# Comprehensive Temporal Protein Dynamics during Postirradiation Recovery in *Deinococcus radiodurans*

**DOI:** 10.1155/2022/1622829

**Published:** 2022-11-11

**Authors:** Yan Xiong, Linyang Wei, Shuchen Xin, Rui Min, Feng Liu, Nuomin Li, Yongqian Zhang

**Affiliations:** ^1^Analysis & Testing Center, Beijing Institute of Technology, Beijing 102488, China; ^2^School of Life Science, Beijing Institute of Technology, Beijing 100081, China

## Abstract

*Deinococcus radiodurans* (*D. radiodurans*) is an extremophile that can tolerate ionizing radiation, ultraviolet radiation, and oxidation. How *D. radiodurans* responds to and survives high levels of ionizing radiation is still not clear. In this study, we performed label-free proteomics to explore the proteome dynamics during postirradiation recovery (PIR). Surprisingly, proteins involved in translation were repressed during the initial hours of PIR. *D. radiodurans* also showed enhanced DNA repair and antioxidative response after 6 kGy of gamma irradiation. Moreover, proteins involved in sulfur metabolism and phenylalanine metabolism were enriched at 1 h and 12 h, respectively, indicating different energy and material needs during PIR. Furthermore, based on these findings, we proposed a novel model to elucidate the possible molecular mechanisms of robust radioresistance in *D. radiodurans*, which may serve as a reference for future radiation repair.

## 1. Introduction

*Deinococcus radiodurans* (*D. radiodurans*) is one of the most radiation-resistant organisms found on Earth [1–3]. The molecular basis of postirradiation recovery (PIR) after a high dose of radiation has become the focus of intense research on the radiation tolerance mechanism of *D. radiodurans*. Previous studies have found that *D. radiodurans* has superb DNA repair ability and a remarkable antioxidative system [4, 5].

At present, most of the evidence regarding the DNA repair mechanism and oxidative stress resistance comes from the transcriptome, knockout mutagenesis, the DNA repair kinetics of mutants, and the activities of DNA repair-related candidate proteins and antioxidant enzymes [6–11]. A few details of the protein changes involved in the *D. radiodurans* radiation-resistant mechanism have also been reported by quantitative proteomics [12–16].

Although these results have shown that several key proteins played an important role in the radiation tolerance mechanism of *D. radiodurans*, they lack deep protein coverage, global protein analysis, and elucidation of the dynamic changes during PIR, which limits further understanding of the biological processes involved in the response to ionizing radiation.

To the best of our knowledge, a comprehensive analysis of the specific radiation tolerance mechanism of *D. radiodurans* has not yet been performed. As the label-free quantitative proteomics method is effective in screening and identifying protein profiles [17, 18], it was applied to conduct a systematic and comprehensive proteomic analysis on *D. radiodurans* at different time points during PIR after 6 kGy *γ*-irradiation. A total of 413 differentially abundant proteins (DAPs, *s*_0_ = 2.0, FDR < 0.05) were reported. The observed profile showed that proteins involved in DNA repair, antioxidative response, sulfur metabolism, and phenylalanine metabolism were significantly enriched. The dataset also resolves the timing of protein induction and repression, indicating the potential primary molecular functions at specific times during PIR. The proteomic changes incorporated in this study preliminarily elucidate the possible mechanism underlying the radiation resistance of *D. radiodurans*.

## 2. Materials and Methods

### 2.1. Strains and Culture Conditions

*D. radiodurans* cells were purchased from the China Common Microorganism Collection Management Center (No. 1.633, CGMCC, Beijing, China). The strains were cultured in TGY liquid medium (1% tryptone, 0.5% glucose, and 0.1% yeast extract) at 30°C with shaking at 150 rpm.

### 2.2. Irradiation Conditions and Postirradiation Recovery

The bacteria were cultured to the early stationary phase (at an OD_600_ of 1.5, 30°C, 150 rpm) and subjected to 6 kGy of ^60^Co *γ*-rays at a dose rate of 30 Gy/min (Peking University, Beijing, China). Another aliquot without irradiation served as the control group. Subsequently, irradiated and control suspensions were centrifuged (10000 × g, 5 min, 4°C) and transferred to fresh TGY at an initial OD_600_ of 0.1 to allow recovery (30°C, 150 rpm). Cell turbidities were collected every 2 hours during PIR. At the required time intervals (0 h, 1 h, 3 h, 6 h, and 12 h), the cells were washed twice with PBS buffer and harvested after centrifugation (10000 × g, 10 min, 4°C). These experiments included three independent biological repeats.

### 2.3. Scanning Electron Microscopy (SEM) and Transmission Electron Microscopy (TEM) Analysis

After irradiation, electron microscopy was performed to analyze cytological morphology [19, 20]. All experimental bacterial strains were collected and fixed with 2.5% glutaraldehyde. The fixed cells were dehydrated by a graded series of ethanol for 15 min each step. For SEM analysis, cells were transferred to isoamyl acetate and dried in a Hitachi Model HCP-2 critical point dryer (Hitachi, Japan) with liquid CO_2_. Pellets for SEM examination were coated with gold and viewed with a Hitachi SU8010SEM instrument (*Hitachi*, Japan). For TEM analysis, specimens were processed as described previously [20]. Ultrathin sections (70–90 nm) were stained with lead citrate and uranyl acetate and observed using a Hitachi H-7650 TEM instrument (Hitachi, Japan).

### 2.4. Protein Extraction and Digestion

The collected bacteria were dissolved in lysis buffer (8 M urea, 2 mM EDTA, 1 mM PMSF) and extracted by ultrasonication in an ice bath (SONICS VCX800, *power*: 800 W, frequency: 20 kHz, treatment time 4 min, vibration 2 s, interval 2 s). The total protein supernatant was centrifuged at 15000 × g for 45 min at 4°C. The protein concentration was optimized using a bicinchoninic acid (BCA) assay kit (Pierce, MA, USA).

For digestion, 50 *μ*g of protein from each sample was used. The protein was reduced by incubating at 56°C for 30 min with 10 mM dithiothreitol (DTT, Sigma). Finally, 50 mM iodoacetamide (IAA, Sigma) was added for alkylation for 30 min. After dilution with 50 mM NH_4_HCO_3_ to decrease the urea concentration to below 2 M, the protein was first digested with trypsin at 37°C overnight at a mass ratio of 50 : 1 (m/m, protein : trypsin) and subsequently at a ratio of 100 : 1 (m/m, protein : trypsin) at 37°C for 4 h.

Finally, digestion was ended by 1% formic acid (*v*/*v*). Peptides were desalted by a Monospin C18 column (Shimadzu, 5010-21700). The desalted eluates were dried, and pellets were stored at -80°C until further analysis.

### 2.5. NanoLC–MS/MS Analysis

Peptides from each sample were dissolved in loading buffer (0.1% FA, *v*/*v*) and centrifuged at 12,000 × g for 10 min. The supernatant was analyzed on a U-3000 nanoLC system (dp92br2) coupled to a Q-Exactive HFX mass spectrometer (Thermo Fisher Scientific, Bremen, Germany). Peptides were separated using a 15 cm house-made C18 reversed-phase column (100 *μ*m inner diameter, 1.9 *μ*m resin) and a 90 min elution gradient. Mobile phase A consisted of 0.1% FA and H_2_O, and mobile phase B consisted of 20% H_2_O and 80% ACN. A 90 min gradient (mobile phase B: 5% at 0 min, 10% at 16 min, 22% at 60 min, 35% at 78 min, 99% at 83 min, 99% at 85 min, 5% at 86 min, and 0% at 90 min) was used at a flow rate of 300 nl/min. The data were acquired in a data-dependent mode. For mass spectrometry parameters, the *m*/*z* range was set to 350-1500 for the MS scan, and the accumulation time was 0.25 s. The top 20 most intense ions in MS1 were selected for MS/MS analysis, and the dynamic exclusion time was 20 s.

### 2.6. Protein Identification and Quantification

The MS/MS data were searched against the *D. radiodurans* database from UniProtKB (https://www.uniprot.org/, uniprot-proteome_UP000002524, last modified on 12/1/2019, 3085 proteins) with MaxQuant software (v 1.6.4.0). The search parameters were as follows: the specific enzyme was trypsin KR_C, which allows up to 4 missing cleavages; carbamidomethyl[C] was set as the fixed modification; oxidation [M] and acetyl [ProteinN-term] were the variable modifications; and the precursor and fragment tolerances were both set to 20 ppm. All protein identification was based on the criteria of a false discovery rate (FDR) less than 1%. The option of matching between runs was enabled with a matching time window of 0.7 min and alignment window of 20 min. The other parameters in MaxQuant were set to the default values. The built-in label-free quantification algorithm (LFQ) in MaxQuant was applied for quantification. The missing values in proteomic datasets were imputed using *NAguideR* [21].

### 2.7. Bioinformatics Analysis

Statistical analysis was performed by Perseus (v.1.6.2.3). The differential proteins were identified at a 5% FDR threshold (*s*0 = 2.0). Furthermore, the functional annotations of differential proteins were analyzed by DAVID 6.8 bioinformatics tools (https://david.ncifcrf.gov/) [22]. Gene Ontology (GO) biological process (GOBP), GO cellular components (GOCC), and GO molecular function (GOMF) terms were identified with FDR < 0.05. STRING (https://www.string-db.org/), Cytoscape (version 3.6.1), plugin ClueGO (version 2.5.4), and Cluepedia (version 1.5.4) were used to show protein–protein interactions (PPIs) of related proteins. A two-sided hypergeometric test with a Benjamini–Hochberg correction was performed to assess enrichment significance. Only results with *p* value < 0.05 are presented. The kappa score of PPI was set to 0.7. KEGG (Kyoto Encyclopedia of Genes and Genomes) was also applied for pathway analysis, and *p* value < 0.05 was considered to be significant using a two-sided hypergeometric test with a Fisher correction.

## 3. Results and Discussion

**Figure 1:**
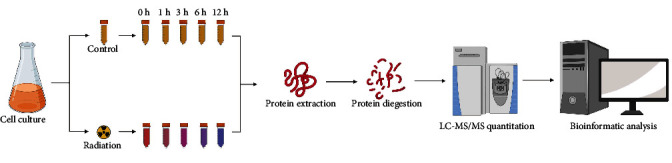
Experimental workflow for differentially abundant proteins in *D. radiodurans* during postirradiation recovery. After radiation, the bacteria in the control and irradiation groups were collected at the required time intervals (0 h, 1 h, 3 h, 6 h, and 12 h) during PIR. Proteins were extracted, digested, and then, subjected to LC–MS/MS analysis. Finally, differentially abundant proteins were evaluated by label-free quantitation.

**Figure 2:**
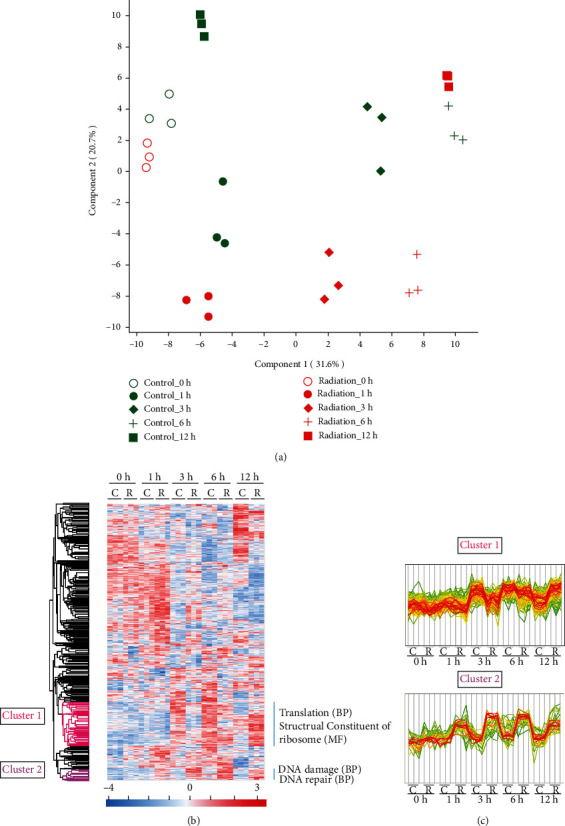
Principal component analysis (PCA) and cluster analysis of proteome data. (a) PCA score biplot of protein percentage data of samples during PIR. The cumulative data variance on the first two PCs was 52.3%. (b) Cluster analysis demonstrating the grouping of the replicate samples and 2 clusters with significantly enriched GO terms. (c) Expression levels are shown in the two clusters (Supplementary Table S8), in which cluster 1 includes translation (GOBP) and structural constituent of ribosome (GOMF) (119 proteins, *p* = 7.5*E* − 3), and cluster 2 includes DNA damage and DNA repair (GOBP) (31 proteins, *p* = 1.8*E* − 4). Proteins belonging to the cluster are shown in green to red based on the distance from the mean of all proteins in the cluster.

The overall experimental workflow is shown in Figure 1. The growth curve during PIR is shown in Figure S1, and quantitative information was obtained for 1942 proteins and 11095 peptides across all samples (Supplementary Table S1-2). The overall reproducibility of these MS-MS data was assessed by performing multivariate statistical analysis. As shown in Figure 2, the first principal component (PC1) captures 31.6% of the variance, followed by PC2 with a 20.7% variance. Visual inspection of the data showed a clear separation of the samples between control and irradiation-treated cells.

**Figure 3:**
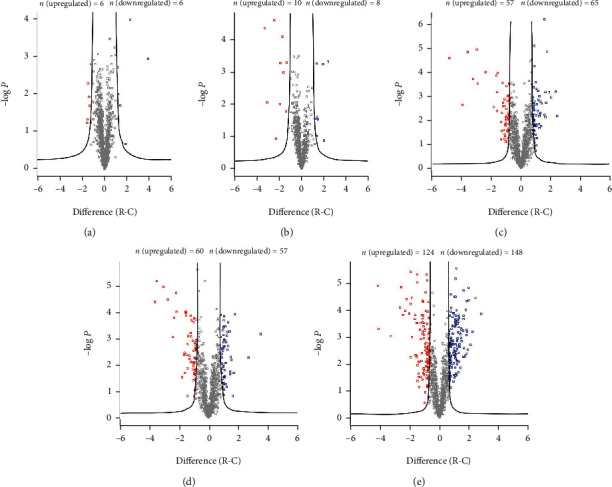
Volcano plots illustrate differentially abundant proteins. Protein abundance (log_2_ fold change) obtained by MS/MS analysis of cells in the control and irradiation groups was plotted against its statistical *p* value. The curve was derived at false discovery rate (FDR) = 0.05 and *s*0 = 2.0. Upregulated proteins are colored red, while downregulated proteins are colored blue.

The numbers of differentially abundant proteins (DAPs) are shown in Table 1 and Figure 3 (Supplementary Table S3-7). As it took time to recover from irradiation, there were few DAPs at 0 h of PIR. The DAPs at 1, 3, 6, and 12 h were subjected to further detailed analysis. We used the DAVID bioinformatics tool to understand the GO clusters of the DAPs at the systems level (Figures 2(b) and 2(c)). According to cluster analysis, upregulated proteins involved in DNA repair (GOBP, cluster 2) at the time intervals of 1, 3, and 6 h; proteins in translation (GOBP, cluster 1) were downregulated at time intervals of 1 and 3 h and while upregulated at 12 h. These dynamic changes indicated that cells slowed down translation and engaged in DNA repair to recover in the early stages of PIR and resumed active growth at later times.

### 3.1. DNA Damage Response

The unusual radioresistance of *D. radiodurans* primarily originates from its efficient DNA repair ability. Proteomic analysis revealed that functional categories of DNA repair were overrepresented in *D. radiodurans* exposed to irradiation compared with the control cells (Figures 2(b) and 2(c)).

**Figure 4:**
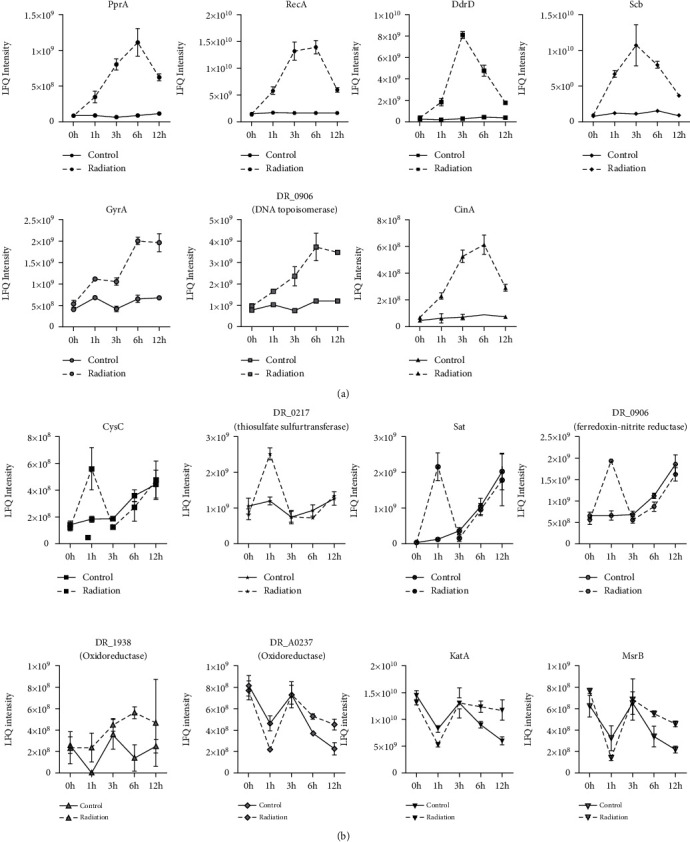
Specific examples of temporal protein profile data. (a) Differentially abundant proteins involved in the DNA damage response. (b) Differentially abundant proteins involved in the antioxidative response. Error bars represent the standard error of triplicate protein relative intensity measurements.

Additionally, Figure 4(a) shows that there were seven DAPs overlapping all the stages of PIR: DNA repair protein PprA, CinA-like protein, protein RecA (recombinase A), DNA damage response protein D (DdrD), single-stranded DNA-binding protein (Ssb), DNA gyrase subunit A (GyrA), and DNA topoisomerase (ATP-hydrolyzing). These proteins were all upregulated and participated in DNA repair.

Single-stranded DNA-binding protein (Ssb) is vital for cell survival in replication and DNA damage repair [23]. Altered Ssb expression significantly affects ionizing radiation tolerance at both the transcript and protein levels [12, 24]. RecA plays a unique role in the repair of DNA damage. It is a recombinase mediating homologous recombination [25]. Comparative proteomics revealed RecA recruitment to the nucleoid of Deinococcus after irradiation-induced DNA damage [26]. PprA is a species-specific radiation-induced protein that ameliorates DNA damage. It plays a critical role in the radiation-induced nonhomologous end-joining repair mechanism [27]. In vivo, PprA interacted with GyrA and DNA topoisomerase to preserve the integrity of the *D. radiodurans* genome after DNA damage [28]. Similar to other Ddr proteins, ddrD gene expression is controlled by the IrrE/DdrO protein pair, and DdrD likely contributes to cell recovery after extensive genotoxic stress [29].

**Figure 5:**
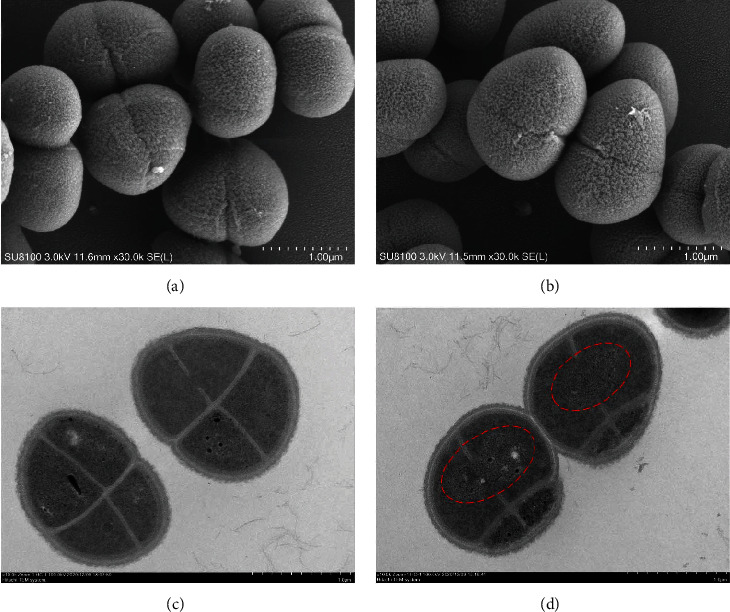
Morphology in *D. radiodurans* after irradiation. (a) SEM images of control cells; (b) SEM images of irradiated cells; (c) TEM images of control cells; (d) TEM images of irradiated cells. DNA spreading between the two compartments through a membranous orifice were marked in dashed red circle.

Investigations with TEM and SEM confirmed this observation. The typical *D. radiodurans* morphology of tetracocci is shown in Figure 5. The ultrastructure of the cell envelope of the radiation group showed that DNA could spread between the two compartments through a membranous orifice (Figure 5(d)). These structural features were conducive to DNA repair in *D. radiodurans* [30].

**Figure 6:**
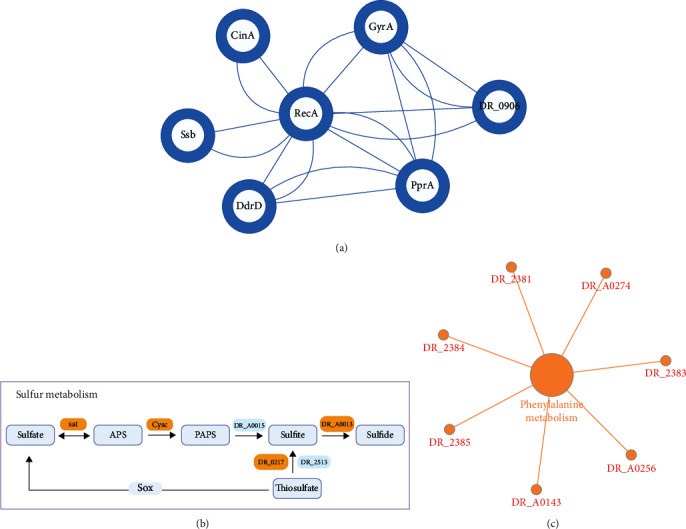
Protein illustration of *D. radiodurans* in response to gamma radiation. (a) Protein interactions of DAPs related to DNA repair. (b) Proteins involved in sulfur metabolism were upregulated (orange) at 1 h of PIR. (c) Proteins involved in phenylalanine metabolism were downregulated (red) at 12 h of PIR.

**Figure 7:**
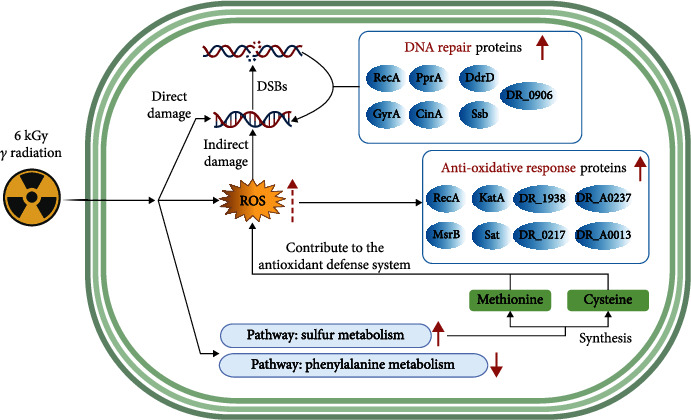
The proposed model elucidates the possible molecular mechanisms of robust radioresistance in *D. radiodurans*. Differentially abundant proteins in the blue ellipse are involved in DNA repair or antioxidative defense during PIR.

Notably, the protein CinA, whose function remains unknown, was upregulated after irradiation. It has been reported that a DNA damage/competence-inducible protein encoded by the cinA ortholog from *Streptococcus pneumonia* could interact physically with RecA [31, 32]. Combined with the PPIs of DAPs (Figure 6(a)), the observed upregulation of CinA may indicate a potential role in the DNA repair system, which needs further study. Altogether, the proteins of *D. radiodurans* that were upregulated in all the stages of PIR indicated extraordinary resistance to the lethal and mutagenic effects of ionizing radiation (Figure 7).

### 3.2. Antioxidative Response

Reactive oxygen species (ROS) generated by ionizing radiation (IR) are deleterious for all organisms, and *D. radiodurans* has evolved robust antioxidant systems to overcome ROS-mediated damage. Our comparative proteomic analysis revealed several differentially abundant proteins related to oxidative stress defense after gamma irradiation (Figure 7).

Proteins involved in the general stress response function to protect and repair damage to cellular structures, such as DNA, the cell envelope, and proteins, and to provide microorganisms the ability to recuperate from the stress they experience (Figure 4(b)). Oxidoreductase, which belongs to the short-chain dehydrogenase/reductase (SDR) family (DR_1938), was upregulated during PIR. Another oxidoreductase (DR_A0237), catalase (KatA), and peptide methionine sulfoxide reductase (MsrB) were differentially upregulated at both 6 h and 12 h. Ferredoxin-nitrite reductase (DR_A0013), thiosulfate sulfurtransferase (DR_0217), sulfate adenylyltransferase (Sat), and adenylyl-sulfate kinase (CysC) were differentially upregulated at 1 h. KatA is an important catalase in the disproportionation of H_2_O_2_ to water and oxygen. *D. radiodurans* with mutagenesis of KatA showed more sensitivity to irradiation [6]. KatA is a well-studied protein that protects *D. radiodurans* from oxidative stress [14]. Because of its sulfur-containing structure, methionine is relatively easily oxidized to yield methionine sulfoxide, leading to conformational changes or inactivation of a protein. Subsequently, methionine sulfoxide can be catalyzed by methionine sulfoxide reductases (Msr proteins) [33, 34]. Therefore, Msr proteins are critical antioxidant enzymes that alleviate the damage caused by oxidative stress [35]. Recent results found that MsrA/B were able to rescue oxidized RecA activities [36]. Moreover, the proteometabolomic response revealed that KatA and MsrA were upregulated under UVC and vacuum conditions [15]. As MsrA and MsrB have complementary stereospecificities in the repair of MetSO in oxidized proteins, we speculated that the upregulation of MsrB contributed to defense against oxidative stresses by reducing methionine sulfoxide residues.

Considering their temporal dynamics during PIR, these proteins upregulated at different time interval during PIR, which indicated multiple strategies were applied in response to oxidative stress. At early stage, enzymes participating in ferredoxin regulation played a role in homeostasis of intracellular metal to resist to ROS-mediated damage, while proteins in deletion of H_2_O_2_ and reversing oxidative forms of sulfur containing amino acids contributed to protection of the bacteria from oxidative stress at late stage.

### 3.3. Sulfur Metabolism and Phenylalanine Metabolism

In addition, KEGG pathway analysis revealed that proteins participating in sulfur metabolism and phenylalanine metabolism were enriched at time intervals of 1 h and 12 h, respectively. Both metabolisms play important roles in protein synthesis, as cysteine and methionine are two essential amino acids that contain sulfur, and phenylalanine is an essential aromatic amino acid.

Cysteine biosynthesis by the sulfate assimilation pathway proceeds by intaking inorganic sulfate into the cell [37], and methionine synthesis is linked to cysteine synthesis [38]. Proteins involved in sulfur metabolism were highly overexpressed at 1 h. They reduce sulfate into sulfide to obtain energy, and sulfide is transferred to a serine moiety to produce cysteine. Meanwhile, methionine and cysteine, as sulfur-containing amino acids, greatly contribute to the antioxidant defense system and are key constituents in the regulation of cell metabolism. Sulfur metabolism plays significant roles in plant and bacterial oxidative stress tolerance [39–41], and we speculate that the overexpressed proteins also contribute to the antioxidative response. Further research is required to elucidate the relevance of these upregulated proteins (Figure 6).

Proteomic analysis also revealed that functional categories of phenylalanine metabolism were overrepresented in *D. radiodurans* during PIR at 12 h (Figure 6). These proteins participate in fatty acid metabolism and the phenylacetate degradation pathway, which are parts of phenylalanine metabolism. The decreased levels indicate the lower degradation of amino acids, which may be beneficial for protein synthesis and helpful for bacterial reproduction.

As shown in Figure 7, our findings showed multiple functional categories of differentially abundant proteins of *D. radiodurans* during PIR. Extensive research has found that the structure of DNA can be damaged directly by high-dose radiation directly or indirectly via consequent ROS accumulation [42, 43]. Thus, increased expression of proteins in the oxidative stress response and DNA repair system was observed. We hypothesized that the elevated level of sulfur metabolism might reduce ROS production and contribute to the antioxidant defense system, as differentially expressed proteins involved in the pathway also functioned in oxidative stress tolerance. Meanwhile, protein temporal dynamics also indicated different protein targets and protein regulation, as well as biological process to be protected at different time interval during PIR.

## 4. Conclusion

*D. radiodurans* is a robust bacterium with high resistance to irradiation. In this study, a comprehensive mapping of protein abundance dynamics during PIR was conducted to investigate its potential resistance capacity. The comparison between irradiation-treated cells and control cells showed a total of 413 DAPs. These proteins involved in DNA repair, antioxidative stress, sulfur metabolism, and phenylalanine metabolism. Several DAPs participated in DNA damage response during all the time intervals, and all the upregulated proteins were related to DNA repair, indicating its efficient DNA repair capacity after irradiation. In addition, proteins with antioxidative response showed upregulated at 1 h, 6 h, and 12 h of PIR. Moreover, proteins participate in sulfur metabolism were overexpressed at 1 h, while proteins related with phenylalanine metabolism were downregulated at 12 h of PIR. The expression level of proteins indicated that energy and material metabolism contribute to the response of *D. radiodurans* to gamma irradiation, and future experiments will be required to elucidate the molecular events.

Overall, these data revealed vivid temporal protein dynamics in *D. radiodurans* during PIR and provided insights to decipher molecular key components in dealing with increased ROS, which are helpful for understanding protein regulation in resistance to irradiation and may serve as a reference for radiation protection.

## Figures and Tables

**Table 1:** Numbers of DAPs during postirradiation recovery.

Postirradiation recovery/h	Upregulated proteins	Downregulated proteins	Differentially abundant proteins (DAPs)
0	6	6	12
1	10	8	18
3	57	65	122
6	60	57	117
12	124	148	272

## Data Availability

The mass spectrometry proteomics data have been deposited to the ProteomeXchange Consortium (http://proteomecentral.proteomexchange.org) via the PRIDE partner repository with the dataset identifier PXD027969.

## Supplementary Materials

Supplementary 1 Figure S1: growth curve of *D. radiodurans* after 6 kGy *γ*-irradiation. After irradiation, suspensions of the control and experimental groups were centrifuged (10000 × g, 5 min, 4°C) and transferred to fresh TGY at an initial OD600 of 0.1 to allow recovery. Cell turbidities were collected every 2 hours during PIR.

Supplementary 2 Tables S1-2: quantitative information across all samples.

Supplementary 3 Tables S3-7: differentially abundant proteins in the comparison control vs. radiation.

Supplementary 4 Table S8: protein IDs in clusters 1 and 2.
