# Supplementary figures and images for: Comprehensive Temporal Protein Dynamics during Postirradiation Recovery in Deinococcus radiodurans
Source: Oxid Med Cell Longev. 2022 Nov 11;2022:1622829. doi: 10.1155/2022/1622829 (PMC9674996; doi:10.1155/2022/1622829)

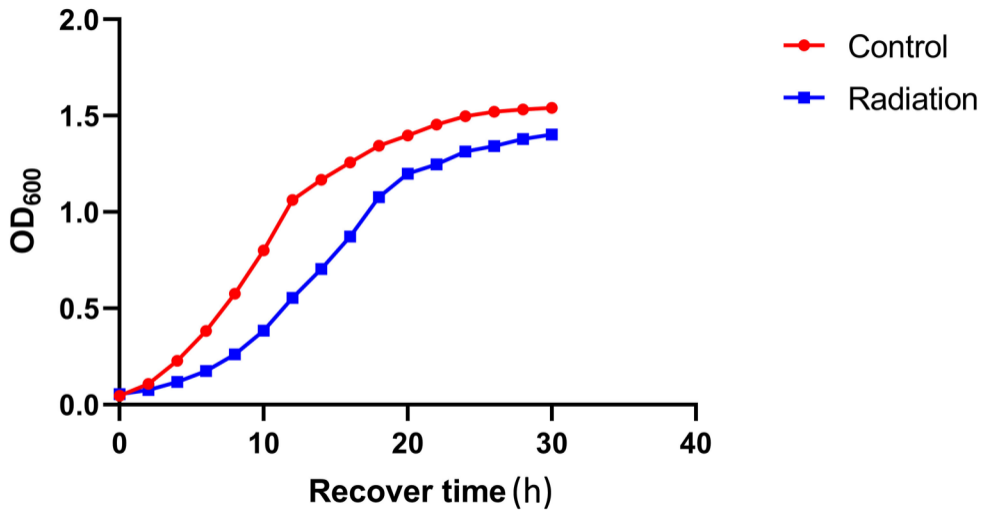

Supplement: Supplementary 1 — Figure S1: growth curve of D. radiodurans after 6 kGy γ-irradiation. After irradiation, suspensions of the control and experimental groups were centrifuged (10000 × g, 5 min, 4°C) and transferred to fresh TGY at an initial OD600 of 0.1 to allow recovery. Cell turbidities were collected every 2 hours during PIR. [file 1622829.f1.pdf]
