# Supplementary material for: Comprehensive Temporal Protein Dynamics during Postirradiation Recovery in Deinococcus radiodurans
Source: Oxid Med Cell Longev. 2022 Nov 11;2022:1622829. doi: 10.1155/2022/1622829 (PMC9674996; doi:10.1155/2022/1622829)
Supplement: Supplementary file 2 [file 1622829.f2.odt]

Table S1 Quantitative proteins across all samples

T: Protein	T: Majority	LFQ intens	LFQ intens	LFQ intens	LFQ intens	LFQ intens	LFQ intens	LFQ intens
sp|O32504	sp|O32504	62211000	1.13E+08	85561000	98169000	1.42E+08	1.05E+08	90504000
sp|O3250	sp|O3250	8.37E+08	6.9E+08	7.45E+08	7.59E+08	7.7E+08	8.66E+08	5.7E+08
sp|O32508	sp|O32508	43663000	52580000	47133000	71634000	69174000	83668000	45188000
sp|O83026	sp|O83026	3.09E+08	4.13E+08	2.77E+08	3.01E+08	2.76E+08	3.21E+08	3.63E+08
sp|O83030	sp|O83030	44895000	56853000	69538000			59918000	84944000
sp|P42443	sp|P42443	1.56E+09	1.59E+09	1.43E+09	1.56E+09	1.54E+09	1.81E+09	1.78E+09
sp|P50933	sp|P50933							
sp|P52027	sp|P52027	4.28E+08	4.33E+08	3.64E+08	4.16E+08	4.61E+08	4.23E+08	4.36E+08
sp|P52153	sp|P52153	2.72E+08	2.78E+08	2.28E+08	4.61E+08	4.78E+08	4.38E+08	3.03E+08
sp|P56860	sp|P56860							
sp|P56861	sp|P56861	1.23E+08	1.75E+08	1.32E+08	1.62E+08	1.89E+08	2.04E+08	1.86E+08
sp|P56864	sp|P56864	20124000	48763000	58207000	1.1E+08	1.21E+08	1.29E+08	2.73E+08
sp|P56867	sp|P56867	1.74E+10	1.81E+10	1.7E+10	1.56E+10	1.43E+10	1.54E+10	1.81E+10
sp|P56905	sp|P56905			84900000	1.38E+08	66106000		
sp|P56925	sp|P56925	5.41E+08	5.46E+08	4.42E+08	3.69E+08	3.58E+08	4.26E+08	5.64E+08
sp|P56926	sp|P56926	3.93E+08	3.98E+08	3.59E+08	3.02E+08	2.73E+08	2.99E+08	3.64E+08
sp|P8358	sp|P8358	2.45E+09	2.48E+09	2.08E+09	2.44E+09	2.2E+09	2.46E+09	3.08E+09
sp|Q4657	sp|Q4657	5.3E+08	4.46E+08	3.99E+08	4.84E+08	4.26E+08	4.9E+08	4.06E+08
sp|Q46578	sp|Q46578	66825000	63689000	59776000	84955000	87719000	98591000	58288000
sp|Q5933	sp|Q5933	1.54E+10	1.44E+10	1.36E+10	7.97E+09	8.95E+09	7.77E+09	1.42E+10
sp|Q9R34	sp|Q9R34	6.88E+09	5.38E+09	5.81E+09	4.63E+09	6.45E+09	5.57E+09	7.41E+09
sp|Q9RR6	sp|Q9RR6	8.95E+09	8.52E+09	8.33E+09	8.45E+09	8.05E+09	8.7E+09	6.49E+09
sp|Q9RR6	sp|Q9RR6	2.65E+08	2.16E+08	2.4E+08	2.03E+08	1.7E+08	1.77E+08	3.18E+08
sp|Q9RR7	sp|Q9RR7	1.08E+08	1.35E+08	1.43E+08	1.39E+08	1.35E+08	93565000	1.1E+08
sp|Q9RR7	sp|Q9RR7	33684000	36503000	39457000	47785000	28075000	41849000	80179000
sp|Q9RR8	sp|Q9RR8							
sp|Q9RR8	sp|Q9RR8							
sp|Q9RR9	sp|Q9RR9	1.65E+08				90927000		1.66E+08
sp|Q9RR9	sp|Q9RR9							
sp|Q9RR9	sp|Q9RR9	5.73E+08	7.85E+08	7.71E+08	6.19E+08	7.69E+08	7.13E+08	7.91E+08
sp|Q9RR9	sp|Q9RR9							
sp|Q9RRB	sp|Q9RRB	1.11E+08	1.18E+08	1.08E+08				1.38E+08
sp|Q9RRB	sp|Q9RRB	4.84E+09	4.9E+09	4.42E+09	2.83E+09	2.52E+09	3.15E+09	7.92E+09
sp|Q9RRB	sp|Q9RRB	2.02E+09	1.72E+09	1.86E+09	2.2E+09	2.02E+09	2.29E+09	1.54E+09
sp|Q9RRC	sp|Q9RRC	79845000	66590000		63054000	63865000	73432000	
sp|Q9RRC	sp|Q9RRC	6.82E+08	7.23E+08	6.55E+08	6.54E+08	6.66E+08	6.57E+08	6.46E+08
sp|Q9RRD	sp|Q9RRD	4.72E+08	3.14E+08	4.55E+08	3.49E+08	3.65E+08	4.05E+08	3.75E+08
sp|Q9RRE	sp|Q9RRE	1.56E+08	1.35E+08	1.67E+08	2.07E+08	1.89E+08	1.73E+08	
sp|Q9RRE	sp|Q9RRE	49292000	76062000	48973000		30748000	74937000	
sp|Q9RRE	sp|Q9RRE							
sp|Q9RRG	sp|Q9RRG	1.95E+09	1.49E+09	1.69E+09				2.88E+09
sp|Q9RRH	sp|Q9RRH	81439000	77184000	63098000	52372000	53924000	58829000	91082000
sp|Q9RRJ1	sp|Q9RRJ1	7.14E+08	8.81E+08	7.85E+08	1.05E+09	1.09E+09	9.34E+08	8.53E+08
sp|Q9RRJ	sp|Q9RRJ	5.8E+08	4.99E+08	5.32E+08	4.21E+08	3.91E+08	4.24E+08	5.12E+08
sp|Q9RRJ5	sp|Q9RRJ5	3.41E+08	4.2E+08	3.46E+08	2.64E+08	2.73E+08	2.65E+08	4.04E+08
sp|Q9RRJ6	sp|Q9RRJ6	65281000			87654000	97974000	44832000	
sp|Q9RRL	sp|Q9RRL	4.04E+08	4.8E+08	4.21E+08	8.95E+08	9.29E+08	6.89E+08	3.54E+08
sp|Q9RRM	sp|Q9RRM	2.05E+08	1.91E+08	1.86E+08	1.94E+08		1.97E+08	1.59E+08
sp|Q9RRM	sp|Q9RRM	2.15E+08	3.63E+08	3.15E+08	3.28E+08	2.68E+08	3.5E+08	

sp|Q9RRP	sp|Q9RRP	3.29E+08	3.36E+08	3.45E+08	2.77E+08	3.29E+08	3.08E+08	3.7E+08
sp|Q9RRQ	sp|Q9RRQ	88092000	84727000	71996000	63991000	71502000	68126000	75754000
sp|Q9RRQ	sp|Q9RRQ	1.05E+09	1.01E+09	9.07E+08	1.09E+09	9.46E+08	1.12E+09	1.1E+09
sp|Q9RRS	sp|Q9RRS	7.22E+08	9.72E+08	9.67E+08	1.13E+09	1.23E+09	9.87E+08	1.14E+09
sp|Q9RRT	sp|Q9RRT	22939000	49536000	47206000	42941000	33533000	22806000	46318000
sp|Q9RRT	sp|Q9RRT	5.3E+08	3.01E+08	3.78E+08	3.08E+08	3.14E+08	3.32E+08	5.87E+08
sp|Q9RRU	sp|Q9RRU							
sp|Q9RRU	sp|Q9RRU							
sp|Q9RRX	sp|Q9RRX	1.19E+09	1.3E+09	1.18E+09	1.53E+09	1.47E+09	1.48E+09	1.72E+09
sp|Q9RRX	sp|Q9RRX	4.59E+08	5.32E+08	5.07E+08	3.35E+08	3.67E+08	3.88E+08	5.27E+08
sp|Q9RRY	sp|Q9RRY	1.98E+08	2.41E+08	2.75E+08	3.91E+08	3.79E+08	3.75E+08	1.76E+08
sp|Q9RRY	sp|Q9RRY							
sp|Q9RS0	sp|Q9RS0		13106000	37008000		8965500	9046500	
sp|Q9RS1	sp|Q9RS1	1.14E+09	9.77E+08	1.01E+09	1.07E+09	9.55E+08	1.15E+09	8.07E+08
sp|Q9RS1	sp|Q9RS1	5.93E+08	6.01E+08	5.11E+08	9.21E+08	8.74E+08	9.06E+08	7.5E+08
sp|Q9RS2	sp|Q9RS2	1.65E+09	1.15E+09	1.42E+09	1.13E+09	1.14E+09	1.12E+09	1.5E+09
sp|Q9RS3	sp|Q9RS3	1.73E+08	1.97E+08	1.5E+08	2.32E+08	2.16E+08	2.38E+08	1.58E+08
sp|Q9RS3	sp|Q9RS3	3.05E+08	2.96E+08	3.03E+08	1.88E+08	1.93E+08	2.11E+08	3.29E+08
sp|Q9RS6	sp|Q9RS6	1.7E+10	1.75E+10	1.41E+10	9.21E+09	7.23E+09	9.57E+09	8.98E+09
sp|Q9RS6	sp|Q9RS6	8.57E+08	8.17E+08	7.45E+08	5.88E+08	5.59E+08	5.82E+08	7.76E+08
sp|Q9RS8	sp|Q9RS8			99178000		1.51E+08	1.1E+08	
sp|Q9RSA	sp|Q9RSA	1.05E+09	1.25E+09	1.14E+09	1.43E+09	1.33E+09	1.45E+09	1.2E+09
sp|Q9RSC	sp|Q9RSC	7.41E+08	6.63E+08	6.04E+08	6.9E+08	6.41E+08	5.9E+08	7.02E+08
sp|Q9RSE	sp|Q9RSE	2.12E+08	2.22E+08	1.89E+08	3.29E+08	3.1E+08	3.07E+08	1.59E+08
sp|Q9RSF	sp|Q9RSF	1.9E+09	1.4E+09	1.39E+09	1.53E+09	1.4E+09	1.67E+09	1.44E+09
sp|Q9RSG	sp|Q9RSG	3.96E+08	3.87E+08	3.6E+08	3.92E+08	4.24E+08	4.13E+08	3.71E+08
sp|Q9RSH	sp|Q9RSH	94085000		79303000				78303000
sp|Q9RSH	sp|Q9RSH		1.24E+08			2.02E+08		
sp|Q9RSI	sp|Q9RSI	1.3E+08	1.28E+08	1.08E+08	1.84E+08	2.38E+08	1.66E+08	2E+08
sp|Q9RSI	sp|Q9RSI	4.66E+08	4.01E+08	3.99E+08	6.71E+08	5.89E+08	5.96E+08	4.52E+08
sp|Q9RSJ	sp|Q9RSJ			14074000				18603000
sp|Q9RSJ	sp|Q9RSJ	9.18E+08	9.95E+08	1.21E+09	1.79E+09	1.69E+09	1.43E+09	1.22E+09
sp|Q9RSJ	sp|Q9RSJ	4.48E+09	4.3E+09	4.26E+09	2.7E+09	3.37E+09	3.37E+09	4.16E+09
sp|Q9RSJ	sp|Q9RSJ			8.92E+08	1.13E+09	1.14E+09	1.13E+09	1.33E+09
sp|Q9RSJ	sp|Q9RSJ	1.42E+09	1.14E+09	1.19E+09	8.36E+08	1.09E+09	1E+09	2.79E+09
sp|Q9RSK	sp|Q9RSK	9.73E+08	8E+08	1.08E+09	4.24E+08	4.77E+08	6.46E+08	4.9E+08
sp|Q9RSK	sp|Q9RSK							
sp|Q9RSK	sp|Q9RSK	2.7E+08	3.47E+08	3.89E+08	2.89E+08	2.63E+08	2.67E+08	
sp|Q9RSK	sp|Q9RSK	7.63E+09	4.84E+09	5.35E+09	5.86E+09	5.61E+09	6.38E+09	8.15E+09
sp|Q9RSL	sp|Q9RSL	41534000	20985000	28704000	10087000	18870000	16504000	44569000
sp|Q9RSL	sp|Q9RSL	4.01E+09	3.31E+09	3.27E+09	1.26E+09	2.07E+09	2.03E+09	5.1E+09
sp|Q9RSL	sp|Q9RSL	3.48E+09	1.93E+09	2.26E+09	8.14E+08	1.3E+09	1.54E+09	5.43E+09
sp|Q9RSL	sp|Q9RSL	1.91E+09	1.65E+09	1.76E+09	2.58E+09	2.72E+09	2.63E+09	1.45E+09
sp|Q9RSL	sp|Q9RSL	5.09E+09	3.91E+09	3.8E+09	1.54E+09	1.99E+09	1.92E+09	6.45E+09
sp|Q9RSL	sp|Q9RSL				42296000			8.08E+09
sp|Q9RSN	sp|Q9RSN	1.94E+09	2.14E+09	2.13E+09	1.13E+09	1.15E+09	1.11E+09	2.43E+09
sp|Q9RSP	sp|Q9RSP	6.81E+08	6.7E+08	5.75E+08	6.98E+08	6.92E+08	5.7E+08	7.34E+08
sp|Q9RSQ	sp|Q9RSQ	2.96E+08	2.73E+08	2.9E+08	1.43E+08	1.68E+08	1.99E+08	2.25E+08
sp|Q9RSQ	sp|Q9RSQ	2.22E+08	2.07E+08	2.17E+08	4.93E+08	2.46E+08	2.13E+08	1.34E+08
sp|Q9RSQ	sp|Q9RSQ		90767000	86583000	31160000	41616000	57416000	91938000
sp|Q9RSR	sp|Q9RSR	2.27E+09	2.29E+09	2.06E+09	2.35E+09	2.08E+09	2.49E+09	2.25E+09
sp|Q9RSR	sp|Q9RSR	1.2E+09	1.08E+09	9.95E+08	9.5E+08	9.75E+08	1.11E+09	1.06E+09
sp|Q9RSS	sp|Q9RSS	36566000	34092000		40951000	40561000	46004000	26091000

sp|Q9RSS	sp|Q9RSS							2.45E+08
sp|Q9RSS	sp|Q9RSS	1.9E+09	2.37E+09	1.95E+09	2.64E+09	2.15E+09	2.5E+09	2.33E+09
sp|Q9RSS	sp|Q9RSS	1.74E+09	1.32E+09	1.33E+09	1.11E+09	1.42E+09	1.27E+09	2.42E+09
sp|Q9RSS	sp|Q9RSS	1.27E+09	1.4E+09	1.51E+09	7.87E+08	9.44E+08	1.28E+09	1.46E+09
sp|Q9RSS	sp|Q9RSS	30564000	29914000	36839000	28343000	26372000	35436000	37831000
sp|Q9RST	sp|Q9RST			31711000	77251000	1.04E+08	79548000	
sp|Q9RSW	sp|Q9RSW							
sp|Q9RSW	sp|Q9RSW	2.3E+08			2.63E+08	2.05E+08	1.64E+08	
sp|Q9RSW	sp|Q9RSW	8.12E+08	7.96E+08	1.07E+09	6.8E+08	4.51E+08	7.5E+08	6.52E+08
sp|Q9RSX	sp|Q9RSX		2.36E+08	1.98E+08	1.8E+08	1.74E+08	1.59E+08	2.03E+08
sp|Q9RSY	sp|Q9RSY	4.76E+09	5.01E+09	5.88E+09	3.35E+09	4.42E+09	2.96E+09	8.42E+09
sp|Q9RSY	sp|Q9RSY	1.65E+08	1.79E+08	1.51E+08	2.76E+08	2.46E+08	3.06E+08	1.14E+08
sp|Q9RSY	sp|Q9RSY							
sp|Q9RSZ	sp|Q9RSZ	6.68E+08	5.53E+08	4.58E+08	8.33E+08	7.33E+08	8.59E+08	5.23E+08
sp|Q9RSZ	sp|Q9RSZ	89838000	54953000	55365000		75993000	84756000	
sp|Q9RT0	sp|Q9RT0	92083000	1.11E+08	1.14E+08	76196000	84642000	1.02E+08	83202000
sp|Q9RT2	sp|Q9RT2	4.04E+09	4.31E+09	3.59E+09	7.09E+09	6.8E+09	5.71E+09	3.64E+09
sp|Q9RT2	sp|Q9RT2	6E+08	4.35E+08	4.39E+08	4.22E+08	5.41E+08	4.08E+08	5.18E+08
sp|Q9RT2	sp|Q9RT2	2.02E+08	2.1E+08	2.21E+08	2.78E+08	3.01E+08	2.85E+08	1.72E+08
sp|Q9RT2	sp|Q9RT2	9.63E+09	9.87E+09	8.63E+09	1.18E+10	1.15E+10	1.31E+10	1.1E+10
sp|Q9RT3	sp|Q9RT3	1.82E+09	2.02E+09	1.96E+09	2.02E+09	2E+09	2.19E+09	1.43E+09
sp|Q9RT4	sp|Q9RT4	4.44E+08	3.45E+08	3.23E+08	4.89E+08	4.11E+08	4.88E+08	5.12E+08
sp|Q9RT5	sp|Q9RT5	2.32E+08	1.67E+08	2.03E+08	1.47E+08	1.55E+08	1.91E+08	
sp|Q9RT6	sp|Q9RT6			96940000		78170000	80953000	89302000
sp|Q9RT6	sp|Q9RT6	1.24E+08	75429000	2.31E+08	2.77E+08	3.61E+08	1.9E+08	1.68E+08
sp|Q9RT7	sp|Q9RT7	1.41E+08	1.41E+08	2.23E+08	2.72E+08	2.24E+08	2.33E+08	
sp|Q9RT9	sp|Q9RT9	4.24E+08	5.2E+08	4.65E+08	3.77E+08	4.39E+08	3.96E+08	4.66E+08
sp|Q9RT9	sp|Q9RT9							
sp|Q9RTA	sp|Q9RTA	75036000	65938000	61644000	69689000	67053000		
sp|Q9RTA	sp|Q9RTA	2.3E+08	1.92E+08	2.27E+08	1.41E+08	2.24E+08	2.36E+08	3.48E+08
sp|Q9RTB	sp|Q9RTB	8.08E+08	7.81E+08	7.51E+08	1.1E+09	9.87E+08	1.08E+09	5.62E+08
sp|Q9RTB	sp|Q9RTB	29405000	30463000	37268000	44117000	35661000	28839000	41994000
sp|Q9RTD	sp|Q9RTD							1.69E+08
sp|Q9RTD	sp|Q9RTD	1.59E+08	1.73E+08	1.68E+08	1.71E+08	1.61E+08	1.36E+08	1.73E+08
sp|Q9RTE	sp|Q9RTE							
sp|Q9RTF	sp|Q9RTF	1.36E+08	99213000	77994000	4.21E+08	4.49E+08	2.53E+08	2.21E+08
sp|Q9RTF	sp|Q9RTF	1.22E+09	1.35E+09	1.28E+09	1.24E+09	1.22E+09	1.24E+09	1.39E+09
sp|Q9RTF	sp|Q9RTF	93261000	1.16E+08	95993000		66276000	1.05E+08	1.09E+08
sp|Q9RTG	sp|Q9RTG	4.81E+08	5.71E+08	5.1E+08	7.87E+08	6.96E+08	7.69E+08	4.01E+08
sp|Q9RTG	sp|Q9RTG							
sp|Q9RTH	sp|Q9RTH	4.38E+08	5.18E+08	4.72E+08	5.93E+08	5.75E+08	5.26E+08	3.94E+08
sp|Q9RTI	sp|Q9RTI	7.33E+08	6.85E+08	7.39E+08	3.94E+08	5.02E+08	5.1E+08	5.49E+08
sp|Q9RTI	sp|Q9RTI6	4.98E+08	5.93E+08	7.12E+08	5.63E+08	5.05E+08	5.25E+08	4.66E+08
sp|Q9RTJ	sp|Q9RTJ3	2.25E+09	1.78E+09	1.86E+09	1.69E+09	1.88E+09	1.48E+09	1.87E+09
sp|Q9RTJ	sp|Q9RTJ5		72885000	55260000		70363000		
sp|Q9RTK	sp|Q9RTK	1.61E+08		1.88E+08	2.06E+08		2.44E+08	1.59E+08
sp|Q9RTK	sp|Q9RTK				1.14E+08			
sp|Q9RTK	sp|Q9RTK							
sp|Q9RTL	sp|Q9RTL	2.16E+08	1.97E+08	1.67E+08	1.93E+08	2.54E+08	1.99E+08	1.73E+08
sp|Q9RTN	sp|Q9RTN			56045000				
sp|Q9RTN	sp|Q9RTN	2.02E+09	2.79E+09	2.34E+09	2.16E+09	2.32E+09	2.1E+09	2.05E+09
sp|Q9RTQ	sp|Q9RTQ	1.31E+09	1.26E+09	1.09E+09	9.43E+08	8.48E+08	9.08E+08	1.38E+09
sp|Q9RTR	sp|Q9RTR							

sp|Q9RTS	sp|Q9RTS							1.54E+08
sp|Q9RTT	sp|Q9RTT	3.75E+08	5.44E+08	4.13E+08	3.3E+08	3.11E+08	2.7E+08	6.69E+08
sp|Q9RTU	sp|Q9RTU							
sp|Q9RTY	sp|Q9RTY	1.05E+08	81815000	86013000				
sp|Q9RTY	sp|Q9RTY							
sp|Q9RTY	sp|Q9RTY	65760000	73491000	76544000	96257000	82515000	58725000	62349000
sp|Q9RU1	sp|Q9RU1	40366000	39474000	47120000			28455000	
sp|Q9RU1	sp|Q9RU1	1.12E+08	87536000	81177000	1.26E+08	1.57E+08	1.38E+08	1.06E+08
sp|Q9RU1	sp|Q9RU1	1.98E+08	1.82E+08	1.75E+08	1.14E+08	1.46E+08	1.5E+08	1.79E+08
sp|Q9RU2	sp|Q9RU2	5.21E+08	5.01E+08	4.6E+08	3.32E+08	4.03E+08	4.15E+08	7.03E+08
sp|Q9RU2	sp|Q9RU2	6.44E+09	5.49E+09	7.81E+09	1.22E+10	9.97E+09	1.23E+10	2.6E+09
sp|Q9RU3	sp|Q9RU3				42821000	59245000	50096000	
sp|Q9RU4	sp|Q9RU4	1.96E+08	1.94E+08	2.09E+08	2E+08	1.55E+08	1.5E+08	
sp|Q9RU6	sp|Q9RU6	1.5E+08	1.7E+08	1.37E+08	1.89E+08	1.96E+08	1E+08	2.75E+08
sp|Q9RU7	sp|Q9RU7	1.24E+08	1.4E+08	1.23E+08	2.15E+08	1.93E+08	1.88E+08	1.61E+08
sp|Q9RU7	sp|Q9RU7	3.02E+08	3.8E+08	3.49E+08	2.7E+08	2.35E+08	1.92E+08	3.46E+08
sp|Q9RU7	sp|Q9RU7	2.12E+09	1.73E+09	2.04E+09	4.32E+09	4.62E+09	3.85E+09	2.89E+09
sp|Q9RU8	sp|Q9RU8	8.42E+09	7.33E+09	8.87E+09	7.3E+09	6.76E+09	8.7E+09	7.12E+09
sp|Q9RU8	sp|Q9RU8	82240000	1.33E+08	1.37E+08				1.06E+08
sp|Q9RU8	sp|Q9RU8	1.92E+09	1.56E+09	1.5E+09	1.06E+09	1.11E+09	1.23E+09	1.48E+09
sp|Q9RU8	sp|Q9RU8	1.78E+08	2.27E+08	2.22E+08	2.68E+08	2.6E+08	2.85E+08	2.61E+08
sp|Q9RU8	sp|Q9RU8	1.4E+08	1.29E+08	1.32E+08				
sp|Q9RU8	sp|Q9RU8		5.15E+08		7.55E+08	5.21E+08	5.33E+08	4E+08
sp|Q9RU8	sp|Q9RU8	32313000	19496000	27618000	95787000	77889000	66758000	26683000
sp|Q9RU9	sp|Q9RU9	42201000		34357000	48945000		54360000	44141000
sp|Q9RUA	sp|Q9RUA	4.65E+08	6.54E+08	5.97E+08	4.08E+08	4.4E+08	3.7E+08	8.22E+08
sp|Q9RUE	sp|Q9RUE	93539000	1.1E+08	1.16E+08	91773000	1.15E+08	68159000	55000000
sp|Q9RUE	sp|Q9RUE	1.54E+09	2.01E+09	2.34E+09	1.66E+09	1.81E+09	1.77E+09	1.43E+09
sp|Q9RUD	sp|Q9RUD	5.94E+08	3.34E+08	4.68E+08	4.5E+08	3.61E+08	3.3E+08	6.13E+08
sp|Q9RUE	sp|Q9RUE	1.14E+08		1.14E+08	1.02E+08		97063000	1.06E+08
sp|Q9RUE	sp|Q9RUE	1.57E+09	2.11E+09	2.49E+09	2.17E+09	1.44E+09	1.32E+09	5.92E+08
sp|Q9RUF	sp|Q9RUF	2.44E+08	1.98E+08	2.34E+08	1.65E+08	1.57E+08	1.79E+08	2.15E+08
sp|Q9RUF	sp|Q9RUF	3.94E+08	4.01E+08	4.21E+08	5.13E+08	4.99E+08	4.7E+08	3.58E+08
sp|Q9RUF	sp|Q9RUF	1.69E+08	1.7E+08	2.78E+08	1.97E+08	1.89E+08	2.02E+08	1.21E+08
sp|Q9RUG	sp|Q9RUG	2.57E+08	2.4E+08	2.34E+08	1.94E+08	1.76E+08	1.89E+08	2.41E+08
sp|Q9RUG	sp|Q9RUG	4.67E+08	3.94E+08	5.73E+08	2.83E+08	3.32E+08	2.87E+08	
sp|Q9RUG	sp|Q9RUG			25877000				
sp|Q9RUH	sp|Q9RUH	42769000		42710000	57956000	49413000		47002000
sp|Q9RUI	sp|Q9RUI	9.75E+08	9.8E+08	6.84E+08	8.13E+08	5.98E+08	6.66E+08	9.02E+08
sp|Q9RUK	sp|Q9RUK	7.42E+08	5.53E+08	5.78E+08	3.19E+08	2.11E+08	4.43E+08	6.03E+08
sp|Q9RUL	sp|Q9RUL	60840000	46787000	63817000		55120000		58061000
sp|Q9RUN	sp|Q9RUN	74966000	83145000					
sp|Q9RUN	sp|Q9RUN	2.36E+08	1.5E+08	2.04E+08	3.55E+08	5.97E+08	2.09E+08	2.13E+08
sp|Q9RUN	sp|Q9RUN	1.09E+09	1.04E+09	9.47E+08	1.24E+09	1.12E+09	1.37E+09	9.34E+08
sp|Q9RUP	sp|Q9RUP	4.88E+08	5.42E+08	5.71E+08	6.16E+08	7.31E+08	5E+08	4.49E+08
sp|Q9RUP	sp|Q9RUP	1.12E+09	1.24E+09	1.13E+09	1.77E+09	1.19E+09	1.74E+09	8.99E+08
sp|Q9RUP	sp|Q9RUP	4.64E+08	3.68E+08	4.26E+08	3.82E+08	3.48E+08	3.79E+08	3.83E+08
sp|Q9RUP	sp|Q9RUP	1.42E+08	1.5E+08	1.47E+08	2.58E+08	1.97E+08	2.28E+08	1.1E+08
sp|Q9RUC	sp|Q9RUC	3.04E+08	3.44E+08	2.76E+08	2.26E+08	2.22E+08	2.09E+08	3.46E+08
sp|Q9RUC	sp|Q9RUC							
sp|Q9RUR	sp|Q9RUR	9.39E+09	9.56E+09	8.88E+09	1.27E+10	1.38E+10	1.24E+10	5.65E+09
sp|Q9RUS	sp|Q9RUS	1.22E+09	1.66E+09	1.37E+09	9.6E+08	1.39E+09	9.9E+08	2.67E+09

sp|Q9RUT	sp|Q9RUT			27078000				
sp|Q9RUV	sp|Q9RUV	1.19E+08	1.69E+08	1.09E+08	96726000	1.15E+08	90510000	1.26E+08
sp|Q9RUV	sp|Q9RUV	9.67E+09	1.05E+10	1.31E+10	6.76E+09	5.07E+09	7.4E+09	3.65E+09
sp|Q9RUV	sp|Q9RUV	1.65E+08	1.68E+08	1.93E+08	5.49E+08	3.88E+08	3.58E+08	1.26E+08
sp|Q9RUV	sp|Q9RUV		25154000	47646000				
sp|Q9RUV	sp|Q9RUV	43360000						57035000
sp|Q9RUV	sp|Q9RUV	2.4E+08	2.51E+08	2.7E+08	2.67E+08	1.99E+08	2.7E+08	1.42E+08
sp|Q9RUV	sp|Q9RUV		15627000	27816000	4655000	9960700		
sp|Q9RUY	sp|Q9RUY	2.44E+08	3.26E+08	4.26E+08	3.74E+08	4.76E+08	5.05E+08	2.08E+08
sp|Q9RUZ	sp|Q9RUZ	5.19E+08	4.88E+08	5.93E+08	1.16E+09	7.57E+08	7.86E+08	3.18E+08
sp|Q9RV1	sp|Q9RV1	7.4E+08	5.24E+08	5.92E+08			6.78E+08	9.23E+08
sp|Q9RV2	sp|Q9RV2	63147000	62211000	56409000	45373000	76543000	48262000	65745000
sp|Q9RV2	sp|Q9RV2	4.77E+09	2.66E+09	2.58E+09	7.94E+09	6.93E+09	4.94E+09	4.72E+09
sp|Q9RV2	sp|Q9RV2	1.17E+09	1.18E+09	1.01E+09	1.13E+09	1.11E+09	1.17E+09	9.26E+08
sp|Q9RV5	sp|Q9RV5	6.88E+08	3.97E+08	5.13E+08	5.41E+08	5.63E+08	6.47E+08	3.34E+08
sp|Q9RV6	sp|Q9RV6	4.61E+08	4.5E+08	3.99E+08	3.99E+08	4.67E+08	4.36E+08	5.49E+08
sp|Q9RV6	sp|Q9RV6	4.08E+08	3.03E+08	3.14E+08	1.16E+09	1.02E+09	1.06E+09	3.35E+08
sp|Q9RV7	sp|Q9RV7	76417000	79043000	75159000	1.18E+08	75326000	85555000	54626000
sp|Q9RV7	sp|Q9RV7		1.22E+08	1.15E+08				
sp|Q9RV8	sp|Q9RV8	1.09E+08	1.44E+08	1.19E+08	1.6E+08	1.53E+08	1.39E+08	1.73E+08
sp|Q9RV9	sp|Q9RV9	7.67E+08	1.05E+09	8.59E+08	2E+09	2.46E+09	1.84E+09	1.46E+09
sp|Q9RV9	sp|Q9RV9	1.93E+08	3.13E+08	2.74E+08	2.8E+08	3.01E+08	2.38E+08	3.7E+08
sp|Q9RV9	sp|Q9RV9	5.59E+08	5.2E+08	5.58E+08	7.76E+08	7.63E+08	8.58E+08	3.35E+08
sp|Q9RVA	sp|Q9RVA	3.75E+08	3.68E+08	3.06E+08	3.95E+08	3.72E+08	4.12E+08	4.34E+08
sp|Q9RVB	sp|Q9RVB	1.37E+08	1.38E+08	1.15E+08	1.89E+08	1.48E+08	1.81E+08	1.56E+08
sp|Q9RVC	sp|Q9RVC	25891000					49895000	
sp|Q9RVC	sp|Q9RVC	2.19E+08	2.9E+08	2.58E+08				2.92E+08
sp|Q9RVC	sp|Q9RVC							
sp|Q9RVC	sp|Q9RVC	1.14E+08	1.11E+08	1.11E+08		1.03E+08	1.31E+08	1.52E+08
sp|Q9RVD	sp|Q9RVD			41645000				
sp|Q9RVD	sp|Q9RVD							
sp|Q9RVE	sp|Q9RVE							
sp|Q9RVE	sp|Q9RVE			20567000				
sp|Q9RVE	sp|Q9RVE	7.87E+08	6.97E+08	7.96E+08	4.54E+08	5.97E+08	5.96E+08	3.17E+08
sp|Q9RVF	sp|Q9RVF							
sp|Q9RVF	sp|Q9RVF		49446000	49806000	58797000	54446000		
sp|Q9RVG	sp|Q9RVG	7.93E+08	6.53E+08	7.14E+08	1.13E+09	9.01E+08	9.93E+08	5.71E+08
sp|Q9RVH	sp|Q9RVH	3.79E+08	4.87E+08	4.1E+08	2.86E+08	2.39E+08	2.47E+08	2.73E+08
sp|Q9RVI	sp|Q9RVI	7.23E+08	8.3E+08	6.77E+08	5.72E+08	4.86E+08	5.8E+08	7.33E+08
sp|Q9RVL	sp|Q9RVL					24967000		
sp|Q9RVM	sp|Q9RVM					45797000		
sp|Q9RVM	sp|Q9RVM	5.85E+08	4.33E+08	4.1E+08	6.77E+08	5.32E+08	6.16E+08	4.67E+08
sp|Q9RVP	sp|Q9RVP	94109000	95326000	83514000	73842000	68509000	82979000	73465000
sp|Q9RVP	sp|Q9RVP	2.55E+09	3.18E+09	3.25E+09	2.22E+09	2.31E+09	2.2E+09	3.03E+09
sp|Q9RVQ	sp|Q9RVQ					1.48E+08	1.45E+08	1.87E+08
sp|Q9RVT	sp|Q9RVT	3.27E+08	4.24E+08	3.58E+08	2.31E+08	1.86E+08	2.21E+08	5.02E+08
sp|Q9RVT	sp|Q9RVT			17437000				
sp|Q9RVT	sp|Q9RVT	32107000	43777000	35536000			27172000	46664000
sp|Q9RVT	sp|Q9RVT		72661000	88204000				
sp|Q9RVV	sp|Q9RVV	1.4E+09	1.47E+09	1.55E+09	1.42E+09	1.4E+09	1.35E+09	1.95E+09
sp|Q9RVW	sp|Q9RVW	2.38E+09	2.43E+09	2.08E+09	1.92E+09	1.79E+09	1.9E+09	2.39E+09

sp|Q9RVX	sp|Q9RVX							
sp|Q9RVY	sp|Q9RVY	5.34E+09	5.82E+09	5.34E+09	4.85E+09	5.07E+09	5.43E+09	4.23E+09
sp|Q9RW	sp|Q9RW	1.4E+08	2.02E+08	2.34E+08	1.36E+08	1.41E+08	1.4E+08	1.55E+08
sp|Q9RW	sp|Q9RW	37177000			42350000	53489000	41077000	
sp|Q9RW1	sp|Q9RW1	5.71E+08	7.23E+08	6.91E+08	6.01E+08	6.77E+08	6.58E+08	6.81E+08
sp|Q9RW2	sp|Q9RW2	1.19E+09	1.04E+09	1.38E+09	1.29E+09	1.33E+09	1.41E+09	2.28E+09
sp|Q9RW4	sp|Q9RW4							
sp|Q9RW4	sp|Q9RW4	76585000	50761000	54101000	58079000	43151000	50288000	
sp|Q9RW5	sp|Q9RW5	77095000	93791000	92511000	83220000	82846000	69652000	75740000
sp|Q9RW6	sp|Q9RW6	2.86E+08	3.97E+08	3.15E+08	2.76E+08	3.37E+08	3.04E+08	3.38E+08
sp|Q9RW6	sp|Q9RW6							
sp|Q9RW6	sp|Q9RW6							
sp|Q9RW7	sp|Q9RW7	1.21E+09	1.4E+09	1.26E+09	1.12E+09	1.13E+09	1.18E+09	1.49E+09
sp|Q9RW9	sp|Q9RW9							
sp|Q9RW9	sp|Q9RW9							
sp|Q9RW9	sp|Q9RW9	2.17E+08	2.23E+08	2.2E+08	1.5E+08	1.31E+08	92964000	1.95E+08
sp|Q9RW9	sp|Q9RW9	1.13E+08	1.02E+08	1.21E+08	1.04E+08	85883000	1.16E+08	2.01E+08
sp|Q9RWA	sp|Q9RWA	4.3E+08	4.73E+08	4.81E+08				
sp|Q9RWB	sp|Q9RWB	5.33E+09	5.56E+09	5.47E+09	5.26E+09	6.13E+09	6.09E+09	4.4E+09
sp|Q9RWB	sp|Q9RWB	2.25E+09	2.46E+09	2.39E+09	2.15E+09	2.06E+09	2.36E+09	3.16E+09
sp|Q9RW	sp|Q9RW	68439000		40429000	45741000		50867000	
sp|Q9RWD	sp|Q9RWD		3.91E+08	3.89E+08	3.51E+08	4.95E+08	2.99E+08	5.89E+08
sp|Q9RWD	sp|Q9RWD							
sp|Q9RW	sp|Q9RW	2.69E+09	2.82E+09	2.54E+09	2.05E+09	2.31E+09	2.53E+09	2.37E+09
sp|Q9RW	sp|Q9RW	9.72E+09	1.18E+10	1.11E+10	9.36E+09	9.84E+09	9.42E+09	8.53E+09
sp|Q9RW	sp|Q9RW	6.29E+09	6.98E+09	6.45E+09	6.08E+09	6.71E+09	5.97E+09	8.77E+09
sp|Q9RW	sp|Q9RW	1.22E+09	1.19E+09	1.06E+09	1.43E+09	1.47E+09	1.15E+09	1.49E+09
sp|Q9RW	sp|Q9RW	3.77E+09	5.2E+09	4.48E+09	3.73E+09	4.94E+09	4.02E+09	8.06E+09
sp|Q9RW	sp|Q9RW	83773000	73187000	64185000	51436000	50149000	57926000	62856000
sp|Q9RW	sp|Q9RW	1.6E+08	1.77E+08	1.56E+08	1.53E+08	84222000		1.32E+08
sp|Q9RWI	sp|Q9RWI	1.12E+08	1.07E+08	93768000		87265000		1.1E+08
sp|Q9RWJ	sp|Q9RWJ							17594000
sp|Q9RWJ	sp|Q9RWJ							
sp|Q9RW	sp|Q9RW	1.35E+08	1.45E+08	1.59E+08	2.03E+08	1.94E+08	2.1E+08	1.37E+08
sp|Q9RW	sp|Q9RW	1.43E+08	1.21E+08	1.38E+08	2.52E+08	2.91E+08	2.89E+08	1.12E+08
sp|Q9RW	sp|Q9RW							
sp|Q9RW	sp|Q9RW		1.54E+08	1.62E+08	1.85E+08	1.53E+08	1.52E+08	
sp|Q9RW	sp|Q9RW	1.31E+08	1.62E+08	1.55E+08		99478000		1.43E+08
sp|Q9RW	sp|Q9RW	2.82E+08	2.24E+08	2.11E+08	2.68E+08	2E+08	2.27E+08	2.87E+08
sp|Q9RW	sp|Q9RW	1.28E+08	1.47E+08	1.29E+08	2.2E+08	2.23E+08	2.16E+08	1.52E+08
sp|Q9RW	sp|Q9RW	3.38E+09	3.25E+09	3.36E+09	3.48E+09	3.78E+09	4.01E+09	2.2E+09
sp|Q9RW	sp|Q9RW	1.56E+09	1.45E+09	1.37E+09	1.34E+09	1.58E+09	1.47E+09	6.65E+08
sp|Q9RW	sp|Q9RW				13292000			
sp|Q9RWS	sp|Q9RWS	4.4E+08	4.29E+08	3.8E+08	3.31E+08	3.24E+08	3.52E+08	4.12E+08
sp|Q9RWS	sp|Q9RWS							
sp|Q9RWU	sp|Q9RWU	3.65E+08	3.95E+08	3.51E+08	4.63E+08	4.39E+08	4.19E+08	2.84E+08
sp|Q9RWV	sp|Q9RWV	2.77E+08	3E+08	2.26E+08	4.5E+08	3.82E+08	3.77E+08	3.09E+08
sp|Q9RWV	sp|Q9RWV	2.94E+08	2.56E+08	2.59E+08	5.23E+08	4.9E+08	4.41E+08	3.42E+08
sp|Q9RX0	sp|Q9RX0		24142000	31552000	35396000	33944000	33953000	
sp|Q9RX1	sp|Q9RX1	2.52E+08	2.53E+08	2.29E+08	1.72E+08	1.52E+08	1.96E+08	2.46E+08
sp|Q9RX1	sp|Q9RX1	9.29E+08	8.93E+08		6.09E+08			8.7E+08

sp|Q9RX2	sp|Q9RX2	1.14E+08	81824000	76917000	86950000	65022000	72166000	1.13E+08
sp|Q9RX2	sp|Q9RX2							
sp|Q9RX2	sp|Q9RX2	2.55E+08	3.3E+08	3.03E+08	3.32E+08	2.7E+08	2.77E+08	2.41E+08
sp|Q9RX3	sp|Q9RX3	1.08E+08	1.45E+08	1.57E+08	2.08E+08	2.21E+08	1.9E+08	81080000
sp|Q9RX3	sp|Q9RX3	98679000	89795000	75383000		1.4E+08	1.24E+08	1.73E+08
sp|Q9RX4	sp|Q9RX4	44952000					37653000	
sp|Q9RX5	sp|Q9RX5	59211000	68329000		45345000	51615000	47228000	61642000
sp|Q9RX5	sp|Q9RX5	48265000	38153000		42746000			
sp|Q9RX6	sp|Q9RX6	4.27E+08	4.64E+08	4.4E+08	4.7E+08	4.26E+08	3.86E+08	3.79E+08
sp|Q9RX8	sp|Q9RX8				3.66E+08	1.28E+08	1.61E+08	
sp|Q9RX9	sp|Q9RX9	97149000						
sp|Q9RX9	sp|Q9RX9							
sp|Q9RXB	sp|Q9RXB	1.11E+08			57460000	51019000	61327000	
sp|Q9RXC	sp|Q9RXC							
sp|Q9RXC	sp|Q9RXC	5.16E+08	6.19E+08	5.4E+08	6.91E+08	6.14E+08	7.11E+08	7.11E+08
sp|Q9RXE	sp|Q9RXE	4.12E+08	4.52E+08	3.8E+08	8.63E+08	5.32E+08	7.56E+08	
sp|Q9RXF	sp|Q9RXF	2.83E+08	2.63E+08	2.18E+08	2.4E+08	2.52E+08	2.57E+08	2.8E+08
sp|Q9RXG	sp|Q9RXG	7.28E+08	7.36E+08	6.11E+08	6.1E+08	5.3E+08	6.43E+08	4.43E+08
sp|Q9RXG	sp|Q9RXG		2.88E+08	2E+08				
sp|Q9RXH	sp|Q9RXH	75306000	89730000	1.57E+08	1.71E+08	1.69E+08	1.82E+08	1.69E+08
sp|Q9RXI7	sp|Q9RXI7	2.36E+08	2.13E+08	2.36E+08	3.6E+08	3.36E+08	4.35E+08	1.7E+08
sp|Q9RXI	sp|Q9RXI	8.55E+09	1.22E+10	9.75E+09	9.1E+09	9.75E+09	8.81E+09	7.55E+09
sp|Q9RXJ0	sp|Q9RXJ0	1.66E+09	1.99E+09	1.68E+09	1.2E+09	1.44E+09	1.46E+09	2.14E+09
sp|Q9RXJ1	sp|Q9RXJ1	3.13E+09	2.86E+09	3.26E+09	1.98E+09	2.88E+09	2.58E+09	2.75E+09
sp|Q9RXJ2	sp|Q9RXJ2	5.15E+08	2.27E+08	3.43E+08	1.73E+08	1.93E+08	1.91E+08	8.81E+08
sp|Q9RXJ3	sp|Q9RXJ3	5.19E+09	3.22E+09	3.76E+09			1.86E+09	6.52E+09
sp|Q9RXJ4	sp|Q9RXJ4		19888000	23683000				21141000
sp|Q9RXJ5	sp|Q9RXJ5							13620000
sp|Q9RXJ6	sp|Q9RXJ6	1.03E+09	1.2E+09	9.34E+08	3.54E+08	4.89E+08	3.58E+08	1.26E+09
sp|Q9RXJ7	sp|Q9RXJ7	5.97E+09	5.27E+09	4.77E+09	4.36E+09	4.04E+09	4.56E+09	7.01E+09
sp|Q9RXJ8	sp|Q9RXJ8			2.16E+08				
sp|Q9RXJ9	sp|Q9RXJ9	3.52E+09	2.62E+09	3.13E+09	3.87E+09	3.15E+09	3.65E+09	3.6E+09
sp|Q9RXK	sp|Q9RXK	4.84E+09	4.16E+09	3.73E+09	1.26E+09	8.84E+08	1.5E+09	7.29E+09
sp|Q9RXK	sp|Q9RXK	1.1E+09	6.62E+08	1.01E+09	1.75E+09	2.46E+09	1.19E+09	9.67E+08
sp|Q9RXK	sp|Q9RXK	3.87E+09	4E+09	4.23E+09	2.28E+09	2.36E+09	2.22E+09	4.69E+09
sp|Q9RXK	sp|Q9RXK	7.75E+08	5.72E+08	9.62E+08	6.82E+08	8.42E+08	8.05E+08	2.88E+09
sp|Q9RXK	sp|Q9RXK	1.48E+09	1.99E+09	1.94E+09	2.46E+09	2.87E+09	2.53E+09	2.73E+09
sp|Q9RXK	sp|Q9RXK	5.74E+09	5.67E+09	6.68E+09	3.42E+09	3.65E+09	3.05E+09	1.34E+10
sp|Q9RXK	sp|Q9RXK							
sp|Q9RXL	sp|Q9RXL	2.6E+08	2.47E+08	2.24E+08	3.98E+08	2.99E+08	2.57E+08	2.75E+08
sp|Q9RXP	sp|Q9RXP	82298000	76073000	80259000	1.73E+08	1.67E+08	1.39E+08	1.02E+08
sp|Q9RXR	sp|Q9RXR							
sp|Q9RXR	sp|Q9RXR	52356000	61853000	34797000	45185000	38750000	36028000	
sp|Q9RXR	sp|Q9RXR	8.4E+08	9.47E+08	7.39E+08	9.56E+08	1.08E+09	9.86E+08	8.65E+08
sp|Q9RXS	sp|Q9RXS	3.02E+08	2.92E+08	2.36E+08	1.94E+08	1.87E+08	2.25E+08	2.21E+08
sp|Q9RXT	sp|Q9RXT			1.42E+08			84151000	
sp|Q9RXT	sp|Q9RXT	1.27E+08	1.89E+08	1.45E+08	1.4E+08	1.28E+08	1.55E+08	
sp|Q9RXT	sp|Q9RXT	3.59E+08	5.14E+08	4.86E+08	5.42E+08	4.75E+08	5.46E+08	4.46E+08
sp|Q9RXT	sp|Q9RXT	8.17E+08	1.1E+09	1.25E+09	1.27E+09	1.07E+09	1.26E+09	5.55E+08
sp|Q9RXU	sp|Q9RXU	1.13E+08	1.25E+08	1.11E+08	1.07E+08	1.21E+08	89487000	1.01E+08
sp|Q9RXV	sp|Q9RXV	1.75E+09	1.53E+09	1.39E+09	1.65E+09	1.65E+09	1.86E+09	1.48E+09

sp|Q9RXW	sp|Q9RXW	76604000	84587000	75193000	90332000	86270000	92598000	68424000
sp|Q9RXX	sp|Q9RXX	1.17E+08	1.28E+08	1.18E+08	1.38E+08	1.36E+08	87152000	
sp|Q9RXY	sp|Q9RXY	4.37E+09	1.14E+10	9.08E+09	4.27E+09	3.41E+09	4.02E+09	1.11E+10
sp|Q9RXY	sp|Q9RXY	3.93E+08	4.58E+08	4.76E+08		1.33E+08	1.09E+08	
sp|Q9RXY	sp|Q9RXY							
sp|Q9RXZ	sp|Q9RXZ							
sp|Q9RY0	sp|Q9RY0	8.8E+08	7.37E+08	7.75E+08	8.89E+08	7.55E+08	7.85E+08	9.4E+08
sp|Q9RY2	sp|Q9RY2	9.5E+09	9.66E+09	8.38E+09	9.17E+09	1.02E+10	8.98E+09	8.39E+09
sp|Q9RY2	sp|Q9RY2	9.52E+08	9.52E+08	7.87E+08	1.09E+09	1.22E+09	1.04E+09	6.51E+08
sp|Q9RY3	sp|Q9RY3	2.55E+09	2.23E+09	2.42E+09	2.86E+09	3.02E+09	2.75E+09	2.18E+09
sp|Q9RY4	sp|Q9RY4	39566000	34048000	31209000		36812000		
sp|Q9RY4	sp|Q9RY4	7.22E+08	6.45E+08	6E+08	7.3E+08	6.71E+08	6.75E+08	5.74E+08
sp|Q9RY4	sp|Q9RY4	1.19E+09	1.3E+09	1.59E+09	1.52E+09	1.64E+09	1.82E+09	2.06E+09
sp|Q9RY5	sp|Q9RY5	2.68E+09	2.06E+09	4.03E+09	2.49E+09	3.41E+09	3.52E+09	2.43E+09
sp|Q9RY5	sp|Q9RY5	7.12E+08	7.8E+08	8.97E+08	7.44E+08	1.04E+09	9.05E+08	1.21E+09
sp|Q9RY5	sp|Q9RY5	1.81E+09	2.06E+09	1.79E+09	1.8E+09	1.96E+09	1.83E+09	3.3E+09
sp|Q9RY6	sp|Q9RY6	3.25E+08	3.42E+08	3.5E+08		1.82E+08		2.78E+08
sp|Q9RY6	sp|Q9RY6	1.56E+08	1.48E+08	1.18E+08	2.25E+08	1.69E+08	1.32E+08	2.02E+08
sp|Q9RY7	sp|Q9RY7	6.04E+08	5.66E+08	5.89E+08	6.83E+08	7.25E+08	8.46E+08	4.88E+08
sp|Q9RY7	sp|Q9RY7	2.78E+08	2.89E+08	2.82E+08	2.49E+08	2.58E+08	2.5E+08	2.52E+08
sp|Q9RY8	sp|Q9RY8							
sp|Q9RYA	sp|Q9RYA							
sp|Q9RYB	sp|Q9RYB	3.56E+09	2.77E+09	3.38E+09	2.54E+09	2.04E+09	2.42E+09	1.54E+09
sp|Q9RYB	sp|Q9RYB	2.59E+08	2.4E+08	2.02E+08	2.34E+08	2.6E+08	2.21E+08	4.09E+08
sp|Q9RYC	sp|Q9RYC	2.45E+08	1.87E+08	1.65E+08	1.55E+08	1.38E+08	1.42E+08	2.81E+08
sp|Q9RYD	sp|Q9RYD							
sp|Q9RYD	sp|Q9RYD	3.24E+08	2.8E+08	2.38E+08	2.28E+08	2.58E+08	2.58E+08	3.04E+08
sp|Q9RYD	sp|Q9RYD	3.54E+08	3.87E+08	3.24E+08	4.11E+08	5.43E+08	4.37E+08	3.72E+08
sp|Q9RYE	sp|Q9RYE	5.13E+08	4.8E+08	4.43E+08	2.01E+08	4.56E+08	4.48E+08	5.38E+08
sp|Q9RYE	sp|Q9RYE							
sp|Q9RYG	sp|Q9RYG	9.36E+08			1.08E+09	7.38E+08	1.03E+09	
sp|Q9RYH	sp|Q9RYH	97012000	1.27E+08	82739000	2.01E+08	1.24E+08	1.83E+08	1.24E+08
sp|Q9RYL	sp|Q9RYL	1.89E+08	1.72E+08	1.44E+08	3E+08	2.31E+08	2.02E+08	1.33E+08
sp|Q9RYM	sp|Q9RYM							
sp|Q9RYM	sp|Q9RYM	4.16E+09	4.03E+09	5.57E+09	8.01E+09	7.34E+09	7.57E+09	1.72E+09
sp|Q9RYM	sp|Q9RYM	2.7E+09	2.54E+09	3.46E+09	3.7E+09	4.35E+09	4.03E+09	1.16E+09
sp|Q9RYP	sp|Q9RYP	2.83E+08	2.44E+08	1.97E+08	2.9E+08	2.44E+08	2.77E+08	2.41E+08
sp|Q9RYR	sp|Q9RYR	1.51E+08	1.21E+08	1.35E+08	1.49E+08	1.1E+08	1.18E+08	
sp|Q9RYR	sp|Q9RYR	1.33E+08	1.76E+08	1.31E+08	1.65E+08	2.27E+08	1.58E+08	1.71E+08
sp|Q9RYU	sp|Q9RYU	4.74E+08	4.44E+08	5.16E+08	5.06E+08	3.3E+08	3.78E+08	3.23E+08
sp|Q9RYU	sp|Q9RYU							
sp|Q9RYV	sp|Q9RYV	5.68E+08	4.7E+08	4.58E+08	4.67E+08	4.83E+08	4.94E+08	4.7E+08
sp|Q9RYW	sp|Q9RYW	1.69E+08	1.9E+08	1.97E+08	2.01E+08	2.1E+08	2.48E+08	2.13E+08
sp|Q9RYX	sp|Q9RYX	1.48E+08	1.92E+08	1.63E+08	2.33E+08	2.06E+08	2.23E+08	1.83E+08
sp|Q9RYY	sp|Q9RYY							
sp|Q9RYZ	sp|Q9RYZ							
sp|Q9RZ0	sp|Q9RZ0	9.02E+08	8E+08	7.39E+08	1.14E+09	1.21E+09	1.29E+09	7.8E+08
sp|Q9RZ0	sp|Q9RZ0		91108000	92806000	84828000		72660000	
sp|Q9RZ0	sp|Q9RZ0	43389000		1.51E+08				
sp|Q9RZ8	sp|Q9RZ8	6.03E+09	7E+09	6.42E+09	1.07E+09	2.61E+09	2.69E+09	4.18E+09
sp|Q9RZA	sp|Q9RZA	1.24E+08	94966000	82319000	86564000	92337000	88535000	1.08E+08

sp|Q9RZE	sp|Q9RZE	82222000	79855000	90484000	76354000	65743000	69717000	89455000
sp|Q9RZL	sp|Q9RZL							
sp|Q9RZN	sp|Q9RZN			44056000	99050000	58427000	39227000	
sp|Q9RZU	sp|Q9RZU							56034000
sp|Q9RZU	sp|Q9RZU	3.62E+08	3.43E+08	2.95E+08	2.93E+08	4.05E+08	3.56E+08	4.19E+08
sp|Q9RZV	sp|Q9RZV	1.7E+08	1.38E+08	1.11E+08	2E+08	1.55E+08	1.73E+08	1.04E+08
sp|Q9WXF	sp|Q9WXF	1.2E+08	1.42E+08	1.46E+08	1.22E+08	1.12E+08	1.18E+08	2.16E+08
sp|Q9X71	sp|Q9X71	6.79E+08	7.28E+08	6.15E+08	6.98E+08	6.18E+08	7.21E+08	7.06E+08
sp|Q9ZNA	sp|Q9ZNA	5.54E+08	5.34E+08	4.95E+08	4.77E+08	3.89E+08	4.44E+08	6.09E+08
tr|Q7DF71	tr|Q7DF71	18169000		19640000				41596000
tr|Q7DF92	tr|Q7DF92							
tr|Q9LBE3	tr|Q9LBE3	6.26E+08	4.78E+08	5.01E+08	3.87E+08	3.85E+08	4E+08	3.22E+08
tr|Q9RR62	tr|Q9RR62	9.12E+08	8.87E+08	8.89E+08	8.78E+08	9.13E+08	8.54E+08	7.24E+08
tr|Q9RR66	tr|Q9RR66	6.11E+08	3.45E+08	3.61E+08	3.19E+08	3.32E+08	2.71E+08	5.39E+08
tr|Q9RR67	tr|Q9RR67							
tr|Q9RR71	tr|Q9RR71	5.96E+08	7.07E+08	6.56E+08	7.62E+08	8.62E+08	7.64E+08	5.9E+08
tr|Q9RR73	tr|Q9RR73	49038000	51287000	44221000			49582000	47450000
tr|Q9RR75	tr|Q9RR75	1.83E+08	2.24E+08	1.76E+08	2.74E+08	2.57E+08	3.05E+08	1.8E+08
tr|Q9RR77	tr|Q9RR77					1.09E+08	97540000	
tr|Q9RR82	tr|Q9RR82	3.43E+08	2.92E+08	2.79E+08	3.76E+08	3.02E+08	4.09E+08	
tr|Q9RR83	tr|Q9RR83	5.05E+08	4.27E+08	4.53E+08	3.17E+08	2.48E+08	3.7E+08	3.44E+08
tr|Q9RR85	tr|Q9RR85	70384000	63036000	91963000				36213000
tr|Q9RR86	tr|Q9RR86	61970000	71953000	54834000	1.17E+08	82857000	67395000	41119000
tr|Q9RR88	tr|Q9RR88							
tr|Q9RR95	tr|Q9RR95	1.63E+08	1.29E+08	1.49E+08	1.35E+08	75893000	66410000	66161000
tr|Q9RR96	tr|Q9RR96	3.08E+08	1.96E+08	1.77E+08				1.9E+08
tr|Q9RR98	tr|Q9RR98	6.04E+08	5.55E+08	5.62E+08	4.28E+08	3.76E+08	4.27E+08	5.79E+08
tr|Q9RR99	tr|Q9RR99	78233000	78749000	69946000	60163000	53490000	65998000	78396000
tr|Q9RRA0	tr|Q9RRA0	8.57E+08	8.24E+08	7.74E+08	1.16E+09	1.06E+09	9.92E+08	8.32E+08
tr|Q9RRA1	tr|Q9RRA1	1.65E+08	2.42E+08	1.23E+08		1.67E+08	2.39E+08	2.8E+08
tr|Q9RRA2	tr|Q9RRA2	4.83E+08	4.49E+08	5.46E+08	1.16E+09	1.1E+09	1.24E+09	5.14E+08
tr|Q9RRA3	tr|Q9RRA3			47113000			47654000	77936000
tr|Q9RRA4	tr|Q9RRA4		1.71E+08	1.68E+08				
tr|Q9RRA5	tr|Q9RRA5							
tr|Q9RRA6	tr|Q9RRA6	2.08E+09	2.29E+09	2.74E+09	3.6E+09	3.53E+09	2.96E+09	1.47E+09
tr|Q9RRA7	tr|Q9RRA7	65449000	71483000	71840000		70876000		58964000
tr|Q9RRA8	tr|Q9RRA8			39003000		49595000		
tr|Q9RRB1	tr|Q9RRB1	63977000	48756000	67619000	42303000		40226000	59810000
tr|Q9RRB2	tr|Q9RRB2							
tr|Q9RRB3	tr|Q9RRB3							
tr|Q9RRB4	tr|Q9RRB4	2.04E+08	2.59E+08	2.23E+08	2.32E+08	2.32E+08	2.37E+08	
tr|Q9RRB8	tr|Q9RRB8	1.05E+08	1.96E+08	2E+08	6.89E+08	4.84E+08	4.82E+08	2.81E+08
tr|Q9RRC0	tr|Q9RRC0			25083000	48362000			33319000
tr|Q9RRC2	tr|Q9RRC2	1.34E+08	1.68E+08					
tr|Q9RRC3	tr|Q9RRC3				27846000			
tr|Q9RRC5	tr|Q9RRC5	1.04E+09	6.67E+08	6.24E+08	8.11E+08	7.04E+08	8.52E+08	3.62E+08
tr|Q9RRC7	tr|Q9RRC7	2.04E+08	2.6E+08	2.44E+08	1.94E+08	1.86E+08	1.89E+08	2.54E+08
tr|Q9RRC9	tr|Q9RRC9							
tr|Q9RRD2	tr|Q9RRD2	2.11E+08	1.84E+08	3.33E+08	2.83E+08	1.93E+08	1.81E+08	
tr|Q9RRD4	tr|Q9RRD4	1.15E+08	1.39E+08	1.2E+08				
tr|Q9RRD5	tr|Q9RRD5	21340000		31469000				

tr|Q9RRD(	tr|Q9RRD(	75278000	70783000	76447000	85102000	60531000	78650000	61592000
tr|Q9RRD9	tr|Q9RRD9			2.02E+08				
tr|Q9RRE0	tr|Q9RRE0	1.08E+08	1.36E+08	1.26E+08	1.83E+08	1.66E+08	1.68E+08	1.59E+08
tr|Q9RRE1	tr|Q9RRE1	2.18E+08	1.98E+08	1.87E+08	1.25E+08	1.75E+08	1.59E+08	1.56E+08
tr|Q9RRE2	tr|Q9RRE2							
tr|Q9RRE3	tr|Q9RRE3	52117000	46239000	39465000	52734000	58182000	43556000	41671000
tr|Q9RRE6	tr|Q9RRE6	28972000	27778000	26500000		31807000	28715000	41275000
tr|Q9RRE7	tr|Q9RRE7	2.55E+08	2.86E+08	2.6E+08	3.53E+08	2.87E+08	3.17E+08	2.21E+08
tr|Q9RRF0	tr|Q9RRF0	4.21E+08	3.62E+08	3.74E+08	3.48E+08	3.5E+08	3.51E+08	3.7E+08
tr|Q9RRF2	tr|Q9RRF2	1.99E+08	2.41E+08	3.07E+08	3.85E+08	4.87E+08	3.28E+08	2.63E+08
tr|Q9RRF4	tr|Q9RRF4	1.05E+08	1.02E+08	93907000	1.09E+08	97849000	88639000	
tr|Q9RRF7	tr|Q9RRF7	8.4E+08	1.11E+09	9.96E+08	8.69E+08	7.8E+08	6.07E+08	8.06E+08
tr|Q9RRG1	tr|Q9RRG1	6.39E+08	6.8E+08	6.2E+08	5.39E+08	5.64E+08	5.68E+08	6.19E+08
tr|Q9RRG3	tr|Q9RRG3	90566000	56088000	54201000	54128000	63064000	56780000	1.61E+08
tr|Q9RRG4	tr|Q9RRG4	4.22E+09	4.65E+09	3.9E+09	4.69E+09	3.62E+09	3.75E+09	3.87E+09
tr|Q9RRG5	tr|Q9RRG5	5.97E+08	6.54E+08	4.91E+08	3.3E+08	2.81E+08	3.32E+08	7.14E+08
tr|Q9RRG6	tr|Q9RRG6	8.91E+08	6.47E+08	7.12E+08	5.17E+08	6.45E+08	6.54E+08	6.75E+08
tr|Q9RRG9	tr|Q9RRG9							
tr|Q9RRH	tr|Q9RRH							
tr|Q9RRH	tr|Q9RRH	2.4E+08	1.9E+08	1.81E+08	7.41E+08	7.72E+08	6.85E+08	1.95E+08
tr|Q9RRH	tr|Q9RRH	84306000	1E+08	71719000	93747000	77148000	68587000	96185000
tr|Q9RRH	tr|Q9RRH	2.22E+08	1.82E+08	1.39E+08	2.04E+08	1.55E+08	1.87E+08	1.72E+08
tr|Q9RRH	tr|Q9RRH	45389000		34342000			31289000	26085000
tr|Q9RRH	tr|Q9RRH	3.39E+08	4.27E+08	6.23E+08	4.82E+08			4.08E+08
tr|Q9RRI1	tr|Q9RRI1	3.06E+08	2.24E+08	1.66E+08	2.32E+08	2.27E+08	2.13E+08	2.1E+08
tr|Q9RRI3	tr|Q9RRI3	2.35E+09	2.71E+09	2.46E+09	3.62E+09	2.94E+09	3.38E+09	1.79E+09
tr|Q9RRI4	tr|Q9RRI4	5.81E+08	3.02E+08	3.97E+08	4.38E+08	5.06E+08	5.26E+08	3.54E+08
tr|Q9RRI6	tr|Q9RRI6	47851000		66231000	1.01E+08	95586000	91652000	79834000
tr|Q9RRI7	tr|Q9RRI7	3.47E+08	3.64E+08	3.16E+08	4.11E+08	3.79E+08	4.31E+08	2.09E+08
tr|Q9RRI9	tr|Q9RRI9	19673000		21387000	24610000	18777000		
tr|Q9RRJ0	tr|Q9RRJ0							
tr|Q9RRJ7	tr|Q9RRJ7	1.55E+09	1.15E+09	1.44E+09	9.9E+08	1.28E+09	1.06E+09	1.4E+09
tr|Q9RRK0	tr|Q9RRK0	66924000	63864000	66695000	2.04E+08	1.37E+08	1.14E+08	71247000
tr|Q9RRK2	tr|Q9RRK2	79812000	59681000	63096000	62558000	85592000	76995000	
tr|Q9RRK3	tr|Q9RRK3					51937000		97823000
tr|Q9RRK6	tr|Q9RRK6	1.77E+08	1.4E+08	1.22E+08		89776000		87631000
tr|Q9RRK8	tr|Q9RRK8							
tr|Q9RRK9	tr|Q9RRK9	1.24E+09	1.36E+09	1.38E+09	1.09E+09	1.46E+09	1.05E+09	1.45E+09
tr|Q9RRL0	tr|Q9RRL0							
tr|Q9RRL1	tr|Q9RRL1							
tr|Q9RRL3	tr|Q9RRL3							
tr|Q9RRL4	tr|Q9RRL4							
tr|Q9RRM	tr|Q9RRM	1.79E+08	2.11E+08	2.13E+08	1.65E+08	1.73E+08	1.92E+08	1E+08
tr|Q9RRM	tr|Q9RRM	2.01E+09	2.11E+09	2.53E+09	7.67E+08	8.13E+08	1.23E+09	2.4E+09
tr|Q9RRM	tr|Q9RRM	72464000	70204000	67630000				
tr|Q9RRN	tr|Q9RRN	66057000	76448000	56509000	57211000	47438000	71933000	94234000
tr|Q9RRN	tr|Q9RRN			80888000	1.77E+08	1.11E+08	1.02E+08	
tr|Q9RRN	tr|Q9RRN							
tr|Q9RRN	tr|Q9RRN							
tr|Q9RRP0	tr|Q9RRP0					10291000		
tr|Q9RRP2	tr|Q9RRP2	88436000	1.55E+08	1.4E+08	1.29E+08	2.02E+08	1.7E+08	1.32E+08

tr|Q9RRP3	tr|Q9RRP3	1.7E+08	3.12E+08	1.71E+08	52815000	66411000	1.03E+08	1.66E+08
tr|Q9RRP4	tr|Q9RRP4	1.9E+09	1.61E+09	1.78E+09	1.42E+09	1.54E+09	1.27E+09	2.28E+09
tr|Q9RRP5	tr|Q9RRP5		5.35E+08	4.43E+08	4.54E+08	4.75E+08	4.54E+08	6.81E+08
tr|Q9RRP8	tr|Q9RRP8	39204000				38448000		
tr|Q9RRP9	tr|Q9RRP9	95348000	4.31E+08	2.3E+08		1.31E+08	1.22E+08	
tr|Q9RRQ	tr|Q9RRQ	1.19E+08	1.04E+08	1.31E+08	78386000	1.09E+08	1.16E+08	
tr|Q9RRQ	tr|Q9RRQ							
tr|Q9RRQ	tr|Q9RRQ	78649000	94088000				54911000	1.4E+08
tr|Q9RRQ	tr|Q9RRQ							
tr|Q9RRQ	tr|Q9RRQ	23054000	32206000	37113000				47975000
tr|Q9RRR0	tr|Q9RRR0	59319000	64051000	55527000			1.59E+08	76920000
tr|Q9RRR3	tr|Q9RRR3	1.08E+09	1.05E+09	9.51E+08	1.04E+09	1.02E+09	9.08E+08	1.26E+09
tr|Q9RRR4	tr|Q9RRR4					32609000	52983000	
tr|Q9RRR8	tr|Q9RRR8	74605000	1.15E+08	95289000	46173000	53395000	69628000	91785000
tr|Q9RRR9	tr|Q9RRR9							
tr|Q9RRS0	tr|Q9RRS0	2.19E+08	2.28E+08	2.6E+08	1.07E+08	1.19E+08	83727000	1.16E+08
tr|Q9RRS1	tr|Q9RRS1							
tr|Q9RRS3	tr|Q9RRS3	72985000	75583000	1E+08	84111000	83359000	75909000	74897000
tr|Q9RRS4	tr|Q9RRS4							
tr|Q9RRS5	tr|Q9RRS5	70258000	58891000	49544000	83124000	71350000	75690000	64492000
tr|Q9RRS6	tr|Q9RRS6					56439000		
tr|Q9RRS7	tr|Q9RRS7	54441000	60975000	64284000				
tr|Q9RRT5	tr|Q9RRT5							
tr|Q9RRT6	tr|Q9RRT6		1.22E+08		1.06E+08	80205000		
tr|Q9RRT7	tr|Q9RRT7					23991000		
tr|Q9RRT9	tr|Q9RRT9	3.3E+08	4.21E+08	3.4E+08	2.94E+08	3.5E+08	3.59E+08	4.15E+08
tr|Q9RRU0	tr|Q9RRU0							
tr|Q9RRU1	tr|Q9RRU1	2.71E+08	2.37E+08	2.74E+08	3.72E+08	3.83E+08	2.89E+08	2.84E+08
tr|Q9RRU4	tr|Q9RRU4			28793000				
tr|Q9RRU9	tr|Q9RRU9	2.04E+08	2.69E+08	2.83E+08	3.72E+08	3.55E+08	3.7E+08	1.33E+08
tr|Q9RRV0	tr|Q9RRV0	3.86E+08	2.73E+08	3.8E+08	3.31E+08	3.57E+08	3.76E+08	2.16E+08
tr|Q9RRV1	tr|Q9RRV1	1.23E+09	6.71E+08	8.31E+08	2.75E+09	3.14E+09	2.68E+09	1.34E+09
tr|Q9RRV2	tr|Q9RRV2	4.7E+08	3.32E+08	3.36E+08	1.43E+09	1.37E+09	1.28E+09	2.81E+08
tr|Q9RRV3	tr|Q9RRV3	4.68E+08	3.42E+08	3.12E+08	6.79E+08	4.61E+08	6.43E+08	2.6E+08
tr|Q9RRV4	tr|Q9RRV4	1.74E+09	1.29E+09	1.56E+09	1.64E+09	1.79E+09	2.2E+09	1.12E+09
tr|Q9RRV7	tr|Q9RRV7	16971000	16396000	18947000	51984000	42839000	50956000	
tr|Q9RRV8	tr|Q9RRV8	2.59E+10	2.53E+10	2.17E+10	1.57E+10	1.17E+10	1.15E+10	2.39E+10
tr|Q9RRW	tr|Q9RRW	1.24E+08	92811000	77835000	94508000	70597000	92626000	
tr|Q9RRW	tr|Q9RRW	5.82E+08	5.78E+08	5.82E+08	7.14E+08	7.86E+08	7.43E+08	7.65E+08
tr|Q9RRW	tr|Q9RRW					92365000	82833000	
tr|Q9RRW	tr|Q9RRW	1.81E+09	1.85E+09	1.9E+09	1.67E+09	1.4E+09	1.66E+09	1.64E+09
tr|Q9RRW	tr|Q9RRW							
tr|Q9RRW	tr|Q9RRW	8.22E+08	1.51E+09	1.51E+09	3.44E+09	3.65E+09	2.52E+09	1.45E+09
tr|Q9RRX0	tr|Q9RRX0	65101000	76687000	58942000	37605000	42650000	54142000	
tr|Q9RRX1	tr|Q9RRX1	2.35E+09	3.34E+09	3.41E+09	2.01E+09	2.27E+09	2.19E+09	2.87E+09
tr|Q9RRX3	tr|Q9RRX3	39112000			48383000	57987000	45527000	
tr|Q9RRX4	tr|Q9RRX4							
tr|Q9RRX6	tr|Q9RRX6		40568000					
tr|Q9RRY2	tr|Q9RRY2	2.48E+08	3.58E+08	3.08E+08	5E+08	4.21E+08	4.55E+08	3.49E+08
tr|Q9RRY3	tr|Q9RRY3		34074000	58366000				36636000
tr|Q9RRY4	tr|Q9RRY4	45241000	33393000	44666000	79331000	64219000	52366000	92821000

tr|Q9RRY5	tr|Q9RRY5	1.22E+08	1.84E+08	1.96E+08	1.79E+08	1.62E+08	2.29E+08	1.69E+08
tr|Q9RRY6	tr|Q9RRY6							
tr|Q9RRY8	tr|Q9RRY8	8544800	5370300	8976200	10316000	10948000	10556000	3466600
tr|Q9RRZ0	tr|Q9RRZ0	2.57E+08	2.19E+08	1.47E+08	3.49E+08	3.04E+08	1.74E+08	3.06E+08
tr|Q9RRZ1	tr|Q9RRZ1	51819000	34495000	36940000	23462000		38479000	
tr|Q9RRZ4	tr|Q9RRZ4	1.21E+08	1.37E+08	1.35E+08	2.46E+08	1.85E+08	2.14E+08	76764000
tr|Q9RRZ5	tr|Q9RRZ5	1.11E+08	1.26E+08	1.03E+08	1.1E+08	1.01E+08	84183000	1.78E+08
tr|Q9RRZ6	tr|Q9RRZ6	72153000	65269000	91835000	43983000	47913000	57465000	1.07E+08
tr|Q9RRZ7	tr|Q9RRZ7	1.34E+08	1.56E+08	1.14E+08		74078000	95502000	
tr|Q9RRZ8	tr|Q9RRZ8				94012000	97537000	93324000	
tr|Q9RS00	tr|Q9RS00	29607000	37965000	38106000	64254000	55512000	50836000	37142000
tr|Q9RS01	tr|Q9RS01							
tr|Q9RS03	tr|Q9RS03							
tr|Q9RS04	tr|Q9RS04	23913000		32939000				
tr|Q9RS05	tr|Q9RS05							
tr|Q9RS07	tr|Q9RS07	1.62E+08	2.13E+08	2.1E+08	1.67E+08	1.67E+08	1.91E+08	2.25E+08
tr|Q9RS08	tr|Q9RS08	7.53E+08	7.44E+08	8.46E+08	6.66E+08	4.64E+08	6.19E+08	6.84E+08
tr|Q9RS09	tr|Q9RS09							
tr|Q9RS10	tr|Q9RS10	4.68E+08	5.21E+08	5.43E+08	4.25E+08	2.96E+08	3.68E+08	3.28E+08
tr|Q9RS11	tr|Q9RS11	2.56E+08	2.2E+08	1.91E+08	1.4E+08	1.21E+08	1.47E+08	3.07E+08
tr|Q9RS13	tr|Q9RS13							
tr|Q9RS15	tr|Q9RS15	1.3E+08	1.23E+08	1.19E+08	97917000	1.01E+08	1.15E+08	1.18E+08
tr|Q9RS17	tr|Q9RS17	4.68E+09	4.68E+09	4.68E+09	4.84E+09	3.96E+09	4.61E+09	3.67E+09
tr|Q9RS18	tr|Q9RS18							
tr|Q9RS21	tr|Q9RS21							
tr|Q9RS25	tr|Q9RS25	4.25E+08	5.41E+08	4.75E+08	7.63E+08	8.46E+08	7.26E+08	6.67E+08
tr|Q9RS26	tr|Q9RS26							
tr|Q9RS28	tr|Q9RS28	2.08E+08	2.23E+08	1.75E+08				3.23E+08
tr|Q9RS29	tr|Q9RS29	1.04E+09	1.03E+09	1E+09	5.29E+08	6.95E+08	6.47E+08	1.76E+09
tr|Q9RS31	tr|Q9RS31	1.01E+08	67397000	85531000	44329000	39883000	61776000	75166000
tr|Q9RS32	tr|Q9RS32		51118000					84298000
tr|Q9RS33	tr|Q9RS33	9.59E+08	9.9E+08	7.49E+08	1.34E+09	1.33E+09	1.28E+09	1.08E+09
tr|Q9RS40	tr|Q9RS40	4.31E+08	4.4E+08	4.26E+08	2.8E+08	2.93E+08	3.01E+08	2.68E+08
tr|Q9RS41	tr|Q9RS41	3.72E+08	3.73E+08	4.16E+08	4.88E+08	4.85E+08	5.53E+08	4E+08
tr|Q9RS46	tr|Q9RS46							
tr|Q9RS47	tr|Q9RS47							
tr|Q9RS49	tr|Q9RS49	4.76E+08	3.95E+08	5.18E+08	1.37E+08			2.22E+08
tr|Q9RS50	tr|Q9RS50							
tr|Q9RS52	tr|Q9RS52	1.5E+08	1.5E+08	1.3E+08	1.56E+08	1.43E+08	1.42E+08	88500000
tr|Q9RS53	tr|Q9RS53							
tr|Q9RS56	tr|Q9RS56	4.38E+08	5.16E+08	4.07E+08	3.3E+08	3.63E+08	3.63E+08	5.82E+08
tr|Q9RS58	tr|Q9RS58							
tr|Q9RS59	tr|Q9RS59			1.59E+08	1.95E+08	1.88E+08	1.97E+08	2.66E+08
tr|Q9RS60	tr|Q9RS60							
tr|Q9RS63	tr|Q9RS63	2.57E+08	2.41E+08	2.24E+08	3.36E+08	2.79E+08	3.05E+08	3.4E+08
tr|Q9RS66	tr|Q9RS66	1.46E+09	1.31E+09	1.18E+09	1.17E+09	1.19E+09	1.14E+09	1.22E+09
tr|Q9RS70	tr|Q9RS70	42109000	34898000		34174000	37255000	34787000	48773000
tr|Q9RS71	tr|Q9RS71	1.69E+09	1.74E+09	1.59E+09	1.4E+09	1.61E+09	1.56E+09	1.53E+09
tr|Q9RS74	tr|Q9RS74						27867000	
tr|Q9RS76	tr|Q9RS76	5.53E+09	4.81E+09	5.46E+09	4.34E+09	3.66E+09	4.65E+09	3.1E+09
tr|Q9RS77	tr|Q9RS77	65850000	78198000	48418000	52133000	48357000		

tr|Q9RS78	tr|Q9RS78							
tr|Q9RS80	tr|Q9RS80	2.07E+08	2.78E+08	2.65E+08	3.22E+08	3.21E+08	3.28E+08	1.32E+08
tr|Q9RS85	tr|Q9RS85	1.05E+08	1.14E+08	93328000	2.69E+08	2.72E+08	2.21E+08	
tr|Q9RS86	tr|Q9RS86							
tr|Q9RS87	tr|Q9RS87	1.32E+09	1.82E+09	1.9E+09	1.05E+09	1.22E+09	1.38E+09	8.08E+08
tr|Q9RS88	tr|Q9RS88	3.11E+08	3.03E+08	2.68E+08	3.29E+08	1.98E+08	50899000	2.58E+08
tr|Q9RS89	tr|Q9RS89	3.06E+08	2.72E+08	2.47E+08	2.52E+08	2.13E+08	2.19E+08	2.55E+08
tr|Q9RS90	tr|Q9RS90	71409000	69303000	65813000	69366000			79025000
tr|Q9RS92	tr|Q9RS92							
tr|Q9RS93	tr|Q9RS93	2.62E+08	2.51E+08	1.94E+08	2.75E+08	2.42E+08	2.81E+08	1.84E+08
tr|Q9RS95	tr|Q9RS95							
tr|Q9RS96	tr|Q9RS96	1.19E+08	88325000	96404000	56124000	44605000	67075000	50988000
tr|Q9RS97	tr|Q9RS97							
tr|Q9RS98	tr|Q9RS98	9.68E+08	9.4E+08	7.62E+08	8.54E+08	8.94E+08	9.53E+08	8.98E+08
tr|Q9RSA2	tr|Q9RSA2	9.62E+08	9.16E+08	8.07E+08	3.48E+08	8.75E+08		1.25E+09
tr|Q9RSA3	tr|Q9RSA3							
tr|Q9RSA4	tr|Q9RSA4							
tr|Q9RSA5	tr|Q9RSA5	2E+10	1.46E+10	1.61E+10	1.84E+10	1.19E+10	1.82E+10	1.67E+10
tr|Q9RSA6	tr|Q9RSA6	4.57E+08	5.57E+08	4.61E+08	1.35E+08	1.8E+08	1.56E+08	4.95E+08
tr|Q9RSA7	tr|Q9RSA7							
tr|Q9RSA8	tr|Q9RSA8		92043000		79326000	91084000	80447000	93869000
tr|Q9RSA9	tr|Q9RSA9							
tr|Q9RSB1	tr|Q9RSB1	29257000	50042000	63611000	40629000	43461000	50621000	56043000
tr|Q9RSB3	tr|Q9RSB3							
tr|Q9RSB4	tr|Q9RSB4							
tr|Q9RSB6	tr|Q9RSB6							
tr|Q9RSB8	tr|Q9RSB8	1.34E+09	1.39E+09	1.14E+09	1.33E+09	1.4E+09	1.53E+09	2.78E+09
tr|Q9RSB9	tr|Q9RSB9	2.53E+08	3.01E+08	2.62E+08	5.06E+08	4.2E+08	5.25E+08	2.02E+08
tr|Q9RSC1	tr|Q9RSC1	1.2E+08	1.05E+08	1.46E+08	1.15E+08	90069000	1.03E+08	
tr|Q9RSC3	tr|Q9RSC3	5.87E+08	1.04E+09	4.81E+08	5.23E+08	5.09E+08	6.54E+08	5.79E+08
tr|Q9RSC6	tr|Q9RSC6	1.06E+08	90016000	1.04E+08	65673000	61025000	56775000	1.3E+08
tr|Q9RSC7	tr|Q9RSC7	34416000						65456000
tr|Q9RSC8	tr|Q9RSC8							
tr|Q9RSD0	tr|Q9RSD0	1.48E+09	1.46E+09	1.44E+09	1.55E+09	1.48E+09	1.42E+09	1.01E+09
tr|Q9RSD1	tr|Q9RSD1							
tr|Q9RSD2	tr|Q9RSD2			66372000				
tr|Q9RSD3	tr|Q9RSD3							
tr|Q9RSD4	tr|Q9RSD4	5.77E+09	4.51E+09	4.48E+09	5.28E+09	4.71E+09	4.83E+09	5.14E+09
tr|Q9RSD6	tr|Q9RSD6	1.11E+08	1.18E+08	1.33E+08	1.55E+08	1.7E+08	1.56E+08	79519000
tr|Q9RSD8	tr|Q9RSD8				1.49E+08	1.68E+08		
tr|Q9RSE0	tr|Q9RSE0							
tr|Q9RSE1	tr|Q9RSE1	3.18E+08	2.47E+08	2.24E+08			1.1E+08	3.72E+08
tr|Q9RSE2	tr|Q9RSE2	2.79E+08	3.28E+08	2.63E+08	2.15E+08	2.55E+08	2.29E+08	3.4E+08
tr|Q9RSE3	tr|Q9RSE3	38263000	37349000	33103000	99244000	89297000	92548000	36225000
tr|Q9RSE4	tr|Q9RSE4	7.57E+08	8.08E+08	7.99E+08	1.34E+08	4.02E+08	2.28E+08	8.83E+08
tr|Q9RSE5	tr|Q9RSE5	5.87E+08	6.63E+08	6.71E+08	2.85E+08	2.11E+08	3.83E+08	5.2E+08
tr|Q9RSE7	tr|Q9RSE7	1.33E+08	1.35E+08	1.4E+08	1.22E+08	1.05E+08	1.26E+08	1.22E+08
tr|Q9RSE8	tr|Q9RSE8					68515000		
tr|Q9RSF1	tr|Q9RSF1							
tr|Q9RSF2	tr|Q9RSF2							
tr|Q9RSF6	tr|Q9RSF6	1.04E+08	1.32E+08	1.02E+08	1.35E+08			1.46E+08

tr|Q9RSF8	tr|Q9RSF8	6.12E+08	5.65E+08	6.49E+08	4.91E+08	5.06E+08	5.25E+08	2.45E+08
tr|Q9RSG2	tr|Q9RSG2					38036000		
tr|Q9RSG3	tr|Q9RSG3							
tr|Q9RSG4	tr|Q9RSG4	1.24E+08	1.44E+08	1.32E+08	66260000	94315000	84092000	95534000
tr|Q9RSG5	tr|Q9RSG5	56400000		56616000	1.1E+08	97233000		62414000
tr|Q9RSG6	tr|Q9RSG6	58640000		39479000		52641000		
tr|Q9RSH0	tr|Q9RSH0	8.43E+08	5.44E+08	6.64E+08	2.67E+08	1.91E+08	2.8E+08	4.86E+08
tr|Q9RSH1	tr|Q9RSH1	3.08E+08	4.18E+08	3.12E+08	3.9E+08	2.07E+08	1.57E+08	3.16E+08
tr|Q9RSH6	tr|Q9RSH6							
tr|Q9RSH8	tr|Q9RSH8	2.99E+08	3.01E+08	3.97E+08		2.07E+08		1.31E+08
tr|Q9RSI0	tr|Q9RSI0							
tr|Q9RSI1	tr|Q9RSI1	41139000	98465000					
tr|Q9RSI2	tr|Q9RSI2							
tr|Q9RSI3	tr|Q9RSI3							
tr|Q9RSI7	tr|Q9RSI7							
tr|Q9RSI8	tr|Q9RSI8							
tr|Q9RSJ0	tr|Q9RSJ0	6.92E+08	1.04E+09		7.5E+08	7.91E+08	8.35E+08	1.55E+09
tr|Q9RSJ1	tr|Q9RSJ1							
tr|Q9RSJ2	tr|Q9RSJ2	5.43E+08	6.07E+08	1.01E+09	4.74E+08	5.49E+08	4.83E+08	
tr|Q9RSJ4	tr|Q9RSJ4	66469000	59857000	44484000	53366000	66760000	64810000	
tr|Q9RSK2	tr|Q9RSK2	6.63E+08	5.88E+08	6.97E+08	7.68E+08	7.37E+08	6.26E+08	5.91E+08
tr|Q9RSK5	tr|Q9RSK5	2.2E+08	2.31E+08	1.61E+08		2E+08	1.8E+08	3.47E+08
tr|Q9RSL6	tr|Q9RSL6	3.35E+08	3.49E+08	3.41E+08	3.61E+08	3.64E+08	4.01E+08	4.17E+08
tr|Q9RSL7	tr|Q9RSL7	5.47E+08	5.66E+08	4.35E+08	2.75E+08	2.48E+08	1.71E+08	4.34E+08
tr|Q9RSL8	tr|Q9RSL8	4.16E+08	3.84E+08	3.96E+08	4.41E+08	4.09E+08	4.09E+08	3.56E+08
tr|Q9RSM	tr|Q9RSM	7.02E+08	8.15E+08	6.31E+08	5.58E+08	5.64E+08	5.64E+08	7.89E+08
tr|Q9RSM	tr|Q9RSM							
tr|Q9RSM	tr|Q9RSM	6.86E+08	5.91E+08	6.12E+08	7.31E+08	8.68E+08	1.04E+09	
tr|Q9RSM	tr|Q9RSM	42571000	78906000	64393000	88319000	80730000	81055000	
tr|Q9RSN0	tr|Q9RSN0	3.08E+08	2.62E+08	2.26E+08	2.3E+08	2.38E+08	2.64E+08	2.21E+08
tr|Q9RSN1	tr|Q9RSN1	73844000	1.02E+08	72416000		78577000		97345000
tr|Q9RSN4	tr|Q9RSN4	5.5E+08	6.25E+08	5.09E+08	6.18E+08	5.85E+08	5.89E+08	7.27E+08
tr|Q9RSN5	tr|Q9RSN5	1.63E+08	1.71E+08	1.51E+08	2.12E+08	1.95E+08	1.83E+08	2.03E+08
tr|Q9RSP0	tr|Q9RSP0	96569000	69428000	76190000	1.37E+08	1.11E+08	1.32E+08	74867000
tr|Q9RSP7	tr|Q9RSP7	1.88E+08				2.11E+08		
tr|Q9RSP8	tr|Q9RSP8	1.72E+08	2.04E+08	1.5E+08	2.89E+08	2.8E+08	2.66E+08	2.25E+08
tr|Q9RSP9	tr|Q9RSP9	1.78E+08	1.68E+08	2.2E+08	1.35E+08	1.74E+08	1.62E+08	1.37E+08
tr|Q9RSQ2	tr|Q9RSQ2							
tr|Q9RSQ4	tr|Q9RSQ4	7.8E+09	7.61E+09	8.9E+09	6.51E+09	6.31E+09	6.16E+09	3.01E+09
tr|Q9RSQ6	tr|Q9RSQ6	5.99E+08		5.96E+08		5.91E+08		
tr|Q9RSR2	tr|Q9RSR2	44664000	32868000	30410000				46490000
tr|Q9RSR6	tr|Q9RSR6							
tr|Q9RSR7	tr|Q9RSR7	4.89E+08	5.71E+08	4.35E+08	4.68E+08	5.25E+08	4.89E+08	5.31E+08
tr|Q9RSR8	tr|Q9RSR8				25674000	35680000	24966000	
tr|Q9RSR9	tr|Q9RSR9	2.27E+08	2.73E+08	2.47E+08	3.67E+08	3.27E+08	3.27E+08	
tr|Q9RSS2	tr|Q9RSS2	18279000			16731000	16423000	22346000	
tr|Q9RSS3	tr|Q9RSS3	1.06E+08	1.41E+08	1.25E+08	2.02E+08	1.59E+08	1.71E+08	2.07E+08
tr|Q9RST1	tr|Q9RST1	4.66E+08	5.03E+08	4.53E+08	5.33E+08	4.58E+08	5.59E+08	4.84E+08
tr|Q9RST2	tr|Q9RST2	4.3E+08	4.36E+08	4.19E+08	4.75E+08	4.51E+08	4.55E+08	3.42E+08
tr|Q9RST3	tr|Q9RST3							
tr|Q9RST7	tr|Q9RST7	8.5E+08	1.01E+09	8.73E+08	7.73E+08	9.27E+08	7.93E+08	9.3E+08

tr|Q9RST9	tr|Q9RST9	1.92E+08	2.06E+08	2.39E+08	2.33E+08	2.7E+08	2.25E+08	2.32E+08
tr|Q9RSU0	tr|Q9RSU0	1.34E+09	1.32E+09	1.03E+09	1.07E+09	1.1E+09	1.13E+09	1.48E+09
tr|Q9RSU1	tr|Q9RSU1	82887000	1.41E+08	1.02E+08	1.75E+08	2.1E+08	1.76E+08	1.46E+08
tr|Q9RSU3	tr|Q9RSU3	4.96E+08	4.84E+08	3.81E+08	7.1E+08	7.13E+08	5.61E+08	6.84E+08
tr|Q9RSU4	tr|Q9RSU4	1.08E+08	89770000	1.03E+08			82568000	98089000
tr|Q9RSU6	tr|Q9RSU6							
tr|Q9RSU7	tr|Q9RSU7	3.4E+09	2.82E+09	2.97E+09	3.42E+09	3.19E+09	3.54E+09	2.79E+09
tr|Q9RSU8	tr|Q9RSU8	1.79E+08	1.89E+08	2.06E+08	50492000		19312000	2.27E+08
tr|Q9RSV0	tr|Q9RSV0							
tr|Q9RSV3	tr|Q9RSV3	2.32E+08	2.66E+08	1.88E+08	2.89E+08	2.68E+08	2.55E+08	3.13E+08
tr|Q9RSV5	tr|Q9RSV5	1.27E+08	1.39E+08	96836000				1.52E+08
tr|Q9RSV7	tr|Q9RSV7	1.34E+08	1.26E+08	1.01E+08	95745000	88684000	95356000	1.59E+08
tr|Q9RSV8	tr|Q9RSV8	58149000	1.14E+08	67382000	83846000	60346000	84712000	53749000
tr|Q9RSW	tr|Q9RSW							
tr|Q9RSW	tr|Q9RSW	7.57E+08	7.53E+08	7.47E+08	8.67E+08	6.49E+08	7.92E+08	5.3E+08
tr|Q9RSW	tr|Q9RSW							81021000
tr|Q9RSX1	tr|Q9RSX1							
tr|Q9RSX3	tr|Q9RSX3	1.34E+08	66137000	60661000				87844000
tr|Q9RSX4	tr|Q9RSX4		92248000		45128000		50994000	78599000
tr|Q9RSX5	tr|Q9RSX5							
tr|Q9RSX6	tr|Q9RSX6	1.21E+08	1.42E+08	1.27E+08	1.18E+08	1.08E+08	1.21E+08	1.12E+08
tr|Q9RSY1	tr|Q9RSY1	4.65E+08	3.37E+08	3.4E+08		3.78E+08		4.92E+08
tr|Q9RSY4	tr|Q9RSY4							
tr|Q9RSY6	tr|Q9RSY6	2.88E+09	3.56E+09	3.36E+09	5.03E+09	4.23E+09	4.47E+09	2.82E+09
tr|Q9RSY7	tr|Q9RSY7	8.44E+08	6.33E+08	5.58E+08	3.92E+08	4.22E+08	5.32E+08	4.52E+08
tr|Q9RSY8	tr|Q9RSY8							
tr|Q9RSY9	tr|Q9RSY9	2.26E+08	1.33E+08	1.61E+08	2.29E+08	4.83E+08	3.31E+08	2.12E+08
tr|Q9RSZ0	tr|Q9RSZ0	1.14E+08	1.21E+08	94651000	1E+08	1.37E+08	1.23E+08	1.76E+08
tr|Q9RSZ3	tr|Q9RSZ3	78159000	78464000	65019000	74852000	71252000	78566000	93717000
tr|Q9RSZ5	tr|Q9RSZ5	3.3E+08	3.2E+08	2.96E+08	1.78E+08	2.47E+08	2.18E+08	3.05E+08
tr|Q9RSZ9	tr|Q9RSZ9	1.83E+09	1.49E+09	1.85E+09	1.23E+09	1.43E+09	1.43E+09	1.46E+09
tr|Q9RT01	tr|Q9RT01			73748000	1.18E+08		93126000	
tr|Q9RT02	tr|Q9RT02	1.97E+08	1.88E+08	2.19E+08	2.58E+08	2.88E+08	3.12E+08	87939000
tr|Q9RT05	tr|Q9RT05	1.49E+08	1.66E+08	1.35E+08	1.47E+08	1.5E+08	1.64E+08	1.94E+08
tr|Q9RT06	tr|Q9RT06	69290000	79845000	65325000	79477000	67859000	70850000	82918000
tr|Q9RT07	tr|Q9RT07	21410000	42104000	36410000	33894000	45659000		
tr|Q9RT08	tr|Q9RT08							
tr|Q9RT09	tr|Q9RT09	1.68E+09	1.98E+09	2.08E+09	2.11E+09	2.21E+09	2.33E+09	1.53E+09
tr|Q9RT11	tr|Q9RT11	97239000	99889000	1.35E+08			1.15E+08	
tr|Q9RT14	tr|Q9RT14	4.4E+08	4.98E+08	5.54E+08	4.02E+08	4.36E+08	5.68E+08	4.96E+08
tr|Q9RT16	tr|Q9RT16							
tr|Q9RT19	tr|Q9RT19							
tr|Q9RT24	tr|Q9RT24	1.68E+09	1.57E+09	1.35E+09	9.95E+08		1.08E+09	1.8E+09
tr|Q9RT26	tr|Q9RT26	4.88E+08	4.74E+08	4.01E+08	4.81E+08	4.49E+08	5.03E+08	3.71E+08
tr|Q9RT28	tr|Q9RT28	5.85E+08	6.52E+08	6.75E+08	4.71E+08	4.36E+08	4.24E+08	1.01E+09
tr|Q9RT29	tr|Q9RT29	1.68E+08	1.81E+08	2.44E+08	2.18E+08	1.83E+08	2.22E+08	1.48E+08
tr|Q9RT30	tr|Q9RT30	2.84E+08	3.22E+08	1.73E+08				3.36E+08
tr|Q9RT31	tr|Q9RT31	98983000		56790000	64491000	56852000	64220000	
tr|Q9RT33	tr|Q9RT33	35740000		36003000	34010000		41784000	
tr|Q9RT34	tr|Q9RT34							
tr|Q9RT35	tr|Q9RT35	1.33E+08		73757000			52523000	1.1E+08

tr|Q9RT36	tr|Q9RT36							
tr|Q9RT37	tr|Q9RT37			54912000		29070000		
tr|Q9RT42	tr|Q9RT42							
tr|Q9RT43	tr|Q9RT43	4.88E+08	4.99E+08	5.02E+08	5.72E+08	5.17E+08	4.93E+08	2.51E+08
tr|Q9RT45	tr|Q9RT45		92814000	76743000		42176000	46983000	80190000
tr|Q9RT46	tr|Q9RT46							
tr|Q9RT49	tr|Q9RT49			28222000				
tr|Q9RT50	tr|Q9RT50			76212000			65647000	
tr|Q9RT51	tr|Q9RT51	34242000	43388000	37657000	1.17E+08	1.16E+08	1.12E+08	49402000
tr|Q9RT53	tr|Q9RT53	3.72E+08	4.5E+08	3.93E+08	6.92E+08	6.98E+08	6.52E+08	3.58E+08
tr|Q9RT58	tr|Q9RT58	1.47E+08	1.86E+08	2E+08	1.22E+08	1.44E+08	1.33E+08	1.54E+08
tr|Q9RT59	tr|Q9RT59							
tr|Q9RT60	tr|Q9RT60							
tr|Q9RT67	tr|Q9RT67							
tr|Q9RT68	tr|Q9RT68	46846000	31655000	34850000	31132000	32460000	26325000	29559000
tr|Q9RT70	tr|Q9RT70	6.57E+08	4.66E+08	7.46E+08	1.56E+09	1.13E+09	1.1E+09	8.26E+08
tr|Q9RT71	tr|Q9RT71	6.18E+08	5.04E+08	4.65E+08	5.05E+08	4.44E+08	4.24E+08	6.2E+08
tr|Q9RT72	tr|Q9RT72	1.37E+08	2.11E+08	2.73E+08	1.09E+08	1.16E+08	2.17E+08	77509000
tr|Q9RT74	tr|Q9RT74	2.52E+08	2.6E+08	2.66E+08	1.74E+08	3.24E+08	1.23E+08	3.6E+08
tr|Q9RT75	tr|Q9RT75	3.66E+08	3.7E+08	3.88E+08	4.65E+08	4.4E+08	4.57E+08	3.47E+08
tr|Q9RT77	tr|Q9RT77							
tr|Q9RT79	tr|Q9RT79							
tr|Q9RT80	tr|Q9RT80					65804000		61949000
tr|Q9RT81	tr|Q9RT81		65863000	57772000	91010000	81485000		
tr|Q9RT82	tr|Q9RT82	69135000	80196000	62716000	1.01E+08	1.07E+08	92984000	53465000
tr|Q9RT87	tr|Q9RT87	1.3E+09	1.36E+09	1.19E+09	1.34E+09	1.43E+09	1.32E+09	1.19E+09
tr|Q9RT92	tr|Q9RT92	30277000	25423000	24589000				22741000
tr|Q9RT93	tr|Q9RT93	43698000	56830000	50260000	65529000	62046000	54528000	54047000
tr|Q9RT94	tr|Q9RT94							
tr|Q9RT95	tr|Q9RT95	76009000	90557000	89933000	60127000	62301000	59829000	1.16E+08
tr|Q9RT96	tr|Q9RT96	32788000	44845000	34554000	62882000	64097000	58459000	34965000
tr|Q9RT97	tr|Q9RT97	1.71E+08	2.11E+08	2.11E+08	2.17E+08	2.39E+08	2.12E+08	2.13E+08
tr|Q9RTA2	tr|Q9RTA2	81161000	77854000	52331000	96656000	84012000	76209000	
tr|Q9RTA3	tr|Q9RTA3		36182000	38021000	54280000	57708000	29322000	54161000
tr|Q9RTA8	tr|Q9RTA8	4.31E+08	3.8E+08	3.52E+08	2.83E+08	2.67E+08	2.92E+08	4.42E+08
tr|Q9RTB3	tr|Q9RTB3	7.28E+08	5.93E+08	4.87E+08	1.6E+09	8.71E+08	1.32E+09	4.32E+08
tr|Q9RTC1	tr|Q9RTC1			3.79E+09	7.75E+09	8.26E+09		
tr|Q9RTC2	tr|Q9RTC2							
tr|Q9RTC3	tr|Q9RTC3							
tr|Q9RTC4	tr|Q9RTC4							
tr|Q9RTC6	tr|Q9RTC6			92583000	81508000	1.37E+08	84319000	
tr|Q9RTC7	tr|Q9RTC7	1.48E+09	1.1E+09	1.13E+09	1.2E+09	7.69E+08	1E+09	1.1E+09
tr|Q9RTC8	tr|Q9RTC8				70431000			
tr|Q9RTC9	tr|Q9RTC9	2.25E+08	2.45E+08	2.12E+08	2.59E+08	2.77E+08	2.91E+08	1.96E+08
tr|Q9RTD1	tr|Q9RTD1				1.46E+08	97872000	1.16E+08	
tr|Q9RTD3	tr|Q9RTD3	6.8E+08	7.54E+08	6.26E+08	9.61E+08	9.55E+08	8.8E+08	7.53E+08
tr|Q9RTD4	tr|Q9RTD4	1.17E+08						
tr|Q9RTD6	tr|Q9RTD6	2.27E+08	2.26E+08	1.72E+08	1.73E+08	1.47E+08	1.54E+08	2.27E+08
tr|Q9RTE1	tr|Q9RTE1		94104000		1.41E+08	1.44E+08	1.13E+08	
tr|Q9RTE2	tr|Q9RTE2	5.56E+08	3.91E+08	4.6E+08	4.76E+08	7.07E+08	6.67E+08	
tr|Q9RTE3	tr|Q9RTE3	9.63E+08	9.63E+08	9.02E+08	8.73E+08	7.28E+08	7.31E+08	1.15E+09

tr|Q9RTE5	tr|Q9RTE5	1.88E+08	1.78E+08	1.71E+08	3.08E+08	2.43E+08	2.83E+08	
tr|Q9RTE8	tr|Q9RTE8	1.43E+08	1.13E+08	1.49E+08		79092000	51831000	1.81E+08
tr|Q9RTE9	tr|Q9RTE9							
tr|Q9RTF0	tr|Q9RTF0							
tr|Q9RTF2	tr|Q9RTF2							
tr|Q9RTF7	tr|Q9RTF7		29424000	45112000	77417000	47864000	57892000	39341000
tr|Q9RTF9	tr|Q9RTF9	47859000	42796000	43294000				53036000
tr|Q9RTG0	tr|Q9RTG0	1.97E+08		1.56E+08	1.36E+08	1.38E+08	1.58E+08	1.32E+08
tr|Q9RTG1	tr|Q9RTG1	4.86E+08	9.62E+08	6.48E+08		6.88E+08	8.53E+08	4.68E+08
tr|Q9RTG6	tr|Q9RTG6							
tr|Q9RTG7	tr|Q9RTG7	1.41E+09	1.11E+09	1.21E+09	1.56E+09	1.22E+09	1.38E+09	1.25E+09
tr|Q9RTG9	tr|Q9RTG9	54447000	57244000	43731000	56357000	44819000	50774000	42617000
tr|Q9RTH0	tr|Q9RTH0							
tr|Q9RTH3	tr|Q9RTH3	1.61E+08	2.07E+08	2.33E+08	1.45E+08	1.67E+08	2.02E+08	2.99E+08
tr|Q9RTH4	tr|Q9RTH4	1.31E+09	1.54E+09	1.6E+09	8.3E+08	7.55E+08	8.02E+08	6.93E+08
tr|Q9RTH7	tr|Q9RTH7							
tr|Q9RTI3	tr|Q9RTI3	2.3E+08	2.62E+08	2.28E+08	2.57E+08	3.27E+08	2.59E+08	3.07E+08
tr|Q9RTI4	tr|Q9RTI4	4.74E+08	5.1E+08	5.44E+08			5.82E+08	5.29E+08
tr|Q9RTI8	tr|Q9RTI8	1.86E+08		2.16E+08				
tr|Q9RTI9	tr|Q9RTI9	1.06E+08	1.24E+08	1.26E+08	2.58E+08	1.98E+08	2.32E+08	2.23E+08
tr|Q9RTJ1	tr|Q9RTJ1	2.5E+08	2.26E+08	2.14E+08	3.27E+08	3.04E+08	3.12E+08	2.15E+08
tr|Q9RTJ4	tr|Q9RTJ4	1.98E+09	1.88E+09	2.65E+09	2.56E+09	2.06E+09	2.36E+09	1.62E+09
tr|Q9RTJ7	tr|Q9RTJ7							
tr|Q9RTJ8	tr|Q9RTJ8							
tr|Q9RTK3	tr|Q9RTK3			26646000	40284000	32019000		
tr|Q9RTK4	tr|Q9RTK4	1.43E+08	1.25E+08	1.33E+08	72954000	1.14E+08	73673000	1.52E+08
tr|Q9RTK5	tr|Q9RTK5	1.03E+09	1.04E+09	1.16E+09	3.84E+08	3.63E+08	4.03E+08	1.23E+09
tr|Q9RTK6	tr|Q9RTK6				7411200			
tr|Q9RTK8	tr|Q9RTK8							
tr|Q9RTL0	tr|Q9RTL0		17872000	33393000				
tr|Q9RTL2	tr|Q9RTL2							
tr|Q9RTL3	tr|Q9RTL3							
tr|Q9RTL4	tr|Q9RTL4	86752000	1.13E+08	94774000	1.59E+08	1.72E+08	1.16E+08	95874000
tr|Q9RTL5	tr|Q9RTL5	17336000	22523000	21453000				
tr|Q9RTL6	tr|Q9RTL6	2.95E+08	3.13E+08	3.16E+08	4.72E+08	5.04E+08	4.18E+08	2.86E+08
tr|Q9RTM	tr|Q9RTM	2.66E+08	2.24E+08	2.13E+08	4.26E+08	4.55E+08	2.91E+08	2.02E+08
tr|Q9RTM	tr|Q9RTM	3.82E+08	3.57E+08	3.45E+08	6.43E+08	6.54E+08	6.33E+08	2.97E+08
tr|Q9RTM	tr|Q9RTM	1.05E+08	1.1E+08	1.23E+08		1.05E+08		1.05E+08
tr|Q9RTM	tr|Q9RTM							
tr|Q9RTM	tr|Q9RTM	1.84E+08	2.24E+08	1.78E+08	1.21E+08	1.08E+08	1.46E+08	2.65E+08
tr|Q9RTN0	tr|Q9RTN0	92177000	1.19E+08	1.05E+08				1.55E+08
tr|Q9RTN3	tr|Q9RTN3	2.93E+09	3.42E+09	2.37E+09	3.86E+09	3.17E+09	3.75E+09	1.44E+09
tr|Q9RTN8	tr|Q9RTN8	2.59E+08	2.25E+08	2.01E+08	1.27E+08	1.39E+08		2.17E+08
tr|Q9RTP0	tr|Q9RTP0	2.07E+08	2.04E+08	1.99E+08	3.68E+08	3.68E+08	3.29E+08	2.42E+08
tr|Q9RTP2	tr|Q9RTP2							
tr|Q9RTP3	tr|Q9RTP3			49174000				
tr|Q9RTP5	tr|Q9RTP5	4.01E+08	4.19E+08	4.53E+08	4.35E+08	3.52E+08	3.9E+08	1.98E+08
tr|Q9RTP6	tr|Q9RTP6							
tr|Q9RTP9	tr|Q9RTP9	80857000	80214000	89992000				
tr|Q9RTQ0	tr|Q9RTQ0	1.83E+09	1.64E+09	1.61E+09	1.11E+09	9.56E+08	1.12E+09	1.9E+09
tr|Q9RTQ1	tr|Q9RTQ1	8.08E+08	9E+08	8.51E+08	8.08E+08	6.17E+08	9.13E+08	7.62E+08

tr|Q9RTQ3	tr|Q9RTQ3	65344000	56138000	85349000	89685000	85465000	89037000	
tr|Q9RTQ5	tr|Q9RTQ5	93005000	1.52E+08	1.07E+08	1.54E+08	1.16E+08	1.15E+08	1.15E+08
tr|Q9RTQ6	tr|Q9RTQ6	1.05E+08	1.04E+08	97746000			45958000	1.27E+08
tr|Q9RTQ7	tr|Q9RTQ7	1.23E+09	1.02E+09	8.84E+08	1.15E+09	1.14E+09	1.14E+09	1.48E+09
tr|Q9RTQ8	tr|Q9RTQ8	89878000		68032000				1.43E+08
tr|Q9RTQ9	tr|Q9RTQ9		62672000	52507000	1.6E+08	1.23E+08	1.33E+08	
tr|Q9RTR1	tr|Q9RTR1							
tr|Q9RTR2	tr|Q9RTR2	4.89E+08	5.03E+08	4.37E+08	5.16E+08	3.83E+08	3.78E+08	4.33E+08
tr|Q9RTR3	tr|Q9RTR3							
tr|Q9RTR4	tr|Q9RTR4	5.46E+08	7.08E+08	6.54E+08	9.26E+08	8.69E+08	8.73E+08	6.65E+08
tr|Q9RTR5	tr|Q9RTR5							
tr|Q9RTR8	tr|Q9RTR8	46207000		51386000	74705000	49382000	51977000	
tr|Q9RTS4	tr|Q9RTS4							
tr|Q9RTS5	tr|Q9RTS5	1.86E+08	2.23E+08	1.8E+08	1.99E+08	2.08E+08	2.02E+08	2.05E+08
tr|Q9RTT1	tr|Q9RTT1							
tr|Q9RTT2	tr|Q9RTT2	1.16E+09	1E+09	9.26E+08	5.54E+08	5.4E+08	6.23E+08	8.3E+08
tr|Q9RTT5	tr|Q9RTT5	1.09E+09	9.84E+08	1.12E+09	1.01E+09	1.05E+09	8.96E+08	6.75E+08
tr|Q9RTT7	tr|Q9RTT7	18979000						
tr|Q9RTU0	tr|Q9RTU0	3.89E+08	3.67E+08	3.31E+08	1.13E+08	66355000	1.41E+08	74420000
tr|Q9RTU1	tr|Q9RTU1	29706000	33112000	25956000				
tr|Q9RTU3	tr|Q9RTU3	50700000	85466000	1.42E+08	1.26E+08	1.49E+08	1.42E+08	
tr|Q9RTU7	tr|Q9RTU7	9.09E+08	8.66E+08	7.38E+08	9.39E+08	9.19E+08	9.37E+08	6.37E+08
tr|Q9RTV1	tr|Q9RTV1	1.73E+08	2.47E+08	4.2E+08	3.46E+08	3.78E+08	3.79E+08	1.32E+08
tr|Q9RTV2	tr|Q9RTV2	1.77E+09	2.72E+09	3.21E+09	7.66E+08	7.64E+08	7.41E+08	1.69E+09
tr|Q9RTV5	tr|Q9RTV5	5.53E+08	5.66E+08	7.8E+08	7.02E+08	6.74E+08	8.05E+08	3.37E+08
tr|Q9RTV6	tr|Q9RTV6	5.14E+08	4.95E+08	4.71E+08	4.77E+08		99096000	7.51E+08
tr|Q9RTV7	tr|Q9RTV7	55035000	55412000	53801000	1.06E+08	45637000	68392000	66019000
tr|Q9RTW	tr|Q9RTW	67726000						
tr|Q9RTW	tr|Q9RTW	1.69E+08	2.35E+08	1.83E+08	98649000	1.26E+08	1.22E+08	1.4E+08
tr|Q9RTW	tr|Q9RTW							
tr|Q9RTW	tr|Q9RTW							
tr|Q9RTW	tr|Q9RTW	1.79E+08	1.76E+08	1.71E+08	2.78E+08	2.7E+08	2.84E+08	1.26E+08
tr|Q9RTW	tr|Q9RTW							
tr|Q9RTX0	tr|Q9RTX0	3.48E+08	3.21E+08	3.11E+08	3.18E+08	3.07E+08	4.12E+08	3.17E+08
tr|Q9RTX3	tr|Q9RTX3							
tr|Q9RTX4	tr|Q9RTX4	1.63E+08	1.96E+08	1.75E+08	2.61E+08	2.32E+08	2.74E+08	1.36E+08
tr|Q9RTX5	tr|Q9RTX5	1.31E+09	1.25E+09	1.15E+09	1.22E+09	1.03E+09	1.11E+09	1.26E+09
tr|Q9RTX6	tr|Q9RTX6	67463000	84108000	81600000	62721000	70128000	64029000	69681000
tr|Q9RTX7	tr|Q9RTX7	1.98E+09	1.85E+09	1.86E+09	1.35E+09	1.05E+09	1.31E+09	1.43E+09
tr|Q9RTX8	tr|Q9RTX8	54700000	41279000					
tr|Q9RTX9	tr|Q9RTX9							
tr|Q9RTY0	tr|Q9RTY0	1E+09	1.27E+09	1.1E+09	1.32E+09	1.49E+09	1.4E+09	9.97E+08
tr|Q9RTY1	tr|Q9RTY1	4.56E+08	4.58E+08	4.46E+08	4E+08	3.24E+08	3.15E+08	5.08E+08
tr|Q9RTY3	tr|Q9RTY3	7.12E+08	6.97E+08	8.24E+08	5.43E+08	5.42E+08	7E+08	3.97E+08
tr|Q9RTY4	tr|Q9RTY4	16471000	21410000	22150000	21137000		15482000	
tr|Q9RTY7	tr|Q9RTY7	1.39E+09	1.55E+09	1.14E+09	7.36E+08	1.08E+09	9.36E+08	1.77E+09
tr|Q9RTY8	tr|Q9RTY8		68934000	1.15E+08	1.02E+08	1.19E+08	1.16E+08	
tr|Q9RTZ0	tr|Q9RTZ0	8.6E+08	7.02E+08	7.08E+08	8.47E+08	9.24E+08	8.32E+08	7.09E+08
tr|Q9RTZ1	tr|Q9RTZ1							1.14E+08
tr|Q9RTZ3	tr|Q9RTZ3	1.74E+08	46751000	1.39E+08	1.99E+08	1.79E+08	2.43E+08	
tr|Q9RTZ4	tr|Q9RTZ4							

tr|Q9RTZ7	tr|Q9RTZ7	3.16E+08	3.27E+08	3.12E+08	4.92E+08	4.56E+08	4.41E+08	2.63E+08
tr|Q9RTZ8	tr|Q9RTZ8	5.29E+08	5.03E+08	4.47E+08	3.86E+08	4.12E+08	4.39E+08	4.66E+08
tr|Q9RTZ9	tr|Q9RTZ9	9.19E+08	8.33E+08	8.59E+08	1.19E+09	1.36E+09	1.23E+09	5.48E+08
tr|Q9RU00	tr|Q9RU00	2.17E+08	2.68E+08	2.15E+08	3.11E+08	3.76E+08	2.85E+08	3.55E+08
tr|Q9RU01	tr|Q9RU01	5.58E+08	6.43E+08	6.12E+08	7.53E+08	7.12E+08	7.02E+08	4.05E+08
tr|Q9RU02	tr|Q9RU02	1.26E+09	1.28E+09	1.15E+09	1.43E+09	1.34E+09	1.29E+09	1.17E+09
tr|Q9RU06	tr|Q9RU06							
tr|Q9RU07	tr|Q9RU07	4.96E+09	4.03E+09	4.15E+09	4.51E+09	5.68E+09	4.5E+09	2.64E+09
tr|Q9RU08	tr|Q9RU08	8.46E+08	9.98E+08	9.36E+08	1.03E+09	1.29E+09	1.07E+09	1.08E+09
tr|Q9RU15	tr|Q9RU15							
tr|Q9RU16	tr|Q9RU16	2.31E+08	2.2E+08	1.63E+08	2.06E+08	2.11E+08	2.1E+08	2.99E+08
tr|Q9RU17	tr|Q9RU17	8.72E+08	8.46E+08	8.82E+08	1.17E+09	1.23E+09	1E+09	8.32E+08
tr|Q9RU22	tr|Q9RU22	2.2E+08	1.37E+08	2.81E+08	1.6E+08	1.91E+08	1.47E+08	2E+08
tr|Q9RU25	tr|Q9RU25	2.34E+08	2.5E+08	2.54E+08	4.12E+08			
tr|Q9RU26	tr|Q9RU26	1.42E+08	1.48E+08	1.11E+08	2.02E+08	1.98E+08	1.5E+08	1.16E+08
tr|Q9RU27	tr|Q9RU27	5.65E+08	6.55E+08	6.91E+08	5.69E+08	6.16E+08	5.74E+08	6.63E+08
tr|Q9RU28	tr|Q9RU28	2.94E+09	3.09E+09	2.38E+09	2.54E+09	2.49E+09	2.51E+09	3.07E+09
tr|Q9RU30	tr|Q9RU30	1.64E+08	1.48E+08	1.58E+08	2.56E+08	1.77E+08	1.79E+08	1.21E+08
tr|Q9RU34	tr|Q9RU34	2.2E+08	1.79E+08	2.69E+08	3.03E+08	2.67E+08	3.54E+08	2.15E+08
tr|Q9RU37	tr|Q9RU37							
tr|Q9RU38	tr|Q9RU38	1.86E+08	1.68E+08	2.01E+08	2.22E+08			
tr|Q9RU39	tr|Q9RU39					24059000		
tr|Q9RU40	tr|Q9RU40							80898000
tr|Q9RU41	tr|Q9RU41	1.6E+08	1.68E+08	1.56E+08	2.31E+08	2.12E+08	2.21E+08	2.7E+08
tr|Q9RU42	tr|Q9RU42				15910000	17696000	26561000	
tr|Q9RU43	tr|Q9RU43	1.07E+09	1.12E+09	1.27E+09	1.26E+09	9.63E+08	1.19E+09	4.04E+08
tr|Q9RU44	tr|Q9RU44							
tr|Q9RU45	tr|Q9RU45							
tr|Q9RU46	tr|Q9RU46							
tr|Q9RU50	tr|Q9RU50	2.19E+08	2.57E+08	2.07E+08	3.41E+08	4.58E+08	3.25E+08	1.55E+08
tr|Q9RU52	tr|Q9RU52	1.21E+09	1.07E+09	1.04E+09	7.62E+08	7.7E+08	9.09E+08	1.24E+09
tr|Q9RU53	tr|Q9RU53	47916000					39763000	
tr|Q9RU54	tr|Q9RU54	3.63E+09	3.96E+09	4.45E+09	4.94E+09	4.48E+09	5.04E+09	2.58E+09
tr|Q9RU55	tr|Q9RU55	3.97E+08	4.31E+08	4.93E+08	2.83E+08	4.49E+08	5.66E+08	1.74E+08
tr|Q9RU56	tr|Q9RU56	6.25E+08	4.81E+08	7.71E+08	4.52E+08	5.82E+08	5.66E+08	4.44E+08
tr|Q9RU57	tr|Q9RU57	6.88E+08	5.39E+08	5.42E+08	4.33E+08	3.66E+08	3.1E+08	6.33E+08
tr|Q9RU58	tr|Q9RU58	3.43E+08	2.57E+08	2.42E+08	2.25E+08	2.3E+08	2.47E+08	2.2E+08
tr|Q9RU59	tr|Q9RU59			70314000	54543000	86474000		
tr|Q9RU60	tr|Q9RU60					1.22E+08	1.19E+08	
tr|Q9RU62	tr|Q9RU62	2.94E+08	3.24E+08	2.56E+08	3.46E+08	3.08E+08	3.19E+08	4.82E+08
tr|Q9RU63	tr|Q9RU63				21724000		21322000	
tr|Q9RU65	tr|Q9RU65	61413000	67500000	46964000			34394000	61550000
tr|Q9RU67	tr|Q9RU67	68261000	63767000	52364000				45966000
tr|Q9RU69	tr|Q9RU69	1.65E+08	1.68E+08	1.69E+08	2.24E+08	1.4E+08	1.93E+08	
tr|Q9RU70	tr|Q9RU70							1.19E+08
tr|Q9RU71	tr|Q9RU71	7.14E+08	8.08E+08	5.84E+08	3.78E+08	3.46E+08	4.26E+08	7.65E+08
tr|Q9RU73	tr|Q9RU73							
tr|Q9RU76	tr|Q9RU76	98221000	1.62E+08	1.49E+08	1.2E+08	1.07E+08	1.15E+08	94981000
tr|Q9RU77	tr|Q9RU77	6.67E+08	7.16E+08	6.54E+08		2.25E+08		4.48E+08
tr|Q9RU78	tr|Q9RU78		1.35E+08					1.03E+08
tr|Q9RU91	tr|Q9RU91	1.15E+08	1.05E+08	1.32E+08	1.38E+08	1.5E+08	1.54E+08	90075000

tr|Q9RU92	tr|Q9RU92	1.61E+08	1.55E+08	1.73E+08	2.85E+08	2.32E+08	2.65E+08	
tr|Q9RU93	tr|Q9RU93	2.67E+08	2.86E+08	2.84E+08	7.08E+08	6.19E+08	8.06E+08	2.53E+08
tr|Q9RU98	tr|Q9RU98	30238000	28545000	35990000	60291000	47249000	69202000	
tr|Q9RU99	tr|Q9RU99							
tr|Q9RUA	tr|Q9RUA				1.53E+08	1.54E+08	1.47E+08	
tr|Q9RUA	tr|Q9RUA	1.44E+08	2.36E+08	3.05E+08	88903000	1.14E+08	1.34E+08	1.43E+08
tr|Q9RUA	tr|Q9RUA	65816000	63859000	65908000	77065000	72900000	93293000	1.21E+08
tr|Q9RUA	tr|Q9RUA	1.76E+09	1.99E+09	1.76E+09	1.77E+09	2.06E+09	1.84E+09	1.36E+09
tr|Q9RUA	tr|Q9RUA	87049000	94902000	72261000				74289000
tr|Q9RUA	tr|Q9RUA	85460000	61780000	69520000	30515000	39392000		53582000
tr|Q9RUA	tr|Q9RUA	2.97E+09	2.95E+09	2.4E+09	1.65E+09	1.37E+09	1.89E+09	2.42E+09
tr|Q9RUB	tr|Q9RUB	1.28E+09	1.26E+09	1.13E+09	1.18E+09	1.74E+09	1.15E+09	1.13E+09
tr|Q9RUB	tr|Q9RUB	3.59E+09	3.27E+09	3.3E+09	3.66E+09	3.25E+09	3.78E+09	2.81E+09
tr|Q9RUB	tr|Q9RUB	41158000	49548000					46725000
tr|Q9RUB	tr|Q9RUB		83749000	71199000			89550000	97777000
tr|Q9RUB	tr|Q9RUB	1.19E+08	1.38E+08	1.27E+08	1.59E+08	1.45E+08	1.67E+08	
tr|Q9RUB	tr|Q9RUB	50567000	64435000	81984000	84657000	75011000	58323000	57453000
tr|Q9RUC	tr|Q9RUC	1.32E+08	1.29E+08	1.37E+08	1.6E+08	1.47E+08	1.35E+08	1.15E+08
tr|Q9RUC	tr|Q9RUC	71938000	1.18E+08	1.15E+08	24179000			
tr|Q9RUC	tr|Q9RUC	71095000						
tr|Q9RUC	tr|Q9RUC	3.67E+08						
tr|Q9RUC	tr|Q9RUC							
tr|Q9RUC	tr|Q9RUC	6.24E+08	7.84E+08	5.93E+08	7.53E+08	7.23E+08	8.68E+08	7.31E+08
tr|Q9RUD	tr|Q9RUD	1.36E+09	1.35E+09	1.5E+09	9.27E+08	1.03E+09	1.16E+09	8.03E+08
tr|Q9RUD	tr|Q9RUD	7.92E+08	6.28E+08	6.56E+08	9.84E+08	8.73E+08	8.07E+08	6.55E+08
tr|Q9RUD	tr|Q9RUD	1.33E+09	1.24E+09	9.53E+08	8.91E+08	8.64E+08	8.92E+08	1.38E+09
tr|Q9RUD	tr|Q9RUD							
tr|Q9RUD	tr|Q9RUD	7.73E+08	7.64E+08	1.12E+09	8.34E+08	8.68E+08	6.68E+08	6.69E+08
tr|Q9RUE0	tr|Q9RUE0	2.92E+09	2.84E+09	3.38E+09	5.08E+09	4.91E+09	4.88E+09	2.03E+09
tr|Q9RUE4	tr|Q9RUE4		31423000					
tr|Q9RUE7	tr|Q9RUE7	1.09E+08		61240000				
tr|Q9RUF4	tr|Q9RUF4	7.35E+08	5.59E+08	5.4E+08	9.72E+08	9.19E+08	9.8E+08	3.43E+08
tr|Q9RUF6	tr|Q9RUF6				1.97E+08	1.44E+08	1.76E+08	
tr|Q9RUF8	tr|Q9RUF8	1.56E+09	1.93E+09	1.5E+09	1.85E+09	1.95E+09	1.81E+09	1.72E+09
tr|Q9RUF9	tr|Q9RUF9	7.45E+08	1.01E+09	8.3E+08	5.58E+08	8.44E+08	6.3E+08	1.08E+09
tr|Q9RUG	tr|Q9RUG	9.27E+08	6.54E+08	6.82E+08	3.07E+08	3.24E+08	2.56E+08	5.16E+08
tr|Q9RUG	tr|Q9RUG	6.62E+08	6.37E+08	6.4E+08	7.37E+08	7E+08	6.36E+08	8.25E+08
tr|Q9RUG	tr|Q9RUG							
tr|Q9RUG	tr|Q9RUG			21280000				
tr|Q9RUG	tr|Q9RUG	6.45E+08	7.28E+08	6.06E+08	1.18E+09	9.91E+08	1.05E+09	7.5E+08
tr|Q9RUH	tr|Q9RUH	1.28E+08	90801000	68339000	54314000		55625000	1.24E+08
tr|Q9RUH	tr|Q9RUH	1.11E+08	1.25E+08	1.35E+08	1.77E+08	1.31E+08	1.53E+08	1.11E+08
tr|Q9RUH	tr|Q9RUH	96348000	97451000	1.02E+08	64192000	68091000		1.21E+08
tr|Q9RUH	tr|Q9RUH	3.61E+08	3.81E+08	3.84E+08	3.49E+08	4.15E+08	3.15E+08	4.52E+08
tr|Q9RUH	tr|Q9RUH	1.07E+08	1.2E+08	93856000		1.83E+08		1.46E+08
tr|Q9RUI0	tr|Q9RUI0	1.95E+08	2.33E+08	3.27E+08	2.15E+08	2.11E+08	2.58E+08	1.91E+08
tr|Q9RUI3	tr|Q9RUI3	43874000	38309000	54637000				58460000
tr|Q9RUI4	tr|Q9RUI4	74170000	66555000			60211000	64715000	60083000
tr|Q9RUI5	tr|Q9RUI5	3.35E+08	3.63E+08	2.73E+08	3.57E+08	2.99E+08	2.93E+08	3.67E+08
tr|Q9RUI7	tr|Q9RUI7							
tr|Q9RUI9	tr|Q9RUI9	2.89E+08	2.82E+08	2.76E+08	2.65E+08	2.6E+08	2.36E+08	2.4E+08

<table>
<tr><td>tr|Q9RUJ1</td><td>tr|Q9RUJ1</td><td>3.63E+08</td><td>3.24E+08</td><td>3.31E+08</td><td>5.12E+08</td><td>5.09E+08</td><td>5.18E+08</td><td>3.01E+08</td></tr>
<tr><td>tr|Q9RUJ2</td><td>tr|Q9RUJ2</td><td>1.95E+08</td><td>2.48E+08</td><td>2.53E+08</td><td>3.16E+08</td><td>3.34E+08</td><td>2.1E+08</td><td>2.5E+08</td></tr>
<tr><td>tr|Q9RUJ3</td><td>tr|Q9RUJ3</td><td></td><td></td><td>25421000</td><td></td><td></td><td>43104000</td><td>54588000</td></tr>
<tr><td>tr|Q9RUJ4</td><td>tr|Q9RUJ4</td><td>2.37E+08</td><td>2.16E+08</td><td>1.69E+08</td><td>1.42E+08</td><td>1.8E+08</td><td>1.7E+08</td><td>1.94E+08</td></tr>
<tr><td>tr|Q9RUJ5</td><td>tr|Q9RUJ5</td><td></td><td></td><td></td><td></td><td></td><td></td><td></td></tr>
<tr><td>tr|Q9RUJ6</td><td>tr|Q9RUJ6</td><td>2.39E+08</td><td>2.37E+08</td><td>1.9E+08</td><td>2.82E+08</td><td>2.8E+08</td><td>2.6E+08</td><td>2.79E+08</td></tr>
<tr><td>tr|Q9RUJ8</td><td>tr|Q9RUJ8</td><td>98560000</td><td>1.26E+08</td><td>1.58E+08</td><td></td><td></td><td></td><td>83454000</td></tr>
<tr><td>tr|Q9RUJ9</td><td>tr|Q9RUJ9</td><td></td><td></td><td></td><td></td><td></td><td></td><td></td></tr>
<tr><td>tr|Q9RUK(</td><td>tr|Q9RUK(</td><td>2.12E+08</td><td>1.85E+08</td><td>1.83E+08</td><td>1.23E+08</td><td>1.63E+08</td><td>1.57E+08</td><td>1.97E+08</td></tr>
<tr><td>tr|Q9RUK'</td><td>tr|Q9RUK'</td><td></td><td></td><td></td><td></td><td></td><td></td><td></td></tr>
<tr><td>tr|Q9RUK:</td><td>tr|Q9RUK:</td><td>6.19E+08</td><td>5.47E+08</td><td></td><td>6.75E+08</td><td>6.38E+08</td><td>4.79E+08</td><td></td></tr>
<tr><td>tr|Q9RUK!</td><td>tr|Q9RUK!</td><td></td><td></td><td></td><td></td><td>11904000</td><td></td><td></td></tr>
<tr><td>tr|Q9RUK</td><td>tr|Q9RUK</td><td>3.23E+08</td><td>3.76E+08</td><td>3.32E+08</td><td>3.6E+08</td><td>2.64E+08</td><td>3.17E+08</td><td>3.74E+08</td></tr>
<tr><td>tr|Q9RUK{</td><td>tr|Q9RUK{</td><td>4.6E+08</td><td>5.3E+08</td><td>4.27E+08</td><td>2.55E+08</td><td>2.91E+08</td><td>2.76E+08</td><td>5.87E+08</td></tr>
<tr><td>tr|Q9RUK(</td><td>tr|Q9RUK(</td><td>3.77E+08</td><td>3.38E+08</td><td>3.33E+08</td><td>6.43E+08</td><td>5.77E+08</td><td>6.89E+08</td><td>2.15E+08</td></tr>
<tr><td>tr|Q9RUL(</td><td>tr|Q9RUL(</td><td>4.16E+08</td><td>3.64E+08</td><td>3.05E+08</td><td>4.27E+08</td><td>3.51E+08</td><td>3.56E+08</td><td>4.06E+08</td></tr>
<tr><td>tr|Q9RUL'</td><td>tr|Q9RUL'</td><td>32166000</td><td></td><td></td><td>37953000</td><td>33563000</td><td></td><td></td></tr>
<tr><td>tr|Q9RUL:</td><td>tr|Q9RUL:</td><td>4.9E+08</td><td>5.81E+08</td><td>3.47E+08</td><td></td><td></td><td></td><td>5.89E+08</td></tr>
<tr><td>tr|Q9RUL‹</td><td>tr|Q9RUL‹</td><td>60071000</td><td>66881000</td><td>82916000</td><td>1.6E+08</td><td>1.34E+08</td><td>1.05E+08</td><td>1.11E+08</td></tr>
<tr><td>tr|Q9RUL!</td><td>tr|Q9RUL!</td><td>1.14E+08</td><td>1.11E+08</td><td>1.21E+08</td><td></td><td>1.26E+08</td><td>1.21E+08</td><td>1.53E+08</td></tr>
<tr><td>tr|Q9RUL(</td><td>tr|Q9RUL(</td><td>6.29E+08</td><td></td><td>5.01E+08</td><td>4.5E+08</td><td>5.9E+08</td><td>6.41E+08</td><td></td></tr>
<tr><td>tr|Q9RUM</td><td>tr|Q9RUM</td><td>5.28E+08</td><td>4.95E+08</td><td>2.97E+08</td><td></td><td></td><td></td><td></td></tr>
<tr><td>tr|Q9RUM</td><td>tr|Q9RUM</td><td>6.63E+08</td><td>5.67E+08</td><td>5.56E+08</td><td>5.38E+08</td><td>7.98E+08</td><td></td><td>7.67E+08</td></tr>
<tr><td>tr|Q9RUM</td><td>tr|Q9RUM</td><td></td><td></td><td></td><td></td><td></td><td></td><td></td></tr>
<tr><td>tr|Q9RUM</td><td>tr|Q9RUM</td><td>99158000</td><td>1.34E+08</td><td>1.21E+08</td><td>1.29E+08</td><td>92568000</td><td>84085000</td><td>1.43E+08</td></tr>
<tr><td>tr|Q9RUM</td><td>tr|Q9RUM</td><td></td><td></td><td></td><td></td><td></td><td></td><td></td></tr>
<tr><td>tr|Q9RUN</td><td>tr|Q9RUN</td><td></td><td></td><td></td><td></td><td></td><td></td><td></td></tr>
<tr><td>tr|Q9RUN</td><td>tr|Q9RUN</td><td>45819000</td><td>42622000</td><td>48934000</td><td>71478000</td><td>96869000</td><td>92067000</td><td>60601000</td></tr>
<tr><td>tr|Q9RUN</td><td>tr|Q9RUN</td><td></td><td></td><td></td><td></td><td></td><td></td><td></td></tr>
<tr><td>tr|Q9RUN</td><td>tr|Q9RUN</td><td></td><td></td><td></td><td></td><td></td><td></td><td></td></tr>
<tr><td>tr|Q9RUN</td><td>tr|Q9RUN</td><td>91977000</td><td>89618000</td><td>87263000</td><td>1.47E+08</td><td>1.35E+08</td><td>1.3E+08</td><td>92152000</td></tr>
<tr><td>tr|Q9RUP(</td><td>tr|Q9RUP(</td><td>2.09E+08</td><td>5.88E+08</td><td>3.96E+08</td><td>4.28E+08</td><td>5.26E+08</td><td>4.47E+08</td><td>6.38E+08</td></tr>
<tr><td>tr|Q9RUP'</td><td>tr|Q9RUP'</td><td>1.54E+10</td><td>1.56E+10</td><td>1.7E+10</td><td>1.6E+10</td><td>1.28E+10</td><td>1.47E+10</td><td>1.04E+10</td></tr>
<tr><td>tr|Q9RUP(</td><td>tr|Q9RUP(</td><td></td><td></td><td></td><td></td><td></td><td></td><td></td></tr>
<tr><td>tr|Q9RUQ</td><td>tr|Q9RUQ</td><td>98455000</td><td>75961000</td><td>67333000</td><td></td><td></td><td></td><td>1.77E+08</td></tr>
<tr><td>tr|Q9RUQ</td><td>tr|Q9RUQ</td><td>8.27E+08</td><td>7.86E+08</td><td></td><td>1.29E+09</td><td>1.37E+09</td><td>1.29E+09</td><td>6.48E+08</td></tr>
<tr><td>tr|Q9RUQ</td><td>tr|Q9RUQ</td><td>2.31E+08</td><td>2.71E+08</td><td>2.98E+08</td><td>1.62E+08</td><td>1.9E+08</td><td>1.27E+08</td><td>3.73E+08</td></tr>
<tr><td>tr|Q9RUQ</td><td>tr|Q9RUQ</td><td>73363000</td><td>59458000</td><td>59059000</td><td></td><td>20881000</td><td>24827000</td><td>57343000</td></tr>
<tr><td>tr|Q9RUR:</td><td>tr|Q9RUR:</td><td>2.78E+08</td><td>1.94E+08</td><td>1.89E+08</td><td>1.79E+08</td><td>1.95E+08</td><td>1.77E+08</td><td>1.21E+08</td></tr>
<tr><td>tr|Q9RUR:</td><td>tr|Q9RUR:</td><td></td><td></td><td></td><td></td><td></td><td></td><td></td></tr>
<tr><td>tr|Q9RUR‹</td><td>tr|Q9RUR‹</td><td>3.94E+08</td><td>4.5E+08</td><td>4.45E+08</td><td>2.45E+08</td><td>2.8E+08</td><td>3.34E+08</td><td>1.74E+08</td></tr>
<tr><td>tr|Q9RUR(</td><td>tr|Q9RUR(</td><td>5.33E+08</td><td>6.13E+08</td><td>5.8E+08</td><td>5.1E+08</td><td>5.39E+08</td><td>5.12E+08</td><td>4.94E+08</td></tr>
<tr><td>tr|Q9RUR</td><td>tr|Q9RUR</td><td>1.34E+09</td><td>1.11E+09</td><td>1.1E+09</td><td>1.5E+09</td><td>1.23E+09</td><td>1.5E+09</td><td>1.04E+09</td></tr>
<tr><td>tr|Q9RUR(</td><td>tr|Q9RUR(</td><td>5.58E+08</td><td>5.02E+08</td><td>4.37E+08</td><td>4.25E+08</td><td>4.61E+08</td><td>4.73E+08</td><td>6.25E+08</td></tr>
<tr><td>tr|Q9RUS'</td><td>tr|Q9RUS'</td><td>3.27E+08</td><td>2.82E+08</td><td>3.12E+08</td><td>3.07E+08</td><td>3.24E+08</td><td>3.78E+08</td><td>4.63E+08</td></tr>
<tr><td>tr|Q9RUS‹</td><td>tr|Q9RUS‹</td><td></td><td></td><td>11155000</td><td>41401000</td><td>43410000</td><td>54188000</td><td>33066000</td></tr>
<tr><td>tr|Q9RUS(</td><td>tr|Q9RUS(</td><td>3.56E+08</td><td>3.84E+08</td><td>2.8E+08</td><td>3.32E+08</td><td>3.19E+08</td><td>3.78E+08</td><td>3.13E+08</td></tr>
<tr><td>tr|Q9RUS:</td><td>tr|Q9RUS:</td><td>54774000</td><td>58274000</td><td>47558000</td><td></td><td>68445000</td><td>62779000</td><td>71146000</td></tr>
<tr><td>tr|Q9RUS!</td><td>tr|Q9RUS!</td><td></td><td></td><td></td><td></td><td></td><td></td><td></td></tr>
<tr><td>tr|Q9RUT(</td><td>tr|Q9RUT(</td><td>12405000</td><td>16195000</td><td>14536000</td><td>43988000</td><td>43114000</td><td>30408000</td><td>33015000</td></tr>
<tr><td>tr|Q9RUT'</td><td>tr|Q9RUT'</td><td>1.88E+08</td><td>1.83E+08</td><td>1.75E+08</td><td>1.32E+08</td><td>1.35E+08</td><td>1.43E+08</td><td>1.8E+08</td></tr>
</table>

tr|Q9RUT4	tr|Q9RUT4		87420000	1.59E+08	86979000	68210000	74713000	1.95E+08
tr|Q9RUT5	tr|Q9RUT5							
tr|Q9RUT9	tr|Q9RUT9	58902000	42757000	39709000	56410000	51806000	59867000	45835000
tr|Q9RUU	tr|Q9RUU	5.2E+08	5.42E+08	4.68E+08	4.14E+08	2.74E+08	4.29E+08	4.83E+08
tr|Q9RUU	tr|Q9RUU	1.6E+09	5.31E+08	1.77E+09	6.71E+08	4.27E+08	5.24E+08	1.3E+09
tr|Q9RUU	tr|Q9RUU	52063000	57260000	51013000	1.47E+08	1.29E+08	1.16E+08	94140000
tr|Q9RUU	tr|Q9RUU	4.42E+08	4.66E+08	4.33E+08	4.12E+08	4E+08	3.95E+08	5.5E+08
tr|Q9RUV4	tr|Q9RUV4	91196000	1.02E+08	1.17E+08	87311000	92543000	1.73E+08	49247000
tr|Q9RUW	tr|Q9RUW	1.33E+08	1.68E+08	1.71E+08		96904000		2.08E+08
tr|Q9RUW	tr|Q9RUW	86435000	76260000	60559000	1.13E+08	98086000	1.02E+08	
tr|Q9RUW	tr|Q9RUW				8232000	9715000	8477500	
tr|Q9RUX0	tr|Q9RUX0		86128000		1.06E+08		97936000	
tr|Q9RUX1	tr|Q9RUX1							
tr|Q9RUX2	tr|Q9RUX2	59277000				75635000	63628000	48836000
tr|Q9RUX3	tr|Q9RUX3							
tr|Q9RUX4	tr|Q9RUX4							
tr|Q9RUX5	tr|Q9RUX5	1.42E+08	2.14E+08	1.55E+08	1.5E+08	1.61E+08	1.46E+08	1.31E+08
tr|Q9RUX7	tr|Q9RUX7				37333000	33518000	44174000	
tr|Q9RUX8	tr|Q9RUX8	1.04E+09	1.2E+09	1.01E+09	1.88E+09	1.45E+09	1.54E+09	1.02E+09
tr|Q9RUX9	tr|Q9RUX9	3.58E+08	1.81E+08	2E+08				
tr|Q9RUY1	tr|Q9RUY1				1.14E+08	96754000	1.95E+08	
tr|Q9RUY2	tr|Q9RUY2	4.59E+09	4.76E+09	4.37E+09	2.98E+09	3.51E+09	3.05E+09	6.27E+09
tr|Q9RUY4	tr|Q9RUY4	1.4E+08	1.35E+08	1.9E+08	1.72E+08	2.07E+08	1.92E+08	1.01E+08
tr|Q9RUY5	tr|Q9RUY5	1.05E+09	7.6E+08	9.74E+08	1.31E+09	9.93E+08	1.09E+09	
tr|Q9RUY7	tr|Q9RUY7							
tr|Q9RUY8	tr|Q9RUY8	8.79E+08	1.02E+09	9.43E+08	6.22E+08	9.71E+08	3.19E+08	9.19E+08
tr|Q9RUZ1	tr|Q9RUZ1	1.9E+09	1.67E+09	1.21E+09	1.48E+09	1.64E+09	2.06E+09	1.53E+09
tr|Q9RUZ2	tr|Q9RUZ2							
tr|Q9RUZ5	tr|Q9RUZ5	1.96E+08	2.31E+08	2E+08	2.34E+08	1.96E+08	2.07E+08	2.06E+08
tr|Q9RUZ6	tr|Q9RUZ6		26618000			30013000		
tr|Q9RUZ7	tr|Q9RUZ7		30632000	34984000				
tr|Q9RUZ8	tr|Q9RUZ8							
tr|Q9RUZ9	tr|Q9RUZ9	96417000	74020000	62365000	82780000	80001000	84248000	84031000
tr|Q9RV00	tr|Q9RV00			94237000				
tr|Q9RV03	tr|Q9RV03	14683000		14171000	48734000	34110000	39209000	
tr|Q9RV05	tr|Q9RV05		95320000	78273000	1.17E+08	1.36E+08	1.43E+08	
tr|Q9RV06	tr|Q9RV06	1.23E+08			78793000	76202000	73031000	1.27E+08
tr|Q9RV10	tr|Q9RV10							
tr|Q9RV11	tr|Q9RV11	99721000	1.12E+08	72427000	1.18E+08	87872000	85566000	3.1E+08
tr|Q9RV13	tr|Q9RV13	69799000						
tr|Q9RV14	tr|Q9RV14							
tr|Q9RV15	tr|Q9RV15	3.84E+08	3.51E+08	2.7E+08	2.77E+08	2.54E+08	2.87E+08	2.79E+08
tr|Q9RV17	tr|Q9RV17	1.96E+08	1.53E+08	1.61E+08	1.9E+08	1.51E+08	1.66E+08	1.58E+08
tr|Q9RV18	tr|Q9RV18	3.24E+08	3.19E+08	3.01E+08	2.36E+08	2.02E+08	2.29E+08	2.48E+08
tr|Q9RV21	tr|Q9RV21		63853000	56525000	72407000	78894000	71750000	
tr|Q9RV26	tr|Q9RV26							
tr|Q9RV27	tr|Q9RV27	3.23E+08	2.78E+08	2.85E+08	2.01E+08	1.15E+08	1.56E+08	2.27E+08
tr|Q9RV29	tr|Q9RV29				44230000			
tr|Q9RV30	tr|Q9RV30							
tr|Q9RV31	tr|Q9RV31	1.36E+08	1.18E+08	1.93E+08	1.22E+08	2.1E+08	1.73E+08	73193000
tr|Q9RV32	tr|Q9RV32	3.12E+08	2.81E+08	2.7E+08	3.17E+08	3.03E+08	2.78E+08	2.78E+08

tr|Q9RV36	tr|Q9RV36	4.73E+08	5.42E+08	6.67E+08	3.26E+08	3.34E+08	3.8E+08	2.74E+08
tr|Q9RV38	tr|Q9RV38							
tr|Q9RV41	tr|Q9RV41	88493000	81218000	66253000	1.76E+08	1.72E+08	2.38E+08	57728000
tr|Q9RV45	tr|Q9RV45	1.18E+10	1E+10	1.33E+10	1.12E+10	1.24E+10	1.12E+10	6.25E+09
tr|Q9RV46	tr|Q9RV46	68540000	98494000	89954000	1.09E+08	97960000	1.02E+08	89283000
tr|Q9RV47	tr|Q9RV47		2.2E+08	2.43E+08	1.71E+08		2.2E+08	
tr|Q9RV48	tr|Q9RV48			1.24E+09				
tr|Q9RV50	tr|Q9RV50	5.36E+08	4.19E+08	4.68E+08		1.64E+08	1.95E+08	5.74E+08
tr|Q9RV52	tr|Q9RV52	1.43E+08	1.37E+08	99643000	1.05E+08	1.01E+08	1.12E+08	1.31E+08
tr|Q9RV53	tr|Q9RV53							
tr|Q9RV55	tr|Q9RV55	1.69E+08	2.76E+08	1.54E+08	6.01E+08	4.91E+08	5.82E+08	
tr|Q9RV56	tr|Q9RV56	45566000	57226000	46555000	65893000	1.16E+08	56540000	67036000
tr|Q9RV57	tr|Q9RV57		1.19E+08	1.42E+08	1.04E+08	1.2E+08	1.35E+08	
tr|Q9RV59	tr|Q9RV59	2.04E+08	1.98E+08	1.74E+08	2.57E+08	2.84E+08	3E+08	2.34E+08
tr|Q9RV63	tr|Q9RV63							
tr|Q9RV67	tr|Q9RV67							
tr|Q9RV68	tr|Q9RV68	2.63E+08	2.62E+08	2.48E+08	3.12E+08	2.86E+08	3.03E+08	3.16E+08
tr|Q9RV70	tr|Q9RV70	2.17E+08	2.06E+08	2.31E+08	7.55E+08	6.87E+08	7.21E+08	
tr|Q9RV73	tr|Q9RV73							
tr|Q9RV74	tr|Q9RV74	41480000	31108000	32287000				
tr|Q9RV75	tr|Q9RV75	1.46E+08	1.24E+08	89043000	1.3E+08	1.19E+08	1.17E+08	1.39E+08
tr|Q9RV78	tr|Q9RV78	5.96E+08	9.24E+08	7.62E+08	5.34E+08	7.09E+08	3.49E+08	6.96E+08
tr|Q9RV82	tr|Q9RV82	1.08E+08	90884000	96905000	75827000		57743000	
tr|Q9RV83	tr|Q9RV83	4.14E+09	4.21E+09	3.68E+09	1.71E+09	1.63E+09	2.17E+09	2.06E+09
tr|Q9RV86	tr|Q9RV86							
tr|Q9RV88	tr|Q9RV88				4.45E+08	3.85E+08		
tr|Q9RV90	tr|Q9RV90	27894000		35457000	54212000	65414000	53291000	
tr|Q9RV92	tr|Q9RV92	59241000		57563000				
tr|Q9RV94	tr|Q9RV94							
tr|Q9RV99	tr|Q9RV99	2.65E+08	2.08E+08	2.03E+08	3.72E+08	3.65E+08	3.16E+08	
tr|Q9RVA2	tr|Q9RVA2	3.05E+08	3.08E+08	3.12E+08	1.99E+08	1.94E+08	2.31E+08	2.99E+08
tr|Q9RVA3	tr|Q9RVA3	34841000	59515000	65074000	4.5E+08	1.83E+08	1E+08	58461000
tr|Q9RVA4	tr|Q9RVA4							
tr|Q9RVA5	tr|Q9RVA5	5.3E+09	5.53E+09	5.12E+09	2.22E+09	2.19E+09	2.36E+09	6.5E+09
tr|Q9RVA7	tr|Q9RVA7	2.8E+08	2.93E+08	2.49E+08	2.27E+08	2.56E+08	2.77E+08	2.85E+08
tr|Q9RVB0	tr|Q9RVB0	1.31E+08			76227000	83755000	93434000	1.61E+08
tr|Q9RVB1	tr|Q9RVB1							
tr|Q9RVB2	tr|Q9RVB2	5.01E+08	4.56E+08	4.24E+08	6.46E+08	5.9E+08	6.26E+08	4.63E+08
tr|Q9RVB3	tr|Q9RVB3	68205000	53154000	51385000				1.17E+08
tr|Q9RVB4	tr|Q9RVB4	3.25E+08	2.4E+08	2.55E+08	4.41E+08	4.04E+08	4.41E+08	2.25E+08
tr|Q9RVB5	tr|Q9RVB5	1.71E+09	2.11E+09	1.52E+09	1.13E+09	1.07E+09	1.13E+09	3.63E+09
tr|Q9RVB8	tr|Q9RVB8							
tr|Q9RVC5	tr|Q9RVC5							
tr|Q9RVC6	tr|Q9RVC6	57847000	76708000	45341000			42097000	90257000
tr|Q9RVD1	tr|Q9RVD1							
tr|Q9RVD2	tr|Q9RVD2	47159000		27186000				
tr|Q9RVD7	tr|Q9RVD7							
tr|Q9RVD8	tr|Q9RVD8							
tr|Q9RVD9	tr|Q9RVD9							
tr|Q9RVE1	tr|Q9RVE1							
tr|Q9RVE5	tr|Q9RVE5	75337000	93482000	95757000	1.45E+08	1.4E+08	1.25E+08	88589000

tr|Q9RVE9	tr|Q9RVE9							48563000
tr|Q9RVF1	tr|Q9RVF1							
tr|Q9RVF3	tr|Q9RVF3							
tr|Q9RVF4	tr|Q9RVF4	24721000		27340000				
tr|Q9RVF7	tr|Q9RVF7	30749000	22095000	41175000	81933000	29577000	35855000	38538000
tr|Q9RVF8	tr|Q9RVF8	9.54E+08	6.22E+08	4.78E+08	5.27E+08	6.01E+08	5.62E+08	9.45E+08
tr|Q9RVG2	tr|Q9RVG2		1271300	1286300		1458600	1347400	1729100
tr|Q9RVG4	tr|Q9RVG4	1.08E+08	1.27E+08	1.35E+08	66031000	51392000	53377000	1.1E+08
tr|Q9RVG6	tr|Q9RVG6	3.3E+09	3.12E+09	3.5E+09	5.69E+09	5.81E+09	5.18E+09	2.08E+09
tr|Q9RVG7	tr|Q9RVG7	2.01E+08	2.12E+08	1.92E+08	3.6E+08	2.7E+08	2.74E+08	3.35E+08
tr|Q9RVG8	tr|Q9RVG8	3.12E+09	2.87E+09	2.54E+09	2.41E+09	2.46E+09	2.66E+09	3.22E+09
tr|Q9RVG9	tr|Q9RVG9							
tr|Q9RVH0	tr|Q9RVH0							
tr|Q9RVH2	tr|Q9RVH2							
tr|Q9RVH6	tr|Q9RVH6							
tr|Q9RVH8	tr|Q9RVH8			54263000	73234000	74046000	63053000	69718000
tr|Q9RVI7	tr|Q9RVI7							
tr|Q9RVI8	tr|Q9RVI8		58031000	58547000				50684000
tr|Q9RVJ0	tr|Q9RVJ0	4.06E+09	4.4E+09	5.53E+09	2.15E+09	2.56E+09	2.47E+09	2.08E+09
tr|Q9RVJ2	tr|Q9RVJ2		3.38E+08	2.47E+08		3.25E+08		1.22E+08
tr|Q9RVJ3	tr|Q9RVJ3	1.52E+08	1.67E+08	1.54E+08	74318000	1.06E+08	1.06E+08	1.89E+08
tr|Q9RVJ4	tr|Q9RVJ4	2.95E+08	3.16E+08	2.68E+08	2.83E+08	3.34E+08		
tr|Q9RVJ7	tr|Q9RVJ7	1.03E+08	1.08E+08	89960000	86582000	1.07E+08	1.21E+08	95935000
tr|Q9RVJ8	tr|Q9RVJ8							
tr|Q9RVK1	tr|Q9RVK1	1.69E+08	2.3E+08	1.62E+08	3.34E+08	2.81E+08	3.04E+08	1.86E+08
tr|Q9RVK5	tr|Q9RVK5	2.25E+08	1.74E+08	1.53E+08	2.1E+08	2.16E+08	2.3E+08	1.9E+08
tr|Q9RVK6	tr|Q9RVK6	1.36E+08	1.24E+08	2.12E+08	1.43E+08	1.48E+08	1.68E+08	1.79E+08
tr|Q9RVK7	tr|Q9RVK7							
tr|Q9RVK8	tr|Q9RVK8	5.11E+09	5.27E+09	4.58E+09	2.55E+09	3.08E+09	2.73E+09	5.93E+09
tr|Q9RVK9	tr|Q9RVK9	2.27E+08	2.07E+08	1.74E+08	1.39E+08	1.23E+08	1.34E+08	2.4E+08
tr|Q9RVL0	tr|Q9RVL0	1.72E+08	1.93E+08	1.88E+08	2.09E+08	1.83E+08	2.08E+08	2E+08
tr|Q9RVL2	tr|Q9RVL2							
tr|Q9RVL5	tr|Q9RVL5	3.1E+08	3.28E+08	2.86E+08				3.45E+08
tr|Q9RVL6	tr|Q9RVL6	4.96E+08	4.02E+08	4.44E+08	4.73E+08	3.16E+08	3.02E+08	8.28E+08
tr|Q9RVL9	tr|Q9RVL9		99576000	1.46E+08	1.17E+08	93918000	87817000	1.06E+08
tr|Q9RVM	tr|Q9RVM	80983000	1.29E+08	84442000	92153000	91702000	91766000	1.14E+08
tr|Q9RVM	tr|Q9RVM	81488000		69422000	1.06E+08	1.16E+08	1.12E+08	
tr|Q9RVM	tr|Q9RVM	8.84E+08	1.14E+09	1.05E+09	8.28E+08	9.17E+08	7.97E+08	9.24E+08
tr|Q9RVN	tr|Q9RVN							
tr|Q9RVN	tr|Q9RVN							
tr|Q9RVN	tr|Q9RVN	1.39E+08	2.21E+08	2.19E+08	3.31E+08	3.44E+08	2.67E+08	4.1E+08
tr|Q9RVN	tr|Q9RVN	1.51E+08	1.51E+08	1.31E+08		76122000		1.51E+08
tr|Q9RVN	tr|Q9RVN	2.73E+08	2.55E+08	3.04E+08	3.93E+08	2.9E+08	2.83E+08	2.7E+08
tr|Q9RVN	tr|Q9RVN		43644000		1.1E+08	1.48E+08	74648000	45719000
tr|Q9RVN	tr|Q9RVN	4.68E+09	3.19E+09	3.87E+09	2.26E+09	2.26E+09	2.93E+09	2.24E+09
tr|Q9RVN	tr|Q9RVN	86974000		55926000		28919000		2.95E+08
tr|Q9RVP0	tr|Q9RVP0	3.55E+08	2.75E+08	2.57E+08	2.05E+08	2.37E+08	2.62E+08	1.69E+08
tr|Q9RVP1	tr|Q9RVP1	2.65E+09	2.8E+09	2.85E+09	2.93E+09	2.61E+09	2.6E+09	1.96E+09
tr|Q9RVP3	tr|Q9RVP3							
tr|Q9RVP5	tr|Q9RVP5	2.55E+09	1.61E+09	2.01E+09	2.28E+09	1.64E+09	2.07E+09	2.79E+09
tr|Q9RVP9	tr|Q9RVP9			23867000				

tr|Q9RVQ	tr|Q9RVQ	3.39E+09	3.23E+09	3.26E+09	9.69E+08	8.51E+08	1.22E+09	3.54E+09
tr|Q9RVQ	tr|Q9RVQ	6.91E+08	6.85E+08	7.49E+08	5.04E+08	6E+08	6.55E+08	6.27E+08
tr|Q9RVQ	tr|Q9RVQ	8.93E+08	9.12E+08	1.29E+09	9.87E+08	8.61E+08	1.09E+09	6.21E+08
tr|Q9RVQ	tr|Q9RVQ	1.09E+09	9.5E+08	1.02E+09	1.08E+09	1.41E+09	1.22E+09	7.98E+08
tr|Q9RVQ	tr|Q9RVQ							
tr|Q9RVQ	tr|Q9RVQ	3.68E+08	3.14E+08	3.79E+08	2.69E+08	2.36E+08	1.91E+08	2.38E+08
tr|Q9RVQ	tr|Q9RVQ							1.34E+08
tr|Q9RVQ	tr|Q9RVQ							
tr|Q9RVQ	tr|Q9RVQ	2.35E+08	1.09E+08	1.4E+08	1.82E+08	87622000	1.07E+08	2.39E+08
tr|Q9RVR1	tr|Q9RVR1							
tr|Q9RVR2	tr|Q9RVR2	6.11E+08	7.01E+08	8.31E+08	2.12E+09	1.73E+09	1.51E+09	6.63E+08
tr|Q9RVR3	tr|Q9RVR3							
tr|Q9RVR4	tr|Q9RVR4							
tr|Q9RVR5	tr|Q9RVR5	5.54E+08	5.08E+08	4.56E+08	6.7E+08	6.3E+08	6.6E+08	5.53E+08
tr|Q9RVR7	tr|Q9RVR7							
tr|Q9RVS0	tr|Q9RVS0	3.95E+09	3.31E+09	3.37E+09	2.75E+09	2.38E+09	2.6E+09	3.86E+09
tr|Q9RVS1	tr|Q9RVS1	1.62E+09	2.07E+09	1.96E+09	1.69E+09	1.76E+09	1.52E+09	2.28E+09
tr|Q9RVS2	tr|Q9RVS2	3.25E+09	2.84E+09	2.77E+09	2.64E+09	2.34E+09	2.7E+09	3.02E+09
tr|Q9RVS4	tr|Q9RVS4							
tr|Q9RVS5	tr|Q9RVS5	2.68E+08	3.33E+08	2.36E+08	3.5E+08	3.33E+08	2.9E+08	2.8E+08
tr|Q9RVS6	tr|Q9RVS6	95349000	77194000	97104000	69936000	62674000	58123000	60534000
tr|Q9RVS8	tr|Q9RVS8	1.55E+10	2.22E+10	1.93E+10	1.7E+10	1.77E+10	1.7E+10	1.72E+10
tr|Q9RVS9	tr|Q9RVS9							
tr|Q9RVT2	tr|Q9RVT2		87630000	79891000	61868000	43493000	55054000	
tr|Q9RVT4	tr|Q9RVT4	2.28E+08	2.52E+08	2.12E+08		2.62E+08		3.21E+08
tr|Q9RVT5	tr|Q9RVT5	3.5E+08	3.22E+08	2.99E+08	3.82E+08	3.39E+08	3.49E+08	2.45E+08
tr|Q9RVT8	tr|Q9RVT8	1.99E+08	2.13E+08	1.58E+08	1.77E+08	1.67E+08	1.72E+08	2.59E+08
tr|Q9RVT9	tr|Q9RVT9	49347000	52744000	39127000	69638000	60249000	71232000	34254000
tr|Q9RVU0	tr|Q9RVU0	3.03E+08	3.67E+08	2.63E+08	2.35E+08	2.99E+08	2.84E+08	7.65E+08
tr|Q9RVU2	tr|Q9RVU2	3.69E+08	3.82E+08	4.25E+08	4.69E+08	4.39E+08	4.36E+08	3.76E+08
tr|Q9RVU4	tr|Q9RVU4	77390000	87021000	44544000	3.03E+08	1.64E+08	2.48E+08	1.29E+08
tr|Q9RVU5	tr|Q9RVU5							
tr|Q9RVU8	tr|Q9RVU8	6.48E+08	7.3E+08	9.13E+08	3.49E+08	4.31E+08	4.18E+08	4.89E+08
tr|Q9RVV0	tr|Q9RVV0	68542000	63957000	63237000	51470000	55461000	43608000	65387000
tr|Q9RVV1	tr|Q9RVV1	3.85E+08	2.59E+08	2.11E+08		1.85E+08		2.89E+08
tr|Q9RVV3	tr|Q9RVV3							
tr|Q9RVV4	tr|Q9RVV4							
tr|Q9RVV5	tr|Q9RVV5							
tr|Q9RVV7	tr|Q9RVV7	1.99E+08	2.42E+08			2.04E+08	1.56E+08	2.81E+08
tr|Q9RVW	tr|Q9RVW	51779000	33464000	57635000	52695000	55112000	78449000	69502000
tr|Q9RVW	tr|Q9RVW	7.68E+08	8.06E+08	7.73E+08	1.01E+09	1.12E+09	9.47E+08	6.53E+08
tr|Q9RVW	tr|Q9RVW	2.25E+08	2.3E+08	1.8E+08	1.6E+08	1.45E+08	1.64E+08	2.77E+08
tr|Q9RVW	tr|Q9RVW	5.47E+08	6.81E+08	5.41E+08	5.98E+08	4.74E+08	4.47E+08	4.8E+08
tr|Q9RVW	tr|Q9RVW							
tr|Q9RVX1	tr|Q9RVX1	3.2E+08	2.25E+08	2.21E+08	2.98E+08	2.46E+08	2.88E+08	1.78E+08
tr|Q9RVX2	tr|Q9RVX2							
tr|Q9RVX5	tr|Q9RVX5				1.07E+08		1.02E+08	
tr|Q9RVX7	tr|Q9RVX7	8.97E+08	9.48E+08	8.89E+08	8.35E+08	7.74E+08	9.57E+08	6.95E+08
tr|Q9RVY0	tr|Q9RVY0				1.56E+08			
tr|Q9RVY2	tr|Q9RVY2		51133000	64527000				54729000
tr|Q9RVY4	tr|Q9RVY4	72328000	75170000	83392000	44213000	47765000	43257000	

tr|Q9RVY5	tr|Q9RVY5							
tr|Q9RVY9	tr|Q9RVY9							
tr|Q9RVZ0	tr|Q9RVZ0			36190000	1.02E+08	71762000	91774000	71180000
tr|Q9RVZ2	tr|Q9RVZ2	5.56E+08	5.86E+08	5.15E+08	2.69E+08	3.88E+08	3.16E+08	4.82E+08
tr|Q9RVZ3	tr|Q9RVZ3	7.8E+08	5.63E+08	4.21E+08	4.03E+08	5.94E+08	6.79E+08	2.75E+08
tr|Q9RVZ4	tr|Q9RVZ4	2.98E+08	3.07E+08	2.64E+08	5.64E+08	4.9E+08	5.05E+08	1.83E+08
tr|Q9RVZ5	tr|Q9RVZ5	81865000	72041000	63952000	1.06E+08	97242000	1.08E+08	63415000
tr|Q9RVZ7	tr|Q9RVZ7				21965000			
tr|Q9RVZ9	tr|Q9RVZ9							
tr|Q9RW0	tr|Q9RW0	41315000	43825000	37793000	47255000	46504000	59736000	59634000
tr|Q9RW0	tr|Q9RW0	8.22E+09	8.34E+09	6.78E+09	6.78E+09	6.52E+09	7.12E+09	7.44E+09
tr|Q9RW0	tr|Q9RW0	1.46E+09	1.73E+09	1.56E+09	1.88E+09	1.27E+09	1.67E+09	1.8E+09
tr|Q9RW0	tr|Q9RW0							
tr|Q9RW0	tr|Q9RW0	85361000	1.02E+08	95330000		1.18E+08	1.33E+08	
tr|Q9RW0	tr|Q9RW0	91245000	1.11E+08	79091000	1.75E+08	1.47E+08	1.79E+08	
tr|Q9RW1	tr|Q9RW1	82753000	68767000	65703000	1.14E+08	92527000	1.06E+08	
tr|Q9RW1	tr|Q9RW1							
tr|Q9RW1	tr|Q9RW1	3.92E+08	4.08E+08	4.28E+08	6.05E+08	7.34E+08	6.2E+08	8.78E+08
tr|Q9RW1	tr|Q9RW1							
tr|Q9RW2	tr|Q9RW2	15658000						
tr|Q9RW2	tr|Q9RW2	2.1E+08	1.95E+08	2.03E+08	1.26E+08	1.27E+08	1.18E+08	1.13E+08
tr|Q9RW2	tr|Q9RW2	1.66E+08	1.95E+08	1.71E+08	1.28E+08	1.28E+08	1.56E+08	
tr|Q9RW2	tr|Q9RW2	1.7E+09	2.25E+09	2.15E+09	3.16E+09	2.9E+09	3.04E+09	1.01E+09
tr|Q9RW2	tr|Q9RW2	3.62E+08	3.34E+08	3.23E+08	3.38E+08	3.36E+08	2.99E+08	3.41E+08
tr|Q9RW2	tr|Q9RW2	2.44E+08	2.89E+08	2.64E+08	2.33E+08	2.36E+08	2.57E+08	2.22E+08
tr|Q9RW3	tr|Q9RW3	83570000	79672000	65422000	1.13E+08	1.07E+08	1.2E+08	67905000
tr|Q9RW3	tr|Q9RW3	1.4E+08	1.52E+08		1.28E+08	1.88E+08	2.05E+08	2.1E+08
tr|Q9RW3	tr|Q9RW3	1.07E+08	1.05E+08	1.01E+08	1.9E+08	1.65E+08	1.97E+08	1.05E+08
tr|Q9RW3	tr|Q9RW3	6.64E+08	7.22E+08	7.74E+08	3.88E+08	5.53E+08	3.83E+08	7.91E+08
tr|Q9RW4	tr|Q9RW4	2.05E+08	1.79E+08	2.11E+08				2.68E+08
tr|Q9RW4	tr|Q9RW4							
tr|Q9RW4	tr|Q9RW4	56589000	84550000	70386000	85226000	88043000	85709000	
tr|Q9RW4	tr|Q9RW4							
tr|Q9RW4	tr|Q9RW4	2.77E+08	2.75E+08	2.48E+08	2.15E+08	2.08E+08		3.54E+08
tr|Q9RW5	tr|Q9RW5							
tr|Q9RW5	tr|Q9RW5	1.22E+09	1.67E+09	1.44E+09	1.78E+09	1.81E+09	1.68E+09	1.19E+09
tr|Q9RW5	tr|Q9RW5							
tr|Q9RW6	tr|Q9RW6	1.85E+08	2.59E+08	3.53E+08	1.46E+08	2.21E+08	1.22E+08	1.7E+08
tr|Q9RW6	tr|Q9RW6	37391000	55990000		1.18E+08	1.12E+08	77205000	44551000
tr|Q9RW6	tr|Q9RW6							
tr|Q9RW7	tr|Q9RW7	4.76E+08	4.57E+08	4.12E+08	5.19E+08	5.4E+08	5.61E+08	3.31E+08
tr|Q9RW7	tr|Q9RW7							
tr|Q9RW7	tr|Q9RW7	3.76E+08	4.12E+08	3.98E+08	1.7E+08	2.23E+08	3.08E+08	2.87E+08
tr|Q9RW7	tr|Q9RW7							
tr|Q9RW8	tr|Q9RW8	2.76E+08	2.68E+08	2.6E+08	2.49E+08	2.32E+08	2.46E+08	2.61E+08
tr|Q9RW8	tr|Q9RW8	1.64E+09	1.67E+09	2.16E+09	1.53E+09	2.25E+09	1.87E+09	8.9E+08
tr|Q9RW8	tr|Q9RW8	6.89E+08	7.22E+08	6.79E+08	7.04E+08	5.97E+08	6.62E+08	5.79E+08
tr|Q9RW8	tr|Q9RW8	40391000	64103000	45981000	39859000			
tr|Q9RW8	tr|Q9RW8	88014000	77061000	74781000	1.55E+08	99982000	1.26E+08	85815000
tr|Q9RW8	tr|Q9RW8	7.75E+08	8.2E+08	6.5E+08	1.53E+09	1.32E+09	1.21E+09	5.84E+08
tr|Q9RW8	tr|Q9RW8							

tr|Q9RW9	tr|Q9RW9	18374000	19508000	13135000	22395000	19042000	17282000	
tr|Q9RW9	tr|Q9RW9	68797000	70363000	59040000	1.57E+08	1.3E+08	1.19E+08	77222000
tr|Q9RW9	tr|Q9RW9		82493000	68395000	79396000	83371000	76584000	
tr|Q9RW9	tr|Q9RW9		1.79E+08					1.6E+08
tr|Q9RW9	tr|Q9RW9	2.17E+08	3.03E+08	3.06E+08	2.34E+08	1.92E+08	2.39E+08	3.6E+08
tr|Q9RWA	tr|Q9RWA	89242000		88961000	1.32E+08	1.73E+08	1.6E+08	
tr|Q9RWA	tr|Q9RWA	5.46E+08	4.73E+08	3.88E+08	4.17E+08	3.45E+08	3.89E+08	5.18E+08
tr|Q9RWA	tr|Q9RWA	29652000			43500000			
tr|Q9RWA	tr|Q9RWA	1.22E+08	73617000	93902000	34849000	49583000	46554000	55085000
tr|Q9RWA	tr|Q9RWA							
tr|Q9RWB	tr|Q9RWB	49745000	54802000	75700000	69150000	53141000	50517000	
tr|Q9RWB	tr|Q9RWB	61179000	68415000	69522000				62301000
tr|Q9RWB	tr|Q9RWB	1.92E+09	1.81E+09	1.75E+09	2.31E+09	1.54E+09	2.07E+09	1.39E+09
tr|Q9RWB	tr|Q9RWB	1.56E+09	1.54E+09	1.73E+09	2.68E+09	2.77E+09	2.28E+09	1.22E+09
tr|Q9RWB	tr|Q9RWB	1.67E+08	1.4E+08	1.25E+08	1.67E+08	1.44E+08	1.66E+08	1.73E+08
tr|Q9RWC	tr|Q9RWC	9.37E+08	6.52E+08	6.76E+08	5.91E+08	3.71E+08	5.71E+08	7.2E+08
tr|Q9RWC	tr|Q9RWC	8.36E+08	8.52E+08	7.44E+08	7.08E+08	7.33E+08	7.87E+08	8.65E+08
tr|Q9RWC	tr|Q9RWC							
tr|Q9RWC	tr|Q9RWC							
tr|Q9RWC	tr|Q9RWC	98605000	1.25E+08	1.06E+08				
tr|Q9RWC	tr|Q9RWC							
tr|Q9RWD	tr|Q9RWD	44864000	33094000	38815000	54602000			45727000
tr|Q9RWD	tr|Q9RWD	1.45E+08	1.51E+08	1.41E+08	1.97E+08	1.45E+08	2.01E+08	1.52E+08
tr|Q9RWD	tr|Q9RWD							
tr|Q9RWE	tr|Q9RWE					2.95E+08		
tr|Q9RWE	tr|Q9RWE			13693000				
tr|Q9RWE	tr|Q9RWE	32515000	31378000	35806000	32959000	22687000	21190000	45205000
tr|Q9RWF	tr|Q9RWF	2.68E+09	2.33E+09	2.24E+09	2.22E+09	2.13E+09	2.16E+09	2.06E+09
tr|Q9RWF	tr|Q9RWF							
tr|Q9RWF	tr|Q9RWF	59919000			54276000	49564000	52841000	59415000
tr|Q9RWF	tr|Q9RWF							2.64E+08
tr|Q9RWF	tr|Q9RWF							
tr|Q9RWF	tr|Q9RWF			25068000	31539000	34847000	34668000	33905000
tr|Q9RWG	tr|Q9RWG							
tr|Q9RWG	tr|Q9RWG	1.64E+09	1.01E+09	1.23E+09	2.19E+09	1.88E+09	1.72E+09	1.05E+09
tr|Q9RWH	tr|Q9RWH	1.51E+09	3.16E+09	2.73E+09	9.09E+08	5.19E+08	8.22E+08	4.57E+09
tr|Q9RWH	tr|Q9RWH	1.36E+09	9.12E+08	1.03E+09	2.54E+09	1.87E+09	2.27E+09	1.05E+09
tr|Q9RWH	tr|Q9RWH		67455000		61391000		59643000	
tr|Q9RWH	tr|Q9RWH				26296000			
tr|Q9RWH	tr|Q9RWH	76395000	52549000	44842000	65108000	83503000		76478000
tr|Q9RWI(	tr|Q9RWI(	82306000	58630000	90985000	1.35E+08	98615000	97165000	85116000
tr|Q9RWI'	tr|Q9RWI'	58820000	44707000					
tr|Q9RWI3	tr|Q9RWI3							
tr|Q9RWI5	tr|Q9RWI5			69189000			1.62E+08	
tr|Q9RWI6	tr|Q9RWI6	60574000	47599000	60169000	32567000	42003000	51459000	35025000
tr|Q9RWI7	tr|Q9RWI7	1.19E+08	1.69E+08	1.37E+08	1.15E+08	1.06E+08	88624000	1.56E+08
tr|Q9RWI8	tr|Q9RWI8							
tr|Q9RWJ1	tr|Q9RWJ1	52224000	62294000	52278000	1.29E+08	92535000	1.51E+08	77261000
tr|Q9RWJ2	tr|Q9RWJ2	2.1E+08		1.98E+08				
tr|Q9RWJ5	tr|Q9RWJ5							
tr|Q9RWJ7	tr|Q9RWJ7	1.64E+08		3.47E+08				

tr|Q9RWK	tr|Q9RWK	1.07E+08		90711000				
tr|Q9RWK	tr|Q9RWK							
tr|Q9RWK	tr|Q9RWK							
tr|Q9RWK	tr|Q9RWK							
tr|Q9RWK	tr|Q9RWK	8E+08	9.61E+08	8.82E+08	7.49E+08	7.59E+08	9.21E+08	
tr|Q9RWK	tr|Q9RWK	70727000	57309000	65898000	31676000	32368000	54455000	30657000
tr|Q9RWL	tr|Q9RWL							
tr|Q9RWL	tr|Q9RWL	2.39E+08	2.26E+08	2.03E+08		1.02E+08	98304000	2.97E+08
tr|Q9RWL	tr|Q9RWL	2.21E+08	1.77E+08	1.64E+08				
tr|Q9RWL	tr|Q9RWL							
tr|Q9RWL	tr|Q9RWL		49416000	46559000	54527000	65668000		65525000
tr|Q9RWL	tr|Q9RWL			44652000	1.36E+08	1.6E+08		57008000
tr|Q9RWL	tr|Q9RWL							
tr|Q9RWL	tr|Q9RWL	1.17E+08	1.04E+08	1.18E+08	1.41E+08	1.3E+08	1.12E+08	1.46E+08
tr|Q9RWM	tr|Q9RWM	3.62E+08	5.02E+08	4.59E+08	3.18E+08	3.85E+08	3.63E+08	6.9E+08
tr|Q9RWM	tr|Q9RWM	86596000	85064000	84757000		87616000		1.14E+08
tr|Q9RWM	tr|Q9RWM							
tr|Q9RWN	tr|Q9RWN	55896000	67346000	55636000	1.58E+08	1.5E+08	1.26E+08	58109000
tr|Q9RWN	tr|Q9RWN	1.19E+08	1.64E+08	1.78E+08	2.26E+08	2.29E+08	2.12E+08	1.18E+08
tr|Q9RWN	tr|Q9RWN	44923000	48042000	46995000		40625000		
tr|Q9RWN	tr|Q9RWN	44228000		44681000	48462000	45918000		
tr|Q9RWP	tr|Q9RWP	1.15E+08	1.71E+08	1.65E+08	1.15E+08	1.22E+08	1.05E+08	1.46E+08
tr|Q9RWP	tr|Q9RWP	1.12E+08	1.17E+08	1.12E+08	1.32E+08	1.18E+08	1.09E+08	1.52E+08
tr|Q9RWP	tr|Q9RWP	3.93E+08	2.96E+08	2.76E+08	3.12E+08	2.12E+08	2.91E+08	2.9E+08
tr|Q9RWP	tr|Q9RWP				3.68E+08	4.54E+08	3.17E+08	2.86E+08
tr|Q9RWP	tr|Q9RWP	9.88E+08	9.83E+08	9.5E+08	1.15E+09	1.08E+09	1.09E+09	8.71E+08
tr|Q9RWQ	tr|Q9RWQ							
tr|Q9RWQ	tr|Q9RWQ							
tr|Q9RWQ	tr|Q9RWQ	3.67E+08	6.13E+08			3.88E+08	3.9E+08	
tr|Q9RWQ	tr|Q9RWQ	4.06E+08	4.16E+08	3.6E+08	3.01E+08	4.46E+08	4.24E+08	6.13E+08
tr|Q9RWQ	tr|Q9RWQ	1.01E+08	1.37E+08	95934000	1.9E+08	1.93E+08	1.67E+08	74275000
tr|Q9RWQ	tr|Q9RWQ	1.82E+08	2.33E+08	2.08E+08	5.57E+08	5.17E+08	3.83E+08	5.27E+08
tr|Q9RWR	tr|Q9RWR							
tr|Q9RWR	tr|Q9RWR							
tr|Q9RWR	tr|Q9RWR							
tr|Q9RWR	tr|Q9RWR							
tr|Q9RWR	tr|Q9RWR	1.51E+08	1.72E+08	1.35E+08	1.52E+08	1.37E+08	1.53E+08	2.23E+08
tr|Q9RWS	tr|Q9RWS	2.26E+08	2.18E+08	2.28E+08	1.79E+08	1.86E+08	2.51E+08	1.2E+08
tr|Q9RWS	tr|Q9RWS							
tr|Q9RWS	tr|Q9RWS			18004000	53478000	34486000	27792000	
tr|Q9RWS	tr|Q9RWS	1.92E+08	1.66E+08	1.62E+08	1.72E+08	1.33E+08	1.65E+08	1.88E+08
tr|Q9RWT	tr|Q9RWT	2.89E+09	2.58E+09	2.51E+09	2.04E+09	2.24E+09	2.4E+09	3.03E+09
tr|Q9RWT	tr|Q9RWT							
tr|Q9RWT	tr|Q9RWT	1.43E+08	1.23E+08	1.16E+08	1E+08	86853000	80171000	1.14E+08
tr|Q9RWT	tr|Q9RWT	4.98E+08	4.35E+08	5.28E+08	3.38E+08	4.23E+08	4.93E+08	4.44E+08
tr|Q9RWT	tr|Q9RWT	7.43E+08	4.72E+08	4.52E+08	1.04E+09	4.26E+08	7E+08	2.62E+08
tr|Q9RWT	tr|Q9RWT	1.07E+08	1.11E+08	1.02E+08	1.97E+08	2.16E+08	1.67E+08	85911000
tr|Q9RWT	tr|Q9RWT	68923000	70944000	70881000	96602000	1E+08	88119000	
tr|Q9RWT	tr|Q9RWT				31869000			
tr|Q9RWT	tr|Q9RWT	42444000	40900000			63162000		
tr|Q9RWU	tr|Q9RWU	1.24E+09	1.21E+09	1.29E+09	8.41E+08	7.82E+08	7.59E+08	1.31E+09

tr|Q9RWU	tr|Q9RWU							
tr|Q9RWU	tr|Q9RWU				28268000	38161000		
tr|Q9RWU	tr|Q9RWU	1.22E+08	83523000	70111000				93304000
tr|Q9RWU	tr|Q9RWU	5.46E+08	4.93E+08	5.25E+08	4.08E+08	4.09E+08	4.15E+08	4.63E+08
tr|Q9RWU	tr|Q9RWU	1.12E+08	1.1E+08	93780000	1.18E+08	1.01E+08	1.05E+08	76412000
tr|Q9RWU	tr|Q9RWU	1.36E+08	1.67E+08	1.43E+08	97135000	1.12E+08	83812000	1.85E+08
tr|Q9RWU	tr|Q9RWU		61926000	41680000				60126000
tr|Q9RWV	tr|Q9RWV	94159000	1.11E+08	84182000	4.54E+08	2.46E+08	3.35E+08	1.19E+08
tr|Q9RWV	tr|Q9RWV	1.07E+08	93018000		1.88E+08	1.17E+08	1.45E+08	
tr|Q9RWV	tr|Q9RWV	6.8E+09	5.6E+09	4.89E+09	7.4E+09	6.29E+09	7.15E+09	2.94E+09
tr|Q9RWV	tr|Q9RWV	4.65E+08	5.24E+08	5.48E+08	9.72E+08	8.03E+08	5.77E+08	3.3E+08
tr|Q9RWV	tr|Q9RWV	1.11E+08	69928000	78778000	89824000	94995000	87046000	85131000
tr|Q9RWV	tr|Q9RWV	1.08E+08	93522000		65125000	1.37E+08		1.09E+08
tr|Q9RWW	tr|Q9RWW	1.68E+08						
tr|Q9RWW	tr|Q9RWW		55274000	42417000			42967000	
tr|Q9RWW	tr|Q9RWW	6.37E+08	9.02E+08	9.31E+08	3.46E+08	5.17E+08	3.94E+08	1.12E+09
tr|Q9RWW	tr|Q9RWW	50695000		49590000	52437000	50710000	36970000	80946000
tr|Q9RWW	tr|Q9RWW	66129000	47914000	45636000		23554000	24642000	1.35E+08
tr|Q9RWW	tr|Q9RWW	2.07E+09	1.55E+09	1.79E+09	7.88E+08	9.85E+08	1.12E+09	1.06E+09
tr|Q9RWW	tr|Q9RWW							
tr|Q9RWX	tr|Q9RWX							
tr|Q9RWX	tr|Q9RWX							
tr|Q9RWX	tr|Q9RWX	2.32E+08	1.22E+08	1.07E+08	1.2E+08	61458000	1.26E+08	1.76E+08
tr|Q9RWX	tr|Q9RWX	1.45E+08	1.65E+08	1.6E+08	2.24E+08	1.82E+08	1.9E+08	1.34E+08
tr|Q9RWX	tr|Q9RWX	41546000	47147000	33897000	36951000		25031000	52678000
tr|Q9RWY	tr|Q9RWY							36014000
tr|Q9RWY	tr|Q9RWY							
tr|Q9RWY	tr|Q9RWY							
tr|Q9RWZ	tr|Q9RWZ							
tr|Q9RX00	tr|Q9RX00							
tr|Q9RX02	tr|Q9RX02	48654000	48466000		63150000	46222000	61496000	
tr|Q9RX07	tr|Q9RX07	89262000	84914000	83540000		33670000		
tr|Q9RX10	tr|Q9RX10	1.28E+09	1.3E+09	1.25E+09	1.82E+09	1.51E+09	1.13E+09	1.45E+09
tr|Q9RX11	tr|Q9RX11							
tr|Q9RX12	tr|Q9RX12	2.47E+08	2.02E+08	1.86E+08	1.22E+08	1.51E+08	1.28E+08	1.93E+08
tr|Q9RX13	tr|Q9RX13		65953000	69392000	46362000		50562000	
tr|Q9RX16	tr|Q9RX16	3.97E+08	3.64E+08	3.19E+08	2.17E+08	2.27E+08	1.65E+08	2.74E+08
tr|Q9RX17	tr|Q9RX17							
tr|Q9RX18	tr|Q9RX18							
tr|Q9RX19	tr|Q9RX19	2.1E+08	2.11E+08	2.11E+08	2.82E+08	2.63E+08	2.65E+08	1.64E+08
tr|Q9RX24	tr|Q9RX24	51444000	71149000	71012000				49077000
tr|Q9RX28	tr|Q9RX28	21537000	19751000	17105000	22370000	17588000	17064000	
tr|Q9RX29	tr|Q9RX29	7.06E+08	5.74E+08	5.47E+08	7.5E+08	7.47E+08	5.81E+08	1.21E+09
tr|Q9RX31	tr|Q9RX31	26952000		43319000				
tr|Q9RX33	tr|Q9RX33	27841000	30861000	32039000	31879000	38070000	31881000	18129000
tr|Q9RX36	tr|Q9RX36	52697000	47916000	39874000		39779000		87405000
tr|Q9RX37	tr|Q9RX37	2.75E+08	2.49E+08	2.95E+08	3.37E+08	2.72E+08	2.73E+08	2.21E+08
tr|Q9RX41	tr|Q9RX41			61042000			50772000	
tr|Q9RX42	tr|Q9RX42							
tr|Q9RX44	tr|Q9RX44		2.31E+08	2.12E+08				
tr|Q9RX47	tr|Q9RX47							

tr|Q9RX48	tr|Q9RX48	4.51E+08	3.73E+08	3.57E+08	3.83E+08	3.43E+08	3.42E+08	3.26E+08
tr|Q9RX49	tr|Q9RX49							
tr|Q9RX52	tr|Q9RX52			83683000				1.98E+08
tr|Q9RX53	tr|Q9RX53							
tr|Q9RX55	tr|Q9RX55	3.62E+08	2.74E+08	2.58E+08	4.34E+08	4.09E+08	5.4E+08	1.97E+08
tr|Q9RX56	tr|Q9RX56	7.53E+08	6.34E+08	7.52E+08	1.81E+09	1.43E+09	1.41E+09	6.16E+08
tr|Q9RX57	tr|Q9RX57	3.02E+08	2.76E+08	2.88E+08	4.45E+08	3.77E+08	4.31E+08	3.65E+08
tr|Q9RX58	tr|Q9RX58							
tr|Q9RX59	tr|Q9RX59	3.47E+08	2.83E+08	3.12E+08	3.69E+08	3.33E+08	3.06E+08	3.57E+08
tr|Q9RX62	tr|Q9RX62							
tr|Q9RX63	tr|Q9RX63				34855000	26678000	28850000	48795000
tr|Q9RX64	tr|Q9RX64	1.46E+08	1.27E+08	1.27E+08	1.39E+08	1.51E+08	1.54E+08	1.71E+08
tr|Q9RX65	tr|Q9RX65	50096000	83781000	79574000	1.4E+08	1.88E+08	1.37E+08	83988000
tr|Q9RX66	tr|Q9RX66							
tr|Q9RX69	tr|Q9RX69	3.09E+08						
tr|Q9RX71	tr|Q9RX71	39461000	51931000	43961000		70954000		60498000
tr|Q9RX72	tr|Q9RX72	8.62E+08	6.26E+08	4.89E+08	4.26E+08	7.51E+08	5.98E+08	7.63E+08
tr|Q9RX73	tr|Q9RX73							
tr|Q9RX76	tr|Q9RX76				46270000	44676000	54943000	
tr|Q9RX79	tr|Q9RX79							
tr|Q9RX80	tr|Q9RX80	2.71E+08	2.14E+08	2.32E+08	2.74E+08	2.27E+08	2.6E+08	1.92E+08
tr|Q9RX81	tr|Q9RX81	25204000	21747000	30703000	40281000	50690000	54829000	
tr|Q9RX83	tr|Q9RX83	2.07E+08	1.26E+08	2.43E+08	3.55E+08	3.56E+08	4.19E+08	1.79E+08
tr|Q9RX84	tr|Q9RX84	16703000		15184000		22001000		
tr|Q9RX85	tr|Q9RX85		52979000		24745000			
tr|Q9RX86	tr|Q9RX86	2.97E+08	3.68E+08	2.78E+08	3.88E+08	3.05E+08	3.5E+08	2.33E+08
tr|Q9RX87	tr|Q9RX87	62158000	99518000	71970000				48411000
tr|Q9RX95	tr|Q9RX95	16907000	28001000	20157000	29820000	24265000	23183000	36249000
tr|Q9RX97	tr|Q9RX97							
tr|Q9RXA0	tr|Q9RXA0							
tr|Q9RXA2	tr|Q9RXA2				54610000	60503000	36822000	
tr|Q9RXA3	tr|Q9RXA3							
tr|Q9RXA4	tr|Q9RXA4	86253000	70741000	1.05E+08	54326000	50363000	59318000	
tr|Q9RXA6	tr|Q9RXA6							
tr|Q9RXA9	tr|Q9RXA9	53975000	55817000	33365000	37417000	29683000	33214000	82498000
tr|Q9RXB0	tr|Q9RXB0	2.68E+08	3.47E+08	3.21E+08	3.91E+08	5.29E+08	4.75E+08	2.56E+08
tr|Q9RXB1	tr|Q9RXB1							
tr|Q9RXB2	tr|Q9RXB2	62725000	54440000	71811000	41125000	49227000	50498000	
tr|Q9RXB3	tr|Q9RXB3							
tr|Q9RXB6	tr|Q9RXB6	4.34E+08	4.58E+08	3.84E+08	3.35E+08	4.48E+08	5.13E+08	2.4E+08
tr|Q9RXB8	tr|Q9RXB8	3.52E+08	2.91E+08	3E+08	5.27E+08	4.96E+08	4.61E+08	2.38E+08
tr|Q9RXB9	tr|Q9RXB9							
tr|Q9RXC2	tr|Q9RXC2						63335000	
tr|Q9RXC3	tr|Q9RXC3	49112000	96065000	85326000	77907000	84920000	78240000	27665000
tr|Q9RXC5	tr|Q9RXC5	3.59E+08	3.86E+08	4.04E+08	3.8E+08	3.64E+08	4.19E+08	2.83E+08
tr|Q9RXD1	tr|Q9RXD1	2.67E+08	3.37E+08	2.57E+08	4.48E+08	3.74E+08	3.46E+08	2.84E+08
tr|Q9RXD2	tr|Q9RXD2	9.33E+08	9.65E+08	1.23E+09	7.65E+08	7.32E+08	6.44E+08	5.47E+08
tr|Q9RXD5	tr|Q9RXD5	1.54E+09	1.49E+09	1.33E+09	1.73E+09	1.72E+09	1.51E+09	2.1E+09
tr|Q9RXD7	tr|Q9RXD7	2.55E+08	2.62E+08	2.08E+08	2.81E+08	2.55E+08	3.07E+08	2.07E+08
tr|Q9RXE3	tr|Q9RXE3					29513000		
tr|Q9RXE5	tr|Q9RXE5	1.81E+08	2.38E+08	1.81E+08		1.1E+08	1.13E+08	2.28E+08

tr|Q9RXE6	tr|Q9RXE6		35555000					33296000
tr|Q9RXE7	tr|Q9RXE7			76511000			96474000	
tr|Q9RXE8	tr|Q9RXE8	80950000	1.2E+08	1.03E+08	1.62E+08	1.62E+08	1.35E+08	1.24E+08
tr|Q9RXE9	tr|Q9RXE9		3.01E+08			2.19E+08		
tr|Q9RXF0	tr|Q9RXF0	7.02E+09	5.78E+09	8.29E+09	7.08E+09	6.3E+09	7.92E+09	5.44E+09
tr|Q9RXF2	tr|Q9RXF2							
tr|Q9RXF3	tr|Q9RXF3			54989000		71463000		
tr|Q9RXF5	tr|Q9RXF5	85862000			1.7E+08			85776000
tr|Q9RXF6	tr|Q9RXF6							
tr|Q9RXF9	tr|Q9RXF9							
tr|Q9RXG0	tr|Q9RXG0	7.86E+08	9.57E+08	9.03E+08	8.34E+08	8.47E+08	7.26E+08	1.14E+09
tr|Q9RXG1	tr|Q9RXG1	4.4E+08	5.17E+08	4.39E+08	5.56E+08	4.3E+08	4.87E+08	7.51E+08
tr|Q9RXG3	tr|Q9RXG3	2.79E+08	2.33E+08	2.15E+08	98678000	1.18E+08	1.1E+08	2.96E+08
tr|Q9RXG7	tr|Q9RXG7		47420000	53318000			37879000	
tr|Q9RXG8	tr|Q9RXG8	1.66E+08	2.06E+08	1.62E+08	2.03E+08	1.97E+08	1.31E+08	
tr|Q9RXG9	tr|Q9RXG9							
tr|Q9RXH0	tr|Q9RXH0	37142000	47052000	44247000	58779000	51463000	55722000	56214000
tr|Q9RXH1	tr|Q9RXH1	29685000	23448000	22651000	23542000			32269000
tr|Q9RXH3	tr|Q9RXH3	62527000	63699000	55345000	1.13E+08	1.16E+08		67311000
tr|Q9RXH4	tr|Q9RXH4	1.43E+08	1.09E+08	1.54E+08	3.07E+08	2.78E+08	1.98E+08	1.1E+08
tr|Q9RXH6	tr|Q9RXH6							
tr|Q9RXH7	tr|Q9RXH7							
tr|Q9RXH8	tr|Q9RXH8	2.77E+08	2.54E+08	2.71E+08	1.39E+08	1.22E+08	1.38E+08	4.53E+08
tr|Q9RXI0	tr|Q9RXI0							
tr|Q9RXI1	tr|Q9RXI1	5.95E+08	5.76E+08	6.06E+08	8.14E+08	5.77E+08	5.26E+08	5.8E+08
tr|Q9RXI3	tr|Q9RXI3	2.36E+08	2.22E+08	2.23E+08	3.49E+08	3.15E+08	2.91E+08	92794000
tr|Q9RXI4	tr|Q9RXI4	62041000	63379000	56067000	1.2E+08	93927000	96647000	59270000
tr|Q9RXI5	tr|Q9RXI5							
tr|Q9RXI6	tr|Q9RXI6							
tr|Q9RXI9	tr|Q9RXI9							
tr|Q9RXK4	tr|Q9RXK4							
tr|Q9RXK9	tr|Q9RXK9	2.54E+08	2.46E+08	1.9E+08	2.33E+08	2.07E+08	2.29E+08	2.03E+08
tr|Q9RXL1	tr|Q9RXL1							
tr|Q9RXL4	tr|Q9RXL4			44541000	70634000	80999000	77241000	
tr|Q9RXL5	tr|Q9RXL5	1.56E+08	2.19E+08	1.51E+08	83356000	87562000	1.2E+08	1.57E+08
tr|Q9RXL6	tr|Q9RXL6	4.21E+08	3.35E+08	3.43E+08	1.88E+08	2.45E+08	1.58E+08	5.38E+08
tr|Q9RXL9	tr|Q9RXL9	1.5E+08	1.09E+08	81965000			66074000	82603000
tr|Q9RXM	tr|Q9RXM	33118000	51805000	58154000	87682000	1.11E+08	75913000	32462000
tr|Q9RXM	tr|Q9RXM	1.89E+09	1.5E+09	1.43E+09	1.28E+09	1.43E+09	1.41E+09	2.21E+09
tr|Q9RXM	tr|Q9RXM	1.76E+08	1.8E+08	1.88E+08	3.28E+08	3.45E+08	2.13E+08	3.44E+08
tr|Q9RXM	tr|Q9RXM	44823000	94392000	63588000	67542000	42109000	74516000	98573000
tr|Q9RXM	tr|Q9RXM			16524000				31507000
tr|Q9RXN0	tr|Q9RXN0		1.88E+08	2.45E+08	2.74E+08	3.01E+08	2.37E+08	1.33E+08
tr|Q9RXN3	tr|Q9RXN3	3.03E+08	3.14E+08	2.3E+08	3.5E+08	3.63E+08	3.72E+08	2.91E+08
tr|Q9RXN7	tr|Q9RXN7		2.56E+08	1.58E+08				
tr|Q9RXN9	tr|Q9RXN9							
tr|Q9RXP2	tr|Q9RXP2	1.35E+08	79798000	1.02E+08	1.03E+08		1.05E+08	1.38E+08
tr|Q9RXP4	tr|Q9RXP4			53086000	52324000	44460000	52471000	
tr|Q9RXP5	tr|Q9RXP5	6.22E+08	4.18E+08	3.93E+08	5.39E+08	3.97E+08	3.61E+08	3.49E+08
tr|Q9RXP7	tr|Q9RXP7	55891000	33917000	28416000	58189000	56816000	75027000	
tr|Q9RXQ	tr|Q9RXQ	1.31E+09	1.15E+09	1.08E+09	1.43E+09	1.33E+09	1.36E+09	1.26E+09

tr|Q9RXQ	tr|Q9RXQ	1.23E+08	97283000	1.39E+08	1.21E+08	2.08E+08	2.15E+08	1.9E+08
tr|Q9RXQ	tr|Q9RXQ	1.62E+08	1.64E+08	1.18E+08	1.22E+08	1.58E+08	1.56E+08	2.38E+08
tr|Q9RXQ	tr|Q9RXQ							
tr|Q9RXQ	tr|Q9RXQ	27288000		16368000		28655000		
tr|Q9RXR1	tr|Q9RXR1	2.45E+08	3.03E+08	2.14E+08	2.04E+08	1.64E+08	2.3E+08	1.69E+08
tr|Q9RXR3	tr|Q9RXR3							
tr|Q9RXR7	tr|Q9RXR7							
tr|Q9RXR8	tr|Q9RXR8							
tr|Q9RXR9	tr|Q9RXR9	1.65E+09	2.23E+09	2.09E+09	1.02E+09	1.15E+09	1.16E+09	1.82E+09
tr|Q9RXS0	tr|Q9RXS0	2.24E+08	1.68E+08	1.39E+08	1.11E+08	1.46E+08	1E+08	2.37E+08
tr|Q9RXS1	tr|Q9RXS1	5.91E+08	4.67E+08	4.87E+08	3.89E+08	3.43E+08	4.42E+08	3.67E+08
tr|Q9RXS5	tr|Q9RXS5							
tr|Q9RXS7	tr|Q9RXS7	3.05E+09	3.45E+09	3.11E+09	2.96E+09	2.97E+09	3.02E+09	3.03E+09
tr|Q9RXT6	tr|Q9RXT6		76481000	63923000		77650000	53015000	
tr|Q9RXT8	tr|Q9RXT8	2.25E+08	3.36E+08	2.29E+08	4.03E+08	3.66E+08	4.2E+08	3.41E+08
tr|Q9RXU0	tr|Q9RXU0	4.79E+08	1.23E+09	7.11E+08	3.34E+08	6.1E+08	3.92E+08	1.09E+09
tr|Q9RXU1	tr|Q9RXU1		25995000	25081000	28305000			
tr|Q9RXU2	tr|Q9RXU2							
tr|Q9RXU3	tr|Q9RXU3		1.87E+08	1.7E+08	2.09E+08	1.59E+08	2.35E+08	1.61E+08
tr|Q9RXU6	tr|Q9RXU6	26375000	52296000	36366000			40765000	51648000
tr|Q9RXU8	tr|Q9RXU8							
tr|Q9RXU9	tr|Q9RXU9							
tr|Q9RXV1	tr|Q9RXV1			20138000			29215000	
tr|Q9RXV5	tr|Q9RXV5		67684000	78372000	37151000	67334000		
tr|Q9RXV6	tr|Q9RXV6	2.07E+08	75998000	64981000	47796000	47720000	51632000	80614000
tr|Q9RXV8	tr|Q9RXV8	8.87E+08	6.78E+08	8.38E+08	6.73E+08	6.94E+08	7.71E+08	5.51E+08
tr|Q9RXV9	tr|Q9RXV9	2.21E+08	2.47E+08	1.86E+08	3.02E+08	2.39E+08	2.53E+08	2.47E+08
tr|Q9RXW	tr|Q9RXW							
tr|Q9RXW	tr|Q9RXW	70071000			68871000	61459000	69970000	
tr|Q9RXW	tr|Q9RXW							
tr|Q9RXW	tr|Q9RXW	6.41E+08	6.69E+08	6.85E+08	3.96E+08	4.86E+08	4.33E+08	
tr|Q9RXW	tr|Q9RXW	56641000	61219000	44048000	64964000		55303000	43317000
tr|Q9RXX2	tr|Q9RXX2	5.66E+08	6.25E+08	5.66E+08	3.26E+08	3.73E+08	2.65E+08	5.31E+08
tr|Q9RXX3	tr|Q9RXX3	5.79E+08	5.5E+08	5.94E+08	6.78E+08	6.49E+08	7.24E+08	9.98E+08
tr|Q9RXX5	tr|Q9RXX5			59342000		57581000		
tr|Q9RXX9	tr|Q9RXX9	61210000	64983000	74018000		46939000		78349000
tr|Q9RXY3	tr|Q9RXY3							
tr|Q9RXY6	tr|Q9RXY6	1.58E+08	1.53E+08	1.09E+08			77064000	1.68E+08
tr|Q9RXY8	tr|Q9RXY8	2.02E+08	2.38E+08	1.79E+08	1.2E+08	1.24E+08	1.08E+08	1.69E+08
tr|Q9RXY9	tr|Q9RXY9	1.14E+08	1.54E+08	1.36E+08	1.28E+08	97902000	1.04E+08	
tr|Q9RXZ2	tr|Q9RXZ2	3.98E+08		2.31E+08		2.33E+08	2.27E+08	
tr|Q9RXZ3	tr|Q9RXZ3	1.38E+08	1.73E+08	1.63E+08	1.73E+08	1.35E+08	2.09E+08	1.67E+08
tr|Q9RXZ4	tr|Q9RXZ4	3.32E+08	4.48E+08	3.71E+08	4.1E+08	3.69E+08	4.45E+08	4.27E+08
tr|Q9RXZ6	tr|Q9RXZ6	34729000						43544000
tr|Q9RXZ9	tr|Q9RXZ9	48519000		51291000	28460000		49097000	
tr|Q9RY00	tr|Q9RY00							
tr|Q9RY01	tr|Q9RY01	1.18E+08	1.07E+08	83881000				1.41E+08
tr|Q9RY02	tr|Q9RY02	2.97E+09	2.89E+09	3.03E+09	2.04E+09	1.83E+09	1.93E+09	1.68E+09
tr|Q9RY03	tr|Q9RY03							
tr|Q9RY04	tr|Q9RY04							
tr|Q9RY08	tr|Q9RY08		1.86E+08					1.93E+08

tr|Q9RY13	tr|Q9RY13	1.67E+08	1.12E+08	1.12E+08	1.41E+08	1.1E+08	1.07E+08	1.27E+08
tr|Q9RY16	tr|Q9RY16	1.19E+08	1.37E+08	1.28E+08		75956000	98395000	1.03E+08
tr|Q9RY20	tr|Q9RY20	6.61E+08	5.8E+08	6.17E+08	5.02E+08	5.1E+08	5.26E+08	3.37E+08
tr|Q9RY21	tr|Q9RY21	4.55E+08	2.51E+08	3.71E+08	6.52E+08	6.45E+08	4.27E+08	2.21E+08
tr|Q9RY22	tr|Q9RY22	76912000	73138000	62753000	72738000	80355000		1.1E+08
tr|Q9RY25	tr|Q9RY25	1.55E+08	2.02E+08	1.6E+08	1.33E+08	1.13E+08	1.45E+08	1.67E+08
tr|Q9RY30	tr|Q9RY30	7.69E+08	7.6E+08	7.76E+08	9.2E+08	8.69E+08	8.55E+08	7.29E+08
tr|Q9RY33	tr|Q9RY33	5.23E+08	7.54E+08	5.62E+08	8.75E+08	8.04E+08	8.05E+08	6.35E+08
tr|Q9RY34	tr|Q9RY34	8.37E+08	4.82E+08	6.58E+08	1.12E+09	1.19E+09	1.09E+09	5.35E+08
tr|Q9RY35	tr|Q9RY35	1.2E+09	9.86E+08	1.02E+09	6.28E+08	3.96E+08	4.86E+08	1.08E+09
tr|Q9RY36	tr|Q9RY36							
tr|Q9RY37	tr|Q9RY37	4.01E+08	4.29E+08	6.62E+08	5.94E+08	6.61E+08	5.68E+08	1.59E+08
tr|Q9RY38	tr|Q9RY38	70405000						46810000
tr|Q9RY39	tr|Q9RY39	50031000	45669000	56049000	35101000	1.03E+08	62991000	
tr|Q9RY43	tr|Q9RY43	1.84E+08		91701000		80254000		1.06E+08
tr|Q9RY46	tr|Q9RY46	8.16E+08	9.07E+08	6.76E+08	1.61E+09	1.42E+09	1.68E+09	9E+08
tr|Q9RY47	tr|Q9RY47	1.2E+08	1.13E+08	94163000	71304000	79356000	73103000	1.03E+08
tr|Q9RY54	tr|Q9RY54	44257000	57891000	42642000	40012000	39063000	45218000	46726000
tr|Q9RY55	tr|Q9RY55	66326000	73369000	53493000	60161000	68082000	66086000	86602000
tr|Q9RY56	tr|Q9RY56	2.83E+09	2.82E+09	3.15E+09	3.08E+09	2.66E+09	3.83E+09	8.6E+08
tr|Q9RY57	tr|Q9RY57			1.13E+08	2.1E+08	1.89E+08	1.68E+08	
tr|Q9RY62	tr|Q9RY62				43493000	47680000	44779000	
tr|Q9RY67	tr|Q9RY67	1.71E+09	1.82E+09	1.75E+09	2.14E+09	2.32E+09	2.38E+09	1.52E+09
tr|Q9RY69	tr|Q9RY69	5.32E+08	4.67E+08	4.54E+08	3E+08	4E+08	3.31E+08	5.97E+08
tr|Q9RY73	tr|Q9RY73	82525000	96132000	93510000	1.16E+08	1.19E+08	1.18E+08	1.57E+08
tr|Q9RY74	tr|Q9RY74	3.15E+08	2.49E+08	2.38E+08	2.11E+08	1.86E+08	1.99E+08	3.57E+08
tr|Q9RY75	tr|Q9RY75	1.46E+08	1.52E+08	1.26E+08	1.43E+08	1.18E+08	1.36E+08	1.39E+08
tr|Q9RY76	tr|Q9RY76							
tr|Q9RY78	tr|Q9RY78	5.65E+08	2.67E+08	4.84E+08	4.63E+08	3.47E+08	3.75E+08	6.01E+08
tr|Q9RY79	tr|Q9RY79	7.63E+08	7.41E+08	7.12E+08	5.81E+08	5.43E+08	6.4E+08	6.36E+08
tr|Q9RY83	tr|Q9RY83	97199000	90841000	97673000	1.69E+08	1.55E+08	1.43E+08	76936000
tr|Q9RY86	tr|Q9RY86	3.73E+08	3.74E+08	3.32E+08	4.01E+08	4.1E+08	3.87E+08	5.25E+08
tr|Q9RY87	tr|Q9RY87							
tr|Q9RY88	tr|Q9RY88		37174000	23310000				86911000
tr|Q9RY92	tr|Q9RY92	2.02E+08		1.7E+08			2.06E+08	
tr|Q9RY95	tr|Q9RY95							
tr|Q9RY96	tr|Q9RY96			46966000				
tr|Q9RY97	tr|Q9RY97							
tr|Q9RY98	tr|Q9RY98							29948000
tr|Q9RYA0	tr|Q9RYA0	76205000	83868000	78506000	26668000	24436000		66190000
tr|Q9RYA5	tr|Q9RYA5	2.8E+08	2.68E+08	2.83E+08	2.65E+08	1.61E+08	1.65E+08	1.85E+08
tr|Q9RYA6	tr|Q9RYA6	4.89E+08	5.01E+08	4.23E+08	3.23E+08	3.47E+08	3.15E+08	3.51E+08
tr|Q9RYA9	tr|Q9RYA9	4.21E+08	5.04E+08	3.89E+08	7.3E+08	6.17E+08	6.59E+08	4.16E+08
tr|Q9RYB8	tr|Q9RYB8	7.49E+08	6.65E+08	7.04E+08	7.02E+08	5.97E+08	6.43E+08	6.88E+08
tr|Q9RYC0	tr|Q9RYC0	1.72E+08	1.25E+08	1.32E+08	1.64E+08	2.02E+08	1.68E+08	1.22E+08
tr|Q9RYC2	tr|Q9RYC2	1.18E+08	3.23E+08	4.22E+08	4E+08	2.64E+08	2.96E+08	2.58E+08
tr|Q9RYC4	tr|Q9RYC4	36834000	59470000	45103000	60173000	69414000	51577000	71822000
tr|Q9RYC5	tr|Q9RYC5		1.27E+08	1.67E+08	2.32E+08	2.47E+08	2.44E+08	
tr|Q9RYC6	tr|Q9RYC6	2.6E+08	2.15E+08	1.79E+08	3.3E+08	2.96E+08	3.05E+08	2.79E+08
tr|Q9RYC7	tr|Q9RYC7	3.58E+08	2.35E+08	2.98E+08	87982000	1.39E+08	98086000	5E+08
tr|Q9RYC9	tr|Q9RYC9							

tr|Q9RYD(	tr|Q9RYD(	4.41E+08	4.86E+08	3.6E+08	4.94E+08	4.57E+08	6.08E+08	4.9E+08
tr|Q9RYD´	tr|Q9RYD´	35269000						
tr|Q9RYD2	tr|Q9RYD2							
tr|Q9RYD3	tr|Q9RYD3					29815000		41096000
tr|Q9RYD7	tr|Q9RYD7	1.24E+08	1.9E+08	1.47E+08	2.7E+08	3.3E+08	2.69E+08	1.32E+08
tr|Q9RYD9	tr|Q9RYD9	1.56E+09	1.55E+09	1.53E+09	2.61E+09	1.93E+09	2.23E+09	1.78E+09
tr|Q9RYE0	tr|Q9RYE0	5.49E+08	4.56E+08	4.73E+08	4.76E+08	4.25E+08	4.87E+08	3.27E+08
tr|Q9RYE1	tr|Q9RYE1	67127000		66572000				
tr|Q9RYE4	tr|Q9RYE4	5.62E+08	4.91E+08	4.78E+08	3.41E+08	3.08E+08	3.01E+08	4.56E+08
tr|Q9RYE5	tr|Q9RYE5	24752000			53141000	47421000		
tr|Q9RYE8	tr|Q9RYE8	8.5E+08	7.55E+08	6.98E+08	6.31E+08	7.34E+08	6.34E+08	5.63E+08
tr|Q9RYE9	tr|Q9RYE9	80294000	70172000	55860000				61424000
tr|Q9RYF0	tr|Q9RYF0	1.88E+08	2.66E+08	1.84E+08	4.83E+08	3.9E+08	3.8E+08	1.51E+08
tr|Q9RYF4	tr|Q9RYF4	1.41E+09	1.43E+09	1.32E+09	1.19E+09	1.15E+09	1.24E+09	1.77E+09
tr|Q9RYF5	tr|Q9RYF5	56916000	52131000	36422000				52544000
tr|Q9RYF8	tr|Q9RYF8	1.25E+08	1.67E+08	1.57E+08	1.06E+08	1.47E+08	1.28E+08	1.79E+08
tr|Q9RYG(	tr|Q9RYG(	29806000	30160000	28478000	38234000	27821000		51270000
tr|Q9RYG´	tr|Q9RYG´							
tr|Q9RYG3	tr|Q9RYG3	34433000	29662000	37926000		41702000	32437000	
tr|Q9RYG4	tr|Q9RYG4	22704000	31015000	27241000	30089000	35316000		41039000
tr|Q9RYG5	tr|Q9RYG5	44484000	54739000	44346000	58195000			77072000
tr|Q9RYG7	tr|Q9RYG7							
tr|Q9RYG8	tr|Q9RYG8		74314000		75663000	81588000	44481000	
tr|Q9RYH(	tr|Q9RYH(			35643000				
tr|Q9RYH´	tr|Q9RYH´	56142000	48820000	50850000	62077000	47228000	44243000	
tr|Q9RYH3	tr|Q9RYH3	23027000	28812000			31610000		
tr|Q9RYH6	tr|Q9RYH6				3.2E+08	3.09E+08	3.06E+08	
tr|Q9RYH8	tr|Q9RYH8	99713000	91639000	1.16E+08	1.84E+08	1.26E+08	1.26E+08	2.05E+08
tr|Q9RYH9	tr|Q9RYH9	1.8E+09	1.87E+09	1.54E+09	1.72E+09	1.82E+09	1.78E+09	1.78E+09
tr|Q9RYI0	tr|Q9RYI0							
tr|Q9RYI1	tr|Q9RYI1	5.96E+08	6.97E+08	6.37E+08	5.48E+08	5.74E+08	5.6E+08	4.23E+08
tr|Q9RYI3	tr|Q9RYI3							
tr|Q9RYI6	tr|Q9RYI6							
tr|Q9RYI7	tr|Q9RYI7	4.35E+08	5E+08	4.42E+08	6.5E+08	5.62E+08	5.93E+08	3.29E+08
tr|Q9RYK2	tr|Q9RYK2							
tr|Q9RYK3	tr|Q9RYK3							
tr|Q9RYK8	tr|Q9RYK8	2.05E+08	2.64E+08	2.4E+08	2.36E+08	2.46E+08	2.72E+08	2.92E+08
tr|Q9RYL0	tr|Q9RYL0	5.33E+09	4.93E+09	4.91E+09	2.99E+09	3.36E+09	2.25E+09	2.6E+09
tr|Q9RYL1	tr|Q9RYL1	1.56E+08	1.27E+08	1.38E+08	2.23E+08	2.19E+08	1.83E+08	96126000
tr|Q9RYL2	tr|Q9RYL2	8.8E+08	7.35E+08	7.76E+08	4.33E+08	5.66E+08	2.6E+08	8.81E+08
tr|Q9RYL3	tr|Q9RYL3	1.54E+08	1.55E+08	1.7E+08				1.21E+08
tr|Q9RYL8	tr|Q9RYL8	51378000	55459000	58651000	43080000	80177000	38681000	64987000
tr|Q9RYM	tr|Q9RYM	3.57E+08	2.48E+08	2.01E+08	2.21E+08	2.42E+08	2.92E+08	3.22E+08
tr|Q9RYM	tr|Q9RYM	7.21E+08	7.33E+08	6.25E+08	7.66E+08	6.51E+08	8.03E+08	6.8E+08
tr|Q9RYM	tr|Q9RYM							
tr|Q9RYN(	tr|Q9RYN(	6.52E+08	8.74E+08	1.18E+09	5.04E+08	2.47E+08	4.78E+08	3.22E+08
tr|Q9RYN´	tr|Q9RYN´							
tr|Q9RYN3	tr|Q9RYN3	1.04E+09	1.21E+09	1.22E+09	1.26E+09	1.48E+09	1.29E+09	1.15E+09
tr|Q9RYN4	tr|Q9RYN4	1.65E+08	1.84E+08	1.85E+08	2.23E+08	1.85E+08	1.87E+08	1.64E+08
tr|Q9RYN5	tr|Q9RYN5	4.02E+08	3.4E+08	4.06E+08	5.52E+08	5.54E+08	4.88E+08	2.04E+08
tr|Q9RYN6	tr|Q9RYN6	3.54E+09	3.87E+09	4.59E+09	4.92E+09	5.18E+09	4.2E+09	1.61E+09

tr|Q9RYN8	tr|Q9RYN8	1.06E+08	86752000	75251000	1.2E+08	1.12E+08	1.16E+08	91700000
tr|Q9RYN9	tr|Q9RYN9	1.01E+08	1.17E+08	88186000	1.07E+08	1.02E+08	98115000	1.01E+08
tr|Q9RYP4	tr|Q9RYP4	3.64E+08	3.5E+08	2.84E+08	4.86E+08	4.08E+08	4.6E+08	4.49E+08
tr|Q9RYP5	tr|Q9RYP5							
tr|Q9RYP6	tr|Q9RYP6	54007000		91209000	54701000	39140000	58997000	
tr|Q9RYP8	tr|Q9RYP8	28348000	35755000	32074000	36123000	36821000	36871000	33457000
tr|Q9RYP9	tr|Q9RYP9	15696000		18088000	25221000	34924000	56736000	26228000
tr|Q9RYQ0	tr|Q9RYQ0	5.83E+09	4.76E+09	5.84E+09	3.8E+09	4.09E+09	4.5E+09	4.22E+09
tr|Q9RYQ1	tr|Q9RYQ1				58236000	94046000	61164000	
tr|Q9RYQ2	tr|Q9RYQ2	2.37E+08	2.79E+08	2.29E+08	2.95E+08	3.29E+08	3.04E+08	
tr|Q9RYQ3	tr|Q9RYQ3	1.36E+08	1.04E+08	81049000	2.4E+08	2.18E+08	2.28E+08	68230000
tr|Q9RYQ4	tr|Q9RYQ4	2.9E+08	3.19E+08	3.11E+08	1.29E+09	1.05E+09	1.03E+09	2.58E+08
tr|Q9RYQ7	tr|Q9RYQ7	34435000	29782000	34845000	34171000	37449000	33953000	
tr|Q9RYQ8	tr|Q9RYQ8	95305000	1.07E+08	89397000	1.07E+08		1.16E+08	81428000
tr|Q9RYQ9	tr|Q9RYQ9							
tr|Q9RYR1	tr|Q9RYR1	2.03E+08	1.87E+08	1.71E+08	1.22E+08	95522000	1.12E+08	3.59E+08
tr|Q9RYR2	tr|Q9RYR2							
tr|Q9RYR6	tr|Q9RYR6							
tr|Q9RYR7	tr|Q9RYR7	4.68E+08	3.81E+08	4.29E+08	5.47E+08	5.43E+08	5.4E+08	2.56E+08
tr|Q9RYS0	tr|Q9RYS0							
tr|Q9RYS1	tr|Q9RYS1	7.05E+08	8.77E+08	8.59E+08	3.82E+08	5.07E+08	5.01E+08	5.96E+08
tr|Q9RYS2	tr|Q9RYS2	1.48E+08	1.41E+08	1.67E+08	1.34E+08	59591000		
tr|Q9RYS3	tr|Q9RYS3	3.66E+08	2.66E+08	3.66E+08		2.68E+08	2.36E+08	
tr|Q9RYS5	tr|Q9RYS5	42633000	36806000	38383000	48356000	56857000	51416000	38836000
tr|Q9RYS6	tr|Q9RYS6	2.13E+08	1.37E+08	1.26E+08			1.27E+08	1.77E+08
tr|Q9RYS7	tr|Q9RYS7	7.42E+08	7.07E+08	6.29E+08	5.82E+08	5.91E+08	6.95E+08	5.87E+08
tr|Q9RYS8	tr|Q9RYS8	7.27E+08	7.54E+08	6.1E+08	5.68E+08	6.89E+08	6.24E+08	4.39E+08
tr|Q9RYS9	tr|Q9RYS9	85378000	1.05E+08	73811000	87008000	1.28E+08	96756000	
tr|Q9RYT0	tr|Q9RYT0							
tr|Q9RYT1	tr|Q9RYT1							
tr|Q9RYT2	tr|Q9RYT2	55774000	43974000	57565000			65650000	
tr|Q9RYT3	tr|Q9RYT3							
tr|Q9RYT4	tr|Q9RYT4							
tr|Q9RYT5	tr|Q9RYT5	3.58E+08	3.1E+08	2.85E+08	5.59E+08	4.32E+08	4.58E+08	2.87E+08
tr|Q9RYT6	tr|Q9RYT6	2.69E+08	2.71E+08	2.04E+08	3.52E+08	2.52E+08	3.48E+08	2.92E+08
tr|Q9RYT8	tr|Q9RYT8	7.38E+08	6.28E+08	7.12E+08	7.58E+08	7.18E+08	1.16E+09	3E+08
tr|Q9RYT9	tr|Q9RYT9	25626000	45750000	35991000		1.12E+08		
tr|Q9RYU2	tr|Q9RYU2					1.03E+08		
tr|Q9RYU9	tr|Q9RYU9	2E+08	2.01E+08	1.74E+08	2.34E+08	2.15E+08	2.21E+08	1.85E+08
tr|Q9RYV0	tr|Q9RYV0	8.78E+08	1.02E+09	8.01E+08	1.05E+09	1E+09	1.01E+09	8.47E+08
tr|Q9RYV3	tr|Q9RYV3	3.98E+08	4.2E+08	5.82E+08	5.43E+08	6.18E+08	5.77E+08	1.81E+08
tr|Q9RYV4	tr|Q9RYV4	6.27E+08	6.16E+08	6.33E+08	4.48E+08	5.32E+08	4.64E+08	2.83E+08
tr|Q9RYV6	tr|Q9RYV6	2.29E+08	2.51E+08	2.3E+08			1.43E+08	3.7E+08
tr|Q9RYV7	tr|Q9RYV7							
tr|Q9RYV8	tr|Q9RYV8							
tr|Q9RYW	tr|Q9RYW	1.03E+08	1.35E+08	1.18E+08	1.15E+08	72546000	1.02E+08	94116000
tr|Q9RYW	tr|Q9RYW	90456000	1.08E+08	95462000	93944000	66803000	70372000	
tr|Q9RYW	tr|Q9RYW	95947000	75757000	66982000			56111000	1.04E+08
tr|Q9RYW	tr|Q9RYW	66342000	72421000	73099000	82494000	82501000	57391000	62653000
tr|Q9RYW	tr|Q9RYW	2.87E+08	3.04E+08	2.46E+08	3.71E+08	2.95E+08	3.44E+08	2.84E+08
tr|Q9RYW	tr|Q9RYW	1.58E+08	1.55E+08		1.97E+08	1.83E+08	1.81E+08	1.79E+08

tr|Q9RYX1	tr|Q9RYX1	1.35E+08			1.94E+08	1.63E+08	1.35E+08	
tr|Q9RYX2	tr|Q9RYX2	76854000	1.18E+08	64243000	46053000	57875000	64131000	1.03E+08
tr|Q9RYX5	tr|Q9RYX5	70288000	65404000				80015000	91442000
tr|Q9RYX6	tr|Q9RYX6	4.76E+08	4.56E+08	4.22E+08	4.76E+08	4.82E+08	4.26E+08	5.84E+08
tr|Q9RYY4	tr|Q9RYY4							
tr|Q9RYY7	tr|Q9RYY7	38421000		35294000	35357000	48648000	34517000	21434000
tr|Q9RYY8	tr|Q9RYY8					8.81E+08		7.96E+08
tr|Q9RYY9	tr|Q9RYY9	2.8E+08	2.16E+08					2.68E+08
tr|Q9RYZ6	tr|Q9RYZ6	3.16E+08	3.6E+08	2.91E+08	3.55E+08	4.67E+08	3.85E+08	6.63E+08
tr|Q9RYZ7	tr|Q9RYZ7	41980000	35532000	39325000	31870000			50719000
tr|Q9RYZ9	tr|Q9RYZ9	70047000	51382000	42785000	48612000	40886000	54314000	51829000
tr|Q9RZ03	tr|Q9RZ03	6.87E+08	2.78E+08		4.23E+08	2.75E+08	2.44E+08	1.52E+08
tr|Q9RZ04	tr|Q9RZ04			65750000				
tr|Q9RZ07	tr|Q9RZ07	9.08E+09	8.36E+09	6.98E+09	4.88E+09	5.26E+09	5.74E+09	7.9E+09
tr|Q9RZ08	tr|Q9RZ08	5.17E+08	4.39E+08	4.7E+08	5.22E+08	5.21E+08	5.81E+08	5.32E+08
tr|Q9RZ09	tr|Q9RZ09	4.95E+08	5.05E+08	5.04E+08	1.99E+08	2.45E+08	2.79E+08	4.47E+08
tr|Q9RZ10	tr|Q9RZ10	5.09E+08	4.77E+08	5.73E+08	4.44E+08	4.99E+08	5.1E+08	2.37E+08
tr|Q9RZ12	tr|Q9RZ12	6.1E+08	7.69E+08	6.76E+08	5.14E+08	5.45E+08	5.23E+08	6.05E+08
tr|Q9RZ13	tr|Q9RZ13	5.16E+08	5.82E+08	4.4E+08	2.9E+08	3.46E+08	3.25E+08	7.03E+08
tr|Q9RZ14	tr|Q9RZ14							
tr|Q9RZ16	tr|Q9RZ16	50452000	61205000	43939000	57991000	73169000	65053000	69139000
tr|Q9RZ18	tr|Q9RZ18	1.04E+09	7.87E+08	9.7E+08	1.06E+09	6.92E+08		8.51E+08
tr|Q9RZ22	tr|Q9RZ22	97021000	83879000	54258000	1.01E+08	1.01E+08	96342000	93708000
tr|Q9RZ23	tr|Q9RZ23		75849000	1.04E+08				
tr|Q9RZ24	tr|Q9RZ24	1.42E+08	1.55E+08	1.05E+08	1.24E+08	1.15E+08	1.32E+08	1.61E+08
tr|Q9RZ27	tr|Q9RZ27							
tr|Q9RZ28	tr|Q9RZ28	34307000	33933000	23883000				34602000
tr|Q9RZ30	tr|Q9RZ30							
tr|Q9RZ32	tr|Q9RZ32	2.76E+08	2.92E+08	2.36E+08	3.65E+08	2.2E+08	1.58E+08	3.42E+08
tr|Q9RZ33	tr|Q9RZ33	1.71E+08	2.49E+08	1.67E+08	2.52E+08	2.76E+08	2.57E+08	2.17E+08
tr|Q9RZ35	tr|Q9RZ35							
tr|Q9RZ49	tr|Q9RZ49							
tr|Q9RZ56	tr|Q9RZ56	6.68E+08	7.94E+08	8.21E+08		5.2E+08	2.02E+08	4.65E+08
tr|Q9RZ67	tr|Q9RZ67							
tr|Q9RZ68	tr|Q9RZ68							
tr|Q9RZ70	tr|Q9RZ70							
tr|Q9RZ74	tr|Q9RZ74							
tr|Q9RZ75	tr|Q9RZ75							
tr|Q9RZ76	tr|Q9RZ76							
tr|Q9RZ77	tr|Q9RZ77							
tr|Q9RZ78	tr|Q9RZ78							
tr|Q9RZ79	tr|Q9RZ79							
tr|Q9RZ80	tr|Q9RZ80							
tr|Q9RZ81	tr|Q9RZ81	59484000	72475000	54719000	75763000	66235000	57647000	81038000
tr|Q9RZ83	tr|Q9RZ83							
tr|Q9RZ85	tr|Q9RZ85	6.79E+08	6.07E+08	6.82E+08	6.71E+08	5.71E+08	5.81E+08	6.4E+08
tr|Q9RZ86	tr|Q9RZ86	4.72E+08	3.56E+08	3.72E+08				
tr|Q9RZ87	tr|Q9RZ87	6.68E+08	6.1E+08	5.01E+08	1.63E+09	1.5E+09	1.18E+09	6.08E+08
tr|Q9RZ88	tr|Q9RZ88	1.58E+08	1.53E+08	1.32E+08	86672000	80566000	87193000	1.82E+08
tr|Q9RZ90	tr|Q9RZ90							
tr|Q9RZ91	tr|Q9RZ91							

tr|Q9RZ93	tr|Q9RZ93	1.25E+08		56980000			54425000	79459000
tr|Q9RZ94	tr|Q9RZ94	49935000	62314000	55866000	55188000	73872000	50724000	59671000
tr|Q9RZ97	tr|Q9RZ97							
tr|Q9RZ99	tr|Q9RZ99	79998000	89833000	76731000	69102000	86865000	75713000	63243000
tr|Q9RZA0	tr|Q9RZA0	1.82E+08	1.95E+08	1.71E+08	2.75E+08	2.51E+08	2.66E+08	1.79E+08
tr|Q9RZA1	tr|Q9RZA1	91530000	90735000	1.25E+08	2.33E+08	1.81E+08	2.3E+08	98048000
tr|Q9RZA3	tr|Q9RZA3	23049000			22923000	21981000		53414000
tr|Q9RZA5	tr|Q9RZA5							
tr|Q9RZA6	tr|Q9RZA6	57933000		73599000	1.11E+08	85016000	90262000	
tr|Q9RZA7	tr|Q9RZA7	2.15E+08	2.02E+08	1.88E+08	2.71E+08	2.86E+08	2.7E+08	1.71E+08
tr|Q9RZA9	tr|Q9RZA9	33492000	31281000	27323000		36678000		54965000
tr|Q9RZB1	tr|Q9RZB1							
tr|Q9RZB2	tr|Q9RZB2	1.22E+08	1.33E+08	1.03E+08	1.07E+08	1.04E+08	1.05E+08	93829000
tr|Q9RZB3	tr|Q9RZB3	1.29E+08	1.33E+08	1.41E+08	1.48E+08	1.16E+08	1.44E+08	1.05E+08
tr|Q9RZB4	tr|Q9RZB4				40610000	48080000	73595000	
tr|Q9RZB5	tr|Q9RZB5							
tr|Q9RZB9	tr|Q9RZB9		23709000	29502000	26415000	16813000		
tr|Q9RZC0	tr|Q9RZC0		56173000					
tr|Q9RZC1	tr|Q9RZC1		79007000					74242000
tr|Q9RZC3	tr|Q9RZC3	5.14E+08	5.92E+08	3.93E+08	3.22E+08	3.03E+08	3.64E+08	3.87E+08
tr|Q9RZC4	tr|Q9RZC4	4.31E+08	3.98E+08	3.99E+08	7.38E+08	5.81E+08	7.09E+08	2.41E+08
tr|Q9RZC5	tr|Q9RZC5	2.95E+09	3.06E+09	3.09E+09	3.51E+09	3.71E+09	3.73E+09	1.85E+09
tr|Q9RZC6	tr|Q9RZC6					26676000	25271000	
tr|Q9RZC7	tr|Q9RZC7	2.12E+09	2.19E+09	2.42E+09	3.75E+09	2.95E+09	3.38E+09	1.55E+09
tr|Q9RZD1	tr|Q9RZD1	4.23E+08	3.73E+08	4.24E+08	2.37E+08	3.16E+08	2.83E+08	3.2E+08
tr|Q9RZD3	tr|Q9RZD3							
tr|Q9RZD4	tr|Q9RZD4					30771000	28167000	
tr|Q9RZD6	tr|Q9RZD6	5.91E+08	7.48E+08	6.27E+08	6.47E+08	5.58E+08	7.7E+08	7.56E+08
tr|Q9RZD7	tr|Q9RZD7							1.72E+08
tr|Q9RZD8	tr|Q9RZD8						21220000	
tr|Q9RZE3	tr|Q9RZE3	2.09E+08	2.9E+08	4E+08	2.26E+08	2.38E+08	3E+08	1.93E+08
tr|Q9RZE4	tr|Q9RZE4	7.51E+08	5.76E+08	6.64E+08	7.95E+08	9.15E+08	7.7E+08	6.58E+08
tr|Q9RZE5	tr|Q9RZE5							
tr|Q9RZE6	tr|Q9RZE6	1.97E+08	1.91E+08	2.46E+08	1.12E+08	1.1E+08	1.46E+08	1.71E+08
tr|Q9RZE8	tr|Q9RZE8	85318000	1.32E+08	1.2E+08	1.2E+08	1.11E+08	1.26E+08	75351000
tr|Q9RZE9	tr|Q9RZE9	97604000	1.09E+08	83958000	1.08E+08	1.38E+08		98876000
tr|Q9RZF1	tr|Q9RZF1							
tr|Q9RZF2	tr|Q9RZF2							
tr|Q9RZF3	tr|Q9RZF3			1.41E+08	2.54E+08	1.91E+08	1.99E+08	
tr|Q9RZF5	tr|Q9RZF5							
tr|Q9RZG0	tr|Q9RZG0							
tr|Q9RZG1	tr|Q9RZG1		37721000	42984000	77964000	63654000		73699000
tr|Q9RZG2	tr|Q9RZG2							
tr|Q9RZG9	tr|Q9RZG9		37178000	1.01E+08				
tr|Q9RZH0	tr|Q9RZH0	3.07E+08	2.34E+08	1.05E+08			1.02E+08	1.97E+08
tr|Q9RZH2	tr|Q9RZH2							
tr|Q9RZH3	tr|Q9RZH3							
tr|Q9RZH5	tr|Q9RZH5	1.76E+08	1.65E+08	1.53E+08	99650000	1.21E+08	1.01E+08	2.08E+08
tr|Q9RZH6	tr|Q9RZH6	77186000	57982000	62107000		54614000		82827000
tr|Q9RZH8	tr|Q9RZH8			84988000				1.2E+08
tr|Q9RZH9	tr|Q9RZH9							

tr|Q9RZI0	tr|Q9RZI0							
tr|Q9RZI1	tr|Q9RZI1							
tr|Q9RZI5	tr|Q9RZI5	73926000	66133000	66012000	47164000	58037000		77110000
tr|Q9RZI6	tr|Q9RZI6							
tr|Q9RZJ1	tr|Q9RZJ1							
tr|Q9RZJ2	tr|Q9RZJ2	8.03E+08	8.11E+08	6.19E+08	8.37E+08	5.93E+08	5.96E+08	5.62E+08
tr|Q9RZK2	tr|Q9RZK2	2.32E+08	1.63E+08	1.69E+08	1.47E+08	1.72E+08	1.66E+08	5.91E+08
tr|Q9RZK3	tr|Q9RZK3							1.34E+08
tr|Q9RZL3	tr|Q9RZL3	98269000	1.28E+08	1.08E+08	1.01E+08	78491000	96669000	91044000
tr|Q9RZL4	tr|Q9RZL4				56094000	30985000	26590000	
tr|Q9RZL6	tr|Q9RZL6	2.94E+08	3.23E+08	4.79E+08	6.7E+08	6.07E+08	6.07E+08	1.62E+08
tr|Q9RZL7	tr|Q9RZL7	6.22E+08	5.1E+08	5.88E+08	8.55E+08	7.58E+08	8.82E+08	4.1E+08
tr|Q9RZL9	tr|Q9RZL9							
tr|Q9RZM	tr|Q9RZM							
tr|Q9RZM	tr|Q9RZM							
tr|Q9RZM	tr|Q9RZM	1.06E+08	1.29E+08	1.08E+08	1.3E+08	1.07E+08	1.15E+08	
tr|Q9RZN1	tr|Q9RZN1							
tr|Q9RZN4	tr|Q9RZN4	1.62E+08	2.13E+08	2.06E+08	6.19E+08	5.58E+08	4.08E+08	2.08E+08
tr|Q9RZP2	tr|Q9RZP2	64911000	84086000	1.01E+08	76347000	75821000	60805000	
tr|Q9RZP3	tr|Q9RZP3	77762000	98033000	95530000	78423000	75208000	74100000	1.16E+08
tr|Q9RZP6	tr|Q9RZP6		28826000	38301000				
tr|Q9RZP7	tr|Q9RZP7	4.55E+08	4.04E+08	3.69E+08	3.01E+08	2.85E+08	2.84E+08	4.99E+08
tr|Q9RZP9	tr|Q9RZP9	60835000		75771000		1.47E+08		
tr|Q9RZQ	tr|Q9RZQ	2.43E+08	3.38E+08	3.39E+08	2.49E+08	2.99E+08	2.16E+08	3.17E+08
tr|Q9RZQ	tr|Q9RZQ	4.81E+08	4.39E+08	5.58E+08	3.92E+08	3.78E+08	3.66E+08	2.47E+08
tr|Q9RZQ	tr|Q9RZQ	9.08E+08	1.03E+09	1.1E+09	1.57E+09	1.53E+09	1.62E+09	8.11E+08
tr|Q9RZQ	tr|Q9RZQ							
tr|Q9RZR2	tr|Q9RZR2							
tr|Q9RZR4	tr|Q9RZR4							
tr|Q9RZS0	tr|Q9RZS0							
tr|Q9RZS3	tr|Q9RZS3							
tr|Q9RZS5	tr|Q9RZS5	62336000	59382000	54715000	70178000			55438000
tr|Q9RZS6	tr|Q9RZS6	1.05E+08	86759000	78283000	76687000			
tr|Q9RZS7	tr|Q9RZS7	6.25E+08	6.03E+08	6.1E+08	5.39E+08	6.02E+08	5.63E+08	5.53E+08
tr|Q9RZT0	tr|Q9RZT0	95028000	96276000	94685000	2.26E+08	2.35E+08	2.13E+08	
tr|Q9RZT2	tr|Q9RZT2	2.61E+08	1.89E+08	2.06E+08	1.48E+08	1.68E+08	1.53E+08	2.04E+08
tr|Q9RZT3	tr|Q9RZT3	75972000	1.15E+08	90643000	1.74E+08	1.64E+08	1.5E+08	1.05E+08
tr|Q9RZT4	tr|Q9RZT4	50142000			50880000	39244000		52164000
tr|Q9RZT5	tr|Q9RZT5							
tr|Q9RZT6	tr|Q9RZT6	93921000	1.31E+08	97117000	1.7E+08	1.28E+08	1.86E+08	1.26E+08
tr|Q9RZT7	tr|Q9RZT7	1.09E+08	96095000				1.34E+08	
tr|Q9RZT9	tr|Q9RZT9				88786000		51563000	
tr|Q9RZU0	tr|Q9RZU0	46032000	65913000	48959000	1.59E+08	2.24E+08	1.91E+08	
tr|Q9RZU4	tr|Q9RZU4	1.7E+08						3.22E+08
tr|Q9RZU6	tr|Q9RZU6							
tr|Q9RZU7	tr|Q9RZU7	1.77E+08	1.7E+08	1.94E+08	1.53E+08	3.25E+08	1.8E+08	7.77E+08
tr|Q9RZV0	tr|Q9RZV0							
tr|Q9RZV1	tr|Q9RZV1		93631000	75226000	1.08E+08	94530000	95607000	94301000
tr|Q9RZV2	tr|Q9RZV2		1.93E+08	1.48E+08		1.34E+08		
tr|Q9RZV3	tr|Q9RZV3	38498000	63500000	70316000	51169000	65355000	56664000	26643000
tr|Q9RZV4	tr|Q9RZV4	4.77E+09	4.25E+09	4.34E+09	3.17E+09	3.45E+09	3.27E+09	4.34E+09

tr|Q9RZV9	tr|Q9RZV9	3.02E+08	2.54E+08	2.27E+08	4.36E+08	3.38E+08	3.83E+08	2.61E+08

LFQ intens	LFQ intens	LFQ intens	LFQ intens	LFQ intens	LFQ intens	LFQ intens	LFQ intens	LFQ intens
90766000	89687000	59380000	63273000	77965000	93112000	95795000	76783000	91420000
6.54E+08	5.65E+08	5.38E+08	5E+08	5.18E+08	4.06E+08	4.92E+08	5.37E+08	6.17E+08
42511000	1.04E+08	92553000	52407000	72094000	84493000	1.02E+08	84193000	71516000
2.87E+08	2.71E+08	2.66E+08	2.85E+08	2.22E+08	2.25E+08	2.16E+08	2.18E+08	3.09E+08
76333000	81365000		80446000	70168000	78060000	59882000	67719000	44900000
1.69E+09	1.66E+09	1.67E+09	1.6E+09	1.72E+09	1.79E+09	1.66E+09	1.48E+09	1.46E+09
4.32E+08	4.78E+08	3.89E+08	3.35E+08	3.47E+08	4.58E+08	4.5E+08	3.74E+08	4.62E+08
3E+08	4.46E+08	3.02E+08	3.08E+08	2.74E+08	3.14E+08	3.83E+08	3.56E+08	3.45E+08
		39762000		32776000	40352000	1.2E+08	71804000	
1.96E+08	1.81E+08	4.07E+08	3.45E+08	3.24E+08	4.77E+08	3.3E+08	5.31E+08	1.07E+08
3.5E+08	4.59E+08	1.29E+09	1E+09	8.31E+08	1.45E+09	2.18E+09	2.44E+09	30053000
2.04E+10	2.2E+10	2.32E+10	2.52E+10	2.56E+10	2.2E+10	1.96E+10	2.32E+10	1.5E+10
59547000		80391000		67399000	1.11E+08	85508000	1.01E+08	
4.71E+08	5.89E+08		3.54E+08	3.44E+08	4E+08	3.88E+08	3.89E+08	5.29E+08
3.79E+08	3.85E+08	3.54E+08	3.35E+08	3.3E+08	4.11E+08	3.91E+08	3.69E+08	3.74E+08
1.98E+09	2.16E+09	4E+09	3.57E+09	3.01E+09	5.51E+09	5.49E+09	5.53E+09	2.45E+09
4.85E+08	3.82E+08	2.68E+08	3.86E+08	3.65E+08	3.63E+08	3.42E+08	3.08E+08	4.17E+08
77533000		83673000	97452000	88789000	98833000	69847000		43918000
1.24E+10	1.24E+10	9.69E+09	8.71E+09	8.61E+09	5.99E+09	6.77E+09	5.28E+09	1.38E+10
6.79E+09	9.18E+09	9.7E+09	8.1E+09	9.03E+09	1.15E+10	1.08E+10	9.47E+09	5.88E+09
7.75E+09	7.3E+09	6.55E+09	6.56E+09	7.56E+09	6.18E+09	6.08E+09	5.66E+09	7.37E+09
3.1E+08	3.7E+08	3.31E+08	3.36E+08	2.9E+08	3.78E+08	3.17E+08	3.51E+08	2.63E+08
1.48E+08	1.3E+08	78563000	95432000	1.52E+08	1.64E+08	1.49E+08	1.08E+08	1.44E+08
84988000	1.16E+08	57564000	79754000	52913000	52077000	35484000	39537000	1.01E+08
				62887000		86281000		
58556000	66264000				99755000		55090000	
9.03E+08	1.04E+09	7.48E+08	6.11E+08	7.57E+08	9.33E+08	8.89E+08	8.6E+08	8.43E+08
1.16E+08	1.31E+08	89756000	1.14E+08	99873000	1.22E+08		99486000	1.35E+08
9.19E+09	8.38E+09	1.02E+10	6.28E+09	8.35E+09	9.22E+09	7.93E+09	7.75E+09	5.29E+09
1.74E+09	1.6E+09	2.21E+09	2.04E+09	2.11E+09	2.15E+09	2.18E+09	2.4E+09	1.67E+09
85324000	65513000	1.05E+08	67440000	69571000	61391000	96137000	82062000	72673000
8.04E+08	7.19E+08	8.78E+08	1.02E+09	1.08E+09	1.12E+09	1.3E+09	1.12E+09	8.48E+08
4.4E+08	3.33E+08	3.36E+08	2.69E+08	3.54E+08	4.51E+08	4.21E+08	3.39E+08	3.16E+08
		2.96E+08	2.48E+08	2.74E+08	2.68E+08	2.76E+08	2.36E+08	
46954000	36593000		38542000					20794000
3.2E+09	3.5E+09	1.33E+09	1.41E+09	1.51E+09	3.43E+08	7E+08	3.9E+08	2E+09
78854000	1.12E+08	65183000	57894000	46611000	55015000	48656000	62025000	82205000
1.24E+09	1.23E+09	9.88E+08	7.74E+08	8.49E+08	1.36E+09	1.06E+09	1.11E+09	9.94E+08
6.18E+08	4.97E+08	6.19E+08	6.52E+08	5.79E+08	6.23E+08	4.98E+08	5.73E+08	4.18E+08
4.13E+08	4.04E+08	4.78E+08	4.64E+08	3.75E+08	4.61E+08	4.42E+08	4.87E+08	3.28E+08
	1.43E+08	1.28E+08	1.53E+08	1.48E+08	1.45E+08		1.08E+08	
4.51E+08	5.37E+08	3.16E+08	3.25E+08	3.37E+08	3.66E+08	4.2E+08	4.53E+08	5.93E+08
1.84E+08	1.86E+08	2.38E+08	2.67E+08	2.26E+08	3.19E+08	2.53E+08	2.7E+08	1.33E+08
	3.22E+08	86577000	3.19E+08	3.49E+08	3.54E+08	4.34E+08	3.85E+08	5.93E+08

2.95E+08	3.32E+08	2.89E+08	2.96E+08	2.98E+08	2.34E+08	4.34E+08	3.92E+08	4.43E+08
69188000	1.11E+08	87382000	86874000	76941000	78070000	81215000	1.04E+08	1.17E+08
1.07E+09	1.14E+09	1.01E+09	9.63E+08	9.8E+08	1.25E+09	1.08E+09	1.17E+09	1.13E+09
1.15E+09	1.51E+09	9E+08	9.27E+08	8.35E+08	1.35E+09	1.24E+09	1.2E+09	1.06E+09
52101000	63689000	26475000	40571000	59139000	58201000	51470000	48065000	52206000
4.66E+08	7.94E+08	5.41E+08	4.36E+08	3.93E+08	4.75E+08	3.24E+08	5.27E+08	5.04E+08
1.48E+09	1.96E+09	1.47E+09	1.36E+09	1.4E+09	1.71E+09	1.85E+09	1.68E+09	1.59E+09
3.3E+08	3.54E+08	2.96E+08	4.75E+08	3.78E+08	3.89E+08	4.45E+08	4.87E+08	4.14E+08
2.1E+08	2.18E+08	1.83E+08	2.75E+08	4.32E+08	3.27E+08	3.87E+08	4.07E+08	2.15E+08
		12996000	12110000	12717000	12827000		7921200	8182400
9.89E+08	9.22E+08	8.6E+08	7.2E+08	8.24E+08	5.24E+08	3.92E+08	4.83E+08	1.09E+09
6.78E+08	7.76E+08	1.15E+09	9.65E+08	1.01E+09	1.02E+09	1.14E+09	1.1E+09	7.02E+08
1.27E+09	1.49E+09	1.18E+09	1.16E+09	1.24E+09	1.18E+09	1.35E+09	1.22E+09	1.4E+09
1.74E+08	1.93E+08	2.35E+08	2.3E+08	1.88E+08	3.28E+08	2.09E+08	2.29E+08	2.08E+08
4.51E+08	3.65E+08	3.29E+08	3.69E+08	4.02E+08	2.78E+08	3.42E+08	3.09E+08	3.45E+08
9.99E+09	9.04E+09	6.1E+09	6E+09	6.78E+09	5.49E+09	6.32E+09	5.93E+09	1.26E+10
7.94E+08	9E+08	6.12E+08	6.31E+08	5.37E+08	4.86E+08	4.77E+08	5.3E+08	8.74E+08
1.15E+08		1.16E+08	1.13E+08	1.05E+08	1.8E+08		1E+08	92515000
1.28E+09	1.23E+09	1.26E+09	1.13E+09	1.26E+09	1.21E+09	1.15E+09	1.26E+09	1.22E+09
6.8E+08	6.12E+08	6.92E+08	6.15E+08	5.71E+08	5.45E+08	5.68E+08	6.4E+08	6.84E+08
1.91E+08	1.91E+08	2.47E+08	2.58E+08	2.3E+08	2.71E+08	3.1E+08	3.2E+08	2.49E+08
1.6E+09	1.5E+09	1.76E+09	1.39E+09	1.52E+09	1.96E+09	2.03E+09	1.75E+09	1.61E+09
3.67E+08	3.94E+08	3.94E+08	3.59E+08	2.93E+08	4.07E+08	3.89E+08	4.18E+08	4.33E+08
1.04E+08	75611000	1.32E+08	1.13E+08	1.1E+08	1.86E+08	1.31E+08		76918000
		97929000	1.18E+08	1.8E+08	2.34E+08		1.5E+08	
1.73E+08	2.34E+08	2.14E+08	1.72E+08	1.7E+08	1.32E+08	1.67E+08	1.53E+08	2.06E+08
4.3E+08	4.47E+08	5.01E+08	4.29E+08	3.97E+08	4.92E+08	4.06E+08	4.91E+08	4.63E+08
12044000	16546000	17945000	14131000	17055000				
1.25E+09	1.22E+09	1.69E+09	1.92E+09	1.77E+09	2.23E+09	2.25E+09	2.09E+09	9.86E+08
6.23E+09	4.27E+09	5.04E+09	6.01E+09	7.76E+09	8.21E+09	6.95E+09	5.57E+09	3.66E+09
1.04E+09	1.06E+09	2.29E+09	1.67E+09	1.63E+09	1.1E+09	1.46E+09	1.66E+09	7.87E+08
1.45E+09	1.99E+09	1.74E+09	1.65E+09	1.92E+09	2.18E+09	2.52E+09	1.78E+09	9.68E+08
1.27E+09	6.37E+08	1.41E+09	1.78E+09	1.57E+09	1.27E+09	7.98E+08	1.25E+09	4.9E+08
2.84E+08		1.92E+08			2.2E+08			2.92E+08
6.58E+09	7.22E+09	1.21E+10	1.1E+10	1.31E+10	1.19E+10	1.14E+10	1.06E+10	4.12E+09
24477000		81655000	48151000	1.2E+08	41433000	96336000	66666000	23759000
6.01E+09	6.7E+09	6.22E+09	4.98E+09	4.96E+09	4.65E+09	4.89E+09	5.12E+09	3.32E+09
2.39E+09	3.9E+09	5.9E+09	2.74E+09	3.53E+09	2.54E+09	7.88E+09	3.44E+09	1.74E+09
2.11E+09	1.84E+09	4.91E+09	4.87E+09	4.56E+09	3.47E+09	2.73E+09	3.87E+09	1.32E+09
7.69E+09	7.7E+09	5.94E+09	5.21E+09	5.07E+09	4.97E+09	6.14E+09	3.72E+09	4.53E+09
6.28E+09		1.29E+09	1.02E+09	1.23E+09	6.81E+08	6.98E+08		
2.58E+09	2.45E+09	1.77E+09	1.75E+09	2.19E+09	2.62E+09	2.15E+09	1.78E+09	1.68E+09
6.82E+08	7.17E+08	8.1E+08	8.8E+08	8.1E+08	1.14E+09	9.67E+08	1.15E+09	7.84E+08
2.8E+08	2.32E+08	2.59E+08	3.05E+08	2.63E+08	2.43E+08	2.46E+08	2.76E+08	3.29E+08
2.07E+08	1.71E+08	2.2E+08	1.81E+08	2.8E+08	2.19E+08	2.49E+08	2.75E+08	1.96E+08
1.75E+08	59267000	92606000	1.03E+08	1.61E+08	1.44E+08	94992000	66869000	
2.47E+09	2.32E+09	2.15E+09	2.07E+09	2.16E+09	2.63E+09	2.75E+09	2.72E+09	2.64E+09
9.99E+08	9.57E+08	1.31E+09	1.27E+09	1.17E+09	1.62E+09	1.4E+09	1.44E+09	1.03E+09
26654000	25354000	24720000	32668000	31361000	41592000	28199000	47203000	39448000

3.36E+08		1.84E+08	2.85E+08	2.43E+08				
2.31E+09	2.32E+09	3.66E+09	4.03E+09	3.35E+09	4.63E+09	4.26E+09	4.61E+09	1.91E+09
2.1E+09	2.51E+09	2.4E+09	2.02E+09	2.45E+09	2.88E+09	3.04E+09	2.4E+09	1.49E+09
1.73E+09	1.31E+09	2.03E+09	3.07E+09	3.32E+09	2.8E+09	1.91E+09	2.44E+09	9.53E+08
12370000	31965000	54544000	46746000	50261000	57272000	1.46E+08	62810000	24562000
		1.4E+08	66235000	59302000	58735000		42609000	
							2.5E+08	1.68E+08
8.89E+08	8.04E+08	1.95E+09	3.31E+09	2.26E+09	1.78E+09	1.44E+09	1.74E+09	5.03E+08
2.11E+08			1.42E+08	1.64E+08	2.28E+08	2.01E+08	1.84E+08	1.94E+08
5.99E+09	5.7E+09	2.27E+09	2.54E+09	3.11E+09	2.54E+09	2.44E+09	1.58E+09	5.15E+09
1.88E+08	1.55E+08	2.83E+08	2.76E+08	2.7E+08	3.83E+08	2.38E+08	3.45E+08	2.03E+08
4.93E+08	6.36E+08	1.03E+09	1.1E+09	9.7E+08	1.38E+09	1.14E+09	1.3E+09	5.66E+08
				58080000				
		95916000	86541000	86991000		98160000	90188000	80973000
5.21E+09	3.81E+09	7.07E+09	6.68E+09	7.36E+09	7.84E+09	7.67E+09	1.03E+10	4.6E+09
4.33E+08	4.09E+08	2.96E+08	4.41E+08	4.45E+08	4.25E+08	5.69E+08	4.41E+08	5.06E+08
2.1E+08	1.94E+08	2.19E+08	3.05E+08	2.41E+08	3.15E+08	2.77E+08	2.58E+08	2.02E+08
8.39E+09	1.29E+10	1.27E+10	9.73E+09	8.84E+09	1.45E+10	1.21E+10	1.6E+10	8.97E+09
1.62E+09	1.47E+09	2.6E+09	3.03E+09	3.09E+09	3.4E+09	3.86E+09	3.65E+09	1.71E+09
4.34E+08	4.78E+08	5.63E+08	4.55E+08	4.52E+08	4.92E+08	5.18E+08	5.44E+08	4.07E+08
1.61E+08		4.15E+08	4.55E+08	4.3E+08	1.91E+08	1.38E+08	1.26E+08	1.05E+08
85879000	82338000	1.07E+08	95671000		1.01E+08		93517000	87958000
1.35E+08	3.04E+08	1.25E+08	1.65E+08	2.42E+08	1.99E+08	4.62E+08	3.08E+08	1.97E+08
2.23E+08	2.14E+08	1.76E+08	1.47E+08	1.6E+08			2.52E+08	2.58E+08
4.49E+08	3.79E+08	3.86E+08	3.86E+08	5E+08	6.09E+08	5.42E+08	4.38E+08	4.68E+08
84255000	91779000		79305000	74799000	80991000	85358000	1.04E+08	88444000
3.04E+08	4.13E+08	2.99E+08	2.83E+08	2.96E+08	2.99E+08	3.75E+08	3.57E+08	2.74E+08
6.37E+08	6.56E+08	5.86E+08	5.76E+08	6.19E+08	5.35E+08	4.43E+08	4.85E+08	7.79E+08
25407000	31267000	24419000	24554000	26587000			27089000	31056000
	1.3E+08							1.48E+08
1.91E+08	1.73E+08	1.58E+08	1.52E+08	1.48E+08	1.2E+08	1.2E+08	1.5E+08	1.61E+08
1.55E+08	2.61E+08	1.18E+08	1.18E+08	1.35E+08	96554000	1.12E+08	1.09E+08	3.37E+08
1.21E+09	1.47E+09	7.63E+08	8.53E+08	8.43E+08	7.63E+08	7.81E+08	6.45E+08	1.55E+09
95601000		85949000	1.11E+08	86887000	86872000	92465000	91107000	94054000
5.07E+08	4.49E+08	1.05E+09	1.18E+09	1.17E+09	1.15E+09	9.88E+08	1.13E+09	4.62E+08
52260000		46000000	54995000		81121000			
5.21E+08	4.08E+08	6.69E+08	5.08E+08	4.65E+08	4.48E+08	4.19E+08	4.41E+08	4.59E+08
6.47E+08	5.33E+08	5.87E+08	7.39E+08	5.84E+08	5.13E+08	3.68E+08	4.18E+08	4.74E+08
5.41E+08	5.04E+08	5.14E+08	3.28E+08	4.29E+08	3.79E+08	3.27E+08	4.01E+08	
1.59E+09	1.78E+09	1.39E+09	1.33E+09	1.38E+09	6.96E+08	7.91E+08	9.03E+08	2.25E+09
				72792000	40860000			
1.17E+08	1.69E+08	1.8E+08	2.51E+08	1.84E+08	3.47E+08	1.5E+08	3.32E+08	
						1.02E+08		
2.11E+08	1.49E+08	1.41E+08	1.47E+08	1.65E+08	1.86E+08	1.57E+08	1.44E+08	2.68E+08
2.95E+09	2.4E+09	2.03E+09	2.1E+09	2.43E+09	2.68E+09	2.36E+09	2.15E+09	2.2E+09
1.32E+09	1.2E+09	1.28E+09	1.17E+09	1.03E+09	1.33E+09	1.28E+09	1.25E+09	1.18E+09
		18333000		12884000				

	1.46E+08							1.68E+08
4.98E+08	6.1E+08	3.23E+08	2.94E+08	3.93E+08	4.98E+08	5.68E+08	2.43E+08	4.91E+08
	1.01E+08			53360000				
	1.17E+08	1.47E+08	1.36E+08	1.27E+08	1.05E+08	85601000	1.6E+08	1.06E+08
76380000	70469000	66350000	27147000	41025000	40582000	42641000	71742000	1.47E+08
	42330000	28432000	57508000	54762000	48319000		34815000	
1E+08	1.21E+08	1.39E+08	1.19E+08	1.31E+08	1.95E+08	1.94E+08		1.1E+08
2.3E+08	1.19E+08	2.09E+08	2.23E+08	2.44E+08	2.86E+08	1.72E+08	2.2E+08	1.25E+08
5.97E+08	7.16E+08	6.47E+08	5.49E+08	4.88E+08	7.3E+08	7.05E+08	7.19E+08	4.54E+08
4.11E+09	3.87E+09	2.99E+09	4.11E+09	4.11E+09	3.22E+09	4.71E+09	3.92E+09	5.53E+09
37025000		25346000	30150000	32667000	58314000	63138000	41704000	
1.96E+08	1.82E+08	1.69E+08	1.23E+08	1.58E+08	1.34E+08	1.18E+08	1.26E+08	2.42E+08
2.37E+08	1.99E+08	3.82E+08	3.84E+08	2.45E+08	4.2E+08	4.16E+08	3.19E+08	2.38E+08
1.44E+08	1.85E+08	1.15E+08	1.97E+08	2.11E+08	2.03E+08	2.49E+08	2.6E+08	1.65E+08
4.6E+08	3.12E+08	2.16E+08	2.74E+08	2.92E+08	2.98E+08	2.56E+08	2.4E+08	4.01E+08
2.1E+09	3.41E+09	6.63E+09	6.27E+09	6.18E+09	7.04E+09	7.06E+09	8.39E+09	2.35E+09
7.99E+09	5.74E+09	1.69E+10	1.69E+10	1.69E+10	1.65E+10	1.26E+10	1.21E+10	3.99E+09
85688000	1.22E+08	1.6E+08	2.01E+08	2.02E+08	1.89E+08	1.18E+08	1.21E+08	1.1E+08
1.64E+09	1.78E+09	1.54E+09	2.09E+09	1.68E+09	1.7E+09	1.8E+09	1.99E+09	1.6E+09
3.06E+08	3.53E+08	3.1E+08	2.92E+08	2.39E+08	2.97E+08	4.06E+08	4.24E+08	2.9E+08
			91738000			1.18E+08	1.13E+08	
		2.28E+08	90591000	2E+08	3.09E+08	2.76E+08		5.94E+08
26997000	46085000	13944000	14367000	18683000	17523000	26830000	32365000	67279000
46978000	60993000						14428000	77005000
7.54E+08	6.66E+08	4.61E+08	4.36E+08	4.96E+08	3.9E+08	3.9E+08	3.48E+08	6.95E+08
1.02E+08	1.03E+08	1.09E+08	1.19E+08	1.19E+08	1.44E+08		97326000	1.22E+08
1.19E+09	1.18E+09	1.59E+09	1.28E+09	1.65E+09	1.05E+09	1.1E+09	7.7E+08	1.51E+09
5.28E+08	6.1E+08	4.71E+08	5.54E+08	5.31E+08	4.96E+08	5.68E+08	6.08E+08	5.22E+08
1.08E+08	1.07E+08	1.07E+08	1.15E+08	94999000	1.13E+08	1.3E+08	1.08E+08	1.09E+08
1.75E+09	8.59E+08	3.78E+08	1.06E+09	7.08E+08	5.3E+08	6.05E+08	5.15E+08	1.98E+09
2.39E+08	2.05E+08	1.75E+08	1.67E+08	1.52E+08	1.79E+08	2E+08	1.93E+08	1.8E+08
4.3E+08	4.38E+08	6.12E+08	6.64E+08	7.1E+08	6.29E+08	4.72E+08	6.04E+08	4.76E+08
1.46E+08	1.35E+08	1.82E+08	2.58E+08	2.1E+08	2.1E+08	1.56E+08	2.32E+08	1.18E+08
2.49E+08	2.45E+08	2.55E+08	2.34E+08	2.33E+08	2.46E+08	1.99E+08	2.18E+08	2.39E+08
4.08E+08		7.09E+08	7.68E+08	6.56E+08	6.57E+08	5.32E+08	4.57E+08	
51980000	55204000	47577000	45965000	51211000	52161000	39393000	49753000	43538000
7.95E+08	9.92E+08	6.3E+08	9.41E+08	5.51E+08	7.36E+08	7.93E+08	7.8E+08	7.76E+08
7.75E+08	5.82E+08	4.43E+08	2.5E+08	3.32E+08	2.46E+08	2.22E+08	1.84E+08	7.85E+08
64039000	43356000	28286000		38493000		41089000		42678000
64670000	94050000						46136000	61709000
2.33E+08	2.83E+08	2.7E+08	1.71E+08	1.96E+08	1.06E+08	1.56E+08	2.64E+08	2.5E+08
1.18E+09	1.05E+09	1.21E+09	1.26E+09	1.23E+09	1.41E+09	1.33E+09	1.54E+09	1.06E+09
5.18E+08	4.57E+08	3.85E+08	3.94E+08	4.42E+08	4.64E+08	5.37E+08	4.15E+08	4.56E+08
1.29E+09	7.86E+08	1.23E+09	1.23E+09	1.22E+09	1.3E+09	1.39E+09	1.64E+09	1.19E+09
5.15E+08	3.95E+08	4.8E+08	3.41E+08	3.26E+08	3.9E+08	2.83E+08	3.94E+08	5.98E+08
1.44E+08	1.61E+08	2.39E+08	2.51E+08	2.41E+08	2.6E+08	2.6E+08	3.13E+08	1.8E+08
3.88E+08	4.43E+08	3.31E+08	2.81E+08	2.52E+08	2.17E+08	2.05E+08	1.96E+08	4.09E+08
6.5E+09	4.81E+09	7.79E+09	6.31E+09	6.43E+09	4.72E+09	4.31E+09	3.84E+09	7.37E+09
2.4E+09	2.19E+09	2.05E+09	2.56E+09	3.01E+09	4.25E+09	3E+09	2.21E+09	1.59E+09

		57310000	31228000	31817000				33947000
1.3E+08	1.38E+08	1.05E+08	1.18E+08	1.18E+08	1.43E+08	1.42E+08	1.69E+08	1.48E+08
1.02E+10	3.04E+09	4.7E+09	9.93E+09	7.3E+09	4.42E+09	4.09E+09	4.61E+09	3.53E+09
2.1E+08	3.16E+08	3.07E+08	2.33E+08	2.81E+08	2.37E+08	4.48E+08	6.1E+08	2.65E+08
				59979000	40105000			
1.94E+08	1.9E+08	2.46E+08	3.34E+08	3.2E+08	3.23E+08	2.8E+08	3.22E+08	1.95E+08
15424000	23797000	44617000	58707000	43713000	85637000	88963000	70455000	24217000
3.54E+08	1.84E+08	3.41E+08	4.6E+08	4.76E+08	4.68E+08	2.76E+08	3.85E+08	1.94E+08
6.04E+08	7.16E+08	4.28E+08	4.1E+08	4.11E+08	4.53E+08	7.13E+08	8.07E+08	7.33E+08
7.01E+08	7.6E+08	7.02E+08	3.79E+08	4.08E+08	5.32E+08	5.51E+08	5.9E+08	7.39E+08
62001000	63520000		65778000	64595000	60400000		71501000	76839000
3.52E+09	3.29E+09	2.62E+09	3.09E+09	4.3E+09	3.18E+09	5.86E+09	6.86E+09	5.2E+09
1.06E+09	9.35E+08	9.68E+08	1.01E+09	1.18E+09	1.39E+09	1.29E+09	1.24E+09	1.09E+09
2.79E+08	2.96E+08	6.39E+08	2.56E+08	4.14E+08	3.35E+08	7.41E+08	4.34E+08	3.18E+08
4.62E+08	6.26E+08	3.15E+08	2.4E+08	2.36E+08	3.97E+08	3.76E+08	3.03E+08	5.14E+08
4.22E+08	4.31E+08	3.38E+08	3.28E+08	2.85E+08	3.6E+08		4.49E+08	3.48E+08
80897000	78015000			85218000	94886000	1.21E+08	1.01E+08	68536000
1.43E+08		2.69E+08	1.67E+08	3.57E+08	5.64E+08	7.58E+08	5.73E+08	1.44E+08
1.51E+08	1.7E+08	1.58E+08	1.36E+08	1.22E+08	2.18E+08	1.21E+08	1.54E+08	1.41E+08
1.48E+09	2.14E+09	6.95E+08	6.95E+08	8.08E+08	8.79E+08	8.19E+08	5.16E+08	1.35E+09
3.1E+08	2.14E+08	1.11E+08	1.52E+08	2.14E+08	1.63E+08	1.27E+08	1.09E+08	3.4E+08
5.8E+08	4.71E+08	4.49E+08	4.9E+08	4.76E+08	3.83E+08	1.45E+08	2.32E+08	4.47E+08
4.1E+08	4.7E+08	5.59E+08	4.02E+08	3.77E+08	5.25E+08	5.25E+08	5.16E+08	4.21E+08
1.44E+08	1.67E+08	1.53E+08	1.74E+08	1.3E+08	2.05E+08	2.3E+08	2.25E+08	1.82E+08
		30244000		45028000	23460000			
2.65E+08	2.75E+08	1.57E+08	2.13E+08	1.93E+08	3.29E+08	2.48E+08	2.21E+08	2.91E+08
					73681000		97247000	
1.21E+08	1.56E+08	91702000	56616000	1.01E+08	49459000		55961000	93745000
	85712000	64363000	85250000	46841000	56083000	84726000	59451000	90112000
		99567000			73888000	89151000	1.35E+08	
11614000		15159000	18924000	18563000				
4.27E+08	4.27E+08	3.66E+08	3.22E+08	3.91E+08	1.49E+08	1.16E+08	91424000	5.66E+08
			33939000		25145000			
5.96E+08	5.96E+08	5.36E+08	5.89E+08	6.84E+08	7.17E+08	6.98E+08	6.16E+08	8.07E+08
3.46E+08	2.19E+08	3.09E+08	3.25E+08	3.23E+08	2.66E+08	2.48E+08	2.55E+08	3.14E+08
7.08E+08	6.67E+08	5.08E+08	5.29E+08	5.45E+08	4.18E+08	5.13E+08	4.94E+08	9.07E+08
		27148000						
5.15E+08	5.42E+08	6.24E+08	5.82E+08	6.04E+08	6.35E+08	5.75E+08	6.95E+08	4.39E+08
77252000	96145000	68798000	74744000	58908000	64732000		73469000	83339000
2.97E+09	2.72E+09	2.05E+09	2.35E+09	2.06E+09	2.11E+09	2.37E+09	2.36E+09	3.05E+09
	1.82E+08	1.59E+08	1.49E+08	1.06E+08		1.38E+08		
3.59E+08	3.92E+08	2.69E+08	2.58E+08	2.63E+08	1.95E+08	2.92E+08	1.72E+08	3.49E+08
		23540000	18937000					
43214000	76148000	34707000		30304000			33290000	
1.15E+08	84358000		84482000	82764000	73447000			75074000
2.18E+09	2.2E+09	2.55E+09	2.4E+09	2.26E+09	2.67E+09	2.34E+09	2.32E+09	1.75E+09
2.75E+09	2.85E+09	3.66E+09	3.58E+09	3.67E+09	4.11E+09	3.54E+09	3.72E+09	1.83E+09

4.15E+09	3.25E+09	5.38E+09	6.1E+09	5.6E+09	9.43E+09	7.82E+09	8.35E+09	3.96E+09
2.34E+08	2.11E+08	1.31E+08	1.87E+08	2.34E+08	1.82E+08	1.76E+08	1.7E+08	2.14E+08
	46643000	40136000	53312000	53696000	77873000	82199000	73824000	49719000
8.01E+08	7.47E+08	5.6E+08	5.55E+08	6E+08	5.88E+08	5.8E+08	5.87E+08	6.74E+08
1.64E+09	1.39E+09	8.81E+08	1.39E+09	1.93E+09	1.38E+09	1.18E+09	1.64E+09	1.77E+09
68738000		79458000	74105000	93336000	1.06E+08	91032000	1.11E+08	59944000
79281000	52713000	1.44E+08	96624000	1.19E+08	1.67E+08	1.06E+08	1.29E+08	67305000
3.3E+08	2.9E+08	3.45E+08	3.84E+08	3.11E+08	3.45E+08	2.94E+08	2.9E+08	3.64E+08
1.4E+09	1.44E+09	8.87E+08	1.01E+09	1.11E+09	1.28E+09	9.86E+08	9.26E+08	1.15E+09
2.24E+08	1.96E+08	1.06E+08	1.12E+08	1.28E+08	1.33E+08	87098000	77118000	2.49E+08
1.85E+08	1.62E+08	2.46E+08	1.2E+08	1.87E+08	2.36E+08	2.18E+08	1.83E+08	1.17E+08
5.76E+08		2.87E+08	4.64E+08	3.97E+08				
4.52E+09	5.81E+09	6.4E+09	6.79E+09	6.22E+09	6.12E+09	5.19E+09	5.69E+09	4.59E+09
3.11E+09	3.61E+09	6.98E+09	5.45E+09	5.65E+09	3.54E+09	2.22E+09	4.11E+09	2.4E+09
	70356000	75085000	74256000	1.07E+08	47366000	1.46E+08	81411000	73402000
3.68E+08	4.51E+08	2.51E+08	2.88E+08	3.07E+08	2.97E+08	4.9E+08	3.68E+08	4.11E+08
2.76E+09	1.66E+09	3.14E+09	4.93E+09	3.61E+09	3.57E+09	2.95E+09	3.2E+09	1.94E+09
1.17E+10	1.18E+10	1.25E+10	1.72E+10	1.62E+10	1.63E+10	1.4E+10	1.39E+10	9.46E+09
8.27E+09	7.76E+09	8.37E+09	8.28E+09	9.88E+09	1.09E+10	1.17E+10	9.46E+09	6.8E+09
1.57E+09	1.62E+09	1.45E+09	1.84E+09	1.87E+09	1.48E+09	1.58E+09	1.63E+09	1.61E+09
6.84E+09	9.2E+09	7.43E+09	6.6E+09	7.08E+09	9.35E+09	8.9E+09	6.62E+09	5.66E+09
61743000		61809000	1.27E+08	1.42E+08	91272000	1.44E+08	44697000	54974000
1.15E+08	1.37E+08	88787000	1E+08	81869000	97731000	1.27E+08	1.12E+08	1.23E+08
1.2E+08	1.1E+08	1.19E+08	1.09E+08	96360000			1.27E+08	1.16E+08
		27021000			23447000		24064000	
1.33E+08	1.3E+08	1.99E+08	2.11E+08	2.01E+08	2.34E+08	2.7E+08	2.89E+08	1.55E+08
1.34E+08	1E+08	1.78E+08	3.74E+08	2.19E+08	1.84E+08	2.12E+08	2.34E+08	1.27E+08
		1.67E+08	2.34E+08	2.34E+08	1.78E+08	1.18E+08	94397000	
1.9E+08		1.38E+08	1.44E+08	1.44E+08	1.46E+08	1.21E+08	1.21E+08	1.6E+08
2.68E+08	3.03E+08	3.35E+08	3.22E+08	2.73E+08		2.67E+08	2.79E+08	
2.03E+08	2.88E+08	2.32E+08	2.41E+08	1.98E+08	1.83E+08	3.37E+08	2.91E+08	1.66E+08
2.8E+09	2.76E+09	3.53E+09	3.92E+09	3.9E+09	4.31E+09	4.28E+09	3.9E+09	2.8E+09
9.63E+08	5.83E+08	1.13E+09	1.11E+09	1.46E+09	1.36E+09	1.45E+09	1.45E+09	9.52E+08
	14186000	14412000	14425000					
4.94E+08	4.5E+08	4E+08	3.48E+08	3.29E+08	3.56E+08	3.42E+08	3.63E+08	4.77E+08
3.97E+08	3.07E+08	4.13E+08	4.24E+08	4.88E+08	5.88E+08	5.27E+08	5.48E+08	3.72E+08
2.74E+08	3.45E+08	4.29E+08	4.22E+08	3.5E+08	5.66E+08	5.13E+08	4.66E+08	3.33E+08
3.34E+08	4.1E+08	3.07E+08	2.68E+08	2.73E+08	2.8E+08	2.66E+08	3.55E+08	3.36E+08
68960000	60661000		60196000	50188000	57795000	61337000	55009000	
2.5E+08	3.61E+08	3.31E+08	2.85E+08	3.25E+08	3.4E+08	4.03E+08	3.85E+08	2.98E+08
1.01E+09	1.65E+09	1.65E+09	1.7E+09	1.65E+09	1.3E+09	1.21E+09	1.03E+09	

85556000	67672000	1.51E+08	78762000	72278000	1.34E+08	1.95E+08	1.51E+08	79576000
2.35E+08	1.97E+08	2.18E+08	2.57E+08	2.4E+08	2.13E+08	2.44E+08	2.79E+08	2.04E+08
99296000	1.23E+08	1.33E+08	1.36E+08	1.32E+08	1.68E+08	1.41E+08	1.52E+08	1.26E+08
1.7E+08	2.36E+08	3.08E+08	2.12E+08	1.94E+08	3.57E+08	3.59E+08	3.48E+08	1.27E+08
				41694000				
87052000	88865000	1.03E+08	85569000	73360000	1.08E+08	1.14E+08	95370000	65171000
		45105000	43357000	38653000	61585000	52890000	63385000	
5.18E+08	4.18E+08	4.21E+08	4.12E+08	4.24E+08	4.22E+08	3.9E+08	3.09E+08	3.97E+08
		1.45E+08	1.05E+08		85793000	34049000	97537000	
								2.2E+08
			76146000	63368000	66280000	1.04E+08	75683000	
6.41E+08	7.35E+08	6.13E+08	5.54E+08	5.58E+08	6.21E+08	6.07E+08	6.95E+08	7.94E+08
3.41E+08	3.34E+08	5.37E+08	4.61E+08	4.51E+08	5.94E+08	5.35E+08	7.01E+08	4.67E+08
2.94E+08	2.75E+08	2.98E+08	3.19E+08	2.66E+08	3.47E+08	4.37E+08	3.71E+08	2.87E+08
4.81E+08	4.72E+08	5.66E+08	3.95E+08	4.18E+08	5.39E+08	5.7E+08	6.45E+08	8.33E+08
	7.73E+08							2.68E+08
1.84E+08		2.83E+08	3.31E+08	2.95E+08	3.41E+08	2.98E+08	2.12E+08	44912000
1.78E+08	1.87E+08	3.79E+08	2.25E+08	2.69E+08	3.81E+08	4.25E+08	4.48E+08	3.28E+08
9.55E+09	8.1E+09	5.29E+09	7.36E+09	9.92E+09	7.9E+09	7.4E+09	7.14E+09	8.36E+09
2.45E+09	2.48E+09	3.77E+09	4.75E+09	4.5E+09	4.68E+09	3.6E+09	3.55E+09	1.62E+09
2.29E+09	3.22E+09	7.77E+09	6.5E+09	7.68E+09	1.27E+09	7.1E+08	4.77E+09	2.33E+09
6.2E+08	8.7E+08	9.84E+08	5.78E+08	6.19E+08	5.97E+08	1.07E+09	6.16E+08	5.78E+08
5.58E+09	6.31E+09	5.6E+09	4.46E+09	4.34E+09	3.72E+09	4.52E+09		4.45E+09
		52770000	43574000	52906000	27268000	39779000	33688000	
		12343000	10310000	11922000	8801900	79087000	9115400	
1.39E+09	1.25E+09	1.79E+09	1.84E+09	2.45E+09	8.35E+08	8.07E+08	2.05E+09	1.08E+09
7.85E+09	8.72E+09	9.94E+09	9.09E+09	7.94E+09	8.94E+09	8.35E+09	8.54E+09	5.57E+09
		2.63E+08	2.01E+08	2.44E+08				
3.96E+09	4.53E+09	6.61E+09	6.25E+09	6.99E+09	5.05E+09	5.99E+09	6.5E+09	3.69E+09
6.11E+09	8.16E+09	3.98E+09	3.58E+09	3.89E+09	4.32E+09	1.97E+09	3.68E+09	3.59E+09
1.62E+09	1.27E+09	1.9E+09	2.93E+09	3.04E+09	1.91E+09	1.55E+09	3.55E+09	1.1E+09
5.29E+09	5E+09	7.71E+09	8.46E+09	7.37E+09	5.87E+09	5.62E+09	6.35E+09	3.25E+09
1.61E+09	4.05E+09	1.25E+09	8.3E+08	9.91E+08	1.82E+09	1.41E+09	1.23E+09	1.74E+09
3.23E+09	3.63E+09	4.56E+09	5.11E+09	5.39E+09	6.2E+09	6.26E+09	5.68E+09	2.08E+09
9.11E+09	1.18E+10	8.62E+09	8.41E+09	8.78E+09	9.35E+09	6.91E+09	7.6E+09	6.54E+09
2.67E+08	2.52E+08	2.44E+08	2.81E+08	2.17E+08	3.17E+08	2.2E+08	3.23E+08	3.31E+08
56257000		2.54E+08	82003000	93032000	2.6E+08	3.4E+08	2.5E+08	84395000
42648000	51214000	56881000	48069000	58469000	73209000		61683000	49349000
8.72E+08	8.51E+08	8.94E+08	7.25E+08	9.43E+08	7.89E+08	1.14E+09	1.14E+09	9.5E+08
3.37E+08	2.14E+08	2.77E+08	2.76E+08	2.48E+08	2.37E+08	1.77E+08	2.67E+08	1.82E+08
			52799000	68016000		1.07E+08	49632000	37535000
1.29E+08		1.04E+08	1.4E+08	1.54E+08			94953000	1.25E+08
5.2E+08	5.45E+08	5.52E+08	6.07E+08	6.02E+08	6.59E+08	7.02E+08	7.42E+08	5.31E+08
9.27E+08	7.59E+08	7.5E+08	1.01E+09	1.04E+09	1.48E+09	1.16E+09	1.16E+09	9.87E+08
1.53E+08	1.67E+08	1.31E+08	1.92E+08	1.68E+08	1.54E+08	1.57E+08	1.92E+08	1.56E+08
1.39E+09	1.96E+09	1.79E+09	1.49E+09	1.49E+09	1.61E+09	1.87E+09	1.63E+09	1.58E+09

1.03E+08	1.08E+08	1.16E+08	1.09E+08	1.03E+08	1.17E+08	1.22E+08	1.15E+08	89878000
1.01E+08	1.16E+08	1.01E+08	1.2E+08	1.17E+08	1.34E+08	1.34E+08	1.28E+08	1.19E+08
5.29E+09	5.74E+09	1E+10	5.88E+09	6.23E+09	8.31E+09	7.79E+09	7.16E+09	1.1E+10
7.99E+08	5.89E+08	8.37E+08	1.06E+09	8.8E+08	2.12E+08	97837000	3.03E+08	2.89E+08
8.59E+08	7.86E+08	8.94E+08	8.07E+08	7.28E+08	9.6E+08	1.12E+09	1.03E+09	8.5E+08
8.03E+09	6.4E+09	8.54E+09	7.83E+09	7.8E+09	7.7E+09	7.26E+09	6.94E+09	7.65E+09
6.55E+08	6.62E+08	7.6E+08	7.17E+08	8.23E+08	1.21E+09	9.49E+08	8.23E+08	9.95E+08
2.65E+09	2.77E+09	3.02E+09	4.32E+09	3.57E+09	3.83E+09	4.41E+09	3.9E+09	2.68E+09
					23513000			
5.96E+08	6.16E+08	6.69E+08	5.41E+08	5.81E+08	6.35E+08	5.24E+08	5.84E+08	6.7E+08
2.2E+09	1.61E+09	2.83E+09	3.34E+09	3.21E+09	3.29E+09	1.58E+09	2.23E+09	1.55E+09
2.34E+09	2.41E+09	9.22E+09	8.86E+09	6.85E+09	3.84E+09	4.62E+09	3.95E+09	1.45E+09
1.09E+09	1.35E+09	1.08E+09	9.97E+08	1.31E+09	1.24E+09	2.14E+09	1.23E+09	1.09E+09
2.3E+09	2.99E+09	5.5E+09	3.38E+09	3.72E+09	3.57E+09	2.19E+09	2.43E+09	1.59E+09
7.5E+08	3.01E+08	4.96E+08	7.1E+08	5.98E+08	3.81E+08	3.08E+08	3.12E+08	2.22E+08
1.7E+08	1.71E+08	2.51E+08	2.32E+08	2.32E+08	2.68E+08	3.74E+08	2.5E+08	1.67E+08
5.25E+08	5.47E+08	5.91E+08	4.82E+08	3.98E+08	5.63E+08	4.65E+08	7.8E+08	7.06E+08
3.26E+08	2.97E+08	2.64E+08	3.01E+08	2.7E+08	2.6E+08	2.75E+08	2.85E+08	3.42E+08
3.49E+09	2.8E+09	2.26E+09	2.46E+09	2.26E+09	1.78E+09	1.84E+09	1.92E+09	3.44E+09
2.89E+08	2.57E+08	1.48E+08	2.61E+08	1.5E+08	2.34E+08	2.58E+08	1.98E+08	2.99E+08
2.5E+08	3.05E+08	1.98E+08	1.84E+08	1.54E+08	1.55E+08	1.83E+08	1.91E+08	2.71E+08
3.41E+08	4.49E+08	3.9E+08	3.11E+08	2.66E+08	4.13E+08	3.59E+08	3.8E+08	3.47E+08
3.81E+08	4.54E+08	4.9E+08	5.4E+08	3.51E+08	5.56E+08	4.49E+08	5.92E+08	4.36E+08
5.21E+08	6.76E+08	5.45E+08	4.73E+08	4.04E+08	6.19E+08	7.36E+08	5.69E+08	6.69E+08
								29400000
	6.73E+08							7.29E+08
1.5E+08	1.63E+08	1.47E+08	2.14E+08	1.68E+08	2.69E+08	2.73E+08	2.66E+08	1.65E+08
1.78E+08	1.84E+08	1.12E+08			1.37E+08	1.49E+08	1.18E+08	1.98E+08
2.9E+09	1.91E+09	2.27E+09	3.21E+09	3.29E+09	3.29E+09	3.18E+09	3.46E+09	3.25E+09
1.7E+09	1.29E+09	1.71E+09	2.23E+09	2.2E+09	2.2E+09	1.69E+09	1.97E+09	2.25E+09
2.23E+08	2.48E+08	1.89E+08	1.8E+08	1.65E+08	1.73E+08	1.95E+08	1.84E+08	2.29E+08
1.07E+08		1.13E+08	1.21E+08	1.12E+08	82626000	79387000	58768000	1.33E+08
1.78E+08	2.01E+08	1.76E+08	1.46E+08	1.69E+08	2.84E+08	2.7E+08	2.42E+08	1.93E+08
4.69E+08	4.15E+08	4.12E+08	4.08E+08	3.8E+08	4.85E+08	3.99E+08	4.13E+08	3.92E+08
	34860000	47211000	44690000	36482000	46013000	40721000	36916000	27739000
4.66E+08	5.36E+08	3.8E+08	4E+08	3.35E+08	3.81E+08	4.82E+08	4.57E+08	5.9E+08
2.9E+08	2.59E+08	3.51E+08	3.67E+08	3.36E+08	4.08E+08	4.22E+08	3.82E+08	2.18E+08
1.79E+08	1.3E+08	1.51E+08	2.54E+08	2.2E+08	2.21E+08	2.39E+08	3.3E+08	1.91E+08
							33752000	
		30844000	35225000	34600000			38434000	
1.02E+09	9.34E+08	7.3E+08	7.96E+08	6.88E+08	1.11E+09	9.79E+08	1.09E+09	1.03E+09
		57845000	93680000	82276000	94497000		78853000	
89084000	82456000	78565000	1.42E+08	1.35E+08	1.56E+08		1.27E+08	56841000
5.69E+09	4.31E+09	6.14E+09	6.84E+09	7.57E+09	6.29E+09	3.46E+09	4.32E+09	3.89E+09
1.16E+08	1.36E+08	2.28E+08	1.74E+08	1.52E+08	2.23E+08	2.05E+08	2E+08	1.02E+08

93055000	1.49E+08	1.61E+08	1.22E+08	1.07E+08	1.36E+08	1.1E+08	1.37E+08	1.39E+08
		57860000	28180000			60355000	38720000	
	78969000	2.52E+08	2.98E+08	2.9E+08	1.01E+08	77818000		
4.11E+08	4.78E+08	3.59E+08	3.13E+08	2.54E+08	2.99E+08	3.29E+08	3.27E+08	4.06E+08
1.39E+08	1.66E+08	1.88E+08	2.11E+08	2.09E+08	1.88E+08	1.82E+08	2.83E+08	1.67E+08
1.32E+08	1.66E+08	1.5E+08	1.52E+08	1.15E+08	1.43E+08	1.59E+08	1.37E+08	1.13E+08
8.23E+08	8.92E+08	7.94E+08	7.73E+08	8.16E+08	8.21E+08	8.82E+08	7.77E+08	6.78E+08
5.99E+08	6.34E+08	5.37E+08	5.02E+08	4.77E+08	6.55E+08	6.94E+08	7.68E+08	6.4E+08
41930000	39501000							57370000
5.71E+08	4.25E+08	5.4E+08	5.33E+08	4.29E+08	3.72E+08	3.24E+08	4.08E+08	4.22E+08
9.57E+08	9.1E+08	7.01E+08	9.02E+08	8.72E+08	8.44E+08	6.77E+08	6.68E+08	9.82E+08
	5.49E+08		3.61E+08	2.5E+08	3.42E+08		3.55E+08	4.8E+08
6.69E+08	6.64E+08	3.65E+08	4.39E+08	3.96E+08	4.2E+08	3.96E+08	4.17E+08	6.15E+08
	58833000	42923000		40400000		57001000	56170000	
2.2E+08	1.91E+08	2.17E+08		1.6E+08	2.09E+08	1.89E+08	2.05E+08	2.44E+08
		1.35E+08						
1.83E+08	1.66E+08	1.65E+08	2.06E+08	2.21E+08		91333000	1.4E+08	2.47E+08
4.46E+08	4.56E+08	3.16E+08	3.81E+08	4.15E+08	4.17E+08	3.18E+08	3.54E+08	3.84E+08
57153000	57333000		17409000	23903000				1.07E+08
68464000	45781000	52408000	59552000	75025000	71818000	57573000	65138000	65608000
97880000	95225000	1.26E+08	1.11E+08	97798000	64491000	43630000	62609000	1.87E+08
2.08E+08	2.18E+08		1.7E+08					2.36E+08
5.38E+08	5.49E+08	4.95E+08	3.97E+08	4.08E+08	3.66E+08	3.16E+08	3.47E+08	5.31E+08
84942000	91693000	1.11E+08	93423000	77882000	93809000	90662000	1.03E+08	95716000
7.93E+08	8.47E+08	9.55E+08	9.11E+08	8.89E+08	7.62E+08	7.83E+08	9.94E+08	7.94E+08
1.31E+08	2.17E+08	1.72E+08	1.13E+08	1.04E+08	1.65E+08		1.52E+08	2.11E+08
4.18E+08	3.86E+08	6.92E+08	6.25E+08	5.31E+08	4E+08	4.88E+08	4.88E+08	5.96E+08
65919000		1.54E+08	66662000	61161000	1.19E+08	99654000	72840000	51918000
					2.3E+08			
1.71E+09	1.74E+09	2.37E+09	3.24E+09	2.41E+09	1.8E+09	2.04E+09	2.48E+09	2.22E+09
	94709000		81770000		81235000	64810000		70392000
	44457000	39659000			54190000		47584000	35218000
65368000	75938000	74626000	59783000	61955000	54006000	39106000	67999000	89971000
	25191000	25943000	32197000	32894000	29341000		30547000	
1.85E+08	94055000	2.57E+08	2.8E+08	2.54E+08	2.94E+08	1.9E+08	2.29E+08	1.36E+08
3.63E+08	5.41E+08	1.01E+08	1.64E+09	4.73E+08	8.79E+08	7.91E+08	1.15E+09	
			38013000	33208000	43968000			
	1.61E+08	1.73E+08			1.5E+08	2.59E+08		
		31781000	67431000	27006000	35021000	65369000	40938000	
7.09E+08	3.76E+08	4.36E+08	5.55E+08	4.46E+08	3.36E+08	2.2E+08		6.85E+08
2.82E+08	2.58E+08	2.5E+08	2.12E+08	2.33E+08	4.12E+08	3.86E+08	2.79E+08	2.29E+08
		2E+08	2.49E+08	2.24E+08				
1.56E+08	2.04E+08		74431000			1.18E+08	1.59E+08	1.43E+08
	26852000			25704000			25325000	67276000

60031000	52680000	80447000	77405000	90849000	80369000	63750000	84788000	65809000
		1.54E+08	1.57E+08	1.38E+08	1.67E+08	1.66E+08	1.71E+08	
1.62E+08	1.52E+08	2.14E+08	2.03E+08	1.9E+08	3.22E+08	3.76E+08	3.51E+08	1.55E+08
1.55E+08	1.72E+08	1.3E+08	1.86E+08	1.13E+08	96076000	90760000	86115000	1.62E+08
49586000	88090000			45138000	28459000	37715000	35215000	61265000
43054000	46176000	39041000	40364000	31461000	35989000		47356000	44298000
2.71E+08	2.35E+08	4.95E+08	4.71E+08	4.9E+08	6.55E+08	6.63E+08	6.97E+08	2.27E+08
4.22E+08	3.52E+08	4.75E+08	4.07E+08	4.64E+08	4.13E+08	4.25E+08	3.84E+08	2.83E+08
2.83E+08	4.27E+08	2.33E+08	4.06E+08	2.39E+08	2.59E+08	2.69E+08	4.62E+08	3.5E+08
1.21E+08		90922000		85718000	91314000		1.07E+08	1.06E+08
1.29E+09		5.94E+08	8.41E+08	9.23E+08	6.54E+08	1.23E+09	1.11E+09	1.13E+09
6.41E+08	7.47E+08	4.45E+08	3.85E+08	3.9E+08	3.64E+08	3.53E+08	3.25E+08	7.57E+08
62217000	78419000	79113000	44347000	63678000	48779000	1.48E+08	61975000	50901000
4.54E+09	4.2E+09	3.62E+09	3.46E+09	3.41E+09	3.22E+09	2.84E+09	3.17E+09	4.17E+09
6.5E+08	5.07E+08	5.49E+08	3.92E+08	4.33E+08	3.21E+08	2.75E+08	2.87E+08	6.14E+08
6.98E+08	4.48E+08	7.27E+08	7.85E+08	7.44E+08	6.26E+08	6.51E+08	5.86E+08	5.93E+08
96623000	80140000	75505000		60145000	50843000		58952000	
2.18E+08	3.14E+08	55881000	82745000	59548000	96645000	1.29E+08	1.79E+08	2.07E+08
91602000	1.08E+08	1.31E+08	1.23E+08	1.06E+08	1.11E+08	1.21E+08	1.5E+08	97552000
1.4E+08	1.62E+08	3.15E+08	2.22E+08	2.45E+08	4.11E+08	3.53E+08	3.61E+08	1.71E+08
31944000								
3.39E+08	4.45E+08	4.4E+08	4.75E+08	3.83E+08	3.64E+08	3.17E+08	4.31E+08	4.65E+08
2.16E+08	1.65E+08	1.69E+08	1.42E+08	1.79E+08	2.05E+08	2.84E+08	1.97E+08	2.09E+08
3.32E+09	4E+09	2.04E+09	1.94E+09	2.64E+09	2.88E+09	3.61E+09	3.37E+09	2.95E+09
3.89E+08	5.39E+08	3.66E+08	3.55E+08	3.87E+08	3.55E+08	3.31E+08	4.7E+08	5.44E+08
73470000		60793000				38837000		
3.41E+08	2.86E+08	3.57E+08	3.5E+08	3.2E+08	6.78E+08	5.9E+08	5.92E+08	3.87E+08
	24592000			20040000				28038000
1.47E+09	1.17E+09	9E+08	9.8E+08	1.07E+09	1.24E+09	1.03E+09	1E+09	1.51E+09
69216000	84593000	85413000	1.23E+08	88663000	57510000			90714000
			45539000	44139000				84325000
	68031000	62399000	51078000	46657000	64434000	82926000	71958000	54737000
1.79E+08	1.29E+08	1.24E+08	99765000	1.39E+08	83145000		1.09E+08	1.44E+08
1.58E+09	1.6E+09	1.63E+09	2.03E+09	2.61E+09	3.19E+09	3.56E+09	3.04E+09	1.51E+09
				26231000			22451000	
1.77E+08	1.46E+08	1.7E+08	2.27E+08	1.84E+08	1.79E+08	1.34E+08	1.63E+08	1.41E+08
2.54E+09	2.48E+09	2.05E+09	1.88E+09	1.79E+09	2.23E+09	2.05E+09	1.88E+09	2.54E+09
		70767000	95087000	70777000	74340000		92985000	67354000
69657000	1.14E+08	1E+08	75524000	64767000	1.04E+08	1.11E+08	96167000	73404000
			93068000	1.04E+08			1.1E+08	
				8163100				18945000
84275000	1.42E+08	1.21E+08	1.43E+08	1.02E+08	1.28E+08	99109000		2.16E+08

1.37E+08	1.57E+08	2E+08	1.73E+08	1.76E+08		88161000	75810000	1.8E+08
2.22E+09	3.22E+09	2.04E+09	2.16E+09	1.86E+09	1.51E+09	1.43E+09	1.7E+09	2.79E+09
6.66E+08	7.21E+08	6.52E+08	4.85E+08	4.87E+08	7.18E+08	7.42E+08	6.66E+08	6.61E+08
72856000	71111000	57066000	71572000	61349000	45712000	50998000	67807000	72569000
		1.32E+08	1.15E+08				1.12E+08	
94923000	70968000						45896000	65497000
1.09E+08			91754000	79051000	1.12E+08	1.04E+08		1.12E+08
39563000	53875000		22785000	42446000	67158000	55442000	46095000	32708000
	93065000	78440000	58435000	50932000		70879000	79576000	70383000
1.07E+09	1.23E+09	1.18E+09	9.15E+08	7.28E+08	1.18E+09	1.24E+09	1.16E+09	1.08E+09
	33070000		26318000	34941000	28589000			
1.02E+08	1.2E+08	73589000	1.17E+08	1.9E+08	1.27E+08	78158000	1.04E+08	87503000
2.44E+08	77622000	78050000	1.44E+08	1.54E+08	1.48E+08	1.5E+08	89238000	1.27E+08
67085000	76632000	87262000	1.06E+08	87257000	77249000	78487000	88489000	80653000
69862000	69637000	88875000	63798000	80014000	73368000	80630000	74551000	67608000
				42867000				
					1.15E+08	1.14E+08	1.17E+08	
	20186000		20384000				18796000	30719000
4.24E+08	3.5E+08	3.58E+08	3.63E+08	3.68E+08	5.17E+08	4.67E+08	4.6E+08	3.04E+08
2.23E+08	1.97E+08	3.93E+08	5.6E+08	4.65E+08	2.79E+08	2.57E+08	3.16E+08	2.62E+08
26779000			29750000					
1.64E+08	1.39E+08		78676000	74951000	2.5E+08			3.05E+08
2.89E+08	3E+08	1.15E+08	1.36E+08		94861000	75794000		2.97E+08
1.15E+09	2.48E+09	5.91E+08	6.42E+08	6.85E+08	5.86E+08	5E+08	4.81E+08	2.49E+09
2.87E+08	3.81E+08	2.49E+08	1.92E+08	1.97E+08	1.58E+08	1.79E+08	1.88E+08	6.19E+08
3E+08	2.95E+08	2.59E+08	2.16E+08	2.42E+08	1.62E+08	1.22E+08	2E+08	5.21E+08
1.46E+09	1.18E+09	9.38E+08	8.14E+08	7.71E+08	5.76E+08	3.99E+08	4.96E+08	1.4E+09
		30323000	14705000	18641000		34651000	26264000	27949000
2.01E+10	2.27E+10	1.31E+10	1.09E+10	1.26E+10	1.04E+10	1.38E+10	1.14E+10	1.93E+10
64944000		93799000	1.33E+08			2.66E+08	2.66E+08	54903000
6.47E+08	7.35E+08	6.34E+08	5.68E+08	5.94E+08	5.77E+08	5.11E+08	5.1E+08	7.45E+08
		45976000	41689000	45879000				
1.91E+09	1.43E+09	1.66E+09	1.88E+09	2E+09	1.86E+09	1.29E+09	1.31E+09	1.7E+09
1.78E+09	2.06E+09	1.64E+09	1.32E+09	1.35E+09	1.59E+09	1.18E+09	1.18E+09	1.97E+09
57345000	34848000	40845000	49698000	55992000	58497000	42104000	38414000	32863000
2.77E+09	2.63E+09	2.16E+09	2.47E+09	2.73E+09	2.87E+09	2.7E+09	2.48E+09	2.98E+09
37213000		32722000	28054000	38840000	38827000	41307000		44758000
66739000	64329000							55239000
3.46E+08	3.14E+08	4.45E+08	3.82E+08	3.93E+08	4.6E+08	5.64E+08	5.52E+08	3.86E+08
			40838000	70353000				
76036000	90866000	1.18E+08	91239000	77649000	90746000	1.08E+08	1.17E+08	64220000

1.64E+08		1.95E+08	2.05E+08	2.33E+08	2.88E+08	2.16E+08	2.55E+08	1.52E+08
7332600		12959000		4296200	10450000	17490000	7956700	6142500
2.28E+08	2.25E+08	1.6E+08	4.12E+08	4.53E+08	1.18E+08	92427000	1.46E+08	2.43E+08
45466000		42399000	41233000		29598000	32056000	35483000	31750000
1.17E+08	93951000	1.39E+08	1.01E+08	1.23E+08	1.17E+08	99858000	1.38E+08	1.56E+08
1.21E+08	1.67E+08	1.52E+08	1.21E+08	1.13E+08	1.6E+08	1.62E+08	1.73E+08	1.65E+08
87920000	1.21E+08	1.15E+08	1.03E+08	85183000	98409000	1.1E+08	1.23E+08	67779000
1.49E+08	2.03E+08	88105000					1.52E+08	2.27E+08
88935000								93227000
34950000	44075000	51506000	38144000	42362000	36566000	61570000	37704000	43090000
24553000		73353000	55500000	20307000	3.94E+08	4.13E+08	4.45E+08	26462000
					1.31E+08	1.26E+08	1.21E+08	
1.89E+08	2.28E+08	1.52E+08	1.31E+08	1.4E+08	1.54E+08	1.19E+08	1.14E+08	1.65E+08
6.52E+08	7.49E+08	4.63E+08	6.56E+08	5.12E+08	4.14E+08		3.76E+08	8.36E+08
4.41E+08	4.4E+08	2.69E+08	4.01E+08	4.19E+08	2.89E+08	3.06E+08	4.13E+08	4.98E+08
3.14E+08	3.79E+08	3.07E+08	2.08E+08	1.67E+08	1.94E+08	1.71E+08	1.84E+08	2.67E+08
1E+08	1.15E+08	1.01E+08	75489000	76371000	30537000		46138000	1.57E+08
4.68E+09	4.25E+09	3.42E+09	3.88E+09	3.77E+09	4.16E+09	4.13E+09	4.19E+09	4.03E+09
					1.2E+08	98184000		
		73544000			54842000			65134000
4.81E+08	4.63E+08	4.94E+08	3.9E+08	3.99E+08	6.29E+08	4.83E+08	5.2E+08	5.26E+08
2.59E+08	3.06E+08	2.07E+08		2.05E+08		2.5E+08		2.86E+08
9E+08	1.25E+09	6.16E+08	6.48E+08	7.05E+08	6.61E+08	8.42E+08	5.67E+08	9.75E+08
91406000	56997000	65889000	87559000	85810000	79152000	51204000	75248000	71743000
1.15E+09	1.39E+09	1.21E+09	1.21E+09	1.1E+09	1.17E+09	1.15E+09	1.17E+09	1.26E+09
2.71E+08	2.4E+08	2.04E+08	2.3E+08	2.06E+08	2.65E+08	1.59E+08	2.09E+08	3.07E+08
3.88E+08	3.07E+08	5.72E+08	5.19E+08	5.14E+08	6.06E+08	6.03E+08	5.51E+08	2.71E+08
2.17E+08	3.41E+08	3.38E+08	2.25E+08				1.9E+08	4.6E+08
1.47E+08	1.39E+08	1.39E+08	1.44E+08	1.35E+08	1.26E+08	81063000	1.45E+08	89844000
6.66E+08	6.19E+08	5.89E+08	5.64E+08	5.01E+08	4.48E+08	4.4E+08	4.24E+08	5.42E+08
2.04E+08	2.49E+08	1.79E+08	1.66E+08	1.6E+08	2.03E+08	2.14E+08	1.57E+08	
2.3E+08	3.25E+08	2.57E+08	2.09E+08	1.92E+08	2.37E+08	1.53E+08	2.35E+08	2.97E+08
1.11E+09	1.27E+09	9.17E+08	8.98E+08	8.58E+08	8.22E+08	9.18E+08	1.01E+09	1.22E+09
	52897000		44103000	37331000	54419000		44344000	44638000
1.46E+09	1.38E+09	1.47E+09	1.52E+09	1.62E+09	1.68E+09	1.31E+09	1.42E+09	1.57E+09
2.61E+09	3.21E+09	2.67E+09	3.6E+09	3.04E+09	1.63E+09	1.36E+09	1.78E+09	5.5E+09
78871000					59649000	65500000	67583000	61578000

1.59E+08	1.31E+08	2.64E+08	2.85E+08	2.72E+08	2.14E+08		1.8E+08	1.73E+08
1.01E+08	1.06E+08	1.23E+08	1.18E+08	1.39E+08	1.81E+08	2.09E+08	1.87E+08	1.19E+08
1.62E+09	9.23E+08	5.96E+08	8.43E+08	9.35E+08	6.23E+08	4.86E+08	5.28E+08	1.84E+09
3.74E+08	2.65E+08	3.8E+08	4.09E+08	3.23E+08	3.26E+08	3.65E+08	5.03E+08	2.82E+08
3.88E+08		2.9E+08	3.24E+08	2.97E+08	2.94E+08	3.39E+08	3.37E+08	4.2E+08
	1.19E+08	1.2E+08	1.16E+08	96087000	1.5E+08	1.58E+08	1.44E+08	92363000
2.68E+08	3.59E+08	2.36E+08	2.05E+08	1.94E+08	1.49E+08	1.68E+08	1.74E+08	3.05E+08
48749000					88821000			
92725000	53994000	62656000	59835000	63485000	91075000	76010000	1.05E+08	68025000
1.01E+09	1.18E+09	6.02E+08	5.56E+08	5.21E+08	5.23E+08	5.7E+08	4.9E+08	1.04E+09
1.15E+09	1.4E+09	1.28E+09	1.36E+09	1.48E+09	9.69E+08	1.17E+09	1.32E+09	1.38E+09
					24673000	34102000		
			17646000					15264000
1.58E+10	1.43E+10	1.43E+10	1.27E+10	1.55E+10	8.97E+09	1.06E+10	1.12E+10	1.6E+10
5.37E+08	4.79E+08	4.93E+08	5.78E+08	4.62E+08	5.14E+08	5.88E+08	5.58E+08	8.66E+08
						52138000	45851000	
95080000	91298000	89923000	85884000	76122000	98669000	98141000	1.09E+08	
55896000	81657000	65104000	48966000	45041000	59376000	55881000	50226000	53248000
1.59E+09	2.9E+09	1.84E+09	1.51E+09	1.27E+09	1.32E+09	1.13E+09	1.39E+09	1.48E+09
4.07E+08	3.74E+08	6.08E+08	4.17E+08	3.51E+08	3.93E+08	4.07E+08	4.13E+08	4.32E+08
1.08E+08	1.13E+08	94631000	1.14E+08	97871000	1.1E+08	1.05E+08		
4.95E+08	6.59E+08	6.95E+08	4.96E+08	5.92E+08	6.38E+08	6.62E+08	4.94E+08	6.93E+08
1.05E+08	1.28E+08	84589000	1.04E+08	1.27E+08	1.2E+08	84012000	97547000	81131000
	51269000							40313000
					44054000	46822000		
1.22E+09	1.12E+09	9.6E+08	1.18E+09	1.22E+09	1.23E+09	1.22E+09	1.32E+09	1.15E+09
		58638000	81112000					
5.77E+09	6.52E+09	4.84E+09	3.43E+09	3.32E+09	2.33E+09	2.6E+09	2.75E+09	6.1E+09
98638000	1.13E+08	1.36E+08	1.33E+08	1.37E+08	1.39E+08	1.11E+08	1.45E+08	89279000
		1.11E+08	1.44E+08	1.05E+08	99795000	1.3E+08	94557000	1.94E+08
2.94E+08	3.54E+08	1.75E+08	1.02E+08	1.09E+08	62033000		64258000	3.57E+08
2.34E+08	2.82E+08	3.14E+08	3.98E+08	4.28E+08	3.14E+08	3.32E+08	3.62E+08	3.03E+08
33724000	36181000	61282000	51720000	59855000	71941000	1.01E+08	99377000	40341000
8.49E+08	7.76E+08	9.21E+08	9.12E+08	8.07E+08	5.71E+08	5.86E+08	5.25E+08	7.22E+08
7.15E+08	5.59E+08	4.85E+08	1.07E+09	8.47E+08	5.16E+08	4.38E+08	6.42E+08	5.46E+08
1.63E+08	1.37E+08	1.55E+08	1.83E+08	1.8E+08	1.67E+08	1.72E+08	1.71E+08	1.21E+08
58580000		1.01E+08	65444000	62835000	72709000	87761000	72328000	38138000
1.44E+08	1.11E+08	94557000	1.23E+08	1.34E+08	1.61E+08	1.7E+08	1.3E+08	1.23E+08

4.1E+08	3.92E+08	7.64E+08	7.35E+08	6.85E+08	6.33E+08	4.31E+08	5.58E+08	5.32E+08
44197000				38198000	44335000			
1.14E+08	92375000	52368000	68553000	1.01E+08	77604000	70145000	58462000	1.02E+08
56923000	57803000	42980000	56327000	37112000			55388000	62727000
46827000		52407000		41209000		67715000		
4.77E+08	5.3E+08	2.3E+08	2.51E+08	1.71E+08	1.44E+08	1.09E+08	1.82E+08	1E+09
3.53E+08	5.1E+08	1.76E+08	1.99E+08	1.97E+08	2.91E+08	2.5E+08	2.41E+08	4.1E+08
1.23E+08	1.16E+08	1.71E+08	4.5E+08	2.94E+08			77856000	2.39E+08
				1.02E+08			66228000	80900000
1.41E+09	2.43E+09	1.5E+09	1.06E+09	9.49E+08	1.2E+09	1.13E+09	1.08E+09	1.13E+09
				89866000				
4.3E+08		3.79E+08	4E+08	4.92E+08	2.35E+08	2.75E+08	1.08E+08	72929000
92615000	89187000	71986000	93940000		60421000	39273000		64479000
4.6E+08	6.56E+08	5.76E+08	7.21E+08	4.68E+08	5.82E+08	5.55E+08	5.52E+08	7.48E+08
2.63E+08	3.11E+08	2.69E+08	1.92E+08		3.08E+08		3.04E+08	
4.13E+08	4.91E+08	2.14E+08	2.46E+08	2.18E+08	3.07E+08	2.24E+08	2.09E+08	4.76E+08
5.14E+08	5.33E+08	5.97E+08	6.17E+08	6.45E+08	3.69E+08	6.62E+08	5.14E+08	4.81E+08
3.53E+08	3.63E+08	6.9E+08	7.26E+08	6.32E+08	5.22E+08	7.04E+08	7.2E+08	2.63E+08
9.29E+08	8.02E+08	1.17E+09	1.16E+09	1.19E+09	9.32E+08	1.48E+09	1.3E+09	7.8E+08
4.24E+08	7.29E+08	8.96E+08	9.53E+08	7.37E+08	8.95E+08	8.97E+08	9.89E+08	8.38E+08
57720000		40107000	59243000	94835000	62436000	58630000	45556000	65927000
2.49E+08	2.68E+08		1.58E+08	1.8E+08	2.56E+08	2.67E+08	2.89E+08	2.66E+08
	1.51E+08	1.02E+08	62916000	64666000				76779000
6.04E+08	6.75E+08	6.92E+08	5.61E+08	5.46E+08	7.3E+08	7.11E+08	7.7E+08	6.11E+08
1.82E+08	1.89E+08	1.72E+08	1.38E+08	1.64E+08	1.58E+08	1.52E+08	1.42E+08	2.1E+08
74095000	93699000	90673000	81522000	70346000	60137000	68180000	66048000	1.02E+08
2.7E+08			3E+08	2.04E+08	2.61E+08		2.52E+08	2.02E+08
1.79E+08	2.02E+08	2.13E+08	1.79E+08	1.97E+08	2.48E+08	2.6E+08	2.5E+08	2.2E+08
1.73E+08	2.25E+08	1.58E+08	1.08E+08	1.39E+08	1.8E+08	1.71E+08	1.59E+08	1.79E+08
2.84E+09	3.1E+09	4.1E+09	6.69E+09	4.42E+09	2.86E+09	2.55E+09	2.65E+09	4.9E+09
4.66E+08		3.66E+08	3.66E+08	3.59E+08		1.65E+09		
	61944000	42658000	29926000	29330000				63779000
4.43E+08	7.16E+08	4.89E+08	4.59E+08	4.21E+08	4.52E+08	4.6E+08	4.29E+08	5.88E+08
				26335000			27036000	
3.24E+08		2.65E+08	2.53E+08	2.86E+08	3.67E+08	3.78E+08	4.17E+08	2.64E+08
			18741000	24536000			18938000	
2.04E+08	2.19E+08	2.57E+08	1.84E+08	1.78E+08	3.18E+08	3.12E+08	3.09E+08	1.71E+08
5.02E+08	5.13E+08	3.74E+08	3.28E+08	3.4E+08	3.06E+08	3.2E+08	2.98E+08	5.7E+08
3.78E+08	3.35E+08	2.7E+08	3.75E+08	3.33E+08	2.62E+08	1.95E+08	2.26E+08	4.42E+08
9.74E+08	9.96E+08	7.97E+08	7.73E+08	9.9E+08	9.75E+08	9.26E+08	9.4E+08	1.23E+09

2.74E+08	3.79E+08	1.35E+08	2.04E+08	1.74E+08	1.76E+08	2.88E+08	2.59E+08	3.32E+08
1.14E+09	1.16E+09	1.19E+09	9.3E+08	9.69E+08	7.45E+08	8.07E+08	7.28E+08	1.33E+09
1.34E+08	1.63E+08	83007000	81490000	1.05E+08	2.27E+08	1.86E+08	1.71E+08	1.29E+08
4.69E+08	4.87E+08	5.82E+08	4.65E+08	3.9E+08	6.31E+08	7.8E+08	6.51E+08	5.06E+08
95407000	1.05E+08	85873000	83406000	93560000	87725000	84353000	80521000	1.03E+08
2.87E+09	3.66E+09	3.39E+09	2.91E+09	2.89E+09	3.15E+09	3.12E+09	2.89E+09	2.88E+09
2.18E+08	2.19E+08	67559000	62139000	1.29E+08	29477000	41850000	42025000	3.47E+08
2.63E+08	3.1E+08	1.87E+08	1.95E+08	1.45E+08	1.96E+08	1.96E+08	1.81E+08	2.46E+08
1.45E+08	1.86E+08	1.09E+08	99569000	82855000	1.18E+08	83154000	1.18E+08	1.48E+08
1.45E+08	2.12E+08	1.38E+08	1.23E+08	1.18E+08	1.33E+08	1.27E+08	1.3E+08	1.56E+08
75721000	89922000	74726000	96584000	93106000	82021000		57465000	59213000
		1.14E+08	1.84E+08	1.57E+08	1.43E+08	1.35E+08	2.62E+08	2.33E+08
8.26E+08	6.42E+08	7.96E+08	1.06E+09	1.04E+09	7.87E+08	6.92E+08	8.19E+08	9.22E+08
	75853000			39190000	64532000			
78457000	95585000	93139000	89470000	1.31E+08	2.02E+08	1.34E+08	1.08E+08	86777000
90460000	75266000	52621000	58114000	65895000	73802000	71619000	70122000	82278000
1.09E+08	1.18E+08	1.16E+08	96733000	1.01E+08	91266000	1.11E+08	1.11E+08	1.21E+08
5.04E+08	5.65E+08	4.38E+08	4.28E+08	3.88E+08	4.84E+08	4E+08	5.59E+08	4.26E+08
3.31E+09	2.63E+09	6.95E+09	6.62E+09	6.64E+09	7.73E+09	5.93E+09	7.24E+09	2.77E+09
6.03E+08	5.52E+08	5.75E+08	6.14E+08	6.19E+08	6.98E+08	6.88E+08	6.77E+08	6.95E+08
1.93E+08	2.83E+08	1.53E+08	1.62E+08	1.74E+08	1.81E+08	1.44E+08	1.84E+08	2.26E+08
1.19E+08	2.01E+08	99227000	1.17E+08	90138000	1.19E+08	1.55E+08	1.19E+08	1.54E+08
95280000	1.03E+08	1.39E+08	1E+08	97016000	1.4E+08	71520000	1.12E+08	89878000
3.17E+08	3.28E+08	2.91E+08	3.14E+08	2.54E+08	3.31E+08	4.26E+08	3.78E+08	2.73E+08
1.25E+09	1.12E+09	1.72E+09	1.33E+09	1.64E+09	2.28E+09	2.66E+09	1.92E+09	1.24E+09
93927000	1.36E+08	2.46E+08	1.94E+08	2.07E+08	3.09E+08	3.04E+08	2.34E+08	
1.93E+08	1.14E+08	2.02E+08	2.53E+08	2.19E+08	2.46E+08	2.85E+08	2.09E+08	1.99E+08
1.36E+08	1.66E+08	1.62E+08	1.46E+08	1.24E+08	1.06E+08	1.59E+08	1.37E+08	1.68E+08
68386000	94868000	1.02E+08	95427000	86452000	87235000	96524000	1E+08	79186000
			29483000		31134000			
1.83E+09	1.65E+09	1.54E+09	1.88E+09	2.01E+09	2.46E+09	2.06E+09	2.26E+09	1.72E+09
1.09E+08		66155000	95372000	82998000	76109000			75417000
3.67E+08	5.38E+08	5.21E+08	9.32E+08	7.18E+08	3.04E+08	1.39E+08	3.91E+08	6.04E+08
		43386000		43898000				
1.64E+09	1.57E+09	1.88E+09	1.85E+09	1.97E+09	2.03E+09	2.78E+09	2.12E+09	1.32E+09
4.07E+08	3.68E+08	4.77E+08	4.52E+08	4.53E+08	5.58E+08	5.28E+08	5.51E+08	4.33E+08
8.3E+08	9.01E+08	5.41E+08	7.23E+08	6.65E+08	7.8E+08	7.28E+08	5.43E+08	6.06E+08
2.34E+08	1.65E+08	1.52E+08	97024000		80261000		90450000	4.06E+08
2.36E+08	5.06E+08	2.11E+08		2.1E+08	3E+08	2.65E+08	1.8E+08	4.07E+08
51594000	93474000	54552000	71302000	48282000	53205000		46604000	67276000
40290000	36952000	45862000	43881000	46229000	56687000	50616000	53361000	36076000
1.28E+08		1.81E+08	1.75E+08	2.16E+08	1.57E+08	1.12E+08	1.23E+08	

		3.66E+08	2.12E+08	2.16E+08	2.43E+08	1.53E+08	1.62E+08	
			77174000	70168000	1.04E+08			
3.68E+08	2.84E+08	1.96E+08	3.1E+08	2.74E+08	1.98E+08	1.27E+08	2.18E+08	3.69E+08
81277000	62904000	93355000	1.31E+08	85903000	61621000	1.69E+08	1.48E+08	90556000
		37589000			32232000			
					78807000			
44837000	55815000	68603000	61898000	45691000	69062000	68704000	74171000	
4.64E+08	4.32E+08	7.42E+08	5.72E+08	6.36E+08	6.89E+08	6.53E+08	6.93E+08	4.45E+08
1.41E+08		3.26E+08	5.13E+08	4.19E+08	2.15E+08			1.45E+08
	28940000	18027000	18688000	20517000	15824000		18606000	28848000
8.75E+08	1E+09	6.19E+08	6.24E+08	6.75E+08	6.13E+08	8.11E+08	1.08E+09	1.33E+09
5.71E+08	5.91E+08	7.19E+08	6.67E+08	6.44E+08	5.99E+08	5.92E+08	7.25E+08	7.6E+08
95523000	90204000	61650000	69520000	89896000				1.69E+08
1.88E+08	3.13E+08	1.85E+08	2.22E+08	1.62E+08	1.23E+08	1.87E+08	1.5E+08	2.29E+08
4.83E+08	5.26E+08	4.31E+08	5.81E+08	4.85E+08	2.97E+08	4.07E+08	5.5E+08	5.84E+08
		24687000	23357000					38558000
	87863000							61500000
63515000	90559000					75878000		79421000
57531000	70658000	57396000	80675000	59738000	47572000	66432000	74551000	84770000
1.22E+09	1.44E+09	1.35E+09	1.2E+09	1.26E+09	1.54E+09	1.44E+09	1.38E+09	1.46E+09
26990000			18581000	23466000		36578000	23317000	18682000
58018000	47286000	67252000	61579000	61560000	60943000	98065000	65241000	63285000
97564000	1.16E+08	78821000	75447000	74661000	62566000	89545000	85797000	98976000
47953000	50593000	35181000	30707000	33857000	27435000	30619000	47740000	48987000
1.75E+08	2.33E+08	2.23E+08	1.98E+08	2.18E+08	2.92E+08	2.9E+08	2.32E+08	2.2E+08
				75382000	1.02E+08	84948000	72727000	71499000
	62861000	74955000	54777000	37392000	92985000	89305000	80932000	41528000
3.17E+08	2.78E+08	2.39E+08	2.32E+08	2.55E+08	1.57E+08	3.62E+08	1.72E+08	2.71E+08
6.77E+08	8.71E+08	8.73E+08	9.59E+08	7.97E+08	8.9E+08	8.27E+08	9.13E+08	7.75E+08
		3.56E+09		2.7E+09			4.15E+09	
				64087000	43759000			
69795000	65608000				44827000			1.38E+08
1.54E+09	1.9E+09	2.18E+09	1.83E+09	1.46E+09	1.08E+09	1.49E+09	1.93E+09	1.57E+09
2.14E+08	2.18E+08	2.77E+08	2.88E+08	3.23E+08	3.66E+08	3.82E+08	3.74E+08	2.44E+08
6.07E+08	7.15E+08	7.15E+08	6.23E+08	7.13E+08	6.53E+08	6.48E+08	6.75E+08	7.96E+08
1.09E+08	55721000				1.19E+08			51284000
1.68E+08	1.87E+08	1.94E+08	1.65E+08	2.58E+08	2.38E+08	2.84E+08	2.33E+08	1.2E+08
						82001000	91269000	
3.59E+08		4.17E+08	2.79E+08	2.42E+08	2.63E+08	2.59E+08	1.8E+08	3.93E+08
1.09E+09	1.08E+09	1.31E+09	1.22E+09	1.23E+09	1.53E+09	1.38E+09	1.46E+09	1E+09

92948000	90358000							2.35E+08
3.44E+08	2.89E+08	95101000	1.11E+08	1.08E+08	52823000	60701000	64199000	4.44E+08
58590000	59129000	94925000	71723000	62286000	62666000	99216000	91180000	48017000
45050000	56863000							
2.35E+08		1.14E+08	1.65E+08	1.1E+08	1.07E+08	82480000	92314000	96848000
6.23E+08	6.05E+08	3.3E+08	5.98E+08	4.78E+08	3.92E+08	5.28E+08	2.15E+08	5.71E+08
1.35E+09	1.4E+09	3.35E+09	3.04E+09	3.46E+09	3.35E+09	4.08E+09	3.47E+09	1.02E+09
48610000		41135000				42818000	45973000	36611000
		7.57E+08					8.2E+08	
1.9E+08	2.1E+08	1.87E+08	1.92E+08	1.74E+08	2.03E+08	2.09E+08	2.03E+08	1.97E+08
1.03E+09	9.75E+08	7.35E+08	7.72E+08	7.96E+08	6.82E+08	5.91E+08	8.2E+08	1.48E+09
2.83E+08	2.52E+08	2.17E+08	2.14E+08	2.26E+08	3.19E+08	3.53E+08	2.87E+08	2.54E+08
4.67E+08	5.12E+08	4.93E+08	4.64E+08	3.61E+08	5.45E+08	4.14E+08		
1.93E+08	2.64E+08	2.14E+08	2.05E+08	1.51E+08	2.12E+08	2.46E+08	2.73E+08	1.43E+08
2.13E+08	2.51E+08	2.73E+08	2.44E+08	2.19E+08	2.62E+08	2.93E+08	3.02E+08	2.64E+08
1.96E+09	2.28E+09	1.37E+09	1.51E+09	1.34E+09	7.26E+08	7.84E+08	9.87E+08	1.88E+09
			40205000	46770000	59443000	79029000	56701000	
1.19E+08	1.42E+08	78558000	1.1E+08	87439000		1.04E+08		1.62E+08
1.62E+09	1.26E+09	1.31E+09	9.58E+08	1.13E+09	1.07E+09	8.54E+08	8.55E+08	1.02E+09
					9629500	21423000		
							32428000	
			14241000	14047000				
1.21E+08	1.26E+08		87323000	1.07E+08	1.33E+08	1.13E+08	95946000	1.27E+08
2.74E+08	4.02E+08	2.45E+08	2.83E+08	3.23E+08	3.06E+08	3.27E+08	3.93E+08	3.75E+08
2.06E+08	2.41E+08	2.75E+08	2.76E+08	1.93E+08	2.97E+08	3.43E+08	3.18E+08	1.83E+08
2.82E+08	3.1E+08	3.74E+08	4.07E+08	3.49E+08	3.03E+08	2.57E+08	3.62E+08	4.33E+08
98163000	1.1E+08	1.2E+08	1.05E+08	98493000	54787000	59103000	79435000	96653000
2.4E+08	1.79E+08	1.42E+08	1.36E+08	1.49E+08			1.03E+08	2.49E+08
1.22E+08	1.98E+08							1.73E+08
2.64E+09	2.44E+09	2.63E+09	2.57E+09	2.79E+09	2.84E+09	4.49E+09	4.31E+09	2.4E+09
2.61E+08	3.34E+08	2.51E+08	1.94E+08	1.7E+08	1.98E+08	2.36E+08	2.39E+08	3.29E+08
2.11E+08	2.56E+08	2.79E+08	2.18E+08	2.08E+08	2.4E+08	2.38E+08	2.51E+08	2.53E+08
							33911000	
1.79E+08	2.22E+08	1.67E+08	1.84E+08	1.77E+08			1.05E+08	6.6E+08
			8028400	6892900				
		84939000	1.04E+08	63586000			58922000	76202000
2.26E+09	2.24E+09	2.12E+09	2.1E+09	1.69E+09	1.61E+09	9.15E+08	1.5E+09	1.52E+09
7.57E+08	8.16E+08	7.12E+08	5.64E+08	5.09E+08	5.34E+08	5.45E+08	6.47E+08	7.38E+08

	82712000	94391000	69436000	90161000	80449000	86683000	74272000	39981000
1.64E+08	1.23E+08	1.06E+08	2.18E+08	1.11E+08	1.07E+08	1.41E+08	1.27E+08	1.49E+08
1.05E+08	1.37E+08	1.05E+08	1.21E+08	91291000	96143000	87591000	77514000	1.5E+08
1.1E+09	1.37E+09	8.99E+08	1.14E+09	8.76E+08	9.87E+08	1.3E+09	1.11E+09	1.22E+09
1.1E+08	1.53E+08	1.22E+08	85554000	83207000		1.17E+08	1.24E+08	
				47174000			45403000	51261000
4.42E+08	4.04E+08	3.25E+08	3.39E+08	3.18E+08	3.72E+08	4.45E+08	4.52E+08	4.17E+08
8.05E+08	7.79E+08	7.95E+08	9.36E+08	8.12E+08	1.21E+09	1.04E+09	1.22E+09	7.6E+08
		43422000	50803000	48213000	32389000	38529000	35853000	41452000
2.13E+08	2.96E+08	1.87E+08	1.9E+08	1.2E+08	2.34E+08	2.32E+08	2.84E+08	
9.93E+08	9.62E+08	1.02E+09	1.21E+09	9.92E+08	1.08E+09	9.79E+08	9.68E+08	8.65E+08
8.18E+08	8.66E+08			5.79E+08		88082000		1E+09
	32938000			42678000		59559000	65945000	
3.79E+08	3.71E+08	1.31E+08	5.02E+08	1.42E+08	4.42E+08	3.82E+08	5.5E+08	3.71E+08
		30721000		55126000	27113000			
51960000		1.43E+08	1.45E+08	1.53E+08	1.36E+08	73999000	74662000	
6.51E+08	6.68E+08	7.9E+08	7.26E+08	7.82E+08	8.68E+08	1.19E+09	1.09E+09	8.21E+08
1.94E+08	1.17E+08	2.56E+08	4.08E+08	3.98E+08	4.51E+08	3.75E+08	4.15E+08	1.42E+08
1.77E+08	2.2E+09	1.4E+09	2.69E+09	9.46E+08	8.62E+08	7.49E+08	7.24E+08	1.62E+09
4.84E+08	6.38E+08	6.86E+08	5.28E+08	5.02E+08	3.76E+08	1.93E+08	3.75E+08	4.53E+08
6.46E+08	7.4E+08	6.44E+08	6.38E+08	5.36E+08	5.69E+08	5.71E+08	6.3E+08	6.22E+08
2.24E+08	1.21E+08	1.03E+08	79697000	74788000	75946000	66544000	81683000	1.3E+08
					87252000			
1.53E+08	2.23E+08	1.8E+08	1.82E+08	2.31E+08	1.94E+08	1.99E+08	1.84E+08	1.78E+08
		1.44E+08				1.39E+08	1.51E+08	1.37E+08
1.39E+08	1.76E+08	1.69E+08	1.42E+08	1.49E+08	1.38E+08	1.37E+08	2.1E+08	1.46E+08
2.82E+08	3.41E+08	3.83E+08	4.63E+08	3.44E+08	1.96E+08	1.83E+08	2.49E+08	4.82E+08
1.92E+08	1.6E+08	1.84E+08	2.23E+08	2.33E+08	2.07E+08	1.58E+08	2.19E+08	1.73E+08
1.23E+09	1.3E+09	1.11E+09	1.1E+09	1.15E+09	9.13E+08	9.83E+08	1.12E+09	1.59E+09
77032000	46895000	82243000	83749000	78278000	90189000	77550000	84494000	61436000
1.97E+09	1.8E+09	1.06E+09	1.03E+09	9.17E+08	7.78E+08	8.85E+08	7.9E+08	2.26E+09
54182000						52456000		
9.15E+08	1.18E+09	6.89E+08	6.41E+08	7.28E+08	5.14E+08	6.14E+08	6.5E+08	1.29E+09
5.37E+08	6.07E+08	4.57E+08	5.03E+08	4.52E+08	5.45E+08	4.48E+08	3.86E+08	4.7E+08
6.87E+08	3.74E+08	7.47E+08	6.04E+08	5.27E+08	8.23E+08	6.08E+08	7.69E+08	5.47E+08
22721000	23623000	16815000	16772000	22697000	18295000	27538000	26996000	31082000
1.19E+09	1.22E+09	9.06E+08	7.07E+08	9.39E+08	9.58E+08	1.75E+09	8.4E+08	1.1E+09
		88126000	1.07E+08	94945000				
7.72E+08	8.71E+08	8.55E+08	7.7E+08	7.24E+08	8.21E+08	9.76E+08	7.89E+08	9E+08
1.12E+08	1.5E+08	1.28E+08	1.09E+08	94034000	1.18E+08		1.15E+08	1.08E+08
	1.63E+08	1.92E+08	2.05E+08	1.91E+08	1.78E+08	1.77E+08	1.99E+08	1.48E+08
					1.05E+08		64127000	

3.23E+08	2.97E+08	4.21E+08	2.37E+08	2.42E+08	1.86E+08	1.8E+08	2.11E+08	3.57E+08
4.38E+08	5.05E+08	3.03E+08	2.68E+08	3.13E+08	2.55E+08	2.52E+08	2.61E+08	4.82E+08
7E+08	6.93E+08	6.45E+08	6.73E+08	6.15E+08	5.49E+08	4.69E+08	5.5E+08	8.2E+08
3.68E+08	3.66E+08	3.23E+08	3.36E+08	2.58E+08	2.35E+08	3.41E+08	2.65E+08	3.68E+08
5.46E+08	4.81E+08	5.14E+08	5.58E+08	5.32E+08	4.76E+08	4.44E+08	4.88E+08	6.9E+08
1.04E+09	9.02E+08	1.04E+09	8.52E+08	9.31E+08	9.23E+08	1.08E+09	1.04E+09	1.18E+09
4.18E+09	3.61E+09	3.35E+09	4.3E+09	4.09E+09	3.33E+09	3.47E+09	3.44E+09	3.92E+09
1.06E+09	1.88E+09	7.92E+08	7.32E+08	9E+08	5.55E+08	5.83E+08	6.84E+08	1.13E+09
1.81E+08	3E+08	3.64E+08	2.67E+08	2.23E+08	4.65E+08	5.39E+08	5.35E+08	2.3E+08
8.58E+08	1.02E+09	8.02E+08	7.49E+08	7.88E+08	9.43E+08	7.83E+08	9.25E+08	9.75E+08
1.41E+08	2.01E+08	2.06E+08	1.81E+08	1.68E+08	2.18E+08	1.54E+08	1.81E+08	1.76E+08
			1.64E+08	1.77E+08				1.8E+08
1.22E+08	1.07E+08	1.59E+08	1.33E+08	1.75E+08	2.94E+08	2.6E+08	2.49E+08	1.56E+08
7.2E+08	6.68E+08	6.79E+08	6.64E+08	7.03E+08	9.49E+08	8.12E+08	7.62E+08	6.33E+08
2.95E+09	3.74E+09	4E+09	3.57E+09	3.24E+09	4.94E+09	4.86E+09	4.53E+09	3.21E+09
1.8E+08	1.18E+08	1.94E+08	1.91E+08	1.71E+08	2.2E+08	1.85E+08	2.14E+08	1.33E+08
1.85E+08	2.91E+08	2.62E+08	2.71E+08	2.83E+08	3.5E+08	2.81E+08	3.35E+08	2.66E+08
2.89E+08	4.51E+08	2.75E+08	2.59E+08	1.91E+08		2.18E+08	2.59E+08	3.51E+08
					36961000	36378000		77213000
2.2E+08	2.5E+08	2.54E+08	2.37E+08	2.23E+08	3E+08	3.29E+08	2.87E+08	1.91E+08
		40479000	61310000	47820000	28849000		48038000	
6.4E+08	5.33E+08	3.19E+08	7.59E+08	6.4E+08	3E+08	2.19E+08	5.21E+08	6.13E+08
		38473000			45793000	41080000	40735000	
		43560000	35690000	34139000			29867000	
2.45E+08	2.12E+08	1.04E+08	1.35E+08	1.47E+08	2.54E+08	2.41E+08	1.97E+08	2.68E+08
9.94E+08	1.34E+09	6.97E+08	6.36E+08	6.68E+08	7.74E+08	6.73E+08	7.46E+08	1.37E+09
	58103000	45919000				51021000		
3.54E+09	2.94E+09	3.75E+09	5.25E+09	5.14E+09	4.65E+09	5.4E+09	4.88E+09	3.36E+09
1.41E+08		3.22E+08	2.28E+08	2.52E+08	1.58E+08	2.35E+08	1.57E+08	3.26E+08
4.51E+08		2.57E+08	3.76E+08	3.92E+08	4.27E+08	2.54E+08	2.14E+08	4.39E+08
6.14E+08	8.96E+08	6.06E+08	5.76E+08	5.33E+08	6.04E+08	5.66E+08	5.93E+08	7.02E+08
2.53E+08	3.06E+08	2.28E+08	2.29E+08	2.08E+08	1.69E+08	3.2E+08	2.6E+08	2.55E+08
	63528000	61088000						99284000
								1.35E+08
3.56E+08	4.27E+08	3.6E+08	3.08E+08	2.74E+08	3.58E+08	3.56E+08	3.17E+08	3.5E+08
		19033000	20684000	16401000				
71587000	83457000	45581000	49942000	43281000		40957000	41771000	69591000
		31558000	44694000	46856000				1.07E+08
1.71E+08	1.25E+08	2.17E+08	3.19E+08	2.95E+08	2.74E+08	2.17E+08	2.67E+08	1.14E+08
1.09E+08	1.18E+08	80406000		68753000	95422000	71131000	1.04E+08	1.26E+08
6.95E+08	7.1E+08	6.42E+08	5.45E+08	5.81E+08	7.1E+08	6.88E+08	7.29E+08	6.89E+08
1.26E+08	84353000	1.03E+08	1.53E+08	1.5E+08	73596000	60785000	70227000	1.17E+08
3.46E+08	4.84E+08	2.66E+08	5.07E+08	3.57E+08				6.57E+08
					2.48E+08			
1.53E+08	85420000	83894000	1.08E+08	69163000	1.02E+08	67618000	1.04E+08	1.09E+08

1.76E+08	1.93E+08	1.42E+08	1.21E+08	99345000	1.03E+08	1.01E+08	1.37E+08	2.74E+08
2.4E+08	3.84E+08	1.62E+08	1.53E+08	1.65E+08	1.81E+08	1.89E+08	2.74E+08	5.64E+08
		32081000		30306000				35601000
64941000	2.06E+08	58922000	63512000	40247000	59029000	55156000		
1.97E+08	1.39E+08	1.8E+08	2.2E+08	2.84E+08	3.2E+08	1.91E+08	1.6E+08	1.82E+08
1.27E+08	1.12E+08	1.14E+08	1.2E+08	91503000	1.04E+08	1E+08	1.16E+08	79093000
1.71E+09	1.35E+09	1.45E+09	1.37E+09	1.43E+09	1.93E+09	1.85E+09	1.83E+09	1.79E+09
80900000	70911000	67667000	75002000	76960000	1.57E+08		98393000	64839000
53282000		62096000	64891000	57762000				76647000
2.59E+09	2.01E+09	1.89E+09	1.8E+09	1.88E+09	1.21E+09	1.15E+09	1.18E+09	2.26E+09
1.34E+09	1.53E+09	8.81E+08	8.28E+08	1.02E+09	6.14E+08	5.72E+08	4.55E+08	1.24E+09
3.1E+09	2.58E+09	2.97E+09	2.16E+09	2.85E+09	2.4E+09	2.59E+09	2.38E+09	3.17E+09
56493000	66610000	45400000			59728000	53701000		54746000
1.12E+08	1.41E+08	1.49E+08	1.78E+08	1.22E+08	1.01E+08	1.36E+08	1.24E+08	80058000
1.42E+08	1.09E+08		70346000		88678000		64649000	87686000
	73388000		51369000	1.02E+08		44870000	67825000	1.35E+08
1.19E+08	1.32E+08	1.37E+08	1.69E+08	1.23E+08	1.11E+08	1.04E+08	94700000	1.17E+08
1.18E+08	84914000	1.15E+08	1.4E+08	1.34E+08	70035000	1.15E+08	65502000	87467000
73931000	1.18E+08							
4.51E+08	5.75E+08	5.12E+08	3.96E+08	4.46E+08	3.08E+08		4.94E+08	4.29E+08
6.49E+08			6.81E+08	4.85E+08	6.94E+08	7.67E+08	6.22E+08	7.01E+08
1.06E+09	8.62E+08	5.46E+08	8.07E+08	7.39E+08	3.65E+08	3.51E+08	4.01E+08	1.38E+09
6.3E+08	7.82E+08	7.18E+08	5.92E+08	4.85E+08	6.08E+08	5.51E+08	6.53E+08	7.72E+08
1.27E+09	1.35E+09	1.18E+09	9.13E+08	1.06E+09	1.15E+09	1.62E+09	1.33E+09	1.12E+09
8.57E+08	7.44E+08	5.36E+08	1.1E+09	7.46E+08	4.5E+08	4E+08	5.24E+08	7.13E+08
2.55E+09	2.36E+09	2.16E+09	2.68E+09	2.55E+09	1.96E+09	2.32E+09	2.42E+09	3.05E+09
			30309000	28928000				
		1.41E+08	60369000	62376000	1.99E+08			
2.86E+08	3.37E+08	1.99E+08	2.44E+08	4.02E+08	1.79E+08	4.72E+08	3.13E+08	3.68E+08
1.63E+09	1.54E+09	1.1E+09	1.2E+09	1.29E+09	1.86E+09	1.72E+09	1.51E+09	1.76E+09
1.26E+09	1.1E+09	9.83E+08	8.24E+08	1.13E+09	1.84E+09	1.68E+09	1.28E+09	9.43E+08
5E+08	7.78E+08	4E+08	4.28E+08	3.74E+08	1.39E+08	1.37E+08	2.02E+08	1.12E+09
8.49E+08	9.53E+08	1.22E+09	1.13E+09	1.18E+09	1.06E+09	1.33E+09	1.16E+09	7.63E+08
			28726000	19625000	21017000			
8.15E+08	8.13E+08	7.71E+08	7.25E+08	7.1E+08	9.82E+08	7.59E+08	9.47E+08	7.5E+08
84986000	78494000	59727000		71204000	68643000	1.35E+08	68424000	76279000
51438000	1.2E+08	1.61E+08	1.07E+08	1.09E+08	1.68E+08	2.58E+08	2.21E+08	78905000
1.06E+08	1.72E+08	94810000	86039000	77261000	1.05E+08	89703000	1.07E+08	1.22E+08
4.53E+08	5.08E+08	4.23E+08	4.23E+08	3.78E+08	5.33E+08	4.96E+08	4.86E+08	5E+08
1.6E+08	1.05E+08	1.41E+08	1.43E+08	1.18E+08				1.45E+08
2.57E+08	1.75E+08	2.21E+08	2.04E+08	1.95E+08	1.72E+08	1.27E+08	1.37E+08	1.91E+08
59515000		54151000	50128000	57992000		57376000		60306000
73207000		86038000	78445000	86085000	1.16E+08	94992000	1.13E+08	64437000
2.82E+08	4.1E+08	2.67E+08	3.13E+08	3.84E+08	2.62E+08	3.5E+08	3.82E+08	5.3E+08
3.18E+08	2.9E+08	2.24E+08	2.41E+08	2.12E+08	2.41E+08	2.3E+08	2.19E+08	2.71E+08

3.44E+08	3.8E+08	4.46E+08	3.67E+08	4.17E+08	4.41E+08	5.25E+08	4.32E+08	4.06E+08
3.58E+08	3.58E+08	1.96E+08	2.02E+08	2.39E+08	2.73E+08	3.56E+08	3.14E+08	3.51E+08
57641000	49373000	40728000	48307000	37304000	50963000	57686000	65612000	43477000
1.81E+08	2.12E+08	1.46E+08	1.53E+08	1.6E+08	1.87E+08	2.28E+08	1.8E+08	1.77E+08
2.22E+08	3.03E+08	2.92E+08	2.38E+08	2.21E+08	2.79E+08	3.26E+08	3.53E+08	3.01E+08
1.04E+08			1.04E+08	1.23E+08	46136000			66438000
2.72E+08	2.13E+08	3.01E+08	3.24E+08	3.36E+08	2.9E+08	2.95E+08	2.61E+08	2.16E+08
		3.48E+08	5.91E+08	4.03E+08				
	23242000	92124000	99578000	1.1E+08	2.87E+08	3.06E+08	1.75E+08	
3.16E+08	3.65E+08	3.43E+08	3.06E+08	2.37E+08	2.89E+08	2.82E+08	2.9E+08	3.28E+08
5.5E+08	5.27E+08	4.95E+08	4.58E+08	4.68E+08	5.71E+08	5.64E+08	5.17E+08	4.47E+08
3.17E+08	2.83E+08	2.36E+08	2.2E+08	2.39E+08	2.12E+08	1.99E+08	2.16E+08	3.7E+08
4.27E+08	3.95E+08	3.4E+08	2.96E+08	2.87E+08	3.14E+08	2.94E+08	2.84E+08	4.51E+08
			22937000	23410000		33320000	30982000	30330000
7E+08	7.36E+08	1.01E+09	8.8E+08	6.24E+08	6.15E+08	6.28E+08	6.44E+08	4.52E+08
	1.35E+08	75882000	1.49E+08	1.73E+08	95702000	2.48E+08	1.93E+08	1.27E+08
1.4E+08	1.38E+08	1.62E+08	1.57E+08	1.33E+08	1.21E+08	1.9E+08	1.12E+08	1.22E+08
5.85E+08	8.93E+08	6E+08	5.56E+08	4.87E+08	5.47E+08	6.53E+08	6.36E+08	1.1E+09
9.88E+08	9.14E+08				9.6E+08			5.41E+08
5.2E+08	8.7E+08	3.82E+08	4.26E+08	3.07E+08	6.19E+08	5.93E+08	4.86E+08	5.45E+08
1.45E+08	1.23E+08	1.32E+08	1.62E+08	1.4E+08	1.39E+08	1.87E+08	1.57E+08	1.5E+08
				34309000				
57516000	65162000	94342000	73824000	64059000	92529000	87194000	1.13E+08	55904000
					96873000	1.05E+08	88674000	
1.07E+08	1.21E+08	1.4E+08	1.16E+08	1.11E+08	1.33E+08	1.28E+08	1.4E+08	96437000
5.05E+08	6.33E+08	3.6E+08	3.03E+08	3E+08	5.29E+08	5.43E+08	4.61E+08	4.29E+08
1.31E+10	1.68E+10	1.48E+10	1.49E+10	1.38E+10	1.33E+10	1.19E+10	1.27E+10	1.7E+10
1.06E+08	1.69E+08	1.05E+08	75549000	68489000	52922000	65307000	90086000	1.18E+08
5.37E+08	9.01E+08	6.13E+08	5.03E+08	4.24E+08	5.81E+08	5.05E+08	6.12E+08	
3.79E+08	4.04E+08	2.76E+08	2.55E+08	2.24E+08	1.84E+08	1.89E+08	2.1E+08	3.64E+08
76742000	63123000	57650000	46565000	58782000	46289000	39530000	51433000	74994000
85798000	1.1E+08	1.58E+08	1.56E+08	1.74E+08				1.43E+08
3.53E+08	1.65E+08	2.73E+08	4.33E+08	3.94E+08	3.14E+08	1.8E+08	2.55E+08	2.15E+08
6.16E+08	5.43E+08	4.24E+08	5.11E+08	4.42E+08	6.47E+08	5.95E+08	6.94E+08	6.58E+08
1.06E+09	9.36E+08	8.74E+08	7.02E+08	6.99E+08	6.86E+08	1.08E+09	9.18E+08	1.02E+09
6.43E+08	8.4E+08	5.95E+08	4.24E+08	3.63E+08	5.63E+08	5.07E+08	5.06E+08	6.88E+08
4.4E+08	9.23E+08	6.27E+08	3.55E+08	4.81E+08	6E+08	4.56E+08	3.82E+08	6.06E+08
18743000	32945000		21456000	29982000	24180000	40623000	44542000	10688000
4.44E+08	3.34E+08	3.55E+08	2.53E+08	2.92E+08	2.1E+08	1.54E+08	2.15E+08	3.1E+08
62763000	67277000	60436000		42629000	38822000			58957000
30126000	38179000	60110000	47222000	41310000	96511000	20887000	46452000	42139000
1E+08	1.3E+08	1.31E+08	89846000	97363000	1.04E+08	1.14E+08	1.22E+08	1.56E+08

2.24E+08	1.36E+08	3.94E+08	3.89E+08	3.53E+08	3.5E+08	3.24E+08	3.26E+08	68946000
39578000	22307000	48547000	32401000	43424000	59172000	1.27E+08	79874000	37221000
4.55E+08	4.01E+08	4.19E+08	3.53E+08	3.1E+08	2.77E+08	4.39E+08	2.75E+08	4.04E+08
1.25E+09	1.22E+09	1.88E+08		3.05E+08	1.5E+08	8.1E+08	4.16E+08	4.55E+09
70091000	66467000	89232000	74705000	73243000	1.17E+08	90210000	76668000	74331000
5.01E+08	4.61E+08	4.34E+08	3.82E+08	3.59E+08	3.95E+08	4.25E+08	4.57E+08	5.24E+08
69087000	42601000	1.66E+08	2.9E+08	1.78E+08	1.27E+08	2.05E+08	2.29E+08	52855000
1.45E+08	2.18E+08		92911000	1.03E+08	1.17E+08	86438000	82793000	1.19E+08
	96702000	79840000	56111000	54425000	73482000	52962000	70082000	95749000
			80489000		84315000			82268000
49218000		94063000	73152000	32662000	83817000	45192000	1.17E+08	49217000
1.69E+08	1.31E+08	1.14E+08	1.31E+08	1.37E+08	1.74E+08	1.65E+08	1.49E+08	1.69E+08
1.18E+09	1.06E+09	1.14E+09	1.49E+09	1.41E+09	1.6E+09	1.61E+09	2.01E+09	1.23E+09
5.3E+09	6.22E+09	3.39E+09	3.9E+09	3.59E+09	5.81E+09	5.15E+09	5.05E+09	5.5E+09
1.59E+08	1.91E+08	1.3E+08	1.33E+08	1.46E+08	1.71E+08	1.33E+08	1.48E+08	1.34E+08
8.42E+08		1E+09	1.01E+09	8.55E+08	9.53E+08	6.44E+08	9.08E+08	6.36E+08
9.06E+08	9.55E+08	9.77E+08	9.45E+08	7.43E+08	6.48E+08	4.97E+08	6.25E+08	7.84E+08
1.71E+09	1.66E+09	1.34E+09	8.5E+08	8.34E+08	9.65E+08	1.01E+09	1.04E+09	1.68E+09
1.65E+08	2.02E+08	2.38E+08	1.87E+08	2.34E+08	1.98E+08	2.06E+08	2.19E+08	2.65E+08
37436000		53139000	34944000	39202000	51818000		37576000	26594000
89425000	99559000	1.1E+08	80537000	88420000	97179000	1.11E+08	1.12E+08	92077000
1.02E+08	1.05E+08							
	26136000	35765000	22954000	26283000	23900000	47108000	52574000	28011000
	1.68E+08	99213000	1.24E+08		1.32E+08	1.02E+08	1.26E+08	1.18E+08
	1.38E+08	1E+08		1.1E+08	1.05E+08		97795000	1.33E+08
2.98E+08	2.77E+08	4.79E+08	4.1E+08	4.27E+08	2.55E+08	3.15E+08	3.35E+08	1.27E+08
	63278000			58238000			53037000	
2.6E+08	2.65E+08	4.47E+08	4.05E+08	4.11E+08	5.03E+08	4.91E+08	5.72E+08	3.13E+08
1.6E+08	1.61E+08	2.08E+08	1.67E+08	1.75E+08	2.12E+08	2.68E+08	1.96E+08	2.14E+08
3.09E+08	2.62E+08	2.42E+08	3.06E+08	3.42E+08	3.28E+08	2.67E+08	2.75E+08	2.11E+08
52129000		58977000	55275000	59604000			71077000	
2.5E+08	1.82E+08	1.6E+08	1.83E+08		2.16E+08	1.73E+08	1.84E+08	1.91E+08
								54228000
				1.74E+08				
1.06E+08	1.15E+08	67851000	98466000	1.31E+08	53343000	72309000	50953000	95473000
3.45E+08	2.8E+08	6.38E+08	6.99E+08	8.65E+08	8.61E+08	4.99E+08	7.97E+08	2.23E+08

3.56E+08	1.36E+08	4.25E+08	3.98E+08	4.58E+08	3.7E+08	1.71E+08	2.05E+08	3.33E+08
60621000	44750000	1.16E+08	90181000	97484000	3.68E+08	3.44E+08	3.58E+08	
6.71E+09	6.98E+09	5.49E+09	8.78E+09	8.14E+09	2.39E+09	3.09E+09	3.7E+09	1.36E+10
83452000	1.16E+08	89766000	1.1E+08	67467000	82502000	88830000	86423000	85439000
2.57E+08		2.75E+08	2.39E+08	1.76E+08	1.61E+08			
	1.82E+09							
7.46E+08	5.64E+08	2.55E+08	4.76E+08	2.77E+08		1.54E+08		3.57E+08
1.6E+08	1.56E+08	1.18E+08	99286000	98782000	1.23E+08	1.37E+08	91037000	1.77E+08
1.97E+08		2.28E+08	2.44E+08	4.74E+08	3.7E+08	3.22E+08	2.57E+08	2.06E+08
51866000	68244000	66560000	62376000	54150000	57814000	46664000	55416000	66699000
1.72E+08	1.82E+08	1.52E+08	2.15E+08	1.89E+08	1.41E+08	2.26E+08	2.76E+08	1.95E+08
2.29E+08	2.76E+08	2.92E+08	3.03E+08	2.55E+08	3.68E+08	3.01E+08	3.99E+08	2.36E+08
2.28E+08	1.91E+08	3.82E+08	3.44E+08	3.45E+08	3.83E+08	3.19E+08	3.2E+08	1.73E+08
1.48E+08	1.46E+08	80932000	1.56E+08	1.7E+08	2.34E+08	62264000	1.26E+08	1.94E+08
	24313000	24781000	38568000	26516000				
1.38E+08	1.59E+08	1.55E+08	1.53E+08	1.5E+08	1.76E+08	1.69E+08	1.52E+08	1.04E+08
1.06E+09	9.94E+08	5.65E+08	7.18E+08	7.21E+08	5.97E+08	7.84E+08	9.95E+08	1.05E+09
85279000		98637000	1.05E+08	1.15E+08	75417000	63897000	86523000	72015000
3.05E+09	1.82E+09	1.71E+09	2.06E+09	2.01E+09	1.07E+09	9.1E+08	1.1E+09	2.34E+09
		3.4E+08			5.8E+08	2.61E+08	3.19E+08	
	64794000	38035000	39146000	39994000	40712000	66256000		62015000
61451000			44472000	45932000				
3.57E+08	5.15E+08	2.38E+08	3.06E+08	3.07E+08	2.69E+08	2.35E+08	3.16E+08	3E+08
3.14E+08	3.01E+08	2.66E+08	2.75E+08	2.49E+08	3.3E+08	3.07E+08	2.93E+08	4.66E+08
1.06E+08	1.74E+08	1.5E+08	1.26E+08	2.6E+08	3.36E+08	2.07E+08	2.89E+08	1.52E+08
7.42E+09	6E+09	9.37E+09	6.19E+09	7.83E+09	7.49E+09	7.24E+09	6.73E+09	4.55E+09
3.53E+08	3.13E+08	2.83E+08	2.31E+08	2.23E+08	2.58E+08	2.37E+08	2.23E+08	2.4E+08
88876000		1.12E+08		66714000	80575000		81280000	1.16E+08
5.27E+08	6.88E+08	5.79E+08	5.34E+08	5.25E+08	5.41E+08	7.47E+08	7.36E+08	5.68E+08
80066000	1.32E+08	31424000	51037000	42135000				1.22E+08
2.36E+08	1.73E+08	1.54E+08	1.35E+08	1.47E+08	1.2E+08	94428000	1.54E+08	3.21E+08
1.75E+09	2.39E+09	1.22E+09	1.08E+09	8.18E+08	1.11E+09	1.02E+09	9.37E+08	2.45E+09
73005000	75503000	67776000	47445000	38189000	48194000	31968000	31912000	76968000
27601000	48495000		31514000	24367000	43869000	40556000	49073000	26587000
38521000								45714000
72912000	99327000	1.27E+08	1.09E+08	1.03E+08	1.38E+08	1.49E+08	1.68E+08	1.05E+08

55112000	44474000	49462000	51513000	41510000	48503000	52981000	62319000	49931000
		34728000	25411000	31297000	36798000	30609000	30730000	22252000
80359000	2.1E+08	30995000	51496000	49691000	30048000	25562000	1.13E+08	87979000
6.72E+08	9.36E+08	6.44E+08	4.65E+08	4.52E+08	6.44E+08	1.14E+09	6.27E+08	7.99E+08
		2405800		1230100	1822000	32055000	4661300	1292600
93592000		60555000	74878000	54348000	44455000		22060000	84885000
2.25E+09	2.77E+09	2.56E+09	2.93E+09	2.67E+09	3.11E+09	2.74E+09	3.1E+09	2.61E+09
3.7E+08	4.91E+08	4.69E+08	5.5E+08	3.54E+08	3.63E+08	3.25E+08	3.85E+08	3.18E+08
2.75E+09	2.72E+09	2.24E+09	2.34E+09	2.08E+09	2.65E+09	2.59E+09	2.74E+09	2.99E+09
			22388000	16788000				32584000
50132000		43694000	41683000	32619000	31985000	62807000	39184000	99677000
42807000	60705000							61446000
2.38E+09	2.22E+09	2.65E+09	3.19E+09	3.09E+09	3.18E+09	2.78E+09	3.49E+09	3.63E+09
3.76E+08	5.26E+08	4.71E+08	1.18E+08	4.29E+08		6.3E+08	5.91E+08	4.15E+08
1.89E+08	1.9E+08	1.65E+08	1.66E+08	2.05E+08	2.96E+08	2.59E+08	1.99E+08	1.72E+08
3.61E+08	3.26E+08	4.79E+08	3.94E+08	3.51E+08	5.58E+08	4.86E+08	4.06E+08	2.87E+08
	75786000	64324000	90638000	81762000	57664000	71569000	62067000	1.13E+08
2.53E+08	2.57E+08	3.03E+08	2.38E+08	2.18E+08	2.4E+08	3.01E+08	3.16E+08	2.7E+08
1.39E+08	1.77E+08	2.13E+08	1.81E+08	1.57E+08	2.11E+08	2.23E+08	2.21E+08	1.63E+08
1.68E+08	1.43E+08	2.51E+08	3.38E+08	3.54E+08	1E+08	84673000	1.18E+08	4.32E+08
	1.36E+08							
4.93E+09	6.09E+09	3.54E+09	3.98E+09	3.77E+09	2.08E+09	2.49E+09	2.29E+09	4.97E+09
2.08E+08	2.08E+08	1.33E+08	1.49E+08	1.35E+08	1.3E+08	1.23E+08	1.51E+08	1.84E+08
2.39E+08	2.59E+08	2.46E+08	2.33E+08	1.72E+08	2.18E+08	2.51E+08	2.41E+08	2.31E+08
3.91E+08	3.61E+08	3.17E+08	1.93E+08	2.08E+08	2.95E+08	2.25E+08		3.7E+08
5.66E+08	8.87E+08	3.31E+08	6.91E+08	2.79E+08	2.14E+08	2.72E+08	3.58E+08	7.29E+08
		71214000		1.15E+08		96290000	1.03E+08	1.25E+08
69022000	1.05E+08	1.2E+08	1.15E+08	87150000	95370000	69874000	74583000	73604000
69818000	90005000			69771000	1.31E+08	1.32E+08	96468000	99296000
1.03E+09	8.5E+08	7.42E+08	7.81E+08	7.26E+08	7.33E+08	7.04E+08	7.09E+08	1.05E+09
3.44E+08	5.18E+08	3.39E+08	3.47E+08	2.62E+08	4.4E+08	4.09E+08	4.39E+08	3.09E+08
1.1E+08	1.38E+08	1.74E+08	1.17E+08	88930000	1.01E+08	1.56E+08	1.09E+08	1.68E+08
2.72E+08	2.58E+08	2.48E+08	2.48E+08	2.21E+08	1.66E+08	2.11E+08	2.17E+08	3.42E+08
	47433000	1.09E+08	90574000	68206000	1.72E+08	1.45E+08	1.1E+08	34917000
3.89E+09	2.51E+09	2.46E+09	3.41E+09	3.29E+09	2.05E+09	1.71E+09	1.81E+09	2.38E+09
	4.61E+08	61408000	51491000	45658000			49775000	2.33E+08
2.28E+08	1.78E+08	2.36E+08	2.32E+08	2.13E+08	2.32E+08	2.48E+08	2.5E+08	1.98E+08
2.62E+09	2.67E+09	3.33E+09	3.28E+09	3.53E+09	3.24E+09	2.96E+09	3.02E+09	2.15E+09
2.51E+09	2.19E+09	1.18E+09	1.16E+09	1.09E+09	1.35E+09	8.89E+08	1.21E+09	2.31E+09
27432000		52678000	53331000	43955000	41501000	51021000	66049000	51462000

4.71E+09	3.06E+09	2.73E+09	2.37E+09	2.45E+09	1.8E+09	1.4E+09	1.5E+09	2.57E+09
8.21E+08	6.26E+08	4.37E+08	6.35E+08	9.3E+08	7.14E+08	8.55E+08	7.11E+08	7.58E+08
9.26E+08	4.62E+08	8.8E+08	1.3E+09	1.39E+09	1.03E+09	9.23E+08	8.35E+08	5.89E+08
7.57E+08	9.29E+08	6.09E+08	6.27E+08	5.61E+08	4.44E+08	6E+08	5.15E+08	9.22E+08
1.97E+08	2.45E+08	1.27E+08	2.14E+08	2.27E+08	52838000	1.16E+08	1.32E+08	4.52E+08
	1.55E+08	1.24E+08		1.07E+08	1.44E+08		1.55E+08	1.57E+08
1.07E+08	2.38E+08	1.05E+08	78056000	81822000			2.03E+08	1.41E+08
8.2E+08	9.82E+08	7.76E+08	8.69E+08	7.12E+08	8.94E+08	8.27E+08	9.81E+08	1.02E+09
5.32E+08	5.15E+08	4.86E+08	3.81E+08	4.9E+08	7.18E+08	6.21E+08	6.33E+08	5.41E+08
3.73E+09	3.59E+09	2.87E+09	3.38E+09	3.12E+09	3.65E+09	3.54E+09	3.18E+09	3.93E+09
2.44E+09	2.1E+09	1.41E+09	1.38E+09	1.77E+09	2.25E+09	2.25E+09	1.81E+09	2.36E+09
3.85E+09	3.77E+09	3.26E+09	3.28E+09	3.18E+09	3.04E+09	2.82E+09	2.97E+09	3.05E+09
2.22E+08	3.2E+08	3.25E+08	3.59E+08	3.9E+08	4.15E+08	6.12E+08	4.46E+08	3.21E+08
81295000	60810000	1.1E+08	1E+08	92478000	1.17E+08	1.14E+08	1.26E+08	94935000
1.78E+10	1.97E+10	1.17E+10	1.05E+10	1.21E+10	1.22E+10	1.14E+10	1.07E+10	1.77E+10
	89602000	61600000	59333000	69038000	70885000			72105000
2.52E+08	3.26E+08	2.46E+08	2.05E+08		2.94E+08	2.8E+08	2.4E+08	2.77E+08
2.19E+08	2.51E+08	2.46E+08	2.76E+08	2.32E+08	2.4E+08	1.96E+08	1.99E+08	2.38E+08
2.25E+08	3.03E+08	2.02E+08	1.69E+08	1.34E+08	2.51E+08	2.57E+08	2.09E+08	2.01E+08
42603000		46622000	32668000	41997000	61078000	37069000	53229000	56283000
4.33E+08	7.22E+08	3.06E+08	2.44E+08	2.85E+08	2.26E+08	2.18E+08	1.8E+08	4E+08
4.08E+08	4.13E+08	3.38E+08	3.17E+08	3.46E+08	4.78E+08	4.28E+08	4.78E+08	3.6E+08
62933000	79586000	1.25E+08	1.06E+08	1.32E+08	1.86E+08	3.61E+08	2.5E+08	84450000
			46601000	1.1E+08				
8.47E+08	7.06E+08	8.46E+08	9.73E+08	1.06E+09	8.86E+08	6.26E+08	7.63E+08	4.97E+08
78781000	65327000	39400000	44125000	48899000	50236000	40792000	45907000	54625000
2.5E+08	5.84E+08			2.76E+08				4.32E+08
				24555000		37387000	33581000	
2.76E+08		2.71E+08	2.51E+08	1.92E+08	2.39E+08	2.07E+08	2.72E+08	
28277000	31481000	2.05E+08	1.27E+08	1.47E+08	1.85E+08	3.36E+08	1.76E+08	35512000
8E+08	8.09E+08	1.13E+09	1.33E+09	1.14E+09	1.04E+09	1.27E+09	1.31E+09	8.73E+08
2.34E+08	2.04E+08	1.94E+08	1.7E+08	1.59E+08	2.04E+08	1.77E+08	2.03E+08	1.86E+08
5.3E+08	4.63E+08	5.85E+08	4.7E+08	4.69E+08	6.01E+08	5.62E+08	5.46E+08	5.63E+08
2.04E+08	2.01E+08	1.57E+08	1.83E+08	2.07E+08	2.59E+08	2.85E+08	3.47E+08	2.73E+08
			22383000	20746000	25477000	24622000	23795000	
				83609000	91501000	1.47E+08	82564000	1.09E+08
8.71E+08	7.8E+08	5.86E+08	6.63E+08	6.44E+08	3.33E+08	4.46E+08	4.48E+08	7.62E+08
							99611000	
65535000				69344000				81941000
36927000		62320000	74870000	44293000	51020000		54365000	47408000

							19921000	
51570000	71683000	74892000	55930000	51720000	58101000	69055000	71459000	54365000
5.66E+08	6.17E+08	3.44E+08	4.69E+08	4.71E+08	5.12E+08	4.41E+08	4.16E+08	6.36E+08
3.65E+08	2.62E+08	3.37E+08	2.24E+08	2.7E+08	2.37E+08	2.3E+08	2.21E+08	5.37E+08
2.55E+08	2.23E+08	2.41E+08	2.72E+08	3.13E+08	3.02E+08	3.75E+08	3.2E+08	2.95E+08
	98493000	75154000	59542000	42293000	64277000	66842000	72323000	81875000
						22488000	18851000	
53890000	64189000	69167000	59840000	58893000	67623000	62524000	70953000	52466000
7.11E+09	6.26E+09	6.85E+09	4.29E+09	4.97E+09	5.57E+09	4.88E+09	5.14E+09	6.71E+09
1.57E+09	1.35E+09	1.71E+09	1.08E+09	1.26E+09	1.43E+09	1.25E+09	1.38E+09	1.5E+09
24207000			32637000					
1.26E+08	1.98E+08	1.96E+08	1.6E+08	1.23E+08	1.65E+08	1.43E+08	1.94E+08	1.14E+08
1E+08	1.87E+08	1.06E+08	82597000	1.02E+08		1.39E+08	1.47E+08	1.15E+08
70012000	1.08E+08	71544000	83600000		74326000		70276000	64748000
7.26E+08	7.73E+08	4.97E+08	4.46E+08	5.42E+08	7.06E+08	5.02E+08	6.17E+08	5.92E+08
								79061000
1.35E+08	1.56E+08	1.18E+08	1.51E+08	1.4E+08	1.27E+08	69827000	1.4E+08	1.55E+08
1.84E+08			1.59E+08	1.52E+08	1.31E+08			2.54E+08
1.76E+09	1.3E+09	1.32E+09	1.26E+09	1.52E+09	2.49E+09	2.65E+09	1.71E+09	1.92E+09
3.35E+08	4.21E+08	2.81E+08	2.69E+08	2.18E+08	2.34E+08	2.65E+08	2.3E+08	4.43E+08
2.55E+08	2.54E+08	2.42E+08	2.54E+08	2.56E+08	2.05E+08	2.29E+08	2.41E+08	2.57E+08
81990000	73535000	1.12E+08	1.05E+08	96356000	1.43E+08	92597000	1.52E+08	80194000
2.34E+08	3.07E+08	2.31E+08	2.08E+08	1.94E+08	2.4E+08	2.54E+08	2.32E+08	2.3E+08
95069000	1.35E+08	1.28E+08	1.12E+08	1.08E+08	1.37E+08	1.03E+08	1.35E+08	1.3E+08
1.4E+09	1.35E+09	6.3E+08	5.83E+08	8.81E+08	6.92E+08	9.45E+08	7.1E+08	1.18E+09
	2.66E+08		1.65E+08			98260000		2.01E+08
91505000			72750000	83856000		1.04E+08	82831000	95828000
2.98E+08	2.19E+08	1.56E+08		1.74E+08	1.77E+08	2.1E+08	2.25E+08	3.31E+08
1.4E+09	1.06E+09	1.83E+09	2.2E+09	2.46E+09	3.12E+09	2.8E+09	2.92E+09	1.41E+09
2.99E+08	1.79E+08	1.45E+08	97561000	2.16E+08	1.42E+08	1.65E+08	94650000	1.26E+08
	71535000	99580000	89354000	74168000	80879000	1.06E+08	1.09E+08	56618000
3.87E+08	3.78E+08	3.39E+08	2.85E+08	2.94E+08	2.93E+08	2.91E+08	2.94E+08	4.82E+08
3.82E+08	2.61E+08	2.79E+08	4.18E+08	3.7E+08	3.25E+08	2.82E+08	3.08E+08	2.55E+08
3.52E+08	3.29E+08	2.73E+08	2.78E+08	2.66E+08	2.1E+08	1.7E+08	2.09E+08	3.71E+08
8.37E+08	9.86E+08	1.09E+09	1.55E+09	1.2E+09	6.88E+08	5.34E+08	8.8E+08	1.35E+09
5.38E+08	6.28E+08	4.66E+08	4.63E+08	4.37E+08	4.91E+08	5.99E+08	5.46E+08	6.78E+08
54553000		69868000	64366000		54869000	74366000	63744000	76851000
1.01E+08	1.23E+08	1.38E+08	1.1E+08	90902000	1.11E+08	1.2E+08	1.23E+08	1.11E+08
5.32E+08	6E+08	5.06E+08	6.57E+08	4.36E+08	4.28E+08	4.3E+08	5.51E+08	7.89E+08

		18340000	15780000	14783000	15768000	16736000		22392000
72192000	92481000	1.85E+08	1.33E+08	1.18E+08	1.71E+08	1.96E+08	2.01E+08	70481000
85348000	68569000	1.54E+08	1.26E+08	1.2E+08	1.24E+08	1.46E+08	1.79E+08	83404000
1.54E+08	1.51E+08		1.75E+08	1.84E+08	2.36E+08	1.72E+08	1.95E+08	1.26E+08
2.68E+08	2.59E+08	4.86E+08	3.97E+08	2.98E+08	4.2E+08	3.49E+08	3.69E+08	2.78E+08
1.34E+08	1.38E+08	1.08E+08	1.18E+08	1.1E+08		1.12E+08	83606000	1.46E+08
4.75E+08	5.44E+08	5.11E+08	5.06E+08	4.21E+08	5.62E+08	4.63E+08	5.41E+08	4.94E+08
38425000			35496000		34006000	32851000	40384000	32928000
49609000	68560000	42141000	37398000	54452000				1.16E+08
56709000	52864000	47657000	45208000	53819000	60763000	69656000	58863000	56288000
93350000	94296000	75218000	76798000	90501000	53696000	67809000	87770000	92431000
1.85E+09	1.6E+09	1.9E+09	1.94E+09	2.01E+09	1.73E+09	1.61E+09	1.87E+09	1.92E+09
1.57E+09	1.48E+09	2.11E+09	2.41E+09	2.57E+09	2.51E+09	2.62E+09	2.6E+09	1.62E+09
2.06E+08	2.46E+08	1.44E+08	1.29E+08	2.02E+08	1.52E+08	1.72E+08	1.9E+08	1.52E+08
6.93E+08	7.39E+08	5.6E+08	5.49E+08	5.42E+08	6.65E+08	8.09E+08	5.49E+08	6.84E+08
8.66E+08	8.85E+08	7.31E+08	6.66E+08	6.64E+08	7.26E+08	6.67E+08	7.54E+08	7.95E+08
53073000						31965000		
1.3E+08	81620000	62975000	97471000	95618000	81310000		1.1E+08	1.05E+08
		47672000		30049000		41595000		
52286000	55488000	84961000	76645000	73706000	70483000	81491000	89784000	38960000
81552000	1.9E+08	2.78E+08	2.87E+08	2.17E+08	1.53E+08	98279000	1.26E+08	1.54E+08
								2.72E+08
41439000	44533000	42627000	33017000	28145000	36634000	38911000	41958000	44854000
2.14E+09	1.86E+09	1.77E+09	1.58E+09	2.01E+09	2.42E+09	2.03E+09	2.08E+09	2.3E+09
44708000	65674000			49563000				58780000
	3.1E+08	2.56E+08				2.49E+08		2.8E+08
35867000				28277000	19378000	32943000	42916000	
1.26E+09	1.56E+09	2.31E+09	2.42E+09	2.43E+09	1.97E+09	2.77E+09	2.85E+09	1.28E+09
4.74E+09	4.59E+09	5.59E+09	3.7E+09	6.51E+09	1.84E+09	1.98E+09	3.65E+09	3.54E+09
8.14E+08	6.94E+08	3.18E+09	1.99E+09	2.77E+09	2.67E+09	5.41E+09	3.48E+09	5.98E+08
53457000								47305000
								29703000
	1.04E+08	64915000	49058000	34588000			56452000	93854000
76005000	1.07E+08	76257000	61984000	60630000	67754000	74571000	55723000	85746000
								44223000
13778000		18198000	16670000	14077000				
2.47E+08		91965000			1.12E+08	1.8E+08		
67268000	22585000	28106000	54608000	35239000	32355000	37504000	39987000	25343000
1.59E+08	1.81E+08	1.27E+08	1.18E+08	1.41E+08	1.75E+08	1.49E+08	1.3E+08	1.74E+08
38703000	36435000	85978000	59052000	52582000	52197000			55179000
								2.05E+08
1.59E+08			2.75E+08	1.84E+08	1.48E+08			

	5.23E+08		4.07E+08	5.03E+08		4.7E+08	3.29E+08	7.18E+08
50483000		35386000	52707000	42207000	22081000			48016000
2.68E+08	3E+08	2.98E+08	2.86E+08	2.28E+08	2.31E+08	2.88E+08	3.26E+08	2.94E+08
2.53E+08	2.84E+08	2.66E+08	2.18E+08	1.93E+08	1.16E+08	1E+08	1.29E+08	2.42E+08
1.01E+08	75633000	95488000	1.27E+08	1.42E+08	1.59E+08	1.18E+08	1.06E+08	73832000
51413000			56050000	55489000				42569000
1.1E+08	1.96E+08	1.32E+08	1.05E+08	96266000	1.23E+08	1.48E+08	1.38E+08	1.52E+08
6.8E+08	6.53E+08	4.43E+08	2.27E+08	3.95E+08	4.34E+08	4.83E+08	4.01E+08	6.43E+08
1.31E+08	1.62E+08	99806000	1.33E+08	1.14E+08	1.39E+08	1.36E+08	1.21E+08	1.54E+08
1.17E+08	86712000	87281000	84113000	1.02E+08	98373000	1.29E+08	1.07E+08	78280000
1.69E+08	1.8E+08	1.56E+08	2.4E+08	2.15E+08	1.91E+08	1.61E+08	1.82E+08	1.55E+08
77629000	57556000	33675000	49816000	73907000		33269000	45033000	58987000
	1.03E+08				78956000	1.44E+08	1.28E+08	
1.54E+08	1.73E+08	1.19E+08	1.1E+08	1.19E+08	1.38E+08	1.33E+08	1.01E+08	1.63E+08
1.06E+08	1.3E+08	1.56E+08	1.11E+08	87398000	1.24E+08	1.26E+08	1.2E+08	1.49E+08
3.17E+08	3.75E+08	3.36E+08	1.93E+08	2.29E+08	2.46E+08	1.73E+08	3E+08	3.55E+08
2.36E+08		4.8E+08	3.33E+08	1.28E+08				
9E+08	1.07E+09	1.02E+09	1.06E+09	9.7E+08	1.18E+09	1.1E+09	1.13E+09	1.06E+09
5.24E+08	5.1E+08	4.77E+08	4.07E+08	4.54E+08	4.81E+08	4.21E+08	4.44E+08	7.59E+08
4.75E+08	4.88E+08	5.52E+08	5.25E+08	4.58E+08	6.42E+08	5.52E+08	6.05E+08	5.13E+08
1.04E+08	91834000	1.04E+08	1.1E+08	1.34E+08	1.32E+08	1.19E+08	1.21E+08	1.04E+08
4.3E+08	7.8E+08	6.96E+08	9.21E+08	6.65E+08	4.54E+08	5.38E+08	6.57E+08	3.61E+08
1.95E+08	1.97E+08	1.5E+08	1.39E+08	1.35E+08	64268000	79079000	74820000	1.41E+08
2.4E+08	1.19E+08	2.2E+08	2.44E+08	1.68E+08	1.57E+08	1.48E+08	1.78E+08	1.77E+08
			12350000	1.03E+08				
1.66E+08	2.08E+08	2.2E+08	1.78E+08	1.73E+08	2.23E+08	1.78E+08	1.87E+08	1.72E+08
2.77E+09	3.62E+09	2.41E+09	2.02E+09	1.93E+09	1.86E+09	1.89E+09	1.74E+09	3.35E+09
1.28E+08	1.27E+08	1.14E+08	82825000	90581000	86049000	83807000	78000000	1.29E+08
4.37E+08	3.88E+08	5.28E+08	3.21E+08	3.48E+08	3.16E+08	4.63E+08	3.47E+08	4.63E+08
4.02E+08	2.47E+08	2.5E+08	3.97E+08	3.24E+08	2.34E+08	2.64E+08	3.66E+08	4.13E+08
82529000		1.85E+08	1.29E+08	1.14E+08	1.52E+08	1.75E+08	1.44E+08	85981000
68692000	67327000	71157000	68121000	62216000	75591000	72378000	90342000	72324000
			33060000	37851000			33015000	31608000
	95563000	78529000			94428000		1.17E+08	42611000
1.05E+09	1.33E+09	8.8E+08	8.44E+08	6.59E+08	7.89E+08	7.56E+08	8.29E+08	1.43E+09

			50886000		25695000	20347000	32125000	
	1.79E+08	61487000		31197000	29276000	1.09E+08	47162000	92264000
2.25E+08	2.12E+08	2.27E+08	2.47E+08	1.78E+08	1.34E+08		1.4E+08	4.35E+08
94733000	51021000	77076000	1.04E+08	95202000	82856000	75383000	90664000	1.14E+08
1.81E+08	1.42E+08	1.1E+08	1.23E+08	1.28E+08	1.41E+08	1.15E+08	85289000	1.57E+08
	79570000	44785000		47166000				71986000
1.34E+08	1.35E+08	3.33E+08	3.64E+08	2.31E+08	3.87E+08	2.77E+08	3.45E+08	93290000
		1.3E+08		1.07E+08	1.5E+08	1.49E+08	1.43E+08	
3.45E+09	3.52E+09	3.35E+09	4.34E+09	3.91E+09	2.36E+09	2.81E+09	3.08E+09	6.72E+09
6.69E+08	5.43E+08	3.83E+08	4.35E+08	4.36E+08	3.35E+08	2.95E+08	3.75E+08	7.03E+08
99208000	1.72E+08	83570000	59546000	47425000	81318000	82364000	85194000	1.62E+08
91200000	1.38E+08	78268000	73484000	70243000	89667000	26919000	81607000	81611000
1.28E+08								
8.62E+08	8.8E+08	4.13E+08	3.55E+08	5.21E+08	5.94E+08	4.91E+08	3.11E+08	9.93E+08
	1.1E+08	77205000	84568000	67343000	85969000	71043000	70403000	61733000
1.08E+08	1.75E+08	2.08E+08	1.4E+08	1.28E+08	1.07E+08	1.04E+08	1.54E+08	1.24E+08
1.31E+09	7.37E+08	2.28E+09	2.12E+09	2.27E+09	1.62E+09	1.43E+09	1.52E+09	9.8E+08
1.86E+08	1.66E+08	2.12E+08	99128000	1.8E+08	1.91E+08	1.99E+08	1.71E+08	2.09E+08
1.57E+08	1.54E+08	2.94E+08	3.58E+08	3.32E+08	3.33E+08	3.49E+08	3.69E+08	1.89E+08
56241000	57820000	1.47E+08	1.37E+08	1.19E+08	1.47E+08	1.38E+08	1.27E+08	43883000
39887000		82087000	52845000	65259000	65123000	80972000	56558000	
45443000		35003000	39770000	34599000		26525000	33268000	
79419000		1.1E+08	47994000	1.15E+08	89875000		67172000	
1.67E+09	1.47E+09	2.07E+09	1.69E+09	2E+09	2.63E+09	1.83E+09	2.02E+09	1.25E+09
1.76E+08	1.81E+08							2.05E+08
44679000		51929000	58933000	52314000	27293000	31749000	58486000	55463000
2.96E+08	2.32E+08	4.2E+08	4.11E+08	5.7E+08	6.15E+08	6.2E+08	6.8E+08	2.82E+08
	70989000		57202000	55114000				
1.87E+08	2.18E+08	2.43E+08	2.38E+08	2.1E+08	2.02E+08	2.22E+08	2.59E+08	1.92E+08
27889000	43504000	64628000	33292000	38162000	39928000	48458000	52774000	
1.09E+09	2.04E+09	1.25E+09	1.01E+09	1.48E+09	6.7E+08	1.11E+09	1.05E+09	1.4E+09
			21962000					47697000
24763000	18103000	21334000	41551000	35128000	44278000	53818000	41499000	51027000
83710000	85094000	78897000	76877000	82246000	71025000	73599000	67897000	55957000
2.64E+08	2.3E+08	1.87E+08	2.08E+08	2.34E+08	2.22E+08	2.48E+08	2.47E+08	2.75E+08
	1.06E+08							
			1.38E+08				1.17E+08	

4.05E+08	3.65E+08	3.55E+08	3.72E+08	3.85E+08	2.99E+08	3.41E+08	4.16E+08	4.33E+08
1.5E+08			2.02E+08					1.45E+08
				69902000			1.1E+08	
2.46E+08	2.34E+08	2.24E+08	1.7E+08	1.64E+08	2.4E+08	1.92E+08	2.29E+08	2.91E+08
7.81E+08	7.74E+08	6.67E+08	8.23E+08	7.45E+08	6.62E+08	6.91E+08	7.73E+08	8.08E+08
3.54E+08	3.92E+08	5.19E+08	3.35E+08	3.85E+08	5.08E+08	5.5E+08	5.42E+08	2.68E+08
4.42E+08	3.9E+08	4.86E+08	4.81E+08	9.64E+08	4.27E+08	4.96E+08	4.7E+08	2.83E+08
39181000			57476000	42372000	35291000	24116000	39494000	
1.35E+08	1.58E+08	1.8E+08	1.6E+08	1.39E+08	1.55E+08		1.8E+08	1.5E+08
98959000	1.37E+08	1.15E+08	2.63E+08	1.04E+08	1.07E+08	95928000	1.73E+08	85077000
	4.28E+08							4.3E+08
56729000	84943000	65509000	99985000	66753000	66341000	1.01E+08	1.15E+08	66156000
7.86E+08	1E+09	6.66E+08	5.38E+08	4.89E+08	5.57E+08	5.85E+08	5.52E+08	9.55E+08
								44956000
2.2E+08	1.48E+08	2.4E+08	2E+08	2.06E+08	2.46E+08	1.78E+08	2.09E+08	1.17E+08
15352000		48120000	29653000	51613000	36266000	81403000	57577000	
2.55E+08	2.25E+08	2.41E+08	2.19E+08	1.99E+08		1.69E+08	2.46E+08	2.26E+08
						23210000	40644000	27938000
54768000	53509000	36958000	36396000	18981000	29254000	36014000		55516000
3.05E+08	3.56E+08	3.17E+08	1.87E+08	2.08E+08	2.03E+08	1.78E+08	1.98E+08	3.89E+08
62908000				57310000				72451000
30194000	38944000	32580000	28418000	26033000		38711000	27525000	37601000
19262000					25140000			
		95550000	1.04E+08	77122000	76051000	41100000	62853000	64735000
74029000	1.07E+08		44061000	55514000	38160000	60135000	44560000	58551000
1.97E+08	2.8E+08	2.01E+08	3.07E+08	1.92E+08	1.71E+08	2.11E+08	1.97E+08	3.87E+08
46162000		45931000	64427000	58053000	57564000		57918000	55353000
2.78E+08	1.73E+08	1.93E+08	1.89E+08	2.21E+08	2.55E+08	2.73E+08		3.03E+08
2.45E+08	2.89E+08	1.84E+08	1.63E+08	1.62E+08	1.26E+08	1.65E+08	1.68E+08	4.51E+08
	42415000			39942000			40430000	
41731000	23258000		18513000	24244000				52688000
4E+08	3.39E+08	4.78E+08	4.61E+08	4.2E+08	5.92E+08	5.2E+08	5.67E+08	3.33E+08
3.06E+08	2.95E+08	2.37E+08	2.66E+08	2.92E+08	3.46E+08	3.28E+08	3.86E+08	3.31E+08
4.66E+08	9.69E+08	3.83E+08	6.95E+08	7.16E+08	1.05E+08	1.61E+08	2.69E+08	1.67E+09
2.21E+09	2.05E+09	2.35E+09	1.54E+09	2.02E+09	2.16E+09	1.69E+09	2.01E+09	1.42E+09
2.52E+08	2.26E+08	1.76E+08	1.85E+08	1.55E+08	1.44E+08	1.22E+08	1.43E+08	2.78E+08
	19422000							
2E+08	2.2E+08	2.43E+08		2E+08	2.11E+08	1.64E+08	2.11E+08	3.19E+08

					35653000			
1.16E+08	1.17E+08	1.64E+08	1.39E+08	1.46E+08	2.29E+08	2.2E+08	2.41E+08	1.07E+08
2.39E+08		2E+08		3.46E+08	3.49E+08	3.98E+08	3.19E+08	
5.34E+09	4.48E+09	2.14E+09	2.37E+09	2.05E+09	3.24E+09	3.79E+09	2.43E+09	6.94E+09
				38544000				57804000
				58566000		57122000	65988000	65739000
91045000	1.02E+08	93233000	95715000			90780000	1E+08	1.25E+08
1.1E+09	1.38E+09	1.47E+09	1.45E+09	1.35E+09	1.35E+09	1.39E+09	1.31E+09	1E+09
6.63E+08	6.78E+08	1.33E+09	7.21E+08	8.16E+08	1.28E+09	1.23E+09	1.27E+09	4.36E+08
2.83E+08	3.04E+08	2.95E+08	2.48E+08	2.7E+08	2.8E+08	1.78E+08	2.49E+08	2.08E+08
51469000	46476000	44041000	48471000	37504000			32118000	
	3.71E+08			2.25E+08	1.92E+08	1.89E+08	1.49E+08	2.03E+08
61307000	69664000	62172000	47719000	45709000	30198000	34490000	38247000	39000000
	41751000	43719000	34030000	26801000	21623000			29076000
64982000	77451000	96667000	74640000	61447000	85118000	88840000	84953000	76817000
1.15E+08	1.06E+08	1.57E+08	1.38E+08	1.39E+08	1.35E+08	2.29E+08	2.66E+08	1.54E+08
3.89E+08	4.9E+08	4.8E+08	4.56E+08	4.49E+08	2.03E+08	1.61E+08	3.03E+08	2.56E+08
5.84E+08	7.36E+08	2.97E+08	3.78E+08	3.44E+08	1.66E+08	2.88E+08	3.21E+08	7.41E+08
1.33E+08	2.01E+08	1.35E+08	1.24E+08	1.23E+08		1E+08	1.17E+08	3.13E+08
1.06E+08	94468000	63142000	74151000	91211000	83354000	91210000	92200000	86380000
2.33E+08	2.78E+08	2.79E+08	2.32E+08	2.3E+08	2.43E+08	2.66E+08	2.85E+08	2E+08
52472000		53095000	48127000	43284000	43898000	67999000	54149000	55877000
1.76E+08	1.83E+08	1.49E+08	1.27E+08	1.2E+08	1.1E+08	1.12E+08	1.11E+08	1.98E+08
3.94E+08	4.42E+08	2.32E+08	2.64E+08	2.93E+08	2.28E+08	2.85E+08	2.33E+08	5.29E+08
		67961000						88135000
47268000	46992000	25298000	35773000	32487000	34962000	33367000	30445000	55054000
1.8E+09	2.12E+09	1.5E+09	1.31E+09	1.14E+09	1.2E+09	1.14E+09	1.16E+09	1.99E+09
4.18E+08	5.87E+08	3.84E+08	4.09E+08	3.06E+08	3.87E+08	3.7E+08	3.96E+08	2.78E+08
1.16E+08	92033000	66599000	74569000	1.13E+08	1.37E+08	1.19E+08		59680000
30067000	34778000	36165000				31286000		
1.54E+08		96045000	1.39E+08	77163000	1.33E+08	89017000	1.31E+08	1.53E+08
2.51E+08	3.66E+08	2.69E+08	2.37E+08	2.28E+08	2.27E+08	2.07E+08	2.12E+08	2.94E+08
				1.42E+08				
1.83E+08	2.04E+08		68599000	61501000				2.66E+08
		45524000	35244000	50841000	44945000	42484000		68567000
3.56E+08	4.37E+08	6.16E+08	5.29E+08	6.36E+08	5.52E+08	8.52E+08	6.89E+08	3.94E+08
			29883000		30888000	44256000	33811000	50110000
1.09E+09	1.21E+09	2.52E+09	2.4E+09	2.29E+09	2.36E+09	2.47E+09	1.98E+09	1.13E+09

1.33E+08	1.87E+08	2E+08	2.13E+08	2.11E+08	1.6E+08	2.71E+08	1.62E+08	1.93E+08
2.22E+08	2.2E+08	1.99E+08	1.37E+08	1.33E+08	2.43E+08	2.26E+08	1.78E+08	1.66E+08
		26491000		19365000	25563000			23042000
2.09E+08	1.49E+08	2.21E+08	1.92E+08	1.75E+08	2.24E+08	1.9E+08	1.9E+08	2.13E+08
1.98E+09	2E+09	1.21E+09	1.1E+09	1.59E+09	1.48E+09	1.29E+09	9.1E+08	1.9E+09
1.73E+08	2.76E+08	1.87E+08	1.33E+08	1.41E+08	1.92E+08	2.42E+08	1.72E+08	2.15E+08
4.99E+08	3.91E+08	3.02E+08	3.75E+08	3.8E+08	3.23E+08	3.44E+08	3.04E+08	4.47E+08
3.15E+09	3.25E+09	3.17E+09	3.6E+09	3.14E+09	3.94E+09	3.62E+09	3.66E+09	3.12E+09
85631000	89279000	1.11E+08	1.18E+08	94949000	58272000	1.07E+08	1.27E+08	73501000
3.9E+08	4.49E+08	5.17E+08	3.66E+08	3.08E+08	4.87E+08	6.36E+08	5.69E+08	3.17E+08
1.59E+09	1.5E+09	4.65E+08	6.2E+08		6.67E+08	1.01E+09	5.25E+08	1.32E+09
				20443000	27440000	16839000		
1.81E+08	1.76E+08	73222000	86157000	95894000	99841000	76197000	38039000	1.74E+08
43718000	63074000	44867000	28104000	28026000				45410000
32621000				22815000	51957000	49344000	56064000	18282000
59608000				42603000				
81136000	87617000	78428000	67382000	30472000	82615000	81240000	79032000	72030000
1.2E+09	5.62E+08	6.16E+08	5.25E+08	5.65E+08		2.93E+08	6.49E+08	4.47E+08
2.52E+08	2.7E+08	2.83E+08	4.38E+08	3.15E+08	3.15E+08	4.89E+08	3.78E+08	3.69E+08
			63005000	49612000			70537000	64689000
		48964000	42592000	34540000			44815000	
7.64E+08	7.63E+08	4.17E+08	5.23E+08	5.52E+08	4.26E+08	3.02E+08	4.33E+08	6.63E+08
60381000	89706000	62249000	74132000		79319000	66570000	82966000	63670000
5.54E+08	4.6E+08	4.27E+08	4.6E+08	3.98E+08	4.71E+08	4.52E+08	3.94E+08	5.52E+08
6.86E+08	1.21E+09	6.6E+08	6.53E+08	5.35E+08	6.01E+08	2.6E+08	5.11E+08	1.11E+09
			45648000	38341000				70594000
1.38E+08	2.03E+08	1.76E+08	1.32E+08	1.25E+08	1.48E+08	1.55E+08	1.63E+08	1.74E+08
1.79E+08	1.54E+08	91492000	1.26E+08	1.66E+08	1.22E+08	1.25E+08	1.01E+08	1.62E+08
1.15E+08	99779000	85968000	1.06E+08	1.27E+08	1.29E+08	1.99E+08	1.76E+08	1.02E+08
1.59E+08	1.26E+08							3.3E+08
1.55E+08	1.54E+08	1.93E+08	2.72E+08	2.4E+08	2.8E+08	2.12E+08	2.33E+08	1.14E+08
5.15E+08	5.19E+08	7.44E+08	7.54E+08	6.57E+08	7.36E+08	7.02E+08	7.01E+08	3.02E+08
49200000	42511000							37969000
	33442000			50320000	34062000			
7.98E+08		4.16E+08	4.72E+08	4.92E+08				
1.48E+08	1.56E+08	1.34E+08	1.15E+08					1.48E+08
2.42E+09	1.85E+09	1.71E+09	1.8E+09	1.76E+09	7.46E+08	6.8E+08	6.92E+08	2.28E+09
		1.49E+08						

1.36E+08	1.88E+08	1.56E+08	1.14E+08	1.34E+08	1.34E+08	1.03E+08	1.24E+08	2E+08
1.08E+08	1.07E+08	59993000	66141000	92816000	1.11E+08	99775000	91448000	1.04E+08
4.21E+08	3.12E+08	5.05E+08	4.37E+08	3.99E+08	5.81E+08	5.2E+08		4.39E+08
4.29E+08	3.36E+08	4.04E+08	5.87E+08	3.85E+08	3.18E+08	5.01E+08	4.79E+08	6.37E+08
98536000	1.16E+08	1.19E+08	98416000	76516000	1.15E+08	1.27E+08	1.16E+08	74915000
1.51E+08		1.53E+08	1.32E+08	1.41E+08	2.06E+08	1.69E+08	1.85E+08	
7.05E+08	6.09E+08	3.46E+08	4.04E+08	3.28E+08	2.53E+08	2.17E+08	2.52E+08	8.41E+08
5.96E+08	6.87E+08	7.21E+08	7.05E+08	7.69E+08	7.09E+08	7.84E+08	7.03E+08	7.29E+08
6.75E+08	7.41E+08	6.41E+08	9.69E+08	7.76E+08	1.04E+09	1E+09	9.02E+08	7.29E+08
1.02E+09	9.68E+08	9.86E+08	8.08E+08	7.64E+08	7.25E+08	5.8E+08	5.11E+08	1.04E+09
3.24E+08	4.66E+08	3.65E+08	4.67E+08	4.79E+08	4.04E+08	2.66E+08	4.45E+08	4.18E+08
56507000			31532000		50755000	82610000		
28113000	46774000		50323000		37112000		39337000	70045000
	91579000	78882000	92028000	74122000	86693000		1.05E+08	99257000
8.54E+08	8.06E+08	4.37E+08	2.17E+08	4.82E+08	3.7E+08	1.09E+09	4.59E+08	1.12E+09
	89143000	67250000	76102000	69210000	83055000		68248000	97002000
42357000	53881000	76689000	57541000	51740000	1.19E+08	1.24E+08	1.07E+08	57750000
80402000	1.11E+08	1.23E+08	80462000	66049000	1.83E+08	1.63E+08	1.46E+08	90182000
2.1E+09	1.68E+09	5.84E+08	8.04E+08	9.06E+08	4.93E+08	3.96E+08	4.82E+08	2.68E+09
		1.75E+08	1.47E+08	1.39E+08	1.69E+08	1.72E+08	1.98E+08	2.06E+08
		53773000	48752000	43571000	47775000	46413000		
1.33E+09	1.35E+09	1.88E+09	1.51E+09	1.83E+09	2.68E+09	2.31E+09	2.13E+09	1.61E+09
6.06E+08	6.29E+08	4.52E+08	4.27E+08	3.48E+08	5.2E+08	4.09E+08	4.58E+08	6.18E+08
1.25E+08	1.53E+08	1.03E+08	1.05E+08	76863000	1E+08	1.13E+08	1.13E+08	1.33E+08
1.79E+08	3.19E+08	1.64E+08	2.28E+08	2.08E+08	2.97E+08	2.45E+08	2.02E+08	2.87E+08
1.54E+08	1.6E+08	1.87E+08	1.83E+08	1.65E+08	1.88E+08	1.8E+08	1.62E+08	1.37E+08
6.37E+08	7.05E+08	4.95E+08	4.72E+08	3.96E+08	3.99E+08	3.65E+08	3.41E+08	5.02E+08
8.09E+08	6.66E+08	1.36E+09	1.41E+09	1.45E+09	1.37E+09	1.01E+09	1.18E+09	5.26E+08
1.1E+08	93885000	1.47E+08	1.41E+08	1.19E+08	1.09E+08	1.11E+08	1.47E+08	1.03E+08
4.51E+08	6.34E+08	4.46E+08	3.27E+08	3.44E+08	3.38E+08	4.45E+08	4.14E+08	5.12E+08
62635000	97468000							58674000
					1.14E+08	1.06E+08	1.07E+08	2.15E+08
						23296000		
20720000						29442000		
	31652000	57720000	62594000	64038000	39764000	57146000	1.04E+08	62085000
2.9E+08	1.84E+08	2.34E+08	2.83E+08	2.74E+08	2.5E+08	1.46E+08	2.12E+08	1.66E+08
4.16E+08	3.51E+08	3.06E+08	2.8E+08	2.77E+08	2.82E+08	3.17E+08	2.95E+08	4.04E+08
4.69E+08	4.6E+08	4.67E+08	4.41E+08	4.24E+08	5.14E+08	4.8E+08	6.01E+08	4.8E+08
6.12E+08	6.77E+08	6.48E+08	4.85E+08	5.05E+08	9.57E+08	1.04E+09	9.95E+08	7.6E+08
1.54E+08	78656000	1.39E+08	1.47E+08	1.41E+08	2.23E+08	2.35E+08	2.66E+08	94812000
3.33E+08	2.37E+08	3.99E+08	2.89E+08	2.41E+08	5.29E+08	4.71E+08	5.59E+08	2.53E+08
	67657000	76585000	51043000	45450000	55744000		79100000	35518000
		1.68E+08	2.14E+08	1.61E+08			1.87E+08	
2.53E+08	3.22E+08	3.03E+08	2.65E+08	2.61E+08	3.84E+08	2.33E+08	3.7E+08	2.62E+08
3.53E+08	4.13E+08	3.8E+08	2.84E+08	2.85E+08	2.78E+08	3.03E+08	2.8E+08	3.45E+08

4.41E+08	5.74E+08	4.93E+08	3.93E+08	3.8E+08	4.93E+08	5.01E+08	5E+08	4.79E+08
								51958000
47466000	55911000	51575000	51714000	37870000	55371000	56235000	47683000	48265000
2.06E+08	1.97E+08	2.48E+08	2.3E+08	2.22E+08	2.4E+08	2.5E+08	2.64E+08	1.69E+08
1.89E+09	2.04E+09	2.14E+09	2.26E+09	2.09E+09	2.44E+09	2.68E+09	2.81E+09	1.99E+09
3.43E+08	3.4E+08	3.5E+08	3.53E+08	3.55E+08	3.28E+08	3.09E+08	2.95E+08	4.38E+08
				60542000				
5.18E+08	5.05E+08	5.31E+08	6.17E+08	5.72E+08	4.24E+08	4.01E+08	5.63E+08	5.36E+08
	36152000	49449000	15740000	26071000		31440000	46827000	35965000
5.75E+08	6.8E+08	7.75E+08	7.81E+08	7.14E+08	7.03E+08	6.53E+08	6.67E+08	6.99E+08
65911000	89530000	89334000		71954000	30104000	96833000	1.12E+08	82579000
1.42E+08	1.94E+08	1.42E+08	1.74E+08	1.63E+08	1.72E+08	2.12E+08	2.27E+08	2.38E+08
1.37E+09	1.32E+09	1.06E+09	8.07E+08	8.68E+08	6.59E+08	7.88E+08	7.89E+08	1.51E+09
43625000	54871000	46741000		32237000	42636000			58412000
1.21E+08	1.39E+08	94165000	91451000	96195000	1.2E+08	1.37E+08	97820000	1.39E+08
		51051000	40382000	37763000	40170000		46582000	38298000
1.72E+08		1.97E+08	2.78E+08	2.27E+08			2.22E+08	
34522000		1.81E+08	2E+08	1.78E+08	1.19E+08	1.08E+08	95067000	58237000
46390000	34505000	95807000	91467000	86175000	80798000	81506000	62638000	30022000
60202000	62218000	1.76E+08	1.64E+08	1.28E+08	1.38E+08	1.43E+08	1.04E+08	48360000
52135000	60116000	56430000	63482000	45078000		39478000	57559000	52086000
	54819000	37999000	46173000	44335000	43656000		56397000	55221000
			44030000	40154000	70170000	54983000	1.33E+08	
				3.83E+08				
1.47E+08	2.17E+08	1.37E+08	1.38E+08	1.19E+08	1.05E+08	1.7E+08	1.98E+08	1.6E+08
1.39E+09	1.13E+09	1.12E+09	1.16E+09	1.15E+09	1.28E+09	1.44E+09	1.67E+09	1.76E+09
						42637000		
5.99E+08	5.02E+08	4.61E+08	4.54E+08	4.71E+08	6.52E+08	6.63E+08	5.78E+08	5.23E+08
4.73E+08	3.58E+08	3.53E+08	4.02E+08	3.62E+08	5.67E+08	5.26E+08	5.5E+08	4.78E+08
2.75E+08	3.06E+08	2.26E+08	1.86E+08	1.59E+08	2.57E+08	3.03E+08	2.65E+08	3.07E+08
3.43E+09	3.17E+09	3.22E+09	4.25E+09	4.32E+09	3.13E+09	2.04E+09	2.85E+09	4.19E+09
2.13E+08	2.45E+08	1.92E+08	2.21E+08	1.28E+08	85295000	2.33E+08	1.47E+08	2.74E+08
7.4E+08	7.93E+08	6.19E+08	6.69E+08	5.19E+08	3.01E+08	3.14E+08	3.32E+08	8.47E+08
1.2E+08	1.06E+08	89856000	32037000					
32993000	59177000	42439000	38929000	28088000				64142000
2.11E+08			1.25E+08	62501000	1.14E+08	1.27E+08	1.57E+08	2.98E+08
7.32E+08	7.7E+08	8.34E+08	7.73E+08	8.2E+08	9.25E+08	8.05E+08	8.48E+08	8.45E+08
3.58E+08	2.79E+08	6.61E+08	1.11E+09	8.85E+08	4.57E+08	3.94E+08	5.01E+08	4.17E+08
1.24E+09	1.44E+09	9.29E+08	1E+09	9.75E+08	9.96E+08	9.67E+08	8.99E+08	1.45E+09
1.64E+08	1.65E+08	1.47E+08	1.33E+08	1.46E+08	1E+08	1.82E+08	1.66E+08	2.22E+08
2.34E+08	56894000	5.11E+08	2.83E+08	3.15E+08	4.38E+08	5.35E+08	3.66E+08	2.98E+08
2.06E+09	2.3E+09	1.83E+09	2.27E+09	1.9E+09	8.24E+08	8.76E+08	1.04E+09	3.84E+09

78555000	74972000	85413000	64276000	82965000	90996000	1.1E+08	97461000	91387000
99151000	1.09E+08		66760000	52922000	60957000	57144000	71620000	1.03E+08
4.12E+08	4.83E+08	4.92E+08	4.24E+08	3.99E+08	5.1E+08	5.28E+08	4.83E+08	4.61E+08
			39884000		37436000			
43553000	40953000	29543000	31666000	30784000	84041000	68783000	70071000	48166000
		16250000	24323000	23349000	39778000	33738000	35763000	
5.33E+09	4.28E+09	2.73E+09	2.55E+09	2.98E+09	2.49E+09	1.9E+09	2.22E+09	4.26E+09
1.73E+08	2.23E+08	1.13E+08	1.04E+08	1.37E+08	2.73E+08	1.95E+08		1.8E+08
58643000	42262000	51792000	41764000	51908000	40693000			95757000
2.69E+08	3.23E+08	1.92E+08	2.26E+08	2.04E+08	2.09E+08	1.81E+08	2.59E+08	4.67E+08
33605000	37491000	32917000	45703000	33209000				
90011000	1.01E+08	85274000	78596000	82314000	89022000	1.04E+08	84213000	1.1E+08
3.05E+08	3.97E+08	3.42E+08	2.68E+08	2.61E+08	2.56E+08	2.39E+08	2.36E+08	2.48E+08
3.14E+08	3.31E+08	2.47E+08	2.08E+08	2.24E+08	2.5E+08	2.67E+08	2.52E+08	4.01E+08
8.35E+08	7.59E+08	3.87E+08	3.65E+08	3.58E+08	1.92E+08	1.95E+08	2.9E+08	8.55E+08
1.45E+08		64440000	76779000	78723000	90285000	48053000		
2.85E+08		3E+08	2.3E+08	2.61E+08				
35298000	39991000	26267000	33897000	30444000	20567000	35430000	31314000	42671000
1.61E+08	1.79E+08						1.06E+08	1.69E+08
6.53E+08	6.21E+08	3.87E+08	4.04E+08	3.81E+08	3.88E+08	3.25E+08	3.91E+08	8.02E+08
7.28E+08	6.02E+08	3.73E+08	4.12E+08	4.22E+08	3.26E+08	2.99E+08	3.31E+08	7.52E+08
1E+08	1.26E+08		63090000	70141000	56962000	53613000	47921000	92908000
45239000								
2.66E+08	3.26E+08	1.71E+08	1.56E+08	1.61E+08	1.36E+08	1.25E+08	1.39E+08	2.9E+08
2.47E+08	2.93E+08	1.69E+08	1.14E+08	1.36E+08	1.45E+08	1.25E+08	1.28E+08	2.72E+08
6.37E+08	3.09E+08	4.51E+08	6.13E+08	5.4E+08	4.22E+08	1.76E+08	4.08E+08	3.36E+08
34283000			32564000					36470000
								1.74E+08
1.96E+08	2.27E+08	2.58E+08	1.65E+08	1.49E+08	2.12E+08	1.89E+08	2.25E+08	2.16E+08
7.79E+08	9.43E+08	7.79E+08	8.62E+08	7.95E+08	1.26E+09	1E+09	1.2E+09	8.99E+08
2.46E+08	1.64E+08	2.25E+08	2.5E+08	2.5E+08	1.09E+08	56822000	72791000	2.71E+08
2.51E+08	2.79E+08	2.47E+08	3.85E+08	2.79E+08	96985000	84855000	1.37E+08	6.22E+08
2.87E+08	2.34E+08	2.3E+08	2.13E+08	2.18E+08	2.36E+08	4.31E+08	2.88E+08	2.4E+08
1.59E+08	2.29E+08	65618000	1.01E+08	1.12E+08	1.01E+08	1.55E+08	1.75E+08	1.55E+08
86978000		99228000					83726000	1.13E+08
1.11E+08	1.78E+08	1.57E+08	1.16E+08	1.02E+08	1.39E+08	1.03E+08	1.28E+08	1.38E+08
62426000	90061000	58284000	55175000	41444000	38516000	39929000	40978000	76782000
2.8E+08	3.32E+08	2.12E+08	1.97E+08	1.99E+08	2.2E+08	2.53E+08	2.24E+08	3.76E+08
1.68E+08	1.83E+08	1.5E+08	1.66E+08	1.12E+08	1.97E+08	2.32E+08	2.04E+08	1.89E+08

1.43E+08				1.14E+08	1.86E+08			1.45E+08
77048000	1.19E+08	80963000	96829000	80519000	96654000	89268000	88636000	1.04E+08
	84753000					97242000	1.09E+08	78737000
4.52E+08	6.37E+08	4.39E+08	4.38E+08	3.89E+08	6.45E+08	6.12E+08	7.94E+08	6.24E+08
60689000	13493000	19557000	52885000	66242000	47513000	60854000	50349000	46026000
							4.12E+08	
2.87E+08	2.75E+08	1.85E+08		1.82E+08	2.4E+08	2.3E+08	2.3E+08	2.42E+08
5.33E+08	1.03E+09	1.29E+09	1.24E+09	1.02E+09	7.18E+08	6.33E+08	9.01E+08	4.51E+08
53524000	51323000	46463000		36567000			62896000	65421000
44868000	68147000	48756000	38899000	46126000	64591000	1.26E+08	66817000	47623000
2.34E+08	1.75E+08	2.34E+08	1.74E+08	2.6E+08	4.42E+08	2.44E+08	3.92E+08	3.61E+08
			67144000	1.08E+08				
8.22E+09	8.42E+09	5.43E+09	4.76E+09	5.04E+09	3.49E+09	3.96E+09	3.14E+09	7.77E+09
6.46E+08	7.6E+08	3.48E+08	3.68E+08	3.44E+08				7.14E+08
5.51E+08	3.88E+08	2.21E+08	2.5E+08	2.7E+08	1.61E+08	1.68E+08	1.47E+08	4.57E+08
3.41E+08	2.52E+08	2.91E+08	3.43E+08	4.3E+08	2.16E+08	1.67E+08	1.89E+08	2.82E+08
6.07E+08	5.41E+08	3.96E+08	4.67E+08	6.62E+08	5.66E+08	6.09E+08	3.85E+08	7.04E+08
6.39E+08	6.98E+08	3.82E+08	4.13E+08	4.45E+08	3.1E+08	3.37E+08	2.86E+08	6.91E+08
71327000	77507000		45454000	57656000		58399000		96091000
7.74E+08	7.96E+08	4.12E+08	4.93E+08	4.99E+08			2.14E+08	9.82E+08
1.08E+08	79770000	82090000	59161000	55845000	67833000			83827000
			69913000	95830000				77013000
1.32E+08	1.85E+08	1.28E+08	1.05E+08	80031000	1.25E+08	1E+08	1.54E+08	1.72E+08
								24708000
42947000	46136000	27800000	26795000	21015000	19996000		31697000	52302000
3.17E+08	2.86E+08	1.7E+08	2E+08	1.64E+08	2.66E+08	2.58E+08	2.22E+08	3.7E+08
2.44E+08	3.44E+08	1.93E+08	2.07E+08	1.49E+08	1.72E+08	1.56E+08	2.06E+08	2.76E+08
6.05E+08	4.62E+08	3.15E+08	5.28E+08	4.59E+08	4.22E+08	2.86E+08	2.69E+08	7.48E+08
			98682000					
65200000	90014000	1.14E+08	88677000	94777000	1.35E+08	1.82E+08	1.35E+08	62594000
5.78E+08	7.28E+08	4.23E+08	5.63E+08	4.7E+08	4.12E+08	4.16E+08	5.11E+08	7.65E+08
4.42E+08	3.5E+08			3.21E+08				3.94E+08
4.52E+08	7.12E+08	5.52E+08	4.58E+08	5.22E+08	5.79E+08	4.31E+08	6.98E+08	8.1E+08
1.56E+08	1.59E+08	1.14E+08	99898000	1.06E+08	1.24E+08	1.09E+08	1.28E+08	1.63E+08

57784000		1.02E+08	61951000	87690000	1.52E+08	93562000	1.07E+08	68224000
53791000	57384000		44315000	39736000	30337000	36222000	41210000	64673000
82559000	69920000	72199000	46263000	61418000	64976000	45744000	50755000	95820000
1.86E+08	1.94E+08	2.02E+08	1.57E+08	1.21E+08		1.85E+08	2.19E+08	1.82E+08
1.1E+08	1.23E+08	1.18E+08	2.12E+08	1.65E+08	1.38E+08	2.4E+08	2.12E+08	1.01E+08
32341000	65200000	60407000	51621000	71067000	93081000	65722000	77381000	43747000
		85908000	31385000	90376000	55825000	27669000	1.2E+08	
1.97E+08	1.79E+08	1.99E+08	1.96E+08	2.31E+08	2.54E+08	2.25E+08	2.55E+08	2.25E+08
57417000	73326000	54393000	35633000	33161000	36727000	31081000	40190000	35996000
1.28E+08	1.05E+08	1.28E+08	1.7E+08	1.43E+08	1.44E+08	1.25E+08	1.55E+08	1.4E+08
1.36E+08	99379000	1.9E+08	2.49E+08	2.98E+08	2.69E+08	2.97E+08	3.39E+08	1.55E+08
41098000	52528000		43403000					
			31387000	41850000	30530000			
40632000			46154000		48805000	55857000	51819000	44652000
			47151000			69515000		65730000
4.6E+08	2.22E+08	3.3E+08	3.23E+08	3.05E+08	3.4E+08	2.66E+08	3.88E+08	4.44E+08
3.02E+08	3.1E+08	3.35E+08	3.79E+08	3.85E+08	6.58E+08	5.65E+08	7.76E+08	4.16E+08
2.45E+09	2.4E+09	1.5E+09	2.47E+09	2.37E+09	4.59E+09	3.87E+09	4.52E+09	2.84E+09
1.89E+09	2.33E+09	1.61E+09	1.75E+09	1.54E+09	3.27E+09	2.77E+09	3.63E+09	2.84E+09
2.87E+08	2.88E+08	2.36E+08	2.09E+08	1.92E+08	1.11E+08	91475000	1.27E+08	5.02E+08
					30087000	16914000		
6.84E+08	5.99E+08	1.11E+09	1.18E+09	1.06E+09	1.61E+09	2E+09	1.97E+09	5.69E+08
			1.31E+08	1.25E+08			1.73E+08	
	82907000	2.22E+08	1.63E+08	1.29E+08	93190000	2.11E+08	1.33E+08	
2.03E+08	1.77E+08	1.42E+08	2.85E+08	2.09E+08	1.45E+08	62000000	1.13E+08	1.65E+08
6.17E+08	9.42E+08	5.48E+08	4.23E+08	4.24E+08	2.38E+08	2.44E+08	2.77E+08	8.33E+08
2.11E+08	1.49E+08	1.12E+08	1.71E+08	1.58E+08	1.56E+08	1.98E+08	1.25E+08	1.73E+08
1.19E+08	63551000	1.05E+08	1.12E+08	1.1E+08	1.6E+08	1.04E+08	1.34E+08	88419000
1.17E+08	1.11E+08	1.67E+08	73441000	1.44E+08	1.52E+08	1.48E+08	1.53E+08	1.18E+08
2.86E+08	1.41E+08		2.19E+08	3.25E+08	2.44E+08	2.31E+08	2.67E+08	
								95842000
46387000	91020000	69877000	98345000	58362000	64682000	85215000	67382000	42934000
45576000								
1.52E+08	1.44E+08	2.09E+08	2.54E+08	2.27E+08	1.36E+08		1.13E+08	4.91E+08
1.85E+08	2.25E+08	1.75E+08	1.39E+08	1.41E+08	2.34E+08	2.49E+08	1.61E+08	1.51E+08
70958000	1.03E+08	1.07E+08	87139000	77498000	79009000	67353000	79532000	82879000
			1.24E+08	45050000			1.55E+08	

17884000			19886000	20248000			17910000	
			18351000	18706000	20989000		28459000	
85553000	81214000	84023000	86518000	78738000	77554000	82082000	84111000	61585000
			16441000	16902000			57087000	
6.7E+08	7.59E+08	8.97E+08	1.09E+09	9.92E+08	8.8E+08	1.13E+09	9.24E+08	6.95E+08
4.65E+08	4.89E+08	1.66E+09	2.99E+09	1.47E+09	4.43E+08	3.37E+08	9.21E+08	1.83E+08
1.13E+08	1.56E+08	2.56E+08	2.89E+08	2.5E+08	79245000	62525000	1.63E+08	
79155000	96483000	49231000	53046000	53761000	45842000	40705000		1.08E+08
3.33E+08	4.57E+08	1.16E+08	2.74E+08	3.52E+08	68513000	4.75E+08	3.76E+08	4.47E+08
5.14E+08	4.73E+08	5.12E+08	5.27E+08	5.59E+08	5.73E+08	4.7E+08	5.36E+08	6.17E+08
	91290000		72078000	91601000				94852000
		77505000	87819000	80492000	65361000		69510000	1.38E+08
2.3E+08	2.67E+08	1.71E+08	1.91E+08	1.35E+08	77335000	59460000	81949000	3.37E+08
			56590000	65779000				
1E+08	1.07E+08	98945000	76193000	89432000	93044000	99773000	88038000	98888000
			75086000	1.09E+08	1E+08			
4.71E+08	6.01E+08	2.01E+09	1.55E+09	1.62E+09	2.96E+09	2.64E+09	1.83E+09	5.3E+08
						76324000		1.62E+08
2.59E+08	3.42E+08	1.1E+08	1.29E+08	1.34E+08	9.77E+08	3.04E+08	3.14E+08	
3.43E+08	2.94E+08	2.1E+08	2.01E+08	2.1E+08	5.93E+08	2.97E+08	5.65E+08	99614000
1.04E+09	8.85E+08	5.07E+08	9.93E+08	8.99E+08	1.15E+09	9.26E+08	1.05E+09	50406000
			54605000					
	55320000							91505000
5.49E+08	7.2E+08	3.8E+08	4.18E+08	4.31E+08	4.14E+08	2.86E+08	3.02E+08	5.67E+08
1.22E+08	1.15E+08	1.49E+08	1.4E+08	1.26E+08	1.2E+08	1.76E+08	1.73E+08	1.17E+08
1.56E+08	1.77E+08	2.95E+08	3.01E+08	3.78E+08	3.41E+08	5.13E+08	4.79E+08	2.51E+08
1.62E+08	1.97E+08	1.15E+08	1.61E+08	1.19E+08	1.71E+08	2.09E+08	1.94E+08	1.27E+08
		49303000	51449000	44841000	37640000	41930000	52093000	61204000
1.09E+08	1.71E+08	1.05E+08	99334000	84307000	90497000	1.13E+08	1.18E+08	1.66E+08
	1.34E+08		1.36E+08	97991000	1.58E+08			1.27E+08
		56038000	73744000	50773000	95411000	54709000	79464000	
85447000		67381000	60062000	48613000	44073000	76765000	46374000	71611000
3.19E+08	3.73E+08	2.54E+08	2.07E+08	3.06E+08	2.09E+08	1.92E+08		
7.94E+08	1.03E+09	2.22E+09	2.39E+09	2.53E+09	7.66E+08	9.91E+08	9.14E+08	1.39E+08
		1.08E+08	89485000	1.08E+08			1.04E+08	
2.58E+08	3.87E+08		2.3E+08			2.08E+08	2.21E+08	
49475000	76697000	18367000	46253000	54011000	41605000	77514000	62357000	1.21E+08
3.45E+09	3.35E+09	2.4E+09	3.64E+09	2.58E+09	2.25E+09	1.48E+09	1.92E+09	3.08E+09

2.47E+08	3.07E+08	3.75E+08	3.3E+08	3.34E+08	3.66E+08	3.66E+08	3.86E+08	2.48E+08

LFQ intens	LFQ intens	LFQ intens	LFQ intens	LFQ intens	LFQ intens	LFQ intens	LFQ intens	LFQ intens
1E+08	63338000	6.7E+08	6.3E+08	5.71E+08	4.44E+08	3.02E+08	2.98E+08	8.67E+08
7.8E+08	6.65E+08	5.31E+08	5.36E+08	4.85E+08	7.02E+08	8.24E+08	7.11E+08	6.89E+08
63676000	72419000	2.72E+08	3.21E+08	2.81E+08	2.4E+08	2.02E+08	2.47E+08	4.73E+08
3.59E+08	3.35E+08	1.84E+08	2.07E+08	1.99E+08	3.72E+08	3.6E+08	2.36E+08	2.38E+08
83342000	73203000	69682000	52379000	54143000				
1.46E+09	1.12E+09	6.15E+09	5.51E+09	6.27E+09	6.62E+09	5.35E+09	5.38E+09	1.51E+10
3.49E+08	4.62E+08	3.98E+08	4.16E+08	4.04E+08	5.08E+08	5.11E+08	5.88E+08	5.46E+08
3.36E+08	3.74E+08	3.18E+08	3.64E+08	2.84E+08	4.11E+08	4.67E+08	4.82E+08	3.69E+08
1.07E+08	1.49E+08	6.34E+08	3.78E+08	6.69E+08	1.39E+08	1.14E+08	1.19E+08	1.81E+08
22917000	37088000	2.6E+09	1.87E+09	2E+09	2.68E+08	64542000	1.51E+08	8.94E+08
1.42E+10	1.46E+10	2.15E+10	1.88E+10	2.04E+10	1.59E+10	1.68E+10	1.57E+10	1.75E+10
94430000		72671000	65785000	59579000				
4.94E+08	5.47E+08	4.21E+08	4.33E+08	3.86E+08	7.36E+08	7.17E+08	6.22E+08	5.27E+08
3.6E+08	3.67E+08	3.59E+08	3.36E+08	3.45E+08	3.62E+08	3.87E+08	3.82E+08	2.82E+08
2.32E+09	2.24E+09	1.27E+10	1.54E+10	1.51E+10	2.55E+09	2.48E+09	1.85E+09	2.89E+09
4.9E+08	3.7E+08	5.25E+08	4.57E+08	6.18E+08	5.71E+08	5.07E+08	5.07E+08	6.99E+08
65114000		89286000	83989000	93453000				
1.28E+10	1.31E+10	5.64E+09	4.81E+09	5.4E+09	1.51E+10	1.42E+10	9.85E+09	1.27E+10
5.75E+09	6.07E+09	8.39E+09	1.03E+10	1.01E+10	9.42E+09	7.39E+09	4.57E+09	9.38E+09
6.67E+09	5.72E+09	8.56E+09	7.48E+09	8.59E+09	7.64E+09	7.83E+09	6.17E+09	6.6E+09
2.58E+08	2.14E+08	3.63E+08	2.68E+08	2.66E+08	3.82E+08	3.74E+08	3.64E+08	2.47E+08
1.86E+08	1.4E+08	1.6E+08	1.52E+08	1.46E+08	1.24E+08	1.31E+08	83758000	1.47E+08
71347000	84334000	1.1E+08	42895000	57085000	2.2E+08			1.11E+08
	60341000			67592000				
6.95E+08	7.55E+08	8.91E+08	7.42E+08	7.67E+08	1.17E+09	1.26E+09	8.52E+08	1.05E+09
		1.06E+08	1.03E+08	1.23E+08		1.67E+08	1.65E+08	1.12E+08
4.85E+09	4.47E+09	6.88E+09	6.29E+09	5.72E+09	6.94E+09	6.85E+09	6.18E+09	6.34E+09
1.74E+09	1.89E+09	3.37E+09	3.59E+09	3.28E+09	1.66E+09	1.62E+09	1.65E+09	1.79E+09
77130000	81617000	68087000	88896000	74427000	78328000	87139000	99607000	1.03E+08
6.1E+08	6.67E+08	1.21E+09	1.18E+09	1.25E+09	5.99E+08	5.51E+08	4.59E+08	6.14E+08
3.04E+08	3.79E+08	4.64E+08	3.42E+08	3.39E+08	3.81E+08			3.42E+08
1.46E+08		3.72E+08	3.49E+08	2.64E+08				2.08E+08
	34909000		23760000					
1.83E+09	1.58E+09	3.28E+08	1.82E+08	3.95E+08	1.4E+09	1.77E+09	1.84E+09	7.65E+08
83652000	86425000	57909000	71984000	70063000	90873000	1.21E+08	1.11E+08	96281000
4.31E+08	5.1E+08	1.22E+09	1.25E+09	1.04E+09	1.11E+09	7.99E+08	1.04E+09	1.31E+09
5.81E+08	5.14E+08	4.91E+08	4.77E+08	5.22E+08	6.45E+08	5.5E+08	5.98E+08	4.52E+08
2.85E+08	3.52E+08	4.29E+08	4.06E+08	4.87E+08	3.78E+08	3.42E+08	3.41E+08	3E+08
		56540000		66349000		1.73E+08	1.21E+08	1.09E+08
4.25E+08	4.93E+08	5.33E+08	8.04E+08	5.61E+08	7.3E+08	4.94E+08	4.89E+08	5.33E+08
2.08E+08		2.34E+08	1.99E+08	1.69E+08				1.55E+08
2.72E+08	1.94E+09	4.84E+08	3.57E+08	3.34E+08				

3.22E+08	4.63E+08	3.14E+08	3.4E+08	3.41E+08	4.52E+08	3.51E+08	4.28E+08	4.32E+08
1.17E+08	1.04E+08	71448000	91029000	1.11E+08	1.24E+08	94185000	1.51E+08	1.25E+08
1.03E+09	1.15E+09	1.22E+09	1.22E+09	1.31E+09	1.16E+09	1.29E+09	1.2E+09	9.39E+08
9.62E+08	1.34E+09	9.62E+08	1.29E+09	9.29E+08	1.56E+09		1.58E+09	1.38E+09
31810000	58212000	71980000	91788000	97997000	61344000			34700000
4.77E+08	5.78E+08	6.47E+08	5.87E+08	4.85E+08	5.71E+08	4.61E+08	5.46E+08	3.64E+08
1.39E+09	1.58E+09	1.41E+09	1.48E+09	1.38E+09	2.18E+09	2.4E+09	2.15E+09	1.68E+09
4.84E+08	3.54E+08	4.05E+08	4.52E+08	4.08E+08	3.93E+08	4.21E+08	4.12E+08	2.52E+08
1.9E+08	2.28E+08	4.19E+08	4.09E+08	3.88E+08	1.6E+08	2.15E+08		1.76E+08
40094000	11561000							13842000
1.04E+09	1.04E+09	1.07E+09	8.91E+08	1.08E+09	1.02E+09	1.07E+09	7.5E+08	6.44E+08
6.52E+08	6.7E+08	1.15E+09	1.18E+09	1.14E+09	8.94E+08	8.24E+08	8.77E+08	9.29E+08
1.48E+09	1.43E+09	1.24E+09	1.24E+09	1.08E+09	1.45E+09	1.72E+09	1.34E+09	1.25E+09
1.71E+08	1.92E+08	2E+08	2.35E+08	2.14E+08	1.83E+08	1.79E+08	1.86E+08	1.3E+08
3.96E+08	4.68E+08	2.76E+08	2.09E+08	2.16E+08	4.64E+08	4.73E+08	2.83E+08	2.85E+08
1.14E+10	1.06E+10	6.84E+09	6.39E+09	6.31E+09	9.79E+09	1.22E+10	1.16E+10	9.72E+09
8.14E+08	8.77E+08	5.37E+08	5.52E+08	5.96E+08	8.81E+08	1.07E+09	1.01E+09	7.05E+08
1.46E+08		99318000	1.38E+08	1.13E+08	1.29E+08	1.36E+08		1.43E+08
1.32E+09	1.33E+09	1.32E+09	1.24E+09	1.18E+09	1.37E+09	1.37E+09	1.38E+09	1.26E+09
7.54E+08	8.49E+08	4.51E+08	4.58E+08	5.14E+08	6.68E+08	6.63E+08	4.99E+08	7.22E+08
2.25E+08	2.34E+08	3.68E+08	3.72E+08	4.03E+08	1.98E+08	2.19E+08	1.77E+08	1.8E+08
1.66E+09	1.67E+09	1.38E+09	1.66E+09	1.48E+09	1.83E+09	2.42E+09	1.98E+09	2.07E+09
4.15E+08	4.67E+08	2.02E+08	2.54E+08	1.93E+08	4.12E+08	4.6E+08	4.26E+08	2.77E+08
95750000		86675000					91771000	1.2E+08
		1.97E+08	1.83E+08	2.29E+08	1.11E+08		91885000	1.96E+08
2.08E+08	2.4E+08	96852000	1.65E+08	1.24E+08	2.82E+08	1.8E+08	2.27E+08	2.54E+08
5.04E+08	5.87E+08	4.83E+08	4.93E+08	5.09E+08	4.81E+08	5.65E+08	5.36E+08	4.06E+08
20657000		4332400		6721700		14003000	13168000	
1.08E+09	9.45E+08	2.06E+09	2.57E+09	2.01E+09	1.5E+09	1.22E+09	1.12E+09	1.72E+09
2.52E+09	2.39E+09	5.25E+09	3.92E+09	5.75E+09	4.19E+09	3.86E+09	3.37E+09	4.53E+09
8.53E+08		1.05E+09	1.1E+09	1.23E+09		9.15E+08		1.08E+09
1.01E+09	7.65E+08	1.32E+09	1.43E+09	1.3E+09	1.74E+09	1.36E+09	1.6E+09	1.78E+09
7.31E+08	6.56E+08	7.71E+08	4.26E+08	6.22E+08	3E+08	2.16E+08	2.95E+08	4.91E+08
2.4E+08	3.1E+08	3.85E+08	3.3E+08	3.56E+08				2.48E+08
6.04E+09	4.88E+09	7.09E+09	7E+09	7.91E+09	5.6E+09	5.95E+09	8.68E+09	7.25E+09
55085000	26534000	18027000	21664000	22429000			23335000	71073000
3E+09	2.51E+09	2.5E+09	2.62E+09	2.33E+09	2.11E+09	1.51E+09	3.67E+09	5.4E+09
2.88E+09	2.35E+09	1.73E+09	1.28E+09	1.71E+09	3.76E+09	3.88E+09		4.22E+09
1.9E+09	1.58E+09	3.21E+09	2.73E+09	3.29E+09	1.95E+09	1.85E+09	1.58E+09	2.32E+09
3.44E+09	2.88E+09	2.19E+09	2.11E+09	2.3E+09	5.3E+09	4.38E+09	6.08E+09	5.35E+09
			47635000		2.11E+09	2.34E+09	2.82E+08	1.27E+09
1.09E+09	1.01E+09	2.19E+09	2.04E+09	2.58E+09	3.05E+09	2.75E+09	2.69E+09	3.66E+09
6.37E+08	7.64E+08	1.02E+09	1.08E+09	1.14E+09	6.56E+08	7.1E+08	6.13E+08	7.01E+08
3.01E+08	2.36E+08	2.15E+08	1.73E+08	2.15E+08	2.17E+08	2.39E+08		2.54E+08
2.26E+08	2.16E+08	3.68E+08	3.26E+08	3.06E+08	2.19E+08	2.95E+08	1.75E+08	2.59E+08
	54856000	1.4E+08	75454000	78979000				1.48E+08
1.97E+09	2.59E+09	2.84E+09	2.6E+09	2.93E+09	2.59E+09	2.35E+09	1.94E+09	1.93E+09
1.12E+09	1.08E+09	1.01E+09	1.04E+09	1.04E+09	1.04E+09	1.17E+09	9.34E+08	7.68E+08
40023000	43215000	47892000	29735000	39654000			40484000	

1.49E+08								
1.58E+09	1.86E+09	3.56E+09	3.89E+09	4.07E+09	2.64E+09	2.25E+09	2.2E+09	2.08E+09
8.71E+08	1.08E+09	1.79E+09	1.94E+09	1.67E+09	2.39E+09	2.2E+09	2.34E+09	2.76E+09
9.87E+08	8.89E+08	2.22E+09	1.47E+09	2.06E+09	1E+09	1.05E+09	1.65E+09	1.16E+09
25541000	11634000	37751000	39337000	48987000	34511000	30347000	34576000	20572000
		1.03E+08	1.09E+08	89763000				48060000
2.65E+08		1.4E+08	1.35E+08	1.2E+08			2.63E+08	2.69E+08
1.02E+09	7.04E+08	1.29E+09	8E+08	1.16E+09	3E+08	3.03E+08	3.13E+08	8.58E+08
		1.75E+08	1.58E+08	1.72E+08				2.08E+08
2.54E+09	2.83E+09	2.95E+09	2.92E+09	2.93E+09	7.12E+09	6.25E+09	5.83E+09	6.41E+09
1.77E+08	1.63E+08	3.05E+08	2.8E+08	3.36E+08		1.73E+08		
5.62E+08	6.03E+08	9.31E+08	9.5E+08	1.07E+09	6.1E+08	6.65E+08	6.88E+08	8.27E+08
		62287000		59843000				
87851000	1.03E+08	83812000	96308000	95622000			99769000	
3.7E+09	4.09E+09	6.3E+09	6.34E+09	6.64E+09	4.01E+09	5.66E+09	5.49E+09	6.09E+09
4.68E+08	5.81E+08	4.19E+08	4.6E+08	4.47E+08	5.38E+08	5.31E+08	5.55E+08	4.33E+08
2.18E+08	2.01E+08	2.61E+08	2.82E+08	2.95E+08	2.41E+08	2.45E+08	1.8E+08	1.94E+08
1.23E+10	1.4E+10	9.38E+09	1.37E+10	1.16E+10	1.52E+10	1.93E+10	1.8E+10	1.25E+10
1.56E+09	1.55E+09	2.68E+09	2.58E+09	2.35E+09	1.76E+09	1.47E+09	1.19E+09	1.42E+09
4.42E+08	4.42E+08	5.86E+08	6.9E+08	6.67E+08	4.86E+08	4.77E+08	4.68E+08	6.17E+08
1.89E+08	1.5E+08	83226000	75490000				88070000	1.33E+08
		64760000	98968000	87342000	85214000		80527000	81709000
2.51E+08	2.21E+08	2.48E+08	3.85E+08	2.56E+08	4.61E+08	5.24E+08	5.32E+08	6.05E+08
2.17E+08	3.23E+08	2.22E+08	3.14E+08	2.25E+08		2.43E+08		
1.99E+08	2.46E+08	4.71E+08	4.32E+08	5E+08	4.1E+08	3.78E+08	5.05E+08	2.74E+08
78224000	78220000	83865000	84295000	81668000				
2.55E+08	2.43E+08	2.23E+08	2.82E+08	2.9E+08	4.12E+08	4.47E+08	3.43E+08	2.72E+08
6.2E+08	8.34E+08	9.86E+08	1.1E+09	9.73E+08	5.22E+08	7.25E+08	6.54E+08	5.82E+08
45557000	32011000	32664000	24639000	25431000	46334000			
1.51E+08	1.76E+08					1.31E+08		
1.59E+08	1.59E+08	1.29E+08	1.17E+08	1.32E+08	1.71E+08	2.13E+08	1.52E+08	1.4E+08
1.77E+08	2.93E+08	2.28E+08	3.28E+08	2.97E+08	3.2E+08	2.51E+08	5.6E+08	2.3E+08
1.2E+09	1.33E+09	1.2E+09	1.24E+09	1.26E+09	1.64E+09	1.45E+09	1.4E+09	1.24E+09
79674000	1.02E+08	61533000		57143000	1.12E+08	1.08E+08		89144000
3.88E+08	3.66E+08	1.34E+09	1.09E+09	1.48E+09	3.31E+08	3.45E+08	3.46E+08	6.27E+08
								44966000
4.98E+08	5.83E+08	4.54E+08	4.72E+08	4.77E+08	5.81E+08	6.9E+08	7.7E+08	5.86E+08
5.67E+08	6.54E+08	5.31E+08	3.14E+08	4.43E+08	4.41E+08		4.43E+08	3.71E+08
2.92E+08	5.79E+08	6.25E+08		2.77E+08	5.07E+08	5.14E+08	3.4E+08	4.37E+08
2.31E+09	2.31E+09	9.52E+08	8.17E+08	1.13E+09	1.66E+09	1.64E+09	1.58E+09	1.21E+09
		40520000	45804000	48756000				
1.81E+08		1.8E+08	2.13E+08	1.27E+08				
		1.01E+08	1.1E+08	1.13E+08				
1.42E+08	2.09E+08	1.22E+08	1.54E+08	1.32E+08	2.04E+08	2.4E+08	1.74E+08	1.95E+08
49597000								
1.92E+09	1.64E+09	1.98E+09	1.95E+09	2.27E+09	3.25E+09	2.68E+09	2.45E+09	2.92E+09
1.21E+09	1.44E+09	9.55E+08	9.79E+08	9.87E+08	1.19E+09	1.68E+09	1.24E+09	8.66E+08
				36489000		40648000		56728000

1.48E+08	2E+08							
2.88E+08	2.37E+08	4.12E+08	4.88E+08	3.33E+08	8.92E+08	8.43E+08	7.51E+08	8.19E+08
1.36E+08	1.43E+08	92416000	1.5E+08	1.12E+08				
63550000	1.28E+08		52110000		1.15E+08	1.3E+08		
	30022000	37230000		27550000				24774000
1.6E+08	1.43E+08	1.15E+08	1.43E+08	1.12E+08	1.74E+08	1.68E+08	1.98E+08	1.48E+08
1.49E+08	1.97E+08	1.95E+08	1.35E+08	1.74E+08	1.64E+08	1.69E+08	2.09E+08	1.89E+08
4.52E+08	5.52E+08	5.34E+08	6.4E+08	5.7E+08	5.5E+08	6.23E+08	4.94E+08	6.45E+08
6.02E+09	6.25E+09	5.41E+09	4.56E+09	4.44E+09	4.25E+09	4.2E+09	2.47E+09	3.83E+09
		44852000	56802000	35420000			35325000	
1.64E+08	1.46E+08	1.32E+08	1.5E+08	1.08E+08		2.13E+08	1.52E+08	1.33E+08
1.37E+08	2.47E+08	1.75E+08	2.13E+08	2.02E+08	2.93E+08	3.29E+08	2.4E+08	2.56E+08
1.18E+08	1.51E+08	1.93E+08	1.87E+08	2E+08	2.02E+08	1.35E+08	1.18E+08	73686000
2.14E+08	2.6E+08	2.49E+08	1.64E+08	2.55E+08	4E+08	4.54E+08	3.06E+08	2.56E+08
1.95E+09	2.07E+09	4.91E+09	4.72E+09	4.8E+09	2E+09	2.19E+09	2.35E+09	3.34E+09
9.31E+09	6.24E+09	9.32E+09	8.3E+09	7.53E+09	3.21E+09	5.39E+09	5.96E+09	1.19E+10
1.18E+08	1.08E+08	1.69E+08	1.2E+08	1.93E+08	1.27E+08	1.27E+08		1.94E+08
1.38E+09	1.6E+09	1.36E+09	1.13E+09	1.42E+09	2.01E+09	1.52E+09	1.2E+09	1.32E+09
3.12E+08	3.93E+08	2.11E+08	2.81E+08	2.51E+08	3.74E+08	3E+08	3E+08	2.65E+08
			1.52E+08					
6.16E+08	6.96E+08		2.74E+08	2.22E+08	5.99E+08	6.73E+08	4.66E+08	2.95E+08
35441000	57842000	23295000	25280000	18201000	46190000	26556000		11679000
	41658000				65844000	66092000		
4.57E+08	6.85E+08	3.53E+08	3.59E+08	3.57E+08	7.55E+08	6.12E+08	6.81E+08	4.79E+08
61338000	99658000	60816000	78887000	73854000			98020000	78225000
2.15E+09	1.09E+09	1.41E+09	1.54E+09	1.23E+09	1.78E+09	1.67E+09	1.1E+09	2.03E+09
6.32E+08	5.65E+08	5.05E+08	6.1E+08	5.68E+08	5.68E+08	6.5E+08	5.58E+08	6.05E+08
88188000	1.24E+08	86655000	1.09E+08	91096000		1.14E+08	92211000	
1.12E+09	1.89E+09	1.46E+09	8.39E+08	8.08E+08	5.79E+08	7.27E+08	2.93E+08	2.75E+08
2.36E+08	2.41E+08	1.35E+08	1.37E+08	1.37E+08	2.36E+08	2.86E+08	1.72E+08	1.55E+08
3.82E+08	4.63E+08	6.21E+08	6.01E+08	6.21E+08	3.56E+08	4.12E+08	3.33E+08	3.31E+08
1.71E+08	1.67E+08	2.08E+08	1.54E+08	1.69E+08	1.35E+08	1.85E+08	1.13E+08	1.78E+08
1.9E+08	2.34E+08	2.11E+08	1.62E+08	1.66E+08	2.53E+08	2.91E+08	2.82E+08	2.61E+08
5.26E+08	5.62E+08	4.55E+08	3.34E+08	2.08E+08				5.44E+08
			39732000	51554000				
45934000	51038000	54090000	49969000	60453000	54246000	60717000	50008000	41391000
7.73E+08	7.79E+08	5.95E+08	5.39E+08	7.32E+08	1.17E+09	9.34E+08	1.31E+09	1.22E+09
7.72E+08	7.38E+08	1.16E+08	1.62E+08	1.41E+08	9.06E+08	5.66E+08	5.81E+08	5.45E+08
	53147000	41522000		39103000				51433000
80864000	64459000							
2.66E+08	2.63E+08	2.1E+08	2.92E+08	1.76E+08	2.91E+08			2.77E+08
1.04E+09	1.21E+09	1.11E+09	1.05E+09	1.19E+09	1.1E+09	1.09E+09	1.04E+09	1.01E+09
4.14E+08	4.55E+08	4.61E+08	4.74E+08	5.1E+08	5.75E+08	5.77E+08	3.98E+08	5.43E+08
9.1E+08	9.82E+08	1.2E+09	1.29E+09	1.42E+09	8.68E+08	8.93E+08	7.16E+08	9.03E+08
5.72E+08	6.81E+08	3.33E+08	3.09E+08	3.71E+08	5.04E+08	4.85E+08	5.33E+08	5.81E+08
1.72E+08	1.69E+08	2.26E+08	2.52E+08	2.65E+08	1.46E+08	1.38E+08	1.25E+08	1.62E+08
3.5E+08	3.5E+08	1.62E+08	1.4E+08	1.61E+08	5.31E+08	3.97E+08	3.81E+08	3.26E+08
1.06E+10	8.14E+09	5E+09	5.95E+09	5.14E+09	6.16E+09	7.07E+09	6.46E+09	9.1E+09
5.53E+08	6.46E+08	1.96E+09	1.71E+09	1.82E+09	2.52E+09	1.99E+09	2.61E+09	2.65E+09

61456000	24162000							44718000
1.06E+08		1.04E+08	89822000	1.02E+08	1.4E+08	1.28E+08	1.16E+08	1.12E+08
6.95E+09	8.11E+09	9.15E+09	6.8E+09	3.88E+09	1.09E+09	4.13E+08	3.79E+09	4.94E+09
1.62E+08	2.97E+08	3.74E+08	5.32E+08	4.56E+08	3.5E+08	1.99E+08	1.51E+08	2.33E+08
					65112000			48920000
1.94E+08	1.73E+08	2.99E+08	2.63E+08	3.51E+08	1.56E+08	1.5E+08	1.51E+08	1.42E+08
	10836000	1.94E+08	1.6E+08	3.68E+08	59994000	17759000	33137000	2.33E+08
3.51E+08	2.27E+08	4.32E+08	4.3E+08	4.22E+08	1.71E+08	94665000	2.03E+08	3.51E+08
4.38E+08	8.06E+08	4.94E+08	6.88E+08	6.26E+08	7.34E+08	7.2E+08	8.63E+08	3.36E+08
8.36E+08	8.36E+08	4.13E+08	4.39E+08	4.13E+08	9.42E+08	9.76E+08	1.08E+09	8.17E+08
70130000	67176000			41963000	57559000	81185000		57458000
3.4E+09	4.82E+09	5.56E+09	8.64E+09	5.61E+09	3.26E+09	2.75E+09	3.88E+09	2.51E+09
8.97E+08	9.28E+08	1.23E+09	1.15E+09	1.18E+09	1.16E+09	1.13E+09	8.86E+08	9.35E+08
6.46E+08	4.31E+08	3.1E+08	3.77E+08	3.28E+08	2.24E+08	3.07E+08	2.3E+08	5.24E+08
3.35E+08	3.77E+08	2.59E+08	2.4E+08	2.33E+08	8.47E+08	7.97E+08	1.01E+09	7.08E+08
4.94E+08	4.67E+08	3.26E+08	3.5E+08	3.57E+08	5.34E+08	5.02E+08	4.16E+08	3.65E+08
71686000		78704000	1.14E+08	1.08E+08	52148000	82636000	67603000	51853000
79755000	43204000	1.6E+08	1.68E+08	2.52E+08		74924000		1.91E+08
1.28E+08	1.74E+08	1.25E+08	1.45E+08	1.3E+08	2.04E+08	1.51E+08	2.07E+08	1.74E+08
6.1E+08	9.49E+08	7.16E+08	8.17E+08	6.65E+08	2.43E+09	1.8E+09	2.57E+09	2.06E+09
1.74E+08	1.84E+08	1.42E+08	1.46E+08	1.44E+08	2.93E+08	2.58E+08	2.44E+08	2.48E+08
3.27E+08	3.25E+08	2.81E+08	1.94E+08	1.93E+08	2.67E+08	2.8E+08	3.53E+08	4.61E+08
4.24E+08	5.01E+08	3.93E+08	4.04E+08	4.15E+08	6E+08	6.13E+08	6.1E+08	5.39E+08
1.53E+08	1.59E+08	1.64E+08	1.9E+08	1.54E+08	2.68E+08	2.25E+08	3.32E+08	2.42E+08
				50578000				
		2.51E+08	2.38E+08	2.17E+08	4.37E+08	4.33E+08	4.76E+08	3.6E+08
	74656000	75327000		1.03E+08				
1.22E+08	1.03E+08	12998000			1.83E+08	1.12E+08	1.13E+08	85856000
	74308000					53870000		46323000
		2.8E+08	2.94E+08	3.29E+08	47954000			92552000
		15471000						
8.52E+08	4.58E+08	2.83E+08	2.03E+08	1.94E+08	3.9E+08	4.01E+08	3.43E+08	6.82E+08
		36228000		42739000				
7.94E+08	6.75E+08	7.37E+08	7.52E+08	7.33E+08	6.04E+08	8.74E+08	5.84E+08	5.48E+08
2.82E+08	2.66E+08	2.46E+08	1.95E+08	2.36E+08	2.64E+08	2.62E+08	2.16E+08	2.34E+08
7.81E+08	8.78E+08	5.6E+08	6.99E+08	6.22E+08	9.43E+08	8.73E+08	6.81E+08	4.25E+08
	31120000							
48506000				32400000				
4.5E+08	4.85E+08	5.57E+08	4.7E+08	5.07E+08	5.14E+08	5.46E+08	4.98E+08	4.82E+08
1.04E+08	1.01E+08				1.16E+08	1.15E+08	1.09E+08	74971000
2.58E+09	3.12E+09	2.36E+09	2.3E+09	2.3E+09	3.3E+09	3.18E+09	2.28E+09	2.3E+09
1.87E+08	1.99E+08		1.21E+08	96662000				
2.3E+08	2.64E+08	1.34E+08	1.55E+08	1.79E+08	4.14E+08	3.47E+08	2.93E+08	3.37E+08
				20990000				
	40146000	33619000		30525000				69823000
							94639000	
1.42E+09	1.45E+09	1.82E+09	1.87E+09	1.83E+09	2E+09	1.82E+09	1.74E+09	1.9E+09
1.88E+09	1.62E+09	2.7E+09	2.39E+09	2.73E+09	2.19E+09	2.25E+09	1.88E+09	2.26E+09

4.79E+09	4.26E+09	1.14E+10	1.05E+10	1.11E+10	3.62E+09	2.99E+09	2.34E+09	2.87E+09
1.33E+08	1.32E+08	1.67E+08	1.49E+08	1.52E+08	2.31E+08	1.56E+08	1.03E+08	1.01E+08
26446000		60376000	48899000	59241000				
6.03E+08	6.42E+08	7.14E+08	7.25E+08	6.25E+08	8.36E+08	8.35E+08	8.62E+08	7.63E+08
1.47E+09	2.02E+09	1.61E+09	1.52E+09	1.71E+09	1.76E+09	2.2E+09	1.4E+09	1.72E+09
66659000	56823000	62297000	76121000	76356000	68536000	49532000		
80681000	52145000	1.06E+08	79687000	73048000				1.2E+08
2.3E+08	3.63E+08	3.73E+08	2.92E+08	3.13E+08	4.36E+08	3.38E+08	3.34E+08	2.51E+08
1.09E+09	1.12E+09	1E+09	9.88E+08	8.63E+08	1.9E+09	1.92E+09	2.02E+09	1.49E+09
1.36E+08	1.48E+08	1.19E+08	1.11E+08	1.28E+08	2.53E+08	2.12E+08	1.54E+08	1.51E+08
1.06E+08	1.15E+08	1.2E+08	95654000	1.02E+08	1.26E+08	1.92E+08	1.71E+08	2.12E+08
6.79E+09	6.64E+09	5.15E+09	5.11E+09	5.33E+09	6.14E+09	6.27E+09	4.48E+09	6.53E+09
2.93E+09	2.91E+09	3.06E+09	2.16E+09	3.43E+09	3.96E+09	3.49E+09	4.38E+09	4.25E+09
		56379000	44789000	63233000	55360000			46961000
2.78E+08	3.82E+08	3.13E+08	2.48E+08	2.38E+08	5.78E+08	4.4E+08	4.8E+08	2.81E+08
2.94E+09	2.15E+09	2.02E+09	1.43E+09	2.44E+09	1.55E+09	1.64E+09	1.55E+09	2.62E+09
1.02E+10	9.4E+09	1.18E+10	1.02E+10	1.08E+10	1.04E+10	1.07E+10	8.22E+09	1.07E+10
5.35E+09	5.73E+09	6.46E+09	6.79E+09	6.68E+09	7.85E+09	7.75E+09	6.72E+09	8.25E+09
1E+09	1.17E+09	1.69E+09	1.56E+09	1.56E+09	1.5E+09	1.36E+09	1.21E+09	1.19E+09
3.84E+09	4.63E+09	4.65E+09	5.45E+09	4.53E+09	9.17E+09	8.27E+09	7.65E+09	9.22E+09
43968000	52716000	72344000	80545000	74212000	69751000	63993000		53084000
1.71E+08	1.72E+08	86766000		50156000		1.34E+08	1.62E+08	1.07E+08
93356000	1.07E+08	78509000	68017000	89364000		1.26E+08	1.09E+08	
			23093000	25019000				
1.2E+08	1.49E+08	2.1E+08	2.88E+08	2.43E+08	1.71E+08	1.44E+08	1.02E+08	1.22E+08
1.66E+08	1.19E+08	3.76E+08	3.33E+08	3.51E+08	94299000	99280000	97749000	1.06E+08
2.04E+08	1.44E+08	1.65E+08	1.3E+08	1.79E+08				1.7E+08
1.38E+08		95431000		1.28E+08			1.88E+08	
2.9E+08	2.96E+08	2.11E+08	2.16E+08	2.04E+08	3.23E+08	3.58E+08	3.89E+08	2.18E+08
1.43E+08	1.87E+08	1.96E+08	1.68E+08	1.76E+08	2.28E+08	1.49E+08	1.62E+08	1.48E+08
3.12E+09	2.13E+09	4.77E+09	5.01E+09	4.72E+09	2.54E+09	2.95E+09	2.06E+09	3.19E+09
1E+09	8.94E+08	2.1E+09	1.81E+09	2.12E+09	6.7E+08	6.96E+08	6.72E+08	9.31E+08
4.31E+08	4.4E+08	3.5E+08	3.11E+08	3.36E+08	4.48E+08	5.27E+08	4.32E+08	3.82E+08
2.66E+08	3.12E+08	6.19E+08	6.41E+08	5.5E+08	4.39E+08	2.84E+08	2.72E+08	3.64E+08
3.18E+08	3.42E+08	3.11E+08	3.45E+08	3.29E+08	3.73E+08	3.76E+08	3.88E+08	3.1E+08
4.21E+08	3.61E+08	2.37E+08	4.44E+08	2.64E+08	3.86E+08	3.66E+08	3.12E+08	3.17E+08
		47143000	51224000	37080000				51011000
2.8E+08	3.3E+08	2.35E+08	2.63E+08	2.83E+08	3.31E+08	3.18E+08	3.39E+08	3.09E+08
	1.1E+09				2.13E+09	1.81E+09		9.08E+08

1.27E+08	1.09E+08	47295000	85273000	65606000	62685000	1.16E+08		1.45E+08
2.26E+08	3.49E+08	2.77E+08	3.05E+08	3.43E+08	2.84E+08	2.91E+08	2.14E+08	1.87E+08
1.55E+08	1.31E+08	1.6E+08	2.06E+08	1.75E+08	1.31E+08	1.11E+08	1.06E+08	1.25E+08
1.3E+08	1.68E+08	87960000	1.23E+08	1.1E+08	1.99E+08	2.34E+08	2.61E+08	1.65E+08
		36185000						
51890000	71311000	62006000	67786000	66331000	88758000	87753000	64060000	84695000
34149000			61809000	56909000				
4.38E+08	4.13E+08	4.36E+08	3.54E+08	3.36E+08	3.67E+08	5.78E+08	3.83E+08	4.38E+08
		88924000	3.13E+08	1.61E+08				
2.45E+08	2.87E+08	95889000	95749000	72924000	1.24E+09	1.37E+09	1.83E+09	2.23E+09
		44082000	69924000	54897000	67526000			41255000
67142000			1.24E+08			62611000		81324000
6.74E+08	8.03E+08	5.05E+08	5.16E+08	5.29E+08	7.94E+08	7.13E+08	6.7E+08	6.67E+08
3.47E+08	4.72E+08	6.57E+08	7.22E+08	6.69E+08				
2.82E+08	2.75E+08	2.28E+08	2.66E+08	2.79E+08	2.8E+08	2.64E+08	2.47E+08	1.78E+08
6.93E+08	7.23E+08	5.57E+08	6.77E+08	6.1E+08	7.78E+08	8.86E+08	5.58E+08	4.18E+08
					6.82E+08	9.11E+08		
28323000	42420000	1.98E+08	1.63E+08	1.45E+08	1.19E+08	49959000		1.17E+08
4.12E+08	3.53E+08	1.72E+09	1.82E+09	1.75E+09	2.04E+09	1.43E+09	2.02E+09	8.25E+09
6.58E+09	6.2E+09	9.21E+09	8.48E+09	9.48E+09	8.41E+09	8.1E+09	7.9E+09	7.98E+09
2.1E+09	1.84E+09	2.9E+09	2.82E+09	3.39E+09	2.2E+09	1.71E+09	1.72E+09	2.38E+09
3.62E+09	2.6E+09	3.64E+09	3.07E+09	3.82E+09	2E+09	1.89E+09	1.89E+09	5.04E+09
7.56E+08	4.04E+08	3.13E+08	2.4E+08	2.98E+08	3.84E+08	4.04E+08	5.38E+08	7.61E+08
5.89E+09	3.83E+09		2.09E+09		5.65E+09	5.29E+09	5.83E+09	5.95E+09
35576000		12527000	15328000				20999000	43321000
				5772900				
1.09E+09	1.05E+09	1.2E+09	8.35E+08	1.46E+09	7.75E+08	6.82E+08	1.51E+09	1.01E+09
4.81E+09	4.71E+09	5.71E+09	4.23E+09	5.95E+09	5.71E+09	6.71E+09	7E+09	6.35E+09
2.95E+08	2.68E+08							2.55E+08
2.94E+09	3.11E+09	4.32E+09	4.56E+09	4.49E+09	3.85E+09	3.51E+09	2.1E+09	3.48E+09
7.58E+09	4.82E+09	1.64E+09	1.2E+09	1.83E+09	4.74E+09	4.93E+09	4.88E+09	3.21E+09
1E+09	1.2E+09	2.03E+09	2.75E+09	2.06E+09	1.37E+09	8.13E+08	1.33E+09	1.5E+09
3.49E+09	3.26E+09	4.06E+09	3.17E+09	4.07E+09	2.93E+09	3.46E+09	3.86E+09	3.51E+09
1.84E+09	9.39E+08	7.78E+08	1.12E+09	9.1E+08		1.12E+09		9.53E+08
1.45E+09	1.69E+09	4.62E+09	4.75E+09	4.47E+09	3.21E+09	2.3E+09	2.33E+09	3.74E+09
4.66E+09	3.9E+09	5.92E+09	4.96E+09	6.03E+09	8.05E+09	8.24E+09	9.47E+09	8.04E+09
2.54E+08	3.22E+08	2.38E+08	3.2E+08	2.87E+08	2.63E+08	2.73E+08		1.94E+08
92583000	93884000	2.28E+08	2.5E+08	2.71E+08	75475000	83369000		1.27E+08
46593000	49859000	51796000	43615000	48737000	75591000	60538000	50360000	52599000
8.65E+08	9.93E+08	9.49E+08	1.15E+09	9.3E+08	8.42E+08	1.01E+09	1.38E+09	8.6E+08
2.43E+08	2.41E+08	2.1E+08	2.41E+08	2.32E+08		1.86E+08	1.91E+08	2.08E+08
	41222000	65091000		52946000				50085000
99671000	1.03E+08	1.84E+08	1.77E+08	2.38E+08				1.27E+08
4.96E+08	5.54E+08	7.71E+08	8.5E+08	9.53E+08	7.01E+08	6.83E+08	6.99E+08	5.81E+08
6.93E+08	7.9E+08	2.46E+09	2.39E+09	2.71E+09	8.53E+08	8.42E+08	5.82E+08	6.59E+08
93509000	96134000	1.93E+08	2.01E+08	1.71E+08	1.84E+08	1.56E+08	1.2E+08	80879000
	1.86E+09	1.65E+09	1.81E+09	1.42E+09	3.07E+09	2.3E+09	2.83E+09	2.68E+09

74838000	92933000	96665000	91971000	1.12E+08	92678000	1.13E+08	1.14E+08	86666000
1.28E+08	1.22E+08	1.38E+08	1.13E+08	1.5E+08	1E+08			83828000
5.41E+09	5.21E+09	6.3E+09	3.47E+09	5.61E+09	7.33E+09	7.43E+09	9.49E+09	8.76E+09
4.15E+08	4.83E+08	3.41E+08	1.11E+08	4.56E+08	4.07E+08	4.62E+08	3.28E+08	5.73E+08
7.77E+08	9.12E+08	7.38E+08	8.53E+08	7.39E+08	9.54E+08	8.36E+08	1.07E+09	6.56E+08
9.1E+09	7.67E+09	1.24E+10	1.34E+10	1.23E+10	9.64E+09	8.8E+09	8.4E+09	9E+09
5.59E+08	7.23E+08	1.69E+09	1.8E+09	1.89E+09	9.3E+08	1.12E+09	5.6E+08	1.33E+09
2.05E+09	1.93E+09	3.54E+09	3.24E+09	3.83E+09	2.3E+09	2.19E+09	2.53E+09	2.05E+09
		42747000	41167000	39715000				17650000
7.69E+08	7.67E+08	5.76E+08	6.65E+08	6.05E+08	7.44E+08	8.22E+08	7.96E+08	7.57E+08
1.13E+09	1.51E+09	1.96E+09	1.8E+09	1.76E+09	1.12E+09	1.13E+09	1.96E+09	2.04E+09
4.64E+09	2.53E+09	3.42E+09	3.26E+09	2.88E+09	2.28E+09	2.29E+09	2.98E+09	5.72E+09
8.1E+08	9.4E+08	3.61E+09	3.84E+09	3.6E+09	6.76E+09	7.11E+09	6.16E+09	1.39E+10
2.34E+09	1.51E+09	2.2E+09	2.66E+09	2.26E+09	2.22E+09	2.26E+09	2.23E+09	3.13E+09
2.4E+08	2.38E+08	3.42E+08	1.51E+08	4.11E+08			2.92E+08	2.95E+08
1.57E+08	1.67E+08	2.86E+08	3.44E+08	3.23E+08	2.05E+08	2.01E+08	2.38E+08	2.21E+08
6.02E+08	8.59E+08	5.57E+08	5.39E+08	7.62E+08	6.54E+08	5.15E+08	6.73E+08	4.87E+08
2.44E+08	2.84E+08	3.2E+08	2.81E+08	3.42E+08	3.24E+08	2.87E+08	2.1E+08	2.25E+08
		1.89E+08	2.22E+08	2.43E+08	2.1E+08	2.59E+08	2.78E+08	3.59E+08
2.9E+09	3.13E+09	2.67E+09	2.29E+09	2.53E+09	2.27E+09	2.78E+09	2.51E+09	2.07E+09
2.62E+08	3.15E+08	1.83E+08	1.92E+08	2.04E+08	2.82E+08	2.98E+08	2.75E+08	2.01E+08
2.94E+08	3.38E+08	1.3E+08	1.36E+08	1.49E+08	2.56E+08	2.72E+08	2.38E+08	1.51E+08
3.11E+08	3.58E+08	3.11E+08	3.23E+08	3.04E+08	3.98E+08	3.86E+08	4.26E+08	4.34E+08
3.76E+08	3.76E+08	5.71E+08	6.41E+08	6.53E+08	4.08E+08	2.87E+08	2.15E+08	4.22E+08
5.78E+08	4.81E+08	8.69E+08	8.93E+08	1E+09	7.45E+08	6.86E+08	1.12E+09	7.11E+08
					49898000			
6.34E+08	6.65E+08				8.87E+08	1E+09	1.53E+09	
1.35E+08	1.42E+08	2.03E+08	2.14E+08	1.83E+08	1.81E+08	3.37E+08	1.88E+08	1.22E+08
2.15E+08	2.18E+08	1.41E+08	1.22E+08		1.53E+08	1.56E+08	1.35E+08	
3.83E+09	3.14E+09	3.61E+09	2.84E+09	3.32E+09	1.66E+09	1.41E+09	1.87E+09	2.09E+09
2.9E+09	2.01E+09	2.22E+09	1.94E+09	2.03E+09	1.28E+09	1.37E+09	1.21E+09	2.05E+09
3.15E+08	3.25E+08	1.53E+08	1.75E+08	1.5E+08	2.84E+08	3.14E+08	3.16E+08	2.21E+08
1.6E+08	1.61E+08	39643000	40237000	39498000		1.99E+08		1.52E+08
1.25E+08	1.47E+08	1.54E+08	1.72E+08	1.67E+08	2.52E+08	1.85E+08	3.26E+08	2.38E+08
3.88E+08	3.93E+08	3.6E+08	3.49E+08	3.9E+08	4.44E+08	3.5E+08	3.22E+08	3.46E+08
		35452000	59962000					
5.33E+08	6.49E+08	3.28E+08	3.11E+08	3.55E+08	5.73E+08	5.87E+08	4.9E+08	4.31E+08
1.06E+08	1.47E+08	1.67E+08	1.65E+08	1.4E+08			3.22E+08	3.98E+08
1.6E+08	2.08E+08	2.09E+08	2.01E+08	2.2E+08				1.43E+08
		25957000						23598000
		28831000		27740000				
8.81E+08	9.44E+08	1.02E+09	9.2E+08	9.78E+08	1.13E+09	1.01E+09	1.27E+09	9.08E+08
80616000	53989000	69773000	63773000	53624000				57778000
1.34E+08	82522000	75339000	99500000	95066000				83923000
3.47E+09	3.41E+09	4.96E+09	5.15E+09	5.5E+09	2.79E+09	2.27E+09	3.1E+09	5.38E+09
93613000	1.04E+08	98511000	1.18E+08	84737000	1.25E+08	1.4E+08	1.34E+08	1.25E+08

1.25E+08	1.1E+08	1.09E+08	1.19E+08	1.26E+08	1.37E+08	1.36E+08	1.41E+08	1.04E+08
		1.38E+08	80128000	1.51E+08				91422000
		3.08E+08	1.69E+08	1.73E+08				61862000
4.38E+08	5.05E+08	3.03E+08	3.34E+08	2.97E+08	5.25E+08	4.6E+08	4E+08	4.03E+08
1.5E+08	1.58E+08	2.29E+08	2.02E+08	3.21E+08		1.53E+08		1.63E+08
1.44E+08	1.52E+08	1.66E+08	1.86E+08	1.75E+08	2.39E+08	2.44E+08	2.42E+08	2.55E+08
7.45E+08	5.75E+08	7.75E+08	7.84E+08	6.39E+08		2.01E+09	1.9E+09	2E+09
5.03E+08	5.49E+08	6.34E+08	6.57E+08	5.55E+08	6.27E+08	6.14E+08	6.59E+08	4.87E+08
22742000	31170000				84777000	61227000	67679000	73888000
5.69E+08	5.59E+08	3.84E+08	3.37E+08	3.75E+08	6.96E+08	7.01E+08	5.39E+08	6.75E+08
6.73E+08	6.71E+08	1.08E+09	9.78E+08	1.05E+09	9.19E+08	7.64E+08	8.17E+08	7.48E+08
	6.58E+08	3.27E+08	3.03E+08	3.26E+08				
6.22E+08	6.63E+08	7.28E+08	6.41E+08	7.12E+08	5.87E+08	6.24E+08	4.49E+08	4.62E+08
		42952000						
	2.5E+08	2.1E+08	2.43E+08	2.17E+08	1.91E+08	1.64E+08	2.03E+08	
90506000			1.59E+08					32280000
2.98E+08	2.23E+08	2.19E+08	1.52E+08	3.68E+08	2.1E+08	1.8E+08	2.04E+08	2.27E+08
4.43E+08	4.82E+08	4.82E+08	3.56E+08	4.33E+08	3.87E+08	5.06E+08	4.97E+08	2.73E+08
83868000	85123000			69497000				
75904000	78443000	66705000	69443000	80357000	83424000	69609000	81800000	47948000
1.46E+08	1.69E+08	1.09E+08	90431000	1.49E+08	99142000	1.24E+08	79670000	1.16E+08
	2.34E+08				2.52E+08			
4.96E+08	4.97E+08	4.77E+08	3.94E+08	5.17E+08	4.96E+08	5.78E+08	6.01E+08	5.62E+08
79552000	1.07E+08		88937000	1.17E+08	78215000	77016000		77246000
8.58E+08	9.74E+08	1.65E+09	2.08E+09	2.24E+09	9.39E+08	9.17E+08	7.01E+08	8.33E+08
1.89E+08	2.28E+08	3.01E+08	2.3E+08	3.45E+08	2.94E+08	3.16E+08		1.37E+08
5.83E+08	7.18E+08	5.61E+08	6.21E+08	5.03E+08	4.99E+08	4.26E+08	5.48E+08	5.35E+08
		54223000	58866000					
2.37E+09	2.25E+09	3.78E+09	2.64E+09	4.02E+09	1.88E+09	1.77E+09	1.12E+09	1.58E+09
78664000	81402000	49065000				75745000		
		55068000	37131000	36213000		48668000		52473000
73135000	94872000	56170000	61221000	74325000	73345000	88764000	82201000	77861000
			28062000					
2E+08	1.61E+08	3.83E+08	2.69E+08	4.07E+08		88331000	1.3E+08	1.56E+08
		5.14E+08	6.34E+08	6.21E+08	1.11E+08	56146000		1.88E+08
		25914000	39765000	26168000				
		3.09E+08		3.79E+08	1.42E+08		1.04E+08	
		41748000	30693000	46927000				
8.82E+08	7.84E+08	5.55E+08	3.59E+08	5.68E+08	4.06E+08	4.58E+08	3.8E+08	4.36E+08
2.47E+08	1.77E+08	3.1E+08	3.5E+08	3.28E+08	3.64E+08	3.31E+08	3.02E+08	3.02E+08
4.36E+08	1.71E+08							2.35E+08
1.23E+08	1.25E+08	1.5E+08	3.44E+08	1.42E+08				
	74421000	67008000	40856000	17915000				

68988000	60529000	77758000	78304000	1E+08	48692000	75992000	45035000	46068000
		2.03E+08	1.45E+08	1.41E+08	1.84E+08	1.67E+08		
1.25E+08	1.6E+08	2.59E+08	3.54E+08	3.01E+08	1.78E+08	1.83E+08	1.42E+08	1.29E+08
2.05E+08	2.17E+08	1.4E+08	83807000	1.4E+08	1.63E+08	2.39E+08	1.58E+08	1.46E+08
80518000	1.03E+08				1.07E+08	88075000	1.25E+08	74561000
36004000	52448000	43585000	38465000	39492000	41997000	33776000		33951000
2.7E+08	2.84E+08	2.96E+08	3.39E+08	2.84E+08	2.41E+08	2.62E+08	1.9E+08	1.78E+08
4.83E+08	3.47E+08	6.27E+08	6.37E+08	5.97E+08	3.47E+08	3.52E+08	3.38E+08	5.15E+08
3.13E+08	3.94E+08	3.14E+08	3.64E+08	4.07E+08	3.22E+08	3.59E+08	4.02E+08	1.79E+08
	89764000	3.32E+08	1.77E+08	2.63E+08				
8.79E+08	1.52E+09	1.15E+09	1.03E+09	1E+09	1.28E+09	8.32E+08	6.69E+08	
6.69E+08	7.65E+08	3.26E+08	3.33E+08	3.13E+08	9.02E+08	8.3E+08	7.71E+08	5.19E+08
77927000	84916000	39315000	64686000	51158000	83823000	1.1E+08		78537000
3.84E+09	4.17E+09	3.98E+09	4.04E+09	4.11E+09	4.55E+09	5.04E+09	4.43E+09	3.33E+09
5.62E+08	5.64E+08	3.51E+08	3.88E+08	4.3E+08	6.97E+08	7.77E+08	7.13E+08	4.62E+08
8.9E+08	7.56E+08	8.44E+08	6.58E+08	7.31E+08	6.39E+08	6.68E+08	5.85E+08	7.13E+08
					94364000	1.43E+08	2.03E+08	1.31E+08
2.47E+08	2.61E+08	3.19E+08	3.25E+08	3.67E+08	3.37E+08	3.06E+08	3.34E+08	1.56E+08
89580000	92902000	1.04E+08	92143000	94919000	1.05E+08		69061000	78146000
1.73E+08	2.37E+08	1.89E+08	1.57E+08	1.75E+08	1.68E+08	1.81E+08	1.21E+08	1.03E+08
				34393000	26217000	30801000		
6.31E+08	5.37E+08	4.41E+08	4.2E+08	3.41E+08	4.87E+08	4.93E+08	4.49E+08	
2.54E+08	2.68E+08	1.84E+08	2.02E+08	1.9E+08	2.34E+08	2.27E+08	2.53E+08	1.84E+08
2.3E+09	2.64E+09	4.24E+09	4.45E+09	4.36E+09	3.14E+09	3.84E+09	1.45E+09	2.58E+09
4.99E+08	6.1E+08	5.2E+08	4.35E+08	3.92E+08	6.44E+08	4.86E+08	5.28E+08	3.38E+08
		72010000	84988000	74458000				50026000
3.36E+08	3.88E+08	6.21E+08	6.6E+08	7.01E+08	3.21E+08	3.23E+08	2.27E+08	2.87E+08
13488000		28143000	41367000	19058000				
1.28E+09	1.35E+09	9.21E+08	1.02E+09	8.53E+08	1.78E+09	1.9E+09	1.54E+09	1.44E+09
78346000	89539000	42792000	47944000	47892000	67547000	75344000	62360000	71266000
90156000	74601000	31464000		40401000				
		55279000	65859000	43825000		71304000		60835000
1.27E+08	1.47E+08	1.16E+08		1.37E+08	1.21E+08	1.71E+08	1.45E+08	1.1E+08
5.88E+08	1.05E+09	7.89E+09	6.92E+09	7.99E+09	1.5E+09	1.18E+09	9.82E+08	1.61E+09
		56745000	54298000	60929000				
1.94E+08	1.84E+08	2.82E+08	1.86E+08	2.61E+08	1.27E+08	92150000	1.05E+08	1.19E+08
1.96E+09	1.96E+09	1.56E+09	1.78E+09	1.33E+09	3E+09	2.98E+09	3.01E+09	2.28E+09
	87242000	1.01E+08		1.13E+08				
70231000	1.03E+08	69498000	86144000	69215000	96813000	1.28E+08	1.02E+08	94998000
		1.63E+08	1.37E+08	1.55E+08				
	10669000	19511000	25220000	10168000				
	83287000	1.5E+08	2.04E+08	1.45E+08	1.68E+08	1.1E+08		1.39E+08

1.51E+08	1.35E+08	2.1E+08	2.23E+08	2.16E+08	1.62E+08	1.5E+08	1.49E+08	1.1E+08
2.48E+09	2.53E+09	1.69E+09	1.79E+09	2E+09	3.05E+09	2.94E+09	2.49E+09	1.86E+09
7.16E+08	8.47E+08	8.91E+08	1.09E+09	1.08E+09	8.71E+08	9.93E+08	7.9E+08	6.74E+08
		70208000	72006000	59499000	74821000			54864000
1.84E+08		1.75E+08						
98020000	73624000	36673000		63180000				
1.41E+08			98010000		1.75E+08	1.82E+08	2.23E+08	1.49E+08
		77531000	71852000	84981000				
		51082000	68063000	57526000	52855000	56208000	60958000	52447000
83732000	1.09E+08	85489000		1.09E+08	89828000	1.25E+08		98892000
1.18E+09	1.36E+09	7.89E+08	1.02E+09	8.54E+08	1.33E+09	1.26E+09	1.16E+09	1.05E+09
		38352000	53727000	57223000				
	1.14E+08	1.33E+08	69651000	96757000	1.12E+08	1.1E+08		1.07E+08
58459000								79534000
91172000	98630000	1.64E+08	84649000	1.15E+08	57104000		1.09E+08	1.56E+08
74981000	1.01E+08	71829000	73176000	85032000	53901000	72169000		38602000
67900000	77762000	78824000	1.03E+08	94146000	1.02E+08	1.03E+08	1.17E+08	1.74E+08
		96170000	1.07E+08	1.08E+08				
46588000	46063000			57154000				
			1.11E+08	1.22E+08				
				12355000				
3.45E+08	3.98E+08	5.02E+08	4.15E+08	4.45E+08	2.64E+08	96000000	2.96E+08	3.08E+08
2.83E+08	2.35E+08	3.26E+08	2.56E+08	3.83E+08	2.44E+08	2.04E+08	1.88E+08	2.48E+08
27490000		28989000	28164000					
		2.45E+08	2.55E+08	2.66E+08	2.06E+08	2.09E+08	2.01E+08	
3.52E+08	3.1E+08	2.25E+08	1.31E+08	96633000	1.56E+08	1.23E+08	2.33E+08	1.41E+08
1.42E+09	1.59E+09	6.74E+08	1E+09	7.01E+08	2.94E+09	1.56E+09	1.5E+09	1.54E+09
5.11E+08	5.38E+08	3.02E+08	3.45E+08	3.78E+08	4.83E+08	4.56E+08	4.44E+08	2.9E+08
3.79E+08	4.79E+08	2.39E+08	2.59E+08	2.46E+08	3.59E+08	3.15E+08	2.83E+08	1.8E+08
1.71E+09	1.3E+09	7.96E+08	6.67E+08	6.97E+08	1.04E+09	8.03E+08	1.2E+09	1E+09
29399000	20126000	15110000	23750000	25151000	12564000	21113000	22392000	27597000
2.03E+10	1.37E+10	6.69E+09	9.04E+09	9.19E+09	2.4E+10	1.72E+10	1.63E+10	2.05E+10
87199000	84266000	1.38E+08		1.01E+08				
5.41E+08	7.28E+08	2.77E+09	3.01E+09	2.8E+09	8.52E+08	7.36E+08	7.61E+08	9.73E+08
		49600000	87186000					
1.45E+09	1.54E+09	2.05E+09	1.39E+09	1.74E+09	1.54E+09	1.68E+09	1.74E+09	1.39E+09
2.09E+09	1.88E+09	2.07E+09	3.33E+09	1.74E+09	3.62E+09	2.78E+09	3.11E+09	3.6E+09
34451000	32748000	57051000	52338000	52667000		48850000	28206000	
2.16E+09	2.31E+09	4.58E+09	4.94E+09	4.79E+09	3.04E+09	2.75E+09	2.47E+09	2.8E+09
56138000			53529000					32942000
52763000	40454000				50030000	50167000	47268000	39479000
4.07E+08	4.07E+08	5.3E+08	6.1E+08	5.13E+08	4.19E+08	3.36E+08	5.22E+08	2.77E+08
					49980000		44668000	
65084000	71028000	71498000	70964000	73544000	77382000	88386000	56384000	67554000

1.16E+08	1.4E+08	2.9E+08	2.45E+08	1.98E+08	1.69E+08	1.4E+08	2.26E+08	1.63E+08
7073400	4945700	4676200	12137000	11140000		10175000		9255900
1.99E+08	1.75E+08	1.4E+08	1.2E+08	1.34E+08	3.09E+08	2.2E+08	2.27E+08	1.52E+08
33977000	36532000	46226000	30373000		37507000			
1.23E+08	1.82E+08	2.13E+08	2.06E+08	2.56E+08	1.27E+08	1.34E+08	1.25E+08	1.09E+08
1.64E+08	2.02E+08	1.77E+08	1.86E+08	2.14E+08	2.58E+08	2.35E+08	3.06E+08	2.14E+08
76184000	89388000	92196000	77982000	87176000				59730000
		93659000		81293000				
93447000	76490000	1.07E+08	1.36E+08	1.17E+08	57869000		98067000	1.01E+08
28349000	39035000	61889000	77648000	68877000		41219000	55038000	42443000
		1.88E+08	1.68E+08	1.8E+08				
		91391000	76661000	92894000				
1.54E+08	1.4E+08	2.15E+08	1.56E+08	1.88E+08	1.95E+08	1.68E+08	1.94E+08	1.25E+08
8.08E+08	1.04E+09	7.02E+08	6.2E+08	8.02E+08	7.14E+08	7.53E+08	5.61E+08	4.45E+08
3.71E+08	4.41E+08	5.11E+08	4.57E+08	5.39E+08	4.74E+08	3.51E+08	2.62E+08	2.95E+08
2.67E+08	3.29E+08	1.46E+08	1.73E+08	1.71E+08	4.11E+08	4.09E+08	4.34E+08	4.17E+08
1.46E+08	1.54E+08	81921000	92971000	82565000		1.32E+08	74497000	1.06E+08
3.83E+09	4.18E+09	2.91E+09	2.7E+09	2.58E+09	4.15E+09	4.31E+09	2.71E+09	2.61E+09
6.59E+08	5.61E+08	6.78E+08	8.04E+08	7.51E+08	6.17E+08	6.37E+08	7.65E+08	4.93E+08
					2.81E+08		3.25E+08	
1.02E+09	8.74E+08	5.59E+08	5E+08	5.08E+08	1.91E+09	1.29E+09	2.32E+09	1.55E+09
73976000	76747000	56320000		55999000		71539000		76697000
49908000		53149000	46606000					
1.22E+09	1.29E+09	1.44E+09	1.51E+09	1.58E+09	1.32E+09	1.2E+09	9.9E+08	1.06E+09
3.05E+08	2.29E+08	4.75E+08	2.64E+08	3.79E+08	2.26E+08	3.78E+08	2.68E+08	2.15E+08
3.46E+08	4.49E+08	5.4E+08	5.97E+08	5.18E+08	3.72E+08	2.83E+08	4.09E+08	4.22E+08
4.79E+08	4.82E+08	2.1E+08		2.41E+08	2.56E+08	2.8E+08		2.23E+08
1.26E+08	1.31E+08	2.32E+08	1.98E+08	2.74E+08	1.74E+08	1.36E+08	1.42E+08	2.32E+08
4.69E+08	5.53E+08	4.73E+08	4.17E+08	4.18E+08	6.76E+08	7.12E+08	6.03E+08	5.01E+08
		1.64E+08	1.71E+08	1.54E+08			3.08E+08	
3.05E+08	3.24E+08	2.11E+08	2.44E+08	2.55E+08	3.88E+08	4.21E+08	4.57E+08	3.23E+08
1.47E+09	1.48E+09	9.3E+08	1.21E+09	1.04E+09	1.47E+09	1.53E+09	1.21E+09	1.18E+09
		58783000	56818000	62964000		39309000		
1.5E+09	1.58E+09	1.72E+09	1.78E+09	1.57E+09	1.62E+09	1.56E+09	1.58E+09	1.48E+09
				38862000	32276000			23963000
5E+09	4.98E+09	3.24E+09	2.87E+09	3.39E+09	3.56E+09	4.31E+09	2.89E+09	3.66E+09
64676000	82498000	45858000		39636000				

3.16E+08	3.19E+08	2.2E+08	2.61E+08	2.05E+08		76869000	1.93E+08	1.5E+08
1.02E+08	1.37E+08	1.45E+08	1.91E+08	1.48E+08	1.53E+08	1.59E+08	1.13E+08	1.57E+08
9.1E+08	1.11E+09	9.55E+08	8.6E+08	8.89E+08	8.94E+08	1.09E+09	7.49E+08	1E+09
2.56E+08	4.19E+08	4.38E+08		5.19E+08				1.97E+08
2.88E+08	3.25E+08	3.12E+08	2.61E+08	3.39E+08	3.87E+08			2.41E+08
93552000	1.13E+08	1.03E+08	1.27E+08	1.12E+08	90172000		2.71E+08	
2.53E+08	3.5E+08	2.29E+08	1.98E+08	2.4E+08	3.98E+08	4.03E+08	3.19E+08	3.87E+08
				52667000				58695000
83948000	88793000	63329000	41892000	47388000		46362000	71534000	65551000
8.96E+08	9.43E+08	5.02E+08	5.96E+08	4.72E+08	1.75E+09	1.43E+09	1.86E+09	1.3E+09
1.09E+09	1.3E+09	1.11E+09	9.39E+08	1.15E+09	1.01E+09	9.81E+08	9.21E+08	9.05E+08
19870000								
				29205000				
1.78E+10	2.13E+10	2.13E+10	1.17E+10	1.19E+10	1.41E+10	1.42E+10	7.06E+09	1.29E+10
3.85E+08	4.42E+08	4.78E+08	4.69E+08	5.25E+08	5.04E+08	7E+08	2.51E+08	4.7E+08
			23491000					
	98642000	84508000	92578000	91934000	92136000	1.09E+08		
		1.87E+08	1.98E+08					
41871000	62845000	35399000	52263000	57050000	65952000	67646000	70804000	58686000
2.09E+09	1.95E+09	1.06E+09	1.24E+09	1.16E+09	3.02E+09	3.06E+09	4.36E+09	2.49E+09
3.57E+08	3.72E+08	3.14E+08	3.34E+08	4.04E+08	4.23E+08	3.16E+08	3.43E+08	6.86E+08
1.4E+08	1.45E+08	1.17E+08	1.46E+08	93848000		1.23E+08	1.21E+08	1.03E+08
5.44E+08	7.27E+08	5.35E+08	6.7E+08	8.03E+08	6.35E+08	7.09E+08	7.15E+08	4.7E+08
73417000	53189000	95487000	58281000	62244000	67154000	56202000	1.04E+08	59870000
			52652000		36427000	34159000	61152000	47555000
		54077000	57407000	50275000				
1.24E+09	1.25E+09	1.51E+09	1.32E+09	1.54E+09	1.08E+09	1.25E+09	1.07E+09	9.45E+08
5.7E+09	7E+09	2.06E+09	2.25E+09	2.35E+09	8.18E+09	9.49E+09	9.54E+09	5.75E+09
1.6E+08	1.24E+08	1.71E+08	1.29E+08	1.29E+08		1.25E+08	1.22E+08	1.15E+08
		86337000	89466000	1.03E+08				1.61E+08
3.27E+08	3.75E+08	62059000	1.33E+08	63924000	4.08E+08	4.34E+08	5.7E+08	2.06E+08
3.3E+08	2.96E+08	2.78E+08	2.68E+08	3.38E+08	3.31E+08	3.88E+08	4.64E+08	3.83E+08
42935000	43644000	80464000	1.09E+08	1.19E+08	51572000	46334000		54413000
9.74E+08	9.62E+08	8.31E+08	4.78E+08	6.58E+08		8.48E+08	8.54E+08	8.33E+08
4.94E+08	6.2E+08	8.38E+08	4.54E+08	8.29E+08	5.03E+08	3.11E+08	3.96E+08	3.28E+08
1.02E+08	1.3E+08	1.76E+08	1.2E+08	1.6E+08	1.31E+08			1.01E+08
		80721000	96154000	88188000				49276000
		47383000						
73462000	1.04E+08	1.18E+08	1.27E+08	1.24E+08	1.83E+08	1.03E+08		1.44E+08

6.29E+08	4.27E+08	4.4E+08	4.33E+08	4.25E+08	3.28E+08	3.95E+08	5.67E+08	4.82E+08
		27335000	39977000	38218000				
1.08E+08	95516000	1.2E+08	88907000	1.11E+08	1.23E+08	1.01E+08	1.04E+08	1.17E+08
67014000	63244000	40407000	23836000	53947000	40148000			
52356000	69617000		48030000	56683000	55462000	72760000		
8.58E+08	6.96E+08	2.18E+08	1.6E+08	2.57E+08	6.64E+08	7.37E+08	5.08E+08	3.34E+08
2.07E+08	2.96E+08	2.97E+08	2.46E+08	2.68E+08	4.03E+08	4.44E+08	3.88E+08	2.35E+08
2.15E+08	1.47E+08	3.58E+08	1.34E+08	3.96E+08	1.25E+08	1.16E+08	1.18E+08	1.22E+08
		85020000	1.19E+08	95919000				
1.36E+09	1.66E+09	9.52E+08	1.26E+09	1.16E+09	2.23E+09	2.19E+09	2.4E+09	1.66E+09
				25758000				
1.12E+09	5.75E+08						4E+08	7.21E+08
68665000	69987000		29520000	26312000			68199000	
7.15E+08	7.5E+08	7.08E+08	6.73E+08	8.15E+08	6.65E+08	6.39E+08	5.29E+08	6.94E+08
	2.82E+08	2.4E+08	2.16E+08	1.99E+08	4.38E+08	3.89E+08	3.36E+08	3.19E+08
3.3E+08	3.01E+08	2.16E+08	2.15E+08	2.25E+08	4.32E+08	4.17E+08	4.66E+08	3.9E+08
4.72E+08	5.31E+08	7.95E+08	7.89E+08	9.2E+08	4.74E+08	4.82E+08	2.89E+08	6.67E+08
3E+08	3.27E+08	8.97E+08	8.41E+08	1.02E+09	3.8E+08	3.17E+08	2.94E+08	4.43E+08
5.61E+08	6.94E+08	1.55E+09	1.52E+09	1.68E+09	9.24E+08	7.75E+08	5.64E+08	8.85E+08
8.12E+08	6.92E+08	1.3E+09	1.25E+09	1.06E+09	1.46E+09			1.05E+09
94455000	47947000	48658000	49042000	46368000				72133000
2.99E+08	3.04E+08	1.47E+08	1.77E+08	1.48E+08	4.55E+08	4.73E+08	4.14E+08	2.14E+08
84574000		41563000	1.07E+08		1.28E+08	1.47E+08	1.27E+08	66074000
6.69E+08	7.56E+08	4.3E+08	5.29E+08	4.59E+08	7.23E+08	8.33E+08	7.91E+08	7.03E+08
1.75E+08	2.16E+08	1.59E+08	2.53E+08	2.31E+08	2.69E+08	2.82E+08	2.31E+08	1.98E+08
1.14E+08	1.33E+08	90096000	94520000	1.05E+08	1.42E+08	1.56E+08	1.55E+08	1.07E+08
			2.18E+08	2.47E+08				
1.86E+08	2.13E+08	2.25E+08	2.84E+08	3.57E+08	1.69E+08	2.31E+08	1.77E+08	2.07E+08
1.94E+08	2.13E+08	1.08E+08	1.36E+08	1.09E+08	2.68E+08	3.01E+08	2.82E+08	2.3E+08
5.22E+09	5.49E+09	6.43E+09	4.53E+09	7.75E+09	3.08E+09	3.75E+09	2.78E+09	3.87E+09
5.39E+08								
48989000	71082000			38614000		45951000		
6.26E+08	7.53E+08	4.35E+08	2.29E+08		7.14E+08	8.44E+08	8.36E+08	5.95E+08
2.96E+08	3.24E+08	2.78E+08	3.35E+08	3.43E+08	3.46E+08	3.46E+08	3.31E+08	2.85E+08
		39670000		26280000				
1.75E+08	1.97E+08	2.26E+08	2.86E+08	2.41E+08	2.46E+08	2.37E+08	2.4E+08	3.2E+08
4.92E+08	5.33E+08	3.3E+08	3.58E+08	3.01E+08	6.2E+08	5.62E+08	5.17E+08	4.37E+08
3.65E+08	3.98E+08	3.35E+08	2.68E+08	3.44E+08	4.89E+08	4.53E+08	4.28E+08	3.2E+08
9.93E+08	9.62E+08	8.42E+08	9.52E+08	9.99E+08	1.36E+09	1.15E+09	9.82E+08	9.41E+08

2.64E+08	3.29E+08	1.42E+08	1.73E+08	1.54E+08	2.59E+08	2.13E+08	2.71E+08	1.56E+08
1.22E+09	1.35E+09	8.65E+08	8.77E+08	1.02E+09	1.44E+09	1.37E+09	1E+09	1.23E+09
2.18E+08		2.6E+08	2.29E+08	2E+08	2.17E+08	1.95E+08	1.76E+08	1.71E+08
5.6E+08	6.87E+08	1.15E+09	1.44E+09	1.24E+09	8.28E+08	6.45E+08	5.26E+08	6.26E+08
92557000	94573000	1.09E+08	74935000	80146000	1.24E+08	1.24E+08	1.26E+08	
3.77E+09	3.5E+09	2.77E+09	2.44E+09	2.35E+09	2.88E+09	3.32E+09	2.9E+09	3.64E+09
82510000	2.38E+08				1.96E+08	1.81E+08	1.4E+08	1.17E+08
3.19E+08	3.32E+08	1.41E+08	1.55E+08	1.46E+08	3.25E+08	2.96E+08	2.56E+08	1.31E+08
1.09E+08	1.71E+08	95871000	1.07E+08	1.08E+08	2.13E+08	2.2E+08	2.3E+08	2.01E+08
1.39E+08	1.87E+08	1.31E+08	75080000	1.24E+08	2.13E+08	2.1E+08	2.21E+08	1.92E+08
63718000	75168000	1.09E+08	67661000	86949000	52911000	75333000	42131000	54163000
1.49E+08		1.58E+08		2.03E+08	1.7E+08			
5.96E+08	6.39E+08	1.1E+09	8.4E+08	1.1E+09	5.33E+08	5.5E+08	4.67E+08	5.28E+08
61147000	62594000				1.21E+08			
1.19E+08	1.4E+08	1.41E+08	1.22E+08	1.14E+08	1.06E+08	1.04E+08		1.32E+08
60033000	55076000	73851000	59897000	72898000		83377000	73722000	60928000
1.33E+08	1.5E+08	96776000			1.28E+08		79292000	62806000
3.47E+08	3.59E+08	6.54E+08	4.2E+08	6.49E+08	4.41E+08	4.42E+08	4.75E+08	3.93E+08
2.69E+09	3.33E+09	5.58E+09	5.81E+09	5.26E+09	2.25E+09	2.69E+09	3.24E+09	3.65E+09
5.22E+08	6.7E+08	5.37E+08	5.19E+08	5.33E+08	4.15E+08	2.96E+08	5.29E+08	3.21E+08
1.52E+08	2.19E+08	2.58E+08	3.34E+08	3.03E+08	3.49E+08	2.75E+08	3.65E+08	3.22E+08
1.17E+08	1.52E+08	1.07E+08	1.08E+08	1.06E+08	2.51E+08	2.02E+08	2.23E+08	1.68E+08
1.04E+08	1.09E+08	1.31E+08	2.34E+08	1.67E+08	1.14E+08	1.08E+08	86719000	1.36E+08
3.55E+08	1.89E+08	4.34E+08	3.16E+08	3.32E+08	4.04E+08	4.47E+08	2.06E+08	4.51E+08
1.87E+09	1.38E+09	1.46E+09	1.72E+09	1.45E+09	1.19E+09	1.18E+09	1.39E+09	2.48E+09
		7.32E+08	6.63E+08	8.67E+08	1.17E+08			1.36E+08
1.3E+08	2.44E+08	3.23E+08	2.16E+08	2.45E+08	96555000	1.41E+08	74123000	1.4E+08
1.2E+08	1.73E+08	1.22E+08	1.43E+08	1.23E+08	2.09E+08	1.83E+08	1.48E+08	1.64E+08
75649000	81046000	59728000	68174000	58619000	1.04E+08	89009000	83262000	81781000
24813000	24605000	32810000	20582000	27886000				
49565000	26788000							
1.38E+09	1.37E+09	2.66E+09	2.39E+09	2.51E+09	1.62E+09	1.6E+09	1.42E+09	1.62E+09
	1.11E+08	96356000		62462000				85236000
5.15E+08	5.18E+08	8.43E+08	5.9E+08	7.75E+08	5.92E+08	6.57E+08	4.31E+08	4.46E+08
1.53E+09	1.45E+09	1.22E+09	9.34E+08	1.29E+09	1.98E+09	1.77E+09	1.88E+09	1.46E+09
4.39E+08	4.21E+08	3.97E+08	3.37E+08	4.51E+08	4.17E+08	4.82E+08	3.8E+08	3.34E+08
4.67E+08	3.63E+08	3.96E+08	3.71E+08	3.43E+08	1.19E+09	1.04E+09	1E+09	8.16E+08
1.74E+08	3.97E+08	1.7E+08	1.9E+08	2.64E+08	2.67E+08	1.35E+08	1.46E+08	
1.33E+08	1.64E+08	83557000	3.33E+08	2.94E+08	3.86E+08	4.63E+08	5E+08	6.26E+08
85940000	1.4E+08	60497000		38985000	75258000	1.18E+08	66542000	
32496000	45843000	46064000	42327000	50555000		52271000	34764000	
1.04E+08	86622000			60725000				69617000

			60802000					
		35737000	48660000	29822000				77952000
4.49E+08	5E+08	2.93E+08	1.95E+08	2.8E+08	2.99E+08	4.25E+08	3.55E+08	2.36E+08
		86246000	1.33E+08	1.02E+08	87842000	1.06E+08	97774000	88298000
35329000	44108000			29150000		40690000	34840000	36201000
						1.79E+08		2.32E+08
62637000	51868000	86691000	90277000	1.05E+08		59759000	45422000	69923000
5.99E+08	5.58E+08	1.15E+09	1.09E+09	1.11E+09	1.15E+09	1.02E+09	9.9E+08	2.09E+09
1.02E+08	1.51E+08				1.54E+08			1.23E+08
26407000	23141000	18776000	21541000	23757000	33224000	27938000		22924000
8.62E+08	1.32E+09	9.86E+08	1.84E+09	7.21E+08	1.07E+09	1.29E+09	1.43E+09	6.9E+08
5.8E+08	8.74E+08	5.72E+08	8.07E+08	7.49E+08	6.3E+08	7.38E+08	8.36E+08	7.29E+08
1.61E+08	1.18E+08	98414000	69824000	1.02E+08	84335000	94654000	72600000	63503000
3.46E+08	2.89E+08	1.52E+08	1.26E+08	1.67E+08	2.37E+08	2.6E+08	2.68E+08	2.26E+08
4.94E+08	6.23E+08	5.21E+08	5.39E+08	4.97E+08	5.16E+08	4.03E+08	3.32E+08	3.9E+08
	29099000					27179000		
								73200000
			87975000	70028000	1.02E+08	99237000		
61733000	87057000	41806000	34849000	41702000	67090000	65994000	42077000	34349000
1.47E+09	1.51E+09	1.22E+09	1.39E+09	1.37E+09	1.56E+09	1.62E+09	1.43E+09	1.38E+09
26039000	20723000	18383000		19139000				20990000
55273000	55318000	50381000	62167000	76944000	76645000	47416000	43375000	52013000
90936000	1.2E+08	55311000	53758000	66199000	1.25E+08	1.16E+08	94392000	46762000
36662000	27830000	24958000	36692000	24212000	59974000	39143000	66559000	40075000
2.02E+08	2.22E+08	3.13E+08	2.27E+08	2.79E+08	1.98E+08	1.65E+08	1.41E+08	1.8E+08
	61363000	1.15E+08	1.04E+08	1.1E+08				
		39870000	42108000	38431000	53984000	58880000	64310000	
3.66E+08	2.43E+08	2.57E+08	1.67E+08	1.65E+08	3.75E+08	4.63E+08	5.94E+08	4E+08
5.14E+08	7.8E+08	1.11E+09	1.47E+09	9.1E+08	7.66E+08	8.01E+08	6.7E+08	4E+08
				2.58E+09				5.51E+09
	37658000	1.08E+08		60555000				
2.23E+09	2.34E+09	1.06E+09	1.04E+09	1.13E+09	1.45E+09	1.82E+09	1.64E+09	1.11E+09
		33976000	58454000					
1.67E+08	1.62E+08	3.56E+08	3.43E+08	3.55E+08	2.05E+08	2.01E+08	1.85E+08	2.02E+08
	1.38E+08							
7.05E+08	8.26E+08	1.23E+09	1.21E+09	1.37E+09	5.71E+08	6.04E+08	6.76E+08	6.22E+08
1.61E+08	1.41E+08	1.79E+08	1.74E+08	1.71E+08	1.43E+08	1.54E+08	2E+08	2.36E+08
		1.23E+08	1.42E+08	1.58E+08		1.23E+08		
6.63E+08	5.34E+08	3.33E+08	2.56E+08	2.34E+08			4.97E+08	4.53E+08
8.64E+08	9.96E+08	1.13E+09	1.03E+09	1.02E+09	1.03E+09	1.11E+09	1.05E+09	8.41E+08

2.4E+08	1.6E+08		33353000		1.32E+08	1.75E+08		72780000
1.41E+08	2.37E+08	55650000	48825000	71592000	2.79E+08	2.75E+08	2.35E+08	1.61E+08
	49727000		63744000	48206000	73934000	63937000		68562000
1.57E+08	1.3E+08		77981000					
5.01E+08	6.58E+08	5.9E+08	3.61E+08	6.21E+08	5.35E+08	5.15E+08	5.37E+08	4.16E+08
1.31E+09	1.17E+09	2.41E+09	1.76E+09	2.92E+09	1.33E+09	1.39E+09	1.39E+09	2.37E+09
39900000	53475000							
	6.44E+08							
2.01E+08	2.32E+08	1.71E+08	1.83E+08	1.57E+08	2.66E+08	2.48E+08	2.22E+08	2.27E+08
1.17E+09	1.41E+09	7.25E+08	9.16E+08	7.64E+08	1.05E+09	1.21E+09	9.83E+08	6.18E+08
1.74E+08	2.41E+08	2.58E+08	2.95E+08	2.49E+08	3.63E+08	2.4E+08	3.21E+08	3.09E+08
7.7E+08		4.56E+08		4.68E+08	5.57E+08	7.5E+08	7.62E+08	5.74E+08
1.37E+08	1.91E+08	2.4E+08	2.35E+08	2.02E+08	4.13E+08	2.75E+08	3.08E+08	3.15E+08
2.32E+08	2.74E+08	2.57E+08	3.14E+08	3.02E+08	2.39E+08	2.85E+08	2.76E+08	2.18E+08
2.68E+09	2.35E+09	1.04E+09	1.03E+09	1.04E+09	1.8E+09	1.51E+09	8.31E+08	1.01E+09
1.66E+08	1.42E+08	80910000	75263000	1.13E+08	1.72E+08	1.77E+08	2.29E+08	95366000
6.45E+08	8.51E+08	8.34E+08	6.22E+08	6.7E+08	1.35E+09	1.53E+09	1.43E+09	7.17E+08
			50010000					
	24425000							
1.15E+08	79275000	1.33E+08	1.24E+08	1.33E+08				1.24E+08
				29101000				
3.59E+08	4.36E+08	2.26E+08	2.07E+08	2.25E+08	3.91E+08	3.87E+08	3.76E+08	2.74E+08
2.34E+08	2.81E+08	2.25E+08	2.22E+08	2.64E+08	2.36E+08	2.1E+08	2.22E+08	
4.05E+08	4.18E+08	3.65E+08	4.33E+08	4.74E+08	3.42E+08	3.89E+08	3.18E+08	2.64E+08
1.34E+08	1.12E+08	75778000	87658000	91780000	1.32E+08	1.24E+08		1.02E+08
1.52E+08	2.22E+08	1.26E+08		97885000	1.68E+08	1.86E+08	1.7E+08	1.02E+08
1.34E+08	2.16E+08				1.79E+08	1.34E+08		
3.63E+09	3.05E+09	2.71E+09	2.73E+09	2.62E+09	1.73E+09	2.73E+09	2.34E+09	2.14E+09
	3.75E+08	1.61E+08	1.74E+08	1.7E+08	3.05E+08	3.43E+08	2.64E+08	2.84E+08
2.52E+08	3.03E+08	2.39E+08	2.35E+08	2.63E+08	3.05E+08	3.29E+08	3.64E+08	3.16E+08
50357000								
3.82E+08	4.59E+08	2E+08	1.43E+08	2.19E+08	2.69E+08	2.95E+08	1.83E+08	2.33E+08
40110000	77691000	71243000		1.4E+08				
1.27E+09	1.45E+09	1.17E+09	9.65E+08	9.04E+08	2.01E+09	2.86E+09	2.16E+09	1.63E+09
1.06E+09	9.87E+08	6.11E+08	6.85E+08	5.7E+08	8.57E+08	9.37E+08	9.38E+08	6.68E+08

		1.12E+08	76822000	1.18E+08	72563000			
1.19E+08	1.27E+08	1.13E+08	1.29E+08	1.38E+08	1.75E+08	1.58E+08	1.9E+08	1.07E+08
1.42E+08	1.63E+08	79574000			1.41E+08	1.77E+08	1.33E+08	
1.17E+09	1.19E+09	1.2E+09	1.25E+09	1.47E+09	1.2E+09	1.28E+09	1.13E+09	1.14E+09
1.01E+08	1.13E+08	66842000			99253000	1.07E+08	1.05E+08	
58501000		90533000	77360000	1.11E+08				71899000
4.22E+08	3.91E+08	3.76E+08	3.92E+08	4.3E+08	4.5E+08	4.09E+08	3.43E+08	2.84E+08
5.99E+08	7.34E+08	9.99E+08	1.16E+09	1.01E+09	8.07E+08	7.96E+08	7.45E+08	7.71E+08
30921000		51520000	49085000	53648000				
1.98E+08	2.36E+08	77978000	96823000	79229000	2.98E+08	2.91E+08	2.84E+08	1.9E+08
7.49E+08	7.16E+08	7.33E+08	5.44E+08	6.68E+08	9.18E+08	9.51E+08	6.57E+08	6.92E+08
6.56E+08	5.6E+08	3.73E+08	1.96E+08	3.69E+08	7.83E+08	8.85E+08	6.17E+08	4.89E+08
	31247000	1.39E+08	1.13E+08					
2.7E+08	2.36E+08	3.16E+08	1.23E+08	1.15E+08		1.44E+08	83926000	2.7E+08
				36813000				
1.04E+08		1.09E+08	1.13E+08	1.31E+08				80643000
7.99E+08	8.19E+08	9.49E+08	9.41E+08	1.05E+09	6.37E+08	6.18E+08	6.06E+08	5.33E+08
1.84E+08	1.77E+08	1.14E+09	8.75E+08	1.11E+09	1.97E+08	1.52E+08	1.43E+08	3.58E+08
1.17E+08	1.61E+09	1.05E+09	8.04E+08	1.2E+09	3.66E+09	4.07E+09	5.77E+09	3.05E+09
1.45E+09	5.63E+08	3.08E+08	2.65E+08	1.59E+08		5.54E+08	2.87E+08	6.88E+08
5.59E+08	6.35E+08	3.79E+08	3.74E+08	4.27E+08	7.91E+08	8.89E+08	9.55E+08	6.91E+08
57871000	1.27E+08	1.36E+08	82390000	67039000	1.13E+08	1.64E+08	36654000	73381000
		71793000		60046000				
1.36E+08	1.26E+08	2.05E+08	2.01E+08	2.14E+08	1.35E+08	1.5E+08	1.57E+08	1.31E+08
1.36E+08	1.57E+08					1.62E+08		1.36E+08
1.68E+08	1.69E+08	1.73E+08	1.3E+08	1.8E+08	1.6E+08	2.01E+08	1.14E+08	1.62E+08
4.01E+08	4.64E+08	3.27E+08	3.07E+08	4.67E+08	3.23E+08	2.94E+08	4.07E+08	3.05E+08
1.58E+08	1.68E+08	2.35E+08	1.79E+08	2.28E+08	1.43E+08	1.37E+08	1.25E+08	1.08E+08
1.4E+09	1.42E+09	1.02E+09	9.88E+08	9.84E+08	1.16E+09	1.43E+09	1.17E+09	9.32E+08
58698000	67101000	81110000	81059000	76216000	72149000	68858000	54272000	63920000
2.04E+09	2.09E+09	5.71E+08	4.56E+08	5.41E+08	1.68E+09	2.06E+09	1.9E+09	1.15E+09
	84839000				1.12E+08			
1.28E+09	1.25E+09	7.1E+08	9.68E+08	8.21E+08	1.24E+09	1.35E+09	1.16E+09	1.16E+09
3.79E+08	4.25E+08	3.21E+08	2.81E+08	3.82E+08	5.61E+08	5.29E+08	4.29E+08	4.28E+08
7.09E+08	5.32E+08	6.26E+08	6.41E+08	6.86E+08	4.39E+08	5.76E+08	5.24E+08	5.19E+08
20198000	23360000	28736000	19585000	29901000				
1.17E+09	9.35E+08	7.64E+08	8.77E+08	6.48E+08	1.51E+09	1.91E+09	1.52E+09	1.94E+09
1.41E+08		73166000	99320000	64213000				99134000
8.06E+08	8.95E+08	6.16E+08	6.36E+08	5.43E+08	1.04E+09	9.57E+08	8.17E+08	9.78E+08
	1.53E+08	72761000	74616000	1.04E+08	1.54E+08	1.88E+08	1.73E+08	1.48E+08
1.71E+08	1.77E+08	88618000	1.41E+08	69204000	1.94E+08	1.49E+08	1.69E+08	1.1E+08

3.34E+08	3.74E+08	1.75E+08	2.6E+08	2.35E+08	3.11E+08	2.85E+08	2.48E+08	2.65E+08
4.73E+08	4.52E+08	2.22E+08	2.09E+08	2.01E+08	5.68E+08	7.03E+08	6.18E+08	5.44E+08
9.7E+08	8.68E+08	6.06E+08	5.27E+08	6.24E+08	7E+08	8.7E+08	5.33E+08	5.9E+08
2.15E+08	3.39E+08	2.65E+08	3.22E+08	3.45E+08	4.06E+08	3.4E+08	2.33E+08	2.86E+08
6.02E+08	6.83E+08	5.11E+08	4.98E+08	5.49E+08	5.28E+08	5.31E+08	3.19E+08	3.46E+08
1.51E+09	1.35E+09	9.49E+08	1.1E+09	8.65E+08	7.19E+08	8.82E+08	1.22E+09	1.37E+09
4.28E+09	3.87E+09	2.74E+09	2.84E+09	2.85E+09	3.91E+09	3.88E+09	3.08E+09	3.84E+09
8.73E+08	9.65E+08	7.68E+08	7.05E+08	6.67E+08	1.42E+09	2.27E+09	1.65E+09	9.81E+08
2.23E+08	3.13E+08	3.04E+08	3.41E+08	3.09E+08	3.39E+08	3.57E+08	3.62E+08	4.41E+08
8.58E+08	8.8E+08	8.12E+08	7.49E+08	8.66E+08	1.17E+09	1.14E+09	1.06E+09	9.93E+08
1.88E+08	2.28E+08	1.7E+08	1.63E+08	1.77E+08	2.07E+08	2.94E+08	3.18E+08	2.47E+08
		6.32E+08	4.24E+08	7.03E+08				2.05E+08
80184000	1.03E+08	2.11E+08	2.49E+08	2.55E+08	1.13E+08	1.14E+08	73781000	1.47E+08
4.68E+08	5.78E+08	6.23E+08	6.57E+08	5.39E+08	8.65E+08	8.28E+08	7E+08	7.99E+08
2.95E+09	3.39E+09	2.88E+09	3.41E+09	2.66E+09	4.79E+09	4.1E+09	3.59E+09	3.8E+09
1.72E+08	1.61E+08	1.87E+08	2.02E+08	2.11E+08	1.14E+08	91324000	1.11E+08	1.54E+08
1.69E+08	3.72E+08	2.73E+08	3.18E+08	3.34E+08	2.44E+08	2.35E+08	2.13E+08	2.31E+08
2.6E+08	3.57E+08	2.14E+08	2.09E+08	2E+08	3.8E+08	3.03E+08	2.96E+08	2.96E+08
								30813000
					84624000		93198000	86382000
1.98E+08	2.18E+08	1.81E+08	1.97E+08	2.49E+08	2.79E+08	3.13E+08	3.73E+08	2.62E+08
			26267000	44154000				
7.5E+08	7.02E+08	1.66E+09	1.06E+09	2.2E+09	3.82E+08	4.66E+08	3.63E+08	4.17E+08
			34273000					37565000
1.45E+08	2.09E+08	3.1E+08	2.8E+08	2.95E+08	2.88E+08	2.68E+08	1.95E+08	2.16E+08
1.24E+09	1.2E+09	8.82E+08	7.76E+08	7.32E+08	1.42E+09	1.53E+09	1.15E+09	8.47E+08
59135000					62019000	58828000		51987000
3.73E+09	4E+09	5.16E+09	4.16E+09	4.46E+09	2.81E+09	3.17E+09	2.43E+09	2.51E+09
5.97E+08	3.46E+08	1E+08	1.3E+08	1.21E+08		1.35E+08	2.01E+08	3.89E+08
3.1E+08	2.59E+08	3.68E+08	2.9E+08	3.01E+08				4.99E+08
6.38E+08	7.15E+08	4.16E+08	3.61E+08	4.35E+08	8.03E+08	7.28E+08	7.17E+08	6.27E+08
2.97E+08	2.68E+08	1.95E+08	2.03E+08	1.84E+08	2.73E+08	2.91E+08	2.53E+08	2.59E+08
69046000	74064000				52410000	74174000		
	1.73E+08	76431000	83484000	80558000	1.74E+08	1.79E+08		
3.92E+08	4.28E+08	2.01E+08	2.43E+08	2.05E+08	5.63E+08	5.44E+08	6.64E+08	4.48E+08
			17988000					
66657000	66267000	34962000			69278000	96328000	74544000	43056000
62783000	51062000	60651000	34284000	47863000				
2E+08	1.64E+08	2.61E+08	1.77E+08	1.75E+08		89715000		1.63E+08
1.07E+08	1.09E+08	55921000		72918000	1.15E+08	1.36E+08	1.38E+08	96484000
6.02E+08	6.5E+08	4.68E+08	3.66E+08	4.42E+08	7.86E+08	8.07E+08	8.67E+08	4.93E+08
1.02E+08	93745000	1.39E+08	1.05E+08	2.25E+08	87517000	89000000		70148000
3.05E+08	5.88E+08				3.2E+08	3.12E+08		2.91E+08
1.26E+08	1.22E+08	73485000	69715000	70903000	1.06E+08	1.14E+08	1.25E+08	71181000

2.56E+08	1.76E+08	92188000	1.36E+08	1.17E+08			2.36E+08	1.34E+08
3.89E+08	4.76E+08	1.16E+08	1.91E+08	1.44E+08	4.26E+08	4.06E+08	2.74E+08	1.79E+08
	34463000		32331000	32266000				
		67622000	80752000	69034000	1.5E+08			
99949000	1.09E+08	1.61E+08	1.09E+08	1.39E+08	1.4E+08	1.34E+08	89148000	1.23E+08
	77370000	83875000	78869000	81804000	86360000	1.03E+08	1.25E+08	90624000
1.47E+09	1.49E+09	1.64E+09	1.76E+09	1.65E+09	1.71E+09	1.79E+09	1.35E+09	1.34E+09
		1.15E+08	92439000			93215000		
65390000	89905000		50279000	47997000	66987000			50877000
2.06E+09	1.79E+09	1.17E+09	1.04E+09	1.03E+09	2.36E+09	2.46E+09	2.27E+09	1.76E+09
8.55E+08	1.1E+09	5.46E+08	4.84E+08	5.24E+08	1.61E+09	1.64E+09	1.5E+09	1.51E+09
3.38E+09	2.87E+09	2.51E+09	2.98E+09	2.83E+09	2.66E+09	3E+09	3.46E+09	4.08E+09
					66855000	66624000	63147000	66465000
		1.11E+08	1.31E+08			1.32E+08		1.2E+08
1.31E+08	93674000	69817000	49252000	54535000	57509000			
42326000	75683000	1.16E+08	93798000	1.01E+08			50235000	
1.38E+08	1.23E+08	1.07E+08	68044000	1.01E+08	1.02E+08	1.09E+08	1.09E+08	89210000
84350000	1.17E+08	1.64E+08	1.23E+08	2.38E+08	80502000	80525000		84823000
61896000	83147000	50419000				52528000		
	4.38E+08	2.11E+08		3.54E+08	6.07E+08			5.31E+08
6.75E+08	7.31E+08	7.01E+08	7.56E+08	6.13E+08		4.97E+08	5.2E+08	
1.03E+09	1.04E+09	7.76E+08	4.89E+08	6.77E+08	7.3E+08	7.67E+08	8.19E+08	5.75E+08
7.54E+08	8.25E+08	3.61E+08	4.04E+08	3.28E+08	7.4E+08	8.49E+08	6.43E+08	6.1E+08
1.16E+09	1.16E+09	8.76E+08	1.08E+09	7.94E+08	1.23E+09	1.29E+09	1.12E+09	1.31E+09
7.59E+08	8.2E+08	6.78E+08	3.23E+08	5.95E+08	4.8E+08	5.62E+08	6.16E+08	4.43E+08
3.28E+09	3.25E+09	2.38E+09	2.35E+09	2.22E+09	2.53E+09	2.29E+09	1.86E+09	1.75E+09
32695000		28040000				58460000		
								1.12E+08
6.91E+08	5.15E+08	3.62E+08	4.99E+08	3.77E+08	3.27E+08	3.85E+08	4.31E+08	4.69E+08
			1.61E+08					
1.07E+09	1.13E+09	1.67E+09	1.68E+09	1.63E+09	1.88E+09	1.86E+09	1.46E+09	1.51E+09
6.13E+08	7.42E+08	1.36E+09	1.17E+09	1.24E+09	1.25E+09	9.92E+08	8.31E+08	1.19E+09
8.26E+08	9.76E+08	1.94E+08	1.62E+08	2.27E+08	9.89E+08	1.06E+09	7.69E+08	4.9E+08
7.93E+08	9.01E+08	9.31E+08	1.01E+09	1.08E+09	1.57E+09	1.6E+09	1.04E+09	1.84E+09
		15823000						
6.84E+08	7.55E+08	5.96E+08	6.02E+08	5.65E+08	7.93E+08	9.7E+08	9.57E+08	6.67E+08
87726000	83908000	89484000	55284000		80593000	98309000	92186000	69832000
1.16E+08	1.15E+08	1.13E+08	1.62E+08	1.34E+08		2.01E+08	1.99E+08	1.53E+08
1.25E+08	1.36E+08		85709000		1.96E+08	1.93E+08		82682000
3.94E+08	5.04E+08	3.56E+08	4E+08	4.5E+08	6.19E+08	5.45E+08	4.2E+08	3.29E+08
1.2E+08	1.6E+08	1.5E+08	1.59E+08	1.3E+08		97643000		1.14E+08
3.39E+08	2.6E+08	2.22E+08	1.37E+08				1.88E+08	2.13E+08
59695000	56204000	41072000						
68437000		84008000	64436000	75153000	39726000	49073000		
3.63E+08	4.49E+08	3.6E+08	3.4E+08	3.76E+08	4.5E+08	4.56E+08	5.5E+08	3.79E+08
3E+08	3.29E+08	2.04E+08	2.1E+08	1.4E+08	3.16E+08	3.48E+08	2.89E+08	2.77E+08

4.28E+08	5.13E+08	2.88E+08	4.78E+08	4.51E+08	4.1E+08	3.84E+08	4E+08	4.54E+08
1.21E+08	1.65E+08	2.14E+08	2.34E+08	2.2E+08	3.71E+08	2.83E+08	2.24E+08	2.09E+08
32193000	43967000	36133000	46084000	49999000	70725000	40942000	60888000	47430000
1.94E+08	2.01E+08	1.36E+08	1.61E+08	1.39E+08	1.44E+08	1.26E+08	1.38E+08	1.14E+08
2.9E+08	4.06E+08	2.39E+08	3.29E+08	2.57E+08	3.61E+08	3.35E+08	3.05E+08	3.05E+08
74152000	54551000	89442000	46994000	68379000				1.03E+08
1.96E+08	2.26E+08	2.41E+08	2.16E+08	2.66E+08	2.12E+08	1.88E+08	2.06E+08	2.08E+08
5.18E+08		3.53E+08		5.36E+08				
		44743000	65830000	80794000				12058000
3.62E+08	3.93E+08	2.59E+08	2.39E+08	2.02E+08	3.74E+08	3.62E+08	3.08E+08	3.59E+08
4.47E+08	4.99E+08	2.97E+08	2.81E+08	2.79E+08	5.56E+08	6.48E+08	6.05E+08	4.27E+08
3.13E+08	2.87E+08	3.82E+08	3.34E+08	3.76E+08	3.47E+08	3.72E+08	3.95E+08	2.78E+08
3.52E+08	4.19E+08	1.88E+08	2.08E+08	1.96E+08	3.9E+08	4.35E+08	3.78E+08	2.66E+08
				26403000				27308000
3.82E+08	5.46E+08	4.14E+08	4.48E+08	4.01E+08	8.79E+08	1.04E+09	7.5E+08	7.05E+08
72978000	1.1E+08	1.49E+08	3.83E+08	1.59E+08	1.81E+08	1.65E+08	1.15E+08	1.52E+08
1.42E+08	1.31E+08				1.38E+08		1.35E+08	
5.01E+08	5.75E+08	4.53E+08	5.07E+08	5.83E+08	8.17E+08	8.73E+08	8.6E+08	7.64E+08
	4.28E+08		5.79E+08			9.6E+08		
5.47E+08	6.6E+08		1.17E+08	2.17E+08	7.54E+08	8.21E+08	9.91E+08	4.61E+08
1.07E+08	1.23E+08	1.63E+08	1.46E+08	1.54E+08	1.42E+08	1.31E+08	1.1E+08	98378000
				31423000				
63103000	72365000	69076000	77472000	62312000	74829000	1.16E+08	1.32E+08	73795000
								70861000
1.05E+08	1.18E+08	1.08E+08	1.17E+08	1.18E+08	1.15E+08	1.1E+08	1.21E+08	1.13E+08
2E+08	3.12E+08	3.68E+08	3.25E+08	2.99E+08	6.72E+08	6.33E+08	6.17E+08	5.76E+08
1.33E+10	1.18E+10	1.09E+10	1.14E+10	1.23E+10	1.38E+10	1.51E+10	8.69E+09	1.27E+10
1.15E+08	1.2E+08	54962000	45727000	52552000	1.42E+08	1.54E+08	1.9E+08	1.06E+08
	9.95E+08	1.92E+08	8.6E+08		1.03E+09	1.4E+09	2.48E+09	1.18E+09
3.94E+08	5.86E+08	1.62E+08	1.54E+08	1.44E+08	4.02E+08	4.2E+08	3.05E+08	2.92E+08
46524000	62625000	39230000	30054000	39654000	50197000	61371000	62336000	43037000
2.35E+08	2.19E+08	2.3E+08	2.1E+08	2.74E+08		1.42E+08		
2.72E+08	2.5E+08	3.99E+08	2.04E+08	3.63E+08	1.44E+08	1.58E+08	2.23E+08	2.24E+08
4.85E+08	4.88E+08	5.41E+08	4.82E+08	5.01E+08	5.82E+08	7.82E+08	6.82E+08	4.41E+08
1.39E+09	1.05E+09	8.88E+08	8.63E+08	8.92E+08	8.42E+08	1.37E+09	8.89E+08	1.09E+09
6.52E+08	7.39E+08	4.19E+08	4.53E+08	3.74E+08	7.44E+08	8.34E+08	8.54E+08	5.75E+08
6.34E+08	5.1E+08	4.57E+08	3.46E+08	4.11E+08	8.53E+08	5.59E+08	5.64E+08	7.17E+08
15669000		64947000	42793000	60888000	44523000			
2.92E+08	3.2E+08	2.7E+08	2.28E+08	2.66E+08	3.01E+08	4.83E+08	4.31E+08	3.28E+08
	65956000	57392000	48597000			72424000		48587000
24864000	49066000	16631000	50908000	32834000				
1.56E+08	1.63E+08	1.03E+08	1.13E+08	1.05E+08	1.51E+08	1.56E+08	1.6E+08	1.67E+08

1.94E+08	96352000	96853000	1.58E+08	85713000	4.33E+08	3.05E+08	2.63E+08	6.29E+08
59876000	67099000	32253000	47338000	45872000	18422000	22952000	35279000	52380000
4.36E+08	5.13E+08	2.81E+08	3.43E+08	2.68E+08	4.1E+08	4.84E+08	4.67E+08	4.54E+08
3.24E+09	2.68E+09	3.71E+08	3.58E+08	3.7E+08	6.91E+08	1.39E+09	6.78E+08	1.59E+08
63363000	71795000	1.14E+08	1.17E+08	1.15E+08	1.43E+08	1.38E+08	1.21E+08	2.46E+08
5.17E+08	5.76E+08	3.24E+08	3.81E+08	3.34E+08	6.22E+08	6.53E+08	5.04E+08	4.35E+08
68744000	69476000	4.22E+08	3.52E+08	5.35E+08				1.19E+08
1.92E+08		51339000			2.03E+08	2.11E+08	1.45E+08	1.4E+08
1.01E+08	1.34E+08	48796000	61936000	56097000	1.18E+08	1.14E+08	1.2E+08	87330000
		83833000	76664000	73920000				
43972000	21277000	65471000	79950000	53950000	67375000	55223000	46705000	83838000
1.28E+08	90193000	1.48E+08	1.51E+08	1.41E+08	1.7E+08	1.66E+08	1.28E+08	1.52E+08
1.09E+09	1.3E+09	1.41E+09	1.34E+09	1.31E+09	1.12E+09	8.91E+08	9.64E+08	7.54E+08
	1.59E+08							
	61981000			51885000				
3.33E+09	3.74E+09	3.73E+09	3.78E+09	4.08E+09	7.84E+09	6.32E+09	7.17E+09	6.25E+09
1.61E+08	1.58E+08	1.05E+08	1.54E+08	1.25E+08	1.7E+08	1.57E+08	1.44E+08	1.25E+08
8E+08	6.03E+08	8.8E+08	1.05E+09	7.91E+08				9.11E+08
1.06E+09	1.03E+09	9.86E+08	7.37E+08	1.07E+09	1.14E+09	1.04E+09	9.59E+08	8.47E+08
2.41E+09	2.17E+09	1.47E+09	1.09E+09	9.59E+08	2.16E+09	1.17E+09	1.39E+09	1.25E+09
2.12E+08	2.36E+08	1.7E+08	1.86E+08	1.73E+08	1.96E+08	2.25E+08	1.4E+08	1.58E+08
		29030000						
		43167000	26639000	35010000				41581000
60979000	78786000	81029000	81827000	80232000	92500000		86249000	77597000
12857000	24044000	20786000	36187000	26205000			26904000	60528000
	1.23E+08	94916000	1.12E+08	99318000				
1.1E+08		89223000		85469000	1.29E+08		1.14E+08	
91816000	1.3E+08	5.01E+08	4.75E+08	4.67E+08	2.12E+08	2.1E+08	2.54E+08	4.01E+08
					60529000		52649000	
3.05E+08	3.78E+08	3.86E+08	3.46E+08	3.67E+08	2.93E+08	3.22E+08	3.23E+08	2.04E+08
1.78E+08	2.24E+08	1.11E+08	1.27E+08	1.25E+08	1.82E+08	2.7E+08	2.48E+08	2.37E+08
2.52E+08	2.68E+08	2.56E+08	1.74E+08	1.92E+08	2.42E+08	2.29E+08	3.14E+08	3.05E+08
59840000	66443000	64884000		63797000				
2.36E+08	2.32E+08	1.98E+08	1.29E+08	1.23E+08	1.1E+08	1.32E+08	2.03E+08	2.34E+08
		19257000						
			3.1E+08	5.58E+08				
1.84E+08	92052000	51550000	86658000	47918000	56887000	17162000	79966000	1.18E+08
2.31E+08	2.1E+08	7.05E+08	4.12E+08	8.05E+08	1.58E+08		2.11E+08	3.42E+08

6.01E+08	4.21E+08	2.41E+08	2.44E+08	2.02E+08			2.37E+08	4.04E+08
56217000	83800000	2.56E+08	3.02E+08	2.61E+08	57780000			30918000
1.16E+10	1.31E+10	8.3E+09	5.85E+09	9.12E+09	9.05E+09	7.16E+09	4.25E+09	6.82E+09
86779000	1.27E+08	58942000	73373000	89225000	1.14E+08	92969000	1.16E+08	1.72E+08
2.42E+08								2.3E+08
	1.54E+09							
3.82E+08	3.92E+08		1.61E+08					
1.48E+08	1.7E+08	64788000	76306000	64760000	1.83E+08	2.64E+08	1.92E+08	1.19E+08
1.89E+08	2.85E+08	3.59E+08	4.47E+08	3.82E+08	1.39E+08	1.39E+08		1.11E+08
63312000	71370000	52921000	59263000	60315000	76044000	64846000		49350000
1.41E+08		1.28E+08	1.39E+08	1.7E+08	1.64E+08	2.46E+08		1.16E+08
2.3E+08	2.78E+08	3.22E+08	3.23E+08	3.09E+08	2.67E+08	2.84E+08	3.05E+08	2.35E+08
3.81E+08	2.43E+08	3.32E+08	3.31E+08	2.77E+08	1.39E+08	1.71E+08	1.75E+08	2.52E+08
1.85E+08		2.01E+08	1.82E+08	2.36E+08	1.3E+08	1.59E+08	1.99E+08	1.31E+08
		24130000	21922000	26124000				
1.51E+08	1.05E+08	1.07E+08	1.24E+08	1.03E+08	1.43E+08	1.07E+08	1.2E+08	1.36E+08
8.83E+08	7.12E+08	7.55E+08	1.11E+09	8.74E+08	9E+08	9.15E+08	5.06E+08	1.05E+09
91365000	88572000	69067000		93508000	84052000			
3.07E+09	2.93E+09	1.22E+09	8.77E+08	1.3E+09	1.77E+09	1.8E+09	2.27E+09	1.28E+09
		6.5E+08	4.85E+08	5.88E+08	7.08E+08	7.33E+08	8.04E+08	1.6E+09
			2.5E+08	2.65E+08	5.72E+08	2.25E+08		2.77E+08
47454000	64518000	53424000	62060000	58695000	76691000	66341000	83974000	57467000
				26528000				
	2.56E+08	2.03E+08	1.88E+08	1.41E+08	3.48E+08	2.86E+08	3.04E+08	2.64E+08
2.37E+08	2.11E+08	3.06E+08	3.01E+08	2.97E+08	4.47E+08	2.92E+08	2.58E+08	2.65E+08
83587000	2.55E+08	1.6E+08	1.61E+08	6.46E+08	87529000	1.06E+08	1.64E+08	2.52E+08
5.33E+09	4.82E+09	6.73E+09	6.26E+09	6.12E+09	6.12E+09	6.5E+09	5.82E+09	6.51E+09
2.86E+08	3.47E+08	2.34E+08	2E+08	1.59E+08	4.47E+08	3.25E+08	5.06E+08	3.99E+08
1.27E+08	1.4E+08	66668000	75103000		1.55E+08	1.78E+08		
5.24E+08	6.43E+08	7.21E+08	7.62E+08	6.82E+08	6.23E+08	6.6E+08	6.92E+08	6.13E+08
1.18E+08	1.03E+08				85706000	85759000	72257000	56230000
3.36E+08	3.26E+08	2.99E+08	2.29E+08	3.79E+08	1.71E+08	2.04E+08	1.63E+08	2.11E+08
5.98E+08	2.84E+09	1.14E+09	1.18E+09	1.02E+09	2.88E+09	5.05E+09	2.8E+09	1.66E+09
63873000	64106000	40167000			91826000	83615000	97178000	83239000
30008000	28549000	30025000	32787000	32118000			27310000	
88336000	1.13E+08	1.3E+08	1.58E+08	1.73E+08	1.11E+08	87295000	74937000	1.01E+08

	50360000	37550000	29466000	34743000	69242000	40315000		22214000
30321000	23095000	35813000	36766000	33502000				
92414000	77586000	1.4E+08	1.53E+08	70182000	73683000			
1.22E+09	1.11E+09	3.29E+08		3.13E+08	1.11E+09	1.17E+09	1.47E+09	1.23E+09
2132300	2062600					1254700		1552900
1.13E+08		47282000		38762000			1.33E+08	78858000
3.5E+09	3.01E+09	2.65E+09	2.63E+09	2.57E+09	2.29E+09	2.47E+09	1.82E+09	1.88E+09
2.57E+08	4.07E+08	2.96E+08	2.66E+08	2.73E+08	3.23E+08	4.12E+08	3.42E+08	3.37E+08
2.35E+09	2.91E+09	1.97E+09	2.35E+09	2.14E+09	3.3E+09	3.4E+09	3.39E+09	2.23E+09
	28316000	22078000	18605000					
59174000		35219000	48972000		80101000	89889000	93439000	98096000
	53873000							
2.9E+09	2.77E+09	3.63E+09	3.17E+09	3.91E+09	2.94E+09	2.9E+09	1.83E+09	3.34E+09
3.6E+08	1.25E+08							
84573000	1.03E+08	1.49E+08	1.48E+08	1.34E+08	2.39E+08	1.74E+08	1.39E+08	2.18E+08
		3.04E+08	3.08E+08	3.3E+08		4.5E+08		
90148000	96831000	91505000	88248000	89295000	83073000	77628000	73615000	64479000
2.21E+08	2.69E+08	1.75E+08	2.27E+08	2.02E+08	2.46E+08	2.75E+08	2.04E+08	2.63E+08
2.01E+08	2.34E+08	1.9E+08	1.96E+08	2.37E+08	2.14E+08	2.49E+08	1.84E+08	2.57E+08
1.41E+08	1.96E+08	1.99E+08	1.66E+08	1.25E+08	1.32E+08	94220000	1.2E+08	1.57E+08
					1.63E+08	1.34E+08		
4.21E+09	4.68E+09	2.05E+09	2.02E+09	2.07E+09	5.57E+09	6.82E+09	4.98E+09	4.04E+09
2.09E+08	2.16E+08	1.23E+08	1.17E+08	1.31E+08	2.01E+08	2.13E+08	2.24E+08	1.41E+08
2.18E+08	2.33E+08	2.21E+08	2.22E+08	2.41E+08	2.54E+08	2.78E+08	2.14E+08	2.08E+08
3.73E+08	3.66E+08				4.08E+08	3.15E+08		
5.77E+08	9.46E+08	2.18E+08	2.14E+08	2.11E+08	7.11E+08	6.29E+08	6.48E+08	3.6E+08
95192000	1.16E+08	76510000	84672000	77983000				
89739000	80135000	69813000	62880000	76068000	1.13E+08	91143000	72586000	78595000
		51045000	54885000				1.62E+08	72245000
8.02E+08	1.01E+09	5.13E+08	6.94E+08	6.48E+08	1.03E+09	1E+09	8.84E+08	8.29E+08
2.24E+08	3.79E+08	3.95E+08	3.34E+08	3.51E+08	4.55E+08	4.88E+08	5.54E+08	3.68E+08
1.98E+08	2.57E+08	87001000	1.14E+08	1.16E+08	1.96E+08	1.57E+08	1.28E+08	1.25E+08
2.46E+08	3.03E+08	2.18E+08	2.11E+08	2.7E+08	2.8E+08	2.76E+08	1.8E+08	1.5E+08
		88460000	54181000	77034000				
2.13E+09	2.41E+09	4.06E+09	2.58E+09	3.68E+09	9.04E+08	1.15E+09	1.69E+09	1.65E+09
1.16E+08	1.08E+08	42901000	72997000	85745000	85594000	1.03E+08		1.14E+08
3.5E+08	2.63E+08	1.75E+08	1.61E+08	1.57E+08	2.41E+08	1.94E+08	1.99E+08	2.45E+08
2.52E+09	2.26E+09	3.97E+09	3.8E+09	3.52E+09	2.87E+09	2.42E+09	2.36E+09	3.43E+09
2.23E+09	1.93E+09	1.52E+09	1.54E+09	1.7E+09	2.79E+09	3.04E+09	2.81E+09	2.05E+09
		46008000						

2.36E+09	2.77E+09	1.73E+09	1.15E+09	1.48E+09	3.4E+09	4.09E+09	4.06E+09	2.36E+09
4.78E+08	5.37E+08	1.19E+09	1.16E+09	1.28E+09	6.4E+08	6.41E+08	6.25E+08	6.92E+08
8.57E+08	7.14E+08	1.9E+09	1.54E+09	1.14E+09	4.13E+08	4.06E+08	7.28E+08	8.72E+08
1.26E+09	1.17E+09	7.42E+08	6.54E+08	7.96E+08	9.93E+08	9.7E+08	9.56E+08	8.16E+08
3.38E+08	3.85E+08	1.54E+08	1.25E+08	1.61E+08	2.77E+08	2.71E+08	1.92E+08	1.82E+08
				1.21E+08	1.44E+08			
2.33E+08	2.21E+08	94375000	1.71E+08	1.29E+08	1.83E+08	1.44E+08	1.53E+08	1.32E+08
9.88E+08	1.31E+09	7.3E+08	7.75E+08	9.42E+08	1.09E+09	1.39E+09	1.01E+09	8.35E+08
4.44E+08	5.13E+08	3.97E+08	6.15E+08	5.46E+08	5.64E+08	6.37E+08	4.95E+08	4.69E+08
3.23E+09	2.84E+09	2.29E+09	2.37E+09	2.76E+09	3.98E+09	4.34E+09	4.05E+09	3.82E+09
1.09E+09	1.49E+09	1.53E+09	1.56E+09	1.3E+09	2.67E+09	2.23E+09	2.06E+09	2.11E+09
2.68E+09	3.27E+09	2.04E+09	2.17E+09	2.08E+09	3.75E+09	3.29E+09	3.37E+09	2.66E+09
2.36E+08	3.54E+08	3.31E+08	3.95E+08	3.43E+08	3.29E+08	2.84E+08	2.53E+08	2.14E+08
1.02E+08	71963000	73938000	47566000	72724000	82364000	78294000	40429000	72422000
1.96E+10	1.96E+10	1.14E+10	1.22E+10	1.15E+10	2.11E+10	2.26E+10	1.78E+10	1.77E+10
		80826000						
		1.84E+08	2.27E+08	2.37E+08	3.21E+08	2.88E+08	3E+08	
3.04E+08	3.1E+08	2.1E+08	1.84E+08	2.81E+08	2.53E+08	2.44E+08	1.92E+08	1.87E+08
2.23E+08	2.51E+08	1.35E+08	1.69E+08	1.48E+08	4.36E+08	4.16E+08	4.71E+08	3.63E+08
55361000	55807000	67499000	55330000	63850000	59495000		45634000	42299000
3.63E+08	4.6E+08	1.12E+08	1.11E+08	1.05E+08	8.13E+08	6.83E+08	8.34E+08	5.47E+08
3.15E+08	2.56E+08	5.68E+08	5E+08	4.94E+08	4.44E+08	4.68E+08	5.19E+08	4.45E+08
1E+08	97873000	2.18E+08	2.22E+08	1.33E+08	1.27E+08	1.55E+08	1.59E+08	98860000
		28196000						
4.94E+08	4.62E+08	6.41E+08	3.96E+08	5.52E+08	4.32E+08	4.72E+08	4.78E+08	4.74E+08
68375000	64671000	44205000	44336000	32076000	57358000	68430000	72300000	
3.62E+08	4.21E+08	5.13E+08		3.65E+08	4.35E+08	5.53E+08		3.22E+08
		21369000		21554000				
	2.34E+08	1.72E+08	2.12E+08	1.98E+08	3.1E+08			2.97E+08
1.54E+08	53608000	1.16E+08	1.04E+08	84823000	19635000	24650000	18351000	42727000
9.54E+08	1E+09	1.64E+09	1.68E+09	1.64E+09	2.38E+09	2.8E+09	1.9E+09	4.04E+09
1.76E+08	1.53E+08	1.67E+08	1.47E+08	1.25E+08	1.34E+08	1.72E+08	2.33E+08	1.8E+08
5.4E+08	5.35E+08	5.17E+08	5.41E+08	5.35E+08	6.44E+08	4.99E+08	5.43E+08	4.59E+08
2.29E+08	2.55E+08	1.97E+08	1.88E+08	1.82E+08	1.97E+08	2.43E+08	2.13E+08	93623000
		24879000		31482000				
				88492000				1.52E+08
9.8E+08	7.62E+08	5.74E+08	4.61E+08	5.62E+08	9.89E+08	9.41E+08	9E+08	7.48E+08
	1.01E+08							
80659000	85191000	47013000	53735000	42719000				68045000

			23216000					
	58186000	52138000	82032000	66230000	87234000	91335000	1.01E+08	90331000
1.89E+08	3.32E+08	4.31E+08	4.21E+08	4.6E+08	5.2E+08	5.2E+08	4.9E+08	4.75E+08
5.69E+08	3.82E+08	3.41E+08	3.52E+08	3.64E+08	4.81E+08	6.05E+08	5.19E+08	3.32E+08
	3.23E+08	3.21E+08	3.41E+08	3.23E+08	1.43E+08			2.08E+08
1.18E+08	1.46E+08	63526000	40724000	47140000	88876000	1.23E+08	1.32E+08	84968000
44692000	59699000	39939000	50851000	41430000	62804000	69379000	64649000	47828000
8.53E+09	7.95E+09	4.53E+09	5.34E+09	5.43E+09	6.65E+09	8.79E+09	8.94E+09	8.27E+09
1.47E+09	1.58E+09	1.42E+09	1.6E+09	1.51E+09	1.24E+09	1.74E+09	2.24E+09	1.83E+09
		29071000						
1.11E+08	1.44E+08	1.56E+08	1.55E+08	1.47E+08	1.65E+08	2.21E+08	1.77E+08	1.54E+08
1.16E+08	1.25E+08	1.11E+08	1.31E+08	96680000		1.39E+08		
		52096000				1.1E+08		82732000
3.25E+08	3.13E+08	4.56E+08	6.55E+08	6.58E+08	8.31E+08	6.53E+08	9.26E+08	5.02E+08
	23475000			21929000				
1.9E+08	96834000	1.16E+08	1.39E+08	1.81E+08	1.13E+08	1.51E+08	83508000	1.31E+08
		1.65E+08		1.9E+08				1.01E+08
1.26E+09	1.93E+09	1.76E+09	1.79E+09	1.29E+09	1.48E+09	1.54E+09	2.18E+09	1.11E+09
3.5E+08	4.43E+08	1.69E+08	2.32E+08	1.85E+08	3.99E+08	3.97E+08	3.26E+08	2.95E+08
2.71E+08	2.58E+08	2.99E+08	2.28E+08	2.61E+08	2.93E+08	2.22E+08	2.45E+08	1.96E+08
97660000	1.02E+08	1.35E+08	1.48E+08	1.51E+08		1.02E+08	85928000	1.16E+08
2.23E+08	2.18E+08		1.77E+08		2.29E+08	2.92E+08	2.42E+08	3.9E+08
1.38E+08	1.52E+08	1.34E+08	1.72E+08	1.41E+08	2.18E+08	1.46E+08	1.75E+08	1.07E+08
4.16E+08	1.38E+09	6.47E+08	4.88E+08	5.28E+08	1.15E+09	1.29E+09	1.02E+09	1.24E+09
1.88E+08	2.98E+08		2.19E+08		2.35E+08	2.47E+08		1.95E+08
		80882000	76758000	67876000				
3.24E+08	4.18E+08		3.33E+08	2.61E+08	1.87E+08	2.25E+08		2.34E+08
9.29E+08	1.15E+09	3.29E+09	3.08E+09	3.47E+09	1.09E+09	1.05E+09	9.14E+08	1.4E+09
81623000	1.59E+08	76350000	64654000	65299000	3.13E+08		1.69E+08	3.11E+08
	54840000	64574000	1.09E+08	1.12E+08			41822000	67386000
4.18E+08	4.64E+08	3.21E+08	3.86E+08	3.57E+08	4.61E+08	3.94E+08	3.58E+08	4.04E+08
3.29E+08	2.85E+08	2.41E+08	1.64E+08	1.63E+08	2.67E+08	2.56E+08	2.76E+08	1.85E+08
2.81E+08	3.6E+08	2.26E+08	2.31E+08	1.93E+08	4.22E+08	4.34E+08	5.82E+08	2.59E+08
1.1E+09	1.28E+09	1.76E+09	1.45E+09	1.99E+09	9.84E+08	1.02E+09	7.16E+08	8.37E+08
8.03E+08	6.99E+08	4.16E+08	3.38E+08	3.74E+08	9.48E+08	7.28E+08	5.56E+08	4.05E+08
66069000	72895000			56721000				
92519000	1.48E+08	94981000	1.29E+08	1E+08	1.31E+08	1.41E+08	1.12E+08	84445000
6.74E+08	8.09E+08	8.42E+08	7.8E+08	8.98E+08	6.95E+08	7.14E+08	7.98E+08	6.57E+08

19431000	24824000	33937000	22126000	16837000				15919000
81748000	97505000	1.35E+08	1.99E+08	1.86E+08	94239000	93081000	1.14E+08	1.04E+08
	88828000	1.13E+08	1.34E+08	1.34E+08				
		1.53E+08	1.48E+08	1.54E+08		1.61E+08	1.55E+08	
2.04E+08	2.25E+08	3.26E+08	2.93E+08	3.56E+08	2.88E+08	2.79E+08	2.54E+08	2.73E+08
	1.55E+08	1.52E+08	1.64E+08	1.74E+08				1.38E+08
5.71E+08	5.64E+08	3.79E+08	3.44E+08	3.36E+08	6.32E+08	6.49E+08	5.29E+08	4.33E+08
		44793000	37823000	48352000		51561000		
1.17E+08	1.03E+08	21167000		28965000	64522000	87359000	60810000	57988000
49025000	53124000	62748000	66736000	63614000		48872000		
75056000	88044000	82534000	80941000		72792000	54457000	46470000	52128000
1.69E+09	1.65E+09	2.13E+09	1.66E+09	1.98E+09	1.5E+09	1.78E+09	1.44E+09	1.47E+09
1.39E+09	1.65E+09	2.58E+09	2.5E+09	2.7E+09	1.58E+09	1.81E+09	1.31E+09	1.43E+09
1.62E+08	1.76E+08	1.36E+08	1.15E+08	1.13E+08	1.6E+08	1.93E+08	1.72E+08	1.36E+08
6.72E+08	6.45E+08	6E+08	3.64E+08	5.23E+08	6.4E+08	8.49E+08	7.08E+08	6.36E+08
8.57E+08	9.2E+08	6.89E+08	6.95E+08	6.88E+08	9.43E+08	8.66E+08	1.04E+09	6.56E+08
	96752000	1.3E+08	1.81E+08	2.1E+08	99365000			
36157000		59513000	56889000	56546000				66928000
1.65E+08	1.67E+08	2.46E+08	2.35E+08	2.9E+08	1.55E+08	1.79E+08	1E+08	3.14E+08
							3.39E+08	
		13880000		18259000				
37431000	27385000	24542000	31420000	33495000		46126000	43438000	41287000
1.79E+09	2.1E+09	2.24E+09	1.95E+09	2.18E+09	2.89E+09	2.65E+09	2.81E+09	1.84E+09
	47128000				54602000	60626000		51651000
					2.66E+08	3.14E+08	2.33E+08	2.23E+08
		38128000	44001000	35418000	34940000	26425000	23485000	
1.35E+09	1.44E+09	1.65E+09	2.16E+09	1.91E+09	1.44E+09	1.47E+09	1.43E+09	1.54E+09
2.75E+09	4.01E+09	1.35E+09	1.44E+09	2E+09	3.81E+09	3.17E+09	3.33E+09	3.43E+09
1.37E+09	8.73E+08	1.5E+09	2.3E+09	2.09E+09	5.05E+08	7.19E+08	5.02E+08	2.45E+09
95092000	48420000				62032000	73253000		57549000
	24813000				22565000			
73315000	1.44E+08							
71923000	82981000	69890000	83033000	79531000	79343000	85153000	81786000	58176000
							65777000	
17977000		42699000	41607000	30442000				
89157000		1.17E+08	77860000	1.58E+08				
55648000	45164000	38833000	41041000	27614000				44544000
1.23E+08	1.16E+08	1.35E+08	1.31E+08	1.27E+08	1.92E+08	1.64E+08	1.83E+08	1.89E+08
98801000	73995000	34954000	55246000		1.09E+08	78146000		62060000
					1.9E+08	2.26E+08	2.47E+08	
2.32E+08								1.89E+08

		59137000	81479000	97012000				
8.68E+08	7.49E+08	3.96E+08	4.62E+08	4E+08	6.48E+08	4.95E+08	8.11E+08	
54887000	42004000	32482000		41575000				44171000
2.34E+08	3.44E+08	1.84E+08	2.12E+08	2.67E+08	2.72E+08	2.8E+08	1.91E+08	1.79E+08
2.26E+08	2.35E+08	84439000	66501000	96195000		2.43E+08	2.2E+08	1.83E+08
		1.95E+08	1.94E+08	1.54E+08		81475000		1E+08
74111000	60142000	89905000		1.02E+08	69236000			
1.42E+08	2.09E+08	96352000	1.07E+08	1.25E+08	1.97E+08	2.13E+08	1.83E+08	1.69E+08
4.02E+08	3.57E+08	4.54E+08	5.18E+08	4.29E+08	8.87E+08	7.25E+08	6.22E+08	5.44E+08
92862000	1.3E+08	1.11E+08	1.13E+08	1.11E+08	1.18E+08	1.22E+08	80314000	74136000
70678000	95518000	1.29E+08	1.72E+08	1.39E+08	1.06E+08			1.04E+08
1.18E+08	1.28E+08	2.03E+08	2E+08	2.09E+08	1.88E+08	1.2E+08	1.45E+08	1.21E+08
		61010000	38192000	38153000	46105000		47908000	55334000
48656000			1.01E+08	70936000				
1.07E+08	1.28E+08	89755000	84165000	93038000	1.7E+08	1.91E+08	1.61E+08	1.63E+08
1.27E+08	1.26E+08	1.35E+08	1.7E+08	1.28E+08	1.82E+08	1.64E+08	1.57E+08	1.12E+08
1.47E+09	4.98E+08	1.2E+08	1.84E+08	1.1E+08	3.43E+08	4.31E+08	3.33E+08	2.42E+08
3.47E+08	2.89E+08	2.63E+08	2.75E+08					
1.06E+09	1.19E+09	1.09E+09	1.02E+09	1.02E+09	1.09E+09	1.1E+09	9.84E+08	9.31E+08
						31223000	38033000	
6.3E+08	6.85E+08	4.79E+08	5.71E+08	5.66E+08		1.01E+09	1.23E+09	9.88E+08
4.91E+08	5.44E+08	3.06E+08	3.56E+08	3.5E+08	5.66E+08	5.13E+08	4.02E+08	3.5E+08
1.11E+08	76891000	2.88E+08	2E+08	2.7E+08	96631000	1.01E+08	86910000	94124000
3.52E+08	5.68E+08	5.15E+08	6.92E+08	4.73E+08		6.8E+08	2.11E+09	6.13E+08
1.38E+08	1.53E+08	43508000	36624000	43777000	1.58E+08	1.14E+08	1.58E+08	1.28E+08
2.2E+08	1.71E+08	1.46E+08	1.43E+08	1.36E+08	97932000	98154000	1.43E+08	2.06E+08
		55035000	2.17E+08	35935000				
1.33E+08	1.67E+08	1.57E+08	1.27E+08	1.68E+08	2.12E+08	2.18E+08	1.97E+08	1.97E+08
2.69E+09	3.86E+09	1.43E+09	1.37E+09	1.44E+09	3.86E+09	4.04E+09	3.99E+09	2.95E+09
1.31E+08	1.15E+08	53269000	79341000	63727000		1.32E+08	99819000	1.31E+08
6.26E+08	4.65E+08	3.04E+08	2.51E+08	3.23E+08	3.54E+08	3.9E+08	5.96E+08	6.68E+08
4.78E+08	1.92E+08	4.84E+08	4.52E+08	6.42E+08	3.53E+08	3.01E+08	1.72E+08	2.9E+08
1.06E+08	1.09E+08	1.23E+08	1.74E+08	1.64E+08	1.38E+08			
61202000	74358000	78702000	97964000	1.09E+08	1.08E+08	98132000	83821000	74834000
		30115000		23015000				
65409000	67136000			1.03E+08	1.21E+08	1.06E+08	92217000	1.11E+08
1.27E+09	1.53E+09	9.34E+08	8.78E+08	1.08E+09	1.27E+09	1.24E+09	1.24E+09	9.82E+08

	19568000	63766000	48236000	80624000				
80736000	75096000	73197000	88187000	70260000	90691000	1.66E+08	1.21E+08	53677000
6.38E+08	4.07E+08	1.03E+08	95089000	1.89E+08	3.36E+08	3.21E+08	2.77E+08	2.65E+08
90293000	1.04E+08	1.12E+08	94123000	1.35E+08	64533000	83591000	66743000	67519000
1.52E+08	1.54E+08	88805000	86984000	1.07E+08	1.98E+08	1.89E+08	1.84E+08	1.36E+08
69428000	87917000					71018000		
79939000	1.23E+08	4.43E+08	5.41E+08	4.82E+08	1.33E+08	1.78E+08	1.6E+08	2.69E+08
	99627000	2.39E+08	2.68E+08	2.22E+08		1.3E+08	1.02E+08	1.31E+08
4.42E+09	4.63E+09	5.42E+09	5.17E+09	7.67E+09	3.24E+09	3.57E+09	2.44E+09	2.92E+09
4.29E+08	7.29E+08	4.79E+08	4.05E+08	4.69E+08	3.78E+08	4.66E+08	2.51E+08	2.7E+08
1.73E+08	1.24E+08	95837000	1.06E+08	77999000	1.2E+08	1.58E+08	93507000	83050000
1.11E+08	85773000	91673000	1.01E+08	85519000	1.7E+08	1.65E+08	1.04E+08	1.27E+08
				1.51E+08				
		51053000	52765000	44385000				
4.77E+08	5.49E+08	4.56E+08	4.13E+08	2.68E+08	1.85E+09	1.25E+09	1.43E+09	9.61E+08
53323000	79751000	79309000	62181000	74162000	1.02E+08	84627000	1.26E+08	83361000
81220000	1.28E+08	83899000	1.44E+08	1.29E+08	1.45E+08	1.32E+08	1.48E+08	2.32E+08
1.34E+09	1.15E+09	1.28E+09	1.01E+09	9.51E+08	6.02E+08	5.59E+08	1.02E+09	1.32E+09
1.68E+08	1.87E+08	97868000	86252000	1.39E+08	1.11E+08	1.81E+08	1.2E+08	1.03E+08
1.35E+08	1.52E+08	1.96E+08	2.67E+08	2.05E+08	1.36E+08	1.25E+08	83449000	88654000
39484000	48789000	37350000	41037000	42432000		59332000		31023000
43924000	40374000			42138000				
45407000	50700000	30981000	39168000	43068000	72190000			36841000
84929000	82632000	1.17E+08	1.02E+08	82288000				
1.11E+09	1.22E+09	1.53E+09	1.32E+09	1.36E+09	1.58E+09	1.53E+09	1.36E+09	1.59E+09
1.67E+08	2.12E+08	87858000	88289000	1.17E+08				
	46354000	58448000	67865000	50722000				68235000
2.72E+08	2.43E+08	3.25E+08	3.11E+08	4.01E+08	2.37E+08	2.97E+08	2.38E+08	2.9E+08
64540000	71387000							
2.51E+08	2.37E+08	2.14E+08	2.68E+08	2.13E+08	2.19E+08	2.13E+08	1.96E+08	2.04E+08
		2.78E+08	1.71E+08	2.82E+08	43135000			
18585000	34699000	38821000	40222000	37734000	31252000	25393000	31831000	23319000
8.15E+08	1.53E+09	8.85E+08	9.61E+08	1.11E+09	2.34E+09	1.27E+09	1.5E+09	1.17E+09
19523000	18981000	79590000	69455000	94373000	27336000	19540000	25036000	26649000
	67238000	64700000	44294000	63522000	50375000	67616000	49141000	53236000
2.57E+08	3.03E+08	2.77E+08	2.74E+08	3.26E+08	2.86E+08	2.7E+08	2.88E+08	1.46E+08
					87805000			71985000
3.08E+08								

4.17E+08	4.77E+08	3.05E+08	3.17E+08	3.54E+08	3.43E+08	4.47E+08	3.41E+08	2.55E+08
1.46E+08	1.41E+08					2.47E+08		
		73594000		74736000				
2.74E+08	2.77E+08	3.24E+08	3.61E+08	3.23E+08	3.46E+08	2.85E+08	2.71E+08	2.31E+08
7.2E+08	7.5E+08	1.11E+09	1.01E+09	1.2E+09	7.25E+08	7.83E+08	5.1E+08	5.33E+08
3.09E+08	3.38E+08	5.17E+08	6.21E+08	5.26E+08	3.44E+08	4.28E+08	4.49E+08	4.79E+08
2.86E+08	3.73E+08	7.26E+08	7.34E+08	7.04E+08	4.03E+08	4.3E+08	3.88E+08	3.87E+08
	31404000	48598000		35376000				
1.53E+08	1.77E+08	1.36E+08	1.6E+08	1.54E+08	1.86E+08	2.06E+08	1.76E+08	1.82E+08
1.18E+08	1.46E+08	1.06E+08	1.51E+08	1.13E+08			31136000	81963000
					4.87E+08			4.45E+08
55458000	78373000	65190000	1.02E+08	92844000	61549000	76101000	42749000	45791000
9.17E+08	9.52E+08	5.09E+08	6.33E+08	6.81E+08	7.43E+08	1E+09	1.13E+09	7.84E+08
			31579000					
2.51E+08	2.2E+08	1.42E+08	1.17E+08		1.69E+08	3.01E+08	1.69E+08	2.26E+08
27801000	17558000			26670000				39169000
2.59E+08	2.03E+08	2.7E+08	2.32E+08	3.24E+08		3.79E+08	4.01E+08	2.71E+08
		1.24E+08	64054000	1.62E+08	57705000			94753000
4.06E+08	4.17E+08	1.08E+08	1.57E+08	1.41E+08	3.16E+08	4.24E+08	4.14E+08	4.56E+08
	96054000	66636000	49617000					
33436000	39116000	26129000	29783000	22100000	21261000	47057000	55023000	34849000
		24932000	21942000					
1.17E+08	67926000							
59958000	49242000	44082000		39159000	1.34E+08	94832000	1.2E+08	86926000
4.22E+08	4.1E+08	3.82E+08	3.19E+08	4.64E+08	3.59E+08	3.56E+08	3.78E+08	2.77E+08
75888000	56468000	46519000	46808000	57038000				56706000
				4915500				
		3.38E+08	3.25E+08	3.44E+08	1.68E+08	1.67E+08	1.31E+08	1.23E+08
4.3E+08	5.05E+08	53995000	68456000	85746000	3.25E+08	3.04E+08	2.59E+08	1.51E+08
		63024000	64139000	76400000	65796000	43542000		
25015000		40705000	31058000	46016000	25790000	26152000	26333000	42031000
3.74E+08	4.27E+08	4.09E+08	3.88E+08	3.58E+08	3.75E+08	4.09E+08	2.89E+08	2.71E+08
2.66E+08	3.91E+08	2.79E+08	3.75E+08	3.11E+08	3.72E+08	2.5E+08	2.69E+08	2.18E+08
1.04E+09	1.59E+09	1.29E+09	1.17E+09	2.09E+09	1.16E+09	9.6E+08	5.83E+08	3.39E+08
1.54E+09	1.59E+09	2.79E+09	1.92E+09	2.44E+09	1.89E+09	2.45E+09	1.88E+09	1.54E+09
2.46E+08	2.61E+08	1.91E+08	1.8E+08	2.01E+08	3.33E+08	3E+08	2.33E+08	1.83E+08
		33872000	44116000	39503000				
1.94E+08	1.78E+08	3.03E+08	3.26E+08	3.34E+08		2.88E+08		

		42952000	35063000					
70462000								
1.26E+08	1.32E+08	1.92E+08	2.42E+08	2.07E+08	1.45E+08	1.47E+08	1.19E+08	1.18E+08
3.01E+08	2E+08		3.08E+08					
6.18E+09	6.61E+09	4.24E+09	4.01E+09	3.99E+09	4.23E+09	4.07E+09	3.28E+09	2.75E+09
		3.58E+08	3.1E+08	3.87E+08				4.97E+08
	68837000	8.25E+08	8.26E+08	8.13E+08				6.34E+08
96409000	1.2E+08	88694000	97374000	1.08E+08	93051000	99022000	85667000	78925000
9.38E+08	1.02E+09	1.21E+09	1.35E+09	1.29E+09	1.42E+09	1.28E+09	1.24E+09	1.52E+09
5.27E+08	5.67E+08	5.43E+08	7.59E+08	6.62E+08	6.16E+08	7.54E+08	1.02E+09	1.09E+09
2.13E+08	2.12E+08	2.01E+08	1.44E+08	2.17E+08	1.67E+08	3.12E+08	3.25E+08	2.74E+08
39510000			29447000	24765000	40314000	47470000		39361000
		1.37E+08				5.51E+08		2.01E+08
	59918000		27227000	25433000	50165000	24760000		
40991000	38646000			16517000	33149000	29309000		37134000
67057000	1.05E+08	86844000	1.13E+08	1.06E+08	1.01E+08	1.16E+08	85297000	1.05E+08
98433000	1.26E+08	1.88E+08	2.42E+08	3.34E+08	1.27E+08	69342000	1.28E+08	1.12E+08
2.25E+08	2.43E+08	2.35E+08	1.52E+08	2.4E+08	2.77E+08	3.01E+08	2.79E+08	2.84E+08
10644000	14167000							
5.69E+08	8.25E+08	2.74E+08	3.42E+08	3.29E+08	6.45E+08	5.61E+08	4.57E+08	3.68E+08
2.38E+08	3.05E+08	1.59E+08	1.35E+08	1.88E+08	1.76E+08	1.85E+08	1.24E+08	1.11E+08
68177000	1E+08	81749000	64345000	75546000	87640000	89612000	92919000	60434000
2.2E+08	2.78E+08	2.34E+08	2.51E+08	2.51E+08	2.2E+08	2.41E+08	1.59E+08	1.65E+08
70078000	70178000	54811000	58862000	65804000				71902000
1.56E+08	1.85E+08	1.06E+08	92874000	1.11E+08	1.42E+08	1.5E+08	1.3E+08	90651000
3.57E+08	3.75E+08	2.7E+08	2.53E+08	2.87E+08	4.77E+08	4.49E+08	5.27E+08	3.83E+08
			1.03E+08		1.21E+08			
40342000	62757000	51103000	66996000	57482000	44271000	47384000	42744000	60571000
2.02E+09	2.16E+09	1.24E+09	1.52E+09	1.47E+09	2.1E+09	2.14E+09	1.97E+09	1.69E+09
1.89E+08	2.87E+08	1.44E+08	98529000	1.66E+08	4.13E+08	3.94E+08	4.73E+08	2.35E+08
	57433000	55932000	71936000		1.51E+08	94694000	1.14E+08	99588000
	35349000	62418000	66702000	55785000		37068000		25913000
1.57E+08	1.6E+08	4.82E+08	4.18E+08	4.69E+08		92507000		1.1E+08
3.49E+08	3.45E+08	2.93E+08	2.69E+08	2.26E+08	4.19E+08	4.62E+08	4.13E+08	3.67E+08
		1.21E+08						
1.36E+08	1.13E+08			53964000	92550000	1.03E+08	86464000	
57441000		40306000	51823000	59992000				
4.36E+08	4.02E+08	4.93E+08	8.32E+08	6.85E+08	3.56E+08	3.93E+08	1.99E+08	3.52E+08
		39579000	77345000	44846000				36313000
1.3E+09	1.27E+09	1.16E+09	1.16E+09	1.19E+09	1.35E+09	1.32E+09	1.39E+09	1.34E+09

80957000	2.93E+08	1.59E+08	1.18E+08	1.37E+08	2.99E+08	76347000	2.11E+08	1.71E+08
1.8E+08	2.27E+08	1.38E+08	1.43E+08	1.17E+08	3.2E+08	2.83E+08	3.72E+08	2.39E+08
18492000		29724000		29294000				
1.88E+08	1.85E+08	2.09E+08	1.87E+08	2.47E+08	1.84E+08	1.8E+08		1.4E+08
1.14E+09	9.52E+08	1.41E+09	1.34E+09	1.5E+09	2.25E+09	1.69E+09	1.57E+09	1.45E+09
2.28E+08	2.29E+08	1.6E+08	1.07E+08	1.12E+08	2.62E+08	3.05E+08	3.68E+08	2.72E+08
4.19E+08	4.03E+08	3.24E+08	2.9E+08	2.98E+08	3.47E+08	4.1E+08	3.12E+08	2.85E+08
2.99E+09	2.9E+09	4.12E+09	4E+09	4.17E+09	3.77E+09	3.81E+09	3.19E+09	3.1E+09
68436000	33856000	73757000	1.08E+08	78433000	92005000			
2.86E+08	4.16E+08	3.9E+08	3.37E+08	3.85E+08	3.51E+08	4.3E+08	3.28E+08	3.32E+08
5.06E+08	6.12E+08	9.4E+08	8.42E+08	9.53E+08	1.34E+09	8.98E+08		
		40922000	38060000	41782000		20552000		21084000
1.49E+08	1.52E+08	2.11E+08	1.64E+08	1.39E+08	2.24E+08	3.31E+08	2.5E+08	1.37E+08
35807000	46634000	41883000		42048000	78536000			
	23835000	70455000	1.04E+08	74871000				50498000
		71544000	36432000	37746000				
78514000	83177000		91465000	47391000	1.02E+08	1.1E+08	1.25E+08	88564000
9.25E+08	8.86E+08	4.58E+08		4.84E+08		5.38E+08		6.74E+08
2.23E+08	2.65E+08	2.98E+08	3.47E+08	2.45E+08		2.92E+08		
		58117000	60248000	66585000				
	34832000		24676000	22220000				
7.14E+08	7.46E+08	2.92E+08		2.37E+08	8.41E+08	5.08E+08	5.16E+08	4.51E+08
48598000	77441000	46675000	64334000	58543000	63948000	81590000	62669000	63000000
5.84E+08	5.41E+08	3.44E+08	3.65E+08	3.44E+08	5.49E+08	5.39E+08	4.37E+08	4.43E+08
7.91E+08	6.9E+08	4.78E+08	4.77E+08	3.29E+08	7.86E+08	6.79E+08	6.91E+08	4.1E+08
				63266000				
93358000	66270000	63873000		58069000	45592000	70686000	62332000	36922000
1.48E+08	1.77E+08	1.18E+08	1.29E+08	1.17E+08	1.99E+08	2.27E+08	2.1E+08	1.54E+08
90321000	80288000	2.19E+08		2.41E+08	1.36E+08	1.46E+08	1.48E+08	1.1E+08
98198000	1.26E+08	1.42E+08	1.74E+08	1.67E+08	1.15E+08	80712000	1E+08	
1.58E+08	1.56E+08				1.54E+08		1.25E+08	
1.31E+08	1.3E+08	1.75E+08	1.4E+08	1.5E+08	1.96E+08	1.66E+08	1.66E+08	1.33E+08
3.15E+08	4.04E+08	7.59E+08	6.97E+08	6.29E+08	4.63E+08	2.16E+08	3.21E+08	4.14E+08
22859000				23031000				
		3.24E+08						
	1.76E+08	47448000	80207000	58806000			1.75E+08	
2.14E+09	2.27E+09	4.86E+08	4.57E+08	5.33E+08	1.93E+09	2.13E+09	1.79E+09	1.71E+09
							2.12E+08	1.76E+08

1.45E+08	1.85E+08	1.63E+08	2.14E+08	3E+08	1.55E+08	1.89E+08	89959000	1.75E+08
67093000	65674000	98892000	80072000	99989000			1.01E+08	76576000
5.66E+08	4.4E+08	4.44E+08		5.12E+08	4.27E+08	4.32E+08	3.75E+08	4.35E+08
4.95E+08	4.78E+08	7E+08	4.95E+08	4.26E+08	2.18E+08	1.26E+08	2.04E+08	1.39E+08
97839000		84101000	92274000	86473000		1.37E+08		
		2.36E+08	1.77E+08	2.27E+08				
7.76E+08	8.46E+08	6.62E+08	4.98E+08	6.46E+08	6.62E+08	7.8E+08	4.98E+08	4.43E+08
5.19E+08	7.47E+08	9.51E+08	1.02E+09	1.04E+09	6.99E+08	7.5E+08	7.52E+08	7.31E+08
5.54E+08	7.2E+08	1.08E+09	1.03E+09	9.52E+08	7.12E+08	8.58E+08	5.68E+08	6.35E+08
7.94E+08	9.25E+08	6.23E+08	4.64E+08	6.86E+08	9.22E+08	9.43E+08	6.54E+08	4.97E+08
5.29E+08	4.42E+08	5.73E+08	4.82E+08	5.53E+08	3.69E+08	2.48E+08	2.31E+08	3E+08
		71427000					57791000	
43486000	53351000	45828000	64121000	79688000	44108000	48896000	29260000	
1.16E+08	1.02E+08	80881000		82822000	89989000	1.14E+08		
5.36E+08	6.09E+08	4.46E+08	8.44E+08	6.32E+08	7.89E+08	6.25E+08	1.01E+09	8.64E+08
89190000	1.43E+08	73414000		73997000	1.36E+08	1.33E+08	99069000	64826000
54971000	55954000	1.26E+08	1.47E+08	1.17E+08	59487000	62583000	46116000	57038000
83441000	1.01E+08	1.36E+08	1.68E+08	1.62E+08	1.56E+08	1.21E+08	1.31E+08	1.45E+08
1.75E+09	2.37E+09	1.12E+09	1.02E+09	9.69E+08	1.84E+09	2.57E+09	1.06E+09	1.03E+09
2.1E+08		1.71E+08	2.25E+08	2E+08	2.62E+08		2.08E+08	1.49E+08
				44939000				
1.21E+09	1.03E+09	2.61E+09	2.9E+09	2.46E+09	1.61E+09	1.69E+09	1.58E+09	2.39E+09
5.82E+08	6.24E+08	3.1E+08	3.23E+08	3.07E+08	7.07E+08	7.53E+08	4.88E+08	5.12E+08
1.36E+08	1.63E+08	99284000	1.17E+08	1.2E+08	1.82E+08	1.61E+08	1.63E+08	1.05E+08
2.85E+08	3.52E+08	2.31E+08	2.06E+08	2.12E+08	3.57E+08	4E+08	4.29E+08	3.14E+08
1.14E+08	1.31E+08	2.12E+08	1.89E+08	2.17E+08	1.59E+08	1.68E+08	1.39E+08	1.48E+08
4.9E+08	6.25E+08	3.24E+08	2.68E+08	2.57E+08	7.3E+08	6.9E+08	6.98E+08	4.55E+08
5.99E+08	5.55E+08	1.42E+09	1.26E+09	1.31E+09	5.46E+08	6.06E+08	5.63E+08	7.66E+08
1.09E+08	1.2E+08	1.53E+08	1.9E+08	1.46E+08	99846000	1.19E+08	95778000	92569000
4.94E+08	6.24E+08	2.12E+08	3.11E+08	2.85E+08	6.79E+08	6.69E+08	7.63E+08	5.62E+08
							1.13E+08	
1.77E+08	2.37E+08		97276000		2.19E+08	2.37E+08		1.88E+08
29248000	39187000	39554000	40501000	65917000				
67405000		88575000	60270000	99558000				92452000
2.42E+08	2.37E+08	3.17E+08	2.55E+08	1.87E+08	2.1E+08	1.47E+08	2.05E+08	2.16E+08
4.12E+08	3.93E+08	2.66E+08	1.81E+08	2.6E+08	4.49E+08	4.1E+08	4.19E+08	2.29E+08
5.23E+08	5E+08	6.97E+08	7.82E+08	7.49E+08	4.55E+08	4.92E+08	5.16E+08	4.07E+08
8.09E+08	7.81E+08	8.9E+08	1.11E+09	9.48E+08	5.43E+08	7.32E+08	9.1E+08	7.37E+08
1.03E+08	87910000	2.83E+08	2.52E+08	3E+08			1.51E+08	1.23E+08
2.62E+08	3.43E+08	2.57E+08	2.01E+08	2.08E+08	2.78E+08	2.25E+08		2.22E+08
46485000	86505000	59295000	94095000	82720000	58714000			
		2.56E+08	2.94E+08	2.22E+08				
2.51E+08	2.69E+08	2.84E+08	4.1E+08	2.93E+08	2.91E+08	3.59E+08	3.78E+08	2.89E+08
2.79E+08	4.36E+08	1.99E+08	1.82E+08	2.11E+08	4.54E+08	5E+08	4.52E+08	4.51E+08

4.88E+08	6.23E+08	5.43E+08	6.13E+08	5.73E+08	6.05E+08	6.25E+08	6.88E+08	5.61E+08
	34084000	33793000						
	53946000	42561000	56641000	40544000	57996000	55199000	64424000	42560000
1.55E+08	1.82E+08	3.36E+08	2.89E+08	2.97E+08	2.28E+08	2.1E+08	2.14E+08	1.74E+08
1.52E+09	1.65E+09	2.93E+09	3.28E+09	3.26E+09	2.24E+09	2.06E+09	2.08E+09	2.07E+09
5E+08	4.84E+08	4.5E+08	3.89E+08	4.91E+08	3.44E+08	3.65E+08	2.65E+08	2.56E+08
76616000	57864000	80943000		71828000				
4.74E+08	5.52E+08	4.43E+08	3.92E+08	7.04E+08	4.79E+08	5.45E+08	4.17E+08	3.38E+08
29325000	35029000	45345000		39651000				
8.02E+08	6.52E+08	1.15E+09	1.01E+09	1.33E+09	6.32E+08	5.38E+08	5.77E+08	5.84E+08
88725000	78593000				58715000			
2.94E+08	2.22E+08	2.38E+08	2.94E+08	3.51E+08	1.76E+08		1.59E+08	1.98E+08
1.39E+09	1.8E+09	7.35E+08	6.66E+08	8.62E+08	1.5E+09	1.48E+09	1.17E+09	9.66E+08
	60431000				61110000	61944000	64352000	
78656000	1.05E+08	1.11E+08	96222000	1.09E+08	1.47E+08	1.61E+08	1.69E+08	1.18E+08
41912000	45229000	33646000		40353000		43991000		
		1.6E+08		1.69E+08				
13062000	24506000	39439000	51036000	45162000				33858000
	24520000	31525000	31356000	27861000		37749000		
		50027000	84056000	51165000	62251000	34024000		
1.6E+08	48535000	66910000						
51511000	65572000				62116000			
		46037000	42916000	49841000				
			50324000					
2.49E+08		4.12E+08	4.72E+08	3.91E+08				
1.56E+08	1.75E+08	1.27E+08	1.52E+08	1.11E+08	2.27E+08	1.77E+08	2.14E+08	1.04E+08
1.85E+09	1.97E+09	1.54E+09	1.59E+09	1.48E+09	1.39E+09	1.42E+09	1.83E+09	1.11E+09
					80843000	75563000	87302000	50645000
4.08E+08	4.5E+08	7.4E+08	6.73E+08	6.6E+08	5.69E+08	6.13E+08	4.5E+08	5.3E+08
3.55E+08	3.93E+08	4.14E+08	3.88E+08	3.76E+08	3.78E+08	3.84E+08	3.28E+08	2.25E+08
3.32E+08	3.74E+08	4.77E+08	5.56E+08	5.35E+08	4.02E+08	3.72E+08	3.61E+08	2.4E+08
6.36E+09	4.55E+09	2.75E+09	2.14E+09	2.37E+09	3.19E+09	3.54E+09	2.22E+09	4.08E+09
1.86E+08	2.57E+08	36873000	71188000	61487000	1.25E+08	1.19E+08	1.01E+08	66462000
9.99E+08	9.33E+08	2.28E+08	1.94E+08	1.96E+08	6.01E+08	8.88E+08	6.83E+08	5.67E+08
84411000	70603000	30298000	28731000	30628000	84148000	75763000		58306000
	3.7E+08	1.73E+08	1.68E+08	1.84E+08	2.67E+08	4.05E+08		1.8E+08
6.32E+08	6.96E+08	1.06E+09	9.76E+08	9.76E+08	9.29E+08	9.99E+08	7.83E+08	9.35E+08
3.36E+08	3.87E+08	1.14E+09	1.04E+09	1.06E+09	3.21E+08	5.75E+08	3.24E+08	4.65E+08
1.34E+09	1.5E+09	1.68E+09	1.75E+09	1.48E+09	1.57E+09	1.35E+09	1.43E+09	1.48E+09
1.65E+08	1.94E+08	2.42E+08	2.22E+08	2.43E+08	2.22E+08	2.22E+08	1.5E+08	1.62E+08
3.86E+08	3.05E+08	1.78E+08	2.67E+08	1.89E+08	1.34E+08	1.14E+08	3.32E+08	6.45E+08
3.42E+09	2.96E+09	1.46E+09	1.67E+09	2.08E+09	2.28E+09	2.54E+09	1.55E+09	1.85E+09

89224000	84590000	90410000	90367000	97238000	64779000	75914000		66428000
1.03E+08	1.32E+08	54611000	66051000	52948000	1.74E+08	1.71E+08	2.89E+08	1.35E+08
4.46E+08	4.79E+08	2.79E+08	4.5E+08	3.36E+08	4.99E+08	5.35E+08	5.05E+08	4.26E+08
		62331000	39375000	43554000				
36466000	50754000	53984000	69523000	52036000	58777000	57816000	67181000	52524000
	27292000	76278000		97482000				41175000
5.01E+09	4.27E+09	2.14E+09	1.55E+09	1.68E+09	4.06E+09	5.18E+09	5.34E+09	3.94E+09
			47312000					
1.66E+08	1.82E+08	1.67E+08	1.56E+08	1.48E+08				2.95E+08
90475000	74864000	70159000	85627000	66120000	76551000	98161000	63062000	28774000
3.95E+08	5.06E+08	4.62E+08	5.26E+08	5.99E+08	3.73E+08	3.31E+08	3.4E+08	2.67E+08
30614000	25996000	29466000		28335000				39383000
82021000	1.18E+08	68489000	1.06E+08	88834000	95976000	90047000	77491000	1.05E+08
1.96E+08	2.06E+08	1.91E+08	2.3E+08	2.22E+08	3.55E+08	3.97E+08	4.71E+08	4.46E+08
3.34E+08	3.44E+08	2.5E+08	2.33E+08	2.37E+08	4.2E+08	3.47E+08	2.76E+08	1.96E+08
6.79E+08	7.79E+08	2.01E+08	2.16E+08	2.36E+08	8.13E+08	7.44E+08	6.37E+08	4.97E+08
1.1E+08	1.28E+08	55228000					2.03E+08	1.45E+08
3.44E+08								
40612000	42548000	23169000	21362000	19597000	48508000	45603000	32540000	40107000
1.67E+08	1.5E+08				1.68E+08	1.66E+08	1.85E+08	
7.06E+08	7.58E+08	2.99E+08	2.66E+08	2.84E+08	7.02E+08	7.38E+08	8.43E+08	5.72E+08
6.33E+08	6.53E+08	3.06E+08	2.92E+08	2.66E+08	6.66E+08	6.51E+08	5.17E+08	4.59E+08
	61672000	54028000			1.31E+08	1.48E+08	1.54E+08	1.33E+08
		80708000						
3.73E+08	2.79E+08	2.41E+08	3.33E+08	2.51E+08	2.41E+08	2.31E+08	2.1E+08	2.88E+08
3.16E+08	2.99E+08	2.05E+08	1.86E+08	1.68E+08	3.33E+08	4.42E+08	5.68E+08	2.77E+08
8.26E+08	5.31E+08	6.02E+08	4.87E+08	4.93E+08	5.97E+08	6.01E+08	5.47E+08	5.8E+08
35891000				49874000				
2.03E+08	2.52E+08	1.81E+08	1.94E+08	1.27E+08	2.61E+08	2.95E+08	1.96E+08	2.25E+08
8.39E+08	8.74E+08	1.02E+09	9.33E+08	8.82E+08	9.03E+08	9.91E+08	7.95E+08	6.68E+08
6.77E+08	3.24E+08	1.18E+08	91459000	1.09E+08	1.88E+08	1.93E+08	1.35E+08	2.18E+08
4.35E+08	5.17E+08	2.15E+08	1.76E+08	3.15E+08	3.34E+08	3.66E+08	2.98E+08	2.4E+08
2.15E+08	2.21E+08	1.61E+08	1.81E+08	1.7E+08	3.11E+08	3.56E+08	2.75E+08	2.45E+08
83649000	1.74E+08	1.31E+08	1.66E+08	1.28E+08	1.93E+08	1.1E+08	59362000	45222000
1.36E+08	1.38E+08	1.67E+08	1.08E+08	80799000				
1.65E+08	1.76E+08	84555000	1.17E+08	1E+08	1.81E+08	1.71E+08	1.63E+08	1.48E+08
85082000	99491000	46085000	47843000	48988000	67268000	90491000	1E+08	49665000
3.13E+08	4.08E+08	1.78E+08	2.27E+08	2.16E+08	2.95E+08	3.69E+08	4.97E+08	2.81E+08
1.81E+08	2.05E+08	1.07E+08	93275000	1.52E+08	1.52E+08		1.53E+08	1.47E+08

		1.41E+08	1.66E+08	1.52E+08	1.84E+08		1.71E+08	
83571000	70924000	46713000	35352000	49038000	99073000	96267000	1.63E+08	97527000
71814000		84353000	1.13E+08		92683000			1.01E+08
5.75E+08	5.87E+08	6.32E+08	7.27E+08	8.42E+08	6.75E+08	7.34E+08	1.05E+09	5.36E+08
36570000	56124000	37765000		48007000	2.15E+08	2.11E+08	2.49E+08	4.87E+08
		6.32E+08	7.23E+08	6.73E+08	6.45E+08	6.72E+08	1.62E+09	9.65E+08
2.64E+08		2.04E+08	3.18E+08	1.87E+08	3.73E+08	3.86E+08	3.26E+08	3.44E+08
5.65E+08	5.33E+08	5.71E+08	5.09E+08	5.59E+08	6.17E+08	7.14E+08	6.21E+08	1.11E+09
39755000	40064000			29956000	55765000			
69137000	58764000	33067000	41526000	41516000	58116000	66496000	52998000	42376000
	3.1E+08	1.89E+08	2.27E+08	2.14E+08	1.49E+08			
1.17E+08								
7.47E+09	8.3E+09	2.66E+09	2.34E+09	2.23E+09	7.42E+09	9.3E+09	8.86E+09	6.97E+09
4.55E+08	4.05E+08	1.89E+08	2.31E+08	1.69E+08	6.41E+08	6.5E+08	6.45E+08	4.02E+08
2.84E+08	2.8E+08	1.28E+08	1.13E+08	1.1E+08	3.71E+08	5.24E+08	3.36E+08	2.91E+08
3.46E+08	3.57E+08	2.57E+08	2.32E+08	2.99E+08	2.49E+08	2.45E+08	2.63E+08	3.26E+08
2.91E+08	3.09E+08	5.66E+08	5.45E+08	6E+08	5.66E+08	6.03E+08	4.66E+08	4.99E+08
3.87E+08	4.38E+08	2.8E+08	2.43E+08	2.96E+08	7.89E+08	7.34E+08	1.07E+09	7.29E+08
76268000	84232000		52848000		90075000	92389000		97486000
5.79E+08	6.86E+08	5.53E+08	5.28E+08	6.51E+08	7.72E+08	8.27E+08	5.66E+08	6.76E+08
90310000	92288000	54249000	75547000	53213000		85874000	72874000	
1.37E+08	82143000							
1.79E+08	2.02E+08	81538000	87439000	62463000	2.3E+08	2.04E+08	2.29E+08	1.75E+08
				23983000				
34629000	48276000				44474000	52566000	43800000	30775000
3.72E+08	4.08E+08	2.8E+08	2.54E+08	2.55E+08	3.52E+08	3.35E+08	2.69E+08	2.23E+08
2.04E+08	2.69E+08	1.65E+08	1.59E+08	1.53E+08	3.63E+08	2.95E+08	3.12E+08	2.77E+08
5.47E+08		3.56E+08		3.42E+08	4.36E+08	5.56E+08	3.69E+08	3.83E+08
		3.18E+08	1.79E+08	1.52E+08				
		1.65E+08	1.59E+08	1.89E+08				
90662000	1.09E+08	1.02E+08	1.15E+08	98798000	84463000	79287000	92333000	1.25E+08
7.34E+08	8.15E+08	4.05E+08	4.64E+08	5.51E+08	6.75E+08	6.89E+08	7.13E+08	5.76E+08
		3.16E+08		2.8E+08			3.28E+08	
7.06E+08	8.44E+08	6.37E+08	8.6E+08	8.91E+08	1.04E+09	8.03E+08	9.89E+08	9.03E+08
1.72E+08	1.66E+08	1.03E+08	97997000	95572000	1.77E+08	2.23E+08	2.44E+08	1.78E+08

		67371000	1.1E+08	80207000				
55696000	55502000	49915000	44086000	46300000		38109000		43237000
85693000	71797000	94017000	79759000	83578000	78130000	77414000	87023000	74639000
1.6E+08	2.21E+08	2.33E+08	2.3E+08	2.7E+08			2.23E+08	
78267000	89839000	2.68E+08	2.03E+08	2.03E+08			1.17E+08	1.55E+08
	44049000	40566000	37925000	31574000	97394000	76200000	86312000	51370000
	96048000	55365000	53501000	63731000				
1.88E+08	1.78E+08	2.46E+08	2.59E+08	2.53E+08	2.12E+08	1.99E+08	2.21E+08	1.5E+08
42417000	43990000	35344000		53500000	62281000	63566000		
98956000	1.08E+08	1.69E+08	1.43E+08	1.9E+08	1.15E+08	1.12E+08	78003000	78734000
1.02E+08	1.39E+08	3.67E+08	3.12E+08	3.39E+08	89462000	56720000	60399000	64006000
						44337000		
		34592000	27858000	23414000				
	49892000	41996000		38777000				
56590000		52116000				73539000		
4.33E+08	4.48E+08	3.1E+08	3.03E+08	3.31E+08	1.56E+08	2.11E+08	2.71E+08	2.56E+08
3.9E+08	3.59E+08	1.32E+09	9.83E+08	1.3E+09	2.9E+08	3.31E+08	2.2E+08	2.71E+08
1.98E+09	1.93E+09	6.88E+09	5.2E+09	5.2E+09	2.7E+09	3.07E+09	2.03E+09	2.09E+09
		20346000		20579000				
3.15E+09	2.91E+09	3.74E+09	3.68E+09	3.39E+09	2.89E+09	2.71E+09	3.27E+09	2.26E+09
4.66E+08	4.6E+08	1.54E+08	1.65E+08	2.27E+08	3.12E+08	3.84E+08	2.77E+08	2.51E+08
			24912000					
4.42E+08	6.73E+08	1.91E+09	1.92E+09	1.97E+09	6.4E+08	5.4E+08	4.79E+08	7.85E+08
		1.95E+08	1.57E+08	1.99E+08				
		65481000	86372000	71961000				1.69E+08
2.03E+08	1.74E+08	2.26E+08	1.8E+08	1.94E+08	1.33E+08	1.6E+08	1.54E+08	2.49E+08
9.43E+08	9.69E+08	4.58E+08	5.18E+08	5.22E+08	1E+09	9.54E+08	1.07E+09	8.59E+08
2.09E+08	1.9E+08	1.53E+08	1.05E+08	96347000	1.92E+08	2.09E+08	2.05E+08	1.36E+08
78157000		1.27E+08	99042000	88214000	1.45E+08	1.27E+08	1.18E+08	1.16E+08
1.29E+08	1.26E+08		1.09E+08	1.39E+08	89006000	1.03E+08	1E+08	85179000
2.58E+08	4.78E+08							
	2.73E+08	3.52E+08	5E+08	3.55E+08				
		1.11E+08		1.19E+08				
50729000	58012000	90795000	1.31E+08	97013000	71029000		53125000	
	64254000	6.04E+09	3.67E+09	1.82E+10			76482000	
4.98E+08	4.15E+08	1.15E+08	1.25E+08	85264000	1.68E+08	1.91E+08	1.8E+08	1.85E+08
		4.01E+09	9.97E+09	7.14E+09				
		5.72E+08	5.65E+08	3.7E+08				
1.44E+08	1.72E+08	2.03E+08	2.12E+08	2.05E+08	2.1E+08	1.9E+08	1.86E+08	1.54E+08
95834000	88332000	1.08E+08	92006000	1.04E+08	99798000	91420000	1.44E+08	71919000
1.45E+08		1.95E+08	2.07E+08	1.99E+08			1.06E+08	

		20626000	22439000	17250000				
		27588000	43618000	39489000				
55939000	70640000	91900000	85486000	87090000	82444000	65300000	70065000	64912000
			15346000	14431000				
7.59E+08	9.46E+08	6.89E+08	7.88E+08	8.04E+08	7.9E+08	6.98E+08	5.72E+08	7.53E+08
3.16E+08	1.88E+08	3.53E+09	1.65E+09	3.96E+09	7.76E+08		5.08E+08	5.88E+08
		2.62E+08	1.72E+08	2.66E+08	40114000			1.15E+08
90646000	96338000	60482000	70264000	84644000	81799000	1.04E+08	73191000	75057000
16292000				18860000				
2.37E+08	4.82E+08	6.88E+08	6.74E+08	8.44E+08	2.91E+08	1.82E+08	2.57E+08	1.39E+08
5.13E+08	5.23E+08	7.97E+08	7.15E+08	8.68E+08	4.29E+08	4.89E+08	4.01E+08	3.43E+08
		2.7E+08	3.48E+08					
		1.14E+08	1.17E+08	1.16E+08				
	1.39E+08	92672000	1.08E+08	1.04E+08				95914000
2.76E+08	3.57E+08	1.68E+08	1.95E+08	1.92E+08	2.69E+08	2.98E+08	2.46E+08	2E+08
72700000	74083000							
1.13E+08	1.14E+08	98682000	1.17E+08	93626000	1.14E+08	1.19E+08		1.05E+08
								39955000
4.56E+08	4.56E+08	5.67E+08	5.75E+08	5.35E+08	6.36E+08	6.55E+08	7.15E+08	9.23E+08
68353000	1.47E+08	2.81E+08	7.06E+08	52499000				
65159000		1.77E+08	1.89E+08	1.93E+08			1.06E+08	56651000
87045000	1.13E+08	2.81E+08	2.93E+08	2.91E+08	65761000	61999000	44197000	66408000
1.26E+08	45690000	1.21E+09	1.08E+09	1.1E+09				85793000
94822000	94984000				64690000		95161000	
5.77E+08	6.94E+08	3.53E+08	3.42E+08	2.94E+08	5.43E+08	8E+08	7.49E+08	6.3E+08
94097000	1.34E+08	1.97E+08	2.05E+08	2.4E+08	1.09E+08	1.15E+08		97075000
2.13E+08	2.08E+08	4.53E+08	3.45E+08	2.86E+08	1.79E+08	1.73E+08	1.93E+08	1.84E+08
1.33E+08	1.4E+08	1.47E+08	2.1E+08	1.6E+08	1.21E+08	1.19E+08	1.05E+08	1.08E+08
	62189000	46411000						
1.43E+08	1.42E+08	98934000	1.32E+08	1.06E+08	1.9E+08	1.49E+08	1.98E+08	1.17E+08
1.23E+08								1.58E+08
		66001000	1.1E+08	68919000				
75212000	83569000	36964000	62498000	36228000		71136000		45980000
		4.07E+08	2.58E+08	3.79E+08				1.35E+08
1.39E+08	1.29E+08	2.17E+09	1.57E+09	2.4E+09	2.28E+08	2.62E+08	1.86E+08	1.08E+09
		1.17E+08	1.37E+08	1.14E+08				98394000
2.24E+08	2.79E+08	2.32E+08	2.33E+08	2.08E+08				2.66E+08
23716000	41964000	1.08E+08	85345000	1.12E+08	69899000	61004000	32578000	49056000
3.97E+09	3.36E+09	2.92E+09	1.68E+09	3.07E+09	2.66E+09	3.39E+09	2.62E+09	2.17E+09

2.58E+08	2.97E+08	3.71E+08	4.15E+08	4.96E+08	2.38E+08			2.17E+08

LFQ intens	LFQ intens	LFQ intens	LFQ intens	LFQ intens	C: Only ide	C: Reverse	C: Potenti	N: Peptide
8.27E+08	7.19E+08	1.24E+09	1.21E+09	8.88E+08				10
6.09E+08	7.71E+08	6.17E+08	5.22E+08	5.09E+08				10
5.48E+08	5.58E+08	5.92E+08	5.53E+08	6.93E+08				16
2.7E+08	1.9E+08	2.04E+08	2.17E+08	1.44E+08				15
		49905000	81588000	60030000				4
1.18E+10	1.27E+10	1.39E+10	1.52E+10	1.27E+10				19
								3
4.86E+08	5.13E+08	5.45E+08	5.78E+08	5.41E+08				14
3.97E+08	4.36E+08	3.39E+08	3.11E+08	3.34E+08				10
								2
3.86E+08	2.53E+08	3.95E+08	3.92E+08	6.42E+08				5
8.24E+08	1.15E+09	1.71E+09	1.1E+09	2.55E+09				9
2.02E+10	2.01E+10	2.57E+10	3.23E+10	2.53E+10				53
82598000	59397000		59786000	71361000				5
5.45E+08	4.84E+08	6.22E+08	6.56E+08	6.3E+08				5
2.78E+08	2.49E+08	3.29E+08	3.5E+08	3.79E+08				23
3.23E+09	2.91E+09	9.03E+09	9.39E+09	9.06E+09				10
8.57E+08	7.94E+08	1.11E+09	9.97E+08	9.31E+08				15
								6
1.32E+10	1.12E+10	1.23E+10	1.33E+10	9.61E+09				25
8.14E+09	9.18E+09	1.05E+10	8.54E+09	1.17E+10				20
6.55E+09	5.89E+09	7.85E+09	7.44E+09	7.88E+09				17
2.85E+08	2.58E+08	4.58E+08	3.47E+08	3.45E+08				8
1.07E+08	77424000	1.88E+08	1.69E+08	1.64E+08				11
1.12E+08	1.3E+08			1.04E+08				3
								3
								2
		86643000	60421000	64442000				3
								2
8.4E+08	9.83E+08	1.1E+09	8.06E+08	9.26E+08				10
								1
96700000	1.08E+08		1.4E+08	1.54E+08				3
7.6E+09	8.62E+09	1.15E+10	1.41E+10	1.04E+10				62
2.21E+09	2.23E+09	2.42E+09	2.56E+09	2.63E+09				14
1.07E+08		77773000	86320000	1.15E+08				3
4.55E+08	5.08E+08	8.12E+08	9.05E+08	9.94E+08				15
3.13E+08	3.55E+08	3.63E+08	3.77E+08	3.71E+08				6
2.38E+08	66786000	4.46E+08	1.93E+08	3.91E+08				7
								2
								3
1.12E+09	1.01E+09	1.05E+09	1.41E+09	1.26E+09				3
89808000	1.04E+08	67310000	88017000	65421000				6
9.78E+08	8.72E+08	9.12E+08	1.5E+09	9.87E+08				5
4.85E+08	4.09E+08	4.22E+08	4.65E+08	4.54E+08				11
2.59E+08	2.66E+08	3.11E+08	3.42E+08	4E+08				7
1.23E+08	1.69E+08	1.16E+08	1.28E+08	1.44E+08				3
4.18E+08	5.15E+08	3.12E+08	4.03E+08	3.52E+08				13
1.97E+08	2.13E+08		2.01E+08	1.48E+08				6
	3.75E+08		2.96E+08	3.51E+08				4

	2.74E+08	3.21E+08			5
1.13E+08	1.26E+08	1.55E+08	1.12E+08	88677000	5
1.11E+09	9.45E+08	1.26E+09	1.37E+09	1.26E+09	9
1.28E+09	1.69E+09	8.12E+08	8.3E+08	1.29E+09	4
65134000	28105000	46134000	57950000	76238000	4
4.96E+08	5.33E+08	4.45E+08	4.62E+08	5.08E+08	5
					1
					1
1.68E+09	1.9E+09	1.63E+09	1.56E+09	1.72E+09	17
3.88E+08	3.24E+08	3.91E+08	3.16E+08	3.21E+08	10
2.32E+08	2E+08	1.66E+08	1.87E+08	2.7E+08	8
					1
11351000	10636000				1
6.87E+08	8.29E+08	1.26E+09	8.46E+08	6.58E+08	11
1.2E+09	1.1E+09	1.4E+09	1.48E+09	1.33E+09	13
1.06E+09	1.18E+09	1.51E+09	1.16E+09	1.27E+09	17
1.89E+08	1.69E+08	1.28E+08	1.52E+08	1.78E+08	5
2.75E+08	2.84E+08	3.08E+08	2.99E+08	3.89E+08	4
8.64E+09	1E+10	7.31E+09	6.51E+09	7.74E+09	8
6.53E+08	6.47E+08	6.56E+08	5.72E+08	6.8E+08	15
85610000				1.33E+08	5
1.42E+09	1.14E+09	1.14E+09	1.3E+09	1.31E+09	10
8.04E+08	7.75E+08	7.84E+08	5.52E+08	5.39E+08	8
2.32E+08	2.04E+08	2.61E+08	2.4E+08	2.36E+08	7
1.65E+09	1.86E+09	2.06E+09	2.05E+09	1.89E+09	14
3.18E+08	3.09E+08	3.11E+08	2.92E+08	2.93E+08	6
92390000	1.27E+08			96554000	2
		1.46E+08	1.78E+08		3
2.07E+08	3.14E+08	2.53E+08	1.66E+08	2.19E+08	9
4.3E+08	4.14E+08	3.9E+08	4.26E+08	4.18E+08	7
14891000	24607000	45056000	9770200	12486000	3
1.74E+09	1.66E+09	1.99E+09	1.66E+09	1.82E+09	15
4.1E+09	3.42E+09	6.04E+09	7.67E+09	5.98E+09	9
1.04E+09	1.06E+09	1.54E+09	1.27E+09	1.7E+09	5
1.48E+09	1.47E+09	1.91E+09	2.01E+09	1.7E+09	10
7.37E+08	6.05E+08	7.11E+08	8.6E+08	9.64E+08	1
					2
2.96E+08	2.62E+08			2.7E+08	4
7.36E+09	8.09E+09	6.39E+09	8.02E+09	7.81E+09	10
81386000	1.03E+08	31264000	28231000	35829000	3
4.27E+09	5E+09	2.99E+09	4.65E+09	2.84E+09	9
2.55E+09	3.71E+09	7.43E+09	3.22E+09	4.39E+09	7
3.04E+09	2.54E+09	3.18E+09	3E+09	2.58E+09	14
5.22E+09	6.16E+09	5.82E+09	5.03E+09	6.01E+09	8
1.95E+09	1.49E+09	1.94E+09	1.86E+09	1.84E+09	1
2.89E+09	2.56E+09	4.39E+09	4.27E+09	3.86E+09	5
6.67E+08	5.82E+08	9.45E+08	1.08E+09	8.32E+08	10
2.08E+08	1.62E+08	1.84E+08	2.15E+08	2.13E+08	9
2.89E+08	5.5E+08	2.52E+08	5.2E+08	4.94E+08	15
95935000	67024000	75217000	1.33E+08	93811000	5
2E+09	1.88E+09	2.58E+09	2.36E+09	2.38E+09	16
8.26E+08	9.32E+08	7.66E+08	1.02E+09	9.94E+08	13
		26082000	18121000	25111000	5

					1
3E+09	2.2E+09	3.18E+09	3.23E+09	2.95E+09	7
1.72E+09	1.98E+09	2.48E+09	2.53E+09	2.69E+09	7
1.32E+09	1.02E+09	1.27E+09	1.64E+09	1.75E+09	9
38986000	28422000		31036000	37934000	6
48706000	66394000			42222000	3
					1
2.61E+08	3.81E+08	2.98E+08		2.81E+08	3
1.35E+09	9.93E+08	8.91E+08	8.44E+08	1.34E+09	7
1.8E+08	1.64E+08	2.49E+08	2.17E+08	1.91E+08	3
4.34E+09	3.83E+09	4.38E+09	4.69E+09	3.41E+09	5
1.76E+08		1.88E+08	1.85E+08	2.14E+08	2
					1
1.02E+09	9.38E+08	1.13E+09	1.14E+09	1.27E+09	14
		1.26E+08			3
1.05E+08		1.03E+08	90798000	51162000	5
5.71E+09	6.62E+09	6.2E+09	6.28E+09	6.34E+09	12
4.17E+08	3.56E+08	7.66E+08	4.25E+08	3.97E+08	9
1.73E+08	1.49E+08	2.09E+08	1.64E+08	1.75E+08	9
1.27E+10	1.35E+10	9.7E+09	1.01E+10	9.79E+09	3
1.49E+09	1.45E+09	1.51E+09	1.79E+09	1.98E+09	14
5.86E+08	6.4E+08	8.81E+08	8.2E+08	7.77E+08	10
1.27E+08	1.16E+08		1.38E+08	89835000	5
99427000					4
6.68E+08	5.67E+08	3.83E+08	3.39E+08	5.68E+08	11
1.4E+08	2.53E+08	71630000	1.11E+08	1.57E+08	3
3.14E+08	2.37E+08	4.15E+08	4.75E+08	4.22E+08	8
					1
68844000		73181000	75701000		5
2.63E+08	3.02E+08	2.94E+08	3.15E+08	2.51E+08	14
7.25E+08	7.18E+08	7.33E+08	9.42E+08	7.24E+08	5
23754000				22765000	3
1.24E+08	1.36E+08				2
1.48E+08	1.26E+08	1.47E+08	2.03E+08	1.48E+08	11
					1
2.04E+08	2.44E+08	1.03E+08	1.33E+08	1.63E+08	4
1.21E+09	1.01E+09	1.17E+09	1E+09	1.04E+09	15
1.15E+08	82370000	96172000	81498000	81851000	5
7.49E+08	5.5E+08	8.68E+08	9.11E+08	9.32E+08	13
	48338000				2
6.58E+08	4.1E+08	3.62E+08	5.1E+08	4.44E+08	10
3.76E+08	3.17E+08	3.72E+08	5.38E+08	4.9E+08	5
6.96E+08	3.56E+08			5.48E+08	7
1.6E+09	1.61E+09	1.29E+09	1.02E+09	1.04E+09	15
	37905000	62790000	66507000	46882000	4
1.21E+08			1.77E+08	1.66E+08	3
					3
					1
1.6E+08	1.23E+08	2.17E+08	2.11E+08	1.74E+08	10
					2
2.62E+09	2.3E+09	2.78E+09	2.95E+09	2.44E+09	23
8.55E+08	7.62E+08	1.02E+09	1.21E+09	1.07E+09	6
80357000	70896000	1.15E+08	1.04E+08	57241000	4

					3
4.12E+08	4.45E+08	7.59E+08	7.89E+08	6.89E+08	5
					2
	36182000	1.13E+08	1.28E+08	73620000	4
					1
72404000	51332000	48919000	53399000	60029000	3
24363000			13284000		5
1.35E+08	1.26E+08	1.36E+08	1.49E+08	1.33E+08	4
1.59E+08	1.27E+08	1.62E+08	1.93E+08	1.85E+08	7
4.42E+08	4.59E+08	1.11E+09	9.55E+08	8.18E+08	12
3.74E+09	3.01E+09	3.31E+09	2.67E+09	3.52E+09	32
	24483000			38499000	5
1.55E+08	1.27E+08	1.43E+08	2.23E+08	1.28E+08	4
2.61E+08	2.22E+08	1.97E+08	2.41E+08	2.77E+08	5
1.2E+08	80159000	85284000	1.09E+08	1.68E+08	6
3.04E+08	2.63E+08	2.62E+08	3.89E+08	3.81E+08	10
3.33E+09	3.37E+09	2E+09	2.65E+09	3.62E+09	12
1.15E+10	1.02E+10	6.47E+09	8.92E+09	8.18E+09	23
1.86E+08	2E+08	1.93E+08	1.63E+08	2.31E+08	5
1.73E+09	1.57E+09	1.98E+09	1.57E+09	1.75E+09	6
3.14E+08	3.21E+08	2.6E+08	1.94E+08	3.17E+08	7
				1.55E+08	2
2.9E+08	4.23E+08		2.48E+08		4
27367000	31596000	13907000		23037000	5
					3
5.47E+08	4.37E+08	5.64E+08	5.91E+08	6.22E+08	10
85306000	93318000	93416000	1.1E+08	83511000	5
1.36E+09	9.09E+08	1.97E+09	1.95E+09	8.7E+08	15
5.76E+08	5.78E+08	7.22E+08	6.04E+08	5.33E+08	4
		71673000		93530000	5
6.05E+08	3.82E+08	2.53E+08	1.32E+08	2.09E+08	17
1.84E+08	1.59E+08	1.76E+08	1.82E+08	1.51E+08	4
4.98E+08	4.54E+08	5.46E+08	5.55E+08	5.8E+08	19
1.66E+08	1.63E+08	67776000	1.09E+08	1.21E+08	8
2.45E+08	2.21E+08	2.57E+08	2.36E+08	2.32E+08	5
4.26E+08	4.03E+08		4.2E+08	4.43E+08	3
		54197000			3
40681000	46693000	41034000	36618000	59889000	5
7.72E+08	6.62E+08	7.29E+08	5.46E+08	6.51E+08	4
5.83E+08	5.33E+08	4.52E+08	4.85E+08	4.34E+08	3
41928000		37187000	49674000		3
					3
3.64E+08	6.08E+08	2.04E+08	2.17E+08	2.5E+08	4
1.23E+09	1.08E+09	1.2E+09	1.18E+09	1.01E+09	15
5.65E+08	5.61E+08	5.7E+08	4.89E+08	4.37E+08	13
1.15E+09	6.94E+08	1.26E+09	9.79E+08	8.1E+08	6
7.23E+08	7.09E+08	5.43E+08	5.1E+08	4.26E+08	9
1.82E+08	1.88E+08	2.12E+08	2.1E+08	2.04E+08	14
2.9E+08	3.28E+08	2.59E+08	2.98E+08	3.09E+08	4
					1
8.99E+09	8.54E+09	5.88E+09	5.69E+09	4.82E+09	41
1.67E+09	1.59E+09	2.66E+09	3.78E+09	2.69E+09	4

47509000	82773000				2
1.24E+08	1.06E+08	1.42E+08	1.19E+08	1.3E+08	3
6.1E+09	4.87E+09	1.81E+09	3.4E+09	3.7E+09	6
2.53E+08	2.74E+08	2.47E+08	1.53E+08	3.08E+08	9
					2
	68132000				2
1.92E+08	1.43E+08	2.08E+08	2.12E+08	2.19E+08	9
2.55E+08	2.77E+08	3.58E+08	4.9E+08	4.03E+08	11
3.94E+08	3.29E+08	3E+08	2.74E+08	3.28E+08	20
6.09E+08	5.52E+08	2.8E+08	1.72E+08	4.51E+08	7
8.01E+08	7.92E+08	6.57E+08	6.22E+08	6.25E+08	5
55143000	64361000	67760000	80697000	53549000	5
4.31E+09	2.91E+09	3.08E+09	3.68E+09	3.35E+09	5
9.04E+08	8.32E+08	1.25E+09	1.08E+09	1.02E+09	13
5.38E+08	7.96E+08	5.57E+08	3.06E+08	3.78E+08	25
4.29E+08	4.15E+08	4.27E+08	4.49E+08	3.85E+08	6
4.02E+08	5.82E+08	3.81E+08	3.63E+08	3.91E+08	4
79489000	78160000	68784000	59252000	70162000	3
1.37E+08		2.91E+08	2.78E+08	2.57E+08	5
1.38E+08	1.39E+08	1.14E+08	1.44E+08	1.34E+08	9
1.12E+09	1.49E+09	1.33E+09	1.59E+09	1.19E+09	6
1.55E+08	1.49E+08	2.72E+08	2.83E+08	2.04E+08	9
4.66E+08	4.13E+08	1.19E+08	1.91E+08	2.27E+08	9
4.88E+08	5.61E+08	5.6E+08	5.26E+08	4.89E+08	8
1.52E+08	1.64E+08	1.71E+08	1.99E+08	2.08E+08	5
				34851000	2
2.69E+08	2.44E+08	3.68E+08	3.86E+08	2.99E+08	8
				85810000	3
1.26E+08		92606000			4
					1
1.41E+08		2.1E+08	1.95E+08	1.71E+08	3
					2
					2
5.96E+08	5.79E+08	3.48E+08	2.58E+08	2.69E+08	7
					1
	20847000				2
7.03E+08	5.43E+08	7.96E+08	5.69E+08	6.75E+08	4
2.98E+08	2.37E+08	3.02E+08	3.12E+08	2.56E+08	11
5.36E+08	4.3E+08	4.72E+08	4.69E+08	5.81E+08	22
					2
	25692000				2
4.51E+08	5.17E+08	5.66E+08	5.24E+08	4.95E+08	8
72838000	58333000		1.12E+08		4
2.24E+09	2.08E+09	2.27E+09	2.23E+09	2.08E+09	11
1.37E+08		1.38E+08			6
3.05E+08	2.92E+08	3.37E+08	3.7E+08	2.61E+08	7
				21966000	5
42744000		44637000	50333000		2
79725000		54449000	1.07E+08	70936000	4
2.02E+09	1.94E+09	2E+09	2.28E+09	2.16E+09	44
2.34E+09	2.43E+09	3.08E+09	3.09E+09	2.7E+09	34

					1
4.08E+09	2.82E+09	5.24E+09	4.3E+09	4.11E+09	13
1.05E+08	82505000	1.49E+08	1.28E+08	2.77E+08	8
41146000		44881000	66075000	47603000	2
6.48E+08	7.17E+08	7.18E+08	8.21E+08	6.3E+08	8
1.23E+09	1.23E+09	1.06E+09	1.05E+09	1.4E+09	6
					2
70580000	70019000	60837000	80360000	79693000	2
81574000	71118000		84579000	49298000	10
2.96E+08	2.45E+08	2.15E+08	2.89E+08	2.74E+08	9
					2
					2
1.1E+09	1.16E+09	1.33E+09	1.57E+09	1.22E+09	16
					1
					1
1.35E+08	1.36E+08	1.75E+08	2.24E+08	1.78E+08	6
1.9E+08	2.39E+08	2.22E+08	2.36E+08	2.29E+08	8
3.75E+08					2
7.44E+09	6.79E+09	5.26E+09	5.73E+09	5.42E+09	14
4.39E+09	4.97E+09	3.26E+09	3.7E+09	4.6E+09	6
71433000	89621000	91123000	49669000	69786000	4
2.46E+08	2.57E+08	2.23E+08	2.26E+08	3.37E+08	4
					1
2.11E+09	2.05E+09	2.68E+09	1.97E+09	2.32E+09	8
1.05E+10	9.6E+09	1.04E+10	9.06E+09	1.11E+10	20
6.78E+09	7.03E+09	1.01E+10	9.19E+09	8.43E+09	28
1.31E+09	1.14E+09	1.54E+09	1.42E+09	1.61E+09	18
6.5E+09	7.76E+09	7.77E+09	8.69E+09	7.67E+09	7
55172000	72270000	72096000	59654000	67556000	12
1.22E+08	1.11E+08	1.03E+08	93762000	1E+08	4
1.04E+08	1.17E+08	87517000	92994000	99501000	3
		20938000		22954000	4
					3
1.2E+08	1.3E+08	1.86E+08	1.49E+08	1.46E+08	18
1.26E+08	1.61E+08	1.21E+08	1.1E+08	1.63E+08	7
					1
1.49E+08	1.63E+08			1.34E+08	5
97052000		1.49E+08			2
	3.45E+08	2.52E+08	2.93E+08	2.5E+08	2
1.41E+08	1.75E+08	1.2E+08	1.2E+08	1.71E+08	4
3.18E+09	2.7E+09	3.48E+09	3.32E+09	3.91E+09	46
1.29E+09	1.22E+09	1.99E+09	1.36E+09	1.48E+09	9
17247000		19920000		18213000	2
4.3E+08	3.79E+08	3.97E+08	3.86E+08	3.69E+08	10
					1
3.28E+08	2.79E+08	4.05E+08	4.6E+08	4.9E+08	18
3.96E+08	3.08E+08	3.2E+08	4.52E+08	3.7E+08	5
3.51E+08	6.03E+08	3.68E+08	2.6E+08	3.82E+08	6
53732000	79200000	1.23E+08	73567000	79890000	6
3.01E+08	2.81E+08	2.79E+08	3.22E+08	2.84E+08	10
1.16E+09	1.06E+09				2

1.41E+08	2.17E+08	76084000	52989000	1.01E+08	2
					1
1.95E+08	2.58E+08	1.66E+08	2.16E+08	2.68E+08	4
1.22E+08	1.49E+08	1.34E+08	1.06E+08	1.16E+08	13
1.05E+08	1.47E+08	1.42E+08	1.79E+08	1.67E+08	4
					1
75969000	1.02E+08	88742000	82372000	1.02E+08	5
				40845000	3
3.96E+08	3.53E+08	3.68E+08	3.76E+08	4.07E+08	8
	80570000				3
2.25E+09	1.19E+09	4.86E+08	6.64E+08	3.65E+08	7
87900000	63101000	83056000	54853000	82835000	4
64559000	89638000	1.49E+08	81288000	79615000	7
					2
6.79E+08	7.37E+08	5.65E+08	6.17E+08	5.77E+08	11
5.06E+08	4.01E+08	6.3E+08	5.49E+08	5.26E+08	4
1.73E+08	1.89E+08	2.84E+08	2.51E+08	2.17E+08	9
5.59E+08	6.06E+08	4.79E+08	4.71E+08	4.99E+08	24
				2.55E+08	1
1.3E+08	67356000	1.04E+08	1.39E+08	1.56E+08	3
7.73E+09	8.32E+09	4.56E+09	5.36E+09	4.4E+09	13
7.65E+09	5.74E+09	7.11E+09	7.77E+09	8.43E+09	16
2.87E+09	2.61E+09	2.74E+09	3.23E+09	2.3E+09	8
5.28E+09	6.17E+09	3.93E+09	4.8E+09	4.74E+09	7
6.37E+08	9.52E+08	7.47E+08	5.15E+08	6.48E+08	6
5.79E+09	8.6E+09	2.7E+09		5.09E+09	2
45431000	46500000			39241000	3
		14508000		9526100	4
1.42E+09	1.15E+09	1.38E+09	1.99E+09	1.83E+09	8
6.48E+09	6.08E+09	7.66E+09	8.03E+09	7.1E+09	8
2.66E+08					3
3.87E+09	4.28E+09	3.64E+09	3.88E+09	4.4E+09	16
3.06E+09	3.53E+09	2.66E+09	3.82E+09	3.38E+09	7
1.24E+09	1.31E+09	8.2E+08	1.46E+09	2.59E+09	14
4.26E+09	3.87E+09	4.15E+09	5.22E+09	4.69E+09	10
8.13E+08	9.26E+08	1.1E+09	1.13E+09	8.06E+08	3
3.65E+09	3.55E+09	5.1E+09	5.09E+09	5.06E+09	33
7.42E+09	6.71E+09	8.1E+09	1.01E+10	1.03E+10	10
					1
2.67E+08	2.21E+08	2.73E+08	2.32E+08	2.67E+08	5
1.34E+08	2E+08	1.85E+08	2E+08	2.41E+08	2
					1
64811000	50210000	51968000	50671000	51330000	4
8.81E+08	8.39E+08		8.28E+08	7.47E+08	8
1.92E+08	1.82E+08	1.96E+08	2.13E+08	1.63E+08	4
48901000		1.47E+08		53134000	4
1.22E+08					2
5.61E+08	6.73E+08	5.49E+08	5.87E+08	6.31E+08	11
7.72E+08	7.43E+08	1.25E+09	1.32E+09	1.47E+09	15
1.06E+08	1.27E+08	1.73E+08	96145000	2.72E+08	6
2.02E+09	2.35E+09		7.96E+08	2.07E+09	1

87790000	97501000	94868000	1.14E+08	97840000	5
96030000	1.22E+08	1.4E+08	99664000	1.46E+08	6
8.77E+09	1.13E+10	8.77E+09	1.15E+10	9.69E+09	4
6.65E+08	5.53E+08	5.08E+08	5.91E+08	9E+08	4
					1
					2
6.58E+08	8.22E+08	9.1E+08	9.65E+08	8.84E+08	16
9.58E+09	8.03E+09	1.02E+10	9.46E+09	7.57E+09	40
1.11E+09	1.03E+09	1.47E+09	1.47E+09	1.24E+09	5
3.16E+09	2.17E+09	3.4E+09	4.08E+09	3.96E+09	6
					2
6.73E+08	7.46E+08	8E+08	7.08E+08	7.1E+08	8
2.22E+09	2.02E+09	1.98E+09	2.21E+09	2.31E+09	11
5.85E+09	4.37E+09	1.05E+10	4.11E+09	2.29E+09	8
8.33E+09	9.95E+09	7.94E+09	7.58E+09	8.38E+09	10
2.92E+09	2.22E+09	2.87E+09	2.56E+09	3.13E+09	6
3.81E+08	2.45E+08	2.6E+08	3.06E+08	3.42E+08	4
2.19E+08	2.83E+08	3.7E+08	3.36E+08	3.25E+08	11
6.93E+08	6.54E+08	7.81E+08	5.67E+08	6.55E+08	5
2.54E+08	1.77E+08	3.29E+08	2.71E+08	2.92E+08	13
3.98E+08	4.92E+08	4.05E+08	4.01E+08	4.43E+08	6
					5
2.51E+09	2.85E+09	1.69E+09	2.03E+09	2.28E+09	17
2.56E+08	2.32E+08	1.87E+08	2.13E+08	1.39E+08	11
1.55E+08	1.59E+08	1.77E+08	2.22E+08	2.16E+08	4
					2
3.46E+08	4.18E+08	3.81E+08	3.53E+08	3.66E+08	7
3.94E+08	3.27E+08	5.43E+08	5.83E+08	4.6E+08	7
7.49E+08	8.25E+08	1.03E+09	7.86E+08	1.01E+09	5
					5
6.69E+08	7.17E+08				6
1.4E+08	2.35E+08	1.73E+08	1.65E+08	2.07E+08	7
1.39E+08	1.2E+08	1.22E+08	1.26E+08	1.23E+08	10
					2
1.9E+09	1.58E+09	1.27E+09	1.75E+09	1.7E+09	33
1.77E+09	1.24E+09	1.04E+09	9.16E+08	9.89E+08	22
2.32E+08	2.51E+08	2.33E+08	1.96E+08	1.95E+08	7
1.11E+08		94245000	1.26E+08	93452000	5
1.83E+08	2.16E+08	1.97E+08		2.08E+08	7
3.79E+08	3.17E+08	3.48E+08	3.45E+08	4.24E+08	4
	35648000			41170000	4
4.13E+08	3.76E+08	4.18E+08	3.97E+08	4.3E+08	11
2.05E+08	3.13E+08	4.4E+08	4.4E+08	4.97E+08	7
1.49E+08	1.33E+08	1.42E+08	1.29E+08	1.47E+08	4
			30461000		3
				31910000	4
9.57E+08	7.26E+08	9.33E+08	1.17E+09	1.01E+09	13
64084000					5
95037000					5
4.58E+09	2.91E+09	3.73E+09	4.99E+09	2.99E+09	9
1.08E+08	1.71E+08	1.51E+08	1.29E+08	1.26E+08	12

1.41E+08	1.24E+08	1.2E+08	1.24E+08	1.25E+08	8
					1
57354000	63713000	1.65E+08		78348000	2
	86772000	80425000		82220000	5
3.2E+08	3.52E+08	4.52E+08	3.82E+08	3.96E+08	6
2.16E+08	1.94E+08	1.91E+08	2.02E+08	1.9E+08	5
2.09E+08	2.1E+08	3.02E+08	3.02E+08	2.45E+08	4
2.11E+09	1.66E+09	1.23E+09	1.39E+09	1.35E+09	5
5.39E+08	5.02E+08	6.25E+08	5.99E+08	5.4E+08	5
1.14E+08	2.18E+08		50865000	73383000	9
					3
5.1E+08	5.34E+08	3.99E+08	4.73E+08	3.63E+08	7
7.17E+08	6.66E+08	9.35E+08	1.04E+09	9.99E+08	16
3.96E+08					3
					2
4.65E+08	4.67E+08	5.82E+08	6.08E+08	5.76E+08	12
		42720000			2
	1.44E+08	1.98E+08	2.1E+08	1.71E+08	5
					1
2.88E+08	2.43E+08	1.53E+08		2.14E+08	4
2.79E+08	2.84E+08	3.33E+08	3.38E+08	3.53E+08	6
		29500000	27478000	35596000	5
55741000	61372000	75234000	65193000	60026000	7
					1
1.1E+08	1.51E+08	96610000	88025000	99812000	11
	1.79E+08			1.69E+08	1
6.16E+08	5.63E+08	4.83E+08	5.7E+08	4.67E+08	8
83364000	84586000	87624000	80811000	1.01E+08	2
1.08E+09	9.86E+08	1.6E+09	1.15E+09	1.33E+09	18
2.22E+08		2.01E+08	1.99E+08	1.26E+08	4
3.77E+08	7.42E+08	4.31E+08	4.68E+08	4.26E+08	4
46805000			67479000	64450000	3
					2
					1
1.94E+09	1.77E+09	2.01E+09	1.96E+09	2.24E+09	19
77770000	89135000	61996000		71687000	4
36146000	67020000		40208000		2
82391000	90215000	81485000	89939000	81588000	8
					2
22452000		29543000			3
1.75E+08	1.14E+08	93314000	2.03E+08	1.54E+08	4
3.63E+08	4.92E+08	4.85E+08	2.58E+08	7.19E+08	2
					7
1.59E+08		2E+08	2.21E+08	2.2E+08	2
	29653000	49865000	29643000	79349000	1
4.96E+08	2.99E+08			2.96E+08	9
2.28E+08	2.18E+08	3.76E+08	4.14E+08	3.6E+08	21
					1
2.21E+08					2
					3
					3

55658000	52349000	80075000	63027000	58760000	5
1.32E+08					4
1.52E+08	47637000	2.32E+08	2.42E+08	1.78E+08	6
1.4E+08	1.29E+08	90066000	1.12E+08	1.37E+08	8
					1
81090000	1.08E+08	48669000	51147000	74115000	2
34706000	28149000	31971000	35428000	31166000	7
2.55E+08	2.15E+08	2.9E+08	2.34E+08	2.86E+08	14
4.37E+08	5.45E+08	5.19E+08	4.88E+08	4.55E+08	7
3.13E+08	3.56E+08	1.64E+08	2.01E+08	5.46E+08	8
			86190000	67584000	4
	5.51E+08	6.93E+08		1.13E+09	4
5.02E+08	5.38E+08	4.31E+08	4.3E+08	4.78E+08	5
74274000	81980000	1.57E+08	58312000	73235000	2
4.16E+09	3.98E+09	3.14E+09	3.82E+09	3.59E+09	17
5.5E+08	6.44E+08	4.93E+08	6.19E+08	5.37E+08	11
7.69E+08	6.52E+08	5.64E+08	6.73E+08	6.83E+08	9
					1
1.35E+08	1.23E+08	59900000	66588000	64562000	4
1.95E+08	2.61E+08	2.62E+08	2.85E+08	2.76E+08	6
97627000	77621000	1.23E+08	1.14E+08	1.11E+08	6
1.38E+08	1.14E+08	1.36E+08	1.68E+08	1.75E+08	4
					2
4.28E+08		4.22E+08			2
2.22E+08	2.66E+08	1.71E+08	1.7E+08	2.01E+08	5
2.27E+09	2.33E+09	3.33E+09	3.14E+09	3.77E+09	9
4.22E+08	4.12E+08	4.17E+08	4.63E+08	4.11E+08	8
			60333000		2
3.39E+08	3.64E+08	3.57E+08	3.31E+08	3.8E+08	10
				22295000	4
					2
1.3E+09	1.32E+09	1.07E+09	1.1E+09	1.19E+09	15
75903000	1.1E+08	55098000	45828000	52957000	5
52829000					3
41975000	53736000	66176000	58565000		4
1.58E+08	1.58E+08	1.1E+08	1.32E+08	1.34E+08	1
					2
1.32E+09	1.28E+09	4.1E+09	3.12E+09	6.25E+09	16
					1
	22748000	30962000	31927000		3
					1
					1
1.37E+08	1.4E+08	1.39E+08	1.36E+08	1.34E+08	10
1.52E+09	1.54E+09	1.99E+09	2.23E+09	2.19E+09	3
23908000					2
96174000	1.1E+08	1.43E+08	1.37E+08	1.33E+08	10
68092000				1.47E+08	2
					1
					1
					2
96606000	2.06E+08	1.48E+08	1.54E+08	94474000	4

1.44E+08	1.08E+08	1.24E+08	1.67E+08	1.19E+08	2
2.67E+09	2.55E+09	2.22E+09	2.01E+09	2.28E+09	14
6.6E+08	6.61E+08	7.57E+08	7.81E+08	6.19E+08	5
58558000	53561000	61333000	71778000	66857000	7
		1.11E+08		1.1E+08	3
					4
					3
1.23E+08			1.09E+08	1.06E+08	3
					3
26757000	30004000	42486000	51923000		4
98443000	98758000	83035000	1.11E+08	97737000	3
1.03E+09	1.04E+09	1.08E+09	9.42E+08	7.57E+08	7
22672000					3
98934000	55152000	86651000	81151000	99866000	6
55004000	85735000	66164000		84298000	5
1.19E+08	97418000	1.11E+08	1.44E+08	1.4E+08	4
					1
51659000	44185000	62074000	41555000	52261000	5
					1
1.27E+08	1.58E+08	1.16E+08	1.5E+08	1.48E+08	8
		56468000	48765000	47255000	3
					2
					1
					2
	19605000				4
3.43E+08	2.81E+08	3.47E+08	4.36E+08	3.8E+08	5
					1
2.55E+08	1.91E+08	2.11E+08	1.92E+08	2.19E+08	12
36466000				25771000	4
		1.56E+08			5
1.97E+08	1.79E+08		1.19E+08	1.25E+08	4
6.72E+08	1.21E+09	4.26E+08	6.93E+08	1.29E+09	10
3.23E+08	3.74E+08	2.14E+08	2.42E+08	1.79E+08	7
2.6E+08	2.23E+08	2.29E+08	2.48E+08	2.12E+08	5
1.18E+09	1.13E+09	7.16E+08	8.68E+08	7.19E+08	13
19257000	26619000	19344000		16105000	4
1.62E+10	1.74E+10	2.22E+10	1.85E+10	1.84E+10	10
86051000	95452000	1.91E+08			2
1.06E+09	1.09E+09	2.23E+09	2.62E+09	2.52E+09	23
					3
1.46E+09	1.2E+09	1.31E+09	1.53E+09	1.49E+09	8
					3
2.25E+09	3.06E+09	1.89E+09	1.85E+09	2.45E+09	4
31906000	29912000	28384000	35165000	27581000	3
2.23E+09	2.17E+09	3.21E+09	3.52E+09	3.43E+09	21
	35371000				4
					1
45006000	49890000	43329000	41997000	46611000	5
3E+08	3.87E+08	3.55E+08	2.97E+08	4.1E+08	7
					5
83037000	75270000	95129000	98803000	76646000	2

1.88E+08	1.32E+08	1.48E+08	1.83E+08	1.87E+08	5
					5
5228000	13407000		5593800	9304600	3
2.91E+08	2.07E+08	1.67E+08	1.06E+08	3.09E+08	6
46719000	36749000		28992000	31371000	4
1.5E+08	1.58E+08	1.27E+08	1.36E+08	1.19E+08	4
2.23E+08	2.57E+08	1.49E+08	1.77E+08	1.46E+08	4
	76626000	69633000	73851000	75312000	6
		89743000			3
98339000	1.15E+08				2
48196000	50005000	47542000	42541000	44422000	7
					4
					3
			60111000	38249000	4
					3
1.09E+08	1.12E+08	97436000	94768000	79408000	7
5.15E+08	3.36E+08	3.48E+08	4.33E+08	3.98E+08	9
					1
2.53E+08	2.14E+08	3.6E+08	4.45E+08	3.07E+08	13
2.88E+08	4.14E+08	2.59E+08	3.32E+08	2.67E+08	5
					2
1.04E+08	1.24E+08	82408000	64005000	63411000	5
2.83E+09	2.22E+09	2.63E+09	3.04E+09	2.5E+09	22
					2
					2
5.43E+08	5.85E+08	5.67E+08	5.87E+08	4.52E+08	4
					1
	2.47E+08	2.13E+08	2.34E+08		5
6.97E+08	9.28E+08	1.45E+09	5.61E+08	7.27E+08	7
41253000			48929000	56941000	4
35260000			79400000		1
1.57E+09	1.48E+09	1.2E+09	1.16E+09	1.46E+09	5
2.38E+08	2.41E+08	1.97E+08	3.22E+08	2.84E+08	3
3.91E+08	3.19E+08	3.78E+08	3.65E+08	3.78E+08	10
					1
					2
3.28E+08	2.14E+08	2.28E+08	2.27E+08	2.68E+08	4
					2
3.08E+08	2.59E+08	3.42E+08	3.38E+08	3.97E+08	11
					2
5.54E+08	5.47E+08	4.74E+08	5.43E+08	5.75E+08	10
					1
	2.1E+08			1.08E+08	4
					2
2.24E+08	3.18E+08	2.2E+08	2.51E+08	2.46E+08	5
1.13E+09	8.81E+08	1.1E+09	8.74E+08	9.06E+08	10
38038000		37832000		44659000	5
1.51E+09	1.39E+09	1.83E+09	1.65E+09	1.66E+09	22
			28451000		2
3.66E+09	3.63E+09	1.9E+09	2.34E+09	2.04E+09	8
		58586000			6

					1
2.37E+08	2.05E+08			2.06E+08	3
1.7E+08	1.6E+08	1.45E+08	1.46E+08	1.3E+08	4
					2
1.15E+09	8.27E+08	7.23E+08	8.57E+08	9.15E+08	10
2.23E+08	3.12E+08	2.69E+08	2.31E+08	3.1E+08	4
2.21E+08		2.43E+08	2.67E+08	2.59E+08	3
95537000					1
					4
3.56E+08	3.45E+08	2.11E+08	2.39E+08	2.16E+08	8
					3
78066000	66221000	43943000	65219000	57916000	2
					1
8.36E+08	1.23E+09	8.18E+08	8.97E+08	6.96E+08	6
1.24E+09	1.06E+09	1.54E+09	1.46E+09	1.43E+09	6
		27469000			3
					2
1.24E+10	1.44E+10	9.75E+09	6.49E+09	1.21E+10	10
4.59E+08	2.79E+08	5.9E+08	4.47E+08	6.98E+08	4
					2
75854000		67738000		76731000	1
					2
63562000	76546000	52856000	54091000	55326000	4
					2
					1
					2
2.36E+09	2.97E+09	1.53E+09	1.95E+09	1.98E+09	4
3.96E+08	6.3E+08	3.01E+08	4E+08	3.8E+08	6
1.23E+08	99635000	1.1E+08	1.03E+08	1.05E+08	3
5.44E+08	4.15E+08	4.34E+08	4.34E+08	6.76E+08	3
58188000	46574000	41628000	90051000	62308000	6
43677000	35249000	31228000	70537000	59964000	2
					2
1.03E+09	1.07E+09	9.78E+08	9.08E+08	1.09E+09	20
					4
					1
					1
5.88E+09	6.74E+09	4.73E+09	4.15E+09	4.91E+09	8
1.17E+08	1.14E+08	49617000	96824000	89861000	11
2.63E+08	1.9E+08	2.38E+08	1.96E+08	1.61E+08	4
					1
2.21E+08	2.53E+08	1.54E+08	1.39E+08	1.76E+08	3
3.22E+08	4.77E+08	6.33E+08	3.74E+08	3.61E+08	7
61243000	73685000	97089000	64524000	64711000	2
8.39E+08	7E+08	7.89E+08	8.78E+08	8.91E+08	5
2.97E+08	3.43E+08	2.5E+08	3.05E+08	9.49E+08	3
1.17E+08	87538000	1.27E+08	1.53E+08	1.29E+08	6
69776000	51670000	93877000	90101000	59318000	2
61486000	1.17E+08			69637000	3
					1
97178000	1.13E+08	1.29E+08	1.42E+08	1.27E+08	5

6.34E+08	4.08E+08	5.14E+08	4.4E+08	3.59E+08	10
		47423000		39351000	2
					1
81991000	67532000	1.4E+08	91592000	93680000	6
48768000		47817000		38037000	2
53971000	66406000		50598000		1
4.68E+08	3.36E+08	3.78E+08	3.42E+08	3.29E+08	7
2.64E+08	2.81E+08	2.81E+08	2.74E+08	2.8E+08	8
					1
2.24E+08	1.35E+08	1.2E+08	1.31E+08	1.52E+08	2
					1
					5
					1
					1
					1
					3
1.44E+09	1.65E+09	1.13E+09	1.35E+09	1.31E+09	3
					2
5.05E+08	2.86E+08		2.02E+08	3.34E+08	2
		51803000	73482000	53888000	4
5.74E+08	6.07E+08	5.34E+08	6.68E+08	6.17E+08	10
3.28E+08	3.62E+08	2.71E+08	3.32E+08	3.54E+08	3
3.09E+08	2.82E+08	2.63E+08	3.31E+08	2.83E+08	8
6.41E+08	6.3E+08	9.19E+08	7.23E+08	9.01E+08	10
5.41E+08	5.64E+08	7.2E+08	6.83E+08	6.66E+08	15
1.04E+09	9.05E+08	1.52E+09	1.44E+09	1.33E+09	17
					2
8.21E+08	1.06E+09		8.96E+08	7.56E+08	8
54516000	57140000	81287000		62371000	4
1.69E+08	2.11E+08				1
1.07E+08	1.18E+08		96632000	81178000	3
5.77E+08	6.48E+08	4.39E+08	4.96E+08	4.57E+08	11
1.74E+08	2E+08	1.98E+08	2.02E+08	1.28E+08	6
1.12E+08	1.37E+08	71925000	68713000	77670000	7
			2.78E+08	2.75E+08	2
2.88E+08	2.15E+08	2.23E+08	1.99E+08	1.83E+08	4
1.98E+08	2.35E+08	1.89E+08	1.56E+08	1.89E+08	4
					2
4.66E+09	4.23E+09	4.06E+09	5.95E+09	6.18E+09	14
	3.58E+08				4
33251000	34143000	36944000	22193000	26081000	6
					1
6.45E+08	7.12E+08	5.31E+08	6.62E+08	4.61E+08	4
					2
2.3E+08	2.66E+08	2.77E+08	2.77E+08	2.37E+08	8
16900000			18572000	17905000	2
3.18E+08	3.87E+08	3.16E+08	3.26E+08	3.13E+08	6
5.06E+08	4.33E+08	5.77E+08	4.58E+08	3.98E+08	9
3.49E+08	2.96E+08	3.8E+08	4.01E+08	3.61E+08	9
					1
9.43E+08	1.07E+09	1.05E+09	1.06E+09	8.17E+08	13

1.92E+08	2.11E+08	82534000		2.66E+08	5
1.78E+09	1.29E+09	1.7E+09	2.03E+09	1.52E+09	9
1.44E+08	1.35E+08		2.02E+08	1.37E+08	2
6.19E+08	6.71E+08	8.91E+08	7.9E+08	7.94E+08	2
87790000	93936000	93017000	96359000	93443000	3
					1
3.14E+09	3.58E+09	2.72E+09	2.61E+09	2.82E+09	13
2.57E+08	2.97E+08	1.59E+08	94771000	1.09E+08	2
					1
1.88E+08	1.61E+08	1.74E+08	2.01E+08	1.77E+08	5
1.32E+08	1.61E+08	1.44E+08	1.69E+08	1.32E+08	7
1.9E+08	1.74E+08	1.47E+08	1.78E+08	1.86E+08	8
91753000	53008000	63821000	47089000	75059000	5
					2
7.89E+08	5.29E+08	7.23E+08	8.75E+08	8.96E+08	11
	97242000			56180000	1
					1
1.26E+08	1.42E+08	1.25E+08	1.33E+08	1.03E+08	5
46396000		80043000	79990000	62700000	2
					1
80766000	81869000	98251000	89530000	84641000	3
4.08E+08	4.54E+08	4.82E+08	5.71E+08	5.77E+08	7
					3
4.38E+09	3.56E+09	4.16E+09	4.92E+09	4.1E+09	16
4.94E+08	3.36E+08	4.71E+08	6.06E+08	6.67E+08	8
					1
3.17E+08	4.15E+08	3.51E+08	2.75E+08	2.87E+08	6
1.22E+08	1.16E+08	1.11E+08	1.41E+08	1.52E+08	5
1.53E+08	1.2E+08	1.41E+08	1.59E+08	1.22E+08	6
4.43E+08	5.47E+08	6.72E+08	4.59E+08	5.31E+08	17
1.52E+09	1.69E+09	1.12E+09	1.25E+09	1.1E+09	6
1.43E+08	1.12E+08	3.07E+08	3.37E+08	3.26E+08	7
1.52E+08	2.03E+08	2.42E+08	1.53E+08	2.36E+08	8
1.44E+08	1.41E+08	1.56E+08	1.59E+08	1.6E+08	13
72037000	84107000	87081000	86174000	88315000	8
					6
					2
1.51E+09	1.26E+09	2.01E+09	1.81E+09	1.85E+09	14
82955000	66611000	1.12E+08	86173000	77680000	6
5.21E+08	5.24E+08	3.09E+08	5.97E+08	5.13E+08	7
					2
					1
1.46E+09	1.26E+09	1.56E+09	2.22E+08	1.22E+09	3
3.82E+08	3.51E+08	3.72E+08	3.81E+08	3.61E+08	7
6.93E+08	7.9E+08	6.04E+08	6.17E+08	4.86E+08	11
	89192000	1.12E+08			6
5.28E+08	5.34E+08	7.37E+08	6.66E+08		4
					4
36729000	34912000		44284000	39104000	3
					1
82375000	90405000	76130000	74156000		5

	2.52E+08	1.72E+08	2.28E+08	1.55E+08	1
59506000	40409000				1
					1
2.41E+08	1.99E+08	1.42E+08	2.13E+08	1.92E+08	5
97652000	1.11E+08	1.93E+08	1.42E+08	1.48E+08	7
					1
		38339000	28725000		3
2.42E+08	2.73E+08	2.03E+08	2.73E+08	1.82E+08	4
67453000	71702000	93530000	74023000	85399000	6
1.92E+09	2E+09	2.08E+09	2.09E+09	1.72E+09	15
1.06E+08	1.09E+08		1.37E+08		5
					1
					1
					1
23310000	34964000	39228000	20870000	28018000	5
7.75E+08	8.84E+08	6.51E+08	3.97E+08	8.17E+08	8
7.49E+08	7.5E+08	8.04E+08	7.46E+08	8.35E+08	7
79668000	70044000	78090000		72727000	5
1.8E+08	2.33E+08	2.13E+08	1.54E+08	1.83E+08	5
5.26E+08	4.2E+08	3.91E+08	3.57E+08	4.86E+08	14
					2
					1
83031000	86369000				2
	1.16E+08	70477000	61673000	72297000	2
66845000	27759000		42234000	27599000	5
1.49E+09	1.51E+09	1.41E+09	1.33E+09	1.42E+09	18
19615000				18132000	4
54453000	45272000	36228000	31231000	53463000	2
					2
64123000	39489000	40569000	49194000	70199000	4
28642000	43307000	12097000	26302000	33754000	4
1.71E+08	2.27E+08	1.85E+08	1.87E+08	1.92E+08	12
		73945000	59841000		4
35397000		21787000	47864000	79586000	2
2.98E+08	3.72E+08	4.28E+08	3.27E+08	2.85E+08	5
1.1E+09	8.26E+08	5.71E+08	7.25E+08	1.11E+09	10
4.13E+09	4.46E+09				3
					2
	36248000			67447000	2
					1
	36694000				2
2.28E+09	2.11E+09	1.11E+09	1.33E+09	1.77E+09	7
					1
1.9E+08	1.58E+08	2.66E+08	2.41E+08	2.37E+08	14
					2
7.78E+08	5.33E+08	7.5E+08	6.8E+08	6.21E+08	8
					3
1.69E+08	1.86E+08	3.57E+08	2.02E+08	1.66E+08	6
85417000	1.03E+08	1.08E+08	62130000		4
4.83E+08	3.49E+08		2.79E+08	1.78E+08	3
9.86E+08	8.9E+08	9.66E+08	1.04E+09	1.14E+09	16

59247000	75350000			31033000	3
1.74E+08	1.52E+08	1.43E+08	1.39E+08	2.3E+08	4
					1
					1
					3
	47628000	68579000		63077000	2
					3
	1.21E+08				1
4.16E+08	5.4E+08	4.62E+08	3.05E+08	6E+08	3
					2
2.32E+09	3.2E+09	3.59E+09	2.58E+09	2.67E+09	15
		28769000		37677000	5
					2
2.06E+08	2.06E+08	1.79E+08	2.55E+08	2.33E+08	7
7.66E+08	7.14E+08	7.01E+08	7.82E+08	9.01E+08	12
					2
2.99E+08	3.26E+08	2.87E+08	2.72E+08	3E+08	11
5.75E+08	5.36E+08		5.33E+08	5.2E+08	3
					2
3.3E+08	3.72E+08	3.12E+08		3.62E+08	6
2.56E+08	2.31E+08	2.59E+08	2.15E+08	2.42E+08	18
1.36E+09	1.16E+09	6.8E+08	6.3E+08	9.95E+08	9
					1
					2
					3
1.33E+08	1.19E+08	80298000	1.15E+08	95130000	4
9.55E+08	7.27E+08	1.27E+09	1.82E+09	1.23E+09	5
					2
					3
36351000	31042000				2
					1
					3
1.25E+08		1.36E+08		64379000	4
					2
3.84E+08	3.31E+08	1.61E+08	1.85E+08	2.76E+08	6
1.96E+08	1.54E+08	1.7E+08	1.87E+08	2.08E+08	8
3.32E+08	2.76E+08	2.71E+08	2.54E+08	2.39E+08	15
1.08E+08	1.11E+08	1.03E+08	86655000	1.06E+08	4
					1
1.17E+08	84966000	96389000	1.23E+08	92678000	11
	58190000			1.15E+08	4
2.91E+09	4.2E+09	1.84E+09	2.03E+09	1.91E+09	6
1.82E+08	2.61E+08	2.48E+08	2.48E+08	2.4E+08	4
2.8E+08	3.41E+08	2.3E+08	2.42E+08	2.19E+08	10
					2
					5
2.57E+08	1.79E+08	1.37E+08	1.44E+08	1.56E+08	10
10547000					3
					5
1.83E+09	1.71E+09	1.14E+09	1.56E+09	1.28E+09	3
7.46E+08	7.09E+08	5.83E+08	4.29E+08	5.82E+08	4

65206000	58229000	56012000		60113000	4
1.09E+08	1E+08	1.41E+08	94993000	1.24E+08	7
	1.2E+08	1.19E+08	1.17E+08	1.01E+08	4
1.09E+09	1.02E+09	1.06E+09	1E+09	1.13E+09	10
	69985000	93796000		1.14E+08	3
90014000	87485000	50025000	52956000	48686000	2
					1
2.73E+08	1.92E+08	3.37E+08	3.38E+08	3.02E+08	6
					1
7.72E+08	8.49E+08	9.27E+08	9.77E+08	9.74E+08	12
					3
36094000		29234000	36451000		5
					1
1.52E+08	1.37E+08	2E+08			3
					1
8.2E+08	7.26E+08	6.83E+08	8.47E+08	8.01E+08	7
	3.25E+08		4.48E+08	4.74E+08	3
	39118000			51077000	1
3.25E+08	70197000	3.04E+08		87543000	2
31433000			28065000		2
	68850000		81441000	75124000	2
6.25E+08	5.12E+08	5.8E+08	5.22E+08	5.9E+08	20
2.83E+08	2.6E+08	5.31E+08	6.72E+08	5.17E+08	9
2.64E+09		1.99E+09	2.41E+09	1.77E+09	1
7.21E+08	4.5E+08	7.23E+08	3.48E+08	2.39E+08	8
6.4E+08	6.25E+08		6.11E+08		3
90332000	1.5E+08	55846000	31331000	75019000	2
					2
1.63E+08	1.26E+08	1.16E+08	1.37E+08	2.32E+08	3
					1
1.85E+08	1.34E+08		99334000		4
1.25E+08	1.76E+08	1.16E+08	1.33E+08	1.36E+08	8
					4
3.37E+08	3.13E+08	1.94E+08	2.51E+08	2.21E+08	9
					1
1.36E+08	98609000	1.18E+08	1.7E+08	1.38E+08	5
1.17E+09	1.09E+09	1.16E+09	1.01E+09	9.82E+08	6
78700000	55937000	39957000	59179000	70772000	6
1.26E+09	1.33E+09	9.25E+08	1.12E+09	1E+09	21
	91302000		74008000	70019000	2
					2
8.77E+08	1.16E+09	8.57E+08	8.06E+08	8.7E+08	8
4.24E+08	3.82E+08	4.5E+08	4.96E+08	4.66E+08	15
5.36E+08	6.74E+08	4.38E+08	5.33E+08	4.73E+08	4
				22011000	1
9.8E+08	1.13E+09	1.91E+09	1.09E+09	1.04E+09	4
85566000	1.07E+08				4
8.85E+08	8.94E+08	7.96E+08	8.62E+08	9.02E+08	7
1.45E+08	1.76E+08	1.45E+08	1.25E+08	1.12E+08	3
1.28E+08	1.4E+08	1.12E+08	88125000	1.18E+08	7
					1

3.13E+08	2.9E+08	3.72E+08	2.44E+08	1.97E+08	5
4.59E+08	5.05E+08	3.13E+08	3.34E+08	3.51E+08	14
6.6E+08	5.99E+08	5.32E+08	5.53E+08	5.42E+08	29
3.6E+08	3.08E+08	3.9E+08	3.32E+08	2.59E+08	4
4.95E+08	4.74E+08	4.46E+08	4.28E+08	4.54E+08	18
1.32E+09	1.78E+09	8.34E+08	9.78E+08	9.97E+08	12
					2
4E+09	3.77E+09	4.25E+09	3.42E+09	3.31E+09	15
9.36E+08	9.5E+08	7.01E+08	7.75E+08	9.53E+08	8
					1
3.44E+08	4.81E+08	4.14E+08	3.61E+08	4.5E+08	2
9.08E+08	8.77E+08	9.03E+08	9.05E+08	7.82E+08	17
2.01E+08	3.18E+08	1.79E+08	2.43E+08	1.66E+08	4
					4
1.78E+08	1.24E+08	2.53E+08	2.23E+08	1.97E+08	4
6.58E+08	6E+08	6.78E+08	8.49E+08	6.73E+08	15
3.69E+09	3.6E+09	3.73E+09	4.04E+09	3.93E+09	13
1.72E+08	1.62E+08	1.47E+08	1.68E+08	1.62E+08	7
3.12E+08	2.7E+08	3.27E+08	1.94E+08	2.19E+08	5
					2
3.7E+08	4.24E+08	3.15E+08	2.99E+08	3.33E+08	1
26258000	32695000	34604000		29340000	2
					3
2.89E+08	2.85E+08	2.63E+08	2.64E+08	2.39E+08	7
19503000		55454000	49411000	54919000	3
6.71E+08	5.17E+08	4.52E+08	6.21E+08	4.78E+08	13
					1
					7
		31824000	30017000	22444000	2
1.89E+08	1.82E+08	2.45E+08	2.02E+08	1.54E+08	10
8.24E+08	8.43E+08	6.73E+08	6.77E+08	5.92E+08	11
	58858000			47159000	1
3.77E+09	3.37E+09	2.77E+09	2.53E+09	3.2E+09	18
3.12E+08	3.08E+08		93577000	1.09E+08	2
3.42E+08	2.08E+08		3.38E+08	2.04E+08	3
6.09E+08	4.87E+08	5.8E+08	4.87E+08	5.66E+08	13
2.31E+08	1.91E+08	3.35E+08	2.35E+08	2.18E+08	5
			59504000		2
					2
3.72E+08	3.79E+08	3.92E+08	3.69E+08	3.92E+08	14
					2
43240000	46288000	65509000	52074000	42321000	4
				64365000	4
1.53E+08	1.39E+08	2.02E+08	1.78E+08	1.77E+08	5
90607000	74303000	75631000	71026000		5
5.25E+08	4.37E+08	5.21E+08	5.28E+08	6.18E+08	5
					2
1.34E+08	71946000	1.13E+08	1.17E+08	1.64E+08	6
2.61E+08		4.18E+08	4E+08	4.73E+08	2
					5
79829000		88865000			6

1.96E+08	1.83E+08	1.61E+08		98086000	7
1.84E+08	1.93E+08	1.35E+08	77582000	1.8E+08	7
		23539000			2
					1
72911000					3
1.36E+08	94127000	1.91E+08	1.83E+08	1.73E+08	5
91355000	87442000	82371000	1E+08	96190000	6
1.37E+09	1.19E+09	1.38E+09	1.43E+09	1.06E+09	19
75357000	68698000			59328000	3
64125000	58691000			41479000	4
1.7E+09	1.59E+09	2.07E+09	2.1E+09	1.75E+09	13
1.2E+09	1.15E+09	1.16E+09	1.16E+09	9.78E+08	7
3.63E+09	4.4E+09	2.6E+09	3.23E+09	2.92E+09	21
50811000	50469000	68321000	58715000		2
1.04E+08		1.26E+08	1.36E+08	1.37E+08	3
	37697000		64126000		6
				68014000	1
92417000	72430000	75593000	86214000	81150000	10
79172000	56391000	71715000	1.08E+08	68842000	5
					3
4.96E+08			4.04E+08		6
					1
		7.42E+08			4
8.39E+08	6.57E+08	5.22E+08	5.59E+08	6.59E+08	13
6.53E+08	5.82E+08	5.16E+08	5.39E+08	5.31E+08	14
1.09E+09	1.06E+09	1.46E+09	1.04E+09	1.06E+09	5
					1
4.01E+08	3.21E+08	3.9E+08	3.94E+08	5.43E+08	10
2.1E+09	2.08E+09	1.67E+09	1.51E+09	2E+09	16
33254000	36621000	26853000	32564000		3
60362000					2
4.99E+08	7.73E+08	7.02E+08	3.23E+08	3.62E+08	6
	1.49E+08				4
1.35E+09	1.21E+09	1.83E+09	1.86E+09	1.46E+09	13
7.86E+08	7.8E+08	1.72E+09	1.97E+09	1.35E+09	7
6.59E+08	5.75E+08	2.87E+08	2.62E+08	3.56E+08	4
1.7E+09	1.85E+09	1.87E+09	1.97E+09	1.69E+09	10
					1
					1
6.16E+08	6.66E+08	4.42E+08	6.71E+08	6.04E+08	15
64814000	73678000	82215000	72294000	81041000	7
1.53E+08	2.03E+08	1.14E+08	91333000	1.26E+08	6
1.67E+08	1.82E+08	1.1E+08		1.06E+08	3
3.88E+08	3.6E+08	4.5E+08	3.62E+08	3.86E+08	4
1.16E+08	78167000	77824000	89112000	93299000	3
2.08E+08		1.13E+08	1.77E+08	1.27E+08	3
					2
			45773000	47921000	5
4.14E+08	3.33E+08	3.74E+08	3.87E+08	2.9E+08	3
					1
2.54E+08	2.3E+08	2.15E+08	1.85E+08	2.47E+08	8

5.11E+08	6.24E+08	3.55E+08	3.61E+08	3.58E+08	3
1.88E+08	1.47E+08	2.09E+08	2.9E+08	2.41E+08	5
52555000	60702000	44923000	53089000	38112000	5
1.3E+08	1.23E+08	1.96E+08	1.09E+08	1.21E+08	4
					1
2.75E+08	2.81E+08	2.82E+08	2.97E+08	2.62E+08	5
76051000					5
					1
2.59E+08	2.02E+08	3.59E+08	3.43E+08	3.5E+08	13
					1
					3
11597000		47366000	21933000	54943000	5
3.16E+08	2.41E+08	1.7E+08	2.42E+08	3.51E+08	7
4.59E+08	4.23E+08	4.52E+08	4.76E+08	4.34E+08	6
3.06E+08	3.25E+08	3.16E+08	3.44E+08	3.38E+08	11
3.39E+08	3.48E+08	2.23E+08	2.49E+08	2.14E+08	15
25881000			28643000		4
8.05E+08	5.48E+08	1.04E+09	1.26E+09	8.03E+08	6
1.14E+08	1.17E+08	1.63E+08	1.92E+08	2.38E+08	4
1.14E+08	1.21E+08	1.09E+08	1.3E+08	1.13E+08	5
6.51E+08	7.11E+08	8.06E+08		6.76E+08	8
7.4E+08		8.81E+08	9.64E+08	1.13E+09	1
3.47E+08	4.32E+08	3.26E+08	3.7E+08	3.62E+08	5
					1
94893000	86013000	1.67E+08	1.15E+08	1.33E+08	6
					3
					3
94507000	76465000	63246000	66414000	62130000	3
					2
					2
1.59E+08	1.27E+08	1.29E+08	1.21E+08	1.37E+08	3
4.37E+08	4.6E+08	4.16E+08	5.53E+08	4.68E+08	5
1.27E+10	1.12E+10	1.17E+10	1.29E+10	9.64E+09	13
					2
1.1E+08	1.39E+08	1.18E+08	93013000	92483000	3
1.08E+09	1.26E+09	9.74E+08		8.4E+08	1
3.88E+08	3.11E+08	2.55E+08	2.74E+08	2.41E+08	7
49901000	50321000	53220000	49620000	53591000	5
1.84E+08	2.46E+08	1.71E+08		1.04E+08	3
					1
2.78E+08	2.05E+08	1.94E+08	2.81E+08	2.54E+08	9
4.25E+08	5.09E+08	2.59E+08	4.73E+08	4.99E+08	11
1E+09	1.07E+09	8.83E+08	8.54E+08	8.04E+08	13
5.4E+08	6.14E+08	5.05E+08	5.8E+08	5.63E+08	4
6.36E+08	6.05E+08	7.02E+08		6.14E+08	5
		42884000			5
3.88E+08	4.19E+08	4.05E+08	5.73E+08	3.76E+08	6
54347000		57505000	50893000	54096000	3
					1
19680000	54486000			27437000	3
1.26E+08	1.37E+08	1.16E+08	1.28E+08	98560000	5

5.52E+08	5.71E+08	4.53E+08	2.76E+08	2.92E+08	5
					1
52574000	1.02E+08	54314000	33354000	46077000	5
3.74E+08	4.13E+08	4.37E+08	4.28E+08	3.98E+08	11
4.08E+08		4.92E+08	4.34E+08	2.54E+08	4
2.2E+08	2.44E+08	2.57E+08	2.71E+08	2.32E+08	9
4.63E+08	4.85E+08	4.61E+08	4.46E+08	3.63E+08	11
1.19E+08	1.38E+08	1.43E+08	1.41E+08	2.98E+08	6
1.02E+08			1.14E+08	1.03E+08	4
85789000	84518000	83348000	83176000	56571000	3
					2
			95670000		3
					3
73896000	1.11E+08	2.27E+08	74822000	83269000	5
					1
					1
1.25E+08	1.14E+08	1.31E+08	1.33E+08	1.34E+08	11
					2
1.08E+09	8.45E+08	1.1E+09	8.86E+08	1.09E+09	7
					2
					3
4.61E+09	5.22E+09	8.41E+09	6.5E+09	5.83E+09	9
1.44E+08	1.23E+08	1.37E+08	96106000	1.09E+08	11
9.72E+08	1.04E+09	6.73E+08	7.96E+08	5.38E+08	3
					1
1.02E+09	1.01E+09	6.67E+08	8.87E+08	8.01E+08	5
1.72E+09	1.67E+09	9.66E+08	9.94E+08	1.02E+09	1
					1
2.2E+08	1.83E+08	1.79E+08	1.9E+08	1.93E+08	9
					2
40940000	34059000	41202000	55299000	41382000	2
					2
86414000	1.26E+08	1.31E+08	1.35E+08	1.17E+08	12
	87738000				2
34569000	29494000	36267000	23747000	31132000	3
83776000	1.09E+08		1.05E+08	79217000	5
	70207000				3
					1
3.71E+08	4.97E+08	2.75E+08	2.75E+08	3.01E+08	18
47829000	42431000		49304000		2
					2
2.64E+08	2.03E+08	2.95E+08	2.2E+08	2.6E+08	9
1.93E+08	2.43E+08	1.72E+08	1.86E+08	2.05E+08	4
2.09E+08	2.73E+08	2.35E+08	2.59E+08	2.81E+08	4
47450000					2
					1
2E+08	1.44E+08	1.33E+08	1.71E+08	1.59E+08	3
			18587000		5
					5
80034000	84870000	19785000	27015000	1E+08	6
5.15E+08	3.56E+08	4.59E+08	6.06E+08	6.98E+08	7

3.92E+08	2.45E+08		2.31E+08		4
					2
25637000	18376000		48326000		4
7.22E+09	5.93E+09	3.96E+09	4.27E+09	5.52E+09	21
89460000	1.07E+08	89315000	1.13E+08	1.15E+08	4
2.11E+08					2
					2
		2.52E+08	3.61E+08	2.56E+08	2
1.34E+08	1.46E+08	1.03E+08	99162000	76253000	4
					1
1.9E+08	1.35E+08	1.51E+08			6
65588000	64700000	50728000	53780000	43014000	10
1.41E+08	1E+08	2.23E+08	1.35E+08	1.15E+08	5
2.69E+08	2.71E+08	2.45E+08	2.88E+08	2.9E+08	10
					1
					3
2.88E+08	2.26E+08	2.11E+08	2.31E+08	1.97E+08	8
1.57E+08	1.2E+08	1.2E+08	1.04E+08	1.6E+08	11
					1
	18227000				5
1.29E+08	1.23E+08	1.05E+08	1.27E+08	1.31E+08	9
1.12E+09	7.76E+08	1.02E+09	3.84E+08	7.38E+08	7
60477000	74436000	50840000	74096000	91637000	4
1.79E+09	1.57E+09	1.47E+09	1.73E+09	1.76E+09	7
2.06E+09	1.68E+09	1.53E+09	1.35E+09	1.31E+09	5
2.93E+08		2.32E+08	2.93E+08		3
44465000	61187000			56964000	3
					4
					3
2.7E+08	2.47E+08	2.43E+08	2.87E+08	2.59E+08	6
2.35E+08	1.64E+08	2.43E+08	3.17E+08	1.92E+08	11
1.24E+08	6.19E+08	1.61E+08	1.94E+08	5.13E+08	4
					1
7.02E+09	7.92E+09	1.13E+10	1.3E+10	8.94E+09	19
3.15E+08	3.99E+08	2.76E+08	3.03E+08	3.09E+08	7
98721000	1.42E+08	82385000	1.03E+08	85483000	2
					1
6.12E+08	6.98E+08	7.37E+08	6.58E+08	8.53E+08	13
77749000	77738000		73512000	52824000	4
2.27E+08	1.67E+08	1.35E+08	1.76E+08	1.72E+08	7
2.54E+09	3.92E+09	2.24E+09	2.76E+09	2.24E+09	5
					2
					1
56434000	54941000	64108000	50320000	43181000	5
					1
	35341000	28764000	25268000	25063000	4
					1
					1
					2
					1
1.34E+08	1.22E+08	1.39E+08	1.03E+08	1.14E+08	12

28069000		36782000		40903000	4
					2
					1
			23153000	23993000	4
74436000	1.04E+08	17739000	28623000	58093000	6
1.04E+09	1.51E+09	1.05E+09	5.94E+08	8.23E+08	2
1260600	2347700			1263600	2
69653000		60826000	41438000	73738000	3
2E+09	1.73E+09	1.97E+09	1.77E+09	1.83E+09	22
4.84E+08	5.09E+08	3.1E+08	2.17E+08	2.71E+08	9
2.05E+09	2.17E+09	2.31E+09	2.63E+09	2.03E+09	13
					2
					2
					1
23304000	24347000				4
67339000				49174000	4
					1
					3
3.13E+09	2.61E+09	3.28E+09	3.4E+09	3.36E+09	22
			5.64E+08	5.49E+08	2
1.67E+08	1.49E+08	2.12E+08	2.85E+08	2.14E+08	9
2.84E+08	2.39E+08	3.6E+08	3.64E+08	3.69E+08	5
83326000	48494000	40807000	81509000	82685000	4
					2
2.55E+08	2.42E+08	1.97E+08	2.27E+08	1.74E+08	9
2.23E+08	2.48E+08	2.53E+08	2.05E+08	1.89E+08	5
2.69E+08	1.13E+08	69914000	1.14E+08	2.15E+08	4
					3
4.08E+09	3.95E+09	4.06E+09	4.35E+09	3.42E+09	17
1.33E+08	1.25E+08	1.44E+08	1.2E+08	1.17E+08	8
2.23E+08	1.98E+08	2.01E+08	2.14E+08	2.09E+08	8
					2
3.01E+08	2.06E+08				7
5.03E+08	5.85E+08	3.02E+08	2.73E+08	4.4E+08	6
78871000	74490000			78155000	4
89685000	77750000	72751000	84723000	81750000	4
			67074000	95566000	4
8.12E+08	7.77E+08	7.84E+08	7.69E+08	6.93E+08	8
					1
					1
2.97E+08	3.33E+08	3.4E+08	3.15E+08	3.56E+08	4
1.51E+08	1.96E+08	1.43E+08	1.2E+08	1.3E+08	6
2.45E+08	1.69E+08	1.99E+08	1.94E+08	2.09E+08	5
	58588000	68235000	81571000	64178000	6
1.96E+09	1.26E+09	2.39E+09	1.91E+09	2.68E+09	10
1.08E+08	90773000	1.24E+08	98971000	95248000	3
2.51E+08	2.49E+08	1.43E+08	1.54E+08	1.61E+08	6
4.24E+09	3.61E+09	3.96E+09	3.56E+09	3.63E+09	29
					1
1.82E+09	1.75E+09	1.22E+09	1.28E+09	1.25E+09	10
					2

2.45E+09	2.14E+09	2.9E+09	3.24E+09	2.7E+09	7
6.18E+08	6.45E+08	1.3E+09	1.02E+09	1.14E+09	12
1.12E+09	7.7E+08	3.98E+08	9.38E+08	1.27E+09	10
6.73E+08	8.37E+08	5.29E+08	4.36E+08	5.01E+08	14
					1
2.11E+08	2.05E+08	1.01E+08		1.55E+08	6
					3
					2
2.13E+08	2.76E+08	1.72E+08	1.54E+08	1.49E+08	3
					1
9.3E+08	1.06E+09	6.05E+08	5.75E+08	7.88E+08	11
					1
					2
4.77E+08	5.25E+08	6.44E+08	5.07E+08	5.27E+08	9
					2
3.57E+09	3.1E+09	3.78E+09	4.1E+09	2.86E+09	13
1.75E+09	1.57E+09	2.41E+09	2.33E+09	1.99E+09	16
3.41E+09	2.81E+09	2.51E+09	2.44E+09	2.64E+09	14
					2
2.66E+08	2.51E+08	2.8E+08	3.93E+08	3.97E+08	6
79810000	1.21E+08	1.5E+08	74962000	77652000	5
1.39E+10	1.5E+10	1.08E+10	1.33E+10	1.16E+10	11
					2
	62976000	66169000		79855000	4
2.7E+08	2.75E+08	2.93E+08	3.2E+08	2.54E+08	3
2.39E+08	1.9E+08	1.76E+08	1.79E+08	1.72E+08	9
2.44E+08	3.02E+08	2.71E+08	2.44E+08	2.46E+08	2
55484000	47424000			47642000	5
4.94E+08	5.28E+08	3.19E+08	3.56E+08	3.55E+08	7
3.35E+08	3.2E+08	4.88E+08	4.84E+08	5.21E+08	9
1.45E+08	1.48E+08	1.65E+08	76748000	1.22E+08	3
					4
4.51E+08	2.95E+08	4.79E+08	4.92E+08	4.34E+08	6
51974000	57739000	46686000	55603000	47524000	9
3.22E+08	4.96E+08				2
					2
					3
					1
2.5E+08	2.58E+08		2.49E+08	2.12E+08	6
1.13E+08	1.97E+08	67358000	46548000	1.04E+08	4
4.14E+09	2.98E+09	3.49E+09	3.47E+09	3.46E+09	22
1.69E+08	1.31E+08	2.23E+08	1.97E+08	1.66E+08	5
4.88E+08	4.5E+08	5.52E+08	5.06E+08	4.51E+08	6
					1
1E+08	98604000	1.23E+08	1.04E+08	1.46E+08	6
					3
	1.06E+08		1.11E+08	1.06E+08	3
6.98E+08	6.11E+08	6.08E+08	6.41E+08	5.42E+08	25
	88705000				2
					2
67743000					2

					1
					1
87078000	1.1E+08	61574000	71538000	73733000	4
5.04E+08	3.68E+08	5.11E+08	5.22E+08	5.01E+08	6
4.41E+08	5.92E+08	1.72E+08		2.84E+08	3
		2.97E+08	2.17E+08	2.86E+08	4
	63028000	59402000	48547000	65342000	7
				20785000	2
					1
62322000	77959000	57906000	55226000	58599000	4
7.83E+09	8.49E+09	5.42E+09	6.31E+09	5.01E+09	9
1.91E+09	2.27E+09	1.31E+09	1.61E+09	1.3E+09	6
					3
1.73E+08	1.59E+08	1.43E+08	1.45E+08	1.33E+08	3
94625000		92754000		1.02E+08	6
70321000	71537000	64493000		49399000	5
					2
6.31E+08	5.31E+08	5.64E+08	6.36E+08	5.91E+08	4
					1
					2
1E+08	74195000	61910000	1.02E+08	54857000	7
	1.19E+08	90480000	92128000	1.54E+08	2
1.87E+09	1.64E+09	1.23E+09	8.19E+08	1.71E+09	7
3.35E+08	3.26E+08	2.91E+08	2.73E+08	2.82E+08	10
2.39E+08	1.85E+08	2.17E+08	2.63E+08	2.46E+08	5
1.1E+08	1.19E+08	1.41E+08	1.63E+08	1.68E+08	4
4.36E+08	4.11E+08	5.01E+08	6.09E+08	5.44E+08	4
1.3E+08	1.18E+08	1.05E+08	1.03E+08	1.13E+08	7
1.51E+09	1.47E+09	8.21E+08	6.47E+08	9.73E+08	6
1.95E+08					6
					1
57872000	81045000	98065000			5
					3
2.74E+08	3.3E+08	5.42E+08	3.49E+08	4.02E+08	3
					1
1.45E+09	1.13E+09	2.04E+09	2.09E+09	1.83E+09	18
					1
1.54E+08	1.49E+08	1.72E+08	2.35E+08	1.34E+08	5
80028000	84465000			63367000	3
					1
3.79E+08	3.56E+08	3.42E+08	3.6E+08	3.21E+08	12
					1
2.38E+08	1.47E+08	1.91E+08	1.62E+08	2.26E+08	6
					1
3.18E+08	2.61E+08	2.75E+08	2.99E+08	2.82E+08	7
9.87E+08	7.43E+08	8.67E+08	9.12E+08	9.39E+08	17
4.91E+08	4.45E+08	4.39E+08	3.44E+08	4.81E+08	13
48329000		63613000		50056000	2
1.53E+08	1.54E+08	1.09E+08	1.02E+08	94145000	5
7.86E+08	9E+08	5.19E+08	5.71E+08	4.35E+08	6
					1

21878000		17779000		20003000	5
1.5E+08	2.04E+08	1.47E+08	1.49E+08	1.49E+08	8
96384000	78529000	1.45E+08	1.16E+08	1.11E+08	3
1.57E+08		1.92E+08	2.02E+08	1.68E+08	3
2.97E+08	2.67E+08	3.21E+08	3.24E+08	2.83E+08	6
1.37E+08		1.22E+08			2
4.32E+08	4.65E+08	4.71E+08	4.43E+08	4.11E+08	10
65274000	45413000	34564000	44821000	38953000	2
69151000	45471000	56864000	26575000	33477000	4
					2
	24078000	18363000		41293000	3
67598000	51145000			65534000	2
1.84E+09	1.45E+09	1.58E+09	2.03E+09	1.76E+09	10
1.65E+09	1.28E+09	1.61E+09	1.76E+09	1.74E+09	10
91555000	1.4E+08	1.47E+08	1.09E+08	1.69E+08	7
5.67E+08	5.79E+08	6.81E+08	6.24E+08	5.75E+08	6
6.92E+08	6.92E+08	6.19E+08	7.44E+08	7.26E+08	5
					1
					2
1.07E+08		75525000	65101000		4
43017000	43715000	45658000	47494000		6
68521000	57316000	80753000	77843000	90421000	6
2.83E+08	2.43E+08	1.51E+08	1.76E+08	1.59E+08	7
					1
	3.7E+08				4
					3
51046000	35659000	42986000	31369000	40927000	5
1.83E+09	1.81E+09	1.6E+09	1.77E+09	1.65E+09	5
					1
	44613000			42142000	4
2.65E+08		2.75E+08	2.63E+08	2.79E+08	2
					1
		33112000		48594000	4
					1
2.01E+09	2E+09	2.06E+09	1.82E+09	1.77E+09	7
7.85E+09	3.28E+09	4.25E+09	4.7E+09	5.88E+09	1
2E+09	2.68E+09	1.94E+09	1.41E+09	1.87E+09	10
62083000		74541000	84096000	79250000	2
19432000					2
93220000	1.36E+08		64943000	48958000	5
57454000	57273000	60599000	59027000	61880000	13
					8
17161000		11574000		18167000	2
	1.56E+08			1.45E+08	1
39861000	47088000			26948000	3
1.13E+08	1.63E+08	1.24E+08	98445000	1.56E+08	3
					1
96338000	88484000	74556000	82074000	69896000	2
		1.67E+08			1
					2
3E+08			1.17E+08		2

					2
					1
					1
18330000					4
	5.02E+08	4.02E+08		4.61E+08	2
39062000	28619000				3
					1
3.1E+08	1.9E+08	2.5E+08	2.39E+08	1.91E+08	3
2.45E+08	2.07E+08	1.92E+08	1.93E+08	1.76E+08	2
					2
96560000	98840000	1.51E+08	1.85E+08	1.53E+08	5
		30078000	36485000		3
					2
1.41E+08	1.72E+08	1.24E+08	1.25E+08	1.17E+08	6
6.05E+08	8.23E+08	9.37E+08	8.05E+08	7.46E+08	3
1.02E+08	94786000	1.2E+08	97837000	1.3E+08	1
					1
1.05E+08	1E+08	1.04E+08	72090000	98458000	3
1.47E+08	1.23E+08	1.42E+08	1.49E+08	1.45E+08	10
40198000				67927000	5
51539000					3
1.41E+08	1.72E+08	1.37E+08	1.83E+08	1.53E+08	5
1.25E+08	1.81E+08	1.75E+08	1.61E+08	1.75E+08	6
3.41E+08	3.6E+08	2.18E+08	2.29E+08	2.65E+08	2
	2.52E+08				5
1.07E+09	1.07E+09	9.47E+08	1.09E+09	9.68E+08	36
					3
					1
6.7E+08	1.16E+09	5.55E+08	6.27E+08	5.84E+08	4
3.71E+08	3.2E+08	3.34E+08	2.97E+08	2.86E+08	10
1.07E+08	1.1E+08	1.41E+08	1.9E+08	1.53E+08	5
2.96E+08	5.94E+08	5.26E+08	5.02E+08	7.4E+08	4
					1
					1
					4
					1
1.75E+08	1.34E+08	1.29E+08	1.2E+08	1.16E+08	5
1.53E+08	2.53E+08	95856000	1.19E+08	1.54E+08	5
					1
		13255000			1
1.75E+08	1.72E+08	1.82E+08	1.85E+08	1.97E+08	11
2.87E+09	3.1E+09	2.14E+09	2.03E+09	2.16E+09	12
					2
1.22E+08	1.15E+08	82436000	1.03E+08	79861000	5
4.15E+08	4.88E+08	3.49E+08	3.58E+08	3.82E+08	4
3.1E+08	2.15E+08	3.94E+08	2.45E+08	2.6E+08	6
95863000	1.59E+08				2
77107000	81750000	59083000	75284000	82807000	3
36469000				36274000	3
89503000	95351000	1.04E+08		88197000	2
9.15E+08	7.77E+08	8.5E+08	1.01E+09	9.06E+08	9

					2
		16490000			5
87091000	97786000		92870000	49074000	4
2.13E+08	2.2E+08	1.78E+08	1.55E+08	1.79E+08	6
88083000	1E+08	59230000	72449000	77759000	5
1.23E+08	1.15E+08	1.33E+08	1.76E+08	1.12E+08	6
		69269000	67871000	61182000	2
3.35E+08	4.26E+08	3.59E+08	3.28E+08	3.3E+08	9
1.39E+08	1.49E+08	1.47E+08	1.32E+08		3
3.24E+09	2.97E+09	4.13E+09	3.71E+09	3.76E+09	14
2.94E+08	3.23E+08	2.07E+08	3.02E+08	3.75E+08	9
1.06E+08	1.01E+08	98601000	1.06E+08	90222000	7
1.81E+08	1.01E+08	1.14E+08	1.3E+08	1.07E+08	5
					4
					4
6E+08	8.07E+08	8.6E+08	8.48E+08	6.92E+08	4
82428000	96995000	68141000	85478000	81578000	3
1.61E+08	1.95E+08	2.02E+08	2.15E+08	1.83E+08	3
1.34E+09	1.35E+09	1.36E+09	1.45E+09	1.38E+09	9
					2
					1
					2
1.18E+08	95730000	1.64E+08	1.6E+08	1.34E+08	11
1.27E+08	1.04E+08	1.39E+08	1.69E+08	1.87E+08	10
44332000	51365000	47447000	61000000	46884000	6
					4
					1
					2
					1
					1
40381000	37658000		38138000	33741000	2
57692000			68916000		4
1.62E+09	1.41E+09	2.07E+09	2.44E+09	2.02E+09	21
					1
	1.46E+08	60459000	1.42E+08		6
47322000	26587000			30733000	3
2.22E+08	1.69E+08	4.31E+08	2.63E+08	2.94E+08	9
63058000			53962000		2
					2
2.44E+08	2.2E+08	2.09E+08	2.16E+08	2.02E+08	13
		69158000	61965000	60897000	1
42231000	36216000	35407000	43094000	44939000	2
1.64E+09	1.03E+09	6.31E+08	7.51E+08	1.48E+09	4
					2
34822000	19746000	52500000	58103000	33084000	7
64485000	41928000	56829000	68843000	56971000	7
2.1E+08	1.8E+08	1.6E+08	1.74E+08	2.17E+08	3
		76421000	72527000	95042000	2
					1
					2
					1

2.66E+08	2.51E+08	3.04E+08	3.29E+08	3.09E+08	11
					3
1.5E+08		2.05E+08	3.47E+08	1.89E+08	6
					3
2.4E+08	2.28E+08	2.44E+08	1.96E+08	1.98E+08	6
6.3E+08	6.01E+08	5.84E+08	6.08E+08	6.93E+08	8
4.61E+08	5.85E+08	4.95E+08	4.65E+08	4.96E+08	12
					1
6.1E+08	4.39E+08	5.07E+08	5.07E+08	5.81E+08	3
					1
		32488000	44841000		1
1.67E+08	1.92E+08	1.7E+08	1.59E+08	1.51E+08	8
1.22E+08	39546000	25032000	1.06E+08		4
					2
		4.14E+08			3
68979000		70214000	45865000	53654000	3
1.11E+09	9.73E+08	7.08E+08	7.66E+08	8.34E+08	6
					2
					3
					3
2.66E+08	2.89E+08	1.52E+08	2.01E+08	1.58E+08	3
34962000	62056000	22905000	22125000	16318000	2
2.18E+08	1.7E+08	3.55E+08	2.48E+08	2.55E+08	7
			37185000		1
56157000	47847000	1.29E+08	1.76E+08	98324000	8
4.27E+08	5.8E+08	2.36E+08	3.19E+08	2.83E+08	1
				55717000	1
31361000	22282000	28377000	26788000	32113000	7
					1
					3
					1
					1
62586000	56007000			61465000	4
					3
79560000	95039000	80237000	81596000	80403000	5
3.18E+08	2.45E+08	1.27E+08	1.47E+08	2.09E+08	11
					1
65107000					3
	11766000				1
1.3E+08	1.71E+08	2.17E+08	5.39E+08	1.72E+08	4
1.8E+08	1.66E+08	93313000	1.13E+08	90208000	13
					1
50691000		43330000		36824000	3
35852000	28594000	26868000	28489000	38887000	4
3.04E+08	2.19E+08	2.6E+08	2.71E+08	2.9E+08	10
2.83E+08	2.08E+08	2.02E+08	2.08E+08	2.85E+08	2
7.06E+08	6.89E+08	5.6E+08	4.02E+08	5.6E+08	5
1.79E+09	1.6E+09	2.23E+09	2.75E+09	2.18E+09	26
2.24E+08	1.92E+08	1.63E+08	1.79E+08	1.55E+08	7
					2
1.73E+08	2.04E+08	1.67E+08	2.74E+08	1.76E+08	2

		43528000			3
					7
1.1E+08	1.46E+08	1.04E+08	1.2E+08	1.25E+08	5
		2.78E+08			4
3.39E+09	2.48E+09	3.18E+09	3.47E+09	2.51E+09	33
5.12E+08	3.58E+08	4.86E+08	6.53E+08	7.73E+08	3
4.18E+08	3.48E+08	6.92E+08	6.49E+08	5.5E+08	4
1.09E+08	95154000	1.01E+08	1.12E+08	1.07E+08	6
					1
					1
1.2E+09	1.53E+09	1.46E+09	1.46E+09	1.43E+09	18
8.92E+08	1.22E+09	6.17E+08	9.74E+08	8.31E+08	5
2.24E+08	2.59E+08	1.84E+08	2.3E+08	1.97E+08	9
46365000			41502000	35194000	3
1.36E+08	2.8E+08	3.18E+08	3.29E+08	2.72E+08	3
					4
56926000	51987000	67428000		41517000	3
46514000	64579000	28461000	35004000	35272000	2
1.09E+08	1.14E+08	76148000	77862000	68074000	3
1.83E+08	2.53E+08	82610000	1.22E+08	2.55E+08	5
					2
					1
2.96E+08	2.59E+08	3.34E+08	3.66E+08	3.44E+08	11
9341800	29091000				1
3.96E+08	3.56E+08	4.09E+08	3.64E+08	4.58E+08	14
1.3E+08	92583000	1.28E+08	91533000	1.04E+08	8
97350000	61731000	52421000	63253000	74532000	7
					1
					3
					3
					1
2.18E+08	1.55E+08	2.13E+08	2.14E+08	1.89E+08	9
					2
54720000	57858000	64754000			4
1.32E+08	1.22E+08	1.46E+08	1.19E+08	1.42E+08	3
2.82E+08	3.44E+08	4.04E+08	4.04E+08	4.18E+08	8
					4
34534000		37107000	40564000	35697000	9
1.77E+09	2.26E+09	1.73E+09	1.94E+09	1.74E+09	20
3.72E+08	2.85E+08	2.27E+08	2.91E+08	2.5E+08	4
66895000	52652000	1.12E+08	1.44E+08	64960000	5
53442000	42501000			27300000	2
1.23E+08	95873000		1.52E+08		8
3.58E+08	4.1E+08	2.85E+08	2.63E+08	2.22E+08	4
					2
					1
		74599000	1.02E+08		4
76860000	66310000		54527000	58859000	4
4.57E+08	4.98E+08	8.07E+08	5.95E+08	5.07E+08	5
32238000	36753000	55220000	47099000		6
1.25E+09	1.27E+09	1.99E+09	2.28E+09	1.8E+09	22

1.56E+08	1.68E+08	1.09E+08	1.45E+08	2.28E+08	6
1.95E+08	2.13E+08	1.52E+08	1.89E+08	1.91E+08	3
					1
26486000					2
1.44E+08	1.31E+08	1.35E+08	1.88E+08	1.63E+08	3
					1
					1
					1
1.24E+09	7.69E+08	1.38E+09	1.44E+09	1.59E+09	5
1.97E+08	3.22E+08	2.12E+08	2.21E+08	2.21E+08	6
2.7E+08	2.22E+08	2.95E+08	3.31E+08	3.72E+08	11
					1
3.24E+09	2.78E+09	3.02E+09	3.62E+09	3.22E+09	17
	1.19E+08	1.1E+08	96121000	89381000	4
4.21E+08	5.13E+08	5.28E+08	5.8E+08	5.49E+08	2
1.26E+09		7.88E+08	1.53E+09		3
		30799000			3
					3
1.34E+08		1.4E+08	1.16E+08	1.26E+08	4
48471000	57589000			45963000	3
					1
					2
93518000	97637000	1.02E+08	1.13E+08	84327000	4
		40408000	47144000		2
75905000	83382000		70976000		3
	5.21E+08	6.23E+08			4
2.4E+08	1.67E+08	2.12E+08		2.26E+08	6
					1
			48388000		4
				38531000	4
4.48E+08	4.44E+08	4.03E+08	4.89E+08	4.6E+08	3
67452000	67394000				3
3.78E+08	3.22E+08	3.96E+08	4.64E+08	4.23E+08	27
5.36E+08	5.45E+08	3.8E+08	4.15E+08	4.9E+08	8
					2
50352000		37836000	35766000		5
					1
1.47E+08	1.65E+08		1.33E+08		2
1.08E+08	47966000	1.28E+08	1.49E+08	1.8E+08	3
72575000	75302000	75913000	61818000	87553000	8
1.5E+08			1.25E+08		1
1.92E+08	1.64E+08		1.48E+08	1.52E+08	3
4.33E+08	5.68E+08	5.08E+08	2.67E+08	6.26E+08	8
					3
				40528000	5
		5.16E+08	5.23E+08	5.07E+08	2
			1.09E+08		2
1.98E+09	1.65E+09	1.38E+09	1.52E+09	1.33E+09	12
					3
					2
					3

2.12E+08	2.07E+08	1.73E+08	1.99E+08	1.96E+08	5
51573000	48198000	74452000	1.01E+08	61965000	4
4.57E+08	4.15E+08	4.86E+08	4.52E+08	3.28E+08	6
4.48E+08	5.33E+08	3.82E+08		8.59E+08	7
39917000		85900000	1.39E+08	1.14E+08	4
1.26E+08		1.85E+08	1.72E+08	1.92E+08	4
4.09E+08	3.77E+08	3.74E+08	4.26E+08	3.71E+08	10
7.57E+08	7E+08	6.66E+08	7.07E+08	7.53E+08	5
8.45E+08	6.26E+08	4.64E+08	4.72E+08	9.13E+08	11
6.41E+08	3.67E+08	1.09E+09	1.06E+09	8.37E+08	3
					2
4.04E+08	3.88E+08	3.2E+08	1.77E+08	2.91E+08	6
	55757000				3
36205000	32755000		29007000	37219000	3
69414000	89081000	62356000		74925000	6
7.17E+08	7.8E+08	8.34E+08	7.68E+08	7.32E+08	6
66284000	63007000	73638000	72385000	79454000	2
81676000	58166000	87947000	1.08E+08	94774000	5
1.25E+08	1.54E+08	1.45E+08	1.91E+08	1.53E+08	7
1.67E+09	1.24E+09	6.33E+08	7.72E+08	1E+09	4
2.21E+08	1.68E+08	1.76E+08	1.91E+08	1.85E+08	2
				44293000	4
1.98E+09	2.06E+09	1.95E+09	1.73E+09	1.86E+09	15
4.67E+08	5.14E+08	5.17E+08	5.51E+08	4.55E+08	5
1.34E+08	1.09E+08	1.28E+08	1.03E+08	1.08E+08	2
2.05E+08	2.99E+08	2.44E+08	2.45E+08	2.55E+08	7
1.77E+08	1.5E+08	1.68E+08	1.97E+08	2E+08	26
					2
6.22E+08	5.21E+08	5.1E+08	5.3E+08	5.29E+08	2
8.59E+08	7.19E+08	8.72E+08	1.09E+09	9.46E+08	23
1.04E+08	1.27E+08	94799000	90062000	1.06E+08	7
5.61E+08	5.96E+08	4.69E+08	4.39E+08	5.22E+08	9
					1
			42385000	38940000	2
1.83E+08	1.32E+08		1.35E+08		3
					1
					3
					2
42223000	61027000	67761000	55765000	76728000	4
76581000	76861000	1.29E+08	91394000	1.16E+08	4
2.62E+08	2.31E+08	2.03E+08	2.94E+08	2.47E+08	4
3.05E+08	2.86E+08	2.98E+08	3.12E+08	3.16E+08	9
4.76E+08	4.2E+08	5.18E+08	5.47E+08	5.6E+08	8
6.49E+08	8.06E+08	5.01E+08	4.5E+08	5.3E+08	15
1.11E+08	93684000	1.12E+08	1.43E+08	1.11E+08	8
1.7E+08	1.53E+08	1.89E+08	2.23E+08	2.39E+08	3
56304000	45751000				4
1.81E+08	2.09E+08	1.71E+08	2.19E+08	1.95E+08	4
2.81E+08	3.53E+08	2.66E+08	3.53E+08	3.78E+08	7
3.22E+08	4.15E+08	4.24E+08	4.34E+08	3.44E+08	3
					1

5.06E+08	6.31E+08	6.01E+08	6E+08	6.05E+08	11
		92065000	48977000	47184000	2
					1
	80144000	61596000	62565000	47598000	2
2.09E+08	2.24E+08	1.8E+08	2.43E+08	2.79E+08	7
2.4E+09	2.11E+09	2.67E+09	2.66E+09	2.96E+09	19
3.05E+08	2.65E+08	2.83E+08	3.17E+08	3.2E+08	8
	88683000	61651000			5
3.95E+08	3.77E+08	4.31E+08	3.8E+08	4.95E+08	6
				89278000	5
6.58E+08	7.61E+08	1.03E+09	6.96E+08	7.87E+08	10
66695000	70253000	73172000	79196000	60471000	7
2.28E+08	1.95E+08	2.71E+08	2.81E+08	2.98E+08	5
1.1E+09	9.71E+08	9.02E+08	1.01E+09	9.16E+08	10
50238000	47431000	39711000	43245000	43212000	3
88307000	85126000	1.46E+08	1.86E+08	1.24E+08	4
	43093000		37483000	55529000	3
		1.84E+08		2.29E+08	5
50236000	28875000	70930000	91885000	93148000	12
35470000		64951000	67155000	85096000	11
		84033000	57164000	1.18E+08	10
					2
					2
42013000	48221000	57623000	45916000	48442000	1
					4
33450000				45133000	1
				3.12E+08	5
1.09E+08	86386000	1.06E+08	89559000	1.33E+08	8
1.12E+09	9.59E+08	1.71E+09	1.29E+09	1.09E+09	6
52963000					3
4.36E+08	3.29E+08	5.93E+08	5.34E+08	4.69E+08	11
					2
					2
3.4E+08	2.54E+08	3E+08	3.32E+08	3.21E+08	20
					3
					1
2.98E+08	1.92E+08	2.01E+08	2.47E+08	2.22E+08	5
3.36E+09	3.33E+09	2.64E+09	1.93E+09	2.13E+09	15
73314000	70374000	60666000	67964000	1.12E+08	5
4.7E+08	4.23E+08	4.41E+08	3.16E+08	3.37E+08	3
					2
45911000	55823000		29661000	39681000	4
1.65E+08	2.3E+08		1.59E+08	1.61E+08	2
9.43E+08	1.17E+09	9.57E+08	1.06E+09	1.03E+09	18
					2
5.65E+08	4.39E+08	4.55E+08	5.11E+08	3.4E+08	2
					1
1.27E+09	1.15E+09	1.45E+09	1.28E+09	1.7E+09	15
1.62E+08	1.5E+08	1.82E+08	2.37E+08	1.87E+08	7
5.04E+08	5.48E+08	1.4E+08	2.14E+08	3.25E+08	4
1.99E+09	2.14E+09	1.68E+09	1.29E+09	1.51E+09	17

78942000	77453000	82988000	68244000	69602000	7
1.12E+08	1.78E+08	87857000	93368000	1.01E+08	4
4.1E+08	3.64E+08	4.29E+08	4.14E+08	4.37E+08	8
					2
					6
39370000	50822000	34933000	37185000	39337000	2
46297000			13210000	71003000	3
4.14E+09	4.17E+09	3.43E+09	3.95E+09	3.41E+09	25
					3
	2.57E+08	2.39E+08	2.23E+08	2.09E+08	5
39347000	37997000	46379000	54029000	40542000	7
3.62E+08	3.35E+08	1.73E+08	1.48E+08	2.29E+08	14
30410000	37344000	41215000			4
96074000	1.06E+08	91469000	95344000	88533000	5
					1
4.37E+08	4.94E+08	5.12E+08	5.96E+08	6.02E+08	3
					1
					1
2.66E+08		2.17E+08			6
					4
5.43E+08	5.4E+08	4.29E+08	5.1E+08	4.19E+08	15
88225000	83621000	1.29E+08	97558000	90976000	5
3.21E+08	2.07E+08				3
36222000	39869000	40017000	28081000	36448000	8
		1.41E+08	1.08E+08	1.39E+08	6
5.28E+08	6.26E+08	4.33E+08	4.91E+08	3.92E+08	14
4.89E+08	4.83E+08	4.2E+08	4.15E+08	4.88E+08	7
1.07E+08	1.06E+08	1.09E+08	1.15E+08	1.04E+08	3
					1
					1
					4
					4
					1
2.57E+08	2.84E+08	2.28E+08	1.65E+08	1.87E+08	12
2.38E+08	2.73E+08	1.84E+08	1.69E+08	1.9E+08	4
4.58E+08	5.46E+08	3.21E+08	3.48E+08	3.11E+08	8
					2
1.07E+08					1
2.57E+08	2.36E+08	2.81E+08	2.98E+08	2.22E+08	7
7.19E+08	6.17E+08	7.02E+08	6.87E+08	7.27E+08	11
2.59E+08	1.7E+08	1.86E+08	1.29E+08	1.12E+08	12
3.08E+08	2.27E+08	1.93E+08	2.35E+08	2.04E+08	8
2.12E+08	1.8E+08	2.56E+08	2.61E+08	2E+08	3
					1
					1
75477000	67379000	95978000	1E+08	1.14E+08	6
99171000					3
1.75E+08	2.48E+08	1.41E+08	1.54E+08	1.53E+08	3
60087000	55981000	45158000	46403000	38109000	10
2.81E+08	3E+08	2.16E+08	2.32E+08	2.11E+08	12
1.69E+08	1.55E+08	1.51E+08	1.41E+08	1.57E+08	3

			1.88E+08		2
73489000	82972000	59019000	71351000	73487000	6
81660000	1.03E+08				2
4.98E+08	6.21E+08	5.23E+08	5.58E+08	5.99E+08	11
					1
7.31E+08	7.37E+08	2.14E+08	2.62E+08	2.35E+08	19
9.47E+08	1.04E+09	8.63E+08	5.42E+08	5.06E+08	4
	2.62E+08	3.33E+08	3.1E+08	2.7E+08	3
8.93E+08	1.17E+09	7.04E+08	5.96E+08	9.56E+08	5
		30306000	37227000		1
38533000	53837000	94482000	39391000		4
2.3E+08	2.37E+08	3E+08	3.31E+08	2.96E+08	4
		1.17E+08			4
6.23E+09	6.08E+09	5.46E+09	5.4E+09	5.14E+09	17
4.36E+08	4.77E+08	4.23E+08	3.94E+08	3.73E+08	6
2.27E+08	2.1E+08	2.81E+08	3E+08	2.9E+08	9
3.28E+08	3.34E+08	2.88E+08	2.86E+08	2.64E+08	9
3.16E+08	2.64E+08	6.34E+08	6.71E+08	5.68E+08	4
4.89E+08	5.99E+08	4.83E+08	5.58E+08	5.35E+08	5
					1
1.07E+08	1.15E+08	85859000	87394000	81064000	4
5.69E+08	4.33E+08	7.31E+08	7.1E+08	5.05E+08	4
63451000	70758000	96444000	75864000	55115000	6
					4
1.31E+08	1.94E+08	1.12E+08	1.13E+08	1.11E+08	5
					3
31116000	36858000	29104000	30901000	28223000	6
					1
1.73E+08	1.95E+08	2.06E+08	2.02E+08	1.73E+08	8
2.88E+08	3.41E+08	2.66E+08	2.12E+08	2.71E+08	5
					1
					2
3.51E+08		3.19E+08	4.68E+08	3.18E+08	3
					1
					1
					1
					1
					1
					1
					2
					1
			1.22E+08		4
					1
1.3E+08	1.34E+08	1.94E+08	1.66E+08	1.6E+08	7
					1
5.72E+08	4.93E+08	6.32E+08	4.45E+08	4.6E+08	8
					4
1.86E+09	1.31E+09	4.54E+08	5.37E+08	5.26E+08	6
1.48E+08	1.9E+08	1.21E+08	1.6E+08	1.57E+08	4
					3
					1

		97390000	54833000	94863000	3
40848000	45560000	81246000	63851000	47294000	4
					2
54430000	59703000	34800000	75405000	66480000	5
1.88E+08	1.89E+08	1.66E+08	1.55E+08	1.69E+08	5
99904000	1.06E+08	1.46E+08	1.56E+08	1.21E+08	10
32929000	34166000	27734000	31460000	35574000	3
					2
92592000					5
1.85E+08	1.93E+08	1.71E+08	1.82E+08	2.08E+08	8
	22465000	39333000	47570000	63981000	5
					2
88149000	81294000	1.16E+08	1.23E+08	1.09E+08	5
1.07E+08	1.26E+08	72827000	1.02E+08	1.81E+08	5
	43072000		79930000		5
					1
					2
51851000		56934000	47146000	46978000	2
					4
3.25E+08	2.46E+08	2.46E+08	3.18E+08	3.68E+08	4
3.53E+08	2.65E+08	3.91E+08	3.17E+08	3.63E+08	9
1.81E+09	1.62E+09	2.05E+09	2.24E+09	2.05E+09	14
					1
2.03E+09	2.56E+09	1.81E+09	1.77E+09	2.03E+09	16
2.69E+08	2.08E+08	1.6E+08	1.56E+08	1.88E+08	11
					1
					2
9.89E+08	8.31E+08	1.44E+09	1.69E+09	1.73E+09	13
	2.03E+08	1.71E+08	1.95E+08	2.52E+08	5
1.91E+08	2.41E+08	1.89E+08	1.43E+08	1.57E+08	6
2.8E+08	2.64E+08	1.36E+08	1.41E+08	2.07E+08	10
1.07E+09	1.12E+09	7.57E+08	6.78E+08	7.98E+08	14
					1
1.46E+08	1.42E+08	67245000	1.49E+08	1.47E+08	10
90368000	76412000		1.13E+08		3
1.06E+08	76986000	1.08E+08	85106000	94970000	5
					2
					1
	2.44E+08				6
					1
			1.07E+08	1.07E+08	2
	91416000		67203000	93013000	2
					1
		3.76E+09	2.31E+09	2.46E+09	6
1.64E+08	1.53E+08	93120000	1.37E+08	1.22E+08	5
					2
		2.21E+08	1.94E+08	1.86E+08	4
1.42E+08	1.15E+08	1.53E+08	1.92E+08	2.33E+08	8
91255000	1.01E+08	85653000	73592000	80585000	3
81700000					3
					1

				17955000	3
					3
75997000	61408000	94569000	90708000	86876000	8
					3
					4
1.03E+09	7.5E+08	1.02E+09	7.88E+08	6.96E+08	12
5.63E+08	7.15E+08	5.77E+08	4.12E+08	6.11E+08	8
87359000	1.26E+08	1.07E+08	73588000	1.05E+08	7
67540000	68071000	76842000	52384000	58239000	3
	16253000				5
1.23E+08	1.02E+08	72892000	91963000	6.8E+08	5
4.6E+08	4.12E+08	3.25E+08	5.37E+08	5.47E+08	16
				88256000	1
					2
					3
1.07E+08	90454000	1.17E+08	96297000		2
					2
2.57E+08	1.66E+08	88522000	1.76E+08	1.43E+08	5
					3
1.02E+08	84382000	86225000	1.04E+08	87192000	5
					3
1.04E+09	1.11E+09	2.13E+09	2.38E+09	2.15E+09	8
					2
49227000	59574000		44148000	68945000	12
69509000	68960000	32386000	42153000	48433000	22
1.72E+08	1.92E+08	2.78E+08	3.18E+08	3.29E+08	24
					4
					2
					2
					2
		32847000	29218000	33313000	4
					2
				1.03E+08	1
3.88E+08	5.17E+08	5.02E+08	6.32E+08	5.36E+08	8
1.16E+08	1.86E+08	1.03E+08	1.16E+08	1.78E+08	8
1.97E+08	1.91E+08	6.12E+08	2.95E+08	3.85E+08	6
1.87E+08	1.21E+08	1.36E+08	1.74E+08	1.75E+08	6
	45580000	52793000			4
					4
1.28E+08	1.51E+08	1.03E+08	83379000	1.05E+08	3
1.56E+08		1.19E+08			2
	45987000			45942000	2
54001000	48894000	47346000	61182000		6
2.19E+08	2.62E+08	1.13E+08	1.51E+08	2.97E+08	6
					2
8.69E+08	1.6E+09	1.08E+09	7.54E+08	8.57E+08	11
					1
87345000	1.02E+08		97106000	96157000	3
2.4E+08	2.42E+08	3.44E+08	3.64E+08	3.35E+08	9
39628000	47037000	31589000	32329000	74853000	8
2.44E+09	2.02E+09	2.41E+09	2.76E+09	2.49E+09	12

2.68E+08	1.86E+08	3.42E+08	3.39E+08	3.51E+08	3

N: Razor +	N: Unique	N: Sequen	N: Unique	N: Unique	N: Mol. we	N: Q-value	N: Score	N: Intensit
10	10	37.3	37.3	37.3	32.21		323.31	3.14E+10
10	10	31.4	31.4	31.4	51.24		323.31	5.04E+10
16	16	45.3	45.3	45.3	42.662		323.31	1.64E+10
15	15	27.7	27.7	27.7	84.894		135.37	2.52E+10
4	4	20.2	20.2	20.2	28.203		33.205	2.78E+09
19	19	62.3	62.3	62.3	38.144		323.31	2.26E+11
3	3	14.8	14.8	14.8	32.164	0.001091	2.7705	1.66E+09
14	14	21.4	21.4	21.4	105.66		323.31	3.18E+10
10	10	34	34	34	47.131		123.71	3.21E+10
2	2	6.7	6.7	6.7	27.751		58.019	6.46E+08
5	5	29.2	29.2	29.2	20.588		115.7	1.38E+10
9	9	28.4	28.4	28.4	42.943		323.31	6.86E+10
53	53	62.1	62.1	62.1	99.354		323.31	1.94E+12
5	5	19.5	19.5	19.5	41.126	0.000617	7.4198	2.61E+09
5	5	27	27	27	30.98		194.26	2.57E+10
23	23	29.1	29.1	29.1	93.47		323.31	4.78E+10
10	10	30.1	30.1	30.1	37.54		312.55	2.9E+11
15	15	20.5	20.5	20.5	112.1		100.93	2.79E+10
6	6	20	20	20	37.894		74.16	2.91E+09
25	25	41.8	41.8	41.8	60.512		323.31	3.68E+11
20	20	43	43	43	44.452		323.31	4.16E+11
17	17	45.3	45.3	45.3	45.541		323.31	4.15E+11
8	8	30.4	30.4	30.4	45.504		157.01	1.69E+10
11	11	32.1	32.1	32.1	49.875		323.31	1.09E+10
3	3	19.8	19.8	19.8	33.597		231.24	2.83E+09
3	3	32	32	32	30.238	0.000717	17.04	1.46E+09
2	2	9.4	9.4	9.4	30.919		48.303	1.41E+09
3	3	23.3	23.3	23.3	23.895	0.00061	6.5486	1.24E+09
2	2	16.4	16.4	16.4	20.417	0.000725	18.336	3.38E+08
10	10	39.9	39.9	39.9	41.797		80.107	3.35E+10
1	1	3.7	3.7	3.7	40.021	0.000554	3.0978	1.34E+08
3	3	13.5	13.5	13.5	43.851	0.000689	13.764	3.89E+09
62	62	65.4	65.4	65.4	123.73		323.31	1.06E+12
14	14	56.7	56.7	56.7	34.038		323.31	1.93E+11
3	3	26.2	26.2	26.2	17.433		36.654	3.07E+09
15	15	31.2	31.2	31.2	66.603		323.31	4.38E+10
6	6	23.8	23.8	23.8	51.76		59.009	1.39E+10
7	7	24.2	24.2	24.2	25.894		50.803	8.57E+09
2	2	10.9	10.9	10.9	37.528		37.077	9.8E+08
3	3	15.5	15.5	15.5	21.78	0.000592	5.2992	1.12E+09
3	3	34.6	34.6	34.6	8.9624		323.31	6.1E+10
6	6	14.4	14.4	14.4	71.125	0.000696	14.616	3.42E+09
5	5	52.9	52.9	52.9	15.017		264.85	4.13E+10
11	11	35.7	35.7	35.7	47.974		128.81	2.41E+10
7	7	38.2	38.2	38.2	25.269		195.44	2.05E+10
3	3	42.4	42.4	42.4	11.4		290.32	3.18E+09
13	13	25	25	25	72.137		323.31	4.44E+10
6	6	11.5	11.5	11.5	63.512	0.00064	9.0655	6.67E+09
4	4	24.7	24.7	24.7	39.458		69.976	1.14E+10

5	5	20.5	20.5	20.5	25.358		37.748	9.82E+09
5	5	20.1	20.1	20.1	33.548	0.00067	12.036	3.64E+09
9	9	46.6	46.6	46.6	26.369		99.156	7.39E+10
4	4	35.8	35.8	35.8	17.032		163.96	4E+10
4	4	24.9	24.9	24.9	26.567		200.97	2.73E+09
5	5	20.1	20.1	20.1	28.297		112.27	1.82E+10
1	1	9.4	9.4	9.4	32.389	0.001628	2.6125	2.26E+08
1	1	5.7	5.7	5.7	17.395	0.000729	18.853	1.33E+09
17	17	32.6	32.6	32.6	86.984		323.31	9.8E+10
10	10	36.6	36.6	36.6	37.548		323.31	2.29E+10
8	8	36.6	36.6	36.6	33.587		310.38	1.29E+10
1	1	5.6	5.6	5.6	32.238	0.00061	6.5607	1.13E+09
1	1	8.2	8.2	8.2	15.592	0.000562	3.3386	3.75E+08
11	11	35.5	35.5	35.5	32.501		67.311	3.42E+10
13	13	34	34	34	49.486		323.31	5.48E+10
17	17	21.9	21.9	21.9	95.985		323.31	7.77E+10
5	5	26.4	26.4	26.4	26.528		91.713	1.1E+10
4	4	47.6	47.6	47.6	17.951		134.83	1.68E+10
8	8	44	44	44	23.019		323.31	4.19E+11
15	15	52.1	52.1	52.1	34.321		323.31	7.84E+10
5	5	23.9	23.9	23.9	24.268		29.38	5.48E+09
10	10	35.1	35.1	35.1	44.109		323.31	1.19E+11
8	8	32	32	32	28.065		96.887	6.09E+10
7	7	18.6	18.6	18.6	48.54		323.31	1.59E+10
14	14	21.9	21.9	21.9	92.612		323.31	5.57E+10
6	6	29.6	29.6	29.6	36.317		70.358	1.82E+10
2	2	16.9	16.9	16.9	17.772	0.000704	15.946	2.71E+09
3	3	11.2	11.2	11.2	47.172		64.239	4.76E+09
9	9	30.5	30.5	30.5	46.272		210.18	1.35E+10
7	7	22.9	22.9	22.9	42.256		323.31	3.62E+10
3	3	25	25	25	12.899	0.000616	7.2782	4.24E+08
15	15	45.5	45.5	45.5	37.196		298.74	1.26E+11
9	9	42.4	42.4	42.4	23.844		119.67	1.42E+11
5	5	32.1	32.1	32.1	13.791		170.52	4.5E+10
10	10	50.8	50.8	50.8	14.227		323.31	9.45E+10
1	1	24.3	24.3	24.3	4.3092		323.31	2.54E+10
2	2	33.8	33.8	33.8	9.3006	0.000647	9.6889	5.96E+09
4	4	14.2	14.2	14.2	21.691		28.88	1.1E+10
10	10	60.9	60.9	60.9	16.86		323.31	3.27E+11
3	3	38.2	38.2	38.2	6.0671	0.000599	5.8666	2.3E+09
9	9	54.1	54.1	54.1	20.977		323.31	1.9E+11
7	7	42.1	42.1	42.1	12.139		158.83	7.89E+10
14	14	73	73	73	19.581		323.31	1.2E+11
8	8	51.1	51.1	51.1	15.048		323.31	1.92E+11
1	1	14.6	14.6	14.6	10.208		44.16	4.28E+10
5	5	47.2	47.2	47.2	23.617		323.31	9.64E+10
10	10	23.6	23.6	23.6	73.942		323.31	3.59E+10
9	9	34.5	34.5	34.5	46.962		276.79	1.14E+10
15	14	26.6	26.6	25.4	75.569		212.66	2.22E+10
5	5	57.3	57.3	57.3	21.147		323.31	4.19E+09
16	16	27.6	27.6	27.6	84.061		323.31	2.05E+11
13	13	30.8	30.8	30.8	57.204		155.24	6.83E+10
5	5	19.7	19.7	19.7	30.79	0.000585	4.7298	1.93E+09

1	1	27.3	27.3	27.3	6.3594		184.46	3.49E+09
7	7	55.3	55.3	55.3	20.967		323.31	1.58E+11
7	7	47.9	47.9	47.9	14.872		323.31	1.36E+11
9	9	52.4	52.4	52.4	24.36		323.31	9.35E+10
6	6	33.7	33.7	33.7	17.754		152.87	2.22E+09
3	3	17.2	17.2	17.2	12.627	0.000693	14.307	2.44E+09
1	1	8.6	8.6	8.6	30.414	0.000649	9.8383	1.56E+08
3	3	20.2	20.2	20.2	19.686	0.000669	11.925	6.13E+09
7	7	49.2	49.2	49.2	13.957		323.31	5.58E+10
3	3	13.9	13.9	13.9	38.056	0.000753	21.747	5.97E+09
5	5	24.8	24.8	24.8	20.551		236.99	2.1E+11
2	2	8.5	8.5	8.5	29.367	0.000661	11.094	7.65E+09
1	1	6.6	6.6	6.6	22.397	0.000589	5.1415	2.51E+08
14	14	37.7	37.7	37.7	44.283		323.31	6.75E+10
3	3	14.7	14.7	14.7	23.025	0.000702	15.675	1.49E+09
5	5	24.1	24.1	24.1	26.491		30.117	4.74E+09
12	12	35.3	35.3	35.3	51.847		323.31	3.25E+11
9	9	33.1	33.1	33.1	34.925		225.42	2.34E+10
9	9	34.6	34.6	34.6	36.031		192.07	1.73E+10
3	3	52.6	52.6	52.6	8.2871		323.31	3.55E+11
14	14	42.3	42.3	42.3	55.067		323.31	1.36E+11
10	10	15.4	15.4	15.4	99.759		323.31	2.29E+10
5	5	39.2	39.2	39.2	22.621		323.31	8.02E+09
4	4	21.5	21.5	21.5	30.471	0.000684	13.403	3.85E+09
11	11	25.2	25.2	25.2	76.43		180.89	1.65E+10
3	3	23.2	23.2	23.2	22.374		46.991	6.28E+09
8	8	23.5	23.5	23.5	55.512		54.568	2.37E+10
1	1	5.1	5.1	5.1	33.443		26.309	3.31E+09
5	5	40.8	40.8	40.8	15.734	0.000668	11.735	2.79E+09
14	14	42.5	42.5	42.5	49.889		323.31	1.67E+10
5	5	35.9	35.9	35.9	22.747		55.823	2.62E+10
3	3	7	7	7	80.005		92.433	1.3E+09
2	2	6.3	6.3	6.3	39.262	0.000625	8.0237	2.57E+09
11	11	31	31	31	45.232		323.31	1.53E+10
1	1	7.2	7.2	7.2	33.592	0.00075	21.489	5.01E+08
4	4	59.2	59.2	59.2	12.774		69.315	1.04E+10
15	15	21.7	21.7	21.7	102.12		323.31	7.31E+10
5	5	13.9	13.9	13.9	50.255	0.00069	13.791	3.44E+09
13	13	29.6	29.6	29.6	64.023		323.31	5.06E+10
2	2	12	12	12	17.738	0.000627	8.1101	8.96E+08
10	10	38.1	38.1	38.1	37.598		323.31	2.38E+10
5	5	33.7	33.7	33.7	22.652		23.488	1.59E+10
7	7	23.9	23.9	23.9	46.176		171.76	1.58E+10
15	15	46.2	46.2	46.2	58.433		323.31	1.02E+11
4	4	20.4	20.4	20.4	35.288	0.000661	10.814	1.57E+09
3	3	11.8	11.8	11.8	42.183	0.00071	16.334	5.35E+09
3	3	9.5	9.5	9.5	40.974	0.0006	5.9154	2.56E+09
1	1	16	16	16	17.648		296.28	5.68E+08
10	10	26.1	26.1	26.1	60.164		68.385	1.19E+10
2	2	13	13	13	30.17	0.000615	7.1022	3.86E+08
23	23	32.6	32.6	32.6	98.001		323.31	2.87E+11
6	6	23.7	23.7	23.7	39.724		295.04	6.38E+10
4	4	9.5	9.5	9.5	57.945	0.000607	6.3102	1.32E+09

3	3	9.9	9.9	9.9	35.469	0.000553	3.0656	6.84E+09
5	5	13.3	13.3	13.3	56.054		304.1	1.51E+10
2	2	10.6	10.6	10.6	37.752	0.001616	2.4047	1.3E+09
4	4	24.4	24.4	24.4	20.719	0.000656	10.603	4.34E+09
1	1	7.9	7.9	7.9	19.44		25.688	1.92E+09
3	3	14.7	14.7	14.7	45.539		55.881	2.8E+09
5	5	33.1	33.1	33.1	30.143		63.141	1.27E+09
4	4	28.5	28.5	28.5	26.92		54.927	7.79E+09
7	7	26.5	26.5	26.5	39.561		170.38	8.37E+09
12	12	30.9	30.9	30.9	59.112		323.31	3.31E+10
32	32	58.6	58.6	58.6	65.62		323.31	4.05E+11
5	5	27.6	27.6	27.6	22.641		58.992	1.37E+09
4	4	23.6	23.6	23.6	18.787		27.089	7.11E+09
5	5	29.5	29.5	29.5	31.017		42.968	1.14E+10
6	6	34.4	34.4	34.4	29.548		323.31	1.19E+10
10	10	36	36	36	36.835		77.276	2.62E+10
12	12	61	61	61	29.755		323.31	2.26E+11
23	23	76.5	76.5	76.5	28.687		323.31	3.8E+11
5	5	33.9	33.9	33.9	26.738		191.18	5.29E+09
6	6	33.3	33.3	33.3	20.62		323.31	7.1E+10
7	7	29	29	29	41.202		223.23	1.19E+10
2	2	10.1	10.1	10.1	40.485	0.00526	1.8458	4.33E+09
4	4	17	17	17	25.032	0.000708	16.251	1.11E+10
5	5	19.7	19.7	19.7	44.737		35.968	2.47E+09
3	3	19.1	19.1	19.1	19.548		37.859	9.85E+08
10	10	23.4	23.4	23.4	56.623		269.6	2.89E+10
5	5	9.4	9.4	9.4	67.644	0.000672	12.168	3.89E+09
15	15	51.6	51.6	51.6	24.606		323.31	6.79E+10
4	4	14.1	14.1	14.1	34.239		99.722	1.47E+10
5	5	17.7	17.7	17.7	42.85		85.423	3.77E+09
17	17	54.3	54.3	54.3	44.741		323.31	5.69E+10
4	4	17.3	17.3	17.3	31.81		323.31	9.74E+09
19	19	35.8	35.8	35.8	75.269		323.31	5.28E+10
8	8	30.4	30.4	30.4	43.351		181.5	1.1E+10
5	5	24	24	24	27.96		79.295	1.46E+10
3	3	10.4	10.4	10.4	40.127		42.26	1.58E+10
3	3	17.1	17.1	17.1	31.125	0.000645	9.4496	1.22E+09
5	5	24.6	24.6	24.6	38.04		141.51	2.77E+09
4	4	27.5	27.5	27.5	24.598		78.54	2.32E+10
3	3	26.1	26.1	26.1	17.661		136.5	2.25E+10
3	3	22	22	22	22.235	0.000592	5.3082	1.86E+09
3	3	6.2	6.2	6.2	68.933	0.000612	6.8454	6.38E+08
4	4	11.9	11.9	11.9	46.517		35.193	8.99E+09
15	15	27.6	27.6	27.6	64.231		154.22	8.19E+10
13	13	44.8	44.8	44.8	42.898		177.8	4.38E+10
6	6	34.4	34.4	34.4	25.21		323.31	4.3E+10
9	9	39.3	39.3	39.3	24.759		119.86	2.85E+10
14	14	17.2	17.2	17.2	120.27		304.98	1.87E+10
4	4	21.1	21.1	21.1	32.74		119.6	1.05E+10
1	1	7.1	7.1	7.1	33.171	0.006753	1.6237	5.04E+08
41	40	90.5	90.5	90.5	30.606		323.31	8.12E+11
4	4	31.5	31.5	31.5	10.003		323.31	9.41E+10

2	2	25	25	25	9.6669	0.001092	2.7736	7.33E+08
3	3	12.9	12.9	12.9	27.935	0.000698	15.121	5.03E+09
6	6	28.4	28.4	28.4	23.478		323.31	1.82E+11
9	9	25.9	25.9	25.9	47.735		323.31	2.1E+10
2	2	26	26	26	10.651		24.939	8.38E+08
2	2	16.9	16.9	16.9	20.481		22.892	1.09E+09
9	9	21.8	21.8	21.8	56.39		293.95	1.86E+10
11	11	26.2	26.2	26.2	57.422		104.49	1.08E+10
20	20	54.7	54.7	54.7	41.684		323.31	3.93E+10
7	7	38.4	38.4	38.4	29.98		216.32	3.97E+10
5	5	17.1	17.1	17.1	34.263		73.395	3.18E+10
5	5	9.8	9.8	9.8	62.706	0.000675	12.476	2.87E+09
5	5	35.9	35.9	35.9	20.263		140.19	1.12E+11
13	13	70	70	70	22.792		323.31	8.2E+10
25	25	70.8	70.8	70.8	30.929		323.31	3.48E+10
6	6	21.7	21.7	21.7	30.118		74.432	1.93E+10
4	4	44.5	44.5	44.5	12.868		151.75	1.81E+10
3	3	7.4	7.4	7.4	48.558	0.000679	12.871	3.38E+09
5	5	14.3	14.3	14.3	55.752		28.407	7.62E+09
9	9	22.3	22.3	22.3	67.036		53.248	6.94E+09
6	6	30.5	30.5	30.5	38.032		134.82	4.02E+10
9	9	18	18	18	64.678		323.31	1.55E+10
9	9	34.2	34.2	34.2	35.018		323.31	2.11E+10
8	8	36.3	36.3	36.3	40.533		323.31	4.64E+10
5	5	35	35	35	20.397		242.91	8.11E+09
2	2	12.1	12.1	12.1	34.081	0.000652	10.208	8.79E+08
8	8	37	37	37	44.917		80.511	8.91E+09
3	3	8.9	8.9	8.9	52.757	0.00059	5.1864	2.03E+09
4	4	14.7	14.7	14.7	33.238	0.000598	5.7049	2.22E+09
1	1	5.7	5.7	5.7	46.673		24.592	1.4E+09
3	3	13.1	13.1	13.1	38.179	0.000707	16.144	3.59E+09
2	2	7.5	7.5	7.5	39.141	0.009734	1.4619	2.64E+08
2	2	10.5	10.5	10.5	37.192	0.000732	19.259	1.38E+08
7	7	50.3	50.3	50.3	22.02		250.34	2.32E+10
1	1	9	9	9	16.041	0.006273	1.7414	4648700
2	2	11.6	11.6	11.6	21.539	0.000569	3.8754	1.35E+09
4	4	13.7	13.7	13.7	30.129		276	2.2E+10
11	11	30.8	30.8	30.8	48.23		150.87	2.23E+10
22	22	31.6	31.6	31.6	94.877		323.31	4.83E+10
2	2	4.7	4.7	4.7	46.363	0.000553	3.0732	6.89E+08
2	2	5.1	5.1	5.1	41.372	0.00528	1.9325	5.67E+08
8	8	29.4	29.4	29.4	36.912		134.42	2.54E+10
4	4	16.6	16.6	16.6	39.755		49.566	2.45E+09
11	11	50	50	50	50.951		323.31	1.75E+11
6	6	17.8	17.8	17.8	37.775		28.009	4.07E+09
7	7	43.3	43.3	43.3	28.216		98.666	1.93E+10
5	5	20.6	20.6	20.6	46.786	0.000688	13.68	5.97E+08
2	2	11.5	11.5	11.5	25.094	0.000604	6.1518	1.34E+09
4	4	15.6	15.6	15.6	37.979	0.000609	6.4263	2.99E+09
44	44	42.6	42.6	42.6	128.73		323.31	2.45E+11
34	34	27.4	27.4	27.4	171.36		323.31	2.58E+11

1	1	6.1	6.1	6.1	24.447	0.001089	2.6816	1.5E+08
13	13	60.9	60.9	60.9	26.57		323.31	2.32E+11
8	8	29.2	29.2	29.2	54.046		323.31	1.38E+10
2	2	17.1	17.1	17.1	31.408		107.03	1.83E+09
8	8	38.1	38.1	38.1	16.86		323.31	4.79E+10
6	6	45.1	45.1	45.1	24.236		27.912	4.28E+10
2	2	17.8	17.8	17.8	8.5817	0.000553	3.0707	1.12E+08
2	2	5.5	5.5	5.5	45.523		47.137	2.6E+09
10	10	46.5	46.5	46.5	34.926		209.93	6.7E+09
9	9	25.2	25.2	25.2	50.916		213.74	1.75E+10
2	2	7.7	7.7	7.7	38.364	0.004253	2.0886	6.55E+08
2	2	10.2	10.2	10.2	43.161	0.001626	2.5497	1.23E+09
16	16	43.1	43.1	43.1	46.613		323.31	7.49E+10
1	1	7	7	7	15.9	0.009709	1.4484	1.28E+08
1	1	9.2	9.2	9.2	22.227		43.659	7.61E+08
6	6	20.9	20.9	20.9	41.162		119.89	9.98E+09
8	8	20.7	20.7	20.7	78.593		323.31	1.31E+10
2	2	9.7	9.7	9.7	37.037	0.000617	7.3404	8.59E+09
14	14	42.7	42.7	42.7	41.628		323.31	3.35E+11
6	6	45.8	45.8	45.8	18.316		323.31	1.82E+11
4	4	30.6	30.6	30.6	17.484		111.64	2.04E+09
4	4	21.6	21.6	21.6	23.484		288.26	1.02E+10
1	1	5.4	5.4	5.4	27.779	0.005244	1.785	3.3E+08
8	8	43.8	43.8	43.8	23.952		196.69	7.22E+10
20	20	52.4	52.4	52.4	51.306		323.31	5.61E+11
28	28	59.5	59.5	59.5	63.583		323.31	6.71E+11
18	18	63.1	63.1	63.1	38.035		323.31	1.46E+11
7	7	43.2	43.2	43.2	20.14		323.31	2.35E+11
12	12	27.7	27.7	27.7	75.223		173.12	4.56E+09
4	4	18.6	18.6	18.6	27.745		33.807	5.12E+09
3	3	9.1	9.1	9.1	43.518	0.000746	21.064	3.38E+09
4	4	11.5	11.5	11.5	51.778	0.000576	4.1534	6.19E+08
3	3	9.2	9.2	9.2	44.876	0.000584	4.6698	3.85E+09
18	18	25.2	25.2	25.2	111.65		323.31	2.04E+10
7	7	38.9	38.9	38.9	34.519		323.31	9.59E+09
1	1	10.2	10.2	10.2	18.725	0.00826	1.5284	41336000
5	5	18.6	18.6	18.6	44.57	0.000594	5.4529	4.52E+09
2	2	9.4	9.4	9.4	34.62		64.563	4.4E+09
2	2	12.4	12.4	12.4	30.823		191.12	8.58E+09
4	4	10.2	10.2	10.2	50.47		296.93	1.17E+10
46	46	73	73	73	57.777		323.31	3.29E+11
9	9	72.6	72.6	72.6	10.234		105.37	5.28E+10
2	2	5.8	5.8	5.8	62.508	0.000637	8.798	3.33E+08
10	10	33.8	33.8	33.8	48.457		323.31	2.75E+10
1	1	13.3	13.3	13.3	12.73	0.000623	7.7907	2.34E+09
18	18	25.1	25.1	25.1	97.269		323.31	4.9E+10
5	5	20.9	20.9	20.9	36.866		131.42	1.7E+10
6	6	22.7	22.7	22.7	46.305		47.016	1.27E+10
6	6	6.4	6.4	6.4	149.28	0.000697	14.896	2.39E+09
10	10	32	32	32	39.092		323.31	2.26E+10
2	2	12.9	12.9	12.9	30.591		142.65	2.76E+10

2	2	12.1	12.1	12.1	24.808		79.114	3.87E+09
1	1	3.6	3.6	3.6	30.819	0.000605	6.2025	3.51E+09
4	4	40.4	40.4	40.4	23.096		168.64	8.58E+09
13	13	29.6	29.6	29.6	54.689		323.31	1.31E+10
4	4	14.4	14.4	14.4	33.268		191.29	7.42E+09
1	1	7.3	7.3	7.3	28.873	0.000682	13.097	7.21E+08
5	5	12.7	12.7	12.7	66.909		43.131	4.4E+09
3	3	14.5	14.5	14.5	27.839	0.001098	2.8875	1.35E+09
8	8	50	50	50	21.109		121.49	2.59E+10
3	3	24.9	24.9	24.9	25.39		130.55	3.33E+09
7	7	36.1	36.1	36.1	23.002		63.827	1.41E+10
4	4	21.3	21.3	21.3	28.135		27.69	1.28E+09
7	7	8.5	8.5	8.5	107.08	0.000556	3.1823	3.07E+09
2	2	14.9	14.9	14.9	37.411	0.000735	19.84	8.16E+09
11	11	37.1	37.1	37.1	46.129		323.31	6.64E+10
4	4	8	8	8	59.301		26.027	1.57E+10
9	9	34.5	34.5	34.5	36.81		193.06	1.51E+10
24	23	33.5	33.5	32.4	90.362		323.31	5.63E+10
1	1	9.5	9.5	9.5	18.437		59.043	7.61E+09
3	3	47.3	47.3	47.3	10.151		323.31	1.01E+10
13	13	54.5	54.5	54.5	21.156		323.31	1.23E+11
16	16	66.4	66.4	66.4	35.148		323.31	3.52E+11
8	8	38.3	38.3	38.3	20.35		211.06	1.39E+11
7	7	38.3	38.3	38.3	12.358		36.69	1.22E+11
6	6	42.5	42.5	42.5	14.232		300.1	2.32E+10
2	2	22.9	22.9	22.9	10.782		323.31	1.15E+11
3	3	40.3	40.3	40.3	7.7599	0.000672	12.145	1.23E+09
4	4	23.4	23.4	23.4	16.095	0.000587	4.8744	2.62E+08
8	8	45	45	45	27.307		323.31	7.94E+10
8	8	38.1	38.1	38.1	15.159		124.01	4.93E+11
3	3	37.9	37.9	37.9	10.822	0.000671	12.107	6.46E+09
16	16	58.2	58.2	58.2	30.044		323.31	3.35E+11
7	7	68.4	68.4	68.4	10.522		323.31	1.25E+11
14	14	66.3	66.3	66.3	22.278		323.31	1.32E+11
10	10	39.8	39.8	39.8	22.437		323.31	3.68E+11
3	3	22.4	22.4	22.4	12.109		246.16	2.99E+10
33	33	51.9	51.9	51.9	76.797		323.31	4.3E+11
10	10	60.9	60.9	60.9	17.941		279.58	2.5E+11
1	1	6.1	6.1	6.1	14.354		196.88	1.47E+09
5	5	15.7	15.7	15.7	52.316		64.52	1.24E+10
2	2	13.7	13.7	13.7	35.742		323.31	4.8E+09
1	1	9	9	9	15.114	0.006746	1.6173	2.05E+08
4	4	21.2	21.2	21.2	29.676	0.000587	4.9038	1.99E+09
8	8	16.6	16.6	16.6	73.527		56.682	2.53E+10
4	4	19.3	19.3	19.3	16.816	0.001619	2.4169	8.97E+09
4	4	15.2	15.2	15.2	26.815	0.000667	11.487	1.95E+09
2	2	17.6	17.6	17.6	23.584		306.6	5.43E+09
11	11	25.2	25.2	25.2	78.366		128.36	3.1E+10
15	15	65.7	65.7	65.7	32.27		323.31	1.24E+11
6	6	28.6	28.6	28.6	50.17		232.98	8.82E+09
1	1	11.5	11.5	11.5	10.006	0.000615	7.2018	7.66E+10

5	5	19.7	19.7	19.7	44.214		53.754	5.61E+09
6	6	50	50	50	21.292		307.4	5.07E+09
4	4	23.3	23.3	23.3	14.733		266.34	2.1E+11
4	4	25.9	25.9	25.9	19.19		93.803	1.49E+10
1	1	4	4	4	34.757	0.004764	1.9899	6.57E+09
2	2	13.8	13.8	13.8	23.66	0.000695	14.497	1.43E+09
16	16	26.7	26.7	26.7	102.75		224.31	6.52E+10
40	40	64	64	64	67.723		323.31	4.71E+11
5	5	20.4	20.4	20.4	24.724		55.348	5.61E+10
6	6	28.6	28.6	28.6	20.475		44.5	1.35E+11
2	2	10.2	10.2	10.2	22.43		61.38	1.01E+09
8	8	26.6	26.6	26.6	28.356		303.27	4.6E+10
11	11	65.8	65.8	65.8	16.065		323.31	6.88E+10
8	8	59.8	59.8	59.8	10.623		38.908	1.13E+11
10	10	43.9	43.9	43.9	32.722		323.31	2.88E+11
6	6	53.9	53.9	53.9	11.672		323.31	1.03E+11
4	4	50	50	50	11.147		29.844	1.06E+10
11	11	27.6	27.6	27.6	47.836		323.31	1.69E+10
5	5	25.5	25.5	25.5	32.656		136.52	1.99E+10
13	13	28.1	28.1	28.1	74.664		323.31	3.19E+10
6	6	52.7	52.7	52.7	20.83	0.00072	17.575	4.69E+09
4	4	17.4	15.4	15.4	45.006	0.000558	3.238	2.89E+08
17	17	50.2	50.2	50.2	44.32		323.31	1.23E+11
11	11	33.3	33.3	33.3	43.973		100.67	1.8E+10
4	4	9	9	9	65.259		31.18	6.55E+09
2	2	13	13	13	26.457	0.000558	3.2256	4.57E+08
7	7	43.5	43.5	43.5	27.522		135.98	1.67E+10
7	7	30.6	30.6	30.6	31.837		145.82	1.92E+10
5	5	37	37	37	24.488		32.011	3.98E+10
5	5	13.5	13.5	13.5	52.041	0.000699	15.337	7.67E+08
6	6	14.2	14.2	14.2	56.409		323.31	2.29E+10
7	7	28.3	28.3	28.3	43.889		60.171	7.68E+09
10	10	15.6	15.6	15.6	106.83		37.363	6.03E+09
2	2	14.7	14.7	14.7	27.174		24.468	3.18E+09
33	33	55.8	55.8	55.8	76.754		323.31	3.51E+11
22	22	65.1	65.1	65.1	54.834		323.31	2.2E+11
7	7	14.4	14.4	14.4	55.904		27.662	1.07E+10
5	5	16.6	16.6	16.6	43.296	0.000719	17.456	3.96E+09
7	7	28.2	28.2	28.2	37.265		48.403	8.79E+09
4	4	27.5	27.5	27.5	21.298		101.91	1.49E+10
4	4	29.5	29.5	29.5	30.897	0.000748	21.274	1.12E+09
11	11	43.9	43.9	43.9	31.077		323.31	3.37E+10
7	7	23	23	23	55.652		224.05	1.07E+10
4	4	23.5	23.5	23.5	31.54		43.129	8.38E+09
3	3	16.1	16.1	16.1	27.59	0.000552	2.9825	4.62E+08
4	4	24.4	24.4	24.4	27.631		42.001	5.76E+08
13	13	31.2	31.2	31.2	60.954		323.31	4.62E+10
5	5	21.3	21.3	21.3	41.572		78.405	2.72E+09
5	5	11.5	11.5	11.5	55.535	0.000683	13.255	2.95E+09
9	9	66.4	66.4	66.4	12.289		223.7	1.84E+11
12	12	24.2	24.2	24.2	81.584		204.78	8.53E+09

8	8	36.2	36.2	36.2	32.891		248.32	8.47E+09
1	1	7.8	7.8	7.8	15.309	0.00627	1.7398	1.7E+08
2	2	14.1	14.1	14.1	26.089		193.91	2.3E+09
5	5	25.7	25.7	25.7	28.142		323.31	3.85E+09
6	6	16.9	16.9	16.9	50.362		165.93	1.83E+10
5	5	26.1	26.1	26.1	32.077		34.976	8.06E+09
4	4	12.4	12.4	12.4	59.798		323.31	9.64E+09
5	5	16.5	16.5	16.5	36.324		45.973	2.56E+10
5	5	38.2	38.2	38.2	23.723		121.73	2.38E+10
9	9	51.3	51.3	51.3	25.779		135.5	2.17E+09
3	3	9.9	9.9	9.9	42.113	0.000563	3.4866	4.17E+08
7	7	36.8	36.8	36.8	27.328		323.31	2.45E+10
16	16	50.6	50.6	50.6	52.34		323.31	8.31E+10
3	3	24.1	24.1	24.1	15.547	0.000716	17	1.03E+10
2	2	7	7	7	44.128	0.000581	4.4248	4.75E+08
12	12	44.1	44.1	44.1	55.899		323.31	5.58E+10
2	2	22.2	22.2	22.2	20.113	0.000648	9.7396	1.46E+09
5	5	26.9	26.9	26.9	27.97	0.000734	19.607	6.9E+09
1	1	1.8	1.8	1.8	91.644	0.000622	7.7375	1.48E+09
4	4	20.4	20.4	20.4	28.646		218.27	9.05E+09
6	6	29.4	29.4	29.4	26.361		49.107	1.55E+10
5	5	31.2	31.2	31.2	27.163		148.88	1.91E+09
7	7	22.4	22.4	22.4	43.503		102.82	5.57E+09
1	1	9.6	9.6	9.6	25.311	0.006257	1.7156	36505000
11	11	29.3	29.3	29.3	50.38		49.277	6.68E+09
1	1	10.6	10.6	10.6	16.984	0.000675	12.473	4.31E+09
8	8	39.6	39.6	39.6	33.721		69.269	2.61E+10
2	2	7.4	7.4	7.4	36.158		165.64	3.62E+09
18	18	45	45	45	53.228		323.31	7.02E+10
4	4	35.1	35.1	35.1	19.115		323.31	6.15E+09
4	4	30.6	30.6	30.6	34.323		323.31	2.11E+10
3	3	30.6	30.6	30.6	13.119		49.863	1.6E+09
2	2	13.9	13.9	13.9	29.558	0.000612	6.8153	5.24E+09
1	1	3.8	3.8	3.8	34.67	0.000596	5.5706	1.34E+09
19	18	51.6	51.6	51.6	36.328		323.31	2.11E+11
4	4	22.5	22.5	22.5	22.83	0.000604	6.1596	2.23E+09
2	2	57.6	57.6	57.6	9.2399	0.000759	22.518	1.38E+09
8	8	17.5	17.5	17.5	64.508		155.66	4.37E+09
2	2	11.4	11.4	11.4	20.568	0.001615	2.3772	5.4E+08
3	3	13.2	13.2	13.2	46.811	0.000673	12.359	6.9E+08
4	4	42.2	42.2	42.2	12.915		28.121	1.04E+10
2	2	29.8	29.8	29.8	14.993		323.31	2.06E+10
7	7	19.6	19.6	19.6	50.789		63.886	1.71E+09
2	2	10.5	10.5	10.5	24.826		30.166	5.77E+09
1	1	8.5	8.5	8.5	29.103	0.000757	22.25	8.76E+08
9	9	33.1	33.1	33.1	37.176		128.46	2.11E+10
21	21	26.5	26.5	26.5	121.55		323.31	2.84E+10
1	1	14.3	14.3	14.3	7.8909		323.31	3.51E+09
2	2	22.9	22.9	22.9	10.884		48.625	4.11E+09
3	3	29.5	29.5	29.5	16.404	0.000657	10.652	4.3E+09
3	3	40.9	40.9	40.9	22.741		41.604	1.08E+09

5	5	21.2	21.2	21.2	38.371		25.78	3.37E+09
4	4	17.5	17.5	17.5	36.139		248.33	4.52E+09
6	6	21.9	21.9	21.9	37.421		323.31	1.26E+10
8	8	34.6	34.6	34.6	40.252		323.31	1.14E+10
1	1	10.4	10.4	10.4	25.258	0.001617	2.4056	24407000
2	2	6.1	6.1	6.1	31.363	0.005756	1.7619	2.78E+09
7	7	19.6	19.6	19.6	74.052		56.482	1.64E+09
14	14	40.3	40.3	40.3	51.204		323.31	3.23E+10
7	7	42	42	42	19.073		292.34	2.27E+10
8	8	25.6	25.6	25.6	52.727		259.67	1.5E+10
4	4	19.1	19.1	19.1	43.477		26.285	3.75E+09
4	4	15.8	15.8	15.8	33.582		172.85	2.58E+10
5	5	26.9	26.9	26.9	29.844		323.31	3.19E+10
2	2	18.6	18.6	18.6	13.709		59.473	2.14E+09
17	17	64	64	64	50.47		323.31	2.36E+11
11	11	62.8	62.8	62.8	20.324		323.31	5.08E+10
9	9	27.3	27.3	27.3	50.63		323.31	3.69E+10
1	1	3.9	3.9	3.9	33.897	0.004772	2.0251	85953000
4	4	11.3	11.3	11.3	58.301		323.31	2.22E+09
6	6	34.9	34.9	34.9	35.523		323.31	1.79E+10
6	6	20.1	20.1	20.1	41.563	0.00074	20.429	4.87E+09
4	4	22.4	22.4	22.4	22.292	0.000758	22.438	9.99E+09
2	2	8.5	8.5	8.5	37.09	0.000696	14.742	9.32E+08
2	2	12	12	12	13.466		57.425	1.43E+10
5	5	22.7	22.7	22.7	28.316		158.08	8.1E+09
9	9	20.7	20.7	20.7	63.806		122.49	1.07E+11
8	8	27.4	27.4	27.4	38.698		49.746	1.7E+10
2	2	9.5	9.5	9.5	30.573	0.000655	10.571	1.94E+09
10	10	15	15	15	75.236		323.31	2.11E+10
4	4	23.7	23.7	23.7	37.253		323.31	6.23E+08
2	2	20.4	20.4	20.4	17.027	0.000604	6.1321	5.23E+08
15	15	45.1	45.1	45.1	38.831		323.31	6.43E+10
5	5	17.8	17.8	17.8	51.18		97.074	4.15E+09
3	3	17.5	17.5	17.5	23.957	0.000603	6.1249	1.71E+09
4	4	16	16	16	30.821		89.001	1.54E+09
1	1	19.3	19.3	19.3	13.234		96.969	4.04E+09
2	2	15.7	15.7	15.7	17.37	0.006253	1.6741	1.4E+08
16	16	59.9	59.9	59.9	40.858		323.31	1.93E+11
1	1	8	8	8	19.625	0.000552	3.0366	5.48E+08
3	3	8.1	8.1	8.1	45.192	0.000575	4.1152	7.37E+08
1	1	4.5	4.5	4.5	25.081	0.000555	3.0982	2.09E+08
1	1	10.3	10.3	10.3	18.213	0.000634	8.5419	1.51E+08
10	10	38.5	38.5	38.5	41.017		278.08	1.38E+10
3	3	25.3	25.3	25.3	17.393		323.31	9.39E+10
2	2	14.5	14.5	14.5	20.353	0.000635	8.6584	2.36E+09
9	9	17.8	16.9	16.9	88.171		125.16	6.77E+09
2	2	64.7	64.7	64.7	6.7295		78.394	3.19E+09
1	1	6.5	6.5	6.5	20.532	0.009769	1.4853	1.62E+09
1	1	14.7	14.7	14.7	11.349		49.828	1.46E+08
2	2	11.4	11.4	11.4	30.617	0.000755	22.047	2.93E+08
4	4	41	41	41	21.064		39.84	4.49E+09

2	2	32.5	32.5	32.5	12.91		65.563	5.42E+09
14	14	30	30	30	64.245		323.31	1.04E+11
5	5	24.2	24.2	24.2	21.052	0.00068	12.907	1.95E+10
7	7	24.4	24.4	24.4	43.122		81.285	2.82E+09
3	3	24.9	24.9	24.9	22.938		96.637	4.14E+09
4	4	15.8	15.8	15.8	28.946		30.378	2.61E+09
3	3	19.8	19.8	19.8	32.011	0.000727	18.627	1.66E+08
3	3	25.4	25.4	25.4	18.755	0.000721	17.684	5.34E+09
3	3	11.2	11.2	11.2	44.531	0.000629	8.2682	1.05E+09
4	4	18.9	18.9	18.9	37.593		23.099	1.94E+09
3	3	15.9	15.9	15.9	37.542		168.64	3.16E+09
7	7	14.3	14.3	14.3	56.209		70.166	5.5E+10
3	3	28.1	28.1	28.1	13.846	0.000559	3.2509	9.16E+08
6	6	25.7	25.7	25.7	40.691		89.746	5.29E+09
5	5	14.6	14.6	14.6	50.901		157.52	2.02E+09
4	4	22.5	22.5	22.5	26.099		143.13	6.41E+09
1	1	12.1	12.1	12.1	17.059	0.000639	9.0298	3.07E+08
5	5	19	19	19	61.246	0.000718	17.313	3.3E+09
1	1	13.2	13.2	13.2	16.741	0.004249	2.0466	6.93E+08
8	8	20.8	20.8	20.8	64.155		58.066	6.63E+09
3	3	21	21	21	21.011	0.000738	20.238	1.23E+09
2	2	10.9	10.9	10.9	35.077		31.172	9.16E+08
1	1	9.8	9.8	9.8	14.924	0.000736	19.925	3.14E+08
2	2	6.8	6.8	6.8	36.016		28.602	2.41E+09
4	4	36.5	36.5	36.5	21.7	0.000632	8.4069	4.31E+08
5	5	38.9	38.9	38.9	23.128		90.14	1.92E+10
1	1	6.1	6.1	6.1	48.18	0.000682	13.043	3.57E+08
12	12	34.6	34.6	34.6	62.023		203.4	2.52E+10
4	4	13.4	13.4	13.4	44.763	0.000758	22.272	6.8E+08
5	5	19.4	19.4	19.4	36.644		23.607	8.17E+09
4	4	31.7	31.7	31.7	25.164		191.78	9.01E+09
10	10	41.4	41.4	41.4	29.318		323.31	6.23E+10
7	7	48.8	48.8	48.8	18.166		109.5	2.12E+10
5	5	17	17	17	36.547		81.981	1.18E+10
13	13	24.3	24.3	24.3	75.492		62.869	5.03E+10
4	4	63.4	63.4	63.4	9.7595	0.000649	9.7976	1.09E+09
10	10	56.9	56.9	56.9	23.293		323.31	4.71E+11
2	2	12.6	12.6	12.6	19.378	0.000587	4.911	3.05E+09
23	23	23.6	23.6	23.6	152.91		323.31	1.18E+11
3	3	17.6	17.6	17.6	15.374	0.000755	21.948	1.58E+09
8	8	22.9	22.9	22.9	49.182		323.31	1E+11
3	3	7.5	7.5	7.5	58.403		29.617	2.81E+09
4	4	53.1	53.1	53.1	16.533		89.613	5.5E+10
3	3	13	13	13	26.085	0.000605	6.1663	2.33E+09
21	21	42.3	42.3	42.3	66.193		323.31	3.44E+11
4	4	15.4	15.4	15.4	40.101	0.0006	5.8672	1.29E+09
1	1	5.7	5.7	5.7	23.29	0.000609	6.3884	
5	5	42.4	42.4	42.4	16.295		25.238	1.28E+09
7	7	28.7	28.7	28.7	43.801		323.31	1.83E+10
5	5	30	30	30	29.499	0.000659	10.753	1.47E+09
2	2	7.7	7.7	7.7	57.503		114.38	3.57E+09

5	5	28.6	28.6	28.6	34.964		114.64	9.48E+09
5	5	18.2	18.2	18.2	45.86	0.000631	8.3372	1.58E+10
3	3	19.8	19.8	19.8	18.324	0.000685	13.489	3.63E+08
6	6	45	45	45	22.863		105.64	1.26E+10
4	4	11	11	11	49.966		36.748	1.52E+09
4	4	27	27	27	25.392		221.56	7.88E+09
4	4	24.6	24.6	24.6	42.811		59.693	7.09E+09
6	6	20.6	20.6	20.6	42.912		41.86	3.16E+09
3	3	22.1	22.1	22.1	34.334		118.94	4.63E+09
2	2	9.1	9.1	9.1	28.248	0.000586	4.8567	2.32E+09
7	7	29.1	29.1	29.1	35.663		191.95	2.88E+09
4	4	11.8	11.8	11.8	52.988	0.000584	4.6546	2.39E+08
3	3	13.9	13.9	13.9	32.696	0.000702	15.774	1.39E+09
4	4	20.7	20.7	20.7	32.041		69.755	4.92E+09
3	3	22.2	22.2	22.2	28.64		60.708	1.12E+09
7	7	24	24	24	38.323		132.4	1.08E+10
9	9	43.6	43.6	43.6	31.535		323.31	2.91E+10
1	1	8.1	8.1	8.1	22.428	0.005236	1.7701	41516000
13	13	37.5	37.5	37.5	35.155		222.27	2.66E+10
5	5	20.2	20.2	20.2	34.938		116.1	1.09E+10
2	2	31.6	31.6	31.6	12.508	0.000567	3.7507	2.38E+09
5	5	15.9	15.9	15.9	38.942		38.071	3.96E+09
22	22	41	41	41	84.409		323.31	3.78E+11
2	2	17	17	17	23.043		208.79	2.98E+09
2	2	14.8	14.8	14.8	17.655	0.003723	2.1187	1.04E+09
4	4	44.4	44.4	44.4	17.544		323.31	2.37E+10
1	1	4.2	4.2	4.2	23.46	0.003727	2.1257	3.92E+08
5	5	6	6	6	93.627	0.000614	6.9766	5.29E+09
7	7	54.5	54.5	54.5	18.991		216.39	2.91E+10
4	4	27.9	27.9	27.9	31.311		323.31	2.79E+09
1	1	16.1	16.1	16.1	15.234	0.000713	16.713	1.62E+09
5	5	56.9	56.9	56.9	11.399		74.844	5.65E+10
3	3	41.7	41.7	41.7	10.669		323.31	1.41E+10
10	10	35.9	35.9	35.9	53.629		323.31	2.23E+10
1	1	7.3	7.3	7.3	17.528	0.00057	3.9067	3.87E+09
2	2	9.7	9.7	9.7	51.415	0.009704	1.4357	7.06E+09
4	4	21	21	21	29.104		67.137	8.04E+09
2	2	10	10	10	25.348	0.008252	1.5207	2.69E+08
11	11	18.4	18.4	18.4	81.944		91.996	1.16E+10
2	2	9.1	9.1	9.1	27.66	0.000562	3.3754	1.33E+09
10	10	24.3	24.3	24.3	74.167		82.822	2.36E+10
1	1	6.4	6.4	6.4	24.67	0.000559	3.2395	3.26E+08
4	4	14.7	14.7	14.7	40.805	0.000679	12.849	6.45E+09
2	2	2.5	2.5	2.5	95.201	0.00214	2.2233	5.85E+08
5	5	23.2	23.2	23.2	39.051		94.827	1.11E+10
10	10	39.8	39.8	39.8	36.49		167.74	4.2E+10
5	5	11.6	11.6	11.6	58.59		38.5	1.81E+09
22	22	36.8	36.8	36.8	71.509		323.31	1.74E+11
2	2	5.7	5.7	5.7	42.27	0.000692	14.078	6.46E+08
8	8	54.6	54.6	54.6	20.437		261.71	1.54E+11
6	6	15.1	15.1	15.1	52.654	0.000706	16.039	1.69E+09

1	1	4.2	4.2	4.2	46.063		73.364	1.79E+08
3	3	40.4	40.4	40.4	15.526		46.223	1.06E+10
4	4	31.8	31.8	31.8	16.578		179.75	8.35E+09
2	2	7.4	7.4	7.4	50.891	0.000555	3.134	73968000
10	10	52.1	52.1	52.1	15.36		242.98	7.4E+10
4	4	12.6	12.6	12.6	40.401		35.739	1.03E+10
3	3	20.1	20.1	20.1	28.774		124.26	1.11E+10
1	1	16.1	16.1	16.1	12.367		76.454	3.59E+09
4	4	16	16	16	30.27	0.000571	4.0143	7.01E+08
8	8	32.8	32.8	32.8	33.498		323.31	1.38E+10
3	3	26.8	26.8	26.8	28.918	0.00072	17.616	1.46E+09
2	2	20.9	20.9	20.9	16.056	0.000581	4.4039	3.16E+09
1	1	20	20	20	8.0345	0.000625	7.9844	3.08E+10
6	6	54.3	54.3	54.3	19.03		323.31	3.64E+10
6	6	36.6	36.6	36.6	20.88		67.949	3.08E+10
3	3	23.6	23.6	23.6	21.055	0.000641	9.1854	4.34E+08
2	2	7	7	7	42.7	0.000556	3.1824	3.57E+08
10	10	63.4	63.4	63.4	20.102		166.5	3.47E+11
4	4	42	42	42	16.619		124.5	1.72E+10
2	2	9.7	9.7	9.7	34.134	0.000626	8.0919	3.97E+08
1	1	5.9	5.9	5.9	37.261		26.208	3.35E+09
2	2	14.7	14.7	14.7	26.788	0.000681	12.967	4.8E+09
4	4	21.8	21.8	21.8	40.309		50.894	2.95E+09
2	2	11.9	11.9	11.9	26.901	0.000664	11.367	4.49E+08
1	1	6.5	6.5	6.5	25.347	0.000664	11.369	3.96E+08
2	2	7.6	7.6	7.6	34.4	0.001101	2.927	1.1E+08
4	4	35.8	35.8	35.8	10.625		43.32	5.25E+10
6	6	34.7	34.7	34.7	29.144		323.31	1.75E+10
3	3	26.5	26.5	26.5	21.84		57.907	4.45E+09
3	3	10.9	10.9	10.9	42.398		323.31	3.08E+10
6	6	19.5	19.5	19.5	64.473		99.88	4.88E+09
2	2	8.1	8.1	8.1	26.256	0.000711	16.379	1.51E+09
2	2	7.2	7.2	7.2	28.948	0.000554	3.077	6.18E+08
20	20	30.3	30.3	30.3	93.808		323.31	7.68E+10
4	4	19.7	19.7	19.7	31.803	0.000565	3.641	7.53E+10
1	1	20	20	20	9.88		34.345	8.54E+08
1	1	5.7	5.7	5.7	23.887	0.002139	2.2146	9.79E+08
8	8	26.2	26.2	26.2	39.579		323.31	2.42E+11
11	11	32.1	32.1	32.1	45.761		147.75	1.11E+10
4	4	18.2	18.2	18.2	44.084		125.21	5.56E+09
1	1	3.5	3.5	3.5	46.457	0.000714	16.829	2.51E+08
3	3	18.6	18.6	18.6	22.963		63.219	7.46E+09
7	7	40.3	40.3	40.3	22.83		141.08	1.26E+10
2	2	12	12	12	20.031	0.000565	3.5901	3.07E+09
5	5	49.6	49.6	49.6	12.916		323.31	3.27E+10
3	3	32.5	32.5	32.5	16.181		284.07	3.33E+10
6	6	76.4	76.4	76.4	16.5		323.31	1.26E+10
2	2	7	7	7	49.355		141.23	1.77E+09
3	3	15.2	15.2	15.2	39.575	0.000556	3.1359	6.23E+08
1	1	11.5	11.5	11.5	15.441	0.000623	7.7741	1.26E+09
5	5	18.9	18.9	18.9	46.071		323.31	4.74E+09

10	10	49.2	49.2	49.2	33.486		323.31	2.28E+10
2	2	11	11	11	18.975		44.268	7.48E+08
1	1	6.2	6.2	6.2	19.086		34.501	1.54E+09
6	6	29.9	29.9	29.9	36.395		186.61	5.78E+09
2	2	12.9	12.9	12.9	26.608		63.735	2.2E+09
1	1	23.7	23.7	23.7	8.2134	0.00068	12.892	1.62E+09
7	7	33.4	33.4	33.4	32.986		179.27	1.45E+10
8	8	26.6	26.6	26.6	50.05		323.31	1.71E+10
1	1	4.9	4.9	4.9	22.12	0.000644	9.3507	3.55E+09
2	2	16.5	16.5	16.5	20.268		37.388	7.92E+09
1	1	7.6	7.6	7.6	20.297	0.00062	7.6241	7.73E+08
5	5	22.7	22.7	22.7	37.623		63.525	1.82E+09
1	1	10.7	10.7	10.7	17.309		29.982	1.72E+08
1	1	8.2	8.2	8.2	20.376	0.000612	6.8148	4.07E+09
1	1	7.8	7.8	7.8	28.2		54.359	2.32E+08
3	3	17	17	17	22.614	0.000749	21.352	4.15E+08
3	3	24	24	24	28.775		125.49	3.97E+10
2	2	6.6	6.6	6.6	57.357	0.006276	1.7519	1.32E+09
2	2	19.3	19.3	19.3	16.058		274.36	1.08E+10
4	4	8.6	8.6	8.6	91.639		66.736	2.53E+09
10	10	41.6	41.6	41.6	40.821		323.31	3.75E+10
3	3	12	12	12	28.15	0.000597	5.6022	7.29E+09
8	8	18.8	18.8	18.8	52.43		58.944	1.68E+10
10	10	44	44	44	27.979		249.68	2.94E+10
15	15	33.5	33.5	33.5	52.221		323.31	5.48E+10
17	17	45.3	45.3	45.3	49.305		323.31	9.43E+10
2	2	10.1	10.1	10.1	33.504	0.001088	2.655	1.23E+08
8	8	21	21	21	59.725		49.312	2.53E+10
4	4	19.5	19.5	19.5	21.12		323.31	2.97E+09
1	1	6.1	6.1	6.1	21.403		29.213	5.81E+09
3	3	16.1	16.1	16.1	38.151		179.88	3.8E+09
11	11	36	36	36	58.968		323.31	4.44E+10
6	6	26.7	26.7	26.7	24.377		56.833	7.54E+09
7	7	35.1	35.1	35.1	30.466		34.411	6.42E+09
2	2	20.1	20.1	20.1	18.534		198.76	6.38E+09
4	4	13.5	13.5	13.5	38.566		323.31	1.06E+10
4	4	47.9	47.9	47.9	12.692		243.52	8.33E+09
2	2	7.8	7.8	7.8	40.135	0.001623	2.5234	2.27E+08
14	14	40.1	40.1	40.1	40.266		323.31	2.8E+11
4	4	38.5	38.5	38.5	14.377	0.000661	10.953	2.2E+10
6	6	21.4	21.4	21.4	35.363	0.000629	8.3151	1.15E+09
1	1	7.9	7.9	7.9	34.747	0.000577	4.221	1.52E+08
4	4	21.6	21.6	21.6	21.075		43.433	2.03E+10
2	2	26.5	26.5	26.5	15.874		32.342	4.63E+08
8	8	25.1	25.1	25.1	52.319		36.54	9.95E+09
2	2	2.9	2.9	2.9	68.814	0.000579	4.3016	5.58E+08
6	6	17.9	17.9	17.9	75.116		323.31	1.53E+10
9	9	21.7	21.7	21.7	70.502		139.21	2.85E+10
9	9	36.1	36.1	36.1	35.852		323.31	2.09E+10
1	1	10	10	10	36.791	0.000707	16.193	2.09E+08
13	13	31.5	31.5	31.5	62.714		323.31	3.72E+10

5	5	32.2	32.2	32.2	28.432		234.73	7.53E+09
9	9	16.1	16.1	16.1	86.943		323.31	1.07E+11
2	2	31	31	31	15.407		323.31	6.46E+09
2	2	21.4	21.4	21.4	16.866		27.424	2.32E+10
3	3	18.5	18.5	18.5	20.704		293.71	4.02E+09
1	1	8.7	8.7	8.7	20.212		83.256	1.08E+08
13	13	35.9	35.9	35.9	49.59		323.31	1.22E+11
2	2	21.7	21.7	21.7	13.222	0.000617	7.349	3.24E+09
1	1	6.5	6.5	6.5	16.381	0.009724	1.4583	2.78E+09
5	5	18.8	18.8	18.8	37.44		137.53	9.59E+09
7	7	25.3	25.3	25.3	48.817		43.355	5.65E+09
8	8	31.4	31.4	31.4	44.617		323.31	9.88E+09
5	5	35.3	35.3	35.3	22.338		66.279	3.56E+09
2	2	37.8	37.8	37.8	8.9433	0.000636	8.7489	4.54E+09
11	11	46.7	46.7	46.7	36.196		323.31	5E+10
1	1	8.3	8.3	8.3	16.469	0.001096	2.8351	2.09E+09
1	1	4.3	4.3	4.3	28.081	0.000552	3.0597	3.14E+09
5	5	23.6	23.6	23.6	29.363		32.649	3.71E+09
2	2	6.3	6.3	6.3	45.802		61.344	2.56E+09
1	1	12.9	12.9	12.9	20.826	0.003198	2.1456	34573000
3	3	13.1	13.1	13.1	30.952		101.3	4.09E+09
7	7	25.8	25.8	25.8	41.377		323.31	2.22E+10
3	3	8.7	8.7	8.7	46.692	0.004762	1.9808	3.49E+08
16	16	31.6	31.6	31.6	69.309		323.31	3.36E+11
8	8	38.5	38.5	38.5	35.037		323.31	3.08E+10
1	1	7.6	7.6	7.6	20.312	0.000569	3.8394	1.84E+08
6	6	16.5	16.5	16.5	34.991	0.00069	13.989	8.71E+09
5	5	43.5	43.5	43.5	17.949		117.57	8.44E+09
6	6	9.8	9.8	9.8	84.141		41.68	5.56E+09
16	16	22.8	21.6	21.6	90.135		323.31	2.92E+10
6	6	48.4	48.4	48.4	18.064		323.31	4.87E+10
7	7	35.8	35.8	35.8	23.79		216.21	9.38E+09
8	8	42.4	42.4	42.4	28.492		209.36	1.61E+10
13	13	21.7	21.7	21.7	92.831		121.35	1.01E+10
8	7	26.7	26.7	23.6	45.066		86.855	6.67E+09
6	6	68.8	68.8	68.8	17.492		104.35	8.63E+08
2	2	7.4	7.4	7.4	19.024	0.001616	2.3874	1.3E+09
14	14	50.4	50.4	50.4	41.086		323.31	9.74E+10
6	6	27.3	27.3	27.3	34.881	0.000599	5.8093	4.38E+09
7	7	22.1	22.1	22.1	56.581		183.07	2.45E+10
2	2	6.9	6.9	6.9	39.925	0.000577	4.2226	3.35E+08
1	1	17.5	17.5	17.5	10.922	0.000687	13.667	1.35E+09
3	3	13.4	13.4	13.4	31.284		140.41	4.41E+10
7	7	42.9	42.9	42.9	26.213		323.31	2.46E+10
11	11	33.6	33.6	33.6	44.469		243.49	2.8E+10
6	6	24.3	24.3	24.3	41.54		142.51	7.15E+09
4	4	14.8	14.8	14.8	30.306		52.721	9.14E+09
4	4	16.8	16.8	16.8	40.846	0.000591	5.2599	2.99E+09
3	3	10.9	10.9	10.9	37.116	0.000724	18.266	1.95E+09
1	1	12.9	12.9	12.9	13.974	0.000616	7.317	5.01E+08
5	5	22.2	22.2	22.2	27.834		27.818	3.56E+09

1	1	10.7	10.7	10.7	28.769		50.457	3E+09
1	1	6.6	6.6	6.6	28.32		34.261	1.34E+09
1	1	8.9	8.9	8.9	17.763		33.336	1.05E+09
5	5	31.9	31.9	31.9	29.523		193.43	1.61E+10
7	7	19.2	19.2	19.2	44.968		215.66	4.48E+09
1	1	8.1	8.1	8.1	13.428		74.346	1.6E+09
3	3	15.4	15.4	15.4	29.857	0.000569	3.8588	1.13E+09
4	4	6.6	6.6	6.6	86.636	0.000626	8.0587	3.16E+09
6	6	30	30	30	40.347		117	4.42E+09
15	15	17.6	17.6	17.6	89.824		323.31	4.42E+10
5	5	31.6	31.6	31.6	26.742		64.228	5.84E+09
1	1	2.8	2.8	2.8	59.684	0.000602	6.0222	84681000
1	1	5.7	5.7	5.7	26.351		56.348	6.82E+08
1	1	2.8	2.8	2.8	47.05	0.000551	2.9753	1.04E+09
5	5	46.2	46.2	46.2	14.48	0.000667	11.545	9.74E+08
8	8	34.6	34.6	34.6	40.722		187.45	2.62E+10
7	7	35.9	35.9	35.9	21.983		285.84	4.12E+10
5	5	19.1	19.1	19.1	36.234		146.79	3.6E+09
5	5	12.2	12.2	12.2	52.745		48.078	8.94E+09
14	14	63.6	63.6	63.6	35.095		323.31	5.13E+10
2	2	14.6	14.6	14.6	22.129	0.000578	4.249	3.36E+08
1	1	9.4	9.4	9.4	20.775		34.852	1.01E+08
2	2	18.5	18.5	18.5	18.115		115.84	1.39E+09
2	2	6.2	6.2	6.2	41.792	0.000618	7.5817	2.8E+09
5	5	36.7	36.7	36.7	24.622		156.11	4.19E+09
18	18	41.2	41.2	41.2	53.141		323.31	1.57E+11
4	4	10.7	10.7	10.7	67.024		48.881	8.4E+08
2	2	45.2	45.2	45.2	8.3483		231.04	3.01E+09
2	2	6.3	6.3	6.3	42.798	0.001095	2.835	46854000
4	4	20.1	20.1	20.1	30.266		237.54	4.05E+09
4	4	28.4	28.4	28.4	35.843		136.19	1.73E+09
12	12	54.2	54.2	54.2	46.395		242.14	1.74E+10
4	4	12.1	12.1	12.1	44.323		25.481	4.15E+09
2	2	15.2	15.2	15.2	23.259		73.918	2.15E+09
5	5	36	36	36	14.53		76.514	1.54E+10
10	10	37.9	37.9	37.9	38.671		323.31	4.4E+10
3	3	28	28	28	10.142	0.000593	5.3761	1.3E+11
2	2	4.2	4.2	4.2	30.029	0.0006	5.8771	
2	2	15.9	15.9	15.9	14.181	0.001093	2.8016	9.64E+08
1	1	6.6	6.6	6.6	16.435	0.000745	20.924	2.75E+09
2	2	29.6	29.6	29.6	16.59		70.403	1.54E+09
7	7	11.6	11.6	11.6	88.464		83.548	6.55E+10
1	1	6.7	6.7	6.7	28.076		61.654	1.27E+09
14	14	32.3	32.3	32.3	49.236		240.01	2.61E+10
2	2	7.8	7.8	7.8	34.006	0.000575	4.1511	2.86E+09
8	8	54.5	54.5	54.5	23.7		323.31	4.18E+10
3	3	20.6	20.6	20.6	32.147	0.000646	9.6129	1.82E+09
6	6	15.2	15.2	15.2	55.835		63.894	6.87E+09
4	4	15.8	15.8	15.8	47.099		46.002	2.97E+09
3	3	10.7	10.7	10.7	28.559	0.000569	3.8771	1.12E+10
16	16	22.1	22.1	22.1	82.283		323.31	5.11E+10

3	3	15.6	15.6	15.6	33.36		107.94	4.05E+09
4	4	15.8	15.8	15.8	38.728		70.263	8E+09
1	1	5.4	5.4	5.4	27.244		84.173	4.6E+08
1	1	15.9	15.9	15.9	20.488	0.000718	17.345	1.98E+08
3	3	21	21	21	22.067	0.005269	1.9203	2.1E+08
2	2	23.1	23.1	23.1	14.937	0.000759	22.482	1.97E+09
3	3	15.7	15.7	15.7	35.568	0.000717	17.207	8.63E+08
1	1	13.2	13.2	13.2	14.279		187.33	3.07E+09
3	3	32	32	32	13.91		51.312	1.82E+10
2	2	27	27	27	12.398	0.000743	20.73	3.61E+08
15	15	39.7	39.7	39.7	44.177		180.95	9.19E+10
5	5	29.2	29.2	29.2	28.411	0.000649	9.772	1.62E+09
2	2	7.9	7.9	7.9	34.962	0.000665	11.385	5.99E+09
7	7	17.8	17.8	17.8	50.812	0.000742	20.649	9.48E+09
12	12	43.3	43.3	43.3	42.119		295.68	7.04E+10
2	2	5.2	5.2	5.2	50.599	0.001097	2.8476	7.37E+08
11	11	42.8	42.8	42.8	48.886		323.31	1.99E+10
3	3	9.6	9.6	9.6	38.29		304.12	1.5E+10
2	2	14.9	14.9	14.9	19.435	0.00056	3.2786	5.09E+09
6	6	12.9	12.9	12.9	82.596		170.66	1.04E+10
18	18	54	54	54	46.392		169.03	1.77E+10
9	9	85.1	85.1	85.1	15.174		323.31	8.19E+10
1	1	5.9	5.9	5.9	23.868	0.000756	22.199	7.85E+08
2	2	13.7	13.7	13.7	29.843	0.001098	2.8713	1.27E+08
3	3	15.2	15.2	15.2	36.925	0.000603	6.0385	1.11E+09
4	4	19.3	19.3	19.3	45.145		64.852	6.06E+09
5	5	23	23	23	35.746		323.31	5.15E+10
2	2	7.5	7.5	7.5	14.852	0.00626	1.7182	1.64E+08
3	2	7.5	7.5	6.2	56.659	0.001613	2.3516	4.1E+08
2	2	21.8	21.8	21.8	14.265	0.001622	2.4822	3.45E+08
1	1	6.2	6.2	6.2	16.986		210.99	8.31E+10
3	3	10.5	10.5	10.5	32.632		114.97	4.79E+08
4	4	29.3	29.3	29.3	30.235		225.11	4.57E+09
2	2	12.2	12.2	12.2	21.367	0.003725	2.1256	5.35E+08
6	6	67.6	67.6	67.6	17.991		323.31	2.29E+10
8	8	26.3	26.3	26.3	51.341		46.81	1.12E+10
15	15	28.2	28.2	28.2	73.271		323.31	4.39E+10
4	4	14.4	14.4	14.4	39.498	0.000561	3.33	3.99E+09
1	1	10.4	10.4	10.4	21.741		138.76	7.02E+08
11	11	25.7	25.7	25.7	50.963	0.000741	20.626	5.87E+09
4	4	22.6	22.6	22.6	45.225		82.422	3.63E+09
6	6	17.9	17.9	17.9	47.068	0.000723	18.009	7.5E+10
4	4	13.2	13.2	13.2	32.186		64.236	9.23E+09
10	10	46.7	46.7	46.7	28.951		67.592	2.22E+10
2	2	29.6	29.6	29.6	13.141	0.000568	3.8035	2.73E+08
5	5	26.8	26.8	26.8	23.262	0.000576	4.2042	1.06E+09
10	10	25.9	25.9	25.9	57.628		211.88	1.2E+10
3	3	14.9	14.9	14.9	41.444	0.000652	10.283	1.41E+08
5	5	34.7	34.7	34.7	18.462		37.781	2.48E+09
3	3	25.2	25.2	25.2	12.815		56.798	4.25E+10
4	4	23.6	23.6	23.6	22.714	0.000633	8.4936	3.37E+10

4	4	14.3	14.3	14.3	35.875		61.294	2.52E+09
7	7	26	26	26	33.268		137.82	7.89E+09
4	4	13.5	13.5	13.5	43.671	0.000666	11.45	4.15E+09
10	10	42	42	42	26.148		323.31	8.66E+10
3	3	16.2	16.2	16.2	36.166	0.00059	5.1488	2.51E+09
2	2	62.8	62.8	62.8	9.0725		62.21	2.67E+09
1	1	12.6	12.6	12.6	13.149	0.000611	6.7081	1.47E+09
6	5	16.4	16.4	14.2	41.459		33.098	1.59E+10
1	1	5.2	5.2	5.2	28.525	0.001611	2.3466	1.03E+09
12	12	31.5	31.5	31.5	63.864		307.59	8.01E+10
3	3	31.9	31.9	31.9	17.694	0.000611	6.8015	5.47E+08
5	5	26.7	26.7	26.7	32.325		23.466	1.66E+09
1	1	17.4	17.4	17.4	12.209		25.084	8.92E+08
3	3	16.6	16.6	16.6	27.598		26.283	5.86E+09
1	1	7.7	7.7	7.7	22.349		28.832	1.58E+08
7	7	27.6	27.6	27.6	35.748		323.31	4.6E+10
3	3	23.3	23.3	23.3	18.302		127.34	2.18E+10
1	1	6.2	6.2	6.2	41.256		50.64	1.07E+09
2	2	8	8	8	24.642	0.001625	2.5395	1.01E+10
2	2	19.1	19.1	19.1	16.319		25.938	6.27E+08
2	2	13.7	13.7	13.7	16.903		290.79	4.28E+09
20	20	45.3	45.3	45.3	76.967		323.31	7.01E+10
9	9	35.3	35.3	35.3	35.49		323.31	4.39E+10
1	1	14.8	14.8	14.8	18.746		225.23	5.97E+10
8	8	37.6	37.6	37.6	33.447		138.94	2.49E+10
3	3	21.1	21.1	21.1	28.917		95.544	1.86E+10
2	2	13.1	13.1	13.1	27.934		171.45	4.38E+09
2	2	14.6	14.6	14.6	17.992	0.000636	8.7662	2.15E+09
3	3	29.7	29.7	29.7	20.25		112.81	5.95E+09
1	1	7.4	7.4	7.4	14.008	0.0032	2.1611	1.38E+09
4	4	6.8	6.8	6.8	76.938	0.000714	16.835	2.89E+09
8	8	26.3	26.3	26.3	45.542		158.1	1.01E+10
4	4	18.3	18.3	18.3	53.246		133.17	7.79E+08
9	9	24.5	24.5	24.5	62.776		323.31	1.66E+10
1	1	1.6	1.6	1.6	86.721	0.003197	2.1438	62724000
5	5	13.9	13.9	13.9	66.895		323.31	1.36E+10
6	6	25.1	25.1	25.1	39.423		139.94	6.18E+10
6	6	17.6	17.6	17.6	56.208		43.2	5.38E+09
21	21	83.3	83.3	83.3	37.281		323.31	7.72E+10
2	2	13.5	13.5	13.5	24.956	0.000678	12.724	1.19E+09
2	2	22	22	22	13.112	0.000561	3.316	1.48E+08
8	8	23.8	23.8	23.8	34.94		89.064	6.27E+10
15	15	41.9	41.9	41.9	56.028		323.31	4.9E+10
4	4	21.7	21.7	21.7	34.324		323.31	2.41E+10
1	1	14.5	14.5	14.5	13.859	0.000688	13.676	6.83E+08
4	4	37.1	37.1	37.1	12.769		34.392	5.61E+10
4	4	47.8	47.8	47.8	16.638		22.848	3.14E+09
7	7	38.6	38.6	38.6	20.581		323.31	5.62E+10
3	3	29.2	29.2	29.2	15.72		41.084	3.62E+09
7	7	28.5	28.5	28.5	48.499	0.000747	21.128	7.24E+09
1	1	4.7	4.7	4.7	37.802		30.159	9.92E+08

5	5	45.1	45.1	45.1	26.891	0.000735	19.856	8.29E+09
14	14	41.1	41.1	41.1	50.683		323.31	4.23E+10
29	29	37.1	37.1	37.1	102.4		323.31	7.83E+10
4	4	18.9	18.9	18.9	33.69		288.02	1.3E+10
18	18	39	39	39	65.599		323.31	3.09E+10
12	12	44	44	44	39.289		299.8	7.39E+10
2	2	15.3	15.3	15.3	20.888	0.0007	15.55	5.8E+08
15	15	59.7	59.7	59.7	32.421		323.31	1.56E+11
8	8	25.5	25.5	25.5	45.195		319.63	4.47E+10
1	1	3.5	3.5	3.5	38.254	0.000568	3.7719	5.9E+09
2	2	32.8	32.8	32.8	13.683	0.00071	16.362	1.17E+10
17	17	59.3	59.3	59.3	48.106		323.31	6.68E+10
4	4	14.3	14.3	14.3	24.627		22.774	8.79E+09
4	4	15.6	15.6	15.6	37.227	0.000662	11.098	8.16E+09
4	4	12.2	12.2	12.2	41.716		285	9.12E+09
15	15	42.2	42.2	42.2	42.024		168.83	6.02E+10
13	13	46.5	46.5	46.5	38.001		323.31	3.09E+11
7	7	21.6	21.6	21.6	34.47		323.31	1.05E+10
5	5	21.6	21.6	21.6	41.501		323.31	1.1E+10
2	2	11.1	11.1	11.1	24.452	0.000711	16.368	1.49E+09
1	1	7.3	7.3	7.3	35.475		27	7.31E+09
2	2	5.3	5.3	5.3	59.182		30.763	5.12E+08
3	3	8.3	8.3	8.3	44.368		29.01	1.04E+09
7	7	19.1	19.1	19.1	59.444		323.31	1.19E+10
3	3	18.8	18.8	18.8	20.458		31.931	1.15E+09
13	13	32	32	32	47.28		323.31	3.61E+10
1	1	2.6	2.6	2.6	63.413		57.796	2.2E+09
7	7	17.7	17.7	17.7	63.57		234.86	6.52E+08
2	2	14.9	14.9	14.9	21.271	0.000745	20.901	7.17E+08
10	10	29.9	29.9	29.9	45.524		158.24	1.85E+10
11	11	22.6	22.6	22.6	59.917		323.31	6.92E+10
1	1	4.8	4.8	4.8	34.436	0.005272	1.9277	1.19E+09
18	18	47.2	47.2	47.2	47.471		323.31	2.1E+11
2	2	12.7	12.7	12.7	11.275		99.257	1.22E+10
3	3	32	32	32	18.663		127.75	1.22E+10
13	13	59.2	59.2	59.2	31.021		323.31	4.14E+10
5	5	13	13	13	76.109		323.31	1.58E+10
2	2	7.4	7.4	7.4	41.151		43.974	1.04E+09
2	2	11.5	11.5	11.5	20.521		25.29	3.35E+09
14	14	17.8	17.8	17.8	116.91		323.31	3.34E+10
2	2	35.8	35.8	35.8	8.8859		39.015	3.94E+08
4	4	14.2	14.2	14.2	46.123	0.00066	10.777	2.35E+09
4	4	52.6	52.6	52.6	14.366		124.46	2.03E+09
5	5	21.9	21.9	21.9	32.183		323.31	1.05E+10
5	5	12.3	12.3	12.3	56.922		35.515	3.14E+09
5	5	26.5	26.5	26.5	27.19		323.31	2.92E+10
2	2	8.3	8.3	8.3	25.726	0.000564	3.5521	72379000
6	6	13	13	13	63.252		116.7	8.25E+09
2	2	18	18	18	14.907		37.945	1.27E+10
5	5	11.7	11.7	11.7	59.54		64.928	2.73E+09
6	6	39.6	39.6	39.6	21.984		118.03	5.11E+09

7	7	22.7	22.7	22.7	47.984		111.87	6.87E+09
7	7	15.1	15.1	15.1	77.931		323.31	1.61E+10
2	2	5	5	5	69.544		36.604	1.09E+09
1	1	1.9	1.9	1.9	50.953	0.001086	2.6279	7.02E+08
3	3	11.9	11.9	11.9	46.524		54.793	1.73E+09
5	5	45.4	45.4	45.4	22.214		323.31	8.13E+09
6	6	20.1	20.1	20.1	41.734		323.31	7.27E+09
19	19	33.1	33.1	33.1	75.807		323.31	1.33E+11
3	3	12.7	12.7	12.7	34.51		28.546	2.28E+09
4	4	30.7	30.7	30.7	17.556	0.000582	4.4943	1.82E+09
13	13	32	32	32	32.287		323.31	1.18E+11
7	7	29.8	29.8	29.8	33.085		323.31	5.44E+10
21	21	38.6	38.6	38.6	55.942		323.31	2.13E+11
2	2	29.7	29.7	29.7	7.9002	0.000573	4.0446	1.34E+09
3	3	20.2	20.2	20.2	24.659	0.000684	13.337	3.68E+09
6	6	17	17	17	53.183		116.43	3.18E+09
1	1	22.9	22.9	22.9	19.744	0.000737	20.118	2.11E+09
10	10	32.5	32.5	32.5	50.821		323.31	9.7E+09
5	5	27.5	27.5	27.5	27.399		38.241	3.26E+09
3	3	18.1	18.1	18.1	19.316	0.000564	3.5244	1.74E+09
6	6	5.5	5.5	5.5	148.34	0.000741	20.579	9.92E+09
1	1	1	1	1	196.16	0.00675	1.62	52728000
4	4	8.3	8.3	8.3	70.914		54.177	1.8E+10
13	13	41.5	41.5	41.5	63.53		323.31	6.25E+10
14	14	43.9	43.9	43.9	44.673		96.644	4.65E+10
5	5	23.1	23.1	23.1	24.567		42.881	4.99E+10
1	1	6.7	6.7	6.7	22.815	0.00056	3.2763	
10	10	34.7	34.7	34.7	45.866		323.31	3.48E+10
16	16	45.7	45.7	45.7	47.265		323.31	1.8E+11
3	3	10.4	10.4	10.4	41.629		29.192	1.01E+09
2	2	20.6	20.6	20.6	19.201		35.298	1.54E+09
6	6	38.2	38.2	38.2	9.2178		186.76	1.91E+10
4	4	14.8	14.8	14.8	33.498		38.024	3.97E+09
13	13	43	43	43	42.492		323.31	1.09E+11
7	7	40.9	40.9	40.9	29.738		138.25	3.28E+10
4	4	32	32	32	22.239		323.31	2.61E+10
10	10	51.1	51.1	51.1	20.489		294.54	7.13E+10
1	1	8.7	8.7	8.7	21.571		38.033	1.37E+09
1	1	11.2	11.2	11.2	14.659	0.009744	1.4663	2.76E+08
15	15	17.3	17.3	17.3	111.16		323.31	4.83E+10
7	7	17.6	17.6	17.6	55.756	0.000643	9.2806	2.86E+09
6	6	19.4	19.4	19.4	53.823		323.31	9.65E+09
3	3	10.4	10.4	10.4	61.594		69.68	3.2E+09
4	4	18.8	18.8	18.8	46.575		323.31	2.68E+10
3	3	9.6	9.6	9.6	37.08		26.7	5.05E+09
3	3	25.5	25.5	25.5	16.639		58.173	7.96E+09
2	2	15.9	15.9	15.9	29.838		41.726	1.42E+09
5	5	37	37	37	23.84		48.657	2.76E+09
3	3	21.4	21.4	21.4	31.89		323.31	1.5E+10
1	1	4.6	4.6	4.6	28.029		85.618	1.03E+10
8	8	32.8	32.8	32.8	28.994		82.628	1.44E+10

3	3	13.4	13.4	13.4	36.041		312.64	1.55E+10
5	5	21.5	21.5	21.5	36.507		31.44	9.54E+09
5	5	25.7	25.7	25.7	25.102	0.000692	14.119	2.15E+09
4	4	17.8	17.8	17.8	30.963		84.632	9.36E+09
1	1	11.5	11.5	11.5	11.682	0.006725	1.5964	95207000
5	5	37.9	37.9	37.9	19.688		323.31	1.67E+10
5	5	44.7	44.7	44.7	22.321		79.619	2.17E+09
1	1	4.9	4.9	4.9	39.725	0.000656	10.596	1.5E+08
13	13	46.6	46.6	46.6	39.408		323.31	2.01E+10
1	1	11.7	11.7	11.7	18.012	0.000651	10.089	6.9E+08
3	3	24.1	24.1	24.1	20.851		32.532	8.25E+09
5	5	36.3	36.3	36.3	20.744		67.472	3.22E+09
7	7	28.9	28.9	28.9	41.674		85.591	1.61E+10
6	6	39.8	39.8	39.8	19.424		323.31	3.12E+10
11	11	21.8	21.8	21.8	63.666		272.44	2.2E+10
15	15	19.6	19.6	19.6	111.12		323.31	3.29E+10
4	4	16.2	16.2	16.2	26.866	0.00625	1.655	8.34E+08
6	6	40.9	40.9	40.9	17.44		48.242	2.51E+10
4	4	25.9	25.9	25.9	23.105		163.17	5.31E+09
5	5	29.6	29.6	29.6	36.071		80.287	3.45E+09
8	8	23.9	23.9	23.9	49.117		132.26	1.99E+10
1	1	10.3	10.3	10.3	15.939	0.000672	12.183	1.7E+10
5	5	22.8	22.8	22.8	33.404	0.000686	13.531	1.39E+10
1	1	3	3	3	43.351	0.009764	1.4841	3.49E+08
6	6	25.3	25.3	25.3	33.671		96.383	8.71E+09
3	3	16.8	16.8	16.8	27.511	0.000563	3.4597	8.74E+08
3	3	8.5	8.5	8.5	56.017	0.000677	12.719	8E+08
3	3	18.4	18.4	18.4	28.116		22.841	3.77E+09
2	2	9	9	9	42.56		22.927	1.47E+09
2	2	18.1	18.1	18.1	17.619	0.001609	2.317	1.12E+09
3	3	24.2	24.2	24.2	19.168		78.434	6.29E+09
5	5	41	41	41	24.572		65.882	2.02E+10
13	13	39.4	39.4	39.4	35.712		323.31	6.37E+11
2	2	9.2	9.2	9.2	37.376	0.000685	13.494	5.94E+09
3	3	31.8	31.8	31.8	14.384		32.533	3.35E+09
1	1	13.6	13.6	13.6	8.3471		323.31	2.92E+10
7	7	39.8	39.8	39.8	27.938		98.12	1.57E+10
5	5	30.5	30.5	30.5	31.18		108.25	2.1E+09
3	3	18.8	18.8	18.8	19.014	0.000752	21.734	4.8E+09
1	1	5.5	5.5	5.5	22.606	0.006764	1.6369	10033000
9	9	24.6	24.6	24.6	43.404		176.13	2.72E+10
11	11	29.2	29.2	29.2	56.587		323.31	3.5E+10
12	12	49	49	49	22.759		271.46	4.89E+10
4	4	17.8	17.8	17.8	36.546	0.000736	19.943	1.85E+10
5	5	34.7	34.7	34.7	28.287		118.2	1.9E+10
5	5	16.8	16.8	16.8	47.69		42.517	1.5E+09
6	6	34.5	34.5	34.5	24.97		260.96	2E+10
3	3	12.5	12.5	12.5	40.82		22.63	2.25E+09
1	1	5.1	5.1	5.1	30.264	0.000665	11.418	8.07E+08
3	3	15.5	15.5	15.5	39.84		233.56	1.93E+09
5	5	27.3	27.3	27.3	26.005		35.749	9.48E+09

5	5	11.9	11.9	11.9	57.686	0.000731	19.036	1.02E+10
1	1	4.8	4.8	4.8	32.456	0.001618	2.4122	38169000
5	5	35.7	35.7	35.7	28.534		48.013	2.75E+09
11	11	18.9	18.9	18.9	58.805		58.977	1.94E+10
4	4	11.4	11.4	11.4	57.533		222.91	2.83E+10
9	9	17.6	17.6	17.6	89.89		63.683	9.95E+09
11	11	21.4	21.4	21.4	91.908		323.31	3.42E+10
6	6	34.2	34.2	34.2	33.971		127.35	1.17E+10
4	4	20.8	20.8	20.8	30.479	0.000639	9.0188	3.57E+09
3	3	20.9	20.9	20.9	15.06	0.000638	8.9263	3.37E+09
2	2	31.3	31.3	31.3	9.3331	0.006732	1.6034	3.07E+08
3	3	18	18	18	26.188		70.69	2.87E+09
3	3	5.2	5.2	5.2	66.826	0.000566	3.6493	1.53E+08
5	5	5.6	5.6	5.6	98.718	0.000733	19.394	2.66E+09
1	1	3.5	3.5	3.5	42.231	0.008265	1.5363	2.7E+09
1	1	20.7	20.7	20.7	8.9633	0.000695	14.513	1.82E+09
11	11	33.9	33.9	33.9	41.543		250.01	1.25E+10
2	2	12.4	12.4	12.4	21.095	0.000561	3.3369	5.06E+08
7	7	32.3	32.3	32.3	45.134		323.31	8.82E+10
2	2	13.5	13.5	13.5	20.269	0.000571	3.986	2.86E+09
3	3	28.6	28.6	28.6	20.141	0.000574	4.1105	7.3E+08
9	9	34.2	34.2	34.2	30.883		298.25	2.39E+11
11	11	43.8	43.8	43.8	37.147		106.47	9.22E+09
3	3	16.4	16.4	16.4	18.903		190.72	2.84E+10
1	1	7.9	7.9	7.9	29.685	0.009674	1.4134	2.16E+08
5	5	22.8	22.8	22.8	19.294		76.219	3.64E+10
1	1	7.5	7.5	7.5	18.069		24.071	3.97E+10
1	1	3.3	3.3	3.3	43.358	0.00062	7.6197	
9	9	20.8	20.8	20.8	65.402		145.37	1.22E+10
2	2	15.4	15.4	15.4	30.654	0.000752	21.7	5.55E+08
2	2	20.4	20.4	20.4	19.415		29.407	1.45E+09
2	2	14.9	14.9	14.9	16.403	0.000634	8.5533	9.75E+08
12	12	24.2	24.2	24.2	86.572		275.16	7.83E+09
2	2	12.4	12.4	12.4	23.527	0.000647	9.6548	1.97E+09
3	3	11.3	11.3	11.3	49.736		168.56	1.46E+09
5	5	29.1	29.1	29.1	44.023		31.73	4.36E+09
3	3	26.8	26.8	26.8	19.642		273.06	3.22E+09
1	1	10.5	10.5	10.5	8.5869	0.005247	1.7928	1.07E+09
18	18	33.2	33.2	33.2	80.109		234.78	1.94E+10
2	2	12.8	12.8	12.8	25.785		61.729	1.47E+09
2	2	22.2	22.2	22.2	17.9	0.000739	20.385	1.7E+08
9	9	32	32	32	31.042		137.75	1.4E+10
4	4	20	20	20	38.183		101.77	8.28E+09
4	4	34.5	34.5	34.5	18.12	0.000589	5.0995	1.02E+10
2	2	17	17	17	17.493		66.734	2.16E+09
1	1	9.1	9.1	9.1	17.959	0.000627	8.1777	1.05E+09
3	3	20.2	20.2	20.2	28.321		108.62	7.07E+09
5	5	8.2	8.2	8.2	108.6	0.000557	3.1924	1.34E+09
5	5	20	20	20	35.767		56.687	3.62E+09
6	6	65.3	65.3	65.3	20.665		323.31	1.02E+10
7	7	18	18	18	66.059		255.51	1.73E+10

4	4	23.5	23.5	23.5	18.757		26.872	1.53E+10
2	2	7.6	7.6	7.6	41.143	0.000645	9.3552	8.25E+08
4	4	7.8	7.8	7.8	62.555	0.00073	18.997	7.24E+09
21	21	76.7	76.7	76.7	36.707		323.31	6.08E+11
4	4	31.4	31.4	31.4	21.25		113.74	5.27E+09
2	2	12.5	12.5	12.5	22.027	0.000608	6.3845	5.73E+09
2	2	20.1	20.1	20.1	19.174	0.000556	3.1914	2.52E+10
2	2	16	16	16	17.313		203.18	1.01E+10
4	4	16.2	16.2	16.2	28.622		253.95	5.42E+09
1	1	7.9	7.9	7.9	15.083		45.562	1.12E+10
6	6	18.5	18.5	18.5	41.959		275.94	1.41E+10
10	9	11.8	11.8	11.1	125.91		37.165	4.49E+09
5	5	26.1	26.1	26.1	33.845		323.31	5.14E+09
10	10	29.4	29.4	29.4	36.987		58.976	2.33E+10
1	1	4.5	4.5	4.5	21.865	0.005277	1.9312	6.94E+08
3	3	10.9	10.9	10.9	39.412		26.505	1.42E+09
8	8	51.9	51.9	51.9	17.652		239.76	1.8E+10
11	11	60.4	60.4	60.4	34.331		323.31	8.25E+09
1	1	4.5	4.5	4.5	28.629	0.004246	2.0372	3.48E+08
5	5	13.8	13.8	13.8	50.72	0.000646	9.5807	7.63E+08
9	9	25.7	25.7	25.7	44.334		153.58	8.7E+09
7	7	30.8	30.8	30.8	31.687		180.57	3.05E+10
4	4	22.2	22.2	22.2	31.95		38.18	2.74E+09
7	7	27.5	27.5	27.5	21.043		196.33	1.17E+11
5	5	19.9	19.9	19.9	26.263		116.7	1.37E+10
3	3	7.8	7.8	7.8	55.492		34.418	1.14E+10
3	3	32.8	32.8	32.8	14.003	0.000651	10.107	1.7E+09
4	4	19.1	19.1	19.1	36.212	0.000726	18.41	8.12E+08
3	3	15.1	15.1	15.1	23.595	0.009269	1.5054	5.68E+09
6	6	22.5	22.5	22.5	49.622		39.477	1.04E+10
11	11	36.8	36.8	36.8	46.055		208.52	1.43E+10
4	4	8.5	8.5	8.5	74.218		300.45	7.97E+09
1	1	16.5	16.5	16.5	12.417	0.000691	14.038	1.35E+09
19	19	46.6	46.6	46.6	42.8		323.31	5.31E+11
7	7	28	28	28	39.699		323.31	1.37E+10
2	2	9.9	9.9	9.9	26.693	0.001091	2.755	2.99E+09
1	1	2.9	2.9	2.9	36.888	0.009699	1.4352	1.41E+09
13	13	22.5	22.5	22.5	82.041		302.46	4.71E+10
4	4	13.2	13.2	13.2	38.311	0.000722	17.877	1.9E+09
7	7	19.8	19.8	19.8	42.388		302.97	1.62E+10
5	5	29.7	29.7	29.7	20.238		127.22	6.31E+10
2	2	24.1	24.1	24.1	15.663	0.00059	5.1582	2.94E+08
1	1	17.9	17.9	17.9	10.649		268.53	6.28E+08
5	5	9.9	9.9	9.9	82.415	0.000733	19.475	2.36E+09
1	1	5.8	5.8	5.8	37.285	0.000572	4.0226	4.66E+08
4	4	25.9	25.9	25.9	25.469		33.015	1.44E+09
1	1	14.5	14.5	14.5	17.819		25.527	7.3E+08
1	1	12.4	12.4	12.4	17.551	0.000713	16.764	1.44E+08
2	2	6.5	6.5	6.5	39.287	0.001621	2.4685	3.4E+08
1	1	5.2	5.2	5.2	31.789	0.000624	7.9064	2.16E+08
12	12	24.3	24.3	24.3	77.002		277	1.2E+10

4	4	28.3	28.3	28.3	23.697		114.88	2.17E+09
2	2	6.1	6.1	6.1	32.592	0.008273	1.5631	1.07E+08
1	1	2.4	2.4	2.4	45.017	0.009719	1.4578	1.16E+09
4	4	18.3	18.3	18.3	42.22	0.000681	12.986	9.71E+08
6	6	29.5	29.5	29.5	40.629		54.683	4.16E+09
2	2	21	21	21	11.874		30.448	2.54E+10
2	2	20.5	20.5	20.5	8.1522	0.00063	8.3255	1.19E+08
3	3	24.3	24.3	24.3	20.864	0.000642	9.2318	2.81E+09
22	22	69.2	69.2	69.2	36.1		323.31	1.89E+11
9	9	29.6	29.6	29.6	45.402		323.31	1.39E+10
13	13	38.4	38.4	38.4	40.975		217.32	9.25E+10
2	2	9.6	9.6	9.6	30.905	0.000554	3.092	5.63E+08
2	2	17.5	17.5	17.5	22.037	0.00058	4.357	62056000
1	1	5.3	5.3	5.3	39.372	0.006743	1.617	1.06E+08
4	4	25.4	25.4	25.4	25.935	0.000728	18.682	9.17E+08
4	4	37.6	37.6	37.6	19.731	0.000732	19.36	2.53E+09
1	1	13.7	13.7	13.7	14.423	0.000621	7.68	2.97E+08
3	3	13.5	13.5	13.5	38.245		80.824	9.55E+08
22	22	76.1	76.1	76.1	40.967		323.31	2.27E+11
2	2	2.8	2.8	2.8	50.309	0.000588	4.9583	1.27E+10
9	9	49.5	49.5	49.5	30.61		63.699	9.26E+09
5	5	25	25	25	27.233		163.73	1.03E+10
4	4	13.6	13.6	13.6	37.877	0.000624	7.8879	4.2E+09
2	2	11.1	11.1	11.1	41.634	0.000579	4.3106	88933000
9	9	27.8	27.8	27.8	48.795		81.077	1.44E+10
5	5	22.6	22.6	22.6	36.822		323.31	1.03E+10
4	4	30.5	30.5	30.5	17.947		211.16	7.62E+09
2	2	5	3.5	3.5	70.292	0.000574	4.1101	2.02E+09
17	17	42.7	42.7	42.7	57.194		323.31	1.39E+11
8	8	24.4	24.4	24.4	44.728		323.31	7.15E+09
8	8	50.2	50.2	50.2	28.151		102.48	1.5E+10
2	2	12	12	12	28.705	0.000555	3.1088	70766000
7	7	26.4	26.4	26.4	32.931	0.000677	12.66	7.71E+09
6	6	28.6	28.6	28.6	32.947		56.641	2.01E+10
4	4	13.1	13.1	13.1	32.725	0.000609	6.5065	3.21E+09
4	4	38.6	38.6	38.6	18.382		323.31	5.01E+09
4	4	40.3	40.3	40.3	16.121		119.81	4.85E+09
8	8	33.5	33.5	33.5	41.098		323.31	7.58E+10
1	1	11.3	11.3	11.3	12.382	0.001624	2.5301	72288000
1	1	12.5	12.5	12.5	11.862		23.081	4.88E+09
4	4	51.7	51.7	51.7	11.933		299.33	1.67E+10
6	6	12.9	12.9	12.9	47.027		34.525	5.1E+09
5	5	22.8	22.8	22.8	41.306		255.3	1.5E+10
6	6	49.3	49.3	49.3	22.866		95.265	2.95E+09
10	10	61.4	61.4	61.4	18.613		323.31	9.92E+10
3	3	20	20	20	17.432	0.000579	4.3233	3.87E+09
6	6	30.5	30.5	30.5	25.741		128.29	1.77E+10
29	29	51	51	51	72.001		323.31	2.45E+11
1	1	5.4	5.4	5.4	21.58	0.000623	7.8062	1.03E+09
10	10	37.8	37.8	37.8	42.471		323.31	1.01E+11
2	2	20.7	20.7	20.7	14.917		27.301	9.08E+08

7	7	49.4	49.4	49.4	23.55		323.31	1.39E+11
12	12	53.8	53.8	53.8	27.259		323.31	6.45E+10
10	10	42.9	42.9	42.9	32.756		323.31	8.09E+10
14	14	43.9	43.9	43.9	46.258		323.31	4.89E+10
1	1	5.3	5.3	5.3	27.793	0.000674	12.463	82848000
6	6	49.8	49.8	49.8	23.329		144.57	8.52E+09
3	3	3.8	3.8	3.8	136.69	0.000734	19.704	4.01E+09
2	2	5.1	5.1	5.1	56.974	0.009759	1.4811	2.39E+08
3	3	8.4	8.4	8.4	55.783		122.54	5.47E+09
1	1	6.2	6.2	6.2	28.282	0.006767	1.6393	2.42E+08
11	11	41.7	41.7	41.7	40.187		323.31	6.79E+10
1	1	3.8	3.8	3.8	37.22	0.000747	21.072	1.06E+09
2	2	7.5	7.5	7.5	35.993	0.000654	10.434	1.19E+09
9	9	20	20	20	55.366		215.69	2.35E+10
2	2	31.2	31.2	31.2	8.605	0.000586	4.8423	2.14E+08
13	13	24	24	24	64.231		323.31	1.68E+11
16	16	61.7	61.7	61.7	29.627		323.31	1.42E+11
14	14	35.9	35.9	35.9	41.287		323.31	2.1E+11
2	2	24	24	24	16.891	0.000751	21.498	7.15E+08
6	6	65.5	65.5	65.5	12.298		189.37	2.56E+10
5	5	21.3	21.3	21.3	36.064		50.903	3.93E+09
11	11	53.9	53.9	53.9	15.313		323.31	9.69E+11
2	2	32.4	32.4	32.4	12.141		32.972	8.96E+08
4	4	37	37	37	21.794		115.53	1.93E+09
3	3	8.9	8.9	8.9	40.003	0.000633	8.457	7.44E+09
9	9	18	18	18	63.392		74.379	1.37E+10
2	2	14.4	14.4	14.4	29.339		23.022	1.11E+10
5	5	7.6	7.6	7.6	71.668	0.000728	18.679	2.62E+09
7	7	32.8	32.8	32.8	34.632		101.46	1.6E+10
9	9	43.3	43.3	43.3	34.73		323.31	1.58E+10
3	3	11.8	11.8	11.8	38.26	0.00676	1.6314	5.11E+09
4	4	20.1	20.1	20.1	26.407	0.000629	8.307	8.87E+08
6	6	37.3	37.3	37.3	23.87		323.31	4.4E+10
9	9	37.3	37.3	37.3	41.218		40.061	3.62E+09
2	2	9.7	9.7	9.7	42.094	0.00064	9.142	7.98E+09
2	2	7.1	7.1	7.1	40.753	0.000608	6.3572	6.59E+08
3	3	18.4	18.4	18.4	37.434		47.591	1.13E+09
1	1	3.5	3.5	3.5	51.741	0.006739	1.608	1.09E+09
6	6	33.2	33.2	33.2	31.271		119.25	7.83E+09
4	4	27.1	27.1	27.1	14.371		323.31	5.35E+09
22	22	33.9	33.9	33.9	73.157		323.31	8.1E+10
5	5	24.7	24.7	24.7	37.373		323.31	1.19E+10
6	6	24.3	24.3	24.3	52.775		323.31	1.93E+10
1	1	9.2	9.2	9.2	16.24	0.00056	3.2887	4.2E+08
6	6	63.8	63.8	63.8	15.516		105.69	1.81E+10
3	3	23.5	23.5	23.5	20.364		32.147	5.32E+08
3	3	23.7	23.7	23.7	28.42		108.99	3.07E+09
25	25	59.1	59.1	59.1	52.642		323.31	6.4E+10
2	2	11.3	11.3	11.3	21.095	0.000708	16.211	2.64E+09
2	2	23.3	23.3	23.3	12.173		39.995	1.9E+09
2	2	10.2	10.2	10.2	13.91	0.000583	4.5541	1.88E+09

1	1	14.1	14.1	14.1	14.523	0.000751	21.642	1.81E+08
1	1	10.6	10.6	10.6	18.952	0.005255	1.8355	3.11E+08
4	4	5.7	5.7	5.7	85.659		41.466	2.95E+09
6	6	38.9	38.9	38.9	19.314		323.31	2.14E+10
3	3	23.7	23.7	23.7	11.881		151.66	1.09E+10
4	4	36.9	36.9	36.9	17.528		26.956	1.21E+10
7	7	24.2	24.2	24.2	51.281		95.353	3.88E+09
2	2	5.9	5.9	5.9	62.11	0.000588	4.953	3.59E+08
1	1	8	8	8	21.282	0.000613	6.8685	4.66E+08
4	4	16.2	16.2	16.2	40.65		323.31	4.83E+09
9	9	46.1	46.1	46.1	30.848		323.31	4.67E+11
6	6	36.9	36.9	36.9	30.735		323.31	1.28E+11
3	3	15.4	15.4	15.4	35.972	0.000619	7.6116	9.3E+08
3	3	7.8	7.8	7.8	60.188		80.795	5.72E+09
6	6	20.7	20.7	20.7	42.628		78.746	4.94E+09
5	5	12.7	12.7	12.7	68.604		30.493	2.5E+09
2	2	13.7	13.7	13.7	17.134	0.000574	4.0705	9.84E+08
4	4	26	26	26	21.938	0.000693	14.206	1.54E+10
1	1	12.4	12.4	12.4	11.152	0.001612	2.3512	1.4E+08
2	2	28.2	28.2	28.2	18.93		37.801	1.98E+09
7	7	20.3	20.3	20.3	39.106		99.753	6.18E+09
2	2	22.7	22.7	22.7	13.017		30.417	4.68E+09
7	7	54.9	54.9	54.9	19.057		323.31	5.43E+10
10	10	17	17	17	75.927		156.88	1.87E+10
5	5	24	24	24	33.693		323.31	1.57E+10
4	4	9.9	9.9	9.9	67.374		39.27	4.56E+09
4	4	18.1	18.1	18.1	38.263		74.742	7.29E+09
7	7	34.8	34.8	34.8	29.994		69.445	7.16E+09
6	6	59.7	59.7	59.7	16.175		89.982	3.71E+10
6	6	25.2	25.2	25.2	50.769		47.01	4.13E+09
1	1	6.6	6.6	6.6	23.521		237.62	9.4E+09
5	5	19.5	19.5	19.5	35.716		66.933	2.28E+09
3	3	9	9	9	28.046		70.658	5.94E+09
3	3	8.6	8.6	8.6	49.499		94.965	1.01E+10
1	1	6.6	6.6	6.6	26.343	0.005266	1.8662	1.31E+08
18	18	51.1	51.1	51.1	56.626		323.31	1.38E+11
1	1	3.2	3.2	3.2	59.158	0.001089	2.713	1.35E+08
5	5	53	53	53	14.533		158.73	7.47E+09
3	3	24.5	24.5	24.5	29.916		75.303	3.64E+09
1	1	21.7	21.7	21.7	6.5299	0.000558	3.2348	58173000
12	12	40.7	40.7	40.7	43.603		289.27	2.06E+10
1	1	11.6	11.6	11.6	24.141	0.00057	3.8947	96750000
6	6	39.9	39.9	39.9	17.078		107.52	1.47E+10
1	1	5.9	5.9	5.9	21.246		23.569	2.47E+09
7	7	37.2	37.2	37.2	34.15		323.31	1.69E+10
17	17	57.8	57.8	57.8	40.066		323.31	9.81E+10
13	13	54	54	54	25.45		260.18	3.21E+10
2	2	20	20	20	16.976		145.63	2.14E+09
5	5	23.2	23.2	23.2	31.02		108.35	5.78E+09
6	6	34.2	34.2	34.2	25.06		51.743	2.43E+10
1	1	22.8	22.8	22.8	10.307	0.000618	7.4223	14881000

5	5	27.1	27.1	27.1	37.121		75.979	1E+09
8	8	32.6	32.6	32.6	61.39		150.17	1.08E+10
3	3	16	16	16	22.631		23.007	3.86E+09
3	3	15.5	15.5	15.5	24.852		73.923	5.23E+09
6	6	18.9	18.9	18.9	42.173		201.41	1.37E+10
2	2	14.6	14.6	14.6	27.175		137.4	4.28E+09
10	10	28.9	28.9	28.9	46.338		323.31	2.93E+10
2	2	19	19	19	18.114		65.316	1.31E+09
4	4	42	42	42	14.772		64.483	2.62E+09
2	2	9.7	9.7	9.7	33.937	0.000613	6.8599	6.58E+08
3	3	10	10	10	38.454		64.909	2.44E+09
2	2	20.5	20.5	20.5	19.753		45.632	2.7E+09
10	10	51.5	51.5	51.5	27.897		173.24	7.04E+10
10	10	38.4	38.4	38.4	29.42		140.23	1.04E+11
7	7	13	13	13	99.775		49.198	5.93E+09
6	6	25.2	25.2	25.2	26.179		110.69	1.84E+10
5	5	17.8	17.8	17.8	32.865		178.57	2.25E+10
1	1	5.2	5.2	5.2	38.224	0.0007	15.416	9.71E+08
2	2	5.7	5.7	5.7	61.935		31.351	1.78E+09
4	4	17.8	17.8	17.8	25.46	0.000595	5.4826	3.27E+09
6	6	8.7	8.7	8.7	120.02	0.000615	7.2327	1.6E+09
6	6	26.1	26.1	26.1	39.748		145.42	3.19E+09
7	7	34	34	34	36.032		323.31	1.43E+10
1	1	15.8	15.8	15.8	10.83	0.009669	1.4098	10472000
4	4	24.7	24.7	24.7	27.272		75.164	3.28E+09
3	3	9	9	9	43.828	0.000739	20.373	1.69E+08
5	5	10.4	10.4	10.4	66.805	0.000651	10.129	2.28E+09
5	5	14.1	14.1	14.1	51.132		34.12	6.69E+10
1	1	4.5	4.5	4.5	22.287	0.003202	2.168	1.59E+10
4	4	25.1	25.1	25.1	21.759	0.00075	21.395	1.58E+09
2	2	7.3	7.3	7.3	37.528	0.00063	8.3214	6.74E+09
1	1	5	5	5	31.266	0.000694	14.418	3.23E+09
4	4	25	25	25	30.903		60.919	1.13E+09
1	1	7.9	7.9	7.9	16.494	0.004244	2.0277	1.57E+09
7	7	80	80	80	12.197		323.31	8.94E+10
1	1	16.8	16.8	16.8	10.023		323.31	9.25E+10
10	10	86.7	86.7	86.7	11.72		323.31	1E+11
2	2	17.5	17.5	17.5	15.364	0.000583	4.6465	2.44E+09
2	2	8.7	8.7	8.7	27.124	0.000627	8.1334	3.88E+08
5	5	22.8	22.8	22.8	39.014		70.083	2.46E+09
13	13	27.9	27.9	27.9	90.998		323.31	6.68E+09
8	8	8.2	8.2	8.2	175.38	0.000637	8.7991	1.39E+09
2	2	5.2	5.2	5.2	73.88	0.000575	4.1193	4.25E+08
1	1	12.5	12.5	12.5	19.481		181.58	2.87E+09
3	3	22.1	22.1	22.1	21.094		99.704	1.7E+09
3	3	28.1	28.1	28.1	17.217		111.45	8.14E+09
1	1	10.3	10.3	10.3	12.73	0.009259	1.4949	1.69E+08
2	2	9.6	9.6	9.6	22.143	0.001101	2.9274	2.81E+09
1	1	9.2	9.2	9.2	16.831		209.05	3.79E+09
2	2	12.8	12.8	12.8	20.042	0.000568	3.7684	2.15E+09
2	2	8.9	8.9	8.9	40.633	0.001087	2.6498	3E+09

2	2	10.1	10.1	10.1	25.92	0.000625	7.956	2.27E+09
1	1	7	7	7	17.807	0.006266	1.7389	1.37E+08
1	1	23.8	23.8	23.8	9.6045	0.000578	4.2502	5.24E+08
4	4	25.4	25.4	25.4	27.397	0.000655	10.472	7.58E+08
2	2	11.8	11.8	11.8	39.037		26.132	1.55E+10
3	3	16.5	16.5	16.5	29.984		37.584	1.71E+09
1	1	3	3	3	45.67	0.001099	2.8929	3.49E+09
3	3	11	11	11	34.906		126.03	1.16E+10
2	2	11.9	11.9	11.9	19.099	0.000662	11.167	6.05E+09
2	2	5.8	5.8	5.8	46.772	0.001094	2.8028	3.48E+08
5	5	26.9	26.9	26.9	32.339		96.053	4.98E+09
3	3	50	50	50	10.519	0.000601	6.0061	2.52E+09
2	2	9.1	9.1	9.1	26.897	0.000594	5.4591	6.74E+08
6	6	15.7	15.7	15.7	49.975		131.44	6.92E+09
3	3	20.9	20.9	20.9	20.874		136.08	2.79E+10
1	1	11.8	11.8	11.8	19.841		87.994	4.32E+09
1	1	16.9	16.9	16.9	8.5399	0.000596	5.5374	6.99E+08
3	3	16.6	16.6	16.6	33.796		32.734	4.64E+09
10	10	46.9	46.9	46.9	38.93		323.31	1.88E+10
5	5	13.3	13.3	13.3	54.22		45.731	1.79E+09
3	3	17.1	17.1	17.1	31.467		63.165	1.76E+09
5	5	22.7	22.7	22.7	41.693		290.31	9.37E+09
6	6	24.4	24.4	24.4	34.274		24.043	5.67E+09
2	2	2.2	2.2	2.2	105.89	0.009264	1.4966	7.95E+09
5	5	30.7	30.7	30.7	27.626		44.198	6.09E+09
36	36	49.3	49.3	49.3	108.42		323.31	1.14E+11
3	3	18	18	18	34.608	0.000639	9.0112	4.91E+08
1	1	5	5	5	25.979	0.00477	1.9934	1.41E+08
4	4	16.7	16.7	16.7	23.563	0.000654	10.461	1.44E+10
10	10	23.9	23.9	23.9	57.561		216.94	2.97E+10
5	5	18.9	18.9	18.9	37.306		323.31	1.21E+10
4	4	28.6	28.6	28.6	21.959		267.23	2.4E+10
1	1	6.2	6.2	6.2	25.175	0.005288	1.9605	1.95E+09
1	1	6.9	6.9	6.9	21.019	0.0011	2.9067	1.65E+08
4	4	10.4	10.4	10.4	58.585	0.000697	14.957	7.05E+08
1	1	7.1	7.1	7.1	21.447	0.001614	2.3599	81510000
5	5	35.6	35.6	35.6	23.702		127.57	7.41E+09
5	5	23	23	23	25.186		133.86	8.56E+09
1	1	11.4	11.4	11.4	18.138	0.000566	3.644	35252000
1	1	12.5	12.5	12.5	25.744		71.168	9.65E+08
11	11	22.9	22.9	22.9	78.758		139.09	1.18E+10
12	12	48.2	48.2	48.2	40.339		289.77	1.1E+11
2	2	10.1	10.1	10.1	33.388	0.000621	7.6908	1.66E+08
5	4	12.5	12.5	10.8	66.581	0.000648	9.719	4.85E+09
4	4	16.5	16.5	16.5	26.476		211.86	1.07E+10
6	6	42.4	42.4	42.4	18.092		182.05	1.78E+10
2	2	12.5	12.5	12.5	28.332		323.31	5.47E+09
3	3	11.6	11.6	11.6	44.325		62.513	3.32E+09
3	3	9.2	9.2	9.2	63.555	0.000632	8.3884	8.14E+08
2	2	19.2	19.2	19.2	27.766		72.436	3.05E+09
9	9	29.5	29.5	29.5	38.729		176.45	6.31E+10

2	2	2.9	2.9	2.9	106.04	0.000597	5.5985	2.33E+09
5	5	21.3	21.3	21.3	43.1		76.464	1.43E+09
4	4	10.6	10.6	10.6	41.137		50.768	3.12E+09
6	6	23.1	23.1	23.1	36.536		115.71	1.03E+10
5	5	34.4	34.4	34.4	19.416		40.021	7.16E+09
6	6	16.2	16.2	16.2	61.321		69.268	7.31E+09
2	2	11.7	11.7	11.7	18.542	0.000573	4.0635	1.01E+09
9	9	44.8	44.8	44.8	28.054		142.18	2.27E+10
3	3	11.5	11.5	11.5	50.748		38.901	4.32E+09
14	14	42.7	42.7	42.7	41.778		256.33	1.71E+11
9	9	40.7	40.7	40.7	40.173		323.31	2.7E+10
7	7	20.7	20.7	20.7	58.398		29.061	5.18E+09
5	5	51.4	51.4	51.4	15.808		24.762	5.98E+09
4	4	21.3	21.3	21.3	26.169		28.13	4.27E+09
4	4	17.2	17.2	17.2	50.497	0.000666	11.445	9.17E+08
4	4	16.9	16.9	16.9	45.705		98.145	3.01E+10
3	3	20	20	20	28.561		38.377	2.98E+09
3	3	14.7	14.7	14.7	49.564		79.58	4.31E+09
9	9	38.5	38.5	38.5	31.867		323.31	8.14E+10
2	2	6.7	6.7	6.7	53.33	0.000622	7.6979	1.19E+09
1	1	6.2	6.2	6.2	24.16	0.000573	4.0668	2.29E+09
2	2	14.7	14.7	14.7	24.986	0.000602	6.0235	56110000
11	11	33.5	33.5	33.5	50.769		62.788	7.49E+09
10	10	16.3	16.3	16.3	119.56		323.31	1.49E+10
6	6	27.7	27.7	27.7	44.135		323.31	5.76E+09
4	4	8.1	8.1	8.1	90.918		85.227	1.35E+09
1	1	5.4	5.4	5.4	76.736	0.000563	3.4613	2.35E+08
2	2	21.4	21.4	21.4	10.926	0.000565	3.5765	1.11E+08
1	1	10.3	10.3	10.3	19.497	0.000653	10.32	53488000
1	1	22.5	22.5	22.5	13.794		29.586	1.45E+09
2	2	7.4	7.4	7.4	48.293		33.224	1.87E+09
4	4	14.3	14.3	14.3	37.335	0.001609	2.3282	2.31E+09
21	21	46.5	46.5	46.5	59.527		323.31	1.24E+11
1	1	5.5	5.5	5.5	27.688	0.000593	5.4093	1.36E+09
6	6	19.6	19.6	19.6	42.44	0.000706	16.057	6.84E+09
3	3	15.9	15.9	15.9	42.727		110.94	2.15E+09
9	9	36.1	36.1	36.1	31.194		66.52	1.53E+10
2	2	3.1	3.1	3.1	59.186	0.006729	1.6002	1.5E+09
2	2	12.2	12.2	12.2	24.669	0.00267	2.1996	8.48E+08
13	13	33.4	33.4	33.4	52.659		71.005	1.68E+10
1	1	7.3	7.3	7.3	28.033		323.31	2.25E+09
2	2	7.3	7.3	7.3	42.062		274.14	1.76E+09
4	4	48.6	48.6	48.6	18.476		323.31	4.16E+10
2	2	9.6	9.6	9.6	35.229		37.713	3.61E+08
7	7	31.7	31.7	31.7	35.25		41.862	2.31E+09
7	7	16	16	16	95.351		144.16	3.5E+09
3	3	14.7	14.7	14.7	33.79		176.9	1.45E+10
2	2	11.1	11.1	11.1	40.44		54.731	2.22E+09
1	1	6.6	6.6	6.6	24.347		236.76	7.04E+09
2	2	7.8	7.8	7.8	28.142		37.184	6.06E+09
1	1	4	4	4	24.822	0.005238	1.7739	1.26E+08

11	11	23.6	23.6	23.6	61.296		323.31	1.64E+10
3	3	6.5	6.5	6.5	89.981	0.000754	21.855	1.47E+09
6	6	7.4	7.4	7.4	108.74	0.000659	10.748	6.35E+09
3	3	12.6	12.6	12.6	32.75	0.000722	17.86	1.39E+09
6	6	15.4	15.4	15.4	69.461		81.456	1.19E+10
8	8	39.4	39.4	39.4	25.434		315.62	7.89E+10
12	12	19.8	19.8	19.8	79.759		269.85	3.99E+10
1	1	7.1	7.1	7.1	15.278	0.000749	21.353	1.57E+09
3	3	12.6	12.6	12.6	23.949		323.31	2.61E+10
1	1	12	12	12	20.715	0.000704	15.9	1.9E+08
1	1	16.7	16.7	16.7	15.006		323.31	1.33E+09
8	8	23.2	23.2	23.2	49.921		87.996	9.24E+09
4	4	17.4	17.4	17.4	30.394		102.9	3.67E+09
2	2	16	16	16	25.977	0.000744	20.894	1.19E+08
3	3	43.7	43.7	43.7	11.65	0.001088	2.6534	7.84E+09
3	3	12.9	12.9	12.9	42.371	0.000598	5.7097	2.61E+09
6	6	21.1	21.1	21.1	49.601		164.97	3.09E+10
1	1	7.5	3.9	3.9	39.675		28.53	2.16E+09
3	3	23.7	23.7	23.7	14.501	0.000622	7.7373	1.21E+09
3	3	7.2	7.2	7.2	49.305	0.000557	3.2018	1.69E+09
3	3	16.7	16.7	16.7	18.265		29.554	8.18E+09
2	2	12.5	12.5	12.5	36.267	0.000637	8.8475	1.66E+09
7	7	34.6	34.6	34.6	24		179.09	9.96E+09
1	1	8.8	8.8	8.8	26.357	0.000737	20.127	7.34E+08
8	8	30.9	30.9	30.9	50.488		158.37	3.02E+09
1	1	12	12	12	25.124		323.31	9.26E+09
1	1	10.8	10.8	10.8	15.31	0.000593	5.4103	1.8E+09
7	7	15.8	15.8	15.8	89.183		32.237	1.65E+09
1	1	18.8	18.8	18.8	9.3853	0.000571	3.9671	6.26E+08
3	3	11.9	11.9	11.9	53.754		45.296	3.64E+08
1	1	26.3	26.3	26.3	8.7647		222.53	5.92E+08
1	1	3.1	3.1	3.1	35.438	0.009714	1.4535	1.27E+09
4	4	27.3	27.3	27.3	23.788	0.000727	18.632	3.21E+09
3	3	26.6	26.6	26.6	15.27	0.008256	1.527	1.44E+08
5	5	33.8	33.8	33.8	22.63		37.091	4.28E+09
11	11	19.3	19.3	19.3	97.926		323.31	1.75E+10
1	1	15.8	15.8	15.8	12.338	0.009684	1.4166	2.38E+08
3	3	12.1	12.1	12.1	33.01	0.000585	4.7405	2.18E+09
1	1	14	14	14	14.905	0.000641	9.1585	88821000
4	4	9.3	9.3	9.3	62.258		155.18	8.53E+09
13	13	28.6	28.6	28.6	76.275		314.66	1.79E+10
1	1	5.7	5.7	5.7	27.733	0.000703	15.85	1.05E+09
3	3	6.8	6.8	6.8	72.655		63.208	1.66E+09
4	4	13.1	13.1	13.1	48.662	0.000699	15.409	1.77E+09
10	10	27.4	27.4	27.4	58.63		323.31	3.1E+10
2	2	23.3	23.3	23.3	14.651		103.41	1.08E+10
5	5	22.9	22.9	22.9	30.662		35.317	3.84E+10
26	26	46.1	46.1	46.1	90.206		323.31	1.37E+11
7	7	25.1	25.1	25.1	32.851		323.31	1.04E+10
2	2	10.7	10.7	10.7	31.877	0.00058	4.3848	4.81E+08
2	2	8.2	8.2	8.2	27.053		28.782	6.89E+09

3	3	18	18	18	28.535		53.058	7.43E+08
7	7	19.9	19.9	19.9	42.481	0.00066	10.799	1.91E+09
5	5	16.8	16.8	16.8	36.567		93.173	7.37E+09
4	4	14.1	14.1	14.1	37.321		158.41	7.22E+09
33	33	58.8	58.8	58.8	64.842		323.31	3.72E+11
3	3	16.7	16.7	16.7	29.658		111.04	6.96E+09
4	4	30.8	30.8	30.8	15.616	0.000716	16.912	1.15E+10
6	6	31	31	31	32.6		23.683	4.41E+09
1	1	7.9	7.9	7.9	20.326	0.000606	6.2112	5.95E+09
1	1	5.3	5.3	5.3	30.864	0.000567	3.7394	4.76E+08
18	18	35.5	35.5	35.5	84.741		323.31	8.53E+10
5	5	40.8	40.8	40.8	16.951		323.31	5.05E+10
9	9	22.8	22.8	22.8	60.533		252.73	1.55E+10
3	3	6.5	6.5	6.5	71.986	0.000601	6.0123	1.42E+09
3	3	23.4	23.4	23.4	20.124	0.000701	15.556	6.5E+09
4	4	26.1	26.1	26.1	21.577	0.000584	4.6893	4.07E+08
3	3	10.3	10.3	10.3	36.523	0.000686	13.584	2.36E+09
2	2	10.7	10.7	10.7	20.718	0.000564	3.5289	1.05E+09
3	3	14.4	14.4	14.4	43.331		132.9	3.4E+09
5	5	18.1	18.1	18.1	42.607		107.41	8.39E+09
2	2	3.2	3.2	3.2	65.544	0.000551	2.9635	9.52E+08
1	1	2.1	2.1	2.1	57.225	0.001623	2.4879	2.21E+09
11	11	27.4	27.4	27.4	51.161		68.238	2.65E+10
1	1	8.2	8.2	8.2	23.706	0.000635	8.7193	1.8E+08
14	14	31.2	31.2	31.2	71.139		163.21	2.69E+10
8	8	18.5	18.5	18.5	56.812		73.427	1.09E+10
7	7	34.4	34.4	34.4	35.502		153.56	5.27E+09
1	1	7.5	7.5	7.5	19.027	0.000674	12.421	1.33E+09
3	3	10.7	10.7	10.7	37.84		99.568	2.79E+08
3	3	22.4	22.4	22.4	22.624	0.000705	16.006	1.55E+08
1	1	15	15	15	16.039		28.759	1.27E+09
9	9	18.8	18.8	18.8	69.42		323.31	1.52E+10
2	2	11.7	11.7	11.7	23.545	0.000608	6.3592	2.32E+08
4	4	62.2	62.2	62.2	10.685		29.036	2.97E+09
3	3	14.2	14.2	14.2	31.576		132.89	6.3E+09
8	8	22.3	22.3	22.3	51.882		323.31	2.23E+10
4	4	25	25	25	27.426	0.000724	18.112	1.41E+09
9	9	42.7	42.7	42.7	36.176		116.35	3.94E+09
20	20	23.3	23.3	23.3	106		308.48	9.04E+10
4	4	23.6	23.6	23.6	23.28		144.48	1.13E+10
5	5	37	37	37	25.438		323.31	3.39E+09
2	2	14.1	14.1	14.1	30.66	0.000725	18.333	1.16E+09
8	8	29.1	29.1	29.1	39.044		137.55	7.93E+09
4	4	17	17	17	31.576		185.51	1.41E+10
2	2	20.2	20.2	20.2	20.536	0.000582	4.492	1.83E+09
1	1	4.7	4.7	4.7	31.14	0.0011	2.9022	2.25E+08
4	4	17.4	17.4	17.4	33.749		55.596	3.2E+09
4	4	19.7	19.7	19.7	29.124	0.000577	4.2162	1.96E+09
5	5	7.8	7.8	7.8	80.613		112.32	1.55E+10
6	6	22.7	22.7	22.7	37.382		28.367	1.68E+09
22	22	28.7	28.7	28.7	104.21		323.31	1.65E+11

6	6	14.6	14.6	14.6	63.66		323.31	7.57E+09
3	3	4.6	4.6	4.6	69.555		323.31	8.23E+09
1	1	4.8	4.8	4.8	38.353	0.000633	8.4382	1.67E+09
2	2	7.6	7.6	7.6	26.604	0.002671	2.2014	7.38E+08
3	3	17.6	17.6	17.6	20.706		166.97	5.95E+09
1	1	10.5	10.5	10.5	16.329	0.000719	17.555	5.1E+08
1	1	4.6	4.6	4.6	29.619	0.002143	2.2558	1.79E+08
1	1	14.2	14.2	14.2	14.463	0.001097	2.8632	2.91E+08
5	5	37.8	37.8	37.8	21.322		206.33	8.58E+10
6	6	19.5	19.5	19.5	43.973		38.198	6.83E+09
11	11	24.6	24.6	24.6	59.682		181.38	1.97E+10
1	1	17.7	17.7	17.7	17.569		94.443	2.05E+08
17	17	50.4	50.4	50.4	40.325		323.31	2.81E+11
4	4	13.4	13.4	13.4	52.396	0.000701	15.654	3.07E+09
2	2	28.1	28.1	28.1	15.108		237.17	1.99E+10
3	3	18.4	18.4	18.4	16.317		184.68	2.86E+10
3	3	12.4	12.4	12.4	38.73	0.000678	12.764	8.2E+08
3	3	14.7	14.7	14.7	29.103	0.000668	11.66	6.2E+08
4	4	17.7	17.7	17.7	33.426		323.31	5.4E+09
3	3	14.7	14.7	14.7	29.216	0.000694	14.494	1.53E+09
1	1	11.5	11.5	11.5	14.411	0.005252	1.8348	1.4E+08
2	2	33.3	33.3	33.3	12.733	0.000644	9.3386	2.61E+08
4	4	16.4	16.4	16.4	38.537	0.000673	12.228	1.8E+09
2	2	21.7	21.7	21.7	26.984		28.179	1.37E+09
3	3	17.1	17.1	17.1	18.681	0.00062	7.6473	3.18E+09
4	4	31.5	31.5	31.5	22.22		64.221	1.76E+10
6	6	14.6	14.6	14.6	66.801	0.000676	12.66	7.95E+09
1	1	5.8	5.8	5.8	24.658	0.00058	4.392	75991000
4	4	5.2	5.2	5.2	86		58.764	1.81E+09
4	4	18.9	18.9	18.9	43.263		203.54	7.92E+08
3	3	28.6	28.6	28.6	19.272		65.573	1.35E+10
3	3	15.8	15.8	15.8	24.242	0.000669	11.792	3.13E+09
27	27	24.4	24.4	24.4	176.92		323.31	3.2E+10
8	8	22.3	22.3	22.3	53.293		282.7	2.42E+10
2	2	12.7	12.7	12.7	25.89		27.166	3.56E+08
5	5	15.6	15.6	15.6	50.233	0.00074	20.412	2.16E+09
1	1	4.3	4.3	4.3	26.164		23.266	1E+08
2	2	12.1	12.1	12.1	30.542	0.000698	15.311	4.28E+09
3	3	53.6	53.6	53.6	9.1661		323.31	5.25E+09
8	8	19.2	19.2	19.2	72.625		69.248	4.92E+09
1	1	6.4	6.4	6.4	18.367		102.58	4.44E+09
3	3	16.9	16.9	16.9	34.889	0.000705	15.978	5.97E+09
8	8	18.9	18.9	18.9	69.279		161.12	2.85E+10
3	3	8.8	8.8	8.8	37.232	0.000557	3.2157	8.1E+08
5	5	17.8	17.8	17.8	43.482	0.000658	10.696	9.54E+08
2	2	11.7	11.7	11.7	23.07		41.052	1.25E+10
2	2	8.1	8.1	8.1	37.226		43.904	2.83E+09
12	12	58.1	58.1	58.1	30.466		323.31	1.17E+11
3	3	11.7	11.7	11.7	42.519		269.94	4.13E+08
2	2	16.8	16.8	16.8	18.738		27.728	2.35E+08
3	3	21.4	21.4	21.4	28.658	0.000644	9.3092	4.89E+09

5	5	10.3	10.3	10.3	57.452	0.000578	4.25	1.03E+10
4	4	23.6	23.6	23.6	14.123		55.879	3.97E+09
6	6	32.7	32.7	32.7	30.502		149.31	1.67E+10
7	7	20.7	20.7	20.7	57.042		297.62	2.58E+10
4	4	13.9	13.9	13.9	42.981		84.555	2.97E+09
4	4	21.1	21.1	21.1	29.031		323.31	6.21E+09
10	10	34.7	34.7	34.7	47.653		175.74	3.11E+10
5	5	32.1	32.1	32.1	19.508		246.28	3.08E+10
11	11	30.6	30.6	30.6	48.964		276.72	7.56E+10
3	3	25.6	25.6	25.6	13.829		211.44	2.44E+10
2	2	9.5	9.5	9.5	31.087	0.000631	8.3388	3.11E+09
6	6	40.1	40.1	40.1	26.849		323.31	2.67E+10
3	3	9.7	9.7	9.7	35.555		118.38	1.31E+09
3	3	15.3	15.3	15.3	31.815		70.76	2.54E+09
6	6	13.6	13.6	13.6	79.149		61.388	2.76E+09
6	6	43.6	43.6	43.6	17.83		233.81	2.98E+10
2	2	11.6	11.6	11.6	25.53	0.000684	13.428	2.67E+09
5	5	9.3	9.3	9.3	62.604		203.58	6.25E+09
7	7	16.6	16.6	16.6	66.085		166.16	9.02E+09
4	4	27.6	27.6	27.6	19.371		148.07	5.1E+10
2	2	5.3	5.3	5.3	56.527	0.000754	21.759	5.32E+09
4	4	17.4	17.4	17.4	28.024		63.079	1.44E+09
15	15	27.1	27.1	27.1	44.514		323.31	1.34E+11
5	5	26.2	26.2	26.2	26.245		32.063	1.62E+10
2	2	12.5	12.5	12.5	29.343		59.322	5.13E+09
7	7	25.4	25.4	25.4	41.504		200.03	1.26E+10
26	26	38.2	38.2	38.2	148.51		323.31	2.33E+10
2	2	19.3	19.3	19.3	21.843	0.000566	3.6762	1.31E+08
2	2	20.7	20.7	20.7	18.951		323.31	2.63E+10
23	23	44.8	44.8	44.8	73.453		323.31	7.93E+10
7	7	42.1	42.1	42.1	32.213		323.31	1.06E+10
9	9	31.6	31.6	31.6	43.377		323.31	3.34E+10
1	1	5.1	5.1	5.1	31.142	0.000663	11.311	1.21E+08
2	2	4.3	4.3	4.3	34.429	0.000597	5.7032	1.4E+09
3	3	5.1	5.1	5.1	64.907	0.000572	4.0195	4.13E+09
1	1	3.2	3.2	3.2	35.161	0.009729	1.4583	4.17E+08
3	3	42.3	42.3	42.3	11.895		23.842	7.17E+08
2	2	7.9	7.9	7.9	18.803	0.000562	3.3744	4.17E+09
4	4	30.3	30.3	30.3	27.021		69.555	1.26E+09
4	4	17.9	17.9	17.9	19.209		27.555	2.65E+09
4	4	34.4	34.4	34.4	20.66		77.781	8.47E+09
9	9	25.6	25.6	25.6	40.9		323.31	1.99E+10
8	8	53.1	53.1	53.1	21.012		298.9	2.59E+10
15	15	45.7	45.7	45.7	55.589		323.31	8.93E+10
8	8	31.1	31.1	31.1	37.04		75.758	8.88E+09
3	3	5.9	5.9	5.9	59.761	0.000628	8.2065	8.57E+09
4	4	23	23	23	28.411	0.000723	17.948	2.04E+09
4	4	16.3	16.3	16.3	44.275	0.000635	8.6912	5.16E+09
7	7	20	20	20	39.174	0.000712	16.709	1.24E+10
3	3	24.9	24.9	24.9	18.113		27.543	1.19E+10
1	1	14.8	14.8	14.8	9.9663	0.009679	1.4164	44353000

11	11	23	23	23	50.599		92.418	2.99E+10
2	2	10.9	10.9	10.9	30.452	0.000756	22.15	9.73E+08
1	1	7.8	7.8	7.8	22.037	0.000605	6.1896	4.47E+08
2	2	21.3	21.3	21.3	15.534	0.006757	1.6257	1.82E+09
7	7	36.8	36.8	36.8	31.145		220.11	1.55E+10
19	19	78.8	78.8	78.8	30.814		323.31	1.59E+11
8	8	20.6	20.6	20.6	48.482		35.005	2.12E+10
5	5	24.3	24.3	24.3	37.095	0.000743	20.814	1.44E+09
6	6	33.8	33.8	33.8	24.564		240.87	2.37E+10
5	5	30.6	30.6	30.6	37.705		54.821	1.33E+09
10	10	28.2	28.2	28.2	42.481		173.45	4.2E+10
7	7	21.9	21.9	21.9	44.657		114.32	3.38E+09
5	5	21	21	21	46.133		223.26	8.33E+09
10	10	38.5	38.5	38.5	41.904		117.19	5.93E+10
3	3	9.6	9.6	9.6	51.784	0.000588	5.0587	1.58E+09
4	4	12.6	12.6	12.6	51.13		209.63	6.48E+09
3	3	15.1	15.1	15.1	31.674		32.302	1.26E+09
5	5	25.7	25.7	25.7	27.746		135.29	3.94E+09
12	7	25.6	25.6	15.6	80.859		323.31	5.99E+09
6	6	22.8	12.7	12.7	81.011		279.95	2.61E+09
10	10	21.3	21.3	21.3	79.581		205.57	4.3E+09
2	2	16.7	16.7	16.7	20.373	0.000657	10.639	3.94E+09
2	2	7.5	7.5	7.5	64.301	0.009739	1.4628	4.24E+09
1	1	5.8	5.8	5.8	41.102		77.953	1.34E+09
4	4	7.1	7.1	7.1	95.438		323.31	1.8E+09
1	1	11.6	11.6	11.6	29.89		61.197	1.08E+09
5	5	16.3	16.3	16.3	41.859		26.503	1.04E+10
8	8	23.4	23.4	23.4	65.161		116.2	1.03E+10
6	6	26.4	26.4	26.4	31.764		323.31	4.77E+10
3	3	13.5	13.5	13.5	55.298		25.344	1E+09
11	11	28.5	28.5	28.5	52.442		310.48	3.94E+10
2	2	7.9	7.9	7.9	45.069	0.000595	5.4654	2.01E+08
2	2	7.5	7.5	7.5	46.56	0.009749	1.4728	4.19E+08
20	20	43.3	43.3	43.3	69.82		323.31	3.54E+10
3	3	6.9	6.9	6.9	114.79	0.000582	4.5299	1.1E+08
1	1	2.1	2.1	2.1	63.408	0.009689	1.4206	1.68E+08
5	5	17.6	17.6	17.6	53.019		323.31	1.83E+10
15	15	57.7	57.7	57.7	32.062		323.31	2.35E+11
5	5	14.8	14.8	14.8	36.174		323.31	6.97E+09
3	3	25.4	25.4	25.4	14.8		69.177	2.37E+10
2	2	19.1	19.1	19.1	14.98	0.00067	12.01	2.01E+09
4	4	32.2	32.2	32.2	32.533		323.31	2.22E+09
2	2	13.7	13.7	13.7	18.272	0.00065	9.9213	6.17E+09
18	18	30.4	30.4	30.4	71.233		323.31	5.63E+10
2	2	7.9	7.9	7.9	46.411	0.000572	4.016	1.67E+08
2	2	27.4	27.4	27.4	11.442		323.31	2.36E+10
1	1	4	4	4	37.126	0.000576	4.1962	1.27E+09
15	15	36.9	36.9	36.9	58.548		323.31	6.59E+10
7	7	18.3	18.3	18.3	63.269		192.11	1.24E+10
4	4	28.1	28.1	28.1	28.294		169.51	1.5E+10
17	17	39.5	39.5	39.5	58.603		323.31	1.3E+11

7	7	21.1	21.1	21.1	51.292		323.31	5.87E+09
4	4	7.9	7.9	7.9	48.171		60.123	5.85E+09
8	8	14	14	14	71.963		40.138	1.83E+10
2	2	3.7	3.7	3.7	56.498	0.000678	12.808	5.08E+08
6	6	26.7	26.7	26.7	44.767		75.582	1.48E+09
2	2	6.6	6.6	6.6	63.013		41.618	3.12E+09
3	3	18.9	18.9	18.9	25.179	0.000668	11.723	1.05E+09
25	25	41.6	41.6	41.6	84.048		323.31	2.54E+11
3	3	14.2	14.2	14.2	30.333	0.000641	9.1599	1.42E+09
5	5	31.1	31.1	31.1	19.535		27.326	7.1E+09
7	7	20.4	20.4	20.4	46.934		223.14	5.22E+09
14	14	27.1	27.1	27.1	84.042		323.31	3.34E+10
4	4	11	11	11	35.058	0.000611	6.7204	1.14E+09
5	5	10	10	10	69.705	0.000585	4.7061	3.31E+09
1	1	4.8	4.8	4.8	36.768	0.000643	9.2739	57983000
3	3	6.3	6.3	6.3	74.588		88.831	1.85E+10
1	1	1.7	1.7	1.7	57.994	0.005274	1.9312	2.38E+09
1	1	7.4	7.4	7.4	16.261	0.005263	1.8506	1.05E+09
6	6	20.4	20.4	20.4	52.793		164.35	1.33E+10
4	4	10.1	10.1	10.1	60.536	0.00073	19.035	1.32E+09
15	15	29.4	29.4	29.4	77.371		319.61	3.29E+10
5	5	23.4	23.4	23.4	36.165		56.225	3.58E+09
3	3	15.9	15.9	15.9	23.1		148.92	5.31E+09
8	8	42.2	42.2	42.2	20.038		53.386	3.47E+09
6	6	18.3	18.3	18.3	36.902	0.000715	16.836	3.99E+09
14	14	25.7	25.7	25.7	79.507		219.96	2.9E+10
7	7	37.2	37.2	37.2	22.647		323.31	3.84E+10
3	3	26.8	26.8	26.8	29.102		42.268	3E+09
1	1	10.3	10.3	10.3	12.597	0.000589	5.0981	7.11E+08
1	1	14.1	14.1	14.1	14.352	0.009694	1.4224	62959000
4	4	32.1	32.1	32.1	28.806		134.85	9.15E+08
4	4	22.5	22.5	22.5	38.937	0.000689	13.736	1.68E+08
1	1	9.4	9.4	9.4	39.035	0.000561	3.3085	2.89E+08
12	12	33.5	33.5	33.5	56.109		180.54	1.42E+10
4	4	24.3	24.3	24.3	31.653		43.682	1.07E+10
8	8	22.3	22.3	22.3	56.898		97.883	2.3E+10
2	2	10.8	10.8	10.8	29.027		67.692	1.06E+09
1	1	4.8	4.8	4.8	35.46	0.000657	10.683	1.76E+09
7	7	16.8	16.8	16.8	58.742		24.258	1.24E+10
11	11	24.2	24.2	24.2	69.389		323.31	8.05E+10
12	12	29.8	29.8	29.8	48.85		110.95	2.01E+10
8	8	25.1	25.1	25.1	48.294		310.71	1.44E+10
3	3	12.8	12.8	12.8	27.024	0.000638	8.8673	6.06E+09
1	1	5.9	5.9	5.9	16.561	0.000614	7.073	1.19E+09
1	1	17.7	17.7	17.7	17.148	0.000683	13.262	2.54E+08
6	6	24.1	24.1	24.1	46.399		79.171	6.71E+09
3	3	19.2	19.2	19.2	42.529	0.00065	9.9198	2.28E+09
3	3	15.5	15.5	15.5	31.521		42.557	4.85E+09
10	10	24.3	24.3	24.3	55.606		62.184	4.2E+09
12	12	18.9	18.9	18.9	100.31		57.56	1.41E+10
3	3	7.4	7.4	7.4	54.583	0.000601	5.9603	5.47E+09

2	2	6	6	6	48.014		55.164	4.18E+09
6	6	27.2	27.2	27.2	29.885	0.000726	18.58	5.44E+09
2	2	10.9	10.9	10.9	28.521		105.67	2.34E+09
11	11	20.8	20.8	20.8	86.382		323.31	6.54E+10
1	1	5.6	5.6	5.6	26.905	0.002138	2.2074	
19	19	16.7	16.7	16.7	179.05		100.18	1.11E+10
4	4	41	41	41	8.946		256.45	1.08E+10
3	3	19	19	19	15.537	0.000691	14.021	8.29E+09
5	5	21.3	21.3	21.3	42.06		168.24	2.55E+10
1	1	17.6	17.6	17.6	11.966		323.31	1.25E+09
4	4	19.5	19.5	19.5	36.919		124.77	2.35E+09
4	4	7.9	7.9	7.9	47.879		25.076	8.88E+09
4	4	22	22	22	32.523		65.126	1.78E+09
17	17	43.2	43.2	43.2	40.182		323.31	3.45E+11
6	6	14.6	14.6	14.6	51.657		73.524	1.27E+10
9	9	36.3	36.3	36.3	28.694		323.31	1.93E+10
9	9	41.8	41.8	41.8	37.993		323.31	1.92E+10
4	4	30.6	30.6	30.6	16.744		73.032	1.94E+10
5	5	27.2	27.2	27.2	26.811		310.8	1.49E+10
1	1	5.4	5.4	5.4	16.653	0.000663	11.201	1.78E+09
4	4	23	23	23	32.82	0.000687	13.6	2.62E+09
4	4	12.7	12.7	12.7	39.204		111.7	2.16E+10
6	6	10.8	10.8	10.8	100.77		167.73	3.8E+09
4	4	26.3	26.3	26.3	21.846	0.000658	10.695	4.4E+09
5	5	21	21	21	36.93		69.322	6.68E+09
3	3	9.9	9.9	9.9	52.286	0.000619	7.6165	4.15E+08
6	6	12.9	12.9	12.9	61.743		26.302	1.09E+09
1	1	7.8	7.8	7.8	19.636		137.35	6.42E+08
8	8	27.4	27.4	27.4	44.901		52.58	1.4E+10
5	5	24.3	24.3	24.3	36.294		323.31	1.35E+10
1	1	6.3	6.3	6.3	27.031		59.341	1.51E+08
2	2	9.8	9.8	9.8	26.909	0.009274	1.5118	1.5E+08
3	3	27.5	27.5	27.5	11.961		245.56	1.24E+10
1	1	9	9	9	18.508	0.000602	6.0325	1.04E+08
1	1	5.1	5.1	5.1	19.629	0.006736	1.6063	4.58E+08
1	1	25.7	25.7	25.7	7.8809	0.000592	5.2648	19253000
1	1	14.1	14.1	14.1	9.8068	0.000709	16.292	1.2E+09
1	1	10.1	10.1	10.1	15.695	0.000703	15.861	6.77E+08
1	1	8.6	8.6	8.6	12.846		51.486	40647000
2	2	16.9	16.9	16.9	15.735	0.000642	9.2538	1.17E+09
1	1	15	15	15	28.384	0.005249	1.8217	45820000
4	4	9	9	9	78.303		56.323	3.54E+09
1	1	5	5	5	25.846		138.52	8.21E+09
7	7	14.7	14.7	14.7	75.532		41.905	5.06E+09
1	1	22.4	22.4	22.4	13.548		60.042	8.64E+08
8	8	19.6	19.6	19.6	54.422		175.91	2.3E+10
4	4	17.7	17.7	17.7	25.158	0.000596	5.5467	7.01E+09
6	6	39.2	39.2	39.2	22.134		72.103	2.85E+10
4	4	17.7	17.7	17.7	37.895		27.711	6.99E+09
3	3	10.5	10.5	10.5	48.393	0.000567	3.6986	7.25E+08
1	1	4	4	4	38.527	0.000646	9.6061	1.22E+09

3	3	7.8	7.8	7.8	67.778		83.532	3.04E+09
4	4	12.8	12.8	12.8	37.081	0.000583	4.6151	2.25E+09
2	2	6	6	6	63.5		83.531	2.72E+08
5	5	19.3	19.3	19.3	30.438		79.645	3.62E+09
5	5	20.5	20.5	20.5	48.315		124.32	7.53E+09
10	10	36.4	36.4	36.4	48.144		114.25	9.49E+09
3	3	53.7	53.7	53.7	12.666		105.71	3.37E+09
2	2	16.1	16.1	16.1	16.604	0.00109	2.7148	3.27E+08
5	5	29.3	29.3	29.3	33.259		53.424	2.52E+09
8	8	22.4	22.4	22.4	51.072		323.31	1.38E+10
5	5	20.9	20.9	20.9	34.062		33.569	1.79E+09
2	2	14.3	14.3	14.3	20.528		24.327	4.2E+08
5	5	20.3	20.3	20.3	32.962		74.282	7.38E+09
5	5	22.3	22.3	22.3	40.884		183.76	1.32E+10
5	5	19.9	19.9	19.9	40.902	0.000618	7.6097	1.67E+09
1	1	4.7	4.7	4.7	38.938		42.052	
2	2	7.4	7.4	7.4	46.651	0.001094	2.8042	5.95E+08
2	2	5.2	5.2	5.2	53.535	0.002144	2.2669	1.82E+09
4	4	14.9	14.9	14.9	60.229		194.03	1.4E+09
4	4	15	15	15	39.392		87.95	1.89E+10
9	9	23.6	23.6	23.6	57.035		125.4	3.38E+10
14	14	38.1	38.1	38.1	48.951		323.31	1.78E+11
1	1	14	14	14	19.418	0.006263	1.7227	3.13E+08
16	16	41.5	41.5	41.5	55.285		323.31	1.75E+11
11	11	23.2	23.2	23.2	78.913		88.886	1.12E+10
1	1	5.6	5.6	5.6	19.525	0.002141	2.2278	2.64E+08
2	2	7.9	7.9	7.9	39.606	0.00161	2.331	4.66E+08
13	13	30.7	30.7	30.7	61.722		323.31	5.8E+10
5	5	17.7	17.7	17.7	49.012		157.6	3.97E+09
6	6	20.9	20.9	20.9	53.513		281.97	4.51E+09
10	10	50	50	50	28.636		323.31	1.42E+10
14	14	37	37	37	41.986		221.25	5.2E+10
1	1	4.2	4.2	4.2	22.812	0.005283	1.935	6.86E+08
10	10	30	30	30	53.277		297.39	9.34E+09
3	3	24.6	24.6	24.6	27.362		37.851	4.49E+09
5	5	17.9	17.9	17.9	26.267		63.347	5.77E+09
2	2	8	8	8	34.916	0.000676	12.641	3.33E+09
1	1	3.5	3.5	3.5	47.626	0.004767	1.9923	1.47E+08
6	6	11.9	11.9	11.9	77.447		274.21	5.66E+09
1	1	11.5	11.5	11.5	12.184	0.00057	3.8969	2.71E+09
2	2	8.5	8.5	8.5	29.45		33.244	2.55E+09
2	2	19.8	19.8	19.8	14.597	0.000715	16.91	2.91E+09
1	1	5	5	5	21.71	0.001095	2.8188	5.8E+09
6	6	39.3	39.3	39.3	28.951		323.31	7.86E+10
5	5	20	20	20	45.016		32.11	6.11E+09
2	2	11.8	11.8	11.8	23.625		253.37	3.15E+10
4	4	29.1	29.1	29.1	19.783		68.39	3.48E+09
8	8	16.2	16.2	16.2	69.439		272.95	9.79E+09
3	3	30.8	30.8	30.8	20.707		101.86	3.93E+09
3	3	16.4	16.4	16.4	27.24	0.000693	14.181	4.07E+09
1	1	5.9	5.9	5.9	20.912	0.005241	1.7742	2.02E+08

3	3	10.1	10.1	10.1	31.459	0.000559	3.2597	3.35E+08
3	3	9.3	9.3	9.3	45.917	0.000581	4.4518	5.16E+08
8	8	13.8	13.8	13.8	105.29		140.87	5.69E+09
3	3	20.5	20.5	20.5	20.719	0.005285	1.958	57506000
4	4	7.4	7.4	7.4	102.76	0.000654	10.415	2.17E+08
12	12	11.4	11.4	11.4	189.17	0.000746	20.956	2.97E+10
8	8	27.5	27.5	27.5	35.079		227.83	3.5E+10
7	7	21.8	21.8	21.8	34.303		137.79	6.06E+09
3	3	28.1	28.1	28.1	22.22		57.898	3.94E+09
5	5	11.2	11.2	11.2	84.166	0.000606	6.2072	5.32E+08
5	5	27.4	27.4	27.4	39.955		72.73	1.82E+10
16	16	30.8	30.8	30.8	79.27		323.31	5.38E+10
1	1	20.4	20.4	20.4	15.717		30.167	2.23E+09
2	2	9	9	9	24.423	0.000709	16.315	1.22E+09
3	3	10.9	10.9	10.9	45.033	0.000591	5.2024	2.59E+08
2	2	8.5	8.5	8.5	36.572		38.97	4.11E+09
2	2	6.4	6.4	6.4	48.54	0.008269	1.5487	6.37E+08
5	5	15.9	15.9	15.9	50.051		323.31	1.05E+10
3	3	16.5	16.5	16.5	27.089	0.000653	10.408	1.44E+09
5	5	24.6	24.6	24.6	43.329		73.421	4.36E+09
3	3	14.3	14.3	14.3	32.521		126.35	8.85E+08
8	8	27.9	27.9	27.9	60.514		323.31	4.11E+10
2	2	36.1	36.1	36.1	9.0412		42.345	4.61E+09
12	12	25.4	25.4	25.4	78.779		323.31	1.59E+10
22	22	42.6	42.6	42.6	94.671		323.31	4.01E+10
24	24	32.5	32.5	32.5	109.78		323.31	7.47E+10
4	4	11.2	11.2	11.2	52.639	0.000552	3.0573	2.29E+09
2	2	13.1	13.1	13.1	18.857	0.000712	16.41	28768000
2	2	6.4	6.4	6.4	75.995	0.000614	7.0723	1.47E+08
2	2	5	5	5	56.18	0.000595	5.4914	2.16E+09
4	4	11.4	11.4	11.4	97.722		38.467	8E+08
2	2	4.2	4.2	4.2	78.567	0.000742	20.631	1.31E+09
1	1	12.2	12.2	12.2	12.56		23.998	2.01E+09
8	8	24.5	24.5	24.5	69.702		208.43	1.79E+10
8	8	15	15	15	91.58		146.09	7.96E+09
6	6	18	18	18	42.143		44.28	1.03E+10
6	6	29	29	29	33.647		323.31	8.3E+09
3	3	10.1	8.4	8.4	45.415		34.012	1.54E+09
4	4	11.8	11.8	11.8	62.023	0.004251	2.0736	1.94E+09
3	3	12.8	12.8	12.8	35.721		166.63	5.9E+09
2	2	16.8	16.8	16.8	14.636		24.48	3.63E+09
2	2	9.7	9.7	9.7	32.827		56.643	1.84E+09
6	6	33.3	33.3	33.3	38.779		113.65	3.56E+09
6	6	24.8	24.8	24.8	27.027		129.03	9.87E+09
2	2	9.9	9.9	9.9	36.144	0.00162	2.4491	2.51E+09
11	11	37.7	37.7	37.7	31.721		269.85	5.29E+10
1	1	3.3	3.3	3.3	35.538	0.000628	8.1826	2.3E+09
3	3	14.7	14.7	14.7	31.825	0.000607	6.2999	2.95E+09
9	9	29	29	29	46.971		71.162	8.85E+09
8	8	58	58	58	23.402		323.31	6.46E+09
11	11	34.7	34.7	34.7	37.977		323.31	1.23E+11

3	3	11.2	11.2	11.2	27.823		73.806	1.28E+10

N: MS/MS count
194
310
174
168
33
440
19
225
225
20
82
231
2696
23
138
461
378
259
73
806
976
731
140
163
88
2
36
23
3
178
4
64
2405
403
73
267
102
54
25
12
137
73
333
174
122
57
283
54
66

79
35
251
122
55
95
5
22
455
229
114
2
21
157
380
319
85
104
643
368
64
337
294
168
209
137
25
33
116
253
30
359
194
85
292
58
32
89
405
23
498
131
271
428
53
257
244
90
163
81
500
262
56

32
341
417
394
50
36
2
39
166
35
498
61
3
360
19
55
376
236
207
334
414
190
90
34
97
63
120
30
35
144
104
46
37
219
29
39
405
64
270
23
207
83
65
471
30
43
22
17
139
6
987
165
27

19
113
7
30
27
83
52
48
104
144
1115
34
104
78
112
160
538
711
50
221
109
9
38
46
24
219
47
287
38
57
223
75
355
113
143
99
27
56
137
156
29
24
27
338
228
113
116
238
117
3
1403
265

15
70
165
143
26
19
202
118
250
193
88
41
84
351
259
93
100
67
46
86
145
123
170
326
81
19
80
44
49
48
40
3
12
168
2
17
115
222
376
35
22
123
69
404
29
167
8
14
22
996
809

9
450
144
28
269
106
6
34
115
115
2
10
204
4
22
66
145
39
684
383
57
93
7
116
1112
1670
400
350
60
60
49
23
14
268
117
1
42
34
64
95
1139
120
7
202
50
307
134
62
49
166
81

78
37
66
211
103
14
77
26
147
32
75
29
31
14
362
122
163
451
48
113
317
536
383
399
173
64
31
23
213
438
87
518
232
359
519
86
1223
359
54
45
54
3
37
106
51
23
48
238
384
121
60

70
78
184
104
9
23
225
1040
138
258
25
215
202
170
604
220
64
171
38
348
41
5
286
186
79
3
146
119
124
21
46
68
50
4
1076
721
101
36
105
113
31
157
107
82
22
22
187
52
55
415
141

157
5
32
27
134
96
95
106
136
56
8
200
481
38
22
295
33
63
28
94
143
47
103
1
95
19
123
68
357
31
165
25
19
13
425
35
14
108
6
17
96
71
40
37
33
140
336
4
43
68
32

48
33
180
148
1
46
69
277
169
153
24
114
152
32
612
328
227
10
37
190
66
80
22
43
124
306
82
10
196
32
12
364
89
33
36
51
11
730
12
17
10
4
152
149
29
114
29
5
7
12
44

53
302
58
49
25
29
8
43
26
62
58
184
16
69
23
82
7
38
102
24
29
22
23
23
125
28
241
12
37
66
164
124
69
182
23
478
30
415
35
358
35
117
62
1026
24
3
41
112
25
59

91
16
37
70
42
79
74
62
26
22
71
16
2
60
21
100
121
3
202
105
3
76
1144
32
12
163
14
22
77
68
37
169
54
141
23
7
61
7
81
16
150
21
50
6
132
147
43
572
11
301
40

6
101
79
5
415
35
48
35
8
153
26
39
76
234
111
37
20
331
93
14
36
15
81
4
15
15
146
126
40
122
64
28
11
423
7
28
24
356
154
62
12
93
124
30
129
83
161
33
5
27
69

158
12
30
129
48
24
127
173
15
57
11
20
4
4
2
9
97
12
37
69
212
34
153
185
392
294
4
114
48
10
76
287
68
113
41
65
80
12
370
12
42
3
122
18
75
25
115
148
169
3
200

86
319
74
93
46
5
320
41
5
92
90
111
58
50
245
9
52
48
34
1
52
124
6
508
146
6
105
144
107
267
228
74
185
168
132
27
18
431
25
106
5
19
110
165
222
84
54
26
70
7
39

36
22
27
146
74
36
32
40
49
233
35
4
5
11
34
135
145
52
108
309
14
7
19
52
83
562
26
40
6
76
62
184
46
28
111
198
83
20
12
13
25
219
36
187
25
196
24
95
35
81
271

58
61
11
15
16
26
11
35
59
16
149
26
10
54
373
6
220
77
32
65
233
225
7
4
26
56
138
4
5
9
102
13
49
16
156
113
275
34
10
82
27
56
48
179
1
10
127
13
39
143
43

54
80
53
336
30
53
7
139
20
272
3
59
20
70
9
193
79
32
42
18
55
300
228
80
133
80
41
52
55
25
41
151
15
150
11
150
218
146
394
27
6
210
363
179
25
182
41
245
38
44
23

84
383
449
88
236
234
9
308
292
25
39
401
113
39
80
339
638
198
96
39
25
17
44
128
47
182
9
41
32
160
340
11
587
46
82
352
119
20
30
185
21
52
43
89
58
179
16
76
63
17
87

85
138
23
3
39
101
109
403
51
38
303
367
573
26
38
41
39
170
59
26
31
1
27
300
205
179
3
207
553
27
21
77
32
303
136
83
91
2
11
317
34
120
41
142
64
59
25
47
99
21
139

103
86
56
80
12
110
40
4
203
17
31
48
77
240
177
290
25
70
69
39
93
55
76
11
87
3
33
56
29
7
88
85
951
14
51
59
118
75
41
1
184
201
312
116
57
53
155
28
25
33
81

60
2
77
133
98
128
236
145
64
61
25
18
7
54
11
19
177
15
332
31
7
370
160
100
4
150
86
3
94
20
31
24
175
32
67
33
52
20
197
12
5
104
97
29
28
28
68
9
25
203
115

103
10
54
915
111
33
19
69
65
16
59
132
58
168
13
8
128
87
10
34
157
76
50
243
60
31
40
32
19
60
171
78
25
1109
73
40
6
225
57
63
127
15
24
43
14
50
20
10
5
15
157

74
7
26
21
89
78
9
44
576
132
260
8
13
2
20
42
1
27
685
24
155
96
42
3
140
61
76
25
426
164
157
3
33
124
43
109
59
268
1
35
133
46
119
40
242
29
216
764
31
285
24

338
394
270
305
5
88
13
4
40
4
348
4
15
198
8
289
514
808
17
109
77
2121
24
46
42
143
72
55
132
155
23
23
249
81
20
26
16
15
63
105
380
127
128
2
107
28
44
395
21
31
44

6
5
68
143
72
76
94
8
5
96
494
468
12
50
65
64
15
88
15
13
53
47
189
129
147
50
54
73
171
31
29
36
55
64
4
607
6
84
68
1
168
4
88
17
175
307
187
59
112
127
2

30
187
56
38
57
36
211
32
79
7
44
65
253
216
63
121
95
21
18
28
23
72
186
1
23
14
65
239
22
23
43
46
45
18
307
140
327
29
15
44
153
17
8
40
55
68
2
31
34
21
19

17
1
4
11
97
63
11
100
69
3
60
20
7
91
130
51
11
72
270
50
37
130
64
14
36
536
12
9
39
208
155
108
2
4
9
6
98
118
3
12
130
358
3
81
27
124
39
68
16
31
213

4
40
63
58
91
103
39
134
28
263
200
75
64
36
13
42
57
83
273
8
3
5
75
164
99
30
3
8
1
29
34
20
496
12
37
62
104
16
5
263
33
72
150
9
78
102
85
35
33
43
15

183
20
32
31
99
239
259
19
111
16
41
115
41
5
37
49
120
22
19
14
68
18
67
20
48
52
38
67
5
14
33
33
33
6
58
195
6
25
7
74
231
31
34
41
199
61
118
611
119
10
66

36
18
96
41
776
51
71
61
22
6
346
218
174
27
48
11
46
40
52
69
31
28
330
11
169
78
89
8
5
14
37
138
9
59
84
168
32
114
282
95
61
38
96
76
6
15
39
38
116
30
697

114
53
29
13
44
27
3
14
281
90
161
6
608
62
53
54
51
12
61
39
5
2
42
27
42
70
47
4
37
27
67
37
283
105
28
38
14
38
54
68
32
48
123
20
11
50
38
287
13
20
35

43
55
115
113
56
42
183
139
179
71
8
217
33
29
44
129
45
117
138
132
41
29
494
81
71
68
340
8
91
483
123
235
22
27
37
23
16
21
34
45
115
175
160
388
120
52
56
44
88
62
3

151
34
17
22
180
669
162
30
149
34
188
52
80
203
24
88
34
37
68
62
75
50
28
21
39
34
27
100
168
29
254
9
1
353
6
8
109
576
62
104
6
67
52
257
2
106
7
372
123
69
398

90
70
124
22
30
73
25
856
18
49
103
259
32
59
2
146
14
34
126
17
265
67
38
82
37
297
159
73
24
4
25
8
7
129
80
103
35
12
116
361
177
123
46
18
4
83
41
49
93
156
39

30
69
36
443
11
156
42
65
123
34
68
67
28
473
103
189
193
67
108
2
41
61
76
38
100
12
42
2
141
84
4
4
77
3
11
1
9
2
5
2
21
36
81
24
171
34
118
101
7
30

54
29
16
66
56
93
97
5
25
140
80
5
94
111
16
3
9
14
33
85
247
453
11
461
119
18
10
232
65
50
193
174
11
109
59
107
22
2
44
27
27
44
23
96
52
63
15
122
103
56
5

24
13
120
4
10
36
83
101
63
8
74
256
11
27
4
62
12
77
16
42
16
131
25
144
332
454
10
21
5
3
20
28
33
123
129
77
111
37
21
81
37
19
61
41
8
188
39
32
62
131
260

Table S2 Quantitative peptides across all samples

T: Proteins	T: Leading	M: Protein	LFQ intens	LFQ intens	LFQ intens	LFQ intens	LFQ intens	LFQ intens
sp|Q9RR6	sp|Q9RR6	65	1.08E+10	1.25E+10	8.55E+09	6.26E+09	5.87E+09	6.24E+09
tr|Q9RY96	tr|Q9RY96	1715			30086000			
tr|Q9RT09	tr|Q9RT09	798	2.68E+08	5.61E+08	5.53E+08	2.9E+08	5.31E+08	3.73E+08
tr|Q9RT09	tr|Q9RT09	798				32755000		
tr|Q9RRK9	tr|Q9RRK9	543						8462200
tr|Q9RRK9	tr|Q9RRK9	543				74369000	67861000	68295000
tr|Q9RSD1	tr|Q9RSD1	693	3.35E+09	4.57E+09	3.57E+09	5.56E+09	3.83E+09	5.06E+09
sp|Q9RY2	sp|Q9RY2	411		1274000	1235000	9513500	7452700	7048000
sp|Q9RY2	sp|Q9RY2	411			14920000	96840000	1.04E+08	86025000
tr|Q9RZB3	tr|Q9RZB3	1896	1.3E+08	1.4E+08	1.7E+08	66847000	64641000	94833000
tr|Q9RTZ0	tr|Q9RTZ0	961	6284000	3200400	3437900	1039300	1731900	1832500
sp|Q9RS6	sp|Q9RS6	112	1.08E+08	1.05E+08	1.07E+08	91899000	61389000	74054000
tr|Q9RRZ6	tr|Q9RRZ6	615	25061000	17029000				
tr|Q9RVK8	tr|Q9RVK8	1248		50435000		70268000		
tr|Q9RUP1	tr|Q9RUP1	1099	5.93E+09	4.46E+09	4.53E+09	2.54E+09	1.96E+09	2.32E+09
tr|Q9RXU6	tr|Q9RXU6	1647	6704000		20444000			12618000
sp|Q9RZE	sp|Q9RZE	455				76131000	46064000	61713000
sp|Q9RS2	sp|Q9RS2	108	1.51E+09	1.12E+09	1.11E+09	7.62E+08	9.44E+08	8.82E+08
sp|Q9RS2	sp|Q9RS2	108		23111000	1.64E+08			
tr|Q9RV94	tr|Q9RV94	1197						
tr|Q9RUX0	tr|Q9RUX0	1129		21562000		25981000		22710000
sp|Q9RRB	sp|Q9RRB	76		2.56E+08	6.54E+08	1.48E+08	1.13E+08	1.64E+08
sp|Q9RRB	sp|Q9RRB	76	2.55E+08	2.9E+08	3.73E+08			
tr|Q9RRS5	tr|Q9RRS5	576	66847000	38779000	37097000	50187000	49456000	54621000
tr|Q9RU17	tr|Q9RU17	976	2.08E+08	1.89E+08	1.76E+08	2.17E+08	2.13E+08	1.83E+08
tr|Q9RU02	tr|Q9RU02	970	1.23E+09	1.2E+09	9.9E+08	2.46E+09	1.91E+09	2.09E+09
tr|Q9RU02	tr|Q9RU02	970	1.93E+09	1.72E+09	1.75E+09	1.32E+09	1.06E+09	1.3E+09
tr|Q9RXG1	tr|Q9RXG1	1588	6.7E+08	9.8E+08	7.42E+08	1.03E+09	8.57E+08	9.73E+08
tr|Q9RXG1	tr|Q9RXG1	1588	4.21E+08	3.17E+08	3.61E+08	4.61E+08	2.38E+08	3.48E+08
tr|Q9RXG1	tr|Q9RXG1	1588						
tr|Q9RSN4	tr|Q9RSN4	741	6.81E+08	8.9E+08	6.68E+08	8.4E+08	8.62E+08	8.79E+08
tr|Q9RSE2	tr|Q9RSE2	701	15824000	8351200	9938800	6191200	9389300	5100700
tr|Q9RSG4	tr|Q9RSG4	713	1.33E+08	1.13E+08	1.19E+08	62071000	58804000	68270000
sp|Q9RRJ5	sp|Q9RRJ5	88	1.5E+08	1.51E+08	1.55E+08	80519000	74854000	83484000
tr|Q9RVX7	tr|Q9RVX7	1318	126610	876840			618940	
tr|Q9RYG4	tr|Q9RYG4	1749						
sp|P56926	sp|P56926	59	1E+08	92004000	86474000	1.04E+08		64065000
sp|Q9RZA	sp|Q9RZA	454	16759000	93123000	98441000	1.2E+08		
tr|Q9RUF9	tr|Q9RUF9	1050	67026000	90602000	78415000	49425000	76337000	69305000
sp|Q9RSA	sp|Q9RSA	114	1.58E+08	2.96E+08	2.79E+08	2.29E+08	1.75E+08	1.53E+08
tr|Q9RSG5	tr|Q9RSG5	714	90230000	59124000	44461000	1.66E+08	1.46E+08	62898000
tr|Q9RSL6	tr|Q9RSL6	732	29367000	21998000	31898000	32948000	38016000	30214000
sp|P56926	sp|P56926	59	6.84E+08	7.11E+08	5.48E+08	4.71E+08	3.87E+08	5.11E+08
tr|Q9RTG9	tr|Q9RTG9	874	46288000	59472000				
tr|Q9RVT5	tr|Q9RVT5	1296	4.54E+08	4.85E+08	4.28E+08	4.59E+08	3.41E+08	3.63E+08
sp|Q9RV5	sp|Q9RV5	265		1950500	1133100	6311500	11962000	9202000
tr|Q9RYW	tr|Q9RYW	1831	1.56E+08	1.36E+08	1.69E+08	2.79E+08	1.6E+08	1.8E+08
tr|Q9RRY2	tr|Q9RRY2	605	17897000	41074000	44777000	47470000	60548000	60783000
tr|Q9RRR4	tr|Q9RRR4	569						26600000

sp|Q9RY2	sp|Q9RY2	412		2.01E+08	1.94E+08	2.44E+08	3.64E+08	1.9E+08
sp|Q9RY2	sp|Q9RY2	412		37640000	60992000			27356000
tr|Q9RWL	tr|Q9RWL	1433				13947000	12781000	
tr|Q9RUB	tr|Q9RUB	1028						
tr|Q9RVX5	tr|Q9RVX5	1317						
tr|Q9RS59	tr|Q9RS59	650						
tr|Q9RWE	tr|Q9RWE	1399	24313000	24045000	20237000	34232000	19684000	19646000
sp|Q9RXF	sp|Q9RXF	369	19826000		34767000			
tr|Q9RX48	tr|Q9RX48	1526	34613000	29639000	33332000	1.08E+08	1.11E+08	1.13E+08
tr|Q9RV82	tr|Q9RV82	1191	10560000	15207000	29634000	27049000		
sp|Q9RRX	sp|Q9RRX	101						
sp|Q9RW(	sp|Q9RW(	342	1.16E+08	53239000	51478000	24685000	35668000	38817000
tr|Q9RZD7	tr|Q9RZD7	1911						
tr|Q9RVK9	tr|Q9RVK9	1249	37514000	30939000	31833000	43033000	22985000	18139000
tr|Q9RV15	tr|Q9RV15	1159				269740	310710	
tr|Q9RSR9	tr|Q9RSR9	754						
tr|Q9RYR7	tr|Q9RYR7	1799	30661000	23672000	24559000	36491000	48234000	34708000
tr|Q9RZ27	tr|Q9RZ27	1857					23084000	32854000
tr|Q9RUJ2	tr|Q9RUJ2	1068						
sp|Q9RSZ	sp|Q9RSZ	159	21347000	15712000	14890000		11604000	
tr|Q9RZJ2	tr|Q9RZJ2	1939						
sp|Q9RVT	sp|Q9RVT	296	2.53E+08	4.35E+08	4.4E+08		1.32E+08	
tr|Q9RV99	tr|Q9RV99	1198	2.77E+08	2.29E+08	2.32E+08	3.58E+08	3.25E+08	3.57E+08
tr|Q9RTY3	tr|Q9RTY3	957	68758000	63943000	72264000	54048000	56034000	59008000
sp|Q9RTA	sp|Q9RTA	175	2.19E+08	1.3E+08	1.51E+08	16606000	18404000	
tr|Q9RT45	tr|Q9RT45	816					6113900	6701400
tr|Q9RRF2	tr|Q9RRF2	515	1.18E+08	1.23E+08	1.34E+08	89799000	77748000	1.05E+08
tr|Q9RYN8	tr|Q9RYN8	1781	65583000	1.13E+08	63377000			92392000
sp|Q9RYU	sp|Q9RYU	444						
sp|Q9RS2	sp|Q9RS2	108		23199000	18788000	40136000	32557000	43615000
sp|Q9RS2	sp|Q9RS2	108						
sp|Q9RSL5	sp|Q9RSL5	137	2.54E+09	1.73E+09	69214000	20175000	19169000	7725500
tr|Q9RUJ2	tr|Q9RUJ2	1068	25242000	46509000	48422000	14567000	12868000	26169000
tr|Q9RRG3	tr|Q9RRG3	519	3046300	900760	765840	532160	880430	692780
tr|Q9RT26	tr|Q9RT26	804	2.65E+08	2.34E+08	5.97E+08	82465000	1.01E+08	1.68E+08
tr|Q9RU07	tr|Q9RU07	972	3.66E+09	5.77E+09	4.84E+09	6.03E+09	6.69E+09	5.42E+09
sp|Q9RRC	sp|Q9RRC	79	4.03E+08	5.97E+08	4.65E+08	4.88E+08	3.82E+08	4.4E+08
tr|Q9RUV4	tr|Q9RUV4	1125	11688000	30505000	39783000	29623000	25979000	49760000
sp|Q9RRX	sp|Q9RRX	101	1.94E+09	1.94E+09	1.77E+09	1.46E+09	1.43E+09	1.51E+09
sp|Q9RV2	sp|Q9RV2	264		43001000	52028000			
tr|Q9RVV7	tr|Q9RVV7	1309						
tr|Q9RSE2	tr|Q9RSE2	701	67271000	68327000	57312000	44349000	52776000	49751000
tr|Q9RXH6	tr|Q9RXH6	1597						
tr|Q9RTK4	tr|Q9RTK4	888	1.6E+08	1.27E+08	1.65E+08	69951000		74555000
tr|Q9RZC1	tr|Q9RZC1	1901						
tr|Q9RY30	tr|Q9RY30	1685						
tr|Q9RZ88	tr|Q9RZ88	1880	2.36E+08	2.3E+08	1.97E+08	1.32E+08	1.18E+08	1.35E+08
tr|Q9RWH	tr|Q9RWH	1409			95534000	54393000	1.05E+08	56633000
sp|Q9RYM	sp|Q9RYM	439	37165000		17541000	10747000		15541000
sp|Q9RYM	sp|Q9RYM	439	5.14E+08	4.97E+08	1.02E+09	2.2E+08	2.21E+08	3.19E+08
tr|Q9RYS7	tr|Q9RYS7	1806	2.74E+08	2.95E+08	2.73E+08	1.36E+08	1.49E+08	2.08E+08
tr|Q9RWE	tr|Q9RWE	1397	59373000		56037000			79426000
tr|Q9RS09	tr|Q9RS09	625						

tr|Q9RVX7	tr|Q9RVX7	1318	35618000			2.58E+08	2.5E+08	2.38E+08
tr|Q9RZD8	tr|Q9RZD8	1912						
tr|Q9RTX8	tr|Q9RTX8	953	65516000	56749000				
tr|Q9RTM	tr|Q9RTM	902						
tr|Q9RXB2	tr|Q9RXB2	1563	24295000	23551000	51010000	17583000	27094000	23752000
sp|Q9RRE	sp|Q9RRE	82	28562000	41018000	32425000	16524000	20213000	29231000
tr|Q9RV86	tr|Q9RV86	1193						
sp|Q9RRM	sp|Q9RRM	92		13234000	21463000	12125000	18946000	16356000
tr|Q9RY36	tr|Q9RY36	1689	1.81E+08	1.11E+08	1.02E+08	51699000	44639000	56165000
tr|Q9RV31	tr|Q9RV31	1167	1.12E+08	1.15E+08	2.88E+08	1.27E+08	2.34E+08	2.38E+08
tr|Q9RYS2	tr|Q9RYS2	1802						
tr|Q9RSP8	tr|Q9RSP8	745	2.1E+08	2.4E+08	2.19E+08	2.51E+08	2.37E+08	2.48E+08
sp|Q5933	sp|Q5933	63	2.84E+08	5.88E+08	1.08E+09	1.05E+08	2.68E+08	1.54E+08
tr|Q9RU54	tr|Q9RU54	997	2.38E+08		1.46E+08			
sp|Q9RXR	sp|Q9RXR	395	41122000	51716000	24319000	45018000		42920000
sp|Q9RRX	sp|Q9RRX	102				18613000	27293000	
tr|Q9RZT3	tr|Q9RZT3	1970	72738000	32807000	40530000			
sp|Q9RTD	sp|Q9RTD	179	73024000	55232000	50452000			
sp|Q9RTD	sp|Q9RTD	179			10193000	9726100	33588000	39704000
tr|Q9RRP4	tr|Q9RRP4	558	20242000	23920000	45286000	57324000	78208000	61718000
tr|Q9RTX8	tr|Q9RTX8	953	21328000	14022000	14080000	16182000	26335000	10639000
sp|Q9RYM	sp|Q9RYM	437		3.17E+08			1.98E+08	
tr|Q9RTQ8	tr|Q9RTQ8	918	87917000	86812000	56054000	34991000	48554000	30751000
tr|Q9RZ88	tr|Q9RZ88	1880	29913000	20158000		16174000	20416000	8088800
tr|Q9RVK8	tr|Q9RVK8	1248	32139000	31045000	49308000		47527000	61596000
tr|Q9RUG	tr|Q9RUG	1052	9.03E+08	9.99E+08	7.59E+08	8.02E+08	6.89E+08	7.06E+08
tr|Q9RUG	tr|Q9RUG	1052			11583000			
tr|Q9RTM	tr|Q9RTM	899	25052000	29857000	4.05E+08	33436000	2.91E+08	2.74E+08
tr|Q9RR86	tr|Q9RR86	477	50306000	41751000	28531000	70504000	57843000	
tr|Q9RR86	tr|Q9RR86	477	95307000	60278000	88417000	29996000	36141000	46483000
tr|Q9RZ94	tr|Q9RZ94	1884	41304000	45025000	47524000	20916000	60834000	17998000
sp|Q9RRP	sp|Q9RRP	93	26481000	9151800	16968000	3805200	7269300	7453600
tr|Q9RSG4	tr|Q9RSG4	713		42045000				
tr|Q9RT33	tr|Q9RT33	809	28711000	32916000	29161000	27222000	24048000	26048000
tr|Q9RTZ9	tr|Q9RTZ9	967	3.74E+08	2.66E+08	4.35E+08	2.34E+08	3.26E+08	4.16E+08
tr|Q9RXX3	tr|Q9RXX3	1661	29553000	33284000	58226000	25435000	22298000	30697000
tr|Q9RT02	tr|Q9RT02	793					601380	
sp|Q9RU8	sp|Q9RU8	219					33790000	46429000
tr|Q9RWK	tr|Q9RWK	1427						
sp|Q9RT3	sp|Q9RT3	166						
tr|Q9RV74	tr|Q9RV74	1188		5522800	4180000	4695000	8653000	7563400
tr|Q9RWS	tr|Q9RWS	1461	1.1E+08	1.24E+08	1.16E+08	1.17E+08	79135000	1.78E+08
sp|Q9RYA	sp|Q9RYA	425						
tr|Q9RYG4	tr|Q9RYG4	1749						
sp|Q9RX9	sp|Q9RX9	363	32358000					
sp|Q9RX9	sp|Q9RX9	363						
sp|Q9RUE	sp|Q9RUE	228						
tr|Q9RUP0	tr|Q9RUP0	1098					10410000	
tr|Q9RTY3	tr|Q9RTY3	957						
tr|Q9RRQ	tr|Q9RRQ	562	21455000	20081000	18416000	15949000	12231000	21882000
tr|Q9RZH5	tr|Q9RZH5	1930					14521000	
tr|Q9RYD0	tr|Q9RYD0	1730						
tr|Q9RYW	tr|Q9RYW	1829	33816000	30919000	32581000	17915000	28317000	18588000

tr|Q9RZP3	tr|Q9RZP3	1953		51447000	40795000	33103000		
tr|Q9RV99	tr|Q9RV99	1198						
tr|Q9RZE5	tr|Q9RZE5	1915	39683000	39704000	32011000	42532000		
tr|Q9RTL6	tr|Q9RTL6	897	1.75E+08		66590000	20762000	16318000	20014000
tr|Q9RUH	tr|Q9RUH	1056	19086000	16202000	13961000	8100500	10238000	
tr|Q9RY21	tr|Q9RY21	1682	24383000		30177000			
tr|Q9RY21	tr|Q9RY21	1682	26523000	65868000	89922000	43240000	96940000	83845000
tr|Q9RU9	tr|Q9RU99	1019	53794000	33057000	45561000	44548000		43863000
tr|Q9RVE9	tr|Q9RVE9	1220						
tr|Q9RS17	tr|Q9RS17	630	4.08E+08	4.42E+08	7.09E+08	4.8E+08	3.51E+08	4.27E+08
tr|Q9RZE3	tr|Q9RZE3	1913	1310000					
tr|Q9RU38	tr|Q9RU38	985	1.95E+08	2.34E+08	1.95E+08	2.07E+08	1.72E+08	34043000
tr|Q9RYS6	tr|Q9RYS6	1805	1.86E+08	1.53E+08	1.56E+08	1.98E+08	1.77E+08	1.77E+08
tr|Q9RWC	tr|Q9RWC	1388			49741000	32973000		
tr|Q9RRR3	tr|Q9RRR3	568	1.53E+09	1.47E+09	1.46E+09	1.65E+09	1.56E+09	1.35E+09
tr|Q9RRP8	tr|Q9RRP8	560				50486000	40735000	
tr|Q9RZQ	tr|Q9RZQ	1957	1.21E+08	1.84E+08	2.31E+08	1.66E+08	1.89E+08	1.82E+08
tr|Q9RUK9	tr|Q9RUK9	1081	1.22E+08	1.49E+08	1.38E+08	2.44E+08	2.82E+08	2.92E+08
tr|Q9RWV	tr|Q9RWV	1484						
sp|Q9RUV	sp|Q9RUV	258		10725000	19129000		22325000	18759000
sp|Q9RU8	sp|Q9RU8	216	64772000	59049000	78572000		65127000	37981000
tr|Q9RUC	tr|Q9RUC	1033						
tr|Q9RTM	tr|Q9RTM	902						
tr|Q9RZC4	tr|Q9RZC4	1903	1.45E+08	1.57E+08	1.73E+08	1.48E+08	1.22E+08	1.99E+08
tr|Q9RW7	tr|Q9RW7	1362	1.69E+08	1.91E+08	1.83E+08	85668000	89594000	1.04E+08
tr|Q9RUY2	tr|Q9RUY2	1139	2.9E+09	4.49E+09	4.13E+09	2.68E+09	3.8E+09	2.68E+09
tr|Q9RX10	tr|Q9RX10	1507	93989000	28286000	25029000	59809000	67528000	11965000
tr|Q9RX10	tr|Q9RX10	1507			33481000			
sp|Q9RW0	sp|Q9RW0	342	12084000	1.49E+08	1.02E+08	70890000	1.9E+08	68742000
tr|Q9RVW	tr|Q9RVW	1310	13082000	7512700	8603300	12319000	11509000	11514000
tr|Q9RXA9	tr|Q9RXA9	1560		22947000	22899000		8167400	16405000
tr|Q9RW3	tr|Q9RW3	1350	1.77E+08	3.68E+08	3.83E+08	3.87E+08	4.86E+08	3.33E+08
sp|Q9RS3	sp|Q9RS3	110	98531000	1.11E+08	1.18E+08	44326000	40213000	44435000
tr|Q9RTW	tr|Q9RTW	945	3.67E+08	3.49E+08	4.79E+08	2.56E+08	3.08E+08	4.41E+08
tr|Q9RTW	tr|Q9RTW	945	57578000	56587000	89148000			
tr|Q9RS15	tr|Q9RS15	629				2.38E+08	2.19E+08	
tr|Q9RUL0	tr|Q9RUL0	1082			41262000			31322000
tr|Q9RZH5	tr|Q9RZH5	1930	44783000			76603000		65868000
sp|Q9RYE	sp|Q9RYE	433			460920		1107700	1106900
tr|Q9RXE8	tr|Q9RXE8	1579	1.47E+08	1.75E+08	1.4E+08	2.62E+08	1.5E+08	2.23E+08
sp|Q9WXF	sp|Q9WXF	461	73412000	74238000		80632000	78797000	75129000
sp|Q9RW0	sp|Q9RW0	325	2.79E+09	2.61E+09	2.29E+09	2.51E+09	2.32E+09	2.26E+09
tr|Q9RV52	tr|Q9RV52	1177	63826000	79960000	62198000	66187000	1.07E+08	68124000
tr|Q9RW8	tr|Q9RW8	1368	45258000	39506000			79867000	
tr|Q9RW8	tr|Q9RW8	1368						21281000
tr|Q9RVN	tr|Q9RVN	1263						
tr|Q9RUQ	tr|Q9RUQ	1103	70747000	72039000	77238000	7733700		
tr|Q9RS01	tr|Q9RS01	619		19730000		13536000		
tr|Q9RXL4	tr|Q9RXL4	1610	31713000	48990000	96686000	45257000	59816000	54383000
sp|Q9RZE	sp|Q9RZE	455	48725000	65496000	88708000	64248000	49788000	60888000
sp|Q9RZV	sp|Q9RZV	460	26093000	2.08E+08	1.61E+08	1.35E+08	1.43E+08	1.39E+08
tr|Q9RRG6	tr|Q9RRG6	522	6343800	1631100	1887000		962600	

tr|Q9RUB	tr|Q9RUB	1028		16050000	23969000	43403000	50398000	58905000
tr|Q9RWA	tr|Q9RWA	1378	79863000	1.21E+08	1.08E+08	2.33E+08	1.91E+08	1.43E+08
tr|Q9RUS7	tr|Q9RUS7	1114	48692000	47704000	34502000	62141000	57859000	50485000
tr|Q9RXU3	tr|Q9RXU3	1646	59976000	2.44E+08	2.12E+08	2.71E+08	2.19E+08	3.19E+08
tr|Q9RRX1	tr|Q9RRX1	601	14792000	1.12E+08	60827000	35418000	76217000	53871000
tr|Q9RXS1	tr|Q9RXS1	1638	2.6E+08	3.69E+08	3.74E+08	1.39E+08	1.92E+08	1.45E+08
tr|Q9RTZ9	tr|Q9RTZ9	967	4.47E+08	3.71E+08	4.2E+08	5.4E+08	6.64E+08	4.75E+08
tr|Q9RYC7	tr|Q9RYC7	1728	4.51E+08	3.86E+08	3.72E+08	89752000	1.59E+08	90983000
tr|Q9RWP	tr|Q9RWP	1449	36044000	22451000	18176000	1.12E+08	1.77E+08	1.49E+08
tr|Q9RZH5	tr|Q9RZH5	1930					20232000	
sp|Q9RYM	sp|Q9RYM	439	6.95E+08	4.33E+08	5.16E+08	98017000	1.45E+08	1.2E+08
tr|Q9RWV	tr|Q9RWV	1484	2.44E+08	1.51E+08	2.13E+08			
tr|Q9RWL	tr|Q9RWL	1433						
sp|Q9RUP	sp|Q9RUP	246	30207000	25324000	45212000	7839900	12952000	7347300
tr|Q9RYN6	tr|Q9RYN6	1780					9208800	
tr|Q9RWQ	tr|Q9RWQ	1453	1.34E+08	1.27E+08	98068000			
tr|Q9RRV0	tr|Q9RRV0	587	1.39E+08	94661000	1.03E+08	2.05E+08	1.61E+08	1.63E+08
tr|Q9RTU7	tr|Q9RTU7	935	52995000	20627000	23782000	21945000	25624000	27094000
tr|Q9RZC1	tr|Q9RZC1	1901						
tr|Q9RRN	tr|Q9RRN	551	1.01E+08	95841000	69203000	45188000	60687000	53034000
sp|Q9RVM	sp|Q9RVM	292	2.63E+08	2.99E+08	2.73E+08	4.17E+08	3.66E+08	4.68E+08
tr|Q9RYF5	tr|Q9RYF5	1744	56289000	69303000	57278000			
tr|Q9RZJ2	tr|Q9RZJ2	1939	54076000	34403000	23987000	24892000	23422000	29609000
sp|O3250	sp|O3250	46						
sp|Q9RUV	sp|Q9RUV	256	55910000					
sp|Q9RX3	sp|Q9RX3	356	39898000	1.15E+08	1.02E+08	69075000	68808000	64601000
sp|Q9RX3	sp|Q9RX3	356			43992000			
tr|Q9RTJ4	tr|Q9RTJ4	884		32597000	69682000	82044000	86263000	1.33E+08
tr|Q9RTJ4	tr|Q9RTJ4	884	50054000	42665000	1.47E+08	5E+08	3.97E+08	4.22E+08
sp|Q9RUA	sp|Q9RUA	224		43523000			58172000	26872000
sp|Q9RUA	sp|Q9RUA	224	30284000	36069000	56434000			
tr|Q9RVP1	tr|Q9RVP1	1267	95947000	70083000	87684000	93283000	55530000	1.28E+08
tr|Q9RU57	tr|Q9RU57	1000		24961000	45112000	40507000	59116000	63651000
tr|Q9RW6	tr|Q9RW6	1360	37034000	34528000	33686000	94246000	1.19E+08	82763000
tr|Q9RVB3	tr|Q9RVB3	1207	25023000	16119000	17424000			
tr|Q9RRH	tr|Q9RRH	526	41977000					
sp|Q9RY0	sp|Q9RY0	410	7.06E+08	5.02E+08	5.54E+08	7.14E+08	6.23E+08	6.56E+08
tr|Q9RX55	tr|Q9RX55	1530	66834000	60340000	1.08E+08	49948000	63039000	1E+08
tr|Q9RRG5	tr|Q9RRG5	521	3.79E+08	5.26E+08	3.5E+08	2.49E+08	2.58E+08	2.79E+08
tr|Q9RW0	tr|Q9RW0	1334	38986000	29712000	19657000	11916000	10451000	13313000
sp|Q9RYM	sp|Q9RYM	438	1.12E+09	1.71E+09	2.61E+09	1.8E+09	1.71E+09	2.34E+09
sp|Q9RYM	sp|Q9RYM	438			35899000	57527000	1.04E+08	1.01E+08
tr|Q9RZQ	tr|Q9RZQ	1957	77808000	74145000	93621000	59038000	51285000	45964000
sp|Q9RUV	sp|Q9RUV	258		7549200		5354900		
tr|Q9RYH1	tr|Q9RYH1	1754				96754000	77111000	80067000
sp|Q9RU1	sp|Q9RU1	207	52338000	45174000	45024000	27208000		14989000
tr|Q9RS10	tr|Q9RS10	626	2.35E+08	3.03E+08	3.65E+08	2.9E+08	1.85E+08	3.27E+08
tr|Q9RTX7	tr|Q9RTX7	952		9443200	21738000			
tr|Q9RX48	tr|Q9RX48	1526						
sp|Q9RXX	sp|Q9RXX	405		16814000	10596000	44281000	57522000	16710000
sp|Q9RV9	sp|Q9RV9	272	881990					

tr|Q9RWU	tr|Q9RWU	1478	4533600	3555300	3934700		3171000	
tr|Q9RXS7	tr|Q9RXS7	1640	16817000	7100400	38987000	23077000	16065000	6770000
tr|Q9RU28	tr|Q9RU28	981	8.2E+08	4.26E+08	8.01E+08	3.14E+08	1.7E+08	4.22E+08
tr|Q9RTR4	tr|Q9RTR4	923	2.1E+08	2.87E+08	2.74E+08	3.09E+08	4.08E+08	3.38E+08
tr|Q9RUS1	tr|Q9RUS1	1111	97986000	19759000	1.51E+08			84841000
tr|Q9RY30	tr|Q9RY30	1685	1.52E+08	1.93E+08	1.82E+08			
tr|Q9RT29	tr|Q9RT29	806	1.4E+08	71355000	2.03E+08	2.93E+08	1.84E+08	2.2E+08
sp|Q9RR9	sp|Q9RR9	73	91541000	75773000	83194000	47232000	65123000	69331000
sp|Q9RT4	sp|Q9RT4	167	6945500	2544800	3480400	38301000	30359000	39192000
tr|Q9RUV4	tr|Q9RUV4	1125						
tr|Q9RW1	tr|Q9RW1	1340	4864600			5872000	8012000	6843400
tr|Q9RSP0	tr|Q9RSP0	743		23027000	23671000	1.05E+08	87870000	80696000
tr|Q9RXI9	tr|Q9RXI9	1606						
tr|Q9RY67	tr|Q9RY67	1701	7.69E+08	1E+09	8.02E+08	1.12E+09	1.43E+09	1.09E+09
sp|Q9RYX	sp|Q9RYX	447	73341000	1.06E+08	1.39E+08	83992000	79997000	1.23E+08
tr|Q9RRS5	tr|Q9RRS5	576				35031000	20736000	
tr|Q9RV83	tr|Q9RV83	1192	1.92E+09	1.95E+09	1.6E+09		6.76E+08	
tr|Q9RYB8	tr|Q9RYB8	1722				1.38E+08	1.15E+08	1.19E+08
sp|Q9RY2	sp|Q9RY2	411	42011000	52333000	38004000	36141000	39649000	43130000
sp|P56926	sp|P56926	59			21838000			
tr|Q9RZV1	tr|Q9RZV1	1981				29180000	23056000	
tr|Q9RRC7	tr|Q9RRC7	501	89146000	1.18E+08	1.18E+08	84940000		75066000
tr|Q9RRC7	tr|Q9RRC7	501						
tr|Q9RSH8	tr|Q9RSH8	719	2.8E+08	3.13E+08	2.64E+08	1.92E+08	3.85E+08	57896000
sp|Q9RRJ1	sp|Q9RRJ1	86	61921000	40744000	41635000	1.2E+08	96685000	90828000
tr|Q9RRM	tr|Q9RRM	549	42720000	1.84E+08	3.56E+08	2.56E+08	3.42E+08	3.32E+08
tr|Q9RYL0	tr|Q9RYL0	1767	7969400	4650600	6405900	4075800	4647600	3790900
tr|Q9RYL0	tr|Q9RYL0	1767						
tr|Q9RT33	tr|Q9RT33	809	28710000		28685000	27421000		43254000
tr|Q9RYL0	tr|Q9RYL0	1767	7032700	2248300	2756100	3267400	5809900	4179500
tr|Q9RYL0	tr|Q9RYL0	1767	76746000	1.46E+08	1.94E+08	37166000	1.39E+08	1.09E+08
sp|Q9RW9	sp|Q9RW9	317	2.06E+08	1.66E+08	1.59E+08	80085000	1.07E+08	98434000
sp|Q9RXJ6	sp|Q9RXJ6	381	1.47E+09	1.34E+09	1.31E+09	2.68E+08	2.89E+08	3.03E+08
sp|Q9RW\	sp|Q9RW\	349			4732800			
sp|Q9RVT	sp|Q9RVT	296	1.42E+08	2.6E+08	2.26E+08	1.57E+08	2.09E+08	1.75E+08
sp|Q9RW\	sp|Q9RW\	348				20457000	1.1E+08	1.29E+08
tr|Q9RRY2	tr|Q9RRY2	605	67000000		50566000			
tr|Q9RZ86	tr|Q9RZ86	1878	8525100		3746200			
sp|Q9RWJ	sp|Q9RWJ	334	49132000	46311000	39612000	4.67E+08	3.62E+08	4.46E+08
tr|Q9RWP	tr|Q9RWP	1449				16913000	17360000	21822000
sp|Q9RU4	sp|Q9RU4	211	37912000	37731000	66924000	40527000	31715000	39815000
tr|Q9RUZ5	tr|Q9RUZ5	1146			13857000		22759000	
tr|Q9RU28	tr|Q9RU28	981	9.66E+08	1.47E+09	8.54E+08	1.09E+09	9.23E+08	1.01E+09
sp|Q9RS8	sp|Q9RS8	113	2.09E+08	2.39E+08	1.99E+08	2.03E+08	3.88E+08	1.64E+08
tr|Q9RTW	tr|Q9RTW	945			2617000		4808500	3201900
tr|Q9RZV4	tr|Q9RZV4	1984	45838000	35421000	96206000			
sp|Q9RU1	sp|Q9RU1	206	40147000	49034000	24564000	1.57E+08	2.11E+08	1.28E+08
tr|Q9RXS0	tr|Q9RXS0	1637	96545000	52560000	35346000	46647000	19794000	34417000
tr|Q9RS56	tr|Q9RS56	648	86911000	1.42E+08	61710000			1.81E+08
sp|Q9RVE	sp|Q9RVE	282						
tr|Q9RYE4	tr|Q9RYE4	1738	3.17E+08	3.27E+08	2.01E+08	2.57E+08	2.76E+08	2.27E+08

sp|Q9RWE	sp|Q9RWE	320	1.11E+08	1.83E+08	3.99E+08		1.91E+08	2.14E+08
sp|O3250	sp|O3250	46						
sp|Q9RWN	sp|Q9RWN	338						
sp|Q9RUV	sp|Q9RUV	258						
sp|Q9RUR	sp|Q9RUR	249		30511000	29493000	49921000	71102000	36151000
tr|Q9RSD6	tr|Q9RSD6	697	27882000	28140000	48444000	31572000	38553000	37593000
tr|Q9RSD6	tr|Q9RSD6	697				75598000	70596000	36459000
sp|Q9RSN	sp|Q9RSN	138	1.18E+08	1.32E+08	1.26E+08	1.05E+08	1.51E+08	1.17E+08
tr|Q9RTK8	tr|Q9RTK8	891						
tr|Q9RUG	tr|Q9RUG	1052						
sp|P56867	sp|P56867	56	1.81E+08	2.52E+08	2.29E+08	64699000	60313000	1.03E+08
sp|Q9RVV	sp|Q9RVV	300	7890700			5034800	7402500	5361400
sp|Q9RVV	sp|Q9RVV	300						
tr|Q9RVE5	tr|Q9RVE5	1219	61138000	40518000	77294000	1.68E+08	1.68E+08	1.47E+08
tr|Q9RVE5	tr|Q9RVE5	1219	31184000		32902000			
sp|Q9RTG	sp|Q9RTG	184	5.92E+08	8.3E+08	6.32E+08	7.5E+08	1.22E+08	1.42E+08
tr|Q9RY46	tr|Q9RY46	1694				53200000	59004000	51091000
sp|Q9RRX	sp|Q9RRX	101	20497000	32633000	18292000	16641000	19484000	22581000
sp|Q9RUY	sp|Q9RUY	259	40532000	18758000	17891000	22050000	21237000	21016000
tr|Q9RT43	tr|Q9RT43	815	2.28E+08	2.2E+08	2.17E+08	2.01E+08	1.6E+08	1.42E+08
tr|Q9RZL7	tr|Q9RZL7	1945				13326000		
tr|Q9RY79	tr|Q9RY79	1708	1.25E+08	1.22E+08	1.06E+08	84553000	77860000	92309000
tr|Q9RX31	tr|Q9RX31	1518	22758000		31073000			
tr|Q9RW3	tr|Q9RW3	1349	44711000	40135000	40209000	65668000	51845000	54318000
tr|Q9RY30	tr|Q9RY30	1685	58405000	51289000	48710000	1.11E+08	1.14E+08	88719000
sp|Q9RR9	sp|Q9RR9	71	30981000	23749000	21465000	19957000	18951000	17131000
tr|Q9RVS5	tr|Q9RVS5	1290						
tr|Q9RY37	tr|Q9RY37	1690	57417000	83505000	1.22E+08	1.15E+08	2.29E+08	1.31E+08
tr|Q9RTJ7	tr|Q9RTJ7	885		1.14E+08	92093000			
sp|Q9RRX	sp|Q9RRX	101						
tr|Q9RV92	tr|Q9RV92	1196	15945000		17695000	23615000		21122000
sp|Q9RRC	sp|Q9RRC	78						
tr|Q9RUB	tr|Q9RUB	1028	1.77E+08	1.42E+08	2.24E+08	67701000	75356000	1.08E+08
sp|Q9RUE	sp|Q9RUE	226	65654000	33053000		60330000	63544000	57829000
tr|Q9RW0	tr|Q9RW0	1332	1.9E+08	1.26E+09	9.15E+08	1.06E+08	1.09E+09	8.76E+08
tr|Q9RYG0	tr|Q9RYG0	1746				21625000	16486000	13552000
sp|Q9RU6	sp|Q9RU6	212						
sp|Q9RRC	sp|Q9RRC	79						
tr|Q9RXC5	tr|Q9RXC5	1570	96713000	48916000	1.21E+08	98980000	79382000	95539000
tr|Q9RWV	tr|Q9RWV	1486		10631000				
tr|Q9RSZ3	tr|Q9RSZ3	789		1.09E+08				
sp|Q9RY7	sp|Q9RY7	423				9634600		
tr|Q9RRA0	tr|Q9RRA0	483		20493000	88762000		75684000	49540000
tr|Q9RUH	tr|Q9RUH	1057	6468800	6965900	7396800	45989000	32605000	38151000
sp|Q9RX3	sp|Q9RX3	356						
tr|Q9RVY5	tr|Q9RVY5	1322				16297000	14996000	
tr|Q9RRP0	tr|Q9RRP0	555				6488600	15927000	
sp|Q9RU2	sp|Q9RU2	209	1.23E+08		56490000	99781000	70082000	1.52E+08
sp|Q9RYH	sp|Q9RYH	435	5550600			28291000	12985000	24539000
tr|Q9RW5	tr|Q9RW5	1357	1.2E+08	1.45E+08	1.21E+08	1.12E+08		98758000
tr|Q9RW5	tr|Q9RW5	1357				36612000	36219000	44331000

tr|Q9RU6	tr|Q9RU62	1004				63672000		
sp|Q9RU1	sp|Q9RU1	205	34551000	33027000	31286000	30860000	28097000	31538000
tr|Q9RX71	tr|Q9RX71	1541	52454000	70680000	61162000	47318000	27245000	43570000
sp|Q9RW	sp|Q9RW7	314	6304800			82744000	1.13E+08	
tr|Q9RWN	tr|Q9RWN	1444			1568000	2449300	3011500	
sp|Q9RSQ	sp|Q9RSQ	141						
tr|Q9RXB	tr|Q9RXB8	1566	63797000	56607000	67221000	2.08E+08	1.53E+08	1.75E+08
sp|Q9RUF	sp|Q9RUF	232		15111000	13591000	29623000	19836000	
tr|Q9RYW	tr|Q9RYW	1826	11965000	48576000	59247000	1.44E+08		13074000
sp|Q9RYM	sp|Q9RYM	439			34489000			
sp|Q9RSF	sp|Q9RSF	117		22571000			24355000	
tr|Q9RXE	tr|Q9RXE3	1575					27007000	
sp|Q9RSK	sp|Q9RSK	129				39406000	32889000	36391000
tr|Q9RZM	tr|Q9RZM	1948						
tr|Q9RSU	tr|Q9RSU3	764	2.57E+08	2.79E+08	2.36E+08	4.71E+08	4.01E+08	3.49E+08
sp|P4244	sp|P42443	49	2.42E+08	4.3E+08	3.42E+08	4.16E+08	5.58E+08	4.65E+08
sp|Q9RT5	sp|Q9RT5	168						
tr|Q9RUP	tr|Q9RUP9	1100		30687000		26926000	30199000	
sp|Q9RUP	sp|Q9RUP	246	65597000	74817000	64072000	76526000	72366000	71284000
tr|Q9RVQ	tr|Q9RVQ	1279	2.08E+08	37054000	2.62E+08	2.59E+08	2.49E+08	2.16E+08
sp|Q9RX5	sp|Q9RX5	360						
sp|Q9RSR	sp|Q9RSR	144	2.42E+08	2.54E+08	2.03E+08	2.7E+08	2.95E+08	2.01E+08
sp|Q9RVM	sp|Q9RVM	292	14988000	7329800	8508000	18711000	16055000	23673000
sp|Q9RSR	sp|Q9RSR	143			36625000			
tr|Q9RUU	tr|Q9RUU	1123				96159000	98139000	64772000
tr|Q9RSA	tr|Q9RSA7	678				28875000	27962000	20351000
tr|Q9RXX	tr|Q9RXX2	1660	15489000	44441000	51926000			
tr|Q9RY2	tr|Q9RY22	1683	36438000	25609000	26440000	35025000	29533000	31143000
tr|Q9RUH	tr|Q9RUH	1057	45004000	53099000	58113000	51257000	36590000	39927000
tr|Q9RT0	tr|Q9RT08	797				86254000	74320000	73679000
tr|Q9RYN	tr|Q9RYN4	1778	98659000	1.1E+08	87313000	1.32E+08	1.31E+08	1.14E+08
tr|Q9RSJ1	tr|Q9RSJ1	727	2.14E+08	1.63E+08	1.96E+08	39981000		
tr|Q9RXL	tr|Q9RXL9	1613		17954000	41599000			21417000
tr|Q9RV5	tr|Q9RV55	1179	1.39E+08	1.7E+08	1.12E+08	1.56E+08	29589000	1.64E+08
tr|Q9RVL	tr|Q9RVL0	1250	66300000	24163000	1.04E+08	1.09E+08	76375000	1.14E+08
sp|Q9RW	sp|Q9RW	330				77359000	57127000	
tr|Q9RRY	tr|Q9RRY4	607	19270000		18348000	56175000	44288000	36215000
tr|Q9RSM	tr|Q9RSM	737						
sp|Q9RSA	sp|Q9RSA	114	1.15E+09	1.09E+09	1.05E+09	1.32E+09	1.37E+09	1.34E+09
tr|Q9RX1	tr|Q9RX19	1514	21945000	18737000	17517000	54687000	33633000	34951000
tr|Q9RUK	tr|Q9RUK	1075						22154000
tr|Q9RTE	tr|Q9RTE8	864						
sp|Q9RXK	sp|Q9RXK	390			14290000			
tr|Q9RTQ	tr|Q9RTQ	914			30975000			
tr|Q9RYV	tr|Q9RYV3	1821	14569000		54283000			
tr|Q9RZ07	tr|Q9RZ07	1845	17637000	7230200	5750000	4095500	6054000	5716900
sp|P5692	sp|P56926	59						
tr|Q9RZH	tr|Q9RZH	1927	58313000	29945000	45963000			
tr|Q9RVA	tr|Q9RVA2	1199	4.35E+08	4.01E+08	3.81E+08	2.6E+08	2.63E+08	66062000
tr|Q9RVA	tr|Q9RVA2	1199						19561000
tr|Q9RSR	tr|Q9RSR9	754		3.64E+08	3.07E+08	4E+08	3.14E+08	3.94E+08

tr|Q9RZ33	tr|Q9RZ33	1861	2.53E+08	3.74E+08	2.38E+08	3.3E+08	3.52E+08	3.06E+08
tr|Q9RV26	tr|Q9RV26	1163	69899000	1.01E+08	79716000	44921000	48150000	
sp|Q9RYY	sp|Q9RYY	448	28151000	15079000				
sp|Q9RSH	sp|Q9RSH	120					3.28E+08	
tr|Q9RVB5	tr|Q9RVB5	1209			818610			
tr|Q9RUX8	tr|Q9RUX8	1136	1.09E+09	1.13E+09	9.31E+08	1.75E+09	1.48E+09	1.43E+09
tr|Q9RRW	tr|Q9RRW	595			5.55E+08	6.41E+08	35273000	74698000
tr|Q9RXX2	tr|Q9RXX2	1660	87882000	1.5E+08	1.29E+08	62253000		62222000
tr|Q9RW7	tr|Q9RW7	1362	34392000	45480000	37967000	1.05E+08	99600000	80890000
sp|Q9RZ0	sp|Q9RZ0	450			5930800			
sp|Q9RRC	sp|Q9RRC	79						
tr|Q9RWQ	tr|Q9RWQ	1453	2.38E+08	2.39E+08	2.16E+08	2.94E+08	2.5E+08	2.57E+08
tr|Q9RWQ	tr|Q9RWQ	1453						
tr|Q9RVS8	tr|Q9RVS8	1292	38205000		32900000	65472000		66971000
tr|Q9RVS8	tr|Q9RVS8	1292		16357000	87526000	32328000	14990000	45466000
tr|Q9RU93	tr|Q9RU93	1017				27733000	16448000	19719000
tr|Q9RVU5	tr|Q9RVU5	1302		16601000	12759000	18503000	15281000	13494000
tr|Q9RTU7	tr|Q9RTU7	935	26264000	21946000	52165000		16275000	16749000
tr|Q9RVJ0	tr|Q9RVJ0	1238	6.71E+08	8.09E+08	1.05E+09	1.04E+08	2.27E+08	1.2E+08
sp|Q9RRB	sp|Q9RRB	76					20920000	
sp|Q9RX3	sp|Q9RX3	356	17220000		1.08E+08			8525300
sp|Q9RR6	sp|Q9RR6	66			20440000			19185000
sp|Q9RYU	sp|Q9RYU	443	4.61E+08	4.35E+08	5.52E+08	4.49E+08	2.51E+08	3.42E+08
tr|Q9RTW	tr|Q9RTW	944						
tr|Q9RV11	tr|Q9RV11	1156	5616400	11153000	4237900	13630000	5594800	4196500
tr|Q9RSD0	tr|Q9RSD0	692	8.28E+08	7.29E+08	7.36E+08	6.98E+08	4.9E+08	5.44E+08
tr|Q9RT97	tr|Q9RT97	843	39780000	52319000	49971000	24388000	36254000	20627000
tr|Q9RRR4	tr|Q9RRR4	569	17796000	13697000	13830000	50293000	43503000	42099000
tr|Q9RTL4	tr|Q9RTL4	895	31156000	54839000	34277000	76583000	1.12E+08	43213000
tr|Q9RS77	tr|Q9RS77	658	28245000	35063000	21307000		11364000	
tr|Q9RSJ4	tr|Q9RSJ4	729	69284000	72921000	64177000			64593000
tr|Q9RSY6	tr|Q9RSY6	784	1.56E+09	3.15E+09	3.05E+09	1.11E+09	1.35E+09	1.28E+09
tr|Q9RSY6	tr|Q9RSY6	784	36972000	57980000	1.09E+08	6845900	26000000	23995000
tr|Q9RUT4	tr|Q9RUT4	1118		28409000	21475000		21962000	29918000
tr|Q9RVH6	tr|Q9RVH6	1234	31148000					25273000
tr|Q9RVG8	tr|Q9RVG8	1230	36932000	24060000	28972000	39808000	33207000	34563000
tr|Q9RSF6	tr|Q9RSF6	709	55033000	1.12E+08	81472000	1.03E+08	1.49E+08	1E+08
tr|Q9RST7	tr|Q9RST7	760			18022000	22511000		24315000
tr|Q9RRH	tr|Q9RRH	525	2E+08	1.75E+08	1.65E+08	3E+08	2.08E+08	2.25E+08
tr|Q9RTU3	tr|Q9RTU3	934	16934000	53049000	1.11E+08	1.33E+08	1.79E+08	1.5E+08
sp|Q9RXT4	sp|Q9RXT4	400	24738000	38514000	56772000	42843000	36619000	32797000
tr|Q9RVG8	tr|Q9RVG8	1230	2.18E+08	1.94E+08	1.69E+08	1.26E+08	1.36E+08	1.49E+08
tr|Q9RVG8	tr|Q9RVG8	1230		30339000	64763000	15617000	62221000	63705000
tr|Q9RTM	tr|Q9RTM	900	1.47E+08	1.19E+08	1.13E+08	76231000	90107000	94435000
sp|O83020	sp|O83020	47	1.42E+08	1.82E+08	1.55E+08	2.76E+08	2.8E+08	2.2E+08
tr|Q9RY02	tr|Q9RY02	1675						
sp|O83020	sp|O83020	47	95680000	1.08E+08	73569000	1.51E+08	1.35E+08	1.52E+08
tr|Q9RXE7	tr|Q9RXE7	1578						
tr|Q9RS70	tr|Q9RS70	654	33628000	32736000	33546000	42512000	35123000	41476000
tr|Q9RVN	tr|Q9RVN	1263						
tr|Q9RY73	tr|Q9RY73	1703	56591000	58222000	64510000		48754000	

sp|Q9RW2	sp|Q9RW2	307	1.72E+09	1.49E+09	1.21E+09	1.1E+09	1.1E+09	1E+09
tr|Q9RV78	tr|Q9RV78	1190	16055000		21837000	72704000	35615000	24267000
sp|Q9RXK	sp|Q9RXK	389	1.63E+08	3.31E+08	3.63E+08	50615000	87348000	47589000
tr|Q9RYW	tr|Q9RYW	1828	1.13E+08	1.27E+08	1.02E+08	46343000	57219000	61073000
tr|Q9RV56	tr|Q9RV56	1180	29668000		44289000	45543000	4.31E+08	37066000
tr|Q9RW3	tr|Q9RW3	1347	59928000	61137000	55896000	1.23E+08	89744000	1.1E+08
tr|Q9RTU7	tr|Q9RTU7	935	2.4E+08	3.77E+08	3.47E+08	8.37E+08	7.16E+08	6.71E+08
tr|Q9RSG4	tr|Q9RSG4	713	89945000	55554000	1.25E+08	51648000	87088000	65426000
tr|Q9RRG1	tr|Q9RRG1	518	15260000		22867000			
tr|Q9RU52	tr|Q9RU52	995	5.46E+08	4.52E+08	4.46E+08	4.03E+08	3.23E+08	3.73E+08
sp|P56905	sp|P56905	57	14702000		17245000			
tr|Q9RZQ	tr|Q9RZQ	1959	1.92E+08	2.14E+08	2.24E+08	4.37E+08	4.85E+08	4.56E+08
sp|Q9RUY	sp|Q9RUY	259			19497000	43216000	39690000	47421000
tr|Q9RUK0	tr|Q9RUK0	1075	1.05E+08	1E+08	83069000	49143000	57321000	71819000
tr|Q9RUK0	tr|Q9RUK0	1075	18646000					
tr|Q9RVA2	tr|Q9RVA2	1199		1.6E+08				
tr|Q9RZ28	tr|Q9RZ28	1858						
tr|Q9RWE	tr|Q9RWE	1399		27140000		26115000	21006000	27095000
tr|Q9RT80	tr|Q9RT80	834	35048000	54497000	35681000	86640000	74597000	63016000
tr|Q9RRF2	tr|Q9RRF2	515	853970		267320			
tr|Q9RY46	tr|Q9RY46	1694	5967600	1950600	1686800	3210500	3757800	3457800
tr|Q9RY46	tr|Q9RY46	1694	816630	599160	1249200		402030	
sp|Q9RX9	sp|Q9RX9	364						
sp|O3250	sp|O3250	45	76370000	43049000	48369000	31573000	29012000	42473000
tr|Q9RW4	tr|Q9RW4	1353	28402000	47220000	6298600	25680000	29640000	26995000
sp|Q9RUP	sp|Q9RUP	245		47993000				1.63E+08
sp|Q9RUP	sp|Q9RUP	245	1.17E+08	1.08E+08	2.22E+08	35434000	38297000	
sp|Q9RW0	sp|Q9RW0	327						
sp|Q9RTY	sp|Q9RTY	204		56446000	55448000	34818000	49740000	32191000
tr|Q9RWB	tr|Q9RWB	1386						
tr|Q9RS56	tr|Q9RS56	648		49453000				
tr|Q9RRW	tr|Q9RRW	595	2421400	1295500	1066800	1187800	2093900	1828800
tr|Q9RW5	tr|Q9RW5	1358				3409400		4040200
sp|Q9RYM	sp|Q9RYM	438			1063800	2268400	3692100	2565000
tr|Q9RUD	tr|Q9RUD	1039	3.03E+08	3.22E+08	3.11E+08	54701000	62017000	59516000
tr|Q9RS41	tr|Q9RS41	641			49328000	39596000	60001000	85117000
sp|Q9RU8	sp|Q9RU8	220	93594000	86600000	77126000		42211000	
tr|Q9RS78	tr|Q9RS78	659					26767000	34937000
tr|Q9RT37	tr|Q9RT37	813	50278000	56430000	1.03E+08	25799000	43503000	28366000
tr|Q9RRZ6	tr|Q9RRZ6	615	67557000	70199000	97954000	29530000	37637000	45222000
sp|Q9RTF	sp|Q9RTF	181						
sp|P56926	sp|P56926	59		4117300	10655000		6401300	5567400
tr|Q9RSU6	tr|Q9RSU6	766		4896500				
sp|Q9RSQ	sp|Q9RSQ	141	90922000	1.28E+08	1.35E+08	6.31E+08	2.76E+08	1.09E+08
sp|Q9RSQ	sp|Q9RSQ	141			18604000	70784000	6076900	79840000
sp|Q9RXK	sp|Q9RXK	387	2.29E+09	1.98E+09	1.96E+09	1.71E+09	1.28E+09	6.07E+08
tr|Q9RY74	tr|Q9RY74	1704	2.73E+08	2.39E+08	1.98E+08	2.24E+08	2.23E+08	2.32E+08
tr|Q9RV29	tr|Q9RV29	1165	20794000	25672000	24452000	28827000	26630000	27503000
sp|Q9RXW	sp|Q9RXW	404	32805000	64828000	57010000	54646000	52493000	40190000
tr|Q9RRI4	tr|Q9RRI4	532	6.17E+08	3.58E+08	3.38E+08	3.75E+08	4.25E+08	3.98E+08
tr|Q9RYQ0	tr|Q9RYQ0	1788			14562000	14660000		8530100

tr|Q9RTZ8	tr|Q9RTZ8	966			45436000	53508000		49950000
tr|Q9RTA2	tr|Q9RTA2	844	41108000	49914000	30135000	45253000		32171000
tr|Q9RRG4	tr|Q9RRG4	520	2.9E+08	33723000	32257000	37822000	22588000	47655000
tr|Q9RXI6	tr|Q9RXI6	1605				16850000		29187000
tr|Q9RXV9	tr|Q9RXV9	1654	44541000		47148000			
tr|Q9RUB	tr|Q9RUB	1028				9009400		6457700
tr|Q9RUB	tr|Q9RUB	1028				30300000	25428000	29911000
tr|Q9RRE0	tr|Q9RRE0	508	1.38E+08	1.97E+08	1.8E+08	1.43E+08	1.12E+08	1.4E+08
sp|Q9RYM	sp|Q9RYM	438		1.11E+08	2.53E+08	6.91E+08	6.81E+08	6.77E+08
tr|Q9RVG6	tr|Q9RVG6	1228	2.72E+08	2.84E+08	2.7E+08	8.53E+08	8.84E+08	6.58E+08
tr|Q9RVG6	tr|Q9RVG6	1228	4.68E+08	4.58E+08	9.27E+08	3.35E+08	3.96E+08	6.83E+08
tr|Q9RS56	tr|Q9RS56	648			1.92E+08	1.75E+08	1.67E+08	57619000
tr|Q9RV48	tr|Q9RV48	1175			14238000			
tr|Q9RVV3	tr|Q9RVV3	1306	23755000	20435000	28424000		36338000	
tr|Q9RZA0	tr|Q9RZA0	1887	1.1E+08	1.18E+08	1.12E+08	1.4E+08	1.17E+08	38732000
tr|Q9RZ86	tr|Q9RZ86	1878	5.17E+08	4.41E+08	4.55E+08	2.3E+08	2.33E+08	3.51E+08
sp|Q9RUE	sp|Q9RUE	226	6.87E+08	1.55E+09	1.24E+09	4.06E+08	6.32E+08	4.32E+08
tr|Q9RRD9	tr|Q9RRD9	507			26032000			
tr|Q9RUU	tr|Q9RUU	1124	2.94E+08	4E+08	3.48E+08	3.51E+08	2.7E+08	4.22E+08
tr|Q9RVS6	tr|Q9RVS6	1291	56969000	53868000	98082000	38037000	41659000	
tr|Q9RRV4	tr|Q9RRV4	591	86624000	85449000	77775000		67795000	60954000
sp|Q9RV9	sp|Q9RV9	272				68922000	37805000	
tr|Q9RYY7	tr|Q9RYY7	1837						9998900
sp|Q9RZA	sp|Q9RZA	454	19493000	9016700	7178400	6676700	10086000	9508500
tr|Q9RZV1	tr|Q9RZV1	1981	96994000	82322000	69570000	98321000	96168000	1.07E+08
tr|Q9RZE6	tr|Q9RZE6	1916		45925000				
tr|Q9RW5	tr|Q9RW5	1357	6.65E+08	1E+09	8.4E+08	6.47E+08	7.9E+08	6.31E+08
tr|Q9RRF4	tr|Q9RRF4	516	59604000	52614000	57625000	60055000	48036000	49680000
tr|Q9RZV4	tr|Q9RZV4	1984	24959000	8543400	9795100	14937000	17570000	11936000
tr|Q9RZV4	tr|Q9RZV4	1984		25112000	82795000	84754000	1.23E+08	69347000
tr|Q9RZV4	tr|Q9RZV4	1984			24931000	40377000	85388000	52626000
tr|Q9RWU	tr|Q9RWU	1479	44352000		34944000			
tr|Q9RY34	tr|Q9RY34	1687			34707000	31850000	54184000	55486000
tr|Q9RS93	tr|Q9RS93	668	93508000	84393000	71768000	1.01E+08	76058000	95279000
tr|Q9RRA7	tr|Q9RRA7	490	28573000	24772000	25299000		14491000	
tr|Q9RRS5	tr|Q9RRS5	576	1.18E+08	62815000	44829000	1.55E+08	1.36E+08	1.45E+08
sp|P42443	sp|P42443	49	62207000	1.47E+08	2.05E+08	1.87E+08	1.7E+08	1.65E+08
tr|Q9RUR9	tr|Q9RUR9	1110	68238000	59886000	59663000	56037000	52589000	52118000
tr|Q9RY79	tr|Q9RY79	1708	4.05E+08	3.04E+08	4.21E+08	85332000	47948000	66168000
sp|Q9RVC	sp|Q9RVC	277	24447000	35256000				
sp|Q9RTI0	sp|Q9RTI0	187	40582000	40873000	43009000	22806000		22888000
tr|Q9RU52	tr|Q9RU52	995	7.11E+08	6.23E+08	6.33E+08	2.27E+08	2.23E+08	2.02E+08
tr|Q9RZ81	tr|Q9RZ81	1875	66420000	60016000	53935000	95118000	88585000	55469000
sp|Q9RUE	sp|Q9RUE	225	99894000	95423000	87107000	1.05E+08	1.13E+08	1.2E+08
tr|Q9RWV	tr|Q9RWV	1484	4.67E+09	5.07E+09	4.07E+09	1.01E+10	8.33E+09	8.93E+09
tr|Q9RY02	tr|Q9RY02	1675	2E+09	2.11E+09	1.82E+09	1.1E+09	7.09E+08	1.09E+09
tr|Q9RWV	tr|Q9RWV	1485	14918000	10352000	14607000			
tr|Q9RW3	tr|Q9RW3	1347			9676500	5777300		
sp|Q9RRL	sp|Q9RRL	90	1.12E+08	1.04E+08	1.08E+08	3.15E+08	2.79E+08	2.7E+08
tr|Q9RVX2	tr|Q9RVX2	1316				16404000		
tr|Q9RUC	tr|Q9RUC	1035	51258000					

tr|Q9RTZ1	tr|Q9RTZ1	962						
tr|Q9RS28	tr|Q9RS28	635			9478900			
tr|Q9RX48	tr|Q9RX48	1526	21117000	28654000	30290000	54352000	74938000	45281000
tr|Q9RX48	tr|Q9RX48	1526						
tr|Q9RUA	tr|Q9RUA	1023				1.11E+08		
tr|Q9RW0	tr|Q9RW0	1335					34889000	58943000
tr|Q9RRZ4	tr|Q9RRZ4	613	1.17E+08	1.02E+08	94663000	1.72E+08	1.16E+08	1.16E+08
tr|Q9RV90	tr|Q9RV90	1195		7639700		12497000		
tr|Q9RWW	tr|Q9RWW	1489		44696000	19223000	50588000		51159000
tr|Q9RYC4	tr|Q9RYC4	1725	47826000	77507000	54772000	96006000	1.09E+08	82925000
tr|Q9RTV7	tr|Q9RTV7	940	43617000	38597000	37452000			
tr|Q9RX72	tr|Q9RX72	1542	1.11E+09	8.92E+08	7.14E+08	7.46E+08	5.43E+08	8.72E+08
sp|Q9RVQ	sp|Q9RVQ	295	1.57E+08	1.12E+08	1.05E+08	1.55E+08	1.28E+08	1.95E+08
tr|Q9RT26	tr|Q9RT26	804		24583000			16317000	
tr|Q9RS28	tr|Q9RS28	635	24098000	18895000	16818000		20741000	
tr|Q9RRW	tr|Q9RRW	598						
tr|Q9RYW	tr|Q9RYW	1829	37375000	35631000	44100000			
tr|Q9RRK9	tr|Q9RRK9	543	1.25E+09	1.12E+09	1.05E+09	1.54E+09	1.47E+09	1.59E+09
sp|Q9RZE	sp|Q9RZE	455	76980000	39404000	57474000	28965000	50190000	
sp|Q9RZE	sp|Q9RZE	455			24275000			
tr|Q9RUF8	tr|Q9RUF8	1049	1.71E+09	1.94E+09	1.37E+09	1.9E+09	1.52E+09	1.91E+09
tr|Q9RSN5	tr|Q9RSN5	742			57111000			
tr|Q9RXV9	tr|Q9RXV9	1654	11675000	10597000	12260000	50958000	26745000	34057000
sp|Q9RW6	sp|Q9RW6	311	97896000	77116000	73206000	61329000	1.43E+08	93630000
tr|Q9RX69	tr|Q9RX69	1540	4.26E+08	4.14E+08	4.37E+08	2.86E+08	4.46E+08	2.87E+08
tr|Q9RS87	tr|Q9RS87	663	8.6E+08	1.87E+09	2.13E+09	3.12E+08	4.42E+08	3.57E+08
sp|Q9RU3	sp|Q9RU3	210						
tr|Q9RYY4	tr|Q9RYY4	1836						
tr|Q9RZG9	tr|Q9RZG9	1926						
tr|Q9RRF7	tr|Q9RRF7	517						
sp|P42443	sp|P42443	49	1.64E+09	1.46E+09	1.14E+09	1.3E+09	1.23E+09	1.45E+09
sp|P56867	sp|P56867	56	2.42E+09	2.65E+09	2.22E+09	6.59E+08	5.77E+08	8.09E+08
tr|Q9RRC7	tr|Q9RRC7	501						
sp|Q9RSH	sp|Q9RSH	119	16622000		11838000			
sp|Q9RSQ	sp|Q9RSQ	141	1.08E+08	81299000	1.12E+08	59634000	64896000	66924000
sp|Q9RU2	sp|Q9RU2	209				49155000	26338000	31772000
tr|Q9RVW	tr|Q9RVW	1311	24645000	37711000	40225000	29209000	1.02E+08	99654000
tr|Q9RXG3	tr|Q9RXG3	1589	1.3E+08	95086000	94509000	24372000	24040000	
tr|Q9RUH	tr|Q9RUH	1058	6190600					
tr|Q9RTE8	tr|Q9RTE8	864	1.49E+08	1.37E+08	1.34E+08		19777000	14644000
tr|Q9RTE8	tr|Q9RTE8	864	99935000	95822000	99099000	1.15E+08	1.54E+08	98995000
tr|Q9RYK8	tr|Q9RYK8	1766	3.05E+08	4.14E+08	3.5E+08	3.7E+08	4.21E+08	4.14E+08
tr|Q9RWM	tr|Q9RWM	1439	1.36E+08	1.36E+08	1.32E+08	62702000	1.34E+08	57700000
sp|O32508	sp|O32508	46						
tr|Q9RVP1	tr|Q9RVP1	1267	23338000		27661000			19528000
tr|Q9RW0	tr|Q9RW0	1333	6.17E+08	7.29E+08	6.12E+08	9.85E+08	5.23E+08	9.45E+08
tr|Q9RW0	tr|Q9RW0	1333	3.51E+09	3.43E+09	3.14E+09	1.28E+09	1.23E+09	1.58E+09
sp|Q9RW0	sp|Q9RW0	325	505620		1324000	1260700		1676600
tr|Q9RV57	tr|Q9RV57	1181	1.24E+08	1.58E+08	1.8E+08	1.28E+08	1.34E+08	1.73E+08
sp|Q9RXJ6	sp|Q9RXJ6	381			15774000	1.1E+08	90496000	62847000
sp|Q9RSY	sp|Q9RSY	157	2.51E+08	2.57E+08	2E+08	1.92E+08	1.73E+08	2.26E+08

tr|Q9RVG8	tr|Q9RVG8	1230			35980000			20513000
tr|Q9RWT	tr|Q9RWT	1465			40622000		42617000	27178000
sp|Q9RW	sp|Q9RW	331	1.17E+08	1.21E+08	1.31E+08		24695000	
sp|P42443	sp|P42443	49	1.33E+08	1.07E+08	1.32E+08	1.22E+08	1.03E+08	1.86E+08
tr|Q9RXF0	tr|Q9RXF0	1581	50362000		86669000			
sp|Q9RUR	sp|Q9RUR	249	28568000	6704100	5837900	3221200	5863500	4760000
sp|Q9RUR	sp|Q9RUR	249	16012000	4971700	3863800		1424700	1026000
tr|Q9RU54	tr|Q9RU54	997	3832000			159300		110250
tr|Q9RXU2	tr|Q9RXU2	1645						3352900
tr|Q9RUU	tr|Q9RUU	1122	46535000	50253000	1.14E+08	48312000	42936000	60373000
tr|Q9RTP5	tr|Q9RTP5	909	3.41E+08	2.48E+08	2.76E+08	6.46E+08	7.26E+08	6.09E+08
tr|Q9RUB	tr|Q9RUB	1028	2.03E+08	2.36E+08	1.89E+08	1.18E+08	1.67E+08	1.25E+08
tr|Q9RVS0	tr|Q9RVS0	1286	9.96E+09	3.17E+09	8.64E+09	5.75E+09	5.59E+09	9.83E+09
tr|Q9RWL	tr|Q9RWL	1430	1.41E+08	1.18E+08	99468000	1.12E+08	1.03E+08	1.18E+08
sp|P56905	sp|P56905	57					16255000	
tr|Q9RT14	tr|Q9RT14	800	6.76E+08	7.05E+08	9.33E+08	5.04E+08	5.83E+08	5.32E+08
tr|Q9RT14	tr|Q9RT14	800	21030000	25066000	49249000		26053000	30677000
tr|Q9RVE5	tr|Q9RVE5	1219	36398000				29754000	
tr|Q9RTF2	tr|Q9RTF2	867						
tr|Q9RWV	tr|Q9RWV	1485	35034000	2.3E+08	2.94E+08	1.46E+08	1.04E+08	88550000
tr|Q9RRK9	tr|Q9RRK9	543	1.67E+09	1.85E+09	1.9E+09	9.07E+08	9.42E+08	7.77E+08
tr|Q9RUY4	tr|Q9RUY4	1140	1.5E+08	10995000	1.24E+08			
tr|Q9RZL4	tr|Q9RZL4	1943	20770000			35569000	40814000	30429000
sp|Q9RSI9	sp|Q9RSI9	122	92559000	1.03E+08	87498000	1.1E+08	1.42E+08	1.15E+08
tr|Q9RXF3	tr|Q9RXF3	1583	71449000	62879000	58819000	86666000	67580000	72838000
tr|Q9RWB	tr|Q9RWB	1385	40123000	58414000	57791000	74226000	48766000	60903000
tr|Q9RUH	tr|Q9RUH	1058	25143000	23880000	19222000	9932900	11632000	
tr|Q9RUF9	tr|Q9RUF9	1050	19551000	74186000	63317000	41263000	80264000	43068000
tr|Q9RUF8	tr|Q9RUF8	1049	1.31E+09	2.16E+09	1.71E+09	1.48E+09	2.08E+09	1.64E+09
tr|Q9RRC7	tr|Q9RRC7	501						
tr|Q9RUD	tr|Q9RUD	1041	7.37E+08	7.65E+08	8.18E+08	4.84E+08	3.77E+08	5.38E+08
tr|Q9RZC7	tr|Q9RZC7	1906	1.89E+09	1.38E+09	1.26E+09	1.02E+09	1.08E+09	9.87E+08
sp|Q9RXR	sp|Q9RXR	395			5624400	5889700	5650500	
tr|Q9RXS7	tr|Q9RXS7	1640	1.36E+09	1.32E+09	1.16E+09	2E+09	1.56E+09	1.73E+09
tr|Q9RWE	tr|Q9RWE	1397						
tr|Q9RVN	tr|Q9RVN	1260	51666000	1.25E+08	93969000	38115000		52978000
sp|Q9RUC	sp|Q9RUC	247	65206000	89166000	93046000			
tr|Q9RUD	tr|Q9RUD	1039	8.11E+08	8.43E+08	1.1E+09	1.61E+09	1.34E+09	7.36E+08
tr|Q9RU06	tr|Q9RU06	971		53597000	40144000			
sp|Q9RRJ	sp|Q9RRJ	87	17522000		22793000	11124000	16427000	
tr|Q9RUS6	tr|Q9RUS6	1113	1.16E+08	1.41E+08	1.11E+08	60855000	59852000	82184000
tr|Q9RRZ1	tr|Q9RRZ1	612	24224000	11826000	12888000	19075000	28822000	23123000
tr|Q9RRZ1	tr|Q9RRZ1	612	23056000		22490000			
tr|Q9RYN6	tr|Q9RYN6	1780	1.27E+08	1.55E+08	1.67E+08	77208000	78012000	88921000
tr|Q9RZT5	tr|Q9RZT5	1972	1.15E+08			1.55E+08		
tr|Q9RX64	tr|Q9RX64	1537	12542000		10817000		16896000	
tr|Q9RTF0	tr|Q9RTF0	866	4236900	3175100				
tr|Q9RTQ9	tr|Q9RTQ9	919	60438000	56961000	48711000	2.73E+08	1.52E+08	1.78E+08
tr|Q9RU01	tr|Q9RU01	969	1.28E+08	1.17E+08	1.09E+08	1.15E+08		1.14E+08
tr|Q9RYA5	tr|Q9RYA5	1719	2.33E+08	1.51E+08	2.02E+08		56214000	78624000
sp|Q9RUE	sp|Q9RUE	229					27928000	20718000

sp|Q9RXT	sp|Q9RXT	401						
sp|Q9RY4	sp|Q9RY4	416				38461000	99197000	49429000
tr|Q9RV47	tr|Q9RV47	1174		27966000	46919000	18519000		32193000
sp|Q9RU2	sp|Q9RU2	209	15799000	1360100	2500600	15404000	22117000	19032000
tr|Q9RXQ	tr|Q9RXQ	1627	1.45E+09	8.51E+08	7.61E+08	2.06E+08	1.83E+08	2.78E+08
sp|Q9RU6	sp|Q9RU6	212	1.31E+08	1.46E+08	1.25E+08	2.09E+08	2.21E+08	1.75E+08
sp|Q9RT5	sp|Q9RT5	168	2.38E+08	1.62E+08	2.68E+08	77223000	1.03E+08	1.35E+08
tr|Q9RSC6	tr|Q9RSC6	689						
tr|Q9RV30	tr|Q9RV30	1166						
sp|Q9RW0	sp|Q9RW0	304	1.02E+08	1.35E+08	1.65E+08	92855000	94958000	64578000
sp|Q9RSQ	sp|Q9RSQ	140						
sp|Q9RW\	sp|Q9RW\	348	23551000	16532000	12323000		1.21E+08	
sp|Q9RYM	sp|Q9RYM	439	39692000	47107000	1.34E+08			
sp|Q9RX3	sp|Q9RX3	356	73402000	1.33E+08	1.72E+08	72803000	1.17E+08	1.08E+08
tr|Q9RVB2	tr|Q9RVB2	1206	8177800	32149000	15872000	33265000		
sp|Q9RVI3	sp|Q9RVI3	289	29800000			30514000	37726000	39720000
sp|Q9RVA	sp|Q9RVA	274	1.92E+08	2.84E+08	2.15E+08	2.15E+08	3.23E+08	2.24E+08
tr|Q9RWS	tr|Q9RWS	1464				62927000	50345000	59922000
tr|Q9RS93	tr|Q9RS93	668	45205000	56125000			10703000	
sp|Q9RU8	sp|Q9RU8	218	1.96E+09	1.56E+09	1.81E+09	8.27E+08	8.43E+08	9.97E+08
tr|Q9RV68	tr|Q9RV68	1185	1.28E+08	93183000	1.55E+08	1.65E+08	1.58E+08	1.52E+08
tr|Q9RT05	tr|Q9RT05	794		2802700	4773100	32061000	43993000	61807000
sp|Q9RY6	sp|Q9RY6	421	1.89E+08	1.26E+08	1.25E+08			43971000
sp|Q9RR7	sp|Q9RR7	67			4001100	3868000		4112500
sp|P42443	sp|P42443	49	71500000	76857000	75231000	60473000	84510000	71047000
tr|Q9RXE8	tr|Q9RXE8	1579				9874200	78303000	79650000
tr|Q9RXE8	tr|Q9RXE8	1579						
sp|Q9RUY	sp|Q9RUY	259						
tr|Q9RS96	tr|Q9RS96	670	1.66E+08	1E+08	1.55E+08	32386000	29809000	53312000
tr|Q9RV57	tr|Q9RV57	1181			2564900	7332700	12070000	11619000
sp|Q9RVA	sp|Q9RVA	274	4.41E+08	3.06E+08	4.1E+08	1.41E+08	1.52E+08	2.37E+08
tr|Q9RS52	tr|Q9RS52	646						
tr|Q9RX10	tr|Q9RX10	1507	2.06E+08	3.56E+08	4.19E+08	38992000	29258000	31043000
tr|Q9RZQ	tr|Q9RZQ	1958	2.87E+08	3.29E+08	3.73E+08	2.05E+08	1.97E+08	2.37E+08
tr|Q9RU92	tr|Q9RU92	1016		44835000	37312000	1.76E+08	1.86E+08	1.74E+08
tr|Q9RR71	tr|Q9RR71	470	83499000	1.14E+08	1.05E+08	1.47E+08	1.98E+08	1.49E+08
tr|Q9RRB1	tr|Q9RRB1	492	66307000	66786000	58637000	90522000		48473000
sp|Q9RRL	sp|Q9RRL	90		34302000		89969000	80683000	75693000
sp|P56867	sp|P56867	56			32827000		11305000	
tr|Q9RYS6	tr|Q9RYS6	1805	16718000					
sp|Q9RWI	sp|Q9RWI	332	45192000	51817000	59998000			
tr|Q9RYG5	tr|Q9RYG5	1750						
tr|Q9RUU	tr|Q9RUU	1124	1.92E+08	1.55E+08	1.33E+08			
sp|Q4657	sp|Q4657	61	1.23E+08	79578000	81543000			
tr|Q9RZ49	tr|Q9RZ49	1863						
tr|Q9RXA0	tr|Q9RXA0	1555			36391000	37019000	35129000	
tr|Q9RUD	tr|Q9RUD	1040	6.54E+08	4.75E+08	5.58E+08	2.13E+08	1.81E+08	1.7E+08
tr|Q9RZQ	tr|Q9RZQ	1957			42907000			27384000
tr|Q9RVS1	tr|Q9RVS1	1287	4.33E+08	5.07E+08	4.1E+08	6.86E+08	8.47E+08	6E+08
tr|Q9RVS1	tr|Q9RVS1	1287	7.31E+08	8.14E+08	1.08E+09	1.82E+08	3.7E+08	3.6E+08
tr|Q9RUP1	tr|Q9RUP1	1099	90131000	1.92E+08	3.94E+08	1.83E+08	4.36E+08	4.15E+08

tr|Q9RUL4	tr|Q9RUL4	1085	3431100	2424200	1474900	2768400		2126600
sp|Q9RWE	sp|Q9RWE	320	5.77E+09	5.86E+09	5.15E+09	7.63E+09	7.17E+09	7.09E+09
sp|Q9RWE	sp|Q9RWE	320	29337000	81849000	1.06E+08		41456000	28018000
tr|Q9RXH8	tr|Q9RXH8	1599	15402000	17181000		25495000	33704000	26002000
tr|Q9RXH8	tr|Q9RXH8	1599	32151000	80931000	1.2E+08	21693000		43689000
tr|Q9RTN0	tr|Q9RTN0	903	1.16E+08	1.01E+08	1.67E+08			
sp|Q9RXK	sp|Q9RXK	387	4.61E+09	4.43E+09	4.38E+09	3.7E+09	3.78E+09	3E+09
sp|Q9RXK	sp|Q9RXK	387	75608000	1.86E+08	3.74E+08	72893000	95860000	95351000
sp|Q9RRE	sp|Q9RRE	81						
sp|Q9RSJ8	sp|Q9RSJ8	126	43340000	7.01E+08	6.79E+08	6.72E+08	7.43E+08	7.65E+08
tr|Q9RTV5	tr|Q9RTV5	938	2.84E+08	2.19E+08	4.14E+08	2.07E+08	2.17E+08	3.09E+08
sp|Q9RXY	sp|Q9RXY	406	5.99E+09	7.09E+09	5.47E+09	5.74E+09	4.2E+09	5.41E+09
sp|Q9RXY	sp|Q9RXY	406						
sp|Q9RVV	sp|Q9RVV	300	39596000	80401000	74384000			
tr|Q9RTV1	tr|Q9RTV1	936						
sp|Q9RXG	sp|Q9RXG	370	1.02E+08	1.25E+08	89770000	52585000	66456000	58310000
tr|Q9RRC0	tr|Q9RRC0	497						
tr|Q9RXC5	tr|Q9RXC5	1570	2.54E+08	2.42E+08	2.44E+08	1.12E+08	1.06E+08	1.3E+08
tr|Q9RZP6	tr|Q9RZP6	1954		9338400	14766000			
tr|Q9RWU	tr|Q9RWU	1480	51397000	46622000	41420000			
tr|Q9RWW	tr|Q9RWW	1490	6.58E+08	6.79E+08	5.22E+08	2.06E+08	2.69E+08	2.21E+08
sp|Q9RS1	sp|Q9RS1	107	92793000	79463000	72939000	3.19E+08	2.52E+08	2.27E+08
tr|Q9RTP5	tr|Q9RTP5	909	14142000	10922000	34368000			
tr|Q9RUU	tr|Q9RUU	1122	2.08E+08	11135000	2.27E+08	1.54E+08		16793000
sp|Q9RXR	sp|Q9RXR	396			27846000		28364000	26639000
tr|Q9RWP	tr|Q9RWP	1449	70068000		1.98E+08		30882000	61982000
tr|Q9RUT0	tr|Q9RUT0	1116	14986000	4501700	20391000	61687000	68721000	43475000
tr|Q9RYT3	tr|Q9RYT3	1812	25224000	6531500				
sp|Q9RR6	sp|Q9RR6	65	2.91E+08	2.96E+08	4.66E+08	4.03E+08	4.33E+08	4.28E+08
sp|Q9RR6	sp|Q9RR6	65			1.21E+08	4.53E+08	4.63E+08	4.26E+08
tr|Q9RSH1	tr|Q9RSH1	717	33759000		93856000	34934000		35172000
sp|Q9RVI3	sp|Q9RVI3	289	1.43E+08	1.37E+08	1.24E+08	66538000	53519000	74036000
tr|Q9RZE4	tr|Q9RZE4	1914			21161000			
tr|Q9RSE1	tr|Q9RSE1	700		24166000	23199000			
sp|P56926	sp|P56926	59	10358000	14151000				
sp|Q9RRJ5	sp|Q9RRJ5	88	2.51E+08	4.19E+08	2.6E+08	2.66E+08	2.68E+08	2.73E+08
tr|Q9RRC7	tr|Q9RRC7	501						
tr|Q9RVS2	tr|Q9RVS2	1288				28124000	29743000	31705000
tr|Q9RSL8	tr|Q9RSL8	734						
tr|Q9RW5	tr|Q9RW5	1357						10312000
tr|Q9RW8	tr|Q9RW8	1367					28346000	
tr|Q9RVJ0	tr|Q9RVJ0	1238			30054000	18359000	33726000	19192000
sp|Q5933	sp|Q5933	63				42107000	92078000	62488000
tr|Q9RRC7	tr|Q9RRC7	501			35776000			
sp|Q9RW0	sp|Q9RW0	326						
sp|Q9RW0	sp|Q9RW0	342				1382400	4105500	2103400
tr|Q9RVG6	tr|Q9RVG6	1228				35388000	27866000	23718000
tr|Q9RRV7	tr|Q9RRV7	592	8187900	16780000	20122000	40286000	51461000	58432000
sp|Q9RSL1	sp|Q9RSL1	134					1333600	
sp|Q9RSL1	sp|Q9RSL1	134			417740			
tr|Q9RXD1	tr|Q9RXD1	1571	3.87E+08	3.8E+08	3.65E+08	6.25E+08	3.16E+08	3.44E+08

tr|Q9RZ16	tr|Q9RZ16	1852	4677900	5185400	6375400	9880700	19023000	
tr|Q9RWZ	tr|Q9RWZ	1503						
tr|Q9RWA	tr|Q9RWA	1379	3.75E+08	3.35E+08	2.89E+08	46950000	3.1E+08	2.69E+08
tr|Q9RV57	tr|Q9RV57	1181						
tr|Q9RVZ2	tr|Q9RVZ2	1325			28255000			
tr|Q9RUE4	tr|Q9RUE4	1045	22578000	31613000				
sp|Q9RSL1	sp|Q9RSL1	134	3.28E+09	3.02E+09	2.63E+09	6.74E+08	8E+08	9.11E+08
sp|Q9RU8	sp|Q9RU8	216	6.82E+08	6.08E+08	1.02E+09	6.56E+08	7.68E+08	8.24E+08
sp|Q9RU8	sp|Q9RU8	216			1.21E+08	38858000	50253000	41291000
tr|Q9RUJ1	tr|Q9RUJ1	1067	1.91E+08	1.67E+08	1.77E+08	3.32E+08	3.12E+08	3.15E+08
tr|Q9RRC5	tr|Q9RRC5	500				44315000		38604000
tr|Q9RUX7	tr|Q9RUX7	1135	28936000	26436000		32263000	35204000	47557000
sp|Q9RS1	sp|Q9RS1	106						35950000
sp|Q9RS1	sp|Q9RS1	106	24406000	42056000	97700000			60810000
tr|Q9RV99	tr|Q9RV99	1198						
sp|Q9RVI3	sp|Q9RVI3	289			18679000			17725000
sp|Q9RU8	sp|Q9RU8	216	12964000	14568000	14321000	20766000	36338000	32563000
sp|Q9RU8	sp|Q9RU8	216				18396000		27520000
tr|Q9RU52	tr|Q9RU52	995	6.53E+08	5.3E+08	5.3E+08	5.77E+08	5.77E+08	5.42E+08
tr|Q9RU52	tr|Q9RU52	995	52089000	54953000	1.13E+08	19426000	44391000	35791000
sp|Q9RZA	sp|Q9RZA	454	28309000	32536000	27030000	26473000	23955000	22471000
tr|Q9RRE7	tr|Q9RRE7	513	16089000	13999000	12939000		27336000	22285000
tr|Q9RRD4	tr|Q9RRD4	504	1.49E+08	1.42E+08	1.22E+08	2.71E+08	2.26E+08	2E+08
tr|Q9RYD9	tr|Q9RYD9	1735	1463500		413920			
tr|Q9RYA6	tr|Q9RYA6	1720						
tr|Q9RWT	tr|Q9RWT	1471	35900000	40033000	41186000	90793000	94292000	87125000
tr|Q9RVR2	tr|Q9RVR2	1281	5.53E+08	5.67E+08	6.73E+08	1.77E+09	1.03E+09	8.7E+08
tr|Q9RVR2	tr|Q9RVR2	1281		13778000	28348000			
tr|Q9RXD5	tr|Q9RXD5	1573	28107000	24675000	42646000	18817000	20826000	
tr|Q9RU57	tr|Q9RU57	1000	50128000	30897000	37519000			
tr|Q9RTM	tr|Q9RTM	898	6754600	5356100	4805300	18324000	22183000	20078000
tr|Q9RVK9	tr|Q9RVK9	1249	2.63E+08	2.56E+08	2.25E+08	1.61E+08	1.57E+08	1.8E+08
sp|Q9RT2	sp|Q9RT2	162	5.3E+09	5.13E+09	4.23E+09	5.62E+09	5.01E+09	5.75E+09
tr|Q9RZD8	tr|Q9RZD8	1912						
tr|Q9RYV6	tr|Q9RYV6	1823	23882000	16079000	16307000	9854300	11858000	11422000
sp|Q9RT4	sp|Q9RT4	167	6.88E+08	8.97E+08	7E+08	1E+09	8.17E+08	8.76E+08
tr|Q9RSR2	tr|Q9RSR2	750						
tr|Q9RZJ1	tr|Q9RZJ1	1938						
tr|Q9RSR9	tr|Q9RSR9	754	4961300	3502200	3177900	8700200	10310000	7308500
tr|Q9RYG5	tr|Q9RYG5	1750	36287000	49273000	34487000			
sp|Q9RYZ	sp|Q9RYZ	449						
tr|Q9RX12	tr|Q9RX12	1509			17915000			
tr|Q9RYK8	tr|Q9RYK8	1766						
sp|Q9RSW	sp|Q9RSW	152						35717000
tr|Q9RZS3	tr|Q9RZS3	1964						
sp|Q9RWH	sp|Q9RWH	328	2.36E+08	1.73E+08	2.05E+08		49398000	
sp|Q9RWH	sp|Q9RWH	328						
sp|Q9RY7	sp|Q9RY7	423	2.83E+08	2.79E+08	2.46E+08	95547000	1.17E+08	1.16E+08
tr|Q9RYZ9	tr|Q9RYZ9	1842	14839000	22469000				
sp|Q9RYL	sp|Q9RYL	436		63837000	53059000	70160000	56411000	
tr|Q9RV11	tr|Q9RV11	1156	16297000	8089500	5266400	36347000	5642700	

tr|Q9RZ23	tr|Q9RZ23	1855	2.12E+08	1.82E+08	1.54E+08	1.95E+08	1.77E+08	1.87E+08
sp|Q9RXF	sp|Q9RXF	369	11289000	6314400	4783200	4982700	5916400	5686700
tr|Q9RYG4	tr|Q9RYG4	1749	42738000	27778000	25849000	14436000	12659000	
tr|Q9RT75	tr|Q9RT75	831			5019700	10928000	11542000	14114000
sp|Q9RUY	sp|Q9RUY	259	1.94E+08	4.97E+08	1.12E+09	5E+08	6.91E+08	7.54E+08
tr|Q9RYP4	tr|Q9RYP4	1783	25530000		20211000	38342000	41504000	31212000
sp|Q4657	sp|Q4657	62	43943000	43757000		56160000		
tr|Q9RXP5	tr|Q9RXP5	1625	59576000	28498000		79107000		
sp|Q9RWH	sp|Q9RWH	329	21489000		2.06E+08	2.78E+08	3.24E+08	3.28E+08
tr|Q9RR75	tr|Q9RR75	472	17600000	41153000	31127000	19743000	41387000	
tr|Q9RVF8	tr|Q9RVF8	1225	9.22E+08	7.95E+08	5.8E+08	1.16E+09	1.08E+09	1.06E+09
tr|Q9RV78	tr|Q9RV78	1190	12032000					
tr|Q9RUK8	tr|Q9RUK8	1080	1.11E+08	2.93E+08	2.31E+08			
tr|Q9RTM	tr|Q9RTM	902				20675000	17902000	
tr|Q9RV15	tr|Q9RV15	1159	4.08E+08	3.3E+08	2.54E+08	1.96E+08	2.08E+08	2.26E+08
tr|Q9RU56	tr|Q9RU56	999	74919000	91144000	1.83E+08	94578000	1.5E+08	1.25E+08
tr|Q9RX95	tr|Q9RX95	1553	8295000		10305000	16223000	10495000	12883000
tr|Q9RRA5	tr|Q9RRA5	488		40922000	35997000		33148000	77855000
tr|Q9RU52	tr|Q9RU52	995	23948000	22925000	37431000	6993400	11768000	13106000
sp|Q9RUE	sp|Q9RUE	226			8652900		7674000	8068800
sp|Q9R34	sp|Q9R34	64	7.57E+08	4.39E+08	4.42E+08	2.25E+08	3.15E+08	3.45E+08
tr|Q9RVU8	tr|Q9RVU8	1303	1.65E+08	1.94E+08	3.03E+08	1.5E+08	1.76E+08	1.81E+08
tr|Q9RRA1	tr|Q9RRA1	484	1.6E+08	2.63E+08	1.69E+08	25274000	2.31E+08	2.26E+08
sp|Q9RW0	sp|Q9RW0	342				557580	868060	698510
sp|Q9RW0	sp|Q9RW0	342			67387000	35646000		47391000
sp|Q9RW0	sp|Q9RW0	342			64146000	40180000		35493000
sp|Q9RY0	sp|Q9RY0	410				34112000	30036000	38104000
tr|Q9RY34	tr|Q9RY34	1687	8.05E+08	51664000	52047000	5.05E+08	56944000	4.62E+08
tr|Q9RZ07	tr|Q9RZ07	1845	2.55E+09	2.06E+09	1.77E+09	1.95E+09	1.56E+09	1.96E+09
tr|Q9RZ07	tr|Q9RZ07	1845			3052700	13299000	17026000	16994000
sp|Q9RUV	sp|Q9RUV	253	6776500		20636000	1.67E+08	95018000	76068000
sp|Q9RUV	sp|Q9RUV	253	15466000	11151000	53064000	1.06E+08	33752000	41959000
sp|Q9RUV	sp|Q9RUV	253			12512000	28757000		57583000
tr|Q9RYS0	tr|Q9RYS0	1800						
sp|Q9RVL	sp|Q9RVL	290	23587000	17371000	14663000		20845000	13396000
tr|Q9RVY4	tr|Q9RVY4	1321	81495000	50185000	41321000	58555000	51419000	39575000
tr|Q9RVY4	tr|Q9RVY4	1321	27196000	48071000	68453000	13578000	18047000	18075000
sp|Q9RXS	sp|Q9RXS	397	3.5E+08	3.06E+08	2.93E+08	1.84E+08	1.44E+08	1.99E+08
tr|Q9RWK	tr|Q9RWK	1429	27512000	19548000	23443000	10359000	10024000	17354000
sp|Q9RSL1	sp|Q9RSL1	135	13399000	16879000	34537000			
tr|Q9RRQ	tr|Q9RRQ	563						
sp|Q9RXC	sp|Q9RXC	366						
tr|Q9RT82	tr|Q9RT82	836	1.03E+08	1.24E+08	1.09E+08	1.12E+08	82501000	1.34E+08
tr|Q9RTK3	tr|Q9RTK3	887	57238000	37744000	29627000	50085000	35015000	
tr|Q9RSZ9	tr|Q9RSZ9	791	14346000	20879000	29929000	16518000	22922000	21096000
tr|Q9RTC7	tr|Q9RTC7	853			8980100	14628000		
sp|Q9RXF	sp|Q9RXF	369						
sp|Q9RXG	sp|Q9RXG	370	7.44E+08	5.36E+08	5.17E+08	5.34E+08	3.65E+08	4.62E+08
sp|Q9RXG	sp|Q9RXG	370		17708000	29239000	7410500	10722000	12849000
tr|Q9RZ23	tr|Q9RZ23	1855		9611500	14986000			
tr|Q9RZV1	tr|Q9RZV1	1981		13908000	6857400	22269000	17257000	11660000

tr|Q9RVK1	tr|Q9RVK1	1244	18584000		16708000	32850000	27557000	23520000
tr|Q9RVK1	tr|Q9RVK1	1244	1.18E+08	2.07E+08	1.83E+08	2.33E+08	1.41E+08	2.13E+08
sp|Q9RS6	sp|Q9RS6	112	2.98E+08	3.75E+08	3.08E+08	1.91E+08	2.76E+08	2.13E+08
tr|Q9RWB	tr|Q9RWB	1384	34569000	58291000	49813000	74637000		53333000
tr|Q9RWK	tr|Q9RWK	1426	8285800	7400700	8302300	28764000	42517000	31970000
tr|Q9RXH	tr|Q9RXH	1594	19278000	14691000	12076000	11037000	11503000	
sp|Q9RVV	sp|Q9RVV	300	2.53E+08	5.7E+08	6.7E+08	1.17E+08	2.46E+08	2.49E+08
sp|Q9RY6	sp|Q9RY6	420		4.87E+08	5.13E+08			
tr|Q9RYM	tr|Q9RYM	1773	62477000	96553000	1.1E+08	41157000	50529000	36881000
tr|Q9RUL9	tr|Q9RUL9	1087	12976000		11333000	16771000	12572000	
tr|Q9RU08	tr|Q9RU08	973	2.04E+08	1.38E+08				
tr|Q9RYK8	tr|Q9RYK8	1766	2.41E+08	2.74E+08	2.1E+08	1.83E+08	1.48E+08	1.85E+08
tr|Q9RTQ7	tr|Q9RTQ7	917	24305000	10777000	11185000	12743000	15637000	15420000
tr|Q9RTQ7	tr|Q9RTQ7	917	1.15E+09	1.26E+09	1.35E+09	3.63E+08	4.59E+08	4.95E+08
sp|Q9RX1	sp|Q9RX1	351	10862000	52573000	56704000	24948000	22835000	22484000
tr|Q9RSN4	tr|Q9RSN4	741	3.01E+08	3.12E+08	3.1E+08	1.36E+08	1.48E+08	1.11E+08
tr|Q9RUH	tr|Q9RUH	1056	1872200					
tr|Q9RTL4	tr|Q9RTL4	895				40159000	23443000	14933000
tr|Q9RYY7	tr|Q9RYY7	1837			11271000			
tr|Q9RSR7	tr|Q9RSR7	752		6761700				
tr|Q9RZT0	tr|Q9RZT0	1968	2.84E+08	3.18E+08	3.37E+08	1E+08	1.82E+08	1.55E+08
tr|Q9RWW	tr|Q9RWW	1488	46587000					
tr|Q9RS66	tr|Q9RS66	653	2.77E+08	2.77E+08	2.61E+08	1.49E+08	1.57E+08	1.84E+08
tr|Q9RS66	tr|Q9RS66	653			47926000	37525000	37689000	42857000
tr|Q9RVG8	tr|Q9RVG8	1230						
tr|Q9RWD	tr|Q9RWD	1396	3374100		5606000			
tr|Q9RT96	tr|Q9RT96	842	24897000	26530000	27160000	42048000	45756000	45212000
tr|Q9RRT6	tr|Q9RRT6	580		20310000		17355000	9092200	
sp|Q9RX1	sp|Q9RX1	352	9.92E+08	1.05E+09	8.2E+08	5.65E+08	6.9E+08	5.17E+08
tr|Q9RTY1	tr|Q9RTY1	956	2.05E+08	1.66E+08	1.87E+08		45557000	
tr|Q9RV11	tr|Q9RV11	1156	93500000	1.11E+08	78310000	99098000	60410000	97305000
tr|Q9RV11	tr|Q9RV11	1156		26321000	20175000			
tr|Q9RS56	tr|Q9RS56	648	47857000	46168000	38528000	35296000	47259000	38289000
tr|Q9RS71	tr|Q9RS71	655	98687000	1.18E+08	1.88E+08			62467000
tr|Q9RRP5	tr|Q9RRP5	559	67118000	57704000	46397000	61691000	73326000	58169000
tr|Q9RXI1	tr|Q9RXI1	1601	2.15E+08	2.5E+08	2.79E+08	1.9E+08	1.68E+08	1.75E+08
tr|Q9RXI1	tr|Q9RXI1	1601			19362000			
sp|Q9RUD	sp|Q9RUD	227	14123000	10203000	7363200	11182000	16109000	12453000
tr|Q9RT95	tr|Q9RT95	841	99339000	1.2E+08	38567000	83883000	56101000	80328000
tr|Q9RZC3	tr|Q9RZC3	1902						
tr|Q9RXQ	tr|Q9RXQ	1627			22760000	64738000	55995000	
sp|Q9RYG	sp|Q9RYG	434						
tr|Q9RRZ7	tr|Q9RRZ7	616	2.82E+08	2.77E+08	2.5E+08	56925000	81006000	92235000
tr|Q9RS00	tr|Q9RS00	618			12972000			
tr|Q9RS41	tr|Q9RS41	641						
sp|P52153	sp|P52153	52		28772000	23000000	34861000	46630000	37175000
sp|Q9RYB	sp|Q9RYB	426		393540		283010		
tr|Q9RR73	tr|Q9RR73	471	18555000	32634000	28988000	39352000		38289000
tr|Q9RXU3	tr|Q9RXU3	1646						
tr|Q9RWP	tr|Q9RWP	1446						
tr|Q9RRW	tr|Q9RRW	595	10309000		6123600	63286000	51880000	62823000

sp|O3250	sp|O3250	46		32887000	23690000	48926000	43480000	62365000
sp|O3250	sp|O3250	46			11400000			
tr|Q9RZ7	tr|Q9RZ7	1871						
tr|Q9RZ7	tr|Q9RZ7	1871						
sp|Q9RUZ	sp|Q9RUZ	260	4.36E+08	4.1E+08	3.73E+08	6.31E+08	5.4E+08	6.4E+08
sp|O8302	sp|O8302	47	72196000	82311000	52740000		89423000	78524000
tr|Q9RV1	tr|Q9RV1	1160	1.35E+08	1.39E+08	1.55E+08			
sp|Q9RT0	sp|Q9RT0	161	34300000	44327000	41216000	28605000	27625000	41894000
tr|Q9RX1	tr|Q9RX1	1507	1.59E+09	1.18E+09	1.02E+09	3.83E+09	3.6E+09	2.98E+09
tr|Q9RX1	tr|Q9RX1	1507	2.11E+08	1.65E+08	3.04E+08		79853000	73971000
tr|Q9RZ3	tr|Q9RZ3	1860			13033000			
tr|Q9RYX	tr|Q9RYX	1834	53794000	42047000	40043000	86583000	91271000	74721000
sp|Q9RRX	sp|Q9RRX	101						
tr|Q9RTT	tr|Q9RTT	931	25773000		13250000	37382000	18359000	21806000
sp|Q9RXY	sp|Q9RXY	406			35845000		5221000	10002000
sp|Q9RSC	sp|Q9RSC	115	6.39E+08	4.7E+08	4.76E+08	3.8E+08	4.65E+08	3.87E+08
tr|Q9RVE1	tr|Q9RVE1	1218	13818000	12692000	14095000			
sp|Q9RU1	sp|Q9RU1	205	19609000	14827000	22342000			
sp|Q9RUY	sp|Q9RUY	259	6053200		37559000	46129000	91787000	54702000
tr|Q9RSQ	tr|Q9RSQ	748	2.21E+09	2.37E+09	3.64E+09			
tr|Q9RSQ	tr|Q9RSQ	748	55530000	96169000	2.75E+08			
tr|Q9RTR1	tr|Q9RTR1	920	1.3E+08	1.13E+08	1.08E+08	55332000	37335000	68239000
tr|Q9RVA	tr|Q9RVA	1200	57874000	82519000	96332000	5.93E+08	2.31E+08	1.26E+08
tr|Q9RT7	tr|Q9RT7	827	49297000	13151000	19796000	1.91E+08	46290000	33449000
tr|Q9RT7	tr|Q9RT7	827			11338000			
sp|Q9RXJ	sp|Q9RXJ	375	6.55E+08	1.02E+09	6.55E+08	3.91E+08	6.19E+08	4.63E+08
sp|Q9RXJ	sp|Q9RXJ	375			2.34E+08			
tr|Q9RVS	tr|Q9RVS	1291	97701000	88489000	68708000	52435000	46881000	42654000
tr|Q9RYM	tr|Q9RYM	1774						
sp|Q9RTN	sp|Q9RTN	195			14963000			
tr|Q9RW3	tr|Q9RW3	1349						
sp|Q9RTJ	sp|Q9RTJ	189						
sp|Q9RW	sp|Q9RW	335	77761000	69832000	72651000	2.01E+08	1.68E+08	1.7E+08
sp|Q9RX6	sp|Q9RX6	361						
sp|Q9RVV	sp|Q9RVV	300		86867000		1.19E+08	2.12E+08	93403000
sp|Q9RVV	sp|Q9RVV	300						
tr|Q9RV5	tr|Q9RV5	1179						
sp|Q9RXI	sp|Q9RXI	374	1.39E+08	1.86E+08	1.61E+08	1.06E+08	1.17E+08	1.2E+08
tr|Q9RW2	tr|Q9RW2	1346	7453200					
tr|Q9RRA	tr|Q9RRA	489			23277000			
tr|Q9RS04	tr|Q9RS04	621			10718000			
sp|P5692	sp|P5692	59	14166000		12695000			
tr|Q9RVT	tr|Q9RVT	1297	2.22E+08	2.08E+08	1.67E+08	2.24E+08	2.64E+08	3.17E+08
tr|Q9RYT	tr|Q9RYT	1812					40251000	
tr|Q9RRP	tr|Q9RRP	561			24406000			
sp|Q9RUF	sp|Q9RUF	231	79981000	57933000	42437000	1.34E+08	1.31E+08	1.33E+08
tr|Q9RTC	tr|Q9RTC	855	21559000	2.83E+08	2.68E+08	90818000	3.26E+08	56048000
tr|Q9RTC	tr|Q9RTC	855			28595000			
tr|Q9RSW	tr|Q9RSW	776	59475000	68919000	58704000	59509000	65113000	62451000
tr|Q9RT7	tr|Q9RT7	831	2.2E+08	1.6E+08	2.73E+08	1.03E+09	5.85E+08	4.59E+08
tr|Q9RVN	tr|Q9RVN	1262		27266000	51338000		48589000	29531000

tr|Q9RRG	tr|Q9RRG	522	3.2E+08	2.94E+08	4.68E+08	19197000	2.17E+08	2.8E+08
tr|Q9RYM	tr|Q9RYM	1772	3.3E+08	2.64E+08	2.13E+08	3.19E+08	3.41E+08	3.52E+08
tr|Q9RYF5	tr|Q9RYF5	1744		9212800	14230000			
tr|Q9RW9	tr|Q9RW9	1375	31783000	58290000	28148000	2.26E+08	35212000	1.99E+08
sp|Q9RU1	sp|Q9RU1	205	23619000					
tr|Q9RTH	tr|Q9RTH	877	2.89E+08	1.25E+08	1.99E+08			
sp|Q9RZE	sp|Q9RZE	455			42130000		52485000	45098000
sp|Q9RX0	sp|Q9RX0	350		9934000	23460000			
sp|Q9RYV	sp|Q9RYV	445		27775000		17182000		28712000
sp|Q9RYV	sp|Q9RYV	445	3.35E+08	2.99E+08	3.56E+08	1.28E+08	1.42E+08	1.24E+08
sp|Q9RX1	sp|Q9RX1	351	2.15E+08	1.66E+08	1.75E+08	91486000	75053000	96690000
tr|Q9RS31	tr|Q9RS31	637	1.15E+08	12399000	1.32E+08	21817000		55297000
tr|Q9RUT	tr|Q9RUT	1117	1.02E+08	88483000	86291000	1.51E+08	2.05E+08	63321000
tr|Q9RX52	tr|Q9RX52	1528	87806000	1.51E+08	77258000	1.55E+08	1.59E+08	1.21E+08
tr|Q9RYQ	tr|Q9RYQ	1788	9555200	2665700	2432900	2597800	4028100	3182000
sp|P5686	sp|P5686	56	1.3E+09	1.53E+09	1.33E+09	2.03E+09	3E+09	2.28E+09
sp|P5686	sp|P5686	56			2637400			4766500
tr|Q9RZA	tr|Q9RZA	1889	57199000	18555000	19766000	39955000	54001000	19136000
tr|Q9RT05	tr|Q9RT05	794	13349000		26613000	29538000	85455000	22265000
tr|Q9RYQ	tr|Q9RYQ	1791						
tr|Q9RSB	tr|Q9RSB	685					12268000	
tr|Q9RUL	tr|Q9RUL	1082	3.92E+08	4.25E+08	2.66E+08	3.52E+08	3.41E+08	3.92E+08
tr|Q9RSC	tr|Q9RSC	689			6202100			
sp|Q9RUV	sp|Q9RUV	257		26055000	62004000		41232000	
sp|Q9RW	sp|Q9RW	318		57175000	59627000	72828000	56923000	62625000
tr|Q9RYW	tr|Q9RYW	1829	17160000		15923000			
tr|Q9RS95	tr|Q9RS95	669						
tr|Q9RTR	tr|Q9RTR	921	3537900		9010200	22982000	24248000	27078000
sp|Q9RY5	sp|Q9RY5	418	26482000	1.75E+08	2.9E+08	42998000	94677000	70480000
sp|Q9RW	sp|Q9RW	345		28046000	48588000	6065500		15723000
tr|Q9RSZ	tr|Q9RSZ	789	80646000	73846000	51630000			
tr|Q9RYV	tr|Q9RYV	1820	53626000	30055000	55949000	1.2E+08	1.27E+08	90739000
sp|Q9RSS	sp|Q9RSS	150		2662900	1033100	4285300	2689300	4609000
sp|Q9RSS	sp|Q9RSS	150					8947700	9479900
tr|Q9RY37	tr|Q9RY37	1690	3.38E+08	3E+08	5.07E+08	2.32E+08	2.5E+08	2.71E+08
sp|Q9RVP	sp|Q9RVP	293	1.39E+08	1.14E+08	1.13E+08	81978000	79762000	90100000
tr|Q9RXN	tr|Q9RXN	1619		12848000	45320000	31908000	35108000	38829000
tr|Q9RYU	tr|Q9RYU	1818	1.31E+08	13406000	1.07E+08	1.04E+08	1.08E+08	
tr|Q9RUA	tr|Q9RUA	1026	1.17E+09	1.28E+09	9.5E+08	1.06E+09	8.5E+08	8.45E+08
tr|Q9RUA	tr|Q9RUA	1026	25695000	1.65E+08	2.21E+08	2.03E+08	2.2E+08	2.13E+08
tr|Q9RTL6	tr|Q9RTL6	897	2.75E+08	3.7E+08	3.35E+08	7.53E+08	6.36E+08	6.59E+08
tr|Q9RT68	tr|Q9RT68	826						7351700
sp|Q9RY3	sp|Q9RY3	413	1.99E+09	1.87E+09	2.32E+09	3.64E+09	3.1E+09	2.91E+09
tr|Q9RXM	tr|Q9RXM	1616						
tr|Q9RVB	tr|Q9RVB	1208					1.65E+08	
sp|Q9RY2	sp|Q9RY2	412				1.31E+08	1.17E+08	18084000
tr|Q9RTX7	tr|Q9RTX7	952	8.87E+08	3.13E+08	3.88E+08	8.62E+08	2.91E+08	9.66E+08
sp|Q9RYE	sp|Q9RYE	433						
tr|Q9RRC	tr|Q9RRC	501	90344000					
tr|Q9RRC	tr|Q9RRC	501			6882200	36400000		
tr|Q9RYH	tr|Q9RYH	1755	21359000	26265000	18820000	37154000	37959000	53342000

sp|Q9RXI	sp|Q9RXI	374	7983900	3930300	3389000	2897900	4543100	3418700
sp|Q9RW	sp|Q9RW	328	8.49E+08	1.06E+09	6.69E+08	1.73E+09	1.42E+09	1.41E+09
sp|Q9RXW	sp|Q9RXW	404	92024000	1.03E+08	91412000	83027000	1.14E+08	1.45E+08
tr|Q9RT28	tr|Q9RT28	805						
sp|Q9RRC	sp|Q9RRC	79		59250000		88045000	74333000	72448000
sp|Q9RRC	sp|Q9RRC	79	1.07E+08	1.26E+08	1.48E+08	94958000	92741000	1.37E+08
sp|Q9RRC	sp|Q9RRC	79					49206000	34456000
tr|Q9RX59	tr|Q9RX59	1534	1.56E+08	1.61E+08	1.11E+08	2.35E+08		1.84E+08
tr|Q9RX59	tr|Q9RX59	1534	5.44E+08	3.17E+08	6.4E+08	1.01E+08	2.15E+08	2.66E+08
tr|Q9RSZ5	tr|Q9RSZ5	790	2.54E+08	3.29E+08	2.8E+08	85349000	1.78E+08	1.88E+08
sp|Q9RXJ9	sp|Q9RXJ9	384	4.85E+08	3.47E+08	3.44E+08	5.14E+08	5.14E+08	4.94E+08
sp|P52027	sp|P52027	51	4.44E+08		3.3E+08	4.31E+08	4.3E+08	4.52E+08
tr|Q9RYY7	tr|Q9RYY7	1837						
tr|Q9RT97	tr|Q9RT97	843	10619000		19852000			
sp|Q9RVI3	sp|Q9RVI3	289	1.58E+08	1.67E+08	1.32E+08	1.12E+08		1.27E+08
tr|Q9RYE9	tr|Q9RYE9	1741	13730000	51736000	11216000		32099000	
sp|Q9RS6	sp|Q9RS6	112	2.9E+08	3.13E+08	2.73E+08	27735000	32254000	39073000
sp|Q9RS6	sp|Q9RS6	112			8006700			
sp|Q9RZ0	sp|Q9RZ0	450	4.43E+08	5.53E+08	5.17E+08	4.06E+08	5.37E+08	5.14E+08
tr|Q9RZH9	tr|Q9RZH9	1933						
tr|Q9RUH	tr|Q9RUH	1057	96095000	1.72E+08	1.62E+08	74587000	1.29E+08	2.5E+08
tr|Q9RSY6	tr|Q9RSY6	784	1.73E+09	2.04E+09	1.63E+09	5.38E+09	4.8E+09	5.02E+09
tr|Q9RSJ0	tr|Q9RSJ0	726	42588000			37159000		40245000
tr|Q9RWU	tr|Q9RWU	1474	3.25E+08	3.04E+08	3.03E+08	2.17E+08	2.09E+08	2.18E+08
tr|Q9RTQ	tr|Q9RTQ	912	1.53E+09	1.28E+09	9.86E+08	8.34E+08	8.56E+08	1E+09
sp|Q9RUS	sp|Q9RUS	250	2.04E+09	2.75E+09	2.37E+09	1.06E+09	2.38E+09	1.83E+09
sp|Q9RUS	sp|Q9RUS	250	73411000	79513000	1.38E+08			
sp|Q9RUS	sp|Q9RUS	250			36230000			
tr|Q9RYP8	tr|Q9RYP8	1786	44888000	60552000	46308000	59698000	58114000	60266000
tr|Q9RSV5	tr|Q9RSV5	771	1.55E+08		1.08E+08			
tr|Q9RWW	tr|Q9RWW	1488	1.25E+08	1.14E+08	1.05E+08	1.51E+08	1.2E+08	1.5E+08
sp|P52027	sp|P52027	51				29649000	18091000	25545000
sp|Q9RSW	sp|Q9RSW	154	1330600					
tr|Q9RTZ8	tr|Q9RTZ8	966	4.51E+08	3.35E+08	5.68E+08	1.31E+08	1.67E+08	2.97E+08
tr|Q9RWP	tr|Q9RWP	1449			21742000			
tr|Q9RWA	tr|Q9RWA	1379			20567000			
tr|Q9RV36	tr|Q9RV36	1169						
tr|Q9RV36	tr|Q9RV36	1169	27719000	35416000	81335000			
tr|Q9RYN4	tr|Q9RYN4	1778	1.5E+08	2.06E+08	2.34E+08	2.25E+08	1.44E+08	1.59E+08
sp|P56867	sp|P56867	56	1.12E+08	1.2E+08	1.55E+08			
tr|Q9RRW	tr|Q9RRW	598	63544000	72410000	97524000	39985000	46842000	52803000
sp|Q9RTQ	sp|Q9RTQ	197	72018000	85003000	62807000			
sp|O83030	sp|O83030	48						
sp|O83030	sp|O83030	48		29811000	33860000		21082000	25188000
tr|Q9RYC5	tr|Q9RYC5	1726	1.06E+08	1.03E+08	91203000	2.86E+08	3.14E+08	2.84E+08
tr|Q9RXM	tr|Q9RXM	1615	39679000	34865000	35012000	3.18E+08	2.45E+08	2.36E+08
sp|Q9RXT	sp|Q9RXT	401	45687000	75447000	1.04E+08	92114000	98336000	1.16E+08
tr|Q9RWP	tr|Q9RWP	1449	4.43E+08	6.71E+08	5.94E+08	6.43E+08	6.31E+08	5.22E+08
tr|Q9RTY1	tr|Q9RTY1	956	3.84E+08	3.17E+08	3.05E+08	4.64E+08	4.21E+08	4.35E+08
tr|Q9RUL1	tr|Q9RUL1	1083	26668000	25000000	25962000	42081000	38921000	34367000
tr|Q9RX53	tr|Q9RX53	1529			41781000	60849000	57703000	59181000

tr|Q9RS1	tr|Q9RS1	626	2.77E+08	3.09E+08	2.65E+08		74981000	9836300
sp|Q9RYR	sp|Q9RYR	442	7296900	30762000			37125000	
tr|Q9RZ0	tr|Q9RZ0	1846						
tr|Q9RXN	tr|Q9RXN	1619		4961600	12365000			
tr|Q9RXZ	tr|Q9RXZ	1669				15689000	17020000	16834000
tr|Q9RWT	tr|Q9RWT	1465	36038000	39227000	88785000	21497000	23678000	22035000
sp|Q9RSR	sp|Q9RSR	144	15723000	21543000	14314000	38323000		
sp|Q9RRJ1	sp|Q9RRJ1	86	6.18E+08	1.32E+09	1.11E+09	1.22E+09	1.58E+09	1.16E+09
sp|Q9RRD	sp|Q9RRD	80		7784700	1.05E+08		1.03E+08	
sp|Q9RTN	sp|Q9RTN	196						
sp|Q9RSF	sp|Q9RSF	117						
tr|Q9RRX1	tr|Q9RRX1	601	1.14E+09	2.75E+09	1.88E+09	1.53E+09	2.07E+09	1.73E+09
tr|Q9RRW	tr|Q9RRW	597	1.38E+08	1.24E+08	8.01E+08	3.94E+08	4.82E+08	4.61E+08
tr|Q9RT3	tr|Q9RT3	811						
tr|Q9RZQ	tr|Q9RZQ	1958						3273700
sp|Q9RR6	sp|Q9RR6	66						
sp|Q9RW	sp|Q9RW	321	1.01E+08	1.03E+08	99580000	1.71E+08	1.5E+08	1.38E+08
tr|Q9RXB	tr|Q9RXB	1565			65311000	1.16E+08	1.19E+08	1.08E+08
tr|Q9RUL	tr|Q9RUL	1087						
tr|Q9RW	tr|Q9RW	1493	11502000	3766200		4362100	4717300	4346700
tr|Q9RW	tr|Q9RW	1493						
sp|Q9RTF	sp|Q9RTF	182				24422000	16780000	19505000
sp|Q9RY	sp|Q9RY	439	1.06E+09	1.26E+09	1.41E+09	3.19E+09	2.46E+09	2.32E+09
tr|Q9RVN	tr|Q9RVN	1264	5.66E+08	6.15E+08	7.37E+08	49143000	1.55E+08	1.27E+08
tr|Q9RSI1	tr|Q9RSI1	721	1.02E+08	1.14E+08		83542000	85731000	70742000
tr|Q9RUU	tr|Q9RUU	1124		4236200	6964100	5339700		
tr|Q9RSZ	tr|Q9RSZ	788	37527000	55745000	52446000	1.12E+08	92637000	86480000
tr|Q9RSZ	tr|Q9RSZ	788	28355000	34806000	35343000			
tr|Q9RUD	tr|Q9RUD	1043	6457700	1427900	1591800	982240	2938000	1559500
tr|Q9RX7	tr|Q9RX7	1545		8114600	14570000		17052000	9358700
tr|Q9RRU	tr|Q9RRU	584			7543600	17355000	15002000	
tr|Q9RXF2	tr|Q9RXF2	1582	38928000	47437000	30915000			
tr|Q9RWT	tr|Q9RWT	1470	36540000	27916000	35088000	64990000	52010000	
tr|Q9RZL7	tr|Q9RZL7	1945	9.01E+08	8.36E+08	8.08E+08	1.17E+09	8.94E+08	1.12E+09
tr|Q9RZV	tr|Q9RZV	1984	5.12E+09	4.75E+09	4.66E+09	3.72E+09	3.74E+09	4.36E+09
tr|Q9RZV	tr|Q9RZV	1984					51966000	64992000
sp|Q9RVY	sp|Q9RVY	303	1.39E+09	1.41E+09	1.15E+09	1.35E+09	1.46E+09	1.42E+09
sp|Q9RVY	sp|Q9RVY	303						
tr|Q9RXD	tr|Q9RXD	1573	1.8E+08	1.62E+08	1.44E+08	87105000	91872000	1.15E+08
tr|Q9RV4	tr|Q9RV4	1172	1.03E+09	1.83E+09	2.06E+09	5.01E+08	8.06E+08	26843000
tr|Q9RV4	tr|Q9RV4	1172	20122000					
tr|Q9RV4	tr|Q9RV4	1172	9.17E+09	1.14E+10	1.16E+10	1.72E+10	1.28E+10	1.54E+10
tr|Q9RZU	tr|Q9RZU	1977	3.04E+08	2.96E+08	2.14E+08	3.08E+08	3.46E+08	3.09E+08
tr|Q9RY4	tr|Q9RY4	1694			6374100	62867000	1.47E+08	1.46E+08
sp|Q9RTH	sp|Q9RTH	186	72878000	91010000	89540000	73735000	81059000	90721000
tr|Q9RSB	tr|Q9RSB	686	1.12E+08	61168000	1.22E+08	60082000	52096000	52937000
tr|Q9RTA	tr|Q9RTA	846	4.73E+08	3.52E+08	3.39E+08	1.46E+08	1.96E+08	1.85E+08
tr|Q9RYT	tr|Q9RYT	1814	27638000	19095000				
tr|Q9RUX	tr|Q9RUX	1137	15827000	6383700	9869500			
tr|Q9RSP	tr|Q9RSP	746	72019000	83026000	28766000	37383000	93692000	1.01E+08
tr|Q9RZR	tr|Q9RZR	1962						

tr|Q9RXC2	tr|Q9RXC2	1568				28100000		
sp|Q9RSN	sp|Q9RSN	138	4.13E+08	4.24E+08	6.64E+08			
tr|Q9RSE8	tr|Q9RSE8	706	19532000	24307000	20603000	81622000	86604000	8346700
tr|Q9RVG	tr|Q9RVG	1229						
tr|Q9RZV2	tr|Q9RZV2	1982						
tr|Q9RUK	tr|Q9RUK	1075			6737300	8309200	9132000	8211200
sp|Q9RSA	sp|Q9RSA	114	9.2E+08	9E+08	7.55E+08	1.49E+09	1.37E+09	1.38E+09
tr|Q9RT35	tr|Q9RT35	811	35109000					1474800
tr|Q9RX29	tr|Q9RX29	1517	92248000	98495000	77704000	81033000	69730000	69847000
tr|Q9RYT2	tr|Q9RYT2	1811			20595000			
tr|Q9RU0	tr|Q9RU0	969	44472000	62598000	25974000			
tr|Q9RZG	tr|Q9RZG	1923	1.13E+08	73045000	88109000	90013000	88413000	90082000
tr|Q9RVR5	tr|Q9RVR5	1284						
tr|Q9RYT8	tr|Q9RYT8	1816						
sp|O3250	sp|O3250	44	30463000	32934000	20975000	59408000	58875000	55360000
tr|Q9RYS1	tr|Q9RYS1	1801		26443000				17246000
tr|Q9RV45	tr|Q9RV45	1172						
sp|Q9RW	sp|Q9RW	342						
tr|Q9RVQ	tr|Q9RVQ	1274	2.1E+08	2.11E+08	2.29E+08	22464000	38757000	
tr|Q9RTX9	tr|Q9RTX9	954					20149000	16875000
tr|Q9RV56	tr|Q9RV56	1180	26745000	25316000	20797000	41229000	56228000	28378000
tr|Q9RY33	tr|Q9RY33	1686	1.39E+08	1.53E+08	1.28E+08	2.91E+08	2.3E+08	2.39E+08
tr|Q9RRV8	tr|Q9RRV8	593				90570000	76267000	1.06E+08
tr|Q9RZ33	tr|Q9RZ33	1861	1.42E+08	1.91E+08	1.26E+08	3.74E+08	2.9E+08	3.49E+08
tr|Q9RZQ	tr|Q9RZQ	1959		82435000	94305000	3.2E+08	3.05E+08	3.13E+08
tr|Q9RZQ	tr|Q9RZQ	1959			31483000			
tr|Q9RTB3	tr|Q9RTB3	847	3.95E+08	4.72E+08	2.95E+08	1.88E+09	82680000	1.28E+09
tr|Q9RVQ	tr|Q9RVQ	1272	47340000	32985000			74124000	71179000
tr|Q9RVQ	tr|Q9RVQ	1272			26484000			21706000
sp|Q9RW2	sp|Q9RW2	307	15824000		12481000	88311000	59287000	54418000
tr|Q9RWY	tr|Q9RWY	1500	18601000					
tr|Q9RUB	tr|Q9RUB	1028	7459100	3939000	4166500	11931000	9486500	9355700
tr|Q9RRM	tr|Q9RRM	548	83975000	1.01E+08	1.19E+08	36440000	50631000	52433000
sp|Q9RRT	sp|Q9RRT	97	19540000	44885000	28514000	31219000	20561000	16650000
tr|Q9RUF6	tr|Q9RUF6	1048						
tr|Q9RUF6	tr|Q9RUF6	1048	10421000		17343000			
tr|Q9RUU	tr|Q9RUU	1123	48798000	55898000	57784000	4.31E+08	3.31E+08	3.15E+08
tr|Q9RUK	tr|Q9RUK	1079	1.63E+08	1.56E+08	1.22E+08	1.93E+08	1.89E+08	1.77E+08
tr|Q9RST7	tr|Q9RST7	760	29219000	41542000	44899000	28071000	47830000	24749000
tr|Q9RV45	tr|Q9RV45	1172	4.66E+08	3.6E+08	3.13E+08	1.86E+08	2.36E+08	1.92E+08
sp|P56867	sp|P56867	56	630250			289490	402640	326770
sp|P56867	sp|P56867	56						
tr|Q9RT09	tr|Q9RT09	798	1.24E+08	1.45E+08	1.29E+08	4.25E+08	3.4E+08	3.58E+08
tr|Q9RU67	tr|Q9RU67	1007						
tr|Q9RUN	tr|Q9RUN	1097	84368000	74857000	89298000	66240000	63721000	73281000
tr|Q9RVS5	tr|Q9RVS5	1290	1.97E+08	4.33E+08	1.67E+08	2.76E+08	2.63E+08	3.04E+08
tr|Q9RVS5	tr|Q9RVS5	1290	79140000	1.07E+08	1.18E+08	31023000	34879000	38777000
tr|Q9RWH	tr|Q9RWH	1409	9810600	4030500	3400200	1944400	4769400	5118500
tr|Q9RYS9	tr|Q9RYS9	1808	27893000	29804000		16852000	17849000	18618000
tr|Q9RRU	tr|Q9RRU	584	1.02E+08	95971000	1.43E+08	1.05E+08	1.22E+08	93958000
sp|Q9RSJ5	sp|Q9RSJ5	123						

tr|Q9RRN	tr|Q9RRN	551						
tr|Q9RVD	tr|Q9RVD	1215	37623000	40982000	43454000		21044000	23923000
tr|Q9RRM	tr|Q9RRM	548	1.4E+08	1.47E+08	1.75E+08	76118000	61053000	1.04E+08
tr|Q9RU7	tr|Q9RU7	1010	3.24E+08	3.63E+08	2.97E+08	1.3E+08	1.44E+08	1.73E+08
tr|Q9RYQ	tr|Q9RYQ	1788	2E+08	2.35E+08	3.16E+08	1.62E+08	2.21E+08	2.05E+08
sp|P5686	sp|P5686	56	2520500	650040	652450		614370	
sp|P5686	sp|P5686	56						
sp|Q9RV5	sp|Q9RV5	265	7547500	2013500	1883800	3193900	4990800	3820300
sp|Q9RV5	sp|Q9RV5	265	12718000		5749700	7898700	4840300	6653400
sp|Q9RV5	sp|Q9RV5	265		14087000	14360000	12168000	17418000	22600000
sp|Q9RV5	sp|Q9RV5	265	1.45E+08	1.64E+08	1.26E+08	2.98E+08	2.8E+08	2.64E+08
tr|Q9RTY1	tr|Q9RTY1	956	25637000	24420000	32858000			
tr|Q9RRB	tr|Q9RRB	495	50757000	79525000	92385000	46159000	55832000	97011000
sp|Q9RWE	sp|Q9RWE	320	69736000	37163000	38761000	58879000	63485000	63137000
tr|Q9RXU	tr|Q9RXU	1645						
sp|Q9RU8	sp|Q9RU8	219	48235000	50946000	38535000	40076000	40014000	44780000
tr|Q9RVP1	tr|Q9RVP1	1267	6462200	9912400		17135000		
tr|Q9RTJ4	tr|Q9RTJ4	884	3.44E+09	4.02E+09	4.85E+09	3.19E+09	3.01E+09	3.19E+09
tr|Q9RYD	tr|Q9RYD	1735	2.65E+08	1.98E+08	1.77E+08	71664000	73395000	77901000
tr|Q9RSK	tr|Q9RSK	730						
tr|Q9RWB	tr|Q9RWB	1385	9.02E+08	8.84E+08	8.95E+08	4.21E+08	3.36E+08	5.11E+08
tr|Q9RTY	tr|Q9RTY	957	2.62E+08	2.88E+08	3.36E+08	1.31E+08	1.73E+08	2.21E+08
tr|Q9RW0	tr|Q9RW0	1332	8.67E+09	7.92E+09	6.67E+09	5.97E+09	4.65E+09	6.49E+09
tr|Q9RS8	tr|Q9RS8	665						
tr|Q9RV6	tr|Q9RV6	1185					22496000	
sp|Q9RXK	sp|Q9RXK	386	1.08E+08	43346000	52220000			
sp|Q9RRB	sp|Q9RRB	76	1.11E+08	2.08E+08	3.68E+08	1.85E+08	1.91E+08	2.3E+08
tr|Q9RS1	tr|Q9RS1	626	59250000	38087000	64466000	20660000	12227000	19452000
tr|Q9RZ07	tr|Q9RZ07	1845			27311000			
tr|Q9RUK	tr|Q9RUK	1080	3.33E+08	4.19E+08	4.26E+08		99527000	1.55E+08
sp|Q9RST	sp|Q9RST	151	3232000	652620	846860	1927200	3010800	1979700
sp|Q9RST	sp|Q9RST	151			5483400	1.4E+08	2.64E+08	1.33E+08
tr|Q9RS41	tr|Q9RS41	641	21720000	24661000	15871000	1.27E+08		
tr|Q9RS31	tr|Q9RS31	637	41006000	65068000	48166000	25667000	21481000	
tr|Q9RWX	tr|Q9RWX	1498	1.33E+08	1.92E+08	1.75E+08	2.47E+08	2.05E+08	2.4E+08
sp|Q9RRC	sp|Q9RRC	79	12352000	5969800	4550000	2505300		
tr|Q9RSY7	tr|Q9RSY7	785	6.51E+08	4.72E+08	5.32E+08	1.2E+08	1.74E+08	2.6E+08
tr|Q9RYN	tr|Q9RYN	1777	2.6E+08	1.54E+08	2.04E+08	1.31E+08		1.29E+08
tr|Q9RV7	tr|Q9RV7	1188	36393000	29779000	26666000			
tr|Q9RY3	tr|Q9RY3	1691	46433000	26988000	23370000	26566000	24841000	31640000
sp|Q9RRQ	sp|Q9RRQ	94						
sp|P5692	sp|P5692	59	1.27E+08	1.21E+08	1.03E+08	2.36E+08	1.88E+08	1.95E+08
sp|P5692	sp|P5692	59	1.83E+08	2.16E+08	2.63E+08		67133000	1.18E+08
tr|Q9RSV	tr|Q9RSV	770	69632000	60074000	82160000	64837000	43198000	53818000
tr|Q9RVZ	tr|Q9RVZ	1327	4.98E+08		7921600	5.74E+08	4.68E+08	5.1E+08
tr|Q9RWY	tr|Q9RWY	1501						
sp|Q9RTJ	sp|Q9RTJ	189	27265000	22443000	1.07E+08	95512000	75286000	1.06E+08
sp|Q9RWE	sp|Q9RWE	321	2.77E+08	1.88E+08	2.6E+08	2.12E+08	2.15E+08	3.45E+08
tr|Q9RWH	tr|Q9RWH	1409	1.27E+09	8.26E+08	1.18E+09	2.04E+09	1.44E+09	1.79E+09
tr|Q9RXP	tr|Q9RXP	1626	25326000		22012000		33692000	34005000
sp|Q9RW	sp|Q9RW	314	6.24E+08	9.23E+08	6.26E+08	7.09E+08	9.29E+08	7.44E+08

tr|Q9RVS2	tr|Q9RVS2	1288	1.45E+09	1.25E+09	1.13E+09	6.63E+08	6.47E+08	9.38E+08
sp|Q9RUV	sp|Q9RUV	257	63378000	1.23E+08	1.2E+08	1.7E+08	1.18E+08	1.4E+08
tr|Q9RV75	tr|Q9RV75	1189		10232000	7353400			
sp|Q9RUV	sp|Q9RUV	258						
tr|Q9RW7	tr|Q9RW7	1364	5.36E+08	3.44E+08	5.34E+08	1.03E+08	1.08E+08	1.11E+08
tr|Q9RW7	tr|Q9RW7	1364	16004000					
tr|Q9RYE9	tr|Q9RYE9	1741	57893000	46053000	35299000	69625000	70399000	51505000
sp|Q9RWH	sp|Q9RWH	329	23831000	9430500	8329700	5386400	7870200	8482100
sp|P52027	sp|P52027	51	4.15E+08	3.96E+08	3.67E+08	3.08E+08	2.73E+08	3.02E+08
tr|Q9RYT6	tr|Q9RYT6	1815	5.07E+08	4.9E+08	3.68E+08	6.19E+08	4.23E+08	5.71E+08
sp|Q9RW(	sp|Q9RW(	305	28694000		37251000	28698000	63443000	27730000
sp|Q9RSJ8	sp|Q9RSJ8	126						
sp|Q9RRJ	sp|Q9RRJ	87					26852000	
tr|Q9RTJ1	tr|Q9RTJ1	883			40895000	16378000		
tr|Q9RXT8	tr|Q9RXT8	1642	4.2E+08	6.12E+08	4.2E+08	8.01E+08	7.15E+08	7.4E+08
sp|Q9RV9	sp|Q9RV9	273					74538000	20070000
tr|Q9RVJ0	tr|Q9RVJ0	1238	9420600	3748100	3957000	1403900	2486600	2403100
tr|Q9RZF3	tr|Q9RZF3	1921						
tr|Q9RRG5	tr|Q9RRG5	521						
tr|Q9RRU1	tr|Q9RRU1	584	3.01E+08	2.59E+08	2.4E+08	3.9E+08	3.76E+08	3.3E+08
tr|Q9RYE1	tr|Q9RYE1	1737			1.3E+08		11055000	
tr|Q9RTX7	tr|Q9RTX7	952	2.38E+08	2.68E+08	1.72E+08			
tr|Q9RXS1	tr|Q9RXS1	1638	24496000			15574000		22104000
tr|Q9RV30	tr|Q9RV30	1166	20457000	28815000	1.97E+08			1.13E+08
tr|Q9RUA	tr|Q9RUA	1026	2.98E+09	4.4E+09	2.81E+09	1.73E+09	1.3E+09	1.67E+09
tr|Q9RTF7	tr|Q9RTF7	868				36098000		
tr|Q9RYL0	tr|Q9RYL0	1767						
tr|Q9RUP1	tr|Q9RUP1	1099						
tr|Q9RRV4	tr|Q9RRV4	591	6.49E+08	4.68E+08	5.79E+08	3.94E+08	4.27E+08	6.58E+08
sp|Q9R34	sp|Q9R34	64				27648000	19931000	
sp|Q9RSJ9	sp|Q9RSJ9	127						9911900
sp|Q9RSJ6	sp|Q9RSJ6	124				23294000	26242000	20362000
tr|Q9RWW	tr|Q9RWW	1488						
tr|Q9RS63	tr|Q9RS63	652	59177000	49776000	64330000	59161000	49224000	53367000
tr|Q9RRC7	tr|Q9RRC7	501				29274000		
tr|Q9RRC7	tr|Q9RRC7	501						
sp|Q9RSJ6	sp|Q9RSJ6	124	29394000	35376000	30947000	3.76E+08	2.84E+08	1.49E+08
tr|Q9RXL6	tr|Q9RXL6	1612	3.47E+08	2.35E+08	1.74E+08	99145000	87892000	88539000
tr|Q9RVL5	tr|Q9RVL5	1252		1.17E+08	1.15E+08	1.19E+08	75852000	
tr|Q9RX16	tr|Q9RX16	1511	3.58E+08	4.55E+08	3.6E+08	2.18E+08	2.29E+08	1.93E+08
tr|Q9RUJ8	tr|Q9RUJ8	1073		14923000				
tr|Q9RWQ	tr|Q9RWQ	1453	63957000	55510000	63342000	55736000	44552000	83732000
tr|Q9RRH	tr|Q9RRH	528	27537000	30969000	33796000	27918000	27310000	24034000
tr|Q9RTD6	tr|Q9RTD6	859	2.13E+08	1.88E+08	1.53E+08		86735000	
tr|Q9RVG9	tr|Q9RVG9	1231		9247100	32559000			
tr|Q9RTV1	tr|Q9RTV1	936	30641000	1.09E+08	79630000	2.13E+08	1.97E+08	1.89E+08
tr|Q9RUA	tr|Q9RUA	1023			38089000			
sp|Q9RT3	sp|Q9RT3	166	3.19E+08	2.85E+08	3.35E+08			14364000
tr|Q9RVW	tr|Q9RVW	1313	31859000		43939000	95797000		48211000
tr|Q9RRX1	tr|Q9RRX1	601	4607700	2309500	2865200		2038300	2218900
tr|Q9RZI6	tr|Q9RZI6	1937				1995500		

tr|Q9RV82	tr|Q9RV82	1191	76521000	61799000	69138000	46117000	31610000	48644000
tr|Q9RXS1	tr|Q9RXS1	1638	8177300	7050600	10704000			
tr|Q9RXX2	tr|Q9RXX2	1660	94395000	1.57E+08	1.19E+08			
tr|Q9RYI1	tr|Q9RYI1	1760	5.47E+08	7.61E+08	6.16E+08	6.12E+08	5.91E+08	5.71E+08
tr|Q9RSG6	tr|Q9RSG6	715	65845000	85753000	46871000	66781000	69437000	50139000
sp|Q9RSS8	sp|Q9RSS8	149	13218000	18866000	11923000		14911000	
sp|Q9RSS8	sp|Q9RSS8	149	1.37E+08	2.55E+08	5.15E+08			
tr|Q9RSD0	tr|Q9RSD0	692	31144000	40412000	54590000	39603000	37293000	29653000
tr|Q9RRX4	tr|Q9RRX4	603						
tr|Q9RV56	tr|Q9RV56	1180	63851000	64549000	52543000			
tr|Q9RSX3	tr|Q9RSX3	778	8063700	3591400	3829400			
tr|Q9RRC7	tr|Q9RRC7	501	1.15E+08	2.94E+08	2.16E+08	1.39E+08	2.05E+08	1.64E+08
tr|Q9RT45	tr|Q9RT45	816	23159000	17512000	18021000			
tr|Q9RZN4	tr|Q9RZN4	1951			27796000			
tr|Q9RTX4	tr|Q9RTX4	949			6403300	16485000	8974400	10319000
sp|Q9RY5	sp|Q9RY5	419	2.24E+09	2.1E+09	1.85E+09	6.04E+08	6.19E+08	6.54E+08
tr|Q9RRG4	tr|Q9RRG4	520	4.93E+09	5.27E+09	3.73E+09	3.44E+09	2.2E+09	2.89E+09
tr|Q9RRG4	tr|Q9RRG4	520						
sp|Q9RSF	sp|Q9RSF	117	57458000	38032000	54751000	30500000	37715000	62059000
tr|Q9RY03	tr|Q9RY03	1676						5248000
sp|Q9RW5	sp|Q9RW5	310				10667000		12462000
tr|Q9RZE4	tr|Q9RZE4	1914	1.02E+09	9.4E+08	8.34E+08	7.23E+08	6.16E+08	1.59E+08
tr|Q9RZE4	tr|Q9RZE4	1914	54577000	71837000	1.17E+08	55158000		
tr|Q9RR82	tr|Q9RR82	474	14261000	46648000	27447000	1.41E+08	87859000	1.29E+08
tr|Q9RVU	tr|Q9RVU	1300		25313000	22105000		29499000	
tr|Q9RX02	tr|Q9RX02	1505	47810000	49406000		1.13E+08	60462000	1.1E+08
tr|Q9RZQ	tr|Q9RZQ	1959	18249000	27558000	71403000	90390000	1.01E+08	2.41E+08
tr|Q9RSD1	tr|Q9RSD1	693						
tr|Q9RV13	tr|Q9RV13	1157	74839000	87422000	22931000			49856000
tr|Q9RT05	tr|Q9RT05	794	31284000	66319000	71383000			
tr|Q9RYZ6	tr|Q9RYZ6	1840						
sp|Q9RTN	sp|Q9RTN	195			56794000		22738000	
tr|Q9RS33	tr|Q9RS33	639	1.14E+08	99979000	1.01E+08	1.48E+08	1.15E+08	1.5E+08
tr|Q9RS33	tr|Q9RS33	639	3.6E+08	4.21E+08	3.58E+08	2.24E+08	1.89E+08	2.54E+08
tr|Q9RY79	tr|Q9RY79	1708	2.43E+08			3.17E+08		2.98E+08
tr|Q9RY79	tr|Q9RY79	1708						27236000
sp|Q9RYD	sp|Q9RYD	431	27483000	27571000	31723000	24106000	44063000	21875000
tr|Q9RX36	tr|Q9RX36	1520	25285000	32192000			16777000	
tr|Q9RWX	tr|Q9RWX	1498	1.2E+08	1.15E+08	1.24E+08			
tr|Q9RU76	tr|Q9RU76	1012	69058000	1.52E+08	1.35E+08	1.13E+08	82065000	1.09E+08
sp|P8358	sp|P8358	60	1476900	284580				
tr|Q9RZD7	tr|Q9RZD7	1911						
tr|Q9RYS6	tr|Q9RYS6	1805						
tr|Q9RZC1	tr|Q9RZC1	1901		59313000		56811000	56009000	41335000
tr|Q9RUB	tr|Q9RUB	1030						
tr|Q9RV59	tr|Q9RV59	1182	71651000	82986000		1.06E+08	1.43E+08	1.19E+08
tr|Q9RY22	tr|Q9RY22	1683						
tr|Q9RVG6	tr|Q9RVG6	1228	49341000	1.27E+08	3.24E+08	3.28E+08	4.49E+08	3.65E+08
tr|Q9RYS3	tr|Q9RYS3	1803					26615000	
tr|Q9RYS3	tr|Q9RYS3	1803			13399000		13372000	12314000
tr|Q9RR96	tr|Q9RR96	480	2.63E+08	1.74E+08	1.6E+08	96462000	1.13E+08	98185000

tr|Q9RXP2	tr|Q9RXP2	1623	1.82E+08	1.96E+08	1.85E+08			
tr|Q9RV11	tr|Q9RV11	1156						
tr|Q9RV11	tr|Q9RV11	1156			11669000			14652000
tr|Q9RUZ9	tr|Q9RUZ9	1150	1.04E+08	74140000	76668000			
tr|Q9RRN	tr|Q9RRN	551						
tr|Q9RWP	tr|Q9RWP	1449	19726000					
tr|Q9RSE2	tr|Q9RSE2	701	16639000		42024000			20863000
sp|Q9RZ0	sp|Q9RZ0	451				8284700	17671000	
tr|Q9RT96	tr|Q9RT96	842	83800000		16300000	34568000	14044000	
tr|Q9RTQ	tr|Q9RTQ	917	2084700			1697900	3306100	
tr|Q9RRB1	tr|Q9RRB1	492				85706000	31268000	
tr|Q9RTJ1	tr|Q9RTJ1	883			14082000			
tr|Q9RUJ3	tr|Q9RUJ3	1069						11716000
sp|Q9RV2	sp|Q9RV2	264	66890000	29090000	48207000	51714000	40445000	
sp|Q9RSL	sp|Q9RSL	134	14516000	865950	1169700	2130800	4481100	663830
sp|Q9RSL	sp|Q9RSL	134	6948000	3003800	2503900	1589900	3539100	3916100
tr|Q9RVZ9	tr|Q9RVZ9	1330	73219000	73442000	69885000	40411000		
sp|Q9RUV	sp|Q9RUV	257				22136000		17382000
tr|Q9RSA5	tr|Q9RSA5	676	63511000	1.21E+08	52773000	2.37E+08	2.46E+08	3.1E+08
sp|Q9RWU	sp|Q9RWU	347	1.15E+08	1.17E+08	1.56E+08	52421000	56864000	77623000
tr|Q9RSD6	tr|Q9RSD6	697	61397000	24002000	21071000	21545000	25184000	22057000
tr|Q9RSD6	tr|Q9RSD6	697	31919000	22946000				
sp|Q9RXL	sp|Q9RXL	392	2.8E+08	2.39E+08	2.17E+08	4.37E+08	3.16E+08	3E+08
tr|Q9RT01	tr|Q9RT01	792						
tr|Q9RRV8	tr|Q9RRV8	593	35491000	17505000	14523000	8871200	14299000	11448000
tr|Q9RTX7	tr|Q9RTX7	952			1.49E+08			
tr|Q9RTN8	tr|Q9RTN8	905				1632100	2228400	
tr|Q9RUF8	tr|Q9RUF8	1049	17809000	10083000	10525000	8182400	8164300	11945000
tr|Q9RRA0	tr|Q9RRA0	483	24982000	40298000		2.2E+08	1.96E+08	1.8E+08
tr|Q9RVW	tr|Q9RVW	1313						
tr|Q9RX10	tr|Q9RX10	1507	77856000	60379000	1.01E+08	25116000	26591000	29117000
tr|Q9RRR3	tr|Q9RRR3	568						
tr|Q9RUL4	tr|Q9RUL4	1085	44314000	81160000	1.02E+08	1.83E+08	1.52E+08	1.24E+08
sp|Q9RT2	sp|Q9RT2	162	2.76E+09	2.89E+09	1.17E+09	3.27E+09	3.3E+09	3.12E+09
tr|Q9RSX6	tr|Q9RSX6	781						
sp|Q9RTN	sp|Q9RTN	196	65807000	2.01E+08	1.94E+08	2.16E+08	4.21E+08	2.04E+08
tr|Q9RTR4	tr|Q9RTR4	923						
tr|Q9RW5	tr|Q9RW5	1357	47216000	4.94E+08	4.98E+08	34826000	30964000	6.36E+08
tr|Q9RWJ5	tr|Q9RWJ5	1422	2.19E+08		1E+08			
tr|Q9RU43	tr|Q9RU43	990					39358000	74952000
tr|Q9RU43	tr|Q9RU43	990			30696000			
tr|Q9RSQ4	tr|Q9RSQ4	748	8.58E+08	1.19E+09	4.24E+09			
sp|Q9RVB	sp|Q9RVB	275			2356400	7358600	16850000	13927000
tr|Q9RSY9	tr|Q9RSY9	787	5854900					
tr|Q9RSR9	tr|Q9RSR9	754			52721000			
tr|Q9RWD	tr|Q9RWD	1394			12468000			
tr|Q9RZJ1	tr|Q9RZJ1	1938			3476900	4228600	5774500	
tr|Q9RYL2	tr|Q9RYL2	1769	1.93E+08	1.8E+08	1.72E+08	35648000		
tr|Q9RXH4	tr|Q9RXH4	1596	29537000	31174000	25698000	36570000	41544000	31883000
sp|Q9RXT	sp|Q9RXT	401		1449900	3634800			
tr|Q9RZ85	tr|Q9RZ85	1877	3.18E+08	3.47E+08	3.51E+08	5.25E+08	3.94E+08	4.45E+08

tr|Q9RZ85	tr|Q9RZ85	1877			54343000	32555000		20261000
tr|Q9RYQ3	tr|Q9RYQ3	1791	47403000		60106000	1.91E+08	1.66E+08	1.24E+08
tr|Q9RXF0	tr|Q9RXF0	1581	82652000	43122000	2.95E+08	1.08E+08	87729000	1.1E+08
tr|Q9RT97	tr|Q9RT97	843	22010000	27776000	31593000			36187000
tr|Q9RUB	tr|Q9RUB	1028	4063400	2124800	1825200	3046500	4029200	3129800
tr|Q9RUB	tr|Q9RUB	1028	2.24E+08	2.62E+08	2.69E+08	1.32E+08	89339000	1.34E+08
sp|Q9RVP	sp|Q9RVP	293			22195000	56660000	51021000	58777000
tr|Q9RS86	tr|Q9RS86	662						
tr|Q9RWP	tr|Q9RWP	1446	43004000	1.42E+08	1.13E+08	1.71E+08		15689000
tr|Q9RT53	tr|Q9RT53	821	1.56E+08	1.81E+08	1.39E+08	84876000	92423000	88807000
tr|Q9RYG5	tr|Q9RYG5	1750						
tr|Q9RX83	tr|Q9RX83	1548	1.23E+08	83916000	93297000	1.09E+08	90614000	1.09E+08
tr|Q9RUM	tr|Q9RUM	1091	1.16E+08	1.2E+08	95640000	92072000	1.01E+08	81511000
tr|Q9RRR3	tr|Q9RRR3	568						
tr|Q9RT09	tr|Q9RT09	798	24176000	17372000	16546000	11000000	12454000	17454000
sp|Q9RRY	sp|Q9RRY	103	33691000	40669000	79769000	1.17E+08	1.07E+08	1.1E+08
tr|Q9RXG(	tr|Q9RXG(	1587	32160000	33459000	39829000	37031000	47592000	51683000
tr|Q9RUC	tr|Q9RUC	1033	1.44E+08	1.32E+08	1.34E+08	1.71E+08	1.45E+08	1.73E+08
tr|Q9RR95	tr|Q9RR95	479	2.11E+08	1.4E+08	1.61E+08		81132000	
sp|Q9RZN	sp|Q9RZN	457			3672700	13735000	17554000	22867000
sp|Q9RUI	sp|Q9RUI	237				88518000	28026000	24659000
tr|Q9RUR4	tr|Q9RUR4	1107	3.54E+08	3.81E+08	4.54E+08	1.69E+08	1.89E+08	2.93E+08
sp|Q9RY7	sp|Q9RY7	423	3.96E+08	4.95E+08	4.25E+08	1.91E+08	1.99E+08	2.02E+08
tr|Q9RYN4	tr|Q9RYN4	1778	38978000	39590000	54629000			
tr|Q9RXF0	tr|Q9RXF0	1581	6017100	1383300	1076600	363850	2787800	
sp|Q9RYR	sp|Q9RYR	441		18597000				
tr|Q9RU07	tr|Q9RU07	972				2.62E+08	2.69E+08	2.38E+08
tr|Q9RRN	tr|Q9RRN	552	61682000	85842000	69806000	1.77E+08	1.29E+08	1.52E+08
tr|Q9RUR1	tr|Q9RUR1	1109	4075000		726340	1040200	852280	
tr|Q9RZS7	tr|Q9RZS7	1967	87397000	82771000	1.18E+08	35806000	66800000	56644000
tr|Q9RS08	tr|Q9RS08	624	1.15E+08	1.04E+08	1.29E+08			
tr|Q9RS49	tr|Q9RS49	644	7.21E+08	5.67E+08	7.31E+08	73138000		11855000
tr|Q9RZG9	tr|Q9RZG9	1926						
tr|Q9RVU(	tr|Q9RVU(	1299	26085000	34026000	55902000	23475000	5783500	15531000
tr|Q9RU65	tr|Q9RU65	1006	31909000	27238000	20382000			10413000
sp|Q9RU6	sp|Q9RU6	212	2.32E+08	2.61E+08	1.61E+08	24077000	22256000	20737000
tr|Q9RVF7	tr|Q9RVF7	1224			61338000	20423000	72612000	84061000
tr|Q9RVP(	tr|Q9RVP(	1266	1.88E+08	1.68E+08	2.22E+08	1.05E+08	1.18E+08	1.5E+08
tr|Q9RTD6	tr|Q9RTD6	859	7732900	5856800	4660700	4171400	4960700	5471700
sp|Q9RYD	sp|Q9RYD	430						
tr|Q9RRW	tr|Q9RRW	599	18547000	38769000	40145000	1.48E+08	1.83E+08	89246000
tr|Q9RRU(	tr|Q9RRU(	583	12268000	9802000	5742300	13671000	9786700	10492000
tr|Q9RUK9	tr|Q9RUK9	1081	2.08E+08	1.66E+08	1.34E+08	3.49E+08	2.85E+08	3.81E+08
tr|Q9RUK9	tr|Q9RUK9	1081				1.56E+09		1.27E+09
tr|Q9RYW	tr|Q9RYW	1830	64198000		54014000	85643000		65991000
tr|Q9RUR9	tr|Q9RUR9	1110	27074000	16557000	51123000			
tr|Q9RY57	tr|Q9RY57	1699	1.4E+08	1.38E+08	1.22E+08	1.74E+08	1.62E+08	2.03E+08
sp|Q9RY4	sp|Q9RY4	416	5111000	2680700				
sp|Q9RXI7	sp|Q9RXI7	373	74606000					
sp|Q9RXI7	sp|Q9RXI7	373			23084000			
sp|Q9RYB	sp|Q9RYB	426			19384000			

tr|Q9RSY4	tr|Q9RSY4	783						
sp|Q9RSQ	sp|Q9RSQ	140	55640000	49303000	44691000	1.26E+08	1.39E+08	1.39E+08
sp|Q9RYH	sp|Q9RYH	435	32620000	37850000	26256000	1.75E+08	59436000	85338000
sp|Q9RTH	sp|Q9RTH	186	8354100	7565600				
tr|Q9RRX6	tr|Q9RRX6	604					23299000	19236000
tr|Q9RT74	tr|Q9RT74	830		38651000	37533000		20294000	13933000
tr|Q9RXH3	tr|Q9RXH3	1595			22869000			
tr|Q9RZQ	tr|Q9RZQ	1958	82252000	1.02E+08	2.26E+08	70995000	52012000	
tr|Q9RRY2	tr|Q9RRY2	605			56263000			21412000
tr|Q9RRA3	tr|Q9RRA3	486			2841700	3082700		2972200
tr|Q9RRA3	tr|Q9RRA3	486						
sp|Q9RTT	sp|Q9RTT	200	8073800			20482000	13355000	23976000
tr|Q9RSV5	tr|Q9RSV5	771			38650000			
tr|Q9RZQ	tr|Q9RZQ	1958	29119000	12260000	14699000			
sp|Q9RW(	sp|Q9RW(	342	47727000	44717000	65338000		57457000	97952000
sp|Q9RW(	sp|Q9RW(	342			2861500	1.76E+08	1.93E+08	48554000
sp|Q9RSY	sp|Q9RSY	156	1.2E+09	1.65E+09	1.27E+09	1.78E+09	2.07E+09	1.44E+09
sp|Q9RSY	sp|Q9RSY	156	5.11E+09	8.94E+09	1.27E+10	1.98E+09	4.12E+09	3.5E+09
tr|Q9RRH	tr|Q9RRH	525			41719000	2.26E+08	2.43E+08	1.81E+08
sp|Q9RUH	sp|Q9RUH	236	13331000		14713000	18268000	19023000	
tr|Q9RY98	tr|Q9RY98	1717	23682000	14601000	20492000	29943000	21137000	27322000
tr|Q9RVM	tr|Q9RVM	1255	65048000	61471000	33045000	82178000	97384000	87470000
tr|Q9RVM	tr|Q9RVM	1255	56509000		61954000			62954000
tr|Q9RXY6	tr|Q9RXY6	1665	1.74E+08	1.61E+08	1.52E+08	96004000	1.04E+08	1.22E+08
tr|Q9RSU(	tr|Q9RSU(	762	6.67E+08	6.36E+08	6.37E+08	8.73E+08	8.23E+08	8.17E+08
tr|Q9RVA2	tr|Q9RVA2	1199						16255000
tr|Q9RVA2	tr|Q9RVA2	1199						
tr|Q9RSH(	tr|Q9RSH(	716			34817000	21855000	20896000	
sp|Q9RUY	sp|Q9RUY	259						16628000
sp|Q9RRJ	sp|Q9RRJ	87	4.58E+08	4.99E+08	3.21E+08	4E+08	3.78E+08	4.06E+08
tr|Q9RS60	tr|Q9RS60	651	31558000	55513000	24248000		16162000	11660000
tr|Q9RXX3	tr|Q9RXX3	1661	49655000	52360000	56300000			
tr|Q9RVL2	tr|Q9RVL2	1251						
tr|Q9RU62	tr|Q9RU62	1004	29116000	28370000	22536000	57100000	44542000	48742000
tr|Q9RT45	tr|Q9RT45	816		1.02E+08	46451000			
tr|Q9RVA5	tr|Q9RVA5	1202	1.11E+08	1.01E+08	1.47E+08			
sp|Q9RSK	sp|Q9RSK	131	2.69E+09	1.83E+09	2.37E+09	9.35E+08	8.43E+08	1.17E+09
tr|Q9RSU4	tr|Q9RSU4	765	1.23E+08	1.03E+08	1.18E+08			47876000
tr|Q9RXF0	tr|Q9RXF0	1581				58243000	1.17E+08	55093000
sp|Q9RU2	sp|Q9RU2	208						
sp|Q9RUR	sp|Q9RUR	249			1.03E+08	86063000	1.79E+08	1.22E+08
sp|Q9RUR	sp|Q9RUR	249			93537000	3.97E+08	3.28E+08	3.06E+08
sp|Q9RX6	sp|Q9RX6	361	36297000	45531000	50935000	21972000	34166000	44374000
sp|Q9RX6	sp|Q9RX6	361	40556000	57598000	89196000		66695000	82210000
tr|Q9RTH3	tr|Q9RTH3	876	82944000	1.05E+08	64544000	71822000	79382000	71748000
tr|Q9RVL6	tr|Q9RVL6	1253	2.26E+08	2.41E+08	2.58E+08	3.27E+08	2.7E+08	2.38E+08
tr|Q9RUX5	tr|Q9RUX5	1134	86253000	3.11E+08	3.04E+08	1.84E+08	1.08E+08	1.75E+08
tr|Q9RRC5	tr|Q9RRC5	500	86830000	74146000	1.08E+08	29660000	33012000	40885000
tr|Q9RY73	tr|Q9RY73	1703	1.08E+08	1.21E+08	69386000	1.86E+08	1.87E+08	1.83E+08
tr|Q9RRX1	tr|Q9RRX1	601	77357000	88080000	80136000	64083000	50687000	60509000
tr|Q9RS85	tr|Q9RS85	661			20288000		36302000	27134000

tr|Q9RVW	tr|Q9RVW	1312	62734000					38951000
sp|Q9RUG	sp|Q9RUG	234	43339000	70087000	66818000	25970000		21019000
sp|Q9RZV	sp|Q9RZV	460	26476000	22695000				
tr|Q9RWF	tr|Q9RWF	1403						
tr|Q9RRH	tr|Q9RRH	527	85805000	84209000	53782000	81379000	69359000	82366000
tr|Q9RW5	tr|Q9RW5	1357			10967000	51016000	74308000	81445000
tr|Q9RSA5	tr|Q9RSA5	676	1.92E+10	1.54E+10	1.52E+10	7.97E+09	8.54E+09	7.87E+09
tr|Q9RSA2	tr|Q9RSA2	673	1.01E+09	1.02E+09	7.83E+08	6E+08	6.15E+08	5.44E+08
tr|Q9RUU	tr|Q9RUU	1122	2.62E+09	1.46E+09	2.08E+09	4.54E+08	2.62E+08	3.94E+08
sp|Q9RRJ5	sp|Q9RRJ5	88	39872000	48194000	44286000			
sp|Q9RTN	sp|Q9RTN	196	1.79E+09	1.49E+09	1.32E+09	7.76E+08	8.3E+08	7.67E+08
tr|Q9RZD6	tr|Q9RZD6	1910	17414000	9.28E+08	5.17E+08	6.26E+08	5.72E+08	7.84E+08
tr|Q9RXL6	tr|Q9RXL6	1612		15823000	17720000			
tr|Q9RVS0	tr|Q9RVS0	1286	93541000	69909000	78135000			
tr|Q9RSY7	tr|Q9RSY7	785	1.06E+09	8.17E+08	7.02E+08	6.8E+08	6.14E+08	6.44E+08
tr|Q9RY25	tr|Q9RY25	1684	2.46E+08	2.39E+08	2.25E+08	90143000	77240000	1.14E+08
tr|Q9RSE5	tr|Q9RSE5	704	8.01E+08	9.48E+08	9.31E+08	3.79E+08	2.8E+08	4.9E+08
tr|Q9RTX6	tr|Q9RTX6	951			1.44E+08		40624000	63631000
tr|Q9RUY4	tr|Q9RUY4	1140		8276100	21017000	10563000	16555000	17739000
tr|Q9RRH	tr|Q9RRH	529	4.95E+08	4.58E+08	4.38E+08	7.09E+08	83368000	79672000
tr|Q9RRH	tr|Q9RRH	529		72158000	3.59E+08	2.05E+08	2.18E+08	2.13E+08
sp|Q9RX1	sp|Q9RX1	351						34707000
sp|Q9RX1	sp|Q9RX1	351						
tr|Q9RRH	tr|Q9RRH	526				13304000		
tr|Q9RUX2	tr|Q9RUX2	1131						
tr|Q9RX66	tr|Q9RX66	1539						
tr|Q9RUN	tr|Q9RUN	1094	57832000	66659000	60325000	1.02E+08	1.21E+08	1.13E+08
tr|Q9RY43	tr|Q9RY43	1693			20435000		21925000	
tr|Q9RXR1	tr|Q9RXR1	1632				35094000		3154800
tr|Q9RUC	tr|Q9RUC	1037				28331000		25276000
tr|Q9RV74	tr|Q9RV74	1188	1265300	629340				
tr|Q9RW2	tr|Q9RW2	1345	36921000	31690000	26212000	42015000		33616000
tr|Q9RY55	tr|Q9RY55	1697	16383000	24657000				
sp|Q9RTH	sp|Q9RTH	186						
tr|Q9RZA7	tr|Q9RZA7	1892		26235000	24483000	28321000	34212000	38428000
sp|Q9RS8	sp|Q9RS8	113			39234000		76986000	
tr|Q9RUX8	tr|Q9RUX8	1136	4.66E+08	5.34E+08	4.65E+08	4.43E+08	4.05E+08	5.29E+08
sp|Q9RSR	sp|Q9RSR	143	1.46E+09	1.73E+09	1.71E+09	1.7E+09	2E+09	1.95E+09
sp|Q9RVY	sp|Q9RVY	303	12525000	4000000	3348600	4680600	6374100	3731800
sp|Q9RVY	sp|Q9RVY	303	3.06E+08	3.26E+08	6.4E+08	2.1E+08	2.21E+08	3.68E+08
tr|Q9RS08	tr|Q9RS08	624		89431000	54968000		12279000	
tr|Q9RY43	tr|Q9RY43	1693						
tr|Q9RX72	tr|Q9RX72	1542						
tr|Q9RYD1	tr|Q9RYD1	1731	17650000	17621000	17921000	10418000		11641000
sp|Q9RYD	sp|Q9RYD	431						
sp|Q9RW5	sp|Q9RW5	310	42811000		55191000			
sp|P50933	sp|P50933	50	7846200					
tr|Q9RTH0	tr|Q9RTH0	875	1.26E+08	3.31E+08	2.66E+08	5.35E+08	4.31E+08	
tr|Q9RRJ7	tr|Q9RRJ7	537	39316000	29428000	70109000	55223000	63780000	35336000
tr|Q9RYC0	tr|Q9RYC0	1723	23822000	13286000	12486000	20828000	21071000	33809000
tr|Q9RTQ1	tr|Q9RTQ1	913	41700000	68863000	70286000	20756000	17157000	

tr|Q9RVS1	tr|Q9RVS1	1287	10550000	9250000	6751700		52122000	7472200
sp|P56867	sp|P56867	56	33806000	39935000	36479000	1733600	24281000	1903400
sp|P56867	sp|P56867	56		22769000	30589000			
tr|Q9RU28	tr|Q9RU28	981	37878000	28196000	26361000			
tr|Q9RYT6	tr|Q9RYT6	1815				27707000	36416000	
tr|Q9RXA0	tr|Q9RXA0	1555		8597400	4740200			
tr|Q9RY79	tr|Q9RY79	1708	54133000	49138000	42855000		67471000	
sp|Q9RV2	sp|Q9RV2	264					10888000	
sp|Q9RUE	sp|Q9RUE	229	8130700	9246500	36392000			
tr|Q9RRN	tr|Q9RRN	552			21457000	53565000	19940000	24075000
tr|Q9RZB3	tr|Q9RZB3	1896		1.11E+08	88740000	1.88E+08	2.27E+08	1.76E+08
sp|Q9RYU	sp|Q9RYU	444						
tr|Q9RVB8	tr|Q9RVB8	1210						
tr|Q9RU76	tr|Q9RU76	1012	34333000		65025000			
sp|Q9RZ0	sp|Q9RZ0	452	10866000	47980000	75455000	23858000	38123000	
sp|Q9RYU	sp|Q9RYU	444	17611000	21232000	14728000	36982000	37618000	32917000
sp|Q9RRJ	sp|Q9RRJ	87		1.36E+08			1.64E+08	91348000
sp|Q9RUE	sp|Q9RUE	229	4.24E+08	7.81E+08	7.81E+08	1.48E+09	7.64E+08	1.1E+09
sp|Q9RUE	sp|Q9RUE	229	1.18E+08	33427000	68668000			
tr|Q9RRN	tr|Q9RRN	554			12867000		14010000	16270000
tr|Q9RSU0	tr|Q9RSU0	762	25041000	15831000	13214000	9211600	11721000	11713000
tr|Q9RRP8	tr|Q9RRP8	560						
tr|Q9RWD	tr|Q9RWD	1395	7601000	6532700	9250100			
sp|Q9RTA	sp|Q9RTA	175			62712000		15862000	
tr|Q9RVF3	tr|Q9RVF3	1222	43582000	44207000	34935000	51540000	35858000	37019000
tr|Q9RZQ	tr|Q9RZQ	1958	1.94E+08	1.35E+08	1.57E+08	3.67E+08	2E+08	2.68E+08
tr|Q9RYE1	tr|Q9RYE1	1737						
sp|Q9RS3	sp|Q9RS3	109	24673000	1.26E+08	83584000	86237000	88346000	1.16E+08
sp|Q9RRX	sp|Q9RRX	101		18955000				
tr|Q9RTQ	tr|Q9RTQ	917	1773600		2223500	5218900	5121100	4014900
tr|Q9RVZ2	tr|Q9RVZ2	1325						
tr|Q9RSA5	tr|Q9RSA5	676		1832700	4398300		2064700	
tr|Q9RSU0	tr|Q9RSU0	762	1.06E+09	1.08E+09	8.54E+08	8.38E+08	8.76E+08	9.24E+08
tr|Q9RZT0	tr|Q9RZT0	1968	83828000	78253000	83777000			
tr|Q9RUD	tr|Q9RUD	1039	8034700	21693000	32217000	23240000		15200000
tr|Q9RUP1	tr|Q9RUP1	1099	2.79E+09	2.86E+09	2.68E+09	3.42E+09	2.64E+09	2.55E+09
tr|Q9RWV	tr|Q9RWV	1487	7163400				14064000	
tr|Q9RYE9	tr|Q9RYE9	1741						
sp|Q9RUN	sp|Q9RUN	242	6912600					
tr|Q9RZK3	tr|Q9RZK3	1941						
tr|Q9RY76	tr|Q9RY76	1706						
tr|Q9RUA	tr|Q9RUA	1022			18224000			
sp|Q9RUR	sp|Q9RUR	249	6.31E+09	5.27E+09	4.51E+09	5.05E+09	4.37E+09	5.35E+09
sp|Q9RUR	sp|Q9RUR	249	1.29E+09	3.99E+08	2.94E+09	1.82E+09	2.28E+09	7.62E+08
sp|Q9RUP	sp|Q9RUP	246			17038000	23888000	21855000	
tr|Q9RY75	tr|Q9RY75	1705	59128000		93856000			
sp|Q9RXT	sp|Q9RXT	398	66372000	73606000	72946000	28750000	32233000	36555000
tr|Q9RTZ9	tr|Q9RTZ9	967	12040000					
tr|Q9RXB8	tr|Q9RXB8	1566	99236000	72253000	66556000	23608000	17047000	14799000
sp|Q9RT2	sp|Q9RT2	163	77044000	70113000	1.5E+08	52332000	70108000	79763000
sp|Q9RW	sp|Q9RW	349	30696000	18742000	19970000	59992000	66787000	54527000

tr|Q9RTZ9	tr|Q9RTZ9	967	6.18E+08	4.27E+08	3.88E+08	3.9E+08	4.08E+08	3.38E+08
sp|Q9RT9	sp|Q9RT9	172	1.09E+08	1.71E+08	1.3E+08	74759000	68185000	87997000
tr|Q9RT36	tr|Q9RT36	812	30874000	30174000	31864000			
sp|Q9RSK	sp|Q9RSK	131				52635000	66089000	39592000
sp|Q9RUG	sp|Q9RUG	233						
tr|Q9RVQ	tr|Q9RVQ	1273	5.17E+08	6.22E+08	8.44E+08	3.58E+08	3.82E+08	4.6E+08
tr|Q9RTE1	tr|Q9RTE1	860	1.23E+08	86245000	73291000	1.13E+08	1.15E+08	1.13E+08
tr|Q9RRW	tr|Q9RRW	594	76941000	55293000	47865000	62196000	69309000	73426000
tr|Q9RXC2	tr|Q9RXC2	1568						36834000
sp|Q9RXU	sp|Q9RXU	402						
tr|Q9RXU3	tr|Q9RXU3	1646			68753000			9306600
tr|Q9RW9	tr|Q9RW9	1373	6636000	30200000		45042000	30795000	31652000
tr|Q9RRP4	tr|Q9RRP4	558	27499000	20115000	23783000	30173000	23440000	32579000
tr|Q9RRP4	tr|Q9RRP4	558	23504000	75785000	1.18E+08	29973000	61329000	62032000
tr|Q9RU02	tr|Q9RU02	970				9027400	7429400	
tr|Q9RV41	tr|Q9RV41	1171		6950500		22761000	31335000	30277000
tr|Q9RUG	tr|Q9RUG	1052	7.35E+08	7.25E+08	6.26E+08	9.23E+08	8.88E+08	8.07E+08
tr|Q9RUG	tr|Q9RUG	1052	1.54E+08	1.31E+08	2.07E+08	62572000	71937000	62400000
tr|Q9RVZ7	tr|Q9RVZ7	1329	13205000	19842000		32183000	19085000	22016000
sp|Q9RV9	sp|Q9RV9	271	5412500		6815200	63009000	22185000	26829000
tr|Q9RXE7	tr|Q9RXE7	1578	20633000		17293000		45159000	40646000
tr|Q9RZ24	tr|Q9RZ24	1856				52511000		45257000
tr|Q9RUX5	tr|Q9RUX5	1134			1.82E+08			
tr|Q9RUA	tr|Q9RUA	1021						
sp|Q9RV2	sp|Q9RV2	264	97155000	95822000	1.23E+08	1.31E+08	1.06E+08	1.17E+08
tr|Q9RT82	tr|Q9RT82	836	34516000	50646000	22547000	66053000	96323000	42486000
tr|Q9RY79	tr|Q9RY79	1708	22416000	65158000	23166000	1.62E+08	1.61E+08	1.47E+08
sp|Q9RUP	sp|Q9RUP	243	69964000	35877000	33486000	27474000	29098000	37453000
tr|Q9RZ28	tr|Q9RZ28	1858						
sp|Q9RUG	sp|Q9RUG	233	1.66E+08	1.76E+08	1.64E+08	1.27E+08	95232000	
tr|Q9RWQ	tr|Q9RWQ	1453	4.26E+08	4.51E+08	3.69E+08	2.72E+08	2.94E+08	2.81E+08
sp|Q9RYM	sp|Q9RYM	438				4911100	7858300	
sp|Q9RY6	sp|Q9RY6	421						
sp|Q9RT2	sp|Q9RT2	162	80646000	49259000	70580000		17445000	
tr|Q9RRZ5	tr|Q9RRZ5	614						
sp|Q9RS1	sp|Q9RS1	107	82864000	40541000		10924000		
tr|Q9RXF0	tr|Q9RXF0	1581	8.68E+08	3.49E+08	9.24E+08	1.31E+09	5.92E+08	1.06E+09
tr|Q9RY30	tr|Q9RY30	1685	4.11E+08	3.43E+08	4.3E+08			
tr|Q9RWQ	tr|Q9RWQ	1454	82066000	98617000	62472000	1.66E+08	1.12E+08	1.32E+08
tr|Q9RW8	tr|Q9RW8	1368	1.32E+08	91127000	1.01E+08	93905000	92283000	1.05E+08
tr|Q9RW8	tr|Q9RW8	1368			25627000	4942600	17377000	11201000
tr|Q9RRK3	tr|Q9RRK3	540						
tr|Q9RXD5	tr|Q9RXD5	1573	2.6E+08	3.24E+08	2.74E+08			
tr|Q9RUG	tr|Q9RUG	1051	1.43E+09	1.15E+09	1.17E+09	5.6E+08	5.93E+08	5.14E+08
sp|O3250	sp|O3250	45						
sp|Q9RSZ	sp|Q9RSZ	159	41020000	49845000	42314000	57846000		56299000
sp|Q9RSZ	sp|Q9RSZ	159						
tr|Q9RZK3	tr|Q9RZK3	1941						
tr|Q9RY03	tr|Q9RY03	1676	36712000	17893000				
sp|Q9RVE	sp|Q9RVE	282	9113500	7280300	6831300			
tr|Q9RTN3	tr|Q9RTN3	904	90982000	98170000	68601000	57436000	61701000	

tr|Q9RUT	tr|Q9RUT1	1117	1.82E+08	1.48E+08	1.23E+08	1.05E+08	1.04E+08	1.09E+08
tr|Q9RWK	tr|Q9RWK	1427						
sp|Q9RYE	sp|Q9RYE	433			16201000			
sp|Q9RYD	sp|Q9RYD	431	1.46E+08	2.02E+08	1.52E+08	3.56E+08	2.77E+08	2.97E+08
tr|Q9RSF8	tr|Q9RSF8	710	2239400	890770	982250	1455100	1735500	1440200
tr|Q9RW2	tr|Q9RW2	1345	1.17E+08	95444000	1.11E+08		56066000	
sp|Q9RUR	sp|Q9RUR	249	11424000	4020400	3265100	3874500	6514000	5001800
sp|Q9RUR	sp|Q9RUR	249	8.1E+09	1.97E+10	2.26E+10	1.15E+10	1.36E+10	1.12E+10
tr|Q9RYH	tr|Q9RYH1	1754	85441000	79247000	80871000	59201000	43722000	40507000
tr|Q9RYE4	tr|Q9RYE4	1738	38001000	28321000	28573000			
tr|Q9RU0	tr|Q9RU0	968	14489000		11726000			
sp|Q9RW	sp|Q9RW6	313				39286000		2.67E+08
sp|Q9RUV	sp|Q9RUV	253	1.52E+10	1.44E+10	2.16E+10	5.7E+09	5.38E+09	8.31E+09
tr|Q9RZQ	tr|Q9RZQ	1958	87388000	54908000	60210000	32564000	17105000	23906000
tr|Q9RWF	tr|Q9RWF	1400	1.84E+08	2.53E+08	2.58E+08	1.35E+08	1.4E+08	1.44E+08
sp|Q9RUE	sp|Q9RUE	228	94657000		96825000	1.18E+08	1.06E+08	91613000
tr|Q9RTU	tr|Q9RTU7	935	1.25E+09	1.43E+09	1.03E+09	8.7E+08	7.61E+08	6.08E+08
tr|Q9RZD	tr|Q9RZD1	1907	4.59E+08	3.37E+08	3.62E+08	3.39E+08	3.29E+08	3.68E+08
tr|Q9RVJ4	tr|Q9RVJ4	1241						
sp|Q9RSL	sp|Q9RSL4	136	6.04E+09	4.39E+09	4.24E+09	3.79E+08	5.37E+08	5.67E+08
sp|Q9RSQ	sp|Q9RSQ	141						
sp|Q9RSQ	sp|Q9RSQ	141	83368000	69876000	1.21E+08	38207000	43827000	58539000
tr|Q9RRE7	tr|Q9RRE7	513				98322000	69377000	90301000
tr|Q9RW5	tr|Q9RW5	1356	5310100		5702500	12832000	10331000	9065600
tr|Q9RYS5	tr|Q9RYS5	1804		48011000	37716000	23013000	27532000	28383000
sp|Q9RRB	sp|Q9RRB	76	18116000	39417000		47116000		
sp|Q9RRB	sp|Q9RRB	76	4.65E+08	6.6E+08	6.08E+08	2.89E+08	2.76E+08	3.61E+08
tr|Q9RSE7	tr|Q9RSE7	705	19033000		18915000			
tr|Q9RTT1	tr|Q9RTT1	928				17129000	11148000	
sp|Q9RX9	sp|Q9RX9	363						
tr|Q9RVP1	tr|Q9RVP1	1267	1.23E+08	1.58E+08	1.43E+08	88524000	79827000	1.27E+08
tr|Q9RYQ	tr|Q9RYQ3	1791						
tr|Q9RTR	tr|Q9RTR8	925				40083000	23663000	28912000
tr|Q9RS17	tr|Q9RS17	630			4006600			
sp|Q9RXI	sp|Q9RXI	374	19570000	42359000	24884000	10007000	25393000	12309000
tr|Q9RTK	tr|Q9RTK3	887			1816600	2266600	2433000	2916300
tr|Q9RRV1	tr|Q9RRV1	588	28383000	14484000	13345000			28442000
tr|Q9RTB	tr|Q9RTB3	847	10713000	8864100	5294100	13193000	12330000	13176000
tr|Q9RRE7	tr|Q9RRE7	513	10277000	19823000	13717000	18815000	12738000	13392000
tr|Q9RUN	tr|Q9RUN	1095	18092000	44715000	23186000			
tr|Q9RSL6	tr|Q9RSL6	732	1.08E+08	72993000	64334000	85511000	75009000	77750000
tr|Q9RXX2	tr|Q9RXX2	1660	57517000	78760000	70606000	43017000	40325000	43380000
tr|Q9RZA6	tr|Q9RZA6	1891						
tr|Q9RWR	tr|Q9RWR	1460	1.38E+08	1.75E+08	80161000	1.11E+08	1.22E+08	1.19E+08
sp|Q9RVC	sp|Q9RVC	278						
sp|Q9RU2	sp|Q9RU2	209			23034000	31316000		22916000
tr|Q9RSM	tr|Q9RSM	735						
tr|Q9RRG	tr|Q9RRG4	520	72601000	1.08E+08	93701000	1.22E+08	1.83E+08	1.51E+08
tr|Q9RYL2	tr|Q9RYL2	1769	1.1E+09	9.03E+08	9.19E+08	5.53E+08	6.3E+08	2.43E+08
sp|Q9RYM	sp|Q9RYM	439		18368000	17494000			
tr|Q9RYN	tr|Q9RYN8	1781		42531000		73069000	53983000	

tr|Q9RXE6	tr|Q9RXE6	1577		27606000				35833000
tr|Q9RSG5	tr|Q9RSG5	714	51213000		50294000			
sp|Q9RZA	sp|Q9RZA	454						
tr|Q9RXI1	tr|Q9RXI1	1601				65703000	76725000	83809000
tr|Q9RTM	tr|Q9RTM	901		12996000	15039000	1.28E+08	97997000	28967000
sp|Q9RVW	sp|Q9RVW	301						
tr|Q9RXS0	tr|Q9RXS0	1637	2910600		2510700		3707500	
tr|Q9RRH	tr|Q9RRH	525					8151000	7531600
sp|Q9RRB	sp|Q9RRB	76	2.1E+09	1.81E+09	1.35E+09	1.68E+09	1.32E+09	1.73E+09
sp|Q9RUP	sp|Q9RUP	246						
sp|Q9RV1	sp|Q9RV1	261	4.73E+08	3.94E+08	3.43E+08	50578000	42920000	7.06E+08
tr|Q9RTY7	tr|Q9RTY7	959	1.83E+09	2.21E+09	1.41E+09	1.47E+09	1.66E+09	1.51E+09
sp|Q9RV5	sp|Q9RV5	265	5713700	2808300	2881700	4599100	6385300	6668700
sp|Q9RV5	sp|Q9RV5	265		28608000	61200000	53181000	1.28E+08	51296000
sp|Q9RV5	sp|Q9RV5	265	1.23E+08	84490000	98474000	80428000	1.03E+08	
sp|Q9RV5	sp|Q9RV5	265	5.61E+08	3.87E+08	5.88E+08	3.33E+08	3E+08	3.68E+08
tr|Q9RYH1	tr|Q9RYH1	1754						
sp|Q9RVT	sp|Q9RVT	296	1.09E+08		12742000		2.86E+08	19281000
sp|Q9RVM	sp|Q9RVM	292						
tr|Q9RX83	tr|Q9RX83	1548	26776000	14134000	47166000	1.28E+08	1.59E+08	1.36E+08
tr|Q9RUM	tr|Q9RUM	1091	877640				896390	598810
sp|Q9RXI	sp|Q9RXI	374	2.61E+08	4.29E+08	3.61E+08	3.65E+08	4.59E+08	3.53E+08
tr|Q9RV57	tr|Q9RV57	1181					14043000	9299900
sp|Q9RU7	sp|Q9RU7	214	19235000	27581000	20697000	21263000	29705000	18008000
tr|Q9RRG4	tr|Q9RRG4	520	1.59E+08	1.62E+08	1.39E+08	2.16E+08	1.47E+08	1.43E+08
tr|Q9RSR9	tr|Q9RSR9	754	14048000				14207000	
sp|Q9RVY	sp|Q9RVY	303				23300000	22708000	18419000
tr|Q9RWG	tr|Q9RWG	1407	4.84E+08	2.39E+08	2.31E+08	3.4E+08	4.74E+08	3.55E+08
tr|Q9RVG6	tr|Q9RVG6	1228			6624100	49398000	1.22E+08	64070000
sp|Q9RYW	sp|Q9RYW	446						
tr|Q9RZK3	tr|Q9RZK3	1941						
tr|Q9RVK1	tr|Q9RVK1	1244		52571000	62369000		59588000	57284000
tr|Q9RTV5	tr|Q9RTV5	938				2.92E+08		2.68E+08
tr|Q9RU50	tr|Q9RU50	994					29355000	40889000
sp|Q9RV5	sp|Q9RV5	265				3621400	3021700	3715400
sp|Q9RXK	sp|Q9RXK	389				37288000	50019000	
sp|Q9RXK	sp|Q9RXK	389	1.14E+08	83095000	1.78E+08		3.05E+08	3.88E+08
sp|Q9RYB	sp|Q9RYB	427	1.43E+08	1.87E+08	1.41E+08	2.19E+08	2.22E+08	1.81E+08
tr|Q9RVK6	tr|Q9RVK6	1246	51445000	52182000	1.1E+08	77842000	72322000	94570000
sp|Q9RXR	sp|Q9RXR	396				22539000		16498000
tr|Q9RTZ9	tr|Q9RTZ9	967	48312000	65190000	1.02E+08	28785000	33423000	91940000
tr|Q9RSD0	tr|Q9RSD0	692	39014000	24259000	21697000	29797000	39535000	29268000
tr|Q9RZV3	tr|Q9RZV3	1983	9234800		8196400	95160000	1.31E+08	1.18E+08
sp|Q9RXK	sp|Q9RXK	385						
tr|Q9RVU2	tr|Q9RVU2	1300	1.75E+08	2.83E+08	1.89E+08	4.5E+08	4.69E+08	3.91E+08
tr|Q9RUU	tr|Q9RUU	1121	38227000	21369000	17590000	7764000	7928500	9629000
sp|Q9RW0	sp|Q9RW0	325						
tr|Q9RVT5	tr|Q9RVT5	1296	25789000	22768000	17362000			
sp|Q9RU7	sp|Q9RU7	213				13588000	16171000	
tr|Q9RSY1	tr|Q9RSY1	782		30831000	25667000	1.1E+08	1.02E+08	1E+08
sp|Q9RW0	sp|Q9RW0	325						

tr|Q9RUN	tr|Q9RUN	1094	10719000	8343800	13644000	21166000	26200000	26170000
sp|Q9RTK	sp|Q9RTK	191	18556000	19728000	31758000	19976000	18469000	28788000
tr|Q9RZV2	tr|Q9RZV2	1982		13312000	10719000		13941000	
tr|Q9RVU0	tr|Q9RVU0	1299				29728000	24038000	16876000
sp|Q9RU1	sp|Q9RU1	206	13078000	6111800	14841000			
tr|Q9RZ07	tr|Q9RZ07	1845			5281500	22448000	26612000	
sp|Q9RUR	sp|Q9RUR	249	5.32E+08	2.47E+08	2.15E+08	8.01E+08	1.11E+09	8.78E+08
sp|Q9RUR	sp|Q9RUR	249	1.05E+08	1.36E+08	1.97E+08	1.17E+08	1.74E+08	1.64E+08
sp|Q9RU2	sp|Q9RU2	209	14577000	19678000	20192000	1.22E+08	63865000	42191000
tr|Q9RRK9	tr|Q9RRK9	543	3.01E+08	2.35E+08	2.37E+08	80594000	2.02E+08	1.17E+08
tr|Q9RRG5	tr|Q9RRG5	521	1.33E+08	1.9E+08	1.53E+08	72167000	53884000	65870000
tr|Q9RUR4	tr|Q9RUR4	1107	47103000	27426000	27192000	13306000	15462000	15003000
tr|Q9RUR4	tr|Q9RUR4	1107			14538000			
tr|Q9RZ93	tr|Q9RZ93	1883			42034000		69346000	66258000
sp|Q9RW9	sp|Q9RW9	316	47686000	46878000	44336000			
tr|Q9RRI3	tr|Q9RRI3	531	605140					279980
tr|Q9RVP1	tr|Q9RVP1	1267	1E+08	83530000	88616000	1.62E+08	1.03E+08	1.14E+08
tr|Q9RVP1	tr|Q9RVP1	1267						
tr|Q9RUA	tr|Q9RUA	1023	1.36E+08	1.86E+08	1.3E+08	1.55E+08	1.24E+08	1.5E+08
tr|Q9RXA4	tr|Q9RXA4	1558	1.5E+08	82510000	1.81E+08	41680000	80793000	1.12E+08
tr|Q9RW2	tr|Q9RW2	1346	2.06E+08	1.73E+08	1.82E+08	29528000		29957000
sp|Q9RYM	sp|Q9RYM	438		24153000	27178000	66796000	61516000	57695000
tr|Q9RXF0	tr|Q9RXF0	1581						
tr|Q9RW7	tr|Q9RW7	1362	97459000	73334000	1.16E+08	79289000	92183000	1.11E+08
tr|Q9RRM	tr|Q9RRM	549	1.59E+09	1.79E+09	1.4E+09	2.04E+09	2.03E+08	1.67E+09
sp|Q9RTA	sp|Q9RTA	175	3222100	1739700	1492000	3131800	3895100	2885900
tr|Q9RVP1	tr|Q9RVP1	1267		22492000	38579000	59897000	56021000	55641000
tr|Q9RTG7	tr|Q9RTG7	873						
tr|Q9RXM	tr|Q9RXM	1616	1.99E+08	2.14E+08	2.24E+08	5.2E+08	3.44E+08	2.46E+08
tr|Q9RVA5	tr|Q9RVA5	1202	35151000	64489000	1.14E+08	37255000	31396000	43407000
tr|Q9RVA5	tr|Q9RVA5	1202			1.11E+08	98686000	51867000	65582000
sp|Q9RVW	sp|Q9RVW	301			14135000		14726000	
tr|Q9RRK9	tr|Q9RRK9	543			11965000			
tr|Q9RUX5	tr|Q9RUX5	1134			24830000	44772000	42759000	43028000
tr|Q9RZE3	tr|Q9RZE3	1913	9613900		12775000	13580000	15142000	22025000
tr|Q9RRF0	tr|Q9RRF0	514				35112000		19816000
tr|Q9RU08	tr|Q9RU08	973	95561000	1.33E+08	1.2E+08	1.75E+08	1.65E+08	1.75E+08
tr|Q9RV45	tr|Q9RV45	1172				54073000	1.68E+08	2.5E+08
tr|Q9RXE7	tr|Q9RXE7	1578	79638000	70830000	74615000	96068000	75237000	1.08E+08
tr|Q9RXE7	tr|Q9RXE7	1578		20911000	23273000			
tr|Q9RXD7	tr|Q9RXD7	1574				36032000	33085000	30649000
tr|Q9RSK2	tr|Q9RSK2	730	2.15E+08	1.87E+08	2.14E+08			
tr|Q9RXC5	tr|Q9RXC5	1570	2.51E+08	2.22E+08	1.67E+08	2.73E+08	2.18E+08	2.58E+08
tr|Q9RW2	tr|Q9RW2	1344						
tr|Q9RVJ0	tr|Q9RVJ0	1238	7.77E+08	1.23E+09	1.02E+09	7.47E+08	1.19E+09	7.8E+08
tr|Q9RW8	tr|Q9RW8	1367	6.29E+08	6.92E+08	7.92E+08	4.8E+08	6.88E+08	6.92E+08
tr|Q9RXM	tr|Q9RXM	1615						
tr|Q9RTM	tr|Q9RTM	899	25533000	25390000	32561000	64573000	51166000	65048000
tr|Q9RRY6	tr|Q9RRY6	609				16013000	14527000	
sp|O83026	sp|O83026	47				9138900		
tr|Q9RY62	tr|Q9RY62	1700						

tr|Q9RXM	tr|Q9RXM	1617		22138000				
tr|Q9RT07	tr|Q9RT07	796	8594900		3970000	6283500	5971400	
tr|Q9RRX1	tr|Q9RRX1	601	1.29E+08	1.37E+08	1.1E+08	2.28E+08	2E+08	9339000
tr|Q9RWQ	tr|Q9RWQ	1455						
sp|Q9RW(	sp|Q9RW(	342	2.87E+08	2.03E+08	2.59E+08	2.35E+08	2.23E+08	2.31E+08
tr|Q9RYV(	tr|Q9RYV(	1820						
tr|Q9RRE6	tr|Q9RRE6	512						24801000
tr|Q9RUX5	tr|Q9RUX5	1134			3311800			
sp|Q9RSS	sp|Q9RSS	150						
tr|Q9RZ86	tr|Q9RZ86	1878						
tr|Q9RYA5	tr|Q9RYA5	1719	14524000	12829000	11724000	42197000	26732000	
tr|Q9RRS7	tr|Q9RRS7	578	47254000	56609000	81107000	31370000	27610000	45720000
sp|Q9RX0	sp|Q9RX0	350						
tr|Q9RSQ4	tr|Q9RSQ4	748	3.82E+09	3.36E+09	2.96E+09	8.13E+09	8E+09	7.7E+09
sp|O32504	sp|O32504	44						
sp|O32504	sp|O32504	44						
sp|Q9RUY	sp|Q9RUY	259				43777000	53043000	36586000
sp|Q9RXT	sp|Q9RXT	401	3E+08	3.62E+08	2.76E+08	1.24E+09	1.1E+09	1.19E+09
tr|Q9RXS7	tr|Q9RXS7	1640	5340900	4104600	8634800	63998000	11894000	4976900
sp|Q9RUN	sp|Q9RUN	242	38388000	34791000	35562000	97189000	71421000	75066000
sp|Q9RUN	sp|Q9RUN	242	61153000	53550000	1.14E+08	35445000	25764000	49144000
sp|Q9RXT	sp|Q9RXT	398						1.37E+08
tr|Q9RUD	tr|Q9RUD	1040						
tr|Q9RXN(	tr|Q9RXN(	1619	82233000	1.16E+08	1.24E+08	71062000	86617000	70331000
sp|Q9RU8	sp|Q9RU8	221				1.89E+08	1.56E+08	1.46E+08
tr|Q9RWP	tr|Q9RWP	1448					12990000	13902000
sp|Q9RVT	sp|Q9RVT	297	42009000					
tr|Q9RT58	tr|Q9RT58	822						
tr|Q9RXB(	tr|Q9RXB(	1561	34138000		24893000	95219000	82389000	75040000
tr|Q9RWM	tr|Q9RWM	1438	84328000	3.63E+08	3.05E+08	1.2E+08	59154000	1.3E+08
tr|Q9RYN3	tr|Q9RYN3	1777	48662000	60933000	59072000	63758000	1.22E+08	1.53E+08
sp|Q9RSI9	sp|Q9RSI9	122	5.92E+08	76524000	4.95E+08	5.25E+08	5.07E+08	5.09E+08
tr|Q9RTQ1	tr|Q9RTQ1	913	66126000	75749000	72783000	1.39E+08	1.38E+08	1.15E+08
tr|Q9RTG1	tr|Q9RTG1	871	37468000	1.17E+08	95140000	71073000	40241000	53709000
tr|Q9RW3	tr|Q9RW3	1349	24185000	16343000	13112000	11469000	12568000	13122000
sp|Q9RXI	sp|Q9RXI	374	18183000		68667000	3.15E+08	5.66E+08	49866000
tr|Q9RTP6	tr|Q9RTP6	910						
tr|Q9RYQ(	tr|Q9RYQ(	1788	98755000	84758000	83336000	2.15E+08	1.73E+08	1.7E+08
tr|Q9RY75	tr|Q9RY75	1705						
tr|Q9RUX5	tr|Q9RUX5	1134	38732000	32032000	23136000	32566000	25187000	25581000
tr|Q9RUS1	tr|Q9RUS1	1111	53153000		67732000			
sp|Q9RRL	sp|Q9RRL	90	32889000	36466000	28565000	92640000		74303000
tr|Q9RRW	tr|Q9RRW	599					7409000	4935000
sp|Q9RUN	sp|Q9RUN	242	65511000	37316000	37561000	63538000	66663000	56681000
tr|Q9RXL6	tr|Q9RXL6	1612	92908000	74578000	91375000	1.02E+08	89486000	1.04E+08
tr|Q9RVT9	tr|Q9RVT9	1298	48238000	47419000	33096000	69182000	53877000	68903000
sp|Q9RXI	sp|Q9RXI	374			15262000	54801000	45756000	52363000
tr|Q9RYH8	tr|Q9RYH8	1757						
tr|Q9RVW	tr|Q9RVW	1311	37795000	40826000	28290000			
tr|Q9RT09	tr|Q9RT09	798	23196000	44255000	40853000	27054000		58015000
tr|Q9RSQ4	tr|Q9RSQ4	748	2.36E+08	2.94E+08	4.08E+08			

sp|Q9RUT	sp|Q9RUT	251	9574900		47834000			
sp|Q9RVE	sp|Q9RVE	284	1.11E+09	8.86E+08	8.5E+08	3.51E+08	4.44E+08	5.95E+08
sp|Q9RW5	sp|Q9RW5	310				28300000	18001000	
tr|Q9RRZ8	tr|Q9RRZ8	617	5850800			9469600	8510400	10387000
tr|Q9RZA5	tr|Q9RZA5	1890				21051000	25796000	
tr|Q9RST2	tr|Q9RST2	758	32782000	24609000	27194000	53596000	46718000	48193000
sp|Q9RXT4	sp|Q9RXT4	400						
sp|Q9RT3	sp|Q9RT3	166	1.56E+08	1.89E+08	1.5E+08	2.28E+08	1.92E+08	2.13E+08
tr|Q9RRP2	tr|Q9RRP2	556	77117000	1.5E+08	1.14E+08	1.68E+08	3.39E+08	
tr|Q9RXF5	tr|Q9RXF5	1584						
tr|Q9RYS8	tr|Q9RYS8	1807	23906000	16943000	20685000	28850000	33701000	23539000
tr|Q9RXX2	tr|Q9RXX2	1660			4609900			
sp|O3250	sp|O3250	44						
tr|Q9RXG8	tr|Q9RXG8	1591						
sp|Q9RVF	sp|Q9RVF	286		33653000	35442000	73373000	48429000	71553000
tr|Q9RTD3	tr|Q9RTD3	857	84785000	1.57E+08	1.66E+08	2.16E+08	2.5E+08	1.66E+08
tr|Q9RX56	tr|Q9RX56	1531	48341000	53485000	51334000	1.45E+08	1.21E+08	1.09E+08
sp|Q5933	sp|Q5933	63			11499000			
tr|Q9RTZ8	tr|Q9RTZ8	966	62847000	59884000	81145000			48501000
tr|Q9RVS8	tr|Q9RVS8	1292	1.18E+09	1.57E+09	1.27E+09	1.37E+09	1.83E+09	1.53E+09
tr|Q9RVS8	tr|Q9RVS8	1292						
tr|Q9RWX	tr|Q9RWX	1495	1.67E+08	1.33E+08	1.22E+08	67779000	49457000	66583000
sp|Q5933	sp|Q5933	63	3488300	2163100	3541600		1634400	1620700
sp|Q9RWK	sp|Q9RWK	335		78994000	86580000			1.01E+08
sp|Q9RWN	sp|Q9RWN	338		6556300	12446000			13522000
tr|Q9RYV3	tr|Q9RYV3	1821		17021000	26719000		23110000	
tr|Q9RU22	tr|Q9RU22	977				14278000		
tr|Q9RZN4	tr|Q9RZN4	1951	58749000	82832000	80784000	2.79E+08	2.89E+08	2.28E+08
tr|Q9RRS3	tr|Q9RRS3	574	10260000	17849000	34322000	18417000	29333000	21230000
tr|Q9RYM	tr|Q9RYM	1773		27651000	15566000		33694000	
tr|Q9RYM	tr|Q9RYM	1773						
tr|Q9RSH1	tr|Q9RSH1	717		10902000	6955700		33892000	
tr|Q9RXL6	tr|Q9RXL6	1612			20832000			
tr|Q9RYI7	tr|Q9RYI7	1763	63431000		56043000	1.24E+08		90423000
sp|Q9RRD	sp|Q9RRD	80	4.06E+08	3.13E+08	3.17E+08	2.98E+08	2.99E+08	3.01E+08
tr|Q9RT50	tr|Q9RT50	819			22323000			12384000
tr|Q9RZT6	tr|Q9RZT6	1973	37546000	51148000	39381000	1.05E+08	60681000	97040000
sp|Q9RTI0	sp|Q9RTI0	187					23173000	
tr|Q9RU37	tr|Q9RU37	984	69169000	65451000	50792000	1.03E+08	81023000	1.05E+08
sp|Q9RU7	sp|Q9RU7	214	6.52E+08	6.54E+08	5.97E+08	3.7E+08	3E+08	3.11E+08
sp|Q9RU2	sp|Q9RU2	209	1.61E+09	1.77E+09	1.9E+09	1.57E+09	1.25E+09	1.53E+09
sp|Q9RU2	sp|Q9RU2	209	1.06E+08	74473000	2.78E+08	1.26E+09	1.28E+09	1.81E+09
tr|Q9RZG9	tr|Q9RZG9	1926		10949000				
tr|Q9RVE5	tr|Q9RVE5	1219						
sp|Q9RRB	sp|Q9RRB	76	3.35E+09	4.14E+09	2.92E+09	3.63E+09	3.18E+09	3.38E+09
tr|Q9RTR2	tr|Q9RTR2	921	1.12E+08	72785000	85576000	1.36E+08	90138000	80100000
tr|Q9RU26	tr|Q9RU26	979	22743000			18599000	23106000	20695000
sp|Q9RW0	sp|Q9RW0	305	17720000	21897000		29404000	23068000	23530000
tr|Q9RYG5	tr|Q9RYG5	1750	25252000	28144000	27402000	45038000	36187000	
tr|Q9RV94	tr|Q9RV94	1197	38918000	40977000	31004000	48003000	71134000	34698000
tr|Q9RS92	tr|Q9RS92	667	21307000	18108000	19378000	73505000	42566000	54237000

tr|Q9RX72	tr|Q9RX72	1542	43178000	48130000	42067000			
tr|Q9RWV	tr|Q9RWV	1484	6.66E+08	4.23E+08	4.48E+08	2.04E+08	3.58E+08	1.85E+08
sp|Q9RRC	sp|Q9RRC	79	1.98E+08	2.07E+08	1.84E+08	1.71E+08	1.2E+08	1.37E+08
tr|Q9RU0	tr|Q9RU0	972	1.71E+08	95297000	95415000	1.77E+08	1.87E+08	1.12E+08
sp|Q9RRM	sp|Q9RRM	92	13946000	28635000	31288000	34091000	25112000	36434000
sp|Q9RVV	sp|Q9RVV	300				94951000		
sp|Q9RVV	sp|Q9RVV	300	2.81E+08	3E+08	4.64E+08	2.41E+08	1.65E+08	2.82E+08
tr|Q9RS92	tr|Q9RS92	667						
tr|Q9RYQ	tr|Q9RYQ	1792		93637000			24945000	
tr|Q9RW0	tr|Q9RW0	1332	40006000			55058000	55160000	
tr|Q9RWB	tr|Q9RWB	1385	46533000	44502000	38002000	1.07E+08	91069000	1.06E+08
tr|Q9RZ07	tr|Q9RZ07	1845	1.76E+09	1.85E+09	1.48E+09	1.23E+09	1.27E+09	1.4E+09
tr|Q9RYI7	tr|Q9RYI7	1763	46385000	64532000	8680400	79692000	58316000	70960000
sp|Q4657	sp|Q4657	61				7.59E+08	3.15E+08	8.03E+08
sp|Q9RTN	sp|Q9RTN	196				3192800	4616200	
tr|Q9RVS2	tr|Q9RVS2	1288				1.02E+08	71194000	6835700
sp|Q9RTN	sp|Q9RTN	196				22379000	33015000	
tr|Q9RVZ5	tr|Q9RVZ5	1328	58845000		38837000	2.21E+08	2.17E+08	2.14E+08
sp|Q9RV5	sp|Q9RV5	265		464400	411670			
sp|Q9RV5	sp|Q9RV5	265				5212800	7616000	7429600
sp|Q9RV5	sp|Q9RV5	265		35336000	59415000	1.14E+08	1.26E+08	1.48E+08
sp|Q9RVY	sp|Q9RVY	303				38839000	43163000	
tr|Q9RUW	tr|Q9RUW	1128				15709000	17406000	13741000
sp|Q9RXG	sp|Q9RXG	370					40669000	56339000
tr|Q9RYD	tr|Q9RYD	1735	1.45E+09	1.29E+09	1.08E+09	2.02E+09	1.64E+09	1.81E+09
tr|Q9RYN	tr|Q9RYN	1780	20251000	10593000	9863400	12481000	15715000	14423000
sp|Q9RY5	sp|Q9RY5	418		13393000	90369000	29079000	86201000	53496000
tr|Q9RUK	tr|Q9RUK	1080	27388000	24302000	19930000		18609000	
sp|Q9RY2	sp|Q9RY2	411	1.66E+08	1.89E+08	2.14E+08			
sp|Q9RY2	sp|Q9RY2	411	24541000	22002000	25095000	21954000		20599000
tr|Q9RVS0	tr|Q9RVS0	1286			8350700			
sp|Q9RYL	sp|Q9RYL	436						
sp|Q9RRX	sp|Q9RRX	101				86521000	74183000	49307000
tr|Q9RV92	tr|Q9RV92	1196						
tr|Q9RRZ	tr|Q9RRZ	613	1.02E+08	1.11E+08	92949000	2E+08	1.93E+08	1.71E+08
sp|Q9RVV	sp|Q9RVV	300			31504000	41322000		42327000
tr|Q9RRR	tr|Q9RRR	570						
tr|Q9RVP	tr|Q9RVP	1266	1.61E+08	81723000	81441000	71846000	1.08E+08	90251000
sp|Q9RTL	sp|Q9RTL	194						
sp|Q9RVC	sp|Q9RVC	276	23315000		19204000	54133000	26346000	43487000
tr|Q9RXI1	tr|Q9RXI1	1601						
tr|Q9RZD	tr|Q9RZD	1907	55420000	32044000	42623000		61720000	32279000
tr|Q9RZV	tr|Q9RZV	1983	50835000		39325000	18403000	34896000	62262000
tr|Q9RZD	tr|Q9RZD	1907			16555000			
sp|Q9RUV	sp|Q9RUV	257	2.75E+08	2.42E+08	2.5E+08	1.98E+08	1.68E+08	2.54E+08
sp|Q9RSS	sp|Q9RSS	145	41193000	40299000	16538000	88945000	29512000	76533000
sp|Q9RU3	sp|Q9RU3	210					33564000	
sp|Q9RU3	sp|Q9RU3	210					14168000	17322000
tr|Q9RUI4	tr|Q9RUI4	1063		12100000		8412100	23176000	
tr|Q9RST1	tr|Q9RST1	757				3.94E+08	2.77E+08	3.34E+08
tr|Q9RRJ7	tr|Q9RRJ7	537	2.95E+08	1.98E+08	3.11E+08	1.45E+08	1.73E+08	2.45E+08

tr|Q9RT93	tr|Q9RT93	839	62660000	93695000	79034000	1.4E+08	1.1E+08	96305000
tr|Q9RTH4	tr|Q9RTH4	877	71687000	1.08E+08	2.1E+08	55948000	95601000	1.18E+08
tr|Q9RU45	tr|Q9RU45	992						19451000
sp|Q9RXT	sp|Q9RXT	398		13197000				
sp|Q9RXT	sp|Q9RXT	398			40133000			
sp|Q9RTH	sp|Q9RTH	186	3.95E+08	5.43E+08	4.23E+08	6.62E+08	6.28E+08	5.6E+08
tr|Q9RTT2	tr|Q9RTT2	929	9.84E+08	9.61E+08	7.96E+08	5.12E+08	5.35E+08	6.32E+08
tr|Q9RTT2	tr|Q9RTT2	929	32312000	31771000	49194000	23542000	24866000	20297000
sp|Q9RVH	sp|Q9RVH	288			5007500			
sp|Q9RRJ	sp|Q9RRJ	87	12506000	8910000	8556700	23630000	22695000	23440000
sp|Q9RRJ	sp|Q9RRJ	87						
tr|Q9RXE8	tr|Q9RXE8	1579		42637000	42280000	60854000	64774000	38762000
tr|Q9RZ35	tr|Q9RZ35	1862						
sp|Q9RV9	sp|Q9RV9	272	11345000		14791000	28737000	21064000	27331000
tr|Q9RT09	tr|Q9RT09	798			50303000	30635000	30725000	
tr|Q9RYH9	tr|Q9RYH9	1758	2.9E+08	2.6E+08	2.46E+08	2.59E+08	2.71E+08	2.34E+08
tr|Q9RUA	tr|Q9RUA	1022	38932000	1.03E+08	52355000	1.73E+08	1.6E+08	1.74E+08
tr|Q9RRI9	tr|Q9RRI9	535						
tr|Q9RVS5	tr|Q9RVS5	1290		2.64E+08	2.01E+08	1.84E+08	1.96E+08	1.91E+08
sp|Q9RRT	sp|Q9RRT	98		4582200	12952000	59666000	82605000	48922000
tr|Q9RTQ	tr|Q9RTQ	917						
tr|Q9RT87	tr|Q9RT87	837	1.95E+08	1.94E+08	1.54E+08	3.53E+08	2.56E+08	3.7E+08
tr|Q9RS10	tr|Q9RS10	626			71363000			34424000
tr|Q9RTF9	tr|Q9RTF9	869				31395000	36841000	28354000
tr|Q9RSB9	tr|Q9RSB9	686	15446000		13433000	38587000	39310000	39924000
tr|Q9RSB9	tr|Q9RSB9	686						
tr|Q9RVX7	tr|Q9RVX7	1318	1.06E+08	93750000	72276000	74823000	68953000	79068000
tr|Q9RVX7	tr|Q9RVX7	1318	30606000	16727000	25636000			
tr|Q9RVM	tr|Q9RVM	1257	1.14E+08	1.47E+08	1.21E+08	1.65E+08	1.02E+08	84104000
sp|Q9RVC	sp|Q9RVC	277	81523000			25278000		
tr|Q9RXQ	tr|Q9RXQ	1627			23980000		18738000	35469000
sp|Q9RTB	sp|Q9RTB	176	7862400	8805000	9103300			8592500
sp|Q9RZE	sp|Q9RZE	455			5062000	12283000		9828300
tr|Q9RVM	tr|Q9RVM	1257	1.2E+09	1.17E+09	8.93E+08	5.22E+08	5.89E+08	5.8E+08
tr|Q9RWL	tr|Q9RWL	1431	1.83E+08	1.92E+08	1.72E+08		19895000	14300000
tr|Q9RUW	tr|Q9RUW	1127	82387000	81273000	55641000	61866000	52366000	56976000
sp|Q9RRD	sp|Q9RRD	80	1.29E+08	1.77E+08	2.51E+08	87019000	69790000	1.34E+08
tr|Q9RXY3	tr|Q9RXY3	1664	6347700		4963100		4351100	
tr|Q9RTI8	tr|Q9RTI8	881	2.14E+08	2.06E+08	1.88E+08	1.54E+08	1.48E+08	1.31E+08
tr|Q9RVS1	tr|Q9RVS1	1287	1.41E+08	2.72E+08	2.94E+08	4.02E+08	3.15E+08	2.21E+08
tr|Q9RV0	tr|Q9RV0	1151				9294200	9038100	9142000
tr|Q9RR71	tr|Q9RR71	470			16116000			
tr|Q9RS17	tr|Q9RS17	630		2.06E+08	1.24E+08	2.32E+08	1.89E+08	2.33E+08
tr|Q9RS17	tr|Q9RS17	630	5.83E+08	4.99E+08	1.13E+09	5.05E+08	4.08E+08	5.9E+08
sp|Q9RRB	sp|Q9RRB	76		28249000	16203000	2.68E+08		2.85E+08
sp|Q9RRB	sp|Q9RRB	76	38405000	62199000	66741000			
sp|Q9RZ0	sp|Q9RZ0	452	7789600					
sp|Q9RRH	sp|Q9RRH	85					41889000	40460000
tr|Q9RW0	tr|Q9RW0	1336		88395000	65780000	99222000	93528000	94978000
tr|Q9RW0	tr|Q9RW0	1336		24605000	23839000			
tr|Q9RSM	tr|Q9RSM	736						

sp|Q9RT6	sp|Q9RT6	169			7560400			
tr|Q9RW1	tr|Q9RW1	1339	3.63E+08	5.06E+08	3.85E+08	3.58E+08	5.81E+08	3.63E+08
tr|Q9RRB	tr|Q9RRB2	493	14325000	12552000	16992000	21697000	37676000	22051000
tr|Q9RZE	tr|Q9RZE3	1913	2.19E+08	2.31E+08	4.23E+08	1.77E+08	3.06E+08	3.29E+08
tr|Q9RWG	tr|Q9RWG	1407	2.19E+08	2.23E+08	2.21E+08	9.36E+08	6.37E+08	6.88E+08
tr|Q9RWG	tr|Q9RWG	1407	1.11E+08	97900000	2.5E+08	61009000	61828000	76101000
tr|Q9RTP	tr|Q9RTP	906	2.29E+08	2.15E+08	1.72E+08	1.82E+08	2.72E+08	2.96E+08
sp|Q9RRY	sp|Q9RRY	103	41739000			1.05E+08	1.13E+08	88269000
tr|Q9RTM	tr|Q9RTM	899	1.28E+08	1.48E+08	1.1E+08	2.83E+08	3.74E+08	3.04E+08
tr|Q9RUS	tr|Q9RUS	1113		6537200		16896000	16972000	11517000
sp|Q9RRX	sp|Q9RRX	102	69201000	76036000	65540000	1.32E+08	1.26E+08	1.22E+08
sp|Q9RRX	sp|Q9RRX	102	20091000	18228000	33822000			11504000
tr|Q9RVP1	tr|Q9RVP1	1267				1.23E+08	1.06E+08	85656000
sp|Q9RVI	sp|Q9RVI3	289				7866100		
tr|Q9RXU	tr|Q9RXU1	1644	10884000	16529000	15841000	30908000	33691000	16130000
tr|Q9RS03	tr|Q9RS03	620	61349000	58691000	63471000			66068000
tr|Q9RRJ7	tr|Q9RRJ7	537			14758000			
tr|Q9RUU	tr|Q9RUU	1124	1.6E+08	1.64E+08	1.43E+08	1.54E+08	1.26E+08	1.38E+08
tr|Q9RV03	tr|Q9RV03	1152			10514000	28920000		17376000
tr|Q9RTH	tr|Q9RTH	877		31536000	73325000	39624000	50411000	38803000
tr|Q9RU7	tr|Q9RU7	1013	2.21E+08	3.14E+08	2.72E+08		72664000	
tr|Q9RSD	tr|Q9RSD	692	1.21E+08	1.04E+08	92141000	3.91E+08	3.4E+08	2.82E+08
tr|Q9RV2	tr|Q9RV29	1165						
tr|Q9RSM	tr|Q9RSM	737	29918000	24424000	32425000	32091000	52465000	43150000
tr|Q9RUA	tr|Q9RUA	1024						
sp|Q9RW	sp|Q9RW9	318	24716000	30244000	28129000	75455000	81095000	69796000
sp|Q9RT4	sp|Q9RT4	167	47901000		28535000			
tr|Q9RU5	tr|Q9RU52	995					23168000	
tr|Q9RYH	tr|Q9RYH	1756	30560000	3.2E+08	1.86E+08	4E+08	3.73E+08	3.67E+08
tr|Q9RVP1	tr|Q9RVP1	1267	8.12E+08	8.01E+08	6.64E+08	1.84E+09	1.67E+09	1.63E+09
tr|Q9RVP1	tr|Q9RVP1	1267	41175000		1.13E+08			20808000
tr|Q9RVP1	tr|Q9RVP1	1267						
tr|Q9RUG	tr|Q9RUG	1055	12792000	15634000	18992000	61455000	69490000	42671000
sp|Q9RRH	sp|Q9RRH	85	37756000	30544000	32214000	23920000	36599000	32256000
tr|Q9RU9	tr|Q9RU9	1015	50729000	28010000	58894000	82099000	80046000	86419000
tr|Q9RZI5	tr|Q9RZI5	1936						
tr|Q9RWU	tr|Q9RWU	1474	90357000	1.18E+08		1.54E+08		1.08E+08
sp|Q9RVV	sp|Q9RVV	300	21673000	45557000		51292000	42629000	40459000
tr|Q9RST	tr|Q9RST9	761		14585000	12907000	24781000	25046000	
tr|Q9RX8	tr|Q9RX85	1550						
tr|Q9RZQ	tr|Q9RZQ	1958	1.15E+08	1.5E+08	2.37E+08	90223000	91320000	1.31E+08
tr|Q9RTC	tr|Q9RTC9	855	87239000	54224000	61074000	1.01E+08	83917000	
tr|Q9RSF8	tr|Q9RSF8	710	3.23E+08	8.9E+08	8.5E+08	7.71E+08	1.05E+08	8.28E+08
tr|Q9RSF8	tr|Q9RSF8	710	1.86E+08	1.71E+08	3.35E+08	1.04E+08	1.21E+08	1.59E+08
tr|Q9RZP	tr|Q9RZP2	1952	52328000	1.69E+08	1.01E+08	81139000	75930000	1.04E+08
sp|Q9RWJ	sp|Q9RWJ	334						
sp|Q9RVM	sp|Q9RVM	292			43733000	42337000	69870000	
sp|Q9RSE	sp|Q9RSE	116	1.43E+08	1.31E+08	1.09E+08	2.76E+08	2.42E+08	2.26E+08
tr|Q9RRZ	tr|Q9RRZ4	613	24272000	38477000	51420000	2.73E+08	66433000	1.08E+08
tr|Q9RUK	tr|Q9RUK	1079	64822000	88377000	59011000	1.31E+08	1.07E+08	80637000
tr|Q9RUK	tr|Q9RUK	1079	24363000	49261000	57091000	15808000		16528000

tr|Q9RTI3	tr|Q9RTI3	879	9398800	14355000	10783000	34646000	45398000	28529000
tr|Q9RRW	tr|Q9RRW	595					26911000	14218000
tr|Q9RRW	tr|Q9RRW	595						
sp|Q9RVC	sp|Q9RVC	277						
sp|Q9RRB	sp|Q9RRB	76	6.53E+08	8.29E+08	1.04E+09	21334000	2.75E+08	3.36E+08
sp|Q9RSF	sp|Q9RSF	117						
sp|Q9RRE	sp|Q9RRE	83				59044000	69088000	71955000
sp|Q9RUV	sp|Q9RUV	258		3491700	3255800			
sp|Q9RW	sp|Q9RW	342		1.1E+08		94078000	1.32E+08	1.02E+08
tr|Q9RVS	tr|Q9RVS	1286	27908000	31313000	33007000	9617700		48282000
tr|Q9RZT	tr|Q9RZT	1970		13206000		37612000	66483000	19216000
tr|Q9RY2	tr|Q9RY2	1681		23603000	23061000		17897000	
tr|Q9RRU	tr|Q9RRU	584	15236000	17330000	28857000			
tr|Q9RZ12	tr|Q9RZ12	1849	8.23E+08	1.12E+09	9.31E+08	5.88E+08	7.13E+08	5.7E+08
sp|Q9RU2	sp|Q9RU2	209	9076400	5893400	10301000	2.74E+08	2.24E+08	1.75E+08
tr|Q9RV31	tr|Q9RV31	1167	16003000	1.73E+08	2.56E+08	1.39E+08	2.4E+08	1.28E+08
sp|Q9RXR	sp|Q9RXR	396		23830000	13784000	19615000	28670000	21703000
tr|Q9RTP	tr|Q9RTP	908	17966000	3834500	11645000	38571000	26421000	30582000
tr|Q9RRX1	tr|Q9RRX1	601	8216900	13608000	8717300	1.19E+08	75748000	99214000
tr|Q9RX5	tr|Q9RX56	1531						
sp|Q9RS1	sp|Q9RS1	106	1.59E+08	1.62E+08	1.22E+08	2.3E+08	2.69E+08	2.37E+08
sp|Q9RTR	sp|Q9RTR	198						
sp|Q9RRB	sp|Q9RRB	76	1.43E+08	5.77E+08	4.67E+08	5.46E+08	4.38E+08	5.55E+08
sp|Q9RVW	sp|Q9RVW	301				18430000	22072000	23462000
sp|Q9RVW	sp|Q9RVW	301	15798000	31323000	56105000			
tr|Q9RXE	tr|Q9RXE	1575				24656000	24084000	26201000
tr|Q9RUG	tr|Q9RUG	1055	2.76E+08	2.08E+08	2.06E+08	4.2E+08	3.9E+08	4.08E+08
tr|Q9RRQ	tr|Q9RRQ	566						
tr|Q9RRG	tr|Q9RRG	521	2.46E+08	1.49E+08	1.81E+08	62772000	50272000	78446000
tr|Q9RW6	tr|Q9RW6	1359				15659000		
sp|Q9RRB	sp|Q9RRB	76	27088000	18011000	34059000	15772000		23180000
sp|Q9RSI	sp|Q9RSI	122	1.49E+08	1.23E+08	1.21E+08	3.6E+08	2.87E+08	2.9E+08
tr|Q9RUK	tr|Q9RUK	1081				74494000		58920000
tr|Q9RS6	tr|Q9RS66	653						10528000
tr|Q9RYT	tr|Q9RYT	1809	24970000	32939000	26085000	94169000	40437000	71116000
sp|Q9RSI	sp|Q9RSI	121	90250000	1.07E+08	86858000	1.63E+08	1.46E+08	1.53E+08
tr|Q9RU4	tr|Q9RU4	988	8375300	11173000	14161000	78077000	27643000	24169000
sp|Q9RV2	sp|Q9RV2	262	22892000	19722000	20199000	15932000	13561000	11065000
tr|Q9RW2	tr|Q9RW2	1345	18501000		22252000			19904000
tr|Q9RZC	tr|Q9RZC	1904		1.18E+08	1.7E+08	1.26E+08	1.6E+08	1.81E+08
tr|Q9RU4	tr|Q9RU4	987					2106200	
sp|Q9RXF	sp|Q9RXF	369	2.2E+08		1.9E+08	2.11E+08	2.09E+08	2.07E+08
tr|Q9RTL6	tr|Q9RTL6	897	57004000	60850000	62396000		58509000	83475000
sp|Q9RW	sp|Q9RW	327		15756000	21156000	11185000	29863000	
tr|Q9RXS1	tr|Q9RXS1	1638	62906000		50551000	65172000	65672000	76370000
tr|Q9RXS1	tr|Q9RXS1	1638						
sp|Q9RXJ1	sp|Q9RXJ1	376				17746000	29302000	30159000
tr|Q9RWB	tr|Q9RWB	1383		30157000		46486000	47623000	39800000
tr|Q9RTC	tr|Q9RTC	855	1.66E+08		1.48E+08	1.24E+08		1.23E+08
sp|P5686	sp|P5686	56	7.17E+08	6.14E+08	7.53E+08	7.15E+08	7.72E+08	6.9E+08
sp|Q9RX2	sp|Q9RX2	355	8858400			57841000	47646000	40572000

sp|Q9RW	sp|Q9RW	335						
tr|Q9RVH	tr|Q9RVH	1234				55945000	41095000	47063000
sp|Q9RV8	sp|Q9RV8	270					28859000	
sp|Q9RVV	sp|Q9RVV	300						
tr|Q9RRE1	tr|Q9RRE1	509	22730000	23865000	16009000		47453000	72916000
tr|Q9RRW	tr|Q9RRW	596					10862000	14940000
tr|Q9RYQ	tr|Q9RYQ	1790	26293000	11815000	11965000	10358000	15855000	15473000
tr|Q9RVX	tr|Q9RVX7	1318	45507000	37236000	46517000	61811000	88550000	73483000
sp|Q9RYY	sp|Q9RYY	448						
tr|Q9RV8	tr|Q9RV83	1192	607500			364130		
sp|Q9RZ0	sp|Q9RZ0	450			2811800	5390900	9578600	7197900
tr|Q9RUK	tr|Q9RUK	1077						
tr|Q9RT2	tr|Q9RT28	805				23070000	13393000	14381000
tr|Q9RT2	tr|Q9RT28	805		20389000	36867000	22934000	29090000	37612000
tr|Q9RRR	tr|Q9RRR	571				13218000		
tr|Q9RRX	tr|Q9RRX3	602		20062000		50667000	36748000	39675000
tr|Q9RXZ	tr|Q9RXZ2	1668	2.76E+08	3E+08	3.48E+08	2.93E+08	4.2E+08	3.48E+08
tr|Q9RUK	tr|Q9RUK	1077	10956000	9483700	12585000	55308000	51143000	23386000
tr|Q9RVP1	tr|Q9RVP1	1267	24014000	3.53E+08	71101000	2.6E+08	2.78E+08	2.14E+08
tr|Q9RTG	tr|Q9RTG7	873	16259000	16780000	15811000	12804000	9460000	13391000
tr|Q9RVQ	tr|Q9RVQ	1272	5.56E+08	5.67E+08	4.52E+08	4.26E+08	4.12E+08	4.39E+08
sp|Q9RS1	sp|Q9RS1	107						15849000
sp|Q9RVW	sp|Q9RVW	301	1.08E+09	9.72E+08	1.06E+08	7.52E+08	6.08E+08	7.62E+08
sp|P5692	sp|P56926	59					1.55E+08	
sp|Q9RYR	sp|Q9RYR	442					30177000	
tr|Q9RUU	tr|Q9RUU	1123	47523000	35191000	52469000	1.43E+08	1.08E+08	1.12E+08
tr|Q9RSY	tr|Q9RSY6	784	2145400	565580	636490			
tr|Q9RTP	tr|Q9RTP5	909			12550000		11202000	17172000
tr|Q9RUA	tr|Q9RUA	1022		31839000	19943000			
tr|Q9RXH	tr|Q9RXH	1595	16337000	9624400				
sp|Q9RW	sp|Q9RW1	306	27160000		23692000		16871000	
tr|Q9RV1	tr|Q9RV18	1161	30007000	45360000	36400000		17832000	31479000
tr|Q9RY67	tr|Q9RY67	1701	67018000	91054000	70092000	1.7E+08	2.03E+08	1.66E+08
tr|Q9RY67	tr|Q9RY67	1701			14650000		12999000	
tr|Q9RUD	tr|Q9RUD	1043			7478200		10310000	
tr|Q9RVQ	tr|Q9RVQ	1276	19060000	29633000	25116000		21690000	8340700
sp|Q9RR7	sp|Q9RR7	67			7569600			
tr|Q9RZL7	tr|Q9RZL7	1945	1.92E+08	1.37E+08	1.64E+08	2.28E+08	2.14E+08	2.99E+08
tr|Q9RX1	tr|Q9RX16	1511						
tr|Q9RT5	tr|Q9RT53	821				5541300	5295600	4747900
tr|Q9RZE	tr|Q9RZE9	1918						
tr|Q9RYQ	tr|Q9RYQ1	1789				38340000	42219000	33819000
tr|Q9RXN	tr|Q9RXN0	1619		32846000				
sp|Q9RW	sp|Q9RW9	317		25172000	24518000	28803000	17817000	
tr|Q9RS8	tr|Q9RS87	663	71781000	73401000	63420000	1.57E+08	1.19E+08	
tr|Q9RS8	tr|Q9RS87	663	6.89E+08	7.49E+08	1.16E+09	5.37E+08	8.63E+08	1E+09
tr|Q9RS8	tr|Q9RS87	663		23395000				22720000
tr|Q9RYQ	tr|Q9RYQ4	1792	52822000					15309000
tr|Q9RXB	tr|Q9RXB0	1561					65267000	
sp|P5686	sp|P56867	56	1.16E+08	1.26E+08	1.08E+08	3.22E+08	2.71E+08	2.69E+08
sp|Q9RX0	sp|Q9RX0	350					5647200	7324900

tr|Q9RXD	tr|Q9RXD2	1572					25315000	
tr|Q9RV4	tr|Q9RV45	1172				2.4E+08	2.19E+08	2.03E+08
tr|Q9RTE	tr|Q9RTE3	862		17197000	14806000	49572000	45715000	35892000
tr|Q9RTE	tr|Q9RTE3	862	91535000		1.01E+08	48282000	40498000	55668000
sp|Q9RS1	sp|Q9RS1	106				85528000	69881000	65119000
tr|Q9RWI	tr|Q9RWI3	1415		4250000	4078100	5527400	4805900	6748200
tr|Q9RRY	tr|Q9RRY8	610	5257500	5540500	6992500	12970000	9836100	10436000
tr|Q9RRY	tr|Q9RRY8	610	5664800	3769800	4879600	3560700	3421100	4217500
tr|Q9RYR	tr|Q9RYR7	1799	2.93E+08	2.17E+08	2.69E+08	2.2E+08	2.08E+08	2.59E+08
tr|Q9RRB	tr|Q9RRB8	496				19895000	15530000	
tr|Q9RVA	tr|Q9RVA3	1200						
sp|Q9RRX	sp|Q9RRX	101				22210000	26807000	27231000
tr|Q9RT0	tr|Q9RT05	794	13477000		12093000	17741000	17313000	23118000
tr|Q9RY9	tr|Q9RY98	1717						
tr|Q9RRI7	tr|Q9RRI7	534	55461000	39548000	32616000	1.38E+08	1.24E+08	1.31E+08
tr|Q9RRI7	tr|Q9RRI7	534			19667000			
tr|Q9RRP	tr|Q9RRP4	558	67221000	68148000	81316000	68867000	63666000	63875000
tr|Q9RSH	tr|Q9RSH0	716	41373000	34055000	32610000	20585000		23491000
sp|Q9RVW	sp|Q9RVW	301				51536000	57400000	48436000
tr|Q9RUB	tr|Q9RUB	1031					19353000	15065000
sp|Q9RYM	sp|Q9RYM	438	5.47E+09	5.7E+09	5.38E+09	9.77E+09	7.13E+09	8.39E+09
tr|Q9RWL	tr|Q9RWL	1434		16647000	17456000	23694000		
sp|Q9RU7	sp|Q9RU7	214	1.74E+08	1.96E+08	1.55E+08	1.73E+08	1.23E+08	1.31E+08
sp|Q9RV9	sp|Q9RV9	271	1E+08	1.64E+08	1.06E+08	3.94E+08	5.98E+08	3.56E+08
tr|Q9RVM	tr|Q9RVM	1257					9048200	12388000
tr|Q9RT5	tr|Q9RT58	822						
sp|Q9RTD	sp|Q9RTD	179				35743000		
sp|Q9RTD	sp|Q9RTD	179	2.35E+08	1.86E+08	2.13E+08		96669000	
tr|Q9RYP	tr|Q9RYP6	1785				55456000	40825000	43498000
sp|Q9RVT	sp|Q9RVT	296			28576000	20637000	16333000	18030000
sp|Q9RU2	sp|Q9RU2	209	82251000	1.04E+08	1.16E+08	2.29E+08	1.56E+08	1.54E+08
sp|Q9RU2	sp|Q9RU2	209			39372000	24416000		
tr|Q9RT07	tr|Q9RT07	796		18887000	18489000		20174000	24265000
tr|Q9RSL6	tr|Q9RSL6	732		3859800	2226100	7494200	6126500	10029000
tr|Q9RS0	tr|Q9RS00	618						
tr|Q9RS0	tr|Q9RS00	618						
tr|Q9RZC	tr|Q9RZC7	1906	1.78E+09	1.78E+09	1.22E+09	4.18E+09	3.29E+09	3.34E+09
tr|Q9RXB	tr|Q9RXB8	1566	77457000	78593000	65606000	1.28E+08	1.43E+08	
sp|Q9RXP	sp|Q9RXP	393	36392000	77983000	59960000	2.07E+08	2.04E+08	1.65E+08
tr|Q9RVR	tr|Q9RVR2	1281	25943000	30942000	30691000	1.99E+08	1.36E+08	1.7E+08
sp|Q9RZU	sp|Q9RZU	458						
sp|Q9RXT	sp|Q9RXT	401	76865000	88383000	1.11E+08	1.55E+08	1.43E+08	1.73E+08
tr|Q9RU0	tr|Q9RU07	972						
tr|Q9RU9	tr|Q9RU93	1017						
tr|Q9RYV	tr|Q9RYV3	1821	38976000	20251000	22834000	12237000	38041000	10537000
sp|Q9RRB	sp|Q9RRB	76						
tr|Q9RTV1	tr|Q9RTV1	936	1.39E+08	1.9E+08	3.53E+08	1.76E+08	2.01E+08	2.43E+08
tr|Q9RRY	tr|Q9RRY2	605	6.72E+08	2.98E+08	3.58E+08	3.93E+08	1.99E+08	3.53E+08
tr|Q9RU3	tr|Q9RU30	982		14509000	6696700	49883000	59418000	47751000
sp|Q9RV6	sp|Q9RV6	266				1.35E+08	1.3E+08	1.29E+08
sp|Q9RV6	sp|Q9RV6	266						

tr|Q9RU1	tr|Q9RU1	976						
tr|Q9RXD	tr|Q9RXD	1573	81798000	94040000	52204000	4.15E+08	3.39E+08	2.92E+08
tr|Q9RV11	tr|Q9RV11	1156						
tr|Q9RY6	tr|Q9RY6	1702	94885000	59393000	72674000	27821000	38521000	28879000
sp|Q9RSZ	sp|Q9RSZ	159	6.82E+08	6.04E+08	8.84E+08	7.2E+08		
tr|Q9RW8	tr|Q9RW8	1367	3.07E+08	1.91E+08	2.2E+08	6.68E+08	8.97E+08	7.58E+08
tr|Q9RW8	tr|Q9RW8	1367	15136000		20740000			15787000
tr|Q9RX72	tr|Q9RX72	1542		19472000	17523000	8300400		
tr|Q9RY54	tr|Q9RY54	1696	10961000			10182000	11529000	
tr|Q9RUC	tr|Q9RUC	1033	28163000	21787000	22593000	25765000		23626000
tr|Q9RVG	tr|Q9RVG	1227	8801400	20962000	12577000	22339000	27833000	19673000
tr|Q9RRY	tr|Q9RRY	608						
tr|Q9RUP	tr|Q9RUP	1099	9.59E+08	8.99E+08	9.6E+08	1.6E+09	1.42E+09	1.29E+09
tr|Q9RVN	tr|Q9RVN	1264				53010000	42101000	44874000
tr|Q9RVN	tr|Q9RVN	1264	89446000	48210000	1.24E+08	1.27E+08	37491000	2.37E+08
tr|Q9RVN	tr|Q9RVN	1264				3.74E+08	3.42E+08	4.83E+08
tr|Q9RZE4	tr|Q9RZE4	1914	16066000	8522700	7635900			
tr|Q9RVK	tr|Q9RVK	1249				16082000		
tr|Q9RYB	tr|Q9RYB	1722	72925000	59619000	53348000	1.74E+08	1.72E+08	1.87E+08
sp|Q9RW	sp|Q9RW	311	15469000		12142000	20514000	20635000	
tr|Q9RTP	tr|Q9RTP	908	18638000			22056000	24348000	
sp|Q9RUA	sp|Q9RUA	224	1.61E+08	2.01E+08	1.61E+08	95577000	90078000	95073000
tr|Q9RT11	tr|Q9RT11	799	81678000	85895000	90967000	1.38E+08	1.3E+08	1.37E+08
tr|Q9RVE	tr|Q9RVE	1220	41986000	27689000	30519000	29126000	25054000	25945000
tr|Q9RT11	tr|Q9RT11	799				16262000		
tr|Q9RY0	tr|Q9RY0	1678						
tr|Q9RT02	tr|Q9RT02	793						
tr|Q9RSM	tr|Q9RSM	738					23749000	21672000
sp|Q9X71	sp|Q9X71	462	2.35E+08	1.19E+08	1.07E+08	69781000	49477000	73638000
tr|Q9RYV	tr|Q9RYV	1822	56528000	37683000		78820000	82245000	1.33E+08
tr|Q9RVT	tr|Q9RVT	1296	53738000	46277000	51568000	69695000	66704000	63204000
tr|Q9RYI3	tr|Q9RYI3	1761						
tr|Q9RWN	tr|Q9RWN	1442	33372000	39297000	33529000	86500000	70592000	75105000
sp|Q9RVI	sp|Q9RVI	289						
tr|Q9RS87	tr|Q9RS87	663	1.13E+09	1.79E+09	1.37E+09	1.87E+09	2.52E+09	2.05E+09
tr|Q9RS87	tr|Q9RS87	663	2.8E+08	3.26E+08	4.26E+08	43969000	94693000	1.21E+08
sp|Q9RY5	sp|Q9RY5	419						
tr|Q9RS87	tr|Q9RS87	663	70850000	78408000	53076000	2.22E+08	1.59E+08	1.99E+08
tr|Q9RU5	tr|Q9RU5	997	21916000	24868000	30697000	71219000	58350000	66543000
tr|Q9RU5	tr|Q9RU5	997	90437000	90263000	2.01E+08	1.7E+08	1.34E+08	2.34E+08
tr|Q9RXK	tr|Q9RXK	1608	92855000	92835000	73927000	70570000	64871000	74986000
tr|Q9RU6	tr|Q9RU6	1004	19398000	23510000	20247000	12455000	14524000	12226000
tr|Q9RTJ4	tr|Q9RTJ4	884	6.12E+08	5.87E+08	5.93E+08	8.99E+08	7.62E+08	8.25E+08
tr|Q9RY6	tr|Q9RY6	1702						13583000
sp|P5686	sp|P5686	56	1.22E+08	1.1E+08	99571000	81551000	63896000	87588000
tr|Q9RYE	tr|Q9RYE	1736	5.11E+08	5.96E+08	4.91E+08	4.98E+08	4.82E+08	4.93E+08
sp|Q9RWJ	sp|Q9RWJ	334						12252000
sp|Q9RS3	sp|Q9RS3	109	48281000	53561000	46007000	99212000	80179000	76253000
tr|Q9RYQ	tr|Q9RYQ	1792	93277000	1.25E+08	1.3E+08	9.46E+08	6.36E+08	7.08E+08
sp|Q9RXC	sp|Q9RXC	367	3.76E+08	3.24E+08	3.26E+08	2.98E+08	2.44E+08	2.87E+08
tr|Q9RWT	tr|Q9RWT	1473				1.33E+08	1.37E+08	1.44E+08

tr|Q9RRN	tr|Q9RRN	553	52775000	52454000	39725000	69285000	71295000	81062000
sp|Q9RYV	sp|Q9RYV	445		29780000	31368000	62415000	30372000	36340000
sp|Q9RV5	sp|Q9RV5	265	4525000	2011900	1699200	2285600	2702200	2568000
sp|Q9RV5	sp|Q9RV5	265		48561000	61418000	86299000	3.73E+08	4.1E+08
tr|Q9RVV	tr|Q9RVV	1304			8290800	12651000	7869100	
sp|Q9RUE	sp|Q9RUE	229	2583200	77395000	82569000	36531000	63473000	50755000
sp|Q9RY7	sp|Q9RY7	423	67164000	60529000	80137000	58030000	60665000	70658000
tr|Q9RUG	tr|Q9RUG	1053	59960000	1.04E+08	47367000			58994000
sp|Q9RSC	sp|Q9RSC	115	28534000		35995000		33087000	
sp|Q9RWJ	sp|Q9RWJ	333						
tr|Q9RWH	tr|Q9RWH	1409	1.28E+08		99029000	2.33E+08	1.6E+08	2.02E+08
tr|Q9RWH	tr|Q9RWH	1409			37392000	55222000	39567000	34948000
tr|Q9RX37	tr|Q9RX37	1521	4.59E+08	4.91E+08	4.88E+08	3.75E+08	3.39E+08	3.16E+08
sp|Q9RVM	sp|Q9RVM	291					47613000	
tr|Q9RV57	tr|Q9RV57	1181						
tr|Q9RUP	tr|Q9RUP	1098						11147000
tr|Q9RR98	tr|Q9RR98	481		14603000	48396000		49139000	
tr|Q9RVG	tr|Q9RVG	1228					29093000	26687000
tr|Q9RXS7	tr|Q9RXS7	1640	1565000			269260	316250	323740
tr|Q9RRY5	tr|Q9RRY5	608	79019000	99721000	1.04E+08	80392000	72682000	55825000
sp|Q9RSE	sp|Q9RSE	116				13261000	14551000	
tr|Q9RS0C	tr|Q9RS0C	618	20573000	17563000	14626000	17510000	13140000	17864000
tr|Q9RW2	tr|Q9RW2	1344				56663000	58140000	72177000
tr|Q9RRV	tr|Q9RRV	587	5.96E+08	4.41E+08	6.1E+08	3.71E+08	5.1E+08	5.91E+08
tr|Q9RRV	tr|Q9RRV	587					33292000	20617000
tr|Q9RYC6	tr|Q9RYC6	1727	16269000	14731000	9928200	16773000	16622000	10461000
tr|Q9RYC6	tr|Q9RYC6	1727						
tr|Q9RRC5	tr|Q9RRC5	500	4.88E+08	3.27E+08	6.22E+08	1.82E+08	2.09E+08	1.58E+08
sp|Q9RU7	sp|Q9RU7	214	1.32E+08	1.76E+08	1.69E+08	54262000	75085000	29759000
tr|Q9RVA7	tr|Q9RVA7	1203						
tr|Q9RR62	tr|Q9RR62	467	1.28E+09	1.44E+09	1.38E+09	7.05E+08	7.97E+08	8.6E+08
tr|Q9RW2	tr|Q9RW2	1342	15104000	11308000	11413000	25970000		26410000
sp|Q5933	sp|Q5933	63			31681000		51096000	
sp|Q9WXF	sp|Q9WXF	461			4020500		6787800	
tr|Q9RS41	tr|Q9RS41	641	1.84E+08	47649000	15489000		24528000	2.3E+08
tr|Q9RUK	tr|Q9RUK	1078						
sp|Q9RRB	sp|Q9RRB	77	1.4E+08	1.24E+08	1.27E+08	1.48E+08	1.36E+08	1.81E+08
tr|Q9RVL9	tr|Q9RVL9	1254	1.52E+08	1.4E+08	1.18E+08	1.15E+08	86344000	1.13E+08
tr|Q9RTF7	tr|Q9RTF7	868	6757900	54349000	71153000	1.03E+08	54896000	82845000
tr|Q9RWN	tr|Q9RWN	1442	45847000	37647000	46879000			
tr|Q9RYA6	tr|Q9RYA6	1720	1.07E+08	69649000	63555000			
tr|Q9RVP5	tr|Q9RVP5	1269				57901000		67167000
sp|Q9RUP	sp|Q9RUP	245	4.1E+08	3.44E+08	3.17E+08			
tr|Q9RYX6	tr|Q9RYX6	1835	2.99E+08	2.78E+08	2.41E+08	2.94E+08	3.16E+08	2.88E+08
tr|Q9RVE5	tr|Q9RVE5	1219	54686000				1.96E+08	
tr|Q9RZ99	tr|Q9RZ99	1886	74471000	74875000	95089000			
sp|Q9RVC	sp|Q9RVC	277	22674000	36157000	29000000			
tr|Q9RRX1	tr|Q9RRX1	601	4.78E+08	8.16E+08	8.23E+08		13206000	6.44E+08
tr|Q9RWA	tr|Q9RWA	1379						
sp|Q9RTD	sp|Q9RTD	179	81676000	1.19E+08	88148000	1.07E+08	1.35E+08	93365000
sp|Q9RTD	sp|Q9RTD	179	56285000	74663000	1.84E+08			32442000

tr|Q9RT94	tr|Q9RT94	840						
tr|Q9RW5	tr|Q9RW5	1357	8642100	16725000	16746000	70827000	60883000	53677000
tr|Q9RRM	tr|Q9RRM	548	88237000	1.14E+08	1.09E+08	4.51E+08	5.08E+08	2.39E+08
tr|Q9RRM	tr|Q9RRM	548	42553000		44825000		12101000	
sp|Q9RRB	sp|Q9RRB	77	6.13E+08	3.37E+08	3.34E+08	6.29E+08	6.32E+08	6.46E+08
sp|O3250	sp|O3250	46						
tr|Q9RUB	tr|Q9RUB	1030		29542000	21879000			11619000
tr|Q9RSA3	tr|Q9RSA3	674						
sp|Q9RTG	sp|Q9RTG	184		34786000		55293000	55692000	49715000
tr|Q9RY75	tr|Q9RY75	1705	13228000					
tr|Q9RRS5	tr|Q9RRS5	576						
sp|Q9RSZ	sp|Q9RSZ	159		1.25E+08		1.69E+08	1.69E+08	1.77E+08
tr|Q9RTX4	tr|Q9RTX4	949	15073000	24242000	20437000	34469000	46806000	39134000
sp|Q9RTN	sp|Q9RTN	196	31289000	40323000	37089000	80205000	61133000	56629000
sp|Q9RU7	sp|Q9RU7	213	1.48E+08	97647000	1.04E+08	35533000	56554000	47966000
tr|Q9RYX6	tr|Q9RYX6	1835	4.2E+08	4.56E+08	3.16E+08	5.28E+08	4.83E+08	4.86E+08
tr|Q9RTQ8	tr|Q9RTQ8	918						
tr|Q9RZV3	tr|Q9RZV3	1983	3194200		11886000			
tr|Q9RR98	tr|Q9RR98	481	32315000		27214000	59887000	44321000	50186000
tr|Q9RUL0	tr|Q9RUL0	1082						
tr|Q9RZ33	tr|Q9RZ33	1861						
sp|Q9RXI7	sp|Q9RXI7	373			3170300	3732800		
sp|Q9RXI7	sp|Q9RXI7	373						
tr|Q9RU00	tr|Q9RU00	968	1.21E+08	1.59E+08	1.37E+08	1.03E+08	76137000	80609000
tr|Q9RXD5	tr|Q9RXD5	1573			21807000			59494000
tr|Q9RVY9	tr|Q9RVY9	1323				18254000		13526000
tr|Q9RTU7	tr|Q9RTU7	935	6.58E+08	6.12E+08	4.55E+08	7.92E+08	5.97E+08	6.77E+08
tr|Q9RRU9	tr|Q9RRU9	586	1.94E+08	1.73E+08	1.69E+08	5.67E+08	5.54E+08	4.75E+08
tr|Q9RS25	tr|Q9RS25	633	1.12E+08	1.14E+08	1.08E+08	2.55E+08	2.31E+08	2E+08
sp|Q9RRM	sp|Q9RRM	91	72928000	73021000	70828000	37565000		45101000
tr|Q9RUT9	tr|Q9RUT9	1120						
tr|Q9RX00	tr|Q9RX00	1504	63164000	61462000			51259000	41401000
tr|Q9RVV0	tr|Q9RVV0	1304	55717000	58524000	54009000	44596000	38308000	37868000
sp|Q9RR6	sp|Q9RR6	65	2.74E+08	3.6E+08	3.09E+08	5.03E+08	5.66E+08	3.78E+08
tr|Q9RVN	tr|Q9RVN	1264	6.15E+09	4.92E+09	4.82E+09	2.26E+09	2.12E+09	2.54E+09
tr|Q9RVP9	tr|Q9RVP9	1270	6112900	17290000	9684900	47083000		31266000
tr|Q9RRI4	tr|Q9RRI4	532	28178000	14600000		49157000	63768000	50883000
tr|Q9RYQ3	tr|Q9RYQ3	1791			49309000	1.72E+08	1.37E+08	1.46E+08
tr|Q9RTZ9	tr|Q9RTZ9	967			17242000	95320000	84474000	69537000
tr|Q9RTZ9	tr|Q9RTZ9	967				15888000	27247000	16201000
tr|Q9RTR4	tr|Q9RTR4	923				38585000		34425000
sp|Q9RS3	sp|Q9RS3	109		2.02E+08	1.74E+08	2.87E+08	3.25E+08	3E+08
tr|Q9RUJ4	tr|Q9RUJ4	1070	61588000	35370000	31732000			
tr|Q9RSE2	tr|Q9RSE2	701						31603000
tr|Q9RSE2	tr|Q9RSE2	701		97461000	1.33E+08	1.01E+08	1.06E+08	1.11E+08
tr|Q9RS63	tr|Q9RS63	652	11642000		8218900	1.11E+08	88487000	93784000
tr|Q9RTL6	tr|Q9RTL6	897	3.14E+08	2.75E+08	2.43E+08	4.27E+08	5.78E+08	2.96E+08
tr|Q9RU27	tr|Q9RU27	980	1.88E+08	3.05E+08	2.78E+08	1.69E+08	2.65E+08	1.89E+08
tr|Q9RYB8	tr|Q9RYB8	1722	1.04E+08	2.01E+08	2.42E+08	1.81E+08	2.55E+08	2.04E+08
tr|Q9RYX2	tr|Q9RYX2	1833	50115000	64503000	55714000	8914200	9747100	
tr|Q9RYI1	tr|Q9RYI1	1760	41638000	26225000	40235000			

tr|Q9RYV	tr|Q9RYV	1820	61051000	7323000	7735700	23258000	26386000	79956000
sp|Q9RUF	sp|Q9RUF	231	32437000	49973000	54527000		24453000	26517000
tr|Q9RZ3	tr|Q9RZ3	1859		20926000	12953000	13650000	30543000	23279000
tr|Q9RUG	tr|Q9RUG	1052				13187000	10389000	
tr|Q9RUG	tr|Q9RUG	1052		1942100	2954200	6637400	4125800	3838400
tr|Q9RSA	tr|Q9RSA	676	1.74E+08	1.64E+08	2.65E+08	2.52E+08	1.28E+08	1.93E+08
tr|Q9RT87	tr|Q9RT87	837	2473400		473580			
sp|Q9RW	sp|Q9RW	345	2.85E+08	2.68E+08	2.24E+08	1.85E+08	1.5E+08	1.91E+08
sp|Q9RR	sp|Q9RR	91						
sp|Q9RYB	sp|Q9RYB	426					751080	
sp|Q9RYB	sp|Q9RYB	426				10812000	55507000	58443000
tr|Q9RY21	tr|Q9RY21	1682					6768300	
sp|Q9RW	sp|Q9RW	327	34374000	15357000	14921000	8583800	14277000	14771000
sp|Q9RUF	sp|Q9RUF	231	2.1E+08	2.3E+08	2.68E+08	3.46E+08	2.82E+08	3.07E+08
sp|Q9RXV	sp|Q9RXV	403	2.97E+09	2.46E+09	2.09E+09	2.69E+09	2.89E+09	3.21E+09
tr|Q9RU4	tr|Q9RU4	991	77282000	81144000	69374000		63609000	1.01E+08
tr|Q9RWN	tr|Q9RWN	1442		40231000	33794000	1.7E+08	48175000	41746000
sp|Q9RY2	sp|Q9RY2	411		19596000	76737000	53027000	60230000	24535000
tr|Q9RSM	tr|Q9RSM	735	7552800	6973500	8314300	34725000	28852000	26771000
tr|Q9RUZ	tr|Q9RUZ	1146	2.49E+08	2.21E+08	2.03E+08	2.85E+08	2.17E+08	2.49E+08
tr|Q9RUJ4	tr|Q9RUJ4	1070	1.28E+08	1.43E+08	1.14E+08	1.96E+08	2.75E+08	1.92E+08
tr|Q9RTJ1	tr|Q9RTJ1	883	31984000	36516000	45326000	43440000	40268000	53824000
tr|Q9RYD	tr|Q9RYD	1735	2.71E+08	2.03E+08	66235000	4.78E+08	4.14E+08	3.59E+08
tr|Q9RTK	tr|Q9RTK	887						
tr|Q9RWE	tr|Q9RWE	1399						
sp|Q9RXK	sp|Q9RXK	389						
sp|Q9RY7	sp|Q9RY7	422	77458000	73244000	55862000	80875000	78559000	80702000
sp|Q9RS1	sp|Q9RS1	107	15376000			67900000	72722000	65985000
tr|Q9RZE	tr|Q9RZE	1917	62426000	89260000	80130000		39812000	75252000
tr|Q9RTP	tr|Q9RTP	906		22518000	24570000	35775000	30130000	38954000
sp|Q9RXJ	sp|Q9RXJ	382	4.1E+08	3.9E+08	3.31E+08	8.46E+08	6.16E+08	7.31E+08
sp|Q9RXJ	sp|Q9RXJ	382		6208900	12031000	51689000	82707000	82562000
tr|Q9RZ87	tr|Q9RZ87	1879				1.8E+08	1.48E+08	
tr|Q9RX71	tr|Q9RX71	1541					35943000	
tr|Q9RUB	tr|Q9RUB	1028	18930000	5526000	5822200		8489000	7959000
sp|P5692	sp|P5692	59						
sp|Q9RS1	sp|Q9RS1	107						
tr|Q9RSV	tr|Q9RSV	772				23754000	33515000	20222000
tr|Q9RZC	tr|Q9RZC	1904	96549000	1.01E+08	1.04E+08	1.75E+08	1.89E+08	1.58E+08
tr|Q9RTU	tr|Q9RTU	934	89976000	92276000	1.07E+08	94522000	80902000	1.03E+08
sp|Q9RSR	sp|Q9RSR	144				5106200		
tr|Q9RUR	tr|Q9RUR	1109			42802000			
sp|Q9RVA	sp|Q9RVA	274			6736300	20922000	52627000	24742000
tr|Q9RRU	tr|Q9RRU	586	54385000		38621000			71502000
tr|Q9RW5	tr|Q9RW5	1357	20274000	24665000				
tr|Q9RYA	tr|Q9RYA	1720				22436000		22711000
tr|Q9RVG	tr|Q9RVG	1230		41353000	30504000			
sp|Q9RUR	sp|Q9RUR	249	28261000	9031000	8027700	3276700	6115800	4138200
tr|Q9RWX	tr|Q9RWX	1497						7386300
tr|Q9RW7	tr|Q9RW7	1362				16851000	17710000	18098000
tr|Q9RW8	tr|Q9RW8	1371	11344000	9876600	16088000			

tr|Q9RWR	tr|Q9RWR	1459						
tr|Q9RVW	tr|Q9RVW	1311	25822000	28391000	22345000	1.36E+08	90576000	1.1E+08
tr|Q9RWC	tr|Q9RWC	1392	8554800	15119000	12989000	39099000		16889000
tr|Q9RTD	tr|Q9RTD	857	53011000	51041000	41217000	83481000	59469000	62623000
tr|Q9RU2	tr|Q9RU2	980	2.51E+08	3.01E+08	2.25E+08	2.28E+08	1.73E+08	2.21E+08
tr|Q9RU5	tr|Q9RU5	997	27455000	29210000	64603000	94673000	49118000	65839000
sp|Q9RT9	sp|Q9RT9	172			4041100	11508000	9983400	9866900
sp|P5686	sp|P5686	56	5.43E+08		4.2E+08	1.29E+09	1.13E+09	1.12E+09
tr|Q9RUY	tr|Q9RUY	1139	2.49E+08	3.13E+08	1.55E+08	1.9E+08	2.05E+08	1.93E+08
tr|Q9RST1	tr|Q9RST1	757	4.43E+08	4.15E+08	3.52E+08	6.58E+08	5.96E+08	5.97E+08
tr|Q9RW6	tr|Q9RW6	1359				36338000	25539000	
sp|Q9RUF	sp|Q9RUF	230	61448000	54268000		86398000	80164000	82367000
tr|Q9RXG	tr|Q9RXG	1587	71241000	62769000	61859000	1.05E+08	89345000	93531000
tr|Q9RSP	tr|Q9RSP	743						
sp|Q9RX3	sp|Q9RX3	357	18162000				16365000	20017000
tr|Q9RX1	tr|Q9RX1	1511			5386000			
sp|Q9RS6	sp|Q9RS6	111	89497000	1.08E+08	78059000	1.12E+08	1.08E+08	1.04E+08
sp|Q9RXY	sp|Q9RXY	406			10546000			
sp|Q9RWI	sp|Q9RWI	332	4836000		3175000		2542200	3679600
sp|Q9RTA	sp|Q9RTA	174	23158000	15827000	23230000	19011000		
sp|Q9RVT	sp|Q9RVT	296	59457000	47326000	38806000	31290000	26941000	30602000
tr|Q9RZ9	tr|Q9RZ9	1886	43833000	52905000	43362000	30811000	40689000	31994000
tr|Q9RYC	tr|Q9RYC	1723				1.2E+08	1.33E+08	
sp|Q9RV9	sp|Q9RV9	273	48876000	35153000	61375000	57406000	69580000	82094000
sp|Q9RW	sp|Q9RW	343	1.34E+08	93952000	94040000	47918000	57370000	75519000
sp|Q9RSQ	sp|Q9RSQ	140				22746000	17287000	
tr|Q9RYD	tr|Q9RYD	1730						
tr|Q9RTE1	tr|Q9RTE1	860				18941000	21909000	17962000
sp|Q9RU	sp|Q9RU	254	46866000	52946000	78772000	3.18E+08	4.8E+08	1.87E+08
tr|Q9RW2	tr|Q9RW2	1345	1.48E+08	1.16E+08	1.1E+08	1.01E+08	94835000	97271000
tr|Q9RYI7	tr|Q9RYI7	1763		18775000	21192000	15275000		17924000
tr|Q9RS71	tr|Q9RS71	655	2.11E+08	1.95E+08	1.79E+08	2.73E+08	2.58E+08	2.21E+08
tr|Q9RTZ	tr|Q9RTZ	967				52089000	36373000	28044000
tr|Q9RVQ	tr|Q9RVQ	1272	27252000	61343000	37916000			
tr|Q9RVQ	tr|Q9RVQ	1272	21413000	38803000	73022000			
tr|Q9RR6	tr|Q9RR6	469						
tr|Q9RTT	tr|Q9RTT	930	1.12E+09	9.97E+08	1.05E+09	1.32E+09	1.13E+09	1.02E+09
tr|Q9RT2	tr|Q9RT2	806	23057000	25345000	20412000	78772000		45187000
sp|Q9R34	sp|Q9R34	64						
tr|Q9RVB	tr|Q9RVB	1206	4866200	7136200	8921300			
tr|Q9RTU	tr|Q9RTU	935						
tr|Q9RWS	tr|Q9RWS	1461	30046000	1.12E+08	39367000	25591000	39183000	70250000
tr|Q9RX8	tr|Q9RX8	1548					3.79E+08	3.32E+08
tr|Q9RT7	tr|Q9RT7	831	1.78E+08	2.13E+08	2.14E+08	3.15E+08	3.47E+08	2.2E+08
tr|Q9RXX	tr|Q9RXX	1660						46969000
sp|Q9RSJ	sp|Q9RSJ	125						
sp|Q9RSJ	sp|Q9RSJ	125	23807000	22158000	59333000		18821000	16266000
tr|Q9RU9	tr|Q9RU9	1016		25380000		13582000	20425000	13687000
tr|Q9RT5	tr|Q9RT5	819						
tr|Q9RYA	tr|Q9RYA	1721	5.15E+08	4.37E+08	3.06E+08	7.18E+08	6.32E+08	6.35E+08
sp|Q9RV	sp|Q9RV	301	12196000	8209300	9495400	9587200	11328000	13135000

tr|Q9RTE5	tr|Q9RTE5	863	1.29E+08	1.67E+08	1.28E+08	1.63E+08	1.3E+08	1.58E+08
sp|Q9RW(	sp|Q9RW(	342		37632000	99196000	32868000	37168000	68982000
sp|Q9RW(	sp|Q9RW(	342				40071000	42774000	61833000
sp|Q9RS1	sp|Q9RS1	107	60676000	1.06E+08	83754000	1.35E+08	1.17E+08	1.19E+08
sp|Q9RS1	sp|Q9RS1	107			15594000	10048000	13066000	23299000
tr|Q9RYN4	tr|Q9RYN4	1778	47367000	53590000	45270000	64103000	55381000	47403000
tr|Q9RW0	tr|Q9RW0	1333	3.15E+08	1.88E+08	1.69E+08	4.74E+08	2.29E+08	3.7E+08
tr|Q9RT45	tr|Q9RT45	816						
sp|Q9RT2	sp|Q9RT2	162						
sp|Q9RT2	sp|Q9RT2	162		17911000	31677000	13159000	36038000	20802000
sp|Q9RY0	sp|Q9RY0	410				91596000	1.28E+08	64203000
tr|Q9RZ32	tr|Q9RZ32	1860	17818000	26969000	25614000	49043000	70080000	
tr|Q9RYT5	tr|Q9RYT5	1814	28864000	22659000	19230000	85157000	94420000	77389000
sp|Q9RXG	sp|Q9RXG	370		22658000	12966000	13428000	16292000	
tr|Q9RXI1	tr|Q9RXI1	1601	2.44E+08	2.47E+08	2.43E+08	5.46E+08	5.98E+08	5E+08
tr|Q9RXQ	tr|Q9RXQ	1629	1.42E+08	1.55E+08	92879000	1.68E+08	1.54E+08	1.65E+08
tr|Q9RTG7	tr|Q9RTG7	873	1.37E+08	1.11E+08	81556000	1.14E+08	84056000	1.06E+08
sp|Q9RYM	sp|Q9RYM	438						
sp|Q9RUE	sp|Q9RUE	225	42392000	44847000	45478000	21202000	21711000	
tr|Q9RX07	tr|Q9RX07	1506	29450000	30474000		40048000	26373000	29942000
tr|Q9RYH9	tr|Q9RYH9	1758			32590000		11723000	15543000
tr|Q9RWT	tr|Q9RWT	1471	88871000	84440000	81932000		1.08E+08	
tr|Q9RTX0	tr|Q9RTX0	947	63730000	52596000	68448000	22473000	28729000	36053000
sp|Q9RVW	sp|Q9RVW	301	1.57E+08	1.25E+08	1.86E+08	1.29E+08	1.21E+08	1.89E+08
sp|Q9RT5	sp|Q9RT5	168	1.43E+08	1.1E+08	97965000	1.44E+08	1.43E+08	1.55E+08
sp|Q9RXT	sp|Q9RXT	400	2048700		1020800	764760	1074100	1071300
tr|Q9RW8	tr|Q9RW8	1370	92079000	78374000	72670000	2.66E+08		1.99E+08
tr|Q9RTR8	tr|Q9RTR8	925			24745000			
sp|Q9RW(	sp|Q9RW(	322	11936000		7197300	9550300	13433000	9308300
tr|Q9RVX7	tr|Q9RVX7	1318	20097000	89139000	43115000	82148000	1.02E+08	1.02E+08
tr|Q9RVX7	tr|Q9RVX7	1318				28646000	46851000	57629000
tr|Q9RV32	tr|Q9RV32	1168	3.71E+08	3.54E+08	3.04E+08	1.93E+08	1.89E+08	2.74E+08
sp|Q9RSL4	sp|Q9RSL4	136	38510000	11593000	16464000	12234000	12433000	16986000
sp|Q9RY5	sp|Q9RY5	419			17221000			14009000
sp|Q9R34	sp|Q9R34	64				2958500	5857500	4041900
tr|Q9RY34	tr|Q9RY34	1687	2.13E+08	1.78E+08	1.59E+08	8.7E+08	5.69E+08	4.73E+08
tr|Q9RT97	tr|Q9RT97	843	27426000	19992000	24396000	24982000	43694000	
sp|Q9RWI	sp|Q9RWI	328					39891000	
sp|Q9RWI	sp|Q9RWI	335	68497000	33127000	46814000		68550000	
tr|Q9RYH(	tr|Q9RYH(	1756						
tr|Q9RUR4	tr|Q9RUR4	1107	1.61E+08	1.9E+08	3.8E+08	2.9E+08	3.08E+08	4.44E+08
tr|Q9RZB2	tr|Q9RZB2	1895	99052000	1.36E+08	94607000	72607000	75664000	79563000
sp|Q9RSI9	sp|Q9RSI9	122	2.93E+08	2.93E+08	2.51E+08	4.77E+08	2.41E+08	4.52E+08
sp|Q9RUP	sp|Q9RUP	245						10352000
sp|Q9RUG	sp|Q9RUG	235			7956700	11726000	17740000	12711000
tr|Q9RVN	tr|Q9RVN	1261						
tr|Q9RVN	tr|Q9RVN	1261			22495000		9038500	
tr|Q9RVS1	tr|Q9RVS1	1287	1.65E+09	1.45E+09	1.2E+09	2.12E+09	1.91E+09	1.99E+09
tr|Q9RVS1	tr|Q9RVS1	1287		48724000	88413000	19286000	77464000	17109000
tr|Q9RRG4	tr|Q9RRG4	520	7.95E+08	1.01E+09	8.33E+08	1.11E+09	8.56E+08	1.01E+09
tr|Q9RXG(	tr|Q9RXG(	1587				63934000	97065000	50530000

tr|Q9RZP3	tr|Q9RZP3	1953	1.04E+08	1.32E+08	1.26E+08	1.07E+08	96824000	1.02E+08
sp|Q9RTF5	sp|Q9RTF5	182	1.34E+09	1.2E+09	1.12E+09	1.81E+09	1.6E+09	1.63E+09
sp|P42443	sp|P42443	49						
sp|Q9RW0	sp|Q9RW0	327	37001000	83310000	1.39E+08	64469000	71340000	55478000
tr|Q9RS17	tr|Q9RS17	630	3.79E+08	4.41E+08	4.42E+08	3.72E+08	3.27E+08	5E+08
tr|Q9RZC7	tr|Q9RZC7	1906	1.36E+08	92543000	85325000	3.91E+08	3.03E+08	3.03E+08
tr|Q9RZC7	tr|Q9RZC7	1906	1.12E+08	1.18E+08	1.39E+08	1.49E+08	1.31E+08	1.73E+08
tr|Q9RTE3	tr|Q9RTE3	862						
tr|Q9RRI3	tr|Q9RRI3	531	80605000	69136000	64993000	1.78E+08	1.61E+08	1.37E+08
sp|Q9RVC	sp|Q9RVC	279	42466000	25244000	25333000		17656000	
tr|Q9RST2	tr|Q9RST2	758	18063000		24315000			
tr|Q9RXK9	tr|Q9RXK9	1608	1.51E+08	1.82E+08	1.41E+08	2.7E+08	2.48E+08	2.32E+08
tr|Q9RYK8	tr|Q9RYK8	1766					18321000	16232000
tr|Q9RTJ1	tr|Q9RTJ1	883	35807000	15170000	15497000	43767000	63365000	46517000
sp|Q9RW	sp|Q9RW	335		22399000	29456000			
tr|Q9RVK9	tr|Q9RVK9	1249	9482200	8906500	6017100	6723300	9740800	6740200
tr|Q9RUB	tr|Q9RUB	1027	6.37E+08	8.34E+08	4.18E+08	2.04E+09	3.45E+09	1.48E+09
tr|Q9RTR5	tr|Q9RTR5	924	22814000	39753000	48813000	32856000	36602000	50227000
sp|Q9RT4	sp|Q9RT4	167	91238000	89679000	76154000	1E+08	26525000	1.17E+08
tr|Q9RSM	tr|Q9RSM	737	5.44E+08	5.62E+08	4.41E+08	8.15E+08	7.53E+08	8.05E+08
tr|Q9RRG1	tr|Q9RRG1	518	6.44E+08	5.6E+08	4.8E+08	4.29E+08	4.17E+08	4.3E+08
tr|Q9RRE6	tr|Q9RRE6	512						
sp|Q9RRJ1	sp|Q9RRJ1	86	2991900			20258000		15953000
sp|Q9RXJ0	sp|Q9RXJ0	375	1.72E+08	2.71E+08	4.1E+08	3.06E+08	3.26E+08	3.68E+08
tr|Q9RRB2	tr|Q9RRB2	493				8369700		
tr|Q9RR83	tr|Q9RR83	475	63550000	54199000	61471000	92614000	71472000	83924000
tr|Q9RXI1	tr|Q9RXI1	1601			42978000			
sp|Q9RTL8	sp|Q9RTL8	194	13853000	5916800	5155200	5512500	7330900	6242300
tr|Q9RYQ0	tr|Q9RYQ0	1788	76222000	1.19E+08	2.17E+08	2.06E+08	1.5E+08	1.94E+08
tr|Q9RT60	tr|Q9RT60	824	51611000		33407000		94173000	
tr|Q9RYX2	tr|Q9RYX2	1833	13766000					
sp|Q9RRJ6	sp|Q9RRJ6	89				57961000	53700000	11325000
tr|Q9RWB	tr|Q9RWB	1385					44693000	58249000
tr|Q9RUQ	tr|Q9RUQ	1104			17768000			
tr|Q9RXB1	tr|Q9RXB1	1562		57009000	46083000	21917000		24871000
tr|Q9RVW	tr|Q9RVW	1311	48602000	50726000	62525000	19178000	16705000	20347000
sp|Q9RY7	sp|Q9RY7	423	32341000	79722000	80135000	65663000	60034000	38572000
tr|Q9RTZ0	tr|Q9RTZ0	961			49697000	48578000	42447000	18557000
sp|Q9RW1	sp|Q9RW1	306	1.19E+08	1.13E+08	1.09E+08	1.39E+08	1.4E+08	1.18E+08
sp|Q9RV9	sp|Q9RV9	272	3143100		6024300	2071900	4877200	4660700
sp|Q9RY8	sp|Q9RY8	424						
tr|Q9RXF5	tr|Q9RXF5	1584						
sp|Q9RV8	sp|Q9RV8	270	70919000	55026000		61540000	64465000	47011000
sp|Q9RSP	sp|Q9RSP	139	34952000	32683000	27032000	1.21E+08		
tr|Q9RV15	tr|Q9RV15	1159			57064000	68876000		71451000
tr|Q9RXC5	tr|Q9RXC5	1570	9428300	77216000	1.19E+08		56608000	39700000
tr|Q9RWW	tr|Q9RWW	1492	67211000	47976000	46181000	20321000	28871000	30932000
tr|Q9RSZ0	tr|Q9RSZ0	788	37956000	1.06E+08	73080000	97406000	1.41E+08	1.28E+08
tr|Q9RY74	tr|Q9RY74	1704		4729800	2447000	5408500	4301800	4986600
tr|Q9RY74	tr|Q9RY74	1704						
tr|Q9RVL0	tr|Q9RVL0	1250	39308000	20727000	17587000	20970000	12738000	20810000

tr|Q9RRA7	tr|Q9RRA7	490	6623800					
sp|Q9RT9	sp|Q9RT9	172						
tr|Q9RZL9	tr|Q9RZL9	1946	34799000	23501000		24970000	50168000	46609000
tr|Q9RW2	tr|Q9RW2	1345			27831000			
sp|Q9RYM	sp|Q9RYM	439	44406000	72409000	83300000	20025000		19716000
tr|Q9RYS7	tr|Q9RYS7	1806	52640000	64293000	51782000	1.49E+08	1.45E+08	1.38E+08
sp|Q9RS6	sp|Q9RS6	112	3.03E+08	2.18E+08	2.45E+08	1.24E+08	1.37E+08	1.23E+08
tr|Q9RSZ5	tr|Q9RSZ5	790						
sp|Q9RT6	sp|Q9RT6	170						
tr|Q9RVD2	tr|Q9RVD2	1214	17524000	28996000	15301000	40572000	51878000	26713000
tr|Q9RZH3	tr|Q9RZH3	1929						
tr|Q9RRK9	tr|Q9RRK9	543				22249000	45489000	
tr|Q9RYB8	tr|Q9RYB8	1722	2.98E+08	2.94E+08	2.49E+08	3.35E+08	2.84E+08	2.59E+08
tr|Q9RWA	tr|Q9RWA	1380	43452000			24335000		
sp|Q9RRX	sp|Q9RRX	101	47675000	13329000	52433000	1.76E+08	1.79E+08	1.35E+08
tr|Q7DF71	tr|Q7DF71	464						15543000
sp|O8302	sp|O8302	47	3.29E+08	3.9E+08	3.08E+08	2.76E+08	2.48E+08	2.52E+08
tr|Q9RX73	tr|Q9RX73	1543	99148000	1.06E+08	1.21E+08	49318000	81971000	37410000
sp|Q9RRB	sp|Q9RRB	77	1.14E+09	1.13E+09	1.05E+09	2.67E+09	2.49E+09	2.1E+09
sp|Q9RY4	sp|Q9RY4	415	2.27E+08	2.06E+08	1.78E+08	3.49E+08	3.79E+08	3.49E+08
sp|Q9RY4	sp|Q9RY4	415	22467000	46443000	88493000	24977000	78537000	60544000
tr|Q9RSD4	tr|Q9RSD4	696	15043000					
tr|Q9LBE3	tr|Q9LBE3	466	50766000	60165000		93545000	79120000	74414000
tr|Q9RRL4	tr|Q9RRL4	547				32363000	35301000	28408000
tr|Q9RXL5	tr|Q9RXL5	1611	72979000		1.21E+08			
tr|Q9RZP6	tr|Q9RZP6	1954						
tr|Q9RS66	tr|Q9RS66	653	1.01E+09	1.11E+09	1.04E+09	1.37E+09	1.3E+09	1.19E+09
sp|Q9RS6	sp|Q9RS6	112	19862000	16163000	15849000	24353000	25258000	22520000
sp|Q9RS6	sp|Q9RS6	112	2.86E+08	2.83E+08	4.81E+08	1.12E+08	73437000	1.58E+08
tr|Q9RY83	tr|Q9RY83	1709	12960000	4709800	24027000		21376000	
sp|Q9RX5	sp|Q9RX5	359				6375900		3403300
tr|Q9RY97	tr|Q9RY97	1716					1.23E+08	1.15E+08
tr|Q9RY97	tr|Q9RY97	1716						
sp|Q9RUC	sp|Q9RUC	247	30890000	40822000	43676000		41961000	
tr|Q9RYE5	tr|Q9RYE5	1739	16597000					
sp|Q9RYV	sp|Q9RYV	445						
tr|Q9RTR4	tr|Q9RTR4	923			15140000		18183000	
tr|Q9RSU0	tr|Q9RSU0	762	7.05E+08	7.95E+08	5.73E+08	7.74E+08	8.47E+08	5.99E+08
sp|Q9RXG	sp|Q9RXG	370						
tr|Q9RS46	tr|Q9RS46	642	42536000	2.41E+08	2.15E+08	41685000	50596000	3.46E+08
tr|Q9RSY9	tr|Q9RSY9	787						
tr|Q9RUU	tr|Q9RUU	1121	53665000	45671000	43781000			
tr|Q9RS15	tr|Q9RS15	629	35739000	43990000	26514000	26779000	29066000	30148000
tr|Q9RVC5	tr|Q9RVC5	1211	20613000	28893000	36715000	66170000	59269000	56382000
tr|Q9RV05	tr|Q9RV05	1153	56608000	46067000	37136000		37398000	51536000
tr|Q9RSZ5	tr|Q9RSZ5	790	91860000	91245000		59679000	77083000	55363000
sp|Q9RTH	sp|Q9RTH	186	1.14E+08	1.44E+08	84530000		1.02E+08	68027000
sp|Q9RXT	sp|Q9RXT	401	1.95E+09	1.65E+09	1.54E+09	1.91E+09	1.71E+09	1.76E+09
tr|Q9RUU	tr|Q9RUU	1124	34115000	49475000	35516000	21811000		13177000
tr|Q9RRT9	tr|Q9RRT9	582	12222000		14346000			
tr|Q9RYB8	tr|Q9RYB8	1722				7557300		6553600

tr|Q9RXW	tr|Q9RXW	1656				10760000	7267100	11674000
sp|Q9RX3	sp|Q9RX3	356	30018000	35057000	22532000	41069000	43670000	39666000
sp|Q9RYW	sp|Q9RYW	446	21009000	37904000	38277000	83250000	97700000	1.01E+08
tr|Q9RTQ6	tr|Q9RTQ6	916	93310000	98601000	74990000	86665000	85849000	81530000
sp|P56905	sp|P56905	57	20586000	13311000	16057000	39992000	39265000	31447000
tr|Q9RRF2	tr|Q9RRF2	515						
tr|Q9RZ79	tr|Q9RZ79	1873						
tr|Q9RYE9	tr|Q9RYE9	1741	64418000	68895000	46015000			
tr|Q9RWI7	tr|Q9RWI7	1418	1.06E+08	47284000	50280000	68435000	26781000	54167000
sp|Q9R34	sp|Q9R34	64	6.94E+08	1.38E+09	1.81E+09	1.77E+09	2.39E+09	2.26E+09
sp|Q9R34	sp|Q9R34	64			71758000	64105000	50772000	1.38E+08
tr|Q9RRD5	tr|Q9RRD5	505			73075000			59316000
tr|Q9RUA	tr|Q9RUA	1025	27385000	16520000	21310000	4650400	9952000	
tr|Q9RXI3	tr|Q9RXI3	1602	1.26E+08	93288000	89861000	2.29E+08	1.86E+08	1.85E+08
tr|Q9RRA6	tr|Q9RRA6	489		17209000	1.41E+08			
tr|Q9RVB2	tr|Q9RVB2	1206	7822800		7847300		8325900	20778000
tr|Q9RRF7	tr|Q9RRF7	517	7.42E+08	9.2E+08	9.4E+08	9.09E+08	7.54E+08	6.35E+08
tr|Q9RXD5	tr|Q9RXD5	1573	51387000	40129000	35198000	53105000	70476000	54954000
tr|Q9RXD5	tr|Q9RXD5	1573	28863000	24753000				
tr|Q9RTJ1	tr|Q9RTJ1	883						
tr|Q9RTJ1	tr|Q9RTJ1	883	23373000		31457000			
tr|Q9RS71	tr|Q9RS71	655	5.15E+08	5.06E+08	3.85E+08	6.95E+08	6.4E+08	7.09E+08
tr|Q9RS71	tr|Q9RS71	655	1.28E+08	2.16E+08	75653000	1.1E+08	1.87E+08	1.64E+08
tr|Q9RWP	tr|Q9RWP	1446	43532000	56977000	58149000	70934000	72744000	71870000
tr|Q9RSG2	tr|Q9RSG2	711					14376000	15759000
tr|Q9RSL8	tr|Q9RSL8	734	78735000	1.38E+08	1.11E+08	3.32E+08	3.76E+08	1.44E+08
tr|Q9RVZ2	tr|Q9RVZ2	1325	44709000		4.81E+08	16565000		
tr|Q9RXU3	tr|Q9RXU3	1646	4760600	5367400		10079000	3723500	
sp|Q9RT6	sp|Q9RT6	169	64136000	56007000	60988000	1.23E+08	95630000	73396000
tr|Q9RZD6	tr|Q9RZD6	1910						
sp|P52027	sp|P52027	51			66001000	80844000	92672000	97298000
tr|Q9RZ79	tr|Q9RZ79	1873						
tr|Q9RVS0	tr|Q9RVS0	1286						
sp|Q9WXF	sp|Q9WXF	461			3504400			
sp|Q9RXX	sp|Q9RXX	405	27558000	14743000	32403000	38104000	52202000	32397000
sp|Q9RXG	sp|Q9RXG	370	51249000	59157000	41408000	98519000	1E+08	92905000
sp|Q9RXG	sp|Q9RXG	370	16512000	19904000	26256000		15471000	18163000
tr|Q9RWF	tr|Q9RWF	1402	82332000	71389000	65797000	44931000	46110000	61492000
sp|Q9RSJ5	sp|Q9RSJ5	123			15216000			
tr|Q9RVR5	tr|Q9RVR5	1284		4490100	6610600	18707000	26945000	30160000
sp|Q9RTI0	sp|Q9RTI0	187		1.19E+08		1.63E+08	1.18E+08	1.14E+08
sp|Q9RYC	sp|Q9RYC	428						
sp|Q9RWH	sp|Q9RWH	331				46033000		
tr|Q9RV31	tr|Q9RV31	1167		33494000	75379000	1.05E+08	1.34E+08	1.29E+08
tr|Q9RVS5	tr|Q9RVS5	1290	2.64E+08	2.99E+08	2.79E+08	4.7E+08	4.44E+08	3.6E+08
sp|Q9RU1	sp|Q9RU1	206	81241000	77580000	47193000	1.19E+08	1.29E+08	1.27E+08
tr|Q9RWF	tr|Q9RWF	1400	2.13E+09	1.95E+09	1.77E+09	2.22E+09	2.47E+09	2.15E+09
tr|Q9RZA0	tr|Q9RZA0	1887						
tr|Q9RZ23	tr|Q9RZ23	1855			6678600			
tr|Q9RUR6	tr|Q9RUR6	1108	17711000	18216000		14071000	11272000	
sp|Q9RUF	sp|Q9RUF	231				16315000	20049000	

tr|Q7DF92	tr|Q7DF92	465						
tr|Q9RS08	tr|Q9RS08	624	4.27E+08	3.58E+08	3.91E+08	4.12E+08	3.45E+08	4.27E+08
tr|Q9RUD	tr|Q9RUD	1040						
sp|P50933	sp|P50933	50	52120000	1E+08	81267000	1.22E+08	1.23E+08	1.42E+08
tr|Q9RW0	tr|Q9RW0	1331	63669000	63586000	57236000	71618000	80808000	88650000
tr|Q9RWW	tr|Q9RWW	1494						
tr|Q9RRX6	tr|Q9RRX6	604	28084000	14224000	14427000	7419000		
sp|Q9RS8	sp|Q9RS8	113					37208000	
tr|Q9RSE8	tr|Q9RSE8	706						
tr|Q9RTU7	tr|Q9RTU7	935	25210000	35538000	38331000		57829000	49479000
tr|Q9RR75	tr|Q9RR75	472		70480000	42505000	1.14E+08	1.01E+08	1.2E+08
tr|Q9RW2	tr|Q9RW2	1345						
sp|Q9RTJ3	sp|Q9RTJ3	189	9547200	16610000	15304000	47808000	13567000	13742000
sp|Q9RX6	sp|Q9RX6	361	2.18E+08	4.39E+08	3.43E+08	2.3E+08	3.46E+08	2.53E+08
tr|Q9RXX9	tr|Q9RXX9	1663	42956000					59362000
tr|Q9RY46	tr|Q9RY46	1694	1.4E+09	1.99E+09	1.79E+09	1.7E+09	2.34E+09	1.52E+09
tr|Q9RU26	tr|Q9RU26	979	1.21E+08	1.86E+08	1.37E+08	1.5E+08	1.81E+08	1.24E+08
tr|Q9RRV3	tr|Q9RRV3	590				21798000	18751000	11620000
tr|Q9RSU1	tr|Q9RSU1	763	1.41E+08	2.38E+08	1.95E+08	2.63E+08	3.38E+08	2.64E+08
tr|Q9RSD0	tr|Q9RSD0	692	2.86E+08	3.68E+08	3.48E+08	1.38E+08	2.41E+08	2.02E+08
tr|Q9RTI3	tr|Q9RTI3	879		24532000		14560000	32561000	
tr|Q9RRU9	tr|Q9RRU9	586						
tr|Q9RU28	tr|Q9RU28	981	38275000	97873000	1.3E+08			
tr|Q9RYN5	tr|Q9RYN5	1779	1.32E+08	1.64E+08	1.29E+08	1.59E+08	2.12E+08	1.56E+08
sp|Q9RU8	sp|Q9RU8	216		17227000	18043000			
sp|Q9RXT	sp|Q9RXT	401	1.03E+08	3.12E+08	1.37E+08	2.43E+08	3.64E+08	2.69E+08
sp|Q9RXT	sp|Q9RXT	401	41934000	7.65E+08	8.6E+08			
tr|Q9RZE8	tr|Q9RZE8	1917	98598000	1.36E+08	1.19E+08	1.42E+08	1.46E+08	1.01E+08
tr|Q9RTU7	tr|Q9RTU7	935						
tr|Q9RXZ9	tr|Q9RXZ9	1672	41402000			31924000	26818000	35849000
tr|Q9RRK0	tr|Q9RRK0	538			21648000	86635000	65931000	45690000
tr|Q9RWP	tr|Q9RWP	1445			45617000	28984000	21218000	28155000
tr|Q9RUH	tr|Q9RUH	1057						
tr|Q9RXS7	tr|Q9RXS7	1640	89873000	1.03E+08	96940000	1.46E+08	1.12E+08	1.14E+08
tr|Q9RVL0	tr|Q9RVL0	1250	19840000	17805000	20300000	61498000	42998000	36457000
tr|Q9RSQ4	tr|Q9RSQ4	748	1.03E+08	1.17E+08	1.32E+08	4.2E+08	3.78E+08	3.74E+08
tr|Q9RUR4	tr|Q9RUR4	1107	3.8E+08	3.57E+08	3.57E+08	2.35E+08	2.47E+08	2.69E+08
tr|Q9RW8	tr|Q9RW8	1371					5430000	5828700
tr|Q9RTT2	tr|Q9RTT2	929	38856000	26108000	26019000	72966000	56244000	85965000
sp|Q9RW0	sp|Q9RW0	327	1.37E+09	1.39E+09	1.16E+09	1.7E+09	1.58E+09	1.47E+09
tr|Q9RVX7	tr|Q9RVX7	1318	30067000	43404000	32083000		34111000	78489000
tr|Q9RVX7	tr|Q9RVX7	1318	35953000	31045000	44161000			
sp|Q9RVC	sp|Q9RVC	277						
tr|Q9RSH1	tr|Q9RSH1	717	16010000	21245000			22836000	
tr|Q9RXG9	tr|Q9RXG9	1592		53162000				
tr|Q9RXR8	tr|Q9RXR8	1635		19008000				
tr|Q9RWA	tr|Q9RWA	1379	1.38E+08	1.57E+08	1.46E+08	1.68E+08	84791000	1.94E+08
tr|Q9RTA8	tr|Q9RTA8	846				26724000	21367000	23610000
tr|Q9RTU7	tr|Q9RTU7	935						10235000
tr|Q9RYT5	tr|Q9RYT5	1814	23927000	27693000	23348000	89345000	88433000	76602000
tr|Q9RYT5	tr|Q9RYT5	1814		62951000		31824000	40052000	60848000

tr|Q9RVG	tr|Q9RVG	1228	2.06E+08	1.63E+08	1.75E+08	1.67E+08	1.51E+08	1.64E+08
tr|Q9RVX7	tr|Q9RVX7	1318			32906000			
sp|Q9RW	sp|Q9RW	335	2089300		1576800	7594500	4825000	
tr|Q9RV32	tr|Q9RV32	1168						1553100
tr|Q9RYM	tr|Q9RYM	1772	21662000		30802000	44425000	43012000	30722000
sp|P52027	sp|P52027	51						
tr|Q9RSU	tr|Q9RSU	763				22162000	17560000	18927000
sp|Q9RTA	sp|Q9RTA	175						
tr|Q9RXB	tr|Q9RXB	1561			25703000			
sp|Q9RW	sp|Q9RW	311	24325000	27025000	28994000	29180000	36249000	35919000
sp|Q9RW	sp|Q9RW	314	2.52E+08	2.82E+08	2.66E+08	1.94E+08	1.69E+08	2.39E+08
tr|Q9RTG	tr|Q9RTG	873					4669500	5096700
tr|Q9RRI4	tr|Q9RRI4	532	89491000	75473000	67691000	1.28E+08	91151000	99270000
sp|Q9RYB	sp|Q9RYB	427	20822000	30120000	14995000	22003000	23668000	16725000
tr|Q9RXQ	tr|Q9RXQ	1627	6.25E+08	6.92E+08	4.88E+08	6.62E+08	5.28E+08	5.52E+08
sp|Q9RXJ	sp|Q9RXJ	375				10188000		
sp|Q9RXJ	sp|Q9RXJ	375						
sp|Q9RW	sp|Q9RW	336	87221000	1.14E+08	1.27E+08	4.3E+08	3.46E+08	3.02E+08
sp|Q9RW	sp|Q9RW	341	85155000	84166000	76896000	72895000	73682000	89810000
tr|Q9RRP	tr|Q9RRP	561	1.47E+08	1.93E+08	1.36E+08	56079000	43688000	69742000
tr|Q9RWP	tr|Q9RWP	1446	29347000	17714000	19071000	20272000	16844000	18535000
tr|Q9RZE	tr|Q9RZE	1913	1.84E+08	2.94E+08	2.55E+08	2.54E+08	2.76E+08	2.38E+08
tr|Q9RU5	tr|Q9RU5	994		46852000		74498000	71089000	1.01E+08
tr|Q9RYX	tr|Q9RYX	1835				56171000	58888000	46649000
sp|Q9RTH	sp|Q9RTH	186	83661000	99434000	1.44E+08	84118000	1.08E+08	1.15E+08
tr|Q9RZA1	tr|Q9RZA1	1888						
sp|Q9RSK	sp|Q9RSK	130	3.3E+08	3.48E+08	3.36E+08	2.48E+08	2.21E+08	2.31E+08
tr|Q9RWF	tr|Q9RWF	1400	77766000	50781000	56010000	86687000	58655000	71255000
tr|Q9RUA	tr|Q9RUA	1026	1.54E+09	1.87E+09	1.34E+09	6.66E+08	5.56E+08	8.31E+08
tr|Q9RUA	tr|Q9RUA	1026		25652000	70352000	1.19E+08	1.12E+08	1.43E+08
sp|Q9RUP	sp|Q9RUP	244				30572000	33964000	27766000
tr|Q9RS98	tr|Q9RS98	672		59700000	56208000	48152000	29556000	59930000
tr|Q9RU0	tr|Q9RU0	969						
tr|Q9RZA	tr|Q9RZA	1892	1.44E+08	1.7E+08	1.28E+08	1.42E+08	1.23E+08	1.07E+08
tr|Q9RZQ	tr|Q9RZQ	1957				23249000		
tr|Q9RSR	tr|Q9RSR	754		25833000	23798000			
sp|Q9RXJ	sp|Q9RXJ	384	1.04E+08	62165000	94268000	1.25E+08	1.29E+08	1.62E+08
sp|Q9RXJ	sp|Q9RXJ	384				39707000	19962000	25606000
tr|Q9RVY	tr|Q9RVY	1319	1.62E+08	1.3E+08	1.11E+08	1.09E+08	1.09E+08	1.22E+08
tr|Q9RRK	tr|Q9RRK	542		7435600	14760000			
tr|Q9RRY	tr|Q9RRY	609			1.99E+09			4.86E+09
tr|Q9RUF	tr|Q9RUF	1047	2.43E+08	2.37E+08	1.89E+08	5.67E+08	3.68E+08	4.08E+08
tr|Q9RUF	tr|Q9RUF	1047		4355100	7079800	8335500	24730000	8947700
sp|Q9RTA	sp|Q9RTA	175				20139000	27260000	26978000
tr|Q9RYQ	tr|Q9RYQ	1792						
sp|Q9RX2	sp|Q9RX2	353	1.11E+08	68150000	85634000	85499000	65934000	73570000
tr|Q9RVJ4	tr|Q9RVJ4	1241		59170000	68657000	23564000	38010000	44934000
tr|Q9RRV	tr|Q9RRV	591						
sp|P5215	sp|P5215	52			52267000		26258000	
sp|Q9RW	sp|Q9RW	322	8161200		5049600	3875500		5414000
tr|Q9RXG	tr|Q9RXG	1587						

sp|Q9RYD	sp|Q9RYD	430						
tr|Q9RXD7	tr|Q9RXD7	1574	1.35E+08	1.31E+08	99886000	2.14E+08	1.72E+08	1.78E+08
sp|Q9RYU	sp|Q9RYU	443	91010000	88268000	94866000	1.38E+08	94178000	95135000
sp|Q9RR9	sp|Q9RR9	73	26676000	75310000	42955000			
tr|Q9RXR1	tr|Q9RXR1	1632	2.66E+08	2.99E+08	2.35E+08	2.27E+08	2.29E+08	3.11E+08
tr|Q9RW7	tr|Q9RW7	1364			26379000			
sp|Q9RTF5	sp|Q9RTF5	182	16598000	7662800	6756000	6640000	7596500	7409500
sp|Q9RW9	sp|Q9RW9	345	2.12E+08	2.2E+08	1.66E+08	1.63E+08	1.75E+08	1.71E+08
tr|Q9RVU2	tr|Q9RVU2	1300					23048000	33770000
sp|Q9RYM	sp|Q9RYM	438	37777000	41708000	49167000	1.5E+08	1.29E+08	1.18E+08
tr|Q9RRZ0	tr|Q9RRZ0	611	2.17E+08	2.29E+08	1.85E+08	4.53E+08	4.43E+08	9922400
tr|Q9RYP4	tr|Q9RYP4	1783	3.68E+08	5.25E+08				
tr|Q9RW2	tr|Q9RW2	1345		6.15E+08		5.58E+08	3.73E+08	4.22E+08
tr|Q9RX16	tr|Q9RX16	1511			10275000	51718000	39680000	35016000
tr|Q9RV82	tr|Q9RV82	1191						
tr|Q9RXX2	tr|Q9RXX2	1660				10428000	10971000	
tr|Q9RS17	tr|Q9RS17	630	6.45E+08	6.2E+08	6E+08	1.51E+09	1.09E+09	1.17E+09
sp|Q9RTK	sp|Q9RTK	192						
tr|Q9RUK5	tr|Q9RUK5	1078					2351600	
sp|Q9RSS	sp|Q9RSS	147	2.1E+08	2.38E+08	1.78E+08	3.64E+08	3.41E+08	3.18E+08
sp|Q9RVB	sp|Q9RVB	275						
tr|Q9RZV2	tr|Q9RZV2	1982	47489000	74793000	95237000	42466000	42948000	47400000
sp|Q9RWH	sp|Q9RWH	328	1.73E+08	1.72E+08	1.45E+08	3.97E+08	3.28E+08	3.15E+08
sp|Q9RWH	sp|Q9RWH	328	40092000		95746000	50559000	67179000	91209000
sp|Q9RT3	sp|Q9RT3	166	1.48E+08	1.49E+08	1.37E+08	65238000	74626000	91311000
sp|Q9RSL4	sp|Q9RSL4	136	11848000	6279400	4465300	8469600	8448800	8600000
sp|Q9RUI	sp|Q9RUI	237	34623000			64851000	61702000	55796000
tr|Q9RTZ3	tr|Q9RTZ3	963	2.11E+08	92563000	1.54E+08	2.29E+08	2.43E+08	1.45E+08
tr|Q9RU01	tr|Q9RU01	969						
tr|Q9RUQ	tr|Q9RUQ	1101						
tr|Q9RUD	tr|Q9RUD	1040		9608300				
sp|Q9RVD	sp|Q9RVD	281						
tr|Q9RVQ	tr|Q9RVQ	1278	27948000	23321000		26562000		30311000
sp|Q9RV5	sp|Q9RV5	265	8497800	5101800	6245000	26920000	18118000	19012000
sp|Q9RV5	sp|Q9RV5	265		7475800	9874700	31737000	44711000	65421000
sp|Q9RRQ	sp|Q9RRQ	95						
tr|Q9RV78	tr|Q9RV78	1190	11795000	5795500	5009600	3840900	5245300	4684400
tr|Q9RYD0	tr|Q9RYD0	1730		32808000	26981000	44009000		40604000
tr|Q9RZA7	tr|Q9RZA7	1892	16399000	11994000	7132900	13527000	13915000	14288000
sp|Q9RTH	sp|Q9RTH	186				28327000		
sp|Q9RW0	sp|Q9RW0	342	40433000		89225000	1.18E+08	99524000	1.12E+08
tr|Q9RWX	tr|Q9RWX	1497	34545000	23982000	30451000			
tr|Q9RUK0	tr|Q9RUK0	1075						
tr|Q9RXA6	tr|Q9RXA6	1559						
sp|Q9RWD	sp|Q9RWD	324	37714000	25717000	29125000	33931000	31553000	26954000
tr|Q9RX59	tr|Q9RX59	1534	1.52E+08	1.45E+08	1.35E+08	2.7E+08	3.48E+08	2.21E+08
tr|Q9RWH	tr|Q9RWH	1409	261600					
tr|Q9RTW	tr|Q9RTW	944						
tr|Q9RV38	tr|Q9RV38	1170	50257000	46015000	43786000	27469000	24871000	19218000
sp|Q9RV2	sp|Q9RV2	262	67665000	56886000	58759000	1.26E+08	1.13E+08	1.17E+08
sp|Q9RVV	sp|Q9RVV	300		4449500	11290000	13533000		14696000

sp|Q9RY4	sp|Q9RY4	416	1.12E+09	1.92E+09	1.72E+09	2.08E+09	1.89E+09	1.98E+09
sp|Q9RW2	sp|Q9RW2	307	72531000	57163000	1.97E+08	2.04E+08	1.49E+08	1.41E+08
tr|Q9RYW	tr|Q9RYW	1830		14724000	20373000	32048000	24183000	25398000
tr|Q9RTW	tr|Q9RTW	946			3187300	2980200	15272000	4557700
tr|Q9RWK	tr|Q9RWK	1424	1.1E+08		88107000	1.46E+08	1.38E+08	1.14E+08
tr|Q9RYS3	tr|Q9RYS3	1803	4.27E+08	3.42E+08	4.58E+08	1.84E+08	2.22E+08	2.34E+08
tr|Q9RT06	tr|Q9RT06	795	1.1E+08					
tr|Q9RW4	tr|Q9RW4	1351			26677000			
sp|Q9RW	sp|Q9RW	335						
tr|Q9RX52	tr|Q9RX52	1528				75491000	46134000	52253000
tr|Q9RSA6	tr|Q9RSA6	677	1.3E+08	2.19E+08	1.53E+08	86276000	1.01E+08	89651000
tr|Q9RZG	tr|Q9RZG	1923						
tr|Q9RSC3	tr|Q9RSC3	688	5.04E+08	1.05E+09	4.4E+08	5.5E+08	5.16E+08	6.79E+08
sp|Q9RUR	sp|Q9RUR	249	3.11E+09	2.95E+09	2.59E+09	5.51E+09	4.52E+09	4.74E+09
sp|Q9RUR	sp|Q9RUR	249			1.98E+08	2.99E+08	3.47E+08	2.86E+08
sp|Q9RUV	sp|Q9RUV	254	3494600		1381400			
tr|Q9RTP6	tr|Q9RTP6	910			8935800			
sp|Q9RX3	sp|Q9RX3	356	99217000	54295000	42975000	1.76E+08	1.52E+08	1.59E+08
tr|Q9RTJ1	tr|Q9RTJ1	883	2.12E+08	2.02E+08	1.75E+08	2.39E+08	2.98E+08	2.98E+08
tr|Q9RTD1	tr|Q9RTD1	856	69886000	25605000	37562000	2.37E+08	74761000	2.19E+08
sp|Q9RUR	sp|Q9RUR	249	9.87E+08	8.55E+08	8.28E+08	2.62E+09	2.26E+09	2.35E+09
tr|Q9RV67	tr|Q9RV67	1184						
tr|Q9RUP9	tr|Q9RUP9	1100	3.01E+08	3.37E+08	2.41E+08	4.07E+08	3.56E+08	4.2E+08
sp|Q9RSX	sp|Q9RSX	155		40428000	32472000	18916000	23260000	16172000
tr|Q9RRE7	tr|Q9RRE7	513	71095000		1.33E+08	68314000	72249000	88375000
tr|Q9RZ81	tr|Q9RZ81	1875						
tr|Q9RSN5	tr|Q9RSN5	742	31068000	42894000	40368000	41044000	53947000	39184000
sp|Q9RXR	sp|Q9RXR	396	1.04E+09	9.58E+08	8.79E+08	9.39E+08	8.28E+08	9.11E+08
tr|Q9RY21	tr|Q9RY21	1682	4.86E+08	3.8E+08	3.18E+08	6.22E+08	7.47E+08	6.25E+08
tr|Q9RW1	tr|Q9RW1	1337	59635000	57608000	63353000	1.51E+08	1.29E+08	1.19E+08
sp|Q9RSL1	sp|Q9RSL1	133	1.33E+08	71745000	64330000	71762000	89877000	80363000
tr|Q9RU91	tr|Q9RU91	1015	22069000	16495000	11750000	1.32E+08	89921000	19087000
tr|Q9RUY5	tr|Q9RUY5	1141	1.66E+08	1.33E+08	1.53E+08	85811000	97995000	1.19E+08
tr|Q9RSA7	tr|Q9RSA7	678		31649000				
tr|Q9RUC	tr|Q9RUC	1033			38237000	31823000	30727000	
tr|Q9RT35	tr|Q9RT35	811			82632000			
tr|Q9RUA	tr|Q9RUA	1026	11247000	8964100	7823300	12685000	16884000	14169000
tr|Q9RUA	tr|Q9RUA	1026	1.23E+09	1.07E+08	2.25E+08	5.27E+08	5.79E+08	1.53E+09
tr|Q9RST9	tr|Q9RST9	761	83222000			55776000		
tr|Q9RRC2	tr|Q9RRC2	498	9438400	7466000		4643600	6195400	5393700
sp|Q9RSL	sp|Q9RSL	132	3496600	1182500	1078800	790610	1349300	788320
sp|Q9RW	sp|Q9RW	326	2.55E+09	2.89E+09	2.19E+09	1.72E+09	1.52E+09	1.73E+09
sp|Q9RW	sp|Q9RW	326			22545000	11690000	15844000	
tr|Q9RWP	tr|Q9RWP	1449			43650000			
sp|Q9RV7	sp|Q9RV7	269						
tr|Q9RYI6	tr|Q9RYI6	1762				37648000	9121500	
sp|Q9RWI	sp|Q9RWI	332	1.28E+08	94315000	81412000	78043000	86995000	
sp|Q9RVP	sp|Q9RVP	293	27568000	26975000	20752000	28491000	28893000	37133000
tr|Q9RSL8	tr|Q9RSL8	734	56866000	51082000	62833000			4653500
tr|Q9RRY6	tr|Q9RRY6	609						
tr|Q9RYI1	tr|Q9RYI1	1760	507520	647040		356980		

tr|Q9RT92	tr|Q9RT92	838	13348000	9236000	6225500			
tr|Q9RW8	tr|Q9RW8	1368	39351000	31888000	39234000		33324000	
sp|Q9RYP	sp|Q9RYP	440			17542000			
tr|Q9RVA2	tr|Q9RVA2	1199				44869000		45001000
sp|Q9RVQ	sp|Q9RVQ	295						
tr|Q9RTJ1	tr|Q9RTJ1	883	67414000		47387000	1.05E+08	84381000	98776000
sp|Q9RXF	sp|Q9RXF	369	37114000	17708000	15299000			
tr|Q9RVS4	tr|Q9RVS4	1289			47421000			
tr|Q9RVP1	tr|Q9RVP1	1267	44829000	41725000	46224000	39187000	22464000	48480000
tr|Q9RUK0	tr|Q9RUK0	1075	68258000	36551000	42233000		73912000	
tr|Q9RTM	tr|Q9RTM	898	93819000	82586000	76564000	75505000	81675000	56727000
sp|Q9RWE	sp|Q9RWE	320	5.96E+08	7.07E+08	7.31E+08	3.08E+08	2.31E+08	1.87E+08
sp|Q9RTF	sp|Q9RTF	183	24459000	30321000	27100000		15543000	36594000
tr|Q9RRV2	tr|Q9RRV2	589	1.12E+08	58703000	91773000	2.64E+08	2.79E+08	2.66E+08
sp|Q9RT9	sp|Q9RT9	172					4198800	
tr|Q9RZD8	tr|Q9RZD8	1912						
sp|Q9RUY	sp|Q9RUY	259	12122000	6640200			6677700	6485100
tr|Q9RSE0	tr|Q9RSE0	699	16391000	29528000	24346000	23112000		
sp|Q9RXK	sp|Q9RXK	389	797820			661230	995940	770660
sp|Q9RXK	sp|Q9RXK	389						
sp|Q9RXK	sp|Q9RXK	389					22042000	
sp|Q9RXK	sp|Q9RXK	389					35858000	35135000
tr|Q9RT79	tr|Q9RT79	833			26680000	33381000	18905000	24744000
tr|Q9RXI3	tr|Q9RXI3	1602	2.89E+08	1.9E+08	2.5E+08	3.86E+08	4.79E+08	2.9E+08
tr|Q9RX65	tr|Q9RX65	1538						
tr|Q9RZL7	tr|Q9RZL7	1945	3.05E+08	2.42E+08	2.46E+08	7.36E+08	5.79E+08	6.72E+08
tr|Q9RUR	tr|Q9RUR	1105	15559000	10012000	11991000	2776600	3777300	3667000
sp|Q9RWI	sp|Q9RWI	331	1.31E+08			1.69E+08	1.56E+08	1.71E+08
tr|Q9RRQ	tr|Q9RRQ	566	4429500		4651700			
tr|Q9RSD0	tr|Q9RSD0	692				64961000	46879000	
sp|Q9RRQ	sp|Q9RRQ	95	56575000			76305000	98707000	1.02E+08
tr|Q9RRB4	tr|Q9RRB4	495	15904000	26994000	23148000	48958000	29193000	32717000
tr|Q9RVG7	tr|Q9RVG7	1229						
tr|Q9RTP0	tr|Q9RTP0	906	847570				586780	
tr|Q9RR71	tr|Q9RR71	470	1.3E+09	1.27E+09	1.02E+09	1.25E+09	1.16E+09	1.15E+09
tr|Q9RTT2	tr|Q9RTT2	929						
tr|Q9RRI7	tr|Q9RRI7	534	4335300		1350200			
tr|Q9RSL8	tr|Q9RSL8	734		10484000		31569000	50247000	38029000
sp|O3250	sp|O3250	45	3.24E+08	4.22E+08	5.41E+08	41157000	33116000	29096000
tr|Q9RTY0	tr|Q9RTY0	955	83708000	55598000	47804000	66165000		59098000
tr|Q9RS56	tr|Q9RS56	648	1.43E+08	1.45E+08				
sp|Q9RWS	sp|Q9RWS	345	4.31E+08	4.28E+08	3.33E+08	3.62E+08	3.66E+08	3.9E+08
sp|Q9RYC	sp|Q9RYC	428	29598000					
tr|Q9RSB8	tr|Q9RSB8	685	78688000	67614000	69498000	1.11E+08	1.48E+08	96065000
tr|Q9RSB8	tr|Q9RSB8	685	1.69E+09	1.79E+09	1.38E+09	1.46E+09	1.57E+09	1.62E+09
tr|Q9RYZ9	tr|Q9RYZ9	1842	63695000	59374000	52131000	60626000	35766000	63029000
tr|Q9RWI0	tr|Q9RWI0	1413				94399000	48112000	
tr|Q9RR62	tr|Q9RR62	467	2.49E+08	1.09E+08	1.39E+08			
sp|Q9RY6	sp|Q9RY6	421	38210000	32551000	36913000		39234000	
tr|Q9RVX7	tr|Q9RVX7	1318	1.23E+09	1.19E+09	1E+09	1.13E+09	9.35E+08	1.04E+09
sp|Q9RU7	sp|Q9RU7	214		18629000	16810000		15334000	8444000

tr|Q9RZD7	tr|Q9RZD7	1911						
tr|Q9RVQ	tr|Q9RVQ	1271	3.61E+09	3.12E+09	2.52E+09	1.13E+09	1.1E+09	1.28E+09
sp|Q9RY0	sp|Q9RY0	410						
tr|Q9RSB1	tr|Q9RSB1	681		18694000	29829000			15361000
tr|Q9RWH	tr|Q9RWH	1412	25313000		14131000	15755000	18247000	
tr|Q9RVB4	tr|Q9RVB4	1208	76014000	60575000	49890000	1.15E+08	71757000	95601000
tr|Q9RZP7	tr|Q9RZP7	1955				14937000		9596300
tr|Q9RW3	tr|Q9RW3	1349		7053900	8549300			
sp|Q9RY6	sp|Q9RY6	420			7042100			
sp|Q9RW7	sp|Q9RW7	314	36989000	19473000	15548000	4819800	6410000	5052400
sp|Q9RR9	sp|Q9RR9	73	1.63E+08					
tr|Q9RUY1	tr|Q9RUY1	1138					25532000	40828000
tr|Q9RS59	tr|Q9RS59	650	2.31E+08	2.28E+08	1.69E+08	2.12E+08	1.86E+08	2.14E+08
tr|Q9RVH	tr|Q9RVH	1234						
tr|Q9RS52	tr|Q9RS52	646						
sp|Q9RZA	sp|Q9RZA	454						
tr|Q9RSU7	tr|Q9RSU7	767	3.16E+09	2.94E+09	2.69E+09	3.25E+09	3.12E+09	3.07E+09
tr|Q9RU92	tr|Q9RU92	1016	2.79E+08	2.3E+08	1.74E+08	2.52E+08	1.82E+08	
tr|Q9RUV4	tr|Q9RUV4	1125		12580000	12527000			
tr|Q9RT87	tr|Q9RT87	837	1.58E+08	1.43E+08	1.56E+08	1.52E+08	1.26E+08	1.25E+08
tr|Q9RVW	tr|Q9RVW	1311	40543000		44286000	71770000	60282000	62821000
tr|Q9RVW	tr|Q9RVW	1311				17556000	26413000	17135000
tr|Q9RUH	tr|Q9RUH	1056	14623000	6117700	5391300	4521400		4436100
tr|Q9RV14	tr|Q9RV14	1158						
tr|Q9RRP9	tr|Q9RRP9	561	28775000	3.28E+08	2.29E+08		2.8E+08	1.17E+08
tr|Q9RRM	tr|Q9RRM	550	26220000	22411000	32058000			
tr|Q9RT97	tr|Q9RT97	843					61732000	34952000
tr|Q9RWB	tr|Q9RWB	1385	1.42E+08	1.01E+08		1.32E+08	81001000	1.34E+08
sp|Q9RU3	sp|Q9RU3	210				61287000	80951000	53737000
sp|Q9RVW	sp|Q9RVW	301	2.27E+09	2.04E+09	1.92E+09	7.89E+08	1.04E+09	5.78E+08
sp|Q9RVW	sp|Q9RVW	301					25234000	20224000
sp|Q9RU8	sp|Q9RU8	216	3650400	1448900	1437400		780920	
tr|Q9RUI9	tr|Q9RUI9	1066	1.16E+08	76519000	74272000	90521000	61998000	82062000
sp|Q9RTA	sp|Q9RTA	175		3.98E+08	3.35E+08			4.39E+08
sp|Q9RXL	sp|Q9RXL	392	1.66E+08	1.81E+08	1.58E+08	2.44E+08	1.9E+08	1.57E+08
tr|Q9RU27	tr|Q9RU27	980	1.43E+08	1.59E+08	2.09E+08	40359000	82268000	66854000
tr|Q9RWW	tr|Q9RWW	1493	1.78E+09	1.62E+09	1.52E+09	3.04E+08	2.95E+08	5.21E+08
tr|Q9RWW	tr|Q9RWW	1493			1.52E+08	34498000		78033000
tr|Q9RYG5	tr|Q9RYG5	1750				34180000		17732000
tr|Q9RT01	tr|Q9RT01	792						
tr|Q9RT01	tr|Q9RT01	792						
sp|Q9RW0	sp|Q9RW0	304	1.38E+08	1.62E+08	1.36E+08	3511400		77058000
tr|Q9RXI3	tr|Q9RXI3	1602	62210000	69395000	29465000	93557000	92290000	88894000
sp|Q9RW	sp|Q9RW	319	30167000	46065000	43109000			
tr|Q9RYF8	tr|Q9RYF8	1745	1.16E+08		93992000	1.25E+08	1.55E+08	1.42E+08
tr|Q9LBE3	tr|Q9LBE3	466		10657000	6682400	45876000		15990000
tr|Q9RYF4	tr|Q9RYF4	1743					32056000	32882000
tr|Q9RXM	tr|Q9RXM	1616		37253000	33595000	41389000		
tr|Q9RXM	tr|Q9RXM	1616	24527000	22809000	41077000			26618000
tr|Q9RY21	tr|Q9RY21	1682						
tr|Q9RYH9	tr|Q9RYH9	1758	71579000	81504000	1.19E+08	79296000	1.2E+08	1.14E+08

sp|P56867	sp|P56867	56	75573000	1.36E+08	84996000		31084000	
tr|Q9RWU	tr|Q9RWU	1474	30910000		27989000			
sp|P52153	sp|P52153	52	70764000	53740000	74493000	3.25E+08	2.56E+08	2.22E+08
tr|Q9RVN	tr|Q9RVN	1260		61515000	58683000	1.16E+08	81961000	75152000
sp|P56867	sp|P56867	56			19875000			
sp|Q9RW(	sp|Q9RW(	326	42665000	76100000	1.23E+08	74152000	80458000	74216000
tr|Q9RXP5	tr|Q9RXP5	1625				26247000	4883700	14569000
tr|Q9RVP1	tr|Q9RVP1	1267	18992000	12796000	10603000	18011000	17102000	13163000
tr|Q9RVP1	tr|Q9RVP1	1267				29713000		
tr|Q9RR67	tr|Q9RR67	469	16852000	18695000	17390000	27117000	42552000	34597000
sp|Q9RRQ	sp|Q9RRQ	94	7683600	13177000		14116000	13896000	12020000
sp|Q9RX3	sp|Q9RX3	356	80396000	69589000	64670000	83774000	77546000	74495000
sp|P56867	sp|P56867	56			67504000	3.18E+08	2.74E+08	2.63E+08
sp|P56867	sp|P56867	56	2.76E+08	74021000	4.2E+08	3.02E+08	3.14E+08	5.46E+08
tr|Q9RZ04	tr|Q9RZ04	1844			6278600			
tr|Q9RVB5	tr|Q9RVB5	1209				8355800		
sp|Q9RUN	sp|Q9RUN	242	75044000	1.02E+08	82404000	3.65E+08	2.69E+08	2.43E+08
tr|Q9RZ08	tr|Q9RZ08	1846		15750000	20223000	32619000	35343000	42998000
tr|Q9RYF0	tr|Q9RYF0	1742	24230000	43657000	22962000	58047000	38515000	49092000
tr|Q9RW0	tr|Q9RW0	1336						
tr|Q9RSU7	tr|Q9RSU7	767	29622000					
sp|Q9RWH	sp|Q9RWH	330		52222000				
tr|Q9RRQ	tr|Q9RRQ	563				26325000	22216000	17025000
sp|Q9RWU	sp|Q9RWU	347	1.3E+08	22455000	85508000		99655000	1.11E+08
tr|Q9RT53	tr|Q9RT53	821		25973000	24051000	89893000	84720000	78458000
tr|Q9RT53	tr|Q9RT53	821				4236500		4167700
tr|Q9RXQ	tr|Q9RXQ	1627	83056000	75076000	54761000	1.51E+08	1.15E+08	1.23E+08
tr|Q9RY02	tr|Q9RY02	1675	3.93E+08	3.74E+08	3.32E+08	3.5E+08	2.53E+08	2.83E+08
sp|Q9RY2	sp|Q9RY2	411			22364000	24787000		19271000
tr|Q9RY86	tr|Q9RY86	1710	85999000	1.06E+08	90585000	1.61E+08	1.64E+08	1.82E+08
tr|Q9RSD6	tr|Q9RSD6	697	27818000	51712000	38005000	1.41E+08	1.14E+08	22347000
sp|Q9RU1	sp|Q9RU1	205		12460000	17530000			8916200
tr|Q9RXM	tr|Q9RXM	1614						
sp|Q9RZ0	sp|Q9RZ0	452						
sp|Q9RRB	sp|Q9RRB	77	1.18E+09	1.11E+09	1.16E+09	1.25E+09	1.14E+09	1.43E+09
tr|Q9RUB	tr|Q9RUB	1031			21741000			
sp|Q9RV9	sp|Q9RV9	271		37734000	29377000	26088000	27735000	21981000
tr|Q9RRH	tr|Q9RRH	527		1.37E+08	1.09E+08		1.39E+08	
tr|Q9RWA	tr|Q9RWA	1382						
tr|Q9RSE2	tr|Q9RSE2	701	33196000	30109000	20609000	26970000	44211000	28039000
tr|Q9RRF2	tr|Q9RRF2	515	61916000	48645000	59734000			
tr|Q9RST7	tr|Q9RST7	760	1.38E+08	1.45E+08	1.13E+08	2.07E+08	1.6E+08	1.75E+08
tr|Q9RST7	tr|Q9RST7	760			25049000		40276000	28698000
tr|Q9RUB	tr|Q9RUB	1031				26668000	23251000	29857000
tr|Q9RX52	tr|Q9RX52	1528						
tr|Q9RUG	tr|Q9RUG	1055			19214000	4431200		
tr|Q9RUZ9	tr|Q9RUZ9	1150	30040000	42278000				
tr|Q9RYX1	tr|Q9RYX1	1832			12513000			27523000
tr|Q9RW8	tr|Q9RW8	1369	38920000	55023000	38934000	57223000	51690000	54548000
tr|Q9RUP1	tr|Q9RUP1	1099	7.85E+08	1.61E+09	1.29E+09	3.09E+08	1.69E+08	3.76E+08
tr|Q9RYG1	tr|Q9RYG1	1747			1.99E+08			20972000

tr|Q9RTN	tr|Q9RTN3	904	10336000	13141000	14186000	55727000	40114000	44991000
tr|Q9RYW	tr|Q9RYW	1826		24615000	10773000			
tr|Q9RS7	tr|Q9RS76	657	1.8E+08	3.51E+08	3.56E+08	20073000	88519000	
tr|Q9RR8	tr|Q9RR86	477	27160000	45464000	43578000	1.5E+08	63887000	
tr|Q9RYG	tr|Q9RYG3	1748						15340000
sp|Q9RW	sp|Q9RWH	330						
sp|Q9RV1	sp|Q9RV1	261	2.24E+08	1.5E+08	1.52E+08			
tr|Q9RSB1	tr|Q9RSB1	681	15500000	34459000		15591000	18751000	16743000
sp|Q9RVV	sp|Q9RVV	300	2.46E+08	1.68E+08	2.09E+08	1.71E+08		92659000
sp|Q9RR7	sp|Q9RR7	67	5296400	1749200	1978000	1597700	2405300	1807900
sp|Q9RYB	sp|Q9RYB	427	1.7E+08	1.6E+08	1.17E+08	75543000	1.07E+08	76061000
sp|Q9RYB	sp|Q9RYB	427						
sp|Q9RUF	sp|Q9RUF	232	63573000	1.13E+08	2.26E+08	2.99E+08	1.65E+08	1.44E+08
sp|Q9RTY	sp|Q9RTY	203	71214000	72391000	64033000	95321000	87604000	77226000
tr|Q9RUG	tr|Q9RUG	1055	46388000	42212000	53216000	72466000	49517000	57039000
tr|Q9RTQ	tr|Q9RTQ6	916			24675000			
tr|Q9RRA	tr|Q9RRA7	490	54316000	53472000	52190000	75705000	87891000	80615000
tr|Q9RVK1	tr|Q9RVK1	1244			3299500			
sp|Q9RVT	sp|Q9RVT	296	3.13E+08	3.64E+08	3.34E+08	1.51E+08	1.28E+08	1.36E+08
tr|Q9RWI	tr|Q9RWI7	1418	53087000	1.09E+08	90060000		80374000	49888000
tr|Q9RS71	tr|Q9RS71	655	1.32E+08	94918000	1.69E+08	17675000	15587000	14655000
tr|Q9RYI7	tr|Q9RYI7	1763	51730000	64074000	48758000	91712000	68693000	81461000
sp|Q9RW	sp|Q9RW9	317	1.45E+08	1.42E+08	1.26E+08	1.97E+08	1.51E+08	
tr|Q9RRI6	tr|Q9RRI6	533	28647000	65866000	70431000	94954000	94341000	92634000
tr|Q9RVN	tr|Q9RVN	1263						
tr|Q9RWP	tr|Q9RWP	1449	3.56E+08	4.37E+08	2.94E+08	3.58E+08	3.46E+08	3.51E+08
sp|Q9RTS	sp|Q9RTS	199	99052000	81759000	76058000		27248000	26505000
tr|Q9RZD	tr|Q9RZD6	1910				33738000		
tr|Q9RRG	tr|Q9RRG4	520	8.73E+08	9.43E+08	8.93E+08		3E+08	
sp|Q9RRC	sp|Q9RRC	79	22589000		17514000	14871000	24483000	13394000
tr|Q9RV3	tr|Q9RV38	1170	18407000		21947000			
sp|Q9RT2	sp|Q9RT2	164	55259000	81803000	76954000	99103000	89543000	79083000
tr|Q9RYG	tr|Q9RYG4	1749						
tr|Q9RS6	tr|Q9RS66	653						
tr|Q9RRE1	tr|Q9RRE1	509	1.59E+08	1.35E+08	1.44E+08		2.29E+08	2.86E+08
tr|Q9RTQ	tr|Q9RTQ1	913			1.43E+08	2.76E+08	2.13E+08	2.29E+08
tr|Q9RXM	tr|Q9RXM	1615	2.18E+08	2.27E+08	2.87E+08			
tr|Q9RXM	tr|Q9RXM	1615		9451700	8929600			
tr|Q9RV7	tr|Q9RV70	1186	14296000		41343000			
sp|Q9RRC	sp|Q9RRC	79	1.12E+08	1.69E+08	1.79E+08	1.07E+08	1.1E+08	82200000
tr|Q9RZF3	tr|Q9RZF3	1921				11547000		
sp|Q9RSX	sp|Q9RSX	155	1.97E+08	2.19E+08	1.59E+08	2.18E+08	1.61E+08	1.91E+08
sp|Q9RSZ	sp|Q9RSZ	159	75167000	68923000	62096000	1.37E+08	1.47E+08	1.17E+08
tr|Q9RSQ	tr|Q9RSQ4	748						
tr|Q9RX17	tr|Q9RX17	1512	53593000	50575000	54647000	77560000	47792000	54506000
tr|Q9RXI1	tr|Q9RXI1	1601	7.65E+08	7.28E+08	6.14E+08	6.97E+08	4.51E+08	4.55E+08
tr|Q9RTD	tr|Q9RTD6	859	34740000	21060000	25029000			
sp|Q9RXR	sp|Q9RXR	396	80266000	1.03E+08	60684000	1.6E+08	1.17E+08	1.15E+08
tr|Q9RRL1	tr|Q9RRL1	545						
sp|Q9RT2	sp|Q9RT2	163	6.28E+08	4.77E+08	4.11E+08	3.99E+08	3.88E+08	3.48E+08
sp|Q9RW	sp|Q9RW9	318	41976000	43276000	35089000		21470000	

tr|Q9RXG	tr|Q9RXG	1587	61403000	79304000	1.47E+08	54989000	45426000	88854000
sp|Q9RTK	sp|Q9RTK	192				33309000		
tr|Q9RU0	tr|Q9RU0	969	5.25E+08		5.32E+08	7.26E+08	6.35E+08	6.03E+08
sp|Q9RRB	sp|Q9RRB	76	6.51E+08	8.83E+08	6.11E+08	3.48E+08	2.22E+08	3.43E+08
tr|Q9RZS7	tr|Q9RZS7	1967	7.48E+08	6.73E+08	6.66E+08	6.66E+08	6.37E+08	6.72E+08
sp|Q9RXX	sp|Q9RXX	405	5453000		1797900			
sp|Q9RXX	sp|Q9RXX	405			32701000			
tr|Q9RVU	tr|Q9RVU	1299	6287200	15710000	19096000			
tr|Q9RTF9	tr|Q9RTF9	869	75689000	68148000	71006000			16648000
tr|Q9RRA	tr|Q9RRA	486	24839000		15953000			15965000
sp|Q9RVW	sp|Q9RVW	301					33807000	
tr|Q9RYH	tr|Q9RYH	1758	1.96E+09	2.14E+09	1.58E+09	1.9E+09	1.84E+09	2.02E+09
sp|Q9RUV	sp|Q9RUV	253		62278000	71853000	76477000	25792000	52024000
sp|Q9RW	sp|Q9RW	342	28931000	10372000	10590000	6085900	11775000	8678200
sp|Q9RW	sp|Q9RW	342	14451000	24242000	38052000	15909000	30717000	18966000
sp|Q9RTF	sp|Q9RTF	183						25913000
tr|Q9RRE7	tr|Q9RRE7	513	2.28E+08	3.29E+08	2.48E+08	2.39E+08	1.75E+08	2.19E+08
sp|Q9RTF	sp|Q9RTF	182	39989000	69884000	1.25E+08		20105000	
tr|Q9RXX2	tr|Q9RXX2	1660	41240000					
sp|Q9RT2	sp|Q9RT2	165	5.71E+08	5.48E+08	4.79E+08	1.2E+09	1.16E+09	1.06E+09
sp|Q9RRB	sp|Q9RRB	75	25874000	48222000	62164000			
tr|Q9RXF0	tr|Q9RXF0	1581				29165000	90732000	68039000
tr|Q9RT87	tr|Q9RT87	837	3.26E+08	4.69E+08	3.45E+08	5.28E+08	6.69E+08	4.42E+08
tr|Q9RT02	tr|Q9RT02	793	22098000		46352000	6594000	52582000	80283000
tr|Q9RU0	tr|Q9RU0	970	24271000	24584000	26281000			12530000
tr|Q9RU5	tr|Q9RU5	1000	37660000	45388000	33908000	1.05E+08	55938000	84467000
tr|Q9RU5	tr|Q9RU5	1000	1.14E+08	64751000	1.23E+08	61233000	60741000	80400000
tr|Q9RVS1	tr|Q9RVS1	1287	79096000	36028000	36189000			
tr|Q9RRK	tr|Q9RRK	543	2.11E+08	6.63E+08	6.18E+08	5.55E+08	6.89E+08	4.62E+08
tr|Q9RYE0	tr|Q9RYE0	1736		12474000		19027000	23264000	20385000
sp|Q9RS6	sp|Q9RS6	112	76736000	1.09E+08	81001000	1.02E+08	2.06E+08	81987000
tr|Q9RYZ6	tr|Q9RYZ6	1840	29045000		38145000	82231000	1.43E+08	92829000
tr|Q9RRX1	tr|Q9RRX1	601	2.6E+09	2.9E+09	2.58E+09	1.88E+09	1.85E+09	1.75E+09
sp|Q9RUP	sp|Q9RUP	243	82836000	65078000	1.11E+08	1.22E+08	46410000	58471000
tr|Q9RTZ8	tr|Q9RTZ8	966			1146500	4483000	6268400	7704900
tr|Q9RW5	tr|Q9RW5	1357	2.1E+08	2.22E+08	1.68E+08	2.75E+08	2.53E+08	2.68E+08
tr|Q9RRP4	tr|Q9RRP4	558	1.26E+08	85042000	1.13E+08			19374000
tr|Q9RSY7	tr|Q9RSY7	785	12983000	69114000	42386000	72574000	60333000	75987000
sp|Q9RUR	sp|Q9RUR	249						
tr|Q9RRV8	tr|Q9RRV8	593			8359600	1.25E+08	1.36E+08	1.3E+08
tr|Q9RSA5	tr|Q9RSA5	676	17539000	37061000	1.39E+08	38741000	60715000	1.21E+08
tr|Q9RVW	tr|Q9RVW	1311						16280000
sp|Q9RY2	sp|Q9RY2	411						
sp|Q9RRB	sp|Q9RRB	76						
sp|Q9RYA	sp|Q9RYA	425						5446100
tr|Q9RU9	tr|Q9RU9	1017	30799000	40848000	36994000	2.23E+08	1.67E+08	2.7E+08
tr|Q9RZI5	tr|Q9RZI5	1936	20189000	19603000	23227000			
tr|Q9RYA5	tr|Q9RYA5	1719	1.79E+08	2.35E+08	1.84E+08	1.84E+08	1.74E+08	2.04E+08
tr|Q9RTZ7	tr|Q9RTZ7	965	4.16E+08	3.35E+08	3.81E+08	3.6E+08	2.86E+08	3.35E+08
tr|Q9RY21	tr|Q9RY21	1682	2.58E+08	97378000	1.78E+08	5.54E+08	5.34E+08	1.23E+08
tr|Q9RS07	tr|Q9RS07	623	2.05E+08	2.19E+08	1.61E+08	1.04E+08	1.19E+08	1.21E+08

tr|Q9RS07	tr|Q9RS07	623	31911000	22854000				
tr|Q9RY57	tr|Q9RY57	1699			17136000	43460000	38185000	24190000
tr|Q9RX10	tr|Q9RX10	1507				27810000		23499000
sp|Q9RU2	sp|Q9RU2	208			23238000			
sp|Q9RU2	sp|Q9RU2	208				33948000	56648000	68691000
tr|Q9RUN	tr|Q9RUN	1096	25760000	24344000	48067000			19855000
sp|Q9RU8	sp|Q9RU8	221		2.51E+08			1.5E+08	
sp|Q9RW4	sp|Q9RW4	308	2327700					
sp|Q9RW4	sp|Q9RW4	308				9202100	13308000	16851000
tr|Q9RZ24	tr|Q9RZ24	1856	1.13E+08	1.46E+08	84375000	98138000	1.14E+08	1.05E+08
sp|Q9RXK	sp|Q9RXK	389	1.1E+09	1.39E+09	1E+09	2.81E+09	2.64E+09	2.41E+09
sp|Q9RXK	sp|Q9RXK	389	29493000	25741000	33164000		18501000	37043000
tr|Q9RVM	tr|Q9RVM	1257	5.08E+08	6.51E+08	5.69E+08	9.34E+08	1.01E+09	8.15E+08
tr|Q9RU1	tr|Q9RU1	976	41576000	87144000	61144000	87394000	1.06E+08	77962000
tr|Q9RV75	tr|Q9RV75	1189	42131000	51435000	17257000	96135000	82381000	86667000
tr|Q9RVF7	tr|Q9RVF7	1224	36326000	24011000	49249000	1.2E+08	31706000	37985000
tr|Q9RXW	tr|Q9RXW	1659	62547000	53950000	22143000	56290000	38292000	58361000
tr|Q9RU26	tr|Q9RU26	979	94646000	77387000	58223000	2.63E+08	1.79E+08	1.81E+08
tr|Q9RSY6	tr|Q9RSY6	784	1.95E+08	2.41E+08	1.81E+08	4.29E+08	2.85E+08	2.99E+08
tr|Q9RSY6	tr|Q9RSY6	784		9459500	13390000	61709000	88944000	77678000
tr|Q9RX19	tr|Q9RX19	1514	1.04E+08	87075000		1.4E+08	1.12E+08	1.12E+08
tr|Q9RX19	tr|Q9RX19	1514	55628000	61326000	1.08E+08	35958000	47192000	60340000
tr|Q9RX95	tr|Q9RX95	1553						
tr|Q9RS63	tr|Q9RS63	652	2.89E+08	2.89E+08	2.67E+08	3.55E+08	3.03E+08	3.25E+08
sp|Q9RX6	sp|Q9RX6	361						
tr|Q9RRZ6	tr|Q9RRZ6	615	12707000			10335000	10602000	
sp|Q9RYH	sp|Q9RYH	435						
tr|Q9RVQ	tr|Q9RVQ	1277	2.86E+08	1.85E+08	1.59E+08	2.27E+08	1.94E+08	2.36E+08
tr|Q9RRV4	tr|Q9RRV4	591					11663000	7684400
tr|Q9RU50	tr|Q9RU50	994			78541000		39113000	12672000
sp|Q9RSZ	sp|Q9RSZ	159	60580000	52649000	51139000	99861000	88524000	92738000
tr|Q9RX53	tr|Q9RX53	1529						
tr|Q9RVK8	tr|Q9RVK8	1248	4.98E+08	7.54E+08	5.5E+08	3.56E+08	5.71E+08	3.39E+08
tr|Q9RTY0	tr|Q9RTY0	955		6.12E+08	4.98E+08	1.17E+09	1.03E+09	1.05E+09
tr|Q9RTY0	tr|Q9RTY0	955	5.48E+08	5.53E+08	4.97E+08	68775000	1.09E+08	1.44E+08
tr|Q9RS53	tr|Q9RS53	647						
tr|Q9RSN4	tr|Q9RSN4	741	57488000	55030000	52716000	1.38E+08	1.22E+08	1.01E+08
tr|Q9RYC2	tr|Q9RYC2	1724	10437000	18238000	15104000	43506000	41262000	36673000
sp|Q9RW7	sp|Q9RW7	314	6.7E+08	6.39E+08	5.37E+08	7E+08	7.35E+08	6.94E+08
sp|Q9RW7	sp|Q9RW7	314	37706000	60778000	2.14E+08	57571000		1.1E+08
tr|Q9RW3	tr|Q9RW3	1347	42120000	36639000	34711000	42990000	47894000	50409000
tr|Q9RUI9	tr|Q9RUI9	1066	3.68E+08	3.51E+08	2.98E+08	2.33E+08	1.98E+08	2.14E+08
tr|Q9RZT3	tr|Q9RZT3	1970						
tr|Q9RWV	tr|Q9RWV	1484	3258400	870360	1102000		2819900	1941500
sp|Q9RRL	sp|Q9RRL	90	33203000		43902000			
tr|Q9RYA5	tr|Q9RYA5	1719				13381000		
sp|Q9RYC	sp|Q9RYC	428	52425000	36112000	35236000	29141000	24012000	23645000
tr|Q9RYL3	tr|Q9RYL3	1770	2.53E+08	1.81E+08	2.75E+08	70247000	82132000	1.1E+08
tr|Q9RSV0	tr|Q9RSV0	769				2.43E+08	2.2E+08	2.44E+08
tr|Q9RRQ	tr|Q9RRQ	563				16308000		
tr|Q9RV88	tr|Q9RV88	1194	5.25E+08	9.34E+08	6.49E+08	5.09E+08	3.36E+08	4.58E+08

tr|Q9RZ94	tr|Q9RZ94	1884	3750900					
tr|Q9RTS5	tr|Q9RTS5	927	41281000	34653000	30951000	59832000	52879000	72421000
tr|Q9RTZ4	tr|Q9RTZ4	964		17536000		58327000	72420000	38709000
tr|Q9RT71	tr|Q9RT71	828	19275000	10980000	15913000	84885000		18523000
tr|Q9RZN4	tr|Q9RZN4	1951				1.3E+08	1.34E+08	1.06E+08
tr|Q9RX19	tr|Q9RX19	1514	1.78E+08	1.82E+08	1.22E+08	2.01E+08	2.36E+08	2.02E+08
tr|Q9RX19	tr|Q9RX19	1514			18194000		20178000	
sp|Q9RYL	sp|Q9RYL	436				2.63E+08	3.02E+08	
tr|Q9RXG8	tr|Q9RXG8	1591	17208000	83939000	75464000	27245000	86832000	63650000
tr|Q9RT34	tr|Q9RT34	810						
sp|Q5933	sp|Q5933	63			7430300			
sp|Q5933	sp|Q5933	63			41751000			
sp|Q9RSI4	sp|Q9RSI4	121					88466000	
tr|Q9RYT2	tr|Q9RYT2	1811	45580000	39925000	48497000	37501000		38306000
sp|Q9RUV	sp|Q9RUV	258	21081000	28033000	24407000		27295000	
tr|Q9RWB	tr|Q9RWB	1383	28543000	23735000	25696000	13300000	18999000	21349000
sp|Q9RVD	sp|Q9RVD	281				11429000		
tr|Q9RW9	tr|Q9RW9	1376					81718000	
tr|Q9RS41	tr|Q9RS41	641	22587000	43219000	38028000	20212000	62178000	66923000
sp|Q9RSR	sp|Q9RSR	143	1.04E+08	1.31E+08	1.22E+08	2.81E+08	1.87E+08	2.09E+08
tr|Q9RZL6	tr|Q9RZL6	1944						
tr|Q9RSA6	tr|Q9RSA6	677		10724000				
tr|Q9RT58	tr|Q9RT58	822	22806000	48502000	56119000		27414000	
sp|Q9RV7	sp|Q9RV7	269	18057000	34857000	27057000	48622000	83566000	40808000
tr|Q9RXP5	tr|Q9RXP5	1625	5.04E+08	4.3E+08	4.18E+08	4.08E+08	3.2E+08	3.38E+08
sp|Q9RU2	sp|Q9RU2	209	18748000	8175600	8833900	6169100	7459400	6359600
tr|Q9RRT6	tr|Q9RRT6	580	1.02E+08	1.2E+08	96134000	1.07E+08	1.03E+08	1E+08
tr|Q9RZQ	tr|Q9RZQ	1958				1.49E+08	1.03E+08	
tr|Q9RZ10	tr|Q9RZ10	1848	2.36E+08	2.83E+08	2.75E+08	1.86E+08	2.13E+08	2.02E+08
tr|Q9RYL0	tr|Q9RYL0	1767	2.93E+09	3E+09	3.31E+09	1.43E+09	1.53E+09	1.38E+09
tr|Q9RUE7	tr|Q9RUE7	1046						
tr|Q9RZK3	tr|Q9RZK3	1941						
sp|Q9RSG	sp|Q9RSG	118	1.16E+08	1.2E+08	1.04E+08	1.45E+08	1.64E+08	1.47E+08
tr|Q9RW2	tr|Q9RW2	1345	85543000	2.1E+08		2.33E+08	2.47E+08	2.33E+08
sp|Q9RU8	sp|Q9RU8	219		19830000	15675000	14200000	18059000	
tr|Q9RZQ	tr|Q9RZQ	1959	99251000	1.06E+08	85818000	87576000	73828000	80055000
sp|Q9RU2	sp|Q9RU2	209	1.04E+09	1.53E+09	2.39E+09	2.98E+09	1.27E+09	1.69E+09
tr|Q9RUC	tr|Q9RUC	1038	42014000	49863000	30717000	32058000		37177000
tr|Q9RU01	tr|Q9RU01	969				57970000	38429000	26967000
tr|Q9RU01	tr|Q9RU01	969	30549000	33266000	67735000		39711000	48662000
tr|Q9RRA0	tr|Q9RRA0	483					35349000	
tr|Q9RW9	tr|Q9RW9	1375						
tr|Q9RZ32	tr|Q9RZ32	1860	35426000	50619000	46111000		31249000	20556000
sp|Q9RXR	sp|Q9RXR	396	51854000		94820000			
tr|Q9RWP	tr|Q9RWP	1449	11298000	22127000	17017000			
sp|Q9RXJ4	sp|Q9RXJ4	379	3806500	3192300	4260300			
tr|Q9RVW	tr|Q9RVW	1311						
tr|Q9RVW	tr|Q9RVW	1311						
tr|Q9RY43	tr|Q9RY43	1693	1.64E+08					
tr|Q9RU17	tr|Q9RU17	976	61836000	74780000	1.39E+08			16934000
tr|Q9RYV0	tr|Q9RYV0	1820	2.21E+08	2.38E+08	2.05E+08	2.82E+08	2.63E+08	2.93E+08

tr|Q9RXG(	tr|Q9RXG(	1587	1.64E+08	1.43E+08	1.45E+08	1.3E+08	1.03E+08	94832000
tr|Q9RYL1	tr|Q9RYL1	1768	1.08E+08	1.2E+08	92758000	1.87E+08	1.52E+08	1.51E+08
sp|Q9RW1	sp|Q9RW1	306					20550000	
tr|Q9RX1(	tr|Q9RX1(	1507	5468700	2792200	3418000		1431300	2112000
tr|Q9RUT1	tr|Q9RUT1	1117	1.01E+08	48216000		98370000	82604000	84765000
sp|Q9RVI3	sp|Q9RVI3	289	3.77E+08	4.44E+08	4.12E+08	3.03E+08	2.99E+08	2.99E+08
tr|Q9RVB2	tr|Q9RVB2	1206	2.93E+08	3.6E+08	3.34E+08	5.79E+08	5.51E+08	6.21E+08
tr|Q9RX52	tr|Q9RX52	1528		33272000		22309000	20123000	20908000
tr|Q9RXI4	tr|Q9RXI4	1603						
tr|Q9RWS	tr|Q9RWS	1464						
tr|Q7DF71	tr|Q7DF71	464						
tr|Q9RW3	tr|Q9RW3	1350	88607000	86969000	96251000	16999000	20030000	15292000
sp|Q9WXF	sp|Q9WXF	461	3.19E+08	3.74E+08	2E+08	1.75E+08	1.8E+08	1.72E+08
sp|Q9RT2	sp|Q9RT2	164		41758000	15654000			
sp|Q9RR9	sp|Q9RR9	73	6.18E+08	6.91E+08	5.78E+08	8.29E+08	8.81E+08	8.31E+08
sp|Q9RVV	sp|Q9RVV	300	44114000	38667000	33575000	63159000	54044000	55750000
sp|Q9RW(	sp|Q9RW(	326	1.93E+08	2.49E+08	2.43E+08	8.07E+08	5.92E+08	5.59E+08
tr|Q9RTP5	tr|Q9RTP5	909	1.38E+08	1.31E+08	1.54E+08	69852000	1.28E+08	73860000
tr|Q9RZC4	tr|Q9RZC4	1903	3.77E+08	3.85E+08	3.67E+08	7.19E+08	5.55E+08	6.29E+08
tr|Q9RXM	tr|Q9RXM	1617	58983000	1.35E+08	87254000	10042000	56994000	30742000
tr|Q9RY37	tr|Q9RY37	1690	6.13E+08	6.25E+08	6.15E+08	3.18E+08	2.73E+08	2.52E+08
sp|Q9RYL	sp|Q9RYL	436	1.46E+08	1.06E+08	78745000	1.42E+08	1.47E+08	1.43E+08
tr|Q9RUM	tr|Q9RUM	1089	98092000	97339000	82760000	1.39E+08	1.22E+08	98081000
tr|Q9RUB	tr|Q9RUB	1031	86564000	1.01E+08	1.07E+08	1.57E+08	1.21E+08	1.75E+08
sp|Q9RY6	sp|Q9RY6	421	2.32E+08	1.95E+08	2.21E+08			
tr|Q9RZ07	tr|Q9RZ07	1845						
tr|Q9RUX8	tr|Q9RUX8	1136	1.89E+08	2E+08	1.83E+08	6.71E+08	6.72E+08	5.24E+08
tr|Q9RXX2	tr|Q9RXX2	1660	2.32E+08	1.93E+08	2.32E+08	9840300	17568000	10624000
tr|Q9RVP(	tr|Q9RVP(	1266	2.93E+08	2.64E+08	2.01E+08	3.13E+08	31077000	2.55E+08
tr|Q9RVP(	tr|Q9RVP(	1266	3.19E+08	3.55E+08	4.28E+08	66801000	91203000	1.39E+08
tr|Q9RZA9	tr|Q9RZA9	1893	36878000		11943000	37774000	26572000	
tr|Q9RU78	tr|Q9RU78	1014	1.51E+08	1.45E+08	1.24E+08	1.55E+08	1.34E+08	1.47E+08
tr|Q9RT42	tr|Q9RT42	814	45800000	45925000	40051000	1.01E+08	98660000	65472000
tr|Q9RUD	tr|Q9RUD	1040	7.24E+08	7.36E+08	5.86E+08	1.15E+09	1.05E+09	1.02E+09
tr|Q9RYS7	tr|Q9RYS7	1806	1.27E+08	1.29E+08	1.01E+08	2.34E+08	2.05E+08	2.1E+08
sp|Q9RYR	sp|Q9RYR	441	1.13E+08	1.21E+08	93304000	97361000	83640000	89851000
tr|Q9RWN	tr|Q9RWN	1441	74475000	69233000	64489000	70895000		80885000
tr|Q9RVG6	tr|Q9RVG6	1228						11641000
tr|Q9RZT4	tr|Q9RZT4	1971	52590000			23098000	20310000	
tr|Q9RZC5	tr|Q9RZC5	1904			66100000	39357000	41417000	53241000
tr|Q9RXH4	tr|Q9RXH4	1596	19834000	22759000	26936000	48682000		60665000
tr|Q9RZD1	tr|Q9RZD1	1907	61594000	34825000	51457000			
sp|Q9RY6	sp|Q9RY6	421				15690000	21003000	26089000
sp|Q9RY0	sp|Q9RY0	410	66362000	66662000	69087000		29388000	
tr|Q9RRP2	tr|Q9RRP2	556			31150000	17582000	18976000	31791000
tr|Q9RZQ	tr|Q9RZQ	1958		23156000			12107000	4.28E+08
tr|Q9RXR9	tr|Q9RXR9	1636				6.44E+08	5.95E+08	5.85E+08
tr|Q9RXR9	tr|Q9RXR9	1636	5.75E+08	7.71E+08	1.55E+09	2.06E+08	6.93E+08	6.45E+08
tr|Q9RXP7	tr|Q9RXP7	1626		2470600	1343300	13427000	13035000	9857000
tr|Q9RXP7	tr|Q9RXP7	1626			59415000		49293000	47295000
sp|Q9RSY	sp|Q9RSY	157	34741000	36849000	33654000	1.11E+08	1.04E+08	1.36E+08

tr|Q9RWC	tr|Q9RWC	1389	68774000	73097000	50577000	78789000	62872000	59323000
tr|Q9RWA	tr|Q9RWA	1379	1.48E+08	97388000	4.21E+08	4.83E+08	4.35E+08	4.63E+08
tr|Q9RTX7	tr|Q9RTX7	952	1.12E+08	1.02E+08	1.02E+08	41136000	34455000	51685000
tr|Q9RUD	tr|Q9RUD	1043		56504000	88275000			
tr|Q9RV7	tr|Q9RV7	1186	21950000		39057000	51139000		89293000
tr|Q9RVL6	tr|Q9RVL6	1253	25997000	21108000	8785400	42840000		
tr|Q9RWH	tr|Q9RWH	1412	87460000			1.36E+08		
tr|Q9RT16	tr|Q9RT16	801						
sp|Q9RW	sp|Q9RW	310	37336000	35653000	40041000	1.1E+08	24475000	17550000
tr|Q9RSH	tr|Q9RSH	717	86697000	2.33E+08	1.49E+08		72066000	55266000
tr|Q9RZI6	tr|Q9RZI6	1937						
tr|Q9RT9	tr|Q9RT9	841	18763000					
sp|Q9RW	sp|Q9RW	341	1209400	301270	299330			
tr|Q9RSM	tr|Q9RSM	738	67791000		73122000	72881000	91590000	82567000
tr|Q9RSM	tr|Q9RSM	738	29895000	84043000	96883000		27924000	54077000
sp|Q9RU	sp|Q9RU	236						
tr|Q9RSX3	tr|Q9RSX3	778						
tr|Q9RTV1	tr|Q9RTV1	936	1.44E+08	1.22E+08	1.56E+08	1.91E+08	2.34E+08	1.75E+08
tr|Q9RUA	tr|Q9RUA	1023				76520000		
tr|Q9RXX5	tr|Q9RXX5	1662	67283000		66966000		57494000	
sp|Q9RTN	sp|Q9RTN	196	58031000	1.13E+08	21738000	89122000	1.74E+08	1.09E+08
tr|Q9RRV4	tr|Q9RRV4	591	1.76E+09	1.29E+09	1.15E+09	7.42E+08	8.08E+08	8.96E+08
tr|Q9RRX1	tr|Q9RRX1	601				31198000	24332000	
tr|Q9RRX1	tr|Q9RRX1	601				16844000	30746000	17326000
tr|Q9RRX1	tr|Q9RRX1	601	41931000	1.09E+08	2.81E+08	3.04E+08	3.6E+08	2.62E+08
sp|P8358	sp|P8358	60			17266000			
sp|Q9RSL	sp|Q9RSL	135	2.68E+09	2.64E+09	2.46E+09	2.71E+09	2.3E+09	2.73E+09
sp|Q9RSL	sp|Q9RSL	135			34353000	50149000	59710000	69065000
tr|Q9RZH8	tr|Q9RZH8	1932			55712000			
tr|Q9RU2	tr|Q9RU2	980	1.05E+08	1.76E+08	66903000	3.62E+08	2.5E+08	2.87E+08
tr|Q9RU2	tr|Q9RU2	980		46290000	1.09E+08	84542000	1.28E+08	85655000
tr|Q9RZ32	tr|Q9RZ32	1860	92122000	1.03E+08	61859000		21723000	27461000
tr|Q9RT75	tr|Q9RT75	831		19322000	21423000	36044000	1.5E+08	
sp|Q9RUE	sp|Q9RUE	229	2.61E+09	1.99E+09				
sp|Q9RW	sp|Q9RW	338	20429000	30459000	37550000	23384000	17607000	19190000
tr|Q9RR62	tr|Q9RR62	467	2.12E+08	2.59E+08	2.37E+08	1.3E+08		1.3E+08
tr|Q9RZV2	tr|Q9RZV2	1982						
tr|Q9RRU	tr|Q9RRU	585		29731000	45979000	30593000	19486000	
tr|Q9RW7	tr|Q9RW7	1362	1.23E+08	1.81E+08		96387000		1.55E+08
tr|Q9RUW	tr|Q9RUW	1126	1.32E+08	1.23E+08	93629000	82858000	66746000	72289000
tr|Q9RUJ6	tr|Q9RUJ6	1072	35873000	34446000	54484000	65455000	78524000	61922000
sp|Q9RS1	sp|Q9RS1	107	3.05E+08	2.57E+08		3.36E+08	2.75E+08	3.31E+08
sp|Q9RSQ	sp|Q9RSQ	142						15957000
tr|Q9RUR	tr|Q9RUR	1110	5.07E+08	5.45E+08	4.17E+08	4.9E+08	5.37E+08	5.09E+08
tr|Q9RZD	tr|Q9RZD	1910	58822000	89987000	62486000	1.53E+08	1.45E+08	1.53E+08
tr|Q9RT87	tr|Q9RT87	837	9161700	18663000	15827000	4722600		8348700
sp|Q9RUK	sp|Q9RUK	238	6.15E+08	4.68E+08	4.4E+08		20283000	
sp|Q9RXJ	sp|Q9RXJ	384			56826000	41424000	25964000	26826000
sp|Q9RXJ	sp|Q9RXJ	384			1.01E+08	1.34E+08	89161000	1.31E+08
tr|Q9RXB	tr|Q9RXB	1566	1.57E+08	1.31E+08	1.31E+08	71609000	2.59E+08	2.45E+08
sp|Q9RXT	sp|Q9RXT	400	5.38E+08	7.3E+08	5.69E+08	6.56E+08	5.6E+08	6.64E+08

sp|Q9RY5	sp|Q9RY5	417	1657400	535980	391180	524530	969610	574550
sp|Q9RY5	sp|Q9RY5	417		56734000	4.31E+08	97078000	1.28E+08	1.56E+08
tr|Q9RZE6	tr|Q9RZE6	1916	1.1E+08	1.49E+08		1.37E+08	1.24E+08	1.3E+08
sp|Q9RTL	sp|Q9RTL	194	1.47E+08	1.42E+08	1.33E+08	1.27E+08	1.41E+08	1.32E+08
sp|Q9RW	sp|Q9RW	349				52519000	94990000	
tr|Q9RXF9	tr|Q9RXF9	1586	26909000					
tr|Q9RTZ9	tr|Q9RTZ9	967	44934000	40343000	41388000	1.17E+08	80424000	1.12E+08
tr|Q9RTZ9	tr|Q9RTZ9	967			19245000	18802000	19122000	17643000
sp|Q9RTJ3	sp|Q9RTJ3	189						
tr|Q9RTR4	tr|Q9RTR4	923	14214000	18959000				
tr|Q9LBE3	tr|Q9LBE3	466	1.85E+08	1.58E+08	1.39E+08	1.8E+08	1.76E+08	1.37E+08
tr|Q9RYR7	tr|Q9RYR7	1799				30789000	32825000	
tr|Q9RY02	tr|Q9RY02	1675	3.74E+08	2.59E+08	3.39E+08	2.06E+08	1.51E+08	2.02E+08
tr|Q9RTX5	tr|Q9RTX5	950	1.04E+09	1.16E+09	8.68E+08	1.45E+09	1.34E+09	1.55E+09
tr|Q9RZQ	tr|Q9RZQ	1960	1.3E+08	1.05E+08	74294000	50222000		
tr|Q9RYP4	tr|Q9RYP4	1783	86679000	72988000	66642000	95084000	75071000	80294000
sp|Q4657	sp|Q4657	61						
tr|Q9RS50	tr|Q9RS50	645						
sp|Q9RSA	sp|Q9RSA	114						
tr|Q9RXY9	tr|Q9RXY9	1667				61208000		37704000
tr|Q9RWQ	tr|Q9RWQ	1455			16095000			
sp|Q9RW0	sp|Q9RW0	327	2.23E+08	2.81E+08	2.56E+08	5.03E+08	3.93E+08	4.5E+08
sp|Q9RW0	sp|Q9RW0	327			49721000	29106000		21641000
tr|Q9RYK8	tr|Q9RYK8	1766		18784000	23019000		20413000	61901000
tr|Q9RXP5	tr|Q9RXP5	1625				72960000		
sp|Q9RRB	sp|Q9RRB	76						
tr|Q9RRX1	tr|Q9RRX1	601			8139400			
tr|Q9RWP	tr|Q9RWP	1449	5.76E+08	5.67E+08	5.41E+08	5.95E+08	5.69E+08	5.63E+08
tr|Q9RWP	tr|Q9RWP	1449	25417000	34644000	64196000			
tr|Q9RVQ	tr|Q9RVQ	1276				43466000		53985000
sp|Q9RRE	sp|Q9RRE	83	33411000		33355000			
tr|Q9RZ10	tr|Q9RZ10	1848	93479000	1.36E+08	2.69E+08	19089000	20094000	32195000
tr|Q9RVL5	tr|Q9RVL5	1252						
tr|Q9RTU7	tr|Q9RTU7	935		20079000	64165000			
tr|Q9RZL7	tr|Q9RZL7	1945				22071000	30625000	29650000
sp|Q9RU1	sp|Q9RU1	207		45690000				
sp|Q9RYM	sp|Q9RYM	438	1.92E+08	1.38E+08	4.73E+08	9.65E+08	4.39E+08	3.93E+08
sp|Q5933	sp|Q5933	63	2.38E+09	2.73E+09	2.28E+09	2.02E+09	1.95E+09	1.79E+09
tr|Q9RYV3	tr|Q9RYV3	1821	65215000	78909000	94148000	46134000	67003000	49015000
tr|Q9RYV3	tr|Q9RYV3	1821			39887000			
tr|Q9RVM	tr|Q9RVM	1255	31925000		37961000			39505000
tr|Q9RWI0	tr|Q9RWI0	1413		9392100		27463000	23578000	
tr|Q9RYL0	tr|Q9RYL0	1767	8.63E+08	1.57E+09	8.01E+08	7.69E+08	8.72E+08	6.17E+08
tr|Q9RYL0	tr|Q9RYL0	1767			29506000	19918000	21944000	
tr|Q9RU45	tr|Q9RU45	992	22653000	22950000	23907000		22358000	
tr|Q9RT06	tr|Q9RT06	795						
tr|Q9RT06	tr|Q9RT06	795						
tr|Q9RX95	tr|Q9RX95	1553						
tr|Q9RYI7	tr|Q9RYI7	1763	56516000	72308000	18600000	67892000	55492000	73974000
tr|Q9RTH4	tr|Q9RTH4	877	1.53E+08	2.09E+08	2.12E+08	1.24E+08	1.33E+08	1.3E+08
tr|Q9RWF	tr|Q9RWF	1402	36704000			30874000	34718000	32034000

tr|Q9RVX1	tr|Q9RVX1	1315	1.03E+08	69143000	2.18E+08	3.5E+08	69477000	3.11E+08
sp|Q9RRY	sp|Q9RRY	103						
sp|Q9RRY	sp|Q9RRY	103	1.95E+08	2.35E+08	2E+08			
sp|Q9RXI	sp|Q9RXI	374	8606300	4173000	4537900			
tr|Q9RTJ1	tr|Q9RTJ1	883	1.42E+08	1.69E+08	1.12E+08	1.52E+08	1.86E+08	1.65E+08
tr|Q9RUT	tr|Q9RUT	1120	21338000	15585000	11709000	17604000	17592000	24643000
tr|Q9RSW	tr|Q9RSW	775	7.52E+08	7.05E+08	7.27E+08	7.01E+08	5.33E+08	6.37E+08
tr|Q9RUC	tr|Q9RUC	1036						
sp|Q9RR7	sp|Q9RR7	67	55761000	55616000	64946000			5494300
tr|Q9RYN	tr|Q9RYN	1775	2E+08	2.19E+08	5.21E+08	54134000		81959000
sp|Q9RYD	sp|Q9RYD	430		27752000	34392000		16027000	
tr|Q9RWW	tr|Q9RWW	1493	1.73E+09	1.14E+09	1.96E+09	5.27E+08	6.27E+08	8.19E+08
tr|Q9RZU	tr|Q9RZU	1979						
tr|Q9RSQ	tr|Q9RSQ	748	19162000		1.33E+08			
sp|Q9RUN	sp|Q9RUN	242			38259000			
tr|Q9RVL	tr|Q9RVL	1250		2.31E+08	2.1E+08	1.6E+08	1.25E+08	1.33E+08
tr|Q9RRW	tr|Q9RRW	595					80071000	
tr|Q9RYD	tr|Q9RYD	1734	2.53E+08	2.91E+08	40458000	19470000	1.45E+08	2.06E+08
tr|Q9RZA1	tr|Q9RZA1	1888	27548000	23529000	52880000	38063000	46485000	50705000
sp|Q9RW	sp|Q9RW	328					38051000	
tr|Q9RSF6	tr|Q9RSF6	709	20118000	21165000	23018000	18582000		
tr|Q9RTY1	tr|Q9RTY1	956	1.67E+08	1.71E+08	1.6E+08		17356000	
sp|Q9RSK	sp|Q9RSK	131	24526000	10791000	10682000	6535000	7451700	8693300
tr|Q9RRV	tr|Q9RRV	591	2.17E+08	1.83E+08	1.39E+08	9.14E+08	8.9E+08	7.29E+08
tr|Q9RRV	tr|Q9RRV	591			34113000	42791000	37551000	60459000
sp|Q9RTQ	sp|Q9RTQ	197	3.53E+08	3.4E+08	2.91E+08	2.69E+08	2.18E+08	2.48E+08
tr|Q9RT7	tr|Q9RT7	827	15649000	27912000	49910000			
sp|Q9RRX	sp|Q9RRX	101	40663000		69651000	1.03E+08	89728000	66831000
tr|Q9RWT	tr|Q9RWT	1468	7893100	5372300	6389700	7218600	9221900	9165200
tr|Q9RT0	tr|Q9RT0	798	1.84E+09	1.63E+09	1.54E+09	2.01E+09	1.75E+09	2.01E+09
sp|Q9RY5	sp|Q9RY5	418	6.16E+08	5.53E+08	6E+08	1.08E+09	9.23E+08	1.18E+09
tr|Q9RWV	tr|Q9RWV	1482				2.87E+08	2.41E+08	3.03E+08
tr|Q9RTX7	tr|Q9RTX7	952	12267000		22909000	8.54E+08		17111000
tr|Q9RY7	tr|Q9RY7	1705	68931000	65854000	73868000			1.21E+08
tr|Q9RVN	tr|Q9RVN	1262	2.4E+08	2.15E+08	2.62E+08	2.68E+08	2.57E+08	2.87E+08
sp|Q9RTI	sp|Q9RTI	187	9.58E+08	7.64E+08	8.15E+08	1.37E+08		1.86E+08
tr|Q9RTI9	tr|Q9RTI9	882	10738000			34469000	22990000	20704000
tr|Q9RS5	tr|Q9RS5	650				82800000	82066000	84077000
tr|Q9RYF4	tr|Q9RYF4	1743	1.31E+08	1.14E+08	1.18E+08	21164000	22673000	23298000
sp|Q9RXK	sp|Q9RXK	387	2.79E+09	2.66E+09	2.66E+09	1.72E+09	1.51E+09	1.74E+09
tr|Q9RX4	tr|Q9RX4	1526	88484000	83096000	72266000	55916000	37045000	50925000
tr|Q9RT9	tr|Q9RT9	840						
tr|Q9RTK	tr|Q9RTK	888	82261000	1.07E+08	1.05E+08	59769000	68406000	82159000
tr|Q9RWA	tr|Q9RWA	1379	82688000	85484000	58380000			
sp|Q9RW	sp|Q9RW	314	20582000	11378000	44091000	25888000		28348000
sp|Q9RU8	sp|Q9RU8	218	6311400			2449300	3027700	2171300
tr|Q9RTP	tr|Q9RTP	911						
tr|Q9RVV	tr|Q9RVV	1304					19468000	
sp|Q9RSA	sp|Q9RSA	114	2.81E+08	2.64E+08	4.06E+08	1E+08	1.68E+08	1.64E+08
tr|Q9RSV7	tr|Q9RSV7	772	26025000	19131000	21764000			1.23E+08
tr|Q9RSE4	tr|Q9RSE4	703	1.65E+08	1.5E+08	1.39E+08	82264000	1.5E+08	1.02E+08

tr|Q9RSE4	tr|Q9RSE4	703			19942000			
tr|Q9RT31	tr|Q9RT31	808	56959000		22938000	67114000	11257000	46286000
tr|Q9RXB(	tr|Q9RXB(	1561	25222000	43657000	32664000			
tr|Q9RYN!	tr|Q9RYN!	1779	51521000	49557000	47426000	52624000	49079000	62644000
tr|Q9RZB3	tr|Q9RZB3	1896	1.02E+08	82841000	98316000	2.3E+08	1.51E+08	2.36E+08
tr|Q9RYL0	tr|Q9RYL0	1767	41014000	2.47E+08	2.18E+08	2.49E+08	5.02E+08	1.7E+08
sp|Q9RW(	sp|Q9RW(	304	1.63E+08	2.44E+08	2.2E+08	1.31E+08	1.75E+08	96520000
tr|Q9RWT	tr|Q9RWT	1469		4115500	2863100	8604700	8524400	5480600
tr|Q9RXF5	tr|Q9RXF5	1584	65894000	46270000	45324000			
tr|Q9RU5(	tr|Q9RU5(	1002	23544000	18351000	84282000	62413000	49117000	
sp|Q9RU2	sp|Q9RU2	209				33667000	47107000	30688000
tr|Q9RZ32	tr|Q9RZ32	1860	1.72E+08	1.48E+08	1.17E+08	1.28E+08		1.8E+08
tr|Q9RUJ8	tr|Q9RUJ8	1073						
tr|Q9RUJ8	tr|Q9RUJ8	1073	19393000	31857000	39807000			
tr|Q9RV9(	tr|Q9RV9(	1195	32220000	30803000	52081000	70461000	86533000	72807000
tr|Q9RZD8	tr|Q9RZD8	1912						
sp|Q9RYP	sp|Q9RYP	440	2.32E+08		1.74E+08	2.76E+08	2.73E+08	2.81E+08
tr|Q9RZC7	tr|Q9RZC7	1906				6665000		9082600
tr|Q9RXB6	tr|Q9RXB6	1565	6313900	4778000	5337700		18540000	21280000
tr|Q9RST3	tr|Q9RST3	759		21701000				15345000
tr|Q9RUK(	tr|Q9RUK(	1075	50090000	43399000	38808000	1.49E+08	1.85E+08	2.21E+08
tr|Q9RX57	tr|Q9RX57	1532	10166000	11906000	9026000	28477000	18927000	25428000
tr|Q9RX57	tr|Q9RX57	1532	2.06E+08	1.69E+08	2.14E+08	3.3E+08	1.55E+08	2.56E+08
sp|P56867	sp|P56867	56						
tr|Q9RUM	tr|Q9RUM	1092		21065000				
tr|Q9RYD(	tr|Q9RYD(	1730						
tr|Q9RYD(	tr|Q9RYD(	1730		22511000			14228000	9900000
tr|Q9RX85	tr|Q9RX85	1550	19381000	37405000	21281000	8355700		
tr|Q9RYL0	tr|Q9RYL0	1767	1.73E+08		3.83E+08			
tr|Q9RUK	tr|Q9RUK	1079	41510000	66959000	68478000	1.28E+08	7238500	1.24E+08
sp|Q9RT9	sp|Q9RT9	173	91029000	81326000	69976000	1.41E+08	1.44E+08	1.43E+08
tr|Q9RZ18	tr|Q9RZ18	1853	7.55E+08	7.16E+08	6.8E+08	2.12E+09	1.78E+09	1.46E+09
sp|Q9RYM	sp|Q9RYM	438		95154000	53720000	1.2E+08	96146000	1.18E+08
sp|Q9RYM	sp|Q9RYM	438	3.76E+09	4.25E+09	5.67E+09	1.74E+09	2.48E+09	2.84E+09
sp|Q9RVY	sp|Q9RVY	303				1.88E+08	1.64E+08	1.62E+08
tr|Q9RYK3	tr|Q9RYK3	1765	21173000	27551000	23824000			
tr|Q9RRW	tr|Q9RRW	597	2.19E+08	1.5E+08	2.19E+08		76520000	1.41E+08
sp|Q9RRB	sp|Q9RRB	76		50419000				72355000
tr|Q9RX1(	tr|Q9RX1(	1507	1.17E+08	1.02E+08	1.03E+08	3.99E+08	3.98E+08	2.84E+08
tr|Q9RSN!	tr|Q9RSN!	742	72549000			1.19E+08	75084000	78701000
tr|Q9RSN!	tr|Q9RSN!	742	41396000	60713000	94947000			
tr|Q9RY79	tr|Q9RY79	1708					6494900	
sp|Q9RV7	sp|Q9RV7	268	27894000	25271000	23239000	79115000	61305000	58573000
tr|Q9RUU	tr|Q9RUU	1121	14700000		10726000	20608000	20169000	20669000
sp|Q9RX2	sp|Q9RX2	355	1.28E+08	2.21E+08	1.9E+08	2.85E+08	2.41E+08	2.46E+08
tr|Q9RX55	tr|Q9RX55	1530				24380000		16491000
sp|Q9RZ0	sp|Q9RZ0	450	3E+08	2.64E+08	2.89E+08	1.76E+08	1.33E+08	2.13E+08
tr|Q9RY83	tr|Q9RY83	1709						
sp|Q9RRB	sp|Q9RRB	75	54647000					
tr|Q9RTR8	tr|Q9RTR8	925	49138000	43125000	52669000	68369000	50603000	45884000
sp|Q9RW5	sp|Q9RW5	310	39489000		45640000		21336000	

sp|Q9RW(	sp|Q9RW(	325	3.16E+08	2.41E+08	3.14E+08	98726000	1.3E+08	1.87E+08
tr|Q9RXF5	tr|Q9RXF5	1584						
tr|Q9RZU4	tr|Q9RZU4	1977						
tr|Q9RXF0	tr|Q9RXF0	1581			7226700	15057000	16010000	22872000
tr|Q9RWI(	tr|Q9RWI(	1413	12858000		14056000	24721000	21399000	26688000
tr|Q9RXI4	tr|Q9RXI4	1603				21200000	12446000	8751800
tr|Q9RXI4	tr|Q9RXI4	1603	21663000	37005000	48044000	64190000	58178000	36864000
tr|Q9RSD(	tr|Q9RSD(	692						
tr|Q9RZC4	tr|Q9RZC4	1903	23020000	27062000	61226000	53322000		62214000
tr|Q9RTZ9	tr|Q9RTZ9	967	1.73E+08	1.96E+08	1.74E+08			4.33E+08
tr|Q9RR62	tr|Q9RR62	467		1.22E+08	1.63E+08	57751000	1.58E+08	72650000
sp|Q9RXI	sp|Q9RXI	374	29400000	2.21E+08	4.68E+08	2.13E+08	1.78E+08	2.32E+08
sp|Q9RVV	sp|Q9RVV	300					20977000	
tr|Q9RRE7	tr|Q9RRE7	513						
tr|Q9RSA5	tr|Q9RSA5	676	1.24E+08	1.24E+08	99118000			1.3E+08
sp|P56867	sp|P56867	56	1.63E+08	1.74E+08	1.28E+08	5.6E+08	4.17E+08	4E+08
sp|P56867	sp|P56867	56	1.57E+09	1.57E+09	1.98E+09	1.59E+09	1.83E+09	2.72E+09
tr|Q9RT26	tr|Q9RT26	804	40970000	34485000	35436000	57615000	54935000	67804000
tr|Q9RRY2	tr|Q9RRY2	605				24837000	25676000	16405000
tr|Q9RUT1	tr|Q9RUT1	1117	29750000	34739000		85490000	83890000	70627000
tr|Q9RUG	tr|Q9RUG	1055	62175000	51826000	44215000	72826000	61832000	64786000
sp|Q9RVV	sp|Q9RVV	300	24686000					
tr|Q9RXG(	tr|Q9RXG(	1587	18474000	28993000		46726000	54564000	43296000
sp|Q9RVH	sp|Q9RVH	288	2.78E+08	2.4E+08	2.43E+08	1.81E+08	1.09E+08	1.23E+08
tr|Q9RWA	tr|Q9RWA	1379						
tr|Q9RYI0	tr|Q9RYI0	1759						
tr|Q9RZ32	tr|Q9RZ32	1860	22778000					
sp|Q9RW(	sp|Q9RW(	342	7893600	4303200	4731800	5709100	5841000	5324700
tr|Q9RYD9	tr|Q9RYD9	1735	20304000	14823000	13910000			45299000
sp|Q9RVW	sp|Q9RVW	301		28189000		6390300		
sp|Q9RVW	sp|Q9RVW	301						
sp|Q9RSL1	sp|Q9RSL1	135	1.76E+08	1.75E+08	1.35E+08	1.88E+08	1.88E+08	1.76E+08
sp|Q9RSL1	sp|Q9RSL1	135						
sp|Q9RSS	sp|Q9RSS	149	1.81E+08	1.66E+08	1.51E+08	2.21E+08	2.01E+08	2.48E+08
tr|Q9RZ99	tr|Q9RZ99	1886	11022000		27133000	25851000	17553000	
tr|Q9RS98	tr|Q9RS98	672			37191000			
tr|Q9RS98	tr|Q9RS98	672	80197000	48533000	97583000	1.53E+08	1.29E+08	1.35E+08
sp|Q9RV9	sp|Q9RV9	271	63002000	50103000	72649000		22855000	
tr|Q9RSJ2	tr|Q9RSJ2	728	15182000	12091000		17463000	17527000	22682000
sp|Q9RXI7	sp|Q9RXI7	373						
tr|Q9RX57	tr|Q9RX57	1532	747620		820000	1952800	1603900	
tr|Q9RX57	tr|Q9RX57	1532	57149000	40183000	39760000		36627000	
sp|Q9RTJ5	sp|Q9RTJ5	190						
tr|Q9RSY1	tr|Q9RSY1	782						
tr|Q9RRT7	tr|Q9RRT7	581						
tr|Q9RTA2	tr|Q9RTA2	844	30223000	35870000				17766000
tr|Q9RXI4	tr|Q9RXI4	1603	20959000	21231000	20625000			
tr|Q9RSN4	tr|Q9RSN4	741		9481200	18970000			
sp|Q9RXT	sp|Q9RXT	400					19278000	21447000
tr|Q9RZC3	tr|Q9RZC3	1902			19733000			
tr|Q9RSR9	tr|Q9RSR9	754	38053000	48420000	25419000	67803000	61187000	56358000

tr|Q9RZQ	tr|Q9RZQ	1960						
tr|Q9RRF4	tr|Q9RRF4	516	25987000	26551000	15482000	30326000	24721000	16097000
tr|Q9RUF8	tr|Q9RUF8	1049	4.76E+08	6.27E+08	5.58E+08	7.56E+08	9.93E+08	7.28E+08
tr|Q9RZ03	tr|Q9RZ03	1843	6.65E+08	4.97E+08			46032000	43151000
tr|Q9RRI1	tr|Q9RRI1	530	86083000	72117000	57903000	70611000	73813000	78418000
sp|Q9RUE	sp|Q9RUE	226		31114000	62655000		17060000	26020000
sp|Q9RUE	sp|Q9RUE	226						
sp|Q9RVH	sp|Q9RVH	288	2.02E+08	2.56E+08	2.35E+08	1.57E+08	1.24E+08	1.18E+08
tr|Q9RRU4	tr|Q9RRU4	585						
sp|P42443	sp|P42443	49				1.92E+08		
sp|P42443	sp|P42443	49	5356600					
tr|Q9RWU	tr|Q9RWU	1475						
tr|Q9RTZ1	tr|Q9RTZ1	962	26437000		21312000			
tr|Q9RUS7	tr|Q9RUS7	1114	46235000		38398000			
tr|Q9RSA6	tr|Q9RSA6	677	5.85E+08	5.59E+08	5.1E+08	78397000	99239000	89727000
tr|Q9RZE4	tr|Q9RZE4	1914			2394100			2230800
tr|Q9RY56	tr|Q9RY56	1698	4.09E+09	3.48E+09	3.95E+09	1.29E+09	1.3E+09	1.6E+09
tr|Q9RWI1	tr|Q9RWI1	1414	60080000	33533000			1.35E+08	18162000
tr|Q9RSL8	tr|Q9RSL8	734	3.44E+08	3.57E+08	2.57E+08	3.74E+08	3.28E+08	3.51E+08
sp|Q9RW0	sp|Q9RW0	342	71545000	77488000	81533000	1.58E+08	1.47E+08	1.58E+08
tr|Q9RS66	tr|Q9RS66	653			32410000			
tr|Q9RYN1	tr|Q9RYN1	1776	1.91E+08					
sp|Q9RUC	sp|Q9RUC	248			21392000			41237000
tr|Q9RXQ	tr|Q9RXQ	1627	37134000	28182000	30601000			
tr|Q9RUF8	tr|Q9RUF8	1049				17307000	17070000	13141000
tr|Q9RS59	tr|Q9RS59	650			13277000			19778000
sp|Q9RSK	sp|Q9RSK	131	65529000	60787000	61471000	60170000	59409000	68356000
sp|Q9RW0	sp|Q9RW0	326	6.15E+08	1.68E+09	1.44E+09	7.26E+08	7.18E+08	6.09E+08
sp|Q9RXU	sp|Q9RXU	402	22885000		26202000	17148000	19909000	11864000
tr|Q9RR98	tr|Q9RR98	481	7.16E+08	9.06E+08	6.76E+08	6.28E+08	5.98E+08	6.8E+08
tr|Q9RSF8	tr|Q9RSF8	710			23115000	23136000	29489000	
tr|Q9RX48	tr|Q9RX48	1526				29440000	22706000	34820000
sp|Q9RUV	sp|Q9RUV	254	2.78E+08	2.57E+08	2.31E+08	2.65E+08	2.6E+08	2.69E+08
sp|Q9RUV	sp|Q9RUV	254			17023000		7769000	
sp|Q9RV6	sp|Q9RV6	267	2.55E+08	2.95E+08	2.27E+08	1.43E+09	1.17E+09	1.36E+09
tr|Q9RVP1	tr|Q9RVP1	1267	1.79E+09	1.94E+09	1.63E+09	1.54E+09	1.16E+09	1.32E+09
tr|Q9RVP1	tr|Q9RVP1	1267		27917000				
sp|Q9RYV	sp|Q9RYV	445	30292000	32168000	24600000		25678000	35010000
tr|Q9RZ22	tr|Q9RZ22	1854			18671000			
tr|Q9RRW	tr|Q9RRW	595	75920000	71707000	75930000	1.29E+08	82894000	1.09E+08
tr|Q9RZ81	tr|Q9RZ81	1875						
tr|Q9RRP8	tr|Q9RRP8	560	10678000				16360000	
tr|Q9RSY6	tr|Q9RSY6	784						
tr|Q9RSR9	tr|Q9RSR9	754		16889000	18033000			
tr|Q9RY79	tr|Q9RY79	1708	72193000	88706000	1.12E+08		49652000	76880000
tr|Q9RX57	tr|Q9RX57	1532	1766800	978720	792290	983850	815620	1304800
sp|Q9RYD	sp|Q9RYD	429						
tr|Q9RWP	tr|Q9RWP	1449	53656000	46718000	53103000	16606000	21447000	
tr|Q9RRC7	tr|Q9RRC7	501	1.51E+08	3.46E+08	2.74E+08	3.27E+08	2.67E+08	1.11E+08
tr|Q9RRC7	tr|Q9RRC7	501						
tr|Q9RYN6	tr|Q9RYN6	1780	33467000	27592000	27237000	19571000	21991000	

tr|Q9RYN	tr|Q9RYN	1780						
tr|Q9RSD	tr|Q9RSD	695	71732000	69357000	57302000	9333700		
sp|Q9RUN	sp|Q9RUN	242			4.07E+08	1.89E+08	1.7E+08	2.39E+08
tr|Q9RXH	tr|Q9RXH	1599	19884000					
tr|Q9RV9	tr|Q9RV9	1198	2.11E+08	2.9E+08	1.94E+08	2.57E+08		
sp|Q9RXJ	sp|Q9RXJ	381				12915000	15660000	12230000
tr|Q9RTY	tr|Q9RTY	960	1.78E+08	1.3E+08	1.75E+08			
tr|Q9RSE	tr|Q9RSE	704	8.1E+08		8.96E+08		3.19E+08	5.89E+08
tr|Q9RZ0	tr|Q9RZ0	1846	77576000	58571000	66833000			
tr|Q9RY9	tr|Q9RY9	1715	28906000	35104000	42878000		26392000	42762000
tr|Q9RX41	tr|Q9RX41	1522	73612000	82565000	93270000	59443000	63889000	59842000
tr|Q9RXQ	tr|Q9RXQ	1628	26673000	27878000	33270000	21768000	19714000	30634000
tr|Q9RRZ	tr|Q9RRZ	615	25799000	21268000	24467000	18677000	15192000	16415000
sp|Q9RSS	sp|Q9RSS	150				1.03E+08	1.06E+08	1.02E+08
sp|Q9RXW	sp|Q9RXW	404		14692000	15310000	14720000		
tr|Q9RXG	tr|Q9RXG	1587	23550000		17402000		15311000	
tr|Q9RZL7	tr|Q9RZL7	1945	3.34E+08	1.74E+08	2.84E+08	2.33E+08	2.46E+08	3.94E+08
sp|Q9RS2	sp|Q9RS2	108						
sp|Q9RU7	sp|Q9RU7	215	1.43E+08	99384000	93035000	1.96E+08	1.74E+08	1.88E+08
sp|Q9RU7	sp|Q9RU7	215	1.57E+09	1.14E+09	1.69E+09	1E+09	8.44E+08	1.18E+09
tr|Q9RV4	tr|Q9RV4	1172	57416000	39617000	57953000	1.91E+08	2.62E+08	2.31E+08
tr|Q9RVW	tr|Q9RVW	1311						
tr|Q9RSD	tr|Q9RSD	697						
sp|Q9RYB	sp|Q9RYB	426	1.68E+09	8.65E+08	9.19E+08	3.35E+08	3.46E+08	3.13E+08
sp|Q9RS6	sp|Q9RS6	112	6.99E+08	6.4E+08	8.12E+08	5.48E+08	4.57E+08	6.12E+08
tr|Q9RVK	tr|Q9RVK	1248		28680000	22051000			
sp|Q9RR6	sp|Q9RR6	66	35135000	47781000	44972000		26617000	20600000
tr|Q9RRG	tr|Q9RRG	522			73181000	1.16E+08	1.12E+08	1.13E+08
tr|Q9RXG	tr|Q9RXG	1590	48985000	43919000	38605000	71250000	43712000	35275000
tr|Q9RS5	tr|Q9RS5	645				20342000		18989000
tr|Q9RVH	tr|Q9RVH	1234		20994000	14807000	28462000	25346000	25654000
tr|Q9RYS7	tr|Q9RYS7	1806		1.22E+08			68462000	
tr|Q9RTX	tr|Q9RTX	950	3.2E+08	3.09E+08	3.07E+08	2.92E+08	2.74E+08	2.56E+08
sp|Q9RT7	sp|Q9RT7	171						
tr|Q9RSY	tr|Q9RSY	784	2.04E+09	3.23E+09	2.54E+09	1.49E+09	1.49E+09	1.99E+09
sp|Q9RY2	sp|Q9RY2	411	1.63E+08		1.18E+08	4.34E+08	7.66E+08	5.09E+08
tr|Q9RSA	tr|Q9RSA	676	6271700	1417200	1171700	722420	1389600	970880
tr|Q9RXI1	tr|Q9RXI1	1601			5399800	10331000		
tr|Q9RWC	tr|Q9RWC	1390	25338000	33198000	28594000	40845000	39682000	30989000
sp|Q9RV9	sp|Q9RV9	272	29775000	92156000	69060000	67711000	1.09E+08	63870000
tr|Q9RUA	tr|Q9RUA	1023	1.08E+08	2.1E+08	1.47E+08	1.55E+08	1.88E+08	1.35E+08
tr|Q9RXV	tr|Q9RXV	1653	44355000	71143000	1.32E+08	62681000	59310000	1.01E+08
tr|Q9RUY	tr|Q9RUY	1139	23577000	32475000	20802000	52635000	37345000	47383000
tr|Q9RWN	tr|Q9RWN	1442	29612000	90849000	1.03E+08	1.37E+08	1.76E+08	1.44E+08
sp|Q9RUP	sp|Q9RUP	243	3.31E+08	2.89E+08	4.49E+08	1.25E+08	1.67E+08	1.26E+08
sp|Q9RUP	sp|Q9RUP	243		19454000	35577000			
tr|Q9RRS1	tr|Q9RRS1	573	16110000		37819000	86765000		
tr|Q9RUC	tr|Q9RUC	1033	61411000	76951000	75316000	47673000	52976000	46596000
tr|Q9RW7	tr|Q9RW7	1362				15191000	14968000	14639000
tr|Q9RVV	tr|Q9RVV	1309	1.27E+08	1.39E+08				
tr|Q9RTU	tr|Q9RTU	935	45881000	39702000	35041000	20604000	24504000	21258000

sp|Q9RRE	sp|Q9RRE	81			51210000			24103000
tr|Q9RZA1	tr|Q9RZA1	1888						
tr|Q9RWR	tr|Q9RWR	1460	42422000		22487000	1.61E+08	1.37E+08	85140000
sp|Q9RW	sp|Q9RW	328	1.5E+09	1.42E+09	1.27E+09	1.13E+09	1.2E+09	1.03E+09
tr|Q9RVM	tr|Q9RVM	1255	86305000	1.38E+08	89676000	1.29E+08	1.1E+08	1.05E+08
tr|Q9RXT	tr|Q9RXT	1642		31793000	23885000	11356000	13897000	
sp|Q4657	sp|Q4657	61		73888000	76233000		39508000	37785000
tr|Q9RUU	tr|Q9RUU	1121	2.92E+08	3.46E+08	3.03E+08		73055000	
tr|Q9RU4	tr|Q9RU4	990				44908000	27673000	7292300
sp|Q9RU7	sp|Q9RU7	215				21708000	20767000	27939000
sp|Q9RZ0	sp|Q9RZ0	451	1.15E+08	1.01E+08	90572000	48476000		69440000
tr|Q9RTM	tr|Q9RTM	899	3.49E+08	3.19E+08	4.26E+08	2.11E+08	2.63E+08	2.77E+08
tr|Q9RUK	tr|Q9RUK	1078						
sp|Q9RUF	sp|Q9RUF	231	67318000	73069000	94956000	1.61E+08	1.23E+08	1.44E+08
sp|Q9RW	sp|Q9RW	304						
tr|Q9RUC	tr|Q9RUC	1033	47193000	40195000	69278000			
tr|Q9RRJ7	tr|Q9RRJ7	537						5756800
tr|Q9RTK	tr|Q9RTK	888	6613900			15629000	14376000	9895400
tr|Q9RYY	tr|Q9RYY	1838				39733000	38709000	34777000
tr|Q9RVH	tr|Q9RVH	1235				32565000		24487000
tr|Q9RUG	tr|Q9RUG	1055	32964000	45290000	49418000			
tr|Q9RWN	tr|Q9RWN	1442			20417000	62771000	71583000	58139000
tr|Q9RU1	tr|Q9RU1	976	1.08E+08		98487000	51145000		48777000
tr|Q9RWK	tr|Q9RWK	1429	62565000	51305000	61265000	50834000	40553000	45962000
tr|Q9RTV1	tr|Q9RTV1	936						
sp|Q9RUL	sp|Q9RUL	239	1.65E+08	1.22E+08	58889000	19337000	87922000	25121000
tr|Q9RX4	tr|Q9RX4	1526		60946000		44310000		49650000
tr|Q9RUD	tr|Q9RUD	1042						
tr|Q9RVX	tr|Q9RVX	1317	53493000	1.32E+08	99373000	80814000	1.31E+08	93366000
sp|Q9RTN	sp|Q9RTN	196			9120100			
tr|Q9RZC	tr|Q9RZC	1904	1.84E+09	1.7E+09	93109000	1.75E+09	2.58E+09	1.89E+09
tr|Q9RST2	tr|Q9RST2	758	3.3E+08	2.86E+08	2.37E+08	1.48E+08	1.44E+08	1.59E+08
tr|Q9RXH	tr|Q9RXH	1599	1.6E+08	1.67E+08	1.53E+08	87221000	68084000	74452000
sp|Q9RZ0	sp|Q9RZ0	450				18030000	13025000	16418000
sp|Q9RZ0	sp|Q9RZ0	450			15393000			
tr|Q9RXP	tr|Q9RXP	1625	24528000	13743000	16323000	11984000	19979000	14863000
tr|Q9RUX	tr|Q9RUX	1133	24740000		35815000	1.69E+08	1.07E+08	66580000
sp|Q9RVH	sp|Q9RVH	288	9032100	3477400	2668900		1653100	1494600
sp|Q9RUF	sp|Q9RUF	230	1.65E+08	1.51E+08	1.5E+08	1.14E+08	83286000	1E+08
sp|Q9RXJ	sp|Q9RXJ	378	3.76E+09	2.89E+09	2.83E+09	1.38E+09	1.51E+09	1.77E+09
sp|Q9RW	sp|Q9RW	327	1.34E+08	1.03E+08	97331000	1.03E+08	1E+08	1.03E+08
tr|Q9RRA	tr|Q9RRA	483	2.72E+08	2.31E+08	2.69E+08	3.87E+08	3.49E+08	2.73E+08
sp|Q9RVP	sp|Q9RVP	294	11378000	4198500	12995000			
tr|Q9RYS1	tr|Q9RYS1	1801	19397000	6342800	38174000	17888000	14123000	
tr|Q9RYS1	tr|Q9RYS1	1801	8252500	24618000	49980000	15084000		18346000
tr|Q9RSM	tr|Q9RSM	737					16979000	
tr|Q9RWT	tr|Q9RWT	1465	1.34E+08	1.15E+08	1.34E+08	1.05E+08	84797000	1.09E+08
tr|Q9RS7	tr|Q9RS7	654	37248000					16117000
tr|Q9RUC	tr|Q9RUC	1034	73140000	1.29E+08	1.39E+08	24848000		
tr|Q9RU1	tr|Q9RU1	976	9.76E+08	9.31E+08	7.78E+08	1.51E+09	1.46E+09	1.43E+09
tr|Q9RU1	tr|Q9RU1	976	34012000	36079000	59242000	22836000	20157000	29536000

tr|Q9RSL7	tr|Q9RSL7	733	6.2E+08	8.81E+08	6E+08	47551000	1.26E+08	1.16E+08
sp|Q9RXJ	sp|Q9RXJ	375						
tr|Q9RR62	tr|Q9RR62	467		23955000				
tr|Q9RYW	tr|Q9RYW	1829						
tr|Q9RXW	tr|Q9RXW	1659	45251000	56695000	42999000	69881000	42058000	55795000
sp|Q9RX4	sp|Q9RX4	358	69248000	51792000	68555000	24556000	45766000	54466000
tr|Q9RU4	tr|Q9RU4	988	11673000	10079000	14452000	8836100	8941000	9406400
sp|Q9RV9	sp|Q9RV9	271	16242000		38726000	67908000		
tr|Q9RYS5	tr|Q9RYS5	1804		17834000	37000000	65445000	95267000	65677000
tr|Q9RVW	tr|Q9RVW	1313	5.8E+08	5.93E+08	5.15E+08	6.24E+08	4.24E+08	5.27E+08
tr|Q9RVW	tr|Q9RVW	1311					22518000	
tr|Q9RVU	tr|Q9RVU	1300		18081000				
tr|Q9RVU	tr|Q9RVU	1300	33350000	30456000	42816000	21180000	20042000	32944000
tr|Q9RRX1	tr|Q9RRX1	601	1.87E+09	2.84E+09	1.67E+09	4.82E+08	3.58E+08	5.7E+08
tr|Q9RYQ	tr|Q9RYQ	1794	12597000	13561000	11854000			
tr|Q9RWA	tr|Q9RWA	1379		10241000	32250000	24997000		29482000
tr|Q9RVU	tr|Q9RVU	1300	74384000	1.24E+08	1.26E+08	13040000		61010000
tr|Q9RXQ	tr|Q9RXQ	1628	5504100		14789000		30394000	37730000
sp|Q9RST	sp|Q9RST	151						
tr|Q9RVJ7	tr|Q9RVJ7	1242	1.14E+08	94683000	1.04E+08	95815000	87967000	1.04E+08
sp|Q9R34	sp|Q9R34	64		2.46E+08	4.07E+08	1.26E+09	1.51E+09	1.35E+09
tr|Q9RU4	tr|Q9RU4	990				1.87E+08	1.32E+08	1.91E+08
tr|Q9RYV6	tr|Q9RYV6	1823	1.73E+08	2.71E+08	2.22E+08			1.44E+08
sp|Q9RXC	sp|Q9RXC	367	23908000	49099000	56332000	90993000	68369000	1.69E+08
sp|Q9RXY	sp|Q9RXY	408	2.9E+08	5.43E+08	4.13E+08	2.09E+08	2.99E+08	2.19E+08
sp|P5692	sp|P5692	58	4.9E+08	4.72E+08	3.86E+08	3.16E+08	2.67E+08	3.54E+08
sp|Q9RW	sp|Q9RW	320			28284000			
tr|Q9RSP	tr|Q9RSP	746	95588000	1.86E+08	3.46E+08	2.38E+08	2.91E+08	2.53E+08
sp|Q9RVG	sp|Q9RVG	287				65620000	76154000	79797000
sp|Q9RXI	sp|Q9RXI	374				30579000		
tr|Q9RVP1	tr|Q9RVP1	1267		32318000	49054000	45627000	51856000	44859000
sp|Q9RXJ7	sp|Q9RXJ7	382	1.09E+09	7.7E+08	8.76E+08	1.48E+08	2.01E+08	1.65E+08
tr|Q9RYF4	tr|Q9RYF4	1743						
tr|Q9RUE	tr|Q9RUE	1046	1.55E+08	68835000	1.11E+08	31065000	36846000	46040000
tr|Q9RRB	tr|Q9RRB	496	1.83E+08	3.33E+08	3.1E+08	1.17E+09	7.69E+08	8.07E+08
tr|Q9RR62	tr|Q9RR62	467			22012000	41061000		29121000
tr|Q9RUK	tr|Q9RUK	1075	2.66E+08	1.84E+08	2.77E+08	1.37E+08	95765000	1.83E+08
tr|Q9RWH	tr|Q9RWH	1408	1.62E+09	3.36E+09	2.85E+09	1.1E+09	6.7E+08	8.32E+08
sp|Q9RSA	sp|Q9RSA	114						
tr|Q9RXW	tr|Q9RXW	1659						
sp|Q9RYD	sp|Q9RYD	431	32055000		20577000			
tr|Q9RZE8	tr|Q9RZE8	1917						
tr|Q9RU5	tr|Q9RU5	997	92670000	43696000	42178000	27841000	38103000	38569000
sp|Q9RS1	sp|Q9RS1	107	36529000	46274000	43750000	53816000	75480000	60327000
tr|Q9RZU	tr|Q9RZU	1979	95875000	1.07E+08	71768000	3.7E+08	4.25E+08	2.67E+08
tr|Q9RZU	tr|Q9RZU	1979					14175000	
tr|Q9RV41	tr|Q9RV41	1171	66561000	65525000	67118000	2.53E+08	2.04E+08	2.88E+08
tr|Q9RY22	tr|Q9RY22	1683			15571000		11341000	
sp|Q9RW	sp|Q9RW	326	25613000	18455000	32762000	68551000	21582000	1.16E+08
tr|Q9RZD	tr|Q9RZD	1907						
tr|Q9RS5	tr|Q9RS5	648	29427000	31248000	29514000	21080000	17815000	17960000

tr|Q9RVE5	tr|Q9RVE5	1219	30095000		9275800	20699000		16959000
tr|Q9RYD7	tr|Q9RYD7	1734	91778000	1.27E+08	1.25E+08	3.36E+08	4.69E+08	3.67E+08
tr|Q9RY67	tr|Q9RY67	1701	1.77E+09	1.48E+09	1.35E+09	3.95E+09	2.77E+09	3E+09
tr|Q9RY67	tr|Q9RY67	1701	51369000	44000000	78744000	55394000	65968000	71734000
tr|Q9RY67	tr|Q9RY67	1701	1.78E+08	2E+08	2.88E+08	54385000	1.28E+08	89825000
tr|Q9RYQ	tr|Q9RYQ	1793						
tr|Q9RYQ	tr|Q9RYQ	1793	21192000		49676000	22568000	32072000	24596000
tr|Q9RRE6	tr|Q9RRE6	512	9648200	7598600	9936100		15754000	15981000
tr|Q9RXS7	tr|Q9RXS7	1640	1.7E+09	1.68E+09	1.59E+09	9.92E+08	7.3E+08	9.29E+08
sp|Q9RWE	sp|Q9RWE	320	3.47E+08	3.46E+08	3.57E+08	3.5E+08	3.65E+08	4.78E+08
sp|Q9RX1	sp|Q9RX1	351	2.58E+08	2.85E+08	1.88E+08	1.76E+08	1.92E+08	1.91E+08
sp|Q9RYD	sp|Q9RYD	429	21835000					
tr|Q9RZT2	tr|Q9RZT2	1969	38340000	26491000	22520000	34336000	28438000	25601000
tr|Q9RTR8	tr|Q9RTR8	925	27421000		27735000			
tr|Q9RWH	tr|Q9RWH	1411	17799000	10532000	13514000	37076000	23994000	
sp|Q9RSW	sp|Q9RSW	153				7199700	4466000	5049300
tr|Q9RX65	tr|Q9RX65	1538	63020000	64915000	79735000	2.2E+08	1.99E+08	1.68E+08
tr|Q9RTI4	tr|Q9RTI4	880	4.91E+08	5.6E+08	5.14E+08	6.96E+08	8.27E+08	5.82E+08
tr|Q9RTI4	tr|Q9RTI4	880			28058000			
tr|Q9RZF1	tr|Q9RZF1	1919		19082000			22560000	13169000
sp|Q9RXU	sp|Q9RXU	402						
tr|Q9RWN	tr|Q9RWN	1442	6080400			27310000	30250000	29741000
tr|Q9RUR9	tr|Q9RUR9	1110	87943000	46221000	47463000			
sp|Q9RU2	sp|Q9RU2	209	22431000	1.84E+08	5.14E+08	8.05E+08	1.46E+09	9.24E+08
sp|Q9RU2	sp|Q9RU2	209				8607800		12520000
tr|Q9RYQ0	tr|Q9RYQ0	1788		33764000	1.95E+08	1.72E+08	2.25E+08	2.52E+08
tr|Q9RVW	tr|Q9RVW	1311			16958000			
sp|Q9RSQ	sp|Q9RSQ	141	38671000	46378000	37126000			19665000
sp|Q9RX0	sp|Q9RX0	350			17650000	35903000	46928000	38886000
sp|Q9RY6	sp|Q9RY6	421	39103000	38441000	26177000	82436000	45188000	62800000
sp|Q9RSL4	sp|Q9RSL4	136	2.99E+08	1.83E+08	2.85E+08	1.17E+08	1.23E+08	1.19E+08
tr|Q9RRV2	tr|Q9RRV2	589	2.97E+08	1.59E+08		3.44E+08	3.19E+08	3.72E+08
tr|Q9RT01	tr|Q9RT01	792			18202000			12384000
tr|Q9RXZ9	tr|Q9RXZ9	1672			48626000			
tr|Q9RT51	tr|Q9RT51	820	4366600	15676000	9752000	42923000	38912000	51434000
tr|Q9RSK2	tr|Q9RSK2	730			34552000			
tr|Q9RRM	tr|Q9RRM	548						
tr|Q9RTY1	tr|Q9RTY1	956	59285000		62467000			
tr|Q9RZD6	tr|Q9RZD6	1910				58498000	34769000	54221000
tr|Q9RVB8	tr|Q9RVB8	1210	12011000	14229000	10729000			
tr|Q9RWB	tr|Q9RWB	1386				48124000	41173000	
tr|Q9RWB	tr|Q9RWB	1386	22855000		44025000	44705000		
tr|Q9RVX2	tr|Q9RVX2	1316					12341000	
tr|Q9RYC4	tr|Q9RYC4	1725				5822100		4584700
tr|Q9RZV3	tr|Q9RZV3	1983	32282000	27306000	20410000	39437000	46980000	68474000
tr|Q9RZC5	tr|Q9RZC5	1904	3.2E+09	2.52E+09	2.51E+09	3.37E+09	2.27E+09	3.33E+09
sp|Q9RSS	sp|Q9RSS	149	77601000	64259000	59729000	33162000	42600000	45407000
tr|Q9RR82	tr|Q9RR82	474	2.51E+08	1.97E+08	1.79E+08	4.21E+08	3.88E+08	4.17E+08
tr|Q9RR82	tr|Q9RR82	474	3.13E+08	3.34E+08	2.65E+08	47621000	43715000	1.26E+08
tr|Q9RW8	tr|Q9RW8	1367		9110600			8969500	7163800
tr|Q9RRR0	tr|Q9RRR0	567	14119000	19752000				

tr|Q9RWN	tr|Q9RWN	1444	28449000		21547000			
tr|Q9RVQ	tr|Q9RVQ	1278				25005000		23883000
tr|Q9RZB5	tr|Q9RZB5	1898						
sp|Q9RUL	sp|Q9RUL	239	21510000	20903000	20456000		20338000	
sp|Q9RVW	sp|Q9RVW	301			16789000			
tr|Q9RRS7	tr|Q9RRS7	578	19332000	20244000	15704000			
tr|Q9RYQ4	tr|Q9RYQ4	1792	3.05E+08	2.6E+08	2.56E+08	1.27E+09	1.31E+09	1.06E+09
tr|Q9RTE3	tr|Q9RTE3	862	36607000	38928000	33094000	77957000		
sp|Q9RR7	sp|Q9RR7	68	13958000	17000000	15394000		11274000	10757000
tr|Q9RYR1	tr|Q9RYR1	1796	1.76E+08	3.06E+08	1.5E+08	85231000	84185000	94239000
tr|Q9RXV9	tr|Q9RXV9	1654						
sp|Q9RVI3	sp|Q9RVI3	289	98315000		62094000		93506000	
tr|Q9RYS1	tr|Q9RYS1	1801	5.82E+08	6.09E+08	5.29E+08	5.44E+08	5.29E+08	6.06E+08
tr|Q9RUQ	tr|Q9RUQ	1101	49062000	33958000	33369000			
tr|Q9RT09	tr|Q9RT09	798	4.57E+08	4.86E+08	7.46E+08	7.95E+08	6.82E+08	9.03E+08
tr|Q9RTX0	tr|Q9RTX0	947						
tr|Q9RUQ	tr|Q9RUQ	1101	99850000	81917000	78793000			
tr|Q9RVS0	tr|Q9RVS0	1286	1.34E+08	1.02E+08	1.94E+08			
sp|Q9RSP	sp|Q9RSP	139						
tr|Q9RVS2	tr|Q9RVS2	1288	1.25E+09	1.34E+09	1.23E+09	1.33E+09	1.06E+09	1.28E+09
tr|Q9RYE5	tr|Q9RYE5	1739	3224000		5395500	22042000	33498000	
tr|Q9RRI9	tr|Q9RRI9	535	24913000	19933000	28803000	16980000	14987000	29484000
tr|Q9RV59	tr|Q9RV59	1182	1.52E+08	1.68E+08	1.56E+08	3.85E+08	3.81E+08	4.08E+08
tr|Q9RV59	tr|Q9RV59	1182					99589000	
tr|Q9RZV9	tr|Q9RZV9	1985	2.79E+08	2.22E+08	2.13E+08	3.19E+08	3.19E+08	3.15E+08
tr|Q9RRD6	tr|Q9RRD6	506	40681000	96205000	34687000	69164000	42230000	51062000
tr|Q9RU78	tr|Q9RU78	1014						
tr|Q9RS92	tr|Q9RS92	667						
tr|Q9RUF8	tr|Q9RUF8	1049	4.18E+08	4.07E+08	3.57E+08	7.35E+08	5.02E+08	5.65E+08
tr|Q9RUF8	tr|Q9RUF8	1049			1.44E+08			
tr|Q9RYQ8	tr|Q9RYQ8	1794						
tr|Q9RXE9	tr|Q9RXE9	1580					17461000	
tr|Q9RSC6	tr|Q9RSC6	689	2E+08	1.58E+08	2.13E+08	68207000	78753000	68953000
tr|Q9RSL8	tr|Q9RSL8	734	4.98E+08	4.64E+08	5.01E+08	1.85E+08	2.43E+08	2.28E+08
tr|Q9RU57	tr|Q9RU57	1000	6.19E+08	2.8E+08	3.16E+08	20690000	1.52E+08	1.18E+08
tr|Q9RYL0	tr|Q9RYL0	1767	3.9E+08	4.56E+08	4.11E+08	4.22E+08	3.41E+08	2.55E+08
sp|Q9RY4	sp|Q9RY4	414	40999000	39167000	33184000	28881000	34761000	30470000
tr|Q9RYQ0	tr|Q9RYQ0	1788	5078800			1402000	1679000	1547500
sp|O32504	sp|O32504	44						
sp|O32504	sp|O32504	44	21924000	1.58E+08	63336000	33887000	27684000	55765000
sp|Q9RRD	sp|Q9RRD	80					34300000	
tr|Q9RS93	tr|Q9RS93	668	33508000		68806000	66778000		
sp|Q9RRJ1	sp|Q9RRJ1	86	2.34E+08	1.07E+08	1.57E+08	12795000	23351000	20828000
tr|Q9RXE9	tr|Q9RXE9	1580		2769800			2180700	
tr|Q9RT11	tr|Q9RT11	799	36407000		74077000			45491000
tr|Q9RSP7	tr|Q9RSP7	744	33419000				21853000	
sp|Q9RUY	sp|Q9RUY	259			11991000			
tr|Q9RZ81	tr|Q9RZ81	1875	15127000	17314000	17324000	29846000	26319000	21997000
tr|Q9RU57	tr|Q9RU57	1000						
sp|Q9RX5	sp|Q9RX5	360	21184000	17892000		15862000		
tr|Q9RX44	tr|Q9RX44	1524		13421000	17678000			

tr|Q9RRP8	tr|Q9RRP8	560	11561000	10272000	8498400			
tr|Q9RRP8	tr|Q9RRP8	560						
tr|Q9RT06	tr|Q9RT06795;1543		75906000	78384000	84446000	29949000	35513000	42462000
tr|Q9RZQ	tr|Q9RZQ	1957	1.28E+08	2.29E+08	2.24E+08	1.89E+08	1.97E+08	1.36E+08
tr|Q9RUB	tr|Q9RUB	1028	1.77E+09	3.01E+09	2.03E+09	1.72E+09	1.45E+09	2.48E+09
tr|Q9RUB	tr|Q9RUB	1028						
tr|Q9RUB	tr|Q9RUB	1028				28923000	22370000	20920000
tr|Q9RY67	tr|Q9RY67	1701	1.11E+09	7.91E+08	7.09E+08	1.79E+09	1.18E+09	1.35E+09
tr|Q9RY67	tr|Q9RY67	1701						
tr|Q9RXD7	tr|Q9RXD7	1574	1.96E+08	2.23E+08	1.45E+08	3.18E+08	2.64E+08	3.23E+08
tr|Q9RXD7	tr|Q9RXD7	1574	28326000	32485000	51316000	23343000	6443500	47433000
tr|Q9RV52	tr|Q9RV52	1177				1.45E+08	1.53E+08	1.6E+08
tr|Q9RRS3	tr|Q9RRS3	574	92595000	85350000	1.11E+08	1.24E+08	71533000	95752000
tr|Q9RYN0	tr|Q9RYN0	1780	7.95E+08	6.56E+08	8.54E+08	1.16E+09	9.9E+08	1.05E+09
tr|Q9RVF4	tr|Q9RVF4	1223						
tr|Q9RXH8	tr|Q9RXH8	1599	2.14E+08	1.74E+08	1.34E+08	45983000	93563000	47049000
tr|Q9RU54	tr|Q9RU54	997						
tr|Q9RZ08	tr|Q9RZ08	1846	32645000	35629000	41785000			
tr|Q9RX24	tr|Q9RX24	1515	61794000	85361000	82757000	53002000	55746000	59743000
sp|Q9RTA	sp|Q9RTA	174			13920000		12043000	8350300
tr|Q9RUB	tr|Q9RUB	1028	4.14E+08	4.45E+08	4.59E+08	7.47E+08	6.35E+08	5.57E+08
tr|Q9RXU0	tr|Q9RXU0	1643						
tr|Q9RZ87	tr|Q9RZ87	1879	27700000	30631000				22134000
tr|Q9RXC3	tr|Q9RXC3	1569	57068000	1.1E+08	85287000	1.17E+08	1.19E+08	1.44E+08
tr|Q9RXC3	tr|Q9RXC3	1569	18683000	63886000	64660000	20756000	34911000	28655000
tr|Q9RT87	tr|Q9RT87	837			7460600			
tr|Q9RXG0	tr|Q9RXG0	1587			7238300		10236000	
tr|Q9RSF8	tr|Q9RSF8	710			19908000	44556000	83700000	39481000
tr|Q9RRD0	tr|Q9RRD0	506					20031000	
tr|Q9RSY7	tr|Q9RSY7	785			10985000			
tr|Q9RZC5	tr|Q9RZC5	1904	1.07E+08	1.65E+08	1.6E+08	3.32E+08	3.86E+08	2.88E+08
tr|Q9RVP1	tr|Q9RVP1	1267			18126000			
tr|Q9RVV0	tr|Q9RVV0	1304		47766000	43905000			44732000
tr|Q9RVV0	tr|Q9RVV0	1304		29375000	48254000	17288000	28582000	21711000
tr|Q9RTJ4	tr|Q9RTJ4	884			21027000			
tr|Q9RYI7	tr|Q9RYI7	1763	15112000	17677000	14952000	54275000	36820000	41409000
tr|Q9RUB	tr|Q9RUB	1028	3.14E+08	2.5E+08	2.49E+08	3.25E+08	2.12E+08	2.63E+08
sp|Q9RXK	sp|Q9RXK	389						
sp|Q9RVY	sp|Q9RVY	303	4.19E+08	5.1E+08	3.96E+08	6.79E+08	6.03E+08	6.81E+08
sp|Q9RY0	sp|Q9RY0	410				8640500		14352000
tr|Q9RTQ1	tr|Q9RTQ1	917	34642000		40651000	78266000	79235000	
tr|Q9RTQ1	tr|Q9RTQ1	917	62651000	63935000	61352000	1.74E+08	2.96E+08	1.41E+08
tr|Q9RVY2	tr|Q9RVY2	1320	22008000	94906000	1.2E+08	84855000	73684000	61275000
sp|Q9RTG	sp|Q9RTG	184	2.72E+08	2.55E+08	2.54E+08	6.89E+08	6.18E+08	6.07E+08
sp|Q9RTG	sp|Q9RTG	184						
tr|Q9RUS6	tr|Q9RUS6	1113	1.19E+08	1.26E+08	1.19E+08	37545000	35967000	82164000
sp|Q9RUR	sp|Q9RUR	249	1776100		569100			
sp|Q9RUE	sp|Q9RUE	225	43983000	70592000				21298000
tr|Q9RUS4	tr|Q9RUS4	1112				33606000	36200000	41717000
sp|Q9RW\	sp|Q9RW\	348						
sp|Q9RSZ	sp|Q9RSZ	160	9214600	5259600	4932500			

tr|Q9RTZ7	tr|Q9RTZ7	965	17473000	22599000	19616000	60387000	53288000	42533000
sp|Q9RUP	sp|Q9RUP	246	86720000	39498000	25250000	1.02E+08	87838000	84229000
sp|Q9RSF	sp|Q9RSF	117			23006000	16796000	15042000	19044000
tr|Q9RVG	tr|Q9RVG	1229	2.44E+08	2.77E+08	2.41E+08	4.95E+08	3.83E+08	3.68E+08
tr|Q9RVG	tr|Q9RVG	1229			13803000			
tr|Q7DF71	tr|Q7DF71	464	59072000					
tr|Q9RZ10	tr|Q9RZ10	1848	7446400		18582000			
sp|Q9RXJ8	sp|Q9RXJ8	383						
tr|Q9RU43	tr|Q9RU43	990						
tr|Q9RRL1	tr|Q9RRL1	545					6314100	
tr|Q9RTH3	tr|Q9RTH3	876						
tr|Q9RSP8	tr|Q9RSP8	745	66542000	1.11E+08	75615000	2.93E+08	2.56E+08	2.7E+08
tr|Q9RU54	tr|Q9RU54	997	1.2E+09	1.45E+09	1.27E+09	2.48E+09	2.37E+09	2.45E+09
sp|Q5933	sp|Q5933	63	5.61E+08	5.35E+08	6.35E+08	1.62E+08	2.22E+08	1.51E+08
tr|Q9RUD	tr|Q9RUD	1043	28525000	38537000	35926000	35778000	25614000	28461000
tr|Q9RW7	tr|Q9RW7	1362						
sp|P56867	sp|P56867	56				20319000		
sp|Q9RSS	sp|Q9RSS	150	35472000	32639000		60105000	30305000	
tr|Q9RS71	tr|Q9RS71	655	7847800					
tr|Q9RYQ0	tr|Q9RYQ0	1788	27882000	41166000	41506000	66405000	62252000	41514000
tr|Q9RTZ1	tr|Q9RTZ1	962				57596000	57557000	57114000
tr|Q9RUD	tr|Q9RUD	1039			55811000			
sp|Q9RWH	sp|Q9RWH	331	91974000	95848000	70438000			
tr|Q9RWP	tr|Q9RWP	1449	21202000	10241000	17382000	53655000	43716000	52170000
tr|Q9RS03	tr|Q9RS03	620	34005000	39248000	30833000	19199000		24086000
sp|Q9RUA	sp|Q9RUA	224	85652000	48742000	61300000			
tr|Q9RTZ8	tr|Q9RTZ8	966	7.91E+08	8.57E+08	6.69E+08	6.65E+08	5.93E+08	3.01E+08
tr|Q9RXM	tr|Q9RXM	1615		11798000	21631000			
tr|Q9RZ85	tr|Q9RZ85	1877	15418000	18907000	11601000			
tr|Q9RYN3	tr|Q9RYN3	1777						
sp|Q9RRG	sp|Q9RRG	84	4.68E+08	4.97E+08	5.49E+08	2.98E+08	3.77E+08	3.2E+08
sp|Q9RW9	sp|Q9RW9	318			10067000			12660000
sp|Q9RXX	sp|Q9RXX	405	1.55E+08	1.95E+08	1.44E+08	1.43E+08	80018000	99870000
tr|Q9RY25	tr|Q9RY25	1684		21383000	14795000			
tr|Q9RVB4	tr|Q9RVB4	1208	1.58E+08	42475000	99817000	1.86E+08	1.76E+08	2.19E+08
tr|Q9RUM	tr|Q9RUM	1089	12643000		1.33E+08			
sp|Q9RTG	sp|Q9RTG	184	30647000	36131000		70506000	46818000	60591000
tr|Q9RTJ8	tr|Q9RTJ8	886	18452000	25336000	24880000			
tr|Q9RW7	tr|Q9RW7	1364	3.6E+08	3.09E+08	3.47E+08	1.3E+08	1.59E+08	2.07E+08
tr|Q9RWV	tr|Q9RWV	1484				18795000	1.04E+08	60673000
tr|Q9RSF8	tr|Q9RSF8	710	63814000	74007000	51017000	1.46E+08	1.22E+08	1.04E+08
sp|Q9RVW	sp|Q9RVW	301	1.1E+08	1.11E+08	1.18E+08	2.5E+08	2.21E+08	2.84E+08
tr|Q9RW2	tr|Q9RW2	1342	1.81E+08	1.57E+08		1.99E+08	1.94E+08	1.66E+08
sp|Q9RW0	sp|Q9RW0	326				1.25E+08	57821000	1.09E+08
tr|Q9RT51	tr|Q9RT51	820						
tr|Q9RTU1	tr|Q9RTU1	933						
tr|Q9RSK5	tr|Q9RSK5	731						
tr|Q9RXF0	tr|Q9RXF0	1581	1.9E+08	2.15E+08	1.38E+09	1.09E+09	9.84E+08	1.49E+09
tr|Q9RU25	tr|Q9RU25	978	56210000	39474000	39482000	34288000		
tr|Q9RU17	tr|Q9RU17	976	21859000	22713000	60853000	12432000	27179000	22883000
tr|Q9RTC6	tr|Q9RTC6	852	32246000	92936000	1.26E+08	1.11E+08	1.34E+08	70800000

tr|Q9RV17	tr|Q9RV17	1160	22943000	6638400		39071000	26596000	
tr|Q9RSE1	tr|Q9RSE1	700	1.18E+08	80089000	97862000	1.05E+08	1.19E+08	81559000
tr|Q9RSW	tr|Q9RSW	775	6.26E+08	6.75E+08	6.08E+08	1.06E+09	8.06E+08	9.51E+08
tr|Q9RSW	tr|Q9RSW	775			37047000			
sp|Q9RV7	sp|Q9RV7	268	15950000		15101000			
tr|Q9RUX8	tr|Q9RUX8	1136	3.08E+08	3.12E+08	2.66E+08			
tr|Q9RS10	tr|Q9RS10	626						
tr|Q9RS10	tr|Q9RS10	626						
tr|Q9RXQ	tr|Q9RXQ	1629						
tr|Q9RXQ	tr|Q9RXQ	1629	1.26E+08	1.18E+08	1.09E+08	31087000		
sp|Q9RSL	sp|Q9RSL	134				47553000	43397000	66357000
sp|Q9RZA	sp|Q9RZA	454			7358400	20882000		10392000
tr|Q9RYA0	tr|Q9RYA0	1718	39137000	36200000	43961000		10513000	
tr|Q9RYW	tr|Q9RYW	1827			7092700	5420700	3466100	3463400
sp|Q9RX1	sp|Q9RX1	351	17314000	31987000				
tr|Q9RY79	tr|Q9RY79	1708	4.71E+08	5.82E+08	6E+08	8.29E+08	7.48E+08	6.87E+08
tr|Q9RYM	tr|Q9RYM	1773	30993000	23670000	21466000	1.15E+08	85913000	96488000
tr|Q9RYM	tr|Q9RYM	1773					30915000	54208000
sp|Q9RYG	sp|Q9RYG	434				19393000	10853000	17866000
tr|Q9RU0	tr|Q9RU0	969	52778000	75337000	1.07E+08	38349000	65050000	54258000
tr|Q9RTZ9	tr|Q9RTZ9	967					5753100	
tr|Q9RRY8	tr|Q9RRY8	610		3319300		5618400	10824000	7924400
tr|Q9RRU	tr|Q9RRU	584						
tr|Q9RUE4	tr|Q9RUE4	1045		25107000	23943000	34623000	27206000	39527000
tr|Q7DF71	tr|Q7DF71	464						
tr|Q9RRY2	tr|Q9RRY2	605	6116800		11926000	61461000	56292000	42101000
tr|Q9RZQ	tr|Q9RZQ	1959	1.07E+08	1E+08	1.16E+08	94591000	84115000	1.1E+08
tr|Q9RT14	tr|Q9RT14	800				42335000	33416000	38384000
tr|Q9RRW	tr|Q9RRW	595	1.28E+08	85476000	1.07E+08	1.14E+08	80900000	1.25E+08
sp|Q9RU2	sp|Q9RU2	209	12420000	6187200	4900800	4842400	7081900	5140300
sp|Q9RWI	sp|Q9RWI	336				15574000	26284000	29587000
tr|Q9RZD4	tr|Q9RZD4	1909	22543000			32834000	29415000	30034000
tr|Q9RS25	tr|Q9RS25	633	63241000	1.03E+08	93515000	1.17E+08	1.53E+08	1.25E+08
tr|Q9RTZ9	tr|Q9RTZ9	967	14826000		17125000	24749000		23783000
tr|Q9RYN3	tr|Q9RYN3	1777	24751000		30028000		36279000	25660000
tr|Q9RXX9	tr|Q9RXX9	1663	34067000	42260000	52746000			
tr|Q9RVS1	tr|Q9RVS1	1287	64437000					
tr|Q9RRC5	tr|Q9RRC5	500						
tr|Q9RT97	tr|Q9RT97	843	1.25E+08	1.19E+08	1.1E+08	1.78E+08	1.32E+08	1.82E+08
sp|Q9RUP	sp|Q9RUP	244						
sp|Q9RXK	sp|Q9RXK	389		2.9E+08	2.4E+08	4.53E+08	5.41E+08	4.17E+08
tr|Q9RYF4	tr|Q9RYF4	1743		44116000	38536000			
tr|Q9RWQ	tr|Q9RWQ	1454	1.82E+08	1.59E+08	1.52E+08	1.08E+08	1.13E+08	1.22E+08
sp|Q9RXT	sp|Q9RXT	400	6248800	10043000	10158000	9531300	13195000	13194000
tr|Q9RXQ	tr|Q9RXQ	1627	60514000	50771000	2.69E+08	2.12E+08	2.28E+08	2.97E+08
sp|Q9RVW	sp|Q9RVW	301			278160	392590	456390	511420
tr|Q9RTU	tr|Q9RTU	933	40607000	49808000	37668000		12220000	
tr|Q9RRJ0	tr|Q9RRJ0	536						
tr|Q9RWV	tr|Q9RWV	1486	5161400	2535700	2978600			
tr|Q9RUR	tr|Q9RUR	1108						
sp|Q9RXG	sp|Q9RXG	370						

tr|Q9RUB	tr|Q9RUB	1031	82741000	1.06E+08	68336000	45637000		35040000
tr|Q9RTZ3	tr|Q9RTZ3	963	1.89E+08	47134000	15544000	54738000	58436000	42507000
tr|Q9RWH	tr|Q9RWH	1412					22394000	22624000
tr|Q9RT87	tr|Q9RT87	837	37928000	49782000	34948000	68936000	55961000	75795000
tr|Q9RVN	tr|Q9RVN	1262	94628000	99109000	1.05E+08	1.14E+08	1.06E+08	1.97E+08
sp|Q9RS6	sp|Q9RS6	112	1.06E+08	1.11E+08	66538000	26907000		
tr|Q9RY13	tr|Q9RY13	1679	37443000					
tr|Q9RWB	tr|Q9RWB	1383	48633000	60827000	66213000	66089000	42259000	24165000
sp|Q9RSL	sp|Q9RSL	133	15585000	8305800	17941000	13212000	18412000	13221000
tr|Q9RZE3	tr|Q9RZE3	1913	1069200					
tr|Q9RUI4	tr|Q9RUI4	1063						
sp|Q9RTN	sp|Q9RTN	196	7.9E+08	6.42E+08	7.62E+08	1.61E+08	2.09E+08	2.82E+08
tr|Q9RYA6	tr|Q9RYA6	1720	4033000		8086100	15015000		24807000
tr|Q9RYA6	tr|Q9RYA6	1720						
tr|Q9RZ32	tr|Q9RZ32	1860	49149000	52208000	74939000	30838000		33045000
tr|Q9RWU	tr|Q9RWU	1476					32794000	
tr|Q7DF71	tr|Q7DF71	464		19138000	15930000			23401000
sp|Q9RXK	sp|Q9RXK	389					17428000	17811000
sp|Q9RXK	sp|Q9RXK	389					15745000	
tr|Q9RRK9	tr|Q9RRK9	543						
sp|Q9RU2	sp|Q9RU2	209	4.45E+08	1.63E+08	2.34E+08	4.72E+08	3.93E+08	4.32E+08
sp|Q9RW	sp|Q9RW	342	2.95E+08	2.04E+08	2.04E+08	1.9E+08	1.71E+08	1.56E+08
sp|Q9RW	sp|Q9RW	342	2.07E+08	3.55E+08	1.19E+09	4.23E+08	6.14E+08	6.4E+08
tr|Q9RV05	tr|Q9RV05	1153						
tr|Q9RS49	tr|Q9RS49	644	13352000	18009000	22890000	28550000	24512000	
tr|Q9RTX7	tr|Q9RTX7	952	2.38E+09	2.05E+09	1.81E+09	1.97E+09	1.12E+09	1.58E+09
sp|Q9RUN	sp|Q9RUN	241	98478000	43136000	54302000	18240000		
sp|Q9RUN	sp|Q9RUN	241						
tr|Q9RU59	tr|Q9RU59	1002				13809000	20810000	13632000
tr|Q9RXI6	tr|Q9RXI6	1605						
tr|Q9RTZ9	tr|Q9RTZ9	967	2.3E+08	3.74E+08	2.88E+08	1.35E+08	1.82E+08	1.8E+08
sp|Q9RSJ8	sp|Q9RSJ8	126			32005000			
sp|Q9RSJ8	sp|Q9RSJ8	126						
tr|Q9RUB	tr|Q9RUB	1028	5.73E+09	5.49E+09	4.93E+09	6.33E+09	5.19E+09	6.56E+09
tr|Q9RWX	tr|Q9RWX	1497						
sp|Q9RSS	sp|Q9RSS	147	4.48E+08	7.17E+08	5.89E+08			66350000
tr|Q9RX16	tr|Q9RX16	1511		894270	572640			536600
tr|Q9RZD	tr|Q9RZD	1910	1.85E+08	2.21E+08	1.77E+08	1.67E+08	1.36E+08	
tr|Q9RVX7	tr|Q9RVX7	1318					769800	686510
tr|Q9RYQ8	tr|Q9RYQ8	1794						
tr|Q9RU9	tr|Q9RU9	1015			1.82E+08	94861000	96973000	1.66E+08
tr|Q9RUA	tr|Q9RUA	1023			13439000	4630700		6006400
sp|Q9RSS	sp|Q9RSS	149						
sp|Q9R34	sp|Q9R34	64			634630			
tr|Q9RSA9	tr|Q9RSA9	680	1.87E+08	1.99E+08	1.64E+08	1.07E+08		78031000
tr|Q9RUE	tr|Q9RUE	1044	2175900			2125800	2966900	2347200
tr|Q9RUE	tr|Q9RUE	1044	2.13E+08	1.81E+08	2.1E+08	2.01E+08	1.72E+08	2.01E+08
sp|Q9R34	sp|Q9R34	64						
tr|Q9RXA4	tr|Q9RXA4	1558	64373000	46354000	65053000	14875000	19710000	19758000
sp|P56867	sp|P56867	56			50503000	54442000	66470000	71250000
tr|Q9RZQ	tr|Q9RZQ	1959				18494000	30197000	28946000

sp|P5202	sp|P5202	51	5559300	8983300		17220000	22563000	12753000
sp|Q9RU	sp|Q9RU	258		2761500				
sp|Q9RRB	sp|Q9RRB	76	65021000					
sp|Q9RRB	sp|Q9RRB	76						17670000
sp|Q9RRB	sp|Q9RRB	76	28648000	79341000	1.44E+08	2.07E+08	2.08E+08	1.85E+08
sp|Q9RRB	sp|Q9RRB	76			22461000			
tr|Q9RUL	tr|Q9RUL	1082	28599000	31277000		1.16E+08	98019000	93203000
tr|Q9RUL	tr|Q9RUL	1082	40903000				19554000	
sp|Q9RW	sp|Q9RW	309	58630000	33745000	39811000	52853000	55245000	46516000
sp|Q9RR	sp|Q9RR	94	34770000	49189000		36484000	30041000	37299000
sp|Q9RSQ	sp|Q9RSQ	141		44238000	39230000	56661000	77333000	51877000
tr|Q9RZE	tr|Q9RZE	1916	73882000	52495000		19524000		17074000
tr|Q9RW9	tr|Q9RW9	1373			3152300	2331500	3474900	2558000
tr|Q9RY9	tr|Q9RY9	1717						
tr|Q9RX1	tr|Q9RX1	1514	33190000	46846000		1.04E+08	80060000	1.12E+08
tr|Q9RX1	tr|Q9RX1	1514				16206000	23982000	28957000
tr|Q9RU4	tr|Q9RU4	990	57351000	76305000	1.5E+08	1.24E+08	1.18E+08	93031000
tr|Q9RRS	tr|Q9RRS	576			8358300			
sp|Q9RU2	sp|Q9RU2	208						
sp|Q9RRP	sp|Q9RRP	93	62439000	50184000	50354000	24075000		
tr|Q9RX5	tr|Q9RX5	1532						
tr|Q9RVQ	tr|Q9RVQ	1271	1.12E+09	1.08E+09	1.09E+09	3.79E+08	2.24E+08	4.01E+08
tr|Q9RWL	tr|Q9RWL	1434						
sp|Q9RRX	sp|Q9RRX	101	3.91E+08	4.11E+08	3.75E+08		6.22E+08	
sp|Q9RTF	sp|Q9RTF	182				54308000	39703000	
tr|Q9RWC	tr|Q9RWC	1388	34133000	16486000	18271000		6070000	
sp|Q9RXK	sp|Q9RXK	389	57674000	2.28E+08	2.06E+08	3.35E+08	4.42E+08	1.52E+08
sp|Q9RU8	sp|Q9RU8	222				1.33E+08	1.03E+08	1.07E+08
tr|Q9RWL	tr|Q9RWL	1435		39510000			20868000	
tr|Q9RSU	tr|Q9RSU	767	87143000	67375000	97510000	3.2E+08	2.64E+08	2.27E+08
tr|Q9RYE4	tr|Q9RYE4	1738		33345000	49248000			
tr|Q9RSM	tr|Q9RSM	735	5.89E+08	8.13E+08	6.8E+08	3.53E+08	4.32E+08	4.3E+08
sp|Q9RR8	sp|Q9RR8	70	38777000	65496000	51350000	61002000		51288000
sp|Q9RW	sp|Q9RW	326	9.39E+09	8.79E+09	7.48E+09	8.45E+09	1E+10	8.17E+09
tr|Q9RZC	tr|Q9RZC	1904			1.35E+08	1.15E+08	1.2E+08	1.49E+08
tr|Q9RVP	tr|Q9RVP	1269		78449000	79840000			
tr|Q9RRZ	tr|Q9RRZ	611						56883000
tr|Q9RRW	tr|Q9RRW	598						
tr|Q9RST1	tr|Q9RST1	757				30686000		
tr|Q9RVR	tr|Q9RVR	1281	1.07E+08	1.61E+08	1.7E+08	2.39E+08	1.64E+08	2.69E+08
tr|Q9RVR	tr|Q9RVR	1281		29487000	83382000	56649000	69505000	66198000
tr|Q9RWC	tr|Q9RWC	1389	3274700					
sp|Q9RR	sp|Q9RR	95	9.71E+08	7.64E+08	7.54E+08	1.04E+09	8.98E+08	1.09E+09
tr|Q9RVL	tr|Q9RVL	1253						
sp|Q9RSP	sp|Q9RSP	139			15014000			
tr|Q9RVQ	tr|Q9RVQ	1273	69830000		43298000	8507200	34642000	26048000
sp|Q5933	sp|Q5933	63						
tr|Q9RVA	tr|Q9RVA	1202	7.08E+08	5.8E+08	5.08E+08	1.79E+08	1.61E+08	2.56E+08
tr|Q9RVA	tr|Q9RVA	1202			17293000			
tr|Q9RR8	tr|Q9RR8	475	51777000	76147000	95381000	35837000	35866000	57104000
sp|Q9RU8	sp|Q9RU8	222	50593000	30801000	42379000	1.52E+08	1.29E+08	1.08E+08

tr|Q9RWC	tr|Q9RWC	1392						
tr|Q9RU7	tr|Q9RU7	1010	3.1E+08	2.93E+08	2.32E+08	2.1E+08	1.67E+08	1.74E+08
tr|Q9RU5	tr|Q9RU5	1001	32409000		59806000			
tr|Q9RSM	tr|Q9RSM	735	1.59E+08	1.99E+08	1.69E+08	1.93E+08	1.6E+08	1.47E+08
tr|Q9RW4	tr|Q9RW4	1355	3.49E+08	3.36E+08	2.97E+08	2.53E+08	2.83E+08	2.32E+08
tr|Q9RRH	tr|Q9RRH	526			4978900			
sp|Q9RRB	sp|Q9RRB	76						
tr|Q9RSL8	tr|Q9RSL8	734						
tr|Q9RRC	tr|Q9RRC	497						
tr|Q9RTY7	tr|Q9RTY7	959	47589000	1.56E+08	4.31E+08	40743000	66181000	57618000
tr|Q9RUD	tr|Q9RUD	1041	1.37E+09	1.45E+09	7.87E+08	1.1E+09	9.95E+08	1.06E+09
sp|Q9RU9	sp|Q9RU9	223	7240500			6450900		8427700
tr|Q9RT9	tr|Q9RT96	842	16230000	8320700	29975000	35950000	34019000	
tr|Q9RYH	tr|Q9RYH	1753	34626000	42076000	55524000			
tr|Q9RRV	tr|Q9RRV	593						
tr|Q9RUZ	tr|Q9RUZ	1146	92111000	1.44E+08	94605000	62615000	58053000	39528000
tr|Q9RRG	tr|Q9RRG	520	5.51E+08	1.5E+09	7.8E+08	1.16E+09	31094000	7.7E+08
tr|Q9RW8	tr|Q9RW8	1366						
tr|Q9RWT	tr|Q9RWT	1467	50237000	50859000	39350000	31373000	28713000	34528000
tr|Q9RYM	tr|Q9RYM	1773	65879000	1.05E+08	55159000	1.42E+08	1.15E+08	1.27E+08
tr|Q9RSB	tr|Q9RSB	686	1.83E+08	2.67E+08	2.43E+08	6.09E+08	5.1E+08	6.43E+08
tr|Q9RWX	tr|Q9RWX	1498			7299000		3339500	
sp|Q9RYX	sp|Q9RYX	447	25791000	34224000	42175000			
tr|Q9RVR	tr|Q9RVR	1285			37099000			
tr|Q9RRA	tr|Q9RRA	491		33558000	48462000	36326000	61915000	36513000
tr|Q9RUC	tr|Q9RUC	1036	2.92E+08	2.81E+08	2.34E+08	2.61E+08	2.27E+08	13443000
tr|Q9RVW	tr|Q9RVW	1311	6.65E+08	6.72E+08	5.63E+08	1.08E+09	9.52E+08	9.55E+08
tr|Q9RVW	tr|Q9RVW	1311		27077000	35021000	12111000		16390000
sp|Q9RUF	sp|Q9RUF	232						29832000
tr|Q9RXR	tr|Q9RXR	1633			32976000			
sp|Q9RW	sp|Q9RW	342	5168000		1065100			
tr|Q9RSM	tr|Q9RSM	735						
tr|Q9RXK	tr|Q9RXK	1608			1.85E+08			
sp|Q9RSQ	sp|Q9RSQ	140	17071000	24634000	33700000			
tr|Q9RYD	tr|Q9RYD	1735						
sp|Q9RUF	sp|Q9RUF	232	97988000	1.06E+08	83598000	1.93E+08	1.64E+08	1.76E+08
tr|Q9RT74	tr|Q9RT74	830	1.59E+08	1.08E+08	97006000	88108000	1.14E+08	90573000
tr|Q9RVG	tr|Q9RVG	1230	32069000	25035000	24783000	42157000	38005000	38922000
sp|Q9RTK	sp|Q9RTK	192	77657000	77673000	57396000	1.32E+08	71374000	89198000
tr|Q9RSK2	tr|Q9RSK2	730	1.56E+08	1.41E+08	2.7E+08			
tr|Q9RXM	tr|Q9RXM	1615			18642000		11564000	
tr|Q9RUV	tr|Q9RUV	1125		48316000	49855000	60814000	66013000	1.59E+08
tr|Q9RTV1	tr|Q9RTV1	936						
tr|Q9RTV1	tr|Q9RTV1	936						
tr|Q9RRW	tr|Q9RRW	595	2.25E+08	3.43E+08	3.46E+08	4.91E+08	4.81E+08	5.23E+08
sp|Q9RVY	sp|Q9RVY	303	6.56E+09	7.56E+09	6.33E+09	3.43E+09	3.05E+09	3.74E+09
tr|Q9RVJ0	tr|Q9RVJ0	1238			48426000	89631000	3.59E+08	77401000
tr|Q9RW8	tr|Q9RW8	1367	1.1E+09	8.64E+08	7.92E+08	3.21E+09	2.37E+09	2.28E+09
tr|Q9RW8	tr|Q9RW8	1367			22722000	30937000	28360000	42835000
tr|Q9RUQ	tr|Q9RUQ	1104						
tr|Q9RVJ0	tr|Q9RVJ0	1238	2.03E+08	2.13E+08	1.98E+08	4E+08	5.67E+08	3.8E+08

tr|Q9RVJ0	tr|Q9RVJ0	1238	96262000	1.23E+08	4.25E+08	25024000	1.51E+08	44394000
tr|Q9RXV9	tr|Q9RXV9	1654	41885000		25792000			
tr|Q9RUX5	tr|Q9RUX5	1134	33174000	29979000	23705000			
sp|Q9RSL1	sp|Q9RSL1	135	41016000				17757000	37959000
tr|Q9RWX	tr|Q9RWX	1499						
tr|Q9RS77	tr|Q9RS77	658				5026200		
tr|Q9RTG1	tr|Q9RTG1	871	3.78E+08	4.43E+08	14323000	14654000	7.12E+08	8.21E+08
sp|Q9RTI6	sp|Q9RTI6	188	5.29E+08	6.63E+08	5.39E+08	5.1E+08	4.57E+08	5.42E+08
tr|Q9RSY7	tr|Q9RSY7	785	40554000	32453000	36405000	18766000	25691000	35021000
tr|Q9RWR	tr|Q9RWR	1458						
tr|Q9RXQ	tr|Q9RXQ	1630	74730000	74289000	53973000			
tr|Q9RR62	tr|Q9RR62	467	3.52E+08	2.83E+08	3.71E+08	1.25E+08	2.01E+08	1.8E+08
tr|Q9RX85	tr|Q9RX85	1550		50719000		40404000	46170000	15344000
tr|Q9RWS	tr|Q9RWS	1464	18614000		10973000			
tr|Q9RU58	tr|Q9RU58	1001	40335000	19304000	21147000	11825000	13792000	13083000
tr|Q9RTQ7	tr|Q9RTQ7	917	4.22E+08	4.23E+08	5.03E+08	1.42E+09	1.54E+09	1.19E+09
tr|Q9RRY3	tr|Q9RRY3	606		2924400				
tr|Q9RZQ	tr|Q9RZQ	1957	24170000	38312000	82293000			81838000
tr|Q9RSD4	tr|Q9RSD4	696	2.12E+08	1.64E+08	2.13E+08	2.66E+08	2.1E+08	2.21E+08
tr|Q9RUX1	tr|Q9RUX1	1130						
tr|Q9RZQ	tr|Q9RZQ	1957						
tr|Q9RVV7	tr|Q9RVV7	1309					19287000	12992000
tr|Q9RUG	tr|Q9RUG	1055	3.34E+08	4.13E+08	3.43E+08	6.1E+08	4.97E+08	5.4E+08
tr|Q9RX36	tr|Q9RX36	1520	46223000				35717000	
sp|Q9RSY1	sp|Q9RSY1	156	3.49E+09	4.93E+09	4.6E+09	9.37E+08	1.06E+09	8.94E+08
sp|Q9RSY1	sp|Q9RSY1	156		29442000	1.38E+08			
tr|Q9RUA	tr|Q9RUA	1026			1181200			
sp|P8358	sp|P8358	60	1.03E+09	1.07E+08	1.01E+08	1.35E+09	1.16E+09	1.35E+09
tr|Q9RUK5	tr|Q9RUK5	1078						
sp|Q9RXK	sp|Q9RXK	385	1.15E+09	6.56E+08	1.08E+09		7687300	
tr|Q9RYT2	tr|Q9RYT2	1811						
sp|Q9RW0	sp|Q9RW0	304						
tr|Q9RZ56	tr|Q9RZ56	1864	50828000	1.39E+08	1.07E+08		45238000	14332000
tr|Q9RTP5	tr|Q9RTP5	909			29238000	48291000	47010000	39171000
tr|Q9RVS5	tr|Q9RVS5	1290					15590000	
tr|Q9RUI5	tr|Q9RUI5	1064	19973000		87528000	1.01E+08	29720000	74565000
tr|Q9RRX1	tr|Q9RRX1	601	48286000	54218000	58107000	18756000	20229000	28068000
sp|Q9RSQ	sp|Q9RSQ	142		21626000	29789000	20398000		
tr|Q9RUA	tr|Q9RUA	1020				60881000	40656000	55864000
tr|Q9RY78	tr|Q9RY78	1707	3.43E+08	25236000	3.38E+08	2.03E+08	1.61E+08	1.79E+08
tr|Q9RXC5	tr|Q9RXC5	1570	1.15E+08	1.57E+08	2.23E+08	77056000	1.18E+08	1.58E+08
tr|Q9RUS1	tr|Q9RUS1	1111	33222000	4.22E+08	3.24E+08	9455500		
tr|Q9RR75	tr|Q9RR75	472	2.26E+08	2.26E+08	1.86E+08	2.69E+08	2.46E+08	2.54E+08
tr|Q9RYN6	tr|Q9RYN6	1780				84416000		
tr|Q9RXK9	tr|Q9RXK9	1608						
tr|Q9RSL6	tr|Q9RSL6	732			24318000			
tr|Q9RVG7	tr|Q9RVG7	1229			6328300			
tr|Q9RUA	tr|Q9RUA	1024	19382000	24574000	19331000	29757000	16176000	17801000
tr|Q9RTR2	tr|Q9RTR2	921	5.08E+08	4.68E+08	4.51E+08	3.41E+08	2.72E+08	3.24E+08
tr|Q9RXC3	tr|Q9RXC3	1569		15057000	15045000			
sp|Q9RSJ6	sp|Q9RSJ6	124	1.07E+09	1.22E+09	1.05E+09	1.81E+09	1.19E+09	1.47E+09

sp|Q9RSJ6	sp|Q9RSJ6	124		11269000	70755000	16907000	28808000	21843000
tr|Q9RUA	tr|Q9RUA	1021		47844000				
sp|Q9RRB	sp|Q9RRB	76	3.11E+09	3.15E+09	2.44E+09	1.2E+09	9.99E+08	1.4E+09
tr|Q9RW5	tr|Q9RW5	1357	34146000	43248000	50136000	68219000	51763000	43245000
tr|Q9RRI6	tr|Q9RRI6	533	25667000		27682000	44790000	39326000	35804000
tr|Q9RSU4	tr|Q9RSU4	765	73652000	79729000	58517000		90885000	86633000
sp|Q9RR6	sp|Q9RR6	65	1.41E+09	1.28E+09	1.31E+09	1.51E+09	1.2E+09	1.46E+09
sp|Q9RV7	sp|Q9RV7	269			9265400			
sp|Q9RSP	sp|Q9RSP	139	8.25E+08	7.28E+08	6.94E+08	3.92E+08	5.1E+08	4.31E+08
sp|Q9RVG	sp|Q9RVG	287		1039700	1441000	1782400	2578400	2459400
sp|Q9RSA	sp|Q9RSA	114	59129000	1.29E+08	2.55E+08	98903000	1.06E+08	1.18E+08
tr|Q9RT09	tr|Q9RT09	798	83514000	1.54E+08	1.36E+08	2.25E+08	3.02E+08	1.85E+08
tr|Q9RT09	tr|Q9RT09	798		40990000	69037000	30781000	46572000	45339000
sp|Q9RWJ	sp|Q9RWJ	333	35999000	35649000	31512000	18708000		
tr|Q9RSU(	tr|Q9RSU(	762	1.36E+09	1.39E+09	1.19E+09	9.12E+08	8.97E+08	8.99E+08
sp|P5692(	sp|P5692(	59	75144000	75738000	1.39E+08	38019000	44602000	49763000
tr|Q9RY2C	tr|Q9RY2C	1681		43784000	63633000	39367000	18259000	29367000
tr|Q9RTR4	tr|Q9RTR4	923	28288000	54025000	44214000			
sp|Q9RZ0	sp|Q9RZ0	450				4544500	6710100	5767000
sp|Q9RR8	sp|Q9RR8	69	69680000	67983000	73836000	1.25E+08	93159000	1.09E+08
tr|Q9RU01	tr|Q9RU01	969		6653000	7238700		15406000	15022000
sp|Q9RUR	sp|Q9RUR	249	1332300				917630	
sp|Q9RUR	sp|Q9RUR	249						
tr|Q9RSA9	tr|Q9RSA9	680						
sp|Q9RXL	sp|Q9RXL	392				61260000	52650000	
tr|Q9RSF8	tr|Q9RSF8	710				53437000	92083000	66155000
sp|Q9RSL(	sp|Q9RSL(	132	29743000	32237000	48721000	36558000	64143000	57735000
tr|Q9RZD8	tr|Q9RZD8	1912			4203300		5096300	
sp|Q9RWI	sp|Q9RWI	329				1132200	1732300	2738400
tr|Q9RU28	tr|Q9RU28	981					19913000	
sp|Q9RUY	sp|Q9RUY	259						
sp|Q9RU7	sp|Q9RU7	214				40028000	71179000	26730000
tr|Q9RUL5	tr|Q9RUL5	1086			24208000		19600000	17517000
tr|Q9RRP4	tr|Q9RRP4	558				9683300		
tr|Q9RRP4	tr|Q9RRP4	558						
sp|Q9RYM	sp|Q9RYM	438			10043000			
tr|Q9RRA8	tr|Q9RRA8	491	45980000		23093000			
sp|Q9RXI	sp|Q9RXI	374	2.68E+08	14647000	4.68E+08	1.4E+09	2.13E+09	1.02E+09
sp|Q9RWI	sp|Q9RWI	335	28909000	27582000	14726000	10654000		
tr|Q9RVA5	tr|Q9RVA5	1202	4.12E+09	5.48E+09	3.89E+09	2.78E+09	2.48E+09	2.58E+09
tr|Q9RTX6	tr|Q9RTX6	951	1817800		2134900	3430800	4914500	3880000
sp|Q9RS1	sp|Q9RS1	106	47315000	50419000	45603000	74146000	86928000	72355000
sp|Q9RUD	sp|Q9RUD	227						
sp|Q9RUD	sp|Q9RUD	227		75743000	1.56E+08		1.43E+08	2.27E+08
tr|Q9RS49	tr|Q9RS49	644	58233000					
tr|Q9RT11	tr|Q9RT11	799	16131000	8974300	13747000			
sp|O3250	sp|O3250	45	4.41E+08	4.37E+08	5.02E+08	1.24E+08	1.77E+08	2.85E+08
tr|Q9RSP(	tr|Q9RSP(	743	55644000	55121000	47834000	1.35E+08	1.03E+08	1.38E+08
tr|Q9RUP1	tr|Q9RUP1	1099	46602000	43891000	55229000	55093000	51669000	
tr|Q9RUP1	tr|Q9RUP1	1099	1.87E+10	2.2E+10	1.7E+10	1.4E+10	8.2E+09	9.59E+09
sp|Q9RZA	sp|Q9RZA	454						

tr|Q9RRW	tr|Q9RRW	595	4.44E+08	3.84E+08	3.24E+08	3.41E+08	3.71E+08	4.14E+08
sp|Q9RXC	sp|Q9RXC	367	56085000	49795000	43769000			22958000
sp|Q9RXC	sp|Q9RXC	367	5955400	14655000	15418000	7400800	9788500	11695000
sp|Q9RW	sp|Q9RW	341	99545000	1.4E+08	1.17E+08	2.46E+08	1.94E+08	1.63E+08
sp|Q9RT6	sp|Q9RT6	170				2252900		1542100
tr|Q9RZE6	tr|Q9RZE6	1916	32933000	35879000	77654000		21181000	
sp|Q9RVY	sp|Q9RVY	303					11030000	
tr|Q9RV68	tr|Q9RV68	1185						
tr|Q9RU28	tr|Q9RU28	981	4.18E+08	4.03E+08	3.44E+08			
sp|Q9RU2	sp|Q9RU2	208	2.04E+08	1.84E+08		1.88E+08	1.98E+08	1.61E+08
sp|Q9RU2	sp|Q9RU2	208		28747000	53236000			
sp|Q9RXJ6	sp|Q9RXJ6	381	3117400	1772500	1324400	2400600	4021700	2747300
tr|Q9RYT8	tr|Q9RYT8	1816	33945000					
tr|Q9RWG	tr|Q9RWG	1406	68855000	57532000	52537000	37685000	37039000	51535000
sp|Q9RUF	sp|Q9RUF	231	16474000	18568000	19307000	22240000	21779000	24669000
tr|Q9RV27	tr|Q9RV27	1164	40709000		8261500	29019000	25688000	37757000
tr|Q9RUB	tr|Q9RUB	1027	1.51E+08	1.03E+08	1.2E+08	20289000	68365000	34213000
tr|Q9RX10	tr|Q9RX10	1507	2.94E+08	3.93E+08	3.47E+08	31712000	1.16E+08	37475000
sp|P5686	sp|P5686	54	12147000	13282000	15843000	2657300		
tr|Q9RT28	tr|Q9RT28	805		15311000	24282000	49680000	18849000	16704000
tr|Q9RVK1	tr|Q9RVK1	1244			1493100			
sp|Q9RW6	sp|Q9RW6	311	77832000	76995000	67301000	74534000	36306000	1.01E+08
tr|Q9RW8	tr|Q9RW8	1368	24672000	10922000	15077000		14971000	
sp|Q9RSJ9	sp|Q9RSJ9	127	8.9E+08	1.05E+09	7.94E+08	1.21E+09	1.18E+09	1.06E+09
sp|Q9RSJ9	sp|Q9RSJ9	127		18715000	81572000	1.12E+08	1.5E+08	1.35E+08
tr|Q9RW3	tr|Q9RW3	1350	10959000	3710100	4643100	2583000	3076500	2894200
tr|Q9RS07	tr|Q9RS07	623	86689000	86087000	68516000	1.58E+08	1.55E+08	1.31E+08
sp|Q9RXK	sp|Q9RXK	389						
sp|Q9RUY	sp|Q9RUY	259						3277900
sp|Q9RUY	sp|Q9RUY	259		29146000	92767000	75206000	10972000	
sp|Q9RUY	sp|Q9RUY	259			91836000	1.42E+08	1.72E+08	1.69E+08
tr|Q9RWF	tr|Q9RWF	1405						
sp|Q9RVY	sp|Q9RVY	303	57243000	1.47E+08	3.06E+08	3.91E+08	3.25E+08	4.65E+08
sp|Q9RVW	sp|Q9RVW	301	7644800	2900400	2379300	1200400	1779900	1450300
sp|Q9RVW	sp|Q9RVW	301						
tr|Q9RV31	tr|Q9RV31	1167	2.41E+08	99932000	1.79E+08	27432000	55753000	1.26E+08
sp|Q9RSQ	sp|Q9RSQ	140	2.73E+08	2.32E+08	2.37E+08	1.82E+08	1.99E+08	1.8E+08
tr|Q9RY86	tr|Q9RY86	1710	1.39E+08	1.58E+08	1.64E+08	70174000	45372000	52901000
tr|Q9RUT4	tr|Q9RUT4	1118		13185000		6916500	7242500	7028600
sp|Q9RXJ9	sp|Q9RXJ9	384	9409700	10756000	8216400	98358000	1.2E+08	95391000
tr|Q9RTB3	tr|Q9RTB3	847			8026100	62997000	61371000	45098000
tr|Q9RZT2	tr|Q9RZT2	1969	1.26E+08	72832000	63314000	39076000	74740000	53602000
tr|Q9RTG7	tr|Q9RTG7	873						
tr|Q9RTG7	tr|Q9RTG7	873	9415900	14641000	40970000		3945800	5726300
tr|Q9RTM	tr|Q9RTM	902	21234000		17233000			
tr|Q9RSX4	tr|Q9RSX4	779		46847000		23674000		29928000
tr|Q9RWV	tr|Q9RWV	1482		27877000	24666000			
sp|Q9RVC	sp|Q9RVC	279	17597000	22782000	22592000		22717000	33679000
tr|Q9RVT2	tr|Q9RVT2	1294						
tr|Q9RT71	tr|Q9RT71	828	2.2E+08	1.73E+08	1.84E+08	1.04E+08	1.13E+08	84019000
tr|Q9RT71	tr|Q9RT71	828	74785000	63210000	1.27E+08	14362000	20702000	24085000

tr|Q9RV4	tr|Q9RV46	1173	9507000		37917000			
sp|Q9RU	sp|Q9RUV	257	2.12E+08	2.01E+08	2.08E+08	3.02E+08	1.86E+08	2.39E+08
sp|Q9RU	sp|Q9RUV	257					15894000	16856000
tr|Q9RW	tr|Q9RWQ	1452		8040700	5734400		8135600	7455300
sp|Q9RSG	sp|Q9RSG	118	68410000	54497000	53381000	86228000	89821000	96596000
tr|Q9RYW	tr|Q9RYW	1827	1.39E+08	1.82E+08	1.4E+08		19616000	18409000
tr|Q9RS1	tr|Q9RS10	626						
tr|Q9RZQ	tr|Q9RZQ	1958	1.07E+08	90297000	93438000	86949000	93288000	71790000
tr|Q9RZQ	tr|Q9RZQ	1958	9569300	7203200	15005000			
sp|Q9RY	sp|Q9RYM	439	1.83E+08	2.88E+08	3.35E+08	3.31E+08	4.68E+08	3.1E+08
sp|Q9RY	sp|Q9RYM	439	45178000	22643000	54836000			
tr|Q9RTY	tr|Q9RTY3	957	7.4E+08	5.36E+08	4.91E+08	6.65E+08	6.03E+08	6.72E+08
tr|Q9RS8	tr|Q9RS88	664						
sp|Q9RXB	sp|Q9RXB	365	52201000	31563000	24038000			
tr|Q9RX1	tr|Q9RX18	1513						
tr|Q9RUG	tr|Q9RUG	1055	6.57E+08	6.63E+08	5.36E+08	8.9E+08	8.57E+08	8.42E+08
tr|Q9RUG	tr|Q9RUG	1055						
tr|Q9RUQ	tr|Q9RUQ	1102	1.11E+09	1.15E+09	1.57E+08	1.81E+09	1.98E+09	1.85E+09
tr|Q9RW8	tr|Q9RW8	1367	8.05E+08	1.08E+09	1.75E+09	1.43E+08	7.29E+08	3.16E+08
tr|Q9RVJ0	tr|Q9RVJ0	1238	3.74E+09	4.22E+09	5.14E+09	42452000	50421000	61610000
tr|Q9RY8	tr|Q9RY86	1710	5.48E+08	3.9E+08	3.55E+08	3.59E+08	2.94E+08	3.67E+08
tr|Q9RYP	tr|Q9RYP4	1783	27013000				85630000	1.11E+08
sp|Q9RSC	sp|Q9RSC	115	9558500	9215100	9409600	9190300	7786400	5470100
tr|Q9RZ1	tr|Q9RZ18	1853	30445000	14738000				
sp|Q9RR	sp|Q9RRM	92	6823700		13224000		3719900	
tr|Q9RZL	tr|Q9RZL6	1944				15964000	14748000	
tr|Q9RWG	tr|Q9RWG	1407	1.94E+09	2.17E+09	1.93E+09	1.24E+09	1.04E+09	1.08E+09
tr|Q9RVP	tr|Q9RVP5	1269	2.92E+09	1.88E+09	1.91E+09	2.96E+08	3.73E+08	4.89E+08
sp|Q9ZN	sp|Q9ZNA	463	22186000		34904000			
tr|Q9RVJ0	tr|Q9RVJ0	1238	48992000	82743000	2.22E+08		28133000	
tr|Q9RRE	tr|Q9RRE7	513	1.05E+08	1.1E+08	1.02E+08		1.18E+08	
tr|Q9RU7	tr|Q9RU76	1012		98083000	83951000		16493000	
tr|Q9RTT	tr|Q9RTT2	929	2.1E+08	1.69E+08	2.78E+08	55725000	66568000	1.24E+08
tr|Q9RXX	tr|Q9RXX3	1661	8.01E+08	1.07E+09	8.42E+08	5.63E+08	6.05E+08	5.73E+08
sp|Q9RV	sp|Q9RVV	300	6.5E+08	4.91E+08	6.33E+08	1.06E+08	92166000	69545000
tr|Q9RWT	tr|Q9RWT	1467	55875000	67494000	81410000	61696000		57419000
sp|Q9RZ0	sp|Q9RZ0	450				52003000		
tr|Q9RW0	tr|Q9RW0	1334		2688100	3343900			4582200
sp|Q9RRB	sp|Q9RRB	76	4.25E+08	4.57E+08	3.76E+08	62358000	70000000	1.4E+08
tr|Q9RVA	tr|Q9RVA5	1202	7.46E+09	8.37E+09	5.82E+09	2.91E+09	2.06E+09	2.56E+09
sp|Q9RXG	sp|Q9RXG	370						
sp|Q9RXG	sp|Q9RXG	370			15124000	9206800		
tr|Q9RTC	tr|Q9RTC9	855						
tr|Q9RYC	tr|Q9RYC5	1726		12698000		17282000	14241000	
sp|Q9RRY	sp|Q9RRY	103				27252000		19163000
tr|Q9RSZ	tr|Q9RSZ9	791	1.42E+09	1.42E+09	1.11E+09	1.82E+09	1.66E+09	1.69E+09
sp|Q9RXE	sp|Q9RXE	368	13377000	18899000	16393000	42336000	19109000	35530000
tr|Q9RZ3	tr|Q9RZ33	1861					9570700	
tr|Q9RZU	tr|Q9RZU	1976	8782600		10274000	12346000	14264000	39563000
sp|Q9RY2	sp|Q9RY2	411	25450000		22273000	28969000	32841000	21522000
sp|P5692	sp|P56926	59	28574000	33421000	30970000		29964000	32322000

tr|Q9RT75	tr|Q9RT75	831						
tr|Q9RYM	tr|Q9RYM	1773	70628000	70537000	59897000	36561000	58194000	39857000
sp|Q9RS6	sp|Q9RS6	112	14572000	21263000	15897000	1.09E+08	1.2E+08	77711000
tr|Q9RT49	tr|Q9RT49	818			17898000			
tr|Q9RYF0	tr|Q9RYF0	1742	38471000		50737000	24365000	30293000	32197000
tr|Q9RRA6	tr|Q9RRA6	489;1984	17255000	23685000	18355000			
tr|Q9RRA6	tr|Q9RRA6	489	19978000	13434000	37151000	28834000	23953000	39785000
tr|Q9RYI1	tr|Q9RYI1	1760	1.69E+08	2.31E+08	2E+08	1.84E+08	2.21E+08	1.64E+08
tr|Q9RT75	tr|Q9RT75	831	1.27E+08	74773000	64592000	76311000	95188000	97752000
sp|Q9RTG	sp|Q9RTG	185	55104000	38377000	64587000		33675000	
tr|Q9RZ18	tr|Q9RZ18	1853	88939000					
tr|Q9RXX9	tr|Q9RXX9	1663			2559500	5429200		
sp|Q9RTN	sp|Q9RTN	196	1.14E+09	9.49E+08	8.97E+08	1.39E+09	1.26E+09	1.29E+09
sp|Q9RTN	sp|Q9RTN	196						
sp|Q9RV2	sp|Q9RV2	262	9414700	7673800	7281100	5795800		7205400
tr|Q9RTA8	tr|Q9RTA8	846	3.34E+08	3.07E+08	2.76E+08	3.89E+08	2.82E+08	3.37E+08
sp|Q9RSL1	sp|Q9RSL1	135	11862000		4664000	11107000		
sp|Q9RSL1	sp|Q9RSL1	135	34652000		44291000	91320000	82950000	63677000
tr|Q9RTI3	tr|Q9RTI3	879					6022200	
tr|Q9RZE4	tr|Q9RZE4	1914	66848000	36631000	70535000			
tr|Q9RRR9	tr|Q9RRR9	571						
tr|Q9RWT	tr|Q9RWT	1465	3.71E+08	40057000	2.87E+08	28270000	3.47E+08	4.3E+08
tr|Q9RRG4	tr|Q9RRG4	520				32680000		
sp|Q9RR6	sp|Q9RR6	65	1442500			696400		835530
sp|Q9RR6	sp|Q9RR6	65	27268000	31968000	49820000			
tr|Q9RV74	tr|Q9RV74	1188	23087000	16862000	17571000			
tr|Q9RVJ4	tr|Q9RVJ4	1241	2.97E+08	2.8E+08	2.5E+08	2.2E+08	1.99E+08	
sp|Q9RRB	sp|Q9RRB	76	3.99E+08	2.94E+08	3.2E+08	1.37E+08	6.52E+08	6.34E+08
tr|Q9RRI4	tr|Q9RRI4	532	1.39E+08	1.07E+08	80341000	77237000	79466000	1.02E+08
sp|Q9RY0	sp|Q9RY0	410	99784000	1.07E+08	98411000	1.31E+08	1.2E+08	1.07E+08
sp|Q9RVW	sp|Q9RVW	301	1.96E+08	5.04E+08	2.26E+08	3.29E+08	3.23E+08	2.48E+08
tr|Q9RSD2	tr|Q9RSD2	694	51814000	41950000	90358000		38997000	18941000
sp|Q9RWU	sp|Q9RWU	347			12484000	72963000		53396000
tr|Q9RZE3	tr|Q9RZE3	1913	9785400	19871000	51485000		16817000	11768000
tr|Q9RVG1	tr|Q9RVG1	1229		24411000				
tr|Q9RSF2	tr|Q9RSF2	708		40425000	37052000	47501000	60405000	48085000
tr|Q9RXX3	tr|Q9RXX3	1661			30832000			
sp|Q9RSI4	sp|Q9RSI4	121	22542000		34752000			
tr|Q9RVU8	tr|Q9RVU8	1303		57400000	1.02E+08		40501000	
tr|Q9RZP7	tr|Q9RZP7	1955		22851000		51467000	46003000	49912000
sp|Q9RUR	sp|Q9RUR	249	2.45E+08	2.91E+08	4.2E+08	2.24E+08	2.64E+08	2.44E+08
sp|Q9RUR	sp|Q9RUR	249	32805000	34781000	2.73E+08	3.46E+08	1.26E+09	7.09E+08
sp|Q9RY2	sp|Q9RY2	411	1.14E+08	95338000	2.4E+08	1.46E+08	1.11E+08	1.99E+08
sp|Q9RY2	sp|Q9RY2	411					17739000	16303000
tr|Q9RXD5	tr|Q9RXD5	1573		3.78E+08	4.95E+08	5.44E+08	5.67E+08	43987000
tr|Q9RYP6	tr|Q9RYP6	1785	61367000	40800000	1E+08	14849000	16856000	27385000
tr|Q9RUM	tr|Q9RUM	1092	20003000	16882000		17256000	9129500	13585000
tr|Q9RWP	tr|Q9RWP	1448	9231500				22579000	
tr|Q9RRR9	tr|Q9RRR9	571						
tr|Q9RS13	tr|Q9RS13	628	1.54E+08	1.14E+08	1.03E+08	54704000	45516000	29682000
sp|Q9RVW	sp|Q9RVW	301			20443000	38985000		14113000

tr|Q9RX95	tr|Q9RX95	1553	3490300					
sp|Q9RZA	sp|Q9RZA	454			18460000	16747000		
sp|Q9RRX	sp|Q9RRX	101	13189000				6275700	
sp|P52153	sp|P52153	52	1.93E+08	1.6E+08	1.46E+08	1.64E+08	1.82E+08	1.79E+08
tr|Q9RYX2	tr|Q9RYX2	1833	61228000	95241000	45109000	38457000		32206000
tr|Q9RVJ4	tr|Q9RVJ4	1241						
sp|Q9RSS	sp|Q9RSS	147	73749000	1.09E+08	74606000	2.98E+08	1.5E+08	1.24E+08
sp|Q9RSR	sp|Q9RSR	143	1.88E+08	1.73E+08	1.35E+08	3.41E+08	2.91E+08	3.27E+08
sp|Q9RSR	sp|Q9RSR	143			5067800	9421000	8130600	11127000
tr|Q9RTR2	tr|Q9RTR2	921	23267000					
tr|Q9RX36	tr|Q9RX36	1520						
tr|Q9RSB1	tr|Q9RSB1	681	41458000		48391000		50587000	68793000
tr|Q9RWH	tr|Q9RWH	1410	35642000	39376000	35198000	93569000	96266000	70973000
tr|Q9RZH2	tr|Q9RZH2	1928						
sp|Q9RXK	sp|Q9RXK	390	19799000	6090700	8829400			
tr|Q9RWW	tr|Q9RWW	1490	4.68E+08	5.97E+08	6.15E+08	2.16E+08	3.38E+08	2.82E+08
sp|Q9RY4	sp|Q9RY4	415	3.19E+08	3.43E+08	2.91E+08	3.81E+08	3.73E+08	3.74E+08
sp|Q9RY4	sp|Q9RY4	415			47181000			27279000
sp|Q9RSJ7	sp|Q9RSJ7	125	5.2E+09	4.38E+09	3.68E+09	2.62E+09	2.83E+09	2.74E+09
tr|Q9RXQ	tr|Q9RXQ	1627	1.39E+08		65696000	24375000	18625000	22574000
tr|Q9RVB4	tr|Q9RVB4	1208	1.54E+08	4.17E+08	3.8E+08	9.23E+08	7.31E+08	7.87E+08
sp|Q9RSL0	sp|Q9RSL0	132	8360200	5025400	9116900	1008300	2793800	2759600
tr|Q9RYT5	tr|Q9RYT5	1814	1.35E+08			2.18E+08	1.52E+08	1.61E+08
tr|Q9RU50	tr|Q9RU50	994	13165000	3442800	3137000	3718500	6495100	4606000
sp|Q9RWI	sp|Q9RWI	335	30274000	26439000	23669000	19109000	15388000	24406000
tr|Q9RU07	tr|Q9RU07	972	3.15E+08	1.66E+08	3.25E+08	29915000	37549000	72299000
sp|Q9RU8	sp|Q9RU8	216	7.37E+09	7.41E+09	5.4E+09	8.13E+09	6.71E+09	7.11E+09
tr|Q9RVP1	tr|Q9RVP1	1267	4.11E+08	3.59E+08	3.22E+08	3.07E+08	2.11E+08	2.57E+08
sp|Q9RUG	sp|Q9RUG	234	4.76E+08	4.27E+08	3.79E+08	3.26E+08	3.39E+08	3.37E+08
tr|Q9RY98	tr|Q9RY98	1717						
sp|Q9RU8	sp|Q9RU8	219	1.77E+08	1.9E+08	1.83E+08	2.36E+08	2.15E+08	2.19E+08
tr|Q9RXS1	tr|Q9RXS1	1638						
tr|Q9RVM	tr|Q9RVM	1257	3.75E+08	1E+09	7.43E+08	4.45E+08	7.64E+08	4.97E+08
tr|Q9RRA1	tr|Q9RRA1	484						
tr|Q9RWC	tr|Q9RWC	1391	60478000		63375000		18264000	21400000
tr|Q9RYV4	tr|Q9RYV4	1822	50581000	47229000	47308000	35892000	53856000	39399000
tr|Q9RYV4	tr|Q9RYV4	1822		18770000	69415000		6998000	
sp|Q9RYL	sp|Q9RYL	436	60886000			80570000	65173000	65765000
tr|Q9RUD	tr|Q9RUD	1039				39372000	43582000	38233000
tr|Q9RR85	tr|Q9RR85	476						
tr|Q9RTQ8	tr|Q9RTQ8	918	10040000		12550000			
tr|Q9RUX5	tr|Q9RUX5	1134	1.32E+08	1.82E+08	1.38E+08	60578000	1.31E+08	34588000
tr|Q9RYN9	tr|Q9RYN9	1782	81472000	55202000	50466000	79205000	62302000	67522000
tr|Q9RXQ	tr|Q9RXQ	1627	81901000	55569000	57417000	72645000	75294000	74632000
sp|Q9RSL1	sp|Q9RSL1	135	1.1E+08	1.11E+08	2.09E+08	1.97E+08	2.18E+08	2.08E+08
tr|Q9RRM	tr|Q9RRM	548	35677000	39183000	41085000	52899000	55257000	49864000
tr|Q9RRM	tr|Q9RRM	548	1.11E+08	23214000	1.34E+08		61605000	91539000
tr|Q9RV05	tr|Q9RV05	1153	88873000	1.21E+08	1.02E+08	71145000	1.07E+08	
sp|Q9RRC	sp|Q9RRC	79	21572000	18857000	16155000	21762000	31257000	22797000
tr|Q9RVU2	tr|Q9RVU2	1300		28112000			93121000	
tr|Q9RXV6	tr|Q9RXV6	1652						

tr|Q9RZ03	tr|Q9RZ03	1843						
tr|Q9RU02	tr|Q9RU02	970			34353000			
sp|Q9RXK	sp|Q9RXK	387	2.15E+08	2.58E+08	2.41E+08		1.12E+08	
sp|Q9RXK	sp|Q9RXK	387			32153000		14237000	
tr|Q9RV18	tr|Q9RV18	1161	4.76E+08	3.65E+08	3.9E+08	1.11E+08	1.14E+08	1.64E+08
sp|Q9RSI4	sp|Q9RSI4	121	1.05E+08	98727000	84123000	2.17E+08	2.57E+08	2.56E+08
tr|Q9RRX1	tr|Q9RRX1	601	2.15E+09	1.82E+09	1.63E+09	1.33E+09	1.45E+09	1.51E+09
tr|Q9RRX1	tr|Q9RRX1	601	1.61E+08	3.36E+08	3.25E+08	70828000	1.09E+08	1.14E+08
tr|Q9RRI4	tr|Q9RRI4	532	42008000	29791000	35005000		34436000	29694000
sp|Q9RRB	sp|Q9RRB	76	1.04E+09	1.63E+09	1.2E+09	8.88E+08	9.81E+08	9.08E+08
sp|Q4657	sp|Q4657	61	3.73E+08	2.95E+08	2.28E+08	4.27E+08	3.5E+08	4.52E+08
sp|P42443	sp|P42443	49	52408000	25100000	38056000			
tr|Q9RWP	tr|Q9RWP	1448	27682000	26900000				
sp|Q9RW(	sp|Q9RW(	327	1.22E+08	33322000	2.01E+08	2.06E+09	2.6E+08	3.31E+08
sp|Q9RVW	sp|Q9RVW	301	2.81E+08	3.21E+08	2.46E+08	1.69E+08	2.08E+08	1.84E+08
sp|Q9RYB	sp|Q9RYB	426	37863000		9527000	753590	2384200	1583200
tr|Q9RVG8	tr|Q9RVG8	1230	7.79E+08	9.02E+08	7.38E+08	4.44E+08	6.16E+08	5.16E+08
tr|Q9RZC7	tr|Q9RZC7	1906		15118000	22787000			
sp|Q9RRY	sp|Q9RRY	103	975890	174260				
sp|Q9RUR	sp|Q9RUR	249						
sp|Q9RUR	sp|Q9RUR	249					15114000	32484000
sp|Q9RUE	sp|Q9RUE	229				12698000	12861000	11395000
sp|Q9RUE	sp|Q9RUE	229						
sp|Q9RY4	sp|Q9RY4	415	5.7E+08	4.8E+08	4.07E+08	7.73E+08	8.08E+08	8.14E+08
tr|Q9RU65	tr|Q9RU65	1006	13613000					
sp|Q9RU8	sp|Q9RU8	216	68944000		68771000	2.24E+08	85855000	2.08E+08
sp|P42443	sp|P42443	49	1.33E+08	1.55E+08	1.35E+08		56452000	
tr|Q9RWJ5	tr|Q9RWJ5	1422	29176000	35863000	24456000	2.77E+08	2.16E+08	2.18E+08
sp|Q9RXC	sp|Q9RXC	367	5.19E+08	7.32E+08	5.26E+08	5.71E+08	4.36E+08	4.87E+08
sp|Q9RRB	sp|Q9RRB	76			22202000		11272000	
tr|Q9RTC9	tr|Q9RTC9	855	79443000	1.06E+08	1.04E+08	61640000	71420000	68717000
tr|Q9RUY1	tr|Q9RUY1	1138						
sp|P8358	sp|P8358	60	1.35E+09	1.32E+09	1.12E+09	8.24E+08	6.55E+08	8.17E+08
tr|Q9RRV8	tr|Q9RRV8	593			76102000	3.49E+08	83511000	
tr|Q9RW4	tr|Q9RW4	1353		1.68E+08	93493000			
tr|Q9RRC7	tr|Q9RRC7	501						
tr|Q9RVL5	tr|Q9RVL5	1252	4.47E+08	4.19E+08	3.29E+08	92175000	1.04E+08	1.22E+08
tr|Q9RVE5	tr|Q9RVE5	1219					42027000	
sp|Q9RS8	sp|Q9RS8	113			3809500		4575100	9732900
tr|Q9RVS8	tr|Q9RVS8	1292	1.77E+10	2.24E+10	1.77E+10	2.15E+10	2.01E+10	2.15E+10
tr|Q9RVS8	tr|Q9RVS8	1292	87131000	2.04E+08	3.45E+08			
tr|Q9RXG3	tr|Q9RXG3	1589	97866000	96877000	97368000	23200000		
tr|Q9RVD8	tr|Q9RVD8	1216			42693000	16942000		
tr|Q9RUH	tr|Q9RUH	1056				9777600		9124900
tr|Q9RUE0	tr|Q9RUE0	1044	2.5E+09	2.38E+09	1.83E+09	6.45E+09	5.83E+09	5.93E+09
sp|Q9RUE	sp|Q9RUE	226	18163000	17253000	15383000	24576000	24350000	26413000
sp|Q9RT2	sp|Q9RT2	163	1972800		816090			
sp|Q9RT2	sp|Q9RT2	163	59597000	36923000	41545000	42386000	28830000	
sp|Q9RT2	sp|Q9RT2	164	3324100	945720	920110	810900	834740	693240
tr|Q9RRW	tr|Q9RRW	597	58081000	73558000	59448000	1.4E+08	1.49E+08	1.38E+08
tr|Q9RRW	tr|Q9RRW	597	4.79E+08	5.51E+08	1.11E+09	4.83E+08	5.1E+08	6.81E+08

tr|Q9RRX3	tr|Q9RRX3	602	48863000					
sp|Q9RSZ	sp|Q9RSZ	159		51079000		7.55E+08	6.61E+08	
sp|Q9RYD	sp|Q9RYD	430	3.71E+08	2.58E+08	2.49E+08	2.71E+08	2.89E+08	3.06E+08
tr|Q9RVG8	tr|Q9RVG8	1230	1.15E+08	1.07E+08	99717000	1.08E+08	87401000	1.01E+08
tr|Q9RST7	tr|Q9RST7	760				18252000	19300000	26755000
tr|Q9RW3	tr|Q9RW3	1349	12943000				10990000	
tr|Q9RV82	tr|Q9RV82	1191						
sp|Q9RV9	sp|Q9RV9	273			25961000	1.42E+08	1.68E+08	1.75E+08
tr|Q9RWP	tr|Q9RWP	1445				2.41E+08	2.71E+08	2.1E+08
tr|Q9RU28	tr|Q9RU28	981	1.58E+09	2.02E+09	1.43E+09	2.14E+09	2.08E+09	1.99E+09
tr|Q9RSX3	tr|Q9RSX3	778						
tr|Q9RS08	tr|Q9RS08	624	1.1E+09	1.01E+09	1.32E+09		2.75E+08	2.65E+08
sp|Q9RSF	sp|Q9RSF	117	18747000	12021000	13906000	11084000	8393200	6731500
tr|Q9RST1	tr|Q9RST1	757		63157000	71452000		27447000	41764000
tr|Q9RRE7	tr|Q9RRE7	513						
tr|Q9RR99	tr|Q9RR99	482	49164000	62494000	50396000	1.03E+08	93028000	94558000
tr|Q9RVJ3	tr|Q9RVJ3	1240	35147000	31614000	30322000	14921000	26131000	17612000
tr|Q9RVJ3	tr|Q9RVJ3	1240	37028000	41524000	1.06E+08	30344000		45082000
tr|Q9RT82	tr|Q9RT82	836	29820000		19740000			
tr|Q9RZ87	tr|Q9RZ87	1879	1.25E+08	1.03E+08	94881000	2.3E+08	2.63E+08	2.44E+08
tr|Q9RTM	tr|Q9RTM	900	43612000	38621000	40941000			
sp|O3250	sp|O3250	46				13069000		
tr|Q9RVS2	tr|Q9RVS2	1288	5.08E+08	3.71E+08	3.55E+08	91928000	1E+08	1.18E+08
sp|Q9RTG	sp|Q9RTG	184	14697000	38404000	24035000			
tr|Q9RUH	tr|Q9RUH	1057	66634000		31081000			
sp|Q9RTG	sp|Q9RTG	184				21534000		
tr|Q9RVG	tr|Q9RVG	1228				75924000	1.09E+08	89766000
tr|Q9RZ04	tr|Q9RZ04	1844				20262000	13715000	10947000
tr|Q9RT97	tr|Q9RT97	843	1.55E+08	1.86E+08	1.9E+08	1.76E+08	1.55E+08	1.62E+08
tr|Q9RYM	tr|Q9RYM	1773	23559000			6135500		
tr|Q9RXW	tr|Q9RXW	1655						
tr|Q9RXN	tr|Q9RXN	1619			13325000	28338000	41293000	18437000
tr|Q9RXN	tr|Q9RXN	1619			50485000			
sp|Q9RUF	sp|Q9RUF	231				3.3E+08		
tr|Q9RV17	tr|Q9RV17	1160	19215000	21044000	31124000		13049000	
tr|Q9RZ78	tr|Q9RZ78	1872						
tr|Q9RUT	tr|Q9RUT	1116	20633000	28172000	30669000	32232000	29914000	
tr|Q9RWQ	tr|Q9RWQ	1451		21946000				
tr|Q9RUU	tr|Q9RUU	1123	26791000	24022000	16786000	31265000	57454000	24948000
tr|Q9RY33	tr|Q9RY33	1686				20719000	30348000	38160000
tr|Q9RY33	tr|Q9RY33	1686				12664000	14439000	
tr|Q9RZH5	tr|Q9RZH5	1930	1.71E+08	1.44E+08	1.43E+08	1.18E+08	1.11E+08	1.13E+08
tr|Q9RY13	tr|Q9RY13	1679	38011000		22998000	58224000	38204000	38555000
tr|Q9RY62	tr|Q9RY62	1700	62877000	86508000	28077000	18324000	23799000	24910000
tr|Q9RX11	tr|Q9RX11	1508				1.33E+08		
tr|Q9RUX1	tr|Q9RUX1	1130						
sp|Q9RRE	sp|Q9RRE	82	44436000	84470000	15673000		33377000	89803000
tr|Q9RRY3	tr|Q9RRY3	606			5536700			
sp|Q9RW0	sp|Q9RW0	327	92337000	32257000	1.48E+08	1.75E+08	2.79E+08	1.6E+08
sp|Q9RT3	sp|Q9RT3	166	16579000	21275000	22669000			
sp|Q9RT5	sp|Q9RT5	168				65666000	48527000	68332000

tr|Q9RUA	tr|Q9RUA	1023	1.86E+09	1.35E+09	1.37E+09	1.47E+09	1.35E+09	1.56E+09
tr|Q9RSZ5	tr|Q9RSZ5	790	6.89E+08	6.1E+08	4.91E+08	3.41E+08	2.73E+08	3.6E+08
sp|O83026	sp|O83026	47						
tr|Q9RZ09	tr|Q9RZ09	1847		38066000	29480000	88584000		
tr|Q9RRS0	tr|Q9RRS0	572	39697000	51253000	48935000	34447000	25717000	
sp|Q9RV8	sp|Q9RV8	270						
sp|Q9RVA	sp|Q9RVA	274	3.9E+08	3.87E+08	2.98E+08	5.73E+08	5.3E+08	5.82E+08
tr|Q9RRP4	tr|Q9RRP4	558						
sp|Q9RXK	sp|Q9RXK	389	6.03E+08	4.19E+08	4.98E+08	1.37E+08	1.42E+08	1.19E+08
tr|Q9RVP3	tr|Q9RVP3	1268	30661000	28435000	19368000	49598000	38330000	66460000
sp|Q9RWN	sp|Q9RWN	340	2.87E+08	2.68E+08	2.32E+08	2.91E+08	2.73E+08	2.56E+08
tr|Q9RXV5	tr|Q9RXV5	1651	35501000	47022000	86231000	56143000	84598000	78182000
tr|Q9RTC9	tr|Q9RTC9	855	63555000	1.22E+08	96009000			
tr|Q9RYS8	tr|Q9RYS8	1807	3.88E+08	3.21E+08	2.35E+08	4.71E+08	4.38E+08	4.22E+08
tr|Q9RYG7	tr|Q9RYG7	1751	1.1E+08	1.05E+08	93043000	1.91E+08	1.7E+08	1.55E+08
tr|Q9RVQ	tr|Q9RVQ	1277						
tr|Q9RZ85	tr|Q9RZ85	1877	7.73E+08	4.24E+08	4.86E+08	1.46E+08	2.84E+08	1.75E+08
tr|Q9RTQ6	tr|Q9RTQ6	916						
tr|Q9RTL5	tr|Q9RTL5	896	18926000	22156000	14506000	43049000		37260000
sp|Q9RTA	sp|Q9RTA	175			26404000			
tr|Q9RW8	tr|Q9RW8	1371	9.17E+08	1.09E+09	5.92E+08	8.68E+08	9.39E+08	8.32E+08
tr|Q9RZ88	tr|Q9RZ88	1880				23269000	23915000	24947000
tr|Q9RXG0	tr|Q9RXG0	1587	2.43E+08	4.26E+08	3.99E+08	4.67E+08	3.75E+08	3.56E+08
tr|Q9RYU9	tr|Q9RYU9	1819	80821000	88105000	69004000	1.01E+08	91593000	1.01E+08
tr|Q9RYU9	tr|Q9RYU9	1819	43155000	53790000	14761000	37269000	40085000	
tr|Q9RY86	tr|Q9RY86	1710	16923000		50260000	28078000	68246000	32670000
tr|Q9RV27	tr|Q9RV27	1164	3.46E+08	4.37E+08	5.45E+08	85624000	1.45E+08	1.91E+08
tr|Q9RXN9	tr|Q9RXN9	1622						
tr|Q9RYS7	tr|Q9RYS7	1806	33352000	18352000	16834000			
sp|Q9RZE	sp|Q9RZE	455	60474000	39961000	67202000	20478000	22552000	10332000
sp|Q9RZV	sp|Q9RZV	460	1E+08	82232000	65049000			
sp|Q9RRQ	sp|Q9RRQ	94	65652000	49557000	46068000	24982000	36858000	32927000
tr|Q9RTM	tr|Q9RTM	902	43690000	55923000				
tr|Q9RYS0	tr|Q9RYS0	1800						
tr|Q9RVS2	tr|Q9RVS2	1288	2.99E+09	2.38E+09	2.14E+09	3.46E+09	2.67E+09	2.8E+09
tr|Q9RVS2	tr|Q9RVS2	1288	14441000	36858000	48438000			8465300
tr|Q9RZQ	tr|Q9RZQ	1958	2.28E+08	2.53E+08	4.24E+08	1.85E+08	1.19E+08	2.09E+08
tr|Q9RVB2	tr|Q9RVB2	1206			14669000			
tr|Q9RXV6	tr|Q9RXV6	1652	3.81E+08			79810000	66368000	89044000
tr|Q9RSZ9	tr|Q9RSZ9	791	2.56E+08	21976000	3.89E+08	18375000	2.08E+08	1.84E+08
tr|Q9RSH1	tr|Q9RSH1	717						
tr|Q9RWT	tr|Q9RWT	1472				28081000	19180000	
tr|Q9RTC7	tr|Q9RTC7	853	1.97E+09	7.29E+08	1.07E+09	1.2E+09	3.59E+08	9.85E+08
sp|Q9RYG	sp|Q9RYG	434	1.51E+09	1.31E+09	1.28E+09	7.47E+08	7.86E+08	8.6E+08
tr|Q9RS52	tr|Q9RS52	646						
sp|Q9RSZ	sp|Q9RSZ	159	2.51E+08	2.22E+08	1.83E+08	5.17E+08	5.41E+08	5.08E+08
tr|Q9RWT	tr|Q9RWT	1465	2.72E+09	2.46E+09	1.96E+09	2.14E+09	2.16E+09	2.19E+09
tr|Q9RVR5	tr|Q9RVR5	1284	7852000	3275200	2531800	1632100	2849600	2853700
tr|Q9RSA2	tr|Q9RSA2	673	29583000	31135000	36627000	8878900		
tr|Q9RRR8	tr|Q9RRR8	570		25534000	57151000	53365000	39164000	57062000
tr|Q9RY55	tr|Q9RY55	1697		22553000	13780000		12823000	

sp|Q9RUV	sp|Q9RUV	254	42244000					
sp|Q9RW	sp|Q9RW	327	7.04E+09	6.17E+09	5.4E+09	4.63E+09	4.38E+09	4.83E+09
tr|Q9RSY	tr|Q9RSY	787	4.55E+08		3.13E+08	57713000	76594000	72049000
tr|Q9RVF	tr|Q9RVF	1224	20647000		15015000	42565000	20359000	24915000
tr|Q9RSJ2	tr|Q9RSJ2	728	7.08E+08	7.94E+08	1.35E+09	5.67E+08	8.38E+08	5.04E+08
tr|Q9RRD	tr|Q9RRD	506	68718000		64957000	82088000	55939000	87862000
tr|Q9RRV	tr|Q9RRV	590	50791000	34286000	32176000			1.06E+08
sp|Q9RXK	sp|Q9RXK	389	1.3E+08	83955000	7.59E+08	5.56E+08	8.72E+08	5.92E+08
sp|Q9RXK	sp|Q9RXK	389	28099000		35136000			
tr|Q9RUR	tr|Q9RUR	1108			1642900			
tr|Q9RY3	tr|Q9RY3	1687	2.81E+08	4.12E+08	3.67E+08	1.73E+08	3.72E+08	2.57E+08
tr|Q9RU2	tr|Q9RU2	981	13482000	25006000	26767000	39235000	76395000	38750000
tr|Q9RSW	tr|Q9RSW	775		86570000	88670000		73325000	60718000
sp|O8302	sp|O8302	47	18169000	26716000	25530000		28827000	
tr|Q9RZ1	tr|Q9RZ1	1853	1.69E+08	1.48E+08	2.65E+08		73542000	
tr|Q9RVZ	tr|Q9RVZ	1329						
sp|Q9RVT	sp|Q9RVT	297			5284300	16913000		
tr|Q9RYN	tr|Q9RYN	1777						
tr|Q9RVS	tr|Q9RVS	1288						
tr|Q9RVS	tr|Q9RVS	1288	18742000	35937000	42541000		54827000	
tr|Q9RU5	tr|Q9RU5	999	6.3E+08	8.01E+08	8.27E+08	2.55E+08	4.25E+08	3.81E+08
sp|Q9RU8	sp|Q9RU8	217		24452000	29331000			
tr|Q9RY3	tr|Q9RY3	1687				12991000	19192000	19454000
tr|Q9RV8	tr|Q9RV8	1192	5.12E+08	5.86E+08	5.06E+08	3.94E+08	3.14E+08	4.52E+08
tr|Q9RV8	tr|Q9RV8	1192			28135000	59996000	62522000	49803000
tr|Q9RSL8	tr|Q9RSL8	734	12138000	5665700	7691600		1298400	
sp|Q9RSV	sp|Q9RSV	154						
tr|Q9RTE	tr|Q9RTE	862	69693000	74102000	60515000	85447000	66859000	61774000
tr|Q9RTE	tr|Q9RTE	862	22691000	28283000	40722000	28398000		39888000
tr|Q9RX7	tr|Q9RX7	1545	12897000	17709000	23930000	24163000	23077000	14369000
tr|Q9RSL8	tr|Q9RSL8	734	1.5E+08	1.72E+08	1.21E+08	1.45E+08	1.24E+08	1.37E+08
tr|Q9RYQ	tr|Q9RYQ	1788	3.48E+09	2.77E+09	3E+09	1.82E+08	1.83E+08	2.86E+08
tr|Q9RVA	tr|Q9RVA	1202	1.5E+09	2.71E+09	1.8E+09	6.9E+08	7.95E+08	6.52E+08
tr|Q9RVA	tr|Q9RVA	1202	1.66E+08		2.34E+08			
sp|Q9RW	sp|Q9RW	325		1.3E+08	2.63E+08			38965000
tr|Q9RV3	tr|Q9RV3	1168			17295000			
sp|Q9RSQ	sp|Q9RSQ	140		49174000	93266000	19115000	58623000	27829000
tr|Q9RSA	tr|Q9RSA	673						
sp|Q9RSR	sp|Q9RSR	143	57706000	46633000	45661000	86255000	83865000	57626000
tr|Q9RUH	tr|Q9RUH	1059	5.75E+08	2.5E+08	7.05E+08	5.81E+08	6.91E+08	5.06E+08
tr|Q9RVM	tr|Q9RVM	1257	6.26E+08	6.71E+08	6.05E+08	6.79E+08	5.55E+08	5.78E+08
tr|Q9RTE2	tr|Q9RTE2	861	4.99E+08	3.54E+08	3.17E+08	5.04E+08	5.72E+08	6.29E+08
sp|Q9RSJ	sp|Q9RSJ	124	1.08E+08	1.43E+08	1.28E+08	1.91E+08	3.13E+08	1.88E+08
sp|Q9RSJ	sp|Q9RSJ	124	45262000	53158000	1.43E+08	75894000	1.3E+08	80391000
tr|Q9RZC	tr|Q9RZC	1906	1.65E+08	1.05E+08	2.92E+08	7.73E+08	2.54E+08	3.15E+08
sp|Q9RU2	sp|Q9RU2	209	34863000		57950000	4.82E+08	5.38E+08	4.72E+08
sp|Q9RTL	sp|Q9RTL	194	885630					
sp|Q9RUP	sp|Q9RUP	246	52853000	47814000	53469000	63718000	54447000	67753000
tr|Q9RRR	tr|Q9RRR	568	1.47E+08	1.53E+08	1.36E+08	3.29E+08	2.89E+08	2.39E+08
tr|Q9RYH	tr|Q9RYH	1756				22902000	20166000	
tr|Q9RZI1	tr|Q9RZI1	1935						

sp|Q9RWH	sp|Q9RWH	330				15703000	16380000	13940000
tr|Q9RXZ4	tr|Q9RXZ4	1670	70878000	72681000	82488000			
tr|Q9RT53	tr|Q9RT53	821		34730000	32689000	54372000	53579000	63284000
sp|Q9RT4	sp|Q9RT4	167	17363000	16668000	15281000		13593000	18641000
tr|Q9RTU7	tr|Q9RTU7	935				16990000	15039000	
tr|Q9RVS2	tr|Q9RVS2	1288	1.56E+08	1.72E+08		5.04E+08	4.61E+08	3.9E+08
tr|Q9RVS2	tr|Q9RVS2	1288	96397000	1.56E+08	2.61E+08	2.77E+08	2.64E+08	2.27E+08
sp|Q9RVP	sp|Q9RVP	294	1.17E+08	3.09E+08	2.29E+08	2.18E+08	3.81E+08	2.5E+08
tr|Q9RVT4	tr|Q9RVT4	1295						
tr|Q9RVT4	tr|Q9RVT4	1295	2.64E+08	9995400	1.83E+08	2.58E+08	2.79E+08	2.23E+08
tr|Q9RST2	tr|Q9RST2	758	33498000	42978000				25504000
tr|Q9RST7	tr|Q9RST7	760	2.68E+08	3.26E+08	2.9E+08	1.14E+08	84770000	88859000
tr|Q9RSU7	tr|Q9RSU7	767	1.22E+08	71568000	71283000			3.78E+08
tr|Q9RUR4	tr|Q9RUR4	1107						
tr|Q9RZC7	tr|Q9RZC7	1906			74453000	23877000	42415000	32956000
tr|Q9RXK9	tr|Q9RXK9	1608	2.72E+08	3.15E+08	2.61E+08	2.42E+08	1.88E+08	2.32E+08
tr|Q9RZ85	tr|Q9RZ85	1877					5.57E+08	
tr|Q9RRP8	tr|Q9RRP8	560						
tr|Q9RTI3	tr|Q9RTI3	879	22248000	23502000	18462000	40918000	31507000	39821000
tr|Q9RWV	tr|Q9RWV	1484					8783200	
tr|Q9RZA0	tr|Q9RZA0	1887					14723000	21187000
tr|Q9RUH	tr|Q9RUH	1060	95951000	1.14E+08	1.04E+08	4.39E+08	3.61E+08	3.54E+08
tr|Q9RU42	tr|Q9RU42	989	33158000	27960000	31000000	20585000	14831000	
tr|Q9RUP1	tr|Q9RUP1	1099	1.35E+08	1.45E+08	2.37E+08	1.88E+08	2.28E+08	2.56E+08
tr|Q9RXL9	tr|Q9RXL9	1613	1.11E+08		1.09E+08			
tr|Q9RTG7	tr|Q9RTG7	873	6.84E+08		6.2E+08	5.57E+08		6.5E+08
tr|Q9RTR4	tr|Q9RTR4	923	7.94E+08	7.29E+08	6.12E+08	4.95E+08	4.43E+08	4.87E+08
sp|Q9RW0	sp|Q9RW0	322						
sp|Q5933	sp|Q5933	63	41970000	35180000	21213000	13916000	19092000	46276000
tr|Q9RYY7	tr|Q9RYY7	1837						
tr|Q9RXE6	tr|Q9RXE6	1577		20072000				
tr|Q9RUD	tr|Q9RUD	1040	34879000	25764000		52379000		65840000
tr|Q9RUR6	tr|Q9RUR6	1108	91042000	1.35E+08	1.81E+08	79949000	77988000	89332000
tr|Q9RUK8	tr|Q9RUK8	1080	2.16E+08	1.84E+08	1.63E+08			91096000
tr|Q9RS40	tr|Q9RS40	640	3.47E+08	3.68E+08	2.95E+08	4.83E+08	3.34E+08	3.84E+08
tr|Q9RY21	tr|Q9RY21	1682	55253000	83839000	1.41E+08			
tr|Q9RWB	tr|Q9RWB	1386	1.18E+09	1.17E+09	1.1E+09	1.19E+09	1.05E+09	9.96E+08
tr|Q9RYY8	tr|Q9RYY8	1838						
tr|Q9RZQ	tr|Q9RZQ	1957						
tr|Q9RUL2	tr|Q9RUL2	1084	96967000	90462000	67060000			
tr|Q9RU01	tr|Q9RU01	969						
tr|Q9RUC	tr|Q9RUC	1034				4646000		
tr|Q9RXF0	tr|Q9RXF0	1581	1.57E+09	1.35E+09	1.87E+09	7.12E+08	6.66E+08	6.46E+08
tr|Q9RXR9	tr|Q9RXR9	1636	3.2E+09	4.11E+09	3.87E+09	1.62E+09	1.79E+09	1.95E+09
tr|Q9RXL9	tr|Q9RXL9	1613						23882000
tr|Q9RU62	tr|Q9RU62	1004						
tr|Q9RVQ	tr|Q9RVQ	1274	8378800		54624000	18996000	32802000	38972000
tr|Q9RW5	tr|Q9RW5	1357	1.69E+08	2E+08	1.37E+08	2.73E+08	2.37E+08	2.54E+08
tr|Q9RW8	tr|Q9RW8	1367			26207000			
tr|Q9RVJ0	tr|Q9RVJ0	1238		18820000	6.47E+08	40474000		35940000
sp|Q9RSJ5	sp|Q9RSJ5	123	10513000	2766300	2890800			

tr|Q9RWT	tr|Q9RWT	1468						
tr|Q9RYT9	tr|Q9RYT9	1817	36393000	58442000	51601000	86439000	1.67E+08	69889000
tr|Q9RYT9	tr|Q9RYT9	1817			86601000			
tr|Q9RUB	tr|Q9RUB	1028			21969000	14621000	23937000	18825000
tr|Q9RXS7	tr|Q9RXS7	1640			10064000	15409000	17030000	14614000
tr|Q9RYA6	tr|Q9RYA6	1720	17697000		35671000	18281000		23712000
tr|Q9RZ07	tr|Q9RZ07	1845	6.76E+09	5.99E+09	4.94E+09	4.26E+09	3.57E+09	4.42E+09
tr|Q9RZ07	tr|Q9RZ07	1845		38270000	49798000			
sp|Q9RXI7	sp|Q9RXI7	373						
sp|Q9RXI7	sp|Q9RXI7	373	24225000	13198000	17129000	35980000	25417000	29781000
sp|Q9RXI7	sp|Q9RXI7	373		27676000	35772000	45426000	59075000	75259000
tr|Q9RUS6	tr|Q9RUS6	1113	1.03E+08	68325000	79514000	42140000	37332000	75645000
sp|Q9RVV	sp|Q9RVV	300	2.91E+09	2.55E+09	2.23E+09	1.95E+09	5.37E+08	1.58E+09
tr|Q9RSZ9	tr|Q9RSZ9	791	7140400	3190600	3109400	2801700	4187900	3196200
tr|Q9RSZ9	tr|Q9RSZ9	791	32029000	82415000	1.48E+08	20472000	24421000	21926000
sp|Q9RSR	sp|Q9RSR	144	1.37E+08					
sp|Q9RW1	sp|Q9RW1	306	1.71E+08	1.37E+08	1.23E+08	92805000	1.04E+08	88893000
tr|Q9RWM	tr|Q9RWM	1438	3.67E+08	2.7E+08	1.99E+08	1.5E+08	1.4E+08	1.56E+08
tr|Q9RZ10	tr|Q9RZ10	1848	51791000	44292000	66040000	69287000	59316000	75031000
sp|Q9RWE	sp|Q9RWE	321	5.31E+08	4.91E+08	3.89E+08	4.38E+08	2.92E+08	2.22E+08
tr|Q9RWC	tr|Q9RWC	1393		19668000	26541000		23408000	23199000
tr|Q9RUZ9	tr|Q9RUZ9	1150	73687000	51396000	91509000		22053000	24268000
tr|Q9RZH5	tr|Q9RZH5	1930	45508000				27989000	
tr|Q9RUF9	tr|Q9RUF9	1050	37601000	28598000	33205000	10840000		10053000
sp|Q9RW9	sp|Q9RW9	315		16144000	22561000			
tr|Q9RY83	tr|Q9RY83	1709	48057000	29044000	28261000	91936000	95608000	64320000
tr|Q9RT87	tr|Q9RT87	837	9.67E+08	8.43E+08	7.07E+08	1.06E+09	9.72E+08	9.84E+08
tr|Q9RT87	tr|Q9RT87	837		22577000	40525000		27580000	18524000
tr|Q9RRI4	tr|Q9RRI4	532						
sp|Q9RS1	sp|Q9RS1	107		16697000	38968000	43234000	59877000	61766000
tr|Q9RTY7	tr|Q9RTY7	959	1.48E+08	2.35E+08	1.55E+08	1.64E+08	1.32E+08	1.33E+08
tr|Q9RWP	tr|Q9RWP	1449			7240100	8866900	6961200	8954400
tr|Q9RYV4	tr|Q9RYV4	1822	3.58E+08	5.06E+08	4.73E+08	1.38E+08	1.51E+08	1.61E+08
sp|Q9RW0	sp|Q9RW0	342	14594000	63875000	2.07E+08	1.5E+08	2.62E+08	2.02E+08
tr|Q9RSH0	tr|Q9RSH0	716	1.12E+09	6.97E+08	7.78E+08	2.28E+08		2.55E+08
tr|Q9RYI7	tr|Q9RYI7	1763	26189000	55563000	83222000	27340000	26519000	
tr|Q9RYN3	tr|Q9RYN3	1777						
tr|Q9RZC5	tr|Q9RZC5	1904	4.57E+08	3.92E+08	3.01E+08	7.69E+08	7.3E+08	8.35E+08
tr|Q9RZC5	tr|Q9RZC5	1904	61898000	1.77E+08	1.56E+08	59700000	1.16E+08	1.3E+08
tr|Q9RX52	tr|Q9RX52	1528			11701000			
tr|Q9RWV	tr|Q9RWV	1482			70705000			
tr|Q9RZQ	tr|Q9RZQ	1958				25962000		
tr|Q9RV45	tr|Q9RV45	1172				1448900	6325100	3889700
tr|Q9RV45	tr|Q9RV45	1172			22331000	24891000		29687000
sp|P56861	sp|P56861	54	82895000	1.42E+08	72854000	1.43E+08	1.59E+08	1.5E+08
tr|Q9RZV4	tr|Q9RZV4	1984						
sp|Q9RRB	sp|Q9RRB	76		29974000	59849000	13178000	13715000	25638000
tr|Q9RS17	tr|Q9RS17	630	4.92E+09	4.84E+09	3.95E+09	3.44E+09	2.78E+09	3.21E+09
tr|Q9RY35	tr|Q9RY35	1688	9.04E+08	9.43E+08	8.59E+08	3.27E+08	2.99E+08	3.42E+08
sp|Q9RUP	sp|Q9RUP	243	2.54E+08	4.7E+08	1.73E+08	3.52E+08	2.63E+08	
tr|Q9RRP2	tr|Q9RRP2	556	1010900	1027100	1178700		764670	

sp|Q9RY2	sp|Q9RY2	411	1.45E+09	6.48E+08	1.2E+09	1.18E+09	1.07E+09	1E+09
sp|Q9RY2	sp|Q9RY2	411	1.45E+08	2.15E+08	2.8E+08	78255000	1.02E+08	1.21E+08
tr|Q9RVX7	tr|Q9RVX7	1318	81888000	87182000	80931000	1.5E+08	1E+08	1.33E+08
tr|Q9RUX5	tr|Q9RUX5	1134					25889000	40880000
sp|P42443	sp|P42443	49						
tr|Q9RSS3	tr|Q9RSS3	756	32188000	52693000	27526000	29033000	32387000	27112000
tr|Q9RU17	tr|Q9RU17	976	12528000	8969800			8801800	
sp|Q9RW0	sp|Q9RW0	327	1.43E+09	1.62E+09	1.29E+09	1.72E+09	1.11E+09	1.26E+09
tr|Q9RW5	tr|Q9RW5	1357	2.85E+08	5.43E+08	4.29E+08	2.98E+08	4.36E+08	3.33E+08
sp|Q9RTN	sp|Q9RTN	196	1.58E+09	2.09E+09	1.82E+09	2.53E+09	3.46E+09	2.41E+09
sp|Q9RTN	sp|Q9RTN	196	9741800	48263000	60649000	10706000		
tr|Q9RSD6	tr|Q9RSD6	697						
tr|Q9RXX3	tr|Q9RXX3	1661						
sp|Q9RZA	sp|Q9RZA	454	21252000	18583000	11544000	20166000		18658000
tr|Q9RXH8	tr|Q9RXH8	1599	26631000	13651000	17056000		3374200	
sp|Q9RUA	sp|Q9RUA	224	5.84E+08	6.62E+08	5.46E+08	5.91E+08	5.77E+08	5.66E+08
tr|Q9RVP5	tr|Q9RVP5	1269						
tr|Q9RXI3	tr|Q9RXI3	1602						
sp|Q9RW0	sp|Q9RW0	342			13437000	1.66E+08	1.71E+08	1.58E+08
sp|Q9RS1	sp|Q9RS1	107	11406000		11092000	27814000	31638000	33386000
sp|Q9RVH	sp|Q9RVH	288	6944900	5516500	5160500	3373200		4900700
tr|Q9RVS2	tr|Q9RVS2	1288	1.51E+09	6.69E+08	8.44E+08	4.19E+08	4.92E+08	4.86E+08
sp|Q9RX5	sp|Q9RX5	360	41230000	30511000		45111000	33891000	76108000
sp|P56867	sp|P56867	56	2.73E+08	3.85E+08	5.36E+08	29321000	42967000	54244000
sp|Q9RY0	sp|Q9RY0	410			26214000	24969000	26514000	
tr|Q9RSD4	tr|Q9RSD4	696	70551000	54799000	59849000	1.33E+08	1.06E+08	92884000
tr|Q9RVA5	tr|Q9RVA5	1202	2.44E+08	2.9E+08	1.98E+08	3.84E+08	3.39E+08	3.06E+08
tr|Q9RVA5	tr|Q9RVA5	1202	3.04E+08	3.48E+08	5.2E+08	1.04E+08	1.19E+08	1.3E+08
tr|Q9RU08	tr|Q9RU08	973	2.21E+08	2.07E+08	2.89E+08	8.34E+08	8.86E+08	7.47E+08
tr|Q9RZV9	tr|Q9RZV9	1985		1.35E+08		1.72E+08	1.22E+08	1.39E+08
tr|Q9RWN	tr|Q9RWN	1443	21786000	31847000	43894000		35649000	
tr|Q9RYL0	tr|Q9RYL0	1767	1.24E+08	1.73E+08	3.53E+08	27647000	30775000	43346000
tr|Q9RX65	tr|Q9RX65	1538					20350000	
tr|Q9RV56	tr|Q9RV56	1180					19452000	
tr|Q9RU41	tr|Q9RU41	988						16327000
tr|Q9RUC	tr|Q9RUC	1033	88005000	1.38E+08	74749000	1.76E+08	1.63E+08	1.01E+08
tr|Q9RZ85	tr|Q9RZ85	1877	19522000	22156000	32565000			
tr|Q9RVT9	tr|Q9RVT9	1298						
tr|Q9RVQ	tr|Q9RVQ	1272	95308000	1.23E+08	2.75E+08	1.61E+08	1.43E+08	2.55E+08
tr|Q9RR62	tr|Q9RR62	467	10645000	4011300	4468400			
tr|Q9RYH1	tr|Q9RYH1	1754					12412000	
tr|Q9RXS7	tr|Q9RXS7	1640	65767000	52772000	83948000	62806000	34137000	89392000
sp|Q9RTI6	sp|Q9RTI6	188					26203000	
tr|Q9RUV4	tr|Q9RUV4	1125	83083000	23326000	98179000	1.42E+08	1.37E+08	2.07E+08
tr|Q9RVP1	tr|Q9RVP1	1267			21846000	23536000	22523000	26890000
tr|Q9RUR7	tr|Q9RUR7	1109	84680000	1.01E+08	88338000	1.95E+08	1.7E+08	1.78E+08
tr|Q9RUR7	tr|Q9RUR7	1109	2.26E+09	1.58E+09	1.95E+09	1.57E+09	1.15E+09	1.52E+09
sp|Q9RYB	sp|Q9RYB	427	2.28E+08	1.16E+08	1.9E+08			
tr|Q9RZP6	tr|Q9RZP6	1954		16046000	23803000			
tr|Q9RSC3	tr|Q9RSC3	688	7.17E+08	5.82E+08	5.12E+08	4.84E+08	5.09E+08	4.98E+08
sp|Q9RY2	sp|Q9RY2	411	2.14E+08	2.46E+08	1.87E+08	6.72E+08	5.32E+08	4.99E+08

tr|Q9RV31	tr|Q9RV31	1167	41381000	88673000	1.44E+08	92204000	1.81E+08	1.66E+08
tr|Q9RY67	tr|Q9RY67	1701	2.05E+08	1.04E+08	3.72E+08	1.91E+08	63722000	79263000
tr|Q9RTN8	tr|Q9RTN8	905	2.27E+08	2.07E+08	1.93E+08	62809000	71284000	
tr|Q9RTQ5	tr|Q9RTQ5	915		20083000	18185000	26748000	26339000	
tr|Q9RSZ5	tr|Q9RSZ5	790	89984000	74435000	79365000	8243100		61329000
sp|Q9RU8	sp|Q9RU8	216				1755300	2998000	2211400
sp|Q9RU8	sp|Q9RU8	216	1.32E+08	2.32E+08	4.02E+08	1.13E+08	1.37E+08	2.92E+08
sp|Q9RS1	sp|Q9RS1	106	8617800	3511800	3099600	2414400	4084200	3195600
sp|Q9RTG	sp|Q9RTG	184	28965000		26054000			
tr|Q9RZC7	tr|Q9RZC7	1906	1.27E+09	1.61E+09	1.37E+09	2.35E+09	1.93E+09	1.99E+09
tr|Q9RR85	tr|Q9RR85	476	23368000		34246000			
sp|Q9RW\	sp|Q9RW\	349	3.3E+08	3.04E+08	2.84E+08	4.78E+08	4.21E+08	4.4E+08
tr|Q9RTI9	tr|Q9RTI9	882						
tr|Q9RVA7	tr|Q9RVA7	1203						
tr|Q9RSQ2	tr|Q9RSQ2	747	11263000	9842200	8969600			8225900
tr|Q9RVQ	tr|Q9RVQ	1274	78512000	43071000	37905000	39485000	64915000	45345000
tr|Q9RRG4	tr|Q9RRG4	520	1.61E+08	1.35E+08	2.46E+08			68963000
tr|Q9RW1	tr|Q9RW1	1337				19341000	16249000	
tr|Q9RU01	tr|Q9RU01	969		15723000	16481000	34354000	62682000	52348000
tr|Q9RSD0	tr|Q9RSD0	692	1.47E+08	1.81E+08	1.68E+08	95489000	1.32E+08	71022000
tr|Q9RVZ5	tr|Q9RVZ5	1328	58084000	57578000	41370000	72582000	61689000	62271000
sp|Q9RYX	sp|Q9RYX	447	1.53E+08	1.8E+08	1.34E+08	2.17E+08	1.85E+08	1.79E+08
tr|Q9RS76	tr|Q9RS76	657	17944000	6039200	35721000	40174000	71525000	13985000
sp|Q9RY7	sp|Q9RY7	423	1.24E+08	89886000	77583000	84945000	90306000	81214000
tr|Q9RXI1	tr|Q9RXI1	1601	26453000	23455000		6541700	31566000	31394000
tr|Q9RZ56	tr|Q9RZ56	1864	7.58E+08	6.6E+08	6.95E+08	3.7E+08	5.18E+08	2.46E+08
tr|Q9RZ56	tr|Q9RZ56	1864		26568000	42885000		32291000	
tr|Q9RTE3	tr|Q9RTE3	862	971510		872360	1780100	1503900	1508900
tr|Q9RT26	tr|Q9RT26	804	67453000					
sp|Q9RY0	sp|Q9RY0	410						
tr|Q9RU73	tr|Q9RU73	1011					16702000	21157000
tr|Q9RYQ0	tr|Q9RYQ0	1788	4.36E+09	3.86E+09	3.21E+09	4.42E+09	4.68E+09	4.3E+09
tr|Q9RSU8	tr|Q9RSU8	768	1.23E+08	1.81E+08	1.39E+08	78643000	1.03E+08	10752000
sp|Q9RY2	sp|Q9RY2	411	26452000	14051000	13326000	18115000	19961000	15325000
tr|Q9RSH0	tr|Q9RSH0	716	1.76E+08	97891000	1.39E+08	59182000	62383000	61282000
tr|Q9RYG4	tr|Q9RYG4	1749	19671000	25973000	23375000	12687000	27457000	10042000
sp|Q9RVH	sp|Q9RVH	288	2.76E+08	2.2E+08	3.05E+08		1.26E+08	1.84E+08
tr|Q9RYS7	tr|Q9RYS7	1806	42296000		23973000			
tr|Q9RWH	tr|Q9RWH	1409	1873400	680380	634360		713400	699920
tr|Q9RXC5	tr|Q9RXC5	1570						
tr|Q9RV50	tr|Q9RV50	1176	3.41E+08	3.62E+08	2.9E+08	1.23E+08	1.17E+08	1.52E+08
sp|Q9RXJ2	sp|Q9RXJ2	377	1.55E+08	61655000	91162000	82459000	88703000	86633000
sp|Q9RVI3	sp|Q9RVI3	289						
tr|Q9RRA6	tr|Q9RRA6	489			36642000			59646000
tr|Q9RTM	tr|Q9RTM	899	3.54E+08	2.83E+08	3.03E+08	6.37E+08	5.43E+08	6.16E+08
tr|Q9RUU	tr|Q9RUU	1124	4.1E+08	3.96E+08	3.9E+08	4.03E+08	3.91E+08	3.62E+08
tr|Q9RWV	tr|Q9RWV	1482	22424000	53424000	34104000	3.25E+08	1.79E+08	2.37E+08
sp|Q9RUT	sp|Q9RUT	251						
tr|Q9RWC	tr|Q9RWC	1393				21438000	22163000	17508000
sp|Q9RU8	sp|Q9RU8	218	40477000	48077000	2.34E+08		26721000	
tr|Q9RXR9	tr|Q9RXR9	1636	15190000		11261000	13910000	36195000	15148000

tr|Q9RVK8	tr|Q9RVK8	1248	35568000	61809000	36508000			
tr|Q9RT77	tr|Q9RT77	832	24471000					
tr|Q9RU71	tr|Q9RU71	1010	59292000	44988000	39473000	25537000	23215000	24733000
tr|Q9RY55	tr|Q9RY55	1697	44994000	39746000	43174000	48104000	24773000	24465000
tr|Q9RTX7	tr|Q9RTX7	952						
tr|Q9RVI8	tr|Q9RVI8	1237		56181000	56173000			
tr|Q9RW1	tr|Q9RW1	1339	28998000		28868000			
tr|Q9RZT9	tr|Q9RZT9	1975				15363000	7554400	4973000
tr|Q9RV70	tr|Q9RV70	1186	20908000	20952000	20069000	51630000	49657000	43593000
tr|Q9RSI7	tr|Q9RSI7	724				47229000	52400000	30313000
tr|Q9RZH8	tr|Q9RZH8	1932	1.23E+08	1.08E+08	88432000	75587000	1.02E+08	79608000
sp|Q9RW9	sp|Q9RW9	345				35585000	28712000	
tr|Q9RT53	tr|Q9RT53	821		53317000	40255000	92946000	84012000	73431000
tr|Q9RT53	tr|Q9RT53	821	20776000	35787000	33902000	37330000	55999000	58129000
tr|Q9RXV1	tr|Q9RXV1	1650			7864500			13019000
sp|Q9RY4	sp|Q9RY4	416		51722000	61598000	81156000		76351000
sp|Q9RY4	sp|Q9RY4	416				48175000		
tr|Q9RSR7	tr|Q9RSR7	752		2.46E+08	2.01E+08	6.08E+08	5.66E+08	6E+08
tr|Q9RSN4	tr|Q9RSN4	741	1.4E+08	1.29E+08	1.04E+08	67557000	48039000	52889000
tr|Q9RYC0	tr|Q9RYC0	1723	83437000	87236000	96693000	97969000		69210000
tr|Q9RZA9	tr|Q9RZA9	1893	55355000	53336000	52177000			
tr|Q9RST1	tr|Q9RST1	757	54623000	49624000	49023000		19840000	
tr|Q9RVQ	tr|Q9RVQ	1274	9.72E+08	7.78E+08	8.06E+08	1.92E+09	1.69E+09	1.69E+09
tr|Q9RRL0	tr|Q9RRL0	544						
sp|Q9RXK	sp|Q9RXK	389	3.15E+08	2.32E+08	2.67E+08			
tr|Q9RY74	tr|Q9RY74	1704	46476000	76082000	73856000	53284000	41147000	41299000
tr|Q9RUY2	tr|Q9RUY2	1139					1311500	
tr|Q9RW8	tr|Q9RW8	1367	79956000	59196000				
sp|Q9RU8	sp|Q9RU8	216					50238000	
sp|Q9RUS	sp|Q9RUS	250			46188000	42993000	86795000	74633000
tr|Q9RZU4	tr|Q9RZU4	1977						
tr|Q9RS47	tr|Q9RS47	643		2.6E+08	3.99E+08	3.31E+08	2.79E+08	2.44E+08
tr|Q9RX19	tr|Q9RX19	1514	72351000			1.53E+08	1.37E+08	
tr|Q9RYD9	tr|Q9RYD9	1735						
tr|Q9RVQ	tr|Q9RVQ	1272				3807200	10334000	4182700
tr|Q9RSB8	tr|Q9RSB8	685				23870000	22109000	29344000
tr|Q9RYH9	tr|Q9RYH9	1758				24326000	29526000	26202000
tr|Q9RZN4	tr|Q9RZN4	1951	24750000	28316000	30237000	47934000	72129000	
tr|Q9RVF7	tr|Q9RVF7	1224				30238000	50570000	37409000
tr|Q9RUB	tr|Q9RUB	1028				7090300	11460000	10108000
sp|Q9RSJ8	sp|Q9RSJ8	126			16993000	1.13E+08	1.08E+08	1.08E+08
sp|Q9RRB	sp|Q9RRB	76				20218000		23992000
tr|Q9RU07	tr|Q9RU07	972				84114000	1.56E+08	1.03E+08
tr|Q9RY75	tr|Q9RY75	1705				3425400	4005200	4335000
tr|Q9RUF8	tr|Q9RUF8	1049				76198000	1.01E+08	88729000
tr|Q9RSL7	tr|Q9RSL7	733						
tr|Q9RSQ4	tr|Q9RSQ4	748				1.21E+08	1.22E+08	1.09E+08
sp|Q9R34	sp|Q9R34	64				24334000	73512000	96918000
tr|Q9RVW	tr|Q9RVW	1310						
sp|Q9RT2	sp|Q9RT2	162						
tr|Q9RXM	tr|Q9RXM	1615						

tr|Q9RRV1	tr|Q9RRV1	588				19425000		
tr|Q9RUC	tr|Q9RUC	1034						
sp|Q9RZ8	sp|Q9RZ8	453						
sp|Q9RXK	sp|Q9RXK	387				22052000	22351000	20078000
sp|Q9RVQ	sp|Q9RVQ	295						6381200
tr|Q9RTC1	tr|Q9RTC1	848			13666000	36260000	41527000	
tr|Q9RRX6	tr|Q9RRX6	604				26874000	27081000	33616000
tr|Q9RXS7	tr|Q9RXS7	1640			33710000	17772000		
tr|Q9RSD1	tr|Q9RSD1	693						
sp|Q9RVE	sp|Q9RVE	283			19312000			
sp|Q9RT0	sp|Q9RT0	161			52494000			
tr|Q9RSL6	tr|Q9RSL6	732	3.75E+08	5.96E+08	4.49E+08	4.06E+08	5.85E+08	5.44E+08
tr|Q9RYY7	tr|Q9RYY7	1837	45262000			34010000	21376000	
tr|Q9RTC8	tr|Q9RTC8	854	33921000	55016000	35771000	88384000	68882000	67186000
tr|Q9RUT5	tr|Q9RUT5	1119						
tr|Q9RYY7	tr|Q9RYY7	1837	30012000		23114000			
tr|Q9RW4	tr|Q9RW4	1351	1.9E+08	1.42E+08	1.08E+08	56333000	40995000	49164000
tr|Q9RTY0	tr|Q9RTY0	955	2322500		691980			
tr|Q9RT75	tr|Q9RT75	831				94724000		1.09E+08
tr|Q9RTN3	tr|Q9RTN3	904	1.65E+09	1.97E+09	1.65E+09	3.68E+09	3.7E+09	4.15E+09
sp|Q9RUE	sp|Q9RUE	226	3519000	1319100	989220	918020	1325500	1113200
sp|Q9RUC	sp|Q9RUC	247	2.39E+08	3.18E+08	1.97E+08	3.24E+08	2.66E+08	3.14E+08
sp|Q9RW0	sp|Q9RW0	327	5.03E+08	4.85E+08	4.25E+08	4.33E+08	4.23E+08	4.45E+08
tr|Q9RRG1	tr|Q9RRG1	518	7.7E+08	7.94E+08	7.59E+08	6.85E+08	7.71E+08	7.95E+08
sp|Q9RW2	sp|Q9RW2	307						
tr|Q9RYK2	tr|Q9RYK2	1764						
tr|Q9RT11	tr|Q9RT11	799	25441000	55881000	45803000			
tr|Q9RY75	tr|Q9RY75	1705		58119000	91017000	1.72E+08	1.05E+08	1.5E+08
tr|Q9RY75	tr|Q9RY75	1705	23806000	16770000	30299000			
tr|Q9RY75	tr|Q9RY75	1705	91955000	1.04E+08	78532000	17811000		42929000
tr|Q9RRN	tr|Q9RRN	551						
tr|Q9RSD0	tr|Q9RSD0	692	67008000	57868000	1.3E+08		16650000	
tr|Q9RYA9	tr|Q9RYA9	1721	2.52E+08	4.12E+08	2.22E+08	2.6E+08	2.32E+08	2.69E+08
sp|Q9RXK	sp|Q9RXK	389	8.23E+08	9.71E+08	4.85E+08	3.16E+08	7.09E+08	3.56E+08
tr|Q9RVA5	tr|Q9RVA5	1202	26976000	9798300	9253400		3765400	4712100
tr|Q9RVA5	tr|Q9RVA5	1202	43263000	1.71E+08	99967000			
tr|Q9RZV4	tr|Q9RZV4	1984			35564000			
sp|Q9RRY	sp|Q9RRY	103	7301300		2310900	1974100		
tr|Q9RWX	tr|Q9RWX	1496	5219400		8153800			
tr|Q9RXB6	tr|Q9RXB6	1565			9538900			
tr|Q9RUX0	tr|Q9RUX0	1129	1.02E+08	89179000	85311000	87266000	1.01E+08	92619000
tr|Q9RRK9	tr|Q9RRK9	543	35864000	33148000	79782000			
tr|Q9RU17	tr|Q9RU17	976	34495000	31237000				28067000
tr|Q9RY86	tr|Q9RY86	1710	77470000	59147000	52038000	57313000	71790000	63112000
tr|Q9RRA2	tr|Q9RRA2	485	21787000	29699000		59009000	63766000	46803000
tr|Q9RTQ1	tr|Q9RTQ1	913	1.64E+09	1.33E+09	1.12E+09	1.68E+09	1.26E+09	1.81E+09
tr|Q9RYB8	tr|Q9RYB8	1722	1.72E+08	2.45E+08	2.21E+08	1.04E+08	1.33E+08	1.68E+08
tr|Q9RTZ7	tr|Q9RTZ7	965			25608000			
tr|Q9RYP4	tr|Q9RYP4	1783			31711000			
tr|Q9RX13	tr|Q9RX13	1510		65735000	31540000	21372000		26377000
sp|Q9RW0	sp|Q9RW0	304						

tr|Q9RYX6	tr|Q9RYX6	1835	3.83E+08	3.15E+08	2.55E+08	2.56E+08	1.89E+08	2.14E+08
sp|Q9RUE	sp|Q9RUE	228			19925000			
tr|Q9RXB3	tr|Q9RXB3	1564			2244300			
sp|Q9RTR	sp|Q9RTR	198						
tr|Q9RWS	tr|Q9RWS	1462				12984000		
tr|Q9RW8	tr|Q9RW8	1370	96663000	76471000	82559000	56540000	60061000	59686000
sp|Q9RWI	sp|Q9RWI	335						
tr|Q9RVW	tr|Q9RVW	1311	30102000					17907000
tr|Q9RYP9	tr|Q9RYP9	1787	34898000		25375000			
tr|Q9RWB	tr|Q9RWB	1387	21519000	34525000	13406000	30752000	28284000	19318000
tr|Q9RUM	tr|Q9RUM	1091		76237000				
tr|Q9RSU0	tr|Q9RSU0	762	2.48E+08	8.92E+08	2.61E+08	9.18E+08	7.54E+08	6.13E+08
tr|Q9RTH0	tr|Q9RTH0	875			25012000			
tr|Q9RY75	tr|Q9RY75	1705	25780000	26251000	19605000	33509000	21933000	31807000
sp|Q9RRH	sp|Q9RRH	85	22087000			22217000		
tr|Q9RXN3	tr|Q9RXN3	1620						
tr|Q9RY75	tr|Q9RY75	1705	16062000				13381000	17190000
sp|P5686	sp|P5686	55	24430000	44537000	62120000	1.2E+08	1.61E+08	1.68E+08
tr|Q9RTX5	tr|Q9RTX5	950	28320000	40109000	63455000			
tr|Q9RZD6	tr|Q9RZD6	1910	1.19E+08	1.1E+08	86138000			
tr|Q9RT50	tr|Q9RT50	819					13919000	
tr|Q9RTY1	tr|Q9RTY1	956	1.24E+08	1.2E+08	1.1E+08	49765000	59468000	57840000
tr|Q9RUR4	tr|Q9RUR4	1107	39351000		30751000			
sp|Q9RSK	sp|Q9RSK	130						
sp|Q9RSK	sp|Q9RSK	130	17503000	40692000	77369000	22603000	30689000	30627000
sp|Q9RYM	sp|Q9RYM	439	1.3E+08	1.59E+08	2.69E+08			
tr|Q9RSM	tr|Q9RSM	737	12498000	14022000	24324000	10255000	13520000	26168000
tr|Q9RTQ5	tr|Q9RTQ5	915	5867800	5439300	16766000	48942000	26803000	26347000
tr|Q9RU69	tr|Q9RU69	1008						
tr|Q9RS70	tr|Q9RS70	654						
tr|Q9RWW	tr|Q9RWW	1492						
tr|Q9RYG1	tr|Q9RYG1	1747						
sp|Q9RXK	sp|Q9RXK	389				2194000		
tr|Q9RS71	tr|Q9RS71	655	4595500	1245900	1198800	1098800	2198400	1601500
tr|Q9RVK5	tr|Q9RVK5	1245	11401000	6549800	5974000	4641100	6063100	5694000
sp|P56867	sp|P56867	56	3.76E+08	2.97E+08	3.03E+08	7.13E+08	8.52E+08	7.31E+08
sp|Q9RSP	sp|Q9RSP	139			22936000			
tr|Q9RVJ3	tr|Q9RVJ3	1240		2196700	4500300			3940800
sp|Q9RXK	sp|Q9RXK	385	4.11E+09	3.05E+09	2.7E+09	2.42E+08	2.49E+08	2.54E+08
sp|O8302	sp|O8302	47	14271000	32524000	44653000	9751400	18552000	27721000
tr|Q9RU91	tr|Q9RU91	1015	47867000	49028000	50895000	47357000	54709000	78797000
sp|Q9RT4	sp|Q9RT4	167	8653200		7187100	13920000	8992600	8413400
tr|Q9RW6	tr|Q9RW6	1360	17002000			73584000	52853000	37005000
tr|Q9RVS0	tr|Q9RVS0	1286	1.79E+09	1.3E+09	1.28E+09	1.26E+09	1.4E+09	1.21E+09
tr|Q9RUL5	tr|Q9RUL5	1086	1.35E+08	1.11E+08	93097000	1.02E+08	95528000	98903000
tr|Q9RUX2	tr|Q9RUX2	1131	22828000		15076000		18479000	24708000
sp|Q9RV5	sp|Q9RV5	265	35252000	39352000	31280000	23674000		32243000
tr|Q9RYS7	tr|Q9RYS7	1806	84747000	1.05E+08	1.04E+08		80161000	58329000
tr|Q9RWS	tr|Q9RWS	1464						
sp|Q9RRY	sp|Q9RRY	103						
tr|Q9RUL5	tr|Q9RUL5	1086			64420000		66979000	55798000

tr|Q9RV59	tr|Q9RV59	1182	6309900			10070000	10755000	
tr|Q9RV86	tr|Q9RV86	1193						
tr|Q9RX36	tr|Q9RX36	1520	49259000	38796000	33186000		43108000	
tr|Q9RRU1	tr|Q9RRU1	584				57556000	65734000	
sp|Q9RVY	sp|Q9RVY	303	4201300	2206800	2098400	1958300	2724900	3649600
tr|Q9RSK2	tr|Q9RSK2	730	70470000	25088000	79387000	1.05E+08	98349000	50750000
tr|Q9RV92	tr|Q9RV92	1196	53608000	48507000	45609000			
tr|Q9RST2	tr|Q9RST2	758	52057000	39144000	43903000			
tr|Q9RRB3	tr|Q9RRB3	494			28088000			
tr|Q9RZ22	tr|Q9RZ22	1854	26973000		25274000			
sp|Q9RVT	sp|Q9RVT	298	26027000	39534000	32768000		28243000	16122000
tr|Q9RUJ3	tr|Q9RUJ3	1069			16301000	52191000	46813000	43285000
tr|Q9RVJ0	tr|Q9RVJ0	1238	3.55E+09	4.41E+09	3.4E+09	4.31E+09	4.75E+09	3.99E+09
tr|Q9RS21	tr|Q9RS21	632				12141000	13677000	17625000
tr|Q9RWP	tr|Q9RWP	1449	31271000	3.29E+08	2.72E+08	1.86E+08	2.02E+08	2.37E+08
tr|Q9RWG	tr|Q9RWG	1407	4.13E+08	3.25E+08	3.23E+08	8.77E+08	8.62E+08	7.93E+08
tr|Q9RU76	tr|Q9RU76	1012	1.17E+08	1.8E+08	1.53E+08	1.93E+08	1.41E+08	
tr|Q9RS33	tr|Q9RS33	639	2.27E+08	2.26E+08	2.01E+08	4.32E+08	4.37E+08	3.74E+08
tr|Q9RYC4	tr|Q9RYC4	1725	4783100	5650700				
sp|Q9RXK	sp|Q9RXK	390	1.55E+09	1.97E+09	1.87E+09	1.29E+09	1.67E+09	1.2E+09
sp|Q9RXK	sp|Q9RXK	390	40945000	47792000	99547000			
tr|Q9RY20	tr|Q9RY20	1681	5.74E+08	5.98E+08	4.82E+08	7.17E+08	6.63E+08	7.15E+08
tr|Q9RW8	tr|Q9RW8	1367						
tr|Q9RSS3	tr|Q9RSS3	756	30861000	1.21E+08	1.08E+08	1.83E+08	1.51E+08	1.53E+08
tr|Q9RW9	tr|Q9RW9	1377	3.53E+08	2.88E+08	2.65E+08	3.16E+08	3.05E+08	2.65E+08
tr|Q9RWN	tr|Q9RWN	1444	29828000	49411000	40226000	59645000	60833000	38882000
tr|Q9RZT3	tr|Q9RZT3	1970	46577000	38441000	37192000		4543300	
tr|Q9RRS5	tr|Q9RRS5	576				11951000	11337000	14952000
tr|Q9RU34	tr|Q9RU34	983	32785000	7555900	1.39E+08	48512000	36912000	1.38E+08
tr|Q9RZI0	tr|Q9RZI0	1934						
tr|Q9RXS1	tr|Q9RXS1	1638	47008000		94142000			
tr|Q9RZT6	tr|Q9RZT6	1973	1.63E+08	2.43E+08	1.03E+08	2.23E+08	2.04E+08	1.61E+08
sp|Q9RRB	sp|Q9RRB	76			8815200			
sp|Q9RRB	sp|Q9RRB	76	6.22E+08	4.5E+08	5.6E+08	1.62E+08	2.47E+08	2.69E+08
tr|Q9RRX1	tr|Q9RRX1	601	1.68E+09	1.37E+09	2.76E+09	2.63E+08	1.76E+09	1.6E+09
tr|Q9RRW	tr|Q9RRW	595				97805000	91282000	
tr|Q9RWF	tr|Q9RWF	1405						13086000
sp|Q9RYR	sp|Q9RYR	442			31767000		20284000	17126000
tr|Q9RTY8	tr|Q9RTY8	960						
tr|Q9RVU0	tr|Q9RVU0	1299	2.3E+08	2.29E+08	1.7E+08	1.62E+08	1.79E+08	1.67E+08
tr|Q9RTM	tr|Q9RTM	898	18005000		25974000			
tr|Q9RY30	tr|Q9RY30	1685			21942000			
tr|Q9RWM	tr|Q9RWM	1440	44836000	26694000	27495000			
sp|Q9RV2	sp|Q9RV2	263	1.33E+08	87249000	1.14E+08	1.58E+08	1.42E+08	1.38E+08
sp|Q9RW	sp|Q9RW	335	19844000	14039000	17269000	16338000		7381200
tr|Q9RVN	tr|Q9RVN	1259	61843000	62814000		2E+08	1.2E+08	2.79E+08
sp|Q9RR9	sp|Q9RR9	73						
sp|Q9RXU	sp|Q9RXU	402	29591000	2.56E+08	59496000	1.21E+08	94190000	73386000
tr|Q9RU77	tr|Q9RU77	1013	6.74E+08	5.55E+08	5.25E+08	2.38E+08	2.73E+08	2.66E+08
tr|Q9RTE5	tr|Q9RTE5	863		20020000	29509000		26548000	21484000
tr|Q9RXU2	tr|Q9RXU2	1645	30429000		22920000			

tr|Q9RWX	tr|Q9RWX	1498	38613000	30224000	22871000			40386000
tr|Q9RTI9	tr|Q9RTI9	882	66062000	72384000	55677000			
tr|Q9RZ09	tr|Q9RZ09	1847	1.3E+08	97504000	1.25E+08	1.62E+08		1.52E+08
tr|Q9RZ09	tr|Q9RZ09	1847	30464000	50275000	67926000			
tr|Q9RS01	tr|Q9RS01	619						
tr|Q9RWU	tr|Q9RWU	1480	3.22E+08	3.03E+08	3.23E+08	48850000	1.3E+08	54222000
tr|Q9RRV3	tr|Q9RRV3	590	3.58E+08	3.03E+08	2.56E+08	8.76E+08	6.05E+08	7.77E+08
tr|Q9RRG5	tr|Q9RRG5	521	3.03E+08	2.6E+08	2.07E+08	1.14E+08	1.06E+08	1.44E+08
tr|Q9RRG5	tr|Q9RRG5	521			50176000		22261000	23410000
sp|Q9RUV	sp|Q9RUV	258						
sp|Q9RUE	sp|Q9RUE	229	3.91E+09	4.08E+09	3.78E+09	1.87E+08	3.88E+08	2.25E+08
sp|P52027	sp|P52027	51	1.26E+08	1.28E+08	1.36E+08	1.15E+08	1.58E+08	1.04E+08
tr|Q9RRV4	tr|Q9RRV4	591	1.46E+08	1.08E+08	2.22E+08			
tr|Q9RXN3	tr|Q9RXN3	1620				1.03E+08		1.05E+08
tr|Q9RXN3	tr|Q9RXN3	1620	1.55E+08	1.31E+08	1.92E+08	66162000	92452000	1.22E+08
tr|Q9RXB2	tr|Q9RXB2	1563	57709000	44734000	37125000	41596000	43465000	41107000
tr|Q9RYE4	tr|Q9RYE4	1738	67141000	50527000	74033000		19613000	
tr|Q9RZT0	tr|Q9RZT0	1968	34041000	33097000	43827000	1.27E+08	1.28E+08	92060000
tr|Q9RYS2	tr|Q9RYS2	1802	1.02E+08	91723000	99180000	41020000	42142000	35526000
tr|Q9RRG9	tr|Q9RRG9	523						
tr|Q9RTX0	tr|Q9RTX0	947	56236000	62149000	52840000	13383000	49997000	11278000
sp|Q9RSQ	sp|Q9RSQ	141			11142000			
tr|Q9RS41	tr|Q9RS41	641	3.2E+08	3.26E+08	2.45E+08	6.09E+08	4.46E+08	4.98E+08
tr|Q9RST7	tr|Q9RST7	760	56514000	46058000	38981000			
tr|Q9RU16	tr|Q9RU16	975	2.97E+08	3.16E+08	2.28E+08	2.85E+08	3.09E+08	2.89E+08
tr|Q9RUY8	tr|Q9RUY8	1143			11233000			
tr|Q9RSF6	tr|Q9RSF6	709	43871000	26260000	17644000			
tr|Q9RWE	tr|Q9RWE	1397				43255000	3.62E+08	2.19E+08
tr|Q9RYA9	tr|Q9RYA9	1721		33419000	36068000	1.2E+08	1.02E+08	55686000
tr|Q9RUI5	tr|Q9RUI5	1064	3.69E+08	2.02E+08	2.19E+08	3.6E+08	3.11E+08	3.5E+08
tr|Q9RUJ3	tr|Q9RUJ3	1069			15981000			
tr|Q9RU17	tr|Q9RU17	976	42413000	55761000	1.38E+08		29700000	
tr|Q9RRC3	tr|Q9RRC3	499	6574200			30561000	25336000	18669000
sp|Q9RU8	sp|Q9RU8	216	14215000	3662500	4586100	5022400	7397600	8043100
tr|Q9RRC5	tr|Q9RRC5	500				1.13E+08		1.39E+08
tr|Q9RRU4	tr|Q9RRU4	585						
tr|Q9RTX0	tr|Q9RTX0	947	80649000	31350000	73649000	1.23E+08	43461000	1.11E+08
tr|Q9RTM	tr|Q9RTM	898				89678000	1.03E+08	36489000
tr|Q9RTM	tr|Q9RTM	898	22813000		48388000			
tr|Q9RW5	tr|Q9RW5	1357	1.26E+09	1.67E+09	1.65E+09	1.72E+09	1.82E+09	1.75E+09
tr|Q9RV30	tr|Q9RV30	1166		70102000				
tr|Q9RUI4	tr|Q9RUI4	1063						
tr|Q9RYC6	tr|Q9RYC6	1727	52337000	36692000	35339000			
tr|Q9RYI1	tr|Q9RYI1	1760		88136000	71242000			
tr|Q9RXD5	tr|Q9RXD5	1573	58889000	43756000		75484000	70864000	55640000
tr|Q9RU06	tr|Q9RU06	971						
tr|Q9RVJ8	tr|Q9RVJ8	1243						
sp|Q9RWH	sp|Q9RWH	330		11503000	27987000	32922000	18583000	51662000
tr|Q9RTU7	tr|Q9RTU7	935	8939100	3586500	3845900	1851400	2615000	2266900
tr|Q9RUG	tr|Q9RUG	1055			23780000			
sp|Q9ZNA	sp|Q9ZNA	463				15604000	11894000	13137000

tr|Q9RYN5	tr|Q9RYN5	1779	20261000		55696000			
sp|Q9RY2	sp|Q9RY2	412	1.51E+09	1.27E+09	9.59E+08	1.32E+09	1.01E+09	1.24E+09
tr|Q9RSS3	tr|Q9RSS3	756	1.47E+08	1.52E+08	1.22E+08	2.62E+08	1.94E+08	2.26E+08
sp|Q9RRX	sp|Q9RRX	102	21527000	24193000	51248000			
tr|Q9RX71	tr|Q9RX71	1541						
tr|Q9RY75	tr|Q9RY75	1705	2.12E+08	1.62E+08	1.77E+08	1.87E+08	1.28E+08	1.59E+08
tr|Q9RT01	tr|Q9RT01	792		88230000	68056000	80599000	79934000	
tr|Q9RX12	tr|Q9RX12	1509						
tr|Q9RUZ5	tr|Q9RUZ5	1146						
tr|Q9RU07	tr|Q9RU07	972	23921000	10628000	10177000	10466000	16012000	15734000
tr|Q9RU07	tr|Q9RU07	972			22853000	84624000	88260000	95259000
tr|Q9RRD9	tr|Q9RRD9	507						
tr|Q9RWI6	tr|Q9RWI6	1417			834820	1578600		
sp|Q9RV2	sp|Q9RV2	264	81849000	73203000	70961000	67181000	64781000	83915000
tr|Q9RXP2	tr|Q9RXP2	1623	77394000	27779000	57114000			
sp|Q9RXY	sp|Q9RXY	407	3.33E+08	3.28E+08	2.76E+08	81487000	90161000	68361000
sp|Q9RWD	sp|Q9RWD	323	3.86E+08	3.73E+08	3.12E+08	3.08E+08	2.92E+08	2.57E+08
tr|Q9RWJ1	tr|Q9RWJ1	1420	46815000	39417000	27809000	43965000	51097000	53691000
tr|Q9RRA0	tr|Q9RRA0	483	23083000	62026000	55716000	3.69E+08	2.37E+08	1.59E+08
tr|Q9RRA0	tr|Q9RRA0	483	31029000	65692000	1.14E+08	31607000	38211000	1.15E+08
tr|Q9RRU9	tr|Q9RRU9	586	1.6E+08	1.72E+08	2.43E+08	2.61E+08	1.64E+08	2.45E+08
tr|Q9RWJ2	tr|Q9RWJ2	1421	2.54E+08	1.7E+08	2.06E+08	1.25E+08	1.12E+08	94246000
tr|Q9RYL8	tr|Q9RYL8	1771	77119000	39474000	59787000			
tr|Q9RVW	tr|Q9RVW	1312	3.77E+08	3.34E+08	4.79E+08	99648000	1.06E+08	1.5E+08
sp|Q9RSQ	sp|Q9RSQ	142		59841000	93482000	29058000	23862000	42404000
tr|Q9RUK5	tr|Q9RUK5	1078				3469200	5857400	
tr|Q9RWP	tr|Q9RWP	1446						
tr|Q9RUA	tr|Q9RUA	1024	97510000	96829000	80670000			
tr|Q9RXB8	tr|Q9RXB8	1566	60797000	62959000	1.07E+08	20760000	41633000	43104000
tr|Q9RYK2	tr|Q9RYK2	1764						
tr|Q9RTC7	tr|Q9RTC7	853	2.9E+08	3.1E+08	2.22E+08	1.62E+08	1.56E+08	1.53E+08
tr|Q9RZQ	tr|Q9RZQ	1958	1.56E+08	1.64E+08	1.92E+08	1.99E+08		41036000
tr|Q9RWH	tr|Q9RWH	1412	31977000	33891000		75739000	1.32E+08	59121000
tr|Q9RV11	tr|Q9RV11	1156	72550000	69238000	48354000	49079000	42379000	49485000
tr|Q9RR85	tr|Q9RR85	476	1.07E+08	70289000	1.16E+08			
sp|Q9RW0	sp|Q9RW0	342	60680000	48800000	46087000	43952000	38028000	49454000
sp|Q9RW0	sp|Q9RW0	342			53288000	2.11E+08	2.35E+08	2.41E+08
tr|Q9RVK8	tr|Q9RVK8	1248	6.75E+08	6.85E+08	5.68E+08	3.01E+08	2.84E+08	3.07E+08
tr|Q9RX85	tr|Q9RX85	1550						
tr|Q9RTJ4	tr|Q9RTJ4	884	1.73E+08	1.05E+08	1.49E+08	1.92E+08	1.66E+08	
sp|P52027	sp|P52027	51	45340000	89445000	63765000	57851000	71213000	57752000
tr|Q9RVL6	tr|Q9RVL6	1253	7.04E+08	5.37E+08	5.06E+08	2.32E+08	2.5E+08	2.12E+08
sp|Q9RY5	sp|Q9RY5	419	6743100	1258300	1829200	1361300	2284400	1683300
tr|Q9RXF0	tr|Q9RXF0	1581	4.49E+09	3.02E+09	4.36E+09	1.92E+09	1.04E+09	1.65E+09
tr|Q9RXF0	tr|Q9RXF0	1581		2196700	8170500	3971700		4476700
tr|Q9RUA	tr|Q9RUA	1023	1.77E+09	1.83E+09	1.48E+09	1.4E+09	1.24E+09	1.22E+09
tr|Q9RVP1	tr|Q9RVP1	1267	2.2E+08	2.57E+08	4.13E+08	2.06E+08	2.23E+08	2.67E+08
tr|Q9RYI7	tr|Q9RYI7	1763						
tr|Q9RTP3	tr|Q9RTP3	908	50801000	41733000	72461000			
tr|Q9RZA1	tr|Q9RZA1	1888	1.17E+08		1.25E+08		1.2E+08	
tr|Q9RUX9	tr|Q9RUX9	1137	2.67E+08	2.28E+08	2.19E+08	1.57E+08	1.86E+08	1.74E+08

sp|Q9RZ0	sp|Q9RZ0	451						
tr|Q9RSP0	tr|Q9RSP0	743					25756000	
tr|Q9RRH	tr|Q9RRH	526	97679000	1.21E+08		85866000	1.02E+08	97986000
sp|Q9RYV	sp|Q9RYV	445	33546000	15364000		8005700		11275000
tr|Q9RYQ4	tr|Q9RYQ4	1792	23346000	35764000	16997000	1.79E+08	1.65E+08	1.26E+08
tr|Q9RYQ4	tr|Q9RYQ4	1792	3.07E+08	4.71E+08	7.92E+08	2.47E+08	3.34E+08	2.54E+08
sp|Q9RSC	sp|Q9RSC	115		3.06E+08	2.89E+08	3.09E+08	3E+08	2.4E+08
tr|Q9RUX2	tr|Q9RUX2	1131	65069000			59706000	56687000	46251000
tr|Q9RWT	tr|Q9RWT	1469	1.2E+08	1.19E+08	1.25E+08	2.07E+08	1.54E+08	1.93E+08
tr|Q9RWT	tr|Q9RWT	1469		19630000	39898000	1.48E+08	1.79E+08	1.35E+08
tr|Q9RVC6	tr|Q9RVC6	1212	40670000	79457000	47237000	38399000	56510000	36867000
tr|Q9RYN8	tr|Q9RYN8	1781	96868000	85932000	75776000	1.67E+08	1.47E+08	1.6E+08
tr|Q9RW4	tr|Q9RW4	1353						
tr|Q9RY55	tr|Q9RY55	1697	58748000	63966000	44943000	56212000	64069000	62154000
sp|Q9RTF5	sp|Q9RTF5	182					28153000	
tr|Q9RYN3	tr|Q9RYN3	1777	8.59E+08	1.01E+09	8.85E+08	1.67E+09	2.07E+09	1.62E+09
tr|Q9RYN3	tr|Q9RYN3	1777	20810000		37389000			
sp|Q9RW0	sp|Q9RW0	327		5777400	5008800	2216300		3638000
tr|Q9RXS1	tr|Q9RXS1	1638		4095300		13090000	5323100	9681700
tr|Q9RTP5	tr|Q9RTP5	909	95377000	89108000	94815000		19095000	
tr|Q9RU45	tr|Q9RU45	992						
tr|Q9RVQ	tr|Q9RVQ	1273				1.54E+08	1.48E+08	1.83E+08
tr|Q9RVS6	tr|Q9RVS6	1291	3262200	1910600	2323900		1686800	1743700
sp|Q9RUY	sp|Q9RUY	259	19132000	28327000	40045000		50210000	1.11E+08
tr|Q9RXX2	tr|Q9RXX2	1660			21586000	25936000	26357000	21566000
tr|Q9RWD	tr|Q9RWD	1394						
tr|Q9RRJ0	tr|Q9RRJ0	536	39924000	39457000	33711000		15810000	
tr|Q9RTP3	tr|Q9RTP3	908						
tr|Q9RY02	tr|Q9RY02	1675		16111000			11494000	
tr|Q9RUF8	tr|Q9RUF8	1049	20045000	23221000	31070000		22681000	29684000
tr|Q9RTI8	tr|Q9RTI8	881	15967000		24645000			
sp|Q9RXT4	sp|Q9RXT4	400	39745000	68501000	57467000	1.68E+08	1.13E+08	1.41E+08
tr|Q9RZB1	tr|Q9RZB1	1894						
sp|Q9RTG	sp|Q9RTG	185				12950000		12519000
sp|Q9RTD	sp|Q9RTD	179	47129000	49552000	41416000	80849000	64193000	
sp|Q9RTB	sp|Q9RTB	177	37118000	44478000	48858000			
tr|Q9RXP2	tr|Q9RXP2	1623				62471000	52359000	50812000
tr|Q9RYS7	tr|Q9RYS7	1806						
tr|Q9RXF0	tr|Q9RXF0	1581	3.71E+08	3.7E+08	3.61E+08	5.82E+08	5.16E+08	5E+08
tr|Q9RXF0	tr|Q9RXF0	1581				12149000		14806000
tr|Q9RVP1	tr|Q9RVP1	1267	21364000		24311000	26336000		19116000
tr|Q9RR95	tr|Q9RR95	479						
tr|Q9RYQ2	tr|Q9RYQ2	1790	3538600		8634300	15665000		8353500
tr|Q9RYF4	tr|Q9RYF4	1743	32766000	26883000	49787000	23530000		27454000
tr|Q9RZC7	tr|Q9RZC7	1906						
tr|Q9RW7	tr|Q9RW7	1362	24430000		15903000	28631000	23726000	29348000
tr|Q9RT74	tr|Q9RT74	830						
tr|Q9RUC	tr|Q9RUC	1035	49085000	57848000	49794000		92062000	
tr|Q9RWU	tr|Q9RWU	1477			41824000			
tr|Q9RT92	tr|Q9RT92	838	35428000	21256000	22161000	18021000	24931000	19754000
tr|Q9RYS2	tr|Q9RYS2	1802						

tr|Q9RX10	tr|Q9RX10	1507						
sp|Q9RY7	sp|Q9RY7	422		23793000	34254000	15078000	25121000	
sp|Q9RSJ7	sp|Q9RSJ7	125	1.35E+08	76712000	90108000			
tr|Q9RSZ5	tr|Q9RSZ5	790						
tr|Q9RWL	tr|Q9RWL	1437						16695000
tr|Q9RV59	tr|Q9RV59	1182				52635000	43541000	47228000
tr|Q9RS87	tr|Q9RS87	663	25999000	12771000	2.04E+08	35640000	48520000	64975000
tr|Q9RS17	tr|Q9RS17	630	2.15E+08	3.32E+08	2.2E+08	7.56E+08	7.98E+08	5.48E+08
tr|Q9RU93	tr|Q9RU93	1017	3.78E+08	3.18E+08	2.84E+08	5.33E+08	6.32E+08	6.66E+08
tr|Q9RW9	tr|Q9RW9	1374	48131000	31929000	32883000	1.1E+08	95909000	94134000
tr|Q9RWX	tr|Q9RWX	1497	28311000	32556000	44688000	34475000	37001000	47804000
sp|Q9RVE	sp|Q9RVE	284	49893000	62447000	1.44E+08	1.64E+08	2.47E+08	2.26E+08
tr|Q9RRW	tr|Q9RRW	596	71650000		51034000	58364000	96136000	61834000
tr|Q9RUI0	tr|Q9RUI0	1061			33457000			
tr|Q9RRC7	tr|Q9RRC7	501	1.45E+08		1.67E+08	1.22E+08		1.18E+08
tr|Q9RSL7	tr|Q9RSL7	733		24121000	16505000	32850000	27696000	
tr|Q9RUF9	tr|Q9RUF9	1050		927480	1713300	3027500	1374000	1647000
sp|Q9RT2	sp|Q9RT2	163	1.16E+08	52964000	89146000	1.05E+08	1.1E+08	1.06E+08
tr|Q9RWF	tr|Q9RWF	1401	1.08E+09	8.98E+08	8.15E+08	1.14E+09	80875000	1.42E+08
sp|Q9RUF	sp|Q9RUF	231						
sp|Q9RXJ2	sp|Q9RXJ2	377	2834000	960340	1332900		1462300	
sp|Q9RXJ2	sp|Q9RXJ2	377	24535000	46497000	1.15E+08		15386000	
tr|Q9RYA9	tr|Q9RYA9	1721				3331100	4373900	3619400
tr|Q9RVR5	tr|Q9RVR5	1284	15863000	10017000	8811900	19970000	18609000	19900000
tr|Q9RT26	tr|Q9RT26	804	2.34E+08	5.27E+08	1.75E+08	2.77E+08	2.83E+08	2.85E+08
tr|Q9RSV8	tr|Q9RSV8	773	42837000	2.49E+08	53509000	54096000	52966000	67465000
tr|Q9RV88	tr|Q9RV88	1194				28949000	21630000	
sp|Q9RTF	sp|Q9RTF	181	1.81E+08	1.63E+08	1.39E+08	6.83E+08	6.37E+08	5.01E+08
tr|Q9RYX6	tr|Q9RYX6	1835	2.02E+08	2E+08	1.84E+08	2.75E+08	3.13E+08	3.17E+08
tr|Q9RZB4	tr|Q9RZB4	1897						
tr|Q9RS80	tr|Q9RS80	660	2.04E+08	2.02E+08	1.82E+08	2.76E+08	2.91E+08	3.07E+08
sp|Q9RXT	sp|Q9RXT	401			5954100			11784000
tr|Q9RTE1	tr|Q9RTE1	860		14705000		26909000		
tr|Q9RRE1	tr|Q9RRE1	509	52950000	65486000	61538000	39751000	81166000	27448000
tr|Q9RVA7	tr|Q9RVA7	1203	1.67E+08	2.1E+08	1.89E+08	1.8E+08	3.52E+08	2.1E+08
tr|Q9RYD0	tr|Q9RYD0	1730	3.28E+08	4.56E+08	2.5E+08	3.95E+08	4.39E+08	4.34E+08
sp|Q9RRC	sp|Q9RRC	79			26317000			
tr|Q9RU27	tr|Q9RU27	980	3.18E+08	3.63E+08	3.36E+08	4.56E+08	5.05E+08	4.73E+08
tr|Q9RXM	tr|Q9RXM	1615	14393000					
tr|Q9RVQ	tr|Q9RVQ	1274	2.11E+08	2.16E+08	1.96E+08	6.38E+08	4.39E+08	5.97E+08
tr|Q9RR62	tr|Q9RR62	467	1.02E+09	9.4E+08	8.5E+08	9.55E+08	8.38E+08	8.99E+08
tr|Q9RR83	tr|Q9RR83	475		48985000	49752000	41385000	43845000	38348000
tr|Q9RZI1	tr|Q9RZI1	1935						
tr|Q9RRC7	tr|Q9RRC7	501						
sp|Q9RX3	sp|Q9RX3	356	25871000	41696000	23691000	97784000	1.02E+08	86036000
tr|Q9RTX4	tr|Q9RTX4	949	2.4E+08	2.13E+08	1.84E+08	2.68E+08	2.36E+08	2.69E+08
sp|Q9RW0	sp|Q9RW0	327	2.48E+08	3.55E+08	3.11E+08	3.96E+08	5.86E+08	4.13E+08
tr|Q9RV68	tr|Q9RV68	1185	5.26E+08	4.57E+08	4.3E+08	5.59E+08	4.55E+08	5.23E+08
tr|Q9RTI4	tr|Q9RTI4	880	53547000	33180000	41029000			41600000
tr|Q9RW0	tr|Q9RW0	1332	1.39E+08	1.42E+08	1.13E+08	2.51E+08	2.26E+08	2.49E+08
tr|Q9RW0	tr|Q9RW0	1332	3.55E+08	3.84E+08	2.99E+08	1.88E+08	2.16E+08	2.39E+08

tr|Q9RRX6	tr|Q9RRX6	604			16068000			
sp|Q9RU2	sp|Q9RU2	209			45349000			
sp|Q9RVT	sp|Q9RVT	297						
tr|Q9RUL0	tr|Q9RUL0	1082		3168500		2366800	3662700	2279000
sp|Q9RY0	sp|Q9RY0	410	88265000	67300000	76358000	2.46E+08	1.79E+08	2.19E+08
tr|Q9RYT2	tr|Q9RYT2	1811	43471000	30850000	45321000			71664000
tr|Q9RSC6	tr|Q9RSC6	689						
tr|Q9RUY5	tr|Q9RUY5	1141	1.01E+09	8.64E+08	8.46E+08	1.17E+09	7.43E+08	8.69E+08
tr|Q9RSZ5	tr|Q9RSZ5	790	10954000			99622000	69074000	82404000
tr|Q9RZD4	tr|Q9RZD4	1909				13486000	18165000	12491000
tr|Q9RRA0	tr|Q9RRA0	483	50937000	56635000	46004000	75035000	66933000	67815000
tr|Q9RWV	tr|Q9RWV	1485	1.82E+08	2.23E+08	1.49E+08	1.69E+08	3.17E+08	1.86E+08
tr|Q9RZJ2	tr|Q9RZJ2	1939	87786000	1.45E+08	1.14E+08	1.75E+08	6436500	1.98E+08
tr|Q9RWT	tr|Q9RWT	1469					13486000	
tr|Q9RUL2	tr|Q9RUL2	1084	5.15E+08	6.47E+08	4.28E+08			
tr|Q9RW8	tr|Q9RW8	1368						
tr|Q9RZ79	tr|Q9RZ79	1873		1.48E+08	3.59E+08		1.73E+08	
tr|Q9RVK5	tr|Q9RVK5	1245	19244000					
tr|Q9RYM	tr|Q9RYM	1773	17939000		17504000			
sp|Q9RW0	sp|Q9RW0	342	26104000		28116000			
tr|Q9RWI0	tr|Q9RWI0	1413		30650000	39624000	1.22E+08		
tr|Q9RYN6	tr|Q9RYN6	1780	2.93E+09	3.34E+09	3.29E+09	7.22E+09	7.1E+09	5.89E+09
tr|Q9RWF	tr|Q9RWF	1402				23242000	20903000	17279000
tr|Q9RXF3	tr|Q9RXF3	1583						
tr|Q9RXM	tr|Q9RXM	1615	5.12E+08	4.72E+08	3.84E+08	1.08E+09	1.03E+09	8.9E+08
tr|Q9RXM	tr|Q9RXM	1615			24813000	22221000	19779000	18863000
tr|Q9RSZ5	tr|Q9RSZ5	790						
tr|Q9RRS0	tr|Q9RRS0	572	2359700	1174100	927670	1357700		2354400
tr|Q9RSU7	tr|Q9RSU7	767	23993000	20185000	22394000	9844600	9948800	12026000
sp|Q9RYM	sp|Q9RYM	439	51258000	44249000	62450000	6.57E+08	4.13E+08	3.85E+08
tr|Q9RXX2	tr|Q9RXX2	1660	26430000	1.13E+08	72401000	45730000	74482000	30173000
tr|Q9RVR2	tr|Q9RVR2	1281	2.74E+08	2.17E+08	2.4E+08	1.59E+09	1.19E+09	8.5E+08
tr|Q9RRA1	tr|Q9RRA1	484	5941100	3637400	2772500			5550300
tr|Q9RYW	tr|Q9RYW	1830	35011000	32191000	18609000	42900000	37384000	37918000
tr|Q9RXZ4	tr|Q9RXZ4	1670	39251000	30818000	54861000	74050000	78729000	78478000
tr|Q9RXZ4	tr|Q9RXZ4	1670	1.05E+08	1.26E+08	1.84E+08	44264000	28227000	
tr|Q9RSZ3	tr|Q9RSZ3	789	37413000	24480000	24512000	31667000	25842000	27900000
tr|Q9RVZ0	tr|Q9RVZ0	1324	43626000	39248000	34536000	1.02E+08	92962000	91366000
tr|Q9RT68	tr|Q9RT68	826	30980000	39365000	31824000	34799000	35739000	21320000
sp|Q9RUV	sp|Q9RUV	256					22607000	
sp|Q9RXF	sp|Q9RXF	369	59769000			53217000		64815000
tr|Q9RYE0	tr|Q9RYE0	1736	76360000	54424000	63160000	1.32E+08	97811000	1.1E+08
tr|Q9RZU4	tr|Q9RZU4	1977						
tr|Q9RZU4	tr|Q9RZU4	1977	59226000					
tr|Q9RRV8	tr|Q9RRV8	593	2.16E+10	2.32E+10	1.85E+10	2.08E+10	1.84E+10	1.84E+10
tr|Q9RRV8	tr|Q9RRV8	593						
tr|Q9RSV3	tr|Q9RSV3	770	1.3E+08	1.1E+08	98582000	1.3E+08	1.13E+08	1.22E+08
tr|Q9RYA9	tr|Q9RYA9	1721	59155000	71476000	69346000	2.49E+08	1.09E+08	1.64E+08
tr|Q9RYA9	tr|Q9RYA9	1721		34541000	57291000			
tr|Q9RV56	tr|Q9RV56	1180;1971	15969000	15418000				
tr|Q9RY54	tr|Q9RY54	1696			8871700	12838000	8713300	

tr|Q9RTA2	tr|Q9RTA2	844	19829000	18929000		20386000	18908000	18487000	
tr|Q9RYY7	tr|Q9RYY7	1837							
tr|Q9RZT2	tr|Q9RZT2	1969							
sp|Q9RZ0	sp|Q9RZ0	450					8906300		
tr|Q9RYW	tr|Q9RYW	1830	12738000		17523000		16030000	10832000	
sp|Q9RRB	sp|Q9RRB	76							
sp|Q9RRB	sp|Q9RRB	76	1.13E+08		1.36E+08			87716000	
sp|Q5933	sp|Q5933	63	2.09E+08	2.01E+08	3.4E+08	72746000	98486000	1.03E+08	
tr|Q9RU9	tr|Q9RU9	1016			1.38E+08				
tr|Q9RTV7	tr|Q9RTV7	940	84706000	86357000	83156000	1.74E+08	67392000	1.02E+08	
sp|Q9RX1	sp|Q9RX1	351			13391000	9919100			
tr|Q9RZH	tr|Q9RZH	1931	80493000	77603000	85785000		13140000		
tr|Q9RY92	tr|Q9RY92	1713	3.13E+08	3.81E+08	2.61E+08	5.29E+08	3.84E+08	4.35E+08	
sp|Q9RW	sp|Q9RW	348	3.97E+08	4.42E+08	3.37E+08	7.11E+08	2.01E+08	2.02E+08	
tr|Q9RXL6	tr|Q9RXL6	1612	1.48E+08	1.11E+08	1.21E+08	66100000	53207000	56518000	
tr|Q9RRK2	tr|Q9RRK2	539	1.11E+08	57163000	61122000	69530000	90651000	87401000	
sp|Q9RXR	sp|Q9RXR	394		31447000	22233000	27884000		46849000	
tr|Q9RZ91	tr|Q9RZ91	1882	49448000	53555000	39060000	43935000	28013000	28888000	
sp|Q9RXJ7	sp|Q9RXJ7	382							
sp|P5686	sp|P56867	56	3.08E+09	5.04E+09	4.64E+09	9.86E+08	9.5E+08	1.45E+09	
tr|Q9RYQ	tr|Q9RYQ	1788	49228000	53139000	55404000	37777000	48161000	46330000	
tr|Q9RYS1	tr|Q9RYS1	1801	4.86E+08	4.29E+08	6.14E+08			12973000	
tr|Q9RY74	tr|Q9RY74	1704	1.64E+08	1.25E+08	1.81E+08	54516000	39747000	44019000	
tr|Q9RT75	tr|Q9RT75	831	54780000	62996000	63856000	42490000	26549000	29236000	
tr|Q9RT75	tr|Q9RT75	831	3907600	5869400	10907000				
sp|Q9RU7	sp|Q9RU7	213	1.15E+08	1.2E+08	1.25E+08	3.54E+08	3.41E+08	2.61E+08	
tr|Q9RXQ	tr|Q9RXQ	1627	10257000	7832900	20692000	10374000	19066000	18794000	
tr|Q9RXB	tr|Q9RXB	1561	32487000		32148000	87508000	1.14E+08	77253000	
tr|Q9RTU	tr|Q9RTU	932	3.03E+08	3.02E+08	2.19E+08	28063000	16122000	35997000	
tr|Q9RYE9	tr|Q9RYE9	1741				25370000			
tr|Q9RTY0	tr|Q9RTY0	955		1.21E+09	9.08E+08	1.18E+09	1.16E+09	1.23E+09	
tr|Q9RV21	tr|Q9RV21	1162		43477000	45103000	33083000	37684000	32058000	
sp|Q9RVC	sp|Q9RVC	276	16778000					38220000	
sp|Q9RW	sp|Q9RW	328	6.82E+08	6.08E+08	9.55E+08	1.2E+09	1.6E+09	9.03E+08	
tr|Q9RRI3	tr|Q9RRI3	531							
sp|Q9RXK	sp|Q9RXK	389	60967000	94504000	72151000	1.53E+08	1.69E+08	1.62E+08	
sp|Q9RXK	sp|Q9RXK	389	1.48E+08	2.63E+08	6.03E+08	1.13E+08	2.05E+08	1.98E+08	
tr|Q9RR71	tr|Q9RR71	470							
tr|Q9RTC3	tr|Q9RTC3	850							
tr|Q9RRD9	tr|Q9RRD9	507			1.39E+08	1.2E+08	1.18E+08	1.12E+08	
tr|Q9RYX6	tr|Q9RYX6	1835	56906000	29379000	71442000	1.48E+08	1.69E+08	89515000	
tr|Q9RZ88	tr|Q9RZ88	1880				1.49E+08	1.42E+08	1.55E+08	
sp|Q9RVE	sp|Q9RVE	283	2310800	1517700	3013400	911250	1931000	2745600	
sp|Q9RT4	sp|Q9RT4	167	85937000		82101000		57391000	70077000	
tr|Q9RWL	tr|Q9RWL	1437	75792000	58279000	49498000	30654000	44882000	39034000	
sp|Q9RX5	sp|Q9RX5	359	30746000	37171000	22155000	35919000	25810000	25245000	
tr|Q9RYY7	tr|Q9RYY7	1837			13110000	39091000	31736000	30859000	
tr|Q9RSR2	tr|Q9RSR2	750	38986000	24036000	34292000				
tr|Q9RU5	tr|Q9RU5	994	2.65E+08	3.52E+08	2.67E+08	4.84E+08	7.2E+08	4.78E+08	
tr|Q9RU5	tr|Q9RU5	994	29518000	1.03E+08	85475000		40676000	35766000	
sp|Q9RRX	sp|Q9RRX	101	7.4E+08	8.15E+08	7.91E+08	9.11E+08	8.91E+08	8.72E+08	

sp|Q9RUP	sp|Q9RUP	243	37531000	65249000	92939000	2.35E+08	91671000	89546000
tr|Q9RRF2	tr|Q9RRF2	515	1.86E+08	2.19E+08	2.76E+08	4.59E+08	5.91E+08	3.64E+08
tr|Q9RWW	tr|Q9RWW	1494						
tr|Q9RYA(	tr|Q9RYA(	1718						
tr|Q9RZV4	tr|Q9RZV4	1984	69148000	1.4E+08	1.56E+08	3.83E+08	4.99E+08	3.46E+08
tr|Q9RS52	tr|Q9RS52	646		60385000	47906000		90495000	
tr|Q9RT3(	tr|Q9RT3(	807		12049000	6376100			
tr|Q9RTJ4	tr|Q9RTJ4	884	2.02E+08	1.66E+08	4.28E+08	18086000	19220000	14533000
sp|Q9RRB	sp|Q9RRB	77				7.82E+08	8.08E+08	7.27E+08
sp|Q9RRB	sp|Q9RRB	77						
sp|P5690!	sp|P5690!	57			18671000			
tr|Q9RYS7	tr|Q9RYS7	1806	8.72E+08	6.53E+08	6.09E+08	8.42E+08	7.68E+08	1.04E+09
tr|Q9RYS7	tr|Q9RYS7	1806	23884000		22526000			
sp|Q9RU2	sp|Q9RU2	208	1.7E+08	2.27E+08	1.85E+08	1.14E+08	1.67E+08	1.11E+08
sp|Q9RT2	sp|Q9RT2	162	1.16E+09	1.14E+09	9.2E+08	2.03E+09	1.51E+09	1.51E+09
tr|Q9RZC6	tr|Q9RZC6	1905			33281000	27538000	50522000	47634000
sp|Q9RXJ8	sp|Q9RXJ8	383	3.38E+08	1.56E+08	1.65E+08	98288000	1.29E+08	1.41E+08
sp|Q9RYR	sp|Q9RYR	442	1.3E+08	1.42E+08	99288000	1.59E+08	2.15E+08	1.81E+08
sp|Q9RVP	sp|Q9RVP	294	3.05E+09	3.21E+09	2.39E+09	2.06E+09	1.89E+09	1.97E+09
tr|Q9RTW	tr|Q9RTW	945	14284000	10819000	18509000	91312000	48007000	53335000
tr|Q9RV06	tr|Q9RV06	1154	58875000			45141000	46676000	45916000
tr|Q9RWT	tr|Q9RWT	1465	2.5E+08	2.35E+08	1.76E+08	1.9E+08	1.46E+08	1.93E+08
sp|Q9RYW	sp|Q9RYW	446					16920000	
tr|Q9RSV5	tr|Q9RSV5	771	28056000	60581000	38658000			
sp|Q9RVV	sp|Q9RVV	300	40845000	36439000	33720000	89738000	78187000	74528000
sp|Q9RVV	sp|Q9RVV	300		1051100	2347300	3699100	5706000	4872500
sp|Q9RVV	sp|Q9RVV	300						
sp|Q9RRB	sp|Q9RRB	77	6.79E+08		6.4E+08	8.58E+08	7.9E+08	
tr|Q9RT05	tr|Q9RT05	794	25367000		42205000	30494000		29362000
sp|Q9RVM	sp|Q9RVM	292					14792000	
tr|Q9RSD6	tr|Q9RSD6	697	2.79E+08	2.16E+08	3E+08	1.75E+08	2.15E+08	2.63E+08
tr|Q9RUJ6	tr|Q9RUJ6	1072	2.37E+08	1.84E+08	1.43E+08	2.23E+08	2.46E+08	2.3E+08
tr|Q9RS86	tr|Q9RS86	662						
tr|Q9RRY6	tr|Q9RRY6	609			39887000		33067000	
tr|Q9RS11	tr|Q9RS11	627						
tr|Q9RSY1	tr|Q9RSY1	782					69173000	
tr|Q9RY67	tr|Q9RY67	1701		19750000				
sp|Q9RU9	sp|Q9RU9	223	33958000		23421000	58301000	66161000	67899000
tr|Q9RXG(	tr|Q9RXG(	1587	4.43E+08	4.75E+08	5.12E+08	2.88E+08	4.92E+08	3.3E+08
tr|Q9RVN	tr|Q9RVN	1265	32231000		18778000		4207700	
tr|Q9RTL2	tr|Q9RTL2	893	2.97E+09	2.82E+09	2.28E+09	4.63E+09	3.26E+09	4.29E+09
tr|Q9RX49	tr|Q9RX49	1527	76990000	70766000	79118000	93801000		99341000
tr|Q9RYW	tr|Q9RYW	1830	95328000	2.03E+08	1.7E+08	1.94E+08	1.51E+08	1.77E+08
tr|Q9RW2	tr|Q9RW2	1342	34687000	49941000	58933000		22641000	12985000
sp|Q9RR6	sp|Q9RR6	66	1.31E+08	1.28E+08	1.17E+08	2.54E+08	2.16E+08	2.36E+08
tr|Q9RW3	tr|Q9RW3	1348						
sp|Q9RS2	sp|Q9RS2	108	97934000	83965000	63521000	3.77E+08	4.42E+08	3.45E+08
sp|Q4657	sp|Q4657	62	13097000		10494000			
tr|Q9RVK8	tr|Q9RVK8	1248	15003000	7056800	6907500	5825200	9073600	7552600
tr|Q9RTA3	tr|Q9RTA3	845						
tr|Q9RUL(	tr|Q9RUL(	1082	4.59E+08	4.18E+08	4.53E+08			

tr|Q9RRK9	tr|Q9RRK9	543	17527000	21863000	2.27E+08	25383000	1.39E+08	26610000
tr|Q9RRK9	tr|Q9RRK9	543						
tr|Q9RTU7	tr|Q9RTU7	935		3465400	7077800	10310000	12663000	12430000
tr|Q9RX10	tr|Q9RX10	1507	2.5E+08	2.5E+08	2.65E+08	3.55E+08	3.43E+08	2.64E+08
tr|Q9RX10	tr|Q9RX10	1507		5109300	27748000			
tr|Q9RVB3	tr|Q9RVB3	1207						
tr|Q9RVS0	tr|Q9RVS0	1286				8312600		
tr|Q9RWU	tr|Q9RWU	1480	9369900	12171000	9944000	15504000	24821000	
tr|Q9RSL8	tr|Q9RSL8	734	1.12E+08	1.04E+08	90517000	3.08E+08	3.5E+08	3.16E+08
tr|Q9RSY7	tr|Q9RSY7	785	44835000	37229000	44734000	89434000	75566000	57596000
sp|Q9RU7	sp|Q9RU7	215	76072000	1.4E+08	1.18E+08	1.32E+09	1.92E+09	6.61E+08
tr|Q9RSQ4	tr|Q9RSQ4	748		54969000	3.63E+08		3.44E+08	2.61E+08
sp|Q9RUF	sp|Q9RUF	231				47611000	60329000	55608000
tr|Q9RXB0	tr|Q9RXB0	1561						
tr|Q9RXB0	tr|Q9RXB0	1561			20926000	19941000	24449000	26241000
tr|Q9RTP0	tr|Q9RTP0	906	4268200					925580
tr|Q9RU62	tr|Q9RU62	1004	70317000	61581000	58076000	99337000	66697000	84019000
tr|Q9RRI1	tr|Q9RRI1	530				1.14E+08		82250000
tr|Q9RYS1	tr|Q9RYS1	1801	5.12E+08	7.96E+08	4.32E+08	5.02E+08	6.03E+08	5.47E+08
tr|Q9RUL0	tr|Q9RUL0	1082	28762000		28672000			
tr|Q9RVA7	tr|Q9RVA7	1203	1.2E+08	1.1E+08	87214000	1.16E+08	1.02E+08	97007000
tr|Q9RZ28	tr|Q9RZ28	1858	41625000	40957000	28362000		14685000	
sp|Q9RSI9	sp|Q9RSI9	122	4.89E+08	4.35E+08	8.08E+08	2E+08	2.23E+08	3.06E+08
tr|Q9RWI1	tr|Q9RWI1	1414	6703600	6740000	4633300			
tr|Q9RUP0	tr|Q9RUP0	1098	2.48E+08	5.36E+08	3.92E+08	3.14E+08	4.04E+08	3.21E+08
tr|Q9RRW	tr|Q9RRW	595	13935000	11211000	20356000			19455000
tr|Q9RSJ0	tr|Q9RSJ0	726						
tr|Q9RVR2	tr|Q9RVR2	1281	1.1E+09	1.05E+09	1.19E+09	1.45E+09	1.59E+09	1.35E+09
tr|Q9RTK5	tr|Q9RTK5	889	6.73E+08	7.05E+08	7.42E+08	4.51E+08	4.35E+08	4.42E+08
tr|Q9RV29	tr|Q9RV29	1165			4833900	8131000		
sp|Q9RWK	sp|Q9RWK	335	39362000	51661000	45178000	78765000	84044000	76625000
tr|Q9RU63	tr|Q9RU63	1005				8971600	9774000	10387000
tr|Q9RY79	tr|Q9RY79	1708		28965000	69874000		28855000	
sp|Q9RUI	sp|Q9RUI	237	1.23E+08	91581000	64266000			
tr|Q9RWY	tr|Q9RWY	1500						
tr|Q9RY47	tr|Q9RY47	1695	82517000	72181000	63956000	62832000	70118000	67183000
tr|Q9RSY4	tr|Q9RSY4	783	26946000	38904000		28742000		18853000
tr|Q9RZT9	tr|Q9RZT9	1975		93256000	88172000	87727000	89879000	1.2E+08
tr|Q9RYQ3	tr|Q9RYQ3	1791						
sp|Q9RWU	sp|Q9RWU	347	2.01E+08	2.5E+08	2.07E+08	2.39E+08	3.37E+08	2.14E+08
tr|Q9RUQ	tr|Q9RUQ	1103						
tr|Q9RVJ0	tr|Q9RVJ0	1238	3.1E+08	2.53E+08	4.8E+08		53935000	
tr|Q9RW8	tr|Q9RW8	1367	28240000		1.17E+08			31331000
tr|Q9RVP5	tr|Q9RVP5	1269	6.22E+08	44417000	5.19E+08	14556000	43453000	30973000
sp|Q9RXJ9	sp|Q9RXJ9	384	2.83E+09	2.83E+09	2.71E+09	5.21E+09	4.89E+09	5.29E+09
tr|Q9RYI1	tr|Q9RYI1	1760	95246000	1.23E+08	1.06E+08	73634000	95993000	83549000
sp|Q9RW6	sp|Q9RW6	312			27342000			
sp|Q9RY2	sp|Q9RY2	411			46748000	97551000	57583000	87529000
sp|Q9RY2	sp|Q9RY2	411				28796000	26196000	
tr|Q9RZT2	tr|Q9RZT2	1969					32839000	58587000
tr|Q9RS29	tr|Q9RS29	636	4.89E+08	5.12E+08	4.66E+08			

tr|Q9RYV0	tr|Q9RYV0	1820	8.44E+08	1.06E+09	8E+08	9.18E+08	8.5E+08	9.89E+08
tr|Q9RTM	tr|Q9RTM	898				1.98E+08	2.55E+08	98483000
tr|Q9RYS0	tr|Q9RYS0	1800						
tr|Q9RZN4	tr|Q9RZN4	1951	1.79E+08	2.1E+08	1.72E+08	5.15E+08	2.43E+08	4.43E+08
tr|Q9RZV2	tr|Q9RZV2	1982			12015000			
tr|Q9RZV2	tr|Q9RZV2	1982						
sp|Q9RRH	sp|Q9RRH	85	61494000	96363000	59371000	31765000	28912000	41832000
tr|Q9RTC9	tr|Q9RTC9	855	70068000	84502000	72123000		1.26E+08	
tr|Q9RUC	tr|Q9RUC	1036	19833000					
tr|Q9RWX	tr|Q9RWX	1496					6503000	
sp|O3250	sp|O3250	45	69527000	42427000	38631000	68737000	79230000	76690000
tr|Q9RWU	tr|Q9RWU	1478	4.88E+08	4.09E+08	3.86E+08	4.37E+08	4.63E+08	4.47E+08
tr|Q9RTX7	tr|Q9RTX7	952	43250000	35945000	31050000	72298000	67328000	77685000
sp|P56867	sp|P56867	56	1.15E+09	1.02E+09	9.61E+08	1.89E+09	1.4E+09	1.39E+09
tr|Q9RXH8	tr|Q9RXH8	1599			20561000			
tr|Q9RYD9	tr|Q9RYD9	1735	22211000	21114000				
tr|Q9RYW	tr|Q9RYW	1830	3.38E+08	3.02E+08	2.18E+08	3.17E+08	2.49E+08	3.11E+08
sp|O3250	sp|O3250	45	8.7E+08	8.5E+08	1.99E+08	1.27E+09	1.33E+09	1.32E+09
sp|O3250	sp|O3250	45	1.65E+08	2.27E+08	2.52E+08	34274000	34817000	74237000
tr|Q9RT53	tr|Q9RT53	821						
tr|Q9RVZ3	tr|Q9RVZ3	1326	6.07E+08	4.57E+08	3.45E+08	7.64E+08	7.21E+08	6.95E+08
tr|Q9RVZ3	tr|Q9RVZ3	1326	21270000	16450000	15428000			
sp|Q9RZA	sp|Q9RZA	454	12841000		6415500	74659000	37683000	47317000
tr|Q9RYQ0	tr|Q9RYQ0	1788	1.88E+09	1.45E+09	2.67E+09	5.99E+08	7.62E+08	7.99E+08
tr|Q9RZB4	tr|Q9RZB4	1897	7088500		7792900			7162500
tr|Q9RSV3	tr|Q9RSV3	770	2.16E+08	3.42E+08		2.74E+08	2.68E+08	2.23E+08
tr|Q9RX56	tr|Q9RX56	1531	4.99E+08	3.34E+08	4.91E+08	7.67E+08	6.77E+08	7.95E+08
tr|Q9RX56	tr|Q9RX56	1531	2.05E+08	1.98E+08	2.73E+08	1.58E+08	1.29E+08	1.77E+08
tr|Q9RRG4	tr|Q9RRG4	520	20358000		8647300	11990000	16730000	11044000
tr|Q9RZ10	tr|Q9RZ10	1848	59408000	50244000	48668000	1.14E+08	1.02E+08	1.02E+08
tr|Q9RZ10	tr|Q9RZ10	1848	6.61E+08	5.32E+08	1.21E+09	1.51E+08	2.04E+08	2.97E+08
tr|Q9RUJ4	tr|Q9RUJ4	1070				56220000	88507000	73944000
tr|Q9RRX1	tr|Q9RRX1	601	5.87E+08	1.2E+09	1.38E+09	3.9E+08	5.04E+08	4.17E+08
tr|Q9RRX1	tr|Q9RRX1	601			3.81E+08			
tr|Q9RVS0	tr|Q9RVS0	1286	1.75E+09	1.72E+09	1.67E+09	1.92E+09	1.44E+09	1.75E+09
tr|Q9RVS0	tr|Q9RVS0	1286	16866000	20973000	34853000		19823000	23652000
sp|Q9RR9	sp|Q9RR9	73			3132200			
tr|Q9RZH5	tr|Q9RZH5	1930	1.45E+08	1.31E+08	1.15E+08	59653000	1.03E+08	68511000
sp|Q9RV2	sp|Q9RV2	264	7.26E+08	7.01E+08	5.2E+08	4.19E+08	3.98E+08	4.27E+08
tr|Q9RRA0	tr|Q9RRA0	483	11622000	4522100	4079600	3189800	4629500	3530600
tr|Q9RTH7	tr|Q9RTH7	878			3140600			
tr|Q9RVL5	tr|Q9RVL5	1252	32917000	23429000	17858000			
sp|Q9RT2	sp|Q9RT2	165	51586000	46960000	80591000	99692000	97910000	1.49E+08
tr|Q9RWS	tr|Q9RWS	1464						
tr|Q9RZD6	tr|Q9RZD6	1910			20849000			
tr|Q9RYT8	tr|Q9RYT8	1816	1.02E+08	64421000	65931000	2.58E+08	2.93E+08	3.11E+08
sp|Q9RVV	sp|Q9RVV	300	44308000	59450000	45488000	47125000	48812000	43100000
sp|Q9RVV	sp|Q9RVV	300	3.72E+08		5.13E+08			
tr|Q9RU78	tr|Q9RU78	1014						
sp|Q9RYU	sp|Q9RYU	444						
tr|Q9RUA	tr|Q9RUA	1023	4.73E+08	5.06E+08	3.77E+08	5.86E+08	5.79E+08	5.96E+08

tr|Q9RUA	tr|Q9RUA	1023	1.19E+08	1.73E+08	2.53E+08	57139000	79383000	77060000
sp|Q9RUG	sp|Q9RUG	235	32567000	45608000	30287000			
sp|Q9RV2	sp|Q9RV2	262						
tr|Q9RZ08	tr|Q9RZ08	1846	3.4E+08	3.46E+08	3.31E+08	4.75E+08	4.7E+08	4.63E+08
tr|Q9RZ08	tr|Q9RZ08	1846	76300000	70337000	1.02E+08			
tr|Q9RTZ8	tr|Q9RTZ8	966	3.41E+08	3.17E+08	2.66E+08	3.36E+08	3.68E+08	3.79E+08
tr|Q9RYK2	tr|Q9RYK2	1764			3692400			
sp|Q9RS6	sp|Q9RS6	112	5.18E+08	6.03E+08	5.02E+08	7.19E+08	6.2E+08	6.48E+08
sp|Q9RS6	sp|Q9RS6	112	99244000	94612000		24578000		
sp|Q9RY0	sp|Q9RY0	410						
sp|Q9RW(	sp|Q9RW(	326					3670000	2173000
tr|Q9RXF6	tr|Q9RXF6	1585	2.66E+08	2.52E+08	1.97E+08	1.96E+08	2.02E+08	2.17E+08
tr|Q9RT58	tr|Q9RT58	822		35995000	56050000	51178000	64275000	31119000
sp|P56926	sp|P56926	59	42261000	31237000	31267000	52583000	74892000	53823000
tr|Q9RYD0	tr|Q9RYD0	1730	96147000	95540000	83552000	1.07E+08	1.09E+08	1.07E+08
tr|Q9RZK3	tr|Q9RZK3	1941						
sp|Q9RU2	sp|Q9RU2	208	6.21E+08	6.25E+08	5.02E+08	4.1E+08	3.75E+08	4.69E+08
sp|Q9X71	sp|Q9X71	462		18002000		11634000	8627500	11087000
tr|Q9RVQ	tr|Q9RVQ	1272						
sp|Q9RY8	sp|Q9RY8	424						
tr|Q9RWP	tr|Q9RWP	1449	5.01E+08	3.95E+08	3.32E+08	6.67E+08	7.11E+08	7.18E+08
tr|Q9RW9	tr|Q9RW9	1377						
tr|Q9RW9	tr|Q9RW9	1377	20166000	30821000	39658000			
tr|Q9RUV4	tr|Q9RUV4	1125	64038000	82198000	2.39E+08	11116000	20353000	26542000
tr|Q9RUW	tr|Q9RUW	1126	83983000	63933000	1.31E+08		43700000	
tr|Q9RTY1	tr|Q9RTY1	956	3.12E+08	2.35E+08	2.37E+08			
tr|Q9RZ85	tr|Q9RZ85	1877	37500000	30336000	50953000		10221000	
tr|Q9RV18	tr|Q9RV18	1161						
tr|Q9RXG9	tr|Q9RXG9	1592					13356000	
sp|Q9RRX	sp|Q9RRX	101				32126000	31792000	33597000
tr|Q9RUX1	tr|Q9RUX1	1130						
tr|Q9RUD	tr|Q9RUD	1040	66337000	47369000	46379000	1.15E+08	1.27E+08	1.13E+08
sp|Q9RY6	sp|Q9RY6	421	94330000	1.15E+08	81293000	77004000	80428000	84998000
tr|Q9RS98	tr|Q9RS98	672	1.23E+09	1.34E+09	1.01E+09	9.85E+08	1.11E+09	1.03E+09
tr|Q9RZ27	tr|Q9RZ27	1857						
tr|Q9RZA6	tr|Q9RZA6	1891	95767000	1.28E+08	1.76E+08			1.44E+08
tr|Q9RX12	tr|Q9RX12	1509	3.24E+08	2.32E+08	2.16E+08	90999000	1.44E+08	
sp|Q9RUE	sp|Q9RUE	229	6469300	3006100	17571000			
tr|Q9RTW	tr|Q9RTW	943		58683000	53325000	59507000	61747000	62579000
tr|Q9RYS7	tr|Q9RYS7	1806	2.69E+08	1.37E+08	2.29E+08	47354000	58675000	81770000
tr|Q9RWI3	tr|Q9RWI3	1415						
tr|Q9RX64	tr|Q9RX64	1537						
tr|Q9RX64	tr|Q9RX64	1537	1.05E+08	58342000	82160000			
tr|Q9RTN8	tr|Q9RTN8	905	44983000	37028000	37273000			
tr|Q9RT14	tr|Q9RT14	800	41386000	49212000	1.06E+08	1E+08	1.38E+08	1.9E+08
tr|Q9RXQ	tr|Q9RXQ	1628	10952000	3451300	3110000	3046700	5624400	5405000
sp|Q9RVC	sp|Q9RVC	277	2.25E+08	2.64E+08	2.26E+08	1.53E+08	2.32E+08	2.01E+08
sp|Q9RV6	sp|Q9RV6	266	2.83E+08	3.67E+08	2.85E+08	2.12E+08	3.71E+08	2.49E+08
sp|Q9RY2	sp|Q9RY2	411	3895900	927910	1000000	657690		760380
tr|Q9RUA	tr|Q9RUA	1023	3128000	4731900	6064100	3852100	5091800	4750800
tr|Q9RY34	tr|Q9RY34	1687						

tr|Q9RY34	tr|Q9RY34	1687	4.63E+08	3.08E+08	4.05E+08		5.11E+08	5.68E+08
tr|Q9RT97	tr|Q9RT97	843					1632600	
tr|Q9RU28	tr|Q9RU28	981	4.82E+08	3.49E+08	3.62E+08	1.21E+08	1.24E+08	1.25E+08
tr|Q9RT05	tr|Q9RT05	794	68129000	74470000	61850000	64622000	75606000	70515000
sp|Q9RSS	sp|Q9RSS	145			85868000			
sp|Q9RXJ3	sp|Q9RXJ3	378			48601000			
sp|P8358	sp|P8358	60	63916000	59031000	51291000	79717000	65106000	1.1E+08
sp|P8358	sp|P8358	60	35332000	1.09E+08	1.16E+08	88087000	1.02E+08	1.16E+08
tr|Q9RS08	tr|Q9RS08	624	18694000	11333000	30719000	31554000	25467000	45396000
sp|Q9RSN	sp|Q9RSN	138	2.16E+09	2.3E+09	2.4E+09	8.54E+08	7.24E+08	1.04E+09
sp|Q9RVI3	sp|Q9RVI3	289	1.03E+08	3.5E+08	2.79E+08	2.57E+08	91362000	2.4E+08
sp|Q9RVP	sp|Q9RVP	294				31791000		
sp|Q9RYE	sp|Q9RYE	432						
sp|Q9RV9	sp|Q9RV9	273						
tr|Q9RUY2	tr|Q9RUY2	1139	4.13E+09	3.76E+09	3.44E+09	3.24E+09	3.42E+09	3.3E+09
tr|Q9RS00	tr|Q9RS00	618						
tr|Q9RXH0	tr|Q9RXH0	1593	22935000	20285000	59157000	29613000	28226000	30030000
tr|Q9RVB2	tr|Q9RVB2	1206	5.13E+08	4.87E+08	4.52E+08	7.35E+08	7.44E+08	7.32E+08
tr|Q9RVB2	tr|Q9RVB2	1206			50272000			
tr|Q9RSB6	tr|Q9RSB6	684		2182800			1607100	
sp|Q9RVT	sp|Q9RVT	297			11223000			
tr|Q9RXB8	tr|Q9RXB8	1566	21202000	33786000	57861000	17418000	29685000	25801000
tr|Q9RYQ	tr|Q9RYQ	1793	41466000	24086000	34154000			
tr|Q9RS92	tr|Q9RS92	667			9238900			
tr|Q9RXI1	tr|Q9RXI1	1601				26455000	27261000	26545000
tr|Q9RWK	tr|Q9RWK	1425			16228000			
sp|Q9RW0	sp|Q9RW0	327	34611000	17153000	17852000	21596000	21874000	21204000
sp|Q9RW9	sp|Q9RW9	318						
tr|Q9RZ07	tr|Q9RZ07	1845	7.37E+09	6.71E+09	5.37E+09	3.72E+09	3.95E+09	4.15E+09
tr|Q9RZ07	tr|Q9RZ07	1845	3.26E+08	3.75E+08	6.04E+08	84799000	1.58E+08	1.78E+08
tr|Q9RSU4	tr|Q9RSU4	765	22097000					
tr|Q9RZD8	tr|Q9RZD8	1912	27524000				15634000	36973000
tr|Q9RW8	tr|Q9RW8	1366						
sp|Q9RSR	sp|Q9RSR	143	6.51E+08	5.52E+08	5.19E+08	3.17E+08	2.59E+08	2.88E+08
tr|Q9RS28	tr|Q9RS28	635					7632600	
tr|Q9RVX1	tr|Q9RVX1	1315	1.6E+08	1.15E+08	1.09E+08	1.37E+08	1.06E+08	1.24E+08
tr|Q9RVJ3	tr|Q9RVJ3	1240	1.22E+08	1.77E+08	1.34E+08	1.31E+08	24319000	1.29E+08
sp|Q9RSJ6	sp|Q9RSJ6	124				18052000		12373000
sp|Q9RR6	sp|Q9RR6	66						
sp|Q9RXJ0	sp|Q9RXJ0	375	1.78E+09	1.63E+09	1.32E+09	1.57E+09	1.61E+09	2.22E+09
sp|Q9RVW	sp|Q9RVW	301	1.93E+09	1.81E+09	1.69E+09	1.34E+09	1.43E+09	1.35E+09
tr|Q9RYE0	tr|Q9RYE0	1736	3E+08	2.51E+08	2.35E+08	3.17E+08	2.85E+08	3.03E+08
tr|Q9RYE0	tr|Q9RYE0	1736	24141000	16772000	30297000		4391600	25249000
tr|Q9RWL	tr|Q9RWL	1435	14576000		60932000	2.64E+08	2.55E+08	2.01E+08
tr|Q9RU27	tr|Q9RU27	980		52788000				
tr|Q9RTM	tr|Q9RTM	902			5696600			
sp|Q9RUI	sp|Q9RUI	237	8.45E+08	1.14E+09	5.96E+08	6.14E+08	6.05E+08	6.58E+08
tr|Q9RTV5	tr|Q9RTV5	938	2.83E+08		1.83E+08	1.26E+09	5.6E+08	1.12E+09
sp|Q9RWI	sp|Q9RWI	329	3.09E+09	6.43E+09	5.72E+09	4.65E+09	5.9E+09	4.57E+09
tr|Q9RSU7	tr|Q9RSU7	767	5.65E+08	4.61E+08	4.41E+08		14156000	3.06E+08
tr|Q9RSI8	tr|Q9RSI8	725						

tr|Q9RSI8	tr|Q9RSI8	725	24493000		38473000			
sp|Q9RR7	sp|Q9RR7	67	1.58E+08	1.14E+08	1.82E+08	1.09E+08	1.03E+08	1.03E+08
tr|Q9RVB2	tr|Q9RVB2	1206						
sp|Q9RZA	sp|Q9RZA	454						
tr|Q9RRD6	tr|Q9RRD6	506	37166000	28252000	28433000	15028000	14212000	13614000
tr|Q9RRA6	tr|Q9RRA6	489	8.27E+08	1.45E+09	1.03E+09	1.74E+09	1.47E+09	1.26E+09
tr|Q9RRA6	tr|Q9RRA6	489	1.18E+08	1.02E+08	4.89E+08		1.58E+08	1.93E+08
tr|Q9RRA6	tr|Q9RRA6	489	22841000					
tr|Q9RWP	tr|Q9RWP	1447	72599000	23462000	35273000	28441000	11513000	30783000
sp|O32508	sp|O32508	46	39462000	47764000	30042000	39644000	52455000	61794000
sp|O32508	sp|O32508	46						
tr|Q9RU73	tr|Q9RU73	1011				19818000		14779000
tr|Q9RRC7	tr|Q9RRC7	501	32582000	50789000	64052000			
tr|Q9RWB	tr|Q9RWB	1387	7255900	6133300	11327000	18414000	11828000	13838000
sp|Q9RU7	sp|Q9RU7	214	46570000	61510000	49790000	67396000	39305000	47815000
sp|Q9RU7	sp|Q9RU7	214			27982000			
tr|Q9RRG5	tr|Q9RRG5	521			12049000			
tr|Q9RRI3	tr|Q9RRI3	531	2.92E+09	2.78E+09	2.32E+09	2.54E+09	2.54E+09	2.58E+09
tr|Q9RZB4	tr|Q9RZB4	1897				11837000	19590000	15292000
tr|Q9RU40	tr|Q9RU40	987						
tr|Q9RW5	tr|Q9RW5	1357	19807000		18601000	63755000	6755000	42770000
tr|Q9RZH0	tr|Q9RZH0	1927	31794000	29570000	41989000	42482000	39752000	39372000
tr|Q9RR62	tr|Q9RR62	467			19864000	26522000	32495000	27952000
tr|Q9RRC2	tr|Q9RRC2	498	4E+08	2.71E+08	3.14E+08	78808000	1.02E+08	1.56E+08
tr|Q9RY75	tr|Q9RY75	1705						
tr|Q9RUG	tr|Q9RUG	1052				11787000	13204000	15817000
tr|Q9RWB	tr|Q9RWB	1385						
tr|Q9RU02	tr|Q9RU02	970					4958100	4597800
tr|Q9RYS1	tr|Q9RYS1	1801						
tr|Q9RTZ8	tr|Q9RTZ8	966					12683000	11408000
tr|Q9RS17	tr|Q9RS17	630				44386000	30338000	48862000
tr|Q9RYY7	tr|Q9RYY7	1837						
tr|Q9RYW	tr|Q9RYW	1831	30877000	32834000		30369000		36666000
tr|Q9RZK2	tr|Q9RZK2	1940	31020000	29332000	34610000	80873000	83419000	83295000
tr|Q9RYY7	tr|Q9RYY7	1837	6882000					10946000
tr|Q9RTB3	tr|Q9RTB3	847				40980000	37190000	46902000
tr|Q9RS52	tr|Q9RS52	646	1.11E+08	1.82E+08	1.51E+08	1.59E+08	1.52E+08	1.17E+08
tr|Q9RUI5	tr|Q9RUI5	1064	33266000	5.19E+08	75423000	27705000	27141000	31259000
sp|Q9RRJ5	sp|Q9RRJ5	88			24266000	36484000	32640000	35567000
tr|Q9RYZ9	tr|Q9RYZ9	1842			8788200	29292000	13715000	23220000
sp|Q9RU2	sp|Q9RU2	209	8.39E+09	8.56E+09	9.26E+09	8.21E+09	5.72E+09	6.45E+09
sp|Q9RU2	sp|Q9RU2	209			89107000	2.43E+08	2.1E+08	2.55E+08
tr|Q9RV29	tr|Q9RV29	1165	15769000	17260000	14425000	20489000		13643000
tr|Q9RVC6	tr|Q9RVC6	1212	38589000	41921000	37971000			18240000
tr|Q9RRJ7	tr|Q9RRJ7	537	31366000	14818000	24008000	9331000		13235000
tr|Q9RWS	tr|Q9RWS	1463	10720000	12359000	24066000	89913000	85417000	34532000
tr|Q9RU22	tr|Q9RU22	977	1.01E+08	1.41E+08	90818000	1.83E+08	1.7E+08	1.53E+08
tr|Q9RU22	tr|Q9RU22	977	1.21E+08	84686000	2.73E+08	41589000		42117000
tr|Q9RTK8	tr|Q9RTK8	891						
tr|Q9RSR2	tr|Q9RSR2	750		6410900	6805700			
tr|Q9LBE3	tr|Q9LBE3	466		1242000		962360	2339500	1456800

tr|Q9LBE3	tr|Q9LBE3	466	3.01E+08	2.06E+08	3.83E+08	82310000	1.42E+08	1.46E+08
sp|Q9RTD	sp|Q9RTD	178	1.44E+08	1.44E+08	1.16E+08	69534000		80359000
tr|Q9RXS7	tr|Q9RXS7	1640	1.51E+09	1.48E+09	1.27E+09	1.28E+09	1.28E+09	1.35E+09
tr|Q9RXS7	tr|Q9RXS7	1640	1.08E+08		2.49E+08	57839000	65097000	64058000
tr|Q9RW9	tr|Q9RW9	1374	38317000	56707000	31472000	2.15E+08	1.99E+08	89190000
sp|Q9RX3	sp|Q9RX3	357	41066000	33404000	29688000	49320000	42088000	44985000
tr|Q9RZ33	tr|Q9RZ33	1861						
tr|Q9RYX2	tr|Q9RYX2	1833	31116000		34803000	40207000		45105000
tr|Q9RX83	tr|Q9RX83	1548			1.3E+08			
tr|Q9RY36	tr|Q9RY36	1689						
tr|Q9RY83	tr|Q9RY83	1709	2.82E+08	2.65E+08	2.61E+08	25788000	64385000	86156000
sp|Q9RSK	sp|Q9RSK	131	3.78E+08	1.73E+08	1.8E+08	2.41E+08	2.55E+08	2.85E+08
sp|Q9RVH	sp|Q9RVH	288	2.81E+08	4.17E+08	3.74E+08	1.74E+08	2.13E+08	1.7E+08
sp|Q9RT4	sp|Q9RT4	167		9575700	8401300	15185000	17088000	16469000
sp|Q9RUF	sp|Q9RUF	232		69394000		97219000	78643000	77078000
tr|Q9RU71	tr|Q9RU71	1010	6.36E+08	8.92E+08	5.5E+08	4.86E+08	3.54E+08	4.68E+08
tr|Q9RU17	tr|Q9RU17	976	2.06E+08	1.82E+08	2.17E+08	1.67E+08	3.91E+08	2.14E+08
tr|Q9RY0C	tr|Q9RY0C	1673	7.01E+08	8.49E+08	5.4E+08	1.89E+08	2.06E+08	2.06E+08
tr|Q9RYV0	tr|Q9RYV0	1820	2.87E+08	4.52E+08	3.71E+08	7.24E+08	7.97E+08	6.26E+08
tr|Q9RYV0	tr|Q9RYV0	1820	1.12E+08	1.01E+08	2.04E+08	72690000	63794000	1.1E+08
tr|Q9RT05	tr|Q9RT05	794	1.38E+08	1.6E+08	1.78E+08	1.81E+08	1.6E+08	1.55E+08
tr|Q9RW4	tr|Q9RW4	1351	30437000					
tr|Q9RRR3	tr|Q9RRR3	568						
sp|Q9RYB	sp|Q9RYB	426			38887000			
sp|Q9RY7	sp|Q9RY7	422	5.84E+08	3.76E+08	4.27E+08	1.51E+09	1.37E+09	1.67E+09
tr|Q9RRC7	tr|Q9RRC7	501	32655000	36183000	36937000			
tr|Q9RUF6	tr|Q9RUF6	1048				27437000	19896000	21351000
tr|Q9RVN	tr|Q9RVN	1261	57493000	40380000	31301000	48731000	41085000	52682000
tr|Q9RYE8	tr|Q9RYE8	1740	3.34E+08	2.38E+08	2.29E+08	4.07E+08	4.13E+08	3.94E+08
tr|Q9RYE8	tr|Q9RYE8	1740	51806000	35466000	1.14E+08	16783000	17272000	27157000
tr|Q9RV74	tr|Q9RV74	1188			36741000			
sp|Q9RVI3	sp|Q9RVI3	289		43763000			64768000	70826000
tr|Q9RUR	tr|Q9RUR	1108	2.41E+08		1.75E+08	3.83E+08	3.39E+08	3.46E+08
tr|Q9RY92	tr|Q9RY92	1713	22496000		19286000			16938000
tr|Q9RYY7	tr|Q9RYY7	1837	1.8E+08	1.74E+08	2.74E+08	47816000	62484000	56080000
sp|Q9RW	sp|Q9RW	328					8118900	
tr|Q9RS53	tr|Q9RS53	647						
tr|Q9RWH	tr|Q9RWH	1412	49511000	33764000	30343000	7360800		
tr|Q9RVV0	tr|Q9RVV0	1304		11971000	40588000			
sp|P56925	sp|P56925	58	4.74E+08	4.76E+08	4.31E+08	3.2E+08	3.79E+08	3.97E+08
tr|Q9RU62	tr|Q9RU62	1004	2.87E+08	3.6E+08	3.13E+08	4.16E+08	3.71E+08	3.56E+08
tr|Q9RUR	tr|Q9RUR	1108	32440000	46922000	68687000		21916000	32098000
tr|Q9RXC5	tr|Q9RXC5	1570				3E+08	3.16E+08	2.98E+08
tr|Q9RYI7	tr|Q9RYI7	1763	23358000			39465000	30963000	25069000
tr|Q9RYI7	tr|Q9RYI7	1763			34778000		31757000	
sp|Q9R34	sp|Q9R34	64	4.58E+09	8.02E+09	5.94E+09	3.62E+09	5.79E+09	4.45E+09
tr|Q9RW7	tr|Q9RW7	1362		1.23E+08				
tr|Q9RWX	tr|Q9RWX	1498					14116000	7920000
tr|Q9RSL7	tr|Q9RSL7	733				20524000		
tr|Q9RUS9	tr|Q9RUS9	1115	34047000	28920000	30769000	41147000	32154000	34757000
tr|Q9RZL4	tr|Q9RZL4	1943			3785000	37786000	35616000	25910000

tr|Q9RZL7	tr|Q9RZL7	1945	91473000	18608000	1.32E+08	1.13E+08	1.22E+08	1.32E+08
sp|Q9RSG	sp|Q9RSG	118	2.06E+08		1.86E+08	1.38E+08	1.5E+08	1.6E+08
tr|Q9RUK	tr|Q9RUK	1079	70470000	64028000	53022000	39148000	50401000	43845000
tr|Q9RV1	tr|Q9RV1	1155	25081000		17534000			
sp|Q9RW	sp|Q9RW	329	2.08E+08	2.71E+08	1.89E+08		3.21E+08	3.33E+08
tr|Q9RYB	tr|Q9RYB	1722	3.33E+08	4.06E+08	3.99E+08	5.2E+08	6.71E+08	4.24E+08
sp|Q9ZNA	sp|Q9ZNA	463	4.6E+08	4.18E+08	5.1E+08	3.65E+08	3.82E+08	4.06E+08
tr|Q9RV0	tr|Q9RV0	1152	9300400	10572000	9548700	68153000	43343000	57512000
tr|Q9RZ1	tr|Q9RZ1	1852	32389000		17885000	29791000	37690000	34288000
tr|Q9RX1	tr|Q9RX1	1511		1.01E+08		79024000		75871000
sp|Q9RVT	sp|Q9RVT	297	10379000	11271000	17566000	9255300	8126200	8720300
sp|Q9RT2	sp|Q9RT2	162				45277000	62246000	55883000
tr|Q9RTU	tr|Q9RTU	935						
tr|Q9RYH	tr|Q9RYH	1757	1.13E+08	84328000	71655000	75290000	56063000	91641000
tr|Q9RXV1	tr|Q9RXV1	1650			21951000	16708000	29536000	18649000
tr|Q9RZC	tr|Q9RZC	1903	3.35E+08	2.99E+08	2.79E+08	7.82E+08	5.99E+08	6.64E+08
tr|Q9RWV	tr|Q9RWV	1486		51952000	50008000	37806000		
tr|Q9RZB	tr|Q9RZB	1899	8341400	18569000	28550000	9841700	16102000	
sp|Q9RTF	sp|Q9RTF	182	12730000	9403800				
sp|Q9RU8	sp|Q9RU8	222			11807000	59099000	43925000	49942000
tr|Q9RY1	tr|Q9RY1	1679	1.08E+08	44579000	78067000			
tr|Q9RY01	tr|Q9RY01	1674	1.24E+08	1.16E+08	91239000	59797000	67156000	63449000
tr|Q9RVN	tr|Q9RVN	1262	2.47E+08	3.3E+08	2.99E+08	22000000	1.95E+08	32971000
sp|Q9RYH	sp|Q9RYH	435						
tr|Q9RUL	tr|Q9RUL	1082	14469000	35327000	14170000		12954000	31357000
tr|Q9RVU	tr|Q9RVU	1299	3.27E+08	4.47E+08	4.64E+08	2.33E+08	3.8E+08	3.26E+08
tr|Q9RXH	tr|Q9RXH	1599	70302000	59704000	93613000	39930000	30102000	82139000
tr|Q9RWB	tr|Q9RWB	1387			16526000			
tr|Q9RW1	tr|Q9RW1	1337			3340000			
sp|Q9RTK	sp|Q9RTK	191						
tr|Q9RXP	tr|Q9RXP	1624				70895000		1.02E+08
tr|Q9RVN	tr|Q9RVN	1261	1.67E+08	1.34E+08	1.16E+08		38536000	
tr|Q9RT87	tr|Q9RT87	837	1.62E+08	1.8E+08	1.6E+08	3.81E+08	2.99E+08	2.27E+08
tr|Q9RVD	tr|Q9RVD	1214	75203000		23581000			
tr|Q9RRR	tr|Q9RRR	570			23032000			
sp|P5692	sp|P5692	59	60703000		73686000	1.97E+08	1.73E+08	1.84E+08
tr|Q9RWP	tr|Q9RWP	1449						
tr|Q9RSP	tr|Q9RSP	745	11368000	22215000	23812000			
tr|Q9RUX	tr|Q9RUX	1134		89917000	97860000	1.2E+08	1.08E+08	1.13E+08
sp|Q9RRL	sp|Q9RRL	90	36043000	87301000	1.07E+08	95391000	1.35E+08	55097000
tr|Q9RWX	tr|Q9RWX	1497			845950			
tr|Q9RU6	tr|Q9RU6	1006	1.09E+08	99624000	99688000			
sp|Q9RYR	sp|Q9RYR	442						
tr|Q9RUQ	tr|Q9RUQ	1103	2.35E+08	2.05E+08	1.9E+08			
tr|Q9RUQ	tr|Q9RUQ	1103	4.29E+08	3.08E+08	4.31E+08		49931000	85428000
tr|Q9RWK	tr|Q9RWK	1428	32458000	42464000	40325000	1.11E+08	1.19E+08	1.06E+08
sp|Q9RYE	sp|Q9RYE	432	3.51E+08	3.12E+08	2.73E+08	31363000	4.59E+08	3.96E+08
tr|Q9RTS5	tr|Q9RTS5	927			25838000			
sp|Q9RRL	sp|Q9RRL	90		16270000				
tr|Q9RYT	tr|Q9RYT	1815				41177000	25766000	31520000
tr|Q9RZ24	tr|Q9RZ24	1856	16640000	14787000	16866000			

tr|Q9RZ97	tr|Q9RZ97	1885						
sp|Q9RVP	sp|Q9RVP	293	15713000	14240000	22721000		6706500	9675800
tr|Q9RWL	tr|Q9RWL	1437	1.13E+08	1.23E+08	1.42E+08	2.05E+08	1.76E+08	1.31E+08
tr|Q9RZP2	tr|Q9RZP2	1952	9232700	8030500	27564000	11395000	11845000	6305400
tr|Q9RWT	tr|Q9RWT	1465	1.42E+08	1.45E+08	1.27E+08	2.35E+08	2.15E+08	2.45E+08
tr|Q9RWT	tr|Q9RWT	1465	1.21E+08	1.8E+08	1.57E+08	2.23E+08		1.47E+08
sp|Q9RTD	sp|Q9RTD	178						
tr|Q9RTK8	tr|Q9RTK8	891			13144000	20546000		46786000
tr|Q9RVB0	tr|Q9RVB0	1204	1.21E+08	1.57E+08	82107000	63104000	70046000	78904000
tr|Q9RSD0	tr|Q9RSD0	692				18017000		
tr|Q9RSD0	tr|Q9RSD0	692	5.18E+08	4.89E+08	5.68E+08	3.77E+08	3.79E+08	5.86E+08
tr|Q9RSD4	tr|Q9RSD4	696	4.34E+08	4.14E+08	3.87E+08	3.08E+08	2.91E+08	3.36E+08
tr|Q9RYD9	tr|Q9RYD9	1735	3.19E+08	2.96E+08	2.77E+08	2.34E+08	1.61E+08	1.91E+08
tr|Q9RYD9	tr|Q9RYD9	1735				78289000	46933000	38833000
tr|Q9RSZ3	tr|Q9RSZ3	789	47793000	71576000	67363000	59218000	65754000	74050000
tr|Q9RYP9	tr|Q9RYP9	1787	9493000	4850600	6350400	25305000	26839000	61243000
tr|Q9RSV7	tr|Q9RSV7	772						
tr|Q9RXV6	tr|Q9RXV6	1652	88661000	1.01E+08	72970000	58862000	70689000	61916000
sp|Q9RUP	sp|Q9RUP	243		36905000	19366000			
tr|Q9RX29	tr|Q9RX29	1517	8.5E+08		6.78E+08	7.61E+08	5.53E+08	6.41E+08
tr|Q9RRI1	tr|Q9RRI1	530	3.28E+08	2.81E+08	1.96E+08	2.5E+08	2.35E+08	2.03E+08
tr|Q9RV67	tr|Q9RV67	1184	62827000	40931000	49281000	91872000	75738000	98398000
tr|Q9RYW	tr|Q9RYW	1829	85770000	69253000	84527000	60097000	74497000	52528000
sp|Q9RZV	sp|Q9RZV	460	10536000	10238000	15050000		31783000	20575000
tr|Q9RU41	tr|Q9RU41	988						
tr|Q9RU40	tr|Q9RU40	987			5271100			
sp|Q9RV9	sp|Q9RV9	271	1.31E+09	1.47E+09	1.1E+09	1.48E+09	1.85E+09	1.96E+09
tr|Q9RTI9	tr|Q9RTI9	882						
tr|Q9RZH0	tr|Q9RZH0	1927	2.61E+08	3.13E+08	15928000			11229000
tr|Q9RYC2	tr|Q9RYC2	1724		29116000		25373000	17715000	22349000
tr|Q9RYZ9	tr|Q9RYZ9	1842	24996000	14413000	11761000	11079000	17011000	15523000
sp|Q9RV6	sp|Q9RV6	266	5.03E+08	4.28E+08	3.99E+08	4.27E+08	4.21E+08	4.72E+08
tr|Q9RVW	tr|Q9RVW	1311	1.97E+08	2.46E+08	2.16E+08	3.08E+08	1.89E+08	2.5E+08
tr|Q9RVW	tr|Q9RVW	1311	32399000		12203000			
tr|Q9RVA3	tr|Q9RVA3	1200		20742000				
tr|Q9RZQ	tr|Q9RZQ	1960		1.24E+08				
tr|Q9RV67	tr|Q9RV67	1184		42307000				
tr|Q9RRA2	tr|Q9RRA2	485	3.48E+08	4.23E+08	4.07E+08	1.89E+09	1.26E+09	1.4E+09
tr|Q9RXE6	tr|Q9RXE6	1577						
tr|Q9RSX1	tr|Q9RSX1	777	96173000	94420000	68719000	1.09E+08	1.63E+08	1.26E+08
tr|Q9RRZ1	tr|Q9RRZ1	612	71669000	56320000	57457000	16416000		35396000
tr|Q9RRZ5	tr|Q9RRZ5	614	16743000	17371000	15763000	19240000	15026000	11980000
tr|Q9RVR2	tr|Q9RVR2	1281						
sp|Q9RXW	sp|Q9RXW	404			26942000	41827000	46369000	37169000
sp|Q9RSZ	sp|Q9RSZ	159	73967000	2.04E+08	46138000	2.64E+08	1.84E+08	3.37E+08
sp|Q9RS3	sp|Q9RS3	110	2.28E+08	2.46E+08	2.89E+08	92843000	1.1E+08	1.46E+08
tr|Q9RWB	tr|Q9RWB	1387		29578000				
sp|P52027	sp|P52027	51	69787000	56634000	54509000	97146000	97015000	87506000
sp|Q9RVA	sp|Q9RVA	274	3.24E+08	3.15E+08	2.9E+08	1.16E+08	1.76E+08	1.33E+08
sp|Q9RV2	sp|Q9RV2	263	1.07E+08	87688000	1.17E+08	28999000	20425000	27864000
sp|Q9RWE	sp|Q9RWE	320	90103000	95428000	1.89E+08	1.44E+08	1.7E+08	1.62E+08

tr|Q9RVG4	tr|Q9RVG4	1227	1.48E+08	1.15E+08	1.04E+08		22531000	43312000
sp|Q9RWU	sp|Q9RWU	347		22730000	22404000	37044000	33708000	27213000
sp|Q9RU1	sp|Q9RU1	206	1.09E+08	1.39E+08	93794000	93816000	1.27E+08	99542000
tr|Q9RVB0	tr|Q9RVB0	1204	33059000			21440000	23319000	26739000
tr|Q9RXI3	tr|Q9RXI3	1602	1.19E+08	87577000	1.19E+08		35247000	
tr|Q9RW8	tr|Q9RW8	1368						7617900
tr|Q9RUZ5	tr|Q9RUZ5	1146			49564000	55181000	46804000	53860000
sp|Q9RSS	sp|Q9RSS	145	35848000	32159000				
sp|Q9RX2	sp|Q9RX2	355	1.85E+08	1.21E+08	2.05E+08	52792000	40850000	63783000
tr|Q9RZQ	tr|Q9RZQ	1959	39793000	41679000	27347000	43386000	49020000	53576000
tr|Q9RZQ	tr|Q9RZQ	1959				24054000	25328000	23207000
tr|Q9RWR	tr|Q9RWR	1456	1.41E+08					
tr|Q9RST9	tr|Q9RST9	761			24357000			
tr|Q9RTX6	tr|Q9RTX6	951	73037000	54494000	68713000	80459000		60564000
sp|Q9RUV	sp|Q9RUV	254	1.13E+08	1.29E+08	1.26E+08	5.32E+08	60388000	2.59E+08
sp|Q9RYM	sp|Q9RYM	439		36731000	5.87E+08	1.76E+09	1.99E+09	1.76E+09
tr|Q9RWX	tr|Q9RWX	1497	82088000	51688000	35640000		16481000	
tr|Q9RZR2	tr|Q9RZR2	1961						
tr|Q9RT50	tr|Q9RT50	819	75036000	77872000	56718000	70294000	71053000	89841000
tr|Q9RYS7	tr|Q9RYS7	1806	10286000	6043900	6184100			
tr|Q9RT29	tr|Q9RT29	806	80054000	67236000	78958000			43373000
tr|Q9RWP	tr|Q9RWP	1449	1.46E+08	67860000	1.66E+08	1.76E+08	40324000	1.04E+08
tr|Q9RUZ9	tr|Q9RUZ9	1150		11472000	14603000			
tr|Q9RZJ1	tr|Q9RZJ1	1938						
sp|Q9RX9	sp|Q9RX9	364						
tr|Q9RWR	tr|Q9RWR	1458					19295000	
tr|Q9RXA0	tr|Q9RXA0	1555						
tr|Q9RT14	tr|Q9RT14	800	15258000	31900000	25402000	22854000	43367000	35795000
tr|Q9RRY5	tr|Q9RRY5	608						
tr|Q9RYD9	tr|Q9RYD9	1735	1.28E+09	1.15E+09	1.02E+09	2.52E+09	1.96E+09	2.3E+09
tr|Q9RUC	tr|Q9RUC	1038	60925000	66918000		92923000	75429000	74671000
tr|Q9RXX9	tr|Q9RXX9	1663	47368000	31597000	40744000		26345000	
tr|Q9RTW	tr|Q9RTW	944	1.52E+08	1.37E+08	98299000		41401000	35879000
tr|Q9RX13	tr|Q9RX13	1510	19719000			80398000	93131000	43967000
tr|Q9RYP4	tr|Q9RYP4	1783	75530000	74178000	54984000		1.07E+08	88472000
sp|Q9RXG	sp|Q9RXG	370	1.04E+09	1.01E+09	7.86E+08	5.09E+08	5.68E+08	5.37E+08
sp|Q9RYD	sp|Q9RYD	431	5.06E+08	4.39E+08	3.17E+08	3.24E+08	4.05E+08	3.57E+08
tr|Q9RT46	tr|Q9RT46	817	85909000	74514000	76008000	59358000	71467000	40996000
tr|Q9RUY8	tr|Q9RUY8	1143	4.79E+08	5.44E+08	4.33E+08	4.3E+08	5.45E+08	2.29E+08
tr|Q9RYL1	tr|Q9RYL1	1768	1.46E+08	1.33E+08	1.4E+08	2.88E+08	2.82E+08	2.38E+08
tr|Q9RVY2	tr|Q9RVY2	1320						
tr|Q9RVB1	tr|Q9RVB1	1205	47750000	35830000	32471000	38937000	47524000	54626000
tr|Q9RUM	tr|Q9RUM	1089	6.79E+08	5.67E+08	4.74E+08	5.22E+08	5.32E+08	
sp|Q9RSS	sp|Q9RSS	147	2.65E+09	2.98E+09	2.57E+09	2.9E+09	2.57E+09	2.95E+09
tr|Q9RY95	tr|Q9RY95	1714		15171000		11525000		
sp|Q9RT3	sp|Q9RT3	166	4.89E+08	3.1E+08	3.35E+08		6.92E+08	8.64E+08
tr|Q9RVK5	tr|Q9RVK5	1245	45206000	77236000	43318000	48852000	41410000	50700000
tr|Q9RS26	tr|Q9RS26	634	26279000	23145000	20601000		13021000	11621000
tr|Q9RY55	tr|Q9RY55	1697	38093000	37402000	26643000		48096000	39320000
tr|Q9RY08	tr|Q9RY08	1678	1.31E+08	1.57E+08	1.24E+08	1.29E+08	1.47E+08	1.11E+08
tr|Q9RWT	tr|Q9RWT	1465	1.17E+09	9.19E+08	8.86E+08	3.5E+08	4.17E+08	3.9E+08

sp|Q9RTY	sp|Q9RTY	204	9141800	11143000	25973000	41556000		5712300
tr|Q9RWU	tr|Q9RWU	1474	5.7E+08	4.3E+08	5.99E+08	2.07E+08	2.8E+08	2.07E+08
tr|Q9RXS0	tr|Q9RXS0	1637	2.61E+08	1.7E+08	1.42E+08	87176000	1.47E+08	72000000
tr|Q9RRE3	tr|Q9RRE3	511	78473000	84045000	64330000	1.12E+08	1.12E+08	99347000
tr|Q9RS29	tr|Q9RS29	636	1.12E+09	1.03E+09	7.66E+08	5.24E+08	5.94E+08	5.61E+08
tr|Q9RU62	tr|Q9RU62	1004						
tr|Q9RT08	tr|Q9RT08	797	15439000	15563000	24873000			
sp|Q9RSL4	sp|Q9RSL4	136	53882000	76549000	1.42E+08		31959000	31311000
tr|Q9RT87	tr|Q9RT87	837	1.15E+09	1.44E+09	9.8E+08	1.01E+09	9.38E+08	1.21E+09
tr|Q9RSI1	tr|Q9RSI1	721						30451000
tr|Q9RZP9	tr|Q9RZP9	1956	87842000	32334000	1.22E+08	4.88E+08	2.45E+08	
tr|Q9RU08	tr|Q9RU08	973	7.81E+08	8.41E+08	6.36E+08	5.92E+08	5.8E+08	5.41E+08
tr|Q9RVW	tr|Q9RVW	1311		25794000		34940000	50121000	36977000
tr|Q9RZE6	tr|Q9RZE6	1916	1.5E+08	1.18E+08	1.03E+08			
tr|Q9RS05	tr|Q9RS05	622						
tr|Q9RU78	tr|Q9RU78	1014		27140000	33215000			
sp|Q9RV2	sp|Q9RV2	264	1.66E+08	1.92E+08	1.78E+08	1.47E+08	1.53E+08	1.5E+08
tr|Q9RRI7	tr|Q9RRI7	534	58394000	63743000	57582000	48041000	56051000	45549000
tr|Q9RUR6	tr|Q9RUR6	1108	1.38E+08	1.3E+08	1.05E+08	1.18E+08	2.17E+08	1.06E+08
sp|Q9RSJ9	sp|Q9RSJ9	127	1.46E+09	1.92E+09	1.7E+09	1.91E+09	2.01E+09	1.81E+09
sp|Q9RSJ9	sp|Q9RSJ9	127			34383000			
tr|Q9RSY9	tr|Q9RSY9	787	44094000	41405000	40163000	57651000	1.7E+08	91339000
tr|Q9RSY9	tr|Q9RSY9	787			29794000			
sp|Q4657	sp|Q4657	61	59694000	53179000	53444000			33377000
tr|Q9RTZ8	tr|Q9RTZ8	966			33457000		52828000	41785000
tr|Q9RZQ	tr|Q9RZQ	1957					16714000	
sp|Q9RYM	sp|Q9RYM	438	21656000	22484000	24630000	25489000	40522000	28110000
sp|Q9RYM	sp|Q9RYM	438	25936000	21317000	41746000	19144000	36857000	25935000
sp|Q9RYM	sp|Q9RYM	438			1.39E+08	57556000	2.22E+08	78047000
sp|Q9RYH	sp|Q9RYH	435			95136000			34043000
tr|Q9RWR	tr|Q9RWR	1457					16495000	19257000
tr|Q9RXA6	tr|Q9RXA6	1559		11460000				
tr|Q9RVJ3	tr|Q9RVJ3	1240	37089000	41572000	42859000	12254000	17727000	
sp|Q9RTJ5	sp|Q9RTJ5	190		31351000	20597000		45725000	
tr|Q9RXD7	tr|Q9RXD7	1574			27518000			
tr|Q9RVK7	tr|Q9RVK7	1247						
tr|Q9RRV8	tr|Q9RRV8	593	7.12E+08	1E+09	1.03E+09	25001000	92816000	55367000
sp|P42443	sp|P42443	49						
sp|Q9RVW	sp|Q9RVW	301			36538000			
tr|Q9RUA	tr|Q9RUA	1021	1.75E+08	3.3E+08	3.72E+08	76454000	1.22E+08	1.15E+08
sp|Q9RW7	sp|Q9RW7	314			31661000		46387000	34335000
sp|O3250	sp|O3250	45		14231000	26692000	31905000	24461000	
tr|Q9RXM	tr|Q9RXM	1615	19566000		17823000	8748600		
tr|Q9RYH8	tr|Q9RYH8	1757	1.2E+08	86268000	85739000		35124000	31862000
sp|Q9RRM	sp|Q9RRM	91			5543400			
tr|Q9RUS4	tr|Q9RUS4	1112	3909300	2879300	2426400	12487000		16305000
sp|Q9RV8	sp|Q9RV8	270	9484900	12523000	9784000	11836000	28405000	14697000
tr|Q9RVB2	tr|Q9RVB2	1206	1.1E+08	77462000	74516000	1.53E+08	1.42E+08	1.48E+08
tr|Q9RUZ5	tr|Q9RUZ5	1146	1.15E+08	96037000	1.04E+08	1.76E+08	1.5E+08	1.72E+08
tr|Q9RX07	tr|Q9RX07	1506					16595000	
sp|Q9RU3	sp|Q9RU3	210	14526000			24406000		

tr|Q9RWB	tr|Q9RWB	1387	45490000	41499000				
tr|Q9RYN3	tr|Q9RYN3	1777		20734000	70033000	57591000	68633000	53853000
tr|Q9RVW	tr|Q9RVW	1310	66119000	55231000	70079000	76089000	68837000	1.31E+08
tr|Q9RVW	tr|Q9RVW	1310	41286000		70335000	50916000	64806000	64978000
tr|Q9RY02	tr|Q9RY02	1675	1.8E+09	1.76E+09	1.42E+09	8.67E+08	1.09E+09	1.02E+09
sp|Q9RU7	sp|Q9RU7	215	1.03E+08	1.2E+08	91770000	2.2E+08	1.98E+08	2.06E+08
tr|Q9RXY9	tr|Q9RXY9	1667	17469000	23853000	21095000			
tr|Q9RZA9	tr|Q9RZA9	1893					6155000	
tr|Q9RRK8	tr|Q9RRK8	542						
tr|Q9RUR0	tr|Q9RUR0	1108	2.25E+08	2.03E+08	1.98E+08	78915000	83057000	1.03E+08
sp|Q9RVW	sp|Q9RVW	301	2520800	13911000	1530200	2346400	2616600	
tr|Q9RYT1	tr|Q9RYT1	1810	8395100		14033000			
tr|Q9RVS1	tr|Q9RVS1	1287	2.98E+08	3.52E+08	2.77E+08	94555000	1.15E+08	98233000
sp|Q9RV9	sp|Q9RV9	272	1.18E+08	1.99E+08	1.86E+08	1.77E+08	2.21E+08	1.52E+08
sp|Q9RRJ	sp|Q9RRJ	87	1.28E+08	1.12E+08	1.74E+08	48526000	64406000	93333000
tr|Q9RVR7	tr|Q9RVR7	1285	4862900		5308200			
sp|Q9RUA	sp|Q9RUA	224	1.09E+08	1.18E+08	88063000		59665000	
sp|Q9RYM	sp|Q9RYM	439	1.79E+08	2.27E+08	3.75E+08			
tr|Q9RVK8	tr|Q9RVK8	1248		2.05E+08	1.53E+08	79989000	1.05E+08	
tr|Q9RY62	tr|Q9RY62	1700				42283000	47018000	34947000
sp|Q9RT6	sp|Q9RT6	170						
tr|Q9RUR4	tr|Q9RUR4	1107	2.86E+08	4.97E+08	4.25E+08	1.51E+08	2.16E+08	2.06E+08
tr|Q9RZU7	tr|Q9RZU7	1979	48436000	64051000	1.05E+08			
sp|Q9RY0	sp|Q9RY0	410						
tr|Q9RST1	tr|Q9RST1	757				12815000	10181000	13107000
sp|Q9RUN	sp|Q9RUN	242	4.92E+08	6.3E+08	5.22E+08	1E+09	1.02E+09	9E+08
tr|Q9RU62	tr|Q9RU62	1004						
tr|Q9RYZ6	tr|Q9RYZ6	1840	3.83E+08	3.09E+08	3.01E+08	2.94E+08	4.11E+08	2.98E+08
sp|Q9RUF	sp|Q9RUF	231	2.45E+08	4.81E+08	3.92E+08	5.22E+08	5.93E+08	5.37E+08
tr|Q9RZU0	tr|Q9RZU0	1976					44763000	
tr|Q9RV14	tr|Q9RV14	1158						46122000
sp|Q9RYZ	sp|Q9RYZ	449						
tr|Q9RUK7	tr|Q9RUK7	1079						9674300
tr|Q9RV99	tr|Q9RV99	1198						
tr|Q9RXG7	tr|Q9RXG7	1590		57944000				
sp|Q9RY0	sp|Q9RY0	410						
sp|P56905	sp|P56905	57				79062000		
tr|Q9RXB8	tr|Q9RXB8	1566	5.04E+08	4.18E+08	3.93E+08	8.79E+08	7.67E+08	6.96E+08
tr|Q9RXB8	tr|Q9RXB8	1566	70235000	74894000	97741000			
tr|Q9RX47	tr|Q9RX47	1525	7318900	12898000		13777000		13232000
sp|Q9RSS	sp|Q9RSS	148	2.21E+08	1.99E+08	2.33E+08		89275000	
tr|Q9RWW	tr|Q9RWW	1493	50632000	46849000	45989000	63335000	66092000	52556000
sp|O8302	sp|O8302	47			6411700		31550000	35283000
tr|Q9RSY9	tr|Q9RSY9	787	36595000	23678000	22195000	54158000	84366000	68167000
sp|Q9RYM	sp|Q9RYM	438		16454000	28433000	79831000	38234000	88066000
tr|Q9RWP	tr|Q9RWP	1449	15552000	16452000	22521000	16911000	19922000	21851000
tr|Q9RVK1	tr|Q9RVK1	1244	10485000	10009000	7466900	34930000	42704000	31154000
sp|Q9RU7	sp|Q9RU7	215						
tr|Q9RRG4	tr|Q9RRG4	520			7799700		17795000	
tr|Q9RRS3	tr|Q9RRS3	574	25952000	26280000	26234000	25037000	35164000	26217000
tr|Q9RUU	tr|Q9RUU	1121	49603000	58325000	56858000		27588000	

sp|Q9RX1	sp|Q9RX1	351	1.22E+08		2.32E+08	1.35E+08		
tr|Q9RZU	tr|Q9RZU	1979	88576000	42586000	1.05E+08			
tr|Q9RTL6	tr|Q9RTL6	897		17725000		43559000	48621000	30950000
sp|Q9RX8	sp|Q9RX8	362					13403000	9404300
sp|Q9RRC	sp|Q9RRC	79	16441000	11220000	8331300	11282000	16907000	9085700
sp|Q9RSR	sp|Q9RSR	144	79138000	37804000	50668000	18538000		
tr|Q9RVS4	tr|Q9RVS4	1289	25352000	20753000	18845000	28906000	36947000	
tr|Q9RYQ	tr|Q9RYQ	1792				1.63E+08		1.3E+08
tr|Q9RYQ	tr|Q9RYQ	1792		26671000	30523000	1.5E+08	1.34E+08	1.77E+08
sp|Q9RX5	sp|Q9RX5	359						
tr|Q9RS03	tr|Q9RS03	620			30886000			
tr|Q9RVZ2	tr|Q9RVZ2	1325	3.23E+08	3.44E+08	3.35E+08	2.53E+08	4.23E+08	2.83E+08
tr|Q9RUI3	tr|Q9RUI3	1062	17986000	15329000	40281000	44807000	27707000	57833000
tr|Q9RW6	tr|Q9RW6	1360		28128000				
tr|Q9RXH	tr|Q9RXH	1596	6931000	11939000	25190000	88572000	80478000	33506000
tr|Q9RYM	tr|Q9RYM	1773	2.24E+08	2.43E+08	2E+08	5.42E+08	5.74E+08	4.27E+08
sp|Q9RWI	sp|Q9RWI	330			15258000			
tr|Q9RUE	tr|Q9RUE	1044	2.09E+09	2.15E+09	1.7E+09	3.9E+09	3.75E+09	3.6E+09
tr|Q9RUE	tr|Q9RUE	1044	90583000	5.48E+08	6.1E+08	1.97E+08		3.78E+08
tr|Q9RW1	tr|Q9RW1	1337		16447000	14979000		19686000	34077000
sp|Q9RW	sp|Q9RW	326	8.24E+08	6.83E+08	9.67E+08	3.91E+08	72948000	7.14E+08
tr|Q9RRA	tr|Q9RRA	483				64083000	75950000	
tr|Q9RRA	tr|Q9RRA	483			23036000			
sp|Q9RUP	sp|Q9RUP	246	73506000	58905000	61765000	2.48E+08	2.44E+08	1.99E+08
tr|Q9RTF2	tr|Q9RTF2	867		26211000				
tr|Q9RZJ1	tr|Q9RZJ1	1938						
tr|Q9RWP	tr|Q9RWP	1448				87678000	1.37E+08	92167000
tr|Q9RYQ	tr|Q9RYQ	1793		8018100	5589900	13048000	11439000	10671000
tr|Q9RTP3	tr|Q9RTP3	908	33340000	28379000	30973000	21038000	25672000	
sp|Q9RUP	sp|Q9RUP	245	4.35E+08	3.21E+08	3.54E+08	2.54E+08	2.63E+08	2.05E+08
tr|Q9RT14	tr|Q9RT14	800	74299000	46301000	83257000		4413100	
tr|Q9RW2	tr|Q9RW2	1341	2276900		2675400	23816000	15244000	8066900
sp|Q9RSZ	sp|Q9RSZ	159		13587000	26626000	1.76E+08	65471000	1.12E+08
tr|Q9RVR2	tr|Q9RVR2	1281	2.68E+08	2.73E+08	2.41E+08	6.63E+08	5.77E+08	4.51E+08
tr|Q7DF71	tr|Q7DF71	464	6111200		5216500			
tr|Q9RTZ3	tr|Q9RTZ3	963						
sp|Q9RW	sp|Q9RW	326	38841000	15673000	12232000	11197000	19439000	18703000
tr|Q9RYG	tr|Q9RYG	1748	26021000	28206000	26917000		41371000	
tr|Q9RYQ	tr|Q9RYQ	1788	1.25E+08	1.01E+08	1.31E+08		28310000	
sp|Q9RWI	sp|Q9RWI	320					2.07E+08	
sp|Q9RSI	sp|Q9RSI	122	78015000	83064000	67761000	1.42E+08	1.39E+08	1.09E+08
sp|Q9RTJ3	sp|Q9RTJ3	189	2.21E+09	1.77E+09	1.84E+09	1.59E+09	1.83E+09	1.53E+09
tr|Q9RXL1	tr|Q9RXL1	1609	18510000		27342000	16849000	13436000	9220100
tr|Q9RW2	tr|Q9RW2	1342			55834000			
sp|Q4657	sp|Q4657	62						
tr|Q9RTA2	tr|Q9RTA2	844	14865000	9156600	8853700	29096000	21828000	20780000
sp|Q9RTA	sp|Q9RTA	175	11583000		7599500		23673000	
tr|Q9RZU	tr|Q9RZU	1976			44177000	1.51E+08		2.52E+08
tr|Q9RU5	tr|Q9RU5	1000	5.34E+08	4.99E+08	4.13E+08	1.97E+08	5.97E+08	3.77E+08
sp|Q9RVQ	sp|Q9RVQ	295						
tr|Q9RYG	tr|Q9RYG	1748	22641000					22785000

tr|Q9RSN3	tr|Q9RSN3	740						
tr|Q9RR75	tr|Q9RR75	472						21793000
sp|Q9RT9	sp|Q9RT9	172	3.38E+08	4.56E+08	3.87E+08	2.26E+08	3.09E+08	2.28E+08
sp|Q9RRT	sp|Q9RRT	97				9384400		
tr|Q9RRS5	tr|Q9RRS5	576						
tr|Q9RXI9	tr|Q9RXI9	1606	27026000			24320000		
sp|O8302	sp|O8302	47	70296000			64768000	54489000	64295000
tr|Q9RR95	tr|Q9RR95	479					99530000	1.19E+08
sp|Q9RSQ	sp|Q9RSQ	141	96906000			2.31E+08		
sp|Q9RRL	sp|Q9RRL	90	4.39E+08	4.67E+08	3.65E+08	6.97E+08	6.66E+08	6.43E+08
tr|Q9RWW	tr|Q9RWW	1490						
tr|Q9RZC3	tr|Q9RZC3	1902	4.53E+08	5.07E+08	3.99E+08	3.58E+08	3.61E+08	3.55E+08
tr|Q9RYG3	tr|Q9RYG3	1748;1749	14831000	31334000	22633000			
sp|Q9RY3	sp|Q9RY3	413	1.55E+08	1.5E+08	1.52E+08	2.67E+08	1.93E+08	2.87E+08
tr|Q9RTR4	tr|Q9RTR4	923	3.1E+08	4.26E+08	4.29E+08	8.46E+08	8.16E+08	8.66E+08
tr|Q9RWX	tr|Q9RWX	1499	39221000	33230000	34172000			
tr|Q9RWX	tr|Q9RWX	1499						
sp|Q9RSQ	sp|Q9RSQ	140						
tr|Q9RRZ1	tr|Q9RRZ1	612	16124000					
tr|Q9RZ09	tr|Q9RZ09	1847	96909000	82788000	77732000	46112000	42559000	49153000
tr|Q9RZ09	tr|Q9RZ09	1847	1.43E+08	2.16E+08	1.37E+08			
tr|Q9RRY3	tr|Q9RRY3	606						
tr|Q9RRY3	tr|Q9RRY3	606						
tr|Q9RYG5	tr|Q9RYG5	1750						
tr|Q9RYH9	tr|Q9RYH9	1758						
sp|Q9RVB	sp|Q9RVB	275	35925000	48880000	45075000	67797000	66066000	81387000
tr|Q9RX33	tr|Q9RX33	1519	17569000	14588000	15660000	20333000	25593000	18559000
tr|Q9RX33	tr|Q9RX33	1519						
tr|Q9RUL4	tr|Q9RUL4	1085				37581000	27532000	
tr|Q9RUL4	tr|Q9RUL4	1085				14550000		
tr|Q9RXW	tr|Q9RXW	1656						34897000
sp|Q9RTA	sp|Q9RTA	175	11044000		15537000	10388000	14057000	19055000
sp|Q9RZ0	sp|Q9RZ0	451				75015000		67150000
tr|Q9RT53	tr|Q9RT53	821						
tr|Q9RWC	tr|Q9RWC	1393					23144000	
tr|Q9RUS7	tr|Q9RUS7	1114	25417000	17357000	49943000		20356000	40561000
tr|Q9RYI7	tr|Q9RYI7	1763	5.3E+08	7E+08	6.77E+08	3.9E+08	88680000	4.07E+08
tr|Q9RRV7	tr|Q9RRV7	592	1692600	441010	627270		810500	772340
tr|Q9RRZ5	tr|Q9RRZ5	614	14558000					
sp|Q9RXB	sp|Q9RXB	365						
tr|Q9RUJ5	tr|Q9RUJ5	1071	7131200	3252500	3195200	2912000	3081100	3462100
tr|Q9RWP	tr|Q9RWP	1449		19762000		26084000	28506000	15161000
tr|Q9RX37	tr|Q9RX37	1521	90506000	94478000	1.08E+08	1.13E+08	84918000	96896000
sp|Q9RVI3	sp|Q9RVI3	289						
tr|Q9RWQ	tr|Q9RWQ	1454	60768000	74814000	62361000	2.01E+08	1.55E+08	1.2E+08
tr|Q9RUJ8	tr|Q9RUJ8	1073		11567000	27169000			
sp|Q9RUR	sp|Q9RUR	249;1109	3.5E+09	3.01E+09	2.42E+09	4.63E+09	3.42E+09	3.58E+09
sp|Q9RSS	sp|Q9RSS	148	87181000	25038000	23339000	18967000	32724000	35725000
sp|Q9RYR	sp|Q9RYR	442	76489000	1.05E+08	76638000	82054000	1.06E+08	87031000
tr|Q9RW2	tr|Q9RW2	1344	1.96E+08	2.28E+08	3.21E+08	2.05E+08		3.43E+08
tr|Q9RUS1	tr|Q9RUS1	1111	13368000	20995000	20100000		19678000	

tr|Q9RZ03	tr|Q9RZ03	1843		10213000		25493000	19751000	19826000
tr|Q9RX33	tr|Q9RX33	1519						
tr|Q9RUA	tr|Q9RUA	1025		3245500	5074900			
tr|Q9RVS1	tr|Q9RVS1	1287	2614100			1232000		1403000
sp|P56925	sp|P56925	58	84394000	15065000	26239000			4977800
tr|Q9RXQ	tr|Q9RXQ	1631	11220000	16238000	9503300		9491400	
sp|Q9RZ0	sp|Q9RZ0	450		16974000				
tr|Q9RYE8	tr|Q9RYE8	1740	26698000	36659000	25724000	66204000	89162000	52350000
tr|Q9RZV2	tr|Q9RZV2	1982				36809000	23617000	25794000
tr|Q9RT07	tr|Q9RT07	796	5190400		21973000		30778000	
tr|Q9RTJ1	tr|Q9RTJ1	883	13273000		14983000	2.15E+08	82812000	
tr|Q9RZ85	tr|Q9RZ85	1877	8550700	13245000	39699000			
tr|Q9RUU	tr|Q9RUU	1124	60760000	52427000	51522000	2.42E+08	1.66E+08	2.59E+08
tr|Q9RRA7	tr|Q9RRA7	490						
tr|Q9RYE8	tr|Q9RYE8	1740	3.91E+08	4.14E+08	3.19E+08	4.27E+08	4.16E+08	4.75E+08
tr|Q9RYE8	tr|Q9RYE8	1740	18797000	42073000	31542000			
tr|Q9RU07	tr|Q9RU07	972	35565000	74424000	1.74E+08	1.29E+08	1.12E+08	1.37E+08
sp|Q9RWN	sp|Q9RWN	336			14072000		14249000	15270000
tr|Q9RZL6	tr|Q9RZL6	1944				1573900	1781700	2434900
tr|Q9RRV2	tr|Q9RRV2	589	59912000	39277000	1.3E+08			
tr|Q9RWT	tr|Q9RWT	1468			884750		1985000	1914200
tr|Q9RST2	tr|Q9RST2	758			24166000			
tr|Q9RTH3	tr|Q9RTH3	876		26014000	64009000			
sp|Q9RW0	sp|Q9RW0	342				12446000	13984000	12387000
tr|Q9RTR5	tr|Q9RTR5	924						
tr|Q9RUK9	tr|Q9RUK9	1081			30640000	78409000	1.05E+08	1.29E+08
tr|Q9RY02	tr|Q9RY02	1675				47897000	75118000	43724000
sp|Q9RXK	sp|Q9RXK	389			17360000	34875000	21246000	35630000
tr|Q9RS25	tr|Q9RS25	633					18669000	
tr|Q9RTX7	tr|Q9RTX7	952		45180000	42638000	25348000		
tr|Q9RTX7	tr|Q9RTX7	952		56034000	87542000			
tr|Q9RW7	tr|Q9RW7	1362	5.37E+08	4.35E+08	3.48E+08	5.74E+08	7.3E+08	5.59E+08
tr|Q9RTX7	tr|Q9RTX7	952	8186800	3522100	3456000	3424800	3386700	4179500
tr|Q9RTX7	tr|Q9RTX7	952	1631100	655160	1059500	1248300	767880	836520
sp|Q9RTD	sp|Q9RTD	179	1.01E+08	79195000	1.08E+08	64636000	44425000	50239000
sp|Q9RY2	sp|Q9RY2	411	89492000	2.29E+08	1.24E+08	1.69E+08	1.36E+08	1.47E+08
tr|Q9RU02	tr|Q9RU02	970	57221000	48832000	77418000	1.27E+08	1.47E+08	1.1E+08
tr|Q9RZD1	tr|Q9RZD1	1907	1.9E+08	1.71E+08	1.86E+08	58013000	51806000	57237000
tr|Q9RUK0	tr|Q9RUK0	1075			16639000			
tr|Q9RUL9	tr|Q9RUL9	1087	81152000		52137000	49621000		
tr|Q9RVV5	tr|Q9RVV5	1308	19181000	25388000	19025000	36346000		39329000
tr|Q9RS04	tr|Q9RS04	621	9496400		13780000			
tr|Q9RWK	tr|Q9RWK	1429	40009000	35785000	54155000	18830000	18768000	50990000
tr|Q9RVQ	tr|Q9RVQ	1271						6579900
tr|Q9RVQ	tr|Q9RVQ	1271			45142000			
tr|Q9RSD4	tr|Q9RSD4	696	45322000	31710000	85811000	17150000	43931000	50793000
tr|Q9RSP9	tr|Q9RSP9	746	1.35E+08	1.92E+08	1.54E+08		54210000	
tr|Q9RXM	tr|Q9RXM	1615	2.03E+08	1.78E+08	1.6E+08	2.6E+08	2.58E+08	2.22E+08
tr|Q9RS10	tr|Q9RS10	626	1019900					
tr|Q9RZD1	tr|Q9RZD1	1907					90933000	79940000
tr|Q9RZ90	tr|Q9RZ90	1881						

sp|Q9RW6	sp|Q9RW6	311	17897000	53848000	61681000	1.45E+08	1.42E+08	1.03E+08
sp|Q9RW6	sp|Q9RW6	311	37862000		48023000			38128000
sp|Q9RSL4	sp|Q9RSL4	136	23875000	20304000				
tr|Q9RRK2	tr|Q9RRK2	539	9328700		13733000		17361000	22700000
tr|Q9RY20	tr|Q9RY20	1681	87861000	84478000	1.17E+08	64218000	95313000	86209000
tr|Q9RYD7	tr|Q9RYD7	1734	38326000		31839000			
sp|Q9RT6	sp|Q9RT6	170					15633000	
tr|Q9RXI9	tr|Q9RXI9	1606				4007900	4424700	3694400
tr|Q9RX83	tr|Q9RX83	1548	13705000	9964200	10001000		8417800	
sp|Q9RZE	sp|Q9RZE	455	40167000	36282000	36706000	61558000	48152000	47253000
tr|Q9RZF3	tr|Q9RZF3	1921			4028100	25501000	19383000	16833000
tr|Q9RW6	tr|Q9RW6	1361						
sp|Q9RVB	sp|Q9RVB	275	1.71E+08	1.45E+08	1.45E+08	2.16E+08	1.76E+08	2.08E+08
tr|Q9RSC6	tr|Q9RSC6	689	20078000	13782000	16882000	9043800	8601000	10642000
sp|Q9RUR	sp|Q9RUR	249	36930000	1.2E+08	51192000	1.06E+08	1.79E+08	93501000
sp|Q9RUR	sp|Q9RUR	249	3.41E+08	3.49E+08	3.11E+08	67639000	1.4E+08	1.53E+08
tr|Q9RXL5	tr|Q9RXL5	1611	67465000	88623000	69586000	51254000	48462000	82976000
tr|Q9RRV3	tr|Q9RRV3	590						
tr|Q9RYR6	tr|Q9RYR6	1798	73436000		58155000	54211000	24729000	
sp|Q9RZU	sp|Q9RZU	459	3186200	3666700	5787800	2686700	3800600	4663000
tr|Q9RZ12	tr|Q9RZ12	1849						
tr|Q9RZ12	tr|Q9RZ12	1849			37001000			
sp|Q9RR7	sp|Q9RR7	68	33758000		42382000	46961000	39808000	54864000
tr|Q9RSB4	tr|Q9RSB4	683				72944000		
tr|Q9RRH	tr|Q9RRH	524		43028000	67616000	31537000	32870000	40522000
sp|Q9RTF5	sp|Q9RTF5	182	1.5E+08	1.14E+08	1.03E+08			
tr|Q9RT06	tr|Q9RT06	795	82135000	76859000	50938000	67849000	83074000	56550000
sp|Q9RU8	sp|Q9RU8	217	10054000	27231000				
tr|Q9RU57	tr|Q9RU57	1000	4.2E+08	3.05E+08	2.8E+08	1.38E+08	1.45E+08	1.39E+08
tr|Q9RUA	tr|Q9RUA	1022	63114000	63581000	51459000			
tr|Q9RV86	tr|Q9RV86	1193						
sp|Q9RW0	sp|Q9RW0	342	1.12E+09	1.01E+09	8.67E+08	4.4E+08	4.75E+08	5.79E+08
sp|Q9RS2	sp|Q9RS2	108		41086000	45532000	84114000	89243000	
tr|Q9RVQ	tr|Q9RVQ	1277	18863000			30778000	12696000	15660000
tr|Q9RTC9	tr|Q9RTC9	855	46865000	47335000	29908000	1.49E+08	1.22E+08	1.34E+08
tr|Q9RTC9	tr|Q9RTC9	855	18056000	27321000	57758000	28874000	31679000	32725000
tr|Q9RVJ4	tr|Q9RVJ4	1241	7812100		8566200			
tr|Q9RXD5	tr|Q9RXD5	1573	3.34E+08	3.39E+08	3E+08	2.32E+08	1.7E+08	2.06E+08
tr|Q9RYQ4	tr|Q9RYQ4	1792					4399600	
tr|Q9RYQ4	tr|Q9RYQ4	1792			22141000			
tr|Q9RRH	tr|Q9RRH	527	1.45E+08	1.33E+08	1.09E+08	1.3E+08	1.11E+08	1.17E+08
sp|Q4657	sp|Q4657	61	14009000	27536000	14802000	23597000	21257000	
tr|Q9RSY8	tr|Q9RSY8	786	36257000		16244000			
tr|Q9RYP5	tr|Q9RYP5	1784						
tr|Q9RUD	tr|Q9RUD	1043	1.03E+09	8.89E+08	1.5E+09	5.93E+08	6.45E+08	4.17E+08
tr|Q9RZL7	tr|Q9RZL7	1945	29962000		24836000			
tr|Q9RWD	tr|Q9RWD	1395	45886000	55316000	69340000			
tr|Q9RRV3	tr|Q9RRV3	590	1.93E+08	1.16E+08	1.09E+08	1.84E+08	1.28E+08	1.45E+08
sp|Q9RYR	sp|Q9RYR	441	39394000	29272000	31798000	43572000	38478000	29916000
tr|Q9RS98	tr|Q9RS98	672	58449000	50812000	53134000	1.06E+08	1.03E+08	1.36E+08
sp|Q9RW	sp|Q9RW	349	66888000	53504000	53263000	1.56E+08	1.58E+08	1.6E+08

tr|Q9RZM	tr|Q9RZM	1947						
tr|Q9RX49	tr|Q9RX49	1527						
tr|Q9RTQ0	tr|Q9RTQ0	912	97574000	1.28E+08	2.03E+08	1.01E+08	79693000	1.1E+08
tr|Q9RTG9	tr|Q9RTG9	874	45288000	46493000	37484000			
tr|Q9RYN4	tr|Q9RYN4	1778	20837000		20010000	19277000	22266000	20517000
tr|Q9RUY8	tr|Q9RUY8	1143	1E+08	98124000	90166000	2.37E+08	2.29E+08	1.51E+08
tr|Q9RUY8	tr|Q9RUY8	1143	69846000	1.05E+08	1.15E+08	17425000	31868000	12762000
sp|Q9RSZ	sp|Q9RSZ	159	5403900	2111200	1820000		2480200	2478900
sp|Q9RSZ	sp|Q9RSZ	159	17065000		29364000			
tr|Q9RV45	tr|Q9RV45	1172	32259000	10893000	11141000	27296000	34953000	41101000
tr|Q9RXV9	tr|Q9RXV9	1654	5.92E+08	4.33E+08	3.48E+08	1.64E+08	1.56E+08	
sp|Q9RWH	sp|Q9RWH	330	10085000	3541300	4323300	1977700	2804400	2481000
tr|Q9RX65	tr|Q9RX65	1538	3577000	5204300	8920300	4229500	19756000	18598000
tr|Q9RT05	tr|Q9RT05	794				18642000		
tr|Q9RSG4	tr|Q9RSG4	713		10024000	44681000			
tr|Q9RX33	tr|Q9RX33	1519		7871500	11745000		15244000	
tr|Q9RVQ	tr|Q9RVQ	1276	4.7E+08	3.41E+08	4.08E+08	1.36E+08	1.79E+08	79016000
tr|Q9RZ07	tr|Q9RZ07	1845						
tr|Q9RV56	tr|Q9RV56	1180	14460000	14495000		17461000	16989000	36316000
tr|Q9RV59	tr|Q9RV59	1182	1.36E+08	1.28E+08	1.01E+08	2.74E+08	2.02E+08	2.63E+08
tr|Q9RV59	tr|Q9RV59	1182			1.07E+08			
sp|Q9RSK	sp|Q9RSK	131	6.61E+08	7.41E+08	9.63E+08	7.51E+08	7.58E+08	1.17E+09
sp|Q9RSK	sp|Q9RSK	131					14031000	
tr|Q9RS18	tr|Q9RS18	631	1.69E+08	1.53E+08	1.44E+08		1.51E+08	
tr|Q9RS05	tr|Q9RS05	622						
sp|P52027	sp|P52027	51	26899000					
sp|P52027	sp|P52027	51			38824000			
tr|Q9RS04	tr|Q9RS04	621						
tr|Q9RVQ	tr|Q9RVQ	1273	471710		221220		263430	296280
sp|Q9RYL	sp|Q9RYL	436				16629000	14580000	14051000
tr|Q9RZB4	tr|Q9RZB4	1897						
tr|Q9RV36	tr|Q9RV36	1169	5.97E+08	6.2E+08	5.92E+08	6.83E+08	4.44E+08	4.81E+08
sp|Q9RW5	sp|Q9RW5	310	56865000	59787000	77095000	47915000	71302000	63093000
sp|Q9RW5	sp|Q9RW5	310				42564000	43652000	29811000
tr|Q9RYV0	tr|Q9RYV0	1820				4315800	8475400	32998000
tr|Q9RRY5	tr|Q9RRY5	608	69413000	1.11E+08	1.48E+08	1.79E+08	1.71E+08	1.76E+08
tr|Q9RT05	tr|Q9RT05	794	56600000	56175000	57464000		25445000	
tr|Q9RW8	tr|Q9RW8	1368						
sp|Q9RW0	sp|Q9RW0	326	3.66E+09	5.41E+09	4.25E+09	3.36E+09	4.34E+09	3.62E+09
tr|Q9RWU	tr|Q9RWU	1481		52055000	28820000			
tr|Q9RVV4	tr|Q9RVV4	1307						
tr|Q9RXD5	tr|Q9RXD5	1573	6302700	2332100	1656100	1898700	3378500	2908900
tr|Q9RS17	tr|Q9RS17	630	7.5E+09	4.59E+09	4.71E+09	4.45E+09	4.02E+09	3.9E+09
tr|Q9RVU2	tr|Q9RVU2	1300			27597000			
tr|Q9RS71	tr|Q9RS71	655	38538000		12311000			
tr|Q9RU62	tr|Q9RU62	1004	2.44E+08	1.67E+08	1.42E+08	1.41E+08	1.04E+08	1.39E+08
tr|Q9RT70	tr|Q9RT70	827				14507000	10885000	13916000
tr|Q9RR95	tr|Q9RR95	479		17516000				
tr|Q9RTQ3	tr|Q9RTQ3	914	9911500	5215600	997800	13281000	12568000	12168000
tr|Q9RWD	tr|Q9RWD	1395		49912000	11529000	12623000		
tr|Q9RUJ3	tr|Q9RUJ3	1069	6116000		4422000			

tr|Q9RRZ7	tr|Q9RRZ7	616						
tr|Q9RRI4	tr|Q9RRI4	532	51713000		41689000			
tr|Q9RWP	tr|Q9RWP	1445	41562000	97045000	82841000	34789000	43424000	34705000
tr|Q9RYB8	tr|Q9RYB8	1722	1.39E+08	1.01E+08	98045000	1E+08	1.01E+08	66215000
tr|Q9RUE0	tr|Q9RUE0	1044	1.63E+08	1.27E+08	1.26E+08	3.28E+08	2.99E+08	2.67E+08
tr|Q9RUE0	tr|Q9RUE0	1044	5.01E+08	7.2E+08	8.9E+08	1.59E+09	1.66E+09	1.27E+09
tr|Q9RY56	tr|Q9RY56	1698						
tr|Q9RWD	tr|Q9RWD	1395	38757000	28977000	27577000	32889000	45221000	92977000
tr|Q9RTY1	tr|Q9RTY1	956	35910000	11311000		1.02E+08	84332000	90610000
tr|Q9RS41	tr|Q9RS41	641	18397000	13377000	15205000			
tr|Q9RUH	tr|Q9RUH	1060	15526000	41662000	5450700			
tr|Q9RTX6	tr|Q9RTX6	951		66119000		38505000	56944000	
tr|Q9RXN1	tr|Q9RXN1	1621		2.5E+08	2.27E+08		49694000	
tr|Q9RRI7	tr|Q9RRI7	534						
tr|Q9RRI7	tr|Q9RRI7	534		28526000	47480000			19167000
tr|Q9RVF4	tr|Q9RVF4	1223	7991400	7273800	6115100	24265000	14944000	17502000
tr|Q9RR95	tr|Q9RR95	479	18542000		13753000			
tr|Q9RYV4	tr|Q9RYV4	1822				16063000		
tr|Q9RYU9	tr|Q9RYU9	1819	2.61E+08	2.18E+08	2E+08	1.61E+08		1.62E+08
sp|Q9RVG	sp|Q9RVG	287						
sp|O3250	sp|O3250	44	27099000	27323000	57020000	18419000	41687000	46260000
tr|Q9RYL2	tr|Q9RYL2	1769	2.11E+08	1.75E+08	1.52E+08	2.02E+08	2.01E+08	90615000
tr|Q9RYX2	tr|Q9RYX2	1833	59627000	49371000	36402000	68036000	72183000	58985000
tr|Q9RX29	tr|Q9RX29	1517	2.93E+08	2.69E+08	2.42E+08	3.85E+08	4.17E+08	2.68E+08
tr|Q9RRV1	tr|Q9RRV1	588				30871000	58375000	52119000
tr|Q9RXI3	tr|Q9RXI3	1602			35130000			
tr|Q9RZN3	tr|Q9RZN3	1950	31514000	32167000	51654000		33754000	67325000
tr|Q9RRA6	tr|Q9RRA6	489	1.56E+09	1.74E+09	2.08E+09	3.89E+09	5.38E+09	2.15E+09
tr|Q9RRA6	tr|Q9RRA6	489			9224600			
tr|Q9RTW	tr|Q9RTW	945	22158000	12740000	17537000	22962000	21003000	35380000
tr|Q9RZE4	tr|Q9RZE4	1914		9079800	11154000			5052600
sp|Q9RTB	sp|Q9RTB	177	15843000		22956000	35710000	31459000	30141000
tr|Q9RWC	tr|Q9RWC	1389						
sp|P50933	sp|P50933	50			14706000		8581500	
tr|Q9RTH4	tr|Q9RTH4	877			73448000		14404000	
tr|Q9RZT0	tr|Q9RZT0	1968	39444000	34040000	20942000	1.05E+08	99377000	1.15E+08
tr|Q9RUF8	tr|Q9RUF8	1049			5870500	30025000	38169000	41719000
tr|Q9RT92	tr|Q9RT92	838	10928000		14993000			
tr|Q9RUJ3	tr|Q9RUJ3	1069		14024000	15887000			
tr|Q9RZQ	tr|Q9RZQ	1959				4.03E+08	5.12E+08	2.88E+08
tr|Q9RYN6	tr|Q9RYN6	1780		22889000	42750000	82845000	1.07E+08	70189000
tr|Q9RU34	tr|Q9RU34	983	2.07E+08	2.02E+08	2.59E+08	3.56E+08	3.09E+08	3.7E+08
tr|Q9RUZ5	tr|Q9RUZ5	1146	14841000	27988000		30596000	36176000	27121000
sp|Q9RSR	sp|Q9RSR	144				31303000	32006000	24864000
tr|Q9RYD2	tr|Q9RYD2	1732	7846500	6045400	8118400		6909900	7547400
tr|Q9RVN	tr|Q9RVN	1263				58822000	74005000	41651000
tr|Q9RS56	tr|Q9RS56	648	83120000	1.24E+08	1.34E+08	1.39E+08	1.53E+08	1.2E+08
tr|Q9RTX7	tr|Q9RTX7	952	8877900	4242700	3830200	2958400	3358400	3259200
tr|Q9RZM	tr|Q9RZM	1949	95411000	1.1E+08	1.06E+08	1.3E+08	1.16E+08	1.23E+08
tr|Q9RRH	tr|Q9RRH	525	28696000	24713000		51473000	36354000	38794000
tr|Q9RY76	tr|Q9RY76	1706						

tr|Q9RVU5	tr|Q9RVU5	1302	25893000	14079000			15240000	
tr|Q9RWX	tr|Q9RWX	1498		8463200	5695500	9091600	9643400	
sp|Q9RUN	sp|Q9RUN	240						
tr|Q9RY02	tr|Q9RY02	1675	5552200		10986000		8046600	3051100
tr|Q9RY02	tr|Q9RY02	1675	87882000		1.68E+08			27555000
tr|Q9RWF	tr|Q9RWF	1405		22743000	20582000	18335000	27063000	29547000
tr|Q9RS97	tr|Q9RS97	671	1.71E+09	1.44E+09	1.29E+09	1.21E+09	1.21E+09	1.32E+09
tr|Q9RU39	tr|Q9RU39	986	3299800				2153900	
tr|Q9RS66	tr|Q9RS66	653	14067000	7534200	8634500	7464400	13283000	8436800
tr|Q9RXM	tr|Q9RXM	1614				20520000	26049000	
tr|Q9RW3	tr|Q9RW3	1349	79370000	1.07E+08	1E+08	2.14E+08	2.06E+08	2.75E+08
tr|Q9RUA	tr|Q9RUA	1020	14270000		12135000	89445000	84224000	85679000
tr|Q9RXH7	tr|Q9RXH7	1598	1.43E+08	1.5E+08		1.07E+08	91642000	1.03E+08
sp|Q9RXC	sp|Q9RXC	366				1.36E+09	1.33E+09	1.05E+09
sp|Q9RVP	sp|Q9RVP	294		10852000	18953000	5145500	14502000	4734200
sp|Q9RSI4	sp|Q9RSI4	121	1.26E+08		54352000	2.02E+08	1.81E+08	1.54E+08
tr|Q9RU62	tr|Q9RU62	1004	2.76E+08	3.09E+08	2.83E+08	2.9E+08	2.43E+08	2.42E+08
tr|Q9RTM	tr|Q9RTM	899				96239000		84060000
sp|Q9RUK	sp|Q9RUK	238	7E+08	5.83E+08	8.8E+08	2.82E+08	3.79E+08	4.56E+08
sp|Q9RYD	sp|Q9RYD	430	1.51E+08	1.32E+08	1.21E+08	92442000	1.05E+08	1.03E+08
sp|Q9RVC	sp|Q9RVC	278						
sp|Q9RTJ3	sp|Q9RTJ3	189						
tr|Q9RR95	tr|Q9RR95	479	67395000	49221000	74370000			
tr|Q9RZI5	tr|Q9RZI5	1936			27140000	20613000		
tr|Q9RTL6	tr|Q9RTL6	897	44326000	26432000	35861000			
tr|Q9RTG6	tr|Q9RTG6	872						
tr|Q9RY37	tr|Q9RY37	1690		24704000	41415000	30721000		
tr|Q9RXM	tr|Q9RXM	1615	3.6E+08	3.22E+08	2.61E+08			
tr|Q9RXM	tr|Q9RXM	1615					6554000	7924200
tr|Q9RX10	tr|Q9RX10	1507	1.06E+08	1.77E+08	1.12E+08			88693000
tr|Q9RT70	tr|Q9RT70	827			36714000			
tr|Q9RRK9	tr|Q9RRK9	543	1.07E+09	9.94E+08	50842000	4.51E+08	4.12E+08	5.54E+08
tr|Q9RRK9	tr|Q9RRK9	543						
tr|Q9RWV	tr|Q9RWV	1485	81397000	80576000	84938000	74983000	88634000	57304000
tr|Q9RYA6	tr|Q9RYA6	1720	4.77E+08	5.4E+08	4.15E+08	3.35E+08	3.67E+08	2.93E+08
tr|Q9RUC	tr|Q9RUC	1036						
tr|Q9RUJ6	tr|Q9RUJ6	1072	1.75E+08	2.76E+08	1.8E+08	3.04E+08	2.73E+08	
tr|Q9RWQ	tr|Q9RWQ	1450	25963000		19167000	26973000		36930000
tr|Q9RZM	tr|Q9RZM	1948						
sp|Q9RX9	sp|Q9RX9	363						
sp|Q9RX9	sp|Q9RX9	363						
sp|Q9RTK	sp|Q9RTK	193		25909000	28752000			
tr|Q9RSV8	tr|Q9RSV8	773	12161000	26356000	10116000	39941000	25978000	27021000
tr|Q9RSV8	tr|Q9RSV8	773	63763000	2.04E+08	1.11E+08	40899000	30784000	61717000
tr|Q9RU54	tr|Q9RU54	997	1E+09	1.04E+09	1.79E+09	7.69E+08	5.03E+08	1.01E+09
tr|Q9RZN3	tr|Q9RZN3	1950						
sp|Q9RYM	sp|Q9RYM	438	1.05E+09	1.03E+09	8.95E+08	2.85E+09	2.75E+09	2.5E+09
sp|Q9RYM	sp|Q9RYM	438			80091000	1.39E+08	1.09E+08	1.23E+08
tr|Q9RUE0	tr|Q9RUE0	1044						
sp|Q9RUV	sp|Q9RUV	252	13962000					
tr|Q9RYF0	tr|Q9RYF0	1742					34162000	

tr|Q9RZK2	tr|Q9RZK2	1940						
tr|Q9RZM	tr|Q9RZM	1948						
sp|Q9RTJ3	sp|Q9RTJ3	189	39728000		52665000			
tr|Q9RXG0	tr|Q9RXG0	1587						
tr|Q9RZJ2	tr|Q9RZJ2	1939						
tr|Q9RT70	tr|Q9RT70	827	1.11E+08	1.18E+08	83528000	48964000		
tr|Q9RZI1	tr|Q9RZI1	1935			10331000		26424000	27035000
tr|Q9RXF2	tr|Q9RXF2	1582						
sp|Q9RV9	sp|Q9RV9	273	23046000	51658000	96352000	1.29E+08	86710000	1.27E+08
tr|Q9RVX7	tr|Q9RVX7	1318	35389000	40614000	36376000	74592000	77465000	76895000
tr|Q9RVX7	tr|Q9RVX7	1318	3.15E+08	3.11E+08	2.78E+08	1.49E+08	27734000	2.14E+08
sp|Q9RV6	sp|Q9RV6	266						
tr|Q9RUW	tr|Q9RUW	1126	2074400					
sp|Q9RW0	sp|Q9RW0	326	30541000	17538000				
sp|Q9RS64	sp|Q9RS64	111	1.11E+10	2.34E+10	9.68E+09	9.41E+09	9.52E+09	1.02E+10
sp|Q9RS8	sp|Q9RS8	113	21673000					
tr|Q9RS88	tr|Q9RS88	664				9.34E+08		2322400
tr|Q9RUY8	tr|Q9RUY8	1143	7.56E+08	8.84E+08	1.04E+09	3.31E+08	8.73E+08	1.16E+08
tr|Q9RYW	tr|Q9RYW	1830	24922000	14674000		24953000	16955000	
tr|Q9RYV8	tr|Q9RYV8	1825	9931600		20139000			
sp|Q9RS0	sp|Q9RS0	105	20917000	29742000	48606000	7738200	14577000	14868000
sp|Q9RXK	sp|Q9RXK	386	5.99E+08	81055000	4.76E+08	1.34E+09	1.23E+09	96227000
sp|Q9RXK	sp|Q9RXK	386						33918000
tr|Q9RZV4	tr|Q9RZV4	1984	1.97E+09	2.12E+09	2.29E+09	1.9E+08	8.95E+08	1.02E+08
tr|Q9RSB3	tr|Q9RSB3	682						
tr|Q9RTP9	tr|Q9RTP9	911	61009000	63350000	1.08E+08			
tr|Q9RW4	tr|Q9RW4	1354	2.58E+08	2.89E+08	3.01E+08		2.79E+08	1.74E+08
tr|Q9RW4	tr|Q9RW4	1354						
tr|Q9RXP7	tr|Q9RXP7	1626	48300000				1.05E+08	95492000
tr|Q9RX44	tr|Q9RX44	1524		4.32E+08	1.47E+08	2.77E+08	2.5E+08	2.32E+08
sp|Q9RXK	sp|Q9RXK	388		72865000		96140000	1.32E+08	84788000
sp|Q9RUE	sp|Q9RUE	225		8843300	19101000			
tr|Q9RSR2	tr|Q9RSR2	750	11502000	11193000	6596100			
tr|Q9RWD	tr|Q9RWD	1394						
tr|Q9RZA6	tr|Q9RZA6	1891			18773000	66889000	72274000	
tr|Q9RVL5	tr|Q9RVL5	1252						
tr|Q9RVS1	tr|Q9RVS1	1287	33026000	29694000	30683000	64136000	48230000	48395000
tr|Q9RUH	tr|Q9RUH	1058	1.03E+08	97932000	90770000	56853000	57944000	68977000
tr|Q9RXK9	tr|Q9RXK9	1608			3709800	4266400	6137300	5197500
sp|Q9RXE	sp|Q9RXE	368				58447000	38879000	
tr|Q9RZD7	tr|Q9RZD7	1911	36599000	75216000	47635000	90884000	93621000	1.07E+08
sp|Q9RXF	sp|Q9RXF	369	98818000	1.18E+08	72594000	59715000	63090000	65162000
tr|Q9RZ87	tr|Q9RZ87	1879	6.17E+08	7.29E+08	5.19E+08	2.18E+09	1.93E+09	1.32E+09
tr|Q9RZ87	tr|Q9RZ87	1879			17860000			
sp|O83020	sp|O83020	47	2.85E+08	3.25E+08	2.3E+08	1.74E+08	1.44E+08	1.54E+08
tr|Q9RRU1	tr|Q9RRU1	584	50535000	36256000	46320000	1.08E+08	90841000	50848000
tr|Q9RWR	tr|Q9RWR	1460	1.48E+08	1.16E+08	1.17E+08	99337000	69219000	88296000
tr|Q9RSR8	tr|Q9RSR8	753				52763000	65913000	50130000
tr|Q9RUG	tr|Q9RUG	1054			44212000	9563500	14092000	16546000
sp|Q9RW0	sp|Q9RW0	327	1.15E+08	89228000	77624000	1.17E+08		
tr|Q9RTN3	tr|Q9RTN3	904	1.08E+08	77229000	97992000	50518000	45244000	55836000

tr|Q9RSC7	tr|Q9RSC7	690	12889000					
tr|Q9RZS3	tr|Q9RZS3	1964	17200000	15424000	21416000	27192000	42871000	31434000
tr|Q9RUG	tr|Q9RUG	1055	1.75E+08	2.53E+08	2.04E+08	4.74E+08	3.91E+08	4.41E+08
tr|Q9RRA6	tr|Q9RRA6	489	7.93E+08			2.43E+09	2.18E+09	2.1E+09
tr|Q9RRA6	tr|Q9RRA6	489	3.43E+08	2.61E+08	5.16E+08	1.52E+08	2.74E+08	3.39E+08
tr|Q9RXG3	tr|Q9RXG3	1589		8548300				
sp|Q9RXK	sp|Q9RXK	386	2.03E+08	1.5E+08	3.42E+08	1.47E+09	2.4E+09	1.3E+09
tr|Q9RV41	tr|Q9RV41	1171	44320000	68662000	43682000	1.18E+08	1.05E+08	1.39E+08
tr|Q9RRK0	tr|Q9RRK0	538	28895000	21752000	29506000	98033000	1.18E+08	58489000
tr|Q9RRK0	tr|Q9RRK0	538	29085000	66791000	64107000		31813000	
tr|Q9RUG	tr|Q9RUG	1052						
tr|Q9RWS	tr|Q9RWS	1461						
tr|Q9RWS	tr|Q9RWS	1461	3.94E+08	3.1E+08	3.6E+08	2.05E+08	1.82E+08	2.5E+08
tr|Q9RWS	tr|Q9RWS	1461				8964600	11548000	11061000
tr|Q9RRU1	tr|Q9RRU1	584	6738700	18092000	28495000		6046300	
tr|Q9RY56	tr|Q9RY56	1698	2.93E+08	87863000	1.34E+08	8.6E+08	6.71E+08	1.08E+09
tr|Q9RUS4	tr|Q9RUS4	1112			12525000	63762000	83687000	40652000
tr|Q9RSI2	tr|Q9RSI2	722						
tr|Q9RWL	tr|Q9RWL	1432	89312000	65292000	61493000			
tr|Q9RU43	tr|Q9RU43	990	57426000	50338000	46480000	29444000	31103000	18450000
tr|Q9RT30	tr|Q9RT30	807	1.75E+08	2.1E+08	1.77E+08	28287000	1.82E+08	33735000
tr|Q9RZT4	tr|Q9RZT4	1971	28158000	37686000	26613000	66985000	34955000	24696000
tr|Q9RUA	tr|Q9RUA	1025	1.04E+08	65722000	66444000	47750000	44092000	50759000
tr|Q9RZ04	tr|Q9RZ04	1844	5362900		5489000			
tr|Q9RU52	tr|Q9RU52	995	84315000	43625000	46893000			
tr|Q9RV55	tr|Q9RV55	1179			16475000	66342000	51578000	47442000
tr|Q9RSA3	tr|Q9RSA3	674	11756000	6692000	4410600	5659800	7395000	6901600
tr|Q9RWB	tr|Q9RWB	1385	76687000	59228000	66361000	1.42E+08	1.12E+08	1.19E+08
tr|Q9RYQ8	tr|Q9RYQ8	1794	40506000			49423000		47624000
tr|Q9RUN	tr|Q9RUN	1093	31913000	40329000		27551000	21443000	
tr|Q9RUR2	tr|Q9RUR2	1105	1.75E+08	1.22E+08	1.07E+08	3.75E+08	3.24E+08	2.79E+08
tr|Q9RX64	tr|Q9RX64	1537		31158000	28537000			
tr|Q9RVG7	tr|Q9RVG7	1229	91389000		65918000			
sp|Q9RY24	sp|Q9RY24	412	3.82E+08	5.37E+08	3.93E+08	6.02E+08	7.98E+08	6.54E+08
tr|Q9RSU7	tr|Q9RSU7	767	24990000	15871000	43757000		3306700	5503600
tr|Q9RWD	tr|Q9RWD	1394	41938000	39671000	23196000	64668000	27684000	25255000
tr|Q9RUM	tr|Q9RUM	1089	69794000	49984000	95615000	32527000		
tr|Q9RWV	tr|Q9RWV	1484		40222000			14517000	
tr|Q9RV56	tr|Q9RV56	1180			18212000		44002000	
tr|Q9RZA0	tr|Q9RZA0	1887	1.47E+08	1.54E+08	1.22E+08	3.01E+08	2.96E+08	2.63E+08
tr|Q9RS08	tr|Q9RS08	624						25892000
tr|Q9RSN0	tr|Q9RSN0	739	3.58E+08	3.41E+08	2.37E+08	2.24E+08	2.56E+08	3.03E+08
tr|Q9RSZ0	tr|Q9RSZ0	788						
tr|Q9RWC	tr|Q9RWC	1393				27718000	32202000	
sp|Q9RUE	sp|Q9RUE	229	2682400	1783200	3076200		749390	
sp|Q9RXJ6	sp|Q9RXJ6	381	53105000		3.79E+08	91116000	81801000	70341000
tr|Q9RWQ	tr|Q9RWQ	1453	89441000	1.01E+08	84198000	12876000	10298000	10764000
tr|Q9RZL7	tr|Q9RZL7	1945	2.5E+08	2.03E+08	2.51E+08	3.03E+08	2.31E+08	2.89E+08
sp|Q9RY2	sp|Q9RY2	411	889340	3904900	3500000		1976700	
tr|Q9RXX2	tr|Q9RXX2	1660	1.31E+08	1.03E+08	1.32E+08	28984000	28733000	51156000
sp|Q9RUY	sp|Q9RUY	259	5209500	1779200	1346600	4518600	4498800	4128200

tr|Q9RXX9	tr|Q9RXX9	1663		24630000		14676000	22119000	25056000
tr|Q9RSP9	tr|Q9RSP9	746		21765000		15090000	21922000	19528000
sp|Q9RU8	sp|Q9RU8	222						
tr|Q9RYI0	tr|Q9RYI0	1759	78219000					
tr|Q9RU42	tr|Q9RU42	989						15115000
sp|Q9RVM	sp|Q9RVM	291	23459000	9194700			20981000	
tr|Q9RZA1	tr|Q9RZA1	1888			8754100			
sp|Q9RXJ7	sp|Q9RXJ7	382	3.52E+09	3.37E+09	2.96E+09	2.93E+09	2.27E+09	2.97E+09
tr|Q9RVP5	tr|Q9RVP5	1269	6.8E+08	6.69E+08	6.04E+08	2.03E+09	2.07E+09	1.46E+09
sp|Q9RR9	sp|Q9RR9	72	5410800		5319000	21988000		
sp|Q9RR6	sp|Q9RR6	66	1.38E+08	1.2E+08	1.34E+08	86091000	78167000	68177000
sp|Q9RT6	sp|Q9RT6	170	3.38E+08	2.54E+08	2.62E+08	99192000	97823000	1.54E+08
tr|Q9RUC	tr|Q9RUC	1036						
sp|Q9RVC	sp|Q9RVC	278	55148000	38842000	42924000		33328000	
tr|Q9RSE3	tr|Q9RSE3	702	28263000	28344000	24867000	71964000	69130000	68174000
tr|Q9RR83	tr|Q9RR83	475	3.96E+08	3.55E+08	3.32E+08		13457000	
tr|Q9RSD8	tr|Q9RSD8	698						6504900
tr|Q9RVV1	tr|Q9RVV1	1305	3.71E+08	2.87E+08	2.64E+08	2.33E+08	2.61E+08	2.5E+08
tr|Q9RWV	tr|Q9RWV	1487	79012000	91616000	82577000		1.33E+08	
sp|Q9RUV	sp|Q9RUV	258			956980			
sp|Q9RZL	sp|Q9RZL	456						
sp|Q9RSI4	sp|Q9RSI4	121		70683000	70923000	56819000	59654000	46333000
tr|Q9RUL2	tr|Q9RUL2	1084	1.19E+08		72816000	48137000	42630000	59201000
tr|Q9RSK2	tr|Q9RSK2	730		15966000		76530000	64519000	60310000
tr|Q9RVJ8	tr|Q9RVJ8	1243		18412000	30421000			
sp|Q9RYD	sp|Q9RYD	431						
tr|Q9RX12	tr|Q9RX12	1509		66722000	52573000	85574000	55883000	57419000
tr|Q9RXB0	tr|Q9RXB0	1561	1.25E+08	1.23E+08	1.22E+08	1.35E+08	1.94E+08	
tr|Q9RW7	tr|Q9RW7	1363			19731000			
tr|Q9RXY9	tr|Q9RXY9	1667				40996000	28814000	
tr|Q9RUI9	tr|Q9RUI9	1066						
tr|Q9RRR3	tr|Q9RRR3	568	3.36E+08	2.85E+08	2.43E+08	2.4E+08	2.09E+08	2.41E+08
tr|Q9RSN4	tr|Q9RSN4	741						68021000
tr|Q9RSU7	tr|Q9RSU7	767		22290000	25579000	18967000		
tr|Q9RXI0	tr|Q9RXI0	1600			6787900			
tr|Q9RYN3	tr|Q9RYN3	1777	86348000	1.26E+08	1.37E+08	1.13E+08	67752000	64188000
tr|Q9RYI7	tr|Q9RYI7	1763	1.54E+08	1.67E+08	2.41E+08	18578000	45471000	30364000
tr|Q9RX85	tr|Q9RX85	1550						
tr|Q9RVK1	tr|Q9RVK1	1244	61221000	68178000	50231000	81013000	59338000	89823000
tr|Q9RRI9	tr|Q9RRI9	535						
tr|Q9RYN6	tr|Q9RYN6	1780	44134000	1.41E+08	1.56E+08	46719000	48123000	39877000
tr|Q9RWU	tr|Q9RWU	1474	1.73E+08	2.13E+08	2.1E+08	2.77E+08	2.22E+08	2.61E+08
tr|Q9RRD5	tr|Q9RRD5	505	20317000		31653000		8583100	
tr|Q9RXQ	tr|Q9RXQ	1627	2.97E+08	2.18E+08	2.27E+08	4.48E+08	4.18E+08	4.69E+08
tr|Q9RU52	tr|Q9RU52	995	3.21E+08	3.52E+08	2.94E+08	5.1E+08	5.04E+08	5.17E+08
tr|Q9RU52	tr|Q9RU52	995		22345000	35568000		17617000	21843000
tr|Q9RTH4	tr|Q9RTH4	877	8.13E+08	8.11E+08	7.71E+08	4.92E+08	3.18E+08	4.25E+08
tr|Q9RYL0	tr|Q9RYL0	1767	2.01E+09	1.95E+09	1.87E+09	2.05E+08	2.68E+08	2.47E+08
tr|Q9RRG	tr|Q9RRG	518	2.35E+08	3.04E+08	2.51E+08	2.19E+08	2.36E+08	2.15E+08
tr|Q9RT74	tr|Q9RT74	830	1.83E+08	2.67E+08	3.06E+08	1.51E+08	4.14E+08	78785000
tr|Q9RS07	tr|Q9RS07	623				88655000	98872000	92627000

tr|Q9RS07	tr|Q9RS07	623	1.09E+08	83437000	1.47E+08	32867000		
tr|Q9RTH4	tr|Q9RTH4	877	5.09E+08	3.56E+08	3.5E+08	91432000	1.74E+08	1.26E+08
tr|Q9RW2	tr|Q9RW2	1345	48878000	47201000	43586000	58037000	74668000	
tr|Q9RUL0	tr|Q9RUL0	1082	9514800	9743800		29453000	15868000	27112000
tr|Q9RY54	tr|Q9RY54	1696		49533000	48186000	28569000	32470000	46256000
sp|Q9RRJ5	sp|Q9RRJ5	88		32662000	52637000	19329000		
tr|Q9RUL0	tr|Q9RUL0	1082	55501000	57038000	63788000			
tr|Q9RS28	tr|Q9RS28	635	2.45E+08	2.47E+08	1.74E+08			
sp|Q9RRM	sp|Q9RRM	91	63132000	60157000	44924000	90419000	1.03E+08	79622000
sp|Q9RYE	sp|Q9RYE	432	49603000	62133000	64484000	25767000	36063000	37213000
tr|Q9RY34	tr|Q9RY34	1687	1.5E+08	2.26E+08	1.82E+08	3.09E+08	4.15E+08	3.3E+08
sp|Q9RWI	sp|Q9RWI	336	82865000	64303000	65309000		1.26E+08	1.51E+08
sp|Q9RRQ	sp|Q9RRQ	95	3.39E+08	2.63E+08	2.6E+08	2.19E+08	2.2E+08	2.12E+08
tr|Q9RV05	tr|Q9RV05	1153		14614000	11555000	22502000	31162000	25473000
tr|Q9RVT5	tr|Q9RVT5	1296			67405000	29529000	20276000	
tr|Q9RSD0	tr|Q9RSD0	692	2056300	1513500	1575800	2319100	3001800	2405000
tr|Q9RVL9	tr|Q9RVL9	1254				12294000	13938000	
tr|Q9RVL9	tr|Q9RVL9	1254			42830000		11941000	23052000
tr|Q9RRE2	tr|Q9RRE2	510		13909000	15700000			
tr|Q9RR98	tr|Q9RR98	481						
tr|Q9RRL1	tr|Q9RRL1	545		18580000				
tr|Q9RZJ2	tr|Q9RZJ2	1939						
tr|Q9RSW	tr|Q9RSW	775			29791000			
tr|Q9RTP0	tr|Q9RTP0	906	1.68E+08	1.92E+08	2.09E+08	1.74E+08	4.01E+08	3.51E+08
tr|Q9RT43	tr|Q9RT43	815	1.42E+08	1.19E+08	2.23E+08	1.14E+08	1.41E+08	1.47E+08
tr|Q9RUI7	tr|Q9RUI7	1065	5.19E+08	4.67E+08	3.74E+08	5.03E+08	5.11E+08	4.89E+08
tr|Q9RXF0	tr|Q9RXF0	1581	13759000	6557600	13721000	10142000	9218800	7132800
tr|Q9RWI1	tr|Q9RWI1	1414				29584000	28789000	26740000
tr|Q9RRV2	tr|Q9RRV2	589			20232000	22930000	20292000	19382000
sp|Q9RSW	sp|Q9RSW	153	2.33E+08	1.98E+08	1.8E+08	2.02E+08	2.45E+08	1.68E+08
tr|Q9RXU1	tr|Q9RXU1	1644		24451000		17230000		
sp|Q9RTJ5	sp|Q9RTJ5	190		75553000	66106000	37889000	51845000	43311000
tr|Q9RVV0	tr|Q9RVV0	1304	35038000	29446000	24032000			
tr|Q9RZA1	tr|Q9RZA1	1888	1.44E+08	1.54E+08	1.47E+08	4.61E+08	3.17E+08	4.54E+08
tr|Q9RT29	tr|Q9RT29	806	22959000	33886000	40286000	22626000	34964000	28224000
tr|Q9RT29	tr|Q9RT29	806		42164000				1.11E+08
tr|Q9RV75	tr|Q9RV75	1189	17120000	7590200		9135900		
tr|Q9RWN	tr|Q9RWN	1442	66347000	81827000	1.24E+08	46109000	93375000	84327000
sp|Q9RV8	sp|Q9RV8	270	14805000		10625000			
sp|Q9RSR	sp|Q9RSR	143	2.77E+08	2.86E+08	2.71E+08	2.17E+08	2.87E+08	2.53E+08
tr|Q9RXI1	tr|Q9RXI1	1601	28939000			43816000		47167000
tr|Q9RUJ2	tr|Q9RUJ2	1068	49478000	61856000	44854000	75513000	72062000	76808000
sp|Q9RVI3	sp|Q9RVI3	289						
tr|Q9RU62	tr|Q9RU62	1004	1541400	5093500	4532800	15597000	14554000	14661000
tr|Q9RTA8	tr|Q9RTA8	846		38382000	32374000			
tr|Q9RSD8	tr|Q9RSD8	698				88454000	1.14E+08	
sp|Q9RTL	sp|Q9RTL	194	2.4E+08	2.58E+08	2.1E+08	2.21E+08	2.45E+08	2.32E+08
tr|Q9RZI0	tr|Q9RZI0	1934	13814000	9869100	14514000			
sp|Q9RUV	sp|Q9RUV	253	15770000		32483000			
tr|Q9RT09	tr|Q9RT09	798	4.38E+08	8.11E+08	6.29E+08	35859000	26024000	1.23E+08
sp|Q9RXI	sp|Q9RXI	374			15259000	15133000	16358000	14088000

tr|Q9RVZ4	tr|Q9RVZ4	1327	55381000	53684000	56490000	90502000	1.02E+08	82723000
tr|Q9RRX0	tr|Q9RRX0	600	30920000	56112000	28720000	52447000	67717000	53608000
tr|Q9RT07	tr|Q9RT07	796	26182000	28223000	34148000	33996000		
sp|Q9RWJ	sp|Q9RWJ	333						
sp|Q9RYB	sp|Q9RYB	427	72175000	89068000	93197000	51214000	33616000	47301000
tr|Q9RTX9	tr|Q9RTX9	954		21091000	31708000	10022000		14241000
tr|Q9RXA2	tr|Q9RXA2	1556	17569000	29593000		89595000	1E+08	61161000
tr|Q9RRR8	tr|Q9RRR8	570					14451000	
tr|Q9RXT6	tr|Q9RXT6	1641			12603000		13190000	
sp|Q9RSR	sp|Q9RSR	143	42933000	35886000			20812000	26591000
tr|Q9RX55	tr|Q9RX55	1530	28904000	47202000	52229000	16598000	28866000	31863000
tr|Q9RWP	tr|Q9RWP	1449						
sp|Q9RXT	sp|Q9RXT	401						
sp|Q9RW0	sp|Q9RW0	342						
sp|P56864	sp|P56864	55						
tr|Q9RUW	tr|Q9RUW	1126					20739000	
sp|Q9RXL	sp|Q9RXL	392	59511000		51474000			
sp|Q9RY5	sp|Q9RY5	418	8.18E+08	9.14E+08	8.06E+08	4.76E+08	6.22E+08	5.77E+08
tr|Q9RW8	tr|Q9RW8	1371						18973000
tr|Q9RRI1	tr|Q9RRI1	530						
sp|Q9RSS4	sp|Q9RSS4	146	2.01E+08	1.37E+08	2.02E+08			
tr|Q9RRE0	tr|Q9RRE0	508	42471000	50608000		51017000	58008000	59539000
tr|Q9RSZ5	tr|Q9RSZ5	790						
sp|Q9RW7	sp|Q9RW7	314		41792000	40446000			
sp|Q9RY6	sp|Q9RY6	420	2.99E+08	2.87E+08	3.3E+08	38870000	88070000	79986000
tr|Q9RUF9	tr|Q9RUF9	1050	6278700					
tr|Q9RTE2	tr|Q9RTE2	861				14817000	21728000	8818700
tr|Q9RVV7	tr|Q9RVV7	1309	59354000		51998000	18061000	1E+08	15624000
tr|Q9RUT4	tr|Q9RUT4	1118				4981100		
tr|Q9RZE3	tr|Q9RZE3	1913	29055000	36407000		25601000	29004000	24933000
tr|Q9RZE3	tr|Q9RZE3	1913		38296000	40281000			
tr|Q9RTP0	tr|Q9RTP0	906						
tr|Q9RZC5	tr|Q9RZC5	1904	40929000	1.31E+08	1.52E+08	35258000	45099000	40990000
tr|Q9RY00	tr|Q9RY00	1673						
tr|Q9RV99	tr|Q9RV99	1198	7144900	7295700	6316900	15546000	17252000	11719000
tr|Q9RTH4	tr|Q9RTH4	877	31384000	93963000	1.13E+08	40784000	60858000	20200000
tr|Q9RTH4	tr|Q9RTH4	877	17594000	42278000	1.11E+08			
tr|Q9RWU	tr|Q9RWU	1477	32283000	28537000	20664000			
tr|Q9RRV4	tr|Q9RRV4	591	98236000	78868000	78755000	86908000	1.14E+08	1.22E+08
sp|Q9RSX	sp|Q9RSX	155	85412000				16011000	
sp|Q9RTI6	sp|Q9RTI6	188	18058000	23217000	53341000	38937000	31428000	22695000
tr|Q9RRT9	tr|Q9RRT9	582		12364000		7576500	8540300	9962200
tr|Q9RWW	tr|Q9RWW	1493		31810000	18365000	3.15E+08	4.78E+08	3.23E+08
tr|Q9RWT	tr|Q9RWT	1471						
sp|Q9RT2	sp|Q9RT2	164	1.25E+08	1.61E+08	1.37E+08			
tr|Q9RUA	tr|Q9RUA	1023	75869000	91377000	70771000	1.41E+08	2.03E+08	1.34E+08
tr|Q9RV78	tr|Q9RV78	1190	85085000	58692000	1.17E+08	81700000	1.24E+08	52049000
tr|Q9RV78	tr|Q9RV78	1190	11393000		52189000			
tr|Q9RY37	tr|Q9RY37	1690	31753000	85281000	1.11E+08	44319000	51784000	21830000
tr|Q9RW5	tr|Q9RW5	1357	95770000	1.75E+08	1.62E+08	50179000	54003000	81367000
sp|Q9RSF0	sp|Q9RSF0	117						

sp|Q9RUA	sp|Q9RUA	224	2.1E+08	4.19E+08	3.84E+08	1.98E+08	2.03E+08	1.62E+08
tr|Q9RU0	tr|Q9RU07	972		77003000	92940000			
tr|Q9RSH	tr|Q9RSH1	717	3.99E+08	4.53E+08	2.73E+08	1.12E+08	1.65E+08	1.06E+08
sp|Q9RWE	sp|Q9RWE	320				15659000	35870000	36088000
sp|Q9RWE	sp|Q9RWE	320			40740000			
sp|Q9RUV	sp|Q9RUV	255	23671000	44720000	81286000	11462000	22250000	
sp|Q9RXH	sp|Q9RXH	372		27951000	58899000	40555000	66746000	55506000
sp|Q9RW5	sp|Q9RW5	310	49073000	79225000	48192000	38513000	57259000	38868000
tr|Q9RR62	tr|Q9RR62	467		22402000			23830000	18979000
tr|Q9RW1	tr|Q9RW1	1338						
tr|Q9RWN	tr|Q9RWN	1442	1.21E+08	2.56E+08	2.57E+08	1.06E+08	1.03E+08	88931000
tr|Q9RVR5	tr|Q9RVR5	1284	4.57E+08	6.27E+08	4.62E+08	4.06E+08	5.62E+08	4.86E+08
sp|Q9RYB	sp|Q9RYB	426						
sp|Q9RVP	sp|Q9RVP	294						
tr|Q9RYN	tr|Q9RYN3	1777	3.36E+08	3.87E+08	3.05E+08	3.37E+08	2.73E+08	2.7E+08
sp|Q9RXT	sp|Q9RXT	400	17613000	13038000	15895000	18485000	25200000	18198000
sp|Q9RYD	sp|Q9RYD	430	65874000	67773000	50762000	45281000	73915000	62123000
tr|Q9RXE7	tr|Q9RXE7	1578	14900000		22831000			
tr|Q9RYX6	tr|Q9RYX6	1835						
sp|Q9RWI	sp|Q9RWI	330						
tr|Q9RYR7	tr|Q9RYR7	1799	1.77E+08	1.64E+08	1.7E+08	1.22E+08	1.82E+08	1.65E+08
tr|Q9RVA2	tr|Q9RVA2	1199	73942000	86304000	83839000	62594000	90723000	64620000
sp|Q9RY3	sp|Q9RY3	413	1.71E+09	1.49E+09	1.62E+09	1.06E+09	1.74E+09	1.08E+09
tr|Q9RX69	tr|Q9RX69	1540	2065900					
tr|Q9RU28	tr|Q9RU28	981	54416000	54146000	47095000	23189000	20858000	28693000
tr|Q9RSW	tr|Q9RSW	775	58578000	50808000	47424000			
tr|Q9RU1	tr|Q9RU17	976	4.47E+08	59896000	4.45E+08	2.82E+08	3.48E+08	3.06E+08
tr|Q9RSZ5	tr|Q9RSZ5	790	27035000	59635000	36050000	35045000		39094000
tr|Q9RTL0	tr|Q9RTL0	892						
tr|Q9RYS7	tr|Q9RYS7	1806		52213000	41752000	26687000	52504000	24719000
tr|Q9RYN8	tr|Q9RYN8	1781	41321000	62545000	50996000	60447000		
tr|Q9RZV3	tr|Q9RZV3	1983	6913400	11870000	10702000	4080900	10392000	
sp|Q9RTA	sp|Q9RTA	175	52231000	51378000	46181000	24457000	46067000	
tr|Q9RY16	tr|Q9RY16	1680					48422000	
tr|Q9RY16	tr|Q9RY16	1680	66851000	77081000	81598000		25970000	40656000
tr|Q9RYH6	tr|Q9RYH6	1756					31062000	19118000
tr|Q9RS98	tr|Q9RS98	672	1.74E+08	1.5E+08	2.27E+08	2.05E+08	2.55E+08	2.94E+08
tr|Q9RSC1	tr|Q9RSC1	687	57632000		54100000	65116000	60943000	66970000
sp|Q9RTG	sp|Q9RTG	184						9098000
tr|Q9RWA	tr|Q9RWA	1379		13498000	12622000			
sp|Q5933	sp|Q5933	63	3.29E+08	4.37E+08	3.66E+08	2.21E+08	3.16E+08	2.22E+08
tr|Q9RZT6	tr|Q9RZT6	1973	8831000					
tr|Q9RU0	tr|Q9RU0	968						
tr|Q9RYS1	tr|Q9RYS1	1801	1.45E+08	1.31E+08	1.63E+08	25773000	41170000	37382000
sp|Q9RUV	sp|Q9RUV	255				31433000	41072000	32064000
sp|Q9RUP	sp|Q9RUP	243	60730000	1.03E+08	68661000	1.17E+08	1.57E+08	74876000
tr|Q9RXZ6	tr|Q9RXZ6	1671	33662000	40072000	41921000	21717000		54797000
sp|Q9RWF	sp|Q9RWF	343	9.79E+08	1.32E+09	1.22E+09	1.04E+09	1.59E+09	1.08E+09
tr|Q9RXA9	tr|Q9RXA9	1560						
tr|Q9RS11	tr|Q9RS11	627					6471200	
sp|Q9RVV	sp|Q9RVV	300	11845000	1.85E+08	2.2E+08	1.07E+08	1.9E+08	1.28E+08

sp|Q9RZ0	sp|Q9RZ0	450		1.26E+08		15073000		
tr|Q9RRR4	tr|Q9RRR4	569				10310000	8230600	
tr|Q9RSV5	tr|Q9RSV5	771	43361000	70717000	53711000	47543000	72796000	49991000
tr|Q9RSD0	tr|Q9RSD0	692		14829000	33414000	2.51E+08	1.68E+08	60127000
tr|Q9RXV8	tr|Q9RXV8	1653	67647000					
sp|Q9RVW	sp|Q9RVW	301					40543000	23158000
sp|Q9RW0	sp|Q9RW0	327	46945000	1.05E+08	1.51E+08	40688000		59185000
sp|Q9RSL4	sp|Q9RSL4	136	1.41E+09	1.95E+09	1.81E+09	3.58E+08	7.47E+08	3.36E+08
tr|Q9RRF4	tr|Q9RRF4	516						
tr|Q9RZ22	tr|Q9RZ22	1854		28846000	31527000	54633000	65483000	60816000
tr|Q9RVT9	tr|Q9RVT9	1298	24360000	28492000	17233000	30271000	27623000	32728000
tr|Q9RVN	tr|Q9RVN	1263		6139000				
tr|Q9RUZ5	tr|Q9RUZ5	1146			10588000			
tr|Q9RSM	tr|Q9RSM	735	75348000	1.37E+08	1.16E+08	91481000	95217000	85915000
tr|Q9RU07	tr|Q9RU07	972	1.23E+08	2.06E+08	3.51E+08	87819000	1.69E+08	1.35E+08
tr|Q9RUM	tr|Q9RUM	1089			10365000			
tr|Q9RW2	tr|Q9RW2	1345	48662000	50929000	51912000		50626000	42125000
tr|Q9RVN	tr|Q9RVN	1258						
sp|Q9RS3	sp|Q9RS3	109	39349000	55588000		22900000	40998000	32766000
tr|Q9RYT5	tr|Q9RYT5	1814				19079000	34406000	28102000
sp|Q9RU8	sp|Q9RU8	216		3588200	3325500		3564200	3798300
sp|Q9RTL8	sp|Q9RTL8	194	37461000	48243000				22118000
sp|Q9RUZ	sp|Q9RUZ	260			25163000			
tr|Q9RTM	tr|Q9RTM	902	17758000					
tr|Q9RV75	tr|Q9RV75	1189						
tr|Q9RXY8	tr|Q9RXY8	1666	2.78E+08	3.02E+08	2.65E+08	1.59E+08	1.92E+08	1.6E+08
sp|Q9RRS	sp|Q9RRS	96	4.66E+08	5.71E+08	4.77E+08	3.65E+08	4.79E+08	4E+08
tr|Q9RVQ	tr|Q9RVQ	1272	1.19E+09	1.11E+09	1.15E+09	3.05E+08	3.32E+08	3.69E+08
tr|Q9RWL	tr|Q9RWL	1434			23998000		20213000	
sp|Q9RVV	sp|Q9RVV	300	19876000	35449000	75714000	71678000	41633000	53519000
tr|Q9RVT2	tr|Q9RVT2	1294	53116000	90130000	62146000	94547000	66673000	68053000
tr|Q9RXY9	tr|Q9RXY9	1667	34506000	42378000	44726000		41984000	25576000
tr|Q9RTZ8	tr|Q9RTZ8	966	4.77E+08	5.18E+08	4.62E+08	1.84E+08	3.83E+08	3.38E+08
tr|Q9RX79	tr|Q9RX79	1545	36634000		45869000	62622000	78160000	56399000
tr|Q9RYD9	tr|Q9RYD9	1735	1.09E+08	1.19E+08	1.28E+08			
tr|Q9RTQ7	tr|Q9RTQ7	917	9.13E+08	1.06E+09	9.1E+08	6.28E+08	8.41E+08	5.8E+08
tr|Q9RV75	tr|Q9RV75	1189		13459000	29118000	32104000	36406000	27991000
tr|Q9RRS3	tr|Q9RRS3	574				42373000		
tr|Q9RUZ9	tr|Q9RUZ9	1150					12704000	
tr|Q9RVW	tr|Q9RVW	1314	7290000	6525500	6640500	18777000	29641000	16965000
sp|Q9RSS	sp|Q9RSS	148	3.42E+08	5.36E+08	2.57E+08	5.05E+08	6.02E+08	5.14E+08
tr|Q9RSZ5	tr|Q9RSZ5	790	60634000					
tr|Q9RZ67	tr|Q9RZ67	1865						
tr|Q9RRU1	tr|Q9RRU1	584	2.37E+08	2.48E+08	2.77E+08	2.79E+08	3.25E+08	2.49E+08
tr|Q9RZU7	tr|Q9RZU7	1979						
tr|Q9RRY5	tr|Q9RRY5	608						
tr|Q9RRR9	tr|Q9RRR9	571	41743000	53294000	63368000			
tr|Q9RS17	tr|Q9RS17	630	6.02E+08	6.42E+08	5.93E+08	7.43E+08	8.05E+08	6.82E+08
sp|Q9RTA	sp|Q9RTA	175	22020000	23026000	23243000	42612000	48343000	23291000
sp|Q9RTA	sp|Q9RTA	175						
tr|Q9RZU0	tr|Q9RZU0	1976		8481600	17275000			6273600

sp|Q9RRX	sp|Q9RRX	102						
tr|Q9RSY6	tr|Q9RSY6	784				42640000	47081000	12901000
tr|Q9RU57	tr|Q9RU57	1000	68201000	1.27E+08	1.05E+08	73050000	85762000	74299000
tr|Q9RV15	tr|Q9RV15	1159						
tr|Q9RUB	tr|Q9RUB	1029	3696200	8844500			4232100	
tr|Q9RVD2	tr|Q9RVD2	1214			24760000			
sp|Q9RVE	sp|Q9RVE	284	1.11E+09	7.42E+08	8.31E+08	1.02E+08	1.42E+08	1.6E+08
tr|Q9RTW	tr|Q9RTW	944						
tr|Q9RU71	tr|Q9RU71	1010						
tr|Q9RRB4	tr|Q9RRB4	495	1.6E+08	1.8E+08	1.58E+08	1.98E+08	2.11E+08	1.72E+08
sp|Q9RSH	sp|Q9RSH	120		42027000	10434000	13651000		
sp|Q9RUF	sp|Q9RUF	231		4823800		13474000		7764800
tr|Q9RZ07	tr|Q9RZ07	1845	6.33E+08	8.41E+08	7.05E+08	3.82E+08	5.06E+08	4.73E+08
tr|Q9RZ07	tr|Q9RZ07	1845						
tr|Q9RV68	tr|Q9RV68	1185					19045000	
sp|Q9RTY	sp|Q9RTY	202			85325000			
sp|Q9RUP	sp|Q9RUP	244						
sp|Q9RY4	sp|Q9RY4	416	2.53E+08	1.99E+08	2.35E+08		1.34E+08	
sp|Q9RY4	sp|Q9RY4	416	21883000	2.53E+08	2.43E+08	33447000	70723000	50918000
tr|Q9RRK9	tr|Q9RRK9	543						
tr|Q9RWT	tr|Q9RWT	1469	1.91E+08	3.77E+08	3.47E+08	2.13E+08	1.72E+08	1.71E+08
tr|Q9RWA	tr|Q9RWA	1379						
tr|Q9RS52	tr|Q9RS52	646	16716000	15628000	13355000	10862000	16081000	
tr|Q9RX76	tr|Q9RX76	1544	60642000	64596000	64123000	49566000	49203000	55292000
tr|Q9RYD9	tr|Q9RYD9	1735						
tr|Q9RW3	tr|Q9RW3	1350	50880000	67032000	51192000	23549000	26334000	40631000
tr|Q9RRH	tr|Q9RRH	527				27248000	44914000	33734000
tr|Q9RU08	tr|Q9RU08	973	1.82E+08	2.76E+08	2.85E+08	72093000	1.46E+08	1.04E+08
tr|Q9RY86	tr|Q9RY86	1710						15591000
tr|Q9RTZ0	tr|Q9RTZ0	961	30536000	61813000	54036000	9401100	33905000	17865000
tr|Q9RY74	tr|Q9RY74	1704	94249000	1.02E+08	1.32E+08			20934000
tr|Q9RRV2	tr|Q9RRV2	589		44976000		76264000	78682000	73673000
sp|Q9RW1	sp|Q9RW1	306	2.69E+08	4.93E+08	3.66E+08	3.57E+08	4.87E+08	1.05E+08
sp|Q9RW1	sp|Q9RW1	306	2.09E+08	3.3E+08	3.69E+08	27140000	44412000	63041000
tr|Q9RUS6	tr|Q9RUS6	1113	56160000	54471000	52191000	64548000	91641000	51559000
sp|Q9RWD	sp|Q9RWD	323		65772000	84568000	53152000	24601000	47000000
tr|Q9RUX5	tr|Q9RUX5	1134	26857000	67820000	54125000	19416000	29271000	20198000
tr|Q9RS93	tr|Q9RS93	668	26045000	46642000	25463000	38580000	68677000	39270000
tr|Q9RW2	tr|Q9RW2	1344	1.44E+08	2.28E+08	1.94E+08	2.23E+08	2.97E+08	2.8E+08
sp|Q9RYM	sp|Q9RYM	438	19719000	35656000	1.19E+08	3.14E+08	4.56E+08	3.39E+08
tr|Q9RRJ7	tr|Q9RRJ7	537				18151000	38611000	20658000
sp|Q9RU4	sp|Q9RU4	211	1.87E+08	2.35E+08	2.08E+08			82049000
sp|Q9RUV	sp|Q9RUV	257				10040000		
tr|Q9RRV8	tr|Q9RRV8	593	47847000	1.33E+08	1E+08		17934000	
tr|Q9RUA	tr|Q9RUA	1026						
tr|Q9RS85	tr|Q9RS85	661	25463000		28532000	34922000	52076000	
sp|Q9RYV	sp|Q9RYV	445				25625000	44466000	35851000
sp|Q9RSC	sp|Q9RSC	115	2E+08	2.09E+08	2.06E+08			
sp|Q9RUR	sp|Q9RUR	249	3.26E+08	4.99E+08	3.24E+08	45836000	1.4E+08	99999000
tr|Q9RTC6	tr|Q9RTC6	852					8683800	14776000
tr|Q9RVJ3	tr|Q9RVJ3	1240	99176000	1.04E+08	1.29E+08	19286000	39190000	45107000

tr|Q9RZ13	tr|Q9RZ13	1850		1.27E+08		20426000		14474000
tr|Q9RW9	tr|Q9RW9	1377						
tr|Q9RV31	tr|Q9RV31	1167	35930000	58706000		51824000	74531000	90627000
tr|Q9RYG7	tr|Q9RYG7	1751		15674000				
sp|Q9RV9	sp|Q9RV9	273	12625000		9137700	12865000		
tr|Q9RVM	tr|Q9RVM	1256						
tr|Q9RZL6	tr|Q9RZL6	1944	2.72E+08	2.49E+08	3.34E+08	89301000	1.68E+08	2.13E+08
tr|Q9RTL3	tr|Q9RTL3	894	19498000	27712000	32468000			
tr|Q9RS29	tr|Q9RS29	636	9621900	12415000	13660000	3821800	7358600	6093600
tr|Q9RZC4	tr|Q9RZC4	1903	3363300		2166900		2022600	
tr|Q9RSZ0	tr|Q9RSZ0	788	1.4E+08	1.36E+08	1.12E+08	66727000	1.02E+08	68217000
tr|Q9RUB	tr|Q9RUB	1027	68206000	75233000	98550000	56859000	95049000	81373000
tr|Q9RUK0	tr|Q9RUK0	1075	74714000	75088000	66295000	93780000	1.41E+08	88519000
tr|Q9RRA4	tr|Q9RRA4	487		6861500	14207000			
tr|Q9RW9	tr|Q9RW9	1376		36697000		21369000		
tr|Q9RS10	tr|Q9RS10	626	1504500					
sp|Q9RW9	sp|Q9RW9	317		52345000	45277000			
tr|Q9RWP	tr|Q9RWP	1445	95811000	1.34E+08	1.19E+08	40629000	1.01E+08	80956000
tr|Q9RTR4	tr|Q9RTR4	923		32095000	35557000	66570000	73539000	59754000
tr|Q9RYS1	tr|Q9RYS1	1801	97848000	1.47E+08	1.65E+08			
tr|Q9RXP4	tr|Q9RXP4	1624	63929000	72540000	49449000	17149000	30292000	
tr|Q9RT80	tr|Q9RT80	834					10671000	
tr|Q9RRR3	tr|Q9RRR3	568	74632000	94691000	85088000	60532000	87817000	63254000
sp|Q9RT6	sp|Q9RT6	170						
sp|Q9RU0	sp|Q9RU0	234			8473700		5498600	
sp|Q9RT0	sp|Q9RT0	161	16692000	14331000	9924100	6133000		
tr|Q9RXP7	tr|Q9RXP7	1626	30267000	34698000	38407000		6753200	
tr|Q9RRT5	tr|Q9RRT5	579	23196000	25796000	21802000	20559000		31436000
tr|Q9RXM	tr|Q9RXM	1617	24728000	28280000	27465000	83596000	58470000	77056000
tr|Q9RXG1	tr|Q9RXG1	1588		21425000	11010000	10653000	18210000	
tr|Q9RXA9	tr|Q9RXA9	1560	47540000	54042000		26579000	61602000	
tr|Q9RZI5	tr|Q9RZI5	1936	48045000					
sp|Q9RUY	sp|Q9RUY	259		88659000	1.04E+08	2E+08	2.77E+08	2.02E+08
sp|Q9RUY	sp|Q9RUY	259	33024000	71951000	99313000	45988000	1.17E+08	1.15E+08
tr|Q9RWB	tr|Q9RWB	1385			15247000			
sp|Q9RXJ0	sp|Q9RXJ0	375	3.05E+08	3.9E+08	4.02E+08	1.87E+08	1.96E+08	2.21E+08
tr|Q9RVJ0	tr|Q9RVJ0	1238	5.38E+08	4.04E+08	6.14E+08	1.09E+08	1.66E+08	2.09E+08
tr|Q9RXL1	tr|Q9RXL1	1609						
tr|Q9RSX3	tr|Q9RSX3	778						
tr|Q9RZP7	tr|Q9RZP7	1955						
tr|Q9RZP7	tr|Q9RZP7	1955						
sp|Q9RXJ2	sp|Q9RXJ2	377	3.79E+08	2.04E+08	2.27E+08	1.58E+08	1.29E+08	1.87E+08
tr|Q9RVP5	tr|Q9RVP5	1269	6.14E+08	3.83E+08	4.24E+08	1.4E+09	1.2E+09	1.36E+09
sp|Q9RU8	sp|Q9RU8	216	37165000	17608000	17541000		15066000	
sp|Q9RU8	sp|Q9RU8	216		79917000	64389000	1.23E+08	1.25E+08	1.3E+08
tr|Q9RVT5	tr|Q9RVT5	1296						
tr|Q9RVW	tr|Q9RVW	1311		23165000			22510000	
tr|Q9RX10	tr|Q9RX10	1507			1.43E+08			5511600
tr|Q9RY33	tr|Q9RY33	1686	5.26E+08	8.53E+08	6.95E+08	7.02E+08	8.66E+08	6.47E+08
tr|Q9RRP5	tr|Q9RRP5	559						
tr|Q9RRP5	tr|Q9RRP5	559						11752000

sp|Q9RSW	sp|Q9RSW	154	1.16E+09	1.33E+09	1.46E+09	1.01E+09	6.82E+08	1.07E+09
sp|Q9RSW	sp|Q9RSW	154		8213800	19013000	15235000		29919000
sp|Q9RRB	sp|Q9RRB	76						
sp|Q9RY5	sp|Q9RY5	419	4.77E+08	6.95E+08	4.55E+08	5.4E+08	4.74E+08	5.16E+08
sp|Q9RY5	sp|Q9RY5	419	80747000	1.72E+08	5.17E+08	8.09E+08	1.01E+09	8.78E+08
tr|Q9RU9	tr|Q9RU98	1018	40149000	54736000	39950000	51996000	48079000	38755000
tr|Q9RT4	tr|Q9RT43	815	2E+08	2.24E+08	2.15E+08	2.04E+08	2.2E+08	2.75E+08
sp|Q9RRE	sp|Q9RRE	81						
sp|Q9RRE	sp|Q9RRE	81				33387000	33051000	36516000
tr|Q9RTE	tr|Q9RTE9	865	55436000	60476000	52808000			
tr|Q9RUD	tr|Q9RUD	1039						
tr|Q9RU0	tr|Q9RU01	969	60066000	47995000	55521000	2.29E+08	2.51E+08	1.32E+08
tr|Q9RTG	tr|Q9RTG7	873						
tr|Q9RX1	tr|Q9RX10	1507	5948100		44924000			
sp|Q9RXR	sp|Q9RXR	395	24371000	35416000	25415000	18439000	15954000	8618200
tr|Q9RZI6	tr|Q9RZI6	1937						
sp|Q9RYB	sp|Q9RYB	426	2.3E+08	1.25E+08	1.54E+08			
tr|Q9RZH	tr|Q9RZH	1929						
tr|Q9RVP	tr|Q9RVP0	1266	3.29E+08	2.94E+08	2.31E+08			
tr|Q9RS71	tr|Q9RS71	655	1.83E+09	1.46E+09	1.58E+09	5.17E+08	6.77E+08	9.11E+08
tr|Q9RS71	tr|Q9RS71	655		1.53E+08	2.24E+08	1.66E+08		3.83E+08
tr|Q9RSL6	tr|Q9RSL6	732	53827000	53968000	41739000			15596000
tr|Q9RY3	tr|Q9RY30	1685						
tr|Q9RXV	tr|Q9RXV5	1651		1.01E+08	93575000	16176000	18392000	
sp|Q9RRB	sp|Q9RRB	76	1.08E+08	1.57E+08	1.75E+08	33768000	38698000	64102000
sp|Q9RU8	sp|Q9RU8	217	1.26E+08	90279000	82402000	2.06E+08	1.83E+08	1.97E+08
tr|Q9RY0	tr|Q9RY08	1678						
tr|Q9RXX	tr|Q9RXX2	1660	63898000		53959000		30393000	
tr|Q9RXX	tr|Q9RXX2	1660	1.43E+08	1.25E+08	1.92E+08			
tr|Q9RZ1	tr|Q9RZ12	1849					37087000	40816000
tr|Q9RVK	tr|Q9RVK6	1246	4.07E+08	2.82E+08	4.21E+08		94671000	
tr|Q9RVK	tr|Q9RVK6	1246			25768000			
tr|Q9RWW	tr|Q9RWW	1493	8.69E+08	5.2E+08	6.42E+08	2.37E+08	3.38E+08	3.86E+08
tr|Q9RWB	tr|Q9RWB	1385				15272000	10785000	
tr|Q9RRG	tr|Q9RRG5	521	2.97E+08	3.35E+08	3.4E+08	1.92E+08	2.64E+08	2.65E+08
tr|Q9RTD	tr|Q9RTD6	859	43562000	1.2E+08	44260000	81805000	46715000	33735000
sp|Q9RXY	sp|Q9RXY	407	1.34E+08	1.16E+08	1.26E+08			11984000
tr|Q9RTJ1	tr|Q9RTJ1	883	11825000			22030000	19836000	16860000
tr|Q9RTY1	tr|Q9RTY1	956			17741000	25486000		
tr|Q9RVT	tr|Q9RVT5	1296	2.34E+08	1.54E+08	2.15E+08	98705000	96879000	1.37E+08
tr|Q9RTZ	tr|Q9RTZ9	967		17837000	22333000		50695000	46775000
tr|Q9RTW	tr|Q9RTW	941	12680000					
tr|Q9RXG	tr|Q9RXG1	1588				6207600	4912100	4081400
tr|Q9RYV	tr|Q9RYV3	1821				1.67E+08	1.22E+08	1.51E+08
tr|Q9RYV	tr|Q9RYV3	1821	1.47E+08	1.79E+08	2.65E+08	2.33E+08	3.03E+08	3.03E+08
tr|Q9RTQ	tr|Q9RTQ9	919		34121000	26520000	63056000	77094000	69769000
tr|Q9RW2	tr|Q9RW2	1343	32467000	31618000	28445000	15320000	17790000	21035000
sp|Q9RUE	sp|Q9RUE	226		3298000	4003300		3580300	2266900
sp|Q9RUE	sp|Q9RUE	226			16902000			
tr|Q9RW6	tr|Q9RW6	1359						
sp|Q9RRT	sp|Q9RRT	97		20215000	20208000	18997000	22913000	

tr|Q9RYD	tr|Q9RYD	1735	2.74E+08	1.78E+08	2.15E+08	1.47E+08	1.65E+08	1.59E+08
sp|Q9RU2	sp|Q9RU2	209	3.81E+08	1.6E+08	5.47E+08	5.25E+08	3.82E+08	1.06E+09
sp|Q9RRB	sp|Q9RRB	76	30935000	36647000	54756000	22503000	11040000	
sp|Q9RRB	sp|Q9RRB	76			12782000			
tr|Q9RZC	tr|Q9RZC7	1906			13112000			
tr|Q9RWC	tr|Q9RWC	1388		25842000	23044000	46509000	36227000	38973000
tr|Q9RUH	tr|Q9RUH	1059	3.44E+08	2.84E+08	2.77E+08	4.88E+08	3.65E+08	4.08E+08
sp|Q9RXK	sp|Q9RXK	386	6.67E+08	6.15E+08	6.68E+08	3.94E+08	3.95E+08	6.25E+08
tr|Q9RU5	tr|Q9RU5	994	90893000	83850000	81888000	1.9E+08	1.86E+08	2.62E+08
tr|Q9RS47	tr|Q9RS47	643	63640000			65114000	47794000	57341000
tr|Q9RVP1	tr|Q9RVP1	1267	1.34E+09	2.04E+09	1.46E+09	3.29E+09	2.71E+09	2.63E+09
tr|Q9RVP1	tr|Q9RVP1	1267	3.38E+08	3.11E+08	6.1E+08	2.62E+08	1.79E+08	2.64E+08
tr|Q9RZ8	tr|Q9RZ8	1878		12872000		8664400		
tr|Q9RSM	tr|Q9RSM	735	7.78E+08	1.02E+09	7.03E+08	6.01E+08	5.56E+08	4.77E+08
tr|Q9RYQ	tr|Q9RYQ	1788	4.3E+09	3.54E+09	3.7E+09	1.91E+09	1.79E+09	2.05E+09
sp|Q9RU8	sp|Q9RU8	216					26997000	
tr|Q9RX1	tr|Q9RX1	1507						12748000
tr|Q9RX1	tr|Q9RX1	1507		23282000	43203000	33871000	47165000	51368000
sp|Q9RTF	sp|Q9RTF	182	1.07E+08	1E+08	92579000	1.68E+08	1.74E+08	1.64E+08
tr|Q9RYX	tr|Q9RYX	1835	5.61E+08	5.87E+08	5.51E+08	4.88E+08	4.56E+08	4.54E+08
tr|Q9RVJ0	tr|Q9RVJ0	1238			32611000			
tr|Q9RWI	tr|Q9RWI	1417	41520000	47669000	99508000	81293000	98011000	1.16E+08
tr|Q9RXX	tr|Q9RXX	1660	15665000	26916000	44317000			
tr|Q9RWS	tr|Q9RWS	1464				19198000		12534000
sp|Q9RRB	sp|Q9RRB	76	4.34E+08	3.79E+08	3.22E+08	2.34E+08	1.84E+08	
sp|Q9RTN	sp|Q9RTN	196	3.71E+08	5.23E+08	4.43E+08	1.39E+08	1.35E+08	2.53E+08
tr|Q9RY7	tr|Q9RY7	1708		13037000	14801000	9738300		
tr|Q9RU0	tr|Q9RU0	970	40603000	34408000		32317000	36572000	37487000
tr|Q9RTN	tr|Q9RTN	903	64702000	36370000	64806000	1.1E+08	49689000	1.28E+08
sp|Q9RR7	sp|Q9RR7	67	59983000	69902000	85368000	93059000	1.1E+08	63620000
sp|Q9RR7	sp|Q9RR7	67	25468000	1.34E+08	1.97E+08			
tr|Q9RT2	tr|Q9RT2	805						
sp|Q9RSR	sp|Q9RSR	143						
sp|Q9RW	sp|Q9RW	327	4.07E+08	4.22E+08	4.23E+08	6.65E+08	5.38E+08	6.13E+08
sp|Q9RSH	sp|Q9RSH	120	66589000	89790000	90308000	73757000	97032000	54573000
tr|Q9RV7	tr|Q9RV7	1190	1.34E+09	1.83E+09	1.51E+09	1.05E+09	8.13E+08	1.07E+09
sp|Q9RY	sp|Q9RY	438	3.55E+08	2.07E+08	2.21E+08	4.42E+08	3.63E+08	3.86E+08
tr|Q9RZU	tr|Q9RZU	1979						
sp|Q9RR8	sp|Q9RR8	69			16082000			
tr|Q9RYD	tr|Q9RYD	1735	1.1E+08	2.42E+08	2.77E+08	2.15E+08	1.34E+08	1.12E+08
tr|Q9RYE	tr|Q9RYE	1736	16361000					
sp|Q9RX1	sp|Q9RX1	351	37590000					68428000
tr|Q9RYR1	tr|Q9RYR1	1796	51916000	52713000	46628000	31861000		
sp|Q9RS2	sp|Q9RS2	108	57547000	78325000	62374000	86123000	1.18E+08	94383000
tr|Q9RSX	tr|Q9RSX	781	1.58E+08	1.67E+08	1.52E+08	1.03E+08	90134000	1.04E+08
tr|Q9RVP1	tr|Q9RVP1	1267						
tr|Q9RYI1	tr|Q9RYI1	1760	5.55E+08	4.85E+08	5.6E+08	3.71E+08	2.63E+08	3.64E+08
sp|Q9RW	sp|Q9RW	311						
tr|Q9RTM	tr|Q9RTM	899	1.37E+08	1.22E+08	1.03E+08	1.79E+08	1.99E+08	1.84E+08
tr|Q9RTM	tr|Q9RTM	899			30280000			
sp|Q9RUY	sp|Q9RUY	259	3.78E+08	4.55E+08	4.39E+08	2.3E+08	2.72E+08	2.65E+08

tr|Q9RTG	tr|Q9RTG	873						
tr|Q9RW9	tr|Q9RW9	1377	1.98E+08	2.21E+08	1.84E+08	1.78E+08	1.44E+08	1.3E+08
tr|Q9RY8	tr|Q9RY86	1710	15726000	17667000	9963200			
tr|Q9RX02	tr|Q9RX02	1505	57589000	54871000	59280000	24054000	37092000	20416000
tr|Q9RXG	tr|Q9RXG	1587	10079000		11007000			
tr|Q9RYM	tr|Q9RYM	1773	2.58E+08	2.58E+08	1.92E+08	2.59E+08	1.93E+08	2.04E+08
tr|Q9RXD	tr|Q9RXD	1573	48153000		29673000			
tr|Q9RVQ	tr|Q9RVQ	1273	37425000	37248000	1.59E+08	1.02E+08	1.6E+08	1.62E+08
sp|Q9RTL	sp|Q9RTL	194						
sp|Q9RY3	sp|Q9RY3	413	36078000	42683000	34383000		31759000	
tr|Q9RYM	tr|Q9RYM	1773						
tr|Q9RWV	tr|Q9RWV	1484				2.18E+08	3.03E+08	2.46E+08
sp|Q9RYP	sp|Q9RYP	440	21403000	19835000	15578000	27167000	19679000	24010000
sp|Q9RVX	sp|Q9RVX	302		37930000	25423000			18977000
sp|Q9RS6	sp|Q9RS6	111						
sp|Q9RYM	sp|Q9RYM	438	1.04E+08	1.1E+08	1.09E+08	3.69E+08	2.37E+08	2.72E+08
sp|Q9RRY	sp|Q9RRY	104			32540000	32049000	44001000	36624000
sp|Q9RSE	sp|Q9RSE	116	2.26E+08	2.38E+08	1.76E+08	2.72E+08	2.36E+08	2.59E+08
tr|Q9RWT	tr|Q9RWT	1470	1.49E+08	1.71E+08	1.51E+08	3.34E+08	4.04E+08	2.8E+08
tr|Q9RZV2	tr|Q9RZV2	1982	3.23E+08	3.41E+08	2.04E+08	2.02E+08	1.4E+08	1.61E+08
tr|Q9RWB	tr|Q9RWB	1386						
sp|Q9RXR	sp|Q9RXR	396			34026000	54787000		59296000
tr|Q9RY38	tr|Q9RY38	1691						
tr|Q9RSU	tr|Q9RSU	762	85183000	1.24E+08	1.08E+08	96573000	1.1E+08	1.34E+08
tr|Q9RSI1	tr|Q9RSI1	721	4362500	14195000	21485000			
sp|Q9RXB	sp|Q9RXB	365	43154000	34030000	36301000	34300000	39988000	45143000
sp|Q9RXG	sp|Q9RXG	370			4726400			
sp|Q9RSJ7	sp|Q9RSJ7	125						
tr|Q9RUR	tr|Q9RUR	1105			14555000			
tr|Q9RVG	tr|Q9RVG	1230				16998000		
tr|Q9RVG	tr|Q9RVG	1230			1.11E+08	25592000	1.99E+08	38296000
tr|Q9RXH	tr|Q9RXH	1593	35744000	48520000	33007000	52932000	45991000	53339000
tr|Q9RTH	tr|Q9RTH	877	1.26E+09	1.2E+09	1.22E+09	8.57E+08	5.46E+08	9.22E+08
tr|Q9RSL7	tr|Q9RSL7	733						3937100
tr|Q9RTQ	tr|Q9RTQ	917	4.72E+08	3.22E+08	3E+08	8.33E+08	7.77E+08	7.39E+08
tr|Q9RYE8	tr|Q9RYE8	1740		14370000	16120000	47638000	46166000	45828000
tr|Q9RV8	tr|Q9RV8	1192			24238000			
tr|Q9RV8	tr|Q9RV8	1192	1.21E+09	1.37E+09	2.36E+09	3.19E+08	4.69E+08	4.64E+08
tr|Q9RRR	tr|Q9RRR	571					12143000	14452000
tr|Q9RY67	tr|Q9RY67	1701	97469000		75255000	1.7E+08	2.04E+08	1.8E+08
tr|Q9RY67	tr|Q9RY67	1701	16568000	14662000	30602000	8384700		
tr|Q9RTR	tr|Q9RTR	923	4E+08	7.45E+08	3.26E+08	5.1E+08	3.28E+08	3.88E+08
tr|Q9RY5	tr|Q9RY55	1697						
tr|Q9RU2	tr|Q9RU2	978						
tr|Q9RTC	tr|Q9RTC2	849						
tr|Q9RTC	tr|Q9RTC2	849						
tr|Q9RTI9	tr|Q9RTI9	882						
sp|Q9RYB	sp|Q9RYB	427	1.95E+08	2.63E+08	1.88E+08	1.47E+08	1.91E+08	
tr|Q9RXX	tr|Q9RXX2	1660		49421000			13186000	
tr|Q9RTM	tr|Q9RTM	899	1.29E+08	1.3E+08	1.48E+08	3.13E+08	2.46E+08	2.87E+08
tr|Q9RUK	tr|Q9RUK	1080	3.34E+08	3.28E+08	2.46E+08	3.26E+08	3.56E+08	3.27E+08

tr|Q9RR9	tr|Q9RR98	481	30612000	15415000	15737000			
tr|Q9RUH	tr|Q9RUH	1057				1.29E+08	1.22E+08	1.29E+08
sp|Q9RX3	sp|Q9RX3	356				42704000		
tr|Q9RWP	tr|Q9RWP	1449				1.3E+08	1.44E+08	
tr|Q9RYE	tr|Q9RYE9	1741		49753000				
sp|Q9RXJ	sp|Q9RXJ4	379		23059000	10764000	23745000		
sp|Q9RXJ	sp|Q9RXJ4	379		14746000	26839000			
sp|Q9RSQ	sp|Q9RSQ	142	55684000	77115000	2.19E+08	18512000	40167000	39202000
sp|Q9RSL	sp|Q9RSL	133			14783000	12374000	16395000	20030000
tr|Q9RX5	tr|Q9RX56	1531	9.08E+08	8.96E+08	8.89E+08	1.53E+09	1.08E+09	1.15E+09
tr|Q9RX5	tr|Q9RX56	1531						
sp|Q9RSJ	sp|Q9RSJ9	127	31111000	19890000	21966000	17172000	18334000	
sp|Q9RSJ	sp|Q9RSJ9	127			65029000	1.52E+08	2.39E+08	56880000
sp|Q9RUP	sp|Q9RUP	246	18114000				19566000	
sp|Q9RSF	sp|Q9RSF	117		58012000				
tr|Q9RY3	tr|Q9RY35	1688	56179000	57346000	53720000		21921000	31771000
tr|Q9RRC	tr|Q9RRC5	500	46075000	33482000	38983000			
sp|Q9RVT	sp|Q9RVT	299	1.26E+08	1.08E+08	1.39E+08	2.82E+08	97116000	1.16E+08
tr|Q9RTR	tr|Q9RTR4	923	5037000					
tr|Q9RXH	tr|Q9RXH8	1599	2.17E+08	2.14E+08	1.89E+08	68940000	58441000	62017000
tr|Q9RW9	tr|Q9RW9	1373	16107000	34954000	28168000		15592000	17307000
tr|Q9RS0	tr|Q9RS05	622						
tr|Q9RRC	tr|Q9RRC7	501	12558000	33051000	29667000	21272000	34472000	12926000
tr|Q9RU2	tr|Q9RU27	980	1.36E+08	1.93E+08	1.78E+08		1.22E+08	1.09E+08
tr|Q9RU2	tr|Q9RU27	980		26533000	60645000	19456000	54724000	29234000
tr|Q9RXF	tr|Q9RXF0	1581				2.09E+08	1.76E+08	1.84E+08
tr|Q9RXF	tr|Q9RXF0	1581			92343000		1.62E+08	1.54E+08
tr|Q9RVP1	tr|Q9RVP1	1267	1.21E+08	47663000	2.36E+08		30554000	41516000
sp|Q9RRG	sp|Q9RRG	84	1.85E+09	1.6E+09	1.79E+09			
tr|Q9RYM	tr|Q9RYM	1773			32126000			
tr|Q9RYS2	tr|Q9RYS2	1802	1.34E+08	1.7E+08	1.51E+08		57692000	
tr|Q9RY5	tr|Q9RY56	1698	91330000	1.4E+08	1.44E+08	1.67E+08	1.9E+08	1.66E+08
sp|Q9R34	sp|Q9R34	64						
sp|Q9RSL	sp|Q9RSL	133	2.85E+08	1.28E+08	2.58E+08	28039000	30176000	
sp|Q9RTF	sp|Q9RTF5	182		37993000	35874000	89665000	91842000	48576000
tr|Q9RWU	tr|Q9RWU	1478	66667000	44730000	66179000	38464000	48419000	
tr|Q9RXQ	tr|Q9RXQ	1628	13517000	18139000	50428000	10980000	17926000	17770000
tr|Q9RWT	tr|Q9RWT	1465	20107000	1.33E+08	1.83E+08	32329000	80417000	50882000
tr|Q9RT87	tr|Q9RT87	837						
sp|P8358	sp|P8358	60				37308000		41419000
tr|Q9RRB	tr|Q9RRB3	494						
tr|Q9RY67	tr|Q9RY67	1701	1.63E+08	2.09E+08	2.6E+08	2.21E+08	3.45E+08	2.96E+08
tr|Q9RY7	tr|Q9RY79	1708	1.56E+08	1.3E+08	1.72E+08	61840000	52284000	87750000
sp|Q9RY2	sp|Q9RY2	411						
tr|Q9RYV	tr|Q9RYV0	1820				64132000	35673000	36980000
tr|Q9RX3	tr|Q9RX33	1519	26390000	51531000	40082000	30464000	35328000	33146000
tr|Q9RUD	tr|Q9RUD	1039	1.48E+09	1.71E+09	1.56E+09	7.48E+08	8.4E+08	1.27E+09
tr|Q9RVJ3	tr|Q9RVJ3	1240						
sp|Q9RTB	sp|Q9RTB	176	47194000	71770000	59357000	32643000	45121000	
tr|Q9RSM	tr|Q9RSM	737		5858200	4715500	11910000	14350000	13254000
sp|Q9RVI	sp|Q9RVI3	289						

tr|Q9RWV	tr|Q9RWV	1482	1.58E+08	1.7E+08	1.63E+08			1.22E+08
sp|P52153	sp|P52153	52	35653000	32321000	18447000		15865000	
tr|Q9RXU9	tr|Q9RXU9	1649					11885000	
tr|Q9RSC3	tr|Q9RSC3	688	7152800		12382000			7061900
tr|Q9RVM	tr|Q9RVM	1256	28117000		22907000	70771000	73974000	60965000
tr|Q9RYV3	tr|Q9RYV3	1821	2.93E+08	2.79E+08	2.56E+08	6.45E+08	5.5E+08	5.98E+08
tr|Q9RYV3	tr|Q9RYV3	1821	26715000	39562000	1.07E+08	1.13E+08	82802000	96074000
sp|Q9RWJ	sp|Q9RWJ	333						13503000
tr|Q9RUC	tr|Q9RUC	1034						
sp|Q9RSP	sp|Q9RSP	139	74006000	62934000	64543000	59577000	75864000	62502000
sp|Q9RSE	sp|Q9RSE	116	90702000		1.05E+08	99983000	98955000	
tr|Q9RSD8	tr|Q9RSD8	698				91468000	89859000	
tr|Q9RRK2	tr|Q9RRK2	539	35931000	23944000	26051000	17449000		15776000
tr|Q9RTZ8	tr|Q9RTZ8	966	1E+08	84892000	67942000	36152000	27274000	36627000
tr|Q9RWY	tr|Q9RWY	1500						16429000
tr|Q9RWQ	tr|Q9RWQ	1453						
sp|Q9RY6	sp|Q9RY6	421				33282000	25786000	16467000
tr|Q9RWC	tr|Q9RWC	1388	55797000	31714000	46404000			
sp|Q9RZV	sp|Q9RZV	460				22793000	11331000	16425000
tr|Q9RSE3	tr|Q9RSE3	702	33302000	31335000	26735000	1.29E+08	1.04E+08	1.15E+08
sp|Q9RU2	sp|Q9RU2	209	3.02E+08	3.03E+08	4.65E+08	93467000	91375000	1.31E+08
sp|Q9RU2	sp|Q9RU2	209				2.08E+08	2.14E+08	2.28E+08
tr|Q9RU34	tr|Q9RU34	983						
tr|Q9RY75	tr|Q9RY75	1705		25467000	29460000	11150000		17205000
tr|Q9RS17	tr|Q9RS17	630	8.47E+08	8.53E+08	7.48E+08	2.1E+08	5.36E+08	2.53E+08
tr|Q9RR62	tr|Q9RR62	467	6600000					
tr|Q9RSA5	tr|Q9RSA5	676	16392000	4818500	4717800	3813500	4260600	4111700
tr|Q9RRU9	tr|Q9RRU9	586						
tr|Q9RX19	tr|Q9RX19	1514			30193000	29654000	35201000	27560000
tr|Q9RR62	tr|Q9RR62	467	23941000	50895000	1.51E+08	1.74E+08	2.02E+08	1.79E+08
tr|Q9RYY9	tr|Q9RYY9	1839		2584800				
tr|Q9RU02	tr|Q9RU02	970	37818000	51786000	48274000	49719000	52208000	50398000
tr|Q9RRR8	tr|Q9RRR8	570	1.56E+08	2.36E+08	1.68E+08	60255000	36703000	46662000
tr|Q9RU43	tr|Q9RU43	990						
tr|Q9RTQ3	tr|Q9RTQ3	914	56387000	71473000	74193000		90896000	94518000
tr|Q9RZD1	tr|Q9RZD1	1907	1.26E+08	31820000	96187000			
tr|Q9RWI0	tr|Q9RWI0	1413	34915000	34685000	27265000	29573000	24783000	18922000
sp|Q9RWH	sp|Q9RWH	330	10019000		29819000	15282000		9629000
tr|Q9RXE9	tr|Q9RXE9	1580						
tr|Q9RRK6	tr|Q9RRK6	541	2.43E+08	2.03E+08	1.7E+08		1.17E+08	51675000
sp|Q9RWH	sp|Q9RWH	328						
sp|Q9RY5	sp|Q9RY5	418				11164000	15524000	14952000
tr|Q9RYD9	tr|Q9RYD9	1735	13789000					
tr|Q9RTE3	tr|Q9RTE3	862						
tr|Q9RTE3	tr|Q9RTE3	862						
tr|Q9RSD1	tr|Q9RSD1	693						
tr|Q9RW9	tr|Q9RW9	1374	1286400		617420	1498700	1007900	1094200
tr|Q9RYS6	tr|Q9RYS6	1805	2455900	872950	822370	662500	986270	877030
tr|Q9RVQ	tr|Q9RVQ	1274	64933000	1.1E+08	1.5E+08	1.63E+08	2.44E+08	1.34E+08
tr|Q9RXB2	tr|Q9RXB2	1563		33517000		18086000	23111000	34921000
tr|Q9RVM	tr|Q9RVM	1257	1.12E+08	89550000	1.64E+08	51730000	54723000	78707000

tr|Q9RUG	tr|Q9RUG	1055	73251000	1.16E+08	96298000	84746000	83745000	1.26E+08
tr|Q9RX36	tr|Q9RX36	1520						
tr|Q9RTM	tr|Q9RTM	899	2.59E+08	5.72E+08	5.12E+08	7.08E+08	6.08E+08	5.25E+08
tr|Q9RZC7	tr|Q9RZC7	1906	76742000	1.17E+08	2.61E+08	72750000	92204000	1.34E+08
tr|Q9RT74	tr|Q9RT74	830	80131000	1.22E+08	1.86E+08			
tr|Q9RVQ	tr|Q9RVQ	1274			43285000			
tr|Q9RYP6	tr|Q9RYP6	1785				54510000	41100000	43633000
tr|Q9RYS2	tr|Q9RYS2	1802	9307100	6028700	17136000	38488000		
sp|Q9RT3	sp|Q9RT3	166			9242100			
tr|Q9RVE5	tr|Q9RVE5	1219	70999000	1.06E+08	1.01E+08	60940000		67573000
sp|Q9RXU	sp|Q9RXU	402	83359000	95869000	86543000	48761000	72963000	69504000
tr|Q9RSH8	tr|Q9RSH8	719	1.6E+08	1.54E+08	2.3E+08			
tr|Q9RUA	tr|Q9RUA	1023		19990000	25338000		29570000	
sp|Q9RW0	sp|Q9RW0	342	1.62E+09	1.76E+09	1.48E+09	1.51E+09	1.24E+09	1.38E+09
sp|Q9RW0	sp|Q9RW0	342	1.89E+08	4.23E+08	8.78E+08	5.61E+08	8.58E+08	8.01E+08
sp|Q9RW0	sp|Q9RW0	326	47066000	54612000	63514000	30131000		17364000
tr|Q9RS15	tr|Q9RS15	629				16919000		
sp|Q9RUV	sp|Q9RUV	254	72010000	79059000	64490000	1.43E+08	88163000	70829000
tr|Q9RXF0	tr|Q9RXF0	1581		1015100		2437700	2949200	2593600
tr|Q9RXF0	tr|Q9RXF0	1581	11857000	4804100	1738300	5104100	10696000	
sp|Q9RVE	sp|Q9RVE	284	70383000	99854000	2.11E+08	27237000	69871000	34166000
tr|Q9RWU	tr|Q9RWU	1474	1.37E+09	1.23E+09	1.13E+09	1.15E+09	1.07E+09	1.07E+09
tr|Q9RWU	tr|Q9RWU	1474	29789000	49482000	37518000			
tr|Q9RVP1	tr|Q9RVP1	1267	1.73E+08	2.29E+08	1E+08	1.8E+08	2.11E+08	2.08E+08
tr|Q9RU54	tr|Q9RU54	997	70028000	1.38E+08	2.35E+08	1.26E+08	1.16E+08	91129000
tr|Q9RRA6	tr|Q9RRA6	489		24700000		54059000	63479000	
tr|Q9RRA6	tr|Q9RRA6	489			35379000			
tr|Q9RRG6	tr|Q9RRG6	522	66198000	32150000	23685000	20952000	26767000	23027000
tr|Q9RVJ0	tr|Q9RVJ0	1238						
tr|Q9RTU7	tr|Q9RTU7	935	2.56E+08	2.02E+08	2.43E+08	1.55E+08	1.51E+08	1.73E+08
tr|Q9RS71	tr|Q9RS71	655	1.17E+08	1.41E+08	1.4E+08	3.44E+08	2.27E+08	2.69E+08
sp|Q9RU7	sp|Q9RU7	214	1.16E+08	12284000	1.01E+08			
sp|Q9RXX	sp|Q9RXX	405		32882000				
tr|Q9RRC0	tr|Q9RRC0	497	18244000	17017000	12234000	64217000		44630000
sp|Q9RT2	sp|Q9RT2	164	66060000	79178000	81387000	55141000	70023000	88397000
sp|Q9RUV	sp|Q9RUV	257						
sp|Q9RUV	sp|Q9RUV	257	1.23E+08	1.12E+08	2.99E+08	73115000	72044000	90630000
tr|Q9RZJ2	tr|Q9RZJ2	1939						
sp|Q9RR6	sp|Q9RR6	66	3E+08	1.83E+08	2.36E+08	30517000	44309000	21911000
tr|Q9RYY7	tr|Q9RYY7	1837						
sp|Q9RXK	sp|Q9RXK	386	42632000	20263000	38424000	1.13E+08	82087000	20265000
tr|Q9RU34	tr|Q9RU34	983		14113000			20066000	31086000
sp|Q9RSR	sp|Q9RSR	144			481730			573240
sp|Q9RVP	sp|Q9RVP	294	58813000	27218000	1.05E+08	78735000	65139000	87786000
tr|Q9RTG7	tr|Q9RTG7	873						
sp|Q9RUE	sp|Q9RUE	229	2.81E+08	4.15E+08	3.87E+08	3.99E+08	3.99E+08	4.14E+08
sp|Q9RY7	sp|Q9RY7	423	11268000	8344000	6610600		5869100	
sp|Q9RY4	sp|Q9RY4	416	11278000	11729000	5973600			
sp|Q9RUE	sp|Q9RUE	229	1.05E+08	1.47E+08	1.39E+08	99209000	67795000	1.14E+08
sp|Q9RUE	sp|Q9RUE	229	1.79E+08	2.67E+08	5.2E+08	63665000	1.52E+08	1.27E+08
sp|Q9RW9	sp|Q9RW9	317	1.05E+08	1.25E+08	1.4E+08	25357000	30478000	29412000

sp|Q9RU8	sp|Q9RU8	218	2814700		626300			
tr|Q9RYU9	tr|Q9RYU9	1819	20637000	29596000	24434000	18446000	26717000	27922000
tr|Q9RV94	tr|Q9RV94	1197	1.28E+08	1.6E+08	1.37E+08	5.41E+08	5.77E+08	4.21E+08
sp|Q9RXT	sp|Q9RXT	401	4.2E+08	6.58E+08	6.66E+08	6.64E+08	4.63E+08	5.68E+08
sp|Q9RXT	sp|Q9RXT	401	3.74E+08	8.1E+08	7.31E+08	68952000	1.64E+08	2.43E+08
tr|Q9RT95	tr|Q9RT95	841		1.04E+08	1.04E+08		56564000	66337000
tr|Q9RSC8	tr|Q9RSC8	691		21718000			21342000	
sp|Q9RVP	sp|Q9RVP	294	4.18E+08	8.82E+08	8.02E+08	4.39E+08	4.23E+08	4.23E+08
tr|Q9RYV3	tr|Q9RYV3	1821	2.23E+08	2.13E+08	2.12E+08	1.97E+08	1.41E+08	1.68E+08
tr|Q9RXN0	tr|Q9RXN0	1619		2.34E+08	1.95E+08	4.33E+08	3.83E+08	3.8E+08
tr|Q9RT72	tr|Q9RT72	829						
sp|Q9RVW	sp|Q9RVW	301	1.89E+08	3.37E+08	2.47E+08	3.67E+08	4.91E+08	3.61E+08
tr|Q9RU17	tr|Q9RU17	976	3.24E+08	3.49E+08	4.64E+08	8.39E+08	7.49E+08	7.54E+08
tr|Q9RZL3	tr|Q9RZL3	1942	1.21E+08	1.41E+08	1.19E+08	86181000	60587000	83159000
sp|Q9RWF	sp|Q9RWF	343		26057000	8025500	36081000	37223000	29085000
sp|Q9RWF	sp|Q9RWF	343	1.74E+08		1.73E+08	1.95E+08	2.23E+08	1.2E+08
sp|Q9RY8	sp|Q9RY8	424						
sp|Q9RRT	sp|Q9RRT	98				2063300	3550400	
tr|Q9RWL	tr|Q9RWL	1436						
tr|Q9RX19	tr|Q9RX19	1514	35061000	33414000	31221000	45289000	31037000	38114000
tr|Q9RSV3	tr|Q9RSV3	770						
tr|Q9RWU	tr|Q9RWU	1481	19501000	17253000	14117000	10566000	11323000	8836800
tr|Q9RWW	tr|Q9RWW	1489	52621000	31480000	40161000			
tr|Q9RU16	tr|Q9RU16	975						
sp|Q9RSL	sp|Q9RSL	133						
tr|Q9RR86	tr|Q9RR86	477		33106000	20191000			
tr|Q9RRM	tr|Q9RRM	548						
tr|Q9RTR5	tr|Q9RTR5	924		2780900		5960300	5314000	4452100
tr|Q9RZC4	tr|Q9RZC4	1903						
sp|Q9RXB	sp|Q9RXB	365	56251000					
tr|Q9RTM	tr|Q9RTM	898					1.07E+08	18784000
sp|Q9RSR	sp|Q9RSR	144	2.41E+08	3.11E+08	2.62E+08	1E+08	1.55E+08	1.25E+08
tr|Q9RVG6	tr|Q9RVG6	1228						
tr|Q9RXV9	tr|Q9RXV9	1654						
tr|Q9RZV0	tr|Q9RZV0	1980	79536000	86710000	63559000	51731000	55629000	64285000
tr|Q9RTE1	tr|Q9RTE1	860	25076000		35919000			
tr|Q9RZJ2	tr|Q9RZJ2	1939				1.11E+08	19527000	62880000
tr|Q9RUF8	tr|Q9RUF8	1049	17310000	14867000	19458000	13839000	13721000	
tr|Q9RW7	tr|Q9RW7	1365	1.58E+08	1.29E+08	1.06E+08		1.01E+08	
tr|Q7DF71	tr|Q7DF71	464	2822700	7894200	11872000		17953000	8142600
tr|Q9RVL0	tr|Q9RVL0	1250			56694000			
tr|Q9RTY8	tr|Q9RTY8	960	27196000	39015000	69657000			24967000
tr|Q9RSC1	tr|Q9RSC1	687	26232000	26627000	51214000		15345000	
sp|Q9RU1	sp|Q9RU1	207	73479000	1.38E+08	1.18E+08	1.42E+08	1.46E+08	1.34E+08
sp|Q9RXG	sp|Q9RXG	370	5.46E+08	7.32E+08	5.07E+08	6.95E+08	6.93E+08	7.47E+08
sp|Q9RXG	sp|Q9RXG	370	52152000		1.37E+08			
tr|Q9RSV8	tr|Q9RSV8	773				17995000		
tr|Q9RRF2	tr|Q9RRF2	515						
sp|Q9RX2	sp|Q9RX2	354	2.56E+08	1.95E+08	1.5E+08	1.35E+08	1.17E+08	1.12E+08
tr|Q9RWX	tr|Q9RWX	1498	2.17E+08	70097000	1.38E+08	3.59E+08	68347000	2.71E+08
sp|P56864	sp|P56864	55						

tr|Q9RUL1	tr|Q9RUL1	1083	12316000			14073000	10246000	
sp|Q9RW6	sp|Q9RW6	313						
tr|Q9RVH0	tr|Q9RVH0	1232			5717700			
sp|Q9RUF	sp|Q9RUF	230						
tr|Q9RYG8	tr|Q9RYG8	1752		65864000		51187000	69815000	6402400
tr|Q9RWI0	tr|Q9RWI0	1413		27259000				
tr|Q9RYQ0	tr|Q9RYQ0	1788	18011000					
sp|Q9RYB	sp|Q9RYB	427				39127000	51485000	50589000
tr|Q9RXH6	tr|Q9RXH6	1597	25235000		26556000	21784000	15077000	21605000
tr|Q9RXM	tr|Q9RXM	1614				7955100		
tr|Q9RZA9	tr|Q9RZA9	1893			25013000			
sp|Q9RXJ5	sp|Q9RXJ5	380						
tr|Q9RZ07	tr|Q9RZ07	1845	21252000	25887000	13585000	29467000	22783000	21267000
tr|Q9RYQ0	tr|Q9RYQ0	1788	10012000	61847000	67126000	72023000	77615000	91978000
sp|Q9RS3	sp|Q9RS3	110	42221000	43964000	68723000		22425000	34454000
sp|P56867	sp|P56867	56	67998000	69985000	54605000	53732000	51902000	48961000
tr|Q9RZA0	tr|Q9RZA0	1887	38224000					
tr|Q9RUL9	tr|Q9RUL9	1087	22643000		12457000	20040000	12839000	23583000
tr|Q9RYD3	tr|Q9RYD3	1733	20427000	31494000	25142000	30183000	34317000	28880000
tr|Q9RVU4	tr|Q9RVU4	1301	29576000	43003000	9466300	7.17E+08	1.71E+08	5.94E+08
sp|Q9RRH	sp|Q9RRH	85	31633000	23584000	30391000	12366000	9648700	17479000
tr|Q9RXT6	tr|Q9RXT6	1641						5789600
tr|Q9RYA0	tr|Q9RYA0	1718	25976000	25939000	21033000	32989000	7919900	18144000
tr|Q9RYA0	tr|Q9RYA0	1718			10650000			
tr|Q9RUY4	tr|Q9RUY4	1140						
tr|Q9RTS5	tr|Q9RTS5	927	1.65E+08	2.63E+08	2.37E+08	1.37E+08	1.59E+08	1.46E+08
tr|Q9RTZ8	tr|Q9RTZ8	966	5205100	6299200	4137300	12088000		9080500
tr|Q9RRF2	tr|Q9RRF2	515				6917300		
tr|Q9RVK6	tr|Q9RVK6	1246	6281600			13048000		11775000
tr|Q9RZI0	tr|Q9RZI0	1934		4231900				7476000
tr|Q9RYY7	tr|Q9RYY7	1837						
tr|Q9RW4	tr|Q9RW4	1354						
tr|Q9RVG9	tr|Q9RVG9	1231	49905000	30510000	37959000			
tr|Q9RRV7	tr|Q9RRV7	592						
tr|Q9RSR2	tr|Q9RSR2	750				29669000		19609000
tr|Q9RVX1	tr|Q9RVX1	1315	1.66E+08	2.29E+08	2.94E+08			43870000
tr|Q9RVG4	tr|Q9RVG4	1227	67852000	93333000	1.42E+08	25731000	29013000	27936000
tr|Q9RY92	tr|Q9RY92	1713						
tr|Q9RS71	tr|Q9RS71	655	3.83E+08					
tr|Q9RVK1	tr|Q9RVK1	1244		4.37E+08	6.67E+08	2.38E+08	2.58E+08	2.63E+08
tr|Q9RUL1	tr|Q9RUL1	1083	1843900					
tr|Q9RS07	tr|Q9RS07	623	42254000	27728000	23395000			
tr|Q9RWJ7	tr|Q9RWJ7	1423	1.63E+08		5.81E+08	1.73E+08	2.06E+08	1.85E+08
sp|Q9RXK	sp|Q9RXK	391	1.17E+08	42985000	35449000	23892000	39785000	43327000
tr|Q9RTG9	tr|Q9RTG9	874	53578000	12351000	23230000	36687000	21055000	40534000
tr|Q9RYI7	tr|Q9RYI7	1763	1.72E+08	1.31E+08	1.31E+08	1.82E+08	1.57E+08	1.48E+08
tr|Q9RXZ9	tr|Q9RXZ9	1672	52953000					
tr|Q9RY79	tr|Q9RY79	1708						
sp|Q9RSS	sp|Q9RSS	147				19098000	15235000	
tr|Q9RS76	tr|Q9RS76	657	24994000			49945000	52306000	41538000
tr|Q9RXD2	tr|Q9RXD2	1572	30339000	48433000		25305000	28792000	

tr|Q9RXZ4	tr|Q9RXZ4	1670	1.41E+08	2.74E+08	2.55E+08	8.9E+08	8.27E+08	7.9E+08
tr|Q9RS28	tr|Q9RS28	635	46369000	48081000	36199000	1.31E+08	99532000	94797000
sp|Q9RSG	sp|Q9RSG	118						
tr|Q9RUI3	tr|Q9RUI3	1062	39983000	27267000	42935000			
sp|Q9RTJ3	sp|Q9RTJ3	189	2129100		697300			
tr|Q9RY30	tr|Q9RY30	1685	1.41E+08	1.6E+08	1.74E+08	1.97E+08	2.03E+08	2.26E+08
sp|Q9RY2	sp|Q9RY2	411	60551000	39715000	41550000	9714900	9553500	16720000
sp|Q9RY2	sp|Q9RY2	411		30336000			12451000	17379000
tr|Q9RS77	tr|Q9RS77	658						
tr|Q9RYX6	tr|Q9RYX6	1835			19764000		18739000	
sp|Q9RRJ	sp|Q9RRJ	87						
tr|Q9RYD9	tr|Q9RYD9	1735	10657000		12510000			
tr|Q9RVQ	tr|Q9RVQ	1279	49134000	57161000	54138000	60559000	82824000	51054000
tr|Q9RY79	tr|Q9RY79	1708		27145000	55316000	16956000	24041000	24909000
tr|Q9RW3	tr|Q9RW3	1348	1.69E+08	1.51E+08	1.71E+08	1.2E+08	1.23E+08	1.08E+08
sp|Q9RW7	sp|Q9RW7	314	1.41E+08	1.57E+08	1.4E+08			
sp|Q9RYM	sp|Q9RYM	438	2.5E+08	3.16E+08	3.72E+08	9.04E+08	6.4E+08	6.71E+08
tr|Q9RVV7	tr|Q9RVV7	1309		92008000				
sp|Q9RY2	sp|Q9RY2	411	3.66E+09	3.45E+09	2.95E+09	2.12E+09	2.56E+09	2.03E+09
tr|Q9RRI3	tr|Q9RRI3	531	6.57E+08	6.83E+08	5.35E+08	8.59E+08	6.51E+08	7.81E+08
tr|Q9RYH8	tr|Q9RYH8	1757	60748000	28446000	63735000	1.69E+08	2.55E+08	1.19E+08
tr|Q9RV05	tr|Q9RV05	1153				6393100	7275900	
tr|Q9RYY7	tr|Q9RYY7	1837						
tr|Q9RRP4	tr|Q9RRP4	558	1.21E+09	1.27E+09	1.21E+09	1.14E+09	1.46E+09	1.16E+09
tr|Q9RXA6	tr|Q9RXA6	1559						
tr|Q9RV30	tr|Q9RV30	1166				41369000	60858000	31203000
sp|Q9RXG	sp|Q9RXG	370				45673000		10987000
tr|Q9RRW	tr|Q9RRW	595			2.38E+08			
tr|Q9RTW	tr|Q9RTW	941	62863000	67792000	44679000	64682000	73563000	51146000
tr|Q9RTM	tr|Q9RTM	899	91509000					
sp|Q9RU2	sp|Q9RU2	209				1.3E+08	1.75E+08	89629000
sp|O3250	sp|O3250	46						
tr|Q9RTZ0	tr|Q9RTZ0	961	7.65E+08	7.15E+08	6.06E+08	5.91E+08	6.84E+08	6.86E+08
tr|Q9RUL2	tr|Q9RUL2	1084				1.07E+08	1.78E+08	1.7E+08
sp|Q9RYL	sp|Q9RYL	436						
sp|Q9RSI9	sp|Q9RSI9	122			21853000		13563000	12068000
tr|Q9RZQ	tr|Q9RZQ	1959			22772000		26414000	31648000
tr|Q9RXK9	tr|Q9RXK9	1608	64731000	60514000	79368000		39848000	
sp|Q9RU7	sp|Q9RU7	215			22275000			
sp|Q9RRJ6	sp|Q9RRJ6	89	12679000			6966200	9393600	9713800
sp|Q9RU0	sp|Q9RU0	233	2.46E+08	2.09E+08	2.16E+08	1.93E+08	1.99E+08	2.22E+08
tr|Q9RSP0	tr|Q9RSP0	743						
tr|Q9RZT2	tr|Q9RZT2	1969	2.82E+08	2.08E+08	2.21E+08			
tr|Q9RU01	tr|Q9RU01	969			1156600		871850	
tr|Q9RSJ4	tr|Q9RSJ4	729	40429000	44579000	9747600	53860000	53887000	62409000
tr|Q9RVG6	tr|Q9RVG6	1228	4.54E+08	3.9E+08	3.47E+08	7.23E+08	5.4E+08	6.17E+08
tr|Q9RVG6	tr|Q9RVG6	1228	17590000	76696000	1.84E+08	66990000	2E+08	72301000
tr|Q9RSY4	tr|Q9RSY4	783						
sp|Q9RUH	sp|Q9RUH	236						
tr|Q9RVS9	tr|Q9RVS9	1293						
sp|O3250	sp|O3250	46	10595000	11246000	14929000	17334000	16782000	19817000

tr|Q9RUY4	tr|Q9RUY4	1140			46020000		79212000	
sp|Q9RXK	sp|Q9RXK	390						
sp|O8302	sp|O8302	47						
sp|Q9RRB	sp|Q9RRB	76	6.79E+08	5.65E+08	5.92E+08	1.03E+09	6.27E+08	8.21E+08
tr|Q9RUU	tr|Q9RUU	1124		31328000	36226000			
sp|Q9RUR	sp|Q9RUR	249	48882000	68956000	95724000	71665000	91656000	90625000
sp|Q9RUR	sp|Q9RUR	249			19346000	4.77E+08	5.8E+08	4.79E+08
tr|Q9RZA7	tr|Q9RZA7	1892	83664000	54748000	65783000	62737000	65160000	93088000
tr|Q9RXF0	tr|Q9RXF0	1581	14102000		27354000	15466000		13595000
tr|Q9RUS6	tr|Q9RUS6	1113	4.91E+08	4.58E+08	3.2E+08	5.88E+08	5.2E+08	6.48E+08
sp|Q9RW(	sp|Q9RW(	327	63977000	88897000	24010000	1.01E+08	1.19E+08	92513000
tr|Q9RYQ4	tr|Q9RYQ4	1792	61459000	69115000	71309000	3.64E+08	3.38E+08	2.82E+08
sp|Q9RSL	sp|Q9RSL	135	24554000	23398000	33776000	56726000	83989000	79844000
tr|Q9RRG6	tr|Q9RRG6	522	28928000	29245000	33157000	38815000	52509000	50846000
tr|Q9RWP	tr|Q9RWP	1449	2.45E+08	1.88E+08	1.62E+08	4.06E+08	4.14E+08	3.45E+08
tr|Q9RS71	tr|Q9RS71	655	2.93E+08	3.76E+08	2.43E+08	5.44E+08	5.44E+08	5.77E+08
tr|Q9RW8	tr|Q9RW8	1366	62487000	49097000	48009000	49023000	47292000	57030000
tr|Q9RT51	tr|Q9RT51	820			6573800	27824000		
tr|Q9RXM	tr|Q9RXM	1615	1.35E+08					
tr|Q9RXY9	tr|Q9RXY9	1667			18191000		32953000	
sp|Q5933	sp|Q5933	63	73646000	1.17E+08	93948000	1.25E+08	1.17E+08	
tr|Q9RXD5	tr|Q9RXD5	1573	29836000	36998000		56389000	50305000	36364000
tr|Q9RXD5	tr|Q9RXD5	1573	26109000					
sp|Q9RXK	sp|Q9RXK	386			14962000			
sp|Q9RVE	sp|Q9RVE	284			1.02E+08		86689000	
tr|Q9RTZ3	tr|Q9RTZ3	963	38336000	29274000	35586000		35753000	
tr|Q9RY79	tr|Q9RY79	1708						
tr|Q9RTG7	tr|Q9RTG7	873						
tr|Q9RS41	tr|Q9RS41	641				29506000	20525000	22376000
tr|Q9RUK9	tr|Q9RUK9	1081		28005000				
sp|Q9RUR	sp|Q9RUR	249	2.01E+09	1.88E+09	1.49E+09	3.08E+09	2.3E+09	2.52E+09
sp|Q9RUR	sp|Q9RUR	249	9.02E+08	1.83E+09	3.66E+09	3.03E+09	3.45E+09	2.91E+09
tr|Q9RYS1	tr|Q9RYS1	1801		2758400	2928100			
tr|Q9RTN3	tr|Q9RTN3	904						
sp|Q9RV8	sp|Q9RV8	270				29135000		
tr|Q9RWP	tr|Q9RWP	1449					20841000	
tr|Q9RWP	tr|Q9RWP	1449	9215500	26618000	29376000	16855000		
tr|Q9RX17	tr|Q9RX17	1512						
sp|Q9RXC	sp|Q9RXC	367	1.32E+08	1.63E+08	1.2E+08	1.46E+08	1.41E+08	1.35E+08
tr|Q9RRV1	tr|Q9RRV1	588						
tr|Q9RVR5	tr|Q9RVR5	1284	2.8E+08	2.67E+08	2.4E+08	2.48E+08	2.31E+08	2.23E+08
tr|Q9RXI1	tr|Q9RXI1	1601	60722000	54935000	67638000			
tr|Q9RVU4	tr|Q9RVU4	1301	9119100	9240500	6907300		15809000	10666000
tr|Q9RZU0	tr|Q9RZU0	1976		21304000		13169000		12191000
tr|Q9RT01	tr|Q9RT01	792				25392000		17659000
tr|Q9RVH8	tr|Q9RVH8	1235				23863000	17707000	23014000
tr|Q9RRP4	tr|Q9RRP4	558		10188000	12804000			
sp|Q9RV5	sp|Q9RV5	265		941830	724080	4091800	6119900	7311300
sp|Q9RUP	sp|Q9RUP	245	11586000	8332300	6994100	9919400	8938500	8829200
sp|Q9RUP	sp|Q9RUP	245				2640900		2029900
sp|Q9RUP	sp|Q9RUP	245	83725000	64626000	1.1E+08	1.93E+08	1.2E+08	1.39E+08

sp|P5692	sp|P5692	59	3.58E+08	3.77E+08	2.64E+08	3.1E+08	2.57E+08	2.66E+08
tr|Q9RVA	tr|Q9RVA5	1202	32754000	10754000	141840	4430100	6677400	5292000
tr|Q9RRP	tr|Q9RRP3	557						
sp|Q9RYM	sp|Q9RYM	439	7.64E+08	5.29E+08	5.52E+08	1.04E+09	1.29E+09	1.06E+09
tr|Q9RSA	tr|Q9RSA3	674						
tr|Q9RUI4	tr|Q9RUI4	1063	73021000	65723000	58680000	44451000	43886000	45829000
sp|Q9RV5	sp|Q9RV5	265		9950900	6306300	16445000	21120000	21352000
tr|Q9RXZ	tr|Q9RXZ3	1669	1.49E+08	36184000	1.25E+08	2.73E+08	2.11E+08	2.5E+08
tr|Q9RUR	tr|Q9RUR	1109	44216000	19418000	13765000	8952900	12781000	13435000
tr|Q9RWE	tr|Q9RWE	1398						
tr|Q9RRG	tr|Q9RRG3	519	76656000	62495000	1.03E+08	97126000	84713000	83683000
tr|Q9RRB	tr|Q9RRB1	492						
tr|Q9RYF4	tr|Q9RYF4	1743				1.03E+08	58840000	1.5E+08
tr|Q9RX8	tr|Q9RX84	1549	24069000	21906000	20882000	21707000	39322000	
tr|Q9RRE	tr|Q9RRE	508	76334000	97594000	90158000	2.08E+08	1.56E+08	1.63E+08
tr|Q9RRE	tr|Q9RRE	508			27301000			
tr|Q9RU2	tr|Q9RU2	977	1.54E+08	1.26E+08	1.26E+08			
tr|Q9RU0	tr|Q9RU0	969						
tr|Q9RUA	tr|Q9RUA	1026	5345600	1648900	1321400	1129800	1709600	1597100
tr|Q9RTC	tr|Q9RTC7	853			10743000		9527300	
tr|Q9RTZ	tr|Q9RTZ7	965						
tr|Q9RXF	tr|Q9RXF3	1583			10361000			
tr|Q9RTX	tr|Q9RTX6	951	63969000	71351000	51495000	69116000	1.26E+08	30109000
tr|Q9RZH	tr|Q9RZH	1931	47934000	33022000	37561000	43650000	46103000	51245000
tr|Q9RY3	tr|Q9RY3	1692	78662000	94053000	94774000	1.45E+08	2.39E+08	1.24E+08
tr|Q9RR9	tr|Q9RR9	481		42423000				
tr|Q9RS0	tr|Q9RS0	624	2332100			17657000	26665000	23040000
tr|Q9RYG	tr|Q9RYG	1747						
tr|Q9RUT	tr|Q9RUT	1120	65228000	37896000	37672000	45256000	38332000	31267000
tr|Q9RVW	tr|Q9RVW	1312	1.63E+08	1.32E+08	1.14E+08	1.57E+08	1.22E+08	1.41E+08
tr|Q9RS1	tr|Q9RS13	628				31812000		
sp|Q9RRB	sp|Q9RRB	77					512870	
sp|Q9RRB	sp|Q9RRB	77			22105000			
tr|Q9RTH	tr|Q9RTH	877	1.68E+09	1.39E+09	1.45E+09	2.25E+08	3.59E+08	3.64E+08
sp|Q9RW	sp|Q9RW	327	4.17E+09	3.94E+09	3.71E+09	1.41E+09	1.59E+09	1.51E+09
sp|Q9RU	sp|Q9RU	254	11411000	14723000	17789000	19701000	10745000	17410000
tr|Q9RRW	tr|Q9RRW	595					42767000	
tr|Q9RYH	tr|Q9RYH	1757				1.13E+08	1.37E+08	1.18E+08
sp|Q9RUF	sp|Q9RUF	231	40511000		63138000	93282000	39546000	80646000
tr|Q9RWV	tr|Q9RWV	1487		49183000		58317000	59356000	76639000
tr|Q9RW5	tr|Q9RW5	1357	27220000		26154000	72859000	40122000	53944000
sp|Q9RW	sp|Q9RW	342	61269000		7327700	34746000		1.05E+08
sp|Q9RW	sp|Q9RW	342			33051000	1.18E+08	1.36E+08	1.02E+08
tr|Q9RWP	tr|Q9RWP	1449						
sp|Q9RRC	sp|Q9RRC	79			27390000			
tr|Q9RSL6	tr|Q9RSL6	732	1.48E+08	1.02E+08	1.02E+08	1.16E+08	96831000	1.56E+08
tr|Q9RS6	tr|Q9RS66	653	41208000	53900000	83431000		42021000	28124000
tr|Q9RXG	tr|Q9RXG	1587	3864400	35232000	20894000	22901000	40425000	19342000
sp|Q9RUA	sp|Q9RUA	224						
tr|Q9RXX	tr|Q9RXX2	1660	4.72E+08	5.59E+08	4.84E+08	4.87E+08	3.47E+08	4.44E+08
sp|Q9RXK	sp|Q9RXK	389	2.03E+08	1.77E+08	1.71E+08	3.6E+08	3.9E+08	4.03E+08

tr|Q9RX81	tr|Q9RX81	1547	18601000	13303000	15780000	12416000	14322000	16474000
tr|Q9RV78	tr|Q9RV78	1190				29437000		
tr|Q9RVU5	tr|Q9RVU5	1302						
tr|Q9RY34	tr|Q9RY34	1687	9.48E+08	7.99E+08	8.51E+08	8.6E+08	7.71E+08	7.55E+08
sp|Q9RSP	sp|Q9RSP	139	18268000	21669000	33001000	87392000	45460000	64466000
tr|Q9RUB	tr|Q9RUB	1028	29151000	36899000	35079000	45932000	67884000	
tr|Q9RUB	tr|Q9RUB	1028	70626000	1.54E+08	3.04E+08	2.58E+08	2.21E+08	1.69E+08
tr|Q9RU57	tr|Q9RU57	1000		24089000	18298000	68189000	59312000	75880000
tr|Q9RSE7	tr|Q9RSE7	705	1.22E+08	1.11E+08	1.18E+08	1E+08	67246000	90973000
tr|Q9RX07	tr|Q9RX07	1506	39322000	35709000	36912000			
tr|Q9RT71	tr|Q9RT71	828	32025000	34023000	30572000	30200000	35660000	24426000
tr|Q9RT45	tr|Q9RT45	816						
sp|P56867	sp|P56867	56	4.73E+08	5.39E+08	9.32E+08	4.88E+08	4.92E+08	3.54E+08
tr|Q9RV70	tr|Q9RV70	1186						
tr|Q9RZB1	tr|Q9RZB1	1894	18016000		14615000			
tr|Q9RVP5	tr|Q9RVP5	1269			12689000	32842000	47102000	34683000
sp|Q9RRB	sp|Q9RRB	77						
tr|Q9RTA8	tr|Q9RTA8	846				39766000	55685000	41243000
sp|Q9RUE	sp|Q9RUE	226			2457100	32421000	80678000	82428000
tr|Q9RU45	tr|Q9RU45	992						
sp|Q9RRL	sp|Q9RRL	90	6.89E+08	6.26E+08	5.9E+08	9.52E+08	9.24E+08	9.31E+08
sp|Q9RRL	sp|Q9RRL	90	1.36E+08	1.89E+08	97855000			45974000
tr|Q9RSY7	tr|Q9RSY7	785		39748000	74974000	59992000	80737000	87394000
tr|Q9RRP4	tr|Q9RRP4	558	40321000	51597000		76116000		
tr|Q9RY75	tr|Q9RY75	1705					19158000	21132000
tr|Q9RY75	tr|Q9RY75	1705	21658000	12651000	26564000			17091000
tr|Q9RUN	tr|Q9RUN	1094		50470000			67198000	47817000
tr|Q9RUA	tr|Q9RUA	1023				23257000	28679000	29434000
tr|Q9RY75	tr|Q9RY75	1705						
sp|Q9RS2	sp|Q9RS2	108					2386700	
tr|Q9RTJ1	tr|Q9RTJ1	883	11659000	10210000	11401000	23105000	16325000	20002000
tr|Q9RVC6	tr|Q9RVC6	1212						
tr|Q9RZ13	tr|Q9RZ13	1850	12552000	6751000	10374000	7380200		8359900
sp|Q9RY5	sp|Q9RY5	417	3.12E+09	2.25E+09	2.41E+09	2.35E+09	2.47E+09	2.38E+09
sp|Q9RY5	sp|Q9RY5	417		18849000	21569000			
tr|Q9RZQ	tr|Q9RZQ	1958	2.81E+08	2.44E+08	2.87E+08	1.87E+08	1.45E+08	1.3E+08
sp|Q9RXC	sp|Q9RXC	367			24233000			
tr|Q9RSV7	tr|Q9RSV7	772						
tr|Q9RW3	tr|Q9RW3	1348	14133000	19997000		5122200	46743000	48216000
tr|Q9RY75	tr|Q9RY75	1705				25664000	24177000	16707000
tr|Q9RY75	tr|Q9RY75	1705			23355000			21150000
tr|Q9RSY7	tr|Q9RSY7	785	5225700	1364700	1460100			1752800
tr|Q9RS52	tr|Q9RS52	646			3276300		5019200	5207100
tr|Q9RZF3	tr|Q9RZF3	1921						
tr|Q9RTI3	tr|Q9RTI3	879	6.26E+08	5.02E+08	4.73E+08	3.65E+08	5.62E+08	3.62E+08
tr|Q9RZA7	tr|Q9RZA7	1892	1.09E+08	88407000	83023000	2.69E+08	2.02E+08	2.02E+08
sp|Q9RSP	sp|Q9RSP	139	1.75E+08	1.7E+08	1.22E+08	2.55E+08	2.75E+08	2.09E+08
tr|Q9RW6	tr|Q9RW6	1359	24555000	28075000	35689000	43202000	57424000	23244000
tr|Q9RW6	tr|Q9RW6	1359	3.22E+08	4.51E+08	5.5E+08	1.26E+08	2.58E+08	2.63E+08
tr|Q9RUZ7	tr|Q9RUZ7	1148						
tr|Q9RY75	tr|Q9RY75	1705	36339000			76095000		

tr|Q9RV5	tr|Q9RV55	1179				73276000	94596000	1.01E+08
tr|Q9RTN	tr|Q9RTN8	905	1.72E+08	1.43E+08	1.3E+08	1.54E+08	1.61E+08	1.56E+08
tr|Q9RX6	tr|Q9RX64	1537	65351000	54068000	68165000	48300000	56591000	55542000
tr|Q9RTX	tr|Q9RTX0	947	2.38E+08	1.95E+08	1.71E+08	79358000	80107000	83024000
tr|Q9RXE	tr|Q9RXE8	1579	8345400		21600000		8465400	24503000
tr|Q9RXX	tr|Q9RXX2	1660						
tr|Q9RZK	tr|Q9RZK3	1941			14232000			
tr|Q9RRC	tr|Q9RRC0	497	7321500		12369000	17049000		
tr|Q9RY0	tr|Q9RY02	1675	1.6E+09	1.79E+09	1.78E+09	2.38E+09	2.5E+09	2.25E+09
sp|Q9RXK	sp|Q9RXK	386	7028400	7533600	8700400			
tr|Q9RTI3	tr|Q9RTI3	879	55890000	1.21E+08	89546000	95128000	1.42E+08	1.02E+08
tr|Q9RWV	tr|Q9RWV	1482	24855000	25653000	25965000	68375000	49663000	51303000
tr|Q9RY0	tr|Q9RY02	1675	5.85E+08	3.53E+08	6.29E+08	87521000	1.07E+08	1.5E+08
tr|Q9RZ2	tr|Q9RZ22	1854						
tr|Q9RT7	tr|Q9RT75	831	5.45E+08	5.24E+08	4.6E+08	7.79E+08	7.13E+08	8.05E+08
tr|Q9RZ7	tr|Q9RZ75	1869						
sp|Q9RW	sp|Q9RW	347	81370000	87217000	98537000	45583000	57979000	52105000
tr|Q9RWA	tr|Q9RWA	1381	1.24E+08	66970000	87581000	42104000		44182000
tr|Q9RWE	tr|Q9RWE	1399			46556000			
tr|Q9RRA	tr|Q9RRA2	485	72527000	72678000	1.11E+08	1.59E+08	1.06E+08	1.81E+08
sp|Q9RU	sp|Q9RUN	242						
sp|Q9RW	sp|Q9RWF	344				15104000		
sp|Q9RW	sp|Q9RWF	344				6020400	7203400	
tr|Q9RSV	tr|Q9RSV7	772			4020000			
sp|Q9RW	sp|Q9RWE	321	90208000	1.06E+08	2.25E+08	1.68E+08	4.22E+08	4.45E+08
sp|Q9RVI	sp|Q9RVI3	289	12789000	23217000	42214000	10310000	17753000	17432000
sp|Q9RRE	sp|Q9RRE	83					48257000	52871000
tr|Q9RWV	tr|Q9RWV	1485	55759000		26388000	29670000	36997000	45979000
tr|Q9RX6	tr|Q9RX64	1537	1.1E+08	86497000	1.06E+08	97676000	99153000	1.05E+08
tr|Q9RUR	tr|Q9RUR4	1107	2057400	1140200	800830	765850	1071400	936640
tr|Q9RYG	tr|Q9RYG5	1750						
tr|Q9RTP	tr|Q9RTP6	910			12185000	7970900		6049200
tr|Q9RX6	tr|Q9RX63	1536	41128000	51701000	33273000	63089000	46634000	51599000
tr|Q9RR7	tr|Q9RR73	471	23492000	15981000	9943900		10937000	11898000
tr|Q9RVS	tr|Q9RVS2	1288	1.09E+08	1.56E+08	1.39E+08	5.07E+08	4.3E+08	3.32E+08
sp|Q9RX6	sp|Q9RX6	361	3.52E+08	3.65E+08	2.92E+08	4.12E+08	3.54E+08	3.86E+08
sp|O3250	sp|O3250	44	4374300		2106300	1423200		
tr|Q9RU4	tr|Q9RU42	989				6138300	8727400	7248200
tr|Q9RT51	tr|Q9RT51	820	37101000	26643000	36718000	98837000	91940000	77479000
tr|Q9RXS	tr|Q9RXS7	1640	3.45E+08	3.4E+08	4.63E+08	2.4E+08	2.73E+08	2.4E+08
tr|Q9RSH	tr|Q9RSH0	716	33365000					
tr|Q9RZH	tr|Q9RZH8	1932						
tr|Q9RWJ1	tr|Q9RWJ1	1420	59229000	36241000	55115000	1.76E+08	1.07E+08	2.31E+08
sp|Q9RW	sp|Q9RW	342	6.65E+08	4.97E+08	4.25E+08	3.55E+08	3.23E+08	3.47E+08
sp|Q9RSL	sp|Q9RSL	135	2.27E+08	1.68E+08	1.68E+08	2.54E+08	2.29E+08	2.34E+08
tr|Q9RWI	tr|Q9RWI6	1417	28151000	35608000	40525000	8277700	11529000	16552000
tr|Q9RWF	tr|Q9RWF	1402				55463000		
sp|Q9RW	sp|Q9RW4	309	41428000	45451000	30892000	41557000		32159000
tr|Q9RYQ	tr|Q9RYQ	1789						99190000
tr|Q9RX8	tr|Q9RX85	1550						
sp|Q9RV8	sp|Q9RV8	270	31650000	59514000	59110000			20904000

tr|Q9RYG3	tr|Q9RYG3	1748						
tr|Q9RYG1	tr|Q9RYG1	1747						
tr|Q9RYG3	tr|Q9RYG3	1748;1749	23920000	18866000				15920000
tr|Q9RUI9	tr|Q9RUI9	1066	24843000		29486000			
sp|Q9RXJ9	sp|Q9RXJ9	384	1.35E+09	1.71E+09	1.5E+09	5.64E+08	7.65E+08	6.28E+08
tr|Q9RSA2	tr|Q9RSA2	673	85437000	79854000				
tr|Q9RRA0	tr|Q9RRA0	483		13194000	11539000			
tr|Q9RVS6	tr|Q9RVS6	1291						
sp|Q9RXK	sp|Q9RXK	386			19147000	24636000	26254000	31830000
tr|Q9RV15	tr|Q9RV15	1159		17975000	16639000	34841000	41985000	34248000
tr|Q9RXI3	tr|Q9RXI3	1602		46775000		92092000	65823000	69516000
tr|Q9RYT5	tr|Q9RYT5	1814		24304000	22796000	13510000		
tr|Q9RRA6	tr|Q9RRA6	489	7.63E+08	5.29E+08	6.86E+08	8.33E+08	1.09E+09	9.91E+08
tr|Q9RW4	tr|Q9RW4	1353	24879000	29553000	26392000			
tr|Q9RWV	tr|Q9RWV	1482			21538000			
tr|Q9RRC9	tr|Q9RRC9	502	1.57E+08	1.94E+08	1.39E+08	1.14E+08	89821000	1.14E+08
tr|Q9RX58	tr|Q9RX58	1533	41902000	45368000	25130000	55463000	35865000	35210000
tr|Q9RUR	tr|Q9RUR	1109	27104000			1.58E+08	1.3E+08	1.22E+08
tr|Q9RUR	tr|Q9RUR	1109	34854000	40466000	60701000	34969000	45051000	78126000
sp|Q9RSJ6	sp|Q9RSJ6	124			23573000	91547000	1.06E+08	90312000
sp|Q9RSJ6	sp|Q9RSJ6	124						
tr|Q9RUQ	tr|Q9RUQ	1103						
tr|Q9RUX8	tr|Q9RUX8	1136	25180000	61939000	46902000	2.24E+08	3.01E+08	1.47E+08
sp|Q9RSL	sp|Q9RSL	135	1.97E+08	1.93E+08	1.82E+08	4.62E+08	4.56E+08	3.38E+08
tr|Q9RZK3	tr|Q9RZK3	1941						
tr|Q9RUI9	tr|Q9RUI9	1066				22454000	20625000	22530000
sp|Q9RTT	sp|Q9RTT	200						
tr|Q9RXT6	tr|Q9RXT6	1641		30762000	31017000			
sp|Q9RY2	sp|Q9RY2	411	14225000	8900400	5648700	16418000	20338000	13995000
tr|Q9RXX2	tr|Q9RXX2	1660	31679000					
tr|Q9RXF0	tr|Q9RXF0	1581				36335000	38758000	36137000
tr|Q9RXF0	tr|Q9RXF0	1581				65056000	57040000	54715000
tr|Q9RRQ	tr|Q9RRQ	566						
tr|Q9RWD	tr|Q9RWD	1395	61787000	56838000	69443000	3714600	9896500	6498800
tr|Q9RWN	tr|Q9RWN	1443	46520000	40510000	27492000	25515000	24775000	
tr|Q9RS71	tr|Q9RS71	655				9032100	13053000	13271000
tr|Q9RUM	tr|Q9RUM	1091	1.11E+08	1.07E+08	1.45E+08	1.72E+08	69495000	70564000
tr|Q9RU25	tr|Q9RU25	978						
tr|Q9RVQ	tr|Q9RVQ	1271	80437000	1.04E+08	1.3E+08	79120000	85710000	1.01E+08
tr|Q9RSM	tr|Q9RSM	735						
sp|Q9RSL4	sp|Q9RSL4	136	3.31E+08	3.84E+08	3.29E+08	3.06E+08	4.37E+08	2.83E+08
sp|Q9RS6	sp|Q9RS6	112	72054000	42146000		1.78E+08	95824000	79811000
tr|Q9RUY7	tr|Q9RUY7	1142						20328000
tr|Q9RW7	tr|Q9RW7	1364	17358000	9595100	8050100	24241000	25255000	21504000
tr|Q9RW7	tr|Q9RW7	1364	72731000	1.03E+08	91122000	25743000	42539000	59678000
tr|Q9RVG2	tr|Q9RVG2	1226	4851900	1636900	1603900	2341800	1956000	1871600
tr|Q9RVG2	tr|Q9RVG2	1226		2111800	2267200		1199300	912720
tr|Q9RRU1	tr|Q9RRU1	584	23759000		17329000	2.9E+08	2.71E+08	3.87E+08
sp|Q9RTU	sp|Q9RTU	201		4018800				
tr|Q9RVE9	tr|Q9RVE9	1220			16451000			
tr|Q9RSE4	tr|Q9RSE4	703	3.35E+08	4.98E+08	4.67E+08		2.33E+08	

tr|Q9RRE6	tr|Q9RRE6	512				14071000		13217000
tr|Q9RYN3	tr|Q9RYN3	1777	35894000	39662000	69037000			34672000
tr|Q9RVA2	tr|Q9RVA2	1199	41255000		44789000	33491000	40274000	41552000
tr|Q9RRR0	tr|Q9RRR0	567				56901000	57802000	1.29E+08
sp|Q9RX6	sp|Q9RX6	361	1.23E+08	1.9E+08	1.44E+08	2.61E+08	2.05E+08	21837000
tr|Q9RS87	tr|Q9RS87	663	1.27E+08	53263000	47339000	78642000		73520000
sp|Q9RXH	sp|Q9RXH	372	1.9E+08	2.44E+08	3E+08	4.61E+08	3.04E+08	3.59E+08
tr|Q9RWQ	tr|Q9RWQ	1453	5941900	5732700	11610000	15998000	34959000	21505000
tr|Q9RZB3	tr|Q9RZB3	1896				26580000	31418000	22450000
tr|Q9RZF3	tr|Q9RZF3	1921						
tr|Q9RT68	tr|Q9RT68	826	12944000	4927400	4035900	4357900	5407500	5022000
tr|Q9RWI1	tr|Q9RWI1	1414						
tr|Q9RWI8	tr|Q9RWI8	1419	32393000		30529000			36785000
sp|Q9RV5	sp|Q9RV5	265	8686000	3623100	2611600	5708200	4400000	497460
tr|Q9RVT5	tr|Q9RVT5	1296		38254000	63417000			
tr|Q9RX36	tr|Q9RX36	1520			20239000	25361000		
sp|Q9RZ8	sp|Q9RZ8	453	21434000		1.25E+08	21036000		85189000
tr|Q9RZH5	tr|Q9RZH5	1930	29824000	25358000	21524000	28793000	21389000	26599000
tr|Q9RZ14	tr|Q9RZ14	1851	72589000	71077000	49650000	64984000	73144000	74710000
tr|Q9RSW	tr|Q9RSW	775						35041000
tr|Q9RUM	tr|Q9RUM	1090	36372000		32573000		21076000	
tr|Q9RTK6	tr|Q9RTK6	890				495740	556600	552510
tr|Q9RTK6	tr|Q9RTK6	890	21103000	26106000	15240000	12768000	18780000	18862000
sp|Q9RVY	sp|Q9RVY	303	2.61E+08	2.35E+08	2.1E+08	63156000	1.1E+08	1.44E+08
tr|Q9RZD7	tr|Q9RZD7	1911						
tr|Q9RXD5	tr|Q9RXD5	1573	23674000	65444000	37496000	1.94E+08	1.36E+08	80536000
tr|Q9RXD5	tr|Q9RXD5	1573				44549000	18670000	31857000
tr|Q9RYN3	tr|Q9RYN3	1777	73742000	87104000	75830000	2.55E+08	5.91E+08	6.49E+08
tr|Q9RXI1	tr|Q9RXI1	1601					11103000	
tr|Q9RW2	tr|Q9RW2	1344	46475000	33665000	40072000			
tr|Q9RXI5	tr|Q9RXI5	1604	1.05E+08	1.15E+08	78135000		94686000	80325000
tr|Q9RRK9	tr|Q9RRK9	543						
tr|Q9RYG1	tr|Q9RYG1	1747	58729000	20936000			37711000	
tr|Q9RTX7	tr|Q9RTX7	952	4305700		5342500			
tr|Q9RV06	tr|Q9RV06	1154						
tr|Q9RRZ6	tr|Q9RRZ6	615						
tr|Q9RZG9	tr|Q9RZG9	1926			4042700			
sp|Q9RXR	sp|Q9RXR	396						
tr|Q9RXL4	tr|Q9RXL4	1610			22340000		18587000	20418000
tr|Q9RSQ4	tr|Q9RSQ4	748	3.92E+09	4.87E+09	7.09E+09	2.09E+08	4.22E+08	2.72E+08
tr|Q9RSY6	tr|Q9RSY6	784			70456000			
tr|Q9RTD6	tr|Q9RTD6	859	59803000	45329000	49167000	29350000	26195000	31315000
tr|Q9RXQ	tr|Q9RXQ	1627	6.88E+08	7.67E+08	6.27E+08	1.32E+09	1.28E+09	1E+09
tr|Q9RXQ	tr|Q9RXQ	1627	1.11E+08	76210000	1.07E+08		48637000	59391000
tr|Q9RT49	tr|Q9RT49	818						
sp|Q9ZNA	sp|Q9ZNA	463	9372800	14457000	21706000	32000000	23219000	31166000
tr|Q9RZ87	tr|Q9RZ87	1879				10865000	12985000	12080000
tr|Q9RTW	tr|Q9RTW	946						
sp|Q9RT4	sp|Q9RT4	167	33991000	27932000	27031000			
tr|Q9RWL	tr|Q9RWL	1437	74138000	68220000	74209000			
tr|Q9RW1	tr|Q9RW1	1337	22617000	16167000	15560000	18766000	17611000	17261000

sp|Q9RY7	sp|Q9RY7	423	1.91E+08	2.18E+08	2.24E+08	3.05E+08	3.21E+08	2.99E+08
tr|Q9RUL2	tr|Q9RUL2	1084		11332000	12372000			
sp|Q9RUZ	sp|Q9RUZ	260						
tr|Q9RVK8	tr|Q9RVK8	1248	2.6E+08	3.15E+08	2.2E+08	3.46E+08	3.5E+08	3.42E+08
tr|Q9RWU	tr|Q9RWU	1479	66807000	53401000	74785000		22846000	15957000
tr|Q9RTV1	tr|Q9RTV1	936	1.99E+08	2.93E+08	4.68E+08	7.08E+08	8.67E+08	4.18E+08
tr|Q9RTN3	tr|Q9RTN3	904				18145000	21336000	11369000
tr|Q9RT28	tr|Q9RT28	805	6237100					
tr|Q9RUG	tr|Q9RUG	1055		36767000		83313000		
tr|Q9RXD5	tr|Q9RXD5	1573	54956000		35737000			
tr|Q9RZT4	tr|Q9RZT4	1971						
tr|Q9RVS0	tr|Q9RVS0	1286	4.79E+08	5.55E+08	5.21E+08	3.19E+08	3.95E+08	3.11E+08
tr|Q9RVS0	tr|Q9RVS0	1286			21357000			
sp|Q9RYD	sp|Q9RYD	431					21156000	
tr|Q9RT75	tr|Q9RT75	831	70031000	86919000	1.18E+08	67980000	65605000	
tr|Q9RRI3	tr|Q9RRI3	531	1.53E+08	1.17E+08	1.29E+08	91506000	59706000	
tr|Q9RWU	tr|Q9RWU	1480		1.47E+08				
tr|Q9RUJ4	tr|Q9RUJ4	1070	2.18E+08	2.76E+08	1.53E+08	77747000	79614000	1.2E+08
tr|Q9RT09	tr|Q9RT09	798	35580000	53024000	91736000			
tr|Q9RTC9	tr|Q9RTC9	855				1.06E+08	91039000	83063000
tr|Q9RTC9	tr|Q9RTC9	855		35504000	53002000			
sp|Q9R34	sp|Q9R34	64						
sp|Q9RY4	sp|Q9RY4	416						
tr|Q9RSV7	tr|Q9RSV7	772	75483000	1.72E+08	1.41E+08	1.56E+08	1.46E+08	1.18E+08
tr|Q9RS70	tr|Q9RS70	654						
tr|Q9RSK2	tr|Q9RSK2	730			16837000	29062000	27707000	
sp|Q9RXF	sp|Q9RXF	369	46005000	2.4E+08	14286000	2.36E+08	2.58E+08	2.41E+08
tr|Q9RV75	tr|Q9RV75	1189	2.5E+08	2.2E+08	2.62E+08	96433000	1.11E+08	1.3E+08
tr|Q9RSD0	tr|Q9RSD0	692		18423000				
tr|Q9RYQ2	tr|Q9RYQ2	1790				10850000	6543900	7077700
tr|Q9RXQ	tr|Q9RXQ	1627						
tr|Q9RUA	tr|Q9RUA	1025						
tr|Q9RWI0	tr|Q9RWI0	1413						
tr|Q7DF92	tr|Q7DF92	465		6674100				
sp|Q9RUN	sp|Q9RUN	241	9280200	15045000	26602000	83258000	98793000	34684000
tr|Q9RZE6	tr|Q9RZE6	1916	1406500				1605000	
tr|Q9RXW	tr|Q9RXW	1657						
tr|Q9RS31	tr|Q9RS31	637		19615000	26657000	54587000		
tr|Q9RWC	tr|Q9RWC	1393						
sp|Q9RRQ	sp|Q9RRQ	95			16937000			
sp|Q9RW0	sp|Q9RW0	304					1.02E+08	79693000
tr|Q9RZ22	tr|Q9RZ22	1854	1.84E+08	1.18E+08	2.32E+08	60818000	47054000	73670000
tr|Q9RYM	tr|Q9RYM	1773	26132000	23131000	35612000			
tr|Q9RWJ7	tr|Q9RWJ7	1423	13227000		12956000			
sp|Q9RW6	sp|Q9RW6	312						
sp|Q9RTR	sp|Q9RTR	198						
tr|Q9RWX	tr|Q9RWX	1499		20248000	15572000			
tr|Q9RR62	tr|Q9RR62	467	1.88E+08	2.87E+08	2.41E+08	3.07E+08	3.86E+08	3.09E+08
tr|Q9RUR	tr|Q9RUR	1109	7.29E+08	7.12E+08	6.52E+08	7.64E+08	5.66E+08	5.72E+08
tr|Q9RUR	tr|Q9RUR	1109		28736000	68040000	76451000	71651000	78992000
tr|Q9RVK7	tr|Q9RWT	1467;1247	93411000	70961000	62939000	66090000	47598000	35915000

tr|Q9RRT7	tr|Q9RRT7	581		14350000			20379000	
tr|Q9RT96	tr|Q9RT96	842			2944900	13189000	9036000	9268300
tr|Q9RT68	tr|Q9RT68	826			44084000	25986000	35668000	32041000
tr|Q9RYY7	tr|Q9RYY7	1837						
tr|Q9RY79	tr|Q9RY79	1708			31199000	20460000		
tr|Q9RWA	tr|Q9RWA	1381	39191000	37322000	46271000		23997000	
sp|P56926	sp|P56926	59	39402000	36441000	41837000	18011000	19838000	23596000
tr|Q9RXW	tr|Q9RXW	1658	45673000	75859000	1.15E+08	88191000	1.17E+08	86199000
tr|Q9RS85	tr|Q9RS85	661	1.07E+08	46373000	59461000	1.39E+08	1.21E+08	1.39E+08
sp|Q9RW9	sp|Q9RW9	345	42822000	51405000	49019000	20361000	18799000	
tr|Q9RRW	tr|Q9RRW	595						
tr|Q9RT26	tr|Q9RT26	804	5.72E+08	5.27E+08	5.19E+08	5.39E+08	4.22E+08	4.99E+08
sp|Q9RSS8	sp|Q9RSS8	149	97047000		89484000	2.52E+08	2.02E+08	2.58E+08
tr|Q9RRS0	tr|Q9RRS0	572	2.39E+08	1.79E+08	1.88E+08			
tr|Q9RTZ9	tr|Q9RTZ9	967	13294000	10806000	8618400	31348000	29277000	25644000
tr|Q9RWA	tr|Q9RWA	1381		58699000	89810000	18262000	44128000	44367000
sp|Q9RRB	sp|Q9RRB	76	49426000	51650000	49168000			
sp|Q9RRB	sp|Q9RRB	76	1.34E+08	1.86E+08	2.36E+08	82580000	82780000	48215000
tr|Q9RVS8	tr|Q9RVS8	1292				1.34E+08	1.05E+08	1.08E+08
tr|Q9RUA	tr|Q9RUA	1026					52290000	38269000
sp|Q9RRJ	sp|Q9RRJ	87	30171000			23426000		
sp|Q9RUR	sp|Q9RUR	249					2328600	
tr|Q9RXQ	tr|Q9RXQ	1627						
sp|Q9RXJ5	sp|Q9RXJ5	380				269480		564880
sp|Q9RW0	sp|Q9RW0	326				2.29E+08	2.07E+08	2.15E+08
tr|Q9RXQ	tr|Q9RXQ	1627		21811000				1.01E+08
sp|Q9RV1	sp|Q9RV1	261						
tr|Q9RYL1	tr|Q9RYL1	1768					66235000	
sp|Q9RYR	sp|Q9RYR	441	50635000	46532000	65893000			
sp|P8358	sp|P8358	60						
sp|Q9RY5	sp|Q9RY5	417				25682000	34100000	34213000
tr|Q9RTZ9	tr|Q9RTZ9	967		7741300	26630000	23450000	73464000	18854000
tr|Q9RWT	tr|Q9RWT	1466	9518400		8222800			
sp|Q9RSK	sp|Q9RSK	131				1.38E+08	1.9E+08	1.32E+08
tr|Q9RVP1	tr|Q9RVP1	1267						
sp|Q9RUR	sp|Q9RUR	249					5330200	
sp|Q9RX8	sp|Q9RX8	362				1.52E+08	1.05E+08	1.06E+08
sp|P56867	sp|P56867	56						
tr|Q9RST7	tr|Q9RST7	760				20858000	29142000	29304000
tr|Q9RRD9	tr|Q9RRD9	507		47375000	51231000		18561000	
tr|Q9RZ28	tr|Q9RZ28	1858						
tr|Q9RU27	tr|Q9RU27	980						
tr|Q9RR83	tr|Q9RR83	475						
tr|Q9RW8	tr|Q9RW8	1368					59618000	57216000
tr|Q9RUU	tr|Q9RUU	1121		7676400	24489000	34199000	32542000	29673000
tr|Q9RTM	tr|Q9RTM	899	13544000		17569000	23739000	21096000	27670000
tr|Q9RZ93	tr|Q9RZ93	1883	31596000	21877000	23064000			12947000
tr|Q9RTM	tr|Q9RTM	900	31067000	48790000	1.19E+08	73463000	70764000	50621000
sp|Q9RY2	sp|Q9RY2	411						
tr|Q9RS89	tr|Q9RS89	665				6547800	10251000	5585600
sp|Q9RXJ9	sp|Q9RXJ9	384					55494000	

tr|Q9RSA2	tr|Q9RSA2	673					9049800	
tr|Q9RWN	tr|Q9RWN	1443					1592700	
tr|Q9RVG6	tr|Q9RVG6	1228			12867000	52800000	50657000	36939000
sp|Q9RXI7	sp|Q9RXI7	373			28222000	34948000	46012000	53529000
sp|Q9RS1	sp|Q9RS1	106					16364000	
sp|Q9RXK	sp|Q9RXK	388	61784000	68550000	1.3E+08	2.18E+08	2.16E+08	2.23E+08
tr|Q9RS17	tr|Q9RS17	630				7308100	11448000	10341000
tr|Q9RTB3	tr|Q9RTB3	847			38904000			
tr|Q9RYQ0	tr|Q9RYQ0	1788				25926000	31073000	
tr|Q9RUI9	tr|Q9RUI9	1066			26149000			
tr|Q9RWW	tr|Q9RWW	1491			31406000		38330000	38337000
sp|Q9RXK	sp|Q9RXK	390						
tr|Q9RUY4	tr|Q9RUY4	1140						
sp|Q9RWU	sp|Q9RWU	347						
sp|Q9RVV	sp|Q9RVV	300			5737900	13773000	20987000	19927000
tr|Q9RRQ	tr|Q9RRQ	564						
sp|Q9RTI6	sp|Q9RTI6	188	21443000	9369300	20290000			5577600
sp|Q9RSQ	sp|Q9RSQ	141	2.01E+08	2.02E+08	1.86E+08	1.88E+08		2.06E+08
sp|Q9RWH	sp|Q9RWH	329	1.1E+09	9.94E+08	9.79E+08	4.42E+08	3.78E+08	4.27E+08
tr|Q9RUL9	tr|Q9RUL9	1087	8.04E+08	8.14E+08	7E+08	6.27E+08	5.85E+08	5.89E+08
tr|Q9RRZ6	tr|Q9RRZ6	615						
tr|Q9RVG6	tr|Q9RVG6	1228	2.32E+09	2.24E+09	2.41E+09	3.63E+09	3.83E+09	2.43E+09
tr|Q9RVG6	tr|Q9RVG6	1228	1.32E+08	2.2E+08	6.04E+08	56615000	1.53E+08	1.31E+08
tr|Q9RT53	tr|Q9RT53	821					14429000	
tr|Q9RS93	tr|Q9RS93	668		44080000	20244000	44249000	35134000	48166000
sp|Q9RT6	sp|Q9RT6	170						
sp|Q9RT6	sp|Q9RT6	170						
tr|Q9RTJ1	tr|Q9RTJ1	883	12585000	61256000	7494900		14461000	
tr|Q9RTJ1	tr|Q9RTJ1	883			22938000			
tr|Q9RW9	tr|Q9RW9	1376	1.7E+08	1.54E+08	1.48E+08			
tr|Q9RT92	tr|Q9RT92	838			27095000			
tr|Q9RS95	tr|Q9RS95	669	42538000	57908000	40077000	36677000	30299000	40674000
tr|Q9RS17	tr|Q9RS17	630						
tr|Q9RYE9	tr|Q9RYE9	1741				69730000		
tr|Q9RRF0	tr|Q9RRF0	514			38550000	34535000	34624000	34635000
tr|Q9RYD7	tr|Q9RYD7	1734	26171000	19417000	19156000	15622000	24708000	29026000
tr|Q9RYD7	tr|Q9RYD7	1734			13281000			
tr|Q9RVW	tr|Q9RVW	1313	1.49E+08	2.51E+08	1.55E+08	1.42E+08	1.24E+08	1.13E+08
sp|Q9RV2	sp|Q9RV2	262		8334500		27506000		20929000
sp|Q4657	sp|Q4657	61						
sp|Q4657	sp|Q4657	62	1441600	730220	394000		511650	
sp|Q4657	sp|Q4657	62	53602000	45935000	93014000	20391000	29009000	32184000
tr|Q9RVZ0	tr|Q9RVZ0	1324			16770000	77906000	50251000	67364000
tr|Q9RVZ0	tr|Q9RVZ0	1324						
sp|Q9RR7	sp|Q9RR7	67						
sp|Q9RXI7	sp|Q9RXI7	373	88366000	2.51E+08	2.15E+08	2.02E+08	1.58E+08	2.18E+08
tr|Q9RY02	tr|Q9RY02	1675		95191000	20538000	42719000	83019000	
tr|Q9RTL0	tr|Q9RTL0	892	12384000	28056000	74769000			
tr|Q9RTX4	tr|Q9RTX4	949	2.64E+08	3.56E+08	1.41E+08	4.34E+08	3.67E+08	4.88E+08
sp|Q9RVI3	sp|Q9RVI3	289	39655000	58054000	35041000			
tr|Q9RZI5	tr|Q9RZI5	1936	95394000	93914000	90146000		48747000	59570000

tr|Q9RR6	tr|Q9RR66	468			1.22E+08	48156000		59665000
tr|Q9RWC	tr|Q9RWQ	1453	64214000	94550000	85250000		1.4E+08	1.25E+08
tr|Q9RVR	tr|Q9RVR	1281			15760000			
tr|Q9RWD	tr|Q9RWD	1394	30387000		28813000			
tr|Q9RSW	tr|Q9RSW	775	23418000	10171000	10960000			8122400
tr|Q9RSW	tr|Q9RSW	775			34087000			
sp|Q9RXJ	sp|Q9RXJ	384	5336200	1396200	1549000	1301100		1821900
tr|Q9RXY	tr|Q9RXY	1665						
sp|Q9RY7	sp|Q9RY7	423	25191000	30898000	34627000		17445000	
tr|Q9RSS	tr|Q9RSS3	756		1391300				
tr|Q9RVL	tr|Q9RVL2	1251						
tr|Q9RYZ	tr|Q9RYZ7	1841	65964000	55307000	64452000	50084000	36345000	32897000
sp|Q9RTB	sp|Q9RTB	176			1.34E+09	1.52E+09		1.58E+09
tr|Q9RY2	tr|Q9RY25	1684	54006000	91623000		1.09E+08	91229000	1.05E+08
tr|Q9RTC	tr|Q9RTC7	853	2.35E+08	2.68E+08	2.38E+08	1.55E+08	1.46E+08	1.21E+08
sp|Q9RV	sp|Q9RV	292	1.12E+08	1.1E+08	96808000	1.4E+08	1.26E+08	1E+08
tr|Q9RU9	tr|Q9RU9	1015	17806000	15718000	17514000	15610000	24492000	14591000
tr|Q9RZA	tr|Q9RZA	1892			9890200		5090000	
tr|Q9RSU	tr|Q9RSU	768	27799000	23351000	34332000	5641200		4875500
tr|Q9RUE	tr|Q9RUE	1044	89736000	91562000	1.56E+08	2.02E+08	2.27E+08	2.78E+08
tr|Q9RZT	tr|Q9RZT	1968						
sp|Q9RW	sp|Q9RW	347	8967200	10451000	7401200	77610000	83093000	37943000
tr|Q9RY7	tr|Q9RY75	1705						
tr|Q9RYM	tr|Q9RYM	1773	37369000	53947000	64137000	73135000	59454000	78462000
tr|Q9RVN	tr|Q9RVN	1260	1.68E+08	25751000	1.6E+08	5.38E+08	3.79E+08	3.54E+08
tr|Q9RVN	tr|Q9RVN	1260		28254000	36602000			
sp|Q9RUZ	sp|Q9RUZ	260	3.17E+08	3.38E+08	3.41E+08	2.91E+08	2.67E+08	2.58E+08
tr|Q9RXH	tr|Q9RXH	1594	22818000	21138000	20550000	28375000		26953000
sp|Q9RR9	sp|Q9RR9	71	2435900		418370		834700	
tr|Q9RXQ	tr|Q9RXQ	1631	25864000		14489000	32976000	34835000	38379000
tr|Q9RTI3	tr|Q9RTI3	879	1.21E+08	1.85E+08	1.61E+08	1.18E+08	1.55E+08	1.16E+08
tr|Q9RS11	tr|Q9RS11	627	1.03E+08	1.15E+08	97578000	2.75E+08	2.78E+08	3.14E+08
tr|Q9RVB	tr|Q9RVB	1206	89221000	78336000	61911000	64631000	59738000	73556000
tr|Q9RVK	tr|Q9RVK	1249	81340000	55603000	49575000			
tr|Q9RZH	tr|Q9RZH	1931						
tr|Q9RYG	tr|Q9RYG	1748;1749						
tr|Q9RYG	tr|Q9RYG5	1750						
tr|Q9RZS	tr|Q9RZS5	1965						
tr|Q9RUC	tr|Q9RUC	1038	10855000					
sp|Q9RXJ	sp|Q9RXJ7	382	472770		133880			
tr|Q9RRV	tr|Q9RRV1	588	3407700	665350	916110	3236900	3741500	
tr|Q9RUZ	tr|Q9RUZ	1147	45312000	29089000	48387000		27557000	50483000
sp|P5686	sp|P56861	54	90358000	1.43E+08	1.17E+08	1.4E+08	1.18E+08	1.53E+08
sp|P5686	sp|P56861	54						
tr|Q9RXM	tr|Q9RXM	1615				19941000	28710000	21242000
sp|Q9RTJ	sp|Q9RTJ3	189	54414000	27078000	27870000	10369000	9949900	10555000
sp|Q9RSL	sp|Q9RSL	133	1.72E+09	1.95E+09	1.66E+09	3.78E+08	8.47E+08	5.63E+08
sp|Q9RW	sp|Q9RW	342	5.75E+09	3.95E+09	4.83E+09	1.65E+09	1.98E+09	2.24E+09
tr|Q9RWT	tr|Q9RWT	1465						
tr|Q9RVE	tr|Q9RVE	1220						
tr|Q9RRW	tr|Q9RRW	599	32516000	33299000	62558000	37410000	46081000	58356000

tr|Q9RY67	tr|Q9RY67	1701	1.21E+08	1.56E+08	2.31E+08	1.84E+08	2.06E+08	1.91E+08
tr|Q9RY67	tr|Q9RY67	1701			73559000	58633000	94104000	83904000
tr|Q9RUM	tr|Q9RUM	1091	14650000		27245000	29931000	26233000	20240000
tr|Q9RUM	tr|Q9RUM	1091			11586000			
sp|Q9RUE	sp|Q9RUE	226			94268000	74210000	1.09E+08	67792000
tr|Q9RSP8	tr|Q9RSP8	745	38414000					
tr|Q9RZQ	tr|Q9RZQ	1958			14170000			
sp|Q9RSS	sp|Q9RSS	149	6.08E+08	5.65E+08	6.19E+08	11485000	40003000	16338000
sp|Q9RVP	sp|Q9RVP	294	2.1E+09	2.25E+09	2.35E+09	1.69E+09	1.59E+09	1.66E+09
sp|P56867	sp|P56867	56	1.3E+09	9.86E+08	1.1E+09	1.9E+08	1.58E+08	2.79E+08
tr|Q9RZ97	tr|Q9RZ97	1885	12586000	9485900	11318000			19429000
tr|Q9RXM	tr|Q9RXM	1617	19214000	16150000				
tr|Q9RZV2	tr|Q9RZV2	1982						
sp|Q9RR9	sp|Q9RR9	74		6663700	6163200			10622000
tr|Q9RZI5	tr|Q9RZI5	1936	14460000		10874000		16811000	
tr|Q9RU01	tr|Q9RU01	969	95199000	91869000	75776000	81187000	72692000	92291000
tr|Q9RTZ9	tr|Q9RTZ9	967	2.02E+08	2.89E+08	2.01E+08	2.17E+08	1.91E+08	2.35E+08
tr|Q9RZD1	tr|Q9RZD1	1907	60508000	62177000	58703000	40729000	64065000	46343000
tr|Q9RYC0	tr|Q9RYC0	1723	30572000	25540000	32818000	23104000	36722000	29462000
tr|Q9RWN	tr|Q9RWN	1441	5764000	6203400	8583200	30333000	25763000	20486000
sp|Q5933	sp|Q5933	63	77300000	75413000	83059000	1.19E+08	1.47E+08	1.06E+08
sp|Q5933	sp|Q5933	63				1771400	3133800	2620800
tr|Q9RWE	tr|Q9RWE	1397	41801000	50113000	49699000			
tr|Q9RZB2	tr|Q9RZB2	1895						
sp|Q9RRB	sp|Q9RRB	76	9.83E+08	5.96E+08	5.84E+08	4.87E+08	5.29E+08	5.77E+08
tr|Q9RZ49	tr|Q9RZ49	1863						
sp|Q9RY7	sp|Q9RY7	423		32144000		18461000		28041000
tr|Q9RZQ	tr|Q9RZQ	1957			23409000			
tr|Q9RTM	tr|Q9RTM	902	2.23E+08	2.72E+08	2.02E+08	1.31E+08	1.18E+08	1.22E+08
tr|Q9RWX	tr|Q9RWX	1498	20433000	24306000	34078000	30820000	29015000	24529000
sp|Q9RY7	sp|Q9RY7	422						
sp|Q9RRB	sp|Q9RRB	77	2.13E+09	2.24E+09	1.87E+09	1.65E+09	1.48E+09	1.61E+09
sp|Q9RSA	sp|Q9RSA	114	2.93E+08	8.9E+08	8.39E+08	1.09E+09	8.84E+08	8.85E+08
sp|Q9RSA	sp|Q9RSA	114		20389000				
tr|Q9RS96	tr|Q9RS96	670	69040000	56497000	48803000	63946000	44226000	57725000
tr|Q9RYC7	tr|Q9RYC7	1728	5196500	5370100				5539300
tr|Q9RUU	tr|Q9RUU	1124						
sp|Q9RRC	sp|Q9RRC	78	1.28E+08	86482000		60318000	96643000	67721000
tr|Q9RXD5	tr|Q9RXD5	1573						
tr|Q9RXZ9	tr|Q9RXZ9	1672	8899200	5538100	16336000	5361300		24876000
tr|Q9RUE0	tr|Q9RUE0	1044	7.43E+08	6.19E+08	7.09E+08	8.98E+08	1.03E+09	9.75E+08
tr|Q9RUE0	tr|Q9RUE0	1044						
tr|Q9RTZ9	tr|Q9RTZ9	967	62546000	42873000	47153000	32278000		36408000
sp|Q9RSE	sp|Q9RSE	116	9172500	19887000	15204000	61526000	70614000	32133000
tr|Q9RU02	tr|Q9RU02	970	1881800	980390	1144600	2195400	2719000	2214200
tr|Q9RUG	tr|Q9RUG	1051	1.09E+08	75294000	91386000	36136000		
tr|Q9RXG3	tr|Q9RXG3	1589		91927000		84631000	61675000	71801000
sp|Q9RV6	sp|Q9RV6	266		17437000	21743000	53657000	36130000	52398000
tr|Q9RUJ1	tr|Q9RUJ1	1067		24620000	22206000			
tr|Q9RSE5	tr|Q9RSE5	704	77142000		54041000	63250000	96320000	29787000
tr|Q9RTL4	tr|Q9RTL4	895						

tr|Q9RT87	tr|Q9RT87	837	26928000	35984000	40790000	34478000	55111000	26249000
sp|Q9RZ0	sp|Q9RZ0	451		26986000	30739000	1.18E+08	35847000	
tr|Q9RVQ	tr|Q9RVQ	1274	8968800	2290700	2529900	1924100	2885300	2620100
sp|Q9RUA	sp|Q9RUA	224		15458000	17917000	9916600	10084000	13439000
tr|Q9RWP	tr|Q9RWP	1449	65203000	48589000	36417000	30669000	32906000	48339000
tr|Q9RSF1	tr|Q9RSF1	707						
tr|Q9RU9	tr|Q9RU9	1016		18245000	31817000	69615000	43918000	47180000
tr|Q9RXX2	tr|Q9RXX2	1660	84443000	71739000	45521000	50000000	49264000	6476000
sp|Q9RXC	sp|Q9RXC	367	5.31E+08	6.53E+08	6.48E+08	1.18E+09	1.08E+09	1.14E+09
sp|Q9RU7	sp|Q9RU7	215				14087000		16558000
tr|Q9RWB	tr|Q9RWB	1385	2.19E+09	2.03E+09	1.82E+09	1.66E+09	1.35E+09	1.46E+09
tr|Q9RZA3	tr|Q9RZA3	1889						
tr|Q9RVJ3	tr|Q9RVJ3	1240			10530000			
tr|Q9RXD5	tr|Q9RXD5	1573		21334000	20076000			
tr|Q9RTQ5	tr|Q9RTQ5	915		67479000		19729000	13862000	16644000
tr|Q9RTQ5	tr|Q9RTQ5	915	1.75E+08	4.31E+08	3.74E+08	1.16E+08	41105000	1.11E+08
tr|Q9RY75	tr|Q9RY75	1705						
sp|Q9RY2	sp|Q9RY2	411						
sp|Q9RY2	sp|Q9RY2	411	4.18E+08	6.33E+08	5.33E+08	2.81E+08	3.85E+08	3.44E+08
tr|Q9RSQ4	tr|Q9RSQ4	748	2.31E+09	2.29E+09	2.52E+09	9.81E+08	1.11E+09	1.1E+09
tr|Q9RSQ4	tr|Q9RSQ4	748					36792000	
tr|Q9RRE6	tr|Q9RRE6	512	34930000	33390000	21503000		20113000	18583000
sp|Q4657	sp|Q4657	61	1.08E+08	1.06E+08	1.05E+08	1.61E+08	1.37E+08	1.09E+08
sp|Q4657	sp|Q4657	61			22534000			
tr|Q9RZM	tr|Q9RZM	1949	88172000	1.13E+08	81408000	97104000	72276000	81116000
tr|Q9RT05	tr|Q9RT05	794						
sp|Q9RSS	sp|Q9RSS	148	7.13E+08	6.56E+08	5.82E+08	2.39E+08	3.08E+08	5.14E+08
tr|Q9RVQ	tr|Q9RVQ	1274	3.84E+08	2.64E+08	2.65E+08	4.39E+08	3.52E+08	3.64E+08
tr|Q9RXX2	tr|Q9RXX2	1660		16840000	27524000			
tr|Q9RX49	tr|Q9RX49	1527						27949000
tr|Q9RRC7	tr|Q9RRC7	501	69538000	61088000	59648000	51888000	40701000	44434000
tr|Q9RWP	tr|Q9RWP	1449	4.4E+08	3.52E+08	4.25E+08	3.38E+08	2.88E+08	3.58E+08
tr|Q9RU9	tr|Q9RU9	1017	2.38E+08	2.19E+08	2.21E+08	3.92E+08		4.09E+08
tr|Q9RRN	tr|Q9RRN	551	51713000	37374000	27468000	47522000	32400000	37970000
tr|Q9RTD3	tr|Q9RTD3	857	30764000	33432000	26961000	58213000	47025000	62540000
sp|Q9RUE	sp|Q9RUE	228	48534000		48446000	44277000		37334000
tr|Q9RUX3	tr|Q9RUX3	1132		3.45E+08	2.86E+08	1.44E+08	1.1E+08	1.47E+08
sp|P42443	sp|P42443	49			9450300			31721000
tr|Q9RZJ2	tr|Q9RZJ2	1939	51662000	94993000	88926000	27640000	38375000	29635000
sp|Q9RRJ6	sp|Q9RRJ6	89	59301000	19188000	38953000			
sp|Q9RWI	sp|Q9RWI	323						
tr|Q9RW9	tr|Q9RW9	1373						
tr|Q9RTK5	tr|Q9RTK5	889	42826000	47993000	54727000		9250300	
tr|Q9RYW	tr|Q9RYW	1829	18867000	18981000	16764000	30333000	24561000	31432000
tr|Q9RVV1	tr|Q9RVV1	1305					4745600	
tr|Q9RT09	tr|Q9RT09	798		1.16E+08	1.03E+08	48926000	75654000	83204000
tr|Q9RZQ	tr|Q9RZQ	1959				26234000		29501000
sp|Q9RR9	sp|Q9RR9	73		40451000	46170000	15571000	21524000	19001000
sp|Q9RRC	sp|Q9RRC	78	27997000	18400000	16152000	14533000	12166000	17556000
tr|Q9RX48	tr|Q9RX48	1526		5119300	3781400	16675000	16615000	17567000
tr|Q9RVU8	tr|Q9RVU8	1303	1.04E+09	1.28E+09	1.34E+09	3.9E+08	6.16E+08	4.72E+08

tr|Q9RTV5	tr|Q9RTV5	938					19925000	
sp|Q9RR6	sp|Q9RR6	65	1.26E+08	75955000	1.2E+08	1.97E+08	2.43E+08	2.72E+08
sp|Q9RS1	sp|Q9RS1	106	1.58E+09	1.54E+09		1.64E+09	1.67E+09	
tr|Q9RTH7	tr|Q9RTH7	878						
tr|Q9RSY1	tr|Q9RSY1	782	5.78E+08	4.96E+08	4.11E+08			
tr|Q9RWL	tr|Q9RWL	1437		86346000	70046000			
tr|Q9RSP0	tr|Q9RSP0	743			1.17E+08			
sp|Q9RY5	sp|Q9RY5	418	2.61E+08	2.92E+08	2.76E+08	5.86E+08	7.57E+08	3.4E+08
tr|Q9RVI7	tr|Q9RVI7	1236	10260000	6052700	16214000			
sp|Q9RW9	sp|Q9RW9	345	1.81E+08	1.74E+08	1.48E+08	69572000	7678300	83868000
sp|Q9RYU	sp|Q9RYU	443	78799000	62807000	64393000			
sp|Q9RYU	sp|Q9RYU	443	93335000	65383000	1.08E+08	47642000	43994000	41115000
tr|Q9RYC0	tr|Q9RYC0	1723	3.28E+08	2.96E+08	2.29E+08		1.4E+08	1.42E+08
tr|Q9RVB5	tr|Q9RVB5	1209			3460000			
tr|Q9RVB5	tr|Q9RVB5	1209	77523000	75823000	75515000			
tr|Q9RYS8	tr|Q9RYS8	1807	9.23E+08	7.59E+08	8.17E+08	2.71E+08	3.18E+08	4.54E+08
sp|Q9RRE	sp|Q9RRE	81	1.37E+08	1.06E+08	1.24E+08	1.2E+08	1.2E+08	1.45E+08
tr|Q9RTX4	tr|Q9RTX4	949		9676800	23100000	18411000	16309000	7945900
tr|Q9RVZ3	tr|Q9RVZ3	1326	5386900	1784400	1534200	1046800	2681300	1234700
sp|Q9RSQ	sp|Q9RSQ	141						
sp|Q9RV6	sp|Q9RV6	267				34074000	23850000	63937000
tr|Q9RVA7	tr|Q9RVA7	1203	64315000	47702000	56436000			
tr|Q9RZQ	tr|Q9RZQ	1959	5932800			5.62E+08	5.39E+08	5.79E+08
tr|Q9RR62	tr|Q9RR62	467	49684000	1.43E+08	2.39E+08	3.5E+08	2.95E+08	3.53E+08
tr|Q9RZQ	tr|Q9RZQ	1958	3.7E+08	3.19E+08	3E+08	2.88E+08	1.99E+08	1.75E+08
sp|Q9RRB	sp|Q9RRB	76	7.14E+08	7E+08	7.18E+08	1.79E+08	1.72E+08	2.92E+08
sp|Q9RRB	sp|Q9RRB	76						
tr|Q9RT49	tr|Q9RT49	818	27229000	24805000	24531000	28807000		22280000
tr|Q9RRS3	tr|Q9RRS3	574		9964800	17692000		22891000	10667000
tr|Q9RWU	tr|Q9RWU	1479	1.71E+08	1.88E+08	1.59E+08	2.19E+08	1.42E+08	1.81E+08
tr|Q9RZB9	tr|Q9RZB9	1899		5538200	6940000	23737000	5064900	3166700
sp|Q9RT2	sp|Q9RT2	164	47342000	73425000	1.13E+08	91551000	27641000	94331000
tr|Q9RVF7	tr|Q9RVF7	1224		58397000				
tr|Q9RUB	tr|Q9RUB	1028				83774000	81144000	87259000
tr|Q9RU56	tr|Q9RU56	999		16434000	33106000	34506000	31930000	46862000
sp|Q9RTG	sp|Q9RTG	184						
tr|Q9RZ04	tr|Q9RZ04	1844			67103000		85800000	77931000
tr|Q9RV90	tr|Q9RV90	1195						
tr|Q9RZ74	tr|Q9RZ74	1868		15962000	8338300	17366000	17697000	21190000
tr|Q9RWU	tr|Q9RWU	1475	1.43E+08	80891000	86976000		59257000	31675000
tr|Q9RS77	tr|Q9RS77	658						
sp|Q9RXZ	sp|Q9RXZ	409				32747000	25197000	29841000
sp|Q9RZU	sp|Q9RZU	459	69938000	63515000	73299000	8230300		
tr|Q9RY78	tr|Q9RY78	1707	8.4E+08	7.47E+08	7.35E+08	8.16E+08	6.12E+08	6.54E+08
tr|Q9RTU7	tr|Q9RTU7	935			30635000	69567000	84643000	82239000
tr|Q9RX12	tr|Q9RX12	1509	1.75E+08	1.81E+08	1.49E+08	1.35E+08	1.64E+08	1.34E+08
tr|Q9RTE3	tr|Q9RTE3	862	22345000	17369000	30907000	18211000	16791000	
tr|Q9RYB8	tr|Q9RYB8	1722	8.54E+08	4.82E+08	4.92E+08	6.54E+08	4.21E+08	5.41E+08
tr|Q9RX69	tr|Q9RX69	1540				46103000		29238000
tr|Q9RUD	tr|Q9RUD	1039	5.53E+08	6.41E+08	8.63E+08	5.98E+08	6.27E+08	9.47E+08
tr|Q9RUD	tr|Q9RUD	1039	64269000	40531000	69493000	22660000		35088000

tr|Q9RVJ0	tr|Q9RVJ0	1238	2.44E+08	2.54E+08	2.3E+08			
tr|Q9RUY2	tr|Q9RUY2	1139	6919800	2692300	2990300			1353400
tr|Q9RR85	tr|Q9RR85	476	50860000	46397000	54032000			
sp|Q9RSS8	sp|Q9RSS8	149	7.88E+08	1.56E+09	2.16E+09	4.02E+08	6.67E+08	6.89E+08
tr|Q9RRG4	tr|Q9RRG4	520	1.24E+09	9.38E+08	1.21E+09	3.21E+08	3.6E+08	4.72E+08
sp|Q9RUN	sp|Q9RUN	242						
sp|Q9RUN	sp|Q9RUN	242			26176000			
sp|Q9RWU	sp|Q9RWU	347		71567000	87418000		53104000	
tr|Q9RX16	tr|Q9RX16	1511			23984000	50103000	61115000	51823000
sp|Q9RZU	sp|Q9RZU	458						
tr|Q9RWP	tr|Q9RWP	1449		29791000	67577000			
tr|Q9RZA1	tr|Q9RZA1	1888			7828600		17244000	
tr|Q9RZA1	tr|Q9RZA1	1888	21923000	48899000	80280000		88736000	68093000
sp|Q9RS6	sp|Q9RS6	111		13630000	15289000	5027700	6965900	7296000
sp|Q9RW9	sp|Q9RW9	318						
tr|Q9RSA8	tr|Q9RSA8	679	78810000	1.59E+08	78187000	1.33E+08	1.52E+08	1.33E+08
sp|Q9RWH	sp|Q9RWH	329						
sp|Q9RU1	sp|Q9RU1	205						
tr|Q9RUJ8	tr|Q9RUJ8	1073	1.59E+08	1.23E+08	1.4E+08			
tr|Q9RZB2	tr|Q9RZB2	1895				34832000	39223000	
sp|Q9RXK	sp|Q9RXK	385				74471000	41756000	58281000
tr|Q9RS10	tr|Q9RS10	626	8.24E+08	8.3E+08	8.25E+08	6.67E+08	4.99E+08	5.55E+08
sp|Q9RSL	sp|Q9RSL	133	6.53E+08	4.13E+08	5.52E+08	30239000		
sp|Q9RSL	sp|Q9RSL	133			12133000			
tr|Q9RVN	tr|Q9RVN	1264	42911000	48751000	93033000	60087000	76242000	1.01E+08
tr|Q9RVN	tr|Q9RVN	1264			56918000	1.71E+08	1.84E+08	1.22E+08
tr|Q9RRU4	tr|Q9RRU4	585			13841000			
sp|Q9RRQ	sp|Q9RRQ	95	2.15E+08	2.35E+08	2.18E+08	1.95E+08	24439000	1.87E+08
tr|Q9RWQ	tr|Q9RWQ	1454	24140000	23341000	12062000	31093000		43180000
tr|Q9RYB8	tr|Q9RYB8	1722	80113000	63427000	74475000	1.6E+08	1.74E+08	1.36E+08
sp|Q9RUE	sp|Q9RUE	226	9.79E+08	6.86E+08	6.81E+08		24889000	28553000
sp|Q9RT2	sp|Q9RT2	164						
tr|Q9RTE3	tr|Q9RTE3	862	1.43E+08	1.24E+08	1.24E+08	89804000	67104000	82606000
tr|Q9RRL3	tr|Q9RRL3	546		50051000		58918000		
tr|Q9RVK8	tr|Q9RVK8	1248	10802000					
sp|Q9RTN	sp|Q9RTN	196	1.36E+08	1.25E+08	1.7E+08	34119000	60648000	74222000
tr|Q9RXB8	tr|Q9RXB8	1566						23263000
tr|Q9RWI0	tr|Q9RWI0	1413					2.04E+08	1.71E+08
tr|Q9RU54	tr|Q9RU54	997						
sp|Q9RUE	sp|Q9RUE	229	15086000	4655700	3968700		5003400	836740
sp|Q9RU7	sp|Q9RU7	215				1141500	3452000	2718700
tr|Q9RYN9	tr|Q9RYN9	1782	1.46E+08	65644000	47428000	1.56E+08	1.51E+08	1.5E+08
tr|Q9RXD7	tr|Q9RXD7	1574	38188000	42925000	45549000			20852000
tr|Q9RZF3	tr|Q9RZF3	1921	87776000	78592000	73978000	44988000	31031000	39447000
sp|Q9RVL	sp|Q9RVL	290				25587000	21646000	
tr|Q9RTZ7	tr|Q9RTZ7	965	37626000	28947000		23007000		
sp|Q9RTJ3	sp|Q9RTJ3	189	1.12E+09	8.27E+08	9.47E+08	1.17E+09	1.15E+09	8.8E+08
sp|P42443	sp|P42443	49					28610000	
tr|Q9RVL5	tr|Q9RVL5	1252						
sp|Q9RUP	sp|Q9RUP	246					19145000	
tr|Q9RZQ	tr|Q9RZQ	1959						

tr|Q9RXX3	tr|Q9RXX3	1661	53945000	38854000	21497000	95860000	97042000	71384000
tr|Q9RSU7	tr|Q9RSU7	767						
tr|Q9RXL4	tr|Q9RXL4	1610			37510000	48871000	52895000	49281000
tr|Q9RSY1	tr|Q9RSY1	782	2.57E+08	1.98E+08	1.88E+08	1.91E+08	1.88E+08	1.78E+08
tr|Q9RT05	tr|Q9RT05	794						
tr|Q9RRC0	tr|Q9RRC0	497	30257000	24960000	22423000		14700000	
tr|Q9RYE4	tr|Q9RYE4	1738	2.84E+08	2.58E+08	2.43E+08	2.46E+08	2.36E+08	2.16E+08
sp|Q9RX0	sp|Q9RX0	350		24917000	30477000	37367000	28283000	30072000
tr|Q9RZR4	tr|Q9RZR4	1962				52824000	30250000	55138000
tr|Q9RS88	tr|Q9RS88	664	38287000	52222000	47009000	60684000	25761000	22863000
sp|Q9RYB	sp|Q9RYB	427					30639000	32581000
tr|Q9RVA7	tr|Q9RVA7	1203				25145000		
sp|Q9RV2	sp|Q9RV2	264	90121000		98443000	1.28E+08	95873000	1.1E+08
sp|Q9RSJ6	sp|Q9RSJ6	124						
sp|Q9RV7	sp|Q9RV7	268	1.16E+08	1.28E+08	1.45E+08	91605000	47837000	63483000
tr|Q9RSC7	tr|Q9RSC7	690	57879000	61592000	48310000			26950000
sp|Q9RSN	sp|Q9RSN	138	6.16E+08	9.1E+08	8.86E+08	4.34E+08	54586000	4.1E+08
tr|Q9RUK9	tr|Q9RUK9	1081	14080000		12030000	20587000	24803000	50221000
tr|Q9RZT0	tr|Q9RZT0	1968	41327000	32378000	31241000		9085700	
tr|Q9RX48	tr|Q9RX48	1526	4.45E+08	4.21E+08	4.32E+08	3.5E+08	3.28E+08	3.19E+08
sp|Q9RR9	sp|Q9RR9	73	32672000	25833000	32545000	27481000	38278000	37198000
tr|Q9RSD4	tr|Q9RSD4	696	13951000		5362600			
sp|Q9RS1	sp|Q9RS1	106	4.08E+08	3.76E+08	3.88E+08	3.01E+08	2.49E+08	3.13E+08
tr|Q9RYN6	tr|Q9RYN6	1780	41268000	17263000	16935000	11101000	14257000	16362000
tr|Q9RU54	tr|Q9RU54	997	1.37E+08	2.47E+08	1.28E+08	1.11E+09	8.49E+08	3.06E+08
tr|Q9RVL6	tr|Q9RVL6	1253		20800000				
tr|Q9RUH	tr|Q9RUH	1056	5832000					25782000
tr|Q9RS88	tr|Q9RS88	664	66547000	46263000	51047000	1.66E+08	47618000	
sp|Q9RW7	sp|Q9RW7	314						
tr|Q9RTU0	tr|Q9RTU0	932	2.72E+08	2.41E+08	2.65E+08	1.85E+08	1.46E+08	1.53E+08
tr|Q9RVB2	tr|Q9RVB2	1206	1.25E+08	1.09E+08	1.04E+08	1.86E+08	1.68E+08	1.51E+08
tr|Q9RVX7	tr|Q9RVX7	1318	1.21E+09	1.02E+09	1.1E+09	1.01E+09	9.19E+08	1.02E+09
tr|Q9RVX7	tr|Q9RVX7	1318	1.69E+08	1.79E+08	2.66E+08		70631000	
tr|Q9RRJ7	tr|Q9RRJ7	537	2.1E+08		2.85E+08			
tr|Q9RYS0	tr|Q9RYS0	1800	45987000		32286000		1.48E+08	
tr|Q9RTZ3	tr|Q9RTZ3	963			18768000			
sp|Q9RSQ	sp|Q9RSQ	141						
sp|Q9RSG	sp|Q9RSG	118	3.35E+08	3.18E+08	2.66E+08	2.78E+08	2.98E+08	3.17E+08
sp|Q9RVC	sp|Q9RVC	277	27796000					
sp|Q9RVD	sp|Q9RVD	281	60297000	59826000	62297000			
sp|Q9RUF	sp|Q9RUF	231	1.82E+08	2.71E+08	3.13E+08	3.51E+08	2.93E+08	2.46E+08
sp|Q9RU6	sp|Q9RU6	212						
tr|Q9RWT	tr|Q9RWT	1467	31650000	17309000	25275000			
tr|Q9RYH8	tr|Q9RYH8	1757	44118000	56785000	93716000			
tr|Q9RV47	tr|Q9RV47	1174	2.34E+08	2.87E+08	2.09E+08	2.55E+08	2.18E+08	2.49E+08
tr|Q9RT02	tr|Q9RT02	793	2.52E+08	2.49E+08	3.5E+08	3.16E+08	2.92E+08	4.1E+08
tr|Q9RT24	tr|Q9RT24	803	1.4E+09	1.48E+09	1.12E+09	1.66E+09	1.26E+09	1.83E+09
tr|Q9RRG5	tr|Q9RRG5	521	5.37E+08	6.78E+08	5.76E+08	7.33E+08	4.96E+08	4.59E+08
tr|Q9RRF4	tr|Q9RRF4	516						
tr|Q9RX83	tr|Q9RX83	1548	4652900	2330200	30000000			
sp|Q9RV7	sp|Q9RV7	269						

tr|Q9RZB3	tr|Q9RZB3	1896	64812000	66937000	46660000			
sp|Q9RVF	sp|Q9RVF	285						
tr|Q9RVH2	tr|Q9RVH2	1233						
sp|Q9RR8	sp|Q9RR8	69						
tr|Q9RR85	tr|Q9RR85	476	36389000	58405000	79147000		18164000	15258000
tr|Q9RS04	tr|Q9RS04	621	27507000	40985000	44518000	23880000	18909000	
sp|Q9RUZ	sp|Q9RUZ	260	1.63E+08	1.9E+08	3.04E+08	1.09E+09	5E+08	4.95E+08
sp|Q9RUF	sp|Q9RUF	230	2.55E+08	1.92E+08	2.14E+08	1.96E+08	1.75E+08	2.17E+08
tr|Q9RUK8	tr|Q9RUK8	1080	3.35E+08	3.55E+08	3.07E+08	1.84E+08	1.62E+08	1.27E+08
tr|Q9RZG1	tr|Q9RZG1	1924			34408000	44498000	41148000	
tr|Q9RT51	tr|Q9RT51	820			29623000	96611000	75546000	73452000
tr|Q9RYR7	tr|Q9RYR7	1799	5.75E+08	4.62E+08	4.17E+08	7.37E+08	8.72E+08	8.32E+08
tr|Q9RXK4	tr|Q9RXK4	1607	51466000	50745000	42583000	66272000	45162000	44858000
tr|Q9RVJ7	tr|Q9RVJ7	1242	73831000	61474000	57611000	55692000	58434000	55505000
tr|Q9RZL3	tr|Q9RZL3	1942	51636000	84156000	61778000	78507000	85013000	75629000
tr|Q9RU69	tr|Q9RU69	1008	811790					
sp|Q9RW9	sp|Q9RW9	318	84719000	85352000	99741000	32883000	37704000	30678000
tr|Q9RU67	tr|Q9RU67	1007	29308000	31977000				
sp|Q9RTF5	sp|Q9RTF5	182	8.68E+08	7.51E+08	5.81E+08	5.13E+08	4.35E+08	4.78E+08
sp|Q9RTF5	sp|Q9RTF5	182			48109000			14779000
tr|Q9RTP2	tr|Q9RTP2	907				37219000	27042000	
sp|Q9RYH	sp|Q9RYH	435	37983000	47241000	36686000	39790000		53099000
tr|Q9RUU	tr|Q9RUU	1123					32929000	
sp|Q9RVW	sp|Q9RVW	301	1.94E+08	2.41E+08	1.7E+08	53969000	44377000	76148000
sp|Q9RVW	sp|Q9RVW	301						
tr|Q9RZE3	tr|Q9RZE3	1913	2.04E+08	2.23E+08	2.09E+08	44729000		1.29E+08
tr|Q9RSR8	tr|Q9RSR8	753						
tr|Q9RWX	tr|Q9RWX	1497		40752000	28468000			
tr|Q9RWX	tr|Q9RWX	1498	13564000	25012000	29816000			
tr|Q9RWV	tr|Q9RWV	1486				1.81E+08	1.64E+08	1.81E+08
tr|Q9RVJ3	tr|Q9RVJ3	1240			30361000			
tr|Q9RZ94	tr|Q9RZ94	1884	50656000	48512000	41523000	72545000	59820000	75230000
tr|Q9RTT5	tr|Q9RTT5	930	3.75E+08	4.02E+08	5.01E+08	3.92E+08	4.65E+08	3.06E+08
tr|Q9RYC0	tr|Q9RYC0	1723			33325000	63431000	67808000	50976000
tr|Q9RU43	tr|Q9RU43	990	1.2E+09	1.27E+09	1.25E+09	1.09E+09	6.93E+08	1.36E+09
tr|Q9RRA0	tr|Q9RRA0	483	1.32E+09	1.06E+09	8.29E+08	7.86E+08	7.84E+08	7.83E+08
tr|Q9RSN3	tr|Q9RSN3	740	96473000	1.58E+08	89914000		52291000	18257000
tr|Q9RUH	tr|Q9RUH	1056			11285000			
tr|Q9RTM	tr|Q9RTM	899				47602000	60679000	76984000
sp|Q9RV7	sp|Q9RV7	269		17959000	17868000			
tr|Q9RYQ0	tr|Q9RYQ0	1788	1.92E+08	1.76E+08	2.3E+08	1.58E+08	1.34E+08	1.86E+08
tr|Q9RX48	tr|Q9RX48	1526	82364000	23004000	10427000		18954000	19625000
tr|Q9RVC6	tr|Q9RVC6	1212						
tr|Q9RTX7	tr|Q9RTX7	952	5.09E+08	5.1E+08	5.04E+08	5.92E+08	3.28E+08	4.88E+08
tr|Q9RY25	tr|Q9RY25	1684					970920	
tr|Q9RSS3	tr|Q9RSS3	756						
sp|Q9RSJ7	sp|Q9RSJ7	125	1.66E+08	2.93E+08	3.13E+08	1.29E+08	1.87E+08	1.87E+08
sp|Q9RSJ7	sp|Q9RSJ7	125			11849000		34581000	16924000
tr|Q9RXC3	tr|Q9RXC3	1569			4466700		2089400	
sp|Q9RSS1	sp|Q9RSS1	148	2.27E+09	2.69E+09	1.94E+09	1.64E+09	2.44E+09	1.71E+09
tr|Q9RRA0	tr|Q9RRA0	483						

tr|Q9RUN	tr|Q9RUN	1095						
tr|Q9RRV(	tr|Q9RRV(	587	8812300		22545000			
tr|Q9RX16	tr|Q9RX16	1511						1.1E+08
tr|Q9RWI1	tr|Q9RWI1	1414						
tr|Q9RXF0	tr|Q9RXF0	1581				1.01E+08	96620000	85431000
tr|Q9RTH3	tr|Q9RTH3	876						1.07E+08
sp|Q9RSZ	sp|Q9RSZ	159	2.54E+08	2.16E+08	2.54E+08	91557000	1.04E+08	1.64E+08
sp|Q9RTN	sp|Q9RTN	196	1.02E+09	1.4E+09	1.18E+09	5.65E+08	8.77E+08	5.7E+08
tr|Q9RXF0	tr|Q9RXF0	1581			34408000	15567000	11713000	
tr|Q9RYW	tr|Q9RYW	1830		16436000	20044000			
tr|Q9RRI3	tr|Q9RRI3	531	12978000	51589000	43168000	5586200	5145800	38225000
tr|Q9RZQ	tr|Q9RZQ	1959	1.75E+08	2.09E+08	3.33E+08	1.06E+08	96252000	1.66E+08
tr|Q9RVW	tr|Q9RVW	1311						
tr|Q9RVW	tr|Q9RVW	1311	45634000	72956000	1.17E+08	12898000	21078000	20791000
tr|Q9RWV	tr|Q9RWV	1484						
sp|Q9RVV	sp|Q9RVV	300						
sp|Q9RXJ5	sp|Q9RXJ5	380	8588600	2638500	2490100	532040	950360	848510
tr|Q9RZA9	tr|Q9RZA9	1893						
sp|Q9RWE	sp|Q9RWE	320	2.55E+08	2.37E+08	3.12E+08		2.51E+08	2.86E+08
tr|Q9RZ76	tr|Q9RZ76	1870						
tr|Q9RWC	tr|Q9RWC	1389	81974000	88760000	90462000	77892000	73969000	87366000
sp|Q9RXT	sp|Q9RXT	401						
tr|Q9RYI7	tr|Q9RYI7	1763	3.66E+08					
sp|Q9RY8	sp|Q9RY8	424						
tr|Q9RZJ2	tr|Q9RZJ2	1939	8924900		8213200	13483000		
sp|Q9RV9	sp|Q9RV9	273						
tr|Q9RSX5	tr|Q9RSX5	780		12136000	9583500			
sp|Q9RY2	sp|Q9RY2	411			7891700	30368000	46718000	38358000
tr|Q9RVD9	tr|Q9RVD9	1217						
sp|Q9RVV	sp|Q9RVV	300				11516000	16030000	
tr|Q9RWV	tr|Q9RWV	1485						
tr|Q9RYH6	tr|Q9RYH6	1756	1.21E+08	1.82E+08			47305000	46758000
tr|Q9RVB3	tr|Q9RVB3	1207	39904000		7414800			
sp|Q9RV9	sp|Q9RV9	273	1.62E+08	4.04E+08	4.12E+08	9.04E+08	6.99E+08	7.04E+08
tr|Q9RSA4	tr|Q9RSA4	675						
tr|Q9RZH3	tr|Q9RZH3	1929						
tr|Q9RUZ8	tr|Q9RUZ8	1149						
tr|Q9RR71	tr|Q9RR71	470	1.08E+08	55974000	73238000	3.7E+08	3.06E+08	3.08E+08
tr|Q9RR71	tr|Q9RR71	470	57660000	3E+08	4.22E+08	1.11E+08		2.61E+08
tr|Q9RR88	tr|Q9RR88	478			14751000			8335400
tr|Q9RZ93	tr|Q9RZ93	1883	89390000			1.03E+08		
tr|Q9RRS6	tr|Q9RRS6	577					59352000	
sp|Q9RSF(	sp|Q9RSF(	117	3046000		15313000	50473000	40292000	24492000
tr|Q9RYS5	tr|Q9RYS5	1804	62072000	54161000	43819000	89306000	98117000	27927000
tr|Q9RYS5	tr|Q9RYS5	1804	31651000		43126000	26597000	29638000	34262000
tr|Q9RU07	tr|Q9RU07	972	3.28E+08	3.7E+08	3.94E+08	1.36E+08	2.83E+08	2.59E+08
tr|Q9RT75	tr|Q9RT75	831	76023000		83352000	20561000	26963000	
tr|Q9RXX5	tr|Q9RXX5	1662			1494100		1542600	1077100
tr|Q9RW9	tr|Q9RW9	1374						
tr|Q9RU58	tr|Q9RU58	1001	3.94E+08	3.42E+08	3.76E+08	2.45E+08	69680000	93234000
tr|Q9RSN5	tr|Q9RSN5	742	1.88E+08	1.58E+08	1.19E+08	1.27E+08	1.45E+08	

tr|Q9RZ79	tr|Q9RZ79	1873						
tr|Q9RY69	tr|Q9RY69	1702	72677000	66148000	94814000	47648000	40186000	50527000
tr|Q9RZE6	tr|Q9RZE6	1916						
tr|Q9RVG6	tr|Q9RVG6	1228	1.91E+08	1.89E+08	1.65E+08	4.27E+08	4.02E+08	3.21E+08
sp|Q9RX8	sp|Q9RX8	362			50275000	3.61E+08	56914000	1.3E+08
tr|Q9RS87	tr|Q9RS87	663			28735000	25851000	53532000	36469000
tr|Q9RWD	tr|Q9RWD	1394						
sp|Q9RVV	sp|Q9RVV	300				42237000	54640000	53746000
tr|Q9RZC1	tr|Q9RZC1	1901	34064000	65481000				
tr|Q9RUZ9	tr|Q9RUZ9	1150	73695000	60548000	39773000	24317000	25960000	26376000
tr|Q9RUL9	tr|Q9RUL9	1087						
tr|Q9RYG5	tr|Q9RYG5	1750						
tr|Q9RU02	tr|Q9RU02	970	33261000	54977000	60285000			
tr|Q9RS71	tr|Q9RS71	655				25188000		
sp|Q9RX6	sp|Q9RX6	361	2.22E+08	2.33E+08	2.2E+08	94362000	15762000	89291000
tr|Q9RY20	tr|Q9RY20	1681	2.35E+08		1.8E+08	36608000	19083000	57251000
tr|Q9RS41	tr|Q9RS41	641	1.28E+08	1.13E+08	1.6E+08		29060000	27682000
sp|Q9RSL1	sp|Q9RSL1	135						
tr|Q9RZC7	tr|Q9RZC7	1906		1.29E+08	3.09E+08		73960000	1.16E+08
tr|Q9RWT	tr|Q9RWT	1473	57137000	48862000	21028000		12446000	
tr|Q9RZQ	tr|Q9RZQ	1958	1.66E+08	1.23E+08	1.56E+08	1.25E+08	1.09E+08	1.18E+08
sp|O8302	sp|O8302	47	65036000	73908000			41359000	41517000
sp|Q9RWH	sp|Q9RWH	328						6368900
tr|Q9RU50	tr|Q9RU50	994						14151000
tr|Q9RZB4	tr|Q9RZB4	1897	65602000	43620000	42032000	52125000	61889000	1.51E+08
tr|Q9RWU	tr|Q9RWU	1478				8050900	9819100	8652500
tr|Q9RWT	tr|Q9RWT	1468	9.63E+08	8.27E+08	8.45E+08	2.24E+08	2.42E+08	3.91E+08
tr|Q9RX86	tr|Q9RX86	1551	3.21E+08	4.54E+08	3.57E+08	4.81E+08	3.53E+08	4.25E+08
tr|Q9RV32	tr|Q9RV32	1168	66471000	64113000	74136000			48120000
tr|Q9RX57	tr|Q9RX57	1532			2272400	7557600		
tr|Q9RXB9	tr|Q9RXB9	1567	43347000	77459000	76345000	34257000	37832000	27463000
tr|Q9RTE3	tr|Q9RTE3	862	2.73E+08	3.9E+08	3.67E+08	1.12E+08	2.02E+08	2.11E+08
sp|Q9RXT	sp|Q9RXT	401				9596800	10469000	15252000
sp|Q9RX0	sp|Q9RX0	350					29034000	28480000
tr|Q9RVR3	tr|Q9RVR3	1282	64249000	68613000	64206000	74882000	32027000	54650000
sp|Q9RS1	sp|Q9RS1	107				1.18E+08		1.03E+08
tr|Q9RT72	tr|Q9RT72	829		3765800	4425900	1453000	1709000	2290600
tr|Q9RRC5	tr|Q9RRC5	500	34962000	1.07E+08	2.16E+08	1.67E+08	1.96E+08	1.92E+08
tr|Q9RT87	tr|Q9RT87	837	16815000	27624000	31849000		18545000	7052200
tr|Q9RT87	tr|Q9RT87	837						
tr|Q9RUL5	tr|Q9RUL5	1086	2922500	3185900	1826500			
sp|Q9RXU	sp|Q9RXU	402	27475000					
tr|Q9RX36	tr|Q9RX36	1520						
tr|Q9RU25	tr|Q9RU25	978	2.48E+08	2.08E+08	2.17E+08	3.34E+08	3.01E+08	3.34E+08
tr|Q9RZC0	tr|Q9RZC0	1900	1.29E+08	61525000	55042000	98097000	1.45E+08	1.32E+08
sp|Q9RYL	sp|Q9RYL	436				4005700	5200100	4669400
tr|Q9RRQ	tr|Q9RRQ	565						
sp|Q9RT3	sp|Q9RT3	166	2.74E+08	2.24E+08	2.6E+08	90987000	1.12E+08	1.77E+08
tr|Q9RWS	tr|Q9RWS	1464	2.3E+08	2.31E+08	2.63E+08	1.64E+08	1.52E+08	1.63E+08
sp|Q9RSS	sp|Q9RSS	145				18234000	31339000	26569000
sp|Q9RTJ3	sp|Q9RTJ3	189	20359000					

sp|Q9RTJ3	sp|Q9RTJ3	189			23764000			
tr|Q9RVX7	tr|Q9RVX7	1318			25369000		43531000	32264000
tr|Q9RYW	tr|Q9RYW	1830						
tr|Q9RRZ0	tr|Q9RRZ0	611	2.35E+08	1.88E+08	2.57E+08	63190000	50413000	1.19E+08
sp|Q9RZU	sp|Q9RZU	459				36789000	26968000	36632000
sp|Q9RYG	sp|Q9RYG	434						
sp|Q9RY2	sp|Q9RY2	411	23845000	31064000	96463000	1.86E+08	2.08E+08	1.85E+08
tr|Q9RT95	tr|Q9RT95	841			13749000			
tr|Q9RYG3	tr|Q9RYG3	1748	29561000		63754000			26851000
tr|Q9RSN4	tr|Q9RSN4	741						
tr|Q9RSN4	tr|Q9RSN4	741	5.15E+08	4.63E+08	5.07E+08	4.29E+08	2.67E+08	3.96E+08
tr|Q9RWI7	tr|Q9RWI7	1418	1.76E+08	1.87E+08	1.53E+08	1.1E+08	1.03E+08	
tr|Q9RUD	tr|Q9RUD	1039	5.64E+08	4.79E+08	6.34E+08	2.05E+08	2.71E+08	3.59E+08
tr|Q9RUD	tr|Q9RUD	1039		15195000	45475000			
tr|Q9RY75	tr|Q9RY75	1705	44827000	46926000	60333000			
sp|Q9RW	sp|Q9RW	335	80327000	1.41E+08	1.43E+08	80834000	1.07E+08	81278000
sp|Q9RVV	sp|Q9RVV	300	24435000	46141000	35234000	38067000	32993000	28759000
sp|Q9RVV	sp|Q9RVV	300	1.19E+09	1.08E+09	1.09E+09	3.42E+08	4.31E+08	3.7E+08
sp|Q9RVV	sp|Q9RVV	300			20722000			
tr|Q9RTK5	tr|Q9RTK5	889	1.07E+09	1.1E+09	1.18E+09	3.43E+08	2.67E+08	3.26E+08
sp|Q9RXJ8	sp|Q9RXJ8	383			43037000			
tr|Q9RUZ9	tr|Q9RUZ9	1150				32048000		
tr|Q9RVQ	tr|Q9RVQ	1273	6.39E+08	5.41E+08	5.85E+08	1.45E+08	1.56E+08	1.24E+08
tr|Q9RUK0	tr|Q9RUK0	1075	49169000	52200000	41889000			
tr|Q9RYP9	tr|Q9RYP9	1787						
sp|Q9RW	sp|Q9RW	336	43928000		42136000	9029100		
sp|Q9RYB	sp|Q9RYB	426	11875000	4801900	6539700	3346400	4123200	3085500
tr|Q9RRV2	tr|Q9RRV2	589	4.83E+08	3.9E+08	3.79E+08	2.04E+09	1.84E+09	1.7E+09
tr|Q9RRV2	tr|Q9RRV2	589	40452000		39964000	22880000	39997000	31564000
tr|Q9RYN6	tr|Q9RYN6	1780	4.71E+08	1.27E+09	1.16E+09	5.89E+08	1.06E+09	5.71E+08
sp|Q9RUN	sp|Q9RUN	242					17336000	
tr|Q9RY43	tr|Q9RY43	1693	95273000		98579000	56038000	53901000	
tr|Q9RYW	tr|Q9RYW	1830	78796000	66217000	79054000	86556000	73921000	90310000
tr|Q9RZD1	tr|Q9RZD1	1907				40825000	39988000	
tr|Q9RYM	tr|Q9RYM	1773	1.19E+08	94854000	79539000	66876000	39733000	66163000
tr|Q9RYM	tr|Q9RYM	1773	38989000	57525000	1E+08		45749000	40690000
tr|Q9RY62	tr|Q9RY62	1700						
sp|Q9RXB	sp|Q9RXB	365						
tr|Q9RS41	tr|Q9RS41	641						
tr|Q9RT97	tr|Q9RT97	843	2.02E+08	1.85E+08	2.11E+08	2.48E+08	2.01E+08	1.97E+08
tr|Q9RRM	tr|Q9RRM	548	81195000	98015000	89359000	30037000	40550000	41290000
tr|Q9RRC0	tr|Q9RRC0	497						
sp|Q9RS3	sp|Q9RS3	110	2.62E+08	2.79E+08	2.5E+08	2.91E+08	2.59E+08	2.39E+08
tr|Q9RSE2	tr|Q9RSE2	701	4.89E+08	4.67E+08	3.85E+08	3.51E+08	2.65E+08	3.14E+08
sp|Q9RW2	sp|Q9RW2	307						
sp|Q9RW0	sp|Q9RW0	327		11844000	19860000			
sp|Q9RW0	sp|Q9RW0	327	25525000	62143000	1.18E+08	26465000	59467000	49652000
sp|P56867	sp|P56867	56	1.31E+09	1.6E+09	1.68E+09	63680000	1.52E+09	1.82E+09
sp|P56867	sp|P56867	56			59834000	3.23E+08	3.34E+08	2.52E+08
tr|Q9RTG7	tr|Q9RTG7	873	1.04E+09	1.2E+09	9.1E+08	1.12E+09	1.09E+09	1.14E+09
sp|P56867	sp|P56867	56	9.03E+09	1.06E+10	9.46E+09	7.85E+09	9.7E+09	6.71E+09

sp|P5686	sp|P56867	56			40525000			
tr|Q9RSH	tr|Q9RSH0	716			43659000			
tr|Q9RYE	tr|Q9RYE8	1740	1.53E+08	80224000	93010000	35387000	50661000	40197000
sp|Q9RW	sp|Q9RW7	314					71413000	74423000
sp|Q9RR9	sp|Q9RR9	73	45253000	1.15E+08	1.19E+08	74299000	1E+08	63801000
tr|Q9RUL	tr|Q9RUL5	1086			19604000			
tr|Q9RSG	tr|Q9RSG3	712	1.02E+08	1.32E+08	1.09E+08	39312000	37949000	57270000
tr|Q9RSU	tr|Q9RSU0	762			71847000			
sp|P5692	sp|P56926	59						
sp|P5692	sp|P56926	59						6632500
tr|Q9RUA	tr|Q9RUA	1023	2.02E+09	2.23E+09	1.77E+09	1.31E+09	1.09E+09	1.26E+09
tr|Q9RUF	tr|Q9RUF4	1047	7169400	2362800				88989
tr|Q9RUF	tr|Q9RUF4	1047	24118000					
sp|Q9RUN	sp|Q9RUN	240	74821000	68059000	84182000			
tr|Q9RVA	tr|Q9RVA5	1202	1.85E+08	2.14E+08	1.98E+08	78346000	63823000	87362000
tr|Q9RX2	tr|Q9RX28	1516	6303300		7721700	16729000	15837000	16844000
tr|Q9RY7	tr|Q9RY79	1708	1.12E+08	1.02E+08	82829000	1.81E+08	1.44E+08	1.55E+08
tr|Q9RY7	tr|Q9RY79	1708	79990000	1.42E+08	1.91E+08	72438000	84000000	88499000
tr|Q9RXX	tr|Q9RXX2	1660	99396000	1.15E+08	1.26E+08			
sp|Q9RW	sp|Q9RWH	335			1.14E+08			
sp|Q9RSG	sp|Q9RSG	118	12061000		37402000		15572000	15163000
sp|Q9RZ8	sp|Q9RZ8	453	6.38E+09	5.92E+09	5.04E+09	62134000	95011000	1.71E+08
sp|Q9RZ8	sp|Q9RZ8	453	1.8E+08	5.83E+08	9.6E+08			
tr|Q9RUZ	tr|Q9RUZ6	1147		16269000		44516000	27758000	
tr|Q9RXS1	tr|Q9RXS1	1638		3037300	3792500	6805000	8626000	19388000
tr|Q9RS2	tr|Q9RS29	636				2980500	4012600	3588500
tr|Q9RZT	tr|Q9RZT3	1970	88237000	1.62E+08	1.06E+08	2.51E+08	2.49E+08	2.14E+08
sp|Q9RV	sp|Q9RVW	301	5.52E+08	4.69E+08	5.38E+08	41534000	59353000	66919000
sp|Q9RY	sp|Q9RYM	438	2.03E+09	3.3E+09	4.48E+09	3.04E+09	2.73E+09	3.51E+09
sp|Q9RXF	sp|Q9RXF	369		31030000	26083000	35639000	28386000	32281000
sp|Q9RW	sp|Q9RW0	326	1.68E+08	1.61E+08	1.63E+08	1.34E+08	1.41E+08	1.51E+08
sp|Q9RW	sp|Q9RW0	326	2.83E+08	4.06E+08	8.25E+08	4.47E+08	6.05E+08	4.88E+08
tr|Q9RT11	tr|Q9RT11	799	52633000		37753000			
sp|Q9RT3	sp|Q9RT3	166	1.79E+08	1.49E+08	1.43E+08	2.42E+08	2.3E+08	
sp|P5215	sp|P52153	52	16424000	16276000	19468000		11340000	
tr|Q9RSP7	tr|Q9RSP7	744	1.32E+08	1.99E+08	1.48E+08	2.52E+08	2.58E+08	2.32E+08
tr|Q9RZP	tr|Q9RZP9	1956						
sp|Q9RU2	sp|Q9RU2	209			1.06E+09	6.72E+09	1.6E+09	8.95E+09
tr|Q9RT87	tr|Q9RT87	837	2853200	57047000	44450000	18343000	16957000	53718000
tr|Q9RT87	tr|Q9RT87	837	3.26E+09	2.88E+09	2.6E+09	1.85E+09	1.26E+09	1.3E+09
tr|Q9RRP	tr|Q9RRP0	555			6760000			
sp|Q9RUL	sp|Q9RUL	239				70076000	62997000	
tr|Q9RRN	tr|Q9RRN	551			6979300			
tr|Q9RWQ	tr|Q9RWQ	1455	76870000	4.24E+08	3.96E+08	5.97E+08	2.24E+08	2.47E+08
sp|Q9RXK	sp|Q9RXK	386						
sp|Q9RXK	sp|Q9RXK	386			1.26E+08	88410000		1.13E+08
tr|Q9RXG	tr|Q9RXG3	1589						
sp|Q9RZN	sp|Q9RZN	457	27005000	53098000	40596000	1.56E+08	65639000	47864000
sp|Q9RXJ	sp|Q9RXJ7	382	4.31E+09	4.51E+09	4.01E+09	1.88E+09	1.58E+09	1.85E+09
tr|Q9RX8	tr|Q9RX85	1550						
tr|Q9RVX	tr|Q9RVX7	1318				26765000	30569000	25886000

tr|Q9RST2	tr|Q9RST2	758	27365000	27695000	43159000			
tr|Q9RUD	tr|Q9RUD	1041			5439100	2557000	8877600	
tr|Q9RSX4	tr|Q9RSX4	779	1.26E+08	1.21E+08	93595000	35454000	29716000	38851000
tr|Q9RR9	tr|Q9RR98	481	3.22E+08	2.9E+08	3.2E+08	1.8E+08	1.71E+08	2.09E+08
sp|Q9RTG	sp|Q9RTG	184			23833000			
sp|Q9RTT	sp|Q9RTT	200		25061000	23484000			
tr|Q9RXD	tr|Q9RXD5	1573	2.11E+08	1.77E+08	1.46E+08	1.66E+08	1.49E+08	1.42E+08
tr|Q9RU2	tr|Q9RU27	980	1.29E+08	1.45E+08	1.69E+08		50779000	
tr|Q9RXZ4	tr|Q9RXZ4	1670		2.89E+08				3.23E+08
tr|Q9RRH	tr|Q9RRH	528	40512000		29621000			28700000
tr|Q9RZF5	tr|Q9RZF5	1922	29030000	30934000	28070000	95680000	51674000	53173000
tr|Q9RW8	tr|Q9RW8	1367	36433000	41697000	97771000		57680000	32336000
sp|Q9RUP	sp|Q9RUP	244	2.15E+08	1.83E+08	2.35E+08	84928000	90222000	1.14E+08
sp|Q9RV5	sp|Q9RV5	265	1.16E+09	8.89E+08	9.58E+08	4.01E+08	3.81E+08	6.91E+08
sp|Q9RV5	sp|Q9RV5	265			21269000			
tr|Q9RSL8	tr|Q9RSL8	734			45189000	17475000	20755000	31144000
tr|Q9RVQ	tr|Q9RVQ	1275				17042000		
sp|Q9RS6	sp|Q9RS6	112	7.22E+08	7.47E+08	4.96E+08	2.55E+08	2.72E+08	2.3E+08
tr|Q9RYT4	tr|Q9RYT4	1813	33639000	22537000	50728000	36025000	24893000	
tr|Q9RV0	tr|Q9RV06	1154	1.47E+08	1.47E+08	1.31E+08	72868000	72426000	67624000
tr|Q9RR71	tr|Q9RR71	470	2.99E+08	3.58E+08	3.18E+08	3.99E+08	3.8E+08	3.51E+08
tr|Q9RWV	tr|Q9RWV	1485	9.13E+08	9.34E+08	8.44E+08	6.91E+08	5.85E+08	5.97E+08
tr|Q9RU9	tr|Q9RU92	1016						
sp|Q9RVF	sp|Q9RVF	286	57333000	45390000	43889000	32101000	52173000	46204000
tr|Q9RSZ3	tr|Q9RSZ3	789						
sp|Q9RSZ	sp|Q9RSZ	160	9899600	4023500	4420600	856970	1516900	2513900
tr|Q9RVW	tr|Q9RVW	1312				96555000		
sp|Q9RXK	sp|Q9RXK	386	2.32E+09	1.6E+09	1.8E+09			
tr|Q9RRM	tr|Q9RRM	548				19529000	25013000	21736000
sp|Q9RU2	sp|Q9RU2	208	3.57E+08	3.55E+08				
tr|Q9RTC1	tr|Q9RTC1	848						
sp|Q9RTF	sp|Q9RTF	182	7.85E+08	1.25E+09	1.04E+09	1.48E+08	3.93E+08	3.91E+08
tr|Q9RUJ2	tr|Q9RUJ2	1068	2.74E+08	2.68E+08	2.71E+08	2.69E+08	3.29E+08	2.52E+08
tr|Q9RUJ2	tr|Q9RUJ2	1068			61886000			
tr|Q9RWT	tr|Q9RWT	1465	6904800	3877700	3609800	3936000	6090900	3841700
tr|Q9RUK	tr|Q9RUK	1081	3.1E+08	2.7E+08	2.85E+08	5.09E+08	4.1E+08	5.19E+08
sp|Q9RRQ	sp|Q9RRQ	95	69237000		72512000	83162000	72372000	77312000
tr|Q9RRX	tr|Q9RRX0	600	50774000	49705000	49591000	13922000	20690000	29949000
tr|Q9RXS1	tr|Q9RXS1	1638	2.52E+08	2.41E+08	2.27E+08	78146000	62522000	81382000
tr|Q9RTP	tr|Q9RTP5	909				28717000		20219000
sp|O3250	sp|O3250	44	19981000		15907000			
tr|Q9RWB	tr|Q9RWB	1386	4.11E+08	3.9E+08	3.77E+08	1.01E+09	9.48E+08	8.65E+08
tr|Q9RWB	tr|Q9RWB	1386			33222000	34894000	48411000	36673000
tr|Q9RY8	tr|Q9RY88	1712		61802000	41641000		32244000	
tr|Q9RY8	tr|Q9RY88	1712	24937000	26760000	15274000			
tr|Q9RUF	tr|Q9RUF8	1049					27124000	16168000
tr|Q9RRH	tr|Q9RRH	524				24157000		19223000
tr|Q9RRH	tr|Q9RRH	524	28093000					
tr|Q9RY8	tr|Q9RY86	1710	1.95E+08	2.48E+08	1.81E+08	5.9E+08	5.84E+08	4.66E+08
tr|Q9RY8	tr|Q9RY86	1710				11903000	13998000	
tr|Q9RWC	tr|Q9RWC	1388	8.56E+08	7.98E+08	7.41E+08	3.44E+08	3.59E+08	3.83E+08

sp|Q9RT3	sp|Q9RT3	166	1.35E+09	2.26E+09	1.9E+09	2.9E+09	2.75E+09	3.01E+09
tr|Q9RY04	tr|Q9RY04	1677						
tr|Q9RUJ9	tr|Q9RUJ9	1074						
tr|Q9RVU	tr|Q9RVU	1299			3737800	14576000	8831800	
tr|Q9RV8	tr|Q9RV8	1194						
tr|Q9RZD	tr|Q9RZD	1907			9618000			
tr|Q9RZE	tr|Q9RZE6	1916						
tr|Q9RZE	tr|Q9RZE6	1916	96738000	81122000	1.71E+08		46729000	69427000
tr|Q9RWU	tr|Q9RWU	1474	2.01E+08	2.75E+08	2.43E+08	64163000	1.59E+08	63858000
tr|Q9RUU	tr|Q9RUU	1123						1847500
tr|Q9RTM	tr|Q9RTM	900						
tr|Q9RUP	tr|Q9RUP	1098	2.1E+08		16694000	1.31E+09	8.14E+08	7.48E+08
sp|P5202	sp|P5202	51	71589000	78898000	26857000	28604000		26717000
tr|Q9RYC	tr|Q9RYC	1729						
tr|Q9RRG	tr|Q9RRG	522		46915000	45362000	27086000		31909000
tr|Q9RVL	tr|Q9RVL	1250	44270000	33504000	50542000	21727000	29557000	44887000
tr|Q9RW4	tr|Q9RW4	1353		9401400	7412000	18888000	17334000	18172000
tr|Q9RW3	tr|Q9RW3	1349	45303000		1.03E+08	1.33E+08	1.2E+08	1.33E+08
tr|Q9RZ2	tr|Q9RZ27	1857						
tr|Q9RRF	tr|Q9RRF	514	59741000	58256000	47682000	1.1E+08	71884000	91371000
tr|Q9RRF	tr|Q9RRF	514	7.9E+08	7.29E+08	8.94E+08	1.74E+08	2.51E+08	2.86E+08
tr|Q9RXB	tr|Q9RXB	1566	19086000	13260000	35131000	41422000	26212000	33154000
tr|Q9RWF	tr|Q9RWF	1403		1.78E+08	2.1E+08	1.77E+08	1.11E+08	
tr|Q9RSG	tr|Q9RSG	711	47910000		24872000		28056000	
sp|Q9RTK	sp|Q9RTK	191	1.69E+08		1.35E+08	2.57E+08		2.51E+08
tr|Q9RUT	tr|Q9RUT	1116	14761000	23743000	23242000			
sp|Q9RW	sp|Q9RW	343	3.08E+08	3.24E+08	2.64E+08	3.76E+08	3.57E+08	4.09E+08
sp|Q9RW	sp|Q9RW	343				8688600	12772000	11086000
sp|Q9RT4	sp|Q9RT4	167						
sp|Q9RV8	sp|Q9RV8	270	1E+08	1.53E+08	1.08E+08	1.99E+08	1.8E+08	1.99E+08
tr|Q9RXQ	tr|Q9RXQ	1628						
sp|Q9RV9	sp|Q9RV9	272	3.39E+08	3.51E+08	2.81E+08	4.07E+08	3.27E+08	3.07E+08
tr|Q9RZJ2	tr|Q9RZJ2	1939				26308000	20709000	26771000
sp|Q9RSA	sp|Q9RSA	114	1.16E+09	1.37E+09	1.36E+09	1.36E+09	1.1E+09	1.47E+09
sp|Q9RY	sp|Q9RY	438	4.86E+08	2.1E+08	8.42E+08	1.01E+09	5.52E+08	7.76E+08
tr|Q9RX7	tr|Q9RX76	1544						20632000
sp|P8358	sp|P8358	60			21859000			
sp|Q9RTF	sp|Q9RTF	183	90560000	1.14E+08	73921000	82929000		75378000
sp|Q9RTJ	sp|Q9RTJ	190						
tr|Q9RZ0	tr|Q9RZ0	1847	33751000	16307000	25438000	45939000	82216000	61188000
tr|Q9RVU	tr|Q9RVU	1299			22783000	33512000	38147000	26195000
tr|Q9RSJ0	tr|Q9RSJ0	726	2.51E+08	1.12E+09	1.61E+08	8.64E+08	2.67E+08	8.88E+08
tr|Q9RZP	tr|Q9RZP7	1955	5.95E+08	5.55E+08	4.81E+08	3.82E+08	3.26E+08	3.42E+08
tr|Q9RVQ	tr|Q9RVQ	1272	45831000		39295000	25306000	37447000	28826000
tr|Q9RUC	tr|Q9RUC	1033	1.31E+08	87570000	1.05E+08	2.27E+08	2.06E+08	60151000
tr|Q9RUC	tr|Q9RUC	1033			63991000			
tr|Q9RW8	tr|Q9RW8	1366	1.01E+08	91715000	98871000	1.32E+08	1.06E+08	1.18E+08
tr|Q9RV11	tr|Q9RV11	1156			2414800	16400000		2906400
sp|P5692	sp|P56926	59	36266000	47890000	51131000			
tr|Q9RVA	tr|Q9RVA	1199						
tr|Q9RYQ	tr|Q9RYQ	1791	1.33E+08	79414000	80071000	2.59E+08	2.19E+08	2.31E+08

sp|Q9RW(	sp|Q9RW(	342	6987500		33648000	1.52E+08	73947000	1.19E+08
tr|Q9RV11	tr|Q9RV11	1156	10764000	21654000	9178500	20164000	17209000	27810000
tr|Q9RV11	tr|Q9RV11	1156	11840000	19519000	8701400		13484000	13498000
tr|Q9RV11	tr|Q9RV11	1156			62443000			19441000
tr|Q9RT02	tr|Q9RT02	793	85523000	94229000	1.15E+08	80602000	1.52E+08	1.04E+08
sp|Q9RRX	sp|Q9RRX	101	34271000	14200000	16265000			
tr|Q9RTX7	tr|Q9RTX7	952	2.08E+08	2.31E+08	2.65E+08			14779000
tr|Q9RU5:	tr|Q9RU52	995	1.78E+09	3.08E+08	1.32E+09	4.38E+08	3.73E+08	1.65E+09
tr|Q9RT97	tr|Q9RT97	843					45955000	47669000
sp|Q9RW!	sp|Q9RW9	318	1.3E+08	1.08E+08	74738000	1.9E+08	1.04E+08	1.55E+08
tr|Q9RT24	tr|Q9RT24	803	2.57E+08	2.76E+08	2.32E+08			
tr|Q9RXU(	tr|Q9RXU(	1643					30046000	
tr|Q9RXU(	tr|Q9RXU(	1643	5.85E+08	5.31E+08	9.27E+08	4.75E+08	8.49E+08	5.69E+08
tr|Q9RRP!	tr|Q9RRP5	559						
tr|Q9RRP!	tr|Q9RRP5	559		5.7E+08	4.78E+08	3.87E+08	3.54E+08	4.07E+08
tr|Q9RVR!	tr|Q9RVR5	1284						
tr|Q9RUU	tr|Q9RUU	1121	1.05E+09	5.88E+08	9.86E+08	9.98E+08	1.13E+09	1.05E+09
tr|Q9RSE4	tr|Q9RSE4	703	6.44E+08	4.87E+08	5.81E+08	28162000	56148000	56150000
sp|Q9RU8	sp|Q9RU8	221	28607000			63105000	43644000	53641000
sp|Q9RU8	sp|Q9RU8	221		3.26E+08	2.81E+08	8.05E+08	5.8E+08	6.91E+08
tr|Q9RTS4	tr|Q9RTS4	926		48632000	28255000			
sp|Q9RRQ	sp|Q9RRQ	95	1.45E+09	9.03E+08	1.2E+09	8.36E+08	8.08E+08	8.36E+08
tr|Q9RTX5	tr|Q9RTX5	950	6367700		14119000			
sp|Q9X71	sp|Q9X71	462	6.12E+08	5.9E+08	4.56E+08	5E+08	4.24E+08	5.02E+08
sp|Q9RXJ7	sp|Q9RXJ7	382	5.88E+09	5.72E+09	2.2E+09	3.53E+09	6.86E+09	7.25E+09
tr|Q9RV3€	tr|Q9RV36	1169	1.99E+08	2.33E+08	3.43E+08	1.13E+08	1.38E+08	1.7E+08
tr|Q9RYC€	tr|Q9RYC6	1727						
sp|Q5933	sp|Q5933	63	1.46E+08	71224000	68141000	34466000	60191000	59222000
sp|Q9RT0	sp|Q9RT0	161	1.23E+08	48003000	36860000	1.63E+08	1.56E+08	1.66E+08
sp|Q9RT0	sp|Q9RT0	161			2.03E+08	14297000		
tr|Q9RZT7	tr|Q9RZT7	1974	1.97E+08	1.72E+08		1.2E+08		2.38E+08
tr|Q9RZT7	tr|Q9RZT7	1974		57038000	62718000			
tr|Q9RRR(	tr|Q9RRR(	567	66003000	51111000	98759000			1.56E+08
tr|Q9RV4€	tr|Q9RV46	1173	15876000	22175000	27939000	30947000		40694000
tr|Q9RUG	tr|Q9RUG	1055				9593100		
tr|Q9RXG!	tr|Q9RXG!	1592				45921000	28209000	30526000
tr|Q9RTZ(	tr|Q9RTZ(	961	5.87E+08	1.25E+09	5.41E+08	2.45E+09	2.28E+09	2.61E+09
tr|Q9RTZ(	tr|Q9RTZ(	961		20732000				
tr|Q9RTZ(	tr|Q9RTZ(	961		61868000	80166000			
tr|Q9RTV€	tr|Q9RTV6	939	4.89E+08	4.22E+08	3.48E+08	3.95E+08	3.75E+08	3.94E+08
sp|Q9RS2	sp|Q9RS2	108	1.88E+08	1.51E+08	1.07E+08	3.36E+08	2.95E+08	3.01E+08
sp|Q9RS2	sp|Q9RS2	108	4.17E+08	3.62E+08	2.84E+08	2.75E+08	2.34E+08	2.97E+08
tr|Q9RRE€	tr|Q9RRE6	512	22852000					
tr|Q9RYI0	tr|Q9RYI0	1759						
tr|Q9RRZ7	tr|Q9RRZ7	616	18831000	18476000	17468000		19112000	23290000
tr|Q9RU9:	tr|Q9RU92	1016			2.07E+08		42338000	69231000
tr|Q9RX57	tr|Q9RX57	1532	5.17E+08	5.03E+08	4.83E+08	5.61E+08	5.23E+08	5.59E+08
tr|Q9RX57	tr|Q9RX57	1532	37607000	72052000	30626000			
tr|Q9RY74	tr|Q9RY74	1704			1.05E+08		72751000	39994000
tr|Q9RVV4	tr|Q9RVV4	1307						
tr|Q9RYQ!	tr|Q9RYQ!	1795						

sp|Q9RUE	sp|Q9RUE	226	2.03E+08	3.71E+08	5.65E+08	2.36E+08	3.18E+08	3.3E+08
tr|Q9RZC0	tr|Q9RZC0	1900		20143000				
tr|Q9RSA6	tr|Q9RSA6	677			15416000	22028000	12616000	13575000
tr|Q9RTL4	tr|Q9RTL4	895	1.19E+08	1.27E+08	1.29E+08	2.17E+08	2.01E+08	1.87E+08
tr|Q9RY75	tr|Q9RY75	1705	27018000	28561000	29977000			
sp|Q9RXK	sp|Q9RXK	387	7.74E+08	7.27E+08	7.67E+08	1.81E+08	2.95E+08	3.19E+08
sp|Q9RXK	sp|Q9RXK	387						
tr|Q9RRA0	tr|Q9RRA0	483	1345000				472490	
tr|Q9RVN	tr|Q9RVN	1264				41837000	41103000	31213000
tr|Q9RUH	tr|Q9RUH	1059			50398000	53186000		
tr|Q9RRA6	tr|Q9RRA6	489						9409600
sp|Q9RTF	sp|Q9RTF	181			24277000		74205000	
tr|Q9RYN6	tr|Q9RYN6	1780	3E+08	4.45E+08	7.43E+08	9.88E+08	9.08E+08	5.2E+08
sp|Q9RT2	sp|Q9RT2	163				1.26E+08	1.04E+08	81787000
tr|Q9RU41	tr|Q9RU41	988	1.46E+08	2.59E+08	1.25E+08	1.44E+08	2.96E+08	3.02E+08
sp|Q9RSF	sp|Q9RSF	117	2136600	1119900	1157200	1233800	1859300	1868300
sp|Q9RUP	sp|Q9RUP	244	1.33E+09	1.45E+09	1.27E+09	2.34E+09	1.79E+09	2.06E+09
sp|Q9RRB	sp|Q9RRB	76	1.33E+08	1.62E+08	2.31E+08	1.12E+08	1.3E+08	1.37E+08
tr|Q9RXC5	tr|Q9RXC5	1570	1.56E+08	1.84E+08	1.35E+08			
tr|Q9RXH4	tr|Q9RXH4	1596	3.73E+08	3.54E+08	4.42E+08	2.02E+08	2.02E+08	4.11E+08
tr|Q9RVP5	tr|Q9RVP5	1269	5.12E+08	5.2E+08	7.62E+08	1.49E+08	1.85E+08	2.1E+08
sp|Q9RVT	sp|Q9RVT	296	29819000	27445000	24581000			
sp|O3250	sp|O3250	45	5.18E+08		4.93E+08	2.82E+08	3E+08	4.45E+08
tr|Q9RZC5	tr|Q9RZC5	1904	7483900	4133300	5180100	3264700	951220	807710
tr|Q9RUL2	tr|Q9RUL2	1084			11200000			
tr|Q9RRV1	tr|Q9RRV1	588	1.41E+09	7.99E+08	1.01E+09	5.16E+09	6E+09	5.59E+09
tr|Q9RRV1	tr|Q9RRV1	588	3.4E+08	2.29E+08	2.04E+08	5.72E+08	5.35E+08	5.63E+08
tr|Q9RVR4	tr|Q9RVR4	1283	47305000	51593000			52344000	
sp|Q9RXT	sp|Q9RXT	399						
tr|Q9RUK1	tr|Q9RUK1	1076	40494000	63689000	80388000			
sp|Q9RWE	sp|Q9RWE	320	3.88E+09	4.05E+09	3.87E+09	3.7E+09	3.77E+09	3.73E+09
sp|Q9RWE	sp|Q9RWE	320	1.61E+08	2.34E+08	3.69E+08	1.18E+08	1.54E+08	1.42E+08
tr|Q9RYI7	tr|Q9RYI7	1763	73864000	1.04E+08	70852000	1.44E+08	1.62E+08	1.69E+08
tr|Q9RR99	tr|Q9RR99	482	76820000	61234000	59905000	22785000	19619000	30353000
tr|Q9RVP5	tr|Q9RVP5	1269				7583400		
sp|Q9RXT4	sp|Q9RXT4	400	89127000	1.52E+08	1.1E+08	72263000	55620000	81738000
tr|Q9RUU	tr|Q9RUU	1124	1926100	8755500	17179000	2651300		4593900
tr|Q9RT93	tr|Q9RT93	839	30771000	19439000	19072000	10927000		14849000
sp|Q9RZU	sp|Q9RZU	458						
tr|Q9RVS1	tr|Q9RVS1	1287	1787600	1193500	837580	666680	604080	866290
tr|Q9RVS1	tr|Q9RVS1	1287			31780000			
tr|Q9RUI4	tr|Q9RUI4	1063	33272000	28891000			34161000	37208000
sp|Q9RTQ	sp|Q9RTQ	197	2.25E+08	2.03E+08	2.37E+08	1.84E+08	1.9E+08	2E+08
tr|Q9RU46	tr|Q9RU46	993				16013000		
tr|Q9RUJ6	tr|Q9RUJ6	1072	37646000					17237000
tr|Q9RUJ6	tr|Q9RUJ6	1072	96239000	87358000	63162000	88787000	61262000	79133000
tr|Q9RW4	tr|Q9RW4	1351	28545000	21223000				
tr|Q9RVK9	tr|Q9RVK9	1249						
sp|Q9RV5	sp|Q9RV5	265	7729100	6218200	6429200	6977500	6760500	7101800
tr|Q9RXS5	tr|Q9RXS5	1639	27443000		39500000			
tr|Q9RS90	tr|Q9RS90	666	1.05E+08	1.04E+08	1.04E+08	1.42E+08		30510000

sp|Q9RXP	sp|Q9RXP	393	2033600	1516600	2229300	1054700	1378200	1473200
tr|Q9RZ10	tr|Q9RZ10	1848	30195000	29877000	34405000	35456000	41708000	58556000
tr|Q9RYU9	tr|Q9RYU9	1819	1.03E+08	1.3E+08	88593000	2.12E+08	1.59E+08	1.7E+08
tr|Q9RTA3	tr|Q9RTA3	845	8673800	41627000	51384000	93794000	1.18E+08	44288000
sp|Q9RW1	sp|Q9RW1	306	5.85E+08	6.02E+08	82008000	1.28E+09	1.17E+09	9.92E+08
sp|Q9RW1	sp|Q9RW1	306	3.27E+08	3.58E+08	3.49E+08	1.1E+08	74562000	1.85E+08
sp|Q9RWN	sp|Q9RWN	336	1.48E+08	1.43E+08	2.4E+08			1.09E+08
tr|Q9RRW	tr|Q9RRW	597	2.1E+09	2.3E+09	2E+09	1.7E+09	1.41E+09	1.89E+09
tr|Q9RRE7	tr|Q9RRE7	513	8767200	7886900	9527600	3898700	6208300	
tr|Q9RRV1	tr|Q9RRV1	588					811980	
tr|Q9RRV1	tr|Q9RRV1	588	45535000	49456000	1.2E+08	1.05E+08	98417000	1.45E+08
tr|Q9RVF1	tr|Q9RVF1	1221			10903000			
tr|Q9RRP4	tr|Q9RRP4	558	2.31E+09	1.86E+09	1.54E+09	1.35E+09	1.52E+09	1.45E+09
tr|Q9LBE3	tr|Q9LBE3	466	52727000	40120000	42777000	26556000	13762000	
sp|Q9RT2	sp|Q9RT2	162			4875900	8837200	14421000	11754000
sp|Q9RWD	sp|Q9RWD	323			12966000	21355000		
tr|Q9RVN	tr|Q9RVN	1265						
sp|Q9RTG	sp|Q9RTG	184		73314000	15129000	2.37E+08	1.59E+08	1.59E+08
sp|Q9RX2	sp|Q9RX2	353	75203000	70266000	45048000	47337000	34020000	
tr|Q9RUX5	tr|Q9RUX5	1134	30827000	44629000	36399000	25643000	39744000	27927000
tr|Q9RVX7	tr|Q9RVX7	1318			16186000	18446000		29042000
tr|Q9RSW	tr|Q9RSW	774						
tr|Q9RUT9	tr|Q9RUT9	1120	19199000	18367000		59516000	51490000	52026000
sp|Q9RW9	sp|Q9RW9	345	68116000	50808000	57351000	1.09E+08	1.21E+08	1.01E+08
sp|Q9RY0	sp|Q9RY0	410	5.38E+08	5.81E+08	5.65E+08	1.13E+09	1.16E+09	1.26E+09
tr|Q9RUN	tr|Q9RUN	1097	93001000	1.1E+08	96886000	2.36E+08	2.25E+08	1.94E+08
sp|Q9RRB	sp|Q9RRB	76						
tr|Q9RVE5	tr|Q9RVE5	1219	36966000	70326000	44461000			
tr|Q9RS93	tr|Q9RS93	668	25987000	2.27E+08	1.7E+08	24717000	21050000	27549000
tr|Q9RS93	tr|Q9RS93	668	4.59E+08	66828000	52408000	48455000	48701000	2.68E+08
tr|Q9RVA7	tr|Q9RVA7	1203		3.18E+08	2.37E+08		1.76E+08	
tr|Q9RS71	tr|Q9RS71	655	1.55E+09	1E+09	7.57E+08	7.91E+08	7.86E+08	7.61E+08
tr|Q9RU67	tr|Q9RU67	1007	50491000	49019000	41966000	74505000	44044000	61256000
tr|Q9RU67	tr|Q9RU67	1007		39184000	37214000			
tr|Q9RW2	tr|Q9RW2	1344	1.37E+09	1.16E+09	8.26E+08	1.23E+09	1.3E+09	1.3E+09
sp|Q9RU7	sp|Q9RU7	213	39010000	36397000	42801000	42432000	43375000	46276000
tr|Q9RUL0	tr|Q9RUL0	1082	4.4E+08	3.5E+08	3.01E+08	1.11E+08	1.18E+08	1.43E+08
tr|Q9RYS1	tr|Q9RYS1	1801	1.26E+08	1.78E+08	1.61E+08	1.31E+08	1.05E+08	1.25E+08
tr|Q9RZE9	tr|Q9RZE9	1918	9583400	13258000	8894300	14196000	12678000	
tr|Q9RZE9	tr|Q9RZE9	1918	85893000	97286000	91555000			
tr|Q9RW8	tr|Q9RW8	1372			3616200			
sp|Q9RRE	sp|Q9RRE	81	1.48E+08	1.31E+08	89818000			
sp|Q9RRE	sp|Q9RRE	81			32483000		29965000	24819000
sp|Q9RS1	sp|Q9RS1	106	71475000	56906000	71274000	73197000	74499000	1.04E+08
sp|Q9RXH	sp|Q9RXH	372						
tr|Q9RX56	tr|Q9RX56	1531	4.16E+08	5.54E+08	6.24E+08	1.84E+09	1.5E+09	1.41E+09
tr|Q7DF71	tr|Q7DF71	464						
tr|Q9RW0	tr|Q9RW0	1331	34018000	39431000	28918000	26811000	25500000	55400000
tr|Q9RW0	tr|Q9RW0	1332	4.96E+08	1.54E+09	1.37E+09	2.67E+09	1.79E+09	2.38E+09
sp|Q9RXI7	sp|Q9RXI7	373						
tr|Q9RRV7	tr|Q9RRV7	592	33608000	16798000	12646000	31363000	22148000	34642000

tr|Q9RYE5	tr|Q9RYE5	1739		21796000		25369000	20154000	
tr|Q9RYT6	tr|Q9RYT6	1815				35945000	35330000	51400000
sp|Q9RXK	sp|Q9RXK	385				1.43E+08	1.7E+08	1.37E+08
tr|Q9RWA	tr|Q9RWA	1381	12550000	5252200	5102300	2340300	3567400	2929600
sp|Q9RY2	sp|Q9RY2	411						
tr|Q9RYN5	tr|Q9RYN5	1779	4.9E+08	3.58E+08	4.89E+08	5.56E+08	5.7E+08	6.11E+08
tr|Q9RSM	tr|Q9RSM	735	63739000	69789000	56907000	68055000	85281000	63118000
tr|Q9RWV	tr|Q9RWV	1487						
tr|Q9RXW	tr|Q9RXW	1658	5.72E+08	6.21E+08	4.69E+08		5.02E+08	4.35E+08
tr|Q9RTQ5	tr|Q9RTQ5	915	24825000	19634000	20104000	35293000		26803000
sp|Q9RTQ	sp|Q9RTQ	197	1.02E+09	1.04E+09	9.06E+08	8.58E+08	7.31E+08	7.76E+08
sp|Q9RWU	sp|Q9RWU	347	79892000		47817000	72464000		56645000
tr|Q9RSE7	tr|Q9RSE7	705	1.68E+08	2.31E+08	1.73E+08	92243000	93398000	1.7E+08
sp|Q9RW6	sp|Q9RW6	311	5.68E+08	6.03E+08	1.87E+08	3.66E+08	3.68E+08	4.54E+08
sp|Q9RSR	sp|Q9RSR	143				80676000		70144000
tr|Q9RYD0	tr|Q9RYD0	1730	35143000	20375000	24940000			1.06E+08
tr|Q9RSJ1	tr|Q9RSJ1	727						
tr|Q9RVK9	tr|Q9RVK9	1249		3657800	3182700		3796600	
tr|Q9RVU2	tr|Q9RVU2	1300	1.08E+08	92350000	99175000	1.43E+08	1.31E+08	1.43E+08
tr|Q9RXE7	tr|Q9RXE7	1578						
tr|Q9RXE7	tr|Q9RXE7	1578			16200000		12187000	14687000
tr|Q9RX53	tr|Q9RX53	1529						
sp|Q9RTF5	sp|Q9RTF5	182	58159000	1.05E+08	98152000		49678000	
sp|Q9RRJ1	sp|Q9RRJ1	86	40450000		37853000			9641800
tr|Q9RYB8	tr|Q9RYB8	1722		20298000	34040000		47929000	
tr|Q9RU70	tr|Q9RU70	1009			19098000			
tr|Q9RS77	tr|Q9RS77	658						
tr|Q9RSF8	tr|Q9RSF8	710			1706500			
tr|Q9RYM	tr|Q9RYM	1774	8614500				25661000	
sp|Q9RUP	sp|Q9RUP	246		1.48E+08	1.36E+08			
tr|Q9RS17	tr|Q9RS17	630	2.29E+08	1.62E+08	1.66E+08	4.18E+08	3.46E+08	3.72E+08
tr|Q9RWW	tr|Q9RWW	1493	2.42E+08	2.17E+08	1.86E+08	99462000	1.01E+08	1.3E+08
tr|Q9RYQ4	tr|Q9RYQ4	1792	35866000	24046000	48868000	60162000	72640000	47247000
tr|Q9RXH4	tr|Q9RXH4	1596	1.15E+08	74313000	82948000	86195000	1.06E+08	1.19E+08
tr|Q9RRV4	tr|Q9RRV4	591	17402000	26200000	28398000	44463000	45512000	46548000
tr|Q9RRV4	tr|Q9RRV4	591				16311000		
tr|Q9RXL6	tr|Q9RXL6	1612	1.45E+08	1.78E+08	1.32E+08	51057000	79450000	57551000
sp|Q9RT6	sp|Q9RT6	169			11620000		5031300	7113100
tr|Q9RWL	tr|Q9RWL	1431	1.14E+08	1.16E+08	95959000	1.31E+08	1.4E+08	1.22E+08
tr|Q9RT28	tr|Q9RT28	805					336950	
sp|Q9RYR	sp|Q9RYR	442	63766000	42700000	32656000	53430000	29526000	70924000
tr|Q9RZE9	tr|Q9RZE9	1918	81903000	76735000	71912000	1.72E+08	2.01E+08	1.77E+08
tr|Q9RUD	tr|Q9RUD	1043	5.32E+08	5.24E+08	6.3E+08	6.32E+08	6.55E+08	6.5E+08
tr|Q9RW8	tr|Q9RW8	1367		29704000	17587000		22300000	
tr|Q9RXQ	tr|Q9RXQ	1627	3E+08	4.45E+08	2.94E+08	3.19E+08	4.12E+08	2.9E+08
sp|Q9RSP	sp|Q9RSP	139	22928000	33723000	30029000	31689000	49092000	27204000
tr|Q9RS17	tr|Q9RS17	630	1.19E+09	1.24E+09	1.18E+09	5.75E+08	4.8E+08	6.15E+08
tr|Q9RXT6	tr|Q9RXT6	1641	50788000	48447000	43079000	57270000	73782000	73060000
tr|Q9RV45	tr|Q9RV45	1172	9.92E+08	8.57E+08	1.2E+09		60671000	57406000
tr|Q9RVX7	tr|Q9RVX7	1318	93313000	94270000	91289000			
tr|Q9RVX7	tr|Q9RVX7	1318	73520000	1.25E+08	1.46E+08	41557000	75370000	89266000

tr|Q9RY7	tr|Q9RY7	1708						
tr|Q9RWI	tr|Q9RWI	1416	57921000	82466000	94807000	1.7E+08	1.45E+08	1.93E+08
sp|Q9R34	sp|Q9R34	64	23031000	32313000	38824000	91397000	61594000	46958000
tr|Q9RZF2	tr|Q9RZF2	1920						
tr|Q9RUD	tr|Q9RUD	1041	35177000	46265000	35696000			
tr|Q9RRI7	tr|Q9RRI7	534			41394000	1.28E+08	90482000	1.37E+08
sp|Q9RY3	sp|Q9RY3	413				21570000	25289000	24106000
tr|Q9RSL6	tr|Q9RSL6	732					27307000	26101000
tr|Q9RST1	tr|Q9RST1	757	4.79E+08	4.87E+08	3.5E+08	3.9E+08	4.14E+08	4.23E+08
sp|O3250	sp|O3250	45	1.09E+08	1.62E+08	1.28E+08	6.59E+08	6.19E+08	5.31E+08
tr|Q9RST7	tr|Q9RST7	760			12005000			
tr|Q9RXL4	tr|Q9RXL4	1610			47977000	99113000	1.17E+08	1.13E+08
tr|Q9RY22	tr|Q9RY22	1683	81767000	95394000	62515000	68989000		
sp|Q9RUC	sp|Q9RUC	233					24773000	31531000
sp|Q9RUC	sp|Q9RUC	233	1.75E+08	1.45E+08	1.61E+08		48099000	39826000
sp|Q9RSI	sp|Q9RSI	121	41739000	40680000	29425000	37119000	43718000	35544000
sp|Q5933	sp|Q5933	63	32666000	16299000	23320000	9657400	9063100	11368000
tr|Q9RWX	tr|Q9RWX	1497	2.76E+08	1.75E+08	2.73E+08	89873000	53993000	79265000
tr|Q9RSE7	tr|Q9RSE7	705	58908000	62796000	61957000	57295000	67634000	88292000
sp|Q9RU2	sp|Q9RU2	209	5.68E+09	4.24E+09	6.07E+09	4.21E+09	2.41E+09	4.42E+09
tr|Q9RRH	tr|Q9RRH	524		6498900				
tr|Q9RWS	tr|Q9RWS	1464	41643000	45621000	35505000	44484000	35438000	35893000
tr|Q9RS76	tr|Q9RS76	657	5.25E+09	5.05E+09	5.04E+09	6.72E+09	6.66E+09	6.22E+09
tr|Q9RS76	tr|Q9RS76	657	1.05E+08	67053000	1.34E+08	30622000	45141000	49897000
sp|Q9RSR	sp|Q9RSR	143			12814000			
sp|Q9RTL	sp|Q9RTL	194	22013000	32967000	31075000	70759000	74947000	46092000
tr|Q9RUT	tr|Q9RUT	1118		13332000				
tr|Q9RUA	tr|Q9RUA	1023						22375000
sp|Q9RZ0	sp|Q9RZ0	452						
tr|Q9RVV	tr|Q9RVV	1307	23211000	14135000				
sp|Q9RUE	sp|Q9RUE	225			38080000		39797000	
tr|Q9RWC	tr|Q9RWC	1393		12497000				
sp|Q9RXK	sp|Q9RXK	388	1.06E+09	2.77E+08	4.91E+08	2.29E+08	1.61E+08	1.89E+08
tr|Q9RX42	tr|Q9RX42	1523	2.94E+08	3.11E+08	2.38E+08	2.77E+08	2.84E+08	3.08E+08
sp|Q9RXJ	sp|Q9RXJ	384				54550000	27098000	80546000
tr|Q9RWV	tr|Q9RWV	1489						
tr|Q9RZI5	tr|Q9RZI5	1936			40901000	42040000	39666000	
sp|Q9RWI	sp|Q9RWI	339	80269000	54928000	58642000		14843000	
sp|Q9RXK	sp|Q9RXK	389						
tr|Q9RYC	tr|Q9RYC	1726			19140000	12061000	23359000	19418000
tr|Q9RUR	tr|Q9RUR	1108	6.75E+08	6.53E+08	5.51E+08	4.37E+08	4.32E+08	4.61E+08
tr|Q9RV52	tr|Q9RV52	1177	91984000	81729000	55704000	99065000	72320000	92540000
tr|Q9RV52	tr|Q9RV52	1177	29171000	22355000				
tr|Q9RVF8	tr|Q9RVF8	1225	6196400	3455700	2867100	1811800	2699800	2148100
tr|Q9RX80	tr|Q9RX80	1546	546990		278680			
tr|Q9RZ28	tr|Q9RZ28	1858						
tr|Q9RWH	tr|Q9RWH	1410		57325000		29961000		28455000
tr|Q9RS08	tr|Q9RS08	624			2.43E+08	40299000	36419000	
tr|Q9RXE	tr|Q9RXE	1576	46093000	67767000	51265000		23786000	30344000
tr|Q9RY83	tr|Q9RY83	1709	38023000	45902000	83847000	1.85E+08	1.55E+08	1.59E+08
tr|Q9RRE7	tr|Q9RRE7	513	23987000	26590000	21315000			

tr|Q9LBE3	tr|Q9LBE3	466	7.87E+08	6.12E+08	6.26E+08	4.31E+08	3.79E+08	4.3E+08
tr|Q9RWL	tr|Q9RWL	1436	70472000					1.76E+08
sp|Q9RY4	sp|Q9RY4	416						
sp|Q9RRX	sp|Q9RRX	102	24200000	20811000	18163000			
sp|Q9RYA	tr|Q9RTR2921;425		1.63E+08	1.81E+08	1.57E+08	2.15E+08	1.53E+08	1.61E+08
tr|Q9RY13	tr|Q9RY13	1679	1.45E+08	1.42E+08	1.2E+08	1.4E+08	1.09E+08	1.11E+08
tr|Q9RTZ8	tr|Q9RTZ8	966	52657000	58389000	69605000	32312000	26118000	55320000
tr|Q9RSU7	tr|Q9RSU7	767	3.98E+08	3.98E+08	3.37E+08	4.77E+08	3.7E+08	4.34E+08
tr|Q9RTX6	tr|Q9RTX6	951						
tr|Q9RT53	tr|Q9RT53	821	35511000	22167000	2.39E+08	6.08E+08	6.22E+08	5.15E+08
sp|Q9RTI6	sp|Q9RTI6	188	3.3E+08				2.66E+08	3.31E+08
sp|Q9RYB	sp|Q9RYB	426					51365000	45836000
tr|Q9RYW	tr|Q9RYW	1831	42362000			54888000	60395000	
sp|Q9RU7	sp|Q9RU7	215	22986000	17834000			5074800	
sp|Q9RW7	sp|Q9RW7	314	1.32E+09	1.22E+09	1.1E+09	8.8E+08	7.64E+08	8.66E+08
tr|Q9RR71	tr|Q9RR71	470			19087000			8752300
tr|Q9RYI6	tr|Q9RYI6	1762	40263000	51609000	39281000	38252000	44373000	
tr|Q9RXG3	tr|Q9RXG3	1589						71537000
tr|Q9RXG3	tr|Q9RXG3	1589	1.39E+08		1.22E+08	42805000		66478000
tr|Q9RWU	tr|Q9RWU	1477	61101000	50313000	51253000		53657000	59516000
tr|Q9RWU	tr|Q9RWU	1477		1.56E+08		1.16E+08		
tr|Q9RS08	tr|Q9RS08	624	3.97E+08	4.06E+08	3.8E+08		37029000	
tr|Q9RT75	tr|Q9RT75	831	1.23E+08	1.06E+08	1.59E+08			
tr|Q9RRK3	tr|Q9RRK3	540					65011000	
tr|Q9RRQ	tr|Q9RRQ	564	1.16E+08	1.03E+08	24432000	33553000	40346000	94595000
tr|Q9RRJ7	tr|Q9RRJ7	537	44999000	3.47E+08	19192000	3.47E+08	5.7E+08	3.29E+08
tr|Q9RRJ7	tr|Q9RRJ7	537	48981000	1.97E+08	87201000	14962000	19908000	27926000
sp|Q9RXK	sp|Q9RXK	390					3506400	
tr|Q9RRG6	tr|Q9RRG6	522	1.08E+09	8.62E+08	9.6E+08	1.76E+08	2.09E+08	1.71E+08
sp|Q9RV1	sp|Q9RV1	261			1.34E+08			
sp|Q9RY3	sp|Q9RY3	413	6042700	3790200	3982100	3611000	2957100	3393800
sp|Q9RTN	sp|Q9RTN	196			14628000	34828000		
tr|Q9RTZ9	tr|Q9RTZ9	967	4.84E+08	4.81E+08	4.75E+08	1.06E+09	1.15E+09	8.97E+08
tr|Q9RS07	tr|Q9RS07	623	1.08E+08	1.56E+08	1.5E+08	1.27E+08	1.14E+08	68711000
tr|Q9RY75	tr|Q9RY75	1705	70345000	89063000	51690000	1.38E+08	65121000	1.2E+08
tr|Q9RY33	tr|Q9RY33	1686		4.54E+08		1.37E+08	1.52E+08	10770000
sp|P42443	sp|P42443	49						
tr|Q9RT97	tr|Q9RT97	843	21186000					
tr|Q9RT28	tr|Q9RT28	805	2.61E+08	3.66E+08	3.28E+08	2.56E+08	3.49E+08	2.66E+08
tr|Q9RT28	tr|Q9RT28	805	35217000	57485000	1.21E+08		54172000	29366000
tr|Q9RTB3	tr|Q9RTB3	847	6.48E+08	6.46E+08	5.98E+08	57017000	60915000	78622000
sp|P56867	sp|P56867	56	2.41E+08	1.21E+08	89742000	49475000	62002000	68445000
tr|Q9RXG9	tr|Q9RXG9	1592						
tr|Q9RRW	tr|Q9RRW	597	7.15E+08	1.16E+09	9.86E+08	8.02E+08	1.04E+09	8.46E+08
tr|Q9RUP0	tr|Q9RUP0	1098						
tr|Q9RXX3	tr|Q9RXX3	1661						
sp|Q9RZ8	sp|Q9RZ8	453				408450		
sp|Q9RZ8	sp|Q9RZ8	453			19063000	1.14E+08	2.09E+08	1.91E+08
sp|Q9RSJ9	sp|Q9RSJ9	127	29817000	7628400	8474500	5689200	10657000	6320500
tr|Q9RYS9	tr|Q9RYS9	1808			24464000			
tr|Q9RWR	tr|Q9RWR	1458		12144000		15972000	22483000	15722000

tr|Q9RWR	tr|Q9RWR	1458			12743000			
tr|Q9RY4	tr|Q9RY43	1693						
tr|Q9RT2	tr|Q9RT26	804	40559000	22591000	19701000	17665000	25918000	22954000
sp|Q4657	sp|Q4657	61						2.55E+09
tr|Q9RT0	tr|Q9RT06	795						
tr|Q9RT31	tr|Q9RT31	808					12870000	21036000
sp|Q9RRJ	sp|Q9RRJ	87	1.01E+08	88979000	85217000			
sp|P4244	sp|P42443	49	1.45E+08	1.6E+08	1E+08	76492000	68395000	93218000
sp|P4244	sp|P42443	49					15094000	
sp|Q9RVT	sp|Q9RVT	299		26582000	24274000			
sp|Q9ZNA	sp|Q9ZNA	463	3.33E+08	3.11E+08	2.51E+08	2.44E+08	2.42E+08	2.18E+08
tr|Q9RUL	tr|Q9RUL	1082	4.03E+08	4870700	4650900	1.92E+08	2.17E+08	1.86E+08
tr|Q9RUS	tr|Q9RUS	1112						
tr|Q9RZ1	tr|Q9RZ13	1850	1.29E+08	85446000	87941000	30110000	28251000	30794000
sp|Q9X71	sp|Q9X71	462						
tr|Q9RYD	tr|Q9RYD	1730	42312000	47975000	42530000	56204000		64538000
tr|Q9RS7	tr|Q9RS70	654		22470000		16453000	24499000	21530000
tr|Q9RRG	tr|Q9RRG	522						8546800
tr|Q9RTH	tr|Q9RTH	877			7879900		7044000	
tr|Q9RS71	tr|Q9RS71	655	1.4E+09	1.47E+09	1.22E+09	1.09E+09	8.69E+08	9.81E+08
tr|Q9RTJ1	tr|Q9RTJ1	883					10303000	
sp|Q9RXB	sp|Q9RXB	365				7482600	10197000	
sp|Q9RW	sp|Q9RW	327	3.98E+09	5.21E+09	4.42E+09	2.24E+09	3.36E+09	2.68E+09
tr|Q9RU4	tr|Q9RU43	990			69063000			
tr|Q9RVJ2	tr|Q9RVJ2	1239	51679000	2.52E+08	1.31E+08	3.13E+08	3.15E+08	69151000
tr|Q9RVJ2	tr|Q9RVJ2	1239	2.28E+08	2.49E+08	1.99E+08		1.73E+08	
tr|Q9RTV	tr|Q9RTV6	939	36944000	22453000	67633000	12301000		
tr|Q9RRW	tr|Q9RRW	595				36755000		39222000
tr|Q9RRW	tr|Q9RRW	595				33858000	32221000	37893000
tr|Q9RUD	tr|Q9RUD	1040	48001000	60117000	99706000			88757000
tr|Q9RUZ	tr|Q9RUZ8	1149	41399000	43969000	31390000	43262000		
sp|Q9RW	sp|Q9RW	325	95855000	1E+08	1.12E+08	1.06E+08	1.41E+08	1.9E+08
tr|Q9RXK	tr|Q9RXK9	1608	4572300	3347600	2416400	2072200	281410	2516100
sp|Q9RVI	sp|Q9RVI3	289	8.09E+08	7.38E+08	5.57E+08	93092000	1.03E+08	82410000
tr|Q9RYX	tr|Q9RYX5	1834						
tr|Q9RZU	tr|Q9RZU	1979						
tr|Q9RVG	tr|Q9RVG	1228	2.02E+08	2.38E+08	4.88E+08	4.22E+08	5.29E+08	4.73E+08
tr|Q9RYV	tr|Q9RYV3	1821		24821000	26856000	7508800	23100000	16563000
sp|Q9RUF	sp|Q9RUF	232					10954000	
sp|Q9RYM	sp|Q9RYM	439		782990	1414500		2033200	
sp|Q9RV5	sp|Q9RV5	265				4.13E+08	1.8E+08	1.6E+08
tr|Q9RSM	tr|Q9RSM	735	1.16E+09	4.96E+08	9.46E+08	8.46E+08	7.12E+08	7.92E+08
tr|Q9RSM	tr|Q9RSM	735	80164000					
tr|Q9RWG	tr|Q9RWG	1407		3301900	3023500		2010500	1363300
tr|Q9RRW	tr|Q9RRW	596						27512000
sp|Q9RSS	sp|Q9RSS	148	25726000	66797000	1.8E+08	1.65E+08	2.92E+08	1.66E+08
tr|Q9RRV	tr|Q9RRV8	593	2906300	17914000	21070000		2590100	
tr|Q9RVN	tr|Q9RVN	1264				23632000	37944000	30212000
tr|Q9RX2	tr|Q9RX28	1516	34312000	32648000	27196000	34525000	25726000	26872000
tr|Q9RZ2	tr|Q9RZ23	1855		17338000	34599000			
tr|Q9RU3	tr|Q9RU39	986					25283000	

tr|Q9RUU	tr|Q9RUU	1123						
tr|Q9RXH8	tr|Q9RXH8	1599	67686000	57585000	56117000			
sp|Q9RR7	sp|Q9RR7	67	23553000	57566000	50169000		47708000	10305000
sp|Q9RW0	sp|Q9RW0	327	1.68E+09	3.35E+09	2.61E+09	1.89E+09	3.08E+09	1.98E+09
sp|Q9RVM	sp|Q9RVM	292	24021000	21085000	17864000		10638000	12527000
sp|Q9RT6	sp|Q9RT6	169	1.05E+08	1.3E+08		1.28E+08	96462000	1.03E+08
tr|Q9RVW	tr|Q9RVW	1313					16386000	17813000
tr|Q9RX13	tr|Q9RX13	1510		41933000	68240000	23226000		35305000
tr|Q9RTT5	tr|Q9RTT5	930	6138100	2619400	2887400	1905100	2463100	
tr|Q9RWX	tr|Q9RWX	1499		61276000	27031000	38000000	32691000	24402000
tr|Q9RTB3	tr|Q9RTB3	847	43977000			1.16E+08	81030000	64700000
tr|Q9RYX2	tr|Q9RYX2	1833			15187000			
tr|Q9RVR1	tr|Q9RVR1	1280				24089000	23373000	22163000
tr|Q9RYE0	tr|Q9RYE0	1736	61025000	27591000	47435000			
sp|Q9RXE	sp|Q9RXE	368	4.88E+08	4.1E+08	3.45E+08	7.75E+08	6.16E+08	7.13E+08
tr|Q9RRE0	tr|Q9RRE0	508	35898000		22529000	19418000		
sp|Q9RYD	sp|Q9RYD	430	7342200	5326600	23586000	93994000	51095000	23529000
tr|Q9RZV3	tr|Q9RZV3	1983	36067000	79351000	1.15E+08	51256000	66437000	45700000
tr|Q9RWP	tr|Q9RWP	1449	9.72E+08	9.69E+08	8.38E+08	1.14E+09	1.07E+09	1.03E+09
tr|Q9RWP	tr|Q9RWP	1449	18234000	24292000	42180000			
tr|Q9RU02	tr|Q9RU02	970				80524000	1.01E+08	94852000
sp|Q9RSY	sp|Q9RSY	158						
tr|Q9RXG0	tr|Q9RXG0	1587		66613000	31455000			
tr|Q9RVK8	tr|Q9RVK8	1248						
tr|Q9RVK8	tr|Q9RVK8	1248			37560000			
tr|Q9RRT7	tr|Q9RRT7	581				22712000		14721000
tr|Q9RZH3	tr|Q9RZH3	1929			8111400	24867000	28204000	35285000
tr|Q9RYD9	tr|Q9RYD9	1735	35767000	67710000	58470000	1.37E+08	1.58E+08	1.28E+08
tr|Q9RXF0	tr|Q9RXF0	1581	6.44E+09	6.08E+09	5.93E+09	1.32E+10	8.09E+09	8.55E+09
tr|Q9RWG	tr|Q9RWG	1407		24510000	61815000	1.19E+08	94669000	76563000
sp|Q9RTL8	sp|Q9RTL8	194				22781000	24860000	
sp|Q9RU1	sp|Q9RU1	207			1.04E+08	59187000		
tr|Q9RUH	tr|Q9RUH	1059						
tr|Q9RSL7	tr|Q9RSL7	733	2.93E+08	3.79E+08	3.46E+08	3.05E+08	2.51E+08	2.94E+08
tr|Q9RV83	tr|Q9RV83	1192	5.9E+09	6.36E+09	5.26E+09	3.03E+09	2.51E+09	2.93E+09
tr|Q9RVQ	tr|Q9RVQ	1276	98210000	1.54E+08	1.59E+08	2.39E+08	2.21E+08	2.52E+08
tr|Q9RT67	tr|Q9RT67	825	47404000		32265000	38268000		35653000
tr|Q9RW1	tr|Q9RW1	1339					28201000	
tr|Q9RRQ	tr|Q9RRQ	562		56769000	57060000		77466000	90364000
tr|Q9RRQ	tr|Q9RRQ	562	1.22E+08	1.03E+08	1.54E+08			42544000
sp|Q9RY7	sp|Q9RY7	423	40920000		32178000	18224000	27172000	14588000
tr|Q9RUG	tr|Q9RUG	1051	32490000	22855000	21603000			9768200
tr|Q9RU78	tr|Q9RU78	1014						
tr|Q9RV45	tr|Q9RV45	1172	1.31E+10	1.09E+10	1.18E+10	4.01E+09	6.27E+09	4.59E+09
tr|Q9RV45	tr|Q9RV45	1172	72652000	62780000	1.27E+08			
tr|Q9RTZ9	tr|Q9RTZ9	967	1.06E+08	77948000	67451000	1.21E+08	1.52E+08	1.31E+08
tr|Q9RTZ9	tr|Q9RTZ9	967	2.11E+08	2.08E+08	2.66E+08	1.28E+08	1.88E+08	1.52E+08
tr|Q9RU30	tr|Q9RU30	982	59785000			71203000	55228000	56331000
tr|Q9RU30	tr|Q9RU30	982	2.47E+08	2.58E+08	2.73E+08		61747000	90602000
tr|Q9RSC8	tr|Q9RSC8	691						
sp|Q9RT2	sp|Q9RT2	163	47549000	38187000	40455000	15353000	37954000	21064000

tr|Q9RRA(	tr|Q9RRA(	483	2.52E+08	2.68E+08	2.27E+08	3.23E+08	2.73E+08	2.28E+08
tr|Q9RS93	tr|Q9RS93	668	75184000		83413000	2.19E+08	2.27E+08	1.76E+08
tr|Q9RUU	tr|Q9RUU	1121	29165000	39914000	32893000		15978000	14382000
tr|Q9RY69	tr|Q9RY69	1702	4.63E+08		4.08E+08	4.37E+08	4.36E+08	4.11E+08
tr|Q9RWD	tr|Q9RWD	1395	2.41E+08	1.88E+08	1.73E+08	4.26E+08	4.35E+08	3.5E+08
sp|Q9RSC	sp|Q9RSC	115	2.09E+08	2.24E+08	1.92E+08	2.86E+08	3.13E+08	3.03E+08
tr|Q9RZA3	tr|Q9RZA3	1889						
sp|Q9RWU	sp|Q9RWU	347						
tr|Q9RXC5	tr|Q9RXC5	1570						
tr|Q9RXM	tr|Q9RXM	1614			55482000	22088000	26926000	60950000
tr|Q9RVL9	tr|Q9RVL9	1254		56362000				
tr|Q9RWV	tr|Q9RWV	1487				47552000	83634000	
sp|Q9RRS	sp|Q9RRS	96	7.61E+08	1.02E+09	9.69E+08	1.27E+09	1.19E+09	1.1E+09
tr|Q9RTV5	tr|Q9RTV5	938				23675000	13767000	26770000
sp|Q9RRJ5	sp|Q9RRJ5	88	1.2E+08	1.32E+08	1.09E+08	77344000	63671000	65645000
tr|Q9RZ99	tr|Q9RZ99	1886	83796000		80372000	1.15E+08	1.19E+08	
tr|Q9RUR3	tr|Q9RUR3	1106						
tr|Q9RSI1	tr|Q9RSI1	721	5257000					
tr|Q9RT33	tr|Q9RT33	809						
sp|Q9RT6	sp|Q9RT6	170	41693000			1.6E+08	1.53E+08	1.21E+08
tr|Q9RVU5	tr|Q9RVU5	1302						
sp|Q9RVI3	sp|Q9RVI3	289						
tr|Q9RTJ1	tr|Q9RTJ1	883			13516000			17207000
tr|Q9RW0	tr|Q9RW0	1331	48824000	41579000	32528000	48067000	37750000	45779000
tr|Q9RTY0	tr|Q9RTY0	955	6.41E+08	1.08E+09	6.36E+08	1.43E+09	1.14E+09	1.21E+09
tr|Q9RRN	tr|Q9RRN	551		40954000	31813000	25891000	20805000	33782000
tr|Q9RWU	tr|Q9RWU	1474	4.69E+08	4.38E+08	5.75E+08	1.63E+08	1.69E+08	1.99E+08
sp|P56864	sp|P56864	55			15439000			
tr|Q9RUZ9	tr|Q9RUZ9	1150	50912000	32985000			37368000	29743000
tr|Q9RUQ	tr|Q9RUQ	1104	37820000	26719000	30119000	12950000	9318100	9294900
tr|Q9RXU6	tr|Q9RXU6	1647		28908000	17674000			
sp|Q9RT3	sp|Q9RT3	166	20675000	33995000	95942000	23078000	38318000	83858000
tr|Q9RSY1	tr|Q9RSY1	782						
tr|Q9RYE8	tr|Q9RYE8	1740						
sp|Q9RU2	sp|Q9RU2	209	3.14E+08	3.09E+08	1.36E+09	3.01E+09	7.35E+09	3.23E+09
sp|Q9RW(	sp|Q9RW(	326		21029000	36902000			
tr|Q9RUA	tr|Q9RUA	1026		66412000			67166000	75682000
tr|Q9RUA	tr|Q9RUA	1026	89879000	74616000	50998000			
tr|Q9RT24	tr|Q9RT24	803	39428000	24638000	25032000	9290700		10513000
tr|Q9RV53	tr|Q9RV53	1178	7.58E+08	7.8E+08	7.57E+08		5.56E+08	1.96E+08
tr|Q9RZ16	tr|Q9RZ16	1852						
tr|Q9RY30	tr|Q9RY30	1685						
sp|Q9RTR	sp|Q9RTR	198	26718000	25578000	17558000			
tr|Q9RWT	tr|Q9RWT	1467						
tr|Q9RVT8	tr|Q9RVT8	1297	1.83E+08	1.91E+08	1.39E+08	1.41E+08	1.44E+08	1.24E+08
tr|Q9RV46	tr|Q9RV46	1173				58732000	61014000	56597000
tr|Q9RWK	tr|Q9RWK	1428	9.21E+08	1.1E+09	1.02E+09	6.69E+08	7.24E+08	7.83E+08
tr|Q9RRW	tr|Q9RRW	595	1.19E+08	1.59E+08	1.53E+08	1.95E+08	2.65E+08	2.19E+08
tr|Q9RRK3	tr|Q9RRK3	540	12392000	13167000	8122900		15382000	18854000
tr|Q9RRE1	tr|Q9RRE1	509	1.62E+08	2.34E+08			1.02E+08	1.23E+08
sp|Q9RYC	sp|Q9RYC	428	2.36E+08	2.04E+08	1.89E+08	1.91E+08	1.82E+08	1.96E+08

tr|Q9RX9	tr|Q9RX95	1553	27613000	45005000	14927000	18542000	37772000	21364000
tr|Q9RTD	tr|Q9RTD4	858	42208000	11065000		13614000		
tr|Q9RS74	tr|Q9RS74	656	3804900					5556700
tr|Q9RS74	tr|Q9RS74	656		25119000		26585000	29483000	23604000
sp|Q9RRJ	sp|Q9RRJ5	88	1.04E+08	1.19E+08	13595000	1.37E+08	1.82E+08	1.68E+08
tr|Q9RX4	tr|Q9RX48	1526	42124000	36398000	29644000		42500000	43732000
tr|Q9RZ9	tr|Q9RZ99	1886	27423000	22662000	18086000	24724000	33464000	50225000
tr|Q9RVH	tr|Q9RVH8	1235		32891000	25861000	55670000	46512000	41402000
tr|Q9RVZ	tr|Q9RVZ5	1328	59834000	62031000	79858000	79853000	85918000	70069000
tr|Q9RTZ	tr|Q9RTZ9	967			2268100			
tr|Q9RYE1	tr|Q9RYE1	1737	21338000	15477000	15402000	12352000	12463000	8822600
tr|Q9RWD	tr|Q9RWD	1395	28302000	31645000	79340000		32653000	
tr|Q9RSN	tr|Q9RSN4	741					35009000	
tr|Q9RRP	tr|Q9RRP3	557	2.37E+08	4.07E+08	2.46E+08	75706000	91728000	1.32E+08
tr|Q9RX1	tr|Q9RX12	1509			1.18E+08			
tr|Q9RXM	tr|Q9RXM	1614	12081000	25909000	21254000	43368000	72772000	41513000
tr|Q9RZL7	tr|Q9RZL7	1945	76656000	81206000	88836000			
tr|Q9RU6	tr|Q9RU62	1004	2.33E+08	2.3E+08	1.84E+08	95341000	96525000	
tr|Q9RZ1	tr|Q9RZ13	1850	95164000	1.92E+08	1.47E+08	61792000	92769000	81151000
sp|Q9RV	sp|Q9RVM	292	3.74E+08	3.28E+08	3.19E+08	3.24E+08	3.95E+08	3.14E+08
sp|Q9RW	sp|Q9RWV	349			4652000	5887600	6580800	
tr|Q9RZL7	tr|Q9RZL7	1945	23227000		20941000			
tr|Q9RVE	tr|Q9RVE5	1219	1.03E+08	1.21E+08	48014000	1.26E+08	44831000	60448000
tr|Q9RZL7	tr|Q9RZL7	1945	11330000	16868000	24468000			
sp|Q9RV	sp|Q9RVV	300	10527000	14404000	19177000	4477400	8256600	6064400
tr|Q9RSM	tr|Q9RSM	738	28095000	42406000	36712000	69001000	89983000	74639000
tr|Q9RYG	tr|Q9RYG0	1746	31757000	36075000	33873000		10993000	
sp|Q9RY0	sp|Q9RY0	410	6956600		6386500	74937000	22183000	20349000
tr|Q9RYQ	tr|Q9RYQ0	1788	3.98E+08	3.3E+08	3.15E+08	4.14E+08	4.54E+08	3.71E+08
tr|Q9RYQ	tr|Q9RYQ0	1788					28078000	
sp|Q9RV	sp|Q9RVW	301						
tr|Q9RV5	tr|Q9RV50	1176	1.97E+08	1.8E+08	1.76E+08		72388000	81320000
sp|Q9RRX	sp|Q9RRX	101	1.37E+08	1.01E+08	99499000	2.86E+08	2.87E+08	2.41E+08
sp|Q9RVI	sp|Q9RVI3	289					1.69E+08	1.68E+08
sp|Q9X71	sp|Q9X71	462	17291000	11688000	11682000	17355000	17075000	16405000
tr|Q9RYY7	tr|Q9RYY7	1837						
tr|Q9RYS	tr|Q9RYS5	1804	48617000	34727000	32006000	28171000	34211000	
tr|Q9RXA	tr|Q9RXA9	1560	63080000	29249000	38378000	48076000	43155000	45502000
tr|Q9RXU	tr|Q9RXU1	1644			32131000			
tr|Q9RXG	tr|Q9RXG7	1590			34809000			19227000
tr|Q9RUN	tr|Q9RUN	1093	39742000		29551000			
tr|Q9RX3	tr|Q9RX37	1521	1.28E+08	1.33E+08	96273000	2.17E+08	1.86E+08	1.68E+08
tr|Q9RV5	tr|Q9RV59	1182		43795000	20116000	9965500	7213300	3416400
tr|Q9RTL5	tr|Q9RTL5	896	11346000	12479000	15689000			
tr|Q9RYB	tr|Q9RYB8	1722	1.7E+08	2.42E+08	1.6E+08	70781000	71557000	89478000
sp|Q9RX1	sp|Q9RX1	351	1.71E+08	1.16E+08	1.02E+08	52304000	85451000	
tr|Q9RUD	tr|Q9RUD	1040	8756900	4221100	4074400	4408100	4395800	4110200
tr|Q9RTP	tr|Q9RTP0	906	82026000		80183000	1.19E+08	88997000	89112000
sp|Q9RSL	sp|Q9RSL	135	17125000	7092400	9251400	5183800	3884500	8021400
tr|Q9RRJ7	tr|Q9RRJ7	537	1.88E+09	1.57E+09	1.26E+09	1.3E+09	1.55E+09	1.39E+09
tr|Q9RY04	tr|Q9RY04	1677						

tr|Q9RVQ	tr|Q9RVQ	1274	1.99E+08	1.44E+08	1.94E+08	1.26E+08	1.15E+08	1.63E+08
sp|Q9RW5	sp|Q9RW5	310						
tr|Q9RS85	tr|Q9RS85	661	86315000	1.57E+08	1.38E+08	4.64E+08	4.56E+08	2.88E+08
sp|Q9RU8	sp|Q9RU8	218	4.37E+08	4.07E+08	3.46E+08	6.59E+08	6.13E+08	5.78E+08
sp|Q9RU8	sp|Q9RU8	218	6.46E+08	4.24E+08	8.18E+08	1.29E+08	1.52E+08	3.6E+08
tr|Q9RUL0	tr|Q9RUL0	1082	11849000		37670000			
sp|Q9RRB	sp|Q9RRB	77	42870000	37234000	39194000	32217000	37450000	29811000
tr|Q9RU08	tr|Q9RU08	973	74422000	53245000	52140000	1.12E+08	89637000	1.06E+08
tr|Q9RU08	tr|Q9RU08	973	16206000	31156000	63186000	17061000	17731000	25700000
tr|Q9RVE5	tr|Q9RVE5	1219	40169000	66495000	58570000	67004000	41692000	35609000
tr|Q9RWE	tr|Q9RWE	1398			11614000		8350300	
tr|Q9RYL1	tr|Q9RYL1	1768	75694000		48857000	78350000	57503000	1.03E+08
tr|Q9RTJ1	tr|Q9RTJ1	883	16338000	12216000	53542000	13477000	3777900	26413000
tr|Q9RZK2	tr|Q9RZK2	1940	10271000					
tr|Q9RZK2	tr|Q9RZK2	1940						
tr|Q9RS52	tr|Q9RS52	646		14427000	15977000			
tr|Q9RYE0	tr|Q9RYE0	1736	1.67E+08	1.82E+08	1.91E+08	33856000	42479000	37945000
tr|Q9RV70	tr|Q9RV70	1186						
tr|Q9RSB1	tr|Q9RSB1	681	10378000			45436000	39271000	53250000
tr|Q9RS56	tr|Q9RS56	648		40208000	43996000			22867000
tr|Q9RXF0	tr|Q9RXF0	1581	2.12E+09	2.77E+09	2.92E+09	3.5E+09	4.45E+09	3.57E+09
tr|Q9RXF0	tr|Q9RXF0	1581	69501000	1.39E+08	2.9E+08			40360000
tr|Q9RYW	tr|Q9RYW	1826			52522000	35865000	53739000	43189000
tr|Q9RTI3	tr|Q9RTI3	879			6835200		11884000	15956000
tr|Q9RTY7	tr|Q9RTY7	959	25395000	21672000	17009000	5097400	13964000	9052100
tr|Q9RYQ2	tr|Q9RYQ2	1790	2.81E+08	3.88E+08	2.93E+08	2.01E+08	2.3E+08	2.14E+08
tr|Q9RTD3	tr|Q9RTD3	857	61504000	1.59E+09	1.15E+08			
tr|Q9RR71	tr|Q9RR71	470	36618000	63290000	58687000	31649000	41350000	36168000
tr|Q9RW2	tr|Q9RW2	1346	56426000	72201000	95601000		31980000	
tr|Q9RWV	tr|Q9RWV	1486				25417000	36027000	23197000
tr|Q9RT06	tr|Q9RT06	795	9166900		15743000	28665000	38910000	21206000
tr|Q9RU30	tr|Q9RU30	982	1.27E+08	1.27E+08	96252000	2.01E+08	1.8E+08	2.09E+08
tr|Q9RUW	tr|Q9RUW	1128	16264000	6547200	6474100	867250	12426000	3014100
sp|Q9RW0	sp|Q9RW0	342	2.66E+09	2.41E+09	2.83E+09	2.22E+09	1.95E+09	3.34E+09
sp|Q9RW0	sp|Q9RW0	342						
sp|Q9RU8	sp|Q9RU8	216		89657000	1.53E+08	89137000	38172000	1.55E+08
tr|Q9RUE0	tr|Q9RUE0	1044		28950000	86042000		43726000	21672000
tr|Q9RTK5	tr|Q9RTK5	889	1.65E+08	1.55E+08	1.68E+08			
tr|Q9RXD5	tr|Q9RXD5	1573	64634000	46928000	51341000			16301000
tr|Q9RS76	tr|Q9RS76	657		6032200	4027900	14852000	17006000	21486000
tr|Q9RWK	tr|Q9RWK	1427	9819400		10856000			
tr|Q9RYD7	tr|Q9RYD7	1734		35179000	42357000			
tr|Q9RVX7	tr|Q9RVX7	1318			48896000			
tr|Q9RT53	tr|Q9RT53	821		45274000	32104000	25634000	40808000	25033000
sp|Q9RYM	sp|Q9RYM	439						39001000
tr|Q9RRW	tr|Q9RRW	597	1.11E+08	88502000	1.21E+08	41739000	15773000	26461000
tr|Q9RXM	tr|Q9RXM	1618			5777500		15459000	15212000
tr|Q9RUF4	tr|Q9RUF4	1047	8.94E+08	6.26E+08	5.61E+08	7.62E+08	9.26E+08	9.52E+08
tr|Q9RUF4	tr|Q9RUF4	1047		1.94E+08	2.57E+08	14954000		
tr|Q9RXU6	tr|Q9RXU6	1647	37741000	40194000		40010000		33570000
tr|Q9RTP9	tr|Q9RTP9	911						

tr|Q9RSD8	tr|Q9RSD8	698	60421000	65227000	67271000	33762000	42710000	34386000
tr|Q9RV45	tr|Q9RV45	1172	2.94E+09	2.62E+09	2.95E+09	3.68E+08	7.38E+08	5.85E+08
tr|Q9RZL7	tr|Q9RZL7	1945	1.23E+08	1.02E+08	77895000	98351000	86023000	1.08E+08
tr|Q9RW9	tr|Q9RW9	1374	11499000	10163000	11898000	23259000	12027000	18370000
tr|Q9RZS0	tr|Q9RZS0	1963	1.72E+08		1.12E+08			
sp|Q9RV2	sp|Q9RV2	264	4.26E+08	5.29E+08	63646000	2.71E+08	3.21E+08	3.16E+08
sp|P56864	sp|P56864	55						
tr|Q9RT82	tr|Q9RT82	836	29842000	32992000	17965000	1.79E+08	50009000	1.48E+08
tr|Q9RWU	tr|Q9RWU	1478	5626900	2460500	2825800			
tr|Q9RTJ4	tr|Q9RTJ4	884	3.58E+08	2.56E+08	2.44E+08	2.32E+08	1.54E+08	1.97E+08
tr|Q9RTP2	tr|Q9RTP2	907						
tr|Q9RSE7	tr|Q9RSE7	705						
tr|Q9RTJ8	tr|Q9RTJ8	886						
tr|Q9RVK5	tr|Q9RVK5	1245	3.14E+08	2.87E+08	2.21E+08	4.17E+08	3.98E+08	3.88E+08
tr|Q9RXC5	tr|Q9RXC5	1570	36773000		36960000	65493000	77501000	72155000
tr|Q9RUY4	tr|Q9RUY4	1140		38359000			44025000	
tr|Q9RYU9	tr|Q9RYU9	1819		14564000	19729000	25561000		22477000
tr|Q9RTQ5	tr|Q9RTQ5	915	41569000	41120000	33986000	66818000	61649000	50973000
tr|Q9RW4	tr|Q9RW4	1351	16459000		21249000			
tr|Q9RUB	tr|Q9RUB	1030	88301000	92623000	83277000	1.06E+08	77432000	90266000
tr|Q9RTM	tr|Q9RTM	902	46343000	87140000	70906000			
tr|Q9RXZ4	tr|Q9RXZ4	1670	3.3E+08	2.94E+08	2.85E+08	3.68E+08		3.4E+08
sp|P56860	sp|P56860	53						
sp|P56860	sp|P56860	53						
tr|Q9RYF0	tr|Q9RYF0	1742						
tr|Q9RZP3	tr|Q9RZP3	1953			41176000	9077700		
tr|Q9RTZ9	tr|Q9RTZ9	967	6.09E+08	5.86E+08	5.35E+08	1.49E+09	1.28E+09	1.19E+09
tr|Q9RW8	tr|Q9RW8	1368	1.31E+08	1.52E+08	1.16E+08	1.91E+08	1.47E+08	1.75E+08
tr|Q9RW8	tr|Q9RW8	1368	40077000	60001000	78319000		47518000	50117000
tr|Q9RX57	tr|Q9RX57	1532	1.1E+08	1.1E+08	1.22E+08	1.64E+08	1.55E+08	1.63E+08
tr|Q9RRC0	tr|Q9RRC0	497						
tr|Q9RZD6	tr|Q9RZD6	1910						
tr|Q9RWX	tr|Q9RWX	1497		11486000	11884000		12787000	
tr|Q9RRX3	tr|Q9RRX3	602				19048000	15364000	23990000
sp|Q9RUZ	sp|Q9RUZ	260	4.92E+08	3.95E+08	3.83E+08	4.44E+08	3.98E+08	4.35E+08
tr|Q9RSZ5	tr|Q9RSZ5	790	11291000	7362900	5595000	3671600	5289100	5169400
tr|Q9RYC7	tr|Q9RYC7	1728						
tr|Q9RU34	tr|Q9RU34	983						
sp|Q9RU1	sp|Q9RU1	207	66749000	35541000	63691000	15222000	30250000	40573000
tr|Q9RYP5	tr|Q9RYP5	1784	18711000	18355000	19187000		49617000	
tr|Q9RZP3	tr|Q9RZP3	1953			26862000			
tr|Q9RYA6	tr|Q9RYA6	1720	5.36E+08	5.27E+08	4.97E+08	3.12E+08	3.19E+08	3.02E+08
tr|Q9RYA6	tr|Q9RYA6	1720	53322000		41786000		34473000	30559000
tr|Q9RU62	tr|Q9RU62	1004	30948000	12817000	45692000			
tr|Q9RYF4	tr|Q9RYF4	1743	54724000	51616000	1.29E+08			28748000
sp|Q9RWU	sp|Q9RWU	347	68153000	73927000	69198000	1.81E+08	1.33E+08	1.52E+08
tr|Q9RV15	tr|Q9RV15	1159	60223000	76857000	54201000	62247000	50050000	57057000
sp|Q9RYX	sp|Q9RYX	447	20610000	22662000	13574000	29642000	29110000	30261000
tr|Q9RXD2	tr|Q9RXD2	1572	1.05E+09	9.18E+08	1.16E+09	6E+08	6.52E+08	5.43E+08
tr|Q9RY54	tr|Q9RY54	1696	60392000	77997000	61787000	61293000	49789000	68041000
tr|Q9RS01	tr|Q9RS01	619						

sp|Q9RVG	sp|Q9RVG	287	7.85E+08	7.27E+08	7.94E+08	1.39E+09	1.11E+09	1.29E+09
tr|Q9RV5	tr|Q9RV5	1179	7937700	11616000	12510000	88122000	54449000	58317000
tr|Q9RYX	tr|Q9RYX	1835	42093000	15846000	28447000	40579000	20735000	31098000
sp|Q9RU6	sp|Q9RU6	212						
sp|Q9RXB	sp|Q9RXB	365				16769000		16533000
tr|Q9RVZ	tr|Q9RVZ	1324						
sp|Q9RSS	sp|Q9RSS	148			2.49E+08			
tr|Q9RW	tr|Q9RW	1491				22021000		8733200
tr|Q9RWH	tr|Q9RWH	1411				9659600		
tr|Q9RW9	tr|Q9RW9	1374	29039000			23800000	23530000	
tr|Q9RTZ	tr|Q9RTZ	963				22221000	10994000	
tr|Q9RRZ	tr|Q9RRZ	613				15508000		4413500
tr|Q9RW2	tr|Q9RW2	1342			50936000			
tr|Q9RYP	tr|Q9RYP	1785			14153000			
tr|Q9RWV	tr|Q9RWV	1483						
sp|Q9RYV	sp|Q9RYV	445	7.53E+08	3.03E+08	2.6E+08	5.76E+08	5.25E+08	3.54E+08
tr|Q9RR8	tr|Q9RR8	477		42768000	33848000	48692000	54137000	36849000
tr|Q9RR8	tr|Q9RR8	477	27242000	40524000	1.1E+08		17391000	
tr|Q9RXS	tr|Q9RXS	1637			14522000	20329000		17657000
tr|Q9RYS	tr|Q9RYS	1804	29970000					
tr|Q9RYS	tr|Q9RYS	1804	59377000		59372000			
tr|Q9RXD	tr|Q9RXD	1572	3.58E+08	3.55E+08	4.4E+08	5.1E+08	6E+08	5.16E+08
tr|Q9RSN	tr|Q9RSN	741	23658000	16349000	15672000	41719000	48567000	46330000
sp|Q9RSC	sp|Q9RSC	115	8.83E+08	9.04E+08	7.4E+08	1.62E+08	1.61E+08	1.33E+08
tr|Q9RVJ7	tr|Q9RVJ7	1242		18968000			13934000	18447000
tr|Q9RX2	tr|Q9RX2	1517						
tr|Q9RSR	tr|Q9RSR	751		14746000			23286000	
sp|Q9RV6	sp|Q9RV6	267				7897600	9807800	5713500
sp|Q9RV6	sp|Q9RV6	267	4.52E+08	2.51E+08	4.14E+08	7.08E+08	7.24E+08	8.01E+08
tr|Q9RVN	tr|Q9RVN	1263	38663000	39180000		1.01E+08	1.47E+08	81534000
sp|P5686	sp|P5686	56	76022000	63471000	67293000			
tr|Q9RR71	tr|Q9RR71	470	95708000	1.13E+08	1.24E+08	1.38E+08	3.35E+08	1.45E+08
tr|Q9RXX	tr|Q9RXX	1660	8762700	5601800	5482300	2597200	3837600	
tr|Q9RSU	tr|Q9RSU	767						
tr|Q9RST7	tr|Q9RST7	760	30410000		62066000	1.32E+08	1.3E+08	1.31E+08
sp|Q9RX3	sp|Q9RX3	357	71788000	83806000	72312000		1.82E+08	
sp|Q9RX3	sp|Q9RX3	357	30269000		25667000			
tr|Q9RU4	tr|Q9RU4	992						
tr|Q9RU4	tr|Q9RU4	992						
sp|Q9RUR	sp|Q9RUR	249	7.06E+09	1.81E+09	3.84E+09	1.02E+10	1.03E+10	9.18E+09
sp|Q9RUR	sp|Q9RUR	249	89830000	1.07E+08	1.08E+08	6.76E+08	2.15E+08	5.29E+08
tr|Q9RW0	tr|Q9RW0	1336	1.12E+08	1.1E+08	68642000	1.96E+08	1.52E+08	1.94E+08
tr|Q9RSU	tr|Q9RSU	764	5.21E+08	5.53E+08	3.69E+08			
tr|Q9RUC	tr|Q9RUC	1034	13840000	9573200	6980300	1731300	1850800	2664400
sp|Q9RSF	sp|Q9RSF	117	2.16E+09	1.65E+09	1.36E+09	1.86E+09	1.5E+09	1.78E+09
sp|Q9RSF	sp|Q9RSF	117	65438000		45105000	11036000	9421500	13450000
tr|Q9RY34	tr|Q9RY34	1687		5058400	75815000	1.02E+08	1.33E+08	1.03E+08
tr|Q9RXY	tr|Q9RXY	1666			18292000			
tr|Q9RX6	tr|Q9RX6	1537		37483000				
tr|Q9RSI3	tr|Q9RSI3	723		3.21E+08	1.47E+08	1.71E+08	1.61E+08	1.63E+08
tr|Q9RUA	tr|Q9RUA	1023		12045000	17562000	16925000	13817000	

tr|Q9RTD3	tr|Q9RTD3	857						
tr|Q9RTD3	tr|Q9RTD3	857			83589000	1.55E+08	3.13E+08	67733000
tr|Q9RYI1	tr|Q9RYI1	1760			43138000	2.43E+08	1.67E+08	1.09E+08
sp|Q9RR7	sp|Q9RR7	68	44041000	48785000	46335000	23332000	29594000	52692000
tr|Q9RVK8	tr|Q9RVK8	1248	4.65E+09	5.56E+08	3.54E+09	3.56E+09	3.71E+09	3.97E+09
tr|Q9RRD2	tr|Q9RRD2	503		42915000		24428000	22688000	21404000
tr|Q9RRD2	tr|Q9RRD2	503	2.5E+08	2.44E+08	4.3E+08	3.2E+08	1.67E+08	1.74E+08
tr|Q9RT53	tr|Q9RT53	821	24085000	17868000	35458000	13346000	19750000	20086000
tr|Q9RVS1	tr|Q9RVS1	1287	2.93E+08	44601000	3675300	3.8E+08	6.67E+08	16766000
tr|Q9RVS1	tr|Q9RVS1	1287	1.58E+09	3.15E+09	2.62E+09	7.11E+08	1.77E+09	8.53E+08
tr|Q9RYC5	tr|Q9RYC5	1726			14519000			
tr|Q9RS77	tr|Q9RS77	658	77988000	63893000	47926000	1.07E+08	89576000	96180000
tr|Q9RVZ5	tr|Q9RVZ5	1328	25573000				19402000	25393000
tr|Q9RY13	tr|Q9RY13	1679	75116000	80046000	83115000	26730000	42473000	38140000
tr|Q9RRB4	tr|Q9RRB4	495	89089000	1.37E+08	97154000	75364000	81602000	88666000
sp|Q9RRB	sp|Q9RRB	75	85354000	1.57E+08	1.42E+08	79599000	59224000	65384000
sp|Q9RZ8	sp|Q9RZ8	453	1.97E+09	2.41E+09	2.08E+09	2.35E+08	6.13E+08	6.2E+08
sp|Q9RZ8	sp|Q9RZ8	453		25122000	2.26E+08	76297000	2.57E+08	1.45E+08
sp|Q9RR6	sp|Q9RR6	65	6.41E+09	5.77E+09	4.91E+09	6.4E+09	5.74E+09	6.29E+09
sp|Q9RR6	sp|Q9RR6	65	1.7E+08	3.2E+08	4.51E+08	31563000	62826000	1.04E+08
sp|Q9RR6	sp|Q9RR6	65			52658000	82593000		78870000
tr|Q9RTD4	tr|Q9RTD4	858	90741000		60400000			97245000
tr|Q9RYD0	tr|Q9RYD0	1730		53013000	29997000	84597000	71893000	
tr|Q9RYD0	tr|Q9RYD0	1730	4.03E+08	6.74E+08	3.49E+08	3.19E+08	4.59E+08	
tr|Q9RR83	tr|Q9RR83	475	2.13E+08	2.6E+08	1.93E+08	67007000	59539000	73000000
tr|Q9RUR6	tr|Q9RUR6	1108	41559000	20125000	62136000	2.78E+08	3.47E+08	3.39E+08
tr|Q9RUR6	tr|Q9RUR6	1108	26051000	37020000	43575000			
sp|Q9RXY	sp|Q9RXY	407					1820700	
tr|Q9RYT8	tr|Q9RYT8	1816	1.6E+09		2.59E+08	6.03E+08	5.39E+08	
tr|Q9RTE8	tr|Q9RTE8	864	25410000			18540000	30312000	18722000
tr|Q9RWQ	tr|Q9RWQ	1452	5.68E+08	5.93E+08		5.41E+08	5.06E+08	5.59E+08
tr|Q9RWQ	tr|Q9RWQ	1452	17338000					
sp|Q9RTT	sp|Q9RTT	200						
sp|Q9RTT	sp|Q9RTT	200	5.28E+08	7.55E+08	5.79E+08	44871000	3.86E+08	2.97E+08
tr|Q9RRW	tr|Q9RRW	599	1.72E+09	1.84E+09	1.89E+09	1.52E+09	1.65E+09	2.25E+09
sp|Q9RY5	sp|Q9RY5	417						
sp|Q9RW0	sp|Q9RW0	327	9.3E+08	2.2E+09	7.46E+08	44960000	1.17E+08	1.72E+08
tr|Q9RT01	tr|Q9RT01	792						
sp|Q9RV2	sp|Q9RV2	263			16413000	24506000	23721000	26453000
tr|Q9RX33	tr|Q9RX33	1519	1046700	529030	490620	560790	588380	524080
tr|Q9RUZ7	tr|Q9RUZ7	1148	26858000	53802000	58213000		14681000	33393000
tr|Q9RX62	tr|Q9RX62	1535				31229000		
tr|Q9RTU7	tr|Q9RTU7	935	3.06E+08	3.14E+08	2.22E+08		6.15E+08	5.61E+08
sp|Q9RY5	sp|Q9RY5	418	4659300	2395600	3413500	4964400	4744500	4771800
sp|Q9RTD	sp|Q9RTD	179	39441000	99898000	50526000	60142000	63522000	61221000
sp|Q9RTD	sp|Q9RTD	179	18956000					
tr|Q9RYI1	tr|Q9RYI1	1760						
tr|Q9RYI1	tr|Q9RYI1	1760	96897000	56019000	1.08E+08		51234000	
tr|Q9RS40	tr|Q9RS40	640	85736000	1.01E+08	1.18E+08	20139000	31731000	28508000
tr|Q9RTG6	tr|Q9RTG6	872						
sp|Q9RRM	sp|Q9RRM	92		7.23E+08	5.5E+08	5.1E+08		5.26E+08

sp|Q9RX5	sp|Q9RX5	359	31500000			24212000	27591000	1.11E+08
sp|Q9RS6	sp|Q9RS6	111	1.29E+10	6.82E+09	6.21E+09	3.24E+09	2.89E+09	3.98E+09
sp|Q9RTF	sp|Q9RTF	183						
tr|Q9RVJ7	tr|Q9RVJ7	1242					76155000	84876000
tr|Q9RTJ4	tr|Q9RTJ4	884	49879000	62431000	91065000			
tr|Q9RXU	tr|Q9RXU	1648						
sp|Q9RSW	sp|Q9RSW	153	12006000				13316000	9425100
tr|Q9RR6	tr|Q9RR66	468	5.58E+08	4.06E+08	3.58E+08	3.6E+08	3.65E+08	3.88E+08
sp|Q9RW	sp|Q9RW	346	77514000	97372000	1.02E+08	83420000	86232000	94666000
tr|Q9RS31	tr|Q9RS31	637				74579000	52328000	63667000
tr|Q9RXA	tr|Q9RXA4	1558						
sp|Q9RTE	sp|Q9RTE	180	8728400	13761000	10432000	22993000	14403000	14639000
tr|Q9RVQ	tr|Q9RVQ	1271	47662000		56667000			
tr|Q9RW0	tr|Q9RW0	1335	1.32E+08	1.44E+08	1.21E+08			
sp|Q9RRB	sp|Q9RRB	76	2.99E+09	3.47E+09	2.46E+09	1.71E+09	1.67E+09	1.98E+09
sp|Q9RRB	sp|Q9RRB	76						
tr|Q9RZ1	tr|Q9RZ13	1850	2.32E+08	2.69E+08	1.94E+08	3.49E+08	3.46E+08	
tr|Q9RU5	tr|Q9RU54	997						
sp|Q9RXS	sp|Q9RXS	397					9167500	10066000
tr|Q9RU0	tr|Q9RU0	968	3.65E+08	7.53E+08	4.22E+08	2.87E+08	4.32E+08	2.92E+08
tr|Q9RRR	tr|Q9RRR	570	27427000	64406000	36244000			
tr|Q9RYW	tr|Q9RYW	1829				31680000	28910000	16482000
sp|Q9RY2	sp|Q9RY2	411					14435000	28102000
tr|Q9RTR	tr|Q9RTR4	923	4.68E+08	1.15E+09	6.21E+08	1.08E+09	1.17E+09	8.06E+08
tr|Q9RTQ	tr|Q9RTQ5	915						
tr|Q9RRA	tr|Q9RRA2	485	9519000	5744400	5710500	4293300	7626400	6530800
tr|Q9RYS5	tr|Q9RYS5	1804		22947000	33450000		20635000	27733000
sp|Q9RTG	sp|Q9RTG	184	1.25E+08	1.58E+08	1.27E+08	2.17E+08	1.47E+08	2.08E+08
tr|Q9RSE4	tr|Q9RSE4	703						
tr|Q9RZC	tr|Q9RZC5	1904	1287500				582500	499920
sp|Q9RSC	sp|Q9RSC	115	8.29E+08	5.71E+08	5.87E+08	7.57E+08	5.56E+08	8.96E+08
tr|Q9RTQ	tr|Q9RTQ	912	83027000	65381000	77005000			38445000
sp|Q9RYZ	sp|Q9RYZ	449						
sp|Q9RYZ	sp|Q9RYZ	449						
tr|Q9RYS	tr|Q9RYS9	1808	96778000	1.26E+08	1.15E+08	91813000	1.88E+08	1.35E+08
tr|Q9RXG	tr|Q9RXG	1591	2.49E+08	1.85E+08	1.63E+08	1.85E+08	1.76E+08	1.61E+08
tr|Q9RUH	tr|Q9RUH	1056	99620000	1.17E+08	1.02E+08	70638000	57238000	79059000
tr|Q9RVM	tr|Q9RVM	1257	98073000	93237000	94408000			
tr|Q9RSD	tr|Q9RSD	692			592020			
tr|Q9RTI9	tr|Q9RTI9	882	1.9E+08	2.23E+08	81267000	5.1E+08	2.3E+08	4.12E+08
tr|Q9RYX1	tr|Q9RYX1	1832	1.41E+08			2.36E+08	1.8E+08	1.6E+08
tr|Q9RWX	tr|Q9RWX	1499	28172000	27633000	21707000	59415000		42522000
sp|Q9RV2	sp|Q9RV2	264	4.88E+08	4.95E+08	4.37E+08	7.41E+08	6.13E+08	6.8E+08
tr|Q9RTM	tr|Q9RTM	902	37416000	33539000				
tr|Q9RST	tr|Q9RST9	761	12669000		30766000			
sp|Q9RV1	sp|Q9RV1	261	5.99E+08	5.05E+08	4.53E+08	5.63E+08	6.08E+08	7.04E+08
tr|Q9RV4	tr|Q9RV48	1175	1.35E+09	1.15E+09	9.31E+08	5E+08	7.22E+08	6E+08
tr|Q9RX6	tr|Q9RX64	1537	72936000	1.08E+08	88998000		1.33E+08	
tr|Q9RSS2	tr|Q9RSS2	755	7564400	6129200		3871600	4807600	12530000
sp|Q9RUP	sp|Q9RUP	246	15396000	43164000	66084000		19310000	10669000
tr|Q9RYC	tr|Q9RYC6	1727	2.42E+08	2.68E+08	2.04E+08	2.88E+08	2.22E+08	2.43E+08

tr|Q9RYI7	tr|Q9RYI7	1763				10607000	12213000	
tr|Q9RUY	tr|Q9RUY1	1138	8673100	8271200	8994600	2.03E+08	1.61E+08	2.06E+08
tr|Q9RXD	tr|Q9RXD5	1573	41956000	58073000	93029000			
sp|Q9RSQ	sp|Q9RSQ	141						
tr|Q9RZV	tr|Q9RZV3	1983						
tr|Q9RZS0	tr|Q9RZS0	1963						
sp|Q9RT2	sp|Q9RT2	163			57346000			
sp|Q9RYB	sp|Q9RYB	426	1.98E+09	3.41E+09	2.18E+09	3.27E+09	2.76E+09	4.48E+08
sp|Q9RYB	sp|Q9RYB	426	1.27E+09	1.28E+09	1.75E+09	3.63E+08		6.53E+08
tr|Q9RXU	tr|Q9RXU9	1649		15165000	23563000			
sp|Q9RXG	sp|Q9RXG	371	3.06E+08	3.56E+08	2.33E+08	3.44E+08		
sp|Q9RVP	sp|Q9RVP	294	2.25E+08	2.37E+08	5.52E+08			
tr|Q9RZU0	tr|Q9RZU0	1976	66257000	57851000	66413000	3.27E+08	3.28E+08	3.08E+08
tr|Q9RZG	tr|Q9RZG9	1926						
tr|Q9RU2	tr|Q9RU26	979	12048000	3860200	3490100	1693000	1676300	1879200
tr|Q9RT07	tr|Q9RT07	796			25524000			
tr|Q9RUC	tr|Q9RUC	1038	3.57E+08	9.01E+08	5.14E+08	9.31E+08	1.09E+09	9.08E+08
tr|Q9RWP	tr|Q9RWP	1449	35576000	14436000	1.04E+08			7756400
tr|Q9RZH	tr|Q9RZH2	1928	71559000	46646000	51484000	50697000	16077000	35741000
tr|Q9RYT5	tr|Q9RYT5	1814	1.68E+08	2.01E+08	2.11E+08		1.1E+08	1.19E+08
sp|Q9RUR	sp|Q9RUR	249	9395300	4372400	3647400	3887700	5662700	6254600
sp|Q9RUR	sp|Q9RUR	249	42630000	25344000	45843000	52288000	58142000	56190000
sp|Q9RUR	sp|Q9RUR	249	1.49E+09	1.42E+09	2.02E+09	1.78E+08	2.92E+08	4.58E+08
tr|Q9RSC6	tr|Q9RSC6	689			30564000	1.45E+08	1.14E+08	
tr|Q9RV70	tr|Q9RV70	1186	2.04E+08	2.03E+08	2.28E+08	8.13E+08	6.14E+08	7.43E+08
tr|Q9RRW	tr|Q9RRW	597	2128700	961270		1337400		1315600
tr|Q9RUN	tr|Q9RUN	1093						
sp|P5686	sp|P56864	55	15749000	74194000	29426000	67854000	55933000	92992000
sp|P5686	sp|P56864	55			31791000			
sp|Q9RTY	sp|Q9RTY	204	84739000	58281000	1.46E+08	75684000	73464000	81684000
tr|Q9RRZ0	tr|Q9RRZ0	611						36958000
tr|Q9RXW	tr|Q9RXW	1656	29757000	25225000				16492000
tr|Q9RYW	tr|Q9RYW	1827				54284000	38924000	50595000
tr|Q9RUG	tr|Q9RUG	1052						
tr|Q9RYN	tr|Q9RYN9	1782	15867000	41750000	20320000	25885000	30553000	19400000
tr|Q9RU2	tr|Q9RU28	981	4.09E+09	3.89E+09	3.07E+09	5.07E+09	3.68E+09	5.07E+09
tr|Q9RU2	tr|Q9RU28	981	1.34E+09	7.67E+08	1.92E+09	1.26E+09	1.53E+09	1.46E+09
sp|P5686	sp|P56867	56	1.3E+10	1.35E+10	1.15E+10	1.89E+10	1.62E+10	3.16E+09
sp|P5686	sp|P56867	56		27367000				
sp|Q9RSR	sp|Q9RSR	144	1.78E+08	1.97E+08	3.63E+08	1.42E+08	1.62E+08	2.67E+08
tr|Q9RY34	tr|Q9RY34	1687	1.03E+09	2.01E+08	8.31E+08	4.06E+08	3.45E+08	4.72E+08
sp|Q9RT5	sp|Q9RT5	168						23783000
tr|Q9RTY1	tr|Q9RTY1	956	89112000	1.28E+08	95127000	97684000	82680000	72550000
tr|Q9RXQ	tr|Q9RXQ	1627	31306000	37303000	49940000			
sp|P4244	sp|P42443	49						
tr|Q9RRY	tr|Q9RRY4	607	61169000	44074000	63282000	51304000	79500000	77077000
tr|Q9RV11	tr|Q9RV11	1156						
tr|Q9RRX0	tr|Q9RRX0	600	49181000	47342000	41308000	21358000	21127000	36283000
tr|Q9RXD	tr|Q9RXD5	1573		2.38E+08		1.61E+08		1.64E+08
tr|Q9RV32	tr|Q9RV32	1168	21805000	14311000	13521000	37285000	23622000	28872000
tr|Q9RX10	tr|Q9RX10	1507	5.25E+08	3.97E+08	3.64E+08	6.37E+08	1.3E+09	3.69E+08

tr|Q9RY55	tr|Q9RY55	1697	59690000	68085000	47683000	43718000	50202000	52104000
tr|Q9RSQ6	tr|Q9RSQ6	749	1.84E+08	1.71E+08	1.54E+08	1.9E+08	2.63E+08	2.11E+08
tr|Q9RVM	tr|Q9RVM	1256	50958000	46651000	34300000	1.29E+08	1.35E+08	1.42E+08
tr|Q9RXL6	tr|Q9RXL6	1612	82223000	45365000	14712000	3.65E+08	4.98E+08	13204000
tr|Q9RWY	tr|Q9RWY	1502						
tr|Q9RRE1	tr|Q9RRE1	509	2.9E+08	30476000	1.33E+08		65448000	
sp|Q9RY2	sp|Q9RY2	411			1.67E+08			
tr|Q9RX10	tr|Q9RX10	1507	2.01E+08	3.5E+08	1.99E+08	1.81E+08	1.53E+08	1.7E+08
tr|Q9RXW	tr|Q9RXW	1657		25667000				28722000
tr|Q9RW2	tr|Q9RW2	1342	1.88E+08	1.32E+08	1.68E+08	58458000	58341000	65341000
tr|Q9RV45	tr|Q9RV45	1172	2.39E+08	1.39E+08	1.38E+08	3.66E+08	4.4E+08	3.9E+08
sp|P56867	sp|P56867	56		29147000	36934000		76072000	32444000
tr|Q9RU60	tr|Q9RU60	1003	1.06E+08	1.06E+08	69724000	74816000	1.82E+08	1.55E+08
sp|Q9RXC	sp|Q9RXC	367		75731000				
tr|Q9RU15	tr|Q9RU15	974		1.56E+08		2.29E+08	1.93E+08	
tr|Q9RZE6	tr|Q9RZE6	1916	2.02E+08	1.74E+08	1.46E+08			
tr|Q9RWF	tr|Q9RWF	1405	11887000		17623000	32260000	25900000	23527000
sp|Q9RYM	sp|Q9RYM	437				17808000		
tr|Q9RR71	tr|Q9RR71	470						
tr|Q9RTX7	tr|Q9RTX7	952	1.7E+08	1.25E+08	1.59E+08	68933000	65384000	75696000
tr|Q9RV15	tr|Q9RV15	1159				1.06E+08	84047000	
tr|Q9RZU6	tr|Q9RZU6	1978						
tr|Q9RVD1	tr|Q9RVD1	1213	26104000			17872000		14756000
sp|Q9RXJ6	sp|Q9RXJ6	381	1.12E+09	9.36E+08	1.38E+09	98888000	1.67E+08	1.93E+08
tr|Q9RY30	tr|Q9RY30	1685	1.03E+09	1.21E+09	1.04E+09	8.72E+08	7.08E+08	8.33E+08
tr|Q9RVP9	tr|Q9RVP9	1270			26168000			
tr|Q9RVL0	tr|Q9RVL0	1250	2.94E+08	2.87E+08	2.39E+08	3.05E+08	2.98E+08	2.73E+08
tr|Q9RZA6	tr|Q9RZA6	1891	20402000		22879000	1.02E+08	52132000	88682000
tr|Q9RZA6	tr|Q9RZA6	1891			33797000	18150000		
sp|Q9RYG	sp|Q9RYG	434	1.61E+08			2.06E+08	1.83E+08	1.8E+08
sp|Q9RYG	sp|Q9RYG	434	59835000	47299000			39185000	65229000
sp|Q9RVQ	sp|Q9RVQ	295						
tr|Q9RVU4	tr|Q9RVU4	1301	27153000	21989000	17386000	30031000	18481000	27202000
tr|Q9RYG3	tr|Q9RYG3	1748						
tr|Q9RSK5	tr|Q9RSK5	731	2.33E+08	2.13E+08	2.16E+08	1.45E+08	1.82E+08	1.21E+08
tr|Q9RUY4	tr|Q9RUY4	1140	41081000	58309000	45640000			
tr|Q9RS17	tr|Q9RS17	630	2.07E+09	1.28E+08	1.43E+08	2.05E+09	1.63E+09	1.9E+09
tr|Q9RTY1	tr|Q9RTY1	956	3.36E+08	3.42E+08	2.85E+08	3.03E+08	2.34E+08	2.88E+08
tr|Q9RVF4	tr|Q9RVF4	1223						
sp|Q9RT3	sp|Q9RT3	166	14989000	47747000	76683000	26440000	56162000	39969000
sp|O8302	sp|O8302	47			26526000			
tr|Q9RVB5	tr|Q9RVB5	1209	2.65E+09	2.86E+09	2.2E+09	1.43E+09	1.43E+09	1.57E+09
tr|Q9RVA2	tr|Q9RVA2	1199	62383000	39594000	66164000	38520000	41177000	44022000
tr|Q9RVK8	tr|Q9RVK8	1248	1.83E+08	1.19E+08	1.19E+08	42207000	69946000	44192000
tr|Q9RZD3	tr|Q9RZD3	1908					11409000	13731000
tr|Q9RTT2	tr|Q9RTT2	929						
tr|Q9RVU8	tr|Q9RVU8	1303	7.25E+08	7.11E+08	7.58E+08	3.93E+08	3.45E+08	3.96E+08
tr|Q9RU70	tr|Q9RU70	1009	48769000		31890000			
tr|Q9RWF	tr|Q9RWF	1400						
tr|Q9RXW	tr|Q9RXW	1658	61538000	53129000	46432000	19114000	19250000	23131000
sp|Q9RYB	sp|Q9RYB	426	1.15E+08	94715000	2.15E+08	9601300	61018000	36821000

tr|Q9RW3	tr|Q9RW3	1350	25187000	31630000	79855000			5301200
sp|Q9R34	sp|Q9R34	64	70632000	18432000	17169000	14591000	21409000	16494000
sp|Q9R34	sp|Q9R34	64	1.14E+09	1.17E+09	1.16E+09	19032000		42377000
tr|Q9RVG	tr|Q9RVG	1229						
sp|Q9RVV	sp|Q9RVV	300	1.1E+09	1.23E+09	1.87E+09	9.71E+08	1.02E+09	1.32E+09
tr|Q9RTV6	tr|Q9RTV6	939	2.48E+08	2.12E+08	2.22E+08			9008800
tr|Q9RS33	tr|Q9RS33	639	1.32E+09	1.22E+09	9.84E+08	1.32E+09	1.11E+09	1.15E+09
sp|Q5933	sp|Q5933	63	37151000	60879000	1.74E+08		28514000	
tr|Q9RWI	tr|Q9RWI	1413	21126000			1.03E+08	59221000	78136000
tr|Q9RR77	tr|Q9RR77	473	70388000	49354000	51537000	1.48E+08	1.19E+08	1.13E+08
tr|Q9RVW	tr|Q9RVW	1313		9224900			18101000	
tr|Q9RSB9	tr|Q9RSB9	686		33635000	55014000			29668000
tr|Q9RRE1	tr|Q9RRE1	509	74918000	73427000	1.08E+08			
tr|Q9RX10	tr|Q9RX10	1507			41479000			
sp|Q9RY4	sp|Q9RY4	416				37387000		31507000
tr|Q9RTB3	tr|Q9RTB3	847						
tr|Q9RZ83	tr|Q9RZ83	1876		60890000	52442000			
tr|Q9RSG4	tr|Q9RSG4	713	69605000		63616000	32817000	61618000	43043000
sp|O3250	sp|O3250	46						
tr|Q9RSS3	tr|Q9RSS3	756	68293000	1.06E+08	48601000	75068000	58407000	61294000
tr|Q9RSV5	tr|Q9RSV5	771						
tr|Q9RTD1	tr|Q9RTD1	856	7501900	6660200	64243000	79151000	89429000	74065000
tr|Q9RVQ	tr|Q9RVQ	1272	6911200	2499600	2109900			
tr|Q9RX19	tr|Q9RX19	1514				30374000	30111000	28798000
tr|Q9RSU0	tr|Q9RSU0	762	9973600		29277000			
tr|Q9RSQ6	tr|Q9RSQ6	749	6.81E+08	7.63E+08	6.44E+08	5.66E+08	4.49E+08	5.18E+08
tr|Q9RSQ6	tr|Q9RSQ6	749	44910000		41284000			
tr|Q9RTM	tr|Q9RTM	899			2252100	6725500	6995300	6939000
tr|Q9RSL8	tr|Q9RSL8	734				29614000		
tr|Q9RSL8	tr|Q9RSL8	734		41277000	43455000	90649000	54497000	60162000
tr|Q9RSL8	tr|Q9RSL8	734	45913000	53456000	98819000	35726000		53477000
tr|Q9RTP9	tr|Q9RTP9	911	23603000	33352000	23584000			
sp|Q9RRT	sp|Q9RRT	98		1.97E+08		1.69E+08	1.04E+08	1.45E+08
tr|Q9RRZ0	tr|Q9RRZ0	611	47773000	46210000	37999000	75460000		53530000
sp|Q9RUN	sp|Q9RUN	241	3.31E+08	2.36E+08	1.95E+08	72132000	87155000	61486000
sp|Q9RT2	sp|Q9RT2	165	1.11E+10	1.19E+10	9.48E+09	1.04E+10	1.02E+10	1.1E+10
sp|Q9RYA	sp|Q9RYA	425						
tr|Q9RS76	tr|Q9RS76	657	4.21E+09	2.53E+09	3.45E+09	1.08E+09	1.1E+09	1.32E+09
tr|Q9RS76	tr|Q9RS76	657			1.19E+08			
sp|Q9RUE	sp|Q9RUE	226	89439000	80674000	70339000	92658000	1.06E+08	1.13E+08
sp|Q9RUE	sp|Q9RUE	226	3.84E+08	8.29E+08	1.18E+09	6.24E+08	9.53E+08	7.2E+08
tr|Q9RTL3	tr|Q9RTL3	894	78969000		51293000		46571000	
tr|Q9RVQ	tr|Q9RVQ	1274				1812500	1967000	1968400
tr|Q9RUB	tr|Q9RUB	1032	76257000	93094000	99894000	1.1E+08	1.08E+08	75177000
tr|Q9RVR2	tr|Q9RVR2	1281		18345000	34546000	30720000	34038000	34731000
tr|Q9RXZ4	tr|Q9RXZ4	1670						
tr|Q9RUK3	tr|Q9RUK3	1077	9.52E+08	7.9E+08	30067000	3.32E+08	4.8E+08	3.1E+08
sp|Q9RTD	sp|Q9RTD	179	90679000	87045000	92794000	1.96E+08	1.33E+08	1.41E+08
sp|Q9RY4	sp|Q9RY4	415	40038000	29320000	23277000	23680000	20750000	22237000
sp|Q9RY4	sp|Q9RY4	415	9.73E+08	7.62E+08	9.9E+08	3.26E+08	3.38E+08	4.87E+08
tr|Q9RTX0	tr|Q9RTX0	947			48658000			

tr|Q9RW4	tr|Q9RW4	1352	3.21E+08	2.44E+08	2.13E+08	1.71E+08		1.66E+08
tr|Q9RSD4	tr|Q9RSD4	696	4.44E+09	3.54E+09	3.47E+09	5.87E+09	7.3E+09	5.39E+09
tr|Q9RSD4	tr|Q9RSD4	696	6.22E+09	5.03E+09	4.36E+09	2.31E+09	3.09E+09	2.84E+09
tr|Q9RUD	tr|Q9RUD	1043	56649000	62316000	2.12E+08	97967000	1.46E+08	65076000
tr|Q9RTI3	tr|Q9RTI3	879	4977200	2506000	2446700			1684800
tr|Q9RTY1	tr|Q9RTY1	956	3.22E+08	2.24E+08	2.31E+08	53103000		48420000
sp|P52153	sp|P52153	52	4.02E+08	2.17E+08	3.29E+08	3.8E+08	6.24E+08	3.37E+08
tr|Q9RSI1	tr|Q9RSI1	721			18719000			
tr|Q9RX41	tr|Q9RX41	1522			13360000			19758000
tr|Q9RZS7	tr|Q9RZS7	1967					6394500	
sp|Q9RW0	sp|Q9RW0	327	8427900	2147800	2419500		1619000	669850
tr|Q9RVN	tr|Q9RVN	1261						
sp|O32508	sp|O32508	46	22849000	11891000		32922000	29333000	34768000
sp|O32508	sp|O32508	46						
tr|Q9RY38	tr|Q9RY38	1691	76890000					
tr|Q9RYQ4	tr|Q9RYQ4	1792	1.64E+08	2.04E+08	1.97E+08	3.22E+08	3.37E+08	3.36E+08
sp|Q9RSF0	sp|Q9RSF0	117						
sp|Q9RXW	sp|Q9RXW	404	91859000	27835000	25949000	32724000	32525000	45401000
tr|Q9RZR2	tr|Q9RZR2	1961						
tr|Q9RZ81	tr|Q9RZ81	1875			18100000			21161000
tr|Q9RRH	tr|Q9RRH	525	2.51E+08	2.23E+08	2.44E+08	1.16E+09	1.13E+09	1.16E+09
sp|Q9RRG	sp|Q9RRG	84	1.55E+09	9.74E+08	1.24E+09			
tr|Q9RZS7	tr|Q9RZS7	1967	35537000	51509000	58714000		33771000	30422000
tr|Q9RWP	tr|Q9RWP	1448	1.5E+08	3.75E+08	7.02E+08	4.38E+08	3.82E+08	4.14E+08
tr|Q9RT45	tr|Q9RT45	816						
tr|Q9RTG9	tr|Q9RTG9	874	8473000	20374000	17411000	53127000	37030000	31680000
tr|Q9RX85	tr|Q9RX85	1550						
sp|Q9RUE	sp|Q9RUE	229	53190000	39091000	47074000			20479000
tr|Q9RZ70	tr|Q9RZ70	1867						
tr|Q9RZL3	tr|Q9RZL3	1942	82570000	78379000	74497000	38239000	23248000	34653000
tr|Q9RRD4	tr|Q9RRD4	504	28175000	28620000	30455000			
tr|Q9RRE1	tr|Q9RRE1	509			17571000	21257000		
tr|Q9RR98	tr|Q9RR98	481		42419000				
tr|Q9RXF0	tr|Q9RXF0	1581				32088000	34188000	27650000
tr|Q9RT29	tr|Q9RT29	806	1.03E+08	1.1E+08	91983000	68485000	62469000	68450000
tr|Q9RYS8	tr|Q9RYS8	1807	64239000	52000000	54982000	5332800		
sp|Q9RXI	sp|Q9RXI	374	2.34E+08	2.45E+08	2.49E+08	2.7E+08	1.73E+08	2.09E+08
tr|Q9RTB3	tr|Q9RTB3	847	66703000		49294000	30628000		44790000
tr|Q9RSB6	tr|Q9RSB6	684						
tr|Q9RYG3	tr|Q9RYG3	1748	40889000					
tr|Q9RX31	tr|Q9RX31	1518	29486000	38265000	42121000			
tr|Q9RU27	tr|Q9RU27	980	8.12E+08	7.56E+08	6.47E+08	7.82E+08	6.44E+08	7.49E+08
tr|Q9RZC5	tr|Q9RZC5	1904	5.38E+08	3.23E+08	3.28E+08		17232000	
tr|Q9RXG0	tr|Q9RXG0	1587	49631000	77054000	57516000			
tr|Q9RSZ5	sp|Q9RXG370;790						77261000	4542600
tr|Q9RV75	tr|Q9RV75	1189	6822300	3603000		2406300		
tr|Q9RTY8	tr|Q9RTY8	960		5619800	26625000	53971000	60796000	63493000
sp|Q9RXG	sp|Q9RXG	370	9957800	27559000	25823000			33298000
tr|Q9RS40	tr|Q9RS40	640	1.29E+08	1.62E+08	1.57E+08	1.75E+08	1.77E+08	1.84E+08
tr|Q9RVY0	tr|Q9RVY0	1319				56124000	13936000	
sp|Q9RT9	sp|Q9RT9	172	1.02E+08	1.17E+08	1.04E+08	94796000	94275000	1.23E+08

tr|Q9RXF0	tr|Q9RXF0	1581	1.14E+09	1.1E+09	1.15E+09	7.82E+08	5.87E+08	7.84E+08
tr|Q9RRG4	tr|Q9RRG4	520	1.53E+09	2.36E+09	1.76E+09	1.95E+09	1.65E+09	3.83E+08
tr|Q9RVZ2	tr|Q9RVZ2	1325	83883000	67181000	69600000			32435000
sp|Q9RRL	sp|Q9RRL	90	28731000	24957000	31449000	82569000	73557000	88397000
tr|Q9RTG1	tr|Q9RTG1	871	2.49E+08	3.6E+08	2.56E+08			
tr|Q9RWQ	tr|Q9RWQ	1453	1.14E+08		92101000	1.13E+08		93994000
tr|Q9RUB	tr|Q9RUB	1029	32334000	28382000	33246000			
tr|Q9RS80	tr|Q9RS80	660	78804000		53389000	2.43E+08	1.38E+08	1.03E+08
tr|Q9RV86	tr|Q9RV86	1193						
tr|Q9RV86	tr|Q9RV86	1193		17751000				
tr|Q9RYL0	tr|Q9RYL0	1767	3.62E+09	2.83E+09	3.82E+09	1E+09	1.36E+09	1.03E+09
tr|Q9RWW	tr|Q9RWW	1489	8297800	6807500	9794900			3809000
tr|Q9RYQ0	tr|Q9RYQ0	1788	9.89E+08	8.6E+08	1.08E+09	4.74E+08	4.07E+08	5.39E+08
tr|Q9RUD	tr|Q9RUD	1039	10903000		12803000	27451000	24012000	29737000
tr|Q9RXF5	tr|Q9RXF5	1584				1.73E+08	1.32E+08	
sp|Q9RRB	sp|Q9RRB	76					468700	402390
sp|Q9RRB	sp|Q9RRB	76			47701000			11933000
sp|Q9RXJ9	sp|Q9RXJ9	384	2.77E+09	2.14E+09	2.14E+09	2.35E+09	1.84E+09	2.25E+09
sp|Q9RXJ9	sp|Q9RXJ9	384	21191000	27598000	40149000		12799000	14096000
tr|Q9RW8	tr|Q9RW8	1370		31103000	29267000			
tr|Q9RYG5	tr|Q9RYG5	1750						
tr|Q9RW0	tr|Q9RW0	1332	1.14E+10	1.17E+10	9.6E+09	7.01E+09	6.81E+09	8E+09
tr|Q9RU45	tr|Q9RU45	992						
sp|Q9RUN	sp|Q9RUN	240	5004900	5593000		3534600	3805600	3563200
tr|Q9RU30	tr|Q9RU30	982	18128000		19083000			
sp|Q9RYP	sp|Q9RYP	440	2.65E+08	2.41E+08	1.77E+08	1.64E+08	1.89E+08	2.03E+08
tr|Q9RSE1	tr|Q9RSE1	700	3.62E+08	2.62E+08	2.39E+08			73790000
tr|Q9RWE	tr|Q9RWE	1398			13451000			
sp|Q9RYM	sp|Q9RYM	439	1.69E+08	1.4E+08	2.2E+08			
tr|Q9RYF0	tr|Q9RYF0	1742	1.39E+08	2.39E+08	1.78E+08	5.8E+08	4.73E+08	4.76E+08
tr|Q9RTM	tr|Q9RTM	899						
tr|Q9RWC	tr|Q9RWC	1392	1.22E+08	1.21E+08	95408000			
tr|Q9RRT9	tr|Q9RRT9	582	1.67E+08	2.57E+08	1.96E+08	1.62E+08	2.23E+08	1.83E+08
tr|Q9RZC3	tr|Q9RZC3	1902	5.85E+08	6.98E+08	4.59E+08	3.76E+08	3.58E+08	4.22E+08
tr|Q9RSV5	tr|Q9RSV5	771			19570000			
tr|Q9RYB8	tr|Q9RYB8	1722	1.04E+09	9.02E+08	7.56E+08	1.07E+09	8.11E+08	1.04E+09
tr|Q9RUT9	tr|Q9RUT9	1120		87844000		79289000		83729000
tr|Q9RVQ	tr|Q9RVQ	1273	3.18E+08	2.54E+08	6.43E+08	2.11E+09	1.06E+09	1.13E+09
tr|Q9RVQ	tr|Q9RVQ	1273	4481200		20413000			
tr|Q9RUE4	tr|Q9RUE4	1045						
tr|Q9RX81	tr|Q9RX81	1547	23281000	29675000	42054000	81150000	59765000	1.03E+08
sp|Q9RS2	sp|Q9RS2	108			1917700	62491000	74593000	76014000
tr|Q9RRB1	tr|Q9RRB1	492		38157000	35799000	17864000		17185000
tr|Q9RRB1	tr|Q9RRB1	492		4174700	5129100			
tr|Q9RWE	tr|Q9RWE	1399	28222000	14952000	22908000	10296000	15834000	15655000
tr|Q9RYG4	tr|Q9RYG4	1749		25265000	34822000			
sp|Q9RSN	sp|Q9RSN	138		36164000	28984000			
tr|Q9RYC4	tr|Q9RYC4	1725						
tr|Q9RWC	tr|Q9RWC	1388		5149700	5758600			
tr|Q9RVA3	tr|Q9RVA3	1200						
sp|Q9RW9	sp|Q9RW9	345	9327300	14859000	29917000		12328000	

tr|Q9RTN(	tr|Q9RTN(	903	1.36E+08	95402000	1.2E+08			
sp|Q9RYV	sp|Q9RYV	445	2.24E+08	2.74E+08	2.01E+08	2.35E+08	2.11E+08	2.15E+08
sp|Q9RTH	sp|Q9RTH	186	1.07E+08	1.01E+08	1.01E+08	23149000	17139000	22568000
tr|Q9RZQ	tr|Q9RZQ	1959	2.95E+08	3.85E+08	3.8E+08	1.58E+09	1.43E+09	1.33E+09
tr|Q9RWV	tr|Q9RWV	1492			53940000			
sp|Q9RW(	sp|Q9RW(	342	1.92E+08	2.15E+08	1.78E+08	3.74E+08	4.72E+08	3.39E+08
sp|Q9RW(	sp|Q9RW(	342	38772000	41718000	1.26E+08	1.27E+08	2.3E+08	2.13E+08
tr|Q9RYQ'	tr|Q9RYQ'	1789				17500000	27038000	22124000
tr|Q9RRS(	tr|Q9RRS(	572	1.76E+08	2.25E+08	3.64E+08	77502000	1.16E+08	1.2E+08
tr|Q9RZD(	tr|Q9RZD(	1910	1.39E+08	2.27E+08	2.06E+08	99434000	1.11E+08	1.23E+08
tr|Q9RS8(	tr|Q9RS8(	665	4.37E+08	4.17E+08	3.82E+08	3.78E+08	3.18E+08	3.55E+08
tr|Q9RYL0	tr|Q9RYL0	1767	4.2E+09	3.43E+09		2.67E+09		1.76E+09
tr|Q9RYT3	tr|Q9RYT3	1812						
tr|Q9RW0	tr|Q9RW0	1331	25016000	35440000	29370000	50810000	28053000	4737400
tr|Q9RWX	tr|Q9RWX	1497				25981000		
tr|Q9RWS	tr|Q9RWS	1464			11798000	23255000	20052000	
tr|Q9RST1	tr|Q9RST1	757	28687000	71284000	1.3E+08	22811000	47705000	59390000
tr|Q9RS71	tr|Q9RS71	655	10772000	20344000	48231000		10919000	
tr|Q9RY3(	tr|Q9RY3(	1688	2.6E+08	1.97E+08	1.56E+08	2.48E+08	1.75E+08	1.62E+08
tr|Q9RXZ(	tr|Q9RXZ(	1671	15391000					
sp|Q9RYE	sp|Q9RYE	432	20339000	21140000	38600000	16341000	20567000	28782000
tr|Q9RYE8	tr|Q9RYE8	1740						
tr|Q9RSW	tr|Q9RSW	775		22736000	60616000		35922000	
tr|Q9RU4(	tr|Q9RU4(	990		2.11E+08	2.8E+08		73640000	
tr|Q9RVS8	tr|Q9RVS8	1292	3.21E+08	6.46E+08	1.11E+09	4.96E+08	6.67E+08	6.83E+08
tr|Q9RUD	tr|Q9RUD	1040	2.48E+08	2.19E+08	2.26E+08			69099000
tr|Q9RZ0(	tr|Q9RZ0(	1847		11577000	13744000			
tr|Q9RZ0(	tr|Q9RZ0(	1847	6.13E+08	3.95E+08	7.09E+08	73936000	1.07E+08	1.26E+08
tr|Q9RVZ2	tr|Q9RVZ2	1325	1.58E+08	2.24E+08	2.11E+08	77994000	1.02E+08	90083000
sp|Q9RYP	sp|Q9RYP	440						1.32E+08
sp|Q9RYP	sp|Q9RYP	440						
tr|Q9RYF4	tr|Q9RYF4	1743	1.46E+09	1.52E+09	1.28E+09	1.14E+09	1.01E+09	1.14E+09
tr|Q9RYV(	tr|Q9RYV(	1823						1756800
tr|Q9RVL0	tr|Q9RVL0	1250	46134000	46912000	59737000	17545000	17609000	
tr|Q9RT0(	tr|Q9RT0(	794	41156000	59052000	37253000	34351000	52705000	39469000
tr|Q9RUU	tr|Q9RUU	1123	1095700					
tr|Q9RUU	tr|Q9RUU	1123	24118000	32526000	16215000			
tr|Q9RVQ	tr|Q9RVQ	1273	7.42E+08	6.87E+08	7.21E+08	5.16E+08	6.42E+08	7.42E+08
tr|Q9RT7(	tr|Q9RT7(	827	1.21E+09	2.89E+08	6.32E+08			
tr|Q9RVA(	tr|Q9RVA(	1201	70246000	50095000	29461000		32885000	
sp|Q9RRB	sp|Q9RRB	77	5.21E+08	4.13E+08	6.07E+08	2.56E+08	47564000	3.93E+08
sp|Q9RXJ1	sp|Q9RXJ1	376	3.4E+08	2.21E+08	2.48E+08	4.89E+08	5.45E+08	6.17E+08
sp|Q9RXJ1	sp|Q9RXJ1	376	75777000	66855000	1.24E+08		86134000	
tr|Q9RVJ0	tr|Q9RVJ0	1238	31284000	32843000	40045000	1211000	11234000	1062800
tr|Q9RVJ0	tr|Q9RVJ0	1238	1.54E+08	1.36E+08	2.17E+08	4294400	22664000	8970800
tr|Q9RV7(	tr|Q9RV7(	1186	49660000	30963000	95777000			
tr|Q9RU6(	tr|Q9RU6(	1008	2.69E+08	2.75E+08	4.79E+08		1.35E+08	2.53E+08
tr|Q9RRS(	tr|Q9RRS(	577					2321000	
sp|P5692(	sp|P5692(	59	92026000	51499000	76479000	37724000	38961000	64969000
tr|Q9RT5(	tr|Q9RT5(	823						
tr|Q9RV5(	tr|Q9RV5(	1180		19233000				

tr|Q9RTE2	tr|Q9RTE2	861	53902000	37710000	72508000	73465000	1.07E+08	99408000
tr|Q9RU57	tr|Q9RU57	1000			21631000			
tr|Q9RTF2	tr|Q9RTF2	867		14501000	20877000			
tr|Q9RXW	tr|Q9RXW	1657						
sp|Q9RW\	sp|Q9RW\	348						
sp|Q9RWI	sp|Q9RWI	336	2150800	981510	512910	632720	497430	903310
tr|Q9RV11	tr|Q9RV11	1156			6432800	43156000	40442000	8167300
tr|Q9RWY	tr|Q9RWY	1500					22858000	
sp|Q9RTI6	sp|Q9RTI6	188		24574000	25496000			
tr|Q9RYR1	tr|Q9RYR1	1796	2.71E+08	1.68E+08	1.54E+08	1.16E+08	92948000	1.15E+08
tr|Q9RUK9	tr|Q9RUK9	1081						
tr|Q9RUD	tr|Q9RUD	1041	1.14E+08	73268000	73218000	44468000	54860000	45901000
tr|Q9RY43	tr|Q9RY43	1693						
tr|Q9RZB2	tr|Q9RZB2	1895	1.21E+08	1E+08	86332000	1.42E+08	1.18E+08	1.09E+08
tr|Q9RVL6	tr|Q9RVL6	1253	1.02E+08	70748000	86171000			
tr|Q9RY75	tr|Q9RY75	1705				34537000		
sp|Q9RZ8	sp|Q9RZ8	453	79129000	20349000				
tr|Q9RXD5	tr|Q9RXD5	1573	2.4E+09	2.22E+09	1.64E+09	3.08E+09	4.11E+09	3.16E+09
sp|Q9RS2	sp|Q9RS2	108	8.17E+08	7.37E+08	1.39E+09	32512000	3.82E+08	6.17E+08
tr|Q9RSH0	tr|Q9RSH0	716	1.21E+08	1.08E+08	1.35E+08		57446000	54303000
tr|Q9RRI3	tr|Q9RRI3	531						
tr|Q9RWI0	tr|Q9RWI0	1413	30591000	22443000	34053000	45446000	43068000	40391000
sp|Q9RYM	sp|Q9RYM	438	1.09E+08	1.43E+08	91706000	2.18E+08	1.82E+08	1.65E+08
sp|Q9RYM	sp|Q9RYM	438	1.54E+09	1.17E+09	1.63E+09	1.63E+09	1.67E+09	2.21E+09
sp|Q9RYM	sp|Q9RYM	438				49355000	56579000	52148000
sp|Q9RUY	sp|Q9RUY	259	68363000	61880000	58833000	1.4E+08	1.5E+08	1.36E+08
sp|Q9RUY	sp|Q9RUY	259	2.45E+08	2.84E+08	4.08E+08	1.69E+08	2.87E+08	3.21E+08
sp|Q9RV2	sp|Q9RV2	264	9.81E+08	9.45E+08	8.82E+08	1.63E+09	1.45E+09	1.4E+09
sp|Q9RV2	sp|Q9RV2	264	1.16E+08	1.41E+08	2.27E+08	46430000		78917000
tr|Q9RUU	tr|Q9RUU	1122						
tr|Q9RZT5	tr|Q9RZT5	1972			21135000	29585000	27239000	28380000
sp|Q9RZ0	sp|Q9RZ0	452	77082000		1.31E+08			1.06E+08
tr|Q9RVG6	tr|Q9RVG6	1228						
sp|Q9RXJ1	sp|Q9RXJ1	376		5920700	4890600			
sp|Q9RWI	sp|Q9RWI	338			7337400		10141000	8399500
tr|Q9RUI9	tr|Q9RUI9	1066	1.68E+08	1.28E+08	2.32E+08		50523000	69967000
sp|Q9RW9	sp|Q9RW9	317	1.79E+08	1.25E+08	1.66E+08			
tr|Q9RVM	tr|Q9RVM	1256	67756000	20953000	32539000	30295000	66610000	67154000
tr|Q9RUD	tr|Q9RUD	1040	55872000	46065000	77067000	55678000	66735000	71747000
tr|Q9RX19	tr|Q9RX19	1514	77231000	63652000	69083000	79195000	69225000	72472000
sp|Q9RSR	sp|Q9RSR	144	6.53E+08	6.91E+08	4.87E+08	6.52E+08	6.57E+08	6.7E+08
sp|Q9RSS	sp|Q9RSS	149	1.97E+08	3.12E+08	4.89E+08	1.98E+08	2.72E+08	4.63E+08
tr|Q9RUA	tr|Q9RUA	1023		49460000	1.27E+08	76692000	1.17E+08	45952000
tr|Q9RVQ	tr|Q9RVQ	1273						
tr|Q9RTL3	tr|Q9RTL3	894						
tr|Q9RZT2	tr|Q9RZT2	1969	32234000					
tr|Q9RZG2	tr|Q9RZG2	1925	2.16E+09	1.33E+09	1.25E+09			
sp|Q9RWI	sp|Q9RWI	339	1.87E+08	1.73E+08	1.36E+08	45611000	90658000	54730000
tr|Q9RYQ0	tr|Q9RYQ0	1788	9.37E+08	6.69E+08	9.22E+08	3.7E+08	3.5E+08	4.16E+08
tr|Q9RTZ9	tr|Q9RTZ9	967	1.28E+08	85757000	92888000	2.6E+08	3.02E+08	2.16E+08
tr|Q9RRV4	tr|Q9RRV4	591	2.57E+08	1.67E+08	1.74E+08	1E+08	1.41E+08	1.3E+08

tr|Q9RYF8	tr|Q9RYF8	1745						
tr|Q9RXX3	tr|Q9RXX3	1661	4.65E+08	1.33E+08				
tr|Q9RSM	tr|Q9RSM	737						
tr|Q9RXG3	tr|Q9RXG3	1589						
tr|Q9RVV0	tr|Q9RVV0	1304	64607000		64288000			
tr|Q9RVB3	tr|Q9RVB3	1207	57911000	46664000	55286000		20186000	
tr|Q9RYQ0	tr|Q9RYQ0	1788			37646000			
sp|Q9RSJ6	sp|Q9RSJ6	124					5150300	
sp|Q9RU8	sp|Q9RU8	216		23781000	39526000	45869000	54876000	57609000
sp|Q9RYM	sp|Q9RYM	439	9.53E+08	8.94E+08	9.27E+08	2.97E+09	1.17E+09	2.24E+09
sp|Q9RYM	sp|Q9RYM	439	1.83E+08	2.38E+08	9.04E+08	1.51E+09	1.77E+09	1.62E+09
tr|Q9RSF1	tr|Q9RSF1	707						
tr|Q9RYI3	tr|Q9RYI3	1761						
sp|Q9RRB	sp|Q9RRB	76	1.52E+09	1.97E+09	1.43E+09	9.85E+08	6.74E+08	9.53E+08
tr|Q9RS0C	tr|Q9RS0C	618	39733000	49604000	29541000	47816000	66008000	
tr|Q9RU08	tr|Q9RU08	973	70882000	1.8E+08	1.75E+08	59237000	2.58E+08	1.59E+08
tr|Q9RW8	tr|Q9RW8	1367		33687000	43889000	17190000	51367000	28979000
tr|Q9RWX	tr|Q9RWX	1498		22723000	22461000		30721000	23608000
tr|Q9RZQ	tr|Q9RZQ	1960						
tr|Q9RZT0	tr|Q9RZT0	1968						
tr|Q9RT71	tr|Q9RT71	828	4.64E+08	3.5E+08	3.69E+08	4.68E+08	4.23E+08	4.08E+08
tr|Q9RW8	tr|Q9RW8	1366	62579000	57987000	55845000	64141000	55854000	64333000
sp|Q9RX3	sp|Q9RX3	356						
tr|Q9RX72	tr|Q9RX72	1542	7668600	3276600	2521100			
tr|Q9RZ10	tr|Q9RZ10	1848	1.95E+08	1.18E+08	1.12E+08	1.33E+08	1.95E+08	1.5E+08
tr|Q9RSU7	tr|Q9RSU7	767	4.82E+08	3.75E+08	5.76E+08	1.86E+08	2.87E+08	2.68E+08
tr|Q9RV75	tr|Q9RV75	1189	2624400	1725200				
sp|Q9RT9	sp|Q9RT9	172	9922500	14684000	13818000	30537000	25614000	
tr|Q9RTR2	tr|Q9RTR2	921						23582000
sp|Q9RUR	sp|Q9RUR	249	2.05E+08	2.09E+08	1.42E+08	5.98E+08	5.07E+08	4.82E+08
sp|Q9RUR	sp|Q9RUR	249					51991000	40651000
sp|Q9RUP	sp|Q9RUP	245	3.02E+08	2.67E+08	2.64E+08	3.67E+08	3.34E+08	3.32E+08
tr|Q9RYL8	tr|Q9RYL8	1771	77721000	93393000	87328000	68647000	1.36E+08	63188000
sp|Q9RSJ6	sp|Q9RSJ6	124	5.87E+08	6.67E+08	7.49E+08	4.76E+08	6.19E+08	4.48E+08
tr|Q9RZA5	tr|Q9RZA5	1890						
sp|P56925	sp|P56925	58						
tr|Q9RZP2	tr|Q9RZP2	1952				22003000	14187000	
sp|Q9RTY	sp|Q9RTY	202	26704000	22739000	25878000	77606000	1.12E+08	1.25E+08
sp|Q9RR9	sp|Q9RR9	73	29298000				11189000	
tr|Q9RSD6	tr|Q9RSD6	697			32998000			
tr|Q9RSL8	tr|Q9RSL8	734	70218000	46188000	93272000	58158000	23782000	26863000
tr|Q9RVS6	tr|Q9RVS6	1291		21564000		46537000	39726000	34940000
tr|Q9RV45	tr|Q9RV45	1172	1.35E+08	1.78E+08	3.43E+08	8.63E+08	1.07E+09	8.59E+08
tr|Q9RV45	tr|Q9RV45	1172				76286000	60441000	40965000
tr|Q9RSM	tr|Q9RSM	735			917920			
tr|Q9RV32	tr|Q9RV32	1168						
tr|Q9RSY6	tr|Q9RSY6	784	1.6E+09	1.81E+09	1.21E+09	2.51E+09	1.88E+09	2.25E+09
sp|Q9RRX	sp|Q9RRX	101	36263000	67438000	53250000	80420000	65912000	85591000
tr|Q9RSQ4	tr|Q9RSQ4	748	49212000		46208000			
sp|Q9RYM	sp|Q9RYM	438				34501000	36843000	
sp|Q9RTF5	sp|Q9RTF5	182	80483000	67112000	95006000	66040000	75647000	39821000

tr|Q9RXF0	tr|Q9RXF0	1581	3.87E+09	2.76E+09	3.3E+09	2.99E+09	2.61E+09	2.94E+09
tr|Q9RS66	tr|Q9RS66	653	6345600	10839000	7986500	28335000	26384000	21986000
tr|Q9RSD6	tr|Q9RSD6	697			18844000	13440000	12487000	7806800
sp|Q9RY5	sp|Q9RY5	417						
sp|Q9RY5	sp|Q9RY5	417			80365000		52523000	
sp|Q9RYM	sp|Q9RYM	438			23176000		38832000	34480000
tr|Q9RR95	tr|Q9RR95	479					50824000	
sp|O83030	sp|O83030	48	83039000	74073000	72491000	64737000	61325000	65657000
sp|Q9RWU	sp|Q9RWU	347	96086000	76687000	77916000	1.71E+08	1.33E+08	1.44E+08
sp|Q9RWN	sp|Q9RWN	337						
tr|Q9RYN8	tr|Q9RYN8	1781		15383000	17717000		16455000	22534000
sp|Q9RU2	sp|Q9RU2	209		12155000	2.07E+08	1.03E+09	1.08E+09	1.08E+09
sp|P56926	sp|P56926	59					14897000	11458000
sp|Q59337	sp|Q59337	63	65204000	37771000	91486000	25656000		
tr|Q9RUQ	tr|Q9RUQ	1103	1.35E+08	1.41E+08	2.02E+08	1.55E+08	1.6E+08	1.09E+08
tr|Q9RWI0	tr|Q9RWI0	1413					17059000	
sp|Q9RSS9	sp|Q9RSS9	150	13687000	9462300	8296300	9751000	9121100	9448700
tr|Q9RTG7	tr|Q9RTG7	873						
tr|Q9RTG7	tr|Q9RTG7	873						
tr|Q9RZS3	tr|Q9RZS3	1964					11788000	
tr|Q9RRA4	tr|Q9RRA4	487	1.58E+08	1.87E+08	1.23E+08	1.63E+08	1.51E+08	1.49E+08
tr|Q9RYN8	tr|Q9RYN8	1781				39411000	21023000	
sp|Q9RUP	sp|Q9RUP	243	2.21E+08	1.83E+08	1.39E+08	1.4E+08		1.64E+08
sp|Q9RRQ	sp|Q9RRQ	95				1.97E+08	1.7E+08	1.85E+08
tr|Q9RVS2	tr|Q9RVS2	1288	1.87E+08	1.6E+08	1.42E+08	80178000	65581000	67811000
tr|Q9RWL	tr|Q9RWL	1431	93073000	97774000	92334000		37940000	33708000
tr|Q9RTC7	tr|Q9RTC7	853		40859000	40152000	1.17E+08	67786000	92301000
sp|Q9RU8	sp|Q9RU8	222				41017000	52694000	
tr|Q9RWP	tr|Q9RWP	1449		26181000	19564000			13122000
tr|Q9RUZ5	tr|Q9RUZ5	1146						
sp|Q9RXK	sp|Q9RXK	389		32885000		52613000		
sp|Q9RRC	sp|Q9RRC	79	2.39E+08	1308700	3.23E+08	9.95E+08		6.93E+08
tr|Q9RZ22	tr|Q9RZ22	1854	27752000	24062000	19162000	78295000	76091000	73575000
sp|Q9RY5	sp|Q9RY5	418						1075700
sp|P52153	sp|P52153	52	68129000	56862000	44819000	73462000	1.04E+08	77461000
tr|Q9RTW	tr|Q9RTW	942	1.84E+08	3.34E+08	1.63E+08	99037000	1.57E+08	1.36E+08
tr|Q9RU91	tr|Q9RU91	1015	16382000	20260000		82302000	54566000	48374000
tr|Q9RS71	tr|Q9RS71	655	29015000	19821000		48872000	36713000	33496000
tr|Q9RS71	tr|Q9RS71	655			18255000		25342000	25115000
sp|Q9RTA	sp|Q9RTA	174	67362000	78713000	55661000	84023000	79010000	
sp|Q9RR8	sp|Q9RR8	70					11300000	9570800
sp|Q9RSJ9	sp|Q9RSJ9	127				16089000	14834000	13667000
sp|Q9RV2	sp|Q9RV2	263		7817500	10078000	77826000	59263000	26268000
tr|Q9RSH1	tr|Q9RSH1	717	2.39E+08	2.28E+08	2.2E+08	3.92E+08	3.47E+08	6.57E+08
tr|Q9RSY6	tr|Q9RSY6	784						
tr|Q9RYN3	tr|Q9RYN3	1777	44275000		39625000	45669000		46933000
sp|Q9RUH	sp|Q9RUH	236				49189000		
tr|Q9RTX7	tr|Q9RTX7	952	25346000	39555000	66292000	42524000		24561000
sp|Q9RYE	sp|Q9RYE	433						
tr|Q9RTH3	tr|Q9RTH3	876						
sp|Q9RWU	sp|Q9RWU	347	91111000	1.73E+08	1.38E+08	4.09E+08	3.9E+08	2.9E+08

tr|Q9RWB	tr|Q9RWB	1386			17877000			
tr|Q9RYS1	tr|Q9RYS1	1801	59978000	47915000	48844000	19346000	30424000	25068000
sp|Q9RXK	sp|Q9RXK	389						
tr|Q9RZ07	tr|Q9RZ07	1845	3.28E+08	3.56E+08	2.96E+08			
tr|Q9RXP4	tr|Q9RXP4	1624			12921000	28252000		12765000
sp|Q9RTB	sp|Q9RTB	176	1.18E+08	1.15E+08	90555000	1.98E+08		1.39E+08
tr|Q9RVG6	tr|Q9RVG6	1228		18896000		42909000	33327000	30819000
tr|Q9RTD6	tr|Q9RTD6	859						
tr|Q9RZP7	tr|Q9RZP7	1955		6318400				
sp|Q9RRB	sp|Q9RRB	76	38174000	22533000	20282000	16954000	19275000	18959000
tr|Q9RXX2	tr|Q9RXX2	1660	16695000					
sp|P56867	sp|P56867	56	7.06E+08	7.93E+08	7.45E+08	1.15E+09	6.09E+08	9.16E+08
tr|Q9RW9	tr|Q9RW9	1373	12777000				16814000	
tr|Q9RV15	tr|Q9RV15	1159						
tr|Q9RYG0	tr|Q9RYG0	1746				25839000	33874000	
sp|Q9RVE	sp|Q9RVE	284	36160000	22225000	9799600	2.86E+08	1.4E+08	1.17E+08
tr|Q9RYT3	tr|Q9RYT3	1812				11234000		
tr|Q9RYI7	tr|Q9RYI7	1763	6901800	3067500	2731700	1485100	2042100	1568800
sp|Q9RXK	sp|Q9RXK	389	3.38E+08	6.98E+08	5.97E+08	8.48E+08	1.41E+09	8.93E+08
tr|Q9RV30	tr|Q9RV30	1166						
tr|Q9RYV3	tr|Q9RYV3	1821	4.84E+08	5.93E+08	1.01E+09	4.56E+08	6.81E+08	5.72E+08
tr|Q9RXB0	tr|Q9RXB0	1561	2.53E+08	4.81E+08	4.64E+08	4.12E+08	5.82E+08	5.03E+08
tr|Q9RU54	tr|Q9RU54	997	82437000	33791000	34392000	32155000	46861000	47837000
tr|Q9RU54	tr|Q9RU54	997	2.48E+08	4.38E+08	7.47E+08	2.35E+08	3.4E+08	3.73E+08
tr|Q9RX56	tr|Q9RX56	1531	2.34E+08	4.35E+08	3.86E+08	2.36E+09	2.06E+09	5.86E+08
tr|Q9RU46	tr|Q9RU46	993						
sp|Q9RWU	sp|Q9RWU	347	26501000			27743000	16135000	
tr|Q9RYC0	tr|Q9RYC0	1723						
tr|Q9RWT	tr|Q9RWT	1469	1.05E+09	3.34E+08	3.71E+08	1.4E+09	52352000	7.36E+08
tr|Q9RU57	tr|Q9RU57	1000	19939000	22569000	39840000	23120000	22896000	46278000
tr|Q9RTW	tr|Q9RTW	945	69675000	59585000	65892000	1.96E+08	1.8E+08	1.65E+08
sp|Q9RVH	sp|Q9RVH	288		42631000	46084000	21062000	23963000	26194000
tr|Q9RUI9	tr|Q9RUI9	1066	99814000	1.49E+08	1.47E+08	2.1E+08	2.53E+08	1.6E+08
tr|Q9RZC5	tr|Q9RZC5	1904	6.62E+08	6.04E+08	8.17E+08			
tr|Q9RTU7	tr|Q9RTU7	935	61752000	47969000	55416000	34936000	25383000	43204000
tr|Q9RRS5	tr|Q9RRS5	576			13068000	22374000	14026000	13301000
tr|Q9RT35	tr|Q9RT35	811						
tr|Q9RT35	tr|Q9RT35	811	51646000	53770000	69606000		32432000	30011000
tr|Q9RZL4	tr|Q9RZL4	1943		22289000				
sp|Q9RW7	sp|Q9RW7	314	71585000	1.07E+08	99593000	84275000		82960000
sp|Q9RW7	sp|Q9RW7	314		26547000	46330000	16783000	43236000	28395000
tr|Q9RRE1	tr|Q9RRE1	509	1.17E+08	1.19E+08	1.41E+08	37503000	41432000	30666000
sp|Q9RUV	sp|Q9RUV	252	52032000	71976000	61614000	56130000	84053000	74518000
tr|Q9RXD2	tr|Q9RXD2	1572	1.98E+08	2.53E+08	3.44E+08	2.06E+08	1.57E+08	1.46E+08
sp|Q9RWE	sp|Q9RWE	321	2.9E+09	3.57E+09	3.23E+09	2.19E+09	2.28E+09	2.56E+09
sp|Q9RWE	sp|Q9RWE	321		13001000	28384000			
sp|Q9RRB	sp|Q9RRB	77	4041700	5250500	11152000	38612000	17301000	23938000
tr|Q9RYL3	tr|Q9RYL3	1770	68804000	95272000	93377000			
tr|Q9RVF4	tr|Q9RVF4	1223	26312000		45595000			
sp|Q9RSQ	sp|Q9RSQ	140	1.73E+08	1.78E+08	1.75E+08	19393000	66826000	1.04E+08
sp|Q9RX3	sp|Q9RX3	356				22304000	20834000	20328000

sp|Q9RT7	sp|Q9RT7	171	2.16E+08	1.95E+08	1.69E+08	1.85E+08	1.74E+08	2.01E+08
tr|Q9RXH	tr|Q9RXH	1595	42211000	94999000	75767000	1.68E+08	1.65E+08	44229000
tr|Q9RSA	tr|Q9RSA	675	10669000			18476000		
sp|Q9RXZ	sp|Q9RXZ	409						
tr|Q9RV4	tr|Q9RV4	1172	2.41E+08	41409000	2.57E+08	1.8E+08	2.37E+08	1.76E+08
tr|Q9RYN	tr|Q9RYN	1778		25346000				
tr|Q9RS1	tr|Q9RS1	626		34841000	23648000	23319000	28494000	19424000
tr|Q9RS1	tr|Q9RS1	626		41318000	15968000			
tr|Q9RXX	tr|Q9RXX	1660			49868000			
tr|Q9RU5	tr|Q9RU5	995						1.01E+08
sp|Q9RYL	sp|Q9RYL	436	37396000	28784000	35679000			
tr|Q9RST2	tr|Q9RST2	758	41130000	59083000	42150000	92466000	88457000	73751000
sp|Q9RW	sp|Q9RW	342	5057300					
tr|Q9RZD	tr|Q9RZD	1910				19116000	23536000	26815000
tr|Q9RUU	tr|Q9RUU	1121	6471400	4055600	4081200		2121400	
tr|Q9RR8	tr|Q9RR8	474	35388000		24106000		21786000	35614000
sp|Q9RVV	sp|Q9RVV	300		16562000	83171000	50560000	47216000	1.08E+08
tr|Q9RXS7	tr|Q9RXS7	1640	9151400					
sp|Q9RYW	sp|Q9RYW	446	64730000	65010000	77265000	43382000	65930000	1.06E+08
tr|Q9RX7	tr|Q9RX7	1542			1.66E+08	1.18E+08	1.71E+08	
tr|Q9RYZ	tr|Q9RYZ	1840						
tr|Q9RYZ	tr|Q9RYZ	1840						
tr|Q9RYW	tr|Q9RYW	1828	63708000	21402000	18356000			18054000
sp|Q9RW	sp|Q9RW	311						
tr|Q9RYG	tr|Q9RYG	1752	91051000	92079000	63915000	91617000	98247000	89595000
tr|Q9RT4	tr|Q9RT4	816				41414000	49868000	58950000
sp|Q9RTJ	sp|Q9RTJ	189	77181000	78472000	1.23E+08	1.69E+08	1.52E+08	1.45E+08
tr|Q9RUS	tr|Q9RUS	1112						
tr|Q9RWB	tr|Q9RWB	1384	66975000	49670000	60021000			
tr|Q9RU6	tr|Q9RU6	1004						11627000
tr|Q9RYY	tr|Q9RYY	1839	2.86E+08	2.19E+08	1.61E+08	2.52E+08	2.31E+08	2.16E+08
tr|Q9RRK	tr|Q9RRK	540				19443000	14439000	
tr|Q9RTZ	tr|Q9RTZ	967	14576000	19104000	14512000	12708000		17088000
tr|Q9RRG	tr|Q9RRG	520	3.16E+08	3.08E+08	2.82E+08	5.61E+08	4.04E+08	4.26E+08
tr|Q9RRG	tr|Q9RRG	520	42182000	28158000	85532000			
tr|Q9RU6	tr|Q9RU6	1008	57964000	8159000	68637000	1.41E+08		1.3E+08
sp|Q9RS2	sp|Q9RS2	108	23834000	8574400	9692100	6813600	8001000	7411000
tr|Q9RSY	tr|Q9RSY	784				57361000	15613000	27301000
tr|Q9RVA	tr|Q9RVA	1202	8.79E+08	9.41E+08	1.23E+09	1.76E+08	2.19E+08	2.65E+08
sp|Q9RU7	sp|Q9RU7	215	3.56E+09	3.42E+09	2.9E+09	5.81E+09	4.98E+09	5.93E+09
tr|Q9RVR	tr|Q9RVR	1284	72818000	73785000	55565000	1.93E+08	1.68E+08	1.53E+08
tr|Q9RVR	tr|Q9RVR	1284			25895000			
tr|Q9RYT	tr|Q9RYT	1816	1.9E+08	1.38E+08	2.11E+08	1.55E+08	1.68E+08	2.08E+08
tr|Q9RVW	tr|Q9RVW	1311	11465000	6703200	5334400	7215900	11251000	8606900
tr|Q9RXN	tr|Q9RXN	1621		24491000	16795000			
tr|Q9RYS	tr|Q9RYS	1807	9.56E+08	7.99E+08	7.85E+08	7.21E+08	6.28E+08	7.38E+08
tr|Q9RZE4	tr|Q9RZE4	1914				42695000	61075000	65423000
tr|Q9RWX	tr|Q9RWX	1497						
sp|Q9RTN	sp|Q9RTN	196	93728000	96849000	1.37E+08	1.19E+08	1.43E+08	1.59E+08
sp|Q9RTN	sp|Q9RTN	196		73599000	3.02E+08	1.21E+08	2.15E+08	1.74E+08
tr|Q9RVH	tr|Q9RVH	1235	55384000		57862000	62937000	63884000	70115000

tr|Q9RUX(	tr|Q9RUX(	1129						38730000
sp|Q9RSQ	sp|Q9RSQ141;551							
tr|Q9RS63	tr|Q9RS63	652	6813800	9766800		66399000	73098000	61372000
sp|Q9RSS	sp|Q9RSS	145	25044000	18402000		27294000	31575000	27967000
tr|Q9RYT8	tr|Q9RYT8	1816	1.75E+08	1.17E+08	2.41E+08	95503000	81605000	1.35E+08
tr|Q9RRI9	tr|Q9RRI9	535	5130900		5245200	14782000	8908800	
tr|Q9RYY7	tr|Q9RYY7	1837		12690000				
tr|Q9RT68	tr|Q9RT68	826				1314400	605060	1136100
sp|Q9RSR	sp|Q9RSR	143	5.72E+08	5.31E+08	4.82E+08	5.45E+08	4.87E+08	5.27E+08
tr|Q9RS29	tr|Q9RS29	636	71400000	20712000	21748000	19811000	21560000	28577000
tr|Q9RWW	tr|Q9RWW	1491	41693000	36633000	38652000	24953000	33117000	27167000
tr|Q9RY54	tr|Q9RY54	1696	48670000	61967000	33917000	38402000	35420000	37028000
tr|Q9RW0	tr|Q9RW0	1332		5040700		4173900	7585200	
tr|Q9RW0	tr|Q9RW0	1332			40689000	86037000	1.16E+08	1.38E+08
tr|Q9RRZ(	tr|Q9RRZ(	611	1.54E+08	1.76E+08		1.1E+08	88293000	98950000
tr|Q9RX16	tr|Q9RX16	1511	27437000	20415000	22626000	15534000	19221000	
tr|Q9RS32	tr|Q9RS32	638	49065000	92613000	47981000	26683000	31512000	29019000
tr|Q9RW7	tr|Q9RW7	1362		41161000				
sp|Q9RYW	sp|Q9RYW	446	2.63E+08			1.62E+08	1.35E+08	1.58E+08
tr|Q9RT02	tr|Q9RT02	793						
tr|Q9RVN	tr|Q9RVN	1264			58744000	30655000	51369000	26721000
tr|Q9RZQ	tr|Q9RZQ	1959	1.35E+08	1.62E+08	1.58E+08	4.01E+08	3.11E+08	3.12E+08
tr|Q9RZQ	tr|Q9RZQ	1959	2.65E+08	2.31E+08	4.84E+08	2.17E+08	2.12E+08	2.93E+08
sp|Q9RSI4	sp|Q9RSI4	121						
tr|Q9RVK9	tr|Q9RVK9	1249	14563000	21802000	15663000		1.03E+08	
tr|Q9RZ8(	tr|Q9RZ8(	1874	4.65E+08	4.21E+08	3.24E+08	3.85E+08	3.26E+08	3.79E+08
tr|Q9RUD	tr|Q9RUD	1043	40948000	24539000	2.5E+08	72209000	71024000	73607000
tr|Q9RZV9	tr|Q9RZV9	1985	50420000	33188000	35520000	97521000	64623000	78697000
sp|Q9RSR	sp|Q9RSR	143	5.19E+08	6.64E+08	5.44E+08	4.83E+08	5.99E+08	4.35E+08
tr|Q9RRA6	tr|Q9RRA6	489	1.94E+09	1.71E+09	1.91E+09	3.13E+09	3.06E+09	2.69E+09
tr|Q9RRA6	tr|Q9RRA6	489				39393000	76107000	
tr|Q9RTX7	tr|Q9RTX7	952	8.63E+08	7.53E+08	8.09E+08	1.17E+08	2.25E+08	1.22E+08
tr|Q9RTX5	tr|Q9RTX5	950	1.16E+09	1.04E+09	1.02E+09	3.75E+08	3.57E+08	3.52E+08
tr|Q9RZC4	tr|Q9RZC4	1903						
sp|Q9RU8	sp|Q9RU8	220	1.5E+08	1.49E+08	1.22E+08	1.15E+08		1.02E+08
tr|Q9RVQ	tr|Q9RVQ	1279	3541200	798960	998570		578390	
tr|Q9RYM	tr|Q9RYM	1773	9.43E+08	8.76E+08	6.26E+08	6.12E+08	5.39E+08	6.16E+08
tr|Q9RY87	tr|Q9RY87	1711	7521000	6211100	6238600	9803700		
sp|Q9RUG	sp|Q9RUG	235						
sp|Q9RXK	sp|Q9RXK	390	5.99E+09	4.9E+09	4.39E+09	3.37E+09	3.1E+09	3.11E+09
sp|Q9RXK	sp|Q9RXK	390		27348000	48134000	18095000	23438000	
tr|Q9RV41	tr|Q9RV41	1171				71989000	48744000	88839000
tr|Q9RTY1	tr|Q9RTY1	956	1.13E+08	1.98E+08	1.64E+08		1.33E+08	1.13E+08
tr|Q9RZ16	tr|Q9RZ16	1852	50435000	81403000	42245000	73315000	87831000	86849000
tr|Q9RRA1	tr|Q9RRA1	484						
tr|Q9RU5(	tr|Q9RU5(	994	17946000	3.61E+08	2.62E+08	49723000		96903000
tr|Q9RT87	tr|Q9RT87	837	9.98E+08	8.18E+08	7.33E+08	1.1E+09	1.07E+09	1.02E+09
tr|Q9RUY2	tr|Q9RUY2	1139	8133600	3945000	4637800	2378200	5016200	2955800
tr|Q9RUY2	tr|Q9RUY2	1139		1.22E+08	2.05E+08		26870000	
tr|Q9RUZ2	tr|Q9RUZ2	1145						
tr|Q9RXM	tr|Q9RXM	1618	11205000		14479000			

tr|Q9RWL	tr|Q9RWL	1435			11723000			
tr|Q9RXA9	tr|Q9RXA9	1560		23929000	17508000	24836000	23599000	17221000
tr|Q9RRT7	tr|Q9RRT7	581			11984000		11411000	4000700
tr|Q9RY83	tr|Q9RY83	1709	15543000	17688000	12542000	1.14E+08		55657000
sp|Q9RX1	sp|Q9RX1	352						
tr|Q9RXI4	tr|Q9RXI4	1603			28786000			
tr|Q9RSH6	tr|Q9RSH6	718	1.58E+08	1.2E+08	1.2E+08	1.52E+08	1.22E+08	1.29E+08
sp|Q9RS1	sp|Q9RS1	106			18894000	20278000		
tr|Q9RSP0	tr|Q9RSP0	743	1.68E+08	1.15E+08	1.33E+08	1.87E+08	1.71E+08	1.91E+08
tr|Q9RYP8	tr|Q9RYP8	1786	51450000	17064000	58154000			
sp|Q9RZU	sp|Q9RZU	458						
tr|Q9RTP0	tr|Q9RTP0	906	81976000	1.28E+08	74138000	2.66E+08	2.76E+08	2.21E+08
tr|Q9RTP0	tr|Q9RTP0	906	32934000		79740000			
tr|Q9RRD6	tr|Q9RRD6	506	2483000	1517600	1327700			
tr|Q9RUT4	tr|Q9RUT4	1118	1.94E+08	1.57E+08	1.81E+08	64546000	81623000	51858000
sp|Q9RWU	sp|Q9RWU	347	82862000	1.12E+08	98298000	34286000		33510000
sp|Q9RR6	sp|Q9RR6	65	1.47E+08	60151000	56434000	34203000	55296000	36904000
sp|P42443	sp|P42443	49						
tr|Q9RYV4	tr|Q9RYV4	1822	5.73E+08	5.7E+08	5.16E+08	4.99E+08	6.09E+08	4.9E+08
tr|Q9RWU	tr|Q9RWU	1479		18053000	27424000	26076000	28910000	23995000
tr|Q9RWV	tr|Q9RWV	1485	1.6E+08	2.22E+08	2.66E+08	1.17E+09	5.17E+08	3.1E+08
tr|Q9RVG6	tr|Q9RVG6	1228	3.73E+09	3.48E+09	3.04E+09	4.62E+09	4.42E+09	4.7E+09
tr|Q9RVG6	tr|Q9RVG6	1228			82091000			
tr|Q9RS18	tr|Q9RS18	631						
tr|Q9RTG7	tr|Q9RTG7	873	1.57E+08	91271000	1.44E+08	5.38E+08	4.59E+08	4.93E+08
sp|Q5933	sp|Q5933	63	1.38E+10	1.34E+10	9.62E+09	7.53E+09	7.37E+09	7.35E+09
sp|Q9RTS	sp|Q9RTS	199	1.08E+08	1.16E+08	1.07E+08	2.32E+08	2.31E+08	2.22E+08
tr|Q9RWY	tr|Q9RWY	1502	24781000					
sp|Q9RSR	sp|Q9RSR	143	3.35E+09	3.24E+09	2.63E+09	2.31E+09	2.36E+09	2.71E+09
tr|Q9RWU	tr|Q9RWU	1476						
tr|Q9RYE4	tr|Q9RYE4	1738	3.98E+08	2.8E+08	2.52E+08	90548000	1.07E+08	1.18E+08
tr|Q9RV21	tr|Q9RV21	1162	41516000	52250000	30781000	84856000	84790000	78616000
sp|Q9RSJ6	sp|Q9RSJ6	124	430440		112840			189740
sp|Q9RSJ6	sp|Q9RSJ6	124	1.04E+08	1.62E+08	2.62E+08	1.13E+08	2.68E+08	1.35E+08
sp|Q9RVV	sp|Q9RVV	300	42392000	34121000	28152000	1.64E+08	1.84E+08	1.43E+08
sp|Q9RVV	sp|Q9RVV	300			490740			
tr|Q9RTZ9	tr|Q9RTZ9	967					46092000	54967000
sp|Q9RRP	sp|Q9RRP	93			19336000			
sp|Q9RVC	sp|Q9RVC	277			14297000			
tr|Q9RZ24	tr|Q9RZ24	1856			17425000			
tr|Q9RXZ9	tr|Q9RXZ9	1672	14016000	34301000	10611000	11053000	10649000	
tr|Q9RTC3	tr|Q9RTC3	850	29548000	23430000	24407000			
tr|Q9RTG9	tr|Q9RTG9	874						
tr|Q9RSF8	tr|Q9RSF8	710			3003900		6636900	
tr|Q9RU69	tr|Q9RU69	1008	76066000	77641000	72337000	1.22E+08	1.15E+08	1.1E+08
sp|Q9RU8	sp|Q9RU8	217	6714800	2671800	1848900	1300500	2271700	1973600
tr|Q9RWU	tr|Q9RWU	1476	99105000	41829000				
sp|Q4657	sp|Q4657	61	64351000	38894000	35372000		17308000	
tr|Q9RY03	tr|Q9RY03	1676						
tr|Q9RSJ4	tr|Q9RSJ4	729	5720200		3076300			
sp|Q9RRB	sp|Q9RRB	76	2575100	741270	506390			

tr|Q9RU0	tr|Q9RU0	969	11632000	8422100	6040400	5506000	10006000	6986300
tr|Q9RZE4	tr|Q9RZE4	1914	4.94E+08	5.45E+08	4.77E+08	7.94E+08	1.05E+09	9.03E+08
tr|Q9RRF7	tr|Q9RRF7	517	67434000	97134000	81499000	58801000	56377000	43050000
sp|Q9RX2	sp|Q9RX2	355			8470100			
sp|Q9RWH	sp|Q9RWH	330						
sp|Q9RYM	sp|Q9RYM	438	3.54E+08	9.46E+08	2.44E+09	2.94E+09	3.54E+09	2.93E+09
tr|Q9RYP6	tr|Q9RYP6	1785	10488000					
sp|Q9RUV	sp|Q9RUV	258			25331000	10124000		
tr|Q9RXS0	tr|Q9RXS0	1637	29448000	24741000	24058000	20680000	22681000	19084000
sp|Q9RWN	sp|Q9RWN	340	14082000	26739000	48794000	10554000	20321000	17599000
tr|Q9RZT5	tr|Q9RZT5	1972						
tr|Q9RSR2	tr|Q9RSR2	750	15774000					
sp|Q9RYB	sp|Q9RYB	427		10835000	9105100		9085200	10633000
tr|Q9RUK0	tr|Q9RUK0	1075						
tr|Q9RXH0	tr|Q9RXH0	1593		33962000			33919000	32922000
sp|Q9RYB	sp|Q9RYB	426	5.1E+08	2.72E+08	4.67E+08	1.39E+08	2.08E+08	2.79E+08
sp|Q9RXJ9	sp|Q9RXJ9	384					2954500	3957300
sp|Q9RY5	sp|Q9RY5	418						
tr|Q9RXM	tr|Q9RXM	1615	1.36E+08	1.11E+08	96700000	95789000	96955000	90815000
tr|Q9RXM	tr|Q9RXM	1615	1.87E+09	1.68E+09	1.54E+09	8.74E+08	1.14E+09	7.18E+08
sp|Q9RY6	sp|Q9RY6	421						
sp|Q9RU8	sp|Q9RU8	219					32140000	
tr|Q9RZC4	tr|Q9RZC4	1903						
sp|Q9RRJ	sp|Q9RRJ	87	1.86E+08	1.68E+08		1.75E+08		
sp|P52027	sp|P52027	51	40392000	36726000	34098000			
tr|Q9RXF2	tr|Q9RXF2	1582						
tr|Q9RVE5	tr|Q9RVE5	1219				28320000		28080000
tr|Q9RW9	tr|Q9RW9	1374	67687000	28851000	29076000		10230000	
tr|Q9RT30	tr|Q9RT30	807	24120000		24615000			
sp|Q9RTF	sp|Q9RTF	183			17212000		8859300	
tr|Q9RUP1	tr|Q9RUP1	1099	2.06E+09	1.78E+09	1.68E+09	2.25E+09	1.92E+09	2.06E+09
tr|Q9RUK9	tr|Q9RUK9	1081	21859000	11501000	12214000	35590000	40665000	27453000
sp|Q9RYB	sp|Q9RYB	426	2.27E+08	82970000	1.23E+08			
tr|Q9RRF0	tr|Q9RRF0	514	81743000	1.2E+08	1.01E+08	76242000	93034000	82742000
tr|Q9RTZ8	tr|Q9RTZ8	966	61066000	38520000	73705000	57674000	30584000	44088000
sp|Q9RU2	sp|Q9RU2	208						
tr|Q9RSK5	tr|Q9RSK5	731	22640000	30104000	9056600		19656000	23459000
tr|Q9RUY4	tr|Q9RUY4	1140	52614000	28702000		14166000	22118000	20407000
tr|Q9RT72	tr|Q9RT72	829	20672000	26725000	31062000		16903000	
tr|Q9RT72	tr|Q9RT72	829		2.36E+08	3.91E+08		34718000	
sp|Q9RXY	sp|Q9RXY	407	19508000	34967000	54060000		16186000	15481000
sp|Q9RVW	sp|Q9RVW	301	8.51E+08	6.59E+08	84423000	6.3E+08	5.04E+08	5.42E+08
sp|Q9RUP	sp|Q9RUP	243						
sp|Q9RUP	sp|Q9RUP	243						
tr|Q9RZ68	tr|Q9RZ68	1866						
sp|Q9RVW	sp|Q9RVW	301	7.07E+08	7E+08	6.66E+08	6.25E+08	5.21E+08	5.75E+08
sp|Q9RSK	sp|Q9RSK	130	2.12E+08	2.04E+08	1.97E+08	2.99E+08	2.34E+08	2.32E+08
tr|Q9RY20	tr|Q9RY20	1681			19468000	22440000		17751000
tr|Q9RST7	tr|Q9RST7	760				26683000	25648000	
tr|Q9RT81	tr|Q9RT81	835		56323000	45604000	83763000	69564000	
sp|Q9RXK	sp|Q9RXK	385	7.77E+08	1.22E+09	8.6E+08	4.35E+08	8.23E+08	5.62E+08

sp|Q9RXK	sp|Q9RXK	385	9158500	34219000	73681000			
tr|Q9RWN	tr|Q9RWN	1442						
tr|Q9RUA	tr|Q9RUA	1020		17433000				12348000
sp|Q9RRX	sp|Q9RRX	102						
sp|P5686	sp|P5686	56	4.76E+08	3.45E+08	3.14E+08	96297000	1.41E+08	1.32E+08
sp|Q9RUP	sp|Q9RUP	246	48590000	38740000	41642000	63513000	62467000	56426000
tr|Q9RWU	tr|Q9RWU	1480	42350000	73382000	1.17E+08	32816000	45397000	24974000
tr|Q9RSZ5	tr|Q9RSZ5	790				2.25E+08	1.99E+08	2.57E+08
sp|Q9RRL	sp|Q9RRL	90			4158300	18582000	30507000	20934000
tr|Q9RVV(	tr|Q9RVV(	1304					17288000	
tr|Q9RVQ	tr|Q9RVQ	1274	17081000	16162000	31801000		18655000	
sp|Q9RV2	sp|Q9RV2	264			28183000	82105000	87104000	83570000
sp|Q9RV2	sp|Q9RV2	263	5.14E+09	4.59E+09	4.04E+09	4.05E+09	3.84E+09	4.44E+09
sp|Q9RTU	sp|Q9RTU	201				1.19E+08		96041000
tr|Q9RXK9	tr|Q9RXK9	1608						21479000
sp|Q9RXG	sp|Q9RXG	370	19569000	14867000	21438000	12018000	11464000	19418000
tr|Q9RUA	tr|Q9RUA	1021	54619000	52997000	96691000	51350000	50948000	75773000
tr|Q9RRZ8	tr|Q9RRZ8	617		7233300	66664000	86782000	84671000	60766000
sp|Q9RSE	sp|Q9RSE	116				2.59E+08	2.42E+08	2.46E+08
sp|Q9RSE	sp|Q9RSE	116			42151000		16476000	
sp|Q9RTN	sp|Q9RTN	196	1.69E+09	4.24E+09	3.13E+09	3.32E+09	3.58E+09	4.09E+09
tr|Q9RVX7	tr|Q9RVX7	1318	58462000	1.17E+08	84420000	66611000	70890000	1.13E+08
sp|Q9RYH	sp|Q9RYH	435	16982000	22123000		41205000	20189000	20473000
sp|Q9RVB	sp|Q9RVB	275	92634000	1.02E+08	66984000	43866000	42839000	49350000
sp|Q9RWI	sp|Q9RWI	335	5637400	11954000	18014000		65350000	
tr|Q9RRH	tr|Q9RRH	525	51356000	39925000	36381000	1.95E+08	2.29E+08	1.78E+08
sp|Q9RY2	sp|Q9RY2	411	1.35E+10	1.74E+10	1.25E+10	7.68E+09	6.39E+09	7.52E+09
sp|Q9RY2	sp|Q9RY2	411		1.14E+08	2.12E+08			14578000
tr|Q9RW3	tr|Q9RW3	1350	1.39E+09	8.01E+08	6.42E+08	2.19E+08	2.95E+08	2.54E+08
sp|Q9RVP	sp|Q9RVP	294	2.27E+09	2.37E+09	2.48E+09	1.52E+09	1.34E+09	1.46E+09
tr|Q9RZ9(	tr|Q9RZ9(	1881	50187000	63317000	33598000	62173000	55302000	65975000
sp|Q9RU2	sp|Q9RU2	209						
tr|Q9RXQ	tr|Q9RXQ	1627	1.6E+08	1.79E+08		2.48E+08	1.76E+08	2.58E+08
tr|Q9RXL6	tr|Q9RXL6	1612	2.06E+08		1.25E+08		1.57E+08	
sp|Q9RXG	sp|Q9RXG	370	47405000	41586000	58730000	33119000	45164000	47939000
tr|Q9RV15	tr|Q9RV15	1159						
tr|Q9RZK2	tr|Q9RZK2	1940						
sp|Q9RVH	sp|Q9RVH	288	1.23E+08	1.29E+08	1.01E+08	1.68E+08	1.5E+08	1.47E+08
sp|Q9RVH	sp|Q9RVH	288		88654000	1.31E+08			
sp|P5215	sp|P5215	52						
tr|Q9RV68	tr|Q9RV68	1185	55918000	45726000	41784000			
sp|Q9RW(	sp|Q9RW(	326	1.02E+08	1E+08	1.36E+08	2.07E+08	2.21E+08	2.4E+08
tr|Q9RW8	tr|Q9RW8	1371	1.69E+08	1.75E+08	1.53E+08	3.62E+08	3.82E+08	2.99E+08
tr|Q9RVX2	tr|Q9RVX2	1316		11145000				
sp|Q9RTH	sp|Q9RTH	186	74168000	95312000	83794000	2.66E+08	1.96E+08	1.78E+08
sp|Q9RYR	sp|Q9RYR	441						
sp|Q9RUP	sp|Q9RUP	246	81479000	44584000	54015000	39632000	51475000	55585000
tr|Q9RSX3	tr|Q9RSX3	778	25868000	90262000	78047000		9580800	
sp|Q9RXT	sp|Q9RXT	400	59745000	22975000	20336000			28931000
tr|Q9RRP4	tr|Q9RRP4	558	1.92E+08	1.76E+08	1.67E+08	1.39E+08	1.46E+08	1.07E+08
tr|Q9RV68	tr|Q9RV68	1185				451200		

tr|Q9RV68	tr|Q9RV68	1185	974780	449980	507570	355760	318160	415800
tr|Q9RSL7	tr|Q9RSL7	733			26420000			
tr|Q9RSL7	tr|Q9RSL7	733						
tr|Q9RUY2	tr|Q9RUY2	1139	82831000	51664000	2301600		3135600	7149700
sp|Q9RRM	sp|Q9RRM	91	87339000	79012000	1.18E+08			
tr|Q9RRK0	tr|Q9RRK0	538	31208000	22179000	59451000			
sp|Q9RSL	sp|Q9RSL	134						28352000
sp|Q9RXI	sp|Q9RXI	374						
sp|Q9RU2	sp|Q9RU2	209			4945900	20345000	36742000	31024000
sp|Q9RSJ9	sp|Q9RSJ9	127				45013000		51407000
tr|Q9RRU1	tr|Q9RRU1	584	14186000	8698200	9633500	5861000	8870200	4127600
sp|Q9RSW	sp|Q9RSW	154	14793000	10526000	10303000	7395400	7861200	9499600
sp|Q9RSW	sp|Q9RSW	154	1.8E+08	1.31E+08	3.13E+08			26609000
sp|Q9RSW	sp|Q9RSW	154			27448000	7719800		
tr|Q9RVX1	tr|Q9RVX1	1315	49907000		1.88E+08	4.48E+08	3.91E+08	3.62E+08
tr|Q9RSF6	tr|Q9RSF6	709						
tr|Q9RUF6	tr|Q9RUF6	1048	2.92E+08	2.54E+08	1.99E+08	2.17E+08	1.43E+08	1.99E+08
tr|Q9RZL7	tr|Q9RZL7	1945		44949000	46126000	45566000	47142000	59047000
tr|Q9RWU	tr|Q9RWU	1480			15891000			
tr|Q9RVK1	tr|Q9RVK1	1244	1.51E+08		1.46E+08			
tr|Q9RV45	tr|Q9RV45	1172	1.34E+09	1.9E+09	3.49E+09	1.15E+10	1.04E+10	7.79E+09
tr|Q9RV45	tr|Q9RV45	1172	5.35E+08	4.63E+08	1.12E+09	3.6E+08	7.68E+08	1.29E+09
tr|Q9RW0	tr|Q9RW0	1336						
sp|Q9RY7	sp|Q9RY7	423	69382000	54723000	64709000	4E+08	2.52E+08	1.94E+08
tr|Q9RU93	tr|Q9RU93	1017	46962000	25971000	52291000	2.01E+08	1.17E+08	2.04E+08
tr|Q9RRC7	tr|Q9RRC7	501		2.4E+08	3.08E+08	33472000	32683000	34535000
tr|Q9RRF2	tr|Q9RRF2	515		16526000	27289000		31787000	38400000
tr|Q9RYB8	tr|Q9RYB8	1722	42242000	21314000	19652000	15818000	19533000	20505000
tr|Q9RRM	tr|Q9RRM	549	2.57E+09	2.38E+09	2.58E+09	4.11E+08	3.95E+08	5.19E+08
sp|Q9RUF	sp|Q9RUF	232		18734000		20172000	19456000	16638000
sp|Q9RUF	sp|Q9RUF	232	1.13E+08	88392000	1.49E+08	39222000	49360000	74137000
tr|Q9RT06	tr|Q9RT06	795	70308000	76729000	69115000	91309000	75448000	92454000
tr|Q9RW3	tr|Q9RW3	1348						
tr|Q9RS11	tr|Q9RS11	627	1E+08	65757000	58999000	24051000	25293000	24691000
sp|Q9RRD	sp|Q9RRD	80				51653000	46274000	86797000
tr|Q9RUU	tr|Q9RUU	1121	3168000		5041600			3568200
tr|Q9RUU	tr|Q9RUU	1121			33361000			
tr|Q9RRY6	tr|Q9RRY6	609	1248200	553840				413430
tr|Q9RZ28	tr|Q9RZ28	1858	18436000	15614000	11169000			
sp|Q9RRX	sp|Q9RRX	102	6.18E+08	1.9E+09	6.66E+08	4.39E+08	3.13E+08	4.34E+08
sp|Q9RU8	sp|Q9RU8	219	61221000	90030000	75774000	91586000	83359000	85327000
sp|Q9RXK	sp|Q9RXK	389	2.31E+09	3.06E+09	3.21E+09	3.19E+09	2.78E+09	3.32E+09
tr|Q9RU50	tr|Q9RU50	994	20439000	11074000	14384000			
tr|Q9RSF6	tr|Q9RSF6	709	33195000	38028000	27672000			
tr|Q9RVV7	tr|Q9RVV7	1309	20216000					
tr|Q9RUP1	tr|Q9RUP1	1099	91667000	78275000	84212000	1.07E+08	93946000	
tr|Q9RUP1	tr|Q9RUP1	1099			13404000	97555000	3.29E+08	3.18E+08
sp|Q9RYE	sp|Q9RYE	432	6.67E+08	6.4E+08	5.3E+08	6.91E+08	6.52E+08	6.68E+08
tr|Q9RUW	tr|Q9RUW	1127	58342000	47412000	41201000	1.36E+08	1.22E+08	1.22E+08
tr|Q9RUW	tr|Q9RUW	1127			17841000			
sp|Q9RR9	sp|Q9RR9	72			46209000			

sp|Q9RYM	sp|Q9RYM	438					4361800	
tr|Q9RS17	tr|Q9RS17	630		36301000	3.07E+08	3.23E+08	4.09E+08	3.6E+08
tr|Q9RRT9	tr|Q9RRT9	582	5.53E+08	7.19E+08	7.33E+08	1.64E+08	2.39E+08	3.24E+08
tr|Q9RWP	tr|Q9RWP	1449						
tr|Q9RU58	tr|Q9RU58	1001	4.09E+08	5.65E+08	4.29E+08	5.04E+08	6.63E+08	4.93E+08
tr|Q9RZL7	tr|Q9RZL7	1945	12455000	4611100	4579700	9642300	14196000	10268000
sp|Q9RUN	sp|Q9RUN	242	7.56E+08	8.2E+08	8.91E+08	1.04E+09	9.39E+08	1.17E+09
sp|Q9RU7	sp|Q9RU7	213	1.14E+08	1.62E+08	1.21E+08	59828000	66122000	74643000
sp|Q9RXR	sp|Q9RXR	395						
sp|Q9RRL	sp|Q9RRL	90		29824000				
tr|Q9RSV7	tr|Q9RSV7	772	1.56E+08	98702000	80030000	37676000	53478000	43561000
sp|Q9RVT	sp|Q9RVT	298	20753000	37074000	22349000	26690000		20308000
tr|Q9RXI1	tr|Q9RXI1	1601	7251000	10278000	23266000			3830100
sp|Q9RXG	sp|Q9RXG	370	95973000	1.13E+08	70905000	77752000	59502000	81075000
sp|Q9RXG	sp|Q9RXG	370				16403000	19057000	19130000
sp|Q9RXJ6	sp|Q9RXJ6	381					21789000	
tr|Q9RVF1	tr|Q9RVF1	1221						
tr|Q9RYC6	tr|Q9RYC6	1727	95019000	56270000	51527000	1.26E+08	1.3E+08	1.31E+08
tr|Q9RWC	tr|Q9RWC	1389	7.63E+08	7.23E+08	5.48E+08	5.62E+08	6.41E+08	6.3E+08
tr|Q9RUS1	tr|Q9RUS1	1111	2.42E+08	1.31E+08	1.31E+08	1.47E+08	1.78E+08	1.43E+08
tr|Q9RZ24	tr|Q9RZ24	1856	1.39E+08	1.7E+08	1.15E+08	1.5E+08	1.29E+08	1.43E+08
tr|Q9RSW	tr|Q9RSW	775	79327000	1.69E+08	1.88E+08	34982000	46144000	1.89E+08
sp|Q9RWN	sp|Q9RWN	341	66083000	1.19E+08	1.01E+08	2.02E+08	2.21E+08	1.9E+08
sp|Q9RSQ	sp|Q9RSQ	142						
sp|Q9RUV	sp|Q9RUV	252	1.43E+08	2.27E+08	1.1E+08	94754000	90575000	63064000
sp|Q9RX9	sp|Q9RX9	364						
tr|Q9RWB	tr|Q9RWB	1387	49214000	25488000	20061000	26317000	24761000	29400000
tr|Q9RXG0	tr|Q9RXG0	1587	8.82E+08	9.04E+08	7.53E+08	7.65E+08	7.07E+08	7.57E+08
tr|Q9RYS6	tr|Q9RYS6	1805	17844000	8645400	7241600			6342700
tr|Q9RTZ8	tr|Q9RTZ8	966	94369000	85078000	68186000	69129000		
sp|Q9RT2	sp|Q9RT2	164	2.62E+08	1.63E+08	1.53E+08	1.26E+08	1.67E+08	1.47E+08
tr|Q9RZ90	tr|Q9RZ90	1881						
tr|Q9RRE7	tr|Q9RRE7	513	1.57E+08	1.63E+08	1.41E+08	1.91E+08	1.71E+08	1.77E+08
tr|Q9RSK2	tr|Q9RSK2	730	8.27E+08	9.1E+08	9.25E+08	9.92E+08	1.04E+09	8.89E+08
tr|Q9RYD3	tr|Q9RYD3	1733					10966000	
sp|Q9RVV	sp|Q9RVV	300						
tr|Q9RV32	tr|Q9RV32	1168	8414600	18515000	17017000		40768000	
tr|Q9RXZ6	tr|Q9RXZ6	1671						
sp|Q9RXI7	sp|Q9RXI7	373	1.71E+08	1.54E+08	1.08E+08	4.18E+08	4.31E+08	5.04E+08
sp|Q9RXI7	sp|Q9RXI7	373	1.42E+08	1.41E+08	2.85E+08			
tr|Q9RXW	tr|Q9RXW	1657			18697000			
tr|Q9RS52	tr|Q9RS52	646	1.27E+08	1.48E+08	1.39E+08	1.17E+08	1.16E+08	1.15E+08
tr|Q9RUR	tr|Q9RUR	1109				11924000	18207000	17108000
tr|Q9RUR	tr|Q9RUR	1109			39974000	70476000	95524000	84350000
tr|Q9RUR	tr|Q9RUR	1109				35466000		
tr|Q9RUL9	tr|Q9RUL9	1087						
sp|Q9RYA	sp|Q9RYA	425	9275400				13995000	7269400
sp|Q9RW0	sp|Q9RW0	304	1.26E+08	1.73E+08	2.36E+08	81160000		1.46E+08
tr|Q9RWB	tr|Q9RWB	1386	2.55E+08	2.18E+08	3.68E+08	1.77E+09	2.18E+09	1.71E+09
tr|Q9RVL5	tr|Q9RVL5	1252				31413000	49257000	41303000
tr|Q9RYT8	tr|Q9RYT8	1816	91627000	97913000	90210000		77283000	94527000

tr|Q9RWU	tr|Q9RWU	1476			3375800	20909000	29937000	19731000
tr|Q9RTY1	tr|Q9RTY1	956			21731000			
tr|Q9RX83	tr|Q9RX83	1548						
sp|Q9RXJ1	sp|Q9RXJ1	376	3.31E+09	2.91E+09	2.17E+09	1.4E+09	1.52E+09	1.7E+09
sp|Q9RXJ1	sp|Q9RXJ1	376			9734800			
sp|Q9RXK	sp|Q9RXK	389		24983000	23761000	1.05E+08	74518000	84442000
sp|Q9RTN	sp|Q9RTN	196	2.37E+08	1.74E+08	1.21E+08	2.32E+08	1.86E+08	1.89E+08
tr|Q9RTZ9	tr|Q9RTZ9	967			11131000			
tr|Q9RW5	tr|Q9RW5	1357	22006000	15452000	11190000	41075000	38797000	29093000
sp|Q9RVA	sp|Q9RVA	274	31498000	24840000	23739000	53770000	58842000	45115000
sp|Q4657	sp|Q4657	62	57825000	52309000	51895000	81599000	62613000	90059000
sp|Q9RSL	sp|Q9RSL	133	4.56E+09	3.32E+09	4.81E+09	1.34E+09	1.63E+09	2.4E+09
tr|Q9RYY7	tr|Q9RYY7	1837				6349800		
tr|Q9RU65	tr|Q9RU65	1006	44592000	33896000	26798000			71208000
tr|Q9RXA3	tr|Q9RXA3	1557	56675000	54927000		69485000	86467000	50928000
tr|Q9RSD0	tr|Q9RSD0	692				41022000	42802000	
tr|Q9RSZ5	tr|Q9RSZ5	790				15577000	14587000	21383000
tr|Q9RSZ5	tr|Q9RSZ5	790			14799000	6594900	8291300	
sp|Q9RRT	sp|Q9RRT	98	98320000	82501000	96319000	61861000	53839000	46098000
tr|Q9RTW	tr|Q9RTW	945	51555000	60409000	70417000	39544000	45558000	25075000
sp|Q4657	sp|Q4657	61	27220000		12690000			
tr|Q9RW2	tr|Q9RW2	1343	1.79E+08	1.61E+08	2.04E+08	1.49E+08	1.24E+08	1.56E+08
tr|Q9RWF	tr|Q9RWF	1404	85337000	2.12E+08	68214000			
tr|Q9RX52	tr|Q9RX52	1528						
tr|Q9RRJ7	tr|Q9RRJ7	537	3.71E+08	3.01E+08	3.45E+08	5.44E+08	6.15E+08	5.04E+08
tr|Q9RRJ7	tr|Q9RRJ7	537		40997000	1.45E+08	50900000	1.27E+08	
sp|Q9RTB	sp|Q9RTB	177	59541000	43143000	38879000	58107000	29032000	41701000
tr|Q9RW8	tr|Q9RW8	1367	1.26E+09	1.22E+09	1.77E+09	9.5E+08	1.83E+09	1.44E+09
tr|Q9RTC9	tr|Q9RTC9	855	14685000	21921000	39899000			
tr|Q9RU37	tr|Q9RU37	984						
sp|Q9RT6	sp|Q9RT6	170	59640000	822940	1.25E+08	2.52E+08	3.73E+08	1.44E+08
sp|Q9RXK	sp|Q9RXK	386	1.14E+09	9.39E+08	8.86E+08	7.26E+08	7.25E+08	6.86E+08
sp|Q9RXK	sp|Q9RXK	386	24589000	22138000	57177000	1.07E+08	2.21E+08	62473000
tr|Q9RTY0	tr|Q9RTY0	955						
tr|Q9RZC7	tr|Q9RZC7	1906				19302000	20555000	
sp|Q9RUV	sp|Q9RUV	254		19993000		18410000	24547000	
tr|Q9RZQ	tr|Q9RZQ	1957	2.92E+08	26937000	2.98E+08	5.43E+08	4.46E+08	3.67E+08
tr|Q9RUF9	tr|Q9RUF9	1050	8.96E+08	1E+09	8.04E+08	7.66E+08	7.41E+08	7.75E+08
sp|Q9RXJ2	sp|Q9RXJ2	377	6317500	1461300	1832500			
sp|Q9RXJ2	sp|Q9RXJ2	377	14169000	51907000	50584000	75654000	1.17E+08	83758000
tr|Q9RWA	tr|Q9RWA	1382						
tr|Q9RWW	tr|Q9RWW	1490	59006000	65724000	77322000	1.17E+08	90239000	81258000
tr|Q9RXM	tr|Q9RXM	1614						
sp|Q9RU8	sp|Q9RU8	216	3.5E+08	3.57E+08	2.6E+08	4.84E+08	5.26E+08	5.02E+08
sp|Q9RU8	sp|Q9RU8	216	3.6E+09	2.91E+09	4.04E+09	1.23E+09	1.67E+09	1.94E+09
tr|Q9RU53	tr|Q9RU53	996	47169000	31090000	29829000	27374000	27090000	34429000
sp|Q9RTY	sp|Q9RTY	202						
tr|Q9RS71	tr|Q9RS71	655	9.77E+08	1.4E+09	1.06E+09	6.01E+08	9.05E+08	7.34E+08
tr|Q9RYS1	tr|Q9RYS1	1801	21699000	12475000	66018000			2594200
tr|Q9RT53	tr|Q9RT53	821	91031000	10418000			1.73E+08	1.38E+08
tr|Q9RSR7	tr|Q9RSR7	752	3.7E+08	4.84E+08	3.86E+08	4.38E+08	5.27E+08	3.69E+08

tr|Q9RSR7	tr|Q9RSR7	752	1.32E+08	1.41E+08	1.64E+08			
tr|Q9RYI7	tr|Q9RYI7	1763	1.38E+08	1.39E+08	1.24E+08	1.38E+08	1.46E+08	1.32E+08
tr|Q9RRZ5	tr|Q9RRZ5	614	1.71E+08	2.1E+08	1.6E+08	1.79E+08	1.69E+08	1.54E+08
tr|Q9RUD	tr|Q9RUD	1043						
tr|Q9RUU	tr|Q9RUU	1124	87398000	60062000	1.39E+08			
sp|Q9RYW	sp|Q9RYW	446			15913000			
tr|Q9RW2	tr|Q9RW2	1342	28361000		30894000			
tr|Q9RZQ	tr|Q9RZQ	1959						
tr|Q9RTI3	tr|Q9RTI3	879	6929500	2213600	2675200	1968300	2578700	2064300
tr|Q9RTI3	tr|Q9RTI3	879		26622000				
tr|Q9RRQ	tr|Q9RRQ	564	64422000	96817000	80496000	30864000	34721000	39541000
tr|Q9RT82	tr|Q9RT82	836			16706000			
sp|Q9RUR	sp|Q9RUR	249				2276100	4920800	3649700
tr|Q9RVT2	tr|Q9RVT2	1294		5992100	7223200	3277200	2050700	3482300
tr|Q9RVZ5	tr|Q9RVZ5	1328	3355900		8941200			8799300
tr|Q9RWH	tr|Q9RWH	1409	1.73E+08	86997000	69795000			
sp|Q9RWH	sp|Q9RWH	328	1.58E+08	2.21E+08	1.7E+08	1.81E+08	2.68E+08	2.02E+08
sp|Q9RWH	sp|Q9RWH	328	1.98E+08	3.59E+08	3.25E+08	73453000	1.44E+08	1.25E+08
sp|Q9RWH	sp|Q9RWH	328			91276000			
tr|Q9RWQ	tr|Q9RWQ	1450						
tr|Q9RSZ3	tr|Q9RSZ3	789	10710000		15113000			
sp|Q9RR6	sp|Q9RR6	65	41665000	49383000	51316000			
sp|Q9RRT	sp|Q9RRT	97	53375000	36402000	39013000	16513000	15945000	12988000
tr|Q9RUJ1	tr|Q9RUJ1	1067	3.91E+08	4.07E+08	3.2E+08	2.7E+08	2.8E+08	2.89E+08
tr|Q9RXD1	tr|Q9RXD1	1571	18446000	30444000	19252000	63330000	33925000	26613000
tr|Q9RY16	tr|Q9RY16	1680	1.16E+08	1.1E+08	1.03E+08			1.25E+08
tr|Q9RY16	tr|Q9RY16	1680	33433000	54227000	53363000		42810000	
tr|Q9RV73	tr|Q9RV73	1187		15448000	9347000		12165000	11913000
sp|Q9RSR	sp|Q9RSR	143				16892000		
tr|Q9RYY8	tr|Q9RYY8	1838	1.55E+08	1.87E+08	1.22E+08		1.67E+08	
tr|Q9RYY8	tr|Q9RYY8	1838						
tr|Q9RYQ0	tr|Q9RYQ0	1788	19220000	7103800	7863100			
tr|Q9RYQ0	tr|Q9RYQ0	1788	54591000	26096000	1.49E+08			
tr|Q9RRG5	tr|Q9RRG5	521	1.76E+08	1.67E+08	1.48E+08	1.26E+08	1.1E+08	1.07E+08
tr|Q9RXV1	tr|Q9RXV1	1650						
tr|Q9RZQ	tr|Q9RZQ	1958	1.62E+08	3.33E+08	2.16E+08	3.07E+08	4.48E+08	2.69E+08
tr|Q9RUX2	tr|Q9RUX2	1131						
tr|Q9RXN3	tr|Q9RXN3	1620	4.29E+08	4E+08	3.41E+08	5.89E+08	6.17E+08	5.74E+08
tr|Q9RT09	tr|Q9RT09	798						
tr|Q9RY39	tr|Q9RY39	1692	24311000	18796000	24621000			
sp|Q9RRT	sp|Q9RRT	98	5.48E+08	5.95E+08	4.45E+08	3.79E+08	3.81E+08	4.31E+08
sp|Q9RUN	sp|Q9RUN	242	8.36E+08	7.4E+08	5.57E+08	6.69E+08	6.61E+08	6.29E+08
sp|Q9RUN	sp|Q9RUN	242						6643000
tr|Q9RXH8	tr|Q9RXH8	1599	2.99E+08	2.6E+08	2.35E+08	1.22E+08	1.11E+08	1.2E+08
sp|Q9RWP	sp|Q9RWP	343	13398000		13147000	94515000	97151000	87812000
sp|Q9RWP	sp|Q9RWP	343				49881000	52611000	58269000
tr|Q9RWS	tr|Q9RWS	1464	23479000		40623000	32331000		
tr|Q9RUL1	tr|Q9RUL1	1083			12395000			
sp|Q9RXJ6	sp|Q9RXJ6	381		1.76E+08	2.23E+08	83800000		92356000
tr|Q9RWI1	tr|Q9RWI1	1414						
sp|Q9RSK	sp|Q9RSK	128	1.42E+09	1.01E+09	1.36E+09	3.58E+08	4.15E+08	6.26E+08

tr|Q9RUA	tr|Q9RUA	1022	43398000			36001000	40624000	
tr|Q9RUA	tr|Q9RUA	1022			17648000			
tr|Q9RRS6	tr|Q9RRS6	577		11608000		58660000	49415000	52386000
tr|Q9RZU7	tr|Q9RZU7	1979	1.11E+08	1.39E+08	1.45E+08	55324000	1.21E+08	71670000
tr|Q9RZU7	tr|Q9RZU7	1979						
tr|Q9RX95	tr|Q9RX95	1553						
sp|Q9RVD	sp|Q9RVD	280	29668000	42560000	41925000	13083000	15539000	
tr|Q9RW4	tr|Q9RW4	1351						
tr|Q9RSD6	tr|Q9RSD6	697	60004000	72071000	44616000	78580000	74172000	66524000
sp|Q9RSS	sp|Q9RSS	147	31500000	33144000	36114000	65801000	38562000	40503000
tr|Q9RUY4	tr|Q9RUY4	1140	79822000	85099000	61433000	1.52E+08	1.45E+08	1.29E+08
tr|Q9RYD7	tr|Q9RYD7	1734	70417000	1.08E+08	1.06E+08	1.19E+08	1.6E+08	1.51E+08
tr|Q9RS58	tr|Q9RS58	649	13475000		10593000			
sp|Q9RXI	sp|Q9RXI	374	6.55E+09	8.95E+09	6.72E+09	9.54E+09	9.68E+09	8.82E+09
sp|Q9RXI	sp|Q9RXI	374	6.94E+08	1.62E+09	1.66E+09		50884000	77925000
tr|Q9RS29	tr|Q9RS29	636	4813600		14881000			
sp|Q9RSR	sp|Q9RSR	144	1.38E+09	1.03E+09	9.79E+08	1.25E+09	9.9E+08	1.26E+09
sp|Q9RSR	sp|Q9RSR	144		13686000				
tr|Q9RT19	tr|Q9RT19	802		70193000	69975000	55509000	1.01E+08	53459000
tr|Q9RWL	tr|Q9RWL	1434		42923000				
tr|Q9RV70	tr|Q9RV70	1186	8464100	14009000	15093000	1.63E+08	2.12E+08	1.57E+08
tr|Q9RW7	tr|Q9RW7	1362	2.14E+08	1.7E+08	2.38E+08			2.17E+08
tr|Q9RSZ5	tr|Q9RSZ5	790	72351000	98281000	87191000	41736000	45816000	47149000
tr|Q9RYV4	tr|Q9RYV4	1822	27794000	38880000	43318000		30096000	23884000
sp|Q9RYW	sp|Q9RYW	446						
tr|Q9RRJ7	tr|Q9RRJ7	537	1.46E+08	1.69E+08	1.65E+08	1.13E+08	1.24E+08	1.13E+08
tr|Q9RV13	tr|Q9RV13	1157						
tr|Q9RVB2	tr|Q9RVB2	1206	14380000			23398000	29086000	26803000
sp|Q9RXJ5	sp|Q9RXJ5	380						
tr|Q9RZA1	tr|Q9RZA1	1888	42341000	43854000	49022000	25362000	43432000	
tr|Q9RYY9	tr|Q9RYY9	1839						
tr|Q9RZH5	tr|Q9RZH5	1930				17050000	21731000	15925000
tr|Q9RYQ2	tr|Q9RYQ2	1790	41237000	56057000	52041000	1.22E+08	1.89E+08	1.4E+08
tr|Q9RZU6	tr|Q9RZU6	1978		30989000	34783000			
tr|Q9RVT4	tr|Q9RVT4	1295	30885000	80251000	52592000		53208000	
tr|Q9RUQ	tr|Q9RUQ	1103		24637000	36961000		14944000	
sp|Q9RUA	sp|Q9RUA	224	43305000	42134000				32199000
tr|Q9RSA5	tr|Q9RSA5	676	6302600	5014700	4860900		5332700	
sp|Q9RVI3	sp|Q9RVI3	289					14683000	15360000
tr|Q9RSM	tr|Q9RSM	735	1957200	5076700	4426600	1179900	3639900	
tr|Q9RTD4	tr|Q9RTD4	858					42581000	42016000
tr|Q9RVS8	tr|Q9RVS8	1292	4.71E+08	1.06E+09	7.53E+08	8.76E+08	1.26E+09	7.2E+08
tr|Q9RVS8	tr|Q9RVS8	1292	4.5E+08	1.15E+09	2.81E+09	1.44E+08	4.5E+08	3.68E+08
tr|Q9RYT8	tr|Q9RYT8	1816	17585000	28795000		53241000	52095000	40361000
sp|Q9RSQ	sp|Q9RSQ	140						9754700
tr|Q9RXP2	tr|Q9RXP2	1623	47275000	31283000	26677000	21873000		28865000
tr|Q9RYG3	tr|Q9RYG3	1748;1749	33817000		48473000	11777000	21661000	26733000
tr|Q9RYG3	tr|Q9RYG3	1748;1749						
sp|Q9RW0	sp|Q9RW0	342	40655000	1.03E+08	2.53E+08	3.82E+08	4.4E+08	4.08E+08
sp|Q9RW0	sp|Q9RW0	342			13993000	4.69E+08	4.17E+08	5.41E+08
sp|Q9RW0	sp|Q9RW0	325			20209000			

sp|Q9RUZ	sp|Q9RUZ	260			29338000			
tr|Q9RYF4	tr|Q9RYF4	1743	1.21E+09	1.37E+09	1.19E+09	1.38E+09	1.42E+09	1.37E+09
tr|Q9RYF4	tr|Q9RYF4	1743						
tr|Q7DF92	tr|Q7DF92	465				11580000		
tr|Q9RVT9	tr|Q9RVT9	1298						
tr|Q9RSY6	tr|Q9RSY6	784		98416000	1.35E+08		98633000	
sp|Q9RVV	sp|Q9RVV	300	5.02E+08	5.76E+08	4.33E+08	6.11E+08	6.16E+08	4.52E+08
sp|Q9RTF	sp|Q9RTF	181		9322900	10308000	29744000	16321000	17278000
sp|Q9RSY	sp|Q9RSY	156	10224000	4527600	3275500	4647100		95770
tr|Q9RY79	tr|Q9RY79	1708	51351000	42707000	36535000	50301000	42774000	49241000
tr|Q9RY79	tr|Q9RY79	1708	13278000		14661000	9977100	7383400	7223900
tr|Q9RXS0	tr|Q9RXS0	1637						
tr|Q9RWQ	tr|Q9RWQ	1452		21807000				
tr|Q9RYF8	tr|Q9RYF8	1745		71359000	90041000	55663000	74257000	71156000
tr|Q9RS17	tr|Q9RS17	630	1.07E+09	1.25E+09	1.02E+09	7.46E+08	5.98E+08	7.03E+08
tr|Q9RWV	tr|Q9RWV	1484	1.61E+08	1.37E+08	1.4E+08	3.1E+08	2.71E+08	2.05E+08
tr|Q9RT30	tr|Q9RT30	807	6.06E+08	1.2E+09				
tr|Q9RUE0	tr|Q9RUE0	1044	5.59E+08	7.34E+08	1.3E+09	52775000	1.04E+08	46224000
sp|Q9RTN	sp|Q9RTN	196				16425000	28071000	
sp|Q9RY0	sp|Q9RY0	410	6.51E+08	5.58E+08	5.58E+08	5.54E+08	3.74E+08	3.95E+08
tr|Q9RVR4	tr|Q9RVR4	1283					38655000	
tr|Q9RWI0	tr|Q9RWI0	1413						
tr|Q9RSY6	tr|Q9RSY6	784	57449000	45608000	49982000	1.64E+08	1.23E+08	1.37E+08
tr|Q9RVA5	tr|Q9RVA5	1202				14509000	9884200	14722000
tr|Q9RWC	tr|Q9RWC	1392						
tr|Q9RYN0	tr|Q9RYN0	1775	7.19E+08	9.93E+08	1.41E+09	6.52E+08	3.27E+08	5.93E+08
tr|Q9RZL7	tr|Q9RZL7	1945						
sp|Q9RR7	sp|Q9RR7	67		90388000	85466000		55792000	
tr|Q9RRE3	tr|Q9RRE3	511	22254000	18272000	15739000	16527000	17258000	12845000
sp|Q9RR6	sp|Q9RR6	65	6.74E+08	9.64E+08	9.21E+08	4.12E+08	4.93E+08	6.02E+08
sp|Q9RR6	sp|Q9RR6	65	20166000	51531000	1.06E+08	55462000	1.24E+08	78774000
tr|Q9RS10	tr|Q9RS10	626	3242500	735730	683110			
tr|Q9RT02	tr|Q9RT02	793	3411800	3853700	2919000	3911500	3194600	2241900
tr|Q9RXF0	tr|Q9RXF0	1581	26316000	14292000	13594000	15224000	20359000	20408000
tr|Q9RVS8	tr|Q9RVS8	1292	1.85E+10	2.25E+10	1.68E+10	1.72E+10	1.4E+10	1.64E+10
tr|Q9RSW	tr|Q9RSW	774	2.77E+08	1.78E+08	1.65E+08	1.99E+08	1.35E+08	1.75E+08
tr|Q9RU07	tr|Q9RU07	972	3.7E+08	1.91E+08	1.87E+08			
tr|Q9RYF8	tr|Q9RYF8	1745	88050000	1.15E+08	1.11E+08	56901000	82086000	61693000
tr|Q9RST7	tr|Q9RST7	760	1.05E+09	1.14E+09	9.75E+08		8.67E+08	9.66E+08
tr|Q9RXV8	tr|Q9RXV8	1653	7.8E+08	6.57E+08	6.35E+08	7.54E+08	8.42E+08	7.88E+08
sp|Q5933	sp|Q5933	63	8.38E+08	9.52E+08	7.64E+08	9.52E+08	9.22E+08	9.44E+08
sp|Q5933	sp|Q5933	63	1.36E+08	1.91E+08	2.86E+08	41907000	1.01E+08	50847000
tr|Q9RZ81	tr|Q9RZ81	1875						
tr|Q7DF71	tr|Q7DF71	464	16649000					
tr|Q9RSX6	tr|Q9RSX6	781	52207000	66780000	59001000	74299000	72373000	78464000
sp|Q9RVA	sp|Q9RVA	274						
tr|Q9RRT9	tr|Q9RRT9	582	93637000	1.02E+08	81851000	1.44E+08	1.21E+08	1.23E+08
tr|Q9RX76	tr|Q9RX76	1544				23049000	23888000	48624000
sp|Q9RR6	sp|Q9RR6	66		2019700	1880700	24108000	6747200	6049000
sp|Q9RUP	sp|Q9RUP	244	38138000	67116000	72639000	1.05E+08		1.79E+08
tr|Q9RTE3	tr|Q9RTE3	862		29280000		51247000	40696000	29034000

tr|Q9RWC	tr|Q9RWC	1391	38426000		59185000	98063000	75004000	60529000
tr|Q9RZE4	tr|Q9RZE4	1914	1.36E+08	90757000	98725000	2.51E+08	2.36E+08	2.21E+08
sp|Q9RTS	sp|Q9RTS	199						
tr|Q9RRC5	tr|Q9RRC5	500		17287000		44076000		39164000
sp|O8302	sp|O8302	47	1.5E+08		1.36E+08	2.17E+08	1.82E+08	2.2E+08
sp|Q4657	sp|Q4657	61						
tr|Q9RYH8	tr|Q9RYH8	1757		5472900		21939000	18187000	9430400
tr|Q9RW8	tr|Q9RW8	1370	27440000	27068000	23323000	71277000	27742000	52132000
sp|P52027	sp|P52027	51						
tr|Q9RRX3	tr|Q9RRX3	602	23946000		14271000	18774000	22827000	17796000
tr|Q9RVN	tr|Q9RVN	1262	28709000	22580000	68599000	1.92E+08	1.3E+08	1.64E+08
tr|Q9RXP4	tr|Q9RXP4	1624				23999000	14735000	14801000
tr|Q9RSM	tr|Q9RSM	735	18422000	18122000	12305000	24612000	33168000	22636000
tr|Q9RWU	tr|Q9RWU	1479	46567000	57556000	59850000	1.31E+08	1.22E+08	1.48E+08
tr|Q9RSB9	tr|Q9RSB9	686	94704000	1.02E+08		93673000	1.14E+08	95439000
tr|Q9RRI7	tr|Q9RRI7	534					9517300	
tr|Q9RT51	tr|Q9RT51	820						
tr|Q9RRG6	tr|Q9RRG6	522	4.24E+08	3.32E+08	3.83E+08	1.05E+09	8.82E+08	1.07E+09
tr|Q9RUK0	tr|Q9RUK0	1075				6335900		
tr|Q9RWP	tr|Q9RWP	1449	1.5E+08	1.44E+08		2.91E+08	2.22E+08	2.62E+08
tr|Q9RS11	tr|Q9RS11	627						
tr|Q9RWA	tr|Q9RWA	1378	44753000		41078000	29217000		
tr|Q9RWN	tr|Q9RWN	1443						
tr|Q9RXD5	tr|Q9RXD5	1573	23809000	26154000	25014000	44616000	30810000	41904000
tr|Q9RW9	tr|Q9RW9	1375		36601000	32000000	42328000	33641000	40309000
tr|Q9RUP1	tr|Q9RUP1	1099	1.02E+08	82050000	93649000	1.15E+08	85748000	1.26E+08
tr|Q9RZC7	tr|Q9RZC7	1906		14103000	13291000			
tr|Q9RXS7	tr|Q9RXS7	1640	1.11E+08	1.7E+08	1.49E+08	2.95E+08	3.31E+08	2.54E+08
tr|Q9RXS7	tr|Q9RXS7	1640		63594000			31895000	37868000
tr|Q9RSK2	tr|Q9RSK2	730	39965000	87327000	93180000		27938000	
tr|Q9RVZ5	tr|Q9RVZ5	1328					6830500	
tr|Q9RYC6	tr|Q9RYC6	1727						
tr|Q9RYW	tr|Q9RYW	1826	20219000	23528000	29598000			
tr|Q9RRE0	tr|Q9RRE0	508		24228000	25066000	30849000		30712000
tr|Q9RYN6	tr|Q9RYN6	1780	3.33E+08	2.68E+08	3.8E+08	5.21E+08	4.91E+08	3.22E+08
sp|O8303	sp|O8303	48	52183000	32788000	37871000			23786000
tr|Q9RRP4	tr|Q9RRP4	558	55063000	30761000				
tr|Q9RTX3	tr|Q9RTX3	948		3890200	3793700	5887300	6590900	8332100
tr|Q9RWP	tr|Q9RWP	1447	4.01E+08	3.98E+08	2E+08	4.08E+08	2.17E+08	2.86E+08
tr|Q9RRS4	tr|Q9RRS4	575	13522000	14816000	14417000	12719000	14796000	13074000
tr|Q9RV63	tr|Q9RV63	1183	40595000	44052000	33167000	39427000	36817000	32151000
sp|Q9RVH	sp|Q9RVH	288	7259500		65985000	44973000		
tr|Q9RRI7	tr|Q9RRI7	534	4.42E+08	4.02E+08	3.47E+08	2.49E+08	2.26E+08	2.29E+08
tr|Q9RZS5	tr|Q9RZS5	1965	71648000	66665000	65520000	1.18E+08	91436000	99414000
tr|Q9RRY3	tr|Q9RRY3	606	61080000	41756000	37650000			
tr|Q9RR71	tr|Q9RR71	470	4.82E+08	4.19E+08	4.12E+08	5.01E+08	4.94E+08	4.41E+08
sp|Q9RY2	sp|Q9RY2	411	39801000	41008000	43975000	78891000	82156000	78045000
sp|Q9RY2	sp|Q9RY2	411			1.31E+08	2.88E+08	2.81E+08	1.96E+08
sp|Q9RY2	sp|Q9RY2	411				18282000		
tr|Q9RXD7	tr|Q9RXD7	1574	85156000	1.05E+08	1.01E+08	20579000	30294000	26760000
tr|Q9RWT	tr|Q9RWT	1472				16463000		19413000

sp|Q9R34	sp|Q9R34	64	48366000	35227000	53412000	28732000	37352000	53816000
sp|Q9R34	sp|Q9R34	64				3.37E+08	3.12E+08	3.23E+08
sp|Q9RWl	sp|Q9RWl	347	50966000	50714000	69302000	5.34E+08	1.42E+08	1.01E+08
tr|Q9RTN(	tr|Q9RTN(	903						
tr|Q9RUF8	tr|Q9RUF8	1049			3187600	37159000	60032000	50795000
tr|Q9RW5	tr|Q9RW5	1357				49605000		
tr|Q9RXL9	tr|Q9RXL9	1613	6585300	1624800	1357400		1012000	662320
tr|Q9RZ09	tr|Q9RZ09	1847	2.84E+08	3.9E+08	3.5E+08	1.05E+08	1.64E+08	1.58E+08
sp|Q9RRU	sp|Q9RRU	100	1.19E+08	95250000	82219000	61538000	44280000	75953000
tr|Q9RRK(	tr|Q9RRK(	538	1.04E+08	75623000	93708000		71053000	
sp|Q9RU2	sp|Q9RU2	209	2.02E+08	2.48E+08	5.96E+08	8.74E+08	7.43E+08	7.23E+08
tr|Q9RXZ3	tr|Q9RXZ3	1669		33256000	41003000	20699000	19120000	29245000
tr|Q9RVX7	tr|Q9RVX7	1318	3240700	2523600	3268000		2552300	2719100
sp|Q9RUF	sp|Q9RUF	231			14126000		14694000	
tr|Q9RXC2	tr|Q9RXC2	1568	52111000	65763000	39940000		74702000	75865000
sp|Q9RVI3	sp|Q9RVI3	289	2.48E+08	2.49E+08	2.02E+08	2.43E+08	2.13E+08	2.48E+08
tr|Q9RUB	tr|Q9RUB	1027						
tr|Q9RTV2	tr|Q9RTV2	937	2.34E+09	3.61E+09	4.24E+09	1.08E+09	1.05E+09	1.04E+09
tr|Q9RS80	tr|Q9RS80	660	83893000	1.21E+08	2.13E+08	1.15E+08	1.42E+08	1.43E+08
tr|Q9RXB8	tr|Q9RXB8	1566	6545100	3057600	1924300	3201400		3465800
tr|Q9RTR3	tr|Q9RTR3	922	77505000	76760000	63611000	31064000	31424000	34147000
tr|Q9RU7(	tr|Q9RU7(	1009				92165000	82984000	96113000
tr|Q9RYV(	tr|Q9RYV(	1820	5.94E+08	7.46E+08	5.15E+08	8.94E+08	1.19E+09	8.56E+08
tr|Q9RWB	tr|Q9RWB	1387			5.47E+08		3.9E+08	
tr|Q9RYC2	tr|Q9RYC2	1724	42244000	4.85E+08	5.36E+08		1.26E+08	1.5E+08
tr|Q9RVC6	tr|Q9RVC6	1212						
tr|Q9RWQ	tr|Q9RWQ	1450						
tr|Q9RTR8	tr|Q9RTR8	925						
tr|Q9RSH1	tr|Q9RSH1	717	35109000	42332000	84149000			
tr|Q9RUH	tr|Q9RUH	1060	95950000	82793000	87207000		44160000	
tr|Q9RSB3	tr|Q9RSB3	682	28141000			46768000	31034000	49559000
tr|Q9RX95	tr|Q9RX95	1553	3396500		6262100	16714000	7338900	8042300
tr|Q9RYL8	tr|Q9RYL8	1771	18215000		30724000			
sp|Q9RTI(	sp|Q9RTI(	187	1.21E+08	1.24E+08	1.32E+08	99029000	1.03E+08	1.41E+08
sp|Q9RTH	sp|Q9RTH	186						
tr|Q9RWI1	tr|Q9RWI1	1414				26055000		
tr|Q9RYI1	tr|Q9RYI1	1760	65309000	53616000	51798000	1.16E+08	90541000	1.06E+08
tr|Q9RUX2	tr|Q9RUX2	1131						
tr|Q9RWH	tr|Q9RWH	1409	9.5E+08	7.95E+08	6.75E+08	1.86E+09	1.75E+09	1.83E+09
tr|Q9RWH	tr|Q9RWH	1409	7721300	5787000	6109000	4037000	6005900	
tr|Q9RVK8	tr|Q9RVK8	1248	4.6E+08	5.47E+08	6.7E+08	2.38E+08	2.81E+08	4.6E+08
tr|Q9RRQ	tr|Q9RRQ	565						
tr|Q9RZH(	tr|Q9RZH(	1927	40814000	20977000	23194000			
tr|Q9RSD(	tr|Q9RSD(	692	15440000	32749000	24974000	19141000	25325000	18150000
tr|Q9RW9	tr|Q9RW9	1377	1761900			1951300		1415100
sp|Q9RVV	sp|Q9RVV	300	1484000		688150		1042500	614910
tr|Q9RTH3	tr|Q9RTH3	876	75647000	1.13E+08	96547000	1.17E+08	1.27E+08	1.61E+08
tr|Q9RTD3	tr|Q9RTD3	857	3.25E+08	2.29E+08	1.55E+08	1.86E+08	2.21E+08	1.19E+08
tr|Q9RTX7	tr|Q9RTX7	952	11086000	6695100	5587300			
tr|Q9RVK5	tr|Q9RVK5	1245			7450800	19116000	11286000	
tr|Q9RSI8	tr|Q9RSI8	725	48890000		27672000			

tr|Q9RZH(	tr|Q9RZH(	1927			28514000			
tr|Q9RV7(	tr|Q9RV7(	1186				6084400	9328300	10098000
tr|Q9RWL	tr|Q9RWL	1432	1.83E+08	1.73E+08	1.57E+08			
tr|Q9RWR	tr|Q9RWR	1460	71298000	75356000	69657000	72186000	75719000	72075000
tr|Q9RXR1	tr|Q9RXR1	1632						
sp|Q9RXG	sp|Q9RXG	370			16106000	22034000	15526000	22804000
sp|Q9RUH	sp|Q9RUH	236	50699000	50383000	50542000	57769000	27477000	40877000
tr|Q9RZS6	tr|Q9RZS6	1966	91746000	90187000	72497000	97677000	87451000	96893000
tr|Q9RV18	tr|Q9RV18	1161	89969000	85001000	64862000	1.69E+08	1.41E+08	1.17E+08
tr|Q9RSZ9	tr|Q9RSZ9	791	46511000	46404000	1.81E+08	19945000		1.47E+08
tr|Q9RY46	tr|Q9RY46	1694			505050	743340	2893800	1157900
tr|Q9RZQ	tr|Q9RZQ	1959				26405000	22376000	36242000
tr|Q9RS15	tr|Q9RS15	629	35082000	29219000	37423000	26943000	32231000	36285000
tr|Q9RSY6	tr|Q9RSY6	784				46127000	54156000	51705000
tr|Q9RV55	tr|Q9RV55	1179	3.07E+08	3.37E+08	3.53E+08	1.65E+08	1.42E+08	1.7E+08
tr|Q9RY83	tr|Q9RY83	1709	2591900	1000300	832520	1468400	1095300	1202400
sp|Q9RRB	sp|Q9RRB	76		20504000	32867000		40009000	72088000
sp|Q9RY2	sp|Q9RY2	411						
tr|Q9RU98	tr|Q9RU98	1018	12016000	11044000	34953000	63887000	43294000	91147000
tr|Q9RRA(	tr|Q9RRA(	483	14486000	14158000	76559000	23363000	81216000	11003000
sp|Q9RSH	sp|Q9RSH	119	66188000	70042000	65302000	98034000	78102000	89577000
tr|Q9RWF	tr|Q9RWF	1400	1.57E+08	1.68E+08	1.94E+08	1.99E+08	2.26E+08	2.05E+08
tr|Q9RXD5	tr|Q9RXD5	1573	48217000		60552000			
tr|Q9RX66	tr|Q9RX66	1539	6436400					
tr|Q9RUD	tr|Q9RUD	1043	45005000	68352000	1.33E+08		40230000	
tr|Q9RSK2	tr|Q9RSK2	730	1.8E+08	1.57E+08	2.88E+08			
sp|Q9RT2	sp|Q9RT2	162	41620000	23143000	59469000	3.81E+08	4.59E+08	2.7E+08
sp|Q9RT2	sp|Q9RT2	162	4.03E+08	4.69E+08	1.04E+09	6.85E+08	5.7E+08	48155000
sp|Q9RRB	sp|Q9RRB	76	40831000	46032000	42771000	77060000	21753000	76841000
tr|Q9RZQ	tr|Q9RZQ	1958	2.26E+08	1.93E+08	2.24E+08	3.64E+08	3.17E+08	3.61E+08
tr|Q9RZQ	tr|Q9RZQ	1958	78834000	58726000	99535000			43196000
tr|Q9RV29	tr|Q9RV29	1165	52308000	40870000	51997000		12342000	
tr|Q9RV03	tr|Q9RV03	1152	11576000		11544000			
sp|Q9RRB	sp|Q9RRB	76						27604000
sp|Q9RRB	sp|Q9RRB	76						
tr|Q9RTW	tr|Q9RTW	942		8904100	10272000			
sp|Q9RRH	sp|Q9RRH	85		15044000	12138000			
tr|Q9RYN8	tr|Q9RYN8	1781	18788000	9066500	6484900	10575000	13403000	11931000
tr|Q9RU55	tr|Q9RU55	998	1.68E+08	81668000	75242000	88974000	1.04E+08	1.12E+08
tr|Q9RU55	tr|Q9RU55	998	5.92E+08	7.24E+08	9.41E+08	2.81E+08	9.09E+08	6.21E+08
tr|Q9RSM	tr|Q9RSM	737						
tr|Q9RUM	tr|Q9RUM	1088	3.74E+08	4.11E+08	3.1E+08	2.67E+08	1.59E+08	2.26E+08
sp|Q9RY6	sp|Q9RY6	420					18370000	
sp|O3250	sp|O3250	44	90743000	1.79E+08	1.49E+08	1.82E+08	2.59E+08	1.72E+08
sp|O3250	sp|O3250	46				37162000	31051000	
tr|Q9RXF0	tr|Q9RXF0	1581	2786600	519470	860040		706270	654280
tr|Q9RWN	tr|Q9RWN	1443	21852000	15141000				10288000
tr|Q9RTC9	tr|Q9RTC9	855	1.88E+08	2.13E+08	1.74E+08	2.16E+08	2.42E+08	2.72E+08
tr|Q9RXX2	tr|Q9RXX2	1660			2.1E+08			81140000
tr|Q9RYY7	tr|Q9RYY7	1837						
tr|Q9RZJ2	tr|Q9RZJ2	1939	1.05E+09	6.27E+08	6.29E+08	5.01E+08	4.22E+08	5.46E+08

sp|Q9RS2	sp|Q9RS2	108	5789200	2673000	2650900	2847500	3584800	2993700
tr|Q9RXB6	tr|Q9RXB6	1565	3.95E+08	5.33E+08	4.86E+08	3.45E+08	4.65E+08	3.87E+08
tr|Q9RUA	tr|Q9RUA	1021		13390000				
tr|Q9RXB0	tr|Q9RXB0	1561						
tr|Q9RZQ	tr|Q9RZQ	1959	5.54E+08	8.07E+08	6.98E+08	5.91E+08	7.86E+08	7.35E+08
tr|Q9RT16	tr|Q9RT16	801						8461800
sp|Q9RU4	sp|Q9RU4	211	27397000	22644000	26654000			
sp|Q9RY5	sp|Q9RY5	418	18575000	56221000	1.05E+08		51962000	16666000
sp|Q9RTA	sp|Q9RTA	174			10940000			
tr|Q9RYV4	tr|Q9RYV4	1822	93683000	81367000	1.66E+08			
sp|Q9RXC	sp|Q9RXC	367	2.04E+08	1.7E+08	1.57E+08	5.03E+08	3.36E+08	3.59E+08
tr|Q9RZQ	tr|Q9RZQ	1958	49021000	56542000	63829000	85835000	98230000	69555000
sp|Q9RZU	sp|Q9RZU	459	4.18E+08	3.79E+08	3.2E+08	3.84E+08	3.87E+08	3.72E+08
tr|Q9RRB3	tr|Q9RRB3	494						
tr|Q9RW8	tr|Q9RW8	1368	1.07E+09	1.45E+09	1.11E+09	1.1E+09	8.68E+08	1E+09
tr|Q9RTV5	tr|Q9RTV5	938	3.6E+08	3.99E+08	4.28E+08	5.7E+08	7.38E+08	5.11E+08
tr|Q9RTV5	tr|Q9RTV5	938			87205000	35832000	35720000	34898000
tr|Q9RYN9	tr|Q9RYN9	1782			24795000	37398000	30424000	27997000
tr|Q9RR95	tr|Q9RR95	479				41259000	34712000	28179000
sp|Q9RSJ7	sp|Q9RSJ7	125	1.22E+08	1.18E+08	1.31E+08			
sp|Q9RSJ7	sp|Q9RSJ7	125			37724000			
sp|Q9RXC	sp|Q9RXC	367	3.62E+08	2.97E+08	2.96E+08	3.89E+08	2.81E+08	3.48E+08
tr|Q9RRX1	tr|Q9RRX1	601	3.88E+08	4.56E+08	7.25E+08	2.68E+08	3.13E+08	3.65E+08
tr|Q9RRX1	tr|Q9RRX1	601			28919000	2.65E+08	2.11E+08	2.4E+08
tr|Q9RYW	tr|Q9RYW	1828						
sp|Q9RTJ3	sp|Q9RTJ3	189	38508000	39929000	43420000			31631000
tr|Q9RT07	tr|Q9RT07	796					26008000	
tr|Q9RUZ9	tr|Q9RUZ9	1150	38640000			89428000	22370000	72905000
tr|Q9RUZ9	tr|Q9RUZ9	1150		4370500	4720600	2255700	2608200	3716900
tr|Q9RTX0	tr|Q9RTX0	947	1.84E+08	1.67E+08	1.86E+08	3.44E+08	2.91E+08	3.24E+08
tr|Q9RTX0	tr|Q9RTX0	947	21228000	39664000	59967000	15294000	30274000	36117000
tr|Q9RRJ7	tr|Q9RRJ7	537		17999000	52304000	37910000	21747000	
sp|P56867	sp|P56867	56	1.24E+09	1.39E+09	1.13E+09	1.28E+09	8.89E+08	1.13E+09
sp|Q9RT2	sp|Q9RT2	162	72628000	50877000	48197000	83939000	1.05E+08	80315000
tr|Q9RSF8	tr|Q9RSF8	710	16005000	33789000	66316000		43798000	19631000
sp|Q9RTQ	sp|Q9RTQ	197			18859000			
tr|Q9RYP6	tr|Q9RYP6	1785						
tr|Q9RZM	tr|Q9RZM	1947	24827000	26804000	28040000			
tr|Q9RUI0	tr|Q9RUI0	1061	1.36E+08	1.23E+08	1.45E+08	2.56E+08	2.72E+08	2.21E+08
tr|Q9RUI0	tr|Q9RUI0	1061	31520000	3.09E+08	4.76E+08	1.54E+08	21859000	2.28E+08
tr|Q9RTJ1	tr|Q9RTJ1	883						
tr|Q9RTW	tr|Q9RTW	945	5785600	2689800	2860200	3716100	6162500	5953000
tr|Q9RZT5	tr|Q9RZT5	1972						
tr|Q9RVD2	tr|Q9RVD2	1214	11807000		17079000			
tr|Q9RU57	tr|Q9RU57	1000	56830000	66266000	68118000	18307000	17876000	30036000
tr|Q9RYN6	tr|Q9RYN6	1780			2485500		2594700	
sp|Q9RUD	sp|Q9RUD	227	5.19E+08	3.89E+08	3.92E+08	1.73E+08	2.16E+08	1.71E+08
tr|Q9RW8	tr|Q9RW8	1369	40983000	22902000	35271000	9019000		
tr|Q9RZU4	tr|Q9RZU4	1977						
tr|Q9RT70	tr|Q9RT70	827	44574000	52342000	77832000	2.7E+08	2.88E+08	1.74E+08
tr|Q9RRJ7	tr|Q9RRJ7	537	30199000	17053000	27734000		16055000	31454000

tr|Q9RXB8	tr|Q9RXB8	1566	29741000	22989000	36181000		30601000	41390000
tr|Q9RS0C	tr|Q9RS0C	618	20656000	31542000	47965000	80912000	54883000	55665000
sp|Q9RRM	sp|Q9RRM	91						
tr|Q9RVQ	tr|Q9RVQ	1276	27209000	16666000	27588000	6132900	9077400	7592100
sp|Q9RZU	sp|Q9RZU	458						
tr|Q9RYL8	tr|Q9RYL8	1771			21874000			
tr|Q9RZ81	tr|Q9RZ81	1875	28087000	51447000	29194000	45388000	40120000	44683000
tr|Q9RRA(	tr|Q9RRA(	483	3.51E+08	3.23E+08	2.63E+08	4.42E+08	4.21E+08	3.87E+08
tr|Q9RXM	tr|Q9RXM	1617						
tr|Q9RTG(	tr|Q9RTG(	870	1.92E+08	1.49E+08	2.08E+08	1.65E+08	1.61E+08	2.12E+08
sp|Q9RT3	sp|Q9RT3	166	16423000	19429000	34298000	6966800	8061500	10121000
sp|Q9RTB	sp|Q9RTB	176	1.25E+08	1.05E+08	1.02E+08	2.42E+08	2.08E+08	2.17E+08
tr|Q9RT43	tr|Q9RT43	815	37799000	48384000	44086000		57569000	54753000
tr|Q9RVI8	tr|Q9RVI8	1237						
tr|Q9RZG1	tr|Q9RZG1	1924	26044000	44758000	14577000	47054000	31277000	49614000
sp|Q9RTQ	sp|Q9RTQ	197	1.13E+09	9.68E+08	9.5E+08	7.15E+08	6.39E+08	7.21E+08
tr|Q9RWT	tr|Q9RWT	1472						
tr|Q9RT75	tr|Q9RT75	831	7.21E+08	4.74E+08	7.52E+08	1.61E+08	1.74E+08	3.49E+08
tr|Q9RV70	tr|Q9RV70	1186						
tr|Q9RRB1	tr|Q9RRB1	492	28619000	10668000				
tr|Q9RRQ	tr|Q9RRQ	565	37031000	37525000	32397000			40027000
tr|Q9RV17	tr|Q9RV17	1160	2.46E+08	2.26E+08	1.5E+08	2.11E+08	1.78E+08	2.11E+08
tr|Q9RYS7	tr|Q9RYS7	1806			10369000			
tr|Q9RTE3	tr|Q9RTE3	862						
tr|Q9RTE3	tr|Q9RTE3	862						
tr|Q9RW0	tr|Q9RW0	1333	71479000	82545000	77516000	77295000	55145000	79144000
tr|Q9RW0	tr|Q9RW0	1333	3.27E+08	4.5E+08	4.3E+08	4.48E+08	3.57E+08	3.87E+08
sp|Q9RU4	sp|Q9RU4	211	1.48E+08	1.53E+08	1.33E+08	1.81E+08	1.39E+08	1.51E+08
tr|Q9RTY4	tr|Q9RTY4	958	24737000	32815000	33526000	46035000		22666000
tr|Q9RUC	tr|Q9RUC	1036						
sp|Q9RRP	sp|Q9RRP	93	5.02E+08	3.73E+08	3.16E+08	4.51E+08	3.67E+08	3.7E+08
tr|Q9RVN	tr|Q9RVN	1261	61217000	53794000	1.1E+08			
tr|Q9RR95	tr|Q9RR95	479	67241000	50327000	64432000	16590000	30503000	15941000
sp|Q9RXT	sp|Q9RXT	401		26894000	43980000	42940000	1.26E+08	44924000
sp|Q9RU8	sp|Q9RU8	219						
sp|Q9RRL	sp|Q9RRL	90	1.82E+08	1.93E+08	1.51E+08	3.13E+08	2.42E+08	2.75E+08
tr|Q9RSU7	tr|Q9RSU7	767						81504000
sp|Q9RW/	sp|Q9RW/	319	5.26E+08	3.95E+08	4.35E+08	65145000	92612000	93541000
sp|Q9RU7	sp|Q9RU7	213			20411000	34788000		32949000
tr|Q9RWV	tr|Q9RWV	1483	1.13E+08	1.08E+08	89456000	2.56E+08	1.68E+08	1.91E+08
sp|Q9RUV	sp|Q9RUV	258						
sp|Q9RSJ6	sp|Q9RSJ6	124	9.04E+08	8.09E+08	1.01E+09	2.13E+09	1.08E+09	1.57E+09
tr|Q9RUQ	tr|Q9RUQ	1104			22677000			
tr|Q9RRW	tr|Q9RRW	594	43730000	31975000	24860000	20994000	10014000	23971000
tr|Q9RYV7	tr|Q9RYV7	1824	81047000	64430000	67521000	26303000	23804000	25317000
tr|Q9RU41	tr|Q9RU41	988					45297000	46182000
sp|Q9RX3	sp|Q9RX3	356	1.11E+08	67452000	79714000	2.56E+08	2.53E+08	1.96E+08
tr|Q9RWM	tr|Q9RWM	1438	3.71E+08	3.94E+08	2.81E+08	4.83E+08	5.5E+08	5.85E+08
sp|Q9RV8	sp|Q9RV8	270						
tr|Q9RYF5	tr|Q9RYF5	1744	41060000		23177000	51971000	31830000	44934000
tr|Q9RWP	tr|Q9RWP	1449						

tr|Q9RYN	tr|Q9RYN3	1777	98129000	2.23E+08	1.66E+08	27411000	44729000	35804000
sp|Q9RVT	sp|Q9RVT	299						
tr|Q9RRN	tr|Q9RRN	551	14118000			18511000	21277000	23600000
tr|Q9RXA	tr|Q9RXA4	1558			24491000	35959000	49778000	51248000
tr|Q9RUC	tr|Q9RUC	1035						
tr|Q9RX57	tr|Q9RX57	1532	14394000	16321000	14673000	71510000	92485000	68313000
tr|Q9RX57	tr|Q9RX57	1532	66764000	22656000	63517000			
tr|Q9RTQ	tr|Q9RTQ6	916	1.17E+08	1.07E+08	70173000			18825000
sp|Q9RW	sp|Q9RW	335	1.44E+08	85211000	1.2E+08		1.55E+08	1.67E+08
sp|Q9RW	sp|Q9RW	335					31568000	
tr|Q9RV4	tr|Q9RV46	1173	1.09E+08	1.57E+08	1.33E+08	1.64E+08	1.35E+08	1.49E+08
tr|Q9RSM	tr|Q9RSM	736		69309000	71679000			
sp|Q9RRU	sp|Q9RRU	99	30542000	35212000	44056000			
tr|Q9RRQ	tr|Q9RRQ	566	18746000	57262000	67301000	25156000	30149000	28444000
tr|Q9RSQ	tr|Q9RSQ6	749	1.04E+08	1.11E+08	1.2E+08	74334000	66106000	1.11E+08
tr|Q9RY6	tr|Q9RY69	1702		50107000	55404000		87727000	
sp|Q9RSS	sp|Q9RSS	147	6.49E+08	7.85E+08	7.05E+08	7.81E+08	6.45E+08	8.35E+08
tr|Q9RWL	tr|Q9RWL	1434		51549000	45303000	65484000	87998000	52573000
sp|Q9RV9	sp|Q9RV9	273	9.45E+08	1.67E+08	1.99E+08	9.96E+08	8.47E+08	1.17E+09
sp|Q9RRP	sp|Q9RRP	93	5539000	8641400	12586000	25987000	44683000	12023000
sp|Q9RR9	sp|Q9RR9	71	45541000				96073000	
sp|Q9RRB	sp|Q9RRB	76						
sp|Q9RRB	sp|Q9RRB	76			13093000	56878000	52781000	56122000
tr|Q9RRV	tr|Q9RRV4	591			1265200		1750900	1869300
tr|Q9RWN	tr|Q9RWN	1442	1.52E+08	1.6E+08	1.82E+08	2.93E+08	2.49E+08	2.44E+08
sp|Q9RXS	sp|Q9RXS	397	68529000	69209000	50003000	55867000	61537000	69653000
sp|Q9RXS	sp|Q9RXS	397	1.15E+08	99064000	92483000	58260000	51335000	66690000
tr|Q9RSD	tr|Q9RSD0	692	1.28E+09	1.28E+09	8.8E+08	1.78E+09	1.32E+09	1.3E+09
tr|Q9RVX	tr|Q9RVX5	1317				44865000		34169000
tr|Q9RVZ	tr|Q9RVZ5	1328						34194000
tr|Q9RS01	tr|Q9RS01	619						
sp|Q9RXK	sp|Q9RXK	387		1.44E+08	1.47E+08	1.69E+08	2.32E+08	2.5E+08
tr|Q9RVQ	tr|Q9RVQ	1276			70486000			
tr|Q9RYI7	tr|Q9RYI7	1763	1.34E+08	1.68E+08	1.23E+08	1.28E+08	1.15E+08	1.18E+08
tr|Q9RXB	tr|Q9RXB8	1566		81014000		1.28E+08	1.55E+08	1.06E+08
sp|Q9RYN	sp|Q9RYM	439	4.49E+09	3.76E+09	4.78E+09	3.27E+09	3.49E+09	5.22E+09
tr|Q9RYE1	tr|Q9RYE1	1737						
tr|Q9RVN	tr|Q9RVN	1265	66010000	39335000	62334000	48223000	56033000	49096000
tr|Q9RSN	tr|Q9RSN3	740	45802000	48758000	40755000	91510000	1.09E+08	
sp|Q9RYE	sp|Q9RYE	433		21378000				
tr|Q9RZD	tr|Q9RZD6	1910	1.03E+08	1.15E+08	1.15E+08	1.49E+08	1.18E+08	1.68E+08
tr|Q9RZK	tr|Q9RZK2	1940						
tr|Q9RRE	tr|Q9RRE6	512						
sp|Q9RRS	sp|Q9RRS	96				9999000	16687000	
sp|Q9RRS	sp|Q9RRS	96	7510800	10682000	13775000			6085700
sp|Q9RU1	sp|Q9RU1	207						
tr|Q9RSE7	tr|Q9RSE7	705	1.46E+08	1.5E+08	1.21E+08	1.75E+08	1.43E+08	1.68E+08
sp|Q9RZA	sp|Q9RZA	454	89093000	73666000	65266000	45158000	56106000	45623000
tr|Q9RZI5	tr|Q9RZI5	1936	13023000		25517000			
sp|Q9RXT	sp|Q9RXT	400	23446000		8172000			
tr|Q9RVT2	tr|Q9RVT2	1294						

sp|P5215	sp|P5215	52	3.1E+08	2.65E+08	1.84E+08	9.2E+08	1.04E+09	4.61E+08
tr|Q9RR8	tr|Q9RR86	477				30763000		
tr|Q9RVH	tr|Q9RVH	1232	7663700	4343200	4025900		2513300	2768600
tr|Q9RU2	tr|Q9RU2	980						
tr|Q9RU2	tr|Q9RU2	980				34003000	20372000	22962000
tr|Q9RXR	tr|Q9RXR	1636			27510000			
tr|Q9RYS	tr|Q9RYS	1807	21188000	18393000	30486000			
tr|Q9RUE	tr|Q9RUE	1044						
tr|Q9RWV	tr|Q9RWV	1486						
tr|Q9RVU	tr|Q9RVU	1303	26763000	47960000	46159000	26395000	38483000	
tr|Q9RVU	tr|Q9RVU	1303		70209000	69374000			40649000
tr|Q9RRG	tr|Q9RRG	520	4.2E+08	3.59E+08	2.38E+08	2.74E+08	2.39E+08	1.81E+08
tr|Q9RYN	tr|Q9RYN	1780	25713000	20049000	28483000	21689000	32977000	25258000
tr|Q9RR6	tr|Q9RR66	468	22845000	13875000	11426000	4048000	11578000	12280000
tr|Q9RWB	tr|Q9RWB	1386	1.43E+08	1.57E+08	2.07E+08	1.51E+08	2.6E+08	1.64E+08
tr|Q9RS5	tr|Q9RS56	648	9.84E+08	3.55E+08	4.11E+08		89070000	3.7E+08
sp|P5686	sp|P5686	56				27279000		20790000
tr|Q9RWQ	tr|Q9RWQ	1455	53119000	55652000	1.22E+08	4.62E+08	5.06E+08	3.22E+08
tr|Q9RUL	tr|Q9RUL	1087			7201000			
tr|Q9RVP	tr|Q9RVP	1266	36180000	45477000	35397000	72294000	74359000	71079000
tr|Q9RX8	tr|Q9RX80	1546	3.06E+08	1.75E+08	2.53E+08	32826000	2.46E+08	2.75E+08
tr|Q9RX8	tr|Q9RX80	1546	76923000	62215000	67582000	33521000	34409000	63474000
tr|Q9RZK	tr|Q9RZK	1940	2E+08	2.05E+08	2.14E+08	1.65E+08	2.31E+08	2.13E+08
tr|Q9RZK	tr|Q9RZK	1940						
tr|Q9RUC	tr|Q9RUC	1033			36714000			
tr|Q9RXF	tr|Q9RXF5	1584				53674000		
tr|Q9RZL4	tr|Q9RZL4	1943						
tr|Q9RXI6	tr|Q9RXI6	1605						
tr|Q9RVG	tr|Q9RVG	1230	992050		392230			
sp|Q9RU8	sp|Q9RU8	217						
sp|Q9RW	sp|Q9RW	335	91594000	96831000	99078000	2.86E+08	2.38E+08	2.37E+08
tr|Q9RT2	tr|Q9RT28	805	5.13E+08	3.6E+08	2.16E+08	4.06E+08	3.75E+08	4.26E+08
sp|Q9RXK	sp|Q9RXK	389			7549600	18343000	24966000	12936000
tr|Q9RUB	tr|Q9RUB	1031			23086000			
sp|Q9RSK	sp|Q9RSK	129	33407000	31497000	20417000		24373000	
tr|Q9RVB	tr|Q9RVB	1206	2.71E+08	3.96E+08	2.14E+08	1.81E+08	1.56E+08	2.62E+08
tr|Q9RTW	tr|Q9RTW	946				20782000	19060000	14553000
sp|Q9RVQ	sp|Q9RVQ	295					14084000	11933000
tr|Q9RZE4	tr|Q9RZE4	1914	96045000	49344000	28528000	5.77E+08	5.46E+08	5.94E+08
tr|Q9RW4	tr|Q9RW4	1355	44347000	43096000	36147000	35372000	29439000	
sp|Q9RSK	sp|Q9RSK	131		52695000	30462000	3.89E+08	92432000	2.62E+08
sp|Q9RSK	sp|Q9RSK	131	6.5E+09	6.02E+09	5.63E+09	9.12E+09	9.24E+09	8.11E+09
tr|Q9RSC1	tr|Q9RSC1	687	1.1E+08	88214000	93452000	1.24E+08	1.17E+08	1.01E+08
tr|Q9RS6	tr|Q9RS60	651						
tr|Q9RVJ0	tr|Q9RVJ0	1238	7.8E+08	8.02E+08		6.49E+08	5.63E+08	5.56E+08
tr|Q9RVJ0	tr|Q9RVJ0	1238		36041000	1.06E+08	14249000	75138000	66154000
tr|Q9RZE	tr|Q9RZE	1913	47239000	30737000	30435000		15089000	
sp|P5686	sp|P5686	55						
sp|P5686	sp|P5686	55						
sp|Q9RTA	sp|Q9RTA	174						
tr|Q9RYC	tr|Q9RYC	1723						

tr|Q9RT31	tr|Q9RT31	808	50703000	42826000	35641000	67966000	52427000	65099000
sp|Q9RT6	sp|Q9RT6	170						
tr|Q9RS17	tr|Q9RS17	630	60381000	28141000	27194000	17423000	27656000	19173000
sp|Q9RUP	sp|Q9RUP	243	3.97E+08	8.66E+08	8E+08	8.32E+08	1.55E+09	8.21E+08
tr|Q9RX33	tr|Q9RX33	1519	8520000	14161000	13182000			
tr|Q9RYA9	tr|Q9RYA9	1721	9044800		13800000	36614000	24711000	3536600
tr|Q9RUX7	tr|Q9RUX7	1135				27990000	20678000	26586000
sp|Q9RYM	sp|Q9RYM	439	2.63E+09	2.62E+09	3.4E+09	1.25E+09	1.46E+09	1.62E+09
tr|Q9RVG8	tr|Q9RVG8	1230	3.14E+09	2.99E+09	2.37E+09	2.32E+09	2.29E+09	
tr|Q9RRB1	tr|Q9RRB1	492			13243000			
tr|Q9RYN4	tr|Q9RYN4	1778	67801000	65450000	82118000	93336000	63309000	87856000
tr|Q9RTC1	tr|Q9RTC1	848	6.94E+09	5.49E+09	4.71E+09	5.2E+09	5.17E+09	6.04E+09
tr|Q9RU17	tr|Q9RU17	976	1.21E+08	92457000	1.02E+08	1.81E+08	1.95E+08	2E+08
tr|Q9RU17	tr|Q9RU17	976			21116000			
tr|Q9RRE7	tr|Q9RRE7	513	79292000	39245000		4.44E+08	26453000	3.57E+08
tr|Q9RUZ9	tr|Q9RUZ9	1150				21920000	22730000	20429000
tr|Q9RXM	tr|Q9RXM	1614	53443000	82362000	1.2E+08	94946000	45039000	62851000
tr|Q9RW8	tr|Q9RW8	1367	5.07E+08	5.06E+08	1.09E+09			55177000
tr|Q9RSL7	tr|Q9RSL7	733	12431000	6223200	5151500	3191300	5141300	3685700
tr|Q9RVI8	tr|Q9RVI8	1237		46174000	47006000	51789000	60600000	
tr|Q9RW8	tr|Q9RW8	1371	55205000	75270000	68637000	1.72E+08	1.33E+08	1.36E+08
tr|Q9RSN5	tr|Q9RSN5	742	24267000	13953000	30352000			
tr|Q9RVA2	tr|Q9RVA2	1199				8955300	7990700	13447000
tr|Q9RRW	tr|Q9RRW	595						8123800
tr|Q9RS71	tr|Q9RS71	655	2.17E+08	3.51E+08	2.96E+08	2.59E+08	4.73E+08	2.93E+08
tr|Q9RZG9	tr|Q9RZG9	1926		13421000	13176000			5499700
sp|Q9RXL	sp|Q9RXL	392						
tr|Q9RUE0	tr|Q9RUE0	1044	30552000	31665000	28138000	99888000	83926000	79934000
sp|Q9RVV	sp|Q9RVV	300	4.81E+08	4.54E+08	4.23E+08	5.83E+08	4.69E+08	5.55E+08
sp|Q9RVV	sp|Q9RVV	300	20194000	17832000	39272000			
tr|Q9RXG0	tr|Q9RXG0	1587	42629000	57611000	1.04E+08			41803000
tr|Q9RRF0	tr|Q9RRF0	514	2.17E+08	1.12E+08	1.21E+08	1.32E+08	2.22E+08	1.51E+08
tr|Q9RRF0	tr|Q9RRF0	514						
tr|Q9RTV5	tr|Q9RTV5	938				29391000		31358000
tr|Q9RW8	tr|Q9RW8	1366						
tr|Q9RW9	tr|Q9RW9	1374	44996000	68651000	51337000	90666000	81385000	67600000
tr|Q9RYE1	tr|Q9RYE1	1737	72534000		89168000			
tr|Q9RU54	tr|Q9RU54	997	6.09E+09	4.67E+09	4.22E+09	5.72E+09	4.96E+09	4.95E+09
tr|Q9RY01	tr|Q9RY01	1674	19957000	19851000	15435000			
tr|Q9RX55	tr|Q9RX55	1530	1.12E+08	86866000	84995000	1.27E+08	1.09E+08	1.57E+08
sp|Q9RXJ1	sp|Q9RXJ1	376	7.28E+08	6.43E+08	7.04E+08	1.98E+08	1.99E+08	3.13E+08
tr|Q9RTE3	tr|Q9RTE3	862	1.22E+09	1.18E+09	8.83E+08	8.75E+08	8.75E+08	8.28E+08
sp|Q9RTY	sp|Q9RTY	202	44395000	39544000	48884000	61011000		
tr|Q9RRI1	tr|Q9RRI1	530	11340000	4986500	4527700	4742300	7078700	5927100
tr|Q9RY96	tr|Q9RY96	1715						
tr|Q9RZE4	tr|Q9RZE4	1914				32241000	16340000	24221000
tr|Q9RZE4	tr|Q9RZE4	1914		61947000	84594000	60924000	64632000	57278000
tr|Q9RXX2	tr|Q9RXX2	1660					20276000	40361000
tr|Q9RSY1	tr|Q9RSY1	782	83534000	44627000	67877000			
tr|Q9RX55	tr|Q9RX55	1530	3.39E+08	2.42E+08	1.75E+08	5.9E+08	5.75E+08	6.37E+08
tr|Q9RZS7	tr|Q9RZS7	1967	24980000	21128000	23078000	40953000	61642000	39589000

tr|Q9RZS7	tr|Q9RZS7	1967			12810000			
tr|Q9RV11	tr|Q9RV11	1156	1107500					
tr|Q9RV11	tr|Q9RV11	1156						
sp|P56861	sp|P56861	54						
sp|Q9RT9	sp|Q9RT9	172	3.54E+08	4.75E+08	5.25E+08	4.26E+08	4.96E+08	4.41E+08
tr|Q9RS15	tr|Q9RS15	629	59728000	55326000	52475000		35857000	40693000
tr|Q9RRW	tr|Q9RRW	595	4.98E+08	4.1E+08	4.14E+08	3.45E+08	4.26E+08	4.37E+08
tr|Q9RW2	tr|Q9RW2	1346	2.88E+08	3.87E+08	2.86E+08	3.12E+08	2.88E+08	3.32E+08
tr|Q9RXQ	tr|Q9RXQ	1627		21284000	12212000		59901000	41149000
tr|Q9RUD	tr|Q9RUD	1040	2.91E+08	3.31E+08	2.91E+08	3.85E+08	3.41E+08	4.09E+08
tr|Q9RYX6	tr|Q9RYX6	1835	41095000	46187000	51529000			
tr|Q9RTQ3	tr|Q9RTQ3	914				23082000	24915000	27251000
tr|Q9RWL	tr|Q9RWL	1437	22910000		16415000	43402000	30873000	44285000
tr|Q9RXE5	tr|Q9RXE5	1576	1.81E+08	1.77E+08	1.36E+08	77208000	1.3E+08	93843000
tr|Q9RWN	tr|Q9RWN	1441	38843000	55485000	40361000	1.76E+08	1.41E+08	1.49E+08
tr|Q9RTZ9	tr|Q9RTZ9	967			36001000	1.55E+08	1.54E+08	1.21E+08
tr|Q9RVD9	tr|Q9RVD9	1217		10012000	9706500	12169000	10858000	
tr|Q9RWW	tr|Q9RWW	1488						
tr|Q9RUG	tr|Q9RUG	1055		15194000	17537000	40267000	40961000	35902000
tr|Q9RSD0	tr|Q9RSD0	692	74236000	55000000	65554000	91506000	59735000	91596000
tr|Q9RTC7	tr|Q9RTC7	853		23557000		44630000	13222000	93340000
tr|Q9RTZ3	tr|Q9RTZ3	963					34250000	
tr|Q9RYW	tr|Q9RYW	1826	34671000	77156000	73278000	18749000	21624000	18535000
tr|Q9RVG7	tr|Q9RVG7	1229	26991000	30007000	30713000			
tr|Q9RSV3	tr|Q9RSV3	770	20501000	38646000	11278000			
sp|P56867	sp|P56867	56				24642000	28337000	
sp|Q9RYP	sp|Q9RYP	440					19831000	
tr|Q9RUY4	tr|Q9RUY4	1140	44985000	55394000	1.12E+08	70386000	91422000	84421000
tr|Q9RS49	tr|Q9RS49	644	1.07E+08	79927000				
sp|Q9RU9	sp|Q9RU9	223	42944000	45258000	36965000			
tr|Q9RYD9	tr|Q9RYD9	1735	12173000	8372800	40189000	1.45E+08	30890000	56299000
tr|Q9RT58	tr|Q9RT58	822	1.98E+08	1.9E+08	1.44E+08	1.64E+08	1.4E+08	1.8E+08
tr|Q9RWU	tr|Q9RWU	1478	2.56E+08	1.82E+08	2.22E+08	1.13E+08	1.05E+08	1.08E+08
tr|Q9RTC4	tr|Q9RTC4	851		1.66E+08	1.36E+08	1.66E+08	1.38E+08	1.47E+08
tr|Q9RVK8	tr|Q9RVK8	1248		44742000	95792000	31972000	36037000	
tr|Q9RWS	tr|Q9RWS	1464	1.53E+08	1.2E+08	1.2E+08			1.75E+08
sp|P56867	sp|P56867	56	6.28E+09	7.03E+09	5.54E+09	3.75E+09	3.28E+09	3.72E+09
tr|Q9RWI1	tr|Q9RWI1	1414						
tr|Q9RXG3	tr|Q9RXG3	1589	3.45E+08	2.29E+08	1.57E+08	99544000	1.11E+08	91994000
tr|Q9RW0	tr|Q9RW0	1334	14873000		16692000	40640000	21163000	24596000
sp|Q9RW5	sp|Q9RW5	310						13353000
tr|Q9RY79	tr|Q9RY79	1708	61520000	50783000	38911000	48012000	34359000	52843000
tr|Q9RTM	tr|Q9RTM	898	2.08E+08	2.26E+08	1.91E+08	4.86E+08	3.73E+08	4.4E+08
tr|Q9RTK4	tr|Q9RTK4	888	1.1E+08			53550000		40609000
tr|Q9RT97	tr|Q9RT97	843	12935000		40003000	1.22E+08	1.84E+08	1.92E+08
sp|Q9RX9	sp|Q9RX9	364						
tr|Q9RUK1	tr|Q9RUK1	1079	6.46E+08	2.46E+08	3.49E+08	6.72E+08	2.63E+08	6.08E+08
tr|Q9RZP7	tr|Q9RZP7	1955						
tr|Q9RWV	tr|Q9RWV	1485	26251000	20249000	12063000	24378000		
sp|Q9RU7	sp|Q9RU7	214	1.59E+08	1.96E+08	1.88E+08		1.36E+08	88793000
tr|Q9RRE7	tr|Q9RRE7	513	57027000	62623000	58747000	1.82E+08		1.66E+08

tr|Q9RRE7	tr|Q9RRE7	513	26995000	45907000	68985000	40778000	35920000	38262000
tr|Q9RWR	tr|Q9RWR	1460	38105000	30346000	59327000		17600000	21424000
tr|Q9RZU7	tr|Q9RZU7	1979	25491000	18053000	25730000	18037000	39843000	20919000
tr|Q9RYW	tr|Q9RYW	1830	20939000	44962000				44524000
tr|Q9RZA7	tr|Q9RZA7	1892	1.45E+08	1.65E+08	1.64E+08	1.28E+08	2.94E+08	1.44E+08
tr|Q9RZF1	tr|Q9RZF1	1919	3.59E+08					
tr|Q9RVJ0	tr|Q9RVJ0	1238	81457000	1.22E+08	2.19E+08	34404000	54961000	45017000
tr|Q9RVJ0	tr|Q9RVJ0	1238			78776000	1.54E+08	2.12E+08	1.43E+08
sp|Q9RRB	sp|Q9RRB	76	5.85E+08	6.42E+08	6.29E+08	2.78E+08	2.39E+08	3.36E+08
tr|Q9RT31	tr|Q9RT31	808	87348000					
tr|Q9RXE9	tr|Q9RXE9	1580	3.27E+08	4.44E+08	3E+08	2.03E+08	1.52E+08	1.96E+08
sp|Q9RZU	sp|Q9RZU	459	30124000	64393000	41457000		1.09E+08	
sp|Q9RRQ	sp|Q9RRQ	94		23390000	28531000	37163000	30915000	28508000
tr|Q9RVX1	tr|Q9RVX1	1315			88864000		37682000	
tr|Q9RTU7	tr|Q9RTU7	935	2.37E+08	1.68E+08	2.22E+08			
tr|Q9RTP0	tr|Q9RTP0	906	1.15E+08	1.15E+08	95108000	1.39E+08	1.83E+08	1.56E+08
tr|Q9RZE4	tr|Q9RZE4	1914	1.43E+08	1.02E+08	88987000			
tr|Q9RU30	tr|Q9RU30	982	12603000	31790000	5541400	72699000	1.06E+08	5641700
tr|Q9RU30	tr|Q9RU30	982	22077000	25712000	61577000	12089000	12091000	15527000
tr|Q9RXI4	tr|Q9RXI4	1603	24639000	16263000	16230000	1.31E+08	1.26E+08	1.02E+08
tr|Q9RYR7	tr|Q9RYR7	1799				29717000		
sp|Q9RW0	sp|Q9RW0	327						
sp|Q9RVC	sp|Q9RVC	279	22793000	26012000	30352000	33227000	28534000	25131000
tr|Q9RS33	tr|Q9RS33	639	1.86E+08	2.57E+08	1.93E+08	5.86E+08	5.24E+08	4.98E+08
tr|Q9RR75	tr|Q9RR75	472			24269000			
sp|Q9RWH	sp|Q9RWH	328	3.38E+08	2.76E+08	3.11E+08	4.88E+08	3.77E+08	4.32E+08
tr|Q9RUD	tr|Q9RUD	1040		12270000	14298000	20622000		16234000
tr|Q9RUD	tr|Q9RUD	1040						
tr|Q9RTP9	tr|Q9RTP9	911		1.06E+08	81587000		17607000	14374000
tr|Q9RU63	tr|Q9RU63	1005			30442000	23457000		
tr|Q9RV70	tr|Q9RV70	1186				18593000		
tr|Q9RYE5	tr|Q9RYE5	1739				88705000		57344000
tr|Q9RRA0	tr|Q9RRA0	483					11824000	
tr|Q9RSV8	tr|Q9RSV8	773						
tr|Q9RWV	tr|Q9RWV	1482	59989000	76772000	51103000	1.05E+08	2.51E+08	2.7E+08
tr|Q9RUX8	tr|Q9RUX8	1136	5.43E+08	8.73E+08	7.48E+08	1.32E+09	8.04E+08	9.85E+08
tr|Q9RY37	tr|Q9RY37	1690	1.24E+08	2.08E+08	2.09E+08	1.03E+09	7.19E+08	6.24E+08
tr|Q9RS29	tr|Q9RS29	636						
tr|Q9RU58	tr|Q9RU58	1001		22563000	20526000			
sp|Q9RTN	sp|Q9RTN	196	1.68E+08	2.91E+08	7.41E+08	1.9E+08	1.63E+08	1.88E+08
tr|Q9RT72	tr|Q9RT72	829	1.81E+08	1.97E+08	2.22E+08	2.89E+08	1.7E+08	2.63E+08
tr|Q9RXY9	tr|Q9RXY9	1667		9611000	14423000			
tr|Q9RYS1	tr|Q9RYS1	1801		25471000	44858000		13417000	9096700
tr|Q9RX97	tr|Q9RX97	1554				29833000	47774000	
tr|Q9RY75	tr|Q9RY75	1705						
tr|Q9RUD	tr|Q9RUD	1039	56902000	77131000	91491000	53839000	1.15E+08	1.11E+08
tr|Q9RW4	tr|Q9RW4	1355	2.05E+08	2.24E+08	1.76E+08			
tr|Q9RU41	tr|Q9RU41	988	69537000	71032000	61368000	98406000	91412000	93610000
sp|Q9RR7	sp|Q9RR7	67						
tr|Q9RXY9	tr|Q9RXY9	1667	59939000		80991000			81109000
tr|Q9RXV8	tr|Q9RXV8	1653						

sp|Q9RY4	sp|Q9RY4	415	3.18E+08		2.46E+08	1.73E+08		1.71E+08
sp|Q9RU7	sp|Q9RU7	215	31621000	5539200	71704000			
tr|Q9RSV5	tr|Q9RSV5	771			29101000			
sp|Q9RT3	sp|Q9RT3	166	8.57E+08	7.18E+08	7.58E+08	7.98E+08	7.04E+08	8.49E+08
tr|Q9RUN	tr|Q9RUN	1096	61644000	66040000	64725000	28768000	31586000	19023000
tr|Q9RVK7	tr|Q9RVK7	1247	85529000	84424000	58382000	85350000	69398000	64581000
tr|Q9RYW	tr|Q9RYW	1829	37877000	48353000	44471000	81977000	79550000	76737000
tr|Q9RYW	tr|Q9RYW	1829	17101000		19421000		21721000	16589000
tr|Q9RRQ	tr|Q9RRQ	562	58455000	58940000	70218000	84799000	78634000	92227000
tr|Q9RYH8	tr|Q9RYH8	1757						
tr|Q9RRD4	tr|Q9RRD4	504	1806100		4845500			
tr|Q9RWA	tr|Q9RWA	1380		23373000	28973000	18974000	14673000	19818000
sp|Q9RXK	sp|Q9RXK	390		10405000	76943000		12691000	
sp|Q9RS6	sp|Q9RS6	111	1.15E+08	5.07E+08	4.14E+08	1.26E+08	1.88E+08	1.44E+08
tr|Q9RRD5	tr|Q9RRD5	505						
tr|Q9RYW	tr|Q9RYW	1829				9608300		4627700
tr|Q9RV27	tr|Q9RV27	1164	38174000	38888000	37849000	59589000	48026000	48425000
tr|Q9RXS1	tr|Q9RXS1	1638	5.03E+08	4.97E+08	4.17E+08	4.06E+08	3.03E+08	4.19E+08
tr|Q9RV0	tr|Q9RV0	1151			1.11E+08	1.01E+08		
tr|Q9RUN	tr|Q9RUN	1097	19196000	19855000	19188000			
tr|Q9RZC7	tr|Q9RZC7	1906	39850000	72445000	71423000	1.02E+08	60855000	96837000
tr|Q9RU0	tr|Q9RU0	969	2.25E+08	1.59E+08	1.7E+08		24673000	
tr|Q9RWV	tr|Q9RWV	1483		3712800		31569000	11423000	
tr|Q9RX07	tr|Q9RX07	1506		16638000	15712000			
tr|Q9RU2	tr|Q9RU2	980			65157000			
tr|Q9RWU	tr|Q9RWU	1476	31863000			41509000	35025000	
tr|Q9RZ32	tr|Q9RZ32	1860	3E+08	2.92E+08	1.72E+08	3.33E+08	2.83E+08	3.69E+08
tr|Q9RXY9	tr|Q9RXY9	1667	4.98E+08					
tr|Q9RSF1	tr|Q9RSF1	707						
tr|Q9RWV	tr|Q9RWV	1484	1.46E+08	2.17E+08	2.31E+08			
tr|Q9RR95	tr|Q9RR95	479						
tr|Q9RZT0	tr|Q9RZT0	1968	11930000				40991000	10902000
sp|Q9RVI3	sp|Q9RVI3	289					88986000	
tr|Q9RYE5	tr|Q9RYE5	1739					29361000	17585000
tr|Q9RYP4	tr|Q9RYP4	1783	4330400		3321600			
sp|O3250	sp|O3250	46	34546000	46175000	41280000			
tr|Q9RW0	tr|Q9RW0	1336				68175000	54584000	68720000
sp|Q9RZ0	sp|Q9RZ0	450		58701000	53502000	1.65E+08	1.53E+08	1.21E+08
tr|Q9RYA9	tr|Q9RYA9	1721			66235000	37124000	45315000	57524000
tr|Q9RRI7	tr|Q9RRI7	534	1.09E+08	1.48E+08	1.42E+08	1.68E+08	1.52E+08	1.8E+08
sp|Q5933	sp|Q5933	63	6.92E+08	4.89E+08	6.17E+08	2.98E+08	1.98E+08	2.42E+08
tr|Q9RVZ4	tr|Q9RVZ4	1327	1.78E+08	2.14E+08	2E+08	4.23E+08	4.67E+08	3.77E+08
tr|Q9RST9	tr|Q9RST9	761	1.59E+08	2.44E+08	2.35E+08	2.82E+08	3.31E+08	2.81E+08
tr|Q9RRC5	tr|Q9RRC5	500	8.48E+08	8.08E+08	13228000	1.28E+09	1.04E+09	1.22E+09
sp|Q9RTJ3	sp|Q9RTJ3	189	2.19E+09	1.79E+09	1.93E+09	1.92E+09	2.13E+09	1.47E+09
sp|Q9RS2	sp|Q9RS2	108	5.96E+08	5.96E+08	7.06E+08	4.45E+08	3.34E+08	3.98E+08
tr|Q9RYI7	tr|Q9RYI7	1763	53340000	62788000	65031000	3.46E+08	3.04E+08	1.55E+08
tr|Q9RRH	tr|Q9RRH	526		33671000	26822000	40858000		32722000
sp|Q9RT7	sp|Q9RT7	171	20100000	22463000	65669000	97627000	63343000	59450000
sp|Q9RZU	sp|Q9RZU	459	2.26E+08	2.14E+08	1.56E+08	2.22E+08		2.18E+08
tr|Q9RVS1	tr|Q9RVS1	1287		39346000				

tr|Q9RXF3	tr|Q9RXF3	1583					52062000	
tr|Q9RTP5	tr|Q9RTP5	909	88238000	96780000	93690000	1.43E+08	52767000	82491000
sp|Q9RSR	sp|Q9RSR	144						
tr|Q9RRI3	tr|Q9RRI3	531	1.23E+08	1.76E+08	2.37E+08	6.46E+08	4.93E+08	5.04E+08
tr|Q9RUK	tr|Q9RUK	1081				32212000	22736000	
sp|Q9RUF	sp|Q9RUF	231	1.43E+08	1.3E+08	1.24E+08	89356000	1.02E+08	1.06E+08
tr|Q9RSV7	tr|Q9RSV7	772	37592000	38085000	51446000	31541000	20643000	27759000
sp|Q9RYB	sp|Q9RYB	426	1.31E+08	1.66E+08	2.16E+08	2.44E+08	1.15E+08	1.79E+08
tr|Q9RV59	tr|Q9RV59	1182	1.64E+08	1.17E+08	1.04E+08		1.33E+08	
tr|Q9RX55	tr|Q9RX55	1530	36370000				41660000	43974000
tr|Q9RTV1	tr|Q9RTV1	936		94274000	2.58E+08		2.08E+08	
tr|Q9RZ03	tr|Q9RZ03	1843	3.09E+08	1.99E+08		4.58E+08	3.58E+08	3.99E+08
tr|Q9RRH	tr|Q9RRH	526	25654000		27097000		30242000	17510000
tr|Q9RR95	tr|Q9RR95	479	12748000		42832000		8959200	9387200
tr|Q9RYE8	tr|Q9RYE8	1740	5.03E+08	4.92E+08	4.38E+08	2.06E+08		11130000
tr|Q9RV59	tr|Q9RV59	1182						
tr|Q9RW2	tr|Q9RW2	1341	12701000	73715000	88785000	87510000		75900000
sp|Q9RXT	sp|Q9RXT	399	2.24E+08	3.1E+08	2.37E+08	2.51E+08	2.12E+08	2.82E+08
sp|Q9RY4	sp|Q9RY4	414	16246000	10837000	9850700		15433000	
tr|Q9RS56	tr|Q9RS56	648						
tr|Q9RVT5	tr|Q9RVT5	1296		9557800				
sp|Q9RUC	sp|Q9RUC	247	77975000	89916000	57536000	35378000	36738000	28720000
tr|Q9RU54	tr|Q9RU54	997						
tr|Q9RZC4	tr|Q9RZC4	1903	1.31E+08	1.03E+08	97640000	2.03E+08	1.73E+08	1.79E+08
tr|Q9RU28	tr|Q9RU28	981	42836000	26349000	19631000	15145000		
sp|Q9RW2	sp|Q9RW2	307					24945000	
sp|Q9RW0	sp|Q9RW0	322	80333000	45093000	46688000	52616000		
tr|Q9RVB4	tr|Q9RVB4	1208	1.79E+08	1.59E+08	1.77E+08	98727000	88444000	1.06E+08
tr|Q9RVB4	tr|Q9RVB4	1208			27255000			
tr|Q9RZE9	tr|Q9RZE9	1918						
tr|Q9RRG1	tr|Q9RRG1	518	1.23E+08	90606000	1.83E+08	30547000	30285000	47360000
tr|Q9RYT5	tr|Q9RYT5	1814				1.64E+08	2.25E+08	1.26E+08
sp|Q9RWU	sp|Q9RWU	347	5.32E+08	4.74E+08	4.37E+08	2.85E+08	2.46E+08	3.49E+08
tr|Q9RRV1	tr|Q9RRV1	588		1047600	1982800		3573900	3077000
tr|Q9RVS0	tr|Q9RVS0	1286	12508000		45766000			
tr|Q9RZ94	tr|Q9RZ94	1884	61211000		57516000			28550000
tr|Q9RRY2	tr|Q9RRY2	605	76510000	1.05E+08	66458000	92191000	83996000	81385000
tr|Q9RSG4	tr|Q9RSG4	713	35654000	42443000	35301000			29396000
tr|Q9RT77	tr|Q9RT77	832				20874000		
tr|Q9RRX6	tr|Q9RRX6	604		22658000				
sp|Q9RWH	sp|Q9RWH	328	12110000	762620	515780		615950	301640
sp|Q9RYB	sp|Q9RYB	427	41456000	35537000	35416000	46676000	34239000	38527000
tr|Q9RU07	tr|Q9RU07	972			26328000			
tr|Q9RTY1	tr|Q9RTY1	956	1.38E+08	93181000		22596000	27525000	28371000
sp|Q9RY8	sp|Q9RY8	424						
sp|Q9RY8	sp|Q9RY8	424						
sp|Q9RUK	sp|Q9RUK	238	2.29E+08	1.92E+08	1.85E+08	63478000	48164000	57758000
tr|Q9RW1	tr|Q9RW1	1338	26734000	25229000	52656000	70223000	49273000	65797000
sp|Q9RTI6	sp|Q9RTI6	188	18961000	18983000	13443000	13509000	14656000	9919200
tr|Q9RU01	tr|Q9RU01	969	1.62E+08	1.86E+08	1.51E+08	2.6E+08	1.91E+08	2.11E+08
tr|Q9RTF9	tr|Q9RTF9	869	16910000	27099000		20908000		54559000

tr|Q9RXY8	tr|Q9RXY8	1666						
tr|Q9RUF9	tr|Q9RUF9	1050						
sp|Q9RVW	sp|Q9RVW	301	57338000	74228000	63472000	1.26E+08	1.1E+08	1.37E+08
tr|Q9RWK	tr|Q9RWK	1427						6781600
sp|Q9RTA	sp|Q9RTA	175			14489000			
tr|Q9RSA2	tr|Q9RSA2	673						
sp|Q9RVV	sp|Q9RVV	300	5029100	3806500	2715400	2532500	2487400	2665100
tr|Q9RSA5	tr|Q9RSA5	676	33564000	72220000	89373000	63095000	1.13E+08	71884000
tr|Q9RXN0	tr|Q9RXN0	1619				15516000	24341000	
tr|Q9RTP5	tr|Q9RTP5	909	8886100	5690700			11620000	19898000
tr|Q9RT81	tr|Q9RT81	835	76324000	56804000	55123000	77348000	71890000	70987000
tr|Q9RWV	tr|Q9RWV	1482				20055000		
tr|Q9RST7	tr|Q9RST7	760	22627000	16328000				
sp|Q9RW0	sp|Q9RW0	342						
tr|Q9RVQ	tr|Q9RVQ	1274	1.55E+08	2.01E+08	1.72E+08	8.4E+08	6.43E+08	5.83E+08
sp|Q9R34	sp|Q9R34	64	1.07E+09	1.01E+09	9.21E+08	2.73E+09	2.55E+09	2.4E+09
sp|Q9R34	sp|Q9R34	64			1.2E+08			
tr|Q9RVT5	tr|Q9RVT5	1296	68683000	61836000	61584000	1.4E+08	1.32E+08	1.08E+08
sp|Q9RXI	sp|Q9RXI	374						
tr|Q9RT43	tr|Q9RT43	815	4.17E+08	4.36E+08	4.21E+08	6.85E+08	5.97E+08	5.66E+08
sp|Q9RX9	sp|Q9RX9	363	11716000				7303500	
tr|Q9RTD3	tr|Q9RTD3	857	7.98E+08	6.92E+08	5.56E+08	7.54E+08	6.7E+08	6.86E+08
tr|Q9RWQ	tr|Q9RWQ	1454	42500000	1.03E+08	76050000	1.47E+08	1.76E+08	1.29E+08
tr|Q9RRU1	tr|Q9RRU1	584	31136000	28533000	27584000	65610000	59338000	
sp|Q9RSP	sp|Q9RSP	139	38319000	42687000	36615000	21943000	35823000	
sp|Q9RU2	sp|Q9RU2	208	27346000	27589000	30223000	16207000	23642000	
sp|Q9RU1	sp|Q9RU1	207	36300000	44042000	24630000	20049000	14890000	18782000
tr|Q9RZS3	tr|Q9RZS3	1964						
tr|Q9RXI4	tr|Q9RXI4	1603	27081000	59615000	50515000	1.23E+08	92773000	97915000
tr|Q9RZQ	tr|Q9RZQ	1959	78897000	69791000	1.28E+08	75967000	98413000	87431000
tr|Q9RZQ	tr|Q9RZQ	1959			49195000		36287000	
tr|Q9RSS2	tr|Q9RSS2	755	19559000		13028000	29307000	22320000	21575000
tr|Q9RUZ1	tr|Q9RUZ1	1144	1.91E+09	1.52E+09	1.39E+09	2.13E+09	2.23E+09	2.19E+09
tr|Q9RU93	tr|Q9RU93	1017	30990000	40771000	44419000	2.14E+08	2.19E+08	1.8E+08
tr|Q9RUX8	tr|Q9RUX8	1136			32354000			28133000
tr|Q9RYT5	tr|Q9RYT5	1814	4.2E+08	3.09E+08	2.71E+08	3.64E+08	3.49E+08	3.59E+08
sp|Q9RWI	sp|Q9RWI	338		1.24E+08	1.12E+08	2.21E+08	1.87E+08	2.07E+08
sp|Q9RRD	sp|Q9RRD	80	4146900	5416400	6631200	30273000	5489900	5854700
tr|Q9RRW	tr|Q9RRW	595						
tr|Q9RRW	tr|Q9RRW	595			27875000			
sp|Q9RRX	sp|Q9RRX	102	33766000	12130000	17063000		14041000	
tr|Q9RVX1	tr|Q9RVX1	1315	2.72E+08	2.05E+08	2.15E+08	3.28E+08	2.52E+08	3.26E+08
tr|Q9RS95	tr|Q9RS95	669						
sp|Q9RRB	sp|Q9RRB	76	1.95E+09	2.15E+09	1.66E+09	9.59E+08	8.37E+08	8.31E+08
tr|Q9RT28	tr|Q9RT28	805	1.6E+08	1.97E+08	2.19E+08		49729000	42288000
tr|Q9RTE5	tr|Q9RTE5	863	1.53E+08	1.32E+08	1.12E+08	3.58E+08	2.67E+08	3.22E+08
tr|Q9RXB0	tr|Q9RXB0	1561	1.75E+08	1.29E+08	1.52E+08	2.1E+08	2.03E+08	2.21E+08
sp|Q9RYV	sp|Q9RYV	445	3.38E+08		2.21E+08	3.52E+08	4.74E+08	3.43E+08
sp|Q9RYV	sp|Q9RYV	445	17626000	38760000	77355000	22235000	66978000	88892000
tr|Q9RTT2	tr|Q9RTT2	929	1.03E+09	9.68E+08	8.45E+08	4.58E+08	3.98E+08	4.45E+08
tr|Q9RYU9	tr|Q9RYU9	1819				77806000	97737000	

sp|Q9RW(	sp|Q9RW(	342	1.45E+08	2.96E+08	2.82E+08	4.28E+08	4.1E+08	4.22E+08
sp|Q9RW(	sp|Q9RW(	342	1E+08	2.62E+08	4.48E+08	1.53E+08	1.92E+08	2.22E+08
tr|Q9RXV1	tr|Q9RXV1	1650			4595800			
tr|Q9RS63	tr|Q9RS63	652						
tr|Q9RU07	tr|Q9RU07	972	1.02E+08	92726000	1.01E+08	1.01E+09	1.01E+09	6.72E+08
tr|Q9RUM	tr|Q9RUM	1092	70200000	55352000	59385000	27622000	21681000	31639000
sp|Q9RWI	sp|Q9RWI	343	2.87E+08	1.41E+08	2.03E+08	1.11E+08	1.42E+08	1.76E+08
sp|Q9RRX	sp|Q9RRX	102	1.86E+08	1.6E+08	1.47E+08	97720000	77796000	96922000
tr|Q9RTK5	tr|Q9RTK5	889			56449000			
tr|Q9RZT3	tr|Q9RZT3	1970	18266000	40573000	26850000			
sp|Q9RSZ	sp|Q9RSZ	160	45781000			1.67E+08	1.29E+08	1.2E+08
tr|Q9RS25	tr|Q9RS25	633	5.91E+08	6.63E+08	5.45E+08	6.99E+08	6.49E+08	7.27E+08
tr|Q9RTB3	tr|Q9RTB3	847				4.83E+08	9.23E+08	4.28E+08
tr|Q9RXM	tr|Q9RXM	1615	1573800	834350	1155100	798750	918680	915720
sp|P8358	sp|P8358	60	1.64E+09	1.93E+09	1.8E+09	2.64E+09	2.68E+09	2.33E+09
sp|Q9RTA	sp|Q9RTA	175	16449000	52671000	45146000	12559000		17354000
tr|Q9RU7(	tr|Q9RU7(	1009						
tr|Q9RSI0	tr|Q9RSI0	720	44748000		55703000		21228000	14750000
sp|Q9RT2	sp|Q9RT2	163	2.92E+08	2.39E+08	2.19E+08	2.43E+08	3.28E+08	2.07E+08
sp|Q9RT2	sp|Q9RT2	164	85113000	68853000	95127000	3.16E+08	2.53E+08	2.2E+08
tr|Q9RVT9	tr|Q9RVT9	1298	42927000	49410000	43474000	69383000	66442000	72000000
tr|Q9RVZ4	tr|Q9RVZ4	1327				30502000		
tr|Q9RUQ	tr|Q9RUQ	1104	62741000	58333000	55986000		20630000	29237000
tr|Q9RUL(	tr|Q9RUL(	1082	1.83E+08	1.95E+08	1.16E+08	4.09E+08	3.87E+08	3.48E+08
tr|Q9RXZ4	tr|Q9RXZ4	1670		23486000	21651000			
tr|Q9RZQ	tr|Q9RZQ	1959	1.02E+09	1.24E+09	1.1E+09	1.58E+09	1.31E+09	1.42E+09
tr|Q9RYS1	tr|Q9RYS1	1801				8746200	11331000	7666800
tr|Q9RUA	tr|Q9RUA	1023	47875000	62733000	68483000	3.5E+08	2.65E+08	2.88E+08
tr|Q9RVQ	tr|Q9RVQ	1271	2.48E+09	2.47E+09	2.25E+09	6.08E+08	5.3E+08	7.54E+08
tr|Q9RVB4	tr|Q9RVB4	1208						
sp|Q9RW9	sp|Q9RW9	345	19168000					
tr|Q9RT14	tr|Q9RT14	800		9872900				
tr|Q9RYW	tr|Q9RYW	1826	96037000	1.28E+08	1.21E+08	1.49E+08	63813000	1.34E+08
tr|Q9RTP5	tr|Q9RTP5	909	63391000	59702000	87818000			
sp|P56867	sp|P56867	56	1.89E+08	2.47E+08	1.52E+08	6.57E+08	4.03E+08	4.63E+08
sp|P56867	sp|P56867	56	1.29E+08	1.42E+08	1.53E+08		1.19E+08	
sp|P56867	sp|P56867	56						47902000
tr|Q9RX19	tr|Q9RX19	1514	12236000	30718000	69285000	13228000	19842000	14479000
tr|Q9RXS7	tr|Q9RXS7	1640	7.58E+08	1.7E+09	1.43E+09	1.08E+09	1.93E+09	1.15E+09
tr|Q9RV75	tr|Q9RV75	1189	71765000	86472000	86900000	97491000	62592000	78057000
tr|Q9RTM	tr|Q9RTM	902	24794000	16953000	23832000	18669000		21397000
tr|Q9RZB2	tr|Q9RZB2	1895	53154000			63757000	71910000	59384000
tr|Q9RRG5	tr|Q9RRG5	521	51246000	1.23E+08	1.07E+08			
tr|Q9RU7(	tr|Q9RU7(	1009						
tr|Q9RZV3	tr|Q9RZV3	1983	22017000	35028000	38727000			
sp|Q9RUE	sp|Q9RUE	228	30332000	31594000	25582000			
sp|Q9RUF	sp|Q9RUF	231	32327000			85268000	79607000	80310000
tr|Q9RYS6	tr|Q9RYS6	1805					11739000	
tr|Q9RV11	tr|Q9RV11	1156						
tr|Q9RXQ	tr|Q9RXQ	1628	66700000	22275000	78921000	1.98E+08	2.87E+08	4.88E+08
tr|Q9RSM	tr|Q9RSM	735						23580000

tr|Q9RSM	tr|Q9RSM	735	65964000	99236000	81222000			
tr|Q9RZP3	tr|Q9RZP3	1953			41820000		24067000	20859000
sp|Q9RXJ9	sp|Q9RXJ9	384	1.32E+09	6.1E+08	1.17E+09	1.67E+09	1.27E+09	1.47E+09
sp|Q9RXJ9	sp|Q9RXJ9	384			77840000			
tr|Q9RZA7	tr|Q9RZA7	1892			29295000	46008000	38312000	50124000
tr|Q9RZP7	tr|Q9RZP7	1955		22566000				
tr|Q9RXQ	tr|Q9RXQ	1627	1.26E+09	1.03E+09	9.03E+08	1.16E+09	1.25E+09	1.29E+09
tr|Q9RXQ	tr|Q9RXQ	1627	74381000	1.15E+08	1.72E+08		30984000	37338000
tr|Q9RT02	tr|Q9RT02	793	1.81E+08	1.37E+08	1.4E+08	5.02E+08	4.03E+08	3.86E+08
tr|Q9RVK8	tr|Q9RVK8	1248	2.21E+08	1.1E+08	1.29E+08			
tr|Q9RZJ2	tr|Q9RZJ2	1939	20881000		14040000			10835000
tr|Q9RVG6	tr|Q9RVG6	1228	3.93E+08	2.84E+08	3.45E+08	7.06E+08	8.1E+08	6.7E+08
tr|Q9RYR2	tr|Q9RYR2	1797	84901000	76914000	87880000	1.5E+08	1.64E+08	1.51E+08
sp|Q9RZ0	sp|Q9RZ0	450	5.79E+08	6.1E+08	5.24E+08	1.16E+09	1.18E+09	1.42E+09
tr|Q9RV92	tr|Q9RV92	1196						
tr|Q9RRB1	tr|Q9RRB1	492	67146000			34627000	43680000	
sp|Q9RY6	sp|Q9RY6	421			13220000	35604000	33905000	
sp|Q9RY6	sp|Q9RY6	421			27785000		20037000	19965000
sp|Q9RVC	sp|Q9RVC	279						
tr|Q9RS66	tr|Q9RS66	653	3.22E+08	3.06E+08	2.51E+08	2.88E+08	2.3E+08	3.35E+08
tr|Q9RRI4	tr|Q9RRI4	532						
tr|Q9RYW	tr|Q9RYW	1830	34068000	30904000				
tr|Q9RUB	tr|Q9RUB	1027	7.33E+08	6.22E+08	5.23E+08	6.14E+08	5.03E+08	4.62E+08
sp|Q9RVT	sp|Q9RVT	299						
sp|Q9RX5	sp|Q9RX5	359	6116100	4888800				
tr|Q9RU17	tr|Q9RU17	976						
sp|P56925	sp|P56925	58				26257000	26300000	77836000
sp|Q9RU8	sp|Q9RU8	216	3345400	976220	1113300	847680	1170800	1541100
sp|Q9RU8	sp|Q9RU8	216	1.02E+08	1.83E+08	4.24E+08	56171000	1.59E+08	54232000
tr|Q9RW8	tr|Q9RW8	1370	22864000					
sp|Q9RR6	sp|Q9RR6	65	758090	6977200	6683900	163450	8757800	
tr|Q9RXW	tr|Q9RXW	1656	1.01E+08		63893000	1.08E+08	1.02E+08	
tr|Q9RUG	tr|Q9RUG	1051	2.27E+08	37708000	89733000			
tr|Q9RVS9	tr|Q9RVS9	1293	46072000		36579000			
tr|Q9RV56	tr|Q9RV56	1180						
tr|Q9RSN4	tr|Q9RSN4	741						
tr|Q9RS52	tr|Q9RS52	646						3173000
tr|Q9RUT1	tr|Q9RUT1	1117		24544000	35032000		11961000	
tr|Q9RU60	tr|Q9RU60	1003				16815000	14156000	13704000
sp|Q9RYY	sp|Q9RYY	448		11609000		10546000		9330900
tr|Q9RYQ8	tr|Q9RYQ8	1794	90434000	1.01E+08	80619000	1.23E+08	1.25E+08	1.11E+08
tr|Q9RSJ4	tr|Q9RSJ4	729			75837000	26246000	43677000	41649000
tr|Q9RWK	tr|Q9RWK	1424	14729000		13137000			
tr|Q9RSL7	tr|Q9RSL7	733						
tr|Q9RXL5	tr|Q9RXL5	1611	1.19E+08	1.24E+08	1E+08	30939000	36107000	39514000
tr|Q9RRN	tr|Q9RRN	551		7293700	6828500			
sp|Q9RS1	sp|Q9RS1	107	5.62E+08	5.08E+08	4.62E+08	9.24E+08	8.87E+08	8.64E+08
sp|Q9RXG	sp|Q9RXG	370	97338000	88730000	80800000	63768000	63693000	75201000
tr|Q9RUY5	tr|Q9RUY5	1141		30884000	1.13E+08	1.84E+08	1.37E+08	1.44E+08
tr|Q9RXM	tr|Q9RXM	1615	33427000			6850100		
tr|Q9RYT5	tr|Q9RYT5	1814	1389200	2163600	3149600		2001500	

tr|Q9RW3	tr|Q9RW3	1347				15837000	16824000	
sp|Q9RSF	sp|Q9RSF	117	1.03E+08	1.13E+08	1.08E+08	2.33E+08	2E+08	2.27E+08
tr|Q9RZQ	tr|Q9RZQ	1957	1.8E+08	2.68E+08	2.1E+08	1.64E+08	2.13E+08	1.68E+08
tr|Q9RS17	tr|Q9RS17	630	4E+08	7.82E+08	7.6E+08	6.97E+08	5.63E+08	5.9E+08
sp|Q9RUE	sp|Q9RUE	229	10469000	3376300	2828200	2065900	3759000	2472500
tr|Q9RTX0	tr|Q9RTX0	947	21159000	15536000	23833000			
sp|P5692	sp|P5692	59	4.34E+08	3.61E+08	3.32E+08	3.21E+08	2.99E+08	2.96E+08
tr|Q9RW8	tr|Q9RW8	1366	3.74E+08	5.03E+08	4.11E+08	1.98E+08	2.03E+08	1.79E+08
sp|Q9RVW	sp|Q9RVW	301	7.13E+08	5.59E+08	5.36E+08	3.5E+08	2.56E+08	3.53E+08
tr|Q9RXM	tr|Q9RXM	1614	27075000	30804000	24575000	72894000	54138000	
sp|Q9RX9	sp|Q9RX9	363						
sp|Q9RS2	sp|Q9RS2	108		42879000				
tr|Q9RST1	tr|Q9RST1	757						
tr|Q9RRP2	tr|Q9RRP2	556	28936000	51584000	1.33E+08		47639000	38987000
tr|Q9RU54	tr|Q9RU54	997		51015000	94136000	55957000	38755000	47090000
tr|Q9RWT	tr|Q9RWT	1466						
tr|Q9RXF0	tr|Q9RXF0	1581	55547000	53663000	77485000	47587000	25546000	38047000
tr|Q9RY30	tr|Q9RY30	1685	24098000	27756000	38909000			
tr|Q9RWV	tr|Q9RWV	1484	1.11E+09	1.02E+09	9.59E+08	1.01E+09	1.09E+09	9.78E+08
tr|Q9RWV	tr|Q9RWV	1484			21743000			
tr|Q9RXP7	tr|Q9RXP7	1626				47285000		54078000
sp|Q9RR6	sp|Q9RR6	65	13808000		25954000	73100000	57177000	42480000
tr|Q9RSD0	tr|Q9RSD0	692	26851000	49661000	39674000	99163000		
tr|Q9RRA6	tr|Q9RRA6	489	98879000	1.08E+08	1.23E+08		82404000	
tr|Q9RZV4	tr|Q9RZV4	1984	29903000	23630000	26816000	14887000		
tr|Q9RZA1	tr|Q9RZA1	1888			3713100		8744000	5052000
tr|Q9RV11	tr|Q9RV11	1156			28285000		5.34E+08	4.74E+08
tr|Q9RYB8	tr|Q9RYB8	1722						
tr|Q9RVQ	tr|Q9RVQ	1272	4.48E+08	7.44E+08	4.92E+08	4.13E+08	5.66E+08	5.07E+08
tr|Q9RW1	tr|Q9RW1	1339	23392000	15466000	25278000	45715000	42561000	47347000
tr|Q9RW0	tr|Q9RW0	1333	1.04E+09	1.22E+09	8.75E+08	1.72E+09	1.57E+09	1.56E+09
tr|Q9RVA5	tr|Q9RVA5	1202	2.06E+09	2.07E+09	2.16E+09			
tr|Q9RY79	tr|Q9RY79	1708	3.55E+08	2.85E+08	3.12E+08	2.32E+08	1.84E+08	2.59E+08
sp|Q9RYL	sp|Q9RYL	436				30511000	17278000	29852000
tr|Q9RTW	tr|Q9RTW	942		25866000	28464000	20487000	19106000	22630000
tr|Q9RY39	tr|Q9RY39	1692	50389000				28445000	29809000
tr|Q9RY47	tr|Q9RY47	1695	59924000	61407000	42396000	27857000	26947000	27385000
tr|Q9RYD1	tr|Q9RYD1	1731	40691000					
tr|Q9RWP	tr|Q9RWP	1446				33562000		10522000
tr|Q9RUE0	tr|Q9RUE0	1044				18699000	15747000	14012000
sp|Q9RUF	sp|Q9RUF	231				24441000		
tr|Q9RZ10	tr|Q9RZ10	1848		28448000	39793000	9227200	26862000	52874000
tr|Q9RYV0	tr|Q9RYV0	1820	6.75E+08	8.09E+08	6.57E+08	5.18E+08	4.69E+08	5.58E+08
tr|Q9RS52	tr|Q9RS52	646	42036000	50373000	40365000	44733000	36479000	46084000
tr|Q9RZD6	tr|Q9RZD6	1910			49876000	34796000		
tr|Q9RX87	tr|Q9RX87	1552	81088000	1.32E+08	1.04E+08	86415000	61724000	93418000
sp|Q9RV9	sp|Q9RV9	272						7582400
sp|Q9RW7	sp|Q9RW7	314			5625700	34418000	41837000	38415000
sp|Q5933	sp|Q5933	63						
tr|Q9RVW	tr|Q9RVW	1312	1.36E+08	1.57E+08	1.25E+08			96268000
tr|Q9RST2	tr|Q9RST2	758	4.97E+08	5.86E+08	5.36E+08	5.41E+08	5.22E+08	5.41E+08

tr|Q9RYL1	tr|Q9RYL1	1768		29144000			56421000	
tr|Q9RV56	tr|Q9RV56	1180	31189000		25624000			35385000
tr|Q9RU76	tr|Q9RU76	1012	1.04E+08	1.37E+08	1.18E+08	1.23E+08	1.09E+08	
tr|Q9RU76	tr|Q9RU76	1012			20881000			
tr|Q9RTH3	tr|Q9RTH3	876			60713000			
tr|Q9RYP4	tr|Q9RYP4	1783	39853000	30413000	27208000			
tr|Q9RVF7	tr|Q9RVF7	1224				3681800	4518000	
sp|Q9RWI	sp|Q9RWI	330	20293000	31382000	34132000			
tr|Q9RXR7	tr|Q9RXR7	1634						
sp|Q9RUP	sp|Q9RUP	243			24884000		50460000	
tr|Q9RTX5	tr|Q9RTX5	950				16789000		
tr|Q9RW2	tr|Q9RW2	1344	93535000	1.31E+08	1.92E+08	4.22E+08	2.2E+08	2.18E+08
sp|Q9RTL8	sp|Q9RTL8	194		75351000				
tr|Q9RTW	tr|Q9RTW	946						
sp|Q9RS6	sp|Q9RS6	111	3.39E+09	3.24E+09	2.93E+09	4.5E+09	61095000	3.49E+09
sp|Q9RS6	sp|Q9RS6	111	1.03E+09	1.15E+09	1.88E+09	5.86E+08	7.61E+08	7.39E+08
tr|Q9RS17	tr|Q9RS17	630	6.2E+08	4.44E+08	7.07E+08	8.27E+08	3.91E+08	53252000
sp|Q9RU2	sp|Q9RU2	208		10106000	14522000			
tr|Q9RX18	tr|Q9RX18	1513	36765000	69805000	64174000	9927100	40289000	43825000
tr|Q9RRM	tr|Q9RRM	550	56610000	69197000	54468000	1.66E+08	1.64E+08	1.27E+08
sp|Q9RVE	sp|Q9RVE	284	12262000	4668900	5657800	3452800	5042600	4875400
tr|Q9RUB	tr|Q9RUB	1027	1.41E+08	2E+08	1.85E+08	1.3E+08	2.4E+08	1.82E+08
tr|Q9RUB	tr|Q9RUB	1027				70249000	1.17E+08	78689000
tr|Q9RRP8	tr|Q9RRP8	560						
tr|Q9RWI0	tr|Q9RWI0	1413	95040000	91768000	1.04E+08	1.16E+08	1.04E+08	1.09E+08
tr|Q9RXS7	tr|Q9RXS7	1640	4.28E+09	4.53E+09	3.35E+09	5.25E+09	4.57E+09	4.31E+09
sp|Q9RVV	sp|Q9RVV	300				28730000	46861000	51997000
tr|Q9RVQ	tr|Q9RVQ	1274	21501000	10554000	35069000			
tr|Q9RT53	tr|Q9RT53	821						
tr|Q9RZ07	tr|Q9RZ07	1845	11363000	12992000	12720000	10869000	11906000	14304000
tr|Q9RWP	tr|Q9RWP	1445		83733000				
tr|Q9RZL6	tr|Q9RZL6	1944	2E+08	3.41E+08	5.34E+08	1.31E+09	1.35E+09	7.24E+08
tr|Q9RT71	tr|Q9RT71	828	1.49E+08	1.6E+08	1.12E+08	1.06E+08	1.25E+08	1.17E+08
sp|Q9RWI	sp|Q9RWI	330	82479000	73270000	52790000		17765000	
tr|Q9RUY4	tr|Q9RUY4	1140			54784000		55444000	63191000
sp|Q9RSI4	sp|Q9RSI4	121			25400000			
tr|Q9RT71	tr|Q9RT71	828	4.71E+08	4.6E+08	3.18E+08	4.63E+08	4.25E+08	4.81E+08
sp|Q9RY7	sp|Q9RY7	422	14133000					
sp|Q9RY7	sp|Q9RY7	423						
sp|Q9RXE	sp|Q9RXE	368						
tr|Q9RW2	tr|Q9RW2	1346	1.25E+08	1.5E+08	1.48E+08	2.39E+08	1.75E+08	1.83E+08
tr|Q9RXI3	tr|Q9RXI3	1602	19074000	14527000	60449000	6347100		6408800
sp|Q4657	sp|Q4657	61	35963000					
tr|Q9RR62	tr|Q9RR62	467	85385000	60016000	58659000	1.27E+08	1.08E+08	1.1E+08
sp|Q9RS3	sp|Q9RS3	109			24378000			
tr|Q9RVV3	tr|Q9RVV3	1306						
sp|Q9RYM	sp|Q9RYM	438			10712000		4657000	
sp|Q9RVA	sp|Q9RVA	274	1.9E+08		78157000	1.74E+08	1.91E+08	1.7E+08
tr|Q9RZL4	tr|Q9RZL4	1943						
tr|Q9RTY0	tr|Q9RTY0	955	2.21E+08	3.58E+08	4.27E+08	1.63E+08	4.9E+08	4.15E+08
sp|Q9RUF	sp|Q9RUF	231	1.19E+08	93425000	98897000	66796000	41033000	

tr|Q9RSM	tr|Q9RSM	735				13429000		
sp|Q9RUF	sp|Q9RUF	231		13331000				
tr|Q9RW0	tr|Q9RW0	1335	28935000	41430000	37115000	69973000	53688000	43233000
tr|Q9RWV	tr|Q9RWV	1486	70224000	40700000	49332000	35673000	38881000	38688000
tr|Q9RT05	tr|Q9RT05	794					9085600	11773000
tr|Q9RSQ2	tr|Q9RSQ2	747						
tr|Q9RRW	tr|Q9RRW	595	3.18E+08	2.15E+08	2.25E+08	3.11E+08	3.26E+08	2.8E+08
tr|Q9RXM	tr|Q9RXM	1614		38208000	45186000	1.14E+08	96223000	90461000
sp|Q9RUP	sp|Q9RUP	246	70813000	1.02E+08	84631000		23088000	
tr|Q9RYQ3	tr|Q9RYQ3	1791	82868000	52533000	50631000	36484000		
tr|Q9RYS8	tr|Q9RYS8	1807	1.49E+08	4.24E+08	3.19E+08	2.09E+08	3.34E+08	2.35E+08
tr|Q9RZS7	tr|Q9RZS7	1967				11930000	7518500	8212000
tr|Q9RZS7	tr|Q9RZS7	1967	18283000		32921000	28716000	38432000	52743000
sp|Q9RWI	sp|Q9RWI	328	63969000	67003000	37220000	1.73E+08	1.19E+08	1.39E+08
tr|Q9RS21	tr|Q9RS21	632	77356000	91960000	20039000			
sp|Q9RV9	sp|Q9RV9	272	2.4E+08	2.37E+08	2.25E+08	2.02E+08	1.63E+08	1.93E+08
tr|Q9RYT5	tr|Q9RYT5	1814	43304000	49717000	74124000		52395000	76589000
tr|Q9RRF7	tr|Q9RRF7	517						
tr|Q9RVA5	tr|Q9RVA5	1202	24503000	34242000	47856000			
sp|P56867	sp|P56867	56	5.18E+09	5.85E+09	4.04E+09	6.47E+09	6.48E+09	7.27E+09
sp|P56867	sp|P56867	56	25791000	79899000	98094000			
tr|Q9RU43	tr|Q9RU43	990	2.36E+08	2.53E+08	28372000	5.37E+08	3.4E+08	3.94E+08
tr|Q9RU43	tr|Q9RU43	990	42780000	39754000	86929000		75499000	88422000
tr|Q9RYN6	tr|Q9RYN6	1780	9.03E+08	1.09E+09	1.27E+09	6.38E+08	7.17E+08	5.94E+08
tr|Q9RUZ9	tr|Q9RUZ9	1150		10191000	32920000	16111000	17425000	40093000

LFQ intens	LFQ intens	LFQ intens	LFQ intens	LFQ intens	LFQ intens	LFQ intens	LFQ intens	LFQ intens
1.02E+10	8.84E+09	9.45E+09	6.69E+09	6.35E+09	5.94E+09	4.69E+09	6.49E+09	6.23E+09
				28676000			27384000	
3.83E+08	5.1E+08	4.39E+08	4.91E+08	6.17E+08	5.18E+08	5.85E+08	3.25E+08	2.87E+08
				33375000	24229000			
			64049000	21516000		29162000		
4.7E+09	3.11E+09	3.6E+09	2.44E+09	2.31E+09	2.32E+09	1.43E+09	1.3E+09	1.36E+09
			8001100	3324100	3181600	7684900	7943100	6222500
			87584000		36106000	36873000	24371000	47816000
1.44E+08	1.74E+08	35624000	1.53E+08	3.08E+08	3.18E+08	2.29E+08	2.74E+08	3.21E+08
5021600	4803100	2995300	3982700	2684400	3413900	2085900	7303300	2546200
86641000	1.21E+08	1.47E+08	79609000	1.01E+08	88698000	75509000	40097000	60960000
			19098000	17495000	9725300	35220000	35122000	37046000
	32625000				76729000	37018000	32736000	35032000
7.88E+09	6.89E+09	9.75E+09	3.93E+09	3.65E+09	3.52E+09	2.67E+09	3.25E+09	3.15E+09
		35312000	31211000	10979000	10993000			
1.8E+09	1.48E+09	1.72E+09	1.29E+09	1.16E+09	1.21E+09	1.06E+09	1.05E+09	1.25E+09
69761000	1.46E+08	56453000	77307000	1.28E+08	1.2E+08	76173000	35016000	74486000
				25988000				
1.31E+09	1.22E+09	1.27E+09	1.29E+09	7.08E+08	9.9E+08	1.12E+09	9.42E+08	8.55E+08
5.53E+08	7.48E+08	2.77E+08	36857000	28475000	3E+08	83340000	2.95E+08	28642000
26375000	40507000	49431000	70828000	47284000	48124000	54400000	52009000	66710000
1.29E+08	1.51E+08	1.5E+08	1.91E+08	1.89E+08	1.74E+08	1.27E+08	1.44E+08	1.85E+08
1.1E+09	1.04E+09	1.09E+09	3.48E+08	9.86E+08	9.43E+08	1.06E+09	8.17E+08	1.14E+09
1.45E+09	1.53E+09	9.05E+08	1.75E+09	8.63E+08	9.99E+08	1.36E+09	1.39E+09	1.27E+09
1.47E+09	1.09E+09	1.46E+09	2.06E+09	1.42E+09	1.49E+09	2.25E+09	1.69E+09	8.55E+08
5.02E+08	5.43E+08	3.77E+08	1.25E+09	5.53E+08	6.9E+08	1.14E+09	1.34E+09	1.18E+09
			1.52E+08		64973000	1.26E+08	49745000	1.42E+08
1.04E+09	7.31E+08	1E+09	8.28E+08	6.9E+08	6.58E+08	9.31E+08	7.32E+08	8.86E+08
14934000	12205000	20963000	33655000	30897000	61858000	23525000	33979000	28503000
51387000	1.17E+08	68230000	62756000	65522000	71160000	63107000		58067000
1.96E+08	1.82E+08	1.78E+08	1.62E+08	2.15E+08	1.83E+08	2.14E+08	1.59E+08	1.82E+08
2222300		1775700						
			17992000	8296700				
			93877000	1.03E+08	93622000	1.08E+08	80824000	99339000
1.33E+08	1.46E+08	2.01E+08	3.23E+08	2.62E+08	44361000	2.52E+08	2.36E+08	2.57E+08
1.26E+08	1.15E+08	1.04E+08	69695000	73904000	1.1E+08	1.93E+08	1.63E+08	98694000
2.31E+08	2.52E+08	1.35E+08	69143000	1.15E+08	1.77E+08	2.3E+08	1.22E+08	95820000
30735000	27838000	84822000	62965000	84478000	57438000	26328000	33783000	87188000
26969000	27576000	27263000	15850000	24216000	28282000			32153000
6.9E+08	5.95E+08	7.56E+08	5.95E+08	5.58E+08	5.46E+08	6.84E+08	7.46E+08	6.63E+08
41180000			38485000					38921000
		3.34E+08	2.52E+08	3.39E+08	3.22E+08			
3269800			14312000	1134900	4945700	5958000	12471000	9444700
1.83E+08	1.66E+08	1.81E+08	1.78E+08	1.62E+08	1.45E+08	1.84E+08	1.98E+08	1.82E+08
49587000	49750000	1.02E+08	63079000	49701000	37625000	1.14E+08	82766000	60159000
					15300000			

2.16E+08	1.22E+08	2.73E+08	1.9E+08	1.44E+08	1.56E+08	2.28E+08	2.18E+08	1.69E+08
25659000	25464000		26174000	11538000	10378000	21713000		19325000
3835900		2179800					3411500	
24747000								
					23792000			
40743000	34556000	37911000	45663000	36036000	22999000	49452000	41349000	41427000
			36152000		52258000	14973000		
26992000	35287000	36040000	1.26E+08	1.34E+08	94158000	29846000		1.18E+08
	24636000					7817000		36266000
						4781900	74201000	4203200
90416000	58771000	73987000	83942000	30559000	54349000	69376000	2.31E+08	70438000
				8452200	11604000			
		21749000						34359000
							1547400	407060
42228000				22208000				
				9911700	10765000			
40828000	50645000	57243000	34605000	33058000	27511000	52270000	55946000	
18691000	18407000	26442000	29954000					
							6217900	
5.29E+08	4.76E+08	4.69E+08	2.16E+08	1.98E+08	2.56E+08	2.04E+08	1.9E+08	1.02E+08
3.49E+08	3.77E+08	5.2E+08	4.06E+08	3.02E+08	3.07E+08	2.46E+08	2.46E+08	3.01E+08
28869000	49768000	23485000	80606000	62329000	44840000	73680000	56606000	70640000
2.65E+08	3.69E+08	3.41E+08	1.93E+08	2.46E+08	2.05E+08	93720000	71819000	1.43E+08
10768000		8938600	8907400	6947500	12393000	14121000	50634000	24106000
75309000	1.12E+08	92918000	74965000	1.04E+08	1.01E+08	88814000	98112000	97491000
67263000	81656000	63870000	63792000	70982000	1.01E+08	65034000		69179000
			33848000	39719000	56412000	36476000	21425000	36650000
7.56E+09	5.21E+09	7.55E+09	1.61E+09	1.39E+09	1.3E+09	8.43E+08	8.91E+08	34900000
55049000	89800000	94279000	10494000	18352000	36250000	15236000	52330000	41630000
4490600	1035500	1928200	2970100	498460	755500	783340	4096000	910680
1.73E+08	2.49E+08		1.36E+08	2.8E+08	2.66E+08	99547000	57820000	1.08E+08
4.1E+09	5.6E+09	4.69E+09	4.01E+09	5.15E+09	4.01E+09	4.49E+09	5.83E+09	5.5E+09
3.52E+08	90319000	4.77E+08	5.9E+08	8.45E+08	9.2E+08	8.3E+08	6.15E+08	7.41E+08
10985000	9687600	7771200	52446000	94068000	61373000	35187000	52477000	65802000
3.23E+09	2.09E+09	2.99E+09	2.33E+09	1.64E+09	1.75E+09	2.34E+09	2.48E+09	2.09E+09
						35488000		
1E+08	92105000	1.18E+08	70850000	39018000	40042000	70394000	73983000	59678000
1.01E+08	1.2E+08	1.32E+08	1.51E+08	1.24E+08	1.16E+08			
				93025000	6550200			
2.66E+08	2.35E+08	2.49E+08	1.77E+08	1.5E+08	1.63E+08	1.96E+08	1.7E+08	2.01E+08
			1.14E+08	1.18E+08	1.3E+08	87975000	16377000	
		15284000	29903000		43985000			36569000
1.28E+08	1.21E+08	14222000	7.82E+08	6.96E+08	4.56E+08	2.57E+08	2.62E+08	2.6E+08
1.88E+08	2.51E+08	1.53E+08	1.06E+08	1.56E+08	1.41E+08	1.48E+08	1.14E+08	1.19E+08
			7375900	4099800	4707900	4611900		5017300

		15588000	11805000	1.02E+08	1.1E+08		84418000	66715000
				14673000				
	53542000						63073000	
	15425000		16858000	28219000	30886000	28992000		19936000
23005000	28582000	36417000		23192000				
		12442000	10223000	25526000		16505000	22897000	
80563000	99989000	98408000	1.39E+08	91320000	1.15E+08	1.01E+08	1.05E+08	77858000
46869000	1.5E+08	42672000	38424000	1.53E+08	1.69E+08		86251000	48974000
2.76E+08	1.57E+08	2.57E+08	1.69E+08	1.99E+08	1.95E+08	2.38E+08	2.7E+08	3.12E+08
2.71E+08	3.05E+08	16722000	1.95E+08	2.69E+08	3.55E+08	1.88E+08		22516000
	2.64E+08		1.43E+08	2.19E+08	1.75E+08	1.55E+08		5561800
73826000	48868000	59505000	58672000	49078000	57899000	88919000		70966000
78674000	58104000	74539000	25299000	15294000	12295000			
1.98E+08	1.45E+08	1.21E+08	44464000	44712000	38048000	15726000	28030000	17894000
					12494000			
			28670000	51700000	42450000	56300000		34125000
34509000	25605000	48767000	23809000		25946000	22639000	15449000	19584000
	3.37E+08		2.64E+08	10557000	2.84E+08	4.02E+08	3.01E+08	
1.79E+08	97488000	2.2E+08	1.56E+08	94023000	83220000	82799000	77777000	1.22E+08
				36717000	84701000			
7.44E+08	8.88E+08	7.94E+08	1.03E+09	1.06E+09	1.12E+09	1.07E+09	8.03E+08	1.02E+09
					7222800			
	61121000		80419000	1.77E+08	1.18E+08	37139000	13160000	33228000
	46092000		40353000	45807000	32971000	39052000	29902000	44112000
	78690000	25434000	72565000	52674000	69483000	64100000	52851000	58526000
63777000				20871000	17525000	12067000	18827000	15650000
26800000	6132000	27166000					16319000	7987000
					40242000		33333000	
	44988000		42655000	34304000	37073000	44934000		52559000
94434000	1.77E+08	58535000	1.53E+08	53189000	2.4E+08	1.76E+08	90378000	1.53E+08
	18302000		27812000	39558000	39998000	25582000	9214600	19969000
							3224500	
				53251000	37610000	34778000	27280000	27047000
	35654000	16300000	31216000	60743000	53221000		47682000	26782000
			10764000	9811900	8041900			4646600
80304000	1.27E+08	1.28E+08	96363000	2.06E+08	1.28E+08	51373000	86424000	71278000
		8591100						
				29510000	45016000			
				11962000	14955000			
	13659000			15451000				
					9461400			
11825000	39953000	11950000	36242000	41263000	14369000		8884300	21630000

								44762000
		37224000						
	32793000	31381000	28667000	24697000	24639000	36750000	34094000	31666000
3.03E+08	2.38E+08	3.86E+08	1.91E+08	2.18E+08	1.81E+08	26959000		
21452000	19690000	20871000	18001000	14441000	16513000	19507000	37002000	23086000
					19913000			
15978000			44830000	1.08E+08	48327000			
55333000								
37153000	42661000	18141000	71764000	80373000	19953000	96413000	30422000	36489000
6.63E+08	6.57E+08	9.66E+08	1.24E+08	2.43E+08	5.24E+08	2.44E+08	6.14E+08	6.68E+08
1290700		792640					1084600	
2.7E+08	3.04E+08	5.75E+08	3.25E+08	3.01E+08	2.4E+08	2E+08	2.78E+08	2.53E+08
1.13E+08	1.84E+08	1.42E+08	1.82E+08	1.52E+08	1.31E+08			1.19E+08
	39134000				28343000			26848000
1.97E+09	1.46E+09	2E+09	1.98E+09	1.45E+09	1.14E+09	1.95E+09	2E+09	1.9E+09
		29796000	85425000	64810000	53296000	60838000	66795000	89846000
79497000	98514000	53793000	40529000	1.15E+08	1.16E+08	1.83E+08	73126000	1.15E+08
1.04E+08	1.02E+08	89559000	40248000	74561000	91926000	93054000	87678000	97794000
					30585000	54089000		
1.53E+08	1.29E+08	80396000	99145000	1.86E+08	1.23E+08	1.47E+08	72068000	66677000
	1.2E+08	54589000	96614000	1.27E+08	1.38E+08	2.53E+08	1.36E+08	2.3E+08
1.46E+08	2.33E+08	1.6E+08	73955000	1.08E+08	1.25E+08	99356000	1.01E+08	96729000
4.71E+09	5.29E+09	7.05E+09	3.52E+09	5.24E+09	4.55E+09	6.93E+09	5.43E+09	5.67E+09
1.81E+08	2.01E+08	2.17E+08	37240000	1.89E+08	2.58E+08	4.26E+08	61424000	1.88E+08
45906000	33979000			33753000	26051000	38661000	26429000	25124000
44143000	77806000	1.09E+08	30495000	33009000	88099000	2.69E+08	1.84E+08	61501000
18698000	4992400	8900400	51666000	6511900	10876000	43020000	3.02E+08	56023000
39649000	45154000	71124000			31576000	28048000	18052000	
3.15E+08	8.03E+08	5.15E+08	3.01E+08	3.5E+08	4.56E+08	6.25E+08	5.66E+08	4.35E+08
1.48E+08	3.32E+08	1.63E+08	1.53E+08	1.7E+08	1.88E+08	73435000	1.55E+08	1.27E+08
1.09E+08	2.24E+08	1.18E+08	1.82E+08	2.66E+08	2.92E+08	1.93E+08	1.49E+08	2.31E+08
	51759000			38247000				
	1.95E+08							
				67331000	53341000			67990000
							61248000	
1383800						1254900	4029400	
96584000	2.02E+08	2.58E+08	1.47E+08	1.31E+08	1.15E+08	3.6E+08	1.67E+08	2.14E+08
1.71E+08	86173000	1.35E+08	95769000	1.08E+08	70362000	1.23E+08	1.01E+08	1.07E+08
2.99E+09	2.91E+09	2.9E+09	3.1E+09		2.86E+09	3.1E+09	2.78E+09	3.15E+09
1.14E+08	77698000	66471000	57300000	69130000	50367000	68351000	1.66E+08	1.73E+08
							36802000	
				27433000	30725000	30205000		
79504000	63758000	49464000	53331000	47396000	43207000			
	17424000	22126000	21939000			21642000		15889000
32601000			64140000	54011000	52440000	50727000		
1.24E+08	68643000	1.22E+08	1.3E+08	91814000	81164000	1.05E+08	1.05E+08	1.11E+08
		1.5E+08	2.37E+08	3.07E+08		2.87E+08	2.71E+08	12545000
2742000	2182100	2398400	2087100	1013100	1692300		5968000	1730000

			49967000	34280000	35646000	22458000	14949000	16021000
	1.06E+08	1.68E+08	1.26E+08	1.27E+08	1.1E+08	81477000	1.02E+08	1E+08
44773000	43542000	42173000	59424000	43600000	41364000	41609000	47292000	56372000
2.1E+08	2.33E+08	2.29E+08	96531000	1.02E+08	1.06E+08	1.29E+08	85143000	81204000
31545000	36956000		47437000	1.89E+08	1.49E+08	84239000	25040000	32367000
3.56E+08	4.65E+08	3.36E+08	1.83E+08	3.32E+08	4.21E+08	5.2E+08	3.42E+08	2.49E+08
1.37E+09	4.88E+08	1.32E+09	5.54E+08	3.87E+08	2.76E+08	3.27E+08	3.36E+08	3.48E+08
6.67E+08	4.92E+08	6.85E+08	6.12E+08	4.09E+08	4.36E+08	3.64E+08	3.85E+08	3.99E+08
10539000	13958000		79657000	1.85E+08	1.42E+08	1.56E+08	1.26E+08	1.22E+08
				40826000		64143000		
4.41E+08	2.42E+08	3.78E+08	3.48E+08	3.43E+08	3.38E+08		2.67E+08	1.83E+08
65633000	64447000	92893000	55617000	1.16E+08	83052000			
10728000	14338000		45141000	48316000	45436000	31386000	9459800	27087000
2.97E+08	2.27E+08	1.91E+08	3.02E+08	3.88E+08	2.48E+08	2.29E+08	2.18E+08	2.44E+08
85265000	1.09E+08	1.29E+08	49620000	54313000		33627000	35088000	
24821000		23028000	35964000	20932000	32297000	45232000	96978000	50669000
62952000	61422000	92648000	1.38E+08	1.02E+08	45900000	1.23E+08	92606000	1.3E+08
1.29E+08	1.84E+08	1.62E+08	4.54E+08	3.37E+08	3.64E+08	4.42E+08	3.74E+08	5.27E+08
63357000	69420000					38352000		
62303000	1.22E+08	60040000	75974000	4.96E+08	41727000	55749000	49263000	49417000
95156000		72155000	38659000		32031000			
87804000	92693000	1.16E+08	18757000	53020000	26917000	76297000	1.11E+08	77531000
					15896000			
7942600			36668000	26816000	29281000	20704000		
			2.08E+08	1.33E+08	1.13E+08			40650000
45694000		49946000		34109000	29961000	49853000	25848000	
				40541000	41873000	34565000		
	54054000		3.01E+08	2.31E+08	2.25E+08	1.23E+08	53985000	67125000
85371000	56244000	1.43E+08	1.05E+08	1.74E+08	1.28E+08	1.56E+08	2.13E+08	1.88E+08
21316000	27029000	34537000	13205000	13318000	11986000			
51157000	38068000		33564000		28352000			
7.34E+08	5.69E+08	6.02E+08	7.16E+08	8.09E+08	5.52E+08	7.99E+08	1.25E+09	1E+09
31091000	73977000	28072000	30252000	66933000	43105000	39219000		43657000
9.84E+08	9.93E+08	8.47E+08	6.97E+08	4.07E+08	4.26E+08	4.15E+08	3.18E+08	3.55E+08
33768000	22623000	24921000	14404000	13105000	8822600	19071000	15245000	15875000
3.83E+08	1.24E+09	3.64E+08	7.25E+08	1.26E+09	1.33E+09	1.26E+09	1.73E+08	9.96E+08
			42063000	24528000	28571000	29018000		17495000
	62020000	34149000		31464000	30116000	82567000		31250000
	26664000	32914000	43954000	53265000	51896000	67340000	60881000	59278000
				29096000				24201000
51122000			53953000	59651000	59182000	68540000	41243000	59677000
1.7E+08	2.45E+08	2.86E+08	1.42E+08	2.58E+08	2.77E+08	1.42E+08	2.77E+08	2.49E+08
			10880000	11194000	9008300			
		21795000				57449000	42014000	
					45209000		36669000	27681000
							1601500	503850

	2003800							
		14048000					45731000	
2.41E+08	8.75E+08	3.87E+08	6.89E+08	7.63E+08	9.37E+08	4.27E+08	1.86E+08	6.02E+08
4.56E+08	3.4E+08	2.79E+08	2.21E+08	3.41E+08	2.91E+08	8.79E+08	2.89E+08	4.51E+08
38895000	67362000		1.48E+08	1.73E+08	2.01E+08	73501000		27438000
1.14E+08	40391000	42033000	75958000	1.86E+08	1.1E+08			
89505000	2.92E+08	1.57E+08	55086000	1.36E+08	3.09E+08	72727000		1.22E+08
24558000	62632000	28118000	67436000	1.37E+08	1.3E+08	74714000	50569000	75445000
		6903900	31736000	22821000	30785000	31604000	38685000	37629000
				30708000	27127000			17978000
			6886700	3535700	6700600	8131900	23730000	11579000
21348000	25071000	31122000		19293000				35695000
					6702200		8762700	
7.57E+08	8.12E+08	7.84E+08	5.13E+08	5.03E+08	7.91E+08	1.47E+09	1E+09	8.57E+08
32137000	1.03E+08		81860000	1.92E+08	1.08E+08	94800000	1.29E+08	1.57E+08
			31251000	18651000	24054000	14122000	15742000	
1.05E+09	1.44E+09	9.07E+08		9.65E+08				5.23E+08
			52191000					
38448000	42437000	49366000				37929000	22549000	
			46750000	13415000	13764000			
1.24E+08		80687000	1.35E+08			1.89E+08	1.24E+08	1.6E+08
								13352000
1.75E+08	1.87E+08	1.39E+08	1.74E+08	2.27E+08	2.85E+08	1.02E+08	72132000	1.41E+08
16481000	43632000	14303000	1.15E+08	58449000	63231000	1.04E+08	97080000	1.16E+08
	52261000		1.96E+08	3.6E+08	3.39E+08	2.12E+08	22246000	1.09E+08
4124200	2252000	2718000	5210600	2618700	4168000	2618000	6406500	4228700
						25422000		
	25645000	17720000	31821000	35019000	37258000	45385000	31444000	33527000
2854100	1683200	3047300	4199800	1820600	2226500	1636000	8088400	2216800
73812000	1.26E+08		1.86E+08	3.32E+08	3.35E+08	1.39E+08		
1.78E+08	2.08E+08	2.05E+08	1.29E+08	1.37E+08	1.08E+08	90596000	88558000	1.32E+08
2.14E+09	2.1E+09	2.06E+09	2.92E+09	2.56E+09	2.63E+09	5.25E+08	3.7E+08	1.81E+09
2.46E+08	2.39E+08	2.43E+08	69855000	81205000	1.64E+08	1.02E+08	1.13E+08	62416000
				14641000				
	37791000				51896000	8616500		
		4636100			3570600			
	64790000	26576000	62660000	61046000	64900000	2.44E+08	68038000	1.96E+08
	47020000		42237000	59514000	50450000	36269000	21996000	29488000
				14916000	17623000	14748000		
7.14E+08	8.08E+08	7.85E+08	1.17E+09	1.06E+09	1.03E+09	1.66E+09	1.28E+09	1.6E+09
1.81E+08	1.78E+08	1.88E+08	1.12E+08	1.63E+08	1.27E+08	1.43E+08	95788000	1.41E+08
	13202000	23309000	32049000	1.9E+08	43208000			
76486000	65542000	76119000	83420000	40669000	46764000	1.3E+08	1.03E+08	92986000
	18033000		20043000	47742000	26990000	24640000		31610000
	1.4E+08			1.15E+08	1.11E+08		1.65E+08	71840000
			11152000					
2.67E+08	2.81E+08	2.81E+08	2.86E+08	3E+08	2.89E+08	3.11E+08	3.23E+08	4.29E+08

35782000			1.3E+08	3.48E+08	3.85E+08	4.27E+08	1.12E+08	1.26E+08
						2E+08		
	16619000			8481200	14474000	18235000		
11392000			32208000	51265000	46334000		14273000	26862000
1.2E+08	1.48E+08	1.38E+08	95997000	83296000	1.39E+08	2.29E+08	1.89E+08	1.22E+08
1.85E+08	2.85E+08	3.3E+08	93659000	2.13E+08	2.53E+08	77095000	1.43E+08	1.67E+08
	5502800	9395900	16181000	5703600	8572100	12864000		12953000
						789090	2703700	1489100
40897000	66794000	83220000	1.07E+08	1.12E+08	89237000	1.37E+08	1.26E+08	1.73E+08
				25629000	23454000	51962000		
5.8E+08	6.7E+08	5.02E+08	1.48E+09	1.61E+09	1.65E+09	1.39E+09	1.49E+09	3.3E+08
			3235500	1211800	2255200	1275400		
	11221000	13750000	15902000	21882000	15527000	26547000	11968000	21349000
16372000	7622400	9495500	17066000	9236500	21187000	17297000	78707000	23514000
95398000	1.37E+08	1.16E+08	84440000	1.34E+08	1.3E+08	71776000	50736000	91399000
80399000	1.35E+08	91453000	2.02E+08	2.3E+08	2.18E+08	2.11E+08	1.55E+08	1.75E+08
				17326000				
36041000	30369000	45328000	52337000	44178000	45042000	41890000	32573000	53892000
58749000	53909000	59146000	28136000	25649000	20228000	25339000	14726000	29022000
1.52E+08	1.39E+08	1.11E+08	20489000		22829000	35999000	26848000	28998000
	888370		1719700			2966700	7638300	
	24892000		86087000	1.46E+08	1.01E+08	70551000		
	1.13E+08			62840000	66833000			
717490							2039400	
	19745000		15673000	18318000		16480000		15756000
				53784000	38630000			
	59512000		53608000	71005000	1.34E+08	36259000		32310000
42933000	37875000	36049000			17183000		22073000	
1.13E+09	1.15E+09	7.66E+08	4.29E+08	4.98E+08	7.05E+08	9.12E+08	5.67E+08	4.78E+08
			13240000			13044000		12469000
					13215000			
40341000	66380000	60216000	1.49E+08	1.42E+08	1.35E+08	1.07E+08	1.08E+08	1.81E+08
				10261000	16896000	20368000	25766000	23406000
						41726000		
			98210000	55529000	49815000			
4521700	3631500		27636000	12788000	19906000	36899000	70204000	51725000
				10990000	15832000			
						12853000		35002000
8247000	4805000	8737700			11122000		6894500	7379000
					9946900		1.22E+08	
					5971200			24089000
72022000	93115000	59790000	1.29E+08	1.99E+08	1.86E+08	1.82E+08	1.29E+08	1.73E+08
			39111000					11785000

				25396000				
26304000	47784000	41371000	43086000	46123000	43849000	54069000	39234000	44041000
80973000	76921000	1.23E+08	91663000	1.46E+08	99907000	92124000	1.37E+08	1.61E+08
				21873000	28304000			25187000
						3846800	13370000	7342000
73052000	65672000	56853000	51406000	41213000	43502000			44202000
	12590000	13913000	17701000	19516000	12801000			13844000
	69713000		8152200	12909000	13750000	11289000	1.03E+08	19186000
			29820000	45764000	28142000			
						33855000	28041000	18283000
		34126000						25364000
			6.82E+08	4.94E+08	4.4E+08	6.83E+08	6.09E+08	6.46E+08
2.77E+08	2.66E+08	2.44E+08	3.53E+08	2.95E+08	2.55E+08	3.98E+08	4.11E+08	4.75E+08
3.74E+08	3.86E+08	2.93E+08	2.25E+08	2.88E+08	4.13E+08	6.68E+08	5.06E+08	3.65E+08
				6944100				
		38157000			27235000	34322000		
	59382000		87138000	1.01E+08	97701000	1E+08		1.01E+08
41617000	25828000	65444000	24771000	23441000	18872000	41800000	56532000	5.46E+08
				4840200				
3.56E+08	2.68E+08	3.24E+08	2.57E+08	2.59E+08	2.83E+08	3.07E+08	3.18E+08	2.39E+08
	7575500		10321000	10098000	19980000	12025000	52240000	21149000
			27897000	70861000	41013000	13841000		
28776000		25960000	55110000	49275000	16994000	75330000	47122000	47906000
17723000	16211000						44700000	43092000
24732000	27986000				24700000			
36849000	42882000	37679000	54023000	39473000	38538000	52631000	52988000	51488000
	53791000		94304000	45174000	52350000	81718000	66759000	78042000
31445000		36319000	60233000	35168000	54348000	57975000	93115000	97858000
87660000	91068000	89824000	88770000	67894000	67197000	59635000	1.06E+08	81501000
				78832000	97888000			44716000
					19037000			
2.2E+08	1.55E+08	1.42E+08	1.68E+08	1.48E+08	1.22E+08	2.04E+08	1.37E+08	2.06E+08
1.01E+08	1.54E+08	1.8E+08	1.43E+08	1.41E+08	98593000	1.05E+08	1.13E+08	1.27E+08
			44575000	1.07E+08	1.63E+08			20865000
			37891000		33579000	36405000	34420000	30613000
1.77E+09	1.36E+09	1.77E+09	1.44E+09	1.23E+09	1.39E+09	1.19E+09	1.28E+09	1.46E+09
	19077000		25268000	24975000	21985000	17374000	16612000	22892000
7653800	21618000			33130000	37639000		10091000	
			10476000	12847000				
				18298000	23273000			
		25276000		19229000	19624000		28241000	
				34485000				
8971600	3974500	9370700		3642800	5697000		12598000	3553600
	35016000			20094000				
27172000	22065000	27309000	17080000		13748000	10881000		6087300
3.62E+08	4.29E+08	4.05E+08	4.42E+08	4.19E+08	3.4E+08	5.03E+08	84172000	4.65E+08
4.04E+08	3.27E+08	3.6E+08	2.83E+08	2.64E+08	2.81E+08	3.55E+08	3.71E+08	4.23E+08

3.58E+08	3.71E+08	5.32E+08	2.74E+08	3.03E+08	2.14E+08	2.43E+08	2.53E+08	2.76E+08
67913000	72411000	48400000	46652000	57872000	59390000	36058000	42417000	26677000
45862000	32272000							
			1.49E+08		1.63E+08			1.43E+08
10252000		1489200		585390	111760		3632700	
1.23E+09	1.1E+09	1.13E+09	1.12E+09	1.2E+09	1.1E+09	1.39E+09	1.61E+09	1.67E+09
	32994000	42377000	7.42E+08	4.85E+08	4.5E+08			
1.2E+08	1.34E+08	1.9E+08		1.05E+08				
32371000	42943000	62611000	37274000	32711000	27826000	30366000	33148000	26540000
3.01E+08	2.56E+08	3.14E+08	3.33E+08	2.88E+08	2.75E+08	3.84E+08	3.89E+08	3.89E+08
					16844000	52245000	43811000	55811000
			10801000	14417000	44734000	11348000		
							6125600	
12665000	14903000		24480000	28549000	48150000	37203000	41873000	27152000
			36951000	25358000	29845000	41489000		
1.44E+08	3E+08	1.85E+08	1.82E+08	4.07E+08	5.28E+08	2.49E+08	1.6E+08	2.54E+08
			31799000					
					29671000			7999200
			21609000		18843000	19886000		
2.3E+08	4.38E+08	3.46E+08	4.42E+08	4.71E+08	4.97E+08	4.47E+08	5.39E+08	5.11E+08
							27574000	34597000
16422000	34030000	24298000	82709000	65601000	48260000		45877000	47872000
7.36E+08	6.25E+08	6.87E+08	4.85E+08	5.37E+08	5.05E+08	5.93E+08	5.19E+08	5.71E+08
69488000	42060000	54762000	25833000	39158000	38282000	69456000	56739000	42414000
		29055000	30164000	34300000	20992000	25492000	30444000	30858000
41370000	63182000	66216000	29040000	43020000	50565000	81338000	69230000	53365000
	30267000		22877000		18844000	11720000	15626000	16817000
	62629000		63256000			74651000	66403000	
5.86E+09	4.7E+09	2.98E+09	6.61E+09	5.53E+09	5.25E+09	5.04E+09	1.86E+09	5.35E+09
18980000	27218000		1.26E+08	1.6E+08	1.28E+08	56500000		25352000
94690000	84425000	1.41E+08	1.61E+08	1.08E+08	1.04E+08	95802000	87778000	84608000
	14552000		14245000	15948000		19922000		
16133000	23930000	22499000	25377000	30432000	19538000	31321000	30930000	33030000
1.26E+08	1.38E+08	1.54E+08	80615000	1.28E+08	1.16E+08	1.98E+08	2.08E+08	1.4E+08
	10642000				10333000	16013000		
1.48E+08	2.02E+08	2.62E+08	82225000	1.22E+08	1.04E+08	68323000		1.28E+08
	18524000		1.49E+08	1.28E+08	1.51E+08	1.35E+08	46680000	39355000
29244000	47222000	37483000	21993000	58048000	47398000		70789000	61463000
2E+08	1.77E+08	1.99E+08	1.45E+08	1.59E+08	1.55E+08	1.36E+08	1.56E+08	1.77E+08
			16373000	15567000		10549000		
1.51E+08	1.36E+08	1.51E+08	82229000	67997000	62553000	48224000	30529000	54728000
1.34E+08	1.21E+08	1.44E+08	1.8E+08	1.7E+08	1.35E+08	1.4E+08	1.42E+08	1.4E+08
		2E+08						
1.76E+08	1.07E+08	1.48E+08	1.87E+08	1.69E+08	1.32E+08	1.06E+08	1.1E+08	1.11E+08
				11668000	12121000			
45142000	32819000	48438000	35249000	32439000	29466000	55222000	58281000	54757000
24055000		16325000				49411000	31061000	
96398000	69608000	63491000	21575000		9130800	38427000	13587000	

2.25E+09	1.67E+09	2.23E+09	1.62E+09	1.33E+09	1.39E+09	1.59E+09	1.59E+09	1.55E+09
					20845000		39853000	24300000
5.21E+08	7.86E+08	1.2E+09	2.23E+08	7.67E+08	1.01E+09	8.45E+08	7.89E+08	7.11E+08
1.76E+08	1.14E+08	1.81E+08	1.27E+08	1.01E+08	89073000	1.65E+08	1.35E+08	1.53E+08
49427000	50027000	51404000	35032000	44452000	35072000	39279000	40157000	39660000
34821000	59923000	54931000	1.33E+08	89912000	97039000	1.41E+08	97979000	1.45E+08
2.07E+08	2.98E+08	3.01E+08	4.56E+08	4.61E+08	4.55E+08	6.3E+08	7.7E+08	9.48E+08
1.68E+08	1.58E+08	1.52E+08	52027000					
5.96E+08	5.16E+08	5.18E+08	2.8E+08	2.38E+08	2.17E+08	3.13E+08	3.09E+08	3.75E+08
			1.42E+08	1.38E+08	1.18E+08	1.4E+08	3.23E+08	1.5E+08
2.19E+08	1.99E+08	2.91E+08	1.64E+08	3.34E+08	2.07E+08	4.33E+08	4.52E+08	3.86E+08
			31459000	24724000	28095000	26068000		10823000
1.94E+08	1.65E+08	2.67E+08	3.01E+08	2.49E+08	2.04E+08	1.59E+08	1.36E+08	1.74E+08
	33322000		35240000		32023000			
						1.69E+08		
31818000	27459000	19599000	20678000	16742000	15131000	31248000	26202000	33787000
76270000	62102000	94409000	71054000	57049000	47316000			19796000
							1587300	
4123900	1591900	2417800	1785800	868000	1296800	1245300	6748000	1611400
			1336900				3115800	865110
41321000	34872000	38330000	32311000	22466000	36010000	27018000	50261000	30612000
	60073000				43775000		52201000	35273000
1.53E+08		1.84E+08	24596000	38366000	35123000			
57015000	1.63E+08	56325000	82240000	1.47E+08	1.13E+08	73015000		
						42507000		
	51102000	48224000		13060000	19905000	43301000	13852000	
39540000	56696000		19368000		33952000	41271000	29886000	20842000
		1434600	2063600	912480	1659400		4282000	1618300
				2934700	2823600	25035000	27660000	19675000
							815390	
	2.45E+08	2.33E+08	3.29E+08		2.02E+08	76488000	52781000	71063000
			52038000	87296000	69519000	63996000	55989000	77168000
85640000	74345000	1.3E+08	76107000	56028000	58900000	94018000	82279000	78323000
							33571000	
37059000	24163000		1.15E+08	1.07E+08	1.13E+08	1.46E+08	38082000	20769000
1.07E+08	1E+08	1.3E+08	1.14E+08	99951000	66058000	87102000	1.09E+08	1.19E+08
				6711900		4246800		
76210000					5354700			4186000
78484000	1.81E+08	83982000	3.1E+08	1.55E+08	3.77E+08	1.76E+08	2.73E+08	5E+08
				33639000	14381000		12987000	
1.9E+09	3E+09	1.93E+09	5.1E+09	5.7E+09	5.5E+09	5.6E+09	3E+09	4.58E+09
3.27E+08	2.55E+08	3.2E+08	2.01E+08	2.15E+08	1.88E+08	2.84E+08	2.68E+08	2.69E+08
28757000	24212000	27304000	17474000				28991000	
68705000	87943000	1.41E+08	1.09E+08	94247000	89370000	91239000	87154000	97860000
6.1E+08	4.43E+08	5.07E+08	4.83E+08	4.28E+08	3.59E+08	3.65E+08		4.83E+08

	28161000				35602000		34517000	25989000
20715000	51391000		3.5E+08	2.58E+08	2.28E+08	1.98E+08	28884000	1.95E+08
			10246000					
	47877000					51631000		
				6183300				
					6506000			
1.38E+08	1.95E+08	1.7E+08	3.51E+08	3.53E+08	2.08E+08	59473000	3.93E+08	3.6E+08
	10694000		1.14E+08	1.29E+08	92384000	42615000		
2.16E+08	2.64E+08	2.94E+08	2.5E+08	3.21E+08	2.72E+08	3.11E+08	3.06E+08	4E+08
1.7E+08	5.43E+08	1.98E+08	1.67E+08	4.59E+08	4.12E+08	2.73E+08	3E+08	3.26E+08
				1.18E+08	1E+08			1.16E+08
		16615000						
	21289000	28737000		21221000	23187000	43908000	34430000	39046000
71471000	81689000	92649000	79277000	39612000			74732000	1.18E+08
2.89E+08	3.67E+08	3E+08	1.9E+08	2.23E+08	2.73E+08	2.56E+08	1.26E+08	1.94E+08
8.99E+08	1.1E+09	5.24E+08	2.18E+08	3.38E+08	6.79E+08	6.38E+08	3.84E+08	2.46E+08
				19374000	18704000			
4.62E+08	5.16E+08	3.66E+08	3.98E+08	4.45E+08	4.74E+08	4.6E+08	5.43E+08	5.73E+08
	55792000		59669000	63528000	71319000	62999000		37628000
23025000		34788000					31173000	
	48627000	27993000	23007000	28182000	67862000	29242000	20841000	45971000
9667300	12934000	10726000		6798500	15033000		16530000	
1.04E+08	1.01E+08	1.05E+08	1.25E+08	99421000	1.03E+08	80209000	87191000	94426000
	36781000	54868000					43351000	
6.65E+08	6.74E+08	5.58E+08	6.07E+08	9.03E+08	1.3E+09	1.89E+09	1.6E+09	1.23E+09
80440000	54201000		73492000	39890000	44996000	53795000	51804000	80324000
14209000	5473800	10494000	10245000	5283400	11404000	7286500	35479000	9702600
	13252000		52117000	73515000	70501000	34918000		
			20106000	52818000	16453000			
			20686000					
			28976000	62922000	44741000	45592000		20252000
42432000	58201000	52899000	65063000	51756000	49348000	45584000	43534000	54998000
				30979000		21459000		
1.45E+08	1.34E+08	64647000	87772000	50670000	69685000	78425000	88264000	1.23E+08
			1.38E+08	2.92E+08	2.06E+08	36053000		17430000
64653000	90697000	1.11E+08	63387000	62021000	66296000	52318000	59365000	70932000
4.52E+08	5.58E+08	2.44E+08	4.88E+08	5.68E+08	5.86E+08	5.23E+08	2.93E+08	2.37E+08
27134000	25052000	27613000	19097000		19133000	47610000	25530000	27045000
18538000	31218000	13064000	9975600	18021000	26771000	30033000		
8.39E+08	6.41E+08	7.9E+08	3.25E+08	3.04E+08	3.04E+08	2.38E+08	1.78E+08	3.18E+08
42722000	25929000	47808000	14030000	45852000	57588000	16545000	1.05E+08	69390000
69512000	97740000	85274000	1.13E+08	1.05E+08	1.03E+08	1.36E+08	1.35E+08	1.24E+08
3.14E+09	3.68E+09	3.43E+09	3.46E+09	4.07E+09	4.01E+09	3.23E+09	3.83E+09	4.73E+09
7.96E+08	1.37E+09	1.12E+09	9.38E+08	8.64E+08	1.02E+09	4.11E+08	4.56E+08	5.01E+08
		22413000			10502000			
52080000	79424000	75194000	83393000	75032000	76223000	87524000	86767000	1.12E+08
			22251000	22841000	18354000	22959000	21802000	23311000
70664000	48114000	74153000						

52020000		59344000		60144000	43755000	30567000		
			25878000	31807000	19329000			9390600
24240000	41107000		39969000	68780000	70034000	14341000	27785000	50999000
			36528000					
						87614000		
			62474000	41067000	30110000	48386000	40263000	47793000
47250000	66128000	51539000	82178000	63058000	74005000	70947000	50145000	73659000
			4359700					5089000
					24955000			
86679000	46083000	82300000	99661000	69215000	61372000	80837000	60791000	1.02E+08
57644000	50307000	63719000	23984000	21257000	19797000	18193000		20813000
1.17E+09	1.01E+09	1.57E+09	9.98E+08	7.5E+08	6.69E+08	8.63E+08	7.09E+08	7.76E+08
1.67E+08	1.35E+08	1.44E+08	1.77E+08	1.55E+08	1.15E+08	1.62E+08	1.34E+08	1.52E+08
	49104000	60178000	29229000	32982000	26780000			33994000
54306000	34744000	42447000	43241000	31369000	36026000		38846000	40842000
	39008000	42113000						
1.1E+09	1.11E+09	9.12E+08	2.18E+09	2.03E+09	1.95E+09	3.51E+09	3.11E+09	3.73E+09
51599000	54723000	82550000	1.31E+08	76800000	74315000	36385000	73436000	98773000
					11975000			
1.26E+09	1.26E+09	1.25E+09	1.24E+09	1.27E+09	1.17E+09	1.67E+09	1.39E+09	1.71E+09
46707000	69857000	82855000		33342000				
9299900	35434000	8805500	19370000	50836000	25467000	30344000	96832000	41813000
50344000	40194000	42626000	87196000	99140000	68251000	53738000	71187000	89735000
3.77E+08	4.01E+08	4.4E+08	1.58E+08	2E+08	2.27E+08	2.29E+08	2.05E+08	1.18E+08
1.11E+09	2.49E+09	1.53E+09	7.08E+08	9.84E+08	1.29E+09	5.43E+08	8.74E+08	6.68E+08
			7539700	13313000	19231000	27602000		
					7697900			
2.56E+09	1.86E+09	3.19E+09	2.41E+09	1.52E+09	1.21E+09	1.98E+09	2.26E+09	1.74E+09
2.91E+09	2.72E+09	2.93E+09	3.12E+09	2.5E+09	2.58E+09	2.04E+09	1.49E+09	1.83E+09
					8510000			
10716000	19228000	7554900	27085000	19569000	15933000	28970000	24105000	17025000
	96188000		72250000	89290000	86755000	1.08E+08		79468000
39375000	53119000	19764000	52585000	1.61E+08	1.31E+08	92652000	1.07E+08	88875000
1.17E+08	1.03E+08	1.48E+08	1.11E+08	1.33E+08	1.34E+08	1.24E+08	71616000	97089000
					6884200			
1.83E+08	2.81E+08	2.47E+08	72619000	93724000	98093000	33942000	23620000	46807000
1.05E+08	1.13E+08	1.46E+08	98406000	64149000	48898000	41786000	52727000	76042000
4.26E+08	4.16E+08	5.87E+08	3.55E+08	3.19E+08	2.68E+08	4.23E+08	5.59E+08	4.48E+08
2.05E+08	2.06E+08	2.56E+08	1.54E+08	2.07E+08	1.79E+08	2.22E+08	2.13E+08	1.9E+08
			39019000		49852000	24586000	19598000	
7.26E+08	6.67E+08	5.37E+08	7.6E+08	4.62E+08	5.71E+08	6.33E+08	5.19E+08	6.66E+08
3.88E+09	3.02E+09	2.15E+09	2.87E+09	1.72E+09	2.38E+09	2.25E+09	2.35E+09	1.83E+09
16825000	2164100	3773300		1393400	2143700	2608600	17596000	2771800
1.52E+08	2.03E+08	2.27E+08	1.94E+08	2.7E+08	2.16E+08	1.71E+08	2.85E+08	3.34E+08
			1.31E+08	1.38E+08	94343000	1.17E+08	63062000	77163000
1.5E+08	2.49E+08	1.56E+08	2.46E+08	3.49E+08	2.96E+08	3.38E+08	2.07E+08	2.86E+08

	21137000			29939000	23367000	33972000	28289000	40151000
55088000	1.03E+08	68410000	45977000	73079000	63389000			46103000
32067000	57902000	37465000	1.03E+08	1.32E+08	1.51E+08	1.06E+08	70537000	1.12E+08
	54218000	38192000						
64612000	12408000	9095200	5620900	2353300	3027100	2080500	17413000	2414800
25371000	9632200	1956500	4823900	2395600	3264900			1438300
3372700	1270700	2629600	2557300	501680	922590		2066100	313300
								10735000
	44345000	57958000					37611000	24860000
1.75E+08	1.92E+08	2.52E+08	1.63E+08	1.49E+08	1.36E+08			1.15E+08
3.6E+08	3.45E+08	2.98E+08	1.3E+08	1.13E+08	1.49E+08	1.8E+08	3.09E+08	9905400
1.43E+09	2.32E+09	5.2E+08	6.74E+08	1.85E+09	1.74E+09	2.94E+09	3.16E+09	1.35E+09
2.03E+08	1.39E+08	1.86E+08	1.7E+08	1.07E+08	1.21E+08	1.62E+08	1.32E+08	1.46E+08
			38563000			45612000	32029000	
7.42E+08	5.68E+08	6.05E+08		1.04E+09	6.58E+08		27107000	20345000
				66210000		45611000		
54528000		62749000						
1.32E+08	1.84E+08	2.55E+08	17422000	31575000	2.47E+08		17424000	54447000
2.41E+09	2.53E+08	8.95E+08	1.7E+08	3.21E+08	7.23E+08	6.47E+08	1.02E+09	3.66E+08
1.95E+08	2.72E+08	3.08E+08	1.02E+08	1.5E+08	1.21E+08	61192000	50656000	68154000
		21858000		13689000	11619000		15445000	
	1.07E+08		1.1E+08	1.09E+08	96453000	1.06E+08	1.03E+08	1.51E+08
	39059000		49082000	47835000	48477000	72727000	63623000	72464000
47255000	53861000	42223000	57186000	61036000	65870000	53217000		57840000
27543000	16231000	25605000	26300000	13954000	15278000	22590000	23363000	27036000
62962000	97405000	82922000	36776000	50905000	83287000	1.78E+08	1.69E+08	1.01E+08
2.61E+09	2.11E+09	1.75E+09	7.07E+08	1.1E+09	1.3E+09	1.9E+09	2.06E+09	1.54E+09
						12217000		
6.67E+08	7.92E+08	6.88E+08	7.01E+08	6.99E+08	7.22E+08	6.83E+08	8.16E+08	7.86E+08
2.04E+09	1.63E+09	2.62E+09	1.05E+09	7.6E+08	6.73E+08	1.2E+09	1.19E+09	1.57E+09
	4340200	6683500	9384000	5726300	10290000	9933200		9711600
1.31E+09	1.16E+09	1.39E+09	24792000	1.3E+09	1.26E+09	1.76E+09	1.59E+09	1.92E+09
	67675000			46576000	39166000		41182000	
1.32E+08	1.18E+08	1.27E+08	83631000	95922000	1.11E+08	1.84E+08	1.64E+08	1.07E+08
98444000	1.24E+08	1E+08	58544000	53384000	59404000	77476000		47145000
8.5E+08	8.8E+08	1.08E+09	1.13E+08	3.57E+08	5.79E+08	87640000	2.48E+08	3.36E+08
	32837000			33438000				
				18010000				
	1.26E+08	1.07E+08	67788000		92828000		56898000	
	21357000	36659000	33511000	22619000	28439000	24388000	22495000	25107000
	22845000							
				1.07E+08	59438000			
	67535000	1.25E+08		1.09E+08		1.53E+08	1.4E+08	1.57E+08
				14333000	11016000			
3964600		19479000		14569000		3360700	11903000	
			52640000		39035000			46655000
52163000	87238000	66041000		93366000	85461000	76471000		61298000
77189000	1.75E+08	73377000	56719000	1.67E+08	1.91E+08	50987000	73882000	42134000

				3093900	34116000	12559000		
	51196000		52545000	56078000	33677000	26146000		
	1616100					5514200	54096000	2233000
2.06E+08	6.15E+08	1E+08	1.5E+09	2.23E+09	1.99E+09	1.34E+09	9.15E+08	1.21E+09
2.55E+08	2.47E+08	3.15E+08	3.9E+08	4.13E+08	2.6E+08	5.7E+08	5.12E+08	4.96E+08
			2.98E+08	2.87E+08	3.05E+08	1.32E+08	79342000	78200000
1.4E+08	2.17E+08	2.89E+08	42296000	1.1E+08	2.23E+08	47047000	1.07E+08	1.17E+08
1.32E+08	10040000	1.35E+08	83843000	1.58E+08	1.71E+08	2.18E+08	1.82E+08	1.36E+08
			40594000	2.01E+08	1.07E+08			15440000
	50340000		34305000	1.39E+08	56498000	41090000	10286000	29835000
	24981000		23984000	44093000	44722000			27716000
						18590000	21852000	
2.83E+08	2.36E+08	2.64E+08	1.55E+08	1.79E+08	2.5E+08	4.84E+08	3.55E+08	2.52E+08
	25710000			50005000		52864000	60069000	
		27980000		74573000	37897000			
1.75E+09	1.84E+09	2.13E+09	2.18E+09	2.44E+09	2.18E+09	1.73E+09	1.82E+09	2.21E+09
35333000	1.1E+08		2.16E+08	1.93E+08	1.87E+08	2.22E+08	81376000	88908000
11640000		22694000	5167000	45368000	27141000	6370700	18261000	11682000
2.55E+08	4.12E+08	3.41E+08	1.64E+08	1.57E+08	1.56E+08	1.11E+08	1.42E+08	1.78E+08
	4347000	6342800		965710	5786400	1654700	2.98E+08	3.72E+08
72443000	1.02E+08	84251000	49449000	71687000	91777000	1.32E+08	75272000	69796000
					7922300		2414300	5861100
55302000	1.17E+08	37059000	65193000	66365000	69093000	82446000	54456000	87118000
			5786400	6753300	12186000			
1.75E+08	3.21E+08	1.32E+08	2.85E+08	3.5E+08	4.02E+08	3.39E+08	2.76E+08	3.1E+08
5.3E+08	2.92E+08	4.78E+08	7.14E+08	6.29E+08	4.44E+08	5.3E+08	3.99E+08	3.94E+08
2.38E+08	2.22E+08	1.95E+08	1.53E+08	1.89E+08	1.54E+08	3.16E+08	1.63E+08	3.03E+08
			53326000	37990000	26680000	29301000	30007000	55622000
1.02E+08	86877000	1.03E+08	32494000	37324000	43248000	80349000	56977000	45101000
50432000	75516000	53855000	48745000	73353000	59855000	55408000	40402000	57685000
31098000	33597000	27528000		34905000	27032000	29129000	40496000	44600000
			44961000	1.06E+08	61170000	22432000		10956000
28930000	44263000	53341000						
52505000	50800000	40082000	48680000	57602000	45588000	41569000	43185000	64387000
			48243000					
2.2E+08	1.99E+08		1.49E+08	1.19E+08	1.02E+08	56675000	1.03E+08	54247000
2.16E+08	1.41E+08	3.05E+08	35841000	23014000	21291000			9156200
					24038000			21953000
							12792000	
6.65E+08	5.74E+08	6.58E+08	4.14E+08	3.11E+08	2.82E+08	2.69E+08	2.01E+08	2.47E+08
77582000		78639000	88508000	70363000	64834000	84830000	1.08E+08	
5.48E+08	4.69E+08	6.78E+08	4.9E+08	4.64E+08	4.73E+08	7.72E+08	6.94E+08	5.15E+08
3.82E+08	1E+09	3.98E+08	3.35E+08	5.64E+08	8.13E+08	8.27E+08	4.39E+08	3.89E+08
35779000	50027000		2.21E+08	1.92E+08	2.06E+08	2.47E+08	70866000	59877000

9676600			5169100	3155600	3715400	5291300	19998000	7395700
4.95E+09	4.96E+09	5.66E+09	7.31E+09	5.35E+09	4.7E+09	5.43E+09	6.08E+09	8.57E+09
	23330000				41403000	35900000		
37166000	34426000	38525000	50603000	48511000	47745000	35932000	52708000	53962000
	1.05E+08		1.52E+08	2.51E+08	2.2E+08	2.08E+08	56692000	1.25E+08
69024000	9996500	78034000		50272000				
7.76E+09	7.25E+09	9.06E+09	1.16E+10	1.23E+10	9.88E+09	8.74E+09	1.05E+10	1.11E+10
71597000	1.02E+08		4.22E+08	7.78E+08	3.03E+08	2.03E+08		1.02E+08
					241920			
8.8E+08	9.29E+08	1E+09	2.45E+09	2.15E+09	2.94E+09	1.64E+09	2.91E+09	2.92E+09
1.22E+08	1.73E+08		2.55E+08	2.98E+08	2.14E+08	1.66E+08	65101000	1.4E+08
6.85E+09	6.46E+09	5.51E+09	1.2E+10	8.62E+09	9.71E+09	1.51E+10	1.31E+10	1.34E+10
22805000	70077000	42896000	1.29E+08	3.17E+08	2.45E+08	1.78E+08	1.76E+08	1.64E+08
	61821000	46977000	39761000	55430000	59607000	71732000	43859000	53675000
22928000	24104000	39179000	30827000		19140000	34950000	36392000	32111000
3.09E+08	3.29E+08	2.85E+08	2.72E+08	3.6E+08	2.58E+08	2.58E+08	2.65E+08	3.67E+08
				68003000	30601000	33788000		16389000
		20553000					12336000	
8.78E+08	8.21E+08	1.27E+09	6.02E+08	4.77E+08	3.5E+08	2.89E+08	3.25E+08	2.98E+08
1.29E+08	1.37E+08	1.24E+08	2.79E+08	2.13E+08	2.19E+08	2.46E+08	2E+08	2.52E+08
				8163800				
3.63E+08	3.06E+08	4.78E+08	8773400			4487000	2.33E+08	25571000
	18219000							
30408000	45881000	57197000	89770000	24554000	62402000	1.52E+08	31069000	67594000
19972000				12877000	18076000			
1.6E+08	3.09E+08	33226000	3.24E+08	3.84E+08	3.17E+08	2E+08	38852000	1.55E+08
			2.23E+08	1E+08	47476000	37035000		14195000
39257000	29260000				25997000	24633000		41960000
1.08E+08	1.28E+08	81325000	63669000	91754000	83284000	47672000	47317000	60120000
					21101000			
15996000	27135000	22967000		10032000	10301000			
3.33E+08	3.26E+08	4.09E+08	4.82E+08	4.68E+08	3.24E+08	4.55E+08	4.34E+08	4.8E+08
			49404000				81191000	64411000
					15402000			
			64520000					
					39728000	1.38E+08	1.01E+08	48291000
				46539000	23209000			
11903000			37335000	10907000	20231000	22294000	26658000	21967000
6063500	1331700	2157500	4146500	1129700	1737600	1115100	7344600	1742300
	618150		6861500	1575800	1843500	2401500	3773700	1667000
4.54E+08	4.46E+08	3.72E+08	4.66E+08	2.92E+08	3.96E+08	5.27E+08	4.23E+08	5.9E+08

12704000								
					14601000			
4.36E+08	4.88E+08	8.48E+08	5.22E+08	5.03E+08	3.99E+08	5E+08	4.44E+08	5.39E+08
	21581000							
					14855000			
34956000		38143000		17166000	12040000			
6.86E+09	5.41E+09	6.15E+09	5.25E+09	4.37E+09	4.34E+09	1.5E+09	2.19E+09	3.18E+09
2.32E+08	5.65E+08	1.02E+08	1.61E+09	2.34E+09	1.94E+09	1.72E+09	6.24E+08	1.2E+09
			1.16E+08	56066000	1.25E+08	42915000		
1.34E+08	94500000	1.85E+08	1.35E+08	2.04E+08	2.44E+08	1.55E+08	3.2E+08	2.6E+08
18911000	32838000	26458000						
		21539000		15537000	23539000	26483000	14463000	23153000
	26312000		11485000	40290000	32776000			
18241000	13481000		98639000	43493000	60885000	1.07E+08	1.76E+08	1.29E+08
9.16E+08	6.18E+08	1.03E+09	5.09E+08	3.83E+08			5.76E+08	5.71E+08
	23868000		26195000	83602000	88988000	67073000	11450000	40551000
	29342000	27330000	1E+08	83088000	68087000	1.18E+08	89025000	1.02E+08
9818700		8654200	39164000	22526000	33109000	40357000	96376000	68844000
1.35E+08	1.34E+08	1.42E+08	1.49E+08	1.16E+08	1.04E+08	1.82E+08	62836000	1.81E+08
4513600			605750		419550		3937200	543680
				14133000				
	63895000	42024000	65514000	64717000	58360000	21781000	70215000	90557000
5.35E+08	5.94E+08	9.04E+08	5.84E+08	5.5E+08	9.45E+08	5.03E+08	5.08E+08	7.06E+08
62533000	50124000	69490000		34897000	75664000	15348000	15653000	
91464000	77305000	1.34E+08	62046000	53417000	41754000	25701000	32956000	42418000
6940600		8550400	11507000	9052900	12158000	10693000	20238000	17949000
2.67E+08	2.54E+08	2.42E+08	1.64E+08	2.02E+08	1.65E+08	1.67E+08	1.51E+08	1.7E+08
4.96E+09	6.3E+09	4.92E+09	9.34E+09	8.47E+09	7.73E+09	1.07E+10	9.01E+09	1.01E+10
				20806000			12098000	
28276000	17116000	15151000	15946000	12055000	13023000	16429000	41571000	21117000
5.68E+08	5.51E+08	7.11E+08	3.63E+08		4.15E+08	3.53E+08	3.73E+08	4.12E+08
			31401000	29382000	38511000			12985000
				21604000	23138000			45688000
			4403300	4778800	7748100	8200900	15943000	21790000
	51363000	34136000	1.26E+08	90619000	95103000	76365000	87478000	64028000
		19738000					8447500	
				39967000		30412000		
2.94E+08	3.84E+08	5.19E+08	4.33E+08	5.48E+08	4.37E+08	1.7E+08	1.88E+08	2.44E+08
1.24E+08	2.69E+08	2.11E+08	1.78E+08	2.22E+08	1.8E+08	1.89E+08	33587000	1.91E+08
				38352000	17159000	35016000		
42494000	69203000							
12761000	58916000	12331000	1.88E+08	77281000	1.01E+08	14114000	26471000	34270000

77686000	1.99E+08	2.18E+08	1.54E+08	1.21E+08	1.19E+08	1.52E+08	1.42E+08	1.81E+08
6412900	4705800	7544500		1145100		6463000	27149000	8917600
1.11E+08	33358000	42585000	82704000	1.45E+08	98556000	70770000	1.07E+08	72210000
87806000	1.45E+08		6.06E+08	8.57E+08	6.05E+08	7.96E+08	28310000	2.46E+08
20805000	21926000	22232000	31721000	22150000	24372000	26294000	28746000	36937000
				86260000	82960000			
16315000	19102000	22407000	49014000	41345000	60913000		84202000	72887000
	29701000		4.62E+08	1.85E+08	4.58E+08	2.11E+08	27408000	65682000
45833000	42291000				32437000	58747000	11847000	13084000
1.44E+09	9.2E+08	1.27E+09	8.48E+08	8.25E+08	5.89E+08	1.09E+09	8.73E+08	8.53E+08
20187000	27885000	33972000	5107900					
1.83E+08	2.28E+08	1.83E+08	1.21E+08	1.6E+08	2.21E+08	3.35E+08	2.76E+08	1.89E+08
	15539000							13753000
4.11E+08	3.5E+08	4.17E+08	4.3E+08	3E+08	3.11E+08	4.18E+08	4.69E+08	4.43E+08
37891000	40283000		45002000	69759000	84694000	75819000	25745000	30237000
	17219000	15702000	7369900	17081000	16243000		21486000	9760900
	84753000	72918000	63470000	56894000	37373000	74750000	44166000	55663000
14705000	12608000		43703000	38702000	40723000	44075000	53116000	45300000
			2348600					
1.13E+09	5.61E+08	8.46E+08	1.09E+09	5.24E+08	7.86E+08	9.88E+08	3.73E+09	1.27E+09
85509000	1.89E+08		3.79E+08	3.49E+08	3.2E+08	2.86E+08	1.37E+08	1.49E+08
3.77E+08	2.12E+08	2.53E+08	2.12E+08	1.7E+08	1.09E+08	1.84E+08	41923000	1.8E+08
		1308400	1160300	633240	868970	963710	5996200	1168900
						37367000		
			33445000	24176000	28222000			
26530000	1.01E+08	64126000	77329000	71204000	66115000	1.24E+08	82258000	99169000
1.32E+09	2.19E+09	1.51E+09	1.49E+09	1.48E+09	1.48E+09	8.92E+08	8.2E+08	9.43E+08
			11097000	4963200	7435000	2326500		
					33599000		18724000	
			6245700	46656000	31418000	4363300	12722000	
							22078000	
27230000	27693000	35222000	31393000	23207000	27634000	24598000	19937000	21914000
55927000	51708000	73642000	63441000	51629000	29914000	42386000		61657000
	9767200		25176000	44161000	26674000	24264000		19182000
1.2E+08	4.22E+08	1.45E+08	3.67E+08	5.35E+08	4.59E+08	4.15E+08		3.27E+08
9028800	17963000		13327000	20936000	12791000	5690000		
	44960000			70638000	82362000			29497000
						42341000		
1.06E+08	1.01E+08	1.13E+08	73809000	1.22E+08	86822000	53507000	1.33E+08	1.27E+08
45033000	42749000	47791000	56514000	54752000	35619000	51202000	68442000	56356000
8509200			50612000	6367600	23014000	33635000	73552000	32183000
			4087600	22609000	26392000		25300000	9805700
11386000	4.38E+08	7665900	5.8E+08	3.85E+08	3.47E+08	5.96E+08	6.08E+08	7.05E+08
			36565000	3740500	7248000	26000000	53181000	19043000
	12252000			51804000	41313000	11156000		
11297000			16038000	9607900	19942000			18541000

	13134000		20893000	11989000	14846000	19816000	16057000	
1.34E+08	3.52E+08	1.84E+08	3.46E+08	2.46E+08	1.72E+08	2.14E+08	3.05E+08	2.15E+08
3.1E+08	2.8E+08	3.57E+08	2.99E+08	2.85E+08	3.13E+08	1.44E+08	2.29E+08	1.98E+08
42421000	74089000	94220000	68111000	72700000	85156000	44760000	93758000	98902000
14811000	20503000	19922000	11740000	17062000	18200000	14646000	28379000	23558000
49537000	38252000	55997000	23777000	21076000	13761000	12969000		
2.1E+08	6.05E+08	1.94E+08	3.46E+08	6.98E+08	1.21E+09	1.13E+09	6.05E+08	5.19E+08
						1.76E+09	1.49E+09	
1.91E+08	1.3E+08	2.73E+08	71794000	90806000	91898000		74317000	
					7449700			10822000
86121000	1.13E+08	68802000	62288000					
3.47E+08	2.45E+08	2.07E+08	1.67E+08	1.14E+08	84079000	1.77E+08	85541000	1.6E+08
19149000	9959000	15868000	15952000	9762200	11583000	13029000	41170000	15261000
1.54E+09	1.97E+09	1.69E+09	5.6E+08	1.4E+09	1.32E+08	1.07E+09	1.22E+09	9.51E+08
39480000	45357000	76760000	32064000	43015000	98389000	65668000	54509000	1.11E+08
3.12E+08	2.97E+08	2.59E+08	3.08E+08	2.88E+08	2.83E+08	3.43E+08	3.25E+08	3.38E+08
							4947700	1346900
		12472000			42647000			
8807400			8782700	9433700	5969100	11380000	13821000	14711000
				9693000	12727000			
2.76E+08	2.18E+08	2.79E+08	2.33E+08	1.73E+08	1.52E+08	1.23E+08	1.23E+08	1.37E+08
	21786000							
3.28E+08	2.68E+08	2.77E+08	1.63E+08	1.95E+08	1.78E+08	1.64E+08	1.8E+08	2.28E+08
			32291000	29668000	31783000	22754000		23357000
35268000	41568000	65221000		27537000	24586000	14817000	30361000	29139000
						18994000	18147000	21077000
1.12E+09	1.05E+09	1.45E+09	1.77E+09	1.37E+09	1.08E+09	1.67E+09	1.8E+09	1.44E+09
3E+08	2.13E+08	2.61E+08	1.21E+08	1.52E+08	1.05E+08		65439000	82240000
3.37E+08	2.43E+08	3.87E+08	4.57E+08	3.14E+08	2.99E+08	2.34E+08	2.46E+08	2.92E+08
			14815000	17528000	14299000			
62817000	37459000	91322000	75077000	65101000	55290000	44593000	47057000	46583000
	82994000			55825000	91912000	43893000		30920000
86261000	69336000	77454000	81927000	51022000	62600000	90821000	89682000	90384000
1.83E+08	2.15E+08	1.78E+08		1.94E+08				
				6968100				
9253900	6082500	7825000	9056000	7654400	10617000	8915900	27604000	12518000
1.59E+08	1.32E+08	1.67E+08	1.12E+08	1.05E+08	99836000	80744000	1.31E+08	1.2E+08
56914000	35306000	92296000						
	7005800			37380000	59845000		78329000	45986000
3.29E+08	2.74E+08	2.75E+08	1.9E+08	1.53E+08	1.33E+08	87952000	1.85E+08	1.54E+08
						1.74E+08		
			36857000	27091000	28747000	21983000	31079000	40260000
28858000		66212000	43174000	69052000	42044000	68200000	58584000	98907000
		27861000						
	4799100	4728000	18654000	24915000	1.04E+08	8687800	5602700	30995000

	30575000				40914000		58036000	39511000
					12498000			
4.74E+08	3.97E+08	5.19E+08	3.44E+08	3.02E+08	2.82E+08	3.53E+08	2.79E+08	3.46E+08
			47697000	54451000	40657000		47955000	56194000
2.58E+08	1.58E+08		2.13E+08	1.5E+08	1.27E+08			1.27E+08
			75168000	53824000	71474000		49600000	48776000
1.63E+09	1.66E+09	1.99E+09	2.03E+09	1.1E+09	1.42E+09	2.69E+09	2.18E+09	2.6E+09
1.48E+08	2.51E+08	92641000	2.17E+08	1.47E+08	1.65E+08	2.42E+08	1.4E+08	1.47E+08
				7526500				
90062000	44697000	93932000	71823000	57461000	53749000	1.14E+08	1.3E+08	1.23E+08
			7475700		6345800			
22975000		39830000	30921000	44892000	54782000	14229000	79298000	79713000
			41493000	39151000	32736000	4762000		
8.43E+08	7.58E+08	1.29E+09	7E+08	7.3E+08	5.74E+08	4.21E+08	4.6E+08	5.7E+08
36994000	28378000	49744000						
			8708400	16560000				
			29798000	77563000	67064000	67358000		
5.24E+08	4.3E+08	5.83E+08	7.83E+08	2.28E+09	1.1E+09			24252000
				1.59E+08	90467000			
1.01E+08	90520000				13618000	79178000	1.06E+08	1.03E+08
86781000	1.43E+08	1.92E+08	1.65E+08	1.67E+08	3.17E+08	3.72E+08	3.03E+08	4.01E+08
12748000	18718000	28211000	1.41E+08	95941000	1.78E+08	1.7E+08	62373000	2.13E+08
				26628000				
1.14E+09	1.45E+09	1.29E+09	1.86E+09	1.69E+09	2.22E+09	2.5E+09	2.7E+09	1.74E+09
	25074000		1.21E+08	3.28E+08	5.11E+08	2.93E+08	13321000	36407000
68444000	81344000	76693000	1.24E+08	96698000	87499000	1.28E+08	1.05E+08	1.1E+08
								10382000
	9170600							
53713000	44921000	38895000	97312000	1.05E+08	1.29E+08	1.28E+08	1.21E+08	1.71E+08
1.15E+08	94074000	88360000	66552000	83484000	1.56E+08	2.98E+08	2.44E+08	1.19E+08
72936000	1.36E+08	55557000	58233000	1.27E+08	1.49E+08	91027000	69481000	73425000
		5776200			2691700		8703200	
				22186000				
						22822000	69565000	88258000
	13408000	15169000				12064000	23483000	
2.25E+08	2.27E+08	2.31E+08	2.41E+08	1.86E+08	1.68E+08	2.86E+08	2.78E+08	2.97E+08
				10843000				
80210000	61262000	28460000	92834000	68706000	61098000	1.05E+08	40528000	97981000
2.51E+08	2.95E+08	2.86E+08	4.1E+08	58672000	72020000	2.67E+08	2.59E+08	82722000
				20595000				
1.21E+08	27045000	1.31E+08	89555000	70116000	58349000	89882000	73843000	35137000
1.44E+08	2.31E+08	3.82E+08	82177000	4.52E+08	3.04E+08	1.58E+08	3.08E+08	5E+08
	23439000			43813000	36165000	17022000		

1.54E+08	3.51E+08	94332000	1.99E+08	4.86E+08	4.58E+08	2.88E+08	1.96E+08	2.49E+08
3.87E+08	2.29E+08	3.13E+08	1.41E+08	1.32E+08	90286000	1.37E+08	1.49E+08	1.37E+08
13675000	14009000	18567000	12953000		6756300			
35035000	32335000	3.58E+08	57347000	45802000	37216000	2.31E+08	3.06E+08	3.29E+08
				24231000				
71963000	1.6E+08	1.28E+08	1.9E+08	1.64E+08	1.58E+08	34474000	29656000	1.17E+08
	34435000				39295000	21083000	23762000	30409000
	34202000	39255000	36134000	38194000	31068000		18989000	
3.78E+08	5.73E+08	6.26E+08	3.73E+08	3.72E+08	3E+08	1.79E+08	2.03E+08	2.5E+08
1.49E+08	1.79E+08	1.94E+08	2.47E+08	1.91E+08	3.12E+08	2.46E+08	3.62E+08	3.36E+08
55896000	76008000	26722000	53819000	65795000	79630000	58830000	28175000	50587000
86499000	1.48E+08	1.89E+08	1.9E+08	34095000	1.25E+08	1.64E+08	1.46E+08	1.67E+08
1.55E+08	1.21E+08		3.32E+08	2.44E+08	2.89E+08	4.94E+08	3.04E+08	4.26E+08
	2451400	3248900	1970100	930560	1800700		3967100	1791600
1.14E+09	1.83E+09	1.85E+09	1.73E+09	2.52E+09	2.98E+09	4.42E+09	3.82E+09	3.77E+09
			5652300	7723600	15053000	16463000	14339000	4669100
1.16E+08	89782000	1.5E+08	1.12E+08	77926000	1.8E+08	94745000	1.26E+08	1.2E+08
18623000		13646000	12432000	8760300	40525000			34507000
			54390000	47343000	20856000	10177000		17690000
			52674000	38656000				
4.01E+08	2.63E+08	3.37E+08	3.8E+08	2.76E+08	2.67E+08	4.09E+08	3.39E+08	3.01E+08
					4176700			
			30940000	20777000	50893000	54454000		
	77166000		48780000	27608000	53045000	47112000	59970000	54927000
	14996000							
			21143000	16495000	7749800	18084000		5573900
	1.32E+08		1.25E+08	2.07E+08	2.22E+08	1.86E+08	48127000	46640000
				22246000				
1.65E+08	1.44E+08	1.85E+08	87035000	68181000	60443000	33374000	24588000	31900000
25532000	51535000	67268000	4659200	2.23E+08	2.14E+08	87740000	1.41E+08	1.74E+08
11708000	2853300		369430	1048700	1904500	12478000	56825000	10325000
			30902000	11057000		23973000		
1.15E+08	2.04E+08		2.46E+08	2.33E+08	2.96E+08	2.98E+08	91256000	77309000
		1.55E+08			5266900			
			10806000	31591000	13214000	26809000		
		1.09E+08	1.17E+08		1E+08	97321000	96887000	91985000
5.66E+08	1.07E+09	6.78E+08	8.03E+08	8.25E+08	7.76E+08	5.59E+08	5.09E+08	6E+08
			1.81E+08	1.51E+08	1.17E+08			
3.39E+08	2.05E+08	2.91E+08	2.74E+08	3.11E+08	1.78E+08	2.76E+08	4.52E+08	6.22E+08
1.46E+09	1.93E+09	2.79E+09	4.55E+09	6.3E+09	4.02E+09	3.81E+09	4.19E+09	4.2E+09
			14112000	21318000	17283000			
	5947700							7696700
					98166000			87847000
1.88E+08	9.69E+08	7.84E+08	4.97E+08	6.16E+08	1.91E+08	1.44E+08	1.8E+08	1.82E+08
							91386000	
				22214000	25687000			
13208000	22495000	33067000	29725000	63809000	54839000	61369000	85857000	1.25E+08

8087000	4492300		6943800	2927800		300600	18940000	4259500
8.25E+08	9.53E+08	8.13E+08	1.29E+09	1.25E+09	1.31E+09	1.36E+09	1.08E+09	1.22E+09
	79979000		1E+08	84540000	83807000	2E+08	1.46E+08	1.36E+08
	39067000	53688000	92520000	1.24E+08	78655000	87817000	93709000	95274000
74357000	2.4E+08	22200000	2.15E+08	3.21E+08	3.92E+08	2.52E+08	76930000	2.03E+08
				48071000	19347000			
1.98E+08	2.13E+08	1.84E+08	2.51E+08	1.9E+08		2.79E+08	2.4E+08	2.63E+08
1.87E+08	5.34E+08	3.26E+08	4.5E+08	9.29E+08	1.07E+09	3.12E+08	8.07E+08	3.42E+08
1.33E+08	2.39E+08		4.63E+08	7.04E+08	5.32E+08	4.52E+08	2.59E+08	4.1E+08
7.59E+08	7.22E+08	8.22E+08	1.64E+09	1.05E+09	1.27E+09	1.35E+09	2.94E+09	1.56E+09
4E+08	3.85E+08	4E+08		3.71E+08	3.58E+08	4.96E+08	4.23E+08	5E+08
							6566200	
26924000		19860000			22193000	12552000	30455000	21586000
1.97E+08	1.43E+08	1.24E+08	1.23E+08	93411000	74630000	91426000	78296000	75312000
42714000	60519000	66625000	52565000	57255000	44682000		58669000	82598000
5.6E+08	3.02E+08	2.81E+08		2.66E+08	2.02E+08	1.33E+08	1.07E+08	1.14E+08
6.96E+08	6.21E+08	5.81E+08	2.13E+08	2.74E+08	3.11E+08	7.17E+08	5.95E+08	4.46E+08
		52752000	64739000				26919000	
2.39E+08		83705000	4.3E+08	1.58E+08	1.67E+08	3.22E+08	6.02E+08	3.72E+08
2.06E+09	1.9E+09	1.61E+09	5.18E+09	4.55E+09	4.53E+09	7.86E+09	6.56E+09	7.35E+09
2.08E+08	85246000	3.39E+08	1.6E+08	1.55E+08	58850000	1.02E+08	1.28E+08	1.42E+08
15488000	24719000	32487000	14286000	10423000	7668300	8193900	7296600	2.67E+08
2.07E+09	2.06E+09	2.34E+09	2.15E+09	1.77E+09	1.53E+09	1.84E+09	1.34E+09	1.65E+09
5.06E+09	4.58E+09	4.01E+09	3.41E+09	4.2E+09	5.18E+09	7.69E+09	5.69E+09	3.8E+09
74106000	1.56E+08	52227000	70270000	1.11E+08	1.81E+08	1.42E+08	1.01E+08	58478000
			14250000		89435000	80887000		
52661000	70266000	67697000	46562000	51801000	49117000	1.53E+08	1.2E+08	1.24E+08
1.56E+08	2.91E+08	2.04E+08	1.28E+08	99339000	1.02E+08	95385000	98691000	1.24E+08
1.42E+08	1.39E+08	1.48E+08	1.65E+08	1.22E+08	1.21E+08	1.8E+08	1.61E+08	2.07E+08
			16232000	13142000	10667000	15731000		17282000
3143500			1217900				3150200	686730
1.23E+08	4.93E+08	1.19E+08	1.44E+08	3.13E+08	3.22E+08	1.02E+08	86709000	1.24E+08
				88789000	1.03E+08	60740000		
57624000	41486000	48713000					17576000	
17209000	45240000		18876000	43949000	59080000	44886000		
1.76E+08	1.65E+08	1.69E+08	2.11E+08	1.76E+08	1.52E+08	1.28E+08	2.31E+08	2.39E+08
1.31E+08	2.31E+08	39248000	1.52E+08	2.98E+08	1.81E+08	1.08E+08	39288000	43734000
40042000	49685000	1.09E+08	28809000	33890000	46093000	57091000	1.37E+08	1.01E+08
48759000	63616000	57956000	78192000	75160000	63430000	65374000	55916000	64283000
	26537000			34623000	16186000	24629000		
1.61E+08	1.19E+08		1.55E+08	1.33E+08	1.11E+08	1.48E+08	1.41E+08	1.96E+08
43734000	60028000	68722000	37392000	47520000	53545000	61717000	90132000	90584000
24695000	49096000		95530000	97208000	88842000	1.05E+08	51452000	60277000
6.82E+08	6.65E+08	1.07E+09	6.54E+08	6.29E+08	4.52E+08	1.09E+08	6.24E+08	6.77E+08
5.21E+08	4.15E+08	5.71E+08	4.98E+08	3.95E+08	3.97E+08	4.3E+08	4.17E+08	5.19E+08
31726000	20503000		27923000	24193000	20937000	33278000	28853000	41427000
		65974000	94529000		64790000	1.25E+08	1.15E+08	1.3E+08

2.57E+08	27297000	3.93E+08	96660000		12531000			
	30557000	38919000	13251000	15297000	21595000	29142000	43157000	30994000
						43028000	39382000	30238000
	21140000		21860000	20609000	46551000	14664000		9463200
					18040000	18316000		
1.66E+09	1.36E+09	1.38E+09	6.81E+08	7.11E+08	1.27E+09	2.43E+09	1.56E+09	1.11E+09
98961000	1.11E+08	71562000		39631000	80934000	1.22E+08	1.19E+08	57693000
2.51E+09	2.15E+09	1.76E+09	1.11E+09	1.23E+09	1.88E+09	3.75E+09	3.1E+09	1.86E+09
7.23E+08	8.1E+08	54540000	3.95E+08	6.4E+08	8.17E+08	9.25E+08	5.96E+08	4.51E+08
						41253000		
				1665200			13016000	7014300
			39276000	32796000	55149000	38246000	46261000	51199000
1.9E+08	1.6E+08	2.58E+08	3.04E+08	2.94E+08	2.74E+08	2.36E+08	2.29E+08	2.71E+08
			76242000	80916000				
5975800			6010700	2483600	2132500	3758800		3577700
	15099000	15761000					47653000	32791000
5.54E+08	1.03E+09	9.61E+08	4.28E+08	9.34E+08	8.05E+08	8.11E+08	8.07E+08	9E+08
3.91E+08	6.73E+08	3.78E+08	2.4E+08	3.53E+08	4.39E+08	25570000		31437000
53637000		47677000	12584000			23827000	51875000	65498000
							5341500	
	49604000	64910000	74348000	59463000	93781000	82635000	73402000	88172000
			33303000	44594000	43025000	35469000		
6472400			1235300		1665400	1117100		1409800
				8286000				
				10494000	33477000	16560000	4736700	
23646000	39855000	21463000	47710000	26043000	35447000			
6.41E+08	8.33E+08	6.88E+08	7.74E+08	6.34E+08	7.39E+08	8.16E+08	5.1E+08	7.51E+08
3.05E+09	4.72E+09	3.4E+09	2.78E+09	3.54E+09	3.28E+09	3.56E+09	2.31E+09	2.89E+09
				17573000	18535000			
1.16E+09	1.18E+09	1.02E+09	1.3E+09	1.73E+09	1.45E+09	2.49E+09	1.98E+09	3.23E+09
				34159000	27543000	20332000		
3.99E+08	3.21E+08	3.2E+08	3.04E+08	1.56E+08	2.48E+08	2.65E+08	2.4E+08	2.18E+08
5.83E+08	1.32E+09	2.29E+08	14675000	1.12E+09	21008000	2.46E+08	41424000	2.46E+08
				29602000				
5.94E+09	8.37E+09	8.02E+09	2.73E+09	5.31E+09	5.63E+09	2.41E+09	2.87E+09	2.88E+09
4.81E+08	3.45E+08	5.44E+08	1.11E+08	61885000	71046000	1.11E+08	1.02E+08	1.07E+08
			41039000		21853000	9339900		23625000
32828000	74463000	29423000	61926000	97604000	77924000	83464000	70797000	66415000
78317000	1.4E+08	1.48E+08	72297000	1.6E+08	1.48E+08	1.02E+08	1.19E+08	1.44E+08
3.48E+08	2.43E+08	1.57E+08	1.58E+08	1.86E+08	2.54E+08	1.42E+08	4.03E+08	1.31E+08
	21763000							
1.04E+08	83090000	1.15E+08	83636000	54939000	83075000	92849000	94689000	69503000
						1722200	7345000	

		12746000						
80913000	70186000	1.1E+08	1.1E+08	3.58E+08	1.65E+08			
36244000	53294000	9246700	1.32E+08	71287000	72722000	73850000	93501000	95305000
					13506000	15282000	17612000	
						51137000	37802000	
			9496400	10505000	9202900	4440400		
8.62E+08	7.24E+08	9.57E+08	9.89E+08	8.09E+08	7.37E+08	9.47E+08	8.74E+08	1.01E+09
17001000	26714000	15860000			4467400	21778000	31959000	16847000
							83155000	
	21219000							
19371000	41181000	25667000		37628000	79251000	30000000	22906000	28903000
	32132000	50915000	1.01E+08	90706000	1.17E+08	1.23E+08	1.57E+08	1.5E+08
33489000	30365000	27191000	36774000	31650000	25403000	31405000	41387000	29864000
22873000	43263000	23357000				13973000		
	14641000			26933000	10829000			
			915860			740200	4522600	714110
1.1E+08	73996000	63526000	1.01E+08	2.08E+08	1.71E+08			
45033000	21741000	41681000	52978000	35714000	30989000	33920000	27934000	28976000
89936000	1.3E+08	1.49E+08	1.73E+08	1.62E+08	1.16E+08	1.62E+08	1.9E+08	1.69E+08
1.52E+08	1.46E+08	2.47E+08	2.06E+08	1.68E+08	1.31E+08	1.41E+08	1.1E+08	1.83E+08
				1.04E+08				
2.9E+08	3.89E+08	1.12E+09	1.4E+09	6.23E+08	5.49E+08	8.57E+08	1.15E+09	1.72E+09
		87681000	46723000	42727000				48444000
	44016000				32358000	35724000		
8189700	12618000	24357000	12730000	28832000	23865000	15104000	44959000	33397000
	21222000		30016000	22751000	16759000		24134000	28349000
4811300	3885700	2320100	9515900	1517300	3462800	5102200	19964000	9425400
40750000	67102000		28460000	46420000	37323000	46952000	21268000	38025000
21907000	42674000	60769000	16496000	36206000	57049000	66603000	69153000	52973000
74786000	42839000	88326000	19106000		20620000		34727000	
2.24E+08	1.46E+08	2.21E+08	1.63E+08	1.53E+08	1.22E+08	1.4E+08	1.31E+08	1.59E+08
41015000	45768000	42088000	22680000	30751000	43033000	44528000	38269000	24088000
3.03E+08	4.66E+08	4.79E+08	1.83E+08	4.25E+08	3.22E+08	78600000	28447000	1.54E+08
1351600	262780	536480		283380	442440	445670	3233600	509250
			27687000	28230000	30939000			
73670000	76588000	1.09E+08	1.46E+08	1.48E+08	1.99E+08	2.15E+08	2.68E+08	3.41E+08
11336000			1879900	4516000	9502300			
77028000	85718000	76144000	81387000	70224000	99098000	86364000	62694000	78055000
2.04E+08	2.03E+08	1.8E+08	2.9E+08	2.43E+08	2.39E+08	3.77E+08	3.35E+08	3.41E+08
39148000	52757000		1.16E+08	97334000	99457000	45830000		35714000
49621000	6171100	17612000	11316000	6639500	8931800	10614000	48529000	9094800
	20423000	24898000		17712000	25994000	13888000	13383000	13011000
66571000	81675000	64266000	2.83E+08	2.85E+08	3.09E+08	1.09E+08	1.08E+08	1.33E+08
24298000	27887000	35798000	10333000	6044200	12039000			

				1090500	2639700		7567500	2618800
52249000	56792000	46068000	33323000		32000000	40666000		
	1.55E+08	74566000	90046000	1.32E+08	1.31E+08	82997000	61405000	71124000
3.67E+08	3.39E+08	2.9E+08	2.89E+08	3.74E+08	3.16E+08	3.69E+08	2.79E+08	3.69E+08
1.66E+08	2.53E+08	97287000			1.43E+08	1.42E+08		
14718000	699620	1571900					4431000	
				9910400				
9377100	2189100	2906400	3234200	1211200	1843900	1188000	9788100	2002100
			13364000		5773200	8176000	9209200	10653000
				5308300	18818000			
99031000	1.04E+08	1.21E+08	1.41E+08	94508000	1.93E+08	1.47E+08	70134000	1.2E+08
	33155000		21549000	44904000	37813000	33987000		27702000
36770000	32912000	9653700	1.2E+08	93262000	75567000	1.05E+08	61681000	41254000
36935000	26169000	36010000	53827000	28003000	43973000	55910000	1.8E+08	65362000
			31475000	23292000	22725000	34362000	53481000	47817000
72896000	63128000	82039000	63009000	50503000	44580000	29885000	52373000	48864000
	31221000	16128000	12174000		19407000	14044000	14631000	20126000
2.13E+09	3.84E+09	3.21E+09	1.06E+09	2.55E+09	2.13E+09	1.09E+09	1.13E+09	1.47E+09
2.77E+08	3.21E+08	4.12E+08	2.91E+08	3.71E+08	2.91E+08	1.98E+08	2.05E+08	1.99E+08
97779000	1.48E+08		1.52E+08	2.13E+08	1.25E+08			
4.01E+08	1.04E+09	5.79E+08	7.13E+08	7.98E+08	8.81E+08	8.39E+08	6.54E+08	6.64E+08
2.56E+08	3.76E+08	2E+08	2.57E+08	2.62E+08	1.97E+08	3.41E+08	1.84E+08	3.05E+08
9.01E+09	7.66E+09	8.19E+09	6.7E+09	4.66E+09	5.04E+09	5.93E+09	6.17E+09	6.37E+09
						21718000		
1.31E+08	1.52E+08	1.2E+08					73996000	
81351000	1.23E+08		5.54E+08	3.97E+08	4.81E+08	4.35E+08	1.33E+08	1.49E+08
15781000	42615000	20180000	28183000	64489000	81337000	25474000	19142000	53392000
2.97E+08	4.36E+08		3.5E+08	4.05E+08	3.79E+08	3.44E+08	2.38E+08	2.61E+08
5447200	1274800			1717900	2326300	2405800	15254000	3274100
			1.67E+08	71000000	40711000	64629000		39208000
	16232000		13197000	4.58E+08	2.88E+08		4.49E+08	
48386000	72757000	46322000	45867000	70647000	67827000	63085000	37375000	48555000
1.38E+08	1.71E+08	1.53E+08	3.01E+08	4.23E+08	4.21E+08	3.61E+08	4.69E+08	4.43E+08
8730200		6054800		3327400	9313600	9802200	20596000	8483000
2.92E+08	4.67E+08	2.7E+08	3.11E+08	5.84E+08	5.21E+08	3.69E+08	2.66E+08	3.25E+08
	1.1E+08	45503000	90469000	1.35E+08	1.81E+08	76277000	57219000	68152000
		19667000	19911000	26726000	21327000	29559000		
23806000	20920000	17714000	28443000	18513000	29789000	25279000	83181000	34429000
	1.17E+08				1.25E+08	1.59E+08	1.19E+08	1.86E+08
62032000	2.33E+08	68952000		1.81E+08	1.66E+08	1.47E+08	1E+08	1.15E+08
			1.04E+08	85552000	96307000	46368000		59758000
	18644000		18705000	15920000	22375000	3.53E+08		21498000
		32599000		28828000	44922000	43517000		37499000
8486100	31423000	79607000			55949000		32202000	1.13E+08
4.95E+08	4.07E+08	3.61E+08	9.85E+08	6.44E+08	7.79E+08	6.96E+08	7.71E+08	6.3E+08
4.75E+08	5.06E+08	2.02E+08	3.07E+09	1.53E+09	2.26E+09	2.98E+09	4.77E+09	3.49E+09
						27508000		
8.41E+08	8.87E+08	9.35E+08	3.79E+08	4.09E+08	4.89E+08	8.53E+08	5.9E+08	4.52E+08

7.73E+08	1.52E+09	7.71E+08	1.22E+09	1.11E+09	1.2E+09	1.22E+09	7.83E+08	9.78E+08
38674000	70350000	78981000	73022000	1.26E+08	1.59E+08	1.36E+08	1.23E+08	1.31E+08
				18946000	20588000	19893000		
5.4E+08	2.03E+08	1.82E+08	3.83E+08	1.18E+08	1.11E+08	4.06E+08	5.04E+08	5.5E+08
			37721000	70643000				
	46378000		27121000			39886000	30947000	36429000
20035000	10341000	15382000	13560000	8457100	12707000	11159000	65616000	15464000
3.89E+08	4.24E+08	3.82E+08	3.71E+08	2.86E+08	3.11E+08	3.78E+08	3.6E+08	3.45E+08
5.54E+08	4.4E+08	5.28E+08	2.93E+08	1.94E+08	2.39E+08	2.68E+08	2.28E+08	2.3E+08
42672000		72233000	59749000	65020000	70736000	1.18E+08	1.21E+08	83295000
					34271000		78064000	52584000
					15472000		24934000	20693000
6.84E+08	6.92E+08	8.99E+08	9.25E+08	6.47E+08	5.88E+08	8.72E+08	1.14E+09	1E+09
4732400	2768900	2733000	6236400	2713100	3027200	3276900	24349000	3934100
					23373000		12427000	
			17532000	15276000	12400000			
3.04E+08	3.04E+08	1.1E+08	3.38E+08	3.92E+08	3.45E+08	2.64E+08	61716000	3.21E+08
45413000								
			3.04E+08	2.03E+08	2.31E+08			
				40958000	18720000			12489000
						16088000	1.62E+08	
3.11E+09	3.38E+09	2.77E+09	2.03E+09	2.06E+09	1.96E+09	1.45E+09	1.47E+09	1.47E+09
	20376000	22472000						
				36311000				
			19165000					
3.25E+08	4.79E+08	2.02E+08	3.25E+08	2.87E+08	2.8E+08	1.98E+08	1.13E+08	1.47E+08
			23918000					
			50615000	27273000	46174000	21328000		
	42002000		47409000	49977000	51320000	46630000		43847000
2.81E+08			2.54E+08			2.43E+08	3.76E+08	
			41570000		29821000	59637000	63681000	45756000
36729000	45660000	73608000	1.39E+08	2.94E+08	1.53E+08	1.82E+08	1.7E+08	2.46E+08
5.51E+08	5.76E+08	7.28E+08	4.48E+08	3.53E+08	3.77E+08	1.47E+08	89402000	3.06E+08
	97760000			78531000	71925000		72696000	80584000
2.57E+08	3.03E+08	2.44E+08	5.49E+08	4.88E+08	5.71E+08	6.04E+08	6.03E+08	8.24E+08
	45150000	37400000	1.34E+08	87270000	86596000	1.13E+08	58968000	83716000
24999000	23808000	26723000	29565000	43051000	45080000	34784000	35921000	40359000
1.66E+08	2.62E+08	1.75E+08						
51012000		56843000		49323000				
44943000	72018000	15695000	79146000	1.01E+08	1.83E+08	2.31E+08	1.8E+08	2.01E+08
					19707000			
2.08E+08	3.31E+08	1.73E+08	3.86E+08	5.39E+08	4.97E+08	4.43E+08	3.1E+08	3.24E+08
	52971000			44452000	75367000	20972000		70481000
2108700	2046400		4322700		1821800		11090000	

	51257000		79213000	82518000	78500000	62852000	49554000	60757000
				11633000	37070000		19608000	
79291000								75831000
3.79E+08	4.08E+08	4.19E+08	5.55E+08	4.65E+08	5.35E+08	6.21E+08	6.9E+08	9.46E+08
68104000	53687000	18461000	70349000	58526000	47454000	63558000	89244000	63562000
	17935000		7696200	22734000	26602000			
71376000	1.7E+08	50145000	1.53E+08	7.7E+08	7.3E+08		20435000	8925100
8085700	31725000	19480000	17560000	40354000	33249000	20991000	26337000	40439000
					56453000			
			3618400	2364300	4592000	2854100	7775100	3360000
2.77E+08	3.47E+08	2.86E+08	1.87E+08	1.82E+08	2.5E+08	4.98E+08	4.5E+08	2.42E+08
			25342000	31700000	31253000			26628000
				19204000				
							4097400	
3.65E+09	3.98E+09	79735000	4.63E+09	4.34E+09	4.66E+09	3.45E+09	2.17E+09	3.05E+09
3.9E+09	5.39E+09	3.89E+09	4.12E+09	2.83E+09	3.17E+09	3.33E+09	3E+09	3.51E+09
			32617000	16630000	22880000			
24407000	42881000		42917000	62577000	68062000	50116000	27392000	38207000
			43206000	41369000	34868000	70770000		
7.21E+08	8.83E+08	6.72E+08	3.86E+08	4.42E+08	4.77E+08	1.81E+08	1.68E+08	2.42E+08
			40876000	52049000	22734000			
	14806000		19148000	21117000	78714000			
		10966000			13985000	22558000		
	39931000		28096000	35570000	30665000	29011000	26576000	34455000
13734000	13901000		72365000	85210000	94708000	52168000	48313000	35970000
1.01E+08	1.01E+08	90515000	73723000	85233000	76702000	67221000	49529000	73414000
36534000			42879000	50781000				
			23246000	12157000		20247000		
	51396000			52199000	44491000	40255000		
								64730000
2.06E+08	2.68E+08	3.14E+08	2.36E+08	2.4E+08	3E+08	2.43E+08	1.68E+08	1.91E+08
			51301000	84559000	91379000			
	34823000		28253000	40175000	54915000	31911000	32833000	45198000
42899000	49883000	37766000	43784000	44249000	46972000	34733000	37814000	28657000
			1.49E+08	1.45E+08	1.48E+08			
43586000	1.1E+08	79320000	1.07E+08	1.08E+08	1.67E+08	65230000	51195000	70478000
3595100		588490	314450	302060	439490		3921100	515390
5365000								
74154000	1.06E+08	59824000	7935900	41329000	10312000		1.15E+08	68183000
	30963000	27551000			35470000			
	84986000		70571000	88104000	92768000	1.84E+08	1.5E+08	1.43E+08
37357000			2.42E+08	2.26E+08	2.12E+08	1.89E+08	87880000	62783000
			16850000	14296000	18438000			
2.15E+08	2.22E+08	2.35E+08		1.65E+08	1.58E+08	1.19E+08	1.17E+08	1.39E+08

1.73E+08	1.71E+08			65166000	91679000			
					9337100			
	21547000		18091000	55335000	50530000	22349000		
1.1E+08	1.13E+08	29000000	1.33E+08	1.04E+08	1.2E+08	1.32E+08	1.45E+08	1.42E+08
							26783000	
				45854000	30709000			27235000
91707000			37113000	51965000	38464000	46255000		
		1068700	1538600	1639800	1459000			
					26854000	31020000		
13148000	19869000		16001000	21512000	21099000	14720000		
38878000	31431000	36992000	60371000	66932000	72874000	71726000	70224000	53806000
14312000	4921700	10640000	22339000	7006000	9530600	12932000	51675000	12915000
10344000	4570600	6592200	12861000	6398700	5185300	5282500	53810000	6654400
35329000	52235000							
	18681000		23256000	26799000	19485000	43321000	65167000	40013000
89740000	1.34E+08	1.07E+08	96384000	1.85E+08	2.78E+08	82845000	32125000	1.85E+08
62859000	1.07E+08	58834000	1.35E+08		1.67E+08	1.21E+08	93778000	97837000
32795000	20590000	22195000	30800000	19543000	25730000	38178000	1.26E+08	41580000
	17512000		30275000	40516000	60827000	60759000		
2.04E+08	2.45E+08	1.85E+08	1.68E+08	2.05E+08	1.92E+08	2.45E+08	1.53E+08	2.88E+08
	22380000		56312000	57955000	67064000	75144000		63083000
31063000	13863000	19144000	21027000	6828300	12287000	11443000	59724000	12430000
	1.6E+08		1.13E+08					
					2464700		30089000	4741300
11086000	9225700	9219500	8005800	6700700	9778800	10948000	22956000	9272700
32317000	63229000	63761000	93536000	91439000	83888000	78119000	88293000	91903000
	46978000		2.01E+08	1.44E+08	1.51E+08	1.56E+08	46740000	11625000
17796000	17569000	27150000						
1.24E+08	1.48E+08	1.88E+08	60373000	2.05E+08	2.51E+08	84968000	2.66E+08	2.42E+08
1.78E+09	2.89E+09	1.95E+09	4.42E+09	3.55E+09	3.58E+09	5.71E+09	4.78E+09	4.78E+09
1.53E+08	2.4E+08	2.6E+08	90822000	1.28E+08	1.75E+08	2.46E+08	2.57E+08	1.71E+08
						13874000		
3.72E+08	3.47E+08	2.77E+08	4.68E+08	4.17E+08	4.89E+08	8.53E+08	7.43E+08	7.2E+08
73517000	1.01E+08	1.13E+08		30539000	69479000			
				38866000				
1.49E+08	98502000	1.28E+08	1.51E+08	1.6E+09	3.19E+08			18432000
			8530500	3313300		1528500		
		8964500						
						43978000	65700000	25008000
		14660000	22868000	28018000	16697000	20907000		22424000
		7179100		5275700	5502100	4182800	35740000	21751000
2.17E+08	1.28E+08	2.62E+08	1.4E+08	1.63E+08	1.34E+08			
	19174000	20519000		19639000	18664000	22668000	22155000	17673000
			3940000	3207700		3571900	6866800	
2.82E+08	3.02E+08	3.16E+08	2.16E+08	3.27E+08	2.87E+08	3.75E+08	2.28E+08	3.13E+08

			37556000	44196000				
35675000	26029000			30536000	32025000	23376000		
50458000	45174000		35997000	85807000	73631000	69471000		33765000
15032000	19714000	25833000			31635000	17229000	55556000	
3441400	2114900	3068900	4010700		1991400	2185600	7062200	
3.2E+08	2.26E+08	1.9E+08	2.25E+08	1.22E+08	1.46E+08	1.83E+08	1.84E+08	1.87E+08
45168000						46110000		55948000
	60213000		18183000					
1.99E+08	1.26E+08	1.82E+08	1.25E+08	85801000	97488000	82055000	72015000	1.47E+08
2.58E+08	1.78E+08	2.78E+08	1.73E+08	1.28E+08	1.21E+08	2.08E+08	2.16E+08	2.12E+08
	9997000		23951000	29685000	28013000		34422000	16519000
	1.04E+08	93635000		89635000	80411000		64892000	
1.49E+08	1.88E+08	1.51E+08	1.51E+08	1.34E+08	1.25E+08	1.1E+08	1.07E+08	1.29E+08
3429200			5130600	12252000		18943000	35732000	15664000
42587000	38401000	54447000	24875000	64545000	1.41E+08	77123000	1.5E+08	1.46E+08
41965000	39496000	41451000	58258000	47457000	83642000	78553000	94614000	86531000
1.04E+08	1.41E+08	96410000	1.26E+08	1.56E+08	1.15E+08	1.55E+08	1.2E+08	1.15E+08
84399000	1.18E+08	1.07E+08	1.25E+08	1.09E+08	1.17E+08	64720000	47340000	53659000
			3880100				5645400	14821000
	11217000		7046900	74562000	40175000	32626000	1.14E+08	2.24E+08
1.59E+08	3.92E+08	1.28E+08	3.99E+08	5.35E+08	4.88E+08	2.84E+08	1.27E+08	2.54E+08
2.65E+08	3.41E+08	2.92E+08	23824000	3.58E+08	3.28E+08	2.51E+08	1.7E+08	2.54E+08
	58496000		53525000	76438000	84087000		60932000	68499000
25415000			843140			960740	5652400	197690
						12561000		
			1.18E+08	64884000	70301000	45878000		
68953000	88041000	66788000		1.13E+08	1.17E+08	1E+08	1.18E+08	1.51E+08
13320000	789970		1560600	255290	401480	446170	2954100	451270
61297000	61966000	55038000	73603000	1.06E+08	63876000	58439000	29864000	33131000
	99485000	54802000		1.61E+08				
2.72E+08	3.3E+08	2.78E+08	2.7E+08	4.49E+08	3.09E+08		88322000	1.44E+08
			27859000	47217000	18776000			
37421000	32056000	42530000	37976000	26477000	21407000	30859000	23382000	19301000
3.66E+08	2.3E+08	52120000	3.73E+08	2.35E+08	2.17E+08	84969000	58403000	81916000
				1E+08	92492000	72419000		
58335000	1.52E+08	27712000	1.91E+08	1.53E+08	1.53E+08	1.99E+08	1.48E+08	1.87E+08
11961000	5059200	6203700	8603300	5668700	8638000	9727800	22081000	11545000
33127000	44100000	55613000	43973000	52360000	44014000	88369000	39576000	38736000
	8213200	8202200	13662000	8089900	10969000	19850000	16058000	18335000
1.17E+08	1.74E+08	1.65E+08	1.82E+08	1.32E+08	1.36E+08	1.14E+08	1.19E+08	1.35E+08
	9.17E+08		2.64E+08	2.3E+08				
	16487000		69294000	42014000	43638000	47642000	62928000	18034000
2.05E+08	1.62E+08	2.18E+08	2.04E+08	1.46E+08	1.53E+08	1.86E+08	1.66E+08	1.88E+08
6001400	3590800	6089500	10911000	5603200			33404000	
							59935000	
				50122000	22764000			

	74320000		71670000		46067000	1.5E+08	1.37E+08	1.16E+08
61961000	64032000	86883000	77624000	1.66E+08	1.6E+08	1.35E+08	2.54E+08	1.58E+08
		6282600					8899200	
				73672000	59346000		23854000	44873000
				18665000	10640000			
		64219000	60799000	72039000	70553000	1.1E+08	59855000	93533000
	36306000		50844000	83516000	36756000	80538000		
			17045000	4411900	4859500	10128000	7277900	10962000
1.01E+08	80897000		1.29E+08		86143000	1.81E+08	1.77E+08	
					32187000		23983000	26515000
5375200	12426000			5592600		18553000	9088500	13010000
37485000			57063000	84455000	56650000			52131000
			80937000	7804400		9362400		
1.75E+09	1.46E+09	1.78E+09	7.27E+08	8.21E+08	1.11E+09	7.53E+08	7.99E+08	7.18E+08
4.74E+09	7.36E+09	1.37E+09	2.49E+09	5.59E+09	6.48E+09	4.07E+09	2.56E+09	1.94E+09
29533000	59595000	64161000	12555000	17843000	11921000	24260000	24965000	51499000
12407000		14313000	15865000	15427000	17461000	15040000	14756000	14735000
20969000	22067000	15650000	17713000	16678000	17275000	22803000	24335000	31376000
43141000	49327000	43097000	49415000	30877000	30087000	87964000	67694000	72192000
					42663000			31100000
1.86E+08	1.71E+08	2.54E+08	2.16E+08	1.38E+08	1.37E+08	1.7E+08	1.8E+08	1.94E+08
8.41E+08	5.25E+08	5.48E+08	5.27E+08	5.07E+08	4.52E+08	4.67E+08	5.58E+08	4.06E+08
					29054000			
				22145000				
5.57E+08	4.18E+08	7.41E+08	7.01E+08	5.57E+08	4.1E+08	6.99E+08	5.37E+08	5.52E+08
27340000	26982000	24153000	24745000	17742000	14205000			16166000
1.19E+08	1E+08	1.18E+08			29154000			23321000
		38154000			29123000			30712000
1.49E+08	95960000	88725000	91481000	89083000	81777000		1.04E+08	1.17E+08
1.03E+08	1.96E+08	62407000	2.3E+08	3.18E+08	2.18E+08	2.12E+08	1.29E+08	1.8E+08
2.75E+09	3.12E+09	1.94E+09	5.09E+09	3.96E+09	4.27E+09	3.74E+09	3.43E+09	3.17E+09
58963000	78182000	85125000	65575000	46210000	1.04E+08	1.01E+08	1.01E+08	1.01E+08
				34093000	52564000			
	1.06E+08	54986000	41210000	41428000	72549000	39010000	62744000	27963000
			78676000	69374000	74120000			
1.76E+08	1.02E+08	1.46E+08	1.04E+08	66540000	63764000		84786000	82945000
5.01E+08	3.25E+08	5.48E+08	2.15E+08	5.86E+08	2.72E+08	1.29E+08	1.65E+08	2.67E+08
1.14E+08	2.71E+08	1.09E+08		1.51E+08	1.53E+08	1.52E+08	2.08E+08	1.57E+08
	65949000		38960000	70880000	48158000			
2.26E+08	1.86E+08	79196000	1.9E+08	1.57E+08	1.18E+08	1.89E+08	1.72E+08	1.55E+08
44863000	59490000	37682000	43396000	55484000	51057000	32927000	49736000	53943000
				12021000				

29585000	49383000	41794000	35277000	55603000	56741000	51773000	41773000	44638000
	55322000		1.02E+08	1.33E+08	1.14E+08	1.22E+08		59590000
	19461000			20497000	26221000			
2.76E+08		3E+08	2.55E+08				2.21E+08	
44038000	49233000		74833000	52635000	63929000	1.17E+08	73795000	85169000
			66901000	58428000	56653000	64957000	6767700	15158000
3.06E+10	1.76E+10	2.42E+10	1.33E+10	9.96E+09	8.74E+09	7.11E+09	7.56E+09	7.03E+09
1.58E+09	1.1E+09	1.42E+09	1.19E+09	1.1E+09	8.57E+08	1.25E+09	1.16E+09	1.32E+09
7.34E+08	1.12E+09	8.09E+08	5.83E+08	7.22E+08	4.71E+08	3.67E+08	5E+08	5.76E+08
59522000	44604000	55875000	4.89E+08	50149000	52211000	36816000		45376000
1.03E+08	3.36E+08	2.13E+08	1.5E+09	1.57E+09	2.76E+08	2.66E+08		1.38E+09
1E+09	7.28E+08	7.5E+08	1.3E+09	1.22E+09	1.15E+09	13700000	2.95E+09	27034000
84526000	1.08E+08	77998000	73620000	92889000	96159000	44957000	30107000	31122000
6.6E+08	8.92E+08	7.57E+08	8.73E+08	8.24E+08	7.05E+08	8.19E+08	8.03E+08	8.61E+08
1.77E+08	2.07E+08	2.15E+08	1.46E+08	1.47E+08	1.57E+08	2E+08	1.37E+08	1.4E+08
7.63E+08	1E+09	7.96E+08	6.68E+08	1.73E+09	1.22E+09	7.28E+08	5.77E+08	9.26E+08
59485000	82876000		79895000		88890000	76607000	52691000	56321000
			15497000	12999000	16355000	10004000		3291600
4.84E+08	4.71E+08	4.56E+08	4.34E+08	4.17E+08	3.86E+08	4.48E+08	3.65E+08	4.42E+08
	40351000		2.42E+08	3.21E+08	2.57E+08	2.28E+08	91299000	59177000
							33041000	
				23582000				
					4121600		7505100	
88374000	95912000	1.24E+08	1.45E+08	97084000	90736000	93711000	1.22E+08	1.33E+08
32789000			17949000	19994000				
					2160000			
							3854500	
	33617000		29271000	26676000	19630000			18619000
				14876000		7881700		
	22048000							
	34706000	34614000		22749000	25006000	33093000		
	50403000				41059000	1E+08		
7.73E+08	7.6E+08	7.58E+08	5.96E+08	7.36E+08	6.8E+08	7.38E+08	2.85E+08	8.01E+08
2.35E+09	2.83E+09	3.12E+09	2.56E+09	2.29E+09	1.71E+09	3.25E+09	2.79E+09	2.89E+09
6485600				3671600	4049200	7050200	27877000	10484000
1.77E+08	3.06E+08	62817000	2.35E+08	4.85E+08	4.47E+08	4.99E+08	3.34E+08	5.22E+08
				55455000	37534000			
		4270100			17773000			
					8593600	7232000	16000000	14373000
		10905000		15753000		16091000		
4.3E+08	3.73E+08	1.74E+08	8.85E+08					8.49E+08
	14770000		49275000	70930000	61537000	63757000		21242000
22399000	13356000	17897000	16898000	9878100	12113000	29525000	79492000	38156000
19651000	45249000					23389000		

16235000		11818000	61878000	56011000	93482000	1.25E+08	9770300	40108000
36158000	37315000	41874000	27621000	38012000	63437000	55976000	65375000	41166000
29555000		40601000		27402000	32972000			
			3448000	6215700				
57647000	80043000	90846000	1.61E+08	1.1E+08	1.05E+08	1.41E+08	97195000	1.39E+08
		11309000	10228000	7012200	8529900	10874000		13041000
					9732400			
				20551000	26871000			
52737000	75907000	97260000	58636000	1.1E+08	1.56E+08	2.06E+08	24516000	1.44E+08
		22668000	19385000			22883000		
						25273000		
	38993000	29957000		20781000				
	35223000		56760000	70198000	58055000	68612000		
67394000		49814000	66888000	68236000	54700000	22231000	67867000	50835000
5.36E+08				1.21E+08		1.93E+08	1.11E+08	2.05E+08
2.87E+08	1.39E+09	4.53E+08	1.88E+08	7.37E+08	3.87E+08	3.25E+08	3.17E+08	6.76E+08
48738000	54090000	99078000						
						23708000	20745000	
21592000	14800000	12832000	12380000	9731900	13378000			9212400
					21460000			
	2565200		13914000	11985000	14590000			3248100
	89126000		82580000	97850000	88584000	31274000		
40061000	48255000	25092000	58320000	61473000	54252000	58824000	54658000	62474000
	67697000		71621000	64457000		2.72E+08	1.13E+08	2.23E+08
67369000	91827000	80332000	1.13E+08	1.4E+08	1.15E+08	1.25E+08	92882000	1.12E+08
35254000	21941000	32927000						
		4020000		3181200	4740500		7003900	4318600
			8602200		6301300	2680800		
1.12E+09	9.31E+08	9.48E+08	1.02E+09	8.19E+08	8.7E+08	5.36E+08	6.36E+08	6.27E+08
	63313000		40444000					
	20888000	15888000	15928000	36044000	25363000	10843000	18306000	20481000
2.16E+09	3.26E+09	2E+09	3.3E+09	3.15E+09	2.95E+09	2.67E+09	2.42E+09	3.64E+09
15223000	11179000				12475000			
					1592300			35670000
5634700	5394400	7617200	4539800				12994000	4802800
			8704500	15620000	5653700			
	9270200			18463000	18607000	7612500	18099000	24585000
	24089000		21492000	42199000	22779000	24175000	14135000	14491000
3.28E+09	5.6E+09	3.3E+09	4.3E+09	4.73E+09	3.67E+09	3.23E+09	2.2E+09	2.55E+09
1.44E+09	2.12E+08	1.23E+08	1.42E+09	1.92E+09	1.93E+09	9.5E+08	7.19E+08	5.79E+08
				65282000	47572000	30750000	31398000	50769000
	28477000			24975000	24740000	21645000		
48924000	93408000	61680000	70334000	96079000	83693000	33849000	36589000	43221000
99097000	1.25E+08	1.42E+08	46312000	41848000	23425000			
1.04E+08	2.07E+08	39090000	1.16E+08	1.75E+08	1.6E+08	1.02E+08	37834000	74646000
28652000	18107000	24991000	49253000	35687000	54138000	45115000	92598000	75091000

5.59E+08	4.2E+08	5.74E+08	2.74E+08	3.09E+08	2.39E+08	1.98E+08	1.93E+08	2.2E+08
91418000	1.71E+08	87595000	96887000	1.21E+08	1.6E+08	1.99E+08	1.86E+08	1.19E+08
77626000	52233000	85294000	4.22E+08	2.7E+08	2.62E+08	2.77E+08	1.83E+08	1.97E+08
			33161000		21828000			
1.42E+08	1E+08							
2.95E+08	3.78E+08		5.73E+08	5.59E+08	5.17E+08	5.51E+08	2.29E+08	2.79E+08
69072000	70694000	83139000	72747000	56064000	71985000	80945000	91250000	81601000
68543000	36176000	52976000	1.23E+08	96436000			6.12E+08	2.14E+08
	26184000	39478000			16702000			16924000
				23215000				
86524000				52043000		20774000		6345800
			6008700	8357900	27384000	25425000	9189400	
	16000000		16924000	22340000	19670000	19342000		20293000
				47583000	65768000	30625000		
					23525000		6040200	
				6983300			40722000	27551000
1.15E+09	1.1E+09	1.41E+09	1.71E+09	1.39E+09	1.29E+09	1.33E+09	1.83E+09	1.5E+09
56433000	1.46E+08	35020000	2.17E+08	3.25E+08	3.16E+08	1.81E+08	98017000	1.57E+08
7549800		21516000	4428500	12646000	11938000	7732200	34722000	28412000
	25129000			18372000	17656000		25658000	
							27012000	31454000
19813000	60354000	47530000	1.18E+08	1.02E+08	1.42E+08	1.37E+08	1.4E+08	95690000
14583000	18022000	12922000	22068000		34349000	38635000	11904000	36937000
23713000	58610000		35966000	32301000	39171000	42856000	56593000	70142000
25726000	13450000	12569000	16928000	13049000	20866000	12223000	44927000	14846000
		28658000						
1.59E+08	1.99E+08	1.69E+08	1.39E+08	1.64E+08	1.45E+08	1.95E+08	1.52E+08	1.53E+08
6.56E+08	5.12E+08	5.62E+08	5.13E+08	4.16E+08	3.89E+08	6.2E+08	7.08E+08	6.66E+08
1.15E+08	1.68E+08	2.97E+08	1.42E+08	2.09E+08	1.64E+08	79159000		1.41E+08
41503000	75899000	43871000	39863000	30026000	42108000	21861000		
7.91E+08	1.01E+09	1.42E+09	68316000		1.41E+08	3.92E+08	1.08E+09	2.62E+08
3.85E+08	2.34E+08	2.78E+08	2.48E+08	3.2E+08	2.05E+08	35062000		
45121000	64145000	57870000	66919000	71391000	83546000	78214000	67755000	60768000
39118000	47560000	51617000	89283000	89528000	1.29E+08	1.03E+08	74679000	1.01E+08
			6603000		6328600	4987300		
				7250700				
	4.57E+08	3.74E+08	3.38E+08	2.43E+08	2.84E+08	2.83E+08	1.55E+08	1.96E+08
1.01E+09	8.97E+08	1.52E+09	7.46E+08	8.05E+08	6.83E+08	2.8E+08	2.94E+08	4.03E+08
37993000	32488000	45733000	73241000	90280000	79552000	92097000	93064000	99023000
		66288000	49926000	46917000	36605000			
49638000	29358000		10351000					
22094000		28861000	10923000	7683300	12042000			
	84691000		59906000	60368000	59887000	67568000	49907000	65456000

1.27E+08	1.43E+08	1.01E+08		80856000	94190000	64272000	80894000	98629000
	16274000							
40955000	1.56E+08	60304000	2.84E+08	2.68E+08	85936000	3.23E+08	2.71E+08	3.23E+08
			1230000				2371600	829830
88983000	61393000	75238000	43064000	55229000	39163000	40159000		32715000
19737000	2695600	4416000	3424000	1389200	2286300	1918800	13427000	2051600
4.78E+09	5.33E+09	5.66E+08	8.81E+09	9.12E+09	7.9E+09	3.43E+09	5.06E+08	9.03E+08
	52006000	94862000	60206000	42259000	37577000	58713000	34542000	47985000
37084000	41718000	37287000	35860000	44586000	35085000	15929000	22537000	27917000
10036000	27708000	9621200	15256000	25008000	14675000			
				36552000	32888000			
5.12E+09	1.18E+10	3.59E+09	5.7E+09	1.03E+10	7.67E+09	5.87E+09	4.39E+09	4.68E+09
44677000	41803000			11848000		1.19E+09		24080000
2.43E+08	2.44E+08	1.23E+08	63820000	1.01E+08	1.34E+08	1.65E+08	1.14E+08	88845000
92376000	94080000	88962000	1.51E+08	1.13E+08	1.03E+08	1.5E+08	1.57E+08	1.51E+08
9.88E+08	1.04E+09	9.66E+08	7.3E+08	7.45E+08	7.23E+08	7.32E+08	8.44E+08	8.87E+08
3.79E+08	3.4E+08	4.21E+08	1.79E+08	1.6E+08	1.41E+08		1.36E+08	1.15E+08
	22077000		33172000	67487000	60735000	54023000		37425000
6.39E+09	9.33E+09	6.69E+09	6.15E+09	5.74E+09	5.8E+09	1.08E+09	7.12E+08	3.12E+09
35600000	50609000		46456000	59449000	52480000	58228000	31876000	31007000
			1.71E+08	1.53E+08	1.75E+08	2.64E+08		2.62E+08
				7823500		8005800	11817000	11643000
27695000							22711000	
1.21E+08	90312000	1.69E+08	1.65E+08	63749000	71009000	1.44E+08	4473600	1.02E+08
2.09E+09	1.97E+09	2E+09	1.47E+09	9.54E+08	1.05E+09	1.67E+09	1.48E+09	9.83E+08
59953000	17216000		1.24E+08	1.4E+08	99234000	1.52E+08	1.07E+08	1.43E+08
	20094000				17265000			17226000
		30548000					53945000	
67558000	1.17E+08		1.07E+08	1.7E+08	3.35E+08	1.85E+08	1.31E+08	1.65E+08
			22724000	20954000	21518000	18709000	20388000	14674000
32345000	35425000	25678000	11505000	16628000	30691000	46927000	39516000	22974000
				3165700	4696500	5640400	6978500	6308100
	23492000		11721000	9365600			11612000	9273500
7942100	9378600	15728000	14309000	10064000	10736000	16796000	22150000	14782000
	17175000	11702000	40625000	48201000	60799000	1.03E+08	96022000	51799000
51507000	40861000		87537000	62970000	64777000	1.09E+08	1.22E+08	1.09E+08
34307000	71937000	39350000	46425000	74713000	57631000	62455000	40115000	46666000
52969000	56597000	37676000	29859000	43050000	41158000	57371000	40590000	
2.13E+08	1.83E+08	2.23E+08	1.44E+08	1.15E+08	1.08E+08	61932000	77850000	81383000
						11922000	15099000	
					12976000			
			39714000		18069000		26576000	24849000
54694000	1.51E+08	89079000	87680000	1.69E+08	1.49E+08	1.29E+08	1.22E+08	1.05E+08
1.11E+09	9.45E+08	1.09E+09	7.36E+08	8.48E+08	6.55E+08	4.48E+08	4.58E+08	4.33E+08
				17274000	11973000		11204000	
	36029000			49818000	63949000	71819000	58361000	68531000

42028000	24091000		33658000			33674000		40592000
97473000	81638000	80042000	35451000		32331000			
		15335000	16531000	13871000	12221000	18416000	21198000	22349000
			15069000	21639000	21368000		31393000	23499000
		23332000	18225000	37319000	58530000	33155000	23196000	70278000
35902000			3310400	2095600	2458000	4727800		
2.01E+09	3.47E+09	3.14E+09	5.43E+09	2.65E+09	3.45E+09	5.23E+09	5.38E+09	4.5E+09
17557000				26987000		34024000		
6.38E+08	4.15E+08	4.89E+08	6.18E+08	23693000	27708000	6.91E+08	6.9E+08	7.23E+08
2.76E+09	1.71E+09	2.58E+09	2.16E+09	1.35E+09	1.21E+09	1.49E+09	1.58E+09	1.48E+09
		2819800	4474100	1890600	2796300		8121000	3445500
			1.37E+08	27375000	87628000	44824000		
42371000	43678000	50043000	1.94E+08	1.6E+08	1.47E+08	1.61E+08	2.08E+08	1.35E+08
1.65E+08	3.61E+08	1.26E+08	5.34E+08	2.7E+08	2.83E+08	3.97E+08	2.92E+08	3.06E+08
1.09E+08	8284800	53164000	1.95E+08	11467000		4628700	9313400	12109000
8959000			32957000	24054000	32908000		69129000	41513000
	480500						4762400	912030
2.44E+08	3.12E+08	2.74E+08	1.98E+08	2.23E+08	3.1E+08	24359000	2.86E+08	2.43E+08
							11466000	
						35949000	26929000	
	1.7E+08	1.57E+08	1.34E+08	74377000	64237000			1.19E+08
	13752000					13661000		
				20580000	14058000		14430000	
1.26E+08	1.06E+08	2.28E+08	3.95E+08	2.56E+08	4.98E+08	4.06E+08	1.02E+09	5.57E+08
			7986800	6983900	23036000	5142500	2359300	
			1.35E+08	1.45E+08	1.33E+08	25430000		16455000
	52879000	70571000		40735000	42564000			
							1.92E+08	
3400600	803090	2806700	2969100			1408300	10679000	2311700
		41009000					31026000	
54378000	2.06E+08	1.02E+08	3.01E+08	8.06E+08	7.47E+08	3.81E+08	4.15E+08	4.54E+08
1.02E+08	1.32E+08	1.02E+08	1.27E+08	1.64E+08	1.47E+08	1.55E+08	1.62E+08	1.76E+08
1.03E+08	96733000	95076000	1.25E+08	1.95E+08	1.35E+08	26370000	56995000	74460000
11960000	25321000		1E+08	68673000	61295000	82909000	60897000	52776000
18752000	15520000	20677000	17883000		24208000	23715000	1.45E+08	28244000
	17696000	9405000	16711000	42972000	13062000	22999000	1.24E+08	55525000
				1.26E+08		77143000	79094000	82826000
3.24E+08	3.61E+08	4.45E+08	3.86E+08	2.98E+08	2.93E+08	4.44E+08	4.05E+08	4.64E+08
19018000	13571000	15442000	12920000	10427000	13779000	10125000	25486000	10008000
				20327000		13812000		
	9104200		19661000	20467000	12138000	11585000		11148000
22000000	56258000	46212000	1.14E+08	63999000	63819000	92350000	1.08E+08	1.02E+08
				3300400				

13964000	10951000	9775400	19972000	18518000	18329000	29314000	21657000	28268000
	13275000		17451000	56021000	30701000	50513000	15689000	35863000
	22302000	45424000		27541000			21031000	20753000
4472400	9840000		6996100	4025200	3469800			
				14222000				
1.03E+08	76093000	1.29E+08	1.92E+08	1.28E+08	2.55E+08	1.56E+08	6.07E+08	2.58E+08
23717000	33524000		3.3E+08	2.5E+08	2.1E+08	61490000	25163000	26036000
					18118000			20606000
99778000	1.06E+08	1.41E+08	1.58E+08	1.5E+08	3.44E+08	6.48E+08	1.62E+09	4.68E+08
81750000	86600000	55145000	1.69E+08	1.24E+08	1.75E+08	52998000		52115000
16688000	12876000	17305000	24073000	16420000	20801000	15477000		22866000
			4992500					
1.06E+08	63788000		85574000	59028000	73971000		1.27E+08	1.04E+08
53048000	42608000	58870000	45804000	33669000		82215000	48823000	37669000
1051300			540800			427580	1080000	333860
	87435000	72614000	1.15E+08	1.23E+08	1.2E+08	1.16E+08	76954000	97485000
96177000	1.35E+08	90708000	81914000	98420000	1.05E+08	1.46E+08	1.45E+08	1.79E+08
			1.76E+08	1.57E+08	1.22E+08	1.53E+08	69181000	1.12E+08
1.69E+08	1.82E+08	1.46E+08	1.59E+08	2.19E+08	1.81E+08	1.03E+08	81824000	87275000
				38767000	47488000			
33487000	36464000	4337400	1.24E+08	66485000	62488000	89860000	1.12E+08	93647000
2.55E+09	1.84E+09	2.38E+09	2.14E+09	1.61E+09	1.44E+09	2.41E+09	2.85E+08	2.29E+09
		2196300	2861200	1631000	2327600	2640200	7887300	3160500
			42712000	65339000	55974000	57397000	32318000	29457000
5.17E+08	9.02E+08	7.79E+08	4.7E+08	5E+08	2.6E+08	4.37E+08	3.42E+08	5.04E+08
29844000	51092000		35205000	2.16E+08	2.69E+08	1.72E+08	43370000	93353000
			1E+08	1.69E+08	2.53E+08	49614000		29863000
	18286000		21079000					
			14822000	35018000	22442000	15378000	12824000	26266000
	21715000		23485000	30567000	24370000	24415000	38541000	42438000
					12675000	23370000		16185000
90378000	1.17E+08		98479000	1.02E+08	95093000	91790000	79254000	1.22E+08
					7885800			
26446000	52465000	40914000	50370000	59904000	63560000	37583000	36116000	42703000
	8124000							
1.47E+08	1.41E+08	1.57E+08	1.86E+08	2.03E+08	1.48E+08	2.15E+08	1.54E+08	1.66E+08
1.37E+08	1.99E+08	1.64E+08	2.77E+08	1.9E+08	1.87E+08	4.47E+08	4.26E+08	4E+08
5.27E+08	5.7E+08	4.08E+08	5.44E+08	8.12E+08	9.69E+08	1.22E+09	1.04E+09	9.51E+08
2.61E+08	2.86E+08	2.44E+08	3.93E+08	5.41E+08	4.5E+08	2.95E+08	1.84E+08	2.96E+08
				9557800				5889900
	23864000	16999000	25876000	46244000	38518000	30834000		24982000
				4636100	6987900	3717900	6226800	6168000

	14919000			26782000	35288000	20876000		
				7290800		5786700		
94138000	1.31E+08	1.37E+08	1.62E+08	1.39E+08	1.31E+08	5206700	1.48E+08	
			65465000					98397000
97936000	1.77E+08		3.75E+08	2.63E+08	2.25E+08	4.26E+08	3.2E+08	4.1E+08
								6413200
					3354400			
			34648000					
			11888000	26001000	17562000	23339000	16790000	
	43030000			46009000	44990000	37976000		45441000
3.25E+09	3.07E+09	4.23E+09	3.72E+09	3.35E+09	2.61E+09	3.74E+09	3.6E+09	3.68E+09
	35315000	28674000		31792000	31635000	31689000		29614000
3.23E+08	60974000	2.91E+08	5.5E+08	86779000	96794000	7.29E+08	8.46E+08	9.74E+08
			1762000	24799000	13687000	10427000	11396000	21925000
			35333000	41263000	45171000	44815000	35140000	46657000
	42744000			89790000	98548000	40083000	46675000	65043000
				5437100			92739000	
69632000	77090000	55475000	54613000	83038000	61467000	44343000	29038000	45021000
				7019100				
			16183000	18514000	14689000			
				19960000	25100000			
33690000		35604000	24333000	25212000	21327000	17559000		26504000
4.21E+08	4.27E+08	5.01E+08	1.27E+08	83753000	1.75E+08	1.95E+08	2.31E+08	1.78E+08
48588000	51592000	55274000	86771000	51256000	31241000	23334000	77940000	
4.98E+08	5.24E+08	4.38E+08	4.2E+08	3.59E+08	3.14E+08	4.14E+08	4.07E+08	4.62E+08
1.18E+08	70932000	1.21E+08	80655000	49705000	44941000	68651000	70303000	64758000
28480000	60736000	57465000	27696000	74949000	62122000	18813000	31493000	30774000
14258000	11773000	13389000	9702300	8686500	14229000	14640000	20953000	16339000
11546000	32494000	80682000	14834000	23286000	1.22E+08	17817000	53833000	82474000
88599000	87061000	1.34E+08	63464000	47058000	48295000	56031000	61850000	75131000
			2024200				18095000	
	27839000		22754000	19169000	20002000	24786000		21942000
	1.04E+08							
			26547000	32813000				37310000
								16033000
27701000	13962000	19323000	34875000	27974000	56978000	55434000	1.68E+08	67072000
33268000				68693000	55263000	48250000		
25492000	37441000	29515000	45733000	26920000	32005000	60782000	33251000	61109000
30410000	35350000	28247000			19368000	7722600	30661000	
			49608000					
55324000	60511000	44222000	14040000	11377000	18289000	14822000		
			28645000	44338000	44685000	59136000	67898000	55007000
			99382000	2.33E+08	1.54E+08			

22783000			95151000	59730000	64111000	27207000		
3.56E+08	8.68E+08	3.7E+08	3.11E+08	3.87E+08	4.14E+08	1.8E+08		1.36E+08
			4219400	8495400	15982000	9248200	18160000	22569000
	5956100							
			17260000	23528000	17332000	20205000	14022000	22648000
23849000	18118000	20422000	15858000	23280000	19722000	23876000		
1.05E+08	1.35E+08	1.03E+08	1.79E+08	2.55E+08	2.58E+08	3.31E+08	1.42E+08	2.57E+08
1.82E+08	1.14E+08	1.57E+08			88118000	1.39E+08		
48196000	18458000	36580000	51351000	62780000	43718000	43984000	60553000	40692000
4274800					7302700			5464500
			23109000	41337000	28040000	21535000	16425000	24739000
1.55E+08	1.14E+08	1.23E+08	1.23E+08	1.76E+08	1.88E+08	1.45E+08	1.56E+08	1.31E+08
	41567000	35436000	39504000	35235000	39822000	43043000		43980000
14828000	54931000			58982000		36953000	15604000	
9.78E+08	1.23E+09	1.01E+09	4.75E+08	5.28E+08	8.15E+08	9.96E+08	8.14E+08	6.42E+08
	1.22E+08	83544000	1.08E+08	1E+08	1.08E+08	94959000	78361000	69734000
4062200	1812500				2276000	3422700	11993000	
			77678000	1.11E+08		88374000		
			45146000	59641000	52581000			
17619000			9057300					4925100
			28574000	21194000	23457000	26778000		15121000
45761000	75417000	56295000	47949000	66692000	50859000	21540000	33384000	36617000
27684000	32847000	39375000		52083000	27393000	10849000	29146000	20927000
	17273000		16231000	20195000	25937000			
				13201000				
		9025700			18973000	12929000	15976000	
	20621000	16607000			18644000			14869000
	61683000	39710000				59706000	54309000	55713000
4.39E+08	3.87E+08	4.27E+08	3.75E+08	2.9E+08	2.9E+08	3.93E+08	4.04E+08	3.84E+08
						23976000		
38956000	37531000	53300000	48883000	40327000	42287000	42651000	48673000	44095000
83187000	97464000	71401000	82306000	70547000	59691000	53693000	50360000	53113000
8.91E+08	8.62E+08	5.79E+08	4.58E+08	4.74E+08	4.44E+08	3.7E+08	4.74E+08	3.86E+08
5.59E+08	1.12E+09	3.86E+08	1.19E+09	1.53E+09	1.48E+09	9.62E+08	6.77E+08	1.53E+09
	20660000	15489000	1.09E+08	1.64E+08	92218000	1.11E+08	67168000	46050000
	19801000							
				13853000				10748000
9.19E+09	6.54E+09	9.95E+09	1.07E+10	5.06E+09	5.72E+09	9E+09	7.75E+09	7.27E+09
55799000	82929000	52276000	75520000	75168000	76841000	74398000	1.08E+08	1.29E+08
	22683000		19344000	20483000	22488000	36154000	28565000	29709000
	23760000	11154000	14106000	25083000		16323000	27350000	27657000
23845000	35613000	28322000	1.23E+08	1.32E+08	1.09E+08	91123000	82300000	81585000
	15953000				10848000		12091000	
10077000		22252000	17903000	16782000	14189000	18365000	18335000	16057000

22321000	46189000	25208000	34277000	45731000	38283000	33167000	30033000	35613000
2.16E+08	2.32E+08	2.05E+08	5.2E+08	6.94E+08	4.73E+08	1.43E+08	1.02E+08	1.93E+08
91875000	1.8E+08	1.26E+08	1.64E+08	1.85E+08	2.06E+08	2.15E+08	2.45E+08	1.83E+08
64314000	1.21E+08	1.12E+08	1.38E+08	1.12E+08	89404000	87926000	74769000	1.13E+08
	16494000		5653500	35653000	43849000	24253000	18225000	52430000
			1.45E+08		94851000	1.83E+08	1.29E+08	1.53E+08
1.55E+08	7.12E+08		4.73E+08	9.08E+08	7.31E+08	4.47E+08	3.59E+08	4.69E+08
			68956000			61852000		
33035000	51428000	42551000	46664000		64197000	58912000	49393000	57102000
2.78E+09	2E+09	2.76E+09	1.34E+09	1.03E+09	9.01E+08	7.81E+08	5.97E+08	6.74E+08
	50857000	36852000						
					52581000		2.53E+08	
	2030100	2994700	1210600	1445600	2169300			
			11424000		17633000		21815000	19837000
				26419000		31300000	41271000	32179000
			2126500	333930	654650	1596900	8541600	2087200
			7235800	1521000	3955700	2727400	2754200	2260300
			1.53E+08		85165000	57120000		40819000
			25787000		17848000			
1.83E+09	1.36E+09	1.4E+09	2.44E+09	1.75E+09	1.78E+09	2.27E+09	2.32E+09	2.48E+09
11595000	10794000	14249000	5769600		3900600	6716000	18935000	
			41701000	51817000	21955000	55376000		
	27979000	28701000	24632000	19202000	19847000	25458000	26340000	26058000
1.7E+08	1.44E+08	2.48E+08	1.67E+08	1.47E+08	1.27E+08	1.48E+08		1.62E+08
		13204000	16247000	16008000	14469000		12800000	13608000
			32786000	26976000	30330000	16916000	28538000	44473000
							29919000	
1.2E+08	1.06E+08	1.44E+08	1.27E+08	98750000	87536000	1.12E+08	97214000	1.1E+08
			36285000	33226000	23067000	47763000	45668000	47917000
10982000		29059000		8813500				
50752000	45583000	71498000	63899000	49521000	67590000	62900000	1.22E+08	92963000
	12159000		18852000					34006000
	32377000		46016000	25936000	29840000	32238000	30408000	30771000
			12030000					
	29481000	26861000	60624000	40542000	31970000	20985000	16421000	26757000
			48239000	49235000	53747000	16631000		38228000
1.35E+08	1.9E+08	1.78E+08	2.45E+08	3.38E+08	2.76E+08	3.05E+08	2.9E+08	
16742000	20844000	45550000	28216000	26734000	20704000	45393000	24770000	47146000
								9867900
				11437000		9567300		
							9168700	26362000
	1.05E+08	1.04E+08	1.18E+08		1.71E+08			86197000
72203000	1.84E+08	31240000	2.65E+08	2.2E+08	1.75E+08	2.61E+08	2.19E+08	2.35E+08

95544000	1.13E+08	1.09E+08	1.13E+08	1.1E+08	1.13E+08	1.24E+08	1.67E+08	1.3E+08
24595000	62720000		55413000	1.21E+08	1.1E+08	44582000	78329000	83457000
			26188000	24278000	18436000	22381000	24732000	21498000
		39657000						
				26341000	18855000			10352000
4.77E+08	5.98E+08	4.61E+08	4.05E+08	3.86E+08	3.84E+08	4.21E+08	3.77E+08	4.23E+08
8.62E+08	9.7E+08	9.49E+08	1.04E+09	1.12E+09	1.04E+09	1.18E+09	1.35E+09	1.19E+09
57835000	54724000	85852000	10444000	31399000	42291000	41388000	64843000	28102000
14271000	15014000	17100000	3523900					
			12954000	12139000	12750000	15998000	13177000	16864000
47922000	32756000	38066000	35430000	36862000	47258000	79470000	61431000	65634000
						50983000	41899000	
11845000	18740000	17515000	15742000	17204000	19692000			
			47321000	70609000	63105000			23687000
3.11E+08	2.85E+08	2.57E+08	2.57E+08	2.37E+08	2E+08	2.28E+08	2.34E+08	2.62E+08
60798000	62409000	1.82E+08	1.88E+08	84423000	1.48E+08	2.1E+08	2.2E+08	2.72E+08
		31536000						
3.8E+08	2.45E+08	3.45E+08	3.25E+08	2.47E+08	2.54E+08	3.39E+08	3.59E+08	3.19E+08
36140000	33742000	38911000	25704000	53861000	71437000	34437000	48805000	62318000
				20566000	14534000			
1.1E+08	1.49E+08	1.57E+08	2.42E+08	2.4E+08	2.76E+08	2.24E+08	2.6E+08	3.15E+08
						34145000	32256000	
	12024000			18569000	17203000			
				26218000	31665000			
96877000	81153000	1.09E+08		51803000	48445000	31172000	40727000	39341000
	12441000							
87366000	83738000	82624000	80548000	76995000	86723000	52101000	95211000	74427000
			16337000	61033000	75249000			
			4780200	10986000	11071000	5024200		6621300
	44351000	79539000	20631000	8391500	31429000	56148000	17243000	35881000
1.35E+09	1.47E+09	1.35E+09	1.02E+09	8.35E+08	6.43E+08	7.3E+08	6.3E+08	6.09E+08
3.26E+08	2.7E+08	3.76E+08	4.99E+08	4.04E+08	2.82E+08	2.46E+08	3.36E+08	3.77E+08
77571000	68586000	69856000	57248000	40721000	38001000	45988000	44890000	46487000
37783000	1.55E+08	31017000	1.08E+08	98597000	1.63E+08	34309000	70266000	1.21E+08
4792200		7265400	13354000			5377600	8753900	
3.27E+08	1.75E+08	2.25E+08	2.43E+08	1.69E+08	1.44E+08	2.35E+08	2.18E+08	2.04E+08
2.31E+08	3.3E+08	3.34E+08	1.96E+08	1.66E+08	2.96E+08	2.29E+08	3.47E+08	3.47E+08
			3223400		3275800	4651200	7040900	5829400
	1.54E+08	1.35E+08	1.04E+08	1.18E+08	1.05E+08	1.21E+08	1.24E+08	1.55E+08
1.15E+08	3.87E+08		3.79E+08	6.21E+08	6.58E+08	5.19E+08	99655000	2.9E+08
	3.23E+08	10063000	4.48E+08		2.39E+08	4.34E+08	2.59E+08	3.92E+08
94748000	1.04E+08		46080000	45988000	54056000	68849000	53907000	59210000
	13925000	12978000	6079100	10216000				13090000
						1.01E+08	1.03E+08	1.08E+08
				13025000	12482000			
				6336900				

				16436000				
7.58E+08	6.04E+08	8.99E+08	3.81E+08	3.64E+08	4.02E+08	7.28E+08	5.76E+08	4.49E+08
28073000	24205000	27934000			17754000	26372000	29256000	28846000
	1.02E+08		42393000	1.8E+08	1.72E+08	79725000	15665000	73497000
3.22E+08	3.86E+08	5.2E+08	8.15E+08	9.21E+08	7.32E+08	7.41E+08	1E+09	9.34E+08
14478000	1.87E+08	34276000	77320000	2.13E+08	2.06E+08	1.06E+08	1.43E+08	2.85E+08
3.2E+08	3.17E+08	4.01E+08	3.22E+08	2.44E+08	2.62E+08	3.29E+08	2.98E+08	2.81E+08
46916000	48725000	67582000	96301000	73809000	69250000	97090000	84224000	95618000
83241000	87129000	1.44E+08	1.7E+08	1.45E+08	1.48E+08	1.46E+08	1.26E+08	1.44E+08
	5962700		8362100	7301800				
	65656000	54970000	95370000	1.04E+08	83458000	96962000	1.01E+08	98586000
			9369500	22890000	18445000	14689000	17058000	19034000
64639000	64235000	74198000	96420000	65554000	65332000	71996000	62523000	70641000
			10989000	9533000	13976000	18464000	14979000	13300000
34586000	42715000	48031000	78159000		35735000		42193000	
1.7E+08	1.28E+08	1.42E+08	1.4E+08	1.1E+08	1.01E+08	1.15E+08	1.2E+08	1.37E+08
			23167000	30762000		22069000	29392000	27290000
			19681000	54866000	42831000	27535000	19832000	
1.56E+08	1.25E+08	1.6E+08	69688000	1.84E+08	1.07E+08			
1.02E+08	1.22E+08	1.58E+08	1.82E+08	1.6E+08	1.36E+08	2.03E+08	1.78E+08	1.99E+08
		32805000						
	4377200		61000000	45851000	50276000	32217000	29397000	21632000
1.38E+08	66244000	97539000	1.8E+08	50112000	74151000	1.29E+08	1.21E+08	1.02E+08
44596000						1.01E+08		
			17703000			14077000	12671000	13717000
3.57E+08	2.84E+08	3.54E+08	2.78E+08	1.77E+08	1.82E+08	3.79E+08	2.86E+08	3.85E+08
9.97E+08	8.98E+08	1.42E+09	1.62E+09	1.19E+09	1.16E+09	1.55E+09	1.42E+09	1.54E+09
	82082000		1.23E+08	1.04E+08	1.53E+08	1.17E+08	91391000	85227000
			15418000		19637000			
21476000		19250000	36942000	76279000	53295000	33184000	40957000	49108000
35253000	34802000	38073000		31562000	22433000	22048000	20365000	24662000
93976000		1.54E+08	54346000	42049000	34044000	50774000		48661000
	91449000		95596000	90115000	80413000	1.08E+08	95401000	91416000
33589000			57739000	42339000	44008000	55841000	45776000	42479000
	21049000		23045000		15073000			24189000
	1.13E+08		30651000			2E+08		1.05E+08
1.17E+08	73357000	1.17E+08	1.38E+08	84116000	65654000	1.07E+08	1.06E+08	1.07E+08
1.51E+08	7.54E+08	1.94E+08	1.38E+09	1.18E+09	9.98E+08	1.11E+09	1.11E+08	2.18E+08
45420000	1.19E+08		3.15E+08	2.92E+08	2.61E+08	2.56E+08	1.56E+08	1.48E+08
40628000			21447000	22784000	38449000	24118000	61357000	60709000
				17946000	16436000	9741900		
86649000	86541000	1.15E+08	62968000	42544000	50448000	64408000	50955000	51175000
1.2E+08	1.26E+08	1.28E+08	1.9E+08	1.7E+08	1.44E+08	2.33E+08	2.55E+08	2.23E+08
10847000	42363000	22459000	40903000	34546000	47351000	26980000	42988000	68367000
1.04E+08	52525000	83178000	1.01E+08	72517000	64290000			
17949000	35150000	28759000	20577000	30883000		24242000		16495000

		22556000	20873000	19889000	13691000	27439000	24016000	33881000
								41934000
	1.18E+08		64329000	82640000	84514000	71268000	78931000	65073000
6.21E+08	1.46E+09	4.69E+08	1.17E+09	8.03E+08	1.52E+09	2.12E+09	1.46E+09	1.08E+09
			10972000		31015000	49785000	59344000	33082000
				2831900	6486600	10344000	24392000	25063000
93393000	76991000		48688000			1.43E+08	1.26E+08	90259000
24137000	26771000	21794000		26544000	30221000	4772700	34917000	40326000
			22253000		20343000	52099000	62247000	51225000
					16896000	14846000		
27938000		21704000	19864000	58402000	28891000			
7.71E+08	8.93E+08	7.35E+08	4.69E+08	6.58E+08	9.47E+08	1.02E+09	9.01E+08	6.41E+08
	8533500	13003000			4642200	10559000	82920000	4249200
			41535000	78136000	1.35E+08	61290000		
	16482000	11860000	8480800	11237000	20515000	15761000	23734000	
9056800			17438000				12569000	14524000
	38841000	20985000	8660800	11392000	43555000	3298200	36839000	68678000
1.25E+08	1.13E+08	1.33E+08	83736000	74857000	75586000	57796000	53627000	54738000
1.37E+09	1.45E+09	1.43E+09	1.68E+09	9.94E+08	1.19E+09	1.63E+08	1.42E+09	1.7E+09
			26467000	21784000	23630000	28032000	24235000	29707000
	29706000			33908000	1.23E+08		50018000	47815000
		6245000	12615000			29773000		
3.05E+08	3.11E+08	2.94E+08	2.86E+08	2.58E+08	2.52E+08	3.71E+08	4.54E+08	4.33E+08
70370000	56489000	48402000	74569000	49366000	87262000	45312000	76004000	46743000
	30485000		34671000	41849000	36970000	32332000		18400000
1.43E+08	1.15E+08	1.44E+08	1.92E+08	1.55E+08	1.37E+08	1.6E+08	1.28E+08	1.7E+08
22872000		22507000	31161000	22306000	14797000	15406000	20535000	23255000
65959000	82885000	72203000	1.11E+08	78401000	91397000	80422000	89238000	70151000
		10279000	46795000	43342000	47494000	20279000	25706000	26394000
	25802000	51030000		28806000	26794000	30431000	50753000	55945000
			22850000					
	1.66E+08		50448000	1.31E+08	1.54E+08	2.61E+08	1.8E+08	1.37E+08
							8890600	
				2.88E+08	2.49E+08			3.68E+08
	40781000		13302000	68313000	59120000	66578000		
	27291000			29647000	25492000	39191000	26231000	
36291000	36835000		37467000	34915000	38034000	40893000	45778000	63352000
			47408000	20648000	13610000			
39206000	37051000	36847000	31762000	26652000	32808000	54672000	45441000	55975000
6.55E+08	7.21E+08	7.41E+08	1.38E+09	2.31E+09	1.87E+09	9.22E+08	7.87E+08	1.09E+09
	10315000	12584000	31504000	14358000	26221000	17598000	17525000	34483000

				18419000	25602000			
	45481000							
				33493000				
			13088000	31779000	11643000	34770000		
22317000	27634000		34303000	36969000	29218000	16938000	24514000	26221000
			7087000	5004200	5818100			
	6983400	10635000			7383100		23560000	
55393000						1.12E+08		
								38529000
			580920				1552800	
				29720000	12791000			
					7533100			9431500
			46434000	22573000	23389000	28553000	17053000	14802000
							20029000	
				23619000	23627000	29402000	27574000	
1.44E+08	2.2E+08	1.37E+08	52617000	91341000	1.18E+08	1.39E+08	96724000	72951000
	16146000	7828100	6469900					
2.14E+08	3.74E+08	2.07E+08	1.2E+08	2.08E+08	3.67E+08	2.85E+08	3.58E+08	2.85E+08
20401000	27472000	21246000	47914000	28240000	30634000	51888000	57393000	45541000
4.61E+08	5.31E+08	5.56E+08	5.05E+08	4.18E+08	4.21E+08	5.5E+08	4.93E+08	5.52E+08
					14122000			
1.09E+09	1.41E+09	1.16E+09	1.78E+09	1.65E+09	1.77E+09	2.1E+09	1.59E+09	1.76E+09
				8195700				
42386000		44001000			82089000	91595000	75286000	
6728100	1613000	1747700	1989800	1020800	1422700	888480	4256000	1107600
		10061000						
			22479000	30643000	32598000	41900000		
19861000		17994000		20148000	9429300			12746000
				16235000				
						31401000		
70197000	79310000	79036000	44307000	47101000	82455000	99004000	78817000	64164000
					6478500			
				8517100	5261200			
							17344000	
75704000	1.15E+08	80609000	1.16E+08	1.72E+08	1.47E+08	1.44E+08	1.01E+08	1.04E+08
			46664000	74517000	46569000	81878000		
			6366300		5889600			
					14705000			
	22019000			13263000	17855000			
				45942000				
1.28E+08	6.29E+08	1.82E+08	2.61E+08	5E+08	5.66E+08	2.41E+08	1.64E+08	1.88E+08
					21664000			
25634000		37986000	29917000		20542000			30019000
1.22E+08	1.21E+08	2.02E+08	1.67E+08	1.31E+08	1.5E+08	1.5E+08	1.37E+08	1.74E+08
					5417900	14402000	8227100	5973300

			12077000	7893500	11393000			
12843000		20721000	44639000	28381000	28562000	60162000	35227000	45304000
	75171000	33542000	92418000	80162000	1.03E+08	1.11E+08	94559000	1E+08
	1496000	2893700	4595600	5365200	5430000	3702300		
	6818500	9974700			2387400	3925400		3512400
4099500	2974800		9043500	1606300	2574400	5729700	18552000	9851200
1.2E+08	1.6E+08	54412000	1.13E+08	1.22E+08	1.27E+08	1.5E+08	1.46E+08	1.57E+08
		23901000	15172000	18845000	19373000		12809000	29461000
	28979000	37598000						
			18229000		13619000	17550000		
			16025000					
	5853600							
27470000	39936000	40624000	77165000	62234000	55629000	1.3E+08	90182000	1.17E+08
				24195000				
63023000	54048000	72683000	93925000	81627000	71276000	1.03E+08	45393000	73184000
	19322000							
	15256000	28028000	26372000	21619000	46232000	74168000	61139000	52429000
2.51E+09	3.63E+09	2.66E+09	2.85E+09	3.81E+09	3.36E+09	3.75E+09	3.42E+09	5.1E+09
	26666000	25475000	67041000	43730000	43919000	61079000		
1.31E+08	1.47E+08	1.35E+08	1.7E+08	1.27E+08	1.15E+08	1.57E+08	1.1E+08	1.5E+08
1.8E+08	1.97E+08	2.97E+08	1.15E+08	1.13E+08	1.25E+08	1.43E+08	1.18E+08	92088000
					10740000	4532600		6509600
	15324000							25755000
1.15E+08	1.79E+08	93789000	1.33E+08	1.65E+08	2.04E+08			1.19E+08
						19543000	14720000	
			7831600	24076000				
71391000	94849000	63719000	60486000	91269000	77148000	1.14E+08	53158000	1.39E+08
4285900			9475000	5710800	8441300	10956000	14701000	15720000
			12147000	14686000	13019000			
				14584000				
1.2E+09	1.03E+09	1.8E+09	1.79E+09	1.53E+09	1.25E+09	3.73E+09	3.34E+09	3.9E+09
98954000	77635000	1.08E+08	58687000	53728000	47411000		52963000	33177000
1.06E+08	54732000	1.46E+08	2.09E+08	1.48E+08	1.39E+08	2.39E+08	2.08E+08	2.23E+08
31694000	56657000	50984000	55509000	73486000	34438000	88745000	64114000	1.36E+08
56569000		81831000	1.99E+08	2.01E+08	1.92E+08	1.2E+08	83480000	1.33E+08
38434000	77391000	31008000	79339000	1.27E+08	90179000	1.79E+08	1.57E+08	1.6E+08
					9532200			
31016000	92587000		2.08E+08	5.45E+08	4.66E+08	3.46E+08	1.46E+08	2.36E+08
2.47E+08	4.23E+08	4.04E+08	1.48E+09	9.35E+08	5.07E+08	5.47E+08	7.27E+08	1.24E+09
9070000		20923000	64551000				23299000	
			40983000	52515000				

		24812000	19350000	14925000				
57666000	1.01E+08	1.03E+08	1.31E+08	89422000	1.07E+08	1.23E+08	1.06E+08	1.29E+08
	20573000		22393000	35818000	42944000	11464000		
65601000	1.1E+08	1.09E+08	68850000	57165000	51959000	59873000	45958000	52581000
7.04E+08	6.37E+08	8.5E+08	1.16E+09	9.19E+08	9.32E+08	1.5E+09		1.48E+09
1.42E+08	1.59E+08	1.84E+08	2.79E+08	2.78E+08	1.76E+08	1.25E+08	1.19E+08	3.1E+08
					20576000			
58313000	53048000	57078000	7737500	5163200	25161000	31503000	56082000	4294200
	10186000	15084000				21388000		22950000
		6380700	14559000	18492000		13526000		
					13332000			
5.9E+08	8.02E+08	5.07E+08	8.18E+08	1.12E+09	9.59E+08	1.02E+09	7.96E+08	1.01E+09
						44610000	34138000	30218000
	75966000		62817000	65665000	48422000	1.87E+08	1.51E+08	1.68E+08
			59359000	1.44E+08	90201000	1.75E+08	32462000	39094000
			9669200	7573600	3899800	20792000	11017000	
			12180000	11896000	15440000		11003000	18195000
		8517100						1.16E+08
		16425000	15346000		13832000	14729000	31331000	16860000
					10624000			
2.17E+08	1.51E+08	1.17E+08	1.05E+08	95528000	1.02E+08	70773000	67708000	67674000
93287000	1.02E+08	84317000	96022000	78295000	90215000	84121000	1E+08	1.18E+08
27726000	38652000	26526000	41622000	36650000	33562000	35017000	42481000	46072000
				11573000				22025000
	18075000	13661000	12304000	13933000	12147000	13182000	14454000	17771000
69570000	86638000	1.31E+08	92070000	67117000	57076000	54681000	56912000	76665000
			33282000	36600000	37352000			
59412000	51377000	48815000	53370000	49932000	45149000			42463000
50735000	53781000	77140000	77071000	76489000	59535000	59556000	51363000	76109000
21088000		17525000						
9.52E+08	1.27E+09	6.66E+08	2.95E+08	4.77E+08	7.06E+08	6.95E+08	2.41E+08	3.95E+08
99857000	2.54E+08	44599000	46463000	86801000	1.23E+08	83470000	45053000	41484000
			11514000	15270000		16982000		
31040000	43405000	52153000	52987000	41395000	49545000	31489000	23742000	35632000
	11511000	11229000	23759000	26767000	40110000	27410000	27827000	37663000
				2.88E+08	1.68E+08	1.51E+08	21779000	1.83E+08
85705000	86137000	75910000	1.11E+08	88082000	91698000	1.03E+08	1.03E+08	1.03E+08
22675000	17741000	22728000	11770000	11235000	10450000		9778500	
5.15E+08	5.13E+08	6.91E+08	4.34E+08	4.26E+08	3.93E+08	2.65E+08	2.65E+08	3.28E+08
2.15E+08	1.51E+08	2.38E+08	1.16E+08	1.03E+08	89476000	85347000	1.11E+08	94521000
3.19E+08	5.61E+08	3.88E+08	3.73E+08	3.99E+08	4E+08	4.55E+08	3.1E+08	45381000
						18642000		12000000
54897000	52306000	75027000	75854000	61803000	59769000		81720000	61388000
91391000	83381000	1.09E+08	66446000	94776000	82955000	91904000	88470000	1.6E+08
4.75E+08	3.55E+08	4.49E+08	2.85E+08	2.5E+08	2.55E+08	2.89E+08	2.67E+08	2.75E+08
						1.73E+08	1.73E+08	1.98E+08

						96861000	1.12E+08	
16478000	19532000	23557000	18674000	35586000	26327000	31921000		36299000
2656100	1472200	2045600	2179800	988120	1785700	1295900	8538000	2188100
7547500			2.76E+08	45584000	1.26E+08	1.16E+08	90087000	1.25E+08
72375000	53860000	80194000	59679000	52619000	59692000	60587000	1.61E+08	79499000
	42149000	37627000	38897000	78522000	57770000	29639000	71518000	69425000
1.11E+08	72113000	1.04E+08	81013000	78547000	72179000	47957000	56721000	61723000
10368000		11907000	14741000	18047000			14985000	30026000
		1.21E+08	2.06E+08			2.41E+08		2.44E+08
			48330000		39802000	28841000		18079000
2.82E+08	3.83E+08	3.07E+08	2.63E+08	3.38E+08	3.78E+08	2.9E+08	2.83E+08	3.33E+08
	65155000			33471000		24142000	31008000	
				8201100				
			19224000					
	440860		674030		371620	498970	2647900	
87265000	88056000		1E+08	91780000	1.26E+08	1.39E+08	1.33E+08	1.15E+08
	12935000		18223000	10226000				
	21344000		12639000	10100000	15560000	23245000	21159000	16237000
2.8E+08	4.54E+08	2.74E+08	1.05E+08	1.97E+08	2.03E+08	86740000	51396000	89768000
	14002000	12241000	21049000	16129000	16306000	13223000	12857000	21457000
				13444000	13455000			10980000
30862000	2.29E+08	34919000	62354000	73829000	72184000	95196000	27534000	65811000
26827000	1.93E+08	32408000	29607000	1.5E+08	1.2E+08	1.58E+08	79156000	93964000
7.5E+08	1.29E+09	8.11E+08	6.07E+08	7.65E+08	1.1E+09	1.09E+09	6.7E+08	5.56E+08
			14678000	16206000	21655000	17547000		27095000
		6200300	5371900	15749000	20782000		6463600	
	3.43E+08		3.8E+08	3.09E+08			2.74E+08	3.18E+08
9091600			11852000	74737000	1.04E+08	39496000		91243000
87718000	1.42E+08	99936000	1.53E+08	1.58E+08	1.65E+08	1.37E+08	1.14E+08	1.57E+08
1.21E+08	1.31E+08	1.06E+08	76405000		76810000	96401000	76237000	95464000
59608000	80935000	86513000	1.25E+08	96978000	81067000	77683000	1.36E+08	1.15E+08
50692000	65937000	60224000	55862000	1.54E+08	72480000			
	88806000	48952000	24891000			15325000		
	3.29E+08	1.77E+08	1.89E+08	2.7E+08	2.42E+08			
4.61E+08	3.22E+08	5.03E+08	92459000	82860000	2.44E+08	1.45E+08	5.26E+08	6.06E+08
						1.77E+08	1.14E+08	
47179000	77441000	62653000				53854000	17430000	38691000
				23600000	35376000	27139000	24895000	22600000
6.59E+08	8.1E+08	11873000	4.4E+08	5.02E+08	7.26E+08	8.36E+08	7.37E+08	6.37E+08
1.31E+08	97979000	1.13E+08	1.44E+08	1.35E+08	1.14E+08	1.08E+08	93172000	1.05E+08
	91816000		68885000	1.2E+08	64681000	46195000	29204000	43903000

8806300	16601000	9301400	24688000	28488000	32899000	48505000	35835000	60309000
72808000	1.05E+08	95302000	1.51E+08	1.39E+08	1.47E+08	1.56E+08	1.65E+08	3.32E+08
							35548000	
1.81E+08	1.63E+08	2.63E+08	6.43E+08	4.13E+08	6.8E+08	6.44E+08	1.2E+09	9.41E+08
10205000	16129000	85749000	66580000	76849000	83484000	62295000	69367000	71889000
						26513000	29395000	
32810000		51575000	60740000	90863000	86789000	1.02E+08	86224000	78370000
							12698000	
1.05E+08	1.11E+08	1.07E+08	1.71E+08	1.92E+08	1.93E+08	2.6E+08	1.83E+08	2.31E+08
		25040000	17062000	9621600	20597000	23810000	13376000	21527000
			36019000	34248000	33944000	28473000	32348000	33395000
1.32E+08	1.04E+08	1.75E+08	60740000	79342000	40207000	37894000	31925000	47327000
6.37E+08	4.47E+08	5.08E+08	4.18E+08	4.01E+08	4.28E+08	7.42E+08	6.4E+08	8.49E+08
52624000	47974000				33676000			
				4125200			7153700	
	31482000	40348000			25047000			24659000
		5571400	5795100	4867000	4538700	5089200		7660000
				44428000	28443000	27580000		26735000
53998000	2.29E+08	2.87E+08	3.01E+08	3.39E+08	2.1E+08	1.03E+08	84295000	80015000
					23695000			
32132000		22367000	18366000	14292000	13648000	19540000	15473000	18151000
4.39E+08	4.8E+08	4.25E+08	4.4E+08	4.85E+08	4.26E+08	7.28E+08	7.1E+08	7.77E+08
84983000	1.11E+08	84178000		44242000	63845000	1.22E+08		
1.09E+08	1.04E+08	68811000	1.53E+08	1.05E+08	98870000	1.72E+08	1.28E+08	1.46E+08
	41478000	37314000	45883000	44030000	59492000	74002000	50029000	59907000
	34194000			20697000				17802000
	49558000	77095000	62811000	56813000	47295000	81765000	67222000	63547000
	60492000	41843000	32913000	31907000	39361000	51829000	30019000	32640000
2.2E+08	2.82E+08	3.18E+08	2E+08	5.09E+08	3.28E+08	2.45E+08	2.52E+08	1.61E+08
1.84E+09	4.77E+09	2.33E+09	3.65E+09	4.5E+09	4.41E+09	2.84E+09	1.85E+09	2.56E+09
	8945800		15534000	19578000	19243000	21345000	22500000	28983000
	38460000	1.63E+08	19752000	25032000	58789000	21954000	32591000	49603000
			44128000	21766000	25696000			
			44713000	30212000		40575000	26927000	23370000
						14345000		10349000
4.22E+08	2.59E+08	60015000	3.47E+08	2.35E+08	1.89E+08	4.01E+08	2.38E+08	3.27E+08
53916000	57858000	91719000	18139000	20329000	14411000			8734400
					19928000	21294000		
			38946000	39866000	85524000	30254000		
		23838000	53571000	43804000	41092000	57505000		14397000
2.25E+08	2.3E+08	2.7E+08	3.18E+08	2.76E+08	3.05E+08	3.23E+08	2.51E+08	2.86E+08
3.31E+08	3.52E+08	2.5E+08	1.11E+08	1.55E+08	2.44E+08	4.39E+08	4.21E+08	2.32E+08
3.4E+08	3.03E+08	3.94E+08	2.94E+08	2.79E+08	9146000	2.87E+08	2.5E+08	2.94E+08
55235000	59517000	78348000		68554000	74978000	54934000		45904000
29443000	30845000		29947000		14557000	25673000		

	8226500		1.12E+08	77338000	74992000	96018000	16014000	21411000
27798000		42367000	40581000	55879000	66882000	58250000	35754000	31278000
30638000	19382000	28880000	27195000	21359000	18937000	26159000	35935000	31689000
						11716000		9083900
			3856000	2138900	3881600	6387100	17505000	11436000
17649000	45561000	24129000	1.84E+08	3.33E+08	3.62E+08	1.15E+08	1.33E+08	1.47E+08
6237400	913240	2324100	2786700	1092700	1912700	1431500	226860	
2.18E+08	3.32E+08	2.52E+08	2.26E+08	2.09E+08	2.14E+08	2.1E+08	1.79E+08	1.78E+08
			2532100		1626500	2924700	11002000	6037500
						501590	2506800	806100
					8996100			
22966000	15822000	20213000	23708000	12423000	21722000	22961000	1.1E+08	23222000
1.69E+08	1.87E+08	1.79E+08	2.66E+08	3.41E+08	4.16E+08	2.85E+08	2.66E+08	3.12E+08
2.53E+09	2.08E+09	3.52E+09	3.04E+09	2.32E+09	2.3E+09	2.6E+09	3.29E+09	2.64E+09
1.34E+08	89654000	90192000	1.63E+08	1.29E+08	1.22E+08	2.05E+08	1.8E+08	1.87E+08
	16016000		90608000	27033000	18738000	66094000		27965000
			71651000	59183000	55573000	22988000		
7546500	3847900		48889000	17796000	31501000	33238000	2.49E+08	1.34E+08
2.06E+08	2.16E+08	3.22E+08		1.72E+08	1.36E+08	2.06E+08	2.41E+08	2.2E+08
1.41E+08	1.46E+08		1.76E+08	1.5E+08	1.42E+08	1.71E+08	2.02E+08	2.56E+08
12779000	20321000		22457000	37228000	11667000	21960000	11147000	18224000
2.8E+08	2.18E+08	3.52E+08	3.64E+08	2.76E+08	2.17E+08	4.13E+08	3.29E+08	4.62E+08
								17128000
						30397000		
53014000	62720000	52990000	1.02E+08	71428000	70036000	81941000	60740000	95775000
				1.54E+08	1.36E+08			1.25E+08
27427000	65686000	20901000	62274000	79080000	64397000	67220000	34299000	63963000
			27980000	33602000	26403000	22281000	33813000	34316000
3.26E+08	7E+08	4.22E+08	7.71E+08	7.49E+08	8.18E+08	1.63E+09	1.47E+09	1.1E+09
			81249000	57388000	86425000	22740000	17413000	20165000
10606000			33900000	12686000	20105000	27793000		
				2683300	4883500			5831600
	15262000		15257000		16118000	11574000	14440000	18476000
80662000	85268000	78792000	36427000	55982000	75026000	1.58E+08	1.25E+08	1.18E+08
43976000	88104000	86445000	1E+08	1.39E+08	1.25E+08	1.13E+08	77212000	93336000
			7595000	4636300	6103400	9884100		16273000
24079000	33882000							36518000
	7217800	31475000	42845000	34692000	15259000	47072000	38714000	21649000
			18857000	6343300				6186900
	15655000					54874000		59173000
				13036000	25531000	57807000	43736000	
7196400	5029200	6043100	4695800	4590200	5640100	2639700	9236400	2599400
			12261000	13741000	6964600		21632000	38924000
	8269600			26009000	14380000			

20162000	22669000		48380000	44497000	49525000	1.54E+08	1.66E+08	1.31E+08
	14180000	9245900	4224700	17116000	17712000	7612800		23919000
	38479000		30440000	32296000	28514000	46244000	35043000	37901000
3.3E+08	2.92E+08	3.04E+08	3.53E+08	2.59E+08	2.6E+08	3.29E+08	3.06E+08	3.24E+08
26986000	66806000	75296000	9767500	81649000	82420000		47122000	73510000
					7493000		7786200	7690800
		6.42E+08	1.44E+09	1.32E+09	1.34E+09	23708000	29379000	1.88E+09
3.76E+08	1.99E+08	2.21E+08	81702000	73581000	48257000	1.17E+08	92882000	92872000
3.85E+08	3.26E+08	4.63E+08	4.07E+08	3.84E+08	3.03E+08	3.23E+08	2.82E+08	
1.45E+08		1.52E+08	99807000	91469000	88808000	1.01E+08	1.2E+08	1.16E+08
	70977000	63540000	91926000	1.25E+08	1.14E+08	96250000	67112000	1.05E+08
27377000	28691000	39462000	48697000	24259000	29166000	36113000	52486000	50142000
			10330000	28515000	34263000		10659000	45129000
59924000				56928000	61135000		42683000	
					10823000			
			3478500	2942800	4514400			
				27216000	17111000			32091000
34477000	29125000	30230000	32233000	30542000	43572000	27482000	1.04E+08	34749000
41420000	52420000	33774000		23261000	36787000	43687000	34036000	27826000
28741000						76906000	84444000	80797000
11279000	7885300		46995000	32664000	31786000	15152000	14548000	8861200
50723000			68681000	74351000	1.07E+08	1.03E+08	1.52E+08	86494000
					9716100			
46647000					37382000	29497000	19614000	50184000
							10394000	
52511000	84264000	1.19E+08	1.64E+08	83220000	1.13E+08	93158000	1.97E+08	3.32E+08
1.4E+08	1.45E+08	1.47E+08	91303000	84871000	81887000			
								18034000
1.44E+08	1.81E+08	1.54E+08	1.53E+08	1.82E+08	1.8E+08	1.74E+08	1.23E+08	2.01E+08
			6451400	13540000	7664200		8660900	
37812000	43473000	24235000		2.03E+08	1.31E+08	61610000	44137000	47096000
			74922000	1.24E+08	1.16E+08	99802000	23490000	55534000
5.8E+08	7.07E+08	7.58E+08	3.31E+08	4.95E+08	4.68E+08	2.22E+08	1.68E+08	
		30318000					11902000	
			787710	262520	431960	1282500	4815000	848320
	8406200			7775100	16136000			
	40977000							28032000
2.64E+08	3.11E+08	3.34E+08	3E+08	2.02E+08		2.39E+08	1.94E+08	
1.58E+08	3.08E+08	2.3E+08	2.6E+08	2.19E+08	2.24E+08	1.39E+08	2.82E+08	3.21E+08
						37778000		
23030000				27334000			41194000	
18363000	22134000		58992000	48124000	44243000	19706000		
				7670000	10837000			
3.64E+08	4.01E+08	4.55E+08	4.45E+08	4E+08	3.86E+08	5.07E+08	4.7E+08	6.04E+08
	5008200	8327400		11521000	11207000	13229000	29899000	9135800

98313000	85812000	1.21E+08						
			88681000	89463000	72371000	29218000		
			22270000					
83288000	98510000	1.07E+08	1.53E+08	1.33E+08	1.34E+08	1.99E+08	1.83E+08	1.95E+08
					15969000	11322000		
51249000	58207000	46094000	33738000	32545000	32842000	26548000	29252000	41814000
3.46E+08	1.41E+08	1.92E+08	4.06E+08	2.06E+08	3.23E+08	3.71E+08	2.78E+08	4.97E+08
		6135300	5941500	14646000	18237000	3758300		6050800
				21956000	33324000	32268000		
	31746000	31927000	17471000	24055000		35864000	33364000	21370000
22809000	15821000		25202000	22012000	24291000	21168000	35479000	33699000
	16863000			9869900	22075000	6096000		17765000
1.9E+08	2.86E+08	3.17E+08	1.1E+08	1.99E+08	2.26E+08	53459000	1.87E+08	1.87E+08
1.91E+08	2E+08	2.42E+08	2.57E+08	1.63E+08	1.35E+08	2.69E+08	2.26E+08	2.48E+08
81526000	1.22E+08	1.09E+08	2.76E+08	2.58E+08	2.51E+08	2.67E+08	2.13E+08	2.71E+08
						86139000	43843000	50207000
10322000	34348000	21678000	28158000		44083000			
	22464000		24141000	25476000	35439000	31247000		23839000
				15539000	17771000			16073000
1.14E+08	79727000	72445000	85979000	77812000	73934000	81752000		
	37468000	35850000	27985000	49113000	48535000	24536000		17946000
1.32E+08	2.06E+08	72524000	2.76E+08	2.6E+08	2.77E+08	3.66E+08	2.26E+08	2.91E+08
61955000	1.13E+08	84258000	4.2E+08	4.3E+08	3.72E+08	1.82E+08	1.28E+08	1.55E+08
				539040	1408200	1715500	6806900	1487100
40488000	57450000	61344000	1.11E+08	1.09E+08	1.01E+08	1.34E+08	1.09E+08	1.43E+08
				24558000	19046000			
9069100		8023500			23892000	9849800	30020000	22266000
30912000	45054000		91153000	31191000	31909000	25940000		18192000
2.59E+08	4.71E+08	2.64E+08	7.59E+08	9.03E+08	8.79E+08	8.48E+08	3.88E+08	5.84E+08
32285000	8519000	6512000	51908000	20883000	16009000	32548000	1.07E+08	29448000
				33591000	30873000	33791000	31358000	
	14549000		2944200	17379000	17910000	19639000	2067500	26231000
1.74E+08	2.56E+08	3.16E+08	2.43E+08	7.02E+08	2.86E+08	3.74E+08	4.96E+08	5.82E+08
				62235000	25763000	43512000	77481000	31825000
	23622000		82329000	1.03E+08	85055000	36666000	39630000	1.08E+08
59530000	2.06E+08	53705000	1.7E+08	4.69E+08	3.54E+08	2.78E+08	1.73E+08	2.6E+08
82192000	1.16E+08	87097000	1.07E+08	1.61E+08	1.37E+08	1.11E+08	88233000	1.13E+08
2.54E+08	2.59E+08	2.07E+08	2.29E+08	2.41E+08	2.29E+08	2.41E+08	1.8E+08	2.46E+08
					18447000			18766000
					5759000	15382000	17479000	12154000
			21958000	13871000				
1.28E+09	1.23E+09	1.1E+09	2.18E+09	1.74E+09	1.66E+09	2.52E+09	2.37E+09	2.5E+09
			32603000	79582000	1.05E+08			
8.13E+08	1E+09	9.14E+08	8.1E+08	7.08E+08	6.97E+08	6.98E+08	7.46E+08	6.75E+08
	99943000	63113000	1.86E+08				1.04E+08	1.09E+08

1.51E+08	1.47E+08	1.58E+08	1.33E+08	1.09E+08	1.22E+08	1.92E+08	1.3E+08	1.12E+08
1.48E+09	1.07E+09	1.41E+09	1.1E+09	8.18E+08	8.58E+08	8.58E+08	8.53E+08	9.1E+08
	46083000			1.53E+08	1.67E+08	1.29E+08	31611000	33476000
1.93E+08	3.2E+08	2.39E+08	2.62E+08	3.63E+08	2.93E+08	2.9E+08	2.84E+08	3.23E+08
63279000	92347000	1.1E+08	78274000	78473000	75149000	1.73E+08	1.23E+08	2.25E+08
	76213000		92428000	92557000	66426000	1.45E+08	41941000	1.17E+08
				22747000	26261000			
54183000	69470000	56462000	19888000	19487000	16150000	20520000	73825000	19516000
55651000	41773000	54626000	47529000	28164000	34429000	17714000		15753000
					11346000			
1.55E+08	1.69E+08	2.37E+08	2.38E+08	2.01E+08	1.81E+08	1.62E+08	1.93E+08	2.75E+08
11401000	7914300	11978000		18932000			72171000	
				19714000		17589000		10806000
						6986400	27324000	
7.05E+08	1.13E+09	1.73E+09	1.12E+09	6.37E+08	5.73E+08	7.16E+08	6.21E+08	4.25E+08
	24917000		17719000	55149000	37590000	36924000		40605000
1.19E+08	99274000	1.47E+08	1.45E+08	1.07E+08	90867000	1.35E+08	1.21E+08	1.25E+08
6.96E+08	5.12E+08	8.31E+08	9.01E+08	6.06E+08	6.07E+08	9.36E+08	9.61E+08	9.67E+08
7.35E+08	5.42E+08	6.71E+08	3.14E+08	2.91E+08	2.69E+08	2.58E+08	2.56E+08	2.62E+08
					11894000			
		9255300		14978000	7611500		7735400	12742000
1.11E+08	1.93E+08	17403000	6.95E+08	1.31E+09	1.01E+09	8E+08	1.4E+08	3.9E+08
						12385000	16984000	18070000
35496000	72801000	49960000	34323000	45877000	51495000	63013000	52462000	59841000
				12177000				
8701000	4372000	6955300	5434800	3963600	5167200	4644200	14059000	4776100
	60010000		80823000	98047000	83883000	66213000		
63646000		78209000						
	28818000			19525000		23573000	33542000	
		31929000		56516000	72426000	26560000		46994000
				23256000	27453000	27057000		
					24225000			
49384000	73722000	70140000	73464000	89221000	80203000			34617000
50407000	1.03E+08	58839000	29513000	40811000	50250000	52050000		83371000
			35256000	47549000	16724000			
1.12E+08	95858000	86214000	72262000	66666000	71168000	61244000	60602000	85706000
			2404000		3035300			
		54757000	38885000	58054000			66480000	43417000
50029000		82110000			41995000	40439000	44755000	43919000
25911000	62222000	64088000	1.28E+08			1.07E+08	90713000	1.78E+08
				61047000	66303000	90145000		83864000
69800000	65134000			51432000	86832000	1.49E+08		96367000
1.43E+08	1.29E+08	2.04E+08	2.39E+08	1.77E+08	1.62E+08	1.19E+08	1.32E+08	1.63E+08
1.45E+08	1.1E+08	2.02E+08	95395000	1.13E+08	76720000	88280000	1.41E+08	1.08E+08
	6129000	15303000	4101700	12076000	6438300	6562000	7077700	
				10138000	8599600	2525800		
27264000	19362000		26449000	24650000	20023000		31170000	

10546000		28137000					8625800	
	38506000	1.29E+08	40617000	75216000	1.07E+08	23803000		68082000
			18507000					
17631000		26926000	84337000	1.66E+08	96933000	30006000	22224000	24721000
36890000	42222000	51992000	34868000	40982000	46026000	24084000	29882000	45126000
3.84E+08	3.17E+08	3.58E+08	1.82E+08	1.83E+08	1.56E+08	1.33E+08	1.04E+08	1.46E+08
	13732000		25119000	28143000	24855000	33496000	32633000	44187000
			39746000	36630000	64727000	34761000		
	2.01E+08			2.6E+08	3.07E+08	2.38E+08	4.05E+08	4.66E+08
45130000	33115000	46420000	43019000	68273000	12317000	52003000	35654000	22166000
32543000	46580000	70552000	1.15E+08	78397000	1.43E+08	1.06E+08	1.01E+08	1.24E+08
			22928000	25518000	7055200		15739000	
4.03E+08	3E+08	2.86E+08	2.75E+08	2.46E+08	1.97E+08	2.19E+08	2.26E+08	2.21E+08
81721000	74260000	1.46E+08	1.13E+08	1.19E+08	1.2E+08	40604000	41270000	49089000
1.2E+09	1.07E+09	1.36E+09	2.37E+09	1.53E+09	1.36E+09	1.46E+09	1.96E+09	1.99E+09
1.71E+08	2.1E+08	2.12E+08	2.09E+08	1.67E+08	1.79E+08	1.69E+08	1.95E+08	2.51E+08
			73042000	70501000	74262000	61944000		9573500
				35439000				
			21382000	39159000	27897000		21520000	36570000
								11419000
1.24E+08	1.83E+08	1.95E+08	1.55E+08	1.4E+08	1.15E+08	1.16E+08	1.39E+08	
				2.02E+08				
1.02E+09	9.89E+08	1.24E+09	1.01E+09	7.63E+08	6.93E+08	8.23E+08	8.77E+08	9.44E+08
			10209000		10406000	12045000	8205700	13051000
85652000	3.18E+08	99472000	1.37E+08	3.41E+08	3.06E+08	1.59E+08	1.18E+08	1.99E+08
34846000	23257000	21613000		20082000	33156000		23859000	
					4339000	12146000	7377500	5892800
1.11E+08			94414000			81813000	85190000	92273000
				72028000	61501000			
			31140000	18729000	30114000			38316000
8.71E+08	6.32E+08	7.52E+08	7.43E+08	5.76E+08	4.31E+08	4.15E+08	3.95E+08	4.61E+08
2.62E+08	2.42E+08	3.07E+08	33903000	2.42E+08	1.88E+08	39723000	3.24E+08	33709000
32417000	53959000	33719000	78043000	62106000				52703000
25613000	37511000	26205000	17581000	15424000	16297000	6545800		10528000
	21678000	25690000		15237000		12415000	42905000	52689000
65359000	49640000	63612000	42428000	37831000	38513000	36529000	49483000	52707000
	52427000		44284000	73577000	62883000	65876000	82713000	96489000
	76169000	42320000		65466000	57984000		54339000	
8.47E+08	1.74E+09	1.15E+09		1.09E+09	1.94E+09	7.75E+08	2.13E+09	2.57E+09
32731000	34324000	31291000	32408000	30363000	22573000	24548000	32927000	31582000
				16263000	16970000			24261000
				2399100	2774500			8395100

				10127000	7740100	6445300		9472300
32849000	23782000	42577000	40183000	37690000	29767000	53801000	44035000	44697000
51519000	76431000	94475000	1.42E+08	1.18E+08	1.18E+08	1.59E+08	2.33E+08	1.81E+08
1.16E+08	92953000	1.1E+08	91651000	71937000	69595000	1.08E+08	97655000	92866000
	14462000	25679000	34200000	30722000	22967000	37415000	55785000	40917000
67198000		65600000			66111000	6514500		1.49E+08
1.66E+08	1.2E+08	1.98E+08	1.8E+08	97788000	38427000	1.51E+08	1.4E+08	1.24E+08
	4.09E+08	27689000	4E+08	2.36E+09	2.03E+09	2.17E+09	4.85E+08	1.36E+09
			99548000	67202000	64620000	54723000		19634000
	43516000		36302000	55444000		32808000	25579000	35814000
11715000	14117000		18414000	18506000	17346000			
56576000	55153000	84764000	27424000	39085000	36935000		31687000	31497000
				1.49E+08	56858000			
				13568000	21544000			
1E+09	9.79E+08	1.05E+09	8.38E+08	8.99E+08	7.48E+08	7.79E+08	1.33E+09	1.26E+09
33771000	56574000	47991000	78000000	52041000	49659000	80208000	53578000	77037000
24665000	41684000		76802000	46625000	57790000	39978000		20761000
35529000		50640000	31054000	25747000	24775000	29155000		42306000
5.21E+08	4.23E+08	4.8E+08	5.82E+08	5.21E+08	5.31E+08	5.63E+08	7.41E+08	1.12E+09
31060000	93121000		1.24E+08	2.53E+08	2.27E+08	2.03E+08	53637000	1.02E+08
69929000	60223000	75125000	91088000	72956000	47881000	76162000	68700000	70466000
	15621000		12776000	11665000	8691200	15176000	13795000	15730000
1.59E+08	1.49E+08	1.92E+08	11592000	3.67E+08	3.02E+08	3.03E+08	3.24E+08	3.56E+08
5.18E+08	5.98E+08	5.26E+08	19337000	21870000		2.89E+08	2.67E+08	
			3453600					
69944000	55952000	76897000	81831000	69300000	60266000	68158000	65465000	77899000
88576000	86679000	1.15E+08	96328000	68723000	69026000	94272000	98122000	82666000
					2525400			
	20267000	22000000			32155000		13691000	36475000
31268000	40743000	40173000	44409000	31385000	35428000	30268000	33873000	47361000
			21716000	14936000	19028000	19943000		20286000
54848000	43883000	65572000			45922000	45026000	43907000	
			14119000	17267000	13276000			
			10714000	6456400	10262000		10452000	1536300
								1.3E+08
			11342000					13411000
16640000		6675300		15046000	11509000		23676000	
			50238000	69378000	61291000	32161000	16840000	26421000
3.41E+08	2.45E+08	4.3E+08	3.57E+08	5.4E+08	5.03E+08	4.81E+08	1E+09	5.77E+08
85429000	70774000	1.09E+08	1.52E+08	1.03E+08	1.13E+08	1.8E+08	1.81E+08	
2.77E+09	2.18E+09	3.48E+09	2.18E+09	1.63E+09	1.47E+09	2.39E+09	2.63E+09	2.4E+09
	20344000	28058000	20297000					22174000
				13764000				
		15208000		11554000				
		12790000	20648000	16314000		20190000		23905000

3.18E+08	3.39E+08	3.81E+08	2.74E+08	3.75E+08	3.4E+08	3.86E+08	2.63E+08	3.32E+08
97032000	93225000	34642000	65670000					
1.1E+08	29343000	1.02E+08	1.22E+08	93005000	92817000	1.18E+08	1.05E+08	1.28E+08
10254000	19948000	14619000	14789000	12414000	15572000	15557000		12597000
	26846000	16390000		23302000	31453000	40358000	36966000	25405000
29733000	50956000	42264000	70888000	55243000	52021000	74690000	66333000	73988000
		10178000		14382000	15462000		7998500	12172000
7.69E+08	4.59E+08	5.26E+08	1.72E+08	1.97E+08	1.9E+08	2.22E+08	2.38E+08	1.47E+08
				57709000		29320000		
1.35E+09	1.45E+09	9.33E+08	4.52E+08	6.34E+08	7.95E+08	7.58E+08	7.11E+08	4.75E+08
20536000	1.44E+08	1.51E+08	1.17E+08	1.09E+08	1.86E+08	3.17E+08	2.97E+08	2.29E+08
2.11E+08	1.87E+08	2.24E+08	1.2E+08	1.15E+08	1.47E+08	3.54E+08	3.49E+08	2.69E+08
2.23E+08	3.62E+08	1.88E+08	79963000	1.73E+08	2.37E+08	3.3E+08	2.87E+08	1.69E+08
	34107000		20035000			44357000	20819000	
37791000	38338000	42792000						
1.04E+08	1.88E+08	2.1E+08	39593000	1.04E+08	1.52E+08	47472000	39405000	44652000
1.1E+08	1.78E+08	1.72E+08	78594000	67131000	91073000	1.16E+08	1.09E+08	53683000
	27304000	20307000		19517000	40309000	47093000	11455000	
1.18E+08	2.24E+08	2.19E+08	2.04E+08	2.39E+08	3.2E+08	6.35E+08	5.14E+08	4.03E+08
	7.37E+08			5.07E+08	7.03E+08	1.11E+09	6.72E+08	6.4E+08
1.48E+08	1.33E+08	1.4E+08	84275000	75568000	1.21E+08	2.34E+08	1.5E+08	1.34E+08
		30306000				26261000		
		41057000	31113000	25092000	33472000	23979000		28527000
34798000	44911000	36650000	32209000	28146000	32044000	30594000	28264000	29475000
		62217000	55860000		70976000		97892000	
58310000	96420000	63888000	1E+08	96901000	81420000	94878000	27730000	1.04E+08
	20083000	21266000	29384000	26536000	25076000	23158000	27086000	30806000
	84744000		83651000	2.02E+08	11854000		14243000	1.29E+08
1.3E+08	2.32E+08	1.42E+08	2.24E+08	2.91E+08	3.18E+08	2.71E+08	1.28E+08	2.22E+08
8415800	13890000	9101500	64020000	46895000	75439000	70496000	83239000	86319000
1.28E+09	1.39E+09	1.31E+09	1.8E+09	1.91E+09	1.87E+09	2.14E+09	1.43E+09	2.11E+09
28771000	33257000		23151000	23776000		12448000		18466000
16341000						22600000	26664000	17491000
12259000	19924000	11547000	21382000			11523000		13173000
			24879000	21447000	12293000	19557000	23839000	17266000
34880000	1.54E+08	1.03E+08	1.62E+08	2.04E+08	2.26E+08	2.11E+08	1.58E+08	1.19E+08
				5542100				
	19300000				16196000	16885000		
	34272000		21309000		21933000	30587000		

1.2E+08	1.13E+08	1.34E+08	1.41E+08	1.12E+08	1.47E+08	1.64E+08	2.9E+08	1.62E+08
								4983100
			2156100	1694300	4702500	7476100	15096000	4415700
	18771000			30350000	21414000			17625000
14406000			7444600	16571000	14816000	18005000		19686000
							4958100	
	27023000	27405000		28431000	26372000	36463000	25213000	22000000
1.26E+08	2.24E+08	1.1E+08	1.86E+08	2.87E+08	2.63E+08	2.56E+08	1.32E+08	2.15E+08
			6088300	4429900	3653400			
23072000	46282000	34989000	64431000	69547000	81699000	69826000	64770000	76627000
				13117000	12937000			14532000
5E+08	5.89E+08	4.82E+08	9.85E+08	9.98E+08	9.18E+08	9.21E+08	7.11E+08	7.45E+08
				8940000		13713000	11146000	
				72857000	65594000	83832000		37397000
79301000	1.26E+08	1.07E+08	1.5E+08	4.7E+08	2.54E+08	2.05E+08	1.83E+08	2.55E+08
49173000	72271000	50041000	1.34E+08	1.2E+08	1.44E+08	1.2E+08	74756000	97993000
	1.08E+08		71032000					45724000
	15741000	19518000	24918000	12929000	14010000	18533000	20248000	16889000
2.21E+08	2.43E+08	1.13E+08	1.34E+08		1.33E+08	1.96E+08	1.26E+08	93353000
	15912000	16430000	31182000	32053000	30928000	16394000	48135000	79707000
46027000	8819600	7929600	1.94E+08	1.05E+08	97393000	1.31E+08	92367000	93725000
2.46E+08	3.14E+08	2.72E+08	2.27E+08	2.75E+08	2.89E+08	2.25E+08	1.58E+08	2.05E+08
17761000	55182000	30166000	62736000	46039000	77289000	63940000	48270000	69652000
1.01E+09	1.58E+09	8.6E+08	8.8E+08	1.11E+09	9.56E+08	5.68E+08	4.8E+08	5.23E+08
			33389000	31041000	21404000			
27540000				45889000	37534000	32819000		
1.68E+08	1.76E+08						77793000	89137000
					18033000	44379000		28329000
20871000	62750000	22532000	1.77E+08	2.73E+08	2.42E+08	1.85E+08	91930000	1.69E+08
			19710000		14666000			
1.45E+08	1.15E+08	75461000	87708000	1.24E+08	1.77E+08	85049000	1.1E+08	1.18E+08
			7804400	11405000	15997000			
				7.98E+09				
86390000	1.41E+08	1.37E+08	1.82E+08	1.23E+08	1.61E+08	1.17E+08		1.37E+08
					16481000	20705000	6575100	
60271000	80438000	22073000	1.37E+08	71731000	71942000	1.38E+08	2.08E+08	1.7E+08
	48825000	20017000	48582000	49335000	56292000	1.18E+08	28790000	54734000
			8885100	7576200	14289000	5595600	17881000	7733700

1.06E+08	1.24E+08	1.06E+08	83149000	79397000	67387000	77947000	54617000	68860000
76036000	88271000	99718000	69876000	71410000	66815000	99588000	76387000	75701000
	48780000			12219000	10183000			
1.79E+08	2.51E+08	1.82E+08	2.4E+08	2.3E+08	2.37E+08	2.47E+08	2.22E+08	2.27E+08
12245000	7395200	9740300	8563200			5387700		
2.19E+08	1.8E+08	2.03E+08	2.23E+08	1.57E+08	1.38E+08	1.54E+08	1.68E+08	1.82E+08
	39775000		47436000	36587000	37689000	45323000		33954000
	16726000	17754000	15762000	25799000	45700000	33257000	36471000	29436000
1.61E+08	1.83E+08	1.77E+08	1.73E+08	1.71E+08	1.42E+08	1.81E+08	1.6E+08	1.97E+08
7.26E+08	7.72E+08	7.55E+08	5.52E+08	4.85E+08	4.49E+08	6.59E+08		5.05E+08
3.08E+08	4.35E+08	3.3E+08	2.3E+08	3.01E+08	3.37E+08	2.1E+08	1.44E+08	1.66E+08
			73770000	93539000	97247000	1.21E+08	72889000	1.25E+08
5.54E+08	5.75E+08	5.08E+08	4.79E+08	4.57E+08	4.26E+08	5.29E+08	3.1E+08	5.67E+08
			14353000				27989000	
			12340000	7467100	10439000	17648000	68385000	19457000
1.95E+08	2.45E+08	1.92E+08	4.16E+08	4.52E+08	4.11E+08	4.24E+08	4.66E+08	5.1E+08
		35013000	39209000					
				36576000	27817000			
1.75E+08	2.15E+08		2.2E+08	2.27E+08	2.32E+08	2.96E+08	2.12E+08	2.39E+08
26464000	63281000			1.59E+08	1.18E+08			
52049000	93167000	58542000	1.5E+08	2.09E+08	2.04E+08	1.59E+08	97363000	1.34E+08
6698400	5275700	6223700	22673000	12311000	14326000	23111000	69524000	25494000
32121000	36744000	25330000	32615000		22126000			45306000
94327000	81920000	1.71E+08	2.34E+08	2.18E+08	1.03E+08	2.5E+08	2.36E+08	2.52E+08
	10671000	20361000	23772000	15534000	16538000	30471000	11362000	28138000
4.36E+08			4.12E+08					
	2957800		11583000	4769400	6041000	8715000	12675000	13621000
			42116000	4465900	14448000	18695000	38041000	45840000
					11151000	13370000		
7353900	3191000	5662400	5150300	2856500	4971500	5713400	21575000	6242900
	36191000			16161000		50746000	51002000	46874000
8171100	6862700	8928100	9489700	6030100	12260000	9463800	22749000	15655000
			1.18E+08	1.29E+08	1.17E+08	2.06E+08	90018000	90334000
26936000	35307000	34521000	37812000		48886000	17704000	16123000	24149000
25036000			10936000				32807000	15593000
2.65E+08	2.09E+08	2.95E+08	4.29E+08	3.12E+08	2.54E+08	3.19E+08	3.6E+08	3.49E+08
			297570				1112800	
			42758000				61077000	51309000
27515000	54779000	37161000	29631000	23509000	28924000	44354000	34779000	35403000
36648000	49377000	61476000	38699000	39222000	33174000	35009000	24306000	40023000
			17752000	14376000	15391000	10880000	3786800	

2.49E+09	2.7E+09	1.3E+09	3.21E+09	3.37E+09	3.54E+09	3.38E+09	1.03E+09	3.15E+09
	1.56E+08	42976000	36655000	1.18E+08	4.64E+08		43174000	1.74E+08
	15251000			10724000		12792000		
7854200		10547000			9381400		5734500	
1.26E+08				1.19E+08	1.1E+08	1.34E+08	95340000	1.2E+08
2.04E+08	3.09E+08	81403000	3.03E+08	2.41E+08	2.69E+08	1.75E+08	1.08E+08	1.3E+08
		1.05E+08					1.2E+08	
68196000								
1.3E+08	1.83E+08	1.29E+08	2.31E+08	3.57E+08	3.2E+08	2.64E+08	2.64E+08	3.35E+08
4.47E+08	4.39E+08	6.26E+08	6.72E+08	5.11E+08	6.05E+08	6.15E+08	6.59E+08	4.72E+08
1.9E+09	2.57E+09	1.85E+09	1.92E+09	2.13E+09	2.16E+09	1.17E+09	1.05E+09	1.32E+09
			73417000	1.1E+08	48746000	33514000		
	2142100	4063900	2635700	1787000	2298100	2492200	7219100	3482500
17992000	42160000	43130000	78029000	69780000	75503000	1.08E+08		1.2E+08
2.78E+08	2.08E+08	2.93E+08	3.21E+08	2.38E+08	1.71E+08	2.69E+08	2.85E+08	2.73E+08
	17300000		27799000		9618000	13903000	13151000	28269000
8.64E+08	7.29E+08	6.77E+08	7.83E+08	6.39E+08	6.06E+08	4.66E+08	3.72E+08	5.36E+08
1.85E+08	2.17E+08	2.47E+08	1.43E+08	1.3E+08	83084000			
25446000	30478000			15691000	21045000	35955000		19879000
	45523000		1.67E+08	2.75E+08	2.18E+08	2.76E+08	80128000	88180000
63193000	40605000	45681000			44167000	44177000		
1.38E+09	1.29E+09	1.31E+09	9.59E+08	8.64E+08	7.89E+08	1.07E+09	1.01E+09	1.24E+09
3.83E+08	3.92E+08	1.29E+08	6.35E+08	4.66E+08	3.64E+08	3.92E+08	5.21E+08	5.36E+08
1.28E+08	67030000	1.54E+08	18888000	1.14E+08	58800000	1.15E+08	86883000	1.05E+08
1.55E+08	1.1E+08	1.9E+08	2.62E+08	1.7E+08	1.82E+08	2.06E+08	5.79E+08	2.39E+08
22325000	15133000	19246000	7950300	8410300	6585300			9558500
	1.68E+08		1.24E+08	1.96E+08	1.55E+08	1.5E+08	81727000	1.26E+08
						36000000	34124000	27375000
			30002000	42901000	35150000			25983000
				1.1E+08	99486000			
9257500	5625400	6470600	8220200	5905500	13165000	4550600	24622000	8659300
1.06E+09	8.66E+08		8.37E+08	1.41E+08	1.77E+09	4.67E+08	3.42E+08	3.03E+08
88911000		74543000				50867000	38241000	
		39043000	25404000			5296000	70873000	
5527900	2245500	4388600	6230400	1982200	2636700	2429900	11138000	4832400
2.14E+09	2.76E+09	2.56E+09	3.41E+09	4.22E+09	3.47E+09	2.97E+09	2.66E+09	3.39E+09
					20464000	17340000		24861000
		44052000			41473000	53452000	60645000	
			1.04E+09		2E+08	9.71E+08	9.82E+08	1.01E+09
			15368000		10720000		34984000	25238000
1.01E+08	1.21E+08	1.28E+08	1.22E+08	1.81E+08	80765000			1.09E+08
25506000	29646000	27940000	25100000	23945000	23882000	18414000	15786000	20594000
20792000	29727000		58463000	1.03E+08	1.19E+08	1.17E+08	1.15E+08	86532000

8189200	10382000		11420000	6489700	9578200	8085100	10707000	11180000
	29531000	25124000						
				27522000				
			34013000	27348000	24918000			
39975000		41521000	57098000	35588000	50633000	47373000	55009000	57343000
23943000	24125000	25714000			22350000		30459000	
	45477000		41094000	49611000	49981000	47267000		
25501000		20355000		45513000	74509000		2.63E+08	72341000
54038000	66443000	61334000	57750000	76443000	58769000	82613000	48430000	55718000
7.46E+08	7.07E+08	1.11E+09	1.1E+09	9.59E+08	8.18E+08	6.86E+08	7.12E+08	8.07E+08
	26996000		17586000	23438000	17955000			
83155000	68229000	1.14E+08	48701000	41312000	33446000	29683000		32417000
			34906000	30517000	28200000			23318000
				4450100	3906300			
20259000				25270000				
			1801200	762150	1026500	1338400		1510500
				8506600	16708000			
1.66E+08	2.05E+08	2.92E+08	2.17E+08	1.88E+08	1.54E+08	1.86E+08	1.84E+08	2E+08
1.55E+08	2.69E+08	1.85E+08	2.52E+08	1.86E+08	2.12E+08	3.68E+08	2.28E+08	3.03E+08
4267800	2459700	3012800	5443900	4691800	7935600			
	1.04E+08		99171000		6636600	86678000	1.3E+08	1.09E+08
				3985400				
				48395000				
85057000	68976000	67038000	63107000		71914000	1.18E+08	92513000	80248000
	18423000	14066000	18761000	26509000	21539000	27523000	16036000	24902000
1.5E+09	1.29E+09	1.45E+09	9.45E+08	8.4E+08	7.13E+08	1.02E+09	8.28E+08	8.71E+08
11844000					19785000			
							7171800	2166600
			7967600	83647000	47561000	34894000	46646000	44435000
2.72E+08	3.3E+08	3.09E+08	3.86E+08	3.71E+08	2.71E+08	1.68E+08	2.9E+08	4.22E+08
76158000	67255000	91718000	41260000	32334000	28521000		24673000	24692000
	2.01E+08	2.28E+08	1.17E+08				23769000	1.27E+08
4.26E+08	3.92E+08	5.17E+08	4.43E+08	3.41E+08	2.9E+08	4.1E+08	4.31E+08	4.19E+08
67088000	39275000	92242000	18511000	20272000		13210000		
	82469000	1.18E+08	1.14E+08	80121000	94801000	35376000		98537000
3.17E+09	1.86E+09	3.41E+09	1.92E+09	1.67E+09	1.36E+09	1.49E+09	1.37E+09	1.6E+09
40988000	46937000	69332000	57168000	54238000	57649000	63147000	87391000	73146000
34286000		70405000			37701000	29340000		
2.83E+08	2.97E+08	4.69E+08	1.01E+08	1.22E+08	1.03E+08	33972000	26768000	39165000
46141000	38072000	48952000	76883000	54396000		86284000	2.03E+08	91842000
1.14E+09	1.28E+09	1.22E+09	1.04E+09	9.64E+08	7.96E+08	4.78E+08	8.48E+08	7.72E+08
				13209000	13229000			

6.28E+09	4.43E+09	5.2E+09	2.98E+09	2.09E+09	2.2E+09	1.87E+09	2.12E+09	2.07E+09
	18366000		11354000	13933000		13200000		
		19882000						
	35239000	19603000	29257000	31406000	31909000	22959000	23347000	29030000
			1.99E+08	79159000	1.46E+08	1.8E+08	64923000	45255000
				12260000	13113000			
27399000	24592000	24431000	10635000	10833000	15182000	5353100	6859000	
4.29E+08	2.89E+08	4.82E+08	1.03E+08					
2.53E+08	2.09E+08	2.6E+08	2.07E+08	1.71E+08	1.42E+08	1.71E+08	1.79E+08	1.79E+08
					2623300			
4.27E+09	3.17E+09	4.49E+09	3.4E+09	2.5E+09	2.35E+09	3.16E+09	3.79E+09	3.25E+09
2.29E+08	2.08E+08	2.74E+08	1.13E+08	1.1E+08	95431000	1.09E+08	1.01E+08	1.52E+08
			20423000	37984000	19993000			
1.61E+08	1.63E+08	1.84E+08	1.6E+08	1.9E+08	1.7E+08	1.21E+08	1.58E+08	1.74E+08
		45058000	58423000	64055000	54282000	54568000	29901000	39826000
			18482000	18449000				
15023000	10193000	12344000	5453200	4671900	7011200	6033400	20833000	8638300
2.4E+08		2.34E+08	2.43E+08	1.13E+08	1.45E+08	1.04E+08	74814000	2.04E+08
			24059000	37305000	22744000	31167000		28717000
				22043000				
	64757000		1.45E+08	1.07E+08	94347000	1.03E+08	83136000	1.2E+08
	39403000		23936000	31012000	29424000	62019000	54397000	41996000
2.57E+09	1.24E+09	1.83E+09	1.63E+09	1.76E+09	6.72E+08	1.16E+09	1.26E+09	9.72E+08
			25851000	11348000	20722000			
7368400	2047600	3687200	4384200	1909700	2087000	1840500	15508000	2382700
33066000	63576000	35998000	47718000	57809000	64142000	53535000	38643000	41728000
2.57E+08	3.39E+08	2.03E+08	3.07E+08	3.17E+08		3.75E+08	3.96E+08	4.68E+08
2.65E+08	2.06E+08	2.45E+08	2.4E+08	2.75E+08	1.71E+08	2.76E+08	2.21E+08	2.44E+08
89836000	1.79E+08		75522000	1.22E+08	1.58E+08	2.56E+08	98660000	86543000
4.84E+08	1.22E+09	7.53E+08	2.69E+09	2.93E+09	2.33E+09	2.01E+09	1.21E+09	1.88E+09
	21308000		4.21E+08	54675000	52571000	1.31E+08	20646000	72670000
1.31E+08	1.12E+08	1.49E+08	25804000	28745000	21369000	23594000		21434000
			34904000	21220000	21595000	31753000	26167000	36290000
49153000	1.04E+08	66329000	1.01E+08	1.04E+08	1.2E+08	89086000	65947000	89294000
9743900	16001000	84459000		10705000	29260000			
	65884000		16413000	32487000	31395000			
1.23E+08	1.19E+08	1.31E+08	1.28E+08	98051000		93415000	1.33E+08	1E+08
			14089000	15565000	20497000	14859000	21708000	14953000
51516000	53506000	48873000	92989000	87571000	1.01E+08	1.25E+08	98686000	1.04E+08
	28848000		63487000	68383000	62519000	74747000	54532000	47703000
	54087000		54550000	92948000	84816000	51781000	45040000	64944000

43697000	1.67E+08	81534000	75732000	82117000	78166000	81917000	42932000	51607000
				35544000				
81518000	1.28E+08	1.42E+08	86436000	99314000	1.4E+08	82728000	1.51E+08	1.39E+08
			59441000		63136000	81165000	76162000	1.05E+08
		5143700					3057600	6305300
	25830000		1.1E+08	2.46E+08	1.5E+08	1.18E+08	24135000	83432000
					9633600			16958000
8863700					17358000		37174000	18965000
				18958000				
	17234000			19092000	21604000			18159000
11063000		12746000	13837000		8134200	14417000	12691000	18745000
				54507000	51401000	57530000	56606000	56340000
61146000	45745000	2.28E+08	2.49E+08	1.99E+08	1.6E+08	1.53E+08	1.34E+08	1.92E+08
85479000	3.71E+08	1.73E+08	3.36E+08	5.46E+08	4.52E+08	3.32E+08	1.73E+08	2.65E+08
				7645800				
50028000	82677000	74063000	1.36E+08	1.66E+08	1.55E+08	1.59E+08	1.67E+08	2.17E+08
			18032000	26714000				
16009000	16992000	22647000	17286000	20763000	18812000	12726000	19020000	23118000
40429000	35163000		26313000		39991000	69817000	73107000	
		26981000						
	1.22E+08		1.29E+08	1.76E+08	1.24E+08	2.25E+08	16886000	1.62E+08
	22021000		35728000	35651000	48323000	46005000	76563000	72013000
67105000	57413000	71348000	2.05E+08	1.64E+08	2.01E+08	1.74E+08	1.47E+08	1.34E+08
3.14E+08	2.95E+08	2.72E+08	1.27E+08	1.7E+08	1.72E+08	74530000	61515000	62733000
	16474000						16863000	
79996000	97158000	1.58E+08	1.31E+08	62481000	80648000	1.53E+08	1.62E+08	1.71E+08
22851000	29314000	46846000	53031000	47371000	30849000	49433000	70628000	84049000
			8764300	21404000	17594000	11108000		10631000
1.27E+09	1.16E+09	8.67E+08	1.35E+09	1.15E+09	1.12E+09	1.29E+09	1.65E+09	1.55E+09
								25537000
1.47E+08	1.23E+08	1.31E+08	2.49E+08	2.33E+08	2.26E+08	3.68E+08	4.09E+08	4.17E+08
		22153000	21568000	27237000		35496000	22076000	33400000
1.08E+08	86555000	1.12E+08	31119000	47353000	33105000			
							1.31E+08	1.31E+08
				14544000	19544000			
1.08E+08			71254000	25836000	23609000			
								18873000
48398000	51247000	77785000	81007000	54324000	46845000	62385000	54926000	70212000
1.8E+08	9.87E+08	6.32E+08	3.27E+08	4.81E+08	2.51E+08	3.13E+08	4.83E+08	4.89E+08
3.6E+08	2.81E+08		3.07E+08	2.15E+08	1.94E+08	41005000	43388000	2.58E+08

10378000	16702000	17603000	19634000	22341000	25914000	20740000	36337000	29934000
12583000			7839600				11305000	
1.88E+08	1.17E+08	2.42E+08	1.01E+08	1.16E+08	1.29E+08			
16551000	29523000	24866000	32233000	59547000	66623000	47561000	75509000	66307000
				19627000				19776000
				20024000				
2.75E+08	2.39E+08	3.05E+08	1.42E+08	85521000	81275000	65389000	57853000	79313000
31259000	30106000	57669000	42833000	32237000	25302000	33313000	19923000	39947000
3.69E+08	2.95E+08	5.85E+08	5.45E+08	8.7E+08	7.87E+08	6.71E+08	3.1E+08	6.59E+08
		2313600	2432300	950130	1655800	1892900	9716500	3360100
1.94E+08	1.49E+08	2.01E+08	1.64E+08	1.47E+08	1.16E+08			1.02E+08
					10618000			
15085000	90220000	36119000	33530000	4.01E+08	3.91E+08	53167000	67995000	1.73E+08
49812000	64091000	87953000	62442000	72810000	74941000	77929000	84330000	78670000
	34893000			32131000	34630000	44070000	30636000	37539000
			9797000	16991000	13944000	11398000		
56588000	59287000	59462000	77266000	60362000	83089000	85391000	1.01E+08	1.12E+08
			8688000	7262600	5470400	4271100		
4.36E+08	2.74E+08	3.92E+08	2.96E+08	2.52E+08	2.15E+08	1.1E+08	1.48E+08	1.51E+08
1.03E+08	1.12E+08	1.39E+08	53571000	65196000	94778000	1.25E+08	1.57E+08	83596000
82889000	1.49E+08		93449000	1.6E+08	1.22E+08	39957000	31504000	41154000
50267000	63247000	42432000	36557000	32043000	38901000	50083000		41118000
1.3E+08	1.44E+08	1.15E+08	1.22E+08	1.03E+08		1.12E+08		1.16E+08
1.24E+08	78498000	48448000	69385000	51651000	50017000		27842000	63983000
				6293100	7784500			
1.92E+08	2.37E+08	1.77E+08	2.71E+08	3.39E+08	3.21E+08	3.61E+08	3.81E+08	3.79E+08
1.63E+08	1.25E+08	1.69E+08	91013000	79501000	74544000	62804000	78159000	65696000
				39799000	39400000		38106000	56038000
5.6E+08	9.04E+08	5.21E+08	6.11E+08	7.24E+08	6.82E+08	3.69E+08	2.63E+08	3.22E+08
22302000	22898000			15200000				
	31749000	50104000			17255000			
80655000	81571000	73026000	37147000	53636000	77377000	1.35E+08	77844000	65679000
			26779000					
1.11E+08	1.25E+08	1.47E+08	1.56E+08	1.37E+08	35893000	81429000	1.34E+08	1.44E+08
			1.54E+08	1.28E+08	1.07E+08	1.16E+08	1.22E+08	1.55E+08
5.33E+08	4.33E+08	6.55E+08	81674000	59693000	60981000		24014000	18051000
1.79E+08	2.13E+08	1.56E+08	60111000	1.07E+08	1.54E+08	2.36E+08	2.84E+08	1.64E+08
	9015700							10340000
2.09E+08	1.59E+08	2.02E+08	2.53E+08	1.83E+08	1.7E+08	2E+08	1.96E+08	2.21E+08
70146000	64657000	1.17E+08	1.55E+08	1.43E+08	1.34E+08	2.13E+08	1.78E+08	1.88E+08
				37249000				
72134000	73305000	86404000	70829000	38678000	36489000	65213000	44541000	46622000
7.85E+08	6.67E+08	1.02E+09	4.43E+08	3.66E+08	4.04E+08	2.41E+08	2.33E+08	3.23E+08
22498000	20576000	20743000	19723000	18821000	29353000			
54742000	72872000	75560000	1.41E+08		1.04E+08	68770000	1.29E+08	1.37E+08
	8569800				8319900			
6.18E+08	5.07E+08	6.23E+08	4.36E+08	3.68E+08	2.99E+08	2.99E+08	3.55E+08	3.87E+08
69673000	62652000	70106000	66288000	52210000	58236000	48577000	50749000	55322000

40101000	1.02E+08	42980000	46293000	1.54E+08	1.67E+08	58259000	63176000	97547000
							27602000	
3.65E+08	5.07E+08	4.55E+08	5.51E+08	5.73E+08	4.8E+08	4.35E+08	5.87E+08	6.09E+08
8.55E+08	1.55E+09	1.92E+09	1.4E+09	1.25E+09	1.77E+09	1.17E+09	1.65E+09	1.53E+09
8.46E+08	6.67E+08	9.35E+08	4.52E+08	3.97E+08	3.94E+08	3.55E+08	4.3E+08	3.89E+08
	2202300		3222700	1429700			7257700	3630800
	17971000				22107000		15542000	13434000
79655000	73904000	81533000						
40548000	25969000	49442000	46798000	41184000	31956000	44128000	38017000	38144000
1.9E+09	1.35E+09	9.31E+08	8.44E+08	1E+09	1.04E+09	1.43E+09	1.85E+09	1.89E+09
	26884000	31083000	43251000	69450000	84201000	24849000	33557000	53385000
24895000	13137000	21931000	17734000	8660800	13123000	16295000	60744000	28004000
			42298000	39791000	36633000	32264000	12566000	14203000
	1.05E+08	1.65E+08						
1.96E+08	2.36E+08	2.94E+08	4.51E+08	4.18E+08	4.21E+08	7.33E+08	8.81E+08	7.6E+08
18777000	36634000		14961000	37902000	53032000	18680000		10144000
	44703000			64976000	29512000		14805000	14619000
5.05E+08	4.48E+08	6.4E+08	8.49E+08	7.21E+08	5.97E+08	9.54E+08	7.61E+08	1.06E+09
	21421000							
5.39E+08	5.89E+08	6.49E+08	3.21E+08	3.93E+08	4.33E+08	8.37E+08	6.52E+08	4.97E+08
14206000		18733000			39287000	9654700	36496000	66618000
	18615000				18970000			
35981000	32525000	31843000	41379000	27065000	31052000	52373000	35586000	47178000
	70042000		97682000	93631000	89083000	1.09E+08		62822000
	48214000	50712000	77678000	27063000	52428000		29125000	
8.85E+08	9.89E+08	1.12E+09	6.79E+08	1.18E+09	1.64E+09	1.76E+09	2.41E+09	1.52E+09
	13554000			11682000	12170000			
62778000	62015000	87815000	70031000	50979000	39432000	56446000	58535000	78473000
60745000	51465000	1.78E+08	2.56E+08	2.67E+08	2.05E+08	1.42E+08	1.61E+08	1.85E+08
4.23E+09	3.12E+09	3.39E+09	2.29E+09	2.09E+09	2.33E+09	2.26E+09	2.39E+09	2.15E+09
	49804000	23977000		12312000	89370000		9739800	63153000
			2533800					
1.93E+08	1.63E+08	98179000	2.6E+08	3.21E+08	3.04E+08	3.75E+08	1.06E+08	3.97E+08
1.21E+08	1.29E+08	1.59E+08	97242000	1.3E+08	1.2E+08	33731000	40982000	71153000
23181000	45875000	33301000	30422000	72418000	54377000	95898000	1.12E+08	83641000
			29632000	9148700	10052000		4897600	6753100
			81344000	62172000	90571000	28209000		
			11946000	12209000	13002000	13026000		
			70873000			30833000		
31830000	30465000	58803000	24733000	27465000	32156000	38238000	45351000	58135000
37706000	30908000	47145000	55748000	49935000	40004000		41665000	48082000
1.96E+08	1.66E+08	2.7E+08	2.79E+08	1.77E+08	1.57E+08	2.22E+08		1.89E+08
3.29E+08	4.34E+08	3.55E+08	2.88E+08	2.47E+08	3.18E+08	1.74E+08	2.36E+08	2.01E+08
80033000	2.62E+08	2.26E+08	1.96E+08	5.56E+08	5.19E+08	3.82E+08	1.68E+09	6.4E+08
3.37E+08	2.26E+08	3.12E+08	2.19E+08	1.53E+08	1.36E+08	2.39E+08	1.51E+08	2.18E+08

			26306000	25493000	21682000	26460000	30777000	35880000
	30395000	17729000	35703000	25738000	29187000	40414000		29836000
		46309000	19961000	54746000	55505000			1.09E+08
	13832000	22834000		77680000	84892000	53658000	44579000	42150000
	18840000			35099000	19190000			
1.79E+08			1.44E+08		1.54E+08	1.46E+08		
		2287600	2122200	1417300	1888000	1764300	9541500	2783300
			8527500			6885300		
1.3E+08	97889000	1.82E+08	80831000	69498000	52218000	63765000	79482000	76752000
2.04E+09	1.64E+09	2.43E+09	4.55E+09	4.11E+09	3.62E+09	4.74E+08	9.43E+08	8.15E+08
	23476000		58694000	1.02E+08	69342000	56261000	21959000	57548000
7.44E+08	1.1E+09	7.84E+08	7.35E+08	7.18E+08	5.46E+08	8E+08	6.02E+08	7.19E+08
57906000	67184000	79600000	40621000	47283000	53570000	1.04E+08	77911000	45736000
93766000	57082000	68986000	92409000	53854000	80149000	68880000	1.2E+08	1.02E+08
46778000	1.12E+08	2.73E+08	37542000	47920000	57050000	29847000	27346000	1.37E+08
63841000	82801000	1.18E+08	28507000	51485000	17709000	35055000	29739000	55129000
47386000	50123000	52604000	1.8E+08	1.97E+08	1.6E+08	1.88E+08	1.66E+08	2.91E+08
1.54E+08	1.81E+08	1.33E+08	3.35E+08	2.99E+08	2.71E+08	4.07E+08	3.02E+08	4.18E+08
			87918000	64970000	92916000	83902000	32853000	30925000
89673000	78851000	86512000	93086000	76511000	74007000	83960000	90915000	1.15E+08
19210000	38503000		38169000	1.07E+08	88354000	56859000		46028000
		32088000						
4.23E+08	3.17E+08	4.74E+08	3.46E+08	2.38E+08	2.35E+08	3.06E+08	2.13E+08	1.83E+08
17936000	14521000	18904000	20659000	17758000	14898000	18545000	18662000	22740000
1.24E+08	1.63E+08	1.34E+08	82010000	66868000	49423000	1.63E+08	2.62E+08	2.02E+08
42543000		55223000		14659000				
62285000	43147000	79006000			88047000	1.47E+08	1.39E+08	1.53E+08
1.34E+09	8.77E+08	1.21E+09	4.21E+08	70123000	4.35E+08		4.2E+08	3.12E+08
4.54E+08	5.53E+08	5.51E+08		4.3E+08			3.52E+08	
2.01E+08	14394000	1.92E+08	1.31E+08	1.87E+08	1.85E+08			94112000
3.58E+08								
83292000		1.1E+08	1.43E+08	12732000	90172000	96658000	1.15E+08	1.22E+08
9340700		15864000	17418000	17612000	13715000	36580000	34507000	34974000
6.72E+08	6.18E+08	7.39E+08	6.61E+08	4.86E+08	4.17E+08	5.93E+08	5.64E+08	1.21E+08
	49641000		25508000	90229000	1.19E+08	48641000	33663000	
43142000	40160000	48207000	56794000	49640000	40157000	68694000	59423000	66386000
3.39E+08	3.3E+08	2.86E+08	1.75E+08	1.97E+08	1.92E+08	2.08E+08	1.81E+08	2.03E+08
1.29E+08								
			1607100		683100		3670000	
58038000	53446000	73664000	49767000	37183000	33440000	31598000	29436000	36894000
99272000	1.64E+08	74755000	63713000	26916000			43624000	30929000
						1.7E+08	2.62E+08	1.72E+08
6.66E+08	8.41E+08	5.74E+08	3.85E+08	4.42E+08	4.34E+08	7.31E+08	2.53E+08	5.49E+08

						5228600	3560600	
29779000	33359000	39495000	46949000		30893000	40736000	39758000	41678000
		24537000	36183000	32074000	33866000	1.21E+08	83711000	70299000
11733000	23483000	20652000	21313000	36538000	65305000	22229000	38136000	70343000
2.01E+08	1.76E+08	2.99E+08	2.33E+08	2.23E+08	1.76E+08	1.88E+08	2.13E+08	2.15E+08
		1.96E+08	1.34E+08			1.63E+08	1.73E+08	1.93E+08
		45336000			39494000	68044000	29653000	66182000
	19266000		35103000	42901000	68898000	62935000	67011000	44183000
7261500							6261200	
	41332000			29151000		39977000	26222000	
26351000		29493000	37612000	31986000	34166000	1.01E+08	86652000	37696000
		14025000	13839000	20001000	30532000	24564000		18495000
23069000			21286000	22177000		38446000	22588000	27605000
				1.4E+08	1.32E+08	1.38E+08	1.23E+08	1.25E+08
	10312000		27511000	10569000	41262000		53174000	53346000
89664000	1.21E+08	1.4E+08	80634000	1.21E+08	1.29E+08	73433000	1.51E+08	2E+08
		12218000			6767000			7809300
	37538000		40449000	84945000	1.06E+08	49070000		
49242000	44937000	64249000	66599000	81512000	1.01E+08	2.51E+08	2.15E+08	1.23E+08
5.21E+08	5.01E+08	5.42E+08	6.84E+08	6.11E+08	5.48E+08	6.31E+08	7.95E+08	7.51E+08
9874800	5502900	6169900	10375000	4047500	5043600	3563000	27732000	6914900
	1.12E+08		1.05E+08	90228000	1.05E+08	1.14E+08	1.23E+08	1.15E+08
1.09E+08	1.79E+08	94029000	97544000	1.34E+08	1.85E+08	1.38E+08	60369000	
8.09E+08	2.05E+09	1.15E+09	1.33E+09	2.23E+09	2.19E+09	1.26E+09	6.73E+08	1.1E+09
36103000	23494000		61095000	76993000	64068000	26277000	19859000	40598000
1.14E+08	1.06E+08	1.21E+08	1.02E+08	1.16E+08	77906000	1.01E+08	1.39E+08	1.04E+08
1.6E+08	2.18E+08	2.91E+08	1.97E+08	1.97E+08	1.4E+08	69278000	1.87E+08	1.84E+08
	16935000			18335000	12014000		18020000	21711000
	91539000			86412000	80524000	65089000	81527000	89235000
3.87E+08	9.64E+08	9.62E+08	4.12E+08	6.59E+08	6.03E+08	4.59E+08	1.44E+09	6.32E+08
40124000	38712000			27739000	22138000	40428000	48065000	26283000
				39595000		26257000		19907000
				10751000	9547400	12224000		
64327000	59472000	40724000	17771000	20370000	28001000	53701000	39621000	27979000
				1.12E+08				1.07E+08
16373000	21893000	26251000		25705000				26198000
6441800	5063500		47092000	24957000	36963000	11140000	12142000	22023000
			1.06E+08	83926000	77441000			
		27978000		40382000	23859000	32489000		36662000
36518000	1.11E+08	1.31E+08	15099000	55064000	69640000	15570000		44014000
2.06E+08	2.24E+08	1.68E+08	1.47E+08	1.77E+08	1.7E+08	2.21E+08	2.06E+08	2.77E+08

1.06E+08	1.63E+08		1.84E+08	1.9E+08	2.1E+08	1.77E+08	1.38E+08	1.87E+08
1.29E+08	1.05E+08	1.17E+08	70580000	1.01E+08	72063000	55568000	52175000	52685000
				1.57E+08	1.6E+08	67064000	32363000	59167000
3650000	2342400	2417000	3285600	2079300	3706700	2803500	7334000	2030900
1.97E+08	1.11E+08	1.51E+08	1.01E+08	85321000				
4.37E+08	5.05E+08	6.93E+08	3.81E+08	3.72E+08	3.35E+08	2.07E+08	5.17E+08	3.71E+08
4.79E+08	8.05E+08	1.22E+09	6.65E+08	7.86E+08	6.9E+08	5.64E+08	1.47E+09	1.03E+09
59988000		84870000					73885000	
3.77E+08	1.92E+08	4.66E+08	99437000	85283000	68644000	22659000	27178000	15538000
3.3E+08	1.81E+08	2.37E+08	2.34E+08	1.97E+08	1.57E+08	1.82E+08	2.39E+08	2.36E+08
				23729000		23529000		
8.87E+08	8.73E+08	1.48E+09	9.23E+08	7.33E+08	6.15E+08	1.08E+09	1.16E+09	8.97E+08
30924000	53820000	33201000	76475000	82062000	61870000	96936000	79381000	96891000
1.35E+08	2.2E+08	1.91E+08	3.12E+08	4.42E+08	4.71E+08	3.81E+08	2.05E+08	2.99E+08
63487000	45686000	56612000	65362000	90895000	89790000			
1.67E+08	2.74E+08	1.9E+08			4.17E+08	7.45E+08	5.8E+08	7.16E+08
1.46E+08	1.77E+08	1.59E+08	87755000	1E+08	1.6E+08	69978000	1.89E+08	25485000
	4.42E+08		29067000	2.99E+08	15230000	2.81E+08	2.06E+08	2.16E+08
83478000	84385000	84920000	41671000	52916000	43662000	46404000	54739000	47149000
71523000	59839000		33206000	39921000	39458000	87176000	79884000	52257000
1.37E+08	96628000	1.06E+08	77166000	52114000	64548000	91841000	63191000	64316000
1.96E+08	2.14E+08		3.24E+08	1.21E+08	1.33E+08	2.5E+08	2.06E+08	1.4E+08
					16380000			
2.16E+08	2.89E+08	2.52E+08	4.17E+08	4.98E+08	3.75E+08	5.47E+08	6.47E+08	7.76E+08
1.83E+08	2.34E+08	1.83E+08	87469000	1.15E+08	94718000	43777000	21455000	28161000
57667000	37486000	3.05E+08	24972000	33549000	23181000	26761000	26817000	31121000
1.79E+08	2.93E+08	94548000	1.5E+08	2.27E+08	2.14E+08	1.68E+08	92147000	1.22E+08
33402000	40891000					48788000	47038000	50554000
57454000	87785000	66544000	2.16E+08			2.84E+08	2.51E+08	2.5E+08
	44364000	52703000	27848000	42638000	33416000		30496000	29605000
5.8E+08	5.55E+08	7.57E+08	8.68E+08	6.17E+08	5.64E+08	6.85E+08	7.33E+08	7.44E+08
1.41E+08	1.2E+08	1.56E+08	92448000	78990000	77654000	68314000	75014000	89779000
1.62E+08	1.39E+08	2.23E+08	1.07E+08	1.08E+08	88267000	88511000	72970000	51574000
	71835000	82397000	56887000	57397000	60426000	67420000	75375000	69821000
39684000			30737000	37691000	39680000	28781000	24780000	35781000
			26985000	37736000	30172000			
26053000	29539000	43882000	73885000	1.36E+08	89148000		43831000	49995000
					9658900	8888700		
1.02E+08	87580000	77138000	35115000	46950000	58023000	54682000	30222000	51921000
			16549000	29508000	27524000			
	2.58E+08	3.92E+08		1.66E+08	1.46E+08	4.91E+08	2.77E+08	4.91E+08
				5.52E+08				
4.37E+08	4.62E+08		9.21E+08	8.78E+08	8.42E+08	7.2E+08	2.17E+08	1.6E+08
			2232400		3336700	4449200	14637000	10844000
22008000	41910000	45500000	96136000	66268000	76675000	1.25E+08	80919000	1.23E+08

63925000	82743000	1.12E+08	87072000	54012000	51073000	77054000	89884000	73089000
5.82E+08	4.74E+08	5.04E+08	6.49E+08	5.12E+08	4.34E+08	5.24E+08	6.28E+08	5.83E+08
64806000			84466000	69761000	63784000			43157000
				61184000	13500000	29442000		
					23264000			
								14290000
1.05E+08		1.15E+08						94252000
31278000		33729000	43020000		44264000			
13446000	47325000	10283000	69211000	16807000	53443000	54797000	18714000	37001000
1.83E+08	2.32E+08	3.28E+08	1.06E+08	1.38E+08	1.06E+08	1.17E+08	1.02E+08	97011000
3931900		870130	493780		499450		2767900	
			44643000	72558000	89597000			24560000
			34142000	1.04E+08	88959000	37251000		27313000
43134000	45864000	42903000	37144000	34139000	38305000	35826000	11501000	38470000
					25071000			
1.5E+08	1.34E+08	1.61E+08	66300000	67332000	1.84E+08	2.41E+08	2.92E+08	3.02E+08
		8503900						
				55797000				
56157000	99112000	16262000	11960000	13364000	22080000	25881000	76126000	16051000
1.68E+09	1.17E+09	2.1E+09	6.85E+08	6.71E+08	5.02E+08	2.08E+08	2.04E+08	3.45E+08
			15151000				14642000	
55833000	29966000		4.05E+08	2.81E+08	3.41E+08	2.34E+08	99772000	43505000
3.34E+09	3.4E+09	3.73E+09	5.79E+09	5.9E+09	5.22E+09	2.73E+09	2.38E+09	5.13E+09
			1.17E+08	1.05E+08	50888000	45316000		10212000
61083000				66406000	10301000			73869000
50020000	1E+08	1.1E+08		67801000	72596000			2.08E+08
			60202000	1.05E+08	68957000	81880000		29820000
1.2E+08	82522000	1.07E+08	61005000	42779000	31163000	27257000	41342000	44587000
19305000	16322000		18813000	29645000	23955000		34282000	30358000
		52940000	6.89E+08	9.92E+08	38978000	79785000	83978000	
	24694000		26276000	27864000	29471000	15222000		
1.47E+08	2.61E+08	1.86E+08	1.05E+08	1.91E+08	1.74E+08	1.62E+08	1.21E+08	1.24E+08
	36705000			31895000	40432000	14306000		
				1.44E+08				
2.5E+08	1.36E+08	2.58E+08	1.07E+08	61096000	70492000	76819000	64727000	69946000
54314000	54484000	71904000	28389000	36928000	55125000	16199000	74841000	77708000
2.48E+08	2.72E+08	2.93E+08	4.5E+08	4.58E+08	4.12E+08	3.49E+08	4.78E+08	4.25E+08
			16090000		13348000	21225000	18823000	19748000
6.42E+08	5.54E+08	8.33E+08	7.22E+08	4.95E+08	4.32E+08	6.67E+08	6.29E+08	5.79E+08
68600000	66039000	72672000	1.88E+08	1.51E+08	1.3E+08	2.19E+08	3.07E+08	3.28E+08
19219000	13895000	31437000		16190000	24474000	23343000	20802000	21233000
1.08E+09	8.71E+08	1.08E+09	5.44E+08	2.3E+08	3.57E+08	2.13E+08	3.36E+08	2.1E+08
			29468000	2.44E+08	2.41E+08	1.13E+08		1.18E+08
			67972000	2.3E+08	1.89E+08	1.69E+08		80167000
1.12E+08	1.13E+08	1.62E+08	21905000	73444000	76058000	13026000	83779000	85059000
9.45E+08	8.68E+08	1.05E+09	9.42E+08	8.26E+08	7.2E+08	9.75E+08	1.03E+09	1.11E+09

2900800	595800	952140	814000	656700	975120	850160	5228300	888750
93260000	1.27E+08		6.54E+08	6.49E+08	6.42E+08	1.9E+08	99008000	68102000
1.42E+08	1.08E+08	1.46E+08	1.4E+08	1.03E+08	83791000	1.17E+08	1.13E+08	1.05E+08
1.52E+08	1.51E+08	1.6E+08	1.17E+08	1.18E+08	1.22E+08	1.15E+08	1.06E+08	1.15E+08
1.56E+08	1.2E+08		35374000				65562000	
45662000	38038000	51396000	34201000		31325000		26474000	37382000
30271000	42778000	41219000	32666000	30306000	20798000	27588000	23156000	28843000
	25983000		25450000	61154000	42287000	25451000	27873000	29942000
1.93E+08	3.28E+08	3.03E+08	3.72E+08	2.52E+08	1.84E+08	1.38E+08	1.49E+08	1.88E+08
1.99E+08	3.14E+08	3.09E+08	1.41E+08	2.76E+08	2.3E+08			43127000
1.09E+09	9.78E+08	1.26E+09	1.37E+09	1.13E+09	9.19E+08	1.13E+09	1.18E+09	1.33E+09
1.1E+08	95591000	1.01E+08	66400000	57289000	59016000	1.09E+08	86429000	53089000
1.12E+08	81861000	1.15E+08	1.17E+08	84884000	74980000	1.25E+08	1.32E+08	1.11E+08
			11655000	16124000	11429000			
					53910000			
	25996000	25430000	33778000	37858000	39998000	43118000	72084000	57547000
	17475000		20412000	14456000	18634000	16401000		
2.36E+08	2.24E+08	1.4E+08	2.7E+08	3.23E+08	2.91E+08	2.53E+08	1.26E+08	2.91E+08
				24898000	49303000	13031000		
19253000	28721000	21932000			14627000	25796000	41468000	
		9246400			6057000			
6.03E+08	5.17E+08	5.79E+08	5.82E+08	4.94E+08	4.21E+08	6.31E+08	6.3E+08	6.52E+08
	24511000			15283000	22149000			
				36598000				
	1.09E+08			73647000	1.11E+08		12844000	
		20156000	31557000	11143000				
					8105200			
16880000						43066000	32519000	
1.02E+08	1.62E+08	3.12E+08	98024000	2.44E+08	3.87E+08	1.66E+08	1.63E+08	3.08E+08
1.84E+09	2.19E+09	1.69E+09	1.73E+09	1.65E+09	1.65E+09	1.18E+09	1.29E+09	1.07E+09
50335000	49457000	56975000	56084000	70432000	54089000		20277000	27930000
								11154000
	29297000		30233000	62468000	34198000	77363000		
5.32E+08	6.96E+08	6.07E+08	6.04E+08	1.08E+09	8.58E+08	3.87E+08	3.89E+08	6.05E+08
				31205000	15626000			
2622200		2904800	2627500	1821400	2845600			3654100
			2747900		2279100	2195700	6712600	6862600
					5912400		6496500	
47421000	51364000	31298000	48262000	69482000	80056000	65270000	42407000	81162000
80964000	1.2E+08	1.03E+08	60497000	1.18E+08	1.12E+08	73557000	1E+08	1.33E+08
		39539000						

1.64E+08	2.11E+08	1.86E+08	1.8E+08	2.37E+08	2.38E+08	3.32E+08	3.12E+08	3.52E+08
					6.51E+08	6.68E+08	6.54E+08	7.63E+08
78365000	1.79E+08	77683000	22195000	3.36E+08	2.54E+08	2.03E+08	1.32E+08	1.81E+08
							5402800	
1.71E+08	1.49E+08	2.14E+08	2.15E+08	1.26E+08	1.11E+08	1.71E+08	2.09E+08	1.87E+08
23835000	16301000	18620000	13656000	10720000	15373000	21054000	48695000	28980000
4.63E+08	6.54E+08	5.42E+08	7.85E+08	9.8E+08	9.33E+08	6.62E+08	4.91E+08	6.93E+08
	12392000				11481000			16217000
	1.17E+08	1.04E+08	47774000	62840000	76525000			27858000
28620000	63832000	40661000	1.11E+08	4.39E+08	2.12E+08	39243000	60791000	45012000
			16765000	23761000	23784000	25934000		
4.05E+08	1.42E+09	2.23E+08	1.76E+09	1.99E+09	2.2E+09	1.34E+09	1.01E+09	1.02E+09
				80701000	45565000			
		80034000		46420000	72948000		50492000	
1.34E+08		1.12E+08						
1.08E+08	2.54E+08	2.12E+08	2.89E+08	2.68E+08	2.62E+08	2.91E+08	1.66E+08	2.22E+08
	17263000		31731000	62928000	42361000	26128000		32604000
			75035000	2.25E+08	1.56E+08			
	30068000		20044000	30269000	22426000	23880000	13981000	21047000
55821000	1.8E+08	78686000	76839000	88131000	87433000			97540000
9871800	9773100	9022600	14056000	13182000	26039000	18832000	31978000	14956000
1.27E+08	1.67E+08	2.46E+08	2.23E+08	1.56E+08	1.34E+08	1.43E+08	1.1E+08	1.51E+08
			19145000	23087000	17294000			17390000
3.4E+08	3.37E+08	2.67E+08	2.65E+08	2.52E+08	2.45E+08	2.77E+08	2.86E+08	3.03E+08
	24191000	32284000			29304000			
		1.32E+08	44089000	50084000	71897000	83554000		83442000
10128000	6868200	5667800	8676700	3300000	4628100	5563000	26090000	7659200
1.83E+09	1.68E+09	1.7E+09	1.81E+09	1.6E+09	1.66E+09	2.22E+09	1.84E+09	2.3E+09
5.68E+08	2.63E+08	2.52E+08	1.63E+09	1.05E+09	1.86E+09	1.31E+09	3.09E+09	1.92E+09
			3.2E+08	3.5E+08	3.08E+08	3.03E+08	2.87E+08	3.66E+08
69269000	68088000	54808000	1.04E+08	1.07E+08	1.05E+08	1.35E+08	75460000	84263000
2.78E+08	2.22E+08	3.24E+08		1.06E+08				
6.91E+08	8.03E+08	7.56E+08	4.85E+08	5.62E+08	5.34E+08	3.72E+08	2.01E+08	3.33E+08
				1.72E+08	1.52E+08		1.43E+08	1.28E+08
1.29E+08	1.45E+08	1.17E+08	73731000	1.05E+08	93667000	39362000	25748000	39771000
4.37E+09	4.32E+09	4.38E+09	4.09E+09	4.35E+09	4.23E+09	4.83E+09	5.51E+09	5.23E+09
44884000	62071000	37459000	39002000	48366000	49421000	48122000	47751000	48604000
1.41E+08	1.08E+08	1.13E+08	79793000	55379000	56502000	71257000	74158000	67319000
	48282000	41271000	45315000	50231000	62437000	63959000	32544000	53038000
10385000		11593000	21297000	59487000		26068000		35693000
			3337800		3304700		14611000	5661600
				21932000				
1.37E+08	3.86E+08	27263000	2.85E+08	3.39E+08	3.59E+08	2.6E+08	1.28E+08	2.31E+08
74484000	1.18E+08	95116000	77137000	1.69E+08	1.17E+08	1.28E+08	70496000	1.11E+08
1.79E+08	1.44E+08	1.26E+08	1.91E+08	1.87E+08	1.97E+08	1.36E+08		14123000

				75758000	7526700			
	46904000	40786000	1.18E+08	75975000	17037000			61958000
56856000	50041000		53777000	37949000	35218000			
46508000	45515000	43939000	2.97E+08	2.12E+08	2.15E+08	2.91E+08	2.72E+08	4.19E+08
2.74E+08	2.22E+08	1.95E+08	76039000	90953000	1.97E+08	3.76E+08	3.21E+08	1.2E+08
2.42E+08	2.56E+08	2.25E+08	1.46E+08	1.8E+08	2.46E+08	2.76E+08	2.93E+08	2.07E+08
4319100					3771400	4195800	6367800	4462100
79429000	97254000	1.06E+08	69027000	75893000	46798000			48809000
	13457000	81558000	56892000	13186000	12709000	9228700	6405800	6932500
1.48E+08	1.41E+08			1.63E+08	1.33E+08	2.3E+08	2.31E+08	2.71E+08
32781000	30963000				27990000	19890000		
43537000	38119000	78409000	47085000	49523000	57307000	49825000	82643000	74490000
		34153000						82882000
1.91E+08	1.57E+08	1.95E+08	1.02E+08	66638000	98521000	72370000	1.53E+08	1.01E+08
				4068100				
4748500			7326300	6971900	10196000		31464000	
	22601000			31481000	36163000	22853000	22713000	
88078000		1.05E+08	2.88E+08	3.25E+08	3.65E+08	56841000	2.07E+08	28331000
4933700	7821200	6612100	26442000	18369000	21178000	25628000	27485000	31101000
2.65E+08	2.54E+08	1.47E+08	4.65E+08	2.14E+08	2.01E+08	4.13E+08	4.46E+08	3.8E+08
					21849000	25890000		9659900
55148000	63797000	61059000		19809000	20024000	22607000	22033000	21693000
			3.43E+08	5.25E+08	3.74E+08	2.73E+08		1.17E+08
82900000	60606000	16214000		51065000			83838000	85144000
1.12E+08	1.16E+08	1.3E+08	1.36E+08	1.21E+08	1.04E+08	1.67E+08	1.9E+08	1.94E+08
6.48E+08	6.59E+08	5.88E+08	3.84E+08	4.12E+08	4.3E+08	3.33E+08	3.82E+08	4.6E+08
7.68E+08	5.7E+08	8.89E+08	8.04E+08	5.21E+08	3.28E+08	5.83E+08	2.36E+08	2.7E+08
1.71E+09	2.44E+09	6.16E+08	9.53E+08	1.4E+09	1.96E+09	2.69E+09	1.61E+09	1.22E+09
			37370000	17346000		23478000		18726000
1.36E+08	2.25E+08	1.07E+08	2.32E+08	3.98E+08	2.91E+08	2.51E+08	1.72E+08	1.32E+08
	69242000		1.29E+08	1E+08	1.49E+08	99316000	81266000	96851000
99748000		1.29E+08	78702000	85102000	1.65E+08	2.59E+08	1.82E+08	2.05E+08
61661000	77045000	60384000	58221000	68351000	56187000	54813000	59832000	51055000
					72434000			
				7964300	37466000			
24852000	19424000	25882000			50616000	56094000	99245000	86995000
	6649400		12881000	5074300	7585200	6750600	19833000	10278000
3.17E+08	2.1E+08	4.6E+08	1.31E+08	1.45E+08	1.11E+08	76570000	85298000	64893000
1.38E+08	2.68E+08	1.56E+08	1.54E+08	1.82E+08	1.78E+08	1.91E+08	1.32E+08	1.65E+08
				14788000				
28173000					6412900			
28893000	40505000	45592000	41330000	56479000	52760000	28775000	38464000	45723000
			34703000	52502000	35195000	68008000	28528000	37575000

1.34E+08	2.5E+08	1.29E+08	3.23E+08	4.38E+08	4.62E+08	2.66E+08	1.58E+08	20618000
				13971000				
				14126000	12141000		10978000	
	13692000			11578000		10069000	11150000	
31664000	55751000	43766000	40391000	95295000	70285000	74666000	69589000	89658000
				21605000	16083000			
				51374000	48011000	31983000	31315000	54583000
				63962000	90932000	1.4E+08	67600000	
95435000	2.21E+08	45915000	2.07E+08	4.1E+08	2.63E+08	2.25E+08	95105000	2.04E+08
	21777000	27199000	24985000	48910000	17690000	38433000	67844000	25320000
					22000000	15349000		
	1.15E+08		78892000	79271000	81426000	45909000	17426000	
1.78E+08	2.26E+08	2.69E+08	2.68E+08	3.52E+08	3.41E+08	3.38E+08	3.48E+08	4.57E+08
9.5E+08	2.17E+09	8.44E+08	2.63E+09	4.6E+09	3.77E+09	2.94E+09	1.96E+09	3.33E+09
16960000	50793000	23456000	70359000	71568000	55932000	80158000		96741000
								25678000
	29230000			34983000	32094000	1.02E+08	1E+08	67146000
48506000	51114000		54879000	42672000	52223000	63395000	48923000	60839000
			47319000			21540000		
51312000	45583000		51895000	52275000	45955000	52800000	45232000	72534000
1.43E+08	2.07E+08	91979000	2.94E+08	2.09E+08	2E+08	1.9E+08	1.31E+08	1.67E+08
								16576000
15465000	25027000		13808000	15808000	11253000			
	2485200	3450500	3549000	3515200	4963700		6905400	3867500
		27161000	25007000	25132000	18406000		20367000	22972000
13754000	28803000	19272000	16544000	14714000	16291000			
	2.42E+08			3.57E+08		8765200	1.09E+08	2.43E+08
				46334000				
1.24E+08	2.12E+08	1.56E+08	3.77E+08	3.91E+08	4.03E+08	4.3E+08	4.15E+08	4.56E+08
	27488000		44826000	47809000	56371000	47247000		18990000
			1.14E+08	29257000	54739000	76894000	61307000	1.09E+08
1.67E+08	2.01E+08	2.85E+08	54237000	76833000	73024000			27863000
	15802000			7780400	13394000	7215900	9900800	
			2072700	451840			3875600	
32271000			79885000	41745000	55996000	80286000	78235000	1E+08
29406000	47466000							
66055000	28537000	72264000	46143000		19664000	39713000	36653000	31287000
	16225000	14994000						
				13876000	26305000			
				16243000				
	47701000		47228000	47398000			62813000	61892000

	15384000	13397000	11138000		15543000	16742000		15857000
6.15E+08	6.62E+08	6.91E+08	5.3E+08	5.3E+08	5.69E+08	9.49E+08	7.62E+08	6.73E+08
51697000	73452000	60281000	56121000		4.12E+08	4.43E+08		4.01E+08
55021000	73078000	41292000	58606000	74560000	72864000	83655000	17334000	75761000
			68198000	16725000	18997000			
			19362000					
97942000	1.43E+08	81089000	1.56E+08	1.89E+08	1.64E+08	1.01E+08	1.14E+08	1.32E+08
	9685000							
							16621000	
36313000	25993000	36459000	40661000	20335000		29217000	21550000	27339000
1.2E+08	74453000	93552000	44557000		42062000	10821000		
8.24E+08	6.97E+08	7.04E+08	3.75E+08	4.02E+08	3.03E+08	4.01E+08	4.75E+08	3.75E+08
1.29E+09	1.91E+09	1.59E+09	1.11E+09	1.26E+09	1.25E+09	5.35E+08	4.87E+08	5.22E+08
70496000	65347000	85368000	35122000	32996000	54734000	42421000	80633000	
3.34E+08	2.98E+08	1.24E+08	5.01E+08	5.88E+08		4.06E+08	2.22E+08	6.92E+08
		43590000	64178000	34645000	47146000	26380000	80338000	58143000
1.4E+08	1.31E+08	1.52E+08						
25794000				26139000	22877000	43144000	54424000	86680000
68432000	53487000	1.16E+08	59271000	56805000	57578000	33310000	34218000	31504000
41847000	22822000	33397000	15563000	9117400	11087000	27564000		14230000
	60238000	54622000	1.42E+08	1.82E+08	1.64E+08	1.29E+08	76315000	1.31E+08
9.24E+08	1.59E+09	1.91E+09	9.88E+08	1.61E+09	1.79E+09	2.23E+09	2.77E+09	2.07E+09
16668000	27021000	17880000	17181000	19339000		29699000		37194000
1.06E+09	1.06E+09	1.04E+09	6.33E+08	4.39E+08	4.05E+08	5.43E+08	5.15E+08	5.31E+08
			22981000			22427000		
3.32E+08	2.12E+08	3.4E+08	3.39E+08	2.39E+08	2.1E+08	3.41E+08	3.19E+08	3.13E+08
				8407000	10947000			
2.57E+08	3.16E+08	3.83E+08	3.71E+08	3.66E+08	2.98E+08	3.66E+08	3.03E+08	4.41E+08
2.24E+09	1.98E+09	2.14E+09	2.15E+09	2.07E+09	2.08E+09	1.92E+09	2.17E+09	2.31E+09
22021000					35701000	34754000		
29781000		43424000		60523000		33651000	26028000	40809000
52092000	36008000	49976000						
67657000	65647000	48022000	34626000	44935000	50771000	27551000	17386000	24470000
				15737000	11109000			
	17820000	27300000	22790000	40590000	31363000	20521000		35496000
				227190	404960			
	25440000			14772000	19922000			
60433000	1.04E+08	49942000	1.58E+08	2.48E+08	2.16E+08	1.84E+08	82153000	1.54E+08
1790000	789450	828310	2130500	776600	1150200	1793400	8325900	1872400
21460000	40724000	21630000	35107000	55840000	51719000	31449000	27949000	29625000
3.9E+08	93005000	3.09E+08	4.56E+08	2.98E+08	3.23E+08	1.85E+08	2.77E+08	4.65E+08
			15336000				17397000	16145000
				15514000	14004000			

	58851000	44672000	43440000	44304000	49502000	77593000	51042000	59618000
	3.37E+08	2.15E+08	3.27E+08	4.15E+08	4.11E+08	4.16E+08	2.59E+08	2.94E+08
		2.82E+08			2.75E+08			
			30104000					
48494000	1.3E+08	32645000	1.17E+08	1.52E+08	1.56E+08	74458000		79553000
		2.5E+08	5.03E+08	7.73E+08	8.94E+08	5.17E+08		4.39E+08
	96867000	1.62E+08	21189000	38727000	48796000			
					72515000	28010000	11590000	31901000
84089000	1.29E+08	1.07E+08	63131000	82359000	86708000	39443000	71625000	85184000
	21664000		38665000	49137000	38719000	38100000	27220000	28744000
	19172000		16008000	28945000	29940000	23633000	16040000	22657000
			49500000	34522000		21653000		11893000
				17325000	16564000		18196000	21610000
				24655000	21559000			21012000
46405000	2.07E+08	80661000	2.31E+08	2.48E+08	2.9E+08	2.23E+08	99839000	1.83E+08
					17815000	7022700		
98689000	59022000		7.35E+08	3.14E+08	4.51E+08	7.32E+08	6.34E+08	6.41E+08
2.79E+09	1.35E+09	3.88E+08	6.48E+09	3.17E+09	2.85E+09	4.83E+09	5.09E+09	98094000
	12812000	17215000	54172000	29025000				
1.48E+09	1.5E+09	1.61E+09	6.42E+08	5.49E+08	5.26E+08	4.17E+08	3.71E+08	5.03E+08
6.68E+08	6.98E+08	7.44E+08	5.12E+08	5.27E+08	4.99E+08	4.62E+08	4.54E+08	5.09E+08
	27114000	30858000		21567000	24959000			
	50626000		25171000	45719000	44767000	44465000	30348000	24933000
			1.12E+08	75308000	73081000	50110000	39591000	23259000
36207000	48731000	43097000	35621000	43852000	37996000	20381000	21841000	16233000
			21096000	18357000	16988000			17161000
			15819000	17874000	17517000		24865000	27252000
	1.29E+08			87585000				
2.93E+08	2.43E+08	1.49E+08	1.55E+08	2.49E+08	3.24E+08	2.06E+08	2.34E+08	2.81E+08
1.12E+09	1.94E+09	1.25E+09	4.34E+09	5.24E+09	5.17E+09	6.35E+09	3.48E+09	3.83E+09
1.04E+09	8.06E+08	6.28E+08		29971000	4.74E+08	7.98E+08	6.62E+08	25480000
8847600	1099100	1411400		556530	850230		2148600	396480
		16196000						
	72328000	40293000	42823000	31539000	34175000	35405000	49580000	33395000
1.52E+08	1.07E+08	93528000	30403000	47383000	85708000	74621000	45618000	31411000
88336000	1.23E+08	98404000	63135000	74106000	1.23E+08	1.97E+08	1.43E+08	1.28E+08
			50603000	23054000	56042000			
		31083000	28995000	40254000	56217000		20436000	
	26922000		30184000	1.74E+08	1.24E+08	77741000	19832000	
2.61E+08	3.63E+08		3.14E+08	3.27E+08	2.83E+08			1.93E+08
	31934000		22838000	54140000				
				26769000	20348000			
60325000	67784000	66913000	68913000	73213000	63051000	45437000	37623000	42220000
1.61E+08	1.96E+08		1.93E+08	2.05E+08	1.71E+08	1.75E+08	1.41E+08	1.93E+08
22345000	16656000	16877000	34451000	25511000	39041000	31066000	71307000	39303000

	56888000			42141000	40286000	53825000		
	22145000	33173000					25406000	
2.11E+09	2.29E+09	2.21E+09	2.48E+09	3.18E+09	2.52E+09	1.82E+09	2.23E+09	2.83E+09
1.52E+08	83049000	1.3E+08	1.52E+08	1.74E+08	1.36E+08	1.3E+08	57244000	1.05E+08
	42879000		46331000	43157000	69659000	56215000	53322000	41006000
45019000	62531000			42110000	54249000			
1.94E+08	2.73E+08	2.2E+08	2.01E+08	2.28E+08	2.36E+08			
			25914000	53894000	30739000	32637000	21553000	29279000
				74351000	80312000	1.06E+08	57925000	72114000
1.53E+08	2.06E+08	2.16E+08	2.25E+08	3.83E+08	3.34E+08	2.12E+08	1.47E+08	2.02E+08
			97129000	88978000	69242000	1.25E+08		
	54142000	61617000	1.07E+08	1.09E+08	1.51E+08	1.06E+08	76961000	95297000
	31540000		88600000	1.08E+08	49058000		19095000	8541000
	9713000	4794100		22822000	9086800		10612000	
28665000								20888000
	36930000	23241000	17081000	21937000	29826000	14707000	8966600	11565000
			32268000	32669000	59208000			
1.05E+08	1.38E+08	95540000	1.07E+08	1.56E+08	1.42E+08			
34290000	36362000	43540000		28122000	38008000	26749000		
54110000	1.51E+08	23510000	48840000	58070000	50157000	16431000		
								29757000
1.57E+08	1.32E+08	1.28E+08	71004000	79203000	1.02E+08	1.43E+08	1.81E+08	1.09E+08
1.14E+09	1.52E+09	1.64E+09	8.77E+08	1.49E+09	1.44E+09	2.65E+09	2.7E+09	4.08E+09
3E+08	2.71E+08	2.78E+08	2.51E+08	2.16E+08	2.42E+08	1.9E+08	2.65E+08	2.1E+08
2.2E+08	2.38E+08	2.37E+08	2.63E+08	2.44E+08	2.12E+08	1.04E+08	72345000	1.6E+08
				12962000	7969900	8676700		14818000
			7705400	9880500				
					24269000		22400000	19345000
94705000	79010000	1.06E+08	38106000	47642000	77505000	48565000	74578000	1.56E+08
3554500			1829600	1153900	1322200	2556000	11061000	2309500
80060000	1.64E+08	84323000	1.34E+08	1.35E+08	1.29E+08	1.71E+08	1.2E+08	1.27E+08
8.71E+09	5.32E+09	9.66E+09	6.77E+09	4.5E+09	4.5E+09	4E+09	4.78E+09	4.2E+09
	1.19E+08	1.11E+08	1.73E+08	1.29E+08	2.02E+08	1.71E+08	3.99E+08	2.17E+08
2.2E+08	2.66E+08	2.7E+08	3.93E+08	3.59E+08	3.63E+08	2.51E+08	3E+08	3.87E+08
	9199800			1.91E+08	29701000		5679900	7181000
				16701000	39699000			14405000
59660000	1.07E+08	66829000	81028000	1.03E+08	1.08E+08	92375000	64237000	81549000
						44945000		
78402000	1.46E+08	69002000	1.55E+08	2.07E+08	1.66E+08	61638000	56028000	90983000
8.5E+08	8.66E+08	1.01E+09	1.15E+09	1.04E+09	8.79E+08	9.86E+08	9.4E+08	1.11E+09
	20001000		40297000	46394000	54747000	26575000		

5.57E+08	7.86E+08	7.16E+08	8.16E+08	9.9E+08	1.11E+09	3.51E+08	4.29E+08	4.77E+08
21366000	33355000	47530000		41435000	70252000		63162000	
15551000					25243000			
39469000	60033000	88406000	68160000	83606000	11702000	77069000	70609000	84275000
	26037000			49595000	57015000	23029000		21184000
8679500	9655000	11316000	16388000	24883000	17490000	12921000	15901000	23928000
	12334000	63841000		11621000	97257000			20776000
			32175000	32827000	37242000			
5.36E+08	5.93E+08	6.06E+08	6.64E+08	5.01E+08	4.92E+08	6.34E+08	6.92E+08	6.86E+08
					14338000	14279000	9408800	
		23253000		15734000	13520000	24550000		24395000
	22311000			31137000	26942000	27184000		
1.14E+09	1.21E+09	8.66E+08	8.91E+08	9.97E+08	1.39E+09	7.43E+08	8.04E+08	1.02E+09
	14149000	16152000	7316700	11143000	10416000		9615300	
40641000	34224000	38816000	36182000	24466000		40684000		
54981000	1.08E+08	71725000	33150000	33853000	76324000	1.16E+08	77448000	89968000
	1.4E+08		13902000	26845000	22688000	38793000	38409000	77920000
			46177000	50377000	90430000	10613000		45057000
1.18E+08	1.02E+08	89030000	89399000	71442000	60538000	48278000	1.06E+08	74743000
1.77E+08	45206000		2.2E+09	1.13E+09	1.1E+09	1.27E+09	4.49E+08	4.03E+08
			2.26E+08	1.99E+08	1.25E+08	1.73E+08	2.18E+08	2.19E+08
3.55E+08	3.4E+08	2.84E+08	2.71E+08	2.82E+08	2.66E+08	2.85E+08	2.28E+08	3.1E+08
		41411000	33004000	1.68E+08	47762000	64361000	59042000	1.06E+08
5.79E+08	4.03E+08	4.59E+08	1.87E+08	1.76E+08	1.79E+08	1.86E+08	1.38E+08	1.19E+08
4.97E+08	3.75E+08	4.57E+08		2.93E+08	2.77E+08	3.12E+08	3.21E+08	3.05E+08
	17827000	23227000			36646000			
1.25E+08	79762000		3.39E+08	2.72E+08	2.96E+08	2.41E+08		
			35068000	18328000	25562000	9427400		10919000
					48910000	43710000		
7.34E+08	7.19E+08	7.84E+08	1.89E+08	16165000	64853000	20623000	1.93E+08	
46248000	71896000	15330000	1.17E+08	70283000	71580000	1.85E+08	40981000	51136000
4.71E+08	6.02E+08	9.05E+08	8932700	2.69E+09	7.8E+08	1.42E+09	1.3E+09	2.02E+09
					12082000	22288000		
1.59E+08	2.59E+08	1.65E+08	2.49E+08	4.15E+08	3.36E+08	4.93E+08	3.41E+08	2.85E+08
4.99E+09	4.8E+09	5.43E+09	6.41E+09	4.29E+09	6.21E+09	2.24E+09	2.04E+09	3.7E+09
			38909000	67996000	63933000		32000000	
	10658000							
					30023000			
38745000	37087000	29670000	55018000	38327000	56821000	48920000	1.42E+08	56316000
	47504000	34445000	32222000	42217000	55944000	78244000	72819000	54102000
3.4E+08	6.47E+08	5.93E+08	1.05E+09	1.4E+09	1.41E+09	8.19E+08	7.2E+08	7.86E+08
				1.97E+08	2.4E+08	76830000	27199000	
42185000	60234000	51157000	91244000	79701000	87802000	3.88E+08	3.78E+08	4.34E+08
20115000			7971400	13803000	7777500	15300000		13861000
11802000	33140000	13509000	49517000	1.8E+08	1.26E+08	1.43E+08	92256000	1.21E+08
36926000	47389000	37881000	40730000	32230000	29526000	27503000	27219000	24075000

		25490000	37032000			15343000	14427000	33047000
1.36E+08	1.17E+08	1.96E+08	2.73E+08	2.58E+08	2E+08	1.95E+08	2.8E+08	3.23E+08
1.27E+09	1.33E+09	1.61E+09	1.91E+09	1.66E+09	1.56E+09	2.11E+09	1.57E+09	2.07E+09
28711000	27053000		1.41E+08	75522000	85666000	1.19E+08	63974000	1.01E+08
2.92E+08	1.26E+08	29559000	38630000	18254000	2.05E+08	95070000	5.99E+08	44030000
	23266000		23061000	52430000	35574000			27584000
12592000	16156000	17655000	15287000	20678000	15827000	17022000	17768000	23808000
1.19E+09	1.68E+09	1.4E+09	1.01E+09	1.4E+09	1.54E+09	1.25E+09	1.32E+09	1.23E+09
	3.13E+08	1.2E+08	4.11E+08	5.36E+08	5.09E+08	4.28E+08	3.58E+08	4.31E+08
3.43E+08	2.41E+08	4.2E+08	3.5E+08	2.84E+08	2.46E+08	3.98E+08	4.54E+08	3.63E+08
	30225000				36213000		46105000	
25751000	15625000			45729000	46692000	35445000	49571000	
26248000		26113000	17824000		11856000			10342000
1.29E+08	1.25E+08	2.26E+08	29465000	3.24E+08	1.2E+08	1.69E+08	1.24E+08	2.32E+08
5.35E+08	4.72E+08	5.16E+08	4.99E+08	4.3E+08	3.56E+08	5.06E+08	5.61E+08	5.06E+08
			8477600	12787000	8267400	13125000	13609000	
73687000				70914000				
			25307000	37011000	42530000	53255000		
1.07E+08	93189000	1.11E+08	35523000	34193000	29209000			
52245000	46986000		2E+08	2.75E+08	3.87E+08	3.45E+08	85229000	40362000
			1.27E+08	1.15E+08	1.62E+08		33013000	
					8713200			
27060000	38566000		17705000	23206000	34347000	48009000		21710000
25741000	31572000	29710000	57787000	75305000	67816000	1.01E+08	70997000	96971000
3.16E+08	7.91E+08	1.63E+08	3.05E+08	4.63E+08	2.39E+08	1.48E+08	77754000	81916000
69830000		80591000		82608000		80949000	94497000	
			27863000	37960000	51163000	41195000	46014000	
					59577000	6476300		26152000
17926000	15328000	32557000	28799000	32621000	29711000	26995000	38088000	50438000
				34076000				
20029000	35290000			73259000	55200000			
			42131000	40677000		50911000	57633000	61102000
		11667000	27078000	22691000	13454000	29182000	35937000	29155000
								22017000
				32655000	39381000	18640000		
				15527000	16452000	35438000	20892000	17114000
			7403400	6863200	5282400			12546000
	11048000		57037000	93852000	85146000	65409000	28236000	59655000
2.08E+09	2.44E+09	2.04E+09	2.41E+09	2.27E+09	1.91E+09	4.93E+09	4.9E+09	6.15E+09
95811000	1.03E+08	1.08E+08	1.88E+08	1.36E+08	2.27E+08	1.93E+08	2.56E+08	1.51E+08
1.45E+08	1.54E+08	2.06E+08	2.03E+08	1.51E+08	1.21E+08	1.25E+08	1.24E+08	1.29E+08
	1.23E+08	39796000		1.08E+08	1.09E+08		20007000	45274000
27526000		22325000		14593000			11317000	

		29802000						
36066000	19323000	26880000	10684000		14067000		21129000	
	20589000				22181000			14922000
					12589000			
2.55E+08	2.3E+08	3.32E+08	1.97E+08	1.69E+08	1.43E+08	2.18E+08	2.13E+08	2.61E+08
47600000	44862000	45529000	57141000	50893000	55182000	58133000	71586000	67923000
	18671000			28458000	50242000	25046000	6726800	13025000
2.93E+08	2.62E+08	7.79E+08	3.32E+08	2.4E+08	2.26E+08	3.72E+08	3.39E+08	3.57E+08
				35662000		1.48E+08		
7.1E+08	5.16E+08	5.14E+08	4.3E+08	3.22E+08	3.05E+08	2.67E+08	2.89E+08	3.07E+08
99370000	65919000	1.17E+08	50865000	31756000	28974000	23324000	20649000	26859000
4.76E+08	5.24E+08	5.76E+08	5.52E+08	5.15E+08	4.41E+08	6.3E+08	6.59E+08	8.82E+08
					12701000			
1.83E+08	98990000	1.36E+08	1.1E+08	1.04E+08	93888000	69638000	96879000	1.5E+08
	1.64E+08	36698000		84345000	1.23E+08	39077000		
1.32E+09	1.7E+09	1.84E+09	1.59E+09	1.54E+09	1.56E+09	1.43E+09	1.97E+09	1.56E+09
		9475000	27372000	9357100	12271000	12144000	26017000	42943000
			14405000		16540000	18075000	26920000	19671000
2.23E+08	1.76E+08	1.92E+08	2.48E+08	3.51E+08	1.98E+08	4.88E+08	2.22E+08	4.9E+08
2.2E+08		1.5E+08						
3.26E+08	2.96E+08	3.3E+08	3.51E+08	3.07E+08	2.82E+08	3.45E+08	3.84E+08	3.87E+08
	22615000	28477000	43127000			40983000		36303000
1.98E+08	3.14E+08	2.18E+08	2.78E+08	3.32E+08	3.13E+08	1.15E+08	3.88E+08	5.02E+08
					13035000			
2.01E+08	1.92E+08	1.51E+08	1.36E+08	1.76E+08	2.46E+08	1.74E+08	1.4E+08	1.4E+08
3.74E+08	5.57E+08	4.6E+08	8.03E+08	8.12E+08	7.06E+08	4.88E+08	4.94E+08	6.67E+08
3.99E+08	4.67E+08	6.37E+08	4.41E+08	5.3E+08	4.73E+08	4.14E+08	5.47E+08	5.86E+08
2.18E+08	3.21E+08	2.41E+08	3.64E+08	5.3E+08	4.57E+08	2.46E+08	60118000	2.26E+08
50034000	31770000	29793000	26125000	38417000	34898000	29726000	37098000	38033000
	1994900	2626000		636740			4310700	1150800
				59973000		46602000		24348000
							15396000	17933000
90223000	1.12E+08	1.13E+08						
3.19E+08	3.58E+08	6.43E+08	1.43E+08	1.89E+08	1.93E+08	68547000	69532000	1.54E+08
	2276400				2809900		7985900	3241000
	46461000		18596000	33895000	50420000	24118000		
	39159000			42482000	25524000	33577000		28264000
			17308000	17320000	21562000	14178000		
20788000	20221000	22651000	37600000	28224000	35693000	52957000	72387000	46826000
			14352000					
			20248000	23147000	15161000	28352000	20019000	29151000
				8398200				5159300

46236000	36162000	84480000	15342000	15855000	22707000	6821000	9597900	
				31075000	24372000	29582000		
	65633000		70114000	72126000	78482000	1E+08	37446000	64150000
2.69E+08	2.61E+08	2.73E+08	77185000	1.19E+08	94784000	4.36E+08	2.42E+08	2.91E+08
1.06E+09	1.44E+09	1.63E+09	1.07E+09	1.28E+09	1.57E+09	8.39E+08	6.64E+08	7.14E+08
7.93E+08	5.17E+08	6.41E+08	1.06E+09	8.2E+08	7.42E+08	6.7E+08	7.42E+08	9.93E+08
1.86E+08	2.12E+08	2.47E+08	2.26E+08	1.93E+08	1.23E+08	1.18E+08	1.61E+08	1.57E+08
				22682000	24085000			
								5995500
80206000	76682000	81380000	79865000	97589000	92662000	85152000	1.06E+08	1.01E+08
2.08E+08	2.84E+08	2.47E+08	2.13E+08	3.7E+08	3.2E+08	1.3E+08	1.11E+08	1.79E+08
				25999000				
2.63E+08		1.41E+08	73676000	1.19E+08		68223000	73413000	61573000
34315000	43754000	40366000		22600000				
58954000	46968000	37786000			63695000		27474000	24367000
	22923000	19626000		13123000	21093000	20780000	17726000	12848000
2.48E+08	3.77E+08	1.42E+08	3.4E+08	1.94E+08	3.6E+08	5.66E+08	5.61E+08	4.24E+08
19890000	50895000				28961000	23184000		10786000
35660000	49550000	33196000	15475000	26574000	40414000	28380000	16664000	17111000
18401000	18917000				15500000			
					11073000		3740700	
			21471000		51410000		19364000	23036000
57392000	1.04E+08	98068000	58721000	1.06E+08	1.32E+08	2.84E+08	1.89E+08	2.58E+08
	14706000							
		80119000			59876000	64368000		52926000
			19611000	39831000	33592000	35511000		
	40872000							
8653600	19131000	19666000	22277000	19487000	21453000	34503000	31847000	38910000
1.07E+08	3.07E+08	1.33E+08	2.46E+08	2.07E+08	2.52E+08	1.23E+08	90372000	1.56E+08
			3340900	1403400	131040	3162100	18060000	1964400
3.08E+08	3.09E+08	2.67E+08	4.5E+08	6.01E+08	5.05E+08	8.7E+08	7.59E+08	8.07E+08
					7795200	9375100	8142500	
				49071000	47972000			66039000
88221000	84716000	1.45E+08		1.43E+08	1.95E+08	96572000	96453000	1.17E+08
1.02E+08	1.13E+08	44725000	42035000	54171000	1.21E+08		80206000	67549000
2.64E+08	2.99E+08	3.28E+08	8.26E+08	7.11E+08	7.26E+08	8.07E+08	7.3E+08	9.7E+08
				45547000	33935000	60698000		
78140000	1.33E+08	59341000	1.18E+08	97573000	1.04E+08	62099000	27950000	49763000
			246020				528740	
					56978000	74285000		
		12689000		27089000		25216000		
					35864000		34060000	
			5716600	6668300	10095000	6785000	21858000	9370900

15587000	18656000	17447000		16999000	13442000	14716000	10544000	16192000
31808000	46564000	46628000	1.44E+08	94874000	86317000	1.16E+08	1.32E+08	1.69E+08
				25226000	20615000	17470000		
4.29E+08	4.77E+08	6.17E+08	6.44E+08	8E+08	4.01E+08	5.52E+08	4.65E+08	5.84E+08
					21478000			
		30515000			15769000			
			9735700		5568600	6286100		6697100
							15008000	
1.12E+08	1.04E+08	80281000	1.36E+08	1E+08	1.23E+08	1.52E+08	2.47E+08	1.95E+08
1.45E+09	1.29E+09	1.26E+09	2.32E+09	2.01E+09	1.67E+09	2.29E+09	1.6E+09	2.04E+09
5.3E+08	5.69E+08	4.74E+08	1.93E+08	2.43E+08	3.06E+08	2.4E+08	1.58E+08	1.23E+08
	31315000	27302000	20290000	29786000	21997000	21709000		14277000
					13928000			
44978000		42089000	62838000	53866000	53359000	53130000	54135000	50136000
15495000	17873000	26113000	12986000	6511300				
17973000	27636000	31286000	13232000	16132000	24032000			
94961000	80447000	1.32E+08	87352000	82558000	75263000			83289000
1.16E+08	80666000	1.28E+08	50202000	54924000	47934000			
								24146000
	38497000		29266000	61398000	46368000	22916000	26717000	
1.51E+08	91770000	53249000	1.16E+08	1.08E+08	1.06E+08	53393000	41636000	49597000
1.09E+09	7.59E+08	9.28E+08	4.93E+08	3.93E+08	3.92E+08	3.97E+08	1.86E+08	1.53E+08
24206000	30813000			29503000				
37454000		24347000	8887400	11068000		12886000	12186000	12540000
2.13E+08	3.42E+08	2.78E+08	3.07E+08	4.55E+08	4.7E+08	3.29E+08	2.31E+08	3.65E+08
	9806000		28285000	12158000	17156000	23101000		12669000
1.28E+08	1.39E+08	1.07E+08	1.57E+08	1.96E+08	1.56E+08	1.89E+08	1.74E+08	1.74E+08
			19911000		20568000	24325000		
1.65E+08	1.76E+08	69046000	1.18E+08	23148000	92635000	1.14E+08	1.46E+08	70642000
	44090000			75960000				
	42617000		1E+08	1.59E+08	88717000	88857000	47858000	66250000
2.71E+08	4.08E+08	2.12E+08	3.08E+08	3.77E+08	3.11E+08	3.27E+08	2.44E+08	2.42E+08
				19900000	15594000			
21025000	46714000		51031000	76534000	59474000	65422000	50092000	77625000
94246000	1.32E+08	1.21E+08	4.28E+08	3.96E+08	6.64E+08	5.97E+08	9.78E+08	8.05E+08
95352000	1.46E+08	1.2E+08	1.17E+08	1.24E+08	1.17E+08	1.25E+08	81084000	1.23E+08
			47381000	35843000				
				62074000				
							11978000	
4.1E+08	7.86E+08	3.04E+08	3.92E+08	5.47E+08	1.49E+08	1.35E+08	2.88E+08	1.49E+08
				8091400	8687000			
	42803000			17310000	37726000	14851000		
39656000	68004000	93055000		14685000	59664000	9241500	45962000	34948000

9478700	18974000		28654000	33583000	41257000		38326000	42196000
1.44E+08	87144000	1.4E+08	52652000	64650000	49095000	53037000		45377000
5.03E+08	7.67E+08	6.14E+08	9.79E+08	1.14E+09	1.13E+09	9.52E+08	8.51E+08	1.01E+09
	18721000	27712000			15132000	24046000		
1.72E+08	1.97E+08	1.51E+08	2.7E+08	3.1E+08	3.61E+08	3.17E+08	2.01E+08	2.32E+08
						10179000		
							3.22E+08	
1.87E+08	1.5E+08	1.95E+08	1.33E+08	59095000	81984000	1.33E+08	1.65E+08	1.25E+08
			1.38E+08	1.55E+08	1.69E+08	1.07E+08	92705000	1.56E+08
			8078700	14101000	35210000		51971000	46393000
35558000	31754000	36434000	36195000	40053000	41026000	18248000	17780000	
				2259800				2459500
13818000			29327000	41028000	29742000	47515000		43567000
8.7E+08	7.72E+08	9.37E+08	1.53E+09	1.38E+09	1.28E+09	1.48E+09	1.37E+09	2.51E+08
16811000	16266000	17118000	81788000	73162000	80474000	94130000	1.19E+08	1.25E+08
			30083000		47585000			24085000
	53033000		61427000	65463000	55095000	81579000	38476000	49051000
		8361300	12118000	10187000		7853400	9639500	8648700
958700			9668000		2114300	6426500	6281700	2416700
		55087000	1.05E+08	1.14E+08	1.09E+08	89091000	55090000	88271000
			30474000	24878000	27423000	39477000		31952000
			3265100	2175600	32018000		19848000	18418000
1.51E+08	1.46E+08	1.43E+08	67556000	95685000	93380000	64468000	59358000	1.23E+08
				24469000	24447000			
	53293000	48862000	56279000	84145000	1.08E+08	44875000	44933000	44740000
	1971900	2315300	5295000	3428700	4559700	5415400	12372000	5965900
						22490000	28628000	32936000
				18244000	19149000	28096000	10869000	
97292000	82337000	61805000	41668000	52867000	71873000	97850000	77621000	72603000
				18024000				
63548000				20724000	21430000			
							29506000	
				39300000				
		92890000	80701000	1.05E+08		1.55E+08	1.39E+08	1.72E+08
					6238300			
3.52E+08	4.29E+08	3.9E+08			6.32E+08	9.82E+08		
	36730000	6950200	20729000		25002000			
69628000	1.42E+08	80632000	1.02E+08	1.24E+08	1.24E+08	1.3E+08	75600000	1.03E+08
9301600	5177400	6649000	9029100	12731000	22906000	37907000	43623000	22760000
	2.68E+08	41057000	6.77E+08	8.26E+08	8.26E+08	6.21E+08	3.65E+08	4.37E+08
		785470	1387100	556540	587480	813830	4129200	943320
16791000	27418000		45378000	16227000	74771000	37896000	25049000	
						3808400	7772200	4657200
	87793000	51206000				1.01E+08		

	1.19E+08	51999000				35599000		21885000
18129000	13050000	18426000	12510000	11337000	8200200	1.75E+08	19725000	2.01E+08
	54335000	46292000	72912000	48951000	53631000	91907000	96534000	1.16E+08
72323000	1.86E+08	79077000	77440000	1.75E+08	71053000	1.35E+08	1.61E+08	1.6E+08
54968000	87623000	62645000	50639000	92686000	72923000			37570000
	22315000	32510000		15441000	21405000			16360000
	57537000	54247000	44987000	49670000	53474000	25452000	96822000	28268000
25895000	13539000	1472600	57256000	23117000	35270000	23367000	69418000	46232000
1959600		315210	500150					
21193000			15173000	18065000	29654000			23302000
4.03E+08	7.99E+08	4.22E+08	5.61E+08	7.7E+08	9E+08	4.63E+08	3.48E+08	5.21E+08
					22885000			22913000
	58056000		44603000	64627000		27597000	29078000	46368000
								19709000
			20545000					
				82087000	82690000	74530000		
1.58E+08	1.73E+08	1.72E+08	1.03E+08	1.06E+08	1.26E+08	1.8E+08	6.13E+08	1.71E+08
1.73E+08	2.54E+08	1.77E+08	3.42E+08	3.19E+08	3.98E+08	3.47E+08	3.47E+08	3.58E+08
99974000	2.92E+08	8736100	3.63E+08	5.3E+08	4.05E+08	7.02E+08	2.16E+08	3.15E+08
						16091000	26635000	
13170000		35695000	18150000					17460000
1.44E+09	2.56E+09	1.72E+09	9.21E+08	6.21E+08	7.22E+08	1.19E+09	1.12E+09	9.71E+08
	51324000	36458000	84013000	50726000	52549000		29856000	54700000
			9106700	4956400	11662000	8230600	25739000	12874000
1.61E+08	1.93E+08	1.55E+08			1.29E+08	1.29E+08	72922000	
50553000	34999000	33332000	1.17E+09	9.99E+08	9.56E+08	6.57E+08	6.08E+08	9.43E+08
4.7E+09	5.08E+09	4.82E+09	4.97E+09	3.44E+09	4.3E+09	4.03E+09	4.08E+09	4.12E+09
					26810000	21711000		13281000
	4.68E+08		7.05E+08	1.64E+08	8.57E+08	9.97E+08	5.65E+08	5.84E+08
4146900		1912900	1877800	873330	1500900	1933200	12785000	1845900
1.23E+08	2.13E+08	1.31E+08	4.2E+08	3.87E+08	3.86E+08	6.47E+08	5.87E+08	7.08E+08
	537470	743530	600670		389680	396330	1447200	
			83793000	89877000	1.03E+08	1.15E+08	79055000	1.22E+08
30079000			27695000			17915000	11784000	
			2467500		882360	1952300	7236800	1940200
11154000	1022300	2499600	2477300			1894100	9980100	1706500
1.79E+08	1.95E+08	1.63E+08	2.23E+08	2.32E+08	2.26E+08	2.15E+08	1.98E+08	2.35E+08
	902160	1333200	1234700			953030	5774100	1430200
86370000	1.52E+08	36809000	1.44E+08	1.15E+08	1.06E+08	1.84E+08	1.45E+08	1.22E+08
	54767000	36455000	55200000	92973000	68819000	39879000	31897000	45971000
			73513000	2.64E+08	82614000	78896000		

13249000		15339000	7258400	18445000	14648000	22367000	21289000	9241900
					12729000	11171000		9494100
	32228000		4.48E+08	1.04E+08	1.59E+08	1.07E+08		
			72592000		39791000			
				42645000	43404000	49615000		52166000
			24693000	57194000	46148000			
	48918000	39701000	96666000	82407000	1.28E+08	1.3E+08	1.15E+08	1.15E+08
			21731000	24059000	28308000			50396000
63929000	46444000	53592000	24221000	23564000	41713000	57125000	14154000	33671000
54302000	63002000	37372000		29112000	37008000	22569000		23537000
				1699700				
33460000	37009000		51048000		45916000	77168000	73974000	61761000
				24157000	24268000	12851000		
24405000	23845000	31442000		1.3E+08	60345000		20091000	
					13988000		12113000	15530000
24768000	45293000	37574000	30763000	39909000	43299000	20631000		19332000
8.83E+08	1.41E+09	7.25E+08	7.67E+08	7.19E+08	8.33E+08	4.6E+08	1.61E+08	3.62E+08
21565000	24652000	28832000		14740000	18503000	13495000		
	1.13E+08						15315000	
12972000	11021000	11504000	9743100	10918000	14015000	11456000	31235000	9291700
1.21E+08	2.36E+08	2.19E+08	1.19E+08	4.48E+08	7.97E+08	8.98E+08	8.53E+08	6.68E+08
		20078000		15760000		11532000		12646000
36400000								
62688000	95548000	92734000	97189000	2.2E+08	2.33E+08	1.16E+08	1.53E+08	2.2E+08
	12335000		32119000					25747000
4.79E+08	5.74E+08	5.56E+08	8.81E+08	1.08E+09	1.08E+09	7.46E+08	7.51E+08	8.75E+08
64935000	43768000	70557000	70903000	74937000	73807000	48981000	1.03E+08	64962000
1.24E+10	9.88E+09	1.45E+10	1.8E+10	1.26E+10	1.07E+10	1.46E+10	1.62E+10	1.45E+10
					1.04E+08		2.65E+08	2.49E+08
	67782000	25468000	39795000	54561000	49751000	69183000	17494000	59421000
38532000				89671000	91870000		11516000	
52655000		71546000						
	1.54E+08		92139000	1.05E+08		1.58E+08	1.55E+08	1.35E+08
			37774000	35861000	32381000	18026000		
							6119600	
7.75E+08	7.29E+08	1.02E+09	1.14E+09	8.72E+08	9.25E+08	9.63E+08	1.09E+09	1.27E+09
		15108000	70484000	48390000	22944000			71935000
					7719300			
	5904200	50363000			21786000		8639200	9676400
4.78E+08	7.79E+08	7.08E+08	2.98E+09	1.11E+09	1.35E+09	1.18E+09	1.05E+09	1.52E+09
					35055000			
	43500000		33101000	66806000	66595000	44869000	32369000	51263000
40838000	44200000	77776000	24272000	23999000	31195000	28269000	41339000	53417000

3.27E+08	3.28E+08	3.67E+08	2.76E+08	2.07E+08	1.98E+08	2.45E+08	2.78E+08	2.79E+08
				51554000	11735000			
1.34E+08	1.87E+08	1.33E+08	2.7E+08	3.46E+08	3.02E+08	2.5E+08	2.96E+08	3.13E+08
3.85E+08	2.64E+08	1.05E+08	1.44E+08	1.79E+08	1.76E+08	1.72E+08	2.7E+08	2.71E+08
							13549000	
			4.21E+08	1.52E+08		3.56E+08	3.33E+08	9760800
	69149000		95323000	3.47E+08	2.11E+08	87899000		58631000
1.3E+09	1.12E+09	1.43E+09	1.45E+09	1.09E+09	8.33E+08	1.67E+09	1.48E+09	1.55E+09
15782000	29495000	22538000	19477000		23636000			2356000
59759000	76843000	75884000	74928000	85105000	59321000	33085000	51592000	75401000
			9297600			9715900	11070000	
1.41E+08	1.41E+08	1.57E+08	1.48E+08	1.17E+08	1.34E+08	71027000	1.29E+08	1.04E+08
5.18E+08	6.37E+08	52537000	26382000	6.78E+08	6.73E+08	5.45E+08	4.71E+08	7.52E+08
			24814000	33118000	29237000			
34337000	38886000	40811000	35298000	24987000	29528000	27428000	39123000	25846000
95913000	64756000	92862000	1.22E+08	1.21E+08	1.05E+08	1.2E+08	1.26E+08	1.29E+08
2.97E+08	5.24E+08	4.5E+08	8.18E+08	5.08E+08	3.86E+08	5.23E+08	5.38E+08	4.97E+08
								16634000
19671000	40881000	33730000	19393000	65341000	63839000	31639000	60615000	61890000
		17554000	18233000	27211000	15006000			
25078000	26991000	66378000	59078000	32820000	24400000	87510000	48409000	70813000
4.23E+08	4.96E+08	4.81E+08	6.32E+08	4.67E+08	4.56E+08	4.11E+08	3.77E+08	4.1E+08
6.55E+08	6.22E+08	7.59E+08	1.09E+09	9.95E+08	7.22E+08	9.12E+08	1.02E+09	1.15E+09
			19661000	60182000	34007000	24025000		17208000
			11068000			37625000		
30406000	26047000	26273000		19845000	25237000			
2855700	3747200	7330700		1419200	2075100			
1.6E+08	1.78E+08	1.09E+08		17278000	15036000		11032000	17118000
				48209000	25894000	26299000		
1.36E+08	1.17E+08	88075000	1.68E+08	1.31E+08	1.29E+08	2.24E+08	1.76E+08	2.06E+08
1.11E+08	58580000	89575000	80807000	71903000	73150000	54755000	1.7E+08	89015000
		12009000	30975000	32504000	30787000	31265000	17794000	35445000
42799000	59470000	57481000	86185000	67906000	72567000	1.12E+08	1.02E+08	1.31E+08
88540000	33335000	75907000	33589000	88033000	40929000			
	9926600	12688000						
		14559000	1.26E+08	1.87E+08	1.07E+08	1.36E+08	1.65E+08	2.36E+08
			60899000	2.72E+08	1.29E+08	13879000		
4.82E+08	4E+08	71450000	3.96E+08	2.75E+08	2.97E+08	29263000	1.57E+08	3.26E+08
6.12E+09	7.02E+09	4.6E+09	4.55E+09	5.26E+09	4.77E+09	9.75E+09	8.09E+09	7.88E+09
				69388000		2.82E+08	32108000	2.13E+08
1.04E+09	1.07E+09	1.22E+09	8.63E+08	6.14E+08	6.56E+08	9.97E+08	1.01E+09	1.04E+09
	35079000				29666000			
5.05E+08	3.1E+08	3.43E+08	2.16E+08	1.95E+08	2.44E+08	5.91E+08	4.56E+08	3.27E+08

1.18E+08	2.18E+08	40681000		4001000	2.25E+08		20883000	1.14E+08
44406000	32214000			37278000		50651000	50427000	49454000
30188000	41076000	44484000	22903000	35259000	24455000			
43368000	40144000			43718000	60260000			42861000
		56715000	1.18E+08	1.41E+08	1.35E+08	1.47E+08	1.12E+08	1.1E+08
	17549000							
2.69E+08	2.98E+08	2.28E+08	2.22E+08	3.02E+08	2.3E+08	1.5E+09	1.34E+09	
4.8E+08	5.35E+08	4.31E+08	5.32E+08	3.3E+08	3.87E+08	4.7E+08	13032000	4.08E+08
24602000	35711000	44374000	40958000	53173000	66144000	34760000	19905000	47545000
					5608900		8933300	12898000
1.35E+08	1.01E+08		70105000	65428000	51594000		50707000	65697000
	3E+08		2.96E+08	3.97E+08	4.35E+08	3.16E+08	2.04E+08	2.32E+08
	33323000	37896000	46795000	41932000	15343000	43463000		
	15617000			16489000	14105000			
31791000	17647000	23086000	17363000	13985000	17716000	13603000	61635000	18908000
7.02E+08	7.64E+08	1.07E+09	7.21E+08	1.06E+09	4.95E+08	8.84E+08	8.59E+08	1.18E+09
5898300				4911100	6947600			
						42620000		
1.22E+08	1.44E+08	1.34E+08	1.13E+08	1.24E+08	1.32E+08	92122000	61829000	80039000
			11396000	22000000	16151000	14661000	11743000	
			42901000	31741000	23129000	37188000	26097000	31442000
3.82E+08	4.4E+08	4.47E+08	4.1E+08	3.78E+08	3.69E+08	6.06E+08	1.9E+08	5.24E+08
1.01E+08	63245000	1.02E+08	68864000	71795000	64601000	57578000	48865000	63798000
9.39E+09	7.37E+09	6.49E+09	1.1E+09	1.62E+09	2.22E+09	1.39E+09	9.57E+08	8.44E+08
17104000	59621000		17450000	39195000	57633000	21832000		
	1984900							
1.06E+08	1.11E+09	1.22E+09	2.13E+09	1.77E+09	1.51E+09	2.61E+09	2.54E+09	2.8E+09
				21959000	16249000			
8.94E+08	1.47E+09	9.96E+08	2.73E+08	3.17E+08	3.31E+08		27788000	
			1806900		7144100			
35592000	63986000	35667000	22604000	72871000	57929000	49233000	27805000	19324000
			10513000		10755000	13679000	16754000	15637000
83253000	74748000	94426000	53607000	71289000	1.04E+08	9106900	67867000	1.49E+08
18208000	39603000	42325000	44864000	83583000	83357000	31320000	20034000	28189000
40214000	30590000	46784000		36238000				
	55048000	5.87E+08						
2.74E+08	3.35E+08	2.56E+08	1.98E+08	2.21E+08	2.22E+08	1.85E+08	1.07E+08	1.17E+08
67490000	1.4E+08	44439000	1.3E+08	2.75E+08	2.37E+08	2.04E+08	1.12E+08	1.49E+08
5.54E+08	4.88E+08	8.37E+08	6.13E+08	4.6E+08		5.72E+08	6.05E+08	5.28E+08
1.85E+08	2.45E+08	2.75E+08	2.11E+08		1.68E+08	1.93E+08	2.18E+08	2.31E+08
						27194000		
				8513800	8233600	4362900	8129600	11098000
	16359000		12637000	15735000	16580000	26039000		16410000
6.08E+08	5.34E+08	4.71E+08	3.33E+08	3.38E+08	3.3E+08	4.39E+08	4.62E+08	4.88E+08
9.78E+08	9.56E+08	8.12E+08	1.33E+09	1.33E+09	1.49E+09	2.44E+09	1.94E+09	1.75E+09

			1.36E+08	1.05E+08	1.02E+08	32947000	18740000	28848000
21514000					87505000	27847000	22727000	33310000
9.84E+09	9.56E+09	1.01E+10	6.84E+09	4.24E+09	5.79E+09	7.04E+09	5.52E+09	5.51E+09
	34647000	29941000	53028000	63075000	52947000	99254000	25558000	92140000
27581000	33905000		21731000				27885000	
1.15E+08	77997000	1.22E+08	70228000	54712000		63577000	69212000	53504000
8.45E+08	1.15E+09	9.39E+08	1.06E+09	9.8E+08	1.17E+09	9.51E+08	6.25E+08	8.38E+08
			19129000		27273000	26440000	31981000	25571000
9.64E+08	9.78E+08	9.55E+08	9.36E+08	1.16E+09	8.71E+08	76849000	1.07E+09	1.2E+09
4026500	947910	1931100	1336900	868550	1447500	2218600	8126000	1728900
			35905000		65381000			29100000
1.58E+08	1.86E+08	1.49E+08	1.22E+08	1.11E+08	1.57E+08	61694000	2.02E+08	2E+08
	21853000		21543000	61328000	64626000	67184000	16705000	38278000
22609000	33949000	17888000	40197000	26641000	36772000	22974000	18358000	18259000
1.68E+09	1.54E+09	1.29E+09	1.17E+09	1E+09	9.64E+08	6.65E+08	8.8E+08	7.85E+08
	47746000		82944000	85642000	71929000	55895000		36210000
29304000	24369000	24671000		11885000	20136000			
	28708000	15438000		20502000	28136000	40654000		
42223000	53218000	36312000	45909000	32713000	31488000		28993000	
			4205300					
795810			323560		333160			
20235000	39507000	5426700	1.61E+08	2.5E+08	2.13E+08	1.39E+08	1.03E+08	1.5E+08
			9434000		5631600	10100000		8055000
			2553600					
			56419000					
			27793000	28492000				
2.56E+08	2.76E+08	4.14E+08	1.41E+08	3.87E+08	1.88E+09	4.07E+08	4.81E+08	1.03E+09
13692000	22602000	62927000	48897000	58321000	43890000	15289000	17171000	14307000
8.02E+09	6.98E+09	8.44E+09	7.93E+09	5.59E+09	6.49E+09	6.69E+09	7.99E+09	8.49E+09
			3357100		3021200	3612900	9810900	11683000
60460000	69242000	54558000	1.29E+08	1E+08	1.49E+08	99316000		96851000
			98174000		2.09E+08	2.26E+08	1.78E+08	
16422000	32681000	17037000	10156000	11494000				
1.43E+08	3.82E+08		81600000	3.41E+08	2.33E+08	1.08E+08	91015000	1.03E+08
54064000	74722000		37277000		34608000	37402000		
44506000	36656000	46273000	43940000	48221000	48140000	40047000	39740000	34513000
2E+10	1.82E+10	2.46E+10	1.62E+10	1.21E+10	1.17E+10	1.3E+10	1.24E+10	1.28E+10
				8056700	6969000			

5.48E+08	4.43E+08	6.9E+08	4.91E+08	3.62E+08	3.43E+08	4.21E+08	3.73E+08	2.88E+08
	42112000	58067000			36566000		36160000	57011000
				4933200	7095500			15175000
1.65E+08	1.53E+08	3.05E+08	3.25E+08	2.16E+08	1.79E+08	2.87E+08	2.15E+08	2.38E+08
			817700		767440	1148300	4318100	3046200
13694000	41781000		19394000	31900000	38147000	35334000		
			5361100					
	74627000		77557000	1.35E+08	1.24E+08	1.17E+08		1.03E+08
		5.93E+08	6.72E+08	2.56E+08	2.67E+08			1.14E+10
3.08E+08		21859000	3.27E+08		40163000	2.74E+08	2.32E+08	2.59E+08
	17132000		24991000	68857000	51266000	43064000		
3515200	1844400	2349300	8535800	3857300	4928600	9020700	65959000	8943100
73022000		39515000						
89065000	62330000	1.11E+08	75775000	46975000	43964000	52205000	48097000	44796000
	22156000		38567000	47266000	36072000	37106000		29821000
38443000	21113000	31948000					32034000	
1.03E+08	1.32E+08	1.21E+08	47594000	36462000	1.05E+08	24561000	31373000	14173000
3.79E+08	4.61E+08	3.75E+08	6.35E+08	6.29E+08	6.52E+08	6.96E+08	5.22E+08	6.12E+08
16730000	20085000	13618000	36464000	40964000	44530000	21568000	18955000	27999000
	13885000		24079000	29426000	22198000	18945000		
	44157000							
16372000	12898000	16130000			9683600	11493000	25894000	11820000
1.1E+09	1.03E+09	1.18E+09	1.71E+09	1.73E+09	1.5E+09	1.33E+09	1.12E+09	1.93E+09
			2.51E+08	1.72E+08	1.9E+08	1.28E+08	13605000	48947000
7263800			4556000	1688600	12287000	2146200	15791000	
72674000	83352000	71326000	66727000	66187000	58592000	80066000	78681000	1.33E+08
			6044900	6005300		11141000		
			3443600	1371500				3527100
			46064000		64946000	71946000		15383000
			96738000	36515000	93106000	84452000		
50112000	52943000		4.02E+08	2.62E+08	2.94E+08	4.11E+08	1.46E+08	1.17E+08
5076700	3120000	5799100	5507700	2950500	3990400	3191200	12398000	4241500
			8666100		10016000	6080400		
1.28E+08	2.54E+08	2.11E+08	58124000	1.18E+08	1.12E+08	27065000	54802000	97075000
2.48E+08	2.95E+08	2.61E+08	2.27E+08	2.25E+08	2.26E+08	2.31E+08	2.22E+08	2.58E+08
2.47E+08	2.64E+08	3.14E+08	1.82E+08	1.48E+08	1.4E+08	43826000	96888000	1.1E+08
			23332000	20253000	29737000	33455000	52527000	40753000
14667000	11829000	16819000	88874000	34087000	21024000	44447000		15459000
9328900	19475000	11686000	19444000	35861000	25839000	12824000	3380500	22040000
68235000	46319000	51368000	81027000		1.43E+08	1.18E+08	3.21E+08	1.8E+08
	578610		1551500			1251500	6309000	
26117000	22698000		1.04E+08	71511000	72829000	94372000	72730000	43972000
45273000	49690000	43503000	22849000	29281000	34913000	37063000	39567000	42249000
32462000	20773000				46157000	48699000	38662000	35418000
			16067000		16662000			8448600
					11982000			
2.51E+08	2.01E+08	2.83E+08	3.15E+08	1.98E+08	1.99E+08	1.57E+08	1.72E+08	1.87E+08
	43879000		66294000	1.34E+08	97619000	42156000		32963000

	24096000				9382500			
1.22E+08	1.8E+08	1.74E+08	2.25E+08	3.39E+08	3.2E+08	3.46E+08	2.53E+08	3.56E+08
		9578100	5873100	4791100	6058400	4698600	14062000	6227600
43438000	44752000	49460000	92463000	71315000	65573000	99151000	84013000	94374000
41272000	1.44E+08	44653000	1.59E+08			30648000	28847000	1.9E+08
52051000	85247000	76418000	69297000	56993000		1.32E+08	55012000	1.2E+08
	4017800		10333000	5785700	4927200	9826200	8318400	15715000
69656000		52507000	1.33E+08	2.13E+08	1.9E+08	2.28E+08	1.76E+08	1.65E+08
	16915000				19627000			
3.26E+08	2.24E+08	3.43E+08	6.51E+08	4.52E+08	6.15E+08	7.28E+08	1.13E+09	7.58E+08
	42614000	22905000	36927000	29869000	31325000	33715000	42251000	40123000
			12564000		13342000			
1.11E+09	7.68E+08	1.02E+09	7.2E+08	5.8E+08	5.34E+08	9.57E+08	9.98E+08	9.42E+08
	13779000				14620000			
1.05E+09	7.38E+08	1.3E+09	8.29E+08	6.49E+08	5.9E+08	8.31E+08	7.12E+08	8.3E+08
2.45E+08	3.41E+08	2.51E+08	2.75E+08	9.02E+08	6.27E+08	1.48E+08	31928000	3.01E+08
1.04E+09	65659000	41161000	1.53E+09	1.89E+09	1.91E+09	21494000	3.15E+08	1.37E+09
6.13E+08	4.94E+08	6.35E+08	4.42E+08	4.01E+08	3.63E+08	3.7E+08	4.41E+08	4.15E+08
1.08E+08	76430000	81662000	91403000	1.41E+08	1.11E+08	94758000	98342000	94458000
	7771400	5889100	6089600	7654300	8499500		8928400	10050000
6272200					25286000			1617500
		27996000	5150200	1929100	19735000	17085000		22337000
2.16E+09	2.32E+09	2.31E+09	2.58E+09	2.51E+09	2.05E+09	1.92E+09	2.1E+09	2.01E+09
4.46E+09	3.06E+09	4.83E+09	6.13E+08	6.84E+08	5E+08	1.73E+08	5.35E+08	8.46E+08
43261000	33476000	35661000		1.86E+08		54455000	66355000	64425000
	20624000		32150000	97780000	96649000	35312000		
94069000	94369000	76799000	1.31E+08	1.55E+08	1.49E+08	1.71E+08	1.7E+08	1.94E+08
48635000					52587000		44085000	
99186000	2.1E+08			3.13E+08	2.75E+08	2.18E+08	1.23E+08	2.02E+08
9.33E+08	5.4E+08	6.21E+08	6.56E+08	5.54E+08	5.6E+08	6.43E+08	7E+08	5.89E+08
1.25E+09	1.48E+09	1.98E+09	7.19E+08	4.84E+08	4.83E+08	2.85E+08	3.48E+08	3.11E+08
1.16E+08	78259000	93795000	1.54E+08	1.02E+08	1.11E+08	1.73E+08	1.54E+08	1.57E+08
	10810000	18901000	27030000		24618000	14412000		
	5149500			6326100	3932300		13379000	7959900
8.13E+08	9.58E+08	9.48E+08	7.51E+08	4.92E+08	6.02E+08	6.57E+08	6.12E+08	5.15E+08
9.14E+09	8.84E+09	7.21E+09	9.43E+09	5.31E+09	6.42E+09	7.53E+09	8.45E+09	7.04E+09
					6941800			
						13399000		
		30000000						14409000
					16434000		13227000	15039000
1.51E+09	1.14E+09	1.2E+09	1.63E+09	1.3E+09	1.13E+09	2.02E+09	1.7E+09	1.96E+09
	9975300	8579900	18912000	19260000	21034000	22626000	18084000	26261000
	26159000		14479000	23386000	11447000			7257900
			7896200	20806000	34448000		9010400	35509000
34367000	36498000			16077000	20649000	46441000	27038000	26452000

74741000	74979000	57525000	27323000	34771000	49317000	63311000	54103000	53868000
	33043000	22026000	38122000	23449000	31001000	17440000	63448000	86700000
	25105000		26150000			21803000		
					14562000			
15734000	10770000	12220000		8142200	8893900	15475000	14161000	10020000
	19719000		24210000	58677000	38811000	22302000	15261000	25394000
1.57E+08	2.17E+08	1.97E+08	1.22E+08	1.24E+08	1.51E+08	2.76E+08	2.76E+08	1.99E+08
	89108000		89545000	1.23E+08	1.05E+08	1.12E+08	43995000	52616000
	44229000		47168000	68915000		77412000		
					3995100		1594000	
1.01E+09	9.55E+08	9.73E+08	1.16E+09	9.86E+08	9.5E+08	9.74E+08	1.04E+09	1.22E+09
6630900	7123000	6410200	5901100	8342400	8326500	5933200		
4.46E+08	3.55E+08	4.44E+08	2.91E+08	2.2E+08	2.04E+08	1.65E+08	1.27E+08	1.81E+08
		6470400			12556000	15448000	82881000	
	21279000		67789000	1.33E+08	1.24E+08	76606000		35389000
					5496000			
27909000	62606000		24966000		12474000			
							3768800	
3.75E+08	3.2E+08	4.32E+08	3.02E+08	2.54E+08	2.38E+08		2.83E+08	2.76E+08
29211000	35012000		30224000	26362000	40511000		28391000	
	594200	1264600	1291800	445050				
			38342000	48855000	43960000	29800000		
	21550000	14677000	7833600					
3.41E+08	3.49E+08	3.57E+08	5.06E+08	4.18E+08	3.59E+08	5.52E+08	5.14E+08	5.7E+08
8.89E+08	6.67E+08	8.54E+08	9.91E+08	8.13E+08	7.22E+08	1.24E+09	1.04E+09	1.04E+09
89901000		79428000	51008000	38701000	49482000	98486000	74213000	67568000
72167000	89280000	76995000	98583000	88670000	1.04E+08	1.14E+08	1.05E+08	1.14E+08
3.44E+08	2.56E+08	2.77E+08	4.29E+08	3.04E+08	3.76E+08	5.31E+08	4.55E+08	4.96E+08
	53586000		70610000	1.04E+08	65772000	67510000		
61461000	56107000	19431000	24522000	65026000	63258000	23239000	24753000	80970000
	18113000		17897000	27568000	14747000			
44208000	39280000	64218000	55715000	43415000	41831000	59268000	51134000	49751000
46224000	50482000	35970000						
	19609000		16867000		15098000			
				18735000	30203000			
21203000	24531000		1.36E+08	98770000	1.05E+08	1.68E+08	1.57E+08	1.04E+08
1.47E+08	2.25E+08	13850000	3.2E+08	2.63E+08	2.24E+08	1.56E+08	26797000	96678000
61770000		77491000	8.13E+08	3.43E+08	1.14E+08	52669000		46303000
73782000	1.01E+08	12694000	1.43E+08	2.21E+08	1.85E+08	1.05E+08	63730000	50061000
			10230000		18856000			
	22565000		17016000	31715000	22637000	17634000		30055000
14359000		15802000	12654000		10062000			
22412000								
							26812000	36133000
76342000	89616000	1.49E+08	1.47E+08	1.15E+08	62009000		34109000	67907000
				63415000	56014000			

14149000		31623000						
			20111000	15141000			32251000	19628000
13503000		15322000	10173000	7523600	10614000	9810100	18063000	
2.24E+08	1.73E+08	2.95E+08	1.61E+08	1.63E+08	1.14E+08	1.64E+08	2.45E+08	1.39E+08
1.05E+08	58394000	1.35E+08	64997000	57069000	45266000	72633000	63348000	53833000
2.07E+08	80002000	2.04E+08	3.44E+08	3.46E+08	2.67E+08	3.86E+08	4.03E+08	4.89E+08
65343000	1.26E+08	88924000	2.28E+08	2.54E+08	2.43E+08	3.63E+08	2.87E+08	3.08E+08
			10189000	8831000	4217900			
51379000		67499000	22411000	10042000	12123000			
61478000		76215000	80521000	59333000	58751000	86463000	80058000	23751000
36851000	31251000	37423000	1.21E+08	76869000	66022000	1.33E+08	1.76E+08	1.21E+08
28086000	7112500	12051000	3672300	1720700	3550500	1073400	5406300	
7.3E+08	6.63E+08	7.07E+08	2.62E+08	2.3E+08	3.43E+08	4.18E+08	3.24E+08	2.11E+08
4.23E+08	2.9E+08	5.67E+08	4.33E+08	3.42E+08	3.2E+08	4.14E+08	3.45E+08	3.58E+08
	24407000		35846000	61762000	36445000	32277000		
3.48E+09	6.84E+09	3.48E+09	8.77E+09	8.66E+09	7.87E+09	7.95E+09	6.91E+09	6.23E+09
1.36E+08	1.34E+08	2.52E+08	86691000	2.19E+08	1.28E+08	50328000	1.17E+08	1.07E+08
4.08E+08	3.75E+08	4.1E+08	1.02E+08	65609000	64010000	82007000	95558000	97171000
9627400	5326900		22182000	12096000	17938000	10101000	27654000	17670000
		1.46E+08	76218000				30157000	38055000
9165900	3523600	6250500	4744700	1483000	2346800	3204100	15153000	3322300
	15936000		33781000	63982000	39087000	34612000	38763000	46521000
1.1E+08	4.63E+08	2.28E+08	1.9E+08	3.04E+08	3.01E+08	81480000	56257000	1.08E+08
9.82E+09	8.9E+09	9.57E+09	1.21E+10	1.11E+10	1.03E+10	1.46E+10	1.28E+10	1.33E+10
2.85E+08	3.75E+08	3.43E+08	3.22E+08	3.28E+08	3.18E+08	4.04E+08	3.86E+08	3.62E+08
8E+08	5.83E+08	7.47E+08	8.28E+08	6.66E+08	6.77E+08	6.85E+08	7.79E+08	7.47E+08
17695000							14569000	
2.52E+08	2.74E+08	4.31E+08	2.75E+08	3.11E+08	2.2E+08	2.82E+08	4.1E+08	4.11E+08
5.62E+08	7.31E+08	5.34E+08	3.59E+08	5.47E+08	4.41E+08	6.54E+08	5.05E+08	5.21E+08
								32159000
31537000	52548000			34812000	40035000	39571000	35477000	31781000
	19589000			30861000	19186000		8918000	15424000
30404000		37527000						
								48314000
	20693000	23571000	12853000	12773000	15071000		23050000	17068000
1.42E+08	2E+08	2.27E+08	1.28E+08	1.17E+08	1.32E+08	1.49E+08	1.33E+08	1.09E+08
34896000	52399000	48673000	46080000	37400000	31810000	53307000	30665000	50018000
1.01E+08	55831000	89311000	1.66E+08	1.19E+08	1.32E+08	1.38E+08	2.82E+08	1.5E+08
	1.42E+08		4.16E+08	6.85E+08	5.27E+08	2.91E+08	1.3E+08	2.05E+08
29950000	38057000		44908000	28537000	29694000	58695000	53035000	40832000
			82740000	1.48E+08	1.55E+08	62480000	29096000	74746000
1.69E+08	1.17E+08	1.67E+08	1.51E+08	1.16E+08	1.09E+08	1.64E+08	1.37E+08	1.36E+08
29636000	24053000	29399000	35364000	27187000	38433000	38920000	86430000	58033000
43337000								

	12001000		10493000	7953700	9368700			
81202000	73618000	71142000	18992000					
2.14E+08	3.12E+08	57654000	64276000	2.48E+08	3.72E+08	2.64E+08	1.73E+08	1.87E+08
			22414000	12712000	17643000	12784000		
2.59E+08	5.73E+08	2.16E+08	2.72E+08	5.08E+08	4.08E+08	3.82E+08	2.2E+08	2.67E+08
1.84E+08	1.01E+08	1.85E+08	1.89E+08	1.36E+08	1.75E+08	1.4E+08	2.42E+08	1.98E+08
2.08E+09	1.89E+09	2.58E+09	2.54E+09	1.91E+09	1.5E+09	2.3E+09	2.99E+09	1.94E+09
76755000	1.65E+08		26979000	3.36E+08	3.02E+08	3.01E+08	64710000	1.63E+08
	26625000	41085000			24878000	20083000	21668000	33990000
1.74E+09	2.55E+09	1.11E+09	1.28E+09	8.98E+08	2.37E+09	3.89E+09	3.11E+09	2.1E+09
1.48E+08	1.89E+08	1.54E+08	3.31E+08	3.08E+08	2.97E+08	3.55E+08	4.19E+08	3.55E+08
13901000	26847000	5734300	63773000	32067000	36385000	12890000		41960000
26978000	1.26E+08	1.51E+08	2.04E+08	3.26E+08	4.93E+08	3.71E+08	1.21E+09	7.96E+08
3.23E+08	4.26E+08	1.82E+08	8.89E+08	7.45E+08	6.61E+08	4.68E+08	5.11E+08	6.31E+08
	1103400							
1.16E+09	8.35E+08	8.55E+08	3.3E+08	3.87E+08	4.87E+08	9.2E+08	7.9E+08	4.87E+08
					13004000	6888500		
		778070	349600				1057000	295420
				8650800				
								14615000
3.18E+08	4.9E+08	4.83E+08	6.55E+08	5.05E+08	5.4E+08	5.5E+08	6E+08	6.12E+08
22736000	25060000	31510000						
	1.47E+08		3.33E+08	3.34E+08	3.26E+08	2.4E+08	2.26E+08	2.36E+08
1.95E+08	1.65E+08	1.53E+08	93658000	98743000	1.29E+08	97948000	71309000	57857000
	23574000	34377000	19990000		11134000	21351000		22691000
7.62E+08	5.86E+08	6.79E+08	6.72E+08	4.9E+08	4.73E+08	5.11E+08	5.73E+08	6.88E+08
			17786000	12773000	11177000	18481000		
56023000	74224000	50797000	44085000	82682000	1.45E+08	1.27E+08	1.04E+08	77970000
1.57E+09	1.53E+09	1.32E+09	1.73E+09	1.73E+09	1.63E+09	1.85E+09	2.11E+09	2.58E+09
				51543000	43938000			
				66025000	67082000	1.45E+08	1.76E+08	1.13E+08
4.67E+08	4.39E+08	3.91E+08	1.18E+08	79921000	91940000	1.4E+08	1.31E+08	1.3E+08
					31603000	43320000		
			9014600	10202000	10398000	16230000		3050800
2.33E+10	1.97E+10	3.4E+10	1.76E+10	1.48E+10	1.32E+10	1.4E+10	1.85E+10	1.58E+10
	1.89E+08			24247000	16326000		27962000	
2.4E+08		2.41E+08		1.66E+08	96781000	1.64E+08	51671000	
				17846000	17746000			
					15697000			12475000
2.75E+09	2.18E+09	2.93E+09	2.25E+09	1.76E+09	1.5E+09	3.05E+09	2.86E+09	2.74E+09
	16121000	18715000	20101000	16743000		19557000	51282000	30742000
1649000			1177600					
25379000			68997000	65575000	30015000	25839000	24219000	41157000
2159600	1116600	1659000	2145100	787640	974950	1242500	6713200	1890600
59426000	68199000	95568000	1.04E+08	92883000	71933000	1.06E+08	75138000	91307000
1.87E+08	4.98E+08	80960000	7.04E+08	1.25E+09	1.23E+09	7.33E+08	4.29E+08	5.83E+08

58836000	50299000		38798000	33069000	35419000	27294000		
35016000		65651000	1.03E+09	4.69E+08	3.92E+08	5.87E+08	5.48E+08	7.09E+08
3.33E+08	3.52E+08	5.18E+08	4.89E+08	4.16E+08	3.08E+08	5.02E+08	4.57E+08	5.17E+08
1.13E+08	1.04E+08	92461000	80813000	84690000	84202000	1.02E+08	93740000	1.1E+08
				18927000			18307000	13522000
		7389300		16302000			10957000	
		31025000						
			56065000	20873000	18446000			
2.4E+08	2.37E+08	2.99E+08	2.47E+08	1.95E+08	57384000	41228000		1.56E+08
2.78E+09	2.25E+09	3.26E+09	3.46E+09	3.18E+09	2.31E+09	3.99E+09	3.99E+09	3.71E+09
36286000	7.97E+08	8.82E+08	5.49E+08	5.93E+08				1.9E+08
19751000	12665000	15021000	14155000	9907900	13153000	15585000	16482000	15744000
					30607000			
	42078000			17893000		32638000		
65477000	46575000	49753000	97930000	65261000	58563000	1.01E+08	87932000	1.01E+08
55962000	49717000	70401000	62554000	56715000	38940000	64917000	60320000	53552000
					11225000			
93504000	61472000	80183000						
96014000	87299000	1.71E+08	1.31E+08	1.09E+08	91062000	80901000	82801000	1.35E+08
28498000	24553000	35321000	76945000	63168000	48638000	17278000	14345000	26110000
					11713000	8639900		15116000
5.12E+08	6.08E+08	7.46E+08	3.72E+08	4.74E+08	3.98E+08	1.71E+08	1.86E+08	2.7E+08
			23812000		33153000		58759000	41538000
14936000	19653000					27751000	21889000	
			37874000	45634000	31900000			
			32620000	21737000	19250000			
								18198000
2.05E+08	1.79E+08	3.17E+08	2.67E+08	2.22E+08	1.49E+08	3.04E+08	3.16E+08	2.56E+08
26425000	8400300	12546000	14177000				31475000	
22095000	21272000	24023000						
9127400	14135000		6620500			13959000	9030700	12797000
	14804000				14469000			
	4.91E+08	5.93E+08						
	15926000		22963000	31673000	20889000			
	29710000		25638000	29991000		29309000	26784000	35196000
	12805000	29981000						
40618000	24212000	36372000	70208000	30654000	26990000	53483000	33623000	25642000
			27281000	25175000	33606000	24792000		19290000
			13755000					
2.11E+08	1.81E+08	2.08E+08	2.1E+08	1.31E+08	1.39E+08	1.82E+08	1.85E+08	1.87E+08
	38485000	37683000	43497000	38799000	36269000	69912000	41802000	73608000
			61028000	65639000	36641000		31610000	
			1.71E+08	1.61E+08	1.42E+08			
					25672000			
	36056000	28625000	17304000	51172000	77172000	12363000	18934000	47574000
					1377700			
1.43E+08	3.69E+08	6.97E+08	1.45E+08	3.52E+08	5.48E+08	3.07E+08	5.49E+08	2.94E+08
	17076000				11919000			
			82816000	1.75E+08	1.68E+08	38328000	51439000	

7.81E+08	9.68E+08	1.06E+09	1.42E+09	1.17E+09	1.12E+09	1.77E+09	1.96E+09	1.69E+09
5.78E+08	5.04E+08	4.97E+08	1.89E+08	5.94E+08	5.41E+08	1.26E+08	6.48E+08	7.8E+08
2.39E+08		2.36E+08				46394000	42090000	
42683000	49865000		15161000	20023000	22805000	29756000	26086000	16501000
				18260000	11806000			
4.54E+08	4.34E+08	5.67E+08	6.61E+08	4.64E+08	3.77E+08	6.27E+08	6.46E+08	6.14E+08
1.44E+09	1.11E+09	1.18E+09	1.26E+09	1.72E+09	1.73E+09	1.46E+09	1.54E+09	1.56E+09
33549000	23404000	43913000	39775000	29272000	37095000	52736000	68895000	48891000
3.01E+08	3.39E+08	3.8E+08	4.42E+08	3.29E+08	3.61E+08	3.81E+08	3.98E+08	3.58E+08
			48480000					
17576000	2.61E+08	1.18E+08	48896000	2.66E+08	7.34E+08		5.86E+08	1.98E+08
3.27E+08	2.82E+08	3.82E+08	3.23E+08	1.85E+08	2.34E+08	1.44E+08	1.8E+08	2.71E+08
1.01E+08	1.02E+08	1.31E+08	2.38E+08	1.94E+08	1.64E+08	1.85E+08	1.7E+08	1.8E+08
29739000		34381000						
8.28E+08	7.19E+08	8.27E+08	4.44E+08	3.88E+08	3.1E+08	2.27E+08	3.29E+08	3.27E+08
	13846000	9664700	27255000		21391000	29485000	23507000	35018000
					13775000			
8.46E+08	6.7E+08	1.1E+09	6.93E+08		4.5E+08	5.12E+08	5.91E+08	6.04E+08
31241000		16037000	5805500					
5.15E+08	4.55E+08	7.39E+08	9.33E+08	6.9E+08	4.34E+08	6.18E+08	6.75E+08	5.83E+08
79158000	69911000	1.08E+08	99538000	66788000	57091000	88106000	67516000	81418000
1.07E+08	40750000	1.46E+08	48556000	48199000	56548000	1.27E+08	81311000	70957000
92497000	26525000	64324000		29581000	59689000	22461000	37187000	52493000
3.39E+08	4.28E+08	2.56E+08	2.21E+08	1.96E+08	2.16E+08	3.48E+08	2.53E+08	2.14E+08
	19573000	43743000	14154000	13284000	11305000			
62181000	30724000	66333000	23047000					
63442000	1E+08	1.52E+08	1.5E+08	1.56E+08	96883000	70972000	72859000	97134000
91998000	98035000	1.81E+08	1.93E+08	1.64E+08	1.29E+08	1.19E+08	94542000	
93529000	85163000	1.62E+08	89112000	78444000	57357000	68135000	64774000	64884000
59542000	32572000	47196000	12327000		16988000			
27523000	19939000	52543000						
2.84E+09	2.84E+09	3.9E+09	3.9E+09	3.76E+09	1.92E+09	2.84E+09	3.92E+09	4.64E+09
	16907000			29540000	31910000	14639000		17918000
38142000	1.67E+08	79880000	54408000	1.52E+08	1.71E+08	1.93E+08	1.13E+08	2.66E+08
				17250000	72227000			
					6661600			
	3E+08	68827000	50851000	37885000	2.64E+08	5.01E+08	3.39E+08	2.31E+08
44794000				34104000	45166000		35479000	29287000
6.3E+08	1.09E+09	2.12E+09	4.4E+09	3.38E+09	1.86E+09	1.4E+09	2.16E+09	3.91E+09
6.89E+08	9.18E+08	8.33E+08	5.22E+08	7.43E+08	6.57E+08	3.38E+08	3.96E+08	4.36E+08
						55849000		
2.27E+08	2.06E+08	2.75E+08	5.28E+08	4.34E+08	3.7E+08	6.15E+08	5.11E+08	6.28E+08
3.4E+09	2.82E+09	3.6E+09	2.14E+09	1.67E+09	1.46E+09	1.83E+09	1.73E+09	1.54E+09
7822300	4671500	7999100	5636200	3071600	4019600	6342700	13419000	6553300
41140000	49491000	68961000	64599000	61059000	82474000	31526000	49131000	55250000
			35811000	2.09E+08	2.01E+08	1.05E+08	11942000	47083000
16541000	14057000	16122000			12808000	35584000	28662000	

1.2E+10	8.51E+09	1.16E+10	9.26E+09	7.27E+09	6.46E+09	1.01E+10	1.05E+10	8.99E+09
4.28E+08	3.84E+08	5.83E+08	2.06E+08	2.07E+08	1.86E+08	62570000	90296000	1.05E+08
10752000	24878000	20572000	16813000	24972000	31824000	15600000	14989000	41288000
2.79E+08	5.16E+08		5.5E+08	6.35E+08	5.62E+08	3.24E+08	24415000	1.39E+08
48222000	50795000	45668000	78838000	80547000	82251000	95861000	88204000	1.06E+08
	28607000		32643000	27003000	33228000	24920000		23122000
1.8E+08	1.47E+08	1.34E+09	1.2E+09	1.4E+09	1.97E+09	3.69E+09	2.97E+09	1.84E+09
					29214000	36412000		
4.24E+08	4.39E+08	2.25E+08	1.11E+08	1.45E+08	2.32E+08	4.71E+08	4.34E+08	2.49E+08
20260000	13553000	18261000	30170000	24306000	31766000	65595000	1.78E+08	51998000
69288000	84219000		59527000	79982000	64235000	88196000	52926000	
	15668000				20325000	22137000		11825000
2E+08	1.48E+08	2.19E+08	59183000	1.17E+08	72498000			
15995000				17312000		16211000		
12604000	22088000	22707000		12916000	18068000	23848000	25266000	
			72797000	50969000	75281000	63634000	55439000	35226000
6.59E+08	9.29E+08	6.49E+08	1.51E+08	3.47E+08	3.23E+08		2.53E+08	1.81E+08
	11831000			24748000	24460000	21460000		8496900
			8463100					
2.07E+08	3.53E+08	1.3E+08	2.38E+08	2.47E+08	2.57E+08		28855000	1.25E+08
			33787000	23289000				
8590500	6364600	7747900	12638000	8147900	10576000	9574100	37307000	12289000
			15329000					19282000
84589000	81835000	82128000	1.14E+08	1.07E+08	1.18E+08	1.84E+08	1.94E+08	1.57E+08
			67627000	68082000	80299000	14459000		
						4679100	17091000	
1.28E+08	1.75E+08	1.89E+08	2.54E+08	3.32E+08	3.26E+08	2.32E+08	2.79E+08	3.19E+08
2.48E+09	3.5E+09	3.24E+09	1.44E+08	1.21E+09	9.41E+08	1.72E+08	71670000	2.68E+08
4.38E+09	3.17E+09	2.87E+09	2.17E+09	1.54E+09	2.18E+09	2.32E+09	2.36E+09	1.33E+09
							1.65E+08	
96600000	1.09E+08	20089000	1.29E+08	2.09E+08	1.84E+08	1.67E+08	43747000	67715000
	33731000		87699000	1.36E+08	77948000	64003000	31903000	
	11712000			77135000	73561000			
							2.42E+08	
36974000	28550000	42533000	80948000	74707000	83071000			1.03E+08
1.11E+09	8.37E+08	1.02E+09	7.35E+08	7.63E+08	6.66E+08	9.65E+08	9.47E+08	8.91E+08
5.21E+08	5E+08	4.63E+08	4.77E+08	4.57E+08	4.24E+08	4.3E+08	4.04E+08	4E+08
5.75E+08	3.84E+08	6.36E+08	3.27E+08	2.18E+08	2.02E+08	2.14E+08	2.19E+08	2.16E+08
1.8E+08	1.89E+08	2.09E+08	1.61E+08	1.88E+08	2.55E+08	4.39E+08	4.46E+08	2.92E+08
			46477000	1.61E+08	1.72E+08	70953000		68602000
39601000	1.39E+08	1.22E+08	1.1E+08	2.75E+08	4.09E+08	1.64E+08	2.59E+08	7.46E+08
		38896000		17517000	19415000	1.08E+08	3.88E+08	40019000
							1265900	
	38002000	31901000	55805000	73751000	77773000	1E+08	56530000	69880000
1.63E+08	1.37E+08	1.44E+08	2E+08	1.47E+08	2.05E+08	2.37E+08	3.74E+08	2.75E+08
				6840100		5562100		10515000

	15896000		23534000					14330000
			49136000					94159000
			80593000	49909000	43489000	93438000	74535000	68195000
			13873000	25343000	16507000	14209000		10711000
2.16E+08	2.35E+08	2.24E+08	2.24E+08	1.96E+08	1.76E+08	1.83E+08	1.61E+08	2.17E+08
63548000	1.57E+08	83242000	67097000	1.51E+08	1.16E+08	1.01E+08	1.36E+08	1.57E+08
2.43E+08	2.64E+08	2.56E+08	1.5E+08	1.78E+08	2.04E+08	2.98E+08	3.08E+08	1.86E+08
3.34E+08	2.64E+08	3.26E+08	2.97E+08	2.03E+08	2E+08	2.87E+08	2.94E+08	2.65E+08
	30193000		12496000		25019000			16705000
2.96E+08	3.38E+08	4.75E+08	2.81E+08	2.12E+08	1.97E+08	2.79E+08	2.93E+08	2.97E+08
78524000	58230000	59484000	1.33E+08	99484000	1.63E+08	2.06E+08	4.94E+08	3.13E+08
			2.4E+08	2.77E+08	2.35E+08	2.29E+08	2.26E+08	2.73E+08
			72716000		53689000	59067000	52612000	64050000
29926000	22584000		34541000	19953000	18244000	26278000		25595000
1.51E+08	1.27E+08	71358000	1.11E+08	1E+08	98217000	1.48E+08	54249000	1.34E+08
47758000	29323000		1.08E+08	63637000	53974000	51116000		30522000
14224000	65048000		2.85E+08	2.02E+08	2.14E+08	2.54E+08	1.2E+08	1.9E+08
1.33E+08			90312000		94921000			
8.26E+08	8.25E+08			1.8E+09	2.5E+09	1.44E+09	2.67E+09	2.51E+09
1E+09	6.76E+08		9.1E+08	7.27E+08	6.31E+08	7.37E+08	6.56E+08	6.38E+08
	21144000	28090000	1.22E+08	97760000	74500000	25001000	22626000	44336000
						10362000		
10138000					15182000	16635000	18598000	
18592000	29307000		25025000	44726000		42139000		40161000
89305000	1.17E+08	1E+08		1.22E+08	85106000	79474000	1.28E+08	1.44E+08
1.14E+08	1.57E+08	98850000	1.74E+08	1.9E+08	1.78E+08	1.72E+08	1.21E+08	1.48E+08
2.52E+08	2.8E+08	2.61E+08	2.6E+08	2.5E+08	2.25E+08	2.75E+08	2.77E+08	2.84E+08
							31825000	
8.91E+08	1.08E+09	8.73E+08	1E+09	1.18E+09	1.41E+09	1.51E+09	1.2E+09	1.42E+09
						3.62E+08		
		1.56E+08	2.33E+08	1.87E+08	1.45E+08	1.31E+08	1.31E+08	1.3E+08
			1.1E+08				55226000	
9.64E+08	1.04E+09	1.12E+09	2.67E+08	3.3E+08	3.46E+08	4.03E+08	5.09E+08	2.85E+08
3.55E+09	4.03E+09	3.22E+09	1.54E+09	1.85E+09	2.85E+09	3.13E+09	3.21E+09	2.17E+09
			16391000	16599000	14000000	8676300	6114700	5252300
1.19E+08	1.27E+08	1.79E+08	2.99E+08	2.22E+08	2.45E+08	3.68E+08	3.57E+08	4.21E+08
			24570000	1.93E+08	1.61E+08	53248000	50896000	
12486000	5426700	9944600	7843600	3991700	3698100	349260		1351500

23942000	42421000			48149000	17417000	17780000	13016000	19666000
				19953000				
7.35E+09	6.09E+09	7.44E+09	4.39E+09	3.67E+09	3.38E+09	2.66E+09	2.98E+09	2.86E+09
				15868000				
5257000	9209100	3969900	31050000	18262000	23365000	37560000	50528000	47405000
3493800			79641000	15580000	39068000	65795000	89145000	82627000
	76070000	36157000	50737000	48268000	64110000	32714000	23766000	27290000
3.7E+09	3.77E+09	3.51E+09	2.03E+09	1.79E+09	1.54E+09	1.6E+09	1.33E+09	1.73E+09
6326800	2525700	3848900	7869900	2368800	3460900	5210000	26687000	5728000
49275000	31064000		25488000	27091000	39273000	39116000	13681000	
			1.97E+08	1.67E+08	1.44E+08			
1.45E+08	1.7E+08	1.52E+08	1.31E+08	1.27E+08	1.96E+08	1.07E+08	2.13E+08	1.51E+08
3.72E+08	3.52E+08	2.88E+08	2.98E+08	1.54E+08	3.19E+08	2.59E+08	4.75E+08	2.25E+08
	30925000		35553000	56805000	62267000	23340000	25072000	15069000
3.26E+08	6.24E+08	4.17E+08	1.15E+09	1.17E+09	1.19E+09	2.37E+08	1.84E+08	4.75E+08
			57880000	40163000	41310000	53254000	56587000	44339000
	70113000		16473000	23106000	18509000		14400000	44154000
14186000	46450000	22736000	48024000	32500000	36556000	34197000	23451000	36931000
34515000	44444000	64798000	70828000	51185000	50791000	48205000	46855000	68505000
7.25E+08	8.24E+08	7.79E+08	9.56E+08	8.54E+08	7.95E+08	1.22E+09	9.34E+08	1.08E+09
			14412000	41670000	44171000	30190000	14852000	11579000
			68697000	60976000	63564000	45079000	5489900	12053000
1.03E+08	1.39E+08	1.14E+08	90792000	94372000	93246000	95171000	1.04E+08	90638000
						6053900		7602100
94515000	80016000	57266000	70846000	1.63E+08	1.26E+08	32010000	18298000	36161000
	9746500		36646000	55969000	54931000	57032000	11008000	13313000
4.84E+08	6.66E+08	5.03E+08	2.26E+08	2.56E+08	2.29E+08	1.65E+08	95589000	1.63E+08
	37149000	13685000		17471000	55808000	9196300	14515000	25426000
3.7E+08	3.01E+08	4.97E+08	5.47E+08	3.72E+08	2.78E+08	6.84E+08	8.37E+08	9.28E+08
20811000	49030000		23449000	87343000	93409000	1.94E+08	87265000	1.82E+08
28273000	26647000			18657000				
46537000	50727000		94949000	1.07E+08	81519000	98633000	50987000	62839000
				4104700		11145000		11977000
4902800	1040100		1238400					
83157000	1.02E+08	89487000	1.68E+08	1.56E+08	1.68E+08	2.99E+08	1.68E+08	2.69E+08
					38250000			
	71350000		1.82E+08	1.39E+08	94643000	90994000	27989000	47372000
5.76E+09	4.42E+09	4.77E+09	2.96E+09	2.58E+09	2.34E+09	3.86E+09	3.37E+09	3.69E+09
1.09E+09	1.1E+09	1.03E+09	8.48E+08	8.08E+08	7.5E+08	7.08E+08	6.72E+08	7.18E+08
1.63E+08	2.38E+08	1.8E+08	1.79E+08				1.57E+08	1.87E+08
1451900	539240		2775600	659210	739800	2277300		

4.66E+08	5.8E+08	1.69E+09	7.06E+08	1.26E+09	1.26E+09	6.04E+08	5.45E+08	7.44E+08
71788000	1.66E+08		1.73E+08	1.66E+08	2.02E+08	1.36E+08	31977000	59000000
51778000	58940000	35728000	36750000	47071000	48409000	14939000		23902000
				15809000		32085000	23066000	19383000
73546000	40211000		26042000	20022000	20917000	27023000		17558000
								8407100
7.97E+08	1.81E+09	7.84E+08	1.36E+09	1.47E+09	1.59E+09	1.86E+09	1.22E+09	1.36E+09
4.3E+08	4.05E+08	1.19E+08	3.05E+08	4.07E+08	5.98E+08	8.62E+08	7.2E+08	6.27E+08
1.91E+09	1.82E+09	2.01E+09	1.25E+09	1.56E+09	1.89E+09	2.92E+09	2.51E+09	1.67E+09
	29950000			37137000	65928000	36141000	22809000	18353000
			10882000	5874700	7441800	7769400		2667900
16027000	19204000	20485000	28234000	33884000	20035000	32643000	34252000	27661000
74451000	47071000	65062000	45247000	31167000	32929000	8855000		24712000
6.23E+08	4.95E+08	4.5E+08	5.3E+08	4.61E+08	3.99E+08	4.99E+08	5.13E+08	4.57E+08
			1.44E+08	23522000	52661000	36422000	45176000	19641000
	40775000	46946000	25580000	51527000	71259000	34361000	66556000	86311000
	5398000			2466500	4357800	3198200	8796500	
1.49E+09	2E+09	3.25E+09	1.8E+09	1.82E+09	1.89E+09	1.91E+09	1.25E+09	1.42E+09
52753000	45262000	51419000	37110000	31953000	37316000	52946000	52407000	61544000
	2.38E+08		1.84E+08	4.01E+08	4.54E+08	68698000		
			16732000	22940000	17009000	26950000		
1.02E+08	72712000	1.08E+08	91596000	58235000	64886000	50827000	57655000	41571000
3.77E+08	3.64E+08	3.27E+08	6.25E+08	3.63E+08	4.39E+08	4.82E+08	4.29E+08	5.31E+08
2.33E+08	4.62E+08	88769000	4.53E+08	4.84E+08	4.94E+08	3.29E+08	1.23E+08	1.95E+08
2.75E+08	3.74E+08	8.21E+08	1.56E+08	1.71E+08	4.61E+08	82321000	3.44E+08	3.69E+08
87710000	1.01E+08	1.22E+08	1.73E+08	1.89E+08	1.91E+08	1.84E+08	1.48E+08	1.79E+08
40858000	59346000	42160000	12132000	45830000	74850000		25242000	25382000
	48870000		80420000	5.92E+08	4.38E+08	1.73E+08	25936000	1.97E+08
			12798000	15571000	15532000	10119000		
				14865000				
			21157000	11091000	8504500	12004000		12188000
99726000	85156000	1.11E+08	24167000				67057000	
	27677000							
					6019500	13572000		
1.53E+08	2.24E+08	1.28E+08	48507000	1.77E+08	3.07E+08	72716000	3.46E+08	5.69E+08
30938000	8063000	25320000	4135500	2378300	2724800	1678500		1622200
				13257000	13254000	12982000		19644000
46219000	47427000	37618000	83021000	1.09E+08	65088000	77564000	94711000	84752000
71633000	8859900	75390000	1.53E+08	1.27E+08	87884000	1.37E+08	33817000	2.23E+08
			57282000		37638000	23869000		
1.04E+08	39634000	44983000	75871000	71056000	62316000	26506000	44455000	38845000
8.33E+08	1.23E+09	17043000	1.23E+09	9.36E+08	8.09E+08	9.91E+08	1.19E+09	1.15E+09
4.85E+08	3.38E+08	6.79E+08	1.06E+08		74843000	20046000		14669000
			63723000	1.14E+08	89745000	51492000		
9.53E+08	6.49E+08	9.66E+08	6.54E+08	4.58E+08	3.93E+08	5.23E+08	5.34E+08	5.22E+08
	1.57E+08	1.16E+08	3.31E+08	3.13E+08	2.58E+08	2.4E+08	2.22E+08	3.41E+08

45337000	20148000		68085000	53139000	89538000	31812000	95127000	22351000
81934000	1.06E+08	93300000	3.6E+08	3.42E+08	2.06E+08	1.8E+08	1.31E+08	1.64E+08
2.61E+08	2.34E+08	3.41E+08	2.34E+08	1.99E+08	1.85E+08	1.41E+08	1.36E+08	1.47E+08
44173000	70400000			5402700	3165600			78516000
			6.46E+08	2.97E+08	4.66E+08	4.38E+08	97021000	1.07E+08
8072700	3219100	5982600		1653500	2332200	1787800		
	31766000				13918000			
1.8E+09	1.64E+09	3.22E+09	1.19E+09	1.2E+09	8.45E+08	2.48E+09	2.46E+09	2.58E+09
	21823000				30480000			
4.2E+08	2.57E+08	4.2E+08	2.85E+08	2.5E+08	2.06E+08	2.39E+08	2.24E+08	2.63E+08
		23191000						
		18364000	18266000		8159500	21516000		13326000
46663000	24679000	38622000	33359000	29107000	31303000	19390000	46264000	21859000
1.1E+08	1.66E+08	63641000	81234000	1.07E+08	94333000			
			31188000	28470000	34476000	5806300		
1.26E+08	1.38E+08	1.56E+08	51488000	91979000	1.18E+08	77009000	1.08E+08	83982000
52512000	51425000	70566000	54755000	43075000	50341000	47538000	48774000	54307000
1.82E+08	1.36E+08	1.75E+08	2.15E+08	1.93E+08	1.97E+08	2.04E+08	2.28E+08	4E+08
12724000	10890000	31824000			27631000			3305300
1.37E+08	1.01E+08	1.57E+08	90981000	79790000	64443000	87066000	69919000	83115000
35084000	17156000	37807000	33068000	36845000			27936000	38228000
5.43E+08	4.97E+08	5.17E+08	3.66E+08	4.67E+08	3.18E+08	3.4E+08	2.55E+08	3.16E+08
	14271000		25693000	34478000	26264000			
1013600	765470		4284300		1062300	2738500	15669000	3947500
85550000	82364000							
4.75E+09	3.53E+09	5.64E+09	2.83E+09	2.14E+09	1.95E+09	2.57E+09	2.27E+09	2.23E+09
2.84E+08	1.71E+08	2.63E+08	43197000	31293000	62857000	56676000	31197000	29147000
12208000	9110700	11244000	12762000	9729000	14822000	10666000	48793000	12725000
83028000	66987000	96922000	47664000	51305000	31342000	27474000	23058000	42984000
34139000	31020000	41538000	92093000	63375000	90787000	91266000	80310000	50447000
46846000		2.73E+08	95425000	2.82E+08	4.42E+08	72845000	2.27E+08	1.72E+08
5306100	865790	1361700	1688700	720650	787670	732970	5118100	914530
6.75E+08		7.68E+08	3.07E+08	3.44E+08				
6.73E+08	6.39E+08	6.42E+08	3.82E+08	4.48E+08	3.5E+08	3.26E+08	2.11E+08	2E+08
1.31E+09	5.35E+08	2.9E+09	1.7E+08	1.1E+08	99057000	1.76E+08	1.67E+08	1.24E+08
14800000	12406000	16516000						
2.38E+08	2.36E+08	2.62E+08	2.81E+08	2.85E+08	2.13E+08	2.45E+08	2.64E+08	3.39E+08
4.77E+08	3.82E+08	4.94E+08	4.18E+08	3.66E+08	3.04E+08	4.34E+08	5.1E+08	3.71E+08
31513000	77692000	87072000	2.04E+08	2.39E+08	1.25E+08	2.33E+08	1.68E+08	2.04E+08
15435000		16718000					6807000	
28013000	15437000	27625000	31244000		18924000	36276000	16796000	31182000
6.48E+08	5.6E+08	1.2E+09	24141000	42023000	31170000	29533000	31649000	
19927000		39420000		4623300	37519000	14485000	31747000	

		19220000						
26382000			19465000	18389000				
48432000	38392000	45440000	35388000	30690000	38364000	52117000	93801000	46362000
40455000	76512000	87834000	43013000	72116000	49679000	1.82E+08	1.43E+08	1.37E+08
					37365000			26108000
33573000	39925000	55732000		21896000				
70386000	60378000	46769000						
			12091000	15413000	11248000	34526000	15810000	25054000
	13394000	15054000		16169000	30932000	25414000		12116000
							14100000	
1.17E+08	1.21E+08	1.11E+08	1.57E+08	1.16E+08	1.12E+08	1.51E+08	1.27E+08	1.62E+08
			56192000		35209000	35657000	54284000	52297000
	41439000	41521000	64385000	50367000	43034000	58272000	64212000	67011000
20195000	10591000	18876000	63577000	60264000	62379000	59005000	8577300	6858300
	10706000				6556000			
	36454000		92405000	1.82E+08	1.51E+08		58187000	75373000
					89827000			
1.69E+08	1.98E+08	1.67E+08	2.33E+08	1.84E+08	2.1E+08	2.77E+08	2.79E+08	3.02E+08
1.87E+08	1.58E+08	1.78E+08	1.01E+08		78322000		81442000	
99966000	1.48E+08	45229000	1.57E+08	1.81E+08	2.24E+08	1.77E+08	1.59E+08	1.6E+08
54154000	43637000	1.25E+08	92091000	60540000	56120000	21424000	11916000	26290000
1.29E+08	1.18E+08	1.66E+08	40180000	32277000	43877000	23376000	25098000	24171000
7.15E+08	7.34E+08	7.66E+08	5.89E+08	5.34E+08	4.27E+08	4.04E+08	3.73E+08	4.2E+08
			47862000			47785000	46748000	54863000
7.1E+08	7.69E+08	1.03E+09	1E+09	1.15E+09	9.13E+08	4.83E+08	3.26E+08	4.87E+08
53173000	23322000			29482000	41740000	38454000	41283000	36092000
	1030200	1497200	1639300	689190	959660	1197600	6675900	1390700
					40802000			
			26773000					
			97529000	8958000	84066000	1.25E+08	79601000	7265400
1.21E+08	36266000		2.44E+08	59195000	1.64E+08	4.92E+08	8.15E+08	5.09E+08
		79664000		71838000	63338000			
					22553000			
			17572000					
	31624000	15448000	28199000	40706000	51236000			30017000
			2.12E+08	72712000	71933000	39787000		26975000
			1.3E+08	34603000	23169000	29359000		
			48646000	29002000		22917000		
				1824300				
			20586000		5231700	9430700		
			21599000	31123000	32839000	31609000		

			26222000					
			19245000	15602000	7957200			
			15754000		10897000			22031000
			28813000	14561000	20261000			
				21883000		25390000		
	11626000		13437000	21374000	18365000			
			28208000	42722000	39974000		48122000	39058000
2.48E+08	5.99E+08	8.39E+08	1.86E+08	61661000	2.46E+08	5.29E+08	3.78E+08	2.73E+08
						35551000		61355000
73343000			69734000	50223000	38033000	46275000	44054000	48936000
17834000	11847000	16037000						
2.77E+08	1.92E+08	3.13E+08	1.28E+08	1.03E+08	67229000	77756000	87718000	96315000
	1288600	1605900						
					2.5E+08			
3.35E+09	1.79E+09	2.8E+09	2.74E+09	2.22E+09	2.67E+09	2.78E+09	4.16E+09	3.78E+09
4523800	865650	1070800	1671200	498940	661490	845790	4740300	970510
3.79E+08	2.76E+08	5.23E+08	3.64E+08	2.58E+08	2.16E+08		3.12E+08	2.82E+08
6.58E+08	5.47E+08	6.14E+08	6.4E+08	5.5E+08	5.05E+08	6.1E+08	9.32E+08	6.83E+08
7.63E+08	7.66E+08	9.39E+08	2.04E+08	1.38E+08	1.05E+08	1.12E+08	99796000	1.1E+08
				10779000				13059000
32025000	33616000	36655000		34885000	21832000			42840000
1.2E+08	1.17E+08	1.49E+08	96685000	1.74E+08	1.35E+08	1.27E+08	1.48E+08	1.85E+08
					20997000	19398000	27389000	
57781000	1.24E+08	50818000	1.04E+08	1.26E+08	78923000	1.05E+08	1.11E+08	1.07E+08
						9473100		
26971000	1.1E+08		36422000	1.28E+08	1.05E+08	42168000		
2.84E+08	2.68E+08	2.49E+08	3.02E+08	2.28E+08	2.39E+08	2.65E+08	3E+08	3.63E+08
1.49E+09	1.34E+09	1.09E+09	3.57E+09	1.39E+09	3.94E+09	3.72E+09	1.02E+09	5.19E+09
27080000	29409000	36011000	42107000	7454200	19518000	27096000	66184000	21767000
1.23E+08	1.53E+08				72793000	1.11E+08		
				1.12E+08	54602000			
					32107000		6055000	7659100
				8488500	9262400			
		8340600		13989000				
1.51E+08	1.1E+08	1.38E+08	94255000	61988000	61985000	78589000	95566000	1.14E+08
18288000	37344000		81392000	1.2E+08	1.03E+08	1.1E+08	28335000	66203000
				40156000	32216000	44371000		
1.22E+08	55397000	80459000	50948000	35505000	53805000	56065000	2.07E+08	66594000
	28788000	36009000		25004000	40873000			41021000
1.25E+09	1.02E+09	1.34E+09	1.14E+09	8.52E+08	6.85E+08	7.41E+08	8.12E+08	9.81E+08
99494000	1.57E+08		1.99E+08	2.41E+08	1.89E+08	2.34E+08	53267000	1.71E+08
1.15E+08	63037000	1.2E+08	68079000	54769000	46994000			
32095000	27362000	17812000	29967000	62808000	42503000	29071000	21359000	55738000

4.11E+08	2.99E+08	5.44E+08	3.02E+08	2.66E+08	2.06E+08	3.96E+08	4.85E+08	4.5E+08
	25219000							
		10483000	6252000	5468600	4894400			
						11891000	12091000	
68818000	83665000	1.08E+08	1.07E+08	81223000	76259000	95494000	92605000	97521000
				35237000	28405000		29891000	26635000
48494000					36403000	86023000	10485000	
15217000	28367000	27797000	54514000	57402000	43445000	45070000	71261000	55801000
40719000	65347000	49680000	76161000	53062000	61249000	67225000	77392000	86252000
4.44E+08	3.77E+08	2.58E+08	5.76E+08	3.42E+08	3.82E+08	23095000	2.98E+08	3.21E+08
			32235000					32065000
25970000	27638000	30335000	31388000	25633000	32352000	36527000	35689000	35244000
15492000	13821000					15056000		15738000
9689700	8527400	9758000	18448000	13390000		13422000	18680000	
2.48E+08	2.8E+08	4.1E+08	1.57E+09	1.01E+09	8.22E+08	1.75E+09	3.18E+09	3.13E+09
34999000	45410000	47429000	47275000	44280000	48681000		47712000	36916000
1.95E+08	1.54E+08	1.71E+08	1.26E+08		68255000	50435000	64379000	90471000
79975000	1.21E+08	93228000	1.36E+08		98621000	58184000	47997000	79486000
51887000	67396000	53969000						
			15378000			20269000		
	71903000		60117000	1.34E+08	1.08E+08			
			14160000	17666000	13011000	19402000		11772000
	18598000		14526000	50071000	24252000	14628000	23835000	12531000
			560080	267090	334530		2114400	
		65137000	20501000		13026000	18624000	7217500	
37313000							16532000	
		2416100	2833600	3523200	3360200		11655000	4145300
2528500	1525600	1883100	2507300	1082100	1098200	1386100	8164000	
10246000	4329000	7552400	5495800	4192700	5361400	5714200	15406000	5937200
2.44E+08	2.45E+08	3.89E+08	8.77E+08	7.61E+08	8.51E+08	8.97E+08	1.64E+09	1.43E+09
	22701000		35511000	19210000	19279000			17361000
1.24E+10	7.75E+09	1.46E+10	1.78E+09	1.07E+09	9.32E+08	7.08E+08	7.38E+08	6.44E+08
			10422000	28909000	15734000	13775000		9960000
34716000	53509000	31744000	35397000	54091000	23616000	37504000	23873000	39120000
9425000		9181500	10159000		9434100	8596800		8509900
17229000		21157000	47160000	28176000	58787000	28957000	31038000	56777000
2.11E+09	1.75E+09	2E+09	1.63E+09	1.33E+09	1.08E+09	1.41E+09	1.52E+09	1.65E+09
1.49E+08	1.21E+08	1.85E+08	1.38E+08	1.1E+08	91639000	1.09E+08	1.29E+08	1.23E+08
15655000	18546000		36243000	31342000	30608000	34049000	36895000	40773000
			33041000	27995000	19629000	14012000		12189000
1.14E+08	1.03E+08	1.55E+08	18488000	13921000	61195000		70655000	
	17186000			8593900	13202000			
		27547000		2519400		23971000	38567000	
	47515000			56537000	49619000	52665000		

				20724000	25304000		12923000	14201000
61070000	65416000	71514000	52907000	37394000	52696000	65870000		58620000
			46215000	66574000	51519000	39544000		49822000
4288400	1742400	2918500	3329300	1565300	1599700	4260800	24209000	4505100
	39007000		28356000	39904000	24618000	45003000	52961000	79859000
	46581000			24959000	27931000			
	43256000	45514000		41383000	31563000			
		10875000		41481000				15695000
	40837000			14580000	13070000	11181000		
66678000	43666000	62896000	22548000	21295000	22291000	48746000	38506000	22087000
		46465000	44350000	42352000	27247000	56937000	52580000	52930000
2.99E+09	2.9E+09	3.05E+09	3.95E+09	4.1E+09	2.79E+09	4.78E+09	4.76E+09	4.92E+09
		17332000	17171000			10548000		
9807600	21958000	14196000	2.69E+08	3.81E+08	17454000	18509000		2.98E+08
4.64E+08	3.69E+08	5.26E+08	7.45E+08	7.04E+08				7.66E+08
1.96E+08	1.59E+08	97346000	21469000	20730000	21487000	87102000	19041000	92724000
3E+08	2.76E+08	3.87E+08	3.71E+08	3.76E+08	3.51E+08	3.38E+08	44254000	52512000
			8188400	6395000		3555300		
3.64E+09	3.49E+09	3.22E+09	2.02E+09	2.71E+09	3.57E+09	4.36E+09	3.72E+09	2.68E+09
	1.16E+08		1.22E+08	1.52E+08	1.85E+08	86386000	51630000	27818000
2E+08	4.28E+08	2.21E+08	4.45E+08	5.99E+08	6.55E+08	5.44E+08	4.23E+08	5.28E+08
				68472000		37972000		
2.03E+08	74169000	2.24E+08	2.38E+08	1.67E+08	1.62E+08	1.49E+08	3.21E+08	3.11E+08
3.71E+08	2.3E+08	2.14E+08	3.48E+08	2.55E+08	3.32E+08	4.23E+08	5.1E+08	4.57E+08
43627000	56744000	93407000	63436000	1.06E+08		1.14E+08	1.62E+08	1.39E+08
64482000	54116000	91467000	19137000	22367000	16806000	9302700		15175000
		15159000	5874700	7375900		10429000		
	32987000	72505000	6043400	10105000	62079000		19169000	1.09E+08
6077900	3002900		5144600	2102300	3163600		12675000	4686300
2.42E+08	2.03E+08	3.21E+08	96885000	1.05E+08	79322000	82178000	1.13E+08	2.12E+08
27599000	39268000	37910000	45369000	24390000	36238000	39420000		31269000
9.15E+08	1.58E+09	5.28E+08	2.37E+09	1.14E+09	1.38E+09	2.54E+08	1.2E+09	1.32E+09
4.79E+09	1.97E+09	2.42E+09	2.87E+08	1.72E+09	1.81E+09	3.58E+08	3.12E+08	2.45E+09
	15999000		51795000		50753000	43071000		60409000
					19452000		11951000	
				17086000				
				27062000	33506000			
6.79E+08	3.65E+08	6.77E+08	3.18E+08	2.47E+08	2.47E+08	1.73E+08	1.26E+08	1.3E+08
				21930000				
39666000	47836000	47733000	36782000	28605000	30224000	22333000	22007000	39779000
1.08E+08	1.3E+08	78342000	1.27E+08	1.24E+08	1.73E+08	1.3E+08	1.57E+08	2.09E+08
	19898000			21035000	24092000	27865000		16725000
1.55E+08	1.27E+08	2.38E+08	88538000	2.6E+08	2.09E+08	61191000	1.49E+08	1.9E+08
21314000	22427000	23012000						
61119000	2.58E+08	1.48E+08	78673000	2.72E+08	4.35E+08	2.23E+08	3.71E+08	3.32E+08
4.04E+08	3E+08	4.83E+08	3.99E+08	4.67E+08	4.23E+08	1.8E+08	1.73E+08	2.36E+08
12739000				47353000	32845000	16461000	28405000	44445000

31353000	22873000	31725000	92846000	71920000	50694000	79964000	74526000	80455000
1E+08	58889000	84144000	83055000	56328000	53482000	1.36E+08	1.15E+08	1.08E+08
40667000	97103000	92269000	1.07E+08		95812000		33651000	59973000
	62665000			40201000				
2.87E+08	2.86E+08	2.09E+08	1.96E+08	2.33E+08	2.71E+08	70143000	63401000	51473000
3.27E+08	2.84E+08	3.63E+08	2.62E+08	2.13E+08	2.54E+08		1.63E+08	2.41E+08
6.76E+08	4.58E+08	6.23E+08	3.65E+08	2.36E+08	2.29E+08	2.39E+08	2.21E+08	1.78E+08
			66730000	71092000	54621000			
	4.04E+08	2.89E+08	24266000	2.2E+08	1.65E+09		1.61E+08	2.21E+08
2.14E+08	1.69E+08	2.06E+08		93763000	1.16E+08		1.74E+08	1.21E+08
92575000	2.02E+08	1.54E+08		35021000	41109000			
		1.82E+08						
34459000			68011000		1.03E+08	84860000	52180000	26624000
61350000	51777000	68250000	51314000	42206000	49136000	53418000	41474000	59377000
	40984000		42865000	68111000	48353000	43399000	30382000	49214000
		41543000	70957000	74212000	79569000	57849000	1.06E+08	1.09E+08
	53680000		60382000	54654000	66847000	77903000		
						13754000	12479000	9695600
62197000	52187000	70284000	86332000	1.09E+08	66107000	12753000	25916000	47308000
					7569800			
4.31E+08	2.77E+08	4.06E+08	6.97E+08	3.52E+08	4.46E+08	7.31E+08	6.09E+08	6.22E+08
58584000	65695000	67443000					34552000	
4.13E+08	2.73E+08	4.38E+08	4.96E+08	3.72E+08	3.1E+08	6.74E+08	7.34E+08	7.78E+08
				25705000	12534000			
69584000	48032000				17393000			10826000
				25502000				
37198000	32441000	37739000	24489000	64859000	29729000			81011000
4.09E+08	2.75E+08	5.17E+08	2.69E+08	2.18E+08	2.33E+08	3.01E+08	4.05E+08	2.14E+08
46309000	53451000	60235000	16793000	19189000	11651000			13231000
	18452000				20310000			
14616000	14858000	37105000	42063000	73928000	32772000	37565000	73365000	49395000
10010000	16354000	9812800	33144000	9422500	10926000	25566000	72406000	25332000
				19303000				
	40479000				56491000			68673000
	27623000	26148000	36972000	36756000	28967000	45810000		43597000
					19102000		7464600	10167000
1.23E+09	1.91E+09	1.47E+09	3.31E+09	3.62E+09	3.57E+09	3.76E+09	4.28E+09	4.28E+09
				1.95E+08	2.02E+08			
1.09E+08	61769000	1.15E+08	25280000	18929000	17870000			
63732000	52453000	60015000	83440000	64638000	97123000	82280000	1.78E+08	1.05E+08
					1.2E+08			
			30475000	40685000	44290000	21764000		9179500
9409000	4210900	6768900	6969500	2381500	3893100	5570200	22799000	
					26787000	22501000		
			11772000	14239000	14197000			

6.27E+08	9.26E+08	6.38E+08	1.04E+09	9.76E+08	1.01E+09	1.16E+09	8.47E+08	1.06E+09
2.55E+08	2.98E+08	2.69E+08	3.34E+08	2.36E+08	2.31E+08	4.25E+08	3.97E+08	3.93E+08
	22624000		12156000	83924000	1.13E+08	28437000		
2.02E+08	1.96E+08	80006000	2.77E+08	2E+08	1.77E+08	2.37E+08	2.31E+08	2.3E+08
	1.23E+08	1.94E+08	3.25E+08	2.14E+08	1.99E+08	2.96E+08	3.5E+08	2.35E+08
12538000	9341100	12689000	11303000	7318200	11315000	10409000	41687000	
			35344000	27363000	10673000			
1291900		340010	2066100			979910	5286400	1796800
48235000	83752000	2.37E+08	89131000	1.56E+08	1.13E+08	99046000	69069000	95696000
68646000	1.1E+08	1.54E+08		32427000	28961000			
9.05E+08	5.33E+08	9.55E+08	8.85E+08	5.55E+08	5.12E+08		70777000	2.78E+08
5.79E+08	3.86E+08	4.76E+08	3.03E+08	2.75E+08	2.51E+08	3.51E+08	3.49E+08	3.21E+08
31593000	33246000	25792000	39763000	37556000	29804000	37845000	33012000	39875000
5417300	69102000	1.01E+08	13022000	1.78E+08	1.35E+08	24696000	30341000	1.97E+08
	25321000	4897200	36399000	1.45E+08	1.13E+08	31909000		75218000
1.41E+08	1.26E+08	1.01E+08	1.6E+08	1.34E+08	1.18E+08	2.49E+08	3.3E+08	2.43E+08
1.76E+08	1.77E+08	1.69E+08	1.35E+08	1.24E+08	1.16E+08	1.22E+08	1.23E+08	1.16E+08
	21501000	37570000	33886000	33133000	27420000			
2.06E+08	2.85E+08	1.71E+08	2.04E+08	2.37E+08	2.9E+08	2.04E+08	1.51E+08	2.01E+08
80021000	1.84E+08	77720000	90733000	1.42E+08	2E+08	1.29E+08	38858000	55217000
	4634200	19851000	95604000	4.21E+08	1.94E+08	2.59E+08	3.58E+08	4.09E+08
96943000	1.16E+08	99502000	20784000	21858000	23306000	2.04E+08	1.51E+08	1.6E+08
	30143000		10546000	26611000	28697000			
						29365000		33859000
4.41E+08	3.13E+08	4.05E+08	3.32E+08	2.49E+08	2.04E+08	1.59E+08	1.17E+08	2.34E+08
46444000	49400000	1.93E+08	1.65E+08	91927000	97986000	2.13E+08	1.65E+08	2.61E+08
				84657000				28615000
1.84E+08	1.58E+08	1.35E+08	2.5E+08	1.95E+08	1.81E+08	1.65E+08	1.77E+08	1.9E+08
	57959000	45085000		18953000				
	54728000	49558000	49263000	71308000	61704000	42633000		
			94306000	84035000	68934000	76230000		34198000
7.26E+08	5.97E+08	6.41E+08	5.02E+08	5.51E+08	3.95E+08	2.44E+08	2.71E+08	3.51E+08
2.51E+08		1.8E+08	1.88E+08	92504000				
81712000	67468000	83110000	32405000	26541000	67684000	95365000	66014000	49431000
9.54E+08	7E+08	8.84E+08	3.86E+08	3.01E+08	2.77E+08	1.76E+08	1.86E+08	1.82E+08
			4537300	2306700	1288800	2761700	12954000	2415600
3.07E+09	3.35E+09	2.9E+09	8.43E+08	7.7E+08	7.23E+08	9.89E+08	1.15E+09	8.62E+08
1.7E+09	1.77E+09	1.37E+09	1.21E+09	1.22E+09	1.19E+09	1.5E+09	1.62E+09	1.54E+09
1.06E+08	1.31E+08		4.85E+08	4.29E+08	4.14E+08	3.94E+08	1.97E+08	2.04E+08
	11747000				8029100	18056000	22071000	
88948000	1.18E+08	1.05E+08						
1.42E+08	1.7E+08	1.44E+08	54600000	66185000	57413000	50898000	38224000	42783000

					4863200			
1.3E+08	1.42E+08	1.33E+08	2.01E+08	1.67E+08	1.38E+08	1.65E+08	1.71E+08	1.87E+08
27308000	19401000	28311000	20179000	13403000	15134000	15633000	35898000	19064000
					10303000		25281000	14301000
2.04E+08	1.97E+08		3.08E+08	4.1E+08	4.28E+08	2.07E+08	65110000	
3.63E+08	4.16E+08	3.19E+08	2.51E+08	2.79E+08	2.3E+08	2.21E+08	1.86E+08	2.46E+08
25667000	31069000	37090000	53523000	52891000	49415000	69987000	66576000	61359000
51803000	88764000	41486000	66144000	91834000	66331000	43779000	52104000	56501000
				32599000		15479000		
76174000	52053000	50139000	45276000	29560000	25469000	24704000	24318000	26255000
84687000	73710000	65795000	87619000	69516000	65833000	95515000	81790000	1.04E+08
	66039000	1.01E+08		68012000	62359000	1.61E+08	1.38E+08	1.25E+08
1.18E+09	1.06E+09	1.77E+09	1.17E+09	9.95E+08	8.72E+08	1.13E+09	8.81E+08	9.96E+08
				26087000				
14910000	5774100	10506000	9320500	6404000	7277700	7875400	44868000	8833700
		4984700	5641600		3298200	9554500	6443700	9490100
28391000	24280000	26801000	12615000	21282000	16853000			
				95338000				
2212100	2182000	1996700	2014200	2152200	2228400	3015900	7098700	4523100
25237000	75513000	27353000	33944000	72585000	1.34E+08	53896000	36323000	92969000
22645000	18949000	30879000	22704000		16193000	19513000	19130000	26098000
	26737000		52472000	47443000	38225000	29637000		29732000
			21467000					
			21775000	20368000	15444000	31088000		11434000
35784000	55518000	73538000	78544000	90964000	84525000	98370000	93588000	98399000
15968000	16221000	21685000	14095000	12132000	21176000	34664000	28491000	
	38938000			35131000	39905000		34955000	
8909100	19863000	37135000	3429600		21612000			6281700
51256000		44902000			21371000	25386000	28020000	
20560000		31847000						
2.33E+08	3.1E+08	2.35E+08	1.34E+08	1.48E+08	1.26E+08	2.2E+08	1.9E+08	2.14E+08
	23204000		28176000	27375000	18681000		26835000	30408000
				10104000				
					3638800			
								15481000
						7567900		
18111000	14488000	42984000		14104000	17734000			19446000
			28183000					
	57663000	1.01E+08	64728000	77415000	57348000			62849000
94427000		1.49E+08			15232000	7855300		8447500
15347000	26227000	18365000		16823000	21724000		78572000	

			24488000	32102000	34251000	36277000	39479000	44441000
	21552000			65865000	42942000	26721000		33152000
1.27E+08	1.64E+08	1.46E+08		47610000	48098000			
				67266000		63422000	61082000	76611000
21498000								
1.01E+08	94694000	95794000	1.72E+08	1.3E+08	1.18E+08	1.81E+08	1.38E+08	1.46E+08
				69100000	57459000			
2.6E+08	2.84E+08	3.3E+08	3.07E+08	3.3E+08	2.44E+08	4.01E+08	3.65E+08	4E+08
2.85E+08	2.22E+08	4.13E+08	1.82E+08	1.49E+08	1.19E+08	1.64E+08	1.51E+08	2.2E+08
65602000	40801000	56402000	1.57E+08	98080000	51225000	1.29E+08	1.13E+08	1.26E+08
	20802000		41037000	25326000	25656000	26107000	19623000	33067000
12011000	12790000		99092000	57681000	1.02E+08	22191000		4658000
48749000	60766000	57448000	43946000	48727000	44283000	60740000	68397000	72876000
				35033000				
1.84E+08	1.78E+08	1.4E+08	1.41E+08	1.65E+08			75978000	1.4E+08
15846000	28610000	28566000	28170000	38368000	26768000	22399000	30611000	30904000
			4568700	2338500	2295300	4861600	6836800	6894200
	10199000	91768000	22742000		36620000			46018000
6.61E+08	9.58E+08	5.53E+08	5.13E+08	5.48E+08	5.95E+08	6.6E+08	1.55E+08	6.95E+08
3163500	1907300	4165600	6131000	1930800	2581700	1825900	11894000	3851700
	89886000		95620000	1.38E+08	1.42E+08	91975000		19353000
					3892700		11834000	
	6394300		12837000	9378100	13422000	13540000		19770000
1.94E+08	1.71E+08	1.74E+08	2.52E+08	2.54E+08	2.04E+08	2.77E+08	2.7E+08	2.99E+08
77711000	62550000	77070000	49556000	31802000	31843000	22190000		30097000
								14039000
3.07E+08	2.54E+08	4.75E+08	1.9E+08	1.91E+08	2.21E+08	1.51E+08	1.73E+08	1.75E+08
2.56E+08	1.94E+08	2.83E+08	3.89E+08	2.96E+08	3E+08	4.95E+08	3.74E+08	6.11E+08
		11522000			12221000		9061900	18670000
2.47E+08	1.61E+08	2.55E+08	2.21E+08	1.37E+08	1.66E+08	1.98E+08		2.05E+08
								14179000
	51067000	57515000	21942000	20351000	16528000		15559000	24968000
2.71E+08	2.64E+08	2.6E+08	1.91E+08	1.7E+08	1.51E+08	2.22E+08	2.49E+08	1.87E+08
3.94E+08	3.25E+08	4.33E+08	3.92E+08	3.11E+08	2.79E+08	3.46E+08	3.98E+08	4.03E+08
			15886000	17414000	26761000			
5.6E+08	4.71E+08	6.5E+08	7.1E+08	6.42E+08	4.76E+08	7.68E+08	7.83E+08	7.13E+08
					22446000		55456000	
	1.94E+08	1.59E+08			82657000		1.68E+08	2.14E+08
8.21E+08	1.01E+09	8.85E+08	8.71E+08	1.15E+09	9.67E+08	8.57E+08	6.35E+08	7.49E+08
66888000	56807000	73043000	67070000			57112000	65870000	58558000
21632000				16083000	16069000			
	22430000		38395000	35073000		48277000	54520000	52266000
1.64E+08	1.79E+08	1.44E+08	2.06E+08	2.22E+08	2.42E+08	3.08E+08	2.1E+08	2.37E+08
4.77E+08	4.17E+08	31869000		3.29E+08	4.15E+08	7.93E+08	6.34E+08	4.54E+08
4.57E+08	4.55E+08	3.7E+08	4.29E+08	4.97E+08	5.16E+08	4.99E+08	5.99E+08	5.59E+08
37389000	32982000	37822000	33612000	36897000	26130000	44004000	22293000	
42486000	1.14E+08	60075000	1.54E+08	1.52E+08	1.4E+08	1.7E+08	1.64E+08	2.05E+08
1.81E+08	2.24E+08	1.13E+08	3.22E+08	2.69E+08	2.54E+08	2.35E+08	1.08E+08	1.47E+08

			27537000		17070000			34735000
				42476000	38028000	38758000		27131000
		2341000			3732900			
65188000	87069000	1.28E+08	1.65E+08	1.3E+08	1.75E+08	1.53E+08	2.26E+08	2.74E+08
	30286000							
						40421000		
2.71E+08	6.49E+08	5.32E+08	1.17E+09	1.16E+09	1.11E+09	1.65E+09	9.27E+08	1.31E+09
	12684000		96796000	65472000	56298000			1.13E+08
			9741400			17384000	16057000	12986000
45323000	45964000			27848000	47074000	69399000	58231000	51571000
1.41E+08	1.78E+08	2.32E+08		1.3E+08		1.14E+08	31190000	38423000
88168000	1.03E+08			88321000	78979000	1.26E+08	2E+08	1.22E+08
4.27E+08	5.54E+08	3.37E+08	1.16E+09	1.15E+09			2.13E+09	1.82E+09
85715000	1.29E+08			1.21E+08	1.45E+08			1.98E+08
		19722000						
			19320000	23825000	19254000	15739000		20993000
24388000					16495000			
48202000	47180000				29427000	74486000	59182000	
2.08E+09	2.58E+09	3.61E+09	2.3E+09	2.31E+09	1.84E+09	1.2E+09	1.12E+09	1.18E+09
4.9E+08	4.28E+08	5.69E+08	7.23E+08	5.27E+08	4.88E+08	6.02E+08	6.55E+08	6.68E+08
				30513000				
			3231300	1501800	2825600			
	1047500	754010	3649500	1993800	1828000	3679600	20781000	3242800
	25467000		16255000	19803000	16362000	12337000		12367000
14839000	14245000	20817000	39950000	46525000	37613000	65182000	51776000	89135000
	69383000		48257000	41951000	36775000	1.06E+08	70085000	66208000
2.65E+08	3.2E+08	4.02E+08	3.84E+08	4.49E+08	2.72E+08	5.26E+08	5.74E+08	4.79E+08
3726200	2405800	3023900		2300800	3436000			3553600
	23954000	29649000	19861000	16317000	18456000	27902000	25225000	
73658000	60196000	96654000	1.33E+08	1.22E+08	99666000	1.23E+08	1.15E+08	1.16E+08
38156000	1.78E+08	76832000	77714000	1.65E+08	2.02E+08	95996000	1.04E+08	1.28E+08
31723000	35261000	33360000	81552000	57085000	55846000	90638000	78699000	90172000
51223000	43833000	68991000	83768000	63176000	47177000	74044000		74694000
25079000		43826000	10325000	15791000	17152000	9508800		13313000
			17042000	17898000	15613000	24723000	18236000	39540000
46817000	59649000	52432000	74162000	1.17E+08	59766000	1.02E+08	80627000	78393000
48486000	57707000	46406000	54285000	55329000	60446000	60574000	53796000	50400000
			46048000	45434000	41438000			
			22441000	69473000	64012000			19477000
2.6E+10	1.68E+10	2.26E+10	2.01E+10	1.24E+10	1.13E+10	1.28E+10	1.32E+10	1.24E+10
			4.68E+08	9.1E+08	2.59E+08	28957000		
1.37E+08	1.06E+08	1.22E+08	66231000	74444000	58338000	77825000	87172000	87335000
62462000	86617000	59778000	70126000	84897000	77352000	86488000	46950000	71302000
12303000		11972000			8258400			13372000
				9909700	14079000	12901000		

			27093000	18543000	18027000	25611000	24223000	23550000
		14464000	3915000	4694700				
					7070600			
26322000	50794000	48979000	14573000	38557000	11401000	5686200		10869000
						60981000	73831000	
	1.74E+08	44055000	1.44E+08	1.21E+08	1.73E+08	1.7E+08	82652000	89713000
	1.14E+08		1.09E+08	1.54E+08	1.91E+08	65914000	17590000	40449000
	14713000	23373000		14971000	14982000			
1.03E+08	3.24E+08	1.75E+08	1.98E+08	1.45E+08	1.36E+08	1.23E+08	1.26E+08	1.51E+08
				10785000	16421000			
1.13E+08	93251000	1.51E+08	1.51E+08	1.3E+08	1.09E+08	96986000	86967000	1.01E+08
						61915000	62798000	64766000
4.37E+08	4.02E+08	5.16E+08	6.86E+08	6.18E+08	5.06E+08	8.48E+08	7.85E+08	7.36E+08
1.79E+08	1.41E+08	2.25E+08	1.03E+08	1.1E+08	84289000	74864000	1.07E+08	79663000
53109000	32473000	48071000	27853000	27977000	31743000	19027000	47563000	19467000
28239000		35244000						
82339000	51981000	1.01E+08	61198000	51588000	50501000	47699000		54681000
					20563000	66308000		
1.97E+09	3.75E+09	2.42E+09	1.88E+09	2.24E+09	2.89E+09	1.93E+09	1.45E+09	1.35E+09
	63671000	30161000		16238000	43374000	39555000	30718000	34914000
2.76E+08	4.08E+08	1.46E+08	57191000	41824000				
96391000	1.23E+08	71938000	1.06E+08	62029000	58851000	1.78E+08	65555000	78654000
67422000	87363000	1.05E+08	74014000	93097000	1.1E+08	57506000	62967000	1.61E+08
					11816000			
84341000	1.41E+08	2.56E+08	1.22E+08	2.39E+08	2.59E+08	1.66E+08	3.41E+08	3.99E+08
			31375000	32800000	28290000			
	29020000	41383000	18410000	38329000	18127000	33254000	38750000	31256000
13244000	3.35E+08	3.35E+08	49049000	6.34E+08	68065000	4.45E+08	3.76E+08	6.26E+08
1.19E+09		1.35E+09	7.51E+08	6.49E+08	5.87E+08	5.16E+08	4.46E+08	
	43781000		24437000	26446000	24996000			25441000
			8044700		40940000	7969100		
2.08E+09	1.05E+09	2.09E+09	5.92E+08	1.1E+09	1.75E+09	6.02E+08	1.49E+09	1.56E+09
1.24E+08	1.36E+08	1.58E+08	1.57E+08	1.27E+08	1.95E+08	3.67E+08	2.7E+08	2.12E+08
60538000	3.69E+08	65407000	1.13E+08	8.11E+08	7.99E+08	3.77E+08	8.14E+08	7.28E+08
			16888000	13282000	14318000	9249900		
2.14E+08	1.84E+08	2.09E+08	2.43E+08	2.09E+08	1.83E+08			3.2E+08
66065000	76847000	1.17E+08	80778000	3.42E+08	1.55E+08	92079000	1.75E+08	1.81E+08
						1.12E+08	95698000	1.14E+08
	1090600		1919700	1797200	2012500	2503600		1730300
1.15E+08	92023000	1.05E+08	1.44E+08	1.29E+08	1.1E+08	1.5E+08	1.19E+08	1.62E+08
1.1E+08	79855000	1.53E+08	84348000	80041000	61427000	61749000	62178000	67194000
48057000	66314000	49565000	61145000	65555000	67212000	1.11E+08	1E+08	1.05E+08
			7709800	13904000		26271000	21022000	25903000
51090000		51314000	37124000	41080000	34380000			
2.21E+08	3.48E+08	2.98E+08	1.3E+08	1.37E+08	1.98E+08	3.08E+08	2.99E+08	2.48E+08
	61998000				28305000	32985000		25277000
1.01E+09	9.19E+08	1.24E+09	1.02E+09	9.58E+08	8.46E+08	1.26E+09	1.48E+09	1.26E+09

			38075000	1.5E+08	1.3E+08	1.12E+08	67157000	1.04E+08
2.66E+08	2.31E+08	4.98E+08	2.68E+08	5.07E+08	2.55E+08	2.88E+08	3.11E+08	5.94E+08
			40265000	28097000		22084000		
	45631000		1.69E+08	2.23E+08	1.12E+08	1.07E+08		73442000
63627000						55923000	61681000	82700000
24522000	11993000	84366000	26868000	18081000	17253000	27533000	23258000	19338000
35809000	71754000		1.37E+08	2.52E+08	1.23E+08	12100000		10424000
					9820900			
3.19E+08	6.88E+08	5.01E+08	4.12E+08	3.52E+08	3.76E+08	3.62E+08	3.97E+08	4.37E+08
2.66E+08	2.62E+08	2.93E+08	1.58E+08	1.41E+08	2.19E+08	4.11E+08	3.82E+08	1.73E+08
4.7E+08	8.83E+08	5.01E+08	1.28E+09	1.7E+09	1.67E+09	2.08E+09	8.98E+08	2E+09
3.71E+08	1.87E+08	3.65E+08	3.34E+08	2.01E+08	2.58E+08	1.47E+08	3.57E+08	3.07E+08
1.33E+08	1.43E+08	1.73E+08	2.49E+08	1.85E+08	1.8E+08	2.47E+08	2.25E+08	2.65E+08
3.99E+09	2.75E+09	3.35E+09	2.46E+09	1.99E+09	1.62E+09	2.27E+09	2.39E+09	2.21E+09
12603000	16585000	30368000	47963000	14609000	25823000	18713000	28429000	80882000
1.25E+08		91230000	60269000	58322000	50806000	39588000		50472000
2.22E+08	2.52E+08	2.59E+08	1.97E+08	2.02E+08		1.52E+08	1.51E+08	1.01E+08
							41849000	21203000
1.15E+08	99835000	84384000			24281000	50428000	41872000	
58645000	56929000	65488000	93066000	71044000	77984000	1.1E+08	87558000	84683000
			3100700	3046400	7052100	3045700		4143900
				21860000	26773000			
6.16E+08	6.08E+08	8.5E+08		7.39E+08	7.37E+08			7.97E+08
	36249000		22952000	29150000	25481000			
74992000	1.83E+08		1.98E+08	2.71E+08	2.56E+08	2.47E+08	1.23E+08	2.15E+08
2.4E+08	1.73E+08	2.4E+08	2.65E+08	1.95E+08	1.75E+08	3.12E+08	2.86E+08	3.38E+08
		63419000	31981000	30242000		25844000		
		69246000	63728000	54044000	42025000		29195000	75039000
20266000	25353000	59379000	11878000	9203000	13922000			
34972000	47029000	63936000				32354000	19661000	19588000
7E+08	7.89E+08	1.1E+09	1.05E+09	1.12E+09	8.36E+08	7.42E+08	8.58E+08	7.26E+08
46568000		87022000	24057000	19844000	16319000			15981000
1.96E+09	2.6E+09	2.72E+09	3.51E+09	3.02E+09	3.01E+09	4.76E+09	3.94E+09	4.88E+09
22318000	9776400	17732000		1.2E+08	10101000	36831000	41611000	80178000
1.98E+08	2.02E+08	71730000		94569000	1.01E+08			
26691000	21660000							
1.19E+08	1.28E+08	1.45E+08	2.76E+08	1.95E+08	1.97E+08	3.02E+08	2.18E+08	2.74E+08
								28342000
4.85E+08	3.83E+08	3.62E+08	1.95E+08	2E+08	84924000	85817000	4.11E+08	2.96E+08
			18703000	13476000	16925000	14214000		
11208000	7047700	8321400	10691000	6727600	8154800	9066000	47592000	11187000
1.99E+08	5.99E+08	2.9E+08	2.53E+08	2.74E+08	2.51E+08		38992000	70953000

94696000	1.24E+08	84178000	88210000	33545000	2.3E+08	2.48E+08	2.87E+08	3.09E+08
			16043000	1640300	8591400	10330000	9833700	7973100
1.34E+08	2.08E+08	1.15E+08	2.54E+08	2.69E+08	2.1E+08	2.75E+08	2.54E+08	2.92E+08
				10113000	13280000			11253000
13362000	20337000	9504000	9903200		10763000	14407000		
1.09E+08	81517000	1.05E+08	4.07E+08	3.01E+08	4.35E+08	3.15E+08	8.73E+08	7.12E+08
32641000	47364000	47977000	28729000	23989000	24770000			
1.76E+08	1.58E+08	1.27E+09	2.55E+08	1.33E+09	1.07E+09	4.69E+08	1.18E+09	1.35E+09
	1.82E+08		23695000	3.63E+08	42424000			
72354000	68395000	77042000	73625000	56729000	61078000	83350000	71491000	55601000
		33541000		28200000				
5.46E+08	4.07E+08	5.86E+08	4.3E+08	3.25E+08	2.91E+08	2.27E+08	2.16E+08	2.55E+08
				31891000	35822000			
1.11E+08	1.22E+08	96509000	98682000	85699000	75330000	69913000	82444000	67533000
48642000	40589000	52280000	44697000	45751000	30893000	16156000		53309000
3.3E+08	5.93E+08	1.88E+08	3.77E+08	6.81E+08	5.49E+08	4.09E+08	2.73E+08	3.75E+08
6E+08	4.86E+08	6.21E+08	3.14E+08	2.77E+08	3.05E+08	5.85E+08	5.62E+08	2E+08
7246700	23256000	23891000	54983000	18536000	29841000	21336000		28201000
44205000	25940000	53682000	29046000	23144000	20010000	27871000		
9.45E+08	8.48E+08	1.22E+09	7.16E+08	19713000	5.52E+08	9.88E+08	7.37E+08	7.51E+08
1.25E+09	1.33E+09	1.35E+09	1.43E+09	9.05E+08	1.04E+09	9.46E+08	1.03E+09	1E+09
	7687500							
79397000	63569000	66601000	80538000	65148000	62397000	92218000	1.81E+08	1.49E+08
			3882900	7713400	1719800			
	42098000		63114000	1.95E+08	1.24E+08	1.07E+08		37388000
83051000	74832000	72708000	56169000	95993000	19822000	52333000	45505000	50616000
12447000	13525000		21346000		16894000	27531000	23186000	
1.26E+08		1.08E+08	87045000	59770000	47334000	67271000	65721000	71942000
34812000	33688000				36938000			56744000
80365000	71092000	61660000	55086000	89867000	54655000	79738000	62486000	67474000
2.59E+08	2.63E+08	2.15E+08	1.33E+08	1.56E+08	1.96E+08	3.64E+08	2.9E+08	2.01E+08
96179000	1.49E+08	1.58E+08	29942000	2.62E+08	3.55E+08	63420000	88897000	1.62E+08
			10628000	41509000	13513000	18859000		
9.43E+08	7.49E+08	1.05E+09	1.45E+08	1.78E+08	2.19E+08	1.57E+08	1.3E+08	1.17E+08
2.52E+09	4.11E+09	4.47E+09	1.01E+10	7.57E+09	7.23E+09	9.96E+09	9.18E+09	1.1E+10
	1.58E+08	78198000	59050000	67797000		1.56E+08	1.14E+08	93386000
			95332000	1.48E+08	1.31E+08	70487000		22996000
					41854000	11422000		
						28451000		
	5.9E+08	6.14E+08			3.96E+08		2.97E+08	

9.27E+08	6.82E+08	9.93E+08	8.55E+08	6.63E+08	6.11E+08	1.26E+09	1.09E+09	1.41E+09
		1.37E+08	1.02E+08		74062000	1.37E+08	1.22E+08	1.14E+08
						1.13E+08	87665000	75337000
5.6E+08	3.33E+08	5.07E+08	2.33E+08	2.86E+08	2.06E+08	1.58E+08		98047000
1.01E+08	76298000	1.38E+08	59953000	41645000	41891000	53876000	50100000	47748000
91418000	87863000	97007000	49615000	53609000	91421000	1.74E+08	1.77E+08	97437000
	19311000	33186000	24874000	15555000	15781000	8312000		
61003000	30799000	46576000	38482000	24799000	30297000	30250000	66137000	37185000
4.82E+08	3.53E+08	1.84E+08	2.36E+08	2.14E+08	1.65E+08	2.11E+08	75961000	2.2E+08
25267000	27429000	31289000	87783000	58637000	97008000	51770000	71758000	83365000
1.83E+09	1.71E+09	2.09E+09	3.12E+09	3.6E+09	3.09E+09	3.36E+09	3.36E+09	3.99E+09
	25451000			22417000	36022000	21003000		
1.01E+08	59839000	1.49E+08	1.29E+08	1.46E+08	1.13E+08	57558000	46614000	54600000
2.09E+08	2.62E+08	2.45E+08	2.26E+08	1.66E+08	2.08E+08	2.51E+08	2.38E+08	2.61E+08
8.38E+08	1.89E+08	9.18E+08	8.46E+08	6.4E+08	5.75E+08	5.53E+08	5.94E+08	6.69E+08
1.11E+08	1.89E+08			97989000	94967000	71814000		47239000
11211000		33632000						
2.61E+08	3.42E+08	2.72E+08	3.27E+08	3.08E+08	3.48E+08	2.64E+08	2.09E+08	2.67E+08
14873000	17275000							
		15674000	69734000	83777000	50416000	1.67E+08	27402000	94648000
6.84E+08	1.47E+09	2.01E+08	5.07E+08	7.89E+08	1.05E+09	4.38E+08	1.23E+08	3.6E+08
	9671800	10823000				5412200		
3.07E+08	2.35E+08	2.81E+08	1.94E+08	1.91E+08	1.33E+08	1.78E+08	1.57E+08	1.55E+08
3.3E+08	5.53E+08	4.69E+08	4.66E+08	6.66E+08	4.66E+08	4.82E+08	4.06E+08	4.95E+08
	1.43E+08		1.09E+08	2.23E+08	1.52E+08	1.29E+08		97084000
24832000	13280000		13784000	7924300		13991000	40511000	15428000
25463000	30382000	44716000	39981000	40205000	40941000	17110000	18520000	28350000
2.52E+08	4.74E+08	1.72E+08	3.68E+08	6.49E+08	5.24E+08	2.07E+08	92947000	1.53E+08
		80283000	61629000	48962000	80388000	71014000	2.19E+08	93643000
1.2E+09	1.11E+09	1.31E+09	5.32E+08	8.06E+08	9.43E+08	1.02E+09	1.07E+09	7.36E+08
			49942000		1.59E+08			
1.27E+09	67008000	1.49E+09	76638000	2.37E+09	1.98E+09	61058000	2.75E+09	2.67E+09
			42678000	41135000	26657000			
			7229100		4362200			
2.01E+08	1.67E+08	2.3E+08	1.18E+08	1.27E+08	99880000	2.01E+08	2.81E+08	1.11E+08
1.01E+09	6.94E+08	1.13E+09	6.15E+08	4.57E+08	3.97E+08	6.87E+08	7.65E+08	6.33E+08
8443200	4821400	8553700	5348500	1962900	3516200	3347100		5124300
			20126000			26235000		
45529000	41480000	56182000	26333000	15854000	15889000	12350000	13351000	
10460000	9974000		1.3E+08	82195000	92367000	1.4E+08	1.58E+08	1.65E+08
			17429000	39898000	22824000	22674000		30702000
		56334000	96730000	63519000	92455000			1.37E+08
78030000	53746000	40618000	38985000	33382000	45758000	43653000	40254000	27789000
			1.01E+08	1.34E+08	6.17E+08	8.68E+08	1.16E+08	
				7240500				
2.85E+08	4.11E+08	3.13E+08	6.16E+08	4.11E+08	3.68E+08	5.03E+08	4.92E+08	5.45E+08

	1.24E+08		1.16E+08	1.7E+08	1.4E+08	1.44E+08		66225000
58829000	48490000	63630000		54676000	73252000			
26298000		34519000						27883000
4.87E+08	4.4E+08	5.69E+08	4.99E+08	3.93E+08	3.38E+08	2.6E+08	1.89E+08	2.46E+08
	64148000			41631000	34202000			
3.12E+08	2.5E+08	2.83E+08	2.61E+08	2.13E+08	2.25E+08	1.68E+08	1.92E+08	1.67E+08
							8680700	
8.07E+08	7.5E+08	1.26E+09	7.29E+08	8.25E+08	5.31E+08	6.5E+08	6.04E+08	6.31E+08
	96697000					54107000	37391000	
				3121000		8474400		
3.45E+08	2.22E+08	3.35E+08	2.21E+08	1.7E+08	1.49E+08	1.86E+08	1.84E+08	1.67E+08
64127000	47776000	73826000	34240000	1.03E+08	82237000			
64874000	40460000	79900000	54936000	34593000	55626000	58579000	88961000	79444000
71655000	88155000	87326000		91865000	80927000	1.16E+08	1.01E+08	1.2E+08
56185000	42433000	62246000	1.13E+08	78577000	82447000	64899000	47243000	64768000
7.44E+08	7.76E+08	8.07E+08	8.09E+08	6.65E+08	5.95E+08	8.4E+08	8.8E+08	7.06E+08
	14965000		10620000	33642000	14733000	14588000		16441000
	3.46E+08	6.28E+08	5.2E+08	3.9E+08	3.39E+08	6E+08	5.64E+08	5.49E+08
				38832000	39404000	20733000		20458000
			44839000	2.57E+08	1.54E+08	23854000		68473000
	51003000			58280000	63860000	53112000		31079000
5.22E+08	3.65E+08	6.37E+08	2.48E+08	2.28E+08	1.66E+08			1.27E+08
				30528000	40288000	17796000		
			15008000	23476000	31559000			
				14993000	9094300			
			17896000	29544000	23097000	19496000		
				17534000				
	2252500							
1.83E+08	1.17E+08		2.14E+08	1.73E+08	1.5E+08	1.71E+08	2.15E+08	8474700
1.46E+09	1.17E+09	1.66E+09	7.89E+08	6.47E+08	5.77E+08	6.25E+08	7.72E+08	5.8E+08
		22753000				17057000		12763000
	1.2E+08		19000000		18056000			
2.8E+08	2.35E+08	1.8E+08	72151000	64569000	1.49E+08	87108000	1.2E+08	1.54E+08
		12611000						
95908000	52208000	60864000	53607000	49003000	43796000	53428000	62989000	61697000
1.12E+08	1.56E+08	76036000	45014000	55460000	49340000	45985000		38912000
	21575000		21372000	12889000	6476300			14511000
				57008000		80612000		62209000
31284000	50230000		40803000	62865000	71052000	42898000	17079000	
30507000	47647000	41644000		14409000	11903000			
	36281000		59779000	1.4E+08	84671000	41694000	35237000	97711000
7628900	3432100	5463500	11384000	4763800	5682500	7679300	36095000	6136800
3.63E+08	3.01E+08	3.33E+08	1.31E+08	1.73E+08	1.73E+08	3.27E+08	2.73E+08	1.99E+08
3.52E+08	3.38E+08	4.59E+08	1.56E+08	1.43E+08	1.65E+08	3.14E+08	3.13E+08	1.72E+08
3547500	855690		1245500	567720	638210		3794300	
			4351000	2466500	2547100	4330200		3796800

1.13E+08	2.12E+08		1.64E+08	2.34E+08	3.93E+08	3.84E+08	17018000	3.03E+08
6.24E+08	5.77E+08	8.75E+08	6.05E+08	4.13E+08	3.5E+08	3.12E+08	3.12E+08	4.45E+08
1.14E+08		74614000	75606000	49841000	47375000	41538000	59786000	52497000
35163000	19515000		35779000	41444000	38763000			
88641000	42994000	37851000	1.32E+08	1.13E+08	1.03E+08	2.22E+08	1.99E+08	2.37E+08
			92980000	2.54E+08	1.85E+08	2.01E+08	62770000	1.2E+08
				45087000	27757000	23572000		24017000
2.8E+09	2.78E+09	2.72E+09	3.18E+09	3.33E+09	2.83E+09	1.49E+09	1.74E+09	2.12E+09
2.23E+08	2.03E+08	2.11E+08	1.7E+08	2.08E+08	2.19E+08	1.87E+08	93311000	91023000
				11176000				
6.06E+09	4.78E+09	4.52E+09	3.94E+09	3.08E+09	2.75E+09	5.09E+09	2.18E+09	4.91E+09
59142000	71169000	86081000	60832000	68565000	47977000	18923000	20195000	30921000
5.95E+08	4.67E+08	6.56E+08		41308000	17507000	28245000	69634000	43453000
			5165200	4103800	5399400	5230700	16289000	7301200
	4781700		7502600	9072300	8960900	9404600		
44171000	40591000	59031000	31633000	30503000	28780000			
			17412000		9530600			
								41157000
21303000	14447000	18723000	26300000	19323000	28942000	25447000	58213000	31121000
68103000								
1.19E+10	7.22E+09	1.08E+10	4.36E+09	3.84E+09	3.1E+09	2.99E+09	3E+09	2.61E+09
2.08E+08	2.86E+08		2.24E+08	2.57E+08	1.98E+08	72847000		44117000
30876000		19372000						
		60802000	3.99E+08	2.88E+08	2.25E+08	1.63E+08	3.66E+08	2.27E+08
7.31E+08	5.77E+08	8.07E+08	3.92E+08	4.1E+08	3.86E+08	2.99E+08	3.21E+08	4.3E+08
94657000	1.07E+08	1.12E+08	84249000	83082000	1.03E+08	1.33E+08	1.49E+08	1.46E+08
25647000	1.98E+08	1.71E+08	1.64E+08	1.74E+08	2.41E+08	4.18E+08	3.47E+08	2.43E+08
			11266000	7640400		19059000		14851000
			11097000	34692000	22387000			
3.47E+09	3.01E+09	3.8E+09	4.99E+09	4.29E+09	3.8E+09	4.95E+09	4.71E+09	5.75E+09
3.61E+09	2.48E+09	4.17E+09	3.54E+09	2.53E+09	2.59E+09	3.62E+09	3.93E+09	3.31E+09
2.75E+08	2.29E+08	3.25E+08	2.07E+08	1.48E+08	1.55E+08	1.71E+08	1.79E+08	1.83E+08
	7980200		27707000	11318000	9401500			10366000
46691000	69147000	19339000	17774000	14325000	83491000	19729000	77120000	1.37E+08
8.61E+08	7.63E+08	1.06E+09	8.19E+08	6.55E+08	5.35E+08	7.61E+08	7.17E+08	6.94E+08
1.67E+08	2.87E+08	8E+08	2.61E+08	2.17E+08	1.85E+08	1.28E+08	4.12E+08	2.75E+08
1.07E+10	8.79E+09	1.14E+10	6.27E+09	7.13E+09	7.44E+09	1.29E+10	1.07E+10	7.23E+09
1.02E+09	6.48E+08	1.48E+09	6.29E+08	5.64E+08	4.8E+08	4.24E+08	5.28E+08	5.02E+08
10901000								

	24975000							
90809000	1.26E+08	82344000	1.32E+08	2.06E+08	1.8E+08	1.39E+08	1.15E+08	1.1E+08
			12976000		11502000			17790000
24561000	32004000	21613000	36786000	31479000	32747000	18658000	17725000	34232000
5.98E+08	7.57E+08	7.9E+08	1.21E+09	1.68E+09	1.06E+09	9.5E+08	9.35E+08	1.19E+09
		89167000	1.23E+08	6.27E+08	5.51E+08	2E+08	1.33E+08	1.6E+08
					1.93E+08			
10312000	23180000	36212000	99635000	54633000	32057000	28602000	35459000	75953000
49381000	37352000	77931000	78460000	71196000	56696000	83080000	50628000	74910000
				18730000				
	53382000			26975000	33748000	44739000		
14770000	22594000	45320000		5088400	17124000		25561000	
20481000	40290000	16927000		63297000	54061000	36972000	36162000	41307000
					13133000			
70608000	2.88E+09	3.88E+09	3.07E+09	2.4E+09	2.07E+09	3.53E+09	2.91E+08	3.34E+09
	8072900	19253000	14971000	12860000				
58686000	39896000	77315000	36527000	30784000	31788000	14903000	15552000	18589000
	11393000	21453000	25935000	50389000	68661000	74810000	1.23E+08	1.61E+08
40988000		38320000	40230000	35961000	34507000	49207000	58047000	49876000
			20188000	13678000	10044000	8268600		
1.61E+08	2.75E+08	1.04E+08	2.49E+08	2.79E+08	2.96E+08	2.38E+08	1.99E+08	1.95E+08
			4611200		4520900	3276500	1988100	
			2985400					
			22209000	24802000	13584000	52160000	42265000	35119000
2.29E+08	2.49E+08	3.01E+08	5.94E+08	5.47E+08	4.39E+08	2.65E+08	2.57E+08	4.52E+08
		14968000	24816000	21783000	19660000	29435000	29979000	28175000
2.53E+08	1.17E+08	1.34E+08		1.28E+08	1.08E+08	1.29E+08	59970000	1.84E+08
3.24E+08	38641000	37133000	39245000	54865000	59526000	3.05E+08	3.02E+08	3.19E+08
			30508000	30749000	24044000	31230000	39034000	43193000
					15623000		7427200	15293000
7.39E+09	7.8E+09	5.25E+09	4.64E+09	6E+09	6.77E+09	5.36E+09	3.41E+09	8.82E+09
				36242000	24832000	47590000		32684000
10024000				9082300		10339000	9903500	13425000
67473000	56081000	1.16E+08	98548000	62485000	44240000	39042000	36666000	33840000
70436000	30911000	56191000	12589000	16049000	10519000		11564000	11375000
				19025000	1.08E+08			19227000
2.54E+08	1.41E+08	2.38E+08	2.47E+08	1.74E+08	1.77E+08	2.46E+08	1.92E+08	2.03E+08
52344000	1.11E+08	26458000	82168000	1.02E+08	79955000	78090000	36522000	53590000
			7471300					4497600
				13305000	10169000			
			1861000		1716500	2202100	3913200	2082700

76702000	2.44E+08	39221000	1.99E+08	2.62E+08	1.76E+08	1.85E+08	88984000	1.39E+08
2.34E+08	1.15E+08	64720000	1.17E+08	1.06E+08		1.22E+08	45423000	1.28E+08
2.24E+09	1.69E+09	2.91E+09	2.21E+09	1.92E+09	1.38E+09	2.36E+09	1.96E+09	1.91E+09
27684000	45225000		99568000	1.67E+08	1.6E+08		26500000	74686000
56766000	60830000	72532000	2.01E+08	1.34E+08	1.39E+08	6251900	2.37E+08	1.56E+08
44988000	51435000	56774000	1.3E+08	80050000	77801000	1.42E+08	1.11E+08	1.15E+08
53330000	37831000	42956000	54781000		43097000	60424000	53054000	73721000
			1.42E+08	1.39E+08	1.25E+08			1.48E+08
1.04E+08	2.27E+08	41820000	1.58E+08	2.13E+08	1.67E+08	1.56E+08	71816000	1.34E+08
4.28E+08	2.62E+08	3.86E+08	5.35E+08	4.33E+08	4.93E+08	4.03E+08	9.31E+08	5.62E+08
4.48E+08	3.59E+08	2.93E+08	1.5E+08	1.89E+08	2.25E+08	2.71E+08	2.53E+08	1.64E+08
		18646000	20949000	15010000	25619000	22239000	37110000	27911000
80044000	51229000	91105000	1.14E+08	62968000	51481000	91319000	68717000	80441000
7.19E+08	5.88E+08	7.72E+08	6.54E+08	5.08E+08	4.92E+08	5.99E+08	7.02E+08	6.76E+08
2.87E+08	2.85E+08	3.35E+08	2.54E+08	2.29E+08	1.95E+08	2.96E+08	2.86E+08	1.19E+08
1.2E+09	8.3E+08	1.08E+09	5.36E+08	3.98E+08	3.72E+08		3.09E+08	2.34E+08
4.64E+08	3.99E+08	6.78E+08	4.86E+08	4.96E+08	3.53E+08	9.34E+08	8.02E+08	8.4E+08
32259000	79165000		48664000	1.35E+08	80108000	98710000	48529000	1.12E+08
2.81E+08	1.78E+08	2.23E+08	2.79E+08	2.28E+08	1.7E+08	1.15E+08	1.6E+08	2.14E+08
				21935000				
45199000		40447000		26837000				
11059000	16639000							
2.28E+08	2.76E+08	3.14E+08	7.28E+08	4.11E+08	4.2E+08	2.92E+08	3.5E+08	4.69E+08
	39890000	39656000		53280000	46545000	43305000		40597000
5272600	4258000	31017000	43738000	33158000	29227000	47107000	82396000	55425000
3.46E+08	3.32E+08	3.77E+08	4.01E+08	3.01E+08	3.33E+08	4.04E+08	3.91E+08	4.13E+08
	24029000		85929000	1.15E+08	96780000	37528000	17674000	32073000
				48959000	34276000			18434000
2.36E+08		2.26E+08	2.17E+08	1.91E+08	1.52E+08	2.58E+08	2.51E+08	2.99E+08
33201000	24509000	43704000	45824000	28280000	24089000	39072000	29181000	30367000
69147000	1.42E+08	1.78E+08	1.98E+08	3.13E+08	3.19E+08	3.32E+08	2.99E+08	2.12E+08
						11163000		
		27112000	53044000	89072000		1.02E+08	97315000	77346000
22643000	34997000	80507000	41877000	27666000	22207000	4169800	7092400	
	28814000		14250000	38968000	13789000			
5.56E+08	4.29E+08	5.66E+08	3.9E+08	3.17E+08	3.16E+08	3.77E+08	3.51E+08	3.67E+08
4.57E+08	3.96E+08	5.09E+08	5.3E+08	4.46E+08	3.24E+08	4.58E+08	5.35E+08	4.91E+08
	21286000		36391000	62932000	45294000	44426000		33366000
			3.56E+08	4.74E+08	4.6E+08	8.08E+08	6.25E+08	
							18314000	
				18987000	13211000			17975000
8.68E+09	7.94E+09	1.43E+10	7.53E+09	7.72E+09	9.21E+09	1.54E+10	1.51E+10	1.1E+10
1.81E+08					27225000			
	32074000	1.15E+08	6362300		80978000	54495000	41995000	4997600
			19833000		21554000			23576000
	41785000	50462000	53398000	35536000	30488000	47568000	41036000	39632000
			10735000					

32280000		2.11E+08						
2.16E+08	2.08E+08	1.93E+08	1.57E+08	1.68E+08	1.17E+08	1.83E+08	1.79E+08	1.9E+08
71085000	66257000	72359000	68440000	47530000	54049000	52892000	59679000	57740000
	44335000		97320000	70961000	94339000	30968000	34253000	33635000
3.7E+08	2.91E+08	3.7E+08	3.84E+08	3.34E+08	2.91E+08	4.06E+08	4.25E+08	4.22E+08
4.05E+08	4.01E+08	4.61E+08	3.06E+08	2.73E+08	3.18E+08	6.61E+08	7.37E+08	5.52E+08
7.44E+08	4.57E+08	7.8E+08	5.74E+08	4.01E+08	3.5E+08	5.33E+08	5.61E+08	6.24E+08
17210000	14834000	33448000	49536000	28865000	28108000	31297000	62374000	79561000
51328000	49224000	64401000					39015000	
			65600000	76427000	74822000	1.28E+08	87354000	87440000
12840000	8619100	2529200	20374000	9461100	12628000	15737000	60276000	24611000
			49893000	43217000	46274000	38647000		
							979160	
86020000	1.02E+08	1.08E+08	79477000	88300000	84406000	74562000	93137000	87854000
15319000	16402000	17795000	38224000	25300000	17850000	50475000	33719000	49531000
2.82E+08	2.24E+08	3.84E+08	3.62E+08	3.27E+08	2.83E+08	6.3E+08	5.36E+08	7.79E+08
63473000	54496000	53436000	56257000	67281000		53871000	59356000	69984000
				40855000	32444000	57149000		
27476000	17470000	25846000	42073000					
			11481000			7023300		
1.01E+08	97679000	2E+08	79204000	58113000	44173000	15374000	13598000	36644000
1.56E+08	1.43E+08	1.65E+08	1.32E+08	1.14E+08		83084000	65152000	58474000
3.29E+08	4.06E+08	2.96E+08	17538000	2.89E+08	16460000	29151000	33760000	39821000
							1.04E+09	1.2E+09
	8817800		29536000	66947000	40460000	23564000	23706000	33666000
5.77E+08	4.92E+08	6.13E+08	1.88E+08	2.07E+08	2.47E+08	3.03E+08	2.52E+08	1.95E+08
26501000	60570000		2.06E+08	1.84E+08	1.7E+08	2.19E+08	1.4E+08	1.56E+08
				8219800	6172100			3147200
	11428000	8411700	7449700					
				18325000	12833000			
2.3E+08	1.52E+08	2.47E+08	1.25E+08	1.02E+08	79305000	60853000	81471000	76172000
1.2E+08	1.27E+08	1.77E+08	1.76E+08	1.88E+08	1.61E+08	1.68E+08	1.74E+08	2.43E+08
	21641000	32920000		24454000	16772000		35949000	31247000
60470000		49012000					49065000	
66104000	63399000	61643000		38603000	39739000	34199000	41073000	43251000
				1E+08		89414000	69274000	1.03E+08
9263500	72982000	82495000	16129000	25776000	49593000	34072000	67766000	56756000
					2039300		5710000	
44478000	61297000		29629000	56345000	47664000			39490000
3.27E+08	2.29E+08	2.92E+08	2.14E+08	1.56E+08	1.36E+08	1.9E+08	1.93E+08	2.01E+08
1.41E+08	3.13E+08	1.11E+08	1.5E+08	2.36E+08	1.85E+08	1.5E+08		95140000
				45380000	57768000		40207000	33114000
3.04E+08	3.29E+08	5.26E+08	5.04E+08	4.32E+08	2.65E+08	4.82E+08	5.36E+08	4.67E+08
29639000	33533000	44944000						
			16012000		12097000			9299300
				10450000	7323800			

56149000			33007000					
	10034000		9043200	11502000	10713000			
1.31E+08	98794000	1.1E+08	1.26E+08	1.02E+08	84875000	1.49E+08	2.07E+08	1.6E+08
				12250000	13251000			
83072000	97494000	1.23E+08	1.26E+08		1.2E+08	1.13E+08	1.08E+08	1.25E+08
1.14E+08	1.58E+08	1.79E+08		1.48E+08	1.24E+08		1.68E+08	1.67E+08
49346000		61795000						
				18392000	14967000	10263000	22961000	29130000
1.81E+08	95328000	1.46E+08	1.07E+08	63812000	59812000	88264000	1.02E+08	80218000
1.67E+08	4.01E+08	1.11E+08	3.51E+08	4.78E+08	4.68E+08	3.83E+08	2.56E+08	3.98E+08
4.05E+08	4.45E+08	5.26E+08	6.7E+08	9.57E+08	6.99E+08	6.74E+08	3.87E+08	6.33E+08
3.28E+08	3.99E+08	3.6E+08	3.59E+08	3.72E+08	3.76E+08	3.18E+08	2.96E+08	3.57E+08
			27562000					
59890000	62692000	69442000				1.48E+08		85455000
14688000		14426000	14377000	17224000	12290000	43811000	33805000	26156000
								9643100
1.14E+08	1.08E+08	1.33E+08	1.11E+08	78444000	79386000	1.2E+08	1.15E+08	1.1E+08
1.97E+09	1.29E+09	1.96E+09	1.38E+09	9.61E+08	9.94E+08	9.98E+08	9.1E+08	1.02E+09
3.01E+08	2.17E+08	2.64E+08	1.68E+08	1.4E+08	94237000	1.91E+08	2.81E+08	1.49E+08
69913000	39501000		49141000	36007000	39916000	55784000	62916000	60184000
76074000	57065000	97401000	48157000	45914000	36932000	33469000	36098000	38555000
11621000	15066000	13893000	19616000		26004000		19963000	28386000
69063000						56771000	52699000	
2.13E+09	1.9E+09	2.2E+09	7.58E+08	7.32E+08	8.64E+08	1.03E+09	8.59E+08	5.93E+08
1.3E+08		98096000	4.73E+08	5.3E+08	5.73E+08			
24513000	17808000	29112000	40506000	21497000	22417000	46926000	45280000	56577000
21262000	13914000	21730000	15128000	8938700	13637000	21177000	66665000	19837000
5.73E+08	4.39E+08	5.71E+08	3.92E+08	2.68E+08	2.32E+08	3.35E+08	3.24E+08	3.55E+08
1.24E+08	2.41E+08	2.3E+08	2.91E+08	3.4E+08	2.63E+08		3.57E+08	3.96E+08
32817000	14625000	21666000						
			46873000	33292000	33837000	16175000		53921000
		6.77E+08						
3.25E+08	2.99E+08	3.59E+08	8.26E+08	6.83E+08	4.28E+08	3.2E+08	4.97E+08	5.48E+08
73581000	85522000	1.23E+08	1.17E+08	84131000	84026000	1.23E+08	1.09E+08	1.07E+08
19376000	50828000		30457000	41548000		21179000	21617000	28048000
24597000	19692000	26567000	43281000	22561000	23951000	24022000	25374000	30678000
25791000	24681000	38157000	29978000	18345000	15627000	38293000	41784000	24488000
34908000	1.29E+08	2.12E+08	3.62E+08	7.93E+08	41135000	6.62E+08	3.78E+08	4.5E+08
1.01E+08	2.23E+08	1.35E+08	1.48E+08	2.79E+08	3.14E+08	1.82E+08	1.49E+08	2.03E+08
	27395000		13502000	32676000	22211000			
63712000	57188000	73118000	64616000	60064000	61990000	66945000	73052000	59155000
5.73E+08	3.98E+08	5.27E+08	4.22E+08	3.84E+08	3.23E+08	4.14E+08	4.22E+08	4.28E+08
73736000	76459000	75389000	66361000	87652000	90876000	73776000	62792000	86052000
8053000	27911000		1.26E+08	1.18E+08	1.09E+08	66207000	9098900	31632000

1.41E+08	1.27E+08	1.44E+08	47368000	59221000	48388000	46270000	40379000	35144000
			22637000	20881000			20338000	26737000
95573000	94279000	94531000	81336000	1.09E+08	1.15E+08	1.5E+08	1.47E+08	1.25E+08
38584000	21380000		29131000		17243000	18611000		20126000
	83133000	1.15E+08				24073000		
64602000	38283000	43515000	57140000	51172000	41878000	41479000	49930000	48543000
47777000		17189000						
55255000	1.18E+08	52861000	67685000	1.94E+08	1.49E+08	1.05E+08	74797000	1.6E+08
32852000	35295000	51037000		45710000	34527000	48096000	42365000	
				23466000				
	1.05E+08			76476000	68559000	1.09E+08	98424000	87563000
				15365000	16559000			
47726000	61522000	35252000	42003000	66394000	52963000	34898000		56927000
25769000	1.25E+08	3.57E+08	1.54E+08	3.03E+08	6.88E+08	1.24E+08	8.19E+08	4.89E+08
			3.43E+08	2.87E+08	2.44E+08	97974000	26320000	1.12E+08
60253000		75508000		8331900	15561000			
1E+08	74130000	1.03E+08	75279000	47854000	50116000	56464000	54641000	43971000
16452000	11702000	14969000	5869700		4219400			
		46648000						
1.41E+08	1.69E+08	3.17E+08	1.6E+08	1.77E+08	1.28E+08	1.93E+08	1.53E+08	2E+08
32801000		27838000		28917000	25557000		40632000	43208000
46521000		35309000				74963000		
68494000	59435000		1.32E+08	78181000	1.54E+08	2.71E+08	2.5E+08	1.43E+08
	1.87E+08	25293000	2.02E+08	1.33E+08	94735000	2.24E+08	1.61E+08	1.86E+08
1.31E+09	1.38E+09	1.98E+09	2.55E+09	2.41E+09	1.64E+09	3.26E+09	3.06E+09	3.38E+09
70994000						97063000		93268000
37315000	28986000	44633000	24399000	31788000	17267000			
2.35E+08	1.47E+08	2.29E+08	1.5E+08		80876000	76740000	1.02E+08	1.09E+08
				11874000	1E+08	18201000		85509000
70364000	65169000	90532000	1E+08	86534000	82689000		1.1E+08	1.07E+08
5.83E+08	7.13E+08	5.77E+08	7.45E+08	6.48E+08	5.95E+08	8.61E+08	9.54E+08	1.2E+09
5.9E+08	4.65E+08	7.07E+08	5.3E+08	4.87E+08	3.03E+08	5.07E+08	4.12E+08	5.14E+08
1.15E+08	89614000	1.31E+08	46349000	25207000	20174000	16496000	21252000	29224000
5.51E+08	4.25E+08	6.74E+08	6.16E+08	5.37E+08	4.04E+08	3.17E+08	2.79E+08	3.26E+08
1.13E+08	2.67E+08	2.7E+08	2.36E+08	3.05E+08	1.66E+08	1E+08	2.89E+08	1.78E+08
20214000	32043000	31658000	1.01E+08	94495000	81920000	96912000	81981000	64681000
8.44E+08	6.2E+08	8.48E+08	3.6E+08	4.16E+08	3.07E+08	5.64E+08	4.28E+08	4.27E+08
5.55E+09	3.65E+09	4.96E+09	5.84E+09	4.14E+09	4.14E+09	5.97E+09	6.26E+09	5.59E+09
36609000	21682000	31333000	27106000	22975000	14154000	23580000	32531000	31026000
3.44E+08	2.92E+08	4.43E+08	5.19E+08	4.32E+08	2.58E+08			5.03E+08
37019000	50105000	41111000	52483000	42038000	38338000	41795000	45109000	55623000
29194000	28415000	36253000			10118000	14964000		10620000
53650000	31203000	35439000		42660000	19873000	71114000	74681000	61633000
2E+08	1.51E+08	2.21E+08	1.87E+08	1.83E+08	1.1E+08	2.04E+08	2.09E+08	2.05E+08
2.08E+09	1.29E+09	2.48E+09	1.24E+09	9.84E+08	8.17E+08	7.79E+08	7.41E+08	7.73E+08

11189000	24887000	9907500			14732000	4350400	15810000	
6.18E+08	4.47E+08	7.14E+08	3.32E+08	3.4E+08	2.37E+08	2.97E+08	2.99E+08	3.11E+08
2.44E+08	2.05E+08	3.75E+08	1.83E+08	1.6E+08	1.38E+08	1.76E+08	1.83E+08	1.63E+08
68280000	86990000	1.05E+08	87947000	77557000	60685000	92671000	1.01E+08	98388000
1.81E+09	1.13E+09	1.76E+09	8.87E+08	6.93E+08	5.99E+08	7.49E+08	7.09E+08	6.65E+08
		25532000		27769000	24218000			
	8801500							
33121000	54479000		1.11E+08	1.56E+08	1.31E+08	45067000	29018000	17923000
1.2E+09	9.94E+08	1.27E+09	1.15E+09	8.45E+08	7.72E+08	1.01E+09	1.17E+09	1.09E+09
		24210000						23329000
25133000	55852000		56071000				1.25E+08	1.61E+08
1.42E+09	9.64E+08	1.83E+09	1.17E+09	8.94E+08	7.42E+08	5.9E+08	5.28E+08	6.31E+08
	18291000					48728000	38349000	
1.43E+08	1.27E+08	1.57E+08		95069000	69478000			
				26302000		1.51E+08	1.48E+08	1.55E+08
23472000						28967000	27149000	
1.21E+08	1.89E+08	1.1E+08	85396000	1.24E+08	1.75E+08	2.48E+08	2.36E+08	1.48E+08
	61921000	75210000	36267000	68744000	55948000	98090000	94361000	52796000
91015000	98888000	1.18E+08	74850000	57766000	74538000	1.6E+08	1.36E+08	1.06E+08
3.81E+09	3.2E+09	2.91E+09	2.04E+09	2.03E+09	2.53E+09	4.04E+09	2.5E+09	2.12E+09
					14457000			
43977000	44220000	60813000	36041000	42589000	45661000	64261000	40069000	40961000
	14977000			15179000	17203000			
	64269000	88763000	22196000			51705000	44843000	
	11495000						37989000	
22188000	14337000	13607000	8964700	8561600	13268000	22656000	40210000	14695000
	12678000			19605000	15664000	15279000		
				21065000	33028000	41491000		
	32499000			32851000	37414000	42974000		
						16065000	13017000	
	15760000		10486000					
32220000	46062000	42400000	28135000	37736000	46380000	48597000	46461000	49450000
					44440000	20988000		
		27906000						
6.92E+08	1.12E+09	9.71E+08	4.57E+08	6.01E+08	7.8E+08	72896000	90827000	5.56E+08
							2598500	
		1.19E+08						
1.88E+08	2.49E+08	1.85E+08	2.16E+08	2.35E+08	2.91E+08	4.9E+08	2.38E+08	1.74E+08
90988000								
	20872000	15065000	6319000	14413000	20479000	11504000	12152000	
	11291000				23282000			22960000
2.01E+08	1.59E+08	2.11E+08	1.55E+08	1.4E+08	1.07E+08	1.14E+08	1.13E+08	1.51E+08
11892000	7943400	8945700	6802900	4186800	6205000	9392000	24132000	12496000
13671000	26307000	21705000		5957300	20744000		18275000	24912000
34186000	53775000	52779000	1.31E+08	1.04E+08	1.29E+08	1.28E+08	1.39E+08	1.58E+08
41959000	47475000	49723000	1.14E+08	1.06E+08	1.27E+08	1.28E+08	1.19E+08	1.56E+08
			1.64E+08	10231000	1.34E+08			1.16E+08
21451000	21345000		20105000			39679000	44961000	31111000

46322000	43294000	37034000		33263000			40939000	52613000
				53630000	44606000			
40492000	96432000	45663000	1.26E+08	2.53E+08	2.39E+08	1.59E+08	1.13E+08	1.38E+08
			21129000	2.18E+08	1.75E+08	1.34E+08	57653000	37568000
1.83E+09	1.74E+09	2.16E+09	1.38E+09	1.05E+09	9.08E+08	4.66E+08	5.16E+08	4.45E+08
1.39E+08	1.26E+08	1.38E+08	3.17E+08	3.04E+08	2.76E+08	3.03E+08	3.05E+08	4.71E+08
	19103000	20474000	11769000	9956600				
1.5E+08	2.52E+08	1.69E+08	1.87E+08	1.75E+08	1.84E+08	2.71E+08	2.43E+08	2.69E+08
	2299700	4030700	4107200	2983700	4512400	3910800		3956100
				8726800	14323000			
2.77E+08	4.3E+08	3.01E+08	1.03E+08	1.74E+08	2.9E+08	2.39E+08	1.39E+08	1.09E+08
1.92E+08	1.69E+08	1.31E+08	68910000	77309000	1.25E+08	1.03E+08	1.09E+08	70629000
59625000	1.37E+08	44976000	1.22E+08	1.72E+08	1.83E+08	1.25E+08	95777000	1.08E+08
	2576100						6712000	10783000
1.44E+08	1.07E+08	1.3E+08	81882000	64897000	55500000	51015000	59116000	47772000
79977000	1.23E+08	1.06E+08	1.3E+08	2.61E+08	2.57E+08			
1.42E+08	1.24E+08	95470000	1.14E+08	1.18E+08	1.01E+08	81273000	69002000	
			29663000	23614000	21440000	35750000	31786000	38726000
13357000	11748000							
1.84E+08	4E+08	2.34E+08	1.68E+08	2.83E+08	3.45E+08	3.41E+08	2.2E+08	1.9E+08
1.09E+08	1.04E+08	1.91E+08	21037000	2.26E+08	93980000			
		3424800					7397800	2959800
				6928300				
6.14E+08	6.04E+08	6.44E+08	9.04E+08	7.54E+08	6.87E+08	9.89E+08	9.63E+08	1.33E+09
1.38E+08			1.24E+08		59941000			30860000
7.04E+08	6.29E+08	1.27E+09	1.32E+09	1.19E+09	9.32E+08	7.69E+08	6.2E+08	8.5E+08
1.53E+08	2.62E+08	2.86E+08	3.04E+08	3.19E+08	7.81E+08	6.51E+08	3.34E+08	4.19E+08
								7183800
				28308000	17881000			
		2879800	16258000	11788000	14832000	13083000	15517000	13454000
					9081600			
	26121000		16025000	18315000	14505000			
	37248000	54484000		17822000				32991000
				6512400				
	40111000		63109000		48106000	62961000		41585000
2.8E+08	3.55E+08	3.19E+08	3E+08	2.35E+08	71909000	2.01E+08	2.36E+08	2.57E+08
31968000				26042000				
					15480000			8193100
	3.28E+08	3.11E+08	4.05E+08		6.17E+08	2.38E+08	2.36E+08	2.7E+08
33958000	62209000			65061000	65648000	40434000	49925000	
27836000		28959000						
30972000	22983000	35522000	32213000	28942000	36449000	28698000	61590000	48156000
	18705000			24550000				52359000
	13339000	23363000	20859000	14973000	13317000	16042000		
12806000	11871000	14736000	27438000	17032000	17394000	27117000	26739000	28583000
19902000	9115700			17565000				
					6760800			9860200
25141000	20349000	24662000	35653000	48844000	31359000	27580000	24874000	28551000
68376000	74318000	62948000		30895000	36128000	40937000		

	2.16E+08		1.67E+08	1.51E+08	1.81E+08	95502000	74122000	1.03E+08
1.45E+08	1.56E+08	2.74E+08	5.64E+08	1.03E+09	5.89E+08	22176000	18509000	67250000
20417000				7681700	28280000			16709000
				10614000				
15687000	10759000	17290000	17931000	15249000	20081000	21172000	47847000	23557000
98852000	44292000	91370000	57270000	49796000	35621000	29379000	41614000	45320000
31854000	23920000	47197000	28501000	27304000	31695000		36898000	41176000
38970000	42550000	39911000				53495000		
62501000	78490000	1.01E+08		17964000		33697000	35781000	
			11810000					
	20616000			22399000				
4.57E+08	4.5E+08	7.46E+08	2.62E+08	3.14E+08	3.82E+08	4.48E+08	4.05E+08	3.03E+08
40048000	36623000	42209000	22038000	29940000	48678000		52662000	54494000
				30969000				
		23973000	31153000	23998000	24863000	34667000	46118000	87878000
2.35E+08	2.08E+08	3.38E+08	4.07E+08	2.97E+08	3.08E+08	3.55E+08	3.11E+08	3.33E+08
					20116000			
1.31E+09	7.02E+08	1.07E+09	1.29E+09	1.11E+09	1.1E+09	1.38E+09	1.96E+09	2.22E+08
1.4E+08	3.12E+08	1.38E+08	1.93E+08	2.88E+08	3.42E+08	2.26E+08	1.45E+08	1.84E+08
					12846000			
1.84E+08	4.43E+08	81107000	4.19E+08	1.03E+09	7.79E+08	5.02E+08	2.74E+08	4.8E+08
			69154000	52443000		77247000	73020000	54941000
					11215000	18168000		
62338000	68522000	96185000	1.73E+08	1.46E+08	1.15E+08	2.08E+08	2.16E+08	2.25E+08
				12843000				
			28291000					
66487000	68353000	63055000	50495000	46970000	41930000	31398000		36366000
		8214900	11694000	9666000	7225300			
	20596000		14621000	26750000	16169000			13251000
5.07E+08				2.59E+08	1.98E+08	1.17E+08	76794000	1.9E+08
	9515600		44238000	94722000	71323000	4105200		11969000
				3685800				
	37198000	44660000	38763000	1.51E+08	1.6E+08	1.42E+08	1.01E+08	1.23E+08
2.84E+08	2.76E+08	3.35E+08	2.91E+08	2.76E+08	2.25E+08	2.67E+08	2.42E+08	2.81E+08
8055700		24508000						
33131000	16522000	23706000	26579000	18099000	25115000	28508000	1.31E+08	31185000
	30284000		1.8E+08	1.44E+08	1.17E+08	84110000	81906000	93289000
49154000	71104000	1.01E+08	1.09E+08	73766000	27070000	1.46E+08	88121000	34788000
1.46E+09	1.45E+09	1.34E+09	1.1E+09	7.39E+08	1.05E+09	5.62E+08	8.63E+08	5.53E+08
	20774000	25744000		30941000	15946000	12869000		8753300
		16269000				23679000	16521000	
9792600			66395000	38925000	38703000	53748000	12557000	73268000
			16632000		11220000			
6.16E+08	5.44E+08	1.07E+09	6.27E+08	5.3E+08	4.54E+08	5.02E+08	4.44E+08	5.72E+08
		44090000	58095000	47661000	21145000			

40924000								
						24530000		
4.68E+08	4.45E+08	4.8E+08	3.29E+08	2.7E+08	4.08E+08	6.29E+08	6.25E+08	4.19E+08
		25452000	20017000					
		34554000		93777000			52960000	66715000
								1.26E+08
3.01E+08	3.47E+08	5.57E+08	4.41E+08	3.04E+08	2.5E+08	4.23E+08	3.32E+08	4.09E+08
3.13E+08	4.53E+08	1.02E+08	2.31E+08	2.62E+08	2.47E+08	2.96E+08	83810000	2.66E+08
			1.09E+08	1.24E+08	85616000	92416000	61205000	70454000
61867000	1.1E+08	85796000	11424000	2.35E+08	3.46E+08	2.83E+08	2.18E+08	2.81E+08
4.59E+08	5.5E+08	6.2E+08	7.21E+08	9.07E+08	5.67E+08	1.1E+09	8.55E+08	9.94E+08
36216000	46003000	40141000	1.01E+08	99670000	93649000	1.19E+08	96325000	86531000
					14009000	20688000		
			22333000				23136000	22432000
83535000	72727000	69694000	47520000	45674000	52236000	28432000	52718000	34368000
1.98E+08	1.81E+08	1.52E+08	59506000	55614000	48925000	55689000	48229000	39082000
				30901000				
75258000	74693000	92383000	53639000	63012000	43088000	71459000	1.34E+08	97965000
8224900	13905000	11237000		34710000	34353000	31648000	25106000	30355000
27329000		28414000	31044000	28757000	26175000	26381000	33694000	41395000
				40535000				
30816000	37037000	31502000	35548000	32396000	22410000	41081000	32684000	36558000
	14201000		37769000	48668000	46913000	44392000	34438000	42286000
69239000		49336000	53544000	81097000	67443000	84844000		76151000
	41930000		32761000		23420000		35321000	
49755000			13983000		14701000	35093000		
5.82E+08	6.55E+08	5.77E+08	4.31E+08	6E+08	4.82E+08	8.26E+08	5.96E+08	6.21E+08
	726830	823810	628400	322250	327480		1484500	
					43674000			71307000
4131300	3521900	2926000	4163900	2924400	3725800	4286800	10418000	5051200
12947000								
	80455000	49566000	61737000		80088000	79089000		
			1.15E+09		1.58E+09			1.2E+09
34916000	44945000	37293000	86133000	83869000	1.07E+08	95741000	89580000	1.06E+08
				33426000	28616000			
1.12E+09	2.32E+09	1.52E+09	1.15E+09	1.68E+09	1.58E+09	9E+08	1.04E+09	1.23E+09
38295000	34269000	57059000	57007000	13151000	40713000	52137000	2.32E+08	57478000
1.09E+08	93582000	77418000				2.08E+08	1.43E+08	
					2.47E+08	1.86E+08	1.2E+08	1.81E+08
43282000	62497000	98613000		14694000	39291000		28853000	17649000

11262000	19230000	14173000	27566000	19763000	26409000	41908000	25246000	34158000
			16506000					
			11418000	4127100	4078700			
3640800		1778300		519130	1139900			1200100
	6940900	4983900		4742200	3.63E+08	3.4E+08	3.4E+08	3.17E+08
			9299600	10163000	10048000	10274000		9884900
29072000	33800000	37880000		32137000	28140000			59165000
	23112000	13897000		8693200	38700000	10872000	1974300	42844000
			13953000	9803400	12393000			
2.63E+08	3.41E+08		4.48E+08	40104000	5.68E+08		34800000	5.94E+08
3.82E+08	3.19E+08	4.07E+08	5.38E+08	4.09E+08	3.29E+08	4.05E+08	4.04E+08	4.02E+08
			21010000	24650000	31794000	16756000		9794600
24697000	18864000		58711000	1.2E+08	1.32E+08	70853000	32751000	
				45883000				
			3060000				1929100	
			20505000	14050000				
			25924000	30010000	23321000	21255000		
			23819000	20561000	9139400			
			17372000		15852000			
			46506000	91123000	55393000	51979000	12173000	33740000
			1.87E+08	84946000	1.08E+08	1.14E+08	55411000	55609000
	24913000	28175000	42669000	30138000	28001000			16957000
33584000	44641000		22128000	43504000	40679000			
3.9E+08	3.99E+08	3.96E+08	3.59E+08	2.8E+08	3.42E+08	2.17E+08	2.66E+08	3.29E+08
8521400	3659100	5953700	3156300	907040	1618100	2278800	10116000	1775700
493210			649350			452120	2979300	778280
66096000	93816000	71331000	76833000	77559000	77033000	51302000	56193000	73632000
99198000	36423000	58096000	1.24E+08	1.71E+08	1.19E+08	91985000	1.3E+08	88009000
			75309000	51332000	85918000	29386000		
73124000	77290000	88522000	53081000	70335000	57760000	28067000	18543000	30032000
	14786000			30766000	30413000	23572000		22012000
		2.45E+08	1.39E+08	1.53E+08	1.25E+08	1.17E+08	1.57E+08	1.52E+08
25257000	34362000	48683000	63740000	47917000	57933000	1.11E+08	1.09E+08	83321000
	8851100		24450000	25412000	23300000	2.02E+08	2.03E+08	2.2E+08
	53925000		24881000	43480000	38478000			
							7870200	7396700
23333000	49783000	59317000		14835000	38779000		15928000	13030000
75746000	1.23E+08		1.91E+08			5.95E+08	3.6E+08	1.72E+08
1.81E+08	1.88E+08	1.92E+08	1.6E+08	1.36E+08	1.45E+08	1.48E+08	1.36E+08	1.93E+08
6353300	514010	976910	361230					420550
		37551000						
			25936000	29039000	16970000			

59921000	58964000	22437000	80140000	71666000	77639000	89278000	32417000	1.13E+08
	36910000		35284000		54858000	67310000	28137000	36270000
37904000	31948000	42851000	28868000	1554900	4279300			
	63653000		1.1E+08	72490000	77295000	1.1E+08	1.02E+08	
				49662000				
			6266800		8424700			
		15799000		7700600	8895100		14825000	
20920000	28520000	27318000	42399000		42997000	68056000	59630000	75500000
		3480200		9728600	23268000		9457400	12877000
1.44E+08	1.93E+08	1.64E+08	2.11E+08	2E+08	1.33E+08	2.31E+08	2.02E+08	2.17E+08
	16634000	10402000	18004000	18182000	18394000	18143000	16319000	20219000
73344000	95796000	61996000	26899000	21114000	61787000	42229000	30796000	18482000
82925000	1.7E+08	52251000	51290000	85201000	1.11E+08	92615000	41789000	34801000
65164000	72630000	63327000	73888000	56029000	60565000	67475000	44502000	47569000
61144000	67946000	72984000	41034000	32518000		48047000		37551000
				4142200	3605000	4313000		
				23835000	29925000			
		32540000	29702000	35905000	36952000	44097000	51406000	47832000
				36186000	40989000			49328000
40968000	88390000		28156000		26146000	54902000	15899000	
1.35E+08	1.32E+08	1.63E+08		74328000	71360000			67039000
68580000	51633000	80325000	96024000	73677000	66963000	71149000	70240000	1.03E+08
42826000	18302000	79080000	33846000				43016000	
3.56E+08	4.1E+08	4.05E+08	3.83E+08	3.43E+08	3.39E+08	3.6E+08	4.61E+08	3.58E+08
1.02E+08	1.14E+08	1.03E+08	87251000	98623000	87262000	70195000	64617000	74797000
8.18E+08	8.42E+08	7.65E+08	8.47E+08	9.6E+08	1.16E+09	1.05E+09	1.37E+09	1.11E+09
13253000	51541000				50605000	54689000	39291000	
26641000		15478000	14752000	12855000	13596000	13602000	50018000	14886000
24582000	39634000	35285000	71194000	67200000	50651000	71362000	72174000	86252000
			26203000	47750000	31597000	26795000		19810000
			18689000		10701000	14099000		7609600
4.83E+08	4.57E+08	4.15E+08	5.72E+08	3.12E+08	4.14E+08	4.6E+08	3.21E+08	3.51E+08
	1327000							
1.76E+08		2.03E+08	4.21E+08	2.16E+08	2.34E+08	3.83E+08	3.77E+08	3.42E+08
9915600	8120100		15070000	15334000	18568000	11129000	17173000	12783000
56692000								
6.33E+08	8.14E+08	7.83E+08	7.59E+08	1.26E+09	7.95E+08	3.95E+08	3.96E+08	5.15E+08
	12207000				19957000			
			39217000	79289000	47894000			
66042000	1.03E+08	93579000	64229000	74289000	74614000	43220000	31027000	49523000
	19649000		28102000	25177000	29771000	19949000	18398000	15927000
54811000	67751000		56088000	54858000	85742000	42166000	40216000	60339000
59017000	80602000	94301000	92805000	68117000	67264000	76679000	50569000	72582000

1.07E+08	2.35E+08	1.19E+08	1.41E+08	2.02E+08	1.44E+08	88104000	31915000	87624000
	45455000							
			14094000		15408000			12938000
1.02E+08	83478000	1.09E+08	1.12E+08	1.03E+08	83226000	1.08E+08	97837000	1.33E+08
37689000	74728000	23220000	41425000	1.08E+08	92043000	32538000	16521000	39351000
1689800	2144300	3290300	6186400		3644400		21760000	5680800
			21786000		24513000		21289000	
12384000	8665900	9406000	11171000	4019500	5975100	5752300	27024000	7263600
3.91E+08	3.96E+08	4.39E+08	2.3E+08	3.34E+08	2.4E+08	2.42E+08	1.97E+08	3.72E+08
11322000	4724700	6580600	10548000	2726000	4352400	6459500	39747000	5488200
			7688800	11837000	14501000	10999000		
	15764000							
					8389800			
					11108000		19227000	15978000
2.27E+08	1.4E+08	1.93E+08	1.53E+08	2.27E+08	1.63E+08	36367000	45847000	82066000
	23901000		21932000	25457000	34885000		18675000	14038000
1.58E+08	1.48E+08	1.62E+08	1.92E+08	1.64E+08	1.69E+08	2.55E+08	1.67E+08	2.25E+08
				53041000	58080000	53718000		
1.79E+08	2.41E+08		3.98E+08	1.76E+09	1.8E+09	1.28E+09	6.71E+08	1.23E+09
				27893000				
1.33E+08	1.37E+08	1.15E+08	1.17E+08	1.04E+08	82411000	1.12E+08	1.06E+08	1.19E+08
	24521000	43440000		18076000	18394000		12272000	
					18096000	1.79E+08	1.52E+08	2.21E+08
3744000	295060	512670	287580			333280	1544000	409760
								10579000
2.32E+08	4.01E+08	2.65E+08	5E+08	5.93E+08	5.8E+08	5.24E+08	2.58E+08	5.02E+08
51124000	39543000		2E+08	1.41E+08	1.25E+08	1.95E+08	97348000	1.47E+08
			48999000	6682600	18599000	27477000	11412000	
1.28E+08	1.01E+08	91115000	1.34E+08	2.34E+08	1.39E+08	2.62E+08	1.03E+08	2.77E+08
56568000	43415000	54825000	55763000	49694000	42225000			39639000
		8753600						
7.21E+09	6.05E+09	6.46E+09	3.19E+09	3.83E+09	5.42E+09	9.4E+09	7.22E+09	4.6E+09
72370000	53018000	75877000	39180000		34730000			
5412600	3286400	4221200	3059100	1993200	3028200	2946000	12681000	4172800
5.95E+09	5.32E+09	5.1E+09	3.97E+09	3.37E+09	3.25E+09	4.74E+09	4.88E+09	5.34E+09
								36849000
27848000					11475000		31462000	
3.13E+08	2.14E+08	3.38E+08	1.72E+08	1.55E+08	1.44E+08	1.25E+08	1.6E+08	1.59E+08
	14405000							
				4160000	8718600	3577600	13212000	7334400
	11762000		17119000		25620000			
	8717800	5117700		4510500	5702000		8241900	7329300

28235000					18583000	20475000		
	36897000				23106000		26919000	
58294000	1.02E+08	59538000	31560000	54796000	61670000	51914000	61071000	34058000
88073000	1.01E+08	1.45E+08	1.2E+08	1.06E+08	1.02E+08	1.15E+08	1.41E+08	1.57E+08
					1.07E+08	81750000	82189000	1.24E+08
4.84E+08	9.39E+08	1.2E+09	5.36E+08	5.97E+08	6.78E+08	3.32E+08	7.92E+08	7.99E+08
53340000		52832000	50425000	97608000	67934000	31186000		64627000
50880000		74991000	58385000	61688000	80043000	83819000	1E+08	
				10568000	21898000	15922000		9157800
			33274000	31900000	29576000			
								40552000
1.12E+08	2.1E+08			2.64E+08	1.38E+08			
	9249200			14019000	16809000	27148000		16645000
			15420000	9696900	10977000	16006000	11366000	13700000
					6509100	10211000		
3.13E+08	2.39E+08	2.5E+08	2.88E+08		1.61E+08			
	27594000		36109000	45690000	48105000	19325000		
1.42E+08	1.55E+08	1.43E+08	1.47E+08	1.75E+08	1.24E+08	70111000	72575000	82843000
78027000	58127000	1.03E+08	77034000	63087000	64217000	79128000	1.02E+08	56315000
5.2E+08	5E+08	9.24E+08	5.68E+08	5.23E+08	7.69E+08	2.99E+08	6.3E+08	5.46E+08
39038000	41226000	43114000	53868000			37054000	36661000	31766000
3E+09	2.32E+09	4.22E+09	2.2E+09	2.56E+09	2.56E+09	1.04E+09	4.13E+09	3.44E+09
				24688000				
	12424000			10859000	13014000	9556200	10735000	13034000
65205000		1.08E+08	59022000					
					17396000			
	24397000		71756000	53847000	45564000	54968000	71021000	70508000
				4932300				
	12418000			7762400	9568100			8873500
	17448000	15513000		29185000			20374000	87070000
		40106000			2805400		10652000	21617000
			17029000	16468000	13385000			
2.24E+08	1.91E+08	2.3E+08	3.53E+08	3.41E+08	3.04E+08	4.02E+08	3.56E+08	3.75E+08
	16360000		22602000		40503000	28577000	13583000	22277000
9958700		16711000	15950000	14860000	14356000	11190000	27902000	13828000
		26891000	62845000	48546000		83287000	81107000	64458000
1.69E+08	1.55E+08	87137000	1.17E+08	1.43E+08	1.97E+08	2.07E+08	1.99E+08	1.55E+08
	5338900	7025300	2373200	681180	1674600		7883000	2004900
1E+08	1.03E+08	87154000	75735000	79590000	73500000	71608000		77248000
	17453000	27999000						

30999000	19480000		7855900		10393000	7108300		7161600
			18700000	7005300		13875000	16974000	18844000
10872000		12426000			4510800			
	40719000				43369000			
33142000	22441000				21050000	30729000	38899000	29540000
8.6E+08	1.19E+09	1.17E+09	1.09E+09	8.53E+08	9.07E+08	1.22E+09	1.34E+09	1.12E+09
							10583000	
9937000	5420500	8287700	6407800	4495000	6619500	6440800	20788000	8767800
85597000	1.14E+08	1.58E+08	1.27E+08	1.03E+08	79201000	1.67E+08	1.26E+08	1.35E+08
	12218000	15221000	29082000	31911000	18414000	26947000	27079000	17138000
1.93E+08	1.2E+08		69011000	62441000		68972000	78943000	
			7.63E+08	4.89E+08	4.08E+08			
	4171100	3060400		2741400	4452100		1994400	5654700
1.27E+08	1.05E+08	1.58E+08	67844000			43295000	98571000	
5.36E+08	3.04E+08	4.05E+08	2.84E+08	2.41E+08	2.74E+08	2.97E+08	2.95E+08	2.68E+08
					83454000			
2.35E+08	3.97E+08	43085000	2.91E+08	2.89E+08	3.26E+08	2.76E+08	81668000	1.74E+08
2.09E+08	1.78E+08	2.03E+08	1.7E+08	1.35E+08	1.12E+08	1.72E+08	1.68E+08	1.47E+08
						28920000		45528000
					4983500			
29622000	30557000	49690000	44785000	56815000	44490000		28166000	
						16395000	36824000	28168000
29852000	37584000			47802000	32513000			
		33358000						
			34612000	49575000	36995000	23123000		
2.82E+08		2.61E+08	3.5E+08	4.02E+08	3.58E+08	3.6E+08		4.17E+08
1.14E+08	1.42E+08	1.05E+08	2.39E+08	3.25E+08	2.26E+08	4.16E+08	2.16E+08	2.47E+08
46161000	25800000				22156000	34318000		
8.97E+08	1.01E+09	6.96E+08	1.33E+09	1.96E+09	1.53E+09	1.79E+08	1.56E+08	1.48E+08
1.08E+08	99706000	1.21E+08	36294000	68583000	50284000	39519000	47327000	37984000
3.21E+08	4.14E+08	3.17E+08	3.3E+08	2.89E+08	2.93E+08	2.92E+08	3.2E+08	2.94E+08
2.8E+08	2.12E+08		3.52E+08	2.83E+08	2.11E+08	3.5E+08	3.41E+08	3.68E+08
			20548000	9702600	9851800			
60197000	56293000		48652000	61031000	86140000	67465000		19886000
13713000		19544000	25642000	24411000	17990000	21329000	24166000	19696000
47015000	1.08E+08	73633000	50206000	1.09E+08	1.1E+08	71057000		
	8.67E+08	42527000	75270000	1.86E+09	1.45E+09	1.01E+09	7.33E+08	1.04E+09
3.1E+08	7.26E+08	4.76E+08	8.25E+08	1.03E+09	8.39E+08	1.09E+09	7.87E+08	1.2E+09
				1.17E+08			15930000	28966000
	12682000	10648000	10582000	12705000	11215000	12497000		17291000
					14972000			

				12456000	8452600			
19465000	24424000	23851000	25719000	50006000	36754000			
			24870000	37869000	30701000	23205000		
86941000	1.04E+08	1.45E+08	1.03E+08	74672000	70827000	51014000	65551000	70000000
				14073000	13586000	22535000	27193000	32079000
					7238900			
	1.25E+08	1.29E+08	75081000	1.44E+08	1.03E+08	71455000		47712000
38416000			39555000					
1.1E+08	1.96E+08		86190000	1.4E+08	1.57E+08	79162000		81693000
			35769000					
		8581200						
				26567000	37509000	40094000		
1.1E+10	5.09E+09	9.25E+09	5.92E+09	4.42E+09	4.86E+09	4.61E+09	6.75E+09	6.79E+09
				9918000				
5.03E+08	5.12E+08	7.22E+08	2.74E+08		1.67E+08	2.29E+08	2.23E+08	2190800
1.84E+09	1.27E+09	1.97E+09	8.36E+08	7.04E+08	5.56E+08	6.11E+08	4.61E+08	4.15E+08
			41058000	1.07E+08				
30811000	16597000		17681000			15560000	34922000	36343000
	6218600		25460000	23347000	24737000	25655000	7982500	12107000
3.99E+08	5.02E+08	4.99E+08	2.19E+09	1.99E+09	1.74E+09	2.5E+09	2.31E+08	2.74E+09
				44301000	26151000	43215000		
2.42E+09	1.64E+09	2.17E+09	9.87E+08	1.8E+09	9.11E+08	2.3E+08	1.57E+08	2.66E+08
				42431000	56586000			
4.18E+08	2.63E+08	3.48E+08	1.5E+08	1.57E+08	1.85E+08	3.04E+08	2.94E+08	1.8E+08
		20918000		13301000			62239000	24678000
3.09E+08	3.4E+08	3.39E+08	1.86E+08	1.65E+08	1.44E+08	1.4E+08	1.33E+08	1.53E+08
1.44E+08	94222000	1.1E+08	74341000	78924000	1.41E+08	2.65E+08	2.47E+08	1.14E+08
				18036000				
9759700	9322600	17251000		4909000	5088100			
	1079700		2082700			1717500	3883200	1269600
							3025300	1.65E+08
34961000		31852000						
26339000	29730000	27320000	40357000	29247000	30097000	51727000	64088000	44901000
1.38E+08	1.05E+08	2.06E+08	1.11E+08	94602000	84374000	1.08E+08	1.04E+08	1.14E+08
			3609700	1985200	3601800	3275700	3638800	
				23230000		21668000		17241000
1.89E+08	92938000	1.19E+08	1.78E+08	1.47E+08	97507000	2.04E+08	1.99E+08	1.77E+08
82582000	89297000	75760000	65300000	85227000	60595000	77081000	72839000	1.61E+08
6.22E+08	4.67E+08	6.88E+08	5.99E+08	4.81E+08	5.23E+08	7.94E+08	4.99E+08	7.38E+08
				18505000	10678000			
2.68E+08	2.32E+08	2.29E+08	2.06E+08	1.99E+08	1.6E+08	1.07E+08	1.4E+08	1.63E+08
43197000	37099000	46665000	67993000	77034000	61909000	57253000	65817000	94435000
1.22E+08	1.36E+08	1.11E+08	88432000	80431000	85754000	34098000	45297000	49041000
					17366000	17577000	24345000	46031000
			17019000	46478000	35270000	40346000		
85242000	1.19E+08	79098000	1.97E+08	1.48E+08	1.51E+08	1.49E+08	1.41E+08	1.98E+08
26447000	57286000	7865400	68129000	51185000	53537000	67525000	81424000	88388000

67437000		39855000						
10966000	22588000	17559000	13824000		20952000	13000000	15142000	
3.13E+08	2.72E+08	2.86E+08	2.54E+08	2.41E+08	2.09E+08	3.89E+08	2.75E+08	3.5E+08
5.61E+08	6.54E+08	7.8E+08	1.1E+09	1.26E+09	1.02E+09			1.53E+09
93040000	2.02E+08		1.72E+08	4.83E+08	3.81E+08	2.45E+08	1.02E+08	1.93E+08
13202000		12756000	9666100	4284400		12156000	16882000	13506000
6.82E+08	5.96E+08	7.99E+08	6.87E+08	5.64E+08	1.34E+09	9.64E+08	1.15E+09	3.58E+09
30266000	40750000	31237000	66472000	91610000	61202000	2.24E+08	3.3E+08	3.04E+08
30276000	29451000		31769000			35398000	25533000	
	85429000				40703000			
					18226000			
1.25E+08	3E+08	65833000	2.61E+08	1.92E+08	1.49E+08	2.13E+08	1.51E+08	2.2E+08
28953000	15604000	29997000	29911000	83091000	50896000	8261500		12232000
1.66E+08	6.51E+08	6.39E+08	1.27E+08	81683000	71514000	57657000	1.55E+08	1.44E+08
19348000	12089000					22264000	24189000	28478000
						32426000		31185000
	1.08E+08	1.79E+08	75291000	62294000	45063000	15942000	17374000	21393000
	48005000	29305000		29735000	23144000			
2.48E+08	2.01E+08	2.17E+08	1.03E+08			2.06E+08	1.85E+08	95342000
42015000	47234000	50802000	45798000	40762000	29411000	21763000	34945000	46409000
76783000	55124000	67564000	52441000	65524000	1.01E+08	43187000	46682000	36543000
					8733100			
1.37E+08	94497000	2.1E+08	38819000					
	14063000		31049000	25764000	21922000	42146000	49799000	35192000
12023000	8676600	10747000	11170000	6127200	7969900	14335000	39743000	13932000
40758000	67803000	65185000	79685000	74929000	73733000	55154000	53531000	71934000
42501000				21628000	36013000	38173000	30111000	25386000
							31040000	
93552000	86013000	81888000	1.55E+08	1.46E+08	1.31E+08	1.26E+08	1.3E+08	1.4E+08
82387000	59713000	1.05E+08						
1.76E+08	1.26E+08	2.61E+08	43680000	44377000				
3.27E+08	3.37E+08	3.33E+08	3.59E+08	4.09E+08	5.6E+08	8.81E+08	6.82E+08	4.92E+08
7749200	12844000		32264000		49749000	13952000	9477300	
57117000			1.41E+08	91657000	92384000	93936000	1.08E+08	1.15E+08
1.12E+08	72031000	1.31E+08	65116000					
			19538000	13296000				
		34772000	37925000	39810000	25552000	39804000	5839300	34869000
1.81E+08	1.66E+08	1.9E+08	1.9E+08	1.33E+08	1.05E+08	2.1E+08	1.64E+08	2.06E+08
3.28E+08	2.77E+08	3.55E+08		1.17E+08	1.15E+08	2.56E+08	3.18E+08	2.86E+08
51455000								41698000
			1366700	598350		1183500	3451500	1389900
7.16E+08	4.88E+08	94657000	5.88E+08	1.01E+08	1.64E+09	40499000	7.41E+08	1.94E+09
1.3E+08	1.03E+08	1.24E+08	1.21E+08	1.04E+08	75252000	99859000	76110000	96483000
3.02E+08	2.47E+08	2.99E+08	2.75E+08	3.19E+08	3.38E+08	2.83E+08	2.93E+08	3.46E+08
9171600	267660	5679800		999260		1594500		1290300
77652000	1.32E+08	54320000	1.02E+08	1.41E+08	94317000	83288000	93915000	87163000
	2134800	3942800	10215000	1793600	2346100	7221100	32781000	8468000

						34229000	38496000	38239000
	24416000	29517000	19399000	15520000		26561000		27577000
62397000		58310000	46792000				44387000	48530000
					14657000			19524000
	16657000	31666000						
				12592000	9344500			
4.24E+09	5.09E+09	5.39E+09	6.78E+09	7.06E+09	5.94E+09	6.25E+09	5.48E+09	6.13E+09
6.36E+08	6.23E+08	9.66E+08	9.28E+08	7.16E+08	6.14E+08	1.09E+09	1E+09	1.31E+09
				9143000				12718000
1.94E+08	1.49E+08	2.02E+08	1.42E+08	1.67E+08	1.36E+08	2.22E+08	1.86E+08	1.73E+08
1.3E+08	2.61E+08	96429000	1.82E+08	2.11E+08	2.41E+08	2.24E+08	1.26E+08	1.78E+08
		15414000	11123000					
73929000	76353000	1.13E+08	72252000	53516000	47442000	67825000	57450000	84791000
33306000	20613000	26399000	52915000	41312000	56734000	47008000	1.06E+08	80341000
5.89E+08	4.41E+08	5.32E+08	3.82E+08	3.02E+08	2.65E+08	4.26E+08	3.4E+08	3.72E+08
40515000		38807000				18106000		
3.07E+08	3.23E+08	5.55E+08	2.4E+08	1.89E+08	1.93E+08	2.17E+08	1.95E+08	2.25E+08
89574000	79295000	1.17E+08	38697000	38273000	46420000	62771000		45634000
								22439000
2.07E+08	1.3E+08	1.8E+08	73601000	67721000	64308000	35083000	48160000	41766000
2E+08	1.67E+08	2.52E+08	1.95E+08	1.47E+08	1.32E+08	1.5E+08	1.34E+08	1.13E+08
	16466000	17322000		17144000	16736000	19194000	35145000	27317000
	15555000			10928000	13141000			
				11677000				9850600
	47198000				75852000			
1.06E+08	92163000	1.02E+08	79880000	88896000	79795000	48774000	35457000	48933000
				11265000	13838000	18601000		11849000
			21528000			30366000		
3.11E+08	3.34E+08	2.99E+08	2.34E+08	2.15E+08	1.74E+08	2.27E+08	2.34E+08	2.26E+08
				17123000		34151000		
		19722000			23132000			
		14245000	14191000	5599600	12720000	6121600		
72608000	96667000	1.1E+08	42622000	1.1E+08	93221000	61944000	1.05E+08	61570000
64134000	1.5E+08		81981000	93292000	18357000	1.13E+08	32509000	47823000
64386000	89916000	1.06E+08	68710000	82139000	87796000	83888000	1.1E+08	1.15E+08
26465000	54214000	47225000			25897000		22658000	
1.92E+08	1.57E+08	1.65E+08	1.44E+08	1.33E+08	1.27E+08	1.7E+08	1.65E+08	1.94E+08
13738000		30735000			19065000			
2.25E+08	2.36E+08	3.07E+08	6.82E+08	5.35E+08	4.71E+08	6.06E+08	7.48E+08	4.61E+08
2.35E+08	2.44E+08	3.09E+08	2.33E+08	2.4E+08	2.07E+08	2.48E+08	2.48E+08	2.87E+08
				22377000	16887000	17862000		
5.07E+08	5.59E+08	5.3E+08	3.24E+08	3.08E+08	3.11E+08	3.57E+08	4.24E+08	3.94E+08
8.87E+08	1.22E+09	1.02E+09	9.45E+08	1.39E+09	1.26E+09	4.99E+08	3.23E+08	6.6E+08
1.38E+08	2.75E+08	2.73E+08	1.65E+08	1.44E+08	1.58E+08	1.54E+08	1.69E+08	1.22E+08
4.99E+08	2.46E+08	4.14E+08	1.8E+08	2.38E+08	1.2E+08	1.23E+08	1.08E+08	1.13E+08
52296000	87037000	81314000		27420000	36788000	46388000	42589000	41501000

53542000	79100000		47200000	47609000	44536000	51838000	23226000	25675000
2.9E+08	2.45E+08	2.94E+08	3.27E+08	2.35E+08	1.93E+08	1.63E+08	1.52E+08	2.31E+08
64472000	44340000	72241000	41538000		40397000	34613000	42685000	35722000
			8493900	9498800	12009000	16928000	24395000	18613000
37018000		35836000	59089000			69836000	61836000	63530000
			34649000	32826000	26435000			
88587000	65816000	63306000	29707000	20018000	19760000			
3.26E+08	2.94E+08	3.39E+08	2.08E+08			1.63E+08	2.27E+08	
80180000	55370000	76669000	1.08E+08	89305000	79318000	1.3E+08	1.11E+08	1.11E+08
53995000	56418000	64499000	17892000	31551000	68373000	65225000	1.14E+08	50250000
1.66E+08	1.66E+08	1.99E+08	1.72E+08	2.24E+08	2.06E+08	4.12E+08	2.83E+08	2.35E+08
30647000	53255000	37760000	79021000		78812000	73261000	74718000	94467000
4.39E+08	3.42E+08	4.42E+08	2.77E+08	2.19E+08	1.97E+08	2.64E+08	2.82E+08	2.46E+08
		28587000	19019000	29439000				21928000
	54343000	89674000	63500000	76458000	61232000			30709000
		1248100	1728300	1442500	1552300	1718400	2090500	2039500
							12841000	19480000
			19464000	58794000	40690000			
			23965000	17677000				
				6096600				20623000
			15400000	15599000		18184000	19697000	
1.89E+08	2.01E+08	2.85E+08	3.13E+08	91188000		3.04E+08	2.7E+08	2.76E+08
28213000	1.07E+08			1.26E+08	98801000	54581000		
5.22E+08	5.62E+08	5.02E+08	5.14E+08	4.37E+08	4.12E+08	6.16E+08		5.56E+08
9876000		8638000	3576900					
2.66E+08	2.31E+08	3.23E+08	2.4E+08	2.47E+08	1.79E+08	2.43E+08	2.58E+08	2.55E+08
	40034000	12796000			19878000	24925000	13246000	
75799000	99004000	79814000		48351000	53164000	38729000		27276000
39689000	55336000	31505000		10556000	12365000	25175000		
81082000	1.2E+08	1.24E+08	94286000	1.56E+08	1.2E+08	1.7E+08	4.55E+08	4.2E+08
35827000	36964000					12850000		
			24492000					28308000
	15799000							
20426000	43931000		63008000	1E+08	56533000	60868000	29717000	73963000
15491000	17399000	13105000				21975000	12495000	
2.06E+08	2.93E+08	1.97E+08	1.35E+08	1.82E+08	2.7E+08	2.49E+08	1.83E+08	3.15E+08
	33465000		2.97E+08	2.38E+08	1.85E+08			
34044000	53119000	62585000	35605000	49675000	56316000	83749000	98743000	52574000
			4118500	21240000	5555300	9665000		17069000
42246000	37321000	26061000		20394000	21803000			
			49794000		66317000	93686000		59434000
1.52E+08	2.46E+08	1.23E+08	98140000	2.59E+08	1.68E+08	2.21E+08	1.81E+08	1.32E+08
	17672000			28690000	20124000	17668000		13221000
	23452000							
31529000	1.37E+08	79710000	41919000	80618000	7.24E+08	6.34E+08	72701000	94100000

26200000	40471000	28824000	24447000	47041000	56876000	52596000	47141000	43935000
32617000	39409000	33496000	29232000	26949000	33853000	50324000	41607000	39052000
20091000	22915000		15687000	16774000		26281000	19769000	
63949000	63947000	40808000	8123000	39404000	25783000		18645000	14946000
				10787000				
30104000	33678000				25723000	44611000		
	51116000		15346000	27169000	58024000	29208000	32847000	28371000
	20979000	9946900			11707000	14467000	11632000	
	32354000			32066000			29214000	22022000
					18715000			
							10406000	
		42930000		34322000	38318000	35930000	38102000	52682000
24683000	27203000	27282000				26589000	16520000	14909000
	67182000							
1.39E+09	1.21E+09	1.46E+09	8.83E+08	8.68E+08	1.1E+09	9.72E+08	8.94E+08	7.03E+08
						25890000		9804300
2.6E+08	4.88E+08	2.75E+08	1.49E+08	2.7E+08	2.54E+08	75028000	25295000	61658000
50526000	52201000	50025000	55984000	54281000	68197000	1.3E+08	98295000	86701000
2.17E+08	4.18E+08	2.16E+08	3.77E+08	7.74E+08	6.26E+08	2.23E+08	1.22E+08	2.31E+08
					14727000			
							5730600	
93896000	61466000	1.02E+08	13258000	41912000	63304000	8265000		27685000
	23472000	28691000						
16459000				1.2E+08	1.16E+08	2.06E+08	54824000	94551000
	28122000		11794000	20495000	23855000			
	14495000	20982000	6912100	13094000	11726000	12361000	10817000	16691000
18787000	54625000	89235000		9942600	37827000		18039000	20405000
								11095000
	79023000	33077000	27900000	33082000	58168000	40406000	4834200	19390000
	74325000				25998000	22802000	25154000	23773000
46833000	33852000	42566000		20984000	48657000		23837000	
	13297000	29654000		7817500	6341200		11954000	8642800
						54333000		
2.23E+08	2.23E+08	2.21E+08	91197000	1.22E+08	1.59E+08	1.52E+08	1.28E+08	1.04E+08
84143000	1.01E+08	82068000	63546000	58742000	72106000	1.51E+08	1.41E+08	92808000
56127000	1.09E+08	1.87E+08	52330000	1.29E+08	1.19E+08	94746000	1.29E+08	1.73E+08
				41770000	20613000	11302000		
10828000	95160000	1.15E+08	9221500	26189000	45470000	33991000	46181000	39569000
1.02E+08	1.47E+08	1.31E+08		1.7E+08	2.7E+08	3.97E+08	2.77E+08	1.75E+08
[truncated: 2,384,718 more chars]
