# Supplementary material for: Comprehensive Temporal Protein Dynamics during Postirradiation Recovery in Deinococcus radiodurans
Source: Oxid Med Cell Longev. 2022 Nov 11;2022:1622829. doi: 10.1155/2022/1622829 (PMC9674996; doi:10.1155/2022/1622829)
Supplement: Supplementary file 3 [file 1622829.f3.odt]

Table S3. Differentially abundant proteins in the comparison Control_0h vs Radia

Uniprot Accession	Gene name	Protein name	LFQ intensity C_0h_1
Q9RR76	gpsA	Glycerol-3-phosp	25.0055599212646
Q9RTF3	gcvH	Glycine cleavage	27.0190315246582
Q9RXH2	rpsO	30S ribosomal pro	26.1662616729736
Q9RRS0	DR_2415	DNA-binding resp	27.7058944702148
Q9RTV2	DR_1654	Uncharacterized p	30.7208576202393
Q9RUT0	potA	Spermidine/putres	23.5644187927246
Q9RUU1	DR_1290	Extracellular solu	30.5729885101318
Q9RVA3	DR_1126	Single-stranded-D	25.0542831420898
Q9RVF7	DR_1072	Acetyl-CoA acety	24.8740367889404
Q9RYN0	DR_A0281	Uncharacterized p	29.2799205780029
Q9RZQ2	DR_B0068	Uncharacterized p	28.8407707214355
Q9RZQ3	DR_B0067	Extracellular nucl	29.7583236694336

ation_0h

Control_0h		
LFQ intensity C_0h_3	LFQ intensity C_0h_4	LFQ intensity R_0h_1
25.1215114593506	25.2337779998779	26.5943431854248
26.5640258789063	26.2168598175049	28.3275318145752
26.4190864562988	27.2291259765625	25.420597076416
27.7614822387695	27.9516048431396	26.9186611175537
31.3398990631104	31.5806903839111	30.5890655517578
23.9490451812744	23.7931270599365	25.3286533355713
28.984245300293	30.7226505279541	32.0842971801758
25.8267498016357	25.9555778503418	27.1752185821533
24.397216796875	25.2952651977539	26.3906555175781
29.703685760498	30.1378498077393	28.6342258453369
28.7109928131104	29.055534362793	26.569845199585
29.9346656799316	30.0284156799316	25.5870914459229

Radiation_0h			
**LFQ intensity R_0h_2**	**LFQ intensity R_0h_3**	**Peptides**	**Razor + unique peptides**
26.0883502960205	26.329610824585	3	3
27.3994178771973	28.1240577697754	4	4
24.7554702758789	25.3382415771484	3	3
26.4420871734619	26.5555229187012	4	4
26.8048896789551	30.5806446075439	1	1
24.567554473877	25.5482196807861	3	3
31.5938816070557	31.3197479248047	4	4
26.3167743682861	27.9270534515381	4	4
26.4616088867188	26.2092933654785	6	6
28.3248729705811	28.5274600982666	2	2
26.3752574920654	26.7576084136963	22	22
26.9121646881104	25.4453754425049	24	24

Unique peptides	Sequence coverage [%]
3	19.8
4	59.2
3	47.3
4	22.5
1	14.8
3	15.5
4	11.4
4	8.5
6	29.5
2	27.4
22	42.6
24	32.5

Unique + razor sequence coverage [%]	Unique sequence coverage [%]
19.8	19.8
59.2	59.2
47.3	47.3
22.5	22.5
14.8	14.8
15.5	15.5
11.4	11.4
8.5	8.5
29.5	29.5
27.4	27.4
42.6	42.6
32.5	32.5

Mol. weight [kDa]	Q-value	Score	MS/MS count	Student's T-test p-value C_R
33.597		231.24	88	0.00161340927783919
12.774		69.315	39	0.0208546942690015
10.151		323.31	113	0.0200541799030878
26.099		143.13	82	0.00195750841198117
18.746		225.23	80	0.215726415359457
39.84		233.56	33	0.0122038473739371
57.533		222.91	98	0.0585892580538098
74.218		300.45	78	0.0483436667911765
40.629		54.683	89	0.00516347064930973
11.442		323.31	106	0.0100788363661427
94.671		323.31	332	0.000103209612238386
109.78		323.31	454	0.00115812856356783

Student's T-test q-value C_R	Student's T-test Difference C_R
	-1.2171516418457
	-1.35036341349284
	1.43338839213054
0.0523636363636364	1.16757011413574
	1.88894907633464
	-1.37927881876628
	-1.57268079121907
	-1.52747853597005
	-1.49834632873535
0.048	1.21163241068522
	2.30152893066406
	3.9255911509196

Fold change C_R	Student's T-test Test statistic C_R
0.430131101798783	-0.563421445048821
0.392193243403687	-0.570961947244392
2.70080296340888	0.601539496435904
2.24633035789445	0.540081195641165
3.70365335774555	0.574890792453357
0.384410908447364	-0.595175414162279
0.336183123739437	-0.604998487491236
0.34688310098142	-0.600496695623283
0.35395887912292	-0.660044381781074
2.31599543662934	0.535229827734849
4.92979935905979	1.07084970436245
15.1956991672433	1.58688741387242

Table S4. Differentially abundant proteins in the comparison Control_1h vs Radia

Uniprot Accession	Gene name	Protein name	LFQ intensity C_1h_2
O32504	pprA	DNA repair prote	26.4314785003662
O32508	cinA	CinA-like protein	25.4294357299805
P42443	recA	Protein RecA	30.726634979248
P56864	sat	Sulfate adenylyltr	28.0254344940186
Q9RSK0	rpmJ	50S ribosomal pro	28.8689136505127
Q9RSL1	rpsE	30S ribosomal pro	32.2472839355469
Q9RSW7	rplT	50S ribosomal pro	29.2811603546143
Q9RT63	recD2	ATP-dependent R	27.3261165618896
Q9RUV2	sodA	Superoxide dismu	31.7645778656006
Q9RXI7	ddrD	DNA damage resp	27.3379020690918
Q9RY51	ssb	Single-stranded D	30.1724796295166
Q9RT53	gyrA	DNA gyrase subu	28.4151821136475
Q9RTV2	DR_1654	Uncharacterized p	30.6537780761719
Q9RVN8	DR_0989	Cationic outer me	31.0584659576416
Q9RVW5	DR_0906	DNA topoisomera	29.2820224761963
Q9RYY7	DR_A0166	Uncharacterized p	24.3533973693848
Q9RZQ2	DR_B0068	Uncharacterized p	27.8793506622314
Q9RZU7	DR_B0014	Hemin ABC trans	29.5341377258301

ation_1h

Control_1h		
**LFQ intensity C_1h_4**	**LFQ intensity C_1h_5**	**LFQ intensity R_1h_1**
26.4356479644775	26.4183959960938	28.725757598877
25.3413333892822	26.6304817199707	27.8384590148926
30.6523265838623	30.6261882781982	32.6249465942383
28.3847560882568	28.773006439209	27.9956645965576
30.2471714019775	29.2477359771729	28.1591854095459
32.4847183227539	32.6420516967773	30.9778747558594
29.7280292510986	29.5827465057373	28.1582698822021
27.009880065918	28.1797332763672	28.781192779541
33.2495460510254	31.502420425415	30.0256462097168
27.4081115722656	27.4805450439453	30.9269828796387
30.0163764953613	30.3341541290283	32.6546096801758
28.7893009185791	28.6850185394287	30.0952186584473
27.3996639251709	31.0373458862305	31.7674617767334
31.8553791046143	31.2240047454834	29.7523860931396
29.5749740600586	29.5906715393066	31.1462516784668
25.8549308776855	23.6857070922852	27.6813716888428
28.354305267334	28.1332015991211	25.9707298278809
29.5640182495117	29.937894821167	27.7642059326172

Radiation_1h			
**LFQ intensity R_1h_2**	**LFQ intensity R_1h_5**	**Peptides**	**Razor + unique peptides**
28.172025680542	28.152961730957	10	10
27.5862770080566	27.88063621521	16	16
32.3178367614746	32.3239669799805	19	19
25.9437351226807	27.171501159668	9	9
27.6849193572998	28.1341323852539	1	1
30.4884586334229	31.7735652923584	9	9
28.175931930542	28.2197895050049	7	7
28.9658718109131	28.9863891601563	11	11
28.6222648620605	31.8192462921143	6	6
30.4165935516357	30.9144191741943	13	13
32.7265930175781	32.5197906494141	10	10
29.9262046813965	29.8822555541992	15	15
31.9208927154541	32.427074432373	1	1
30.1018695831299	30.6530952453613	10	10
31.3825740814209	30.8226680755615	22	22
27.6549224853516	27.8915710449219	19	19
25.8857421875	25.3974456787109	22	22
27.9642200469971	27.4707279205322	11	11

Unique peptides	Sequence coverage [%]
10	37.3
16	45.3
19	62.3
9	28.4
1	24.3
9	54.1
7	49.2
11	25.2
6	28.4
13	54.5
10	43.9
15	17.6
1	14.8
10	61.4
22	33.9
19	16.7
22	42.6
11	37.7

Unique + razor sequence coverage [%]	Unique sequence coverage [%]
37.3	37.3
45.3	45.3
62.3	62.3
28.4	28.4
24.3	24.3
54.1	54.1
49.2	49.2
25.2	25.2
28.4	28.4
54.5	54.5
43.9	43.9
17.6	17.6
14.8	14.8
61.4	61.4
33.9	33.9
16.7	16.7
42.6	42.6
37.7	37.7

Mol. weight [kDa]	Q-value	Score	MS/MS count	Student's T-test p-value C_R
32.21		323.31	194	0.000515179509302508
42.662		323.31	174	0.00987004874198435
38.144		323.31	440	7.73724264902888E-05
42.943		323.31	231	0.0989793672713828
4.3092		323.31	58	0.0291106512979906
20.977		323.31	498	0.0244829107735922
13.957		323.31	166	0.000534545270154583
76.43		180.89	97	0.0167393265178668
23.478		323.31	165	0.133459670884745
21.156		323.31	317	4.22780795687908E-05
32.722		323.31	604	2.36565196970989E-05
89.824		323.31	233	0.000487601418361546
18.746		225.23	80	0.116176554015435
18.613		323.31	242	0.0276151143453075
73.157		323.31	380	0.00101971756339637
179.05		100.18	156	0.00853682995391005
94.671		323.31	332	0.000460635276131317
31.721		269.85	188	0.000549264441501866

Student's T-test q-value C_R	Student's T-test Difference C_R
	-1.92174084981282
	-1.96804046630859
	-1.75386683146159
0.0454117647058824	1.35743204752604
0.0145714285714286	1.46186129252116
0.01275	1.3780517578125
0.017	1.34598159790039
0.0136	-1.40590794881185
0.0185454545454545	2.01646232604981
	-3.34381230672201
	-2.45932769775391
0.0156923076923077	-1.33805910746256
0.0096	-2.34154701232911
0.0497777777777778	1.21016629536946
0.0106666666666667	-1.63460858662923
	-3.11127662658691
	2.37097994486491
	1.94563229878743

Fold change C_R	Student's T-test Test statistic C_R
0.263935835740306	-0.878346619983728
0.255599963201035	-0.811280219907323
0.296505992692968	-0.832900152111012
2.56228694231242	0.515345122529401
2.75463523646163	0.599363802156048
2.59917137054321	0.576191089706767
2.54203095387579	0.631066346360911
0.377380570137387	-0.596881357217103
4.04590466628207	0.656134092303004
0.098494548971528	-1.53876739016217
0.181831279102131	-1.16558404010161
0.395552444409446	-0.628494123881157
0.197298649724672	-0.73841644520988
2.31364303951845	0.513362320286687
0.322057772077514	-0.746119242581289
0.115721061992011	-1.1759343406096
5.1729238174067	1.06549255415981
3.85206567090026	0.887033527941592

Table S5. Differentially abundant proteins in the comparison Control_3h vs Radia

Uniprot Accession	Gene name	Protein name	LFQ intensity C_3h_2
O32504	pprA	DNA repair prote	25.8234729766846
O32508	cinA	CinA-like protein	26.4637756347656
P42443	recA	Protein RecA	30.6365966796875
Q46577	uvrA	UvrABC system p	27.9992713928223
Q9RR76	gpsA	Glycerol-3-phosp	25.7786636352539
Q9RRC4	argS	Arginine--tRNA l	29.7102375030518
Q9RRH3	rqkA	DNA damage-res	25.9579925537109
Q9RSJ8	rpsK	30S ribosomal pro	31.0952816009521
Q9RSK0	rpmJ	50S ribosomal pro	30.3940715789795
Q9RSL3	rplF	50S ribosomal pro	32.1942520141602
Q9RSS8	rplA	50S ribosomal pro	30.9190464019775
Q9RSS9	rplJ	50S ribosomal pro	25.7009162902832
Q9RSW7	rplT	50S ribosomal pro	30.859790802002
Q9RSY2	DR_1987	Uncharacterized	31.0794734954834
Q9RT38	glpK	Glycerol kinase	31.2733097076416
Q9RT57	lutC	Lactate utilization	28.6294040679932
Q9RT63	recD2	ATP-dependent R	26.9009265899658
Q9RTF3	gcvH	Glycine cleavage	26.8091907501221
Q9RTG5	infB	Translation initiat	29.9737758636475
Q9RTI0	leuD2	3-isopropylmalate	29.1294002532959
Q9RU72	prmA	Ribosomal protein	26.7820682525635
Q9RU79	rpsB	30S ribosomal pro	32.6265144348145
Q9RU88	nuoC	NADH-quinone o	27.7622413635254
Q9RUN5	hisS	Histidine--tRNA l	28.0069942474365
Q9RUP6	tal	Probable transald	28.8391208648682
Q9RUW4	proS	Proline--tRNA lig	27.8747863769531
Q9RUW8	rsr	60 kDa SS-A/Ro r	25.4110908508301
Q9RV66	panC	Pantothenate synt	28.2296314239502
Q9RV96	hemE	Uroporphyrinogen	29.3734340667725
Q9RVE7	hpf	Ribosome hiberna	28.4452953338623
Q9RVY3	DR_0888	Uncharacterized	32.3236999511719
Q9RW01	purH	Bifunctional purin	26.9624557495117
Q9RWM0	era	GTPase Era	27.4060039520264
Q9RXH2	rpsO	30S ribosomal pro	28.0784168243408
Q9RXI7	ddrD	DNA damage resp	28.4966316223145
Q9RXJ9	rplB	50S ribosomal pro	32.6223945617676
Q9RXK1	rplD	50S ribosomal pro	30.8208446502686
Q9RXK2	rplC	50S ribosomal pro	32.8431854248047
Q9RY51	ssb	Single-stranded D	30.0090522766113
Q9RY64	rplU	50S ribosomal pro	28.88623046875
Q9X719	ruvB	Holliday junctio	29.5644550323486
Q9RRB4	rsfS	Ribosomal silenci	27.9372501373291
Q9RRE0	mltG	Endolytic murein	27.6746520996094
Q9RRE7	DR_2545	NMO domain-con	28.8839321136475
Q9RRH4	DR_2517	Uncharacterized p	25.7358551025391

Q9RRH7	DR_2514	PlsC domain-cont	28.2306385040283
Q9RRS3	DR_2412	GHL10 domain-c	26.3788509368896
Q9RRS5	dnaX	DNA polymerase	26.4052734375
Q9RRU1	DR_2394	N-acetylmuramoy	28.5512752532959
Q9RRW1	DR_2374	Ribonucleoside-d	29.2408409118652
Q9RRW9	DR_2363	Usp domain-conta	30.6091117858887
Q9RRZ5	DR_2334	Uncharacterized p	27.1819610595703
Q9RS52	uvrB	UvrABC system p	27.0542411804199
Q9RS93	DR_2234	DAGKc domain-c	27.8124370574951
Q9RS98	DR_2229	Uncharacterized p	29.1643257141113
Q9RSC6	DR_2199	ATP-dependent D	26.3339672088623
Q9RSE1	DR_2183	Uncharacterized p	27.3834381103516
Q9RSE5	DR_2179	Uncharacterized p	28.8539142608643
Q9RSU8	DR_2024	Uncharacterized p	26.0096454620361
Q9RSY6	DR_1983	Ribosomal protein	32.6951560974121
Q9RSY9	DR_1980	Epimerase domain	27.1875419616699
Q9RT35	DR_1931	Hydrolase_4 dom	27.4331665039063
Q9RT53	gyrA	DNA gyrase subu	29.4668827056885
Q9RT58	DR_1907	Fumarate reductas	28.2819328308105
Q9RTI9	DR_1775	DNA helicase	27.6720905303955
Q9RTX4	DR_1627	Oligoendopeptida	27.457950592041
Q9RTZ8	DR_1599	Serine protease	28.173885345459
Q9RU69	DR_1525	Carbohydrate kina	27.6917896270752
Q9RUA2	DR_1490	Uncharacterized p	27.4252643585205
Q9RUC2	DR_1467	Oxidoreductase, s	26.7820682525635
Q9RUD9	DR_1448	Uncharacterized p	28.9984111785889
Q9RUF4	DR_1432	Uncharacterized p	27.5706558227539
Q9RUQ1	DR_1331	Uncharacterized p	29.1918067932129
Q9RUU2	DR_1289	DNA helicase	26.4110584259033
Q9RV15	accD	Acetyl-coenzyme	28.7342796325684
Q9RV32	bipA	50S ribosomal sul	29.2481441497803
Q9RV41	DR_1189	Methylmalonyl-C	26.7943916320801
Q9RV55	DR_1175	Sensor histidine k	27.7667350769043
Q9RVB5	DR_1114	Heat shock protei	30.1832885742188
Q9RVF8	DR_1071	YCII domain-con	29.2634925842285
Q9RVK6	DR_1021	DUF4384 domain	27.9059257507324
Q9RVN8	DR_0989	Cationic outer me	31.1985301971436
Q9RVN9	DR_0988	Uncharacterized p	25.8719234466553
Q9RVQ8	DR_0964	Carboxypeptidase	26.6492443084717
Q9RVT8	DR_0934	Transcriptional re	27.5926342010498
Q9RVU0	DR_0932	Polyprenyl syntha	28.1888618469238
Q9RVU8	DR_0924	Uncharacterized p	29.656644821167
Q9RVW5	DR_0906	DNA topoisomera	30.0698413848877
Q9RVX1	DR_0900	DUF3197 domain	27.2269248962402
Q9RW33	DR_0836	NurA domain-con	27.7850036621094
Q9RW38	DR_0831	Uncharacterized p	29.2315082550049
Q9RW72	DR_0797	N-acetyltransfera	28.0570335388184
Q9RWM2	DR_0644	Uncharacterized p	28.7212657928467

Q9RWQ3	DR_0613	DUF1989 domain	28.8288688659668
Q9RWV9	DR_0556	CoA_binding don	26.2219200134277
Q9RWW4	DR_0551	Glutaryl-CoA deh	28.6214618682861
Q9RWX7	DR_0538	Uncharacterized p	28.129768371582
Q9RWX8	DR_0537	Uncharacterized p	27.1300621032715
Q9RX16	DR_0499	3-hydroxyisobuty	28.6458148956299
Q9RX65	DR_0450	Uncharacterized p	26.7785625457764
Q9RX85	DR_0430	3'-phosphate/5'-hy	25.13938331604
Q9RX86	DR_0429	Cytochrome-relate	28.2420444488525
Q9RXC5	DR_0390	DhaL domain-con	28.8331604003906
Q9RXQ2	DR_0257	Pyruvate dehydro	31.2334651947021
Q9RY46	DR_0105	Uncharacterized p	28.7020664215088
Q9RY79	DR_0071	Peptidyl-prolyl ci	30.3377742767334
Q9RYG3	DR_A0354	Methyl-accepting	27.4298191070557
Q9RYL1	DR_A0301	Methylamine utili	27.5181827545166
Q9RYN0	DR_A0281	Uncharacterized p	29.2989826202393
Q9RYT5	DR_A0223	4-hydroxyphenyla	27.3522033691406
Q9RYT6	DR_A0222	TDP-glucose-4,6-	27.3321914672852
Q9RYY7	DR_A0166	Uncharacterized p	24.2211818695068
Q9RZ87	DR_A0067	CoA transferase,	29.0397796630859
Q9RZB2	DR_A0042	Glucose-1-phosph	26.9364070892334
Q9RZB3	DR_A0041	dTDP-glucose 4,6	27.5034732818604
Q9RZE4	DR_A0005	Alcohol dehydrog	29.0294418334961
Q9RZK2	DR_B0125	Iron ABC transpo	30.6249675750732
Q9RZK3	DR_B0124	Iron-chelator utili	27.9304981231689
Q9RZQ1	DR_B0069	Serine protease, s	26.7092514038086
Q9RZQ2	DR_B0068	Uncharacterized p	27.6444396972656
Q9RZQ3	DR_B0067	Extracellular nucl	28.9165859222412
Q9RZU7	DR_B0014	Hemin ABC trans	31.0446586608887

ation_3h

Control_3h		
**LFQ intensity C_3h_3**	**LFQ intensity C_3h_4**	**LFQ intensity R_3h_2**
25.9150867462158	26.2163238525391	29.6917400360107
25.6432571411133	26.1033763885498	28.818546295166
30.5783977508545	30.6838665008545	33.8182182312012
28.5242118835449	28.4441509246826	29.3810882568359
26.2490539550781	25.6571197509766	26.7239036560059
29.9240818023682	30.0055770874023	29.1926288604736
25.7869110107422	25.4741668701172	26.5207481384277
30.6338272094727	30.6048774719238	30.0019588470459
30.7314167022705	30.5445289611816	28.871000289917
32.1808319091797	32.0854072570801	31.1111660003662
31.5147914886475	31.6265354156494	30.1122245788574
25.4783401489258	25.582935333252	24.2941780090332
31.6227893829346	31.0705432891846	29.6766891479492
31.2440547943115	31.5336170196533	32.5771598815918
31.498384475708	31.5267791748047	30.4045639038086
28.762622833252	28.6811027526855	26.9897766113281
27.300163269043	27.8512668609619	29.1731700897217
26.8181190490723	27.0083847045898	27.779878616333
30.1376056671143	30.1225051879883	29.2237987518311
29.4613513946533	29.1222324371338	28.4673099517822
27.5566921234131	27.6513004302979	26.1348876953125
32.5466918945313	32.5243759155273	31.6361198425293
26.4328651428223	27.5762901306152	28.1365795135498
27.3502635955811	27.5478973388672	28.0463008880615
28.3454933166504	28.2812690734863	29.1151790618896
28.3133277893066	28.2520980834961	27.0852718353271
25.8070297241211	25.3815593719482	27.7968063354492
27.8359317779541	27.816349029541	29.4001121520996
29.3718147277832	29.5891551971436	30.9398574829102
28.2628879547119	28.5418930053711	29.3458309173584
32.5069885253906	32.3831405639648	31.4203624725342
27.4800815582275	27.8011932373047	26.5859184265137
28.4789257049561	27.7045764923096	26.6538982391357
28.3003807067871	28.1385345458984	26.7995891571045
27.7472076416016	28.0016899108887	32.941047668457
32.5403327941895	32.7029151916504	31.6968955993652
31.4459381103516	31.5030364990234	30.4854850769043
32.977668762207	32.7796440124512	31.7094402313232
29.8924388885498	30.2893409729004	33.6930160522461
29.4031429290771	29.1565589904785	28.1339378356934
29.5261173248291	29.6046829223633	30.8971366882324
28.0625	27.9216156005859	27.2135448455811
27.5966205596924	27.4997520446777	26.9452533721924
28.8116111755371	28.8696193695068	27.4090843200684
26.3021678924561	25.8275489807129	27.2191886901855

27.7278671264648	27.865966796875	26.6168079376221
26.6622085571289	26.3787670135498	25.2021713256836
25.9270076751709	26.2537498474121	27.3727703094482
29.0606708526611	28.7917861938477	27.8876247406006
29.0803241729736	29.1466655731201	29.8571529388428
30.2964687347412	30.3329811096191	31.7455501556396
26.8450565338135	26.7481689453125	27.6757297515869
27.0980834960938	27.0133972167969	27.7884922027588
27.6113300323486	27.5330429077148	28.5287265777588
29.0511054992676	28.95631980896	30.2791900634766
26.6264495849609	26.9186611175537	25.8353290557861
26.6082305908203	26.698429107666	27.6194686889648
29.9932098388672	29.6585025787354	28.2896919250488
25.8889961242676	26.9478206634521	26.8016872406006
32.624095916748	32.6287956237793	31.7648944854736
27.2750644683838	27.3755893707275	28.2610950469971
27.3813781738281	27.6877250671387	26.0529365539551
29.0905055999756	29.2436351776123	30.9599590301514
28.9330902099609	28.6415481567383	26.8713836669922
27.6143531799316	27.1736946105957	28.2288055419922
27.7340850830078	27.7982292175293	26.6811027526855
27.9997024536133	28.2196502685547	29.0192966461182
28.2485294342041	28.1359920501709	27.2784366607666
27.7154178619385	28.0802993774414	26.8717346191406
27.0622940063477	27.0003471374512	26.3379516601563
30.0332813262939	29.4749298095703	28.7225360870361
27.8637237548828	28.5817947387695	28.805627822876
28.9063282012939	28.6598300933838	30.1305046081543
26.1547012329102	26.1261882781982	27.8757247924805
28.5946636199951	28.6130180358887	27.6045589447021
29.3807373046875	29.6883087158203	28.350980758667
26.4263210296631	26.5386619567871	24.8819427490234
27.8607082366943	28.8203430175781	26.720645904541
30.0103874206543	29.6070499420166	30.6287975311279
28.7920341491699	28.7513637542725	30.1997604370117
28.332576751709	28.4011688232422	27.2250862121582
31.6659393310547	31.6163177490234	30.6197319030762
25.6178169250488	25.4443645477295	26.7605304718018
26.2180061340332	26.2859859466553	26.9723510742188
27.3287734985352	26.9947471618652	28.4336452484131
27.8608264923096	28.0876522064209	29.0264911651611
29.8576126098633	29.9758262634277	28.819185256958
30.3080196380615	30.0837268829346	31.9129581451416
27.450496673584	27.6234512329102	26.4803600311279
27.6285362243652	27.5296955108643	28.5387134552002
29.1187229156494	29.7146644592285	30.203498840332
28.6387557983398	28.4626770019531	27.4621696472168
27.7586898803711	28.5575695037842	29.0178642272949

28.6004180908203	28.7586250305176	29.880256652832
26.1309261322021	26.0658512115479	26.9233169555664
28.4039344787598	28.9563751220703	29.8403205871582
28.4136905670166	28.3067817687988	26.4016819000244
27.0342273712158	26.822380065918	24.8868350982666
28.6144924163818	29.0866146087646	28.1129188537598
27.9702129364014	26.6285362243652	26.2884693145752
25.1172771453857	24.1780529022217	26.4976692199707
27.4778461456299	27.6329097747803	28.7643947601318
28.7798480987549	28.6448173522949	28.0130786895752
31.1587028503418	31.0925750732422	30.3186168670654
27.6903247833252	28.8455352783203	29.6864719390869
30.3940715789795	30.4321117401123	29.5128059387207
27.5752086639404	27.4070587158203	25.0129928588867
27.7215595245361	26.9316806793213	25.986026763916
30.0451812744141	29.7218189239502	28.7916316986084
27.2149353027344	27.2651710510254	28.1037464141846
26.7692604064941	27.0171203613281	28.0477046966553
25.6563549041748	25.9812431335449	28.860502243042
28.7710552215576	28.9588108062744	29.7507266998291
27.3371353149414	27.0952682495117	26.2304840087891
27.8930759429932	28.1490421295166	25.9317035675049
28.6564922332764	28.6597270965576	29.678014755249
31.4784622192383	30.4569911956787	29.1310195922852
28.1041965484619	27.897180557251	26.7714023590088
26.9408931732178	26.9924812316895	25.7555980682373
27.5834102630615	27.6457462310791	25.9848537445068
29.8866367340088	29.7443065643311	26.3543567657471
31.1545448303223	31.2384281158447	30.0126533508301

Radiation_3h			
**LFQ intensity R_3h_3**	**LFQ intensity R_3h_5**	**Peptides**	**Razor + unique peptides**
29.6234169006348	29.4223194122314	10	10
29.0286254882813	29.054500579834	16	16
33.456356048584	33.5688743591309	19	19
29.6753597259521	29.5642738342285	15	15
26.741626739502	26.9498252868652	3	3
28.7597694396973	28.9213600158691	15	15
26.4203414916992	26.6308975219727	6	6
29.950325012207	29.9774646759033	5	5
29.4567165374756	29.1716690063477	1	1
31.5018520355225	31.2399654388428	14	14
30.3018207550049	29.9288883209229	9	9
25.2164535522461	24.7605056762695	6	6
30.330846786499	29.886739730835	7	7
32.0159454345703	31.8343200683594	5	5
30.476146697998	30.4382724761963	14	14
26.9250164031982	26.7927799224854	5	5
29.3143005371094	29.0777816772461	11	11
27.6044883728027	27.8618927001953	4	4
29.4799480438232	29.035327911377	13	13
28.4844303131104	28.2419090270996	5	5
26.8361740112305	26.2563610076904	6	6
31.6347808837891	31.65185546875	12	12
28.113317489624	28.6571731567383	4	4
28.439640045166	29.1794490814209	4	4
29.4292411804199	29.4021663665771	9	9
27.5176582336426	27.0902290344238	9	9
27.9267711639404	28.0441131591797	11	11
28.6754264831543	28.6284313201904	6	6
30.059175491333	30.4699516296387	6	6
29.1510524749756	29.1082897186279	7	7
31.9269828796387	31.3952980041504	13	13
26.6506156921387	26.2979774475098	8	8
26.9049510955811	27.2652606964111	7	7
26.9577045440674	26.0053024291992	3	3
32.8483428955078	32.9541091918945	13	13
31.8486042022705	31.9954357147217	16	16
30.208158493042	30.2916469573975	14	14
31.9870548248291	31.8505802154541	10	10
32.9565010070801	33.2115707397461	10	10
28.5057830810547	27.8706188201904	4	4
30.9719276428223	30.6301002502441	5	5
27.3842620849609	26.7701416015625	4	4
27.1822452545166	25.505578994751	6	6
27.9285793304443	27.6769409179688	14	14
27.5376415252686	27.9607448577881	6	6

27.0420780181885	26.7635726928711	4	4
25.6225166320801	25.3970527648926	5	5
26.9161586761475	27.2353496551514	8	8
27.9256954193115	27.5099029541016	12	12
29.9820976257324	30.0257778167725	23	23
31.0695209503174	31.5090446472168	4	4
27.7309799194336	27.9388198852539	4	4
28.196949005127	27.9500465393066	11	11
28.4092864990234	28.3618545532227	8	8
29.6387386322021	30.1923713684082	6	6
25.7942180633545	25.4730205535889	6	6
27.7197322845459	27.9166126251221	3	3
28.1452560424805	28.3527069091797	3	3
27.9373054504395	28.1440887451172	2	2
32.0272941589355	31.7306079864502	16	16
28.2386322021484	28.6288833618164	6	6
26.2957038879395	26.4298992156982	5	5
30.8374824523926	30.89475440979	15	15
26.6635665893555	26.704906463623	5	5
28.2974967956543	28.4719295501709	6	6
27.0228443145752	26.5552158355713	5	5
28.7749557495117	28.9108791351318	14	14
27.1898040771484	27.0485363006592	5	5
27.0198802947998	26.4881057739258	5	5
26.2384872436523	25.7489624023438	5	5
28.5780544281006	28.2582683563232	10	10
28.8939437866211	29.5265827178955	6	6
30.0139827728271	30.2354640960693	1	1
27.7155494689941	27.8634872436523	9	9
27.9785289764404	27.5959796905518	9	9
28.9397449493408	28.4077072143555	7	7
24.6117248535156	24.1313190460205	4	4
27.5050640106201	27.0097732543945	6	6
31.2449054718018	31.8683166503906	5	5
29.9508819580078	30.4960041046143	2	2
28.0050144195557	26.7516193389893	4	4
30.8669548034668	30.2285995483398	10	10
26.6913909912109	26.4357604980469	3	3
27.6645832061768	28.0409297943115	3	3
27.8604125976563	28.1677742004395	2	2
28.8802852630615	28.9771919250488	7	7
28.7490310668945	28.1376056671143	6	6
31.9463214874268	31.4717445373535	22	22
26.5785961151123	26.5551433563232	6	6
28.701042175293	28.6150894165039	4	4
30.4887447357178	30.4518928527832	6	6
27.8252944946289	27.1330070495605	6	6
29.1723117828369	29.6162815093994	3	3

29.3196926116943	30.1148319244385	4	4
27.4306964874268	26.5870628356934	5	5
29.1599540710449	29.5879402160645	4	4
26.9234313964844	26.6378231048584	10	10
25.4018459320068	25.6142826080322	6	6
27.7237739562988	27.3342399597168	9	9
26.8613586425781	25.2370281219482	4	4
25.7429618835449	25.5119247436523	8	8
28.6705055236816	29.1126461029053	1	1
28.1803970336914	27.7077388763428	10	10
30.2229690551758	30.2397937774658	22	22
29.4178009033203	29.5389919281006	6	6
29.6773433685303	29.4223384857178	23	23
25.582218170166	24.783317565918	12	12
26.1275844573975	26.0685386657715	5	5
29.072603225708	28.7098751068115	2	2
27.9370803833008	28.0825347900391	12	12
27.8240203857422	28.0257511138916	4	4
29.444408416748	29.4579105377197	19	19
30.7911033630371	30.2844944000244	6	6
26.393440246582	26.2766456604004	5	5
26.6692562103271	26.9138774871826	5	5
29.9961757659912	30.0598201751709	14	14
29.0689945220947	29.4123783111572	8	8
26.3804531097412	26.908618927002	7	7
25.5529460906982	25.8281803131104	12	12
26.0506973266602	26.0392570495605	22	22
27.3550624847412	27.5200576782227	24	24
29.6946811676025	30.5789375305176	11	11

Unique peptides	Sequence coverage [%]
10	37.3
16	45.3
19	62.3
15	20.5
3	19.8
15	31.2
6	14.4
5	32.1
1	24.3
14	73
9	52.4
6	33.7
7	49.2
5	24.8
14	42.3
5	39.2
11	25.2
4	59.2
13	29.6
5	33.7
6	34.4
12	61
4	17
4	11.9
9	39.3
9	21.8
11	26.2
6	21.7
6	30.5
7	50.3
13	60.9
8	29.2
7	38.9
3	47.3
13	54.5
16	58.2
14	66.3
10	39.8
10	43.9
4	50
5	16.5
4	42.2
6	21.9
14	40.3
6	34.9

4	22.4
5	19
8	20.8
12	34.6
23	23.6
4	53.1
4	24.6
11	18.4
8	32.8
6	54.3
6	19.5
3	18.6
3	32.5
2	21.7
16	31.6
6	16.5
5	22.2
15	17.6
5	31.6
6	12.9
5	13.9
14	41.1
5	21.9
5	45.4
5	27.5
10	34.7
6	38.2
1	13.6
9	17.6
9	32
7	18
4	7.8
6	18.5
5	29.7
2	21
4	30.5
10	61.4
3	20
3	8.4
2	14.4
7	32.8
6	37.3
22	33.9
6	63.8
4	18.1
6	59.7
6	39.9
3	20.9

4	16.7
5	51.4
4	16.9
10	16.3
6	27.7
9	36.1
4	17.4
8	30.9
1	12
10	27.4
22	28.7
6	43.6
23	44.8
7	25.6
5	14.8
2	27.4
12	33.5
4	24.3
19	16.7
6	39.2
5	20.3
5	22.3
14	37
8	27.5
7	21.8
12	25.4
22	42.6
24	32.5
11	37.7

Unique + razor sequence coverage [%]	Unique sequence coverage [%]
37.3	37.3
45.3	45.3
62.3	62.3
20.5	20.5
19.8	19.8
31.2	31.2
14.4	14.4
32.1	32.1
24.3	24.3
73	73
52.4	52.4
33.7	33.7
49.2	49.2
24.8	24.8
42.3	42.3
39.2	39.2
25.2	25.2
59.2	59.2
29.6	29.6
33.7	33.7
34.4	34.4
61	61
17	17
11.9	11.9
39.3	39.3
21.8	21.8
26.2	26.2
21.7	21.7
30.5	30.5
50.3	50.3
60.9	60.9
29.2	29.2
38.9	38.9
47.3	47.3
54.5	54.5
58.2	58.2
66.3	66.3
39.8	39.8
43.9	43.9
50	50
16.5	16.5
42.2	42.2
21.9	21.9
40.3	40.3
34.9	34.9

22.4	22.4
19	19
20.8	20.8
34.6	34.6
23.6	23.6
53.1	53.1
24.6	24.6
18.4	18.4
32.8	32.8
54.3	54.3
19.5	19.5
18.6	18.6
32.5	32.5
21.7	21.7
31.6	31.6
16.5	16.5
22.2	22.2
17.6	17.6
31.6	31.6
12.9	12.9
13.9	13.9
41.1	41.1
21.9	21.9
45.4	45.4
27.5	27.5
34.7	34.7
38.2	38.2
13.6	13.6
17.6	17.6
32	32
18	18
7.8	7.8
18.5	18.5
29.7	29.7
21	21
30.5	30.5
61.4	61.4
20	20
8.4	8.4
14.4	14.4
32.8	32.8
37.3	37.3
33.9	33.9
63.8	63.8
18.1	18.1
59.7	59.7
39.9	39.9
20.9	20.9

16.7	16.7
51.4	51.4
16.9	16.9
16.3	16.3
27.7	27.7
36.1	36.1
17.4	17.4
30.9	30.9
12	12
27.4	27.4
28.7	28.7
43.6	43.6
44.8	44.8
15.6	25.6
14.8	14.8
27.4	27.4
33.5	33.5
24.3	24.3
16.7	16.7
39.2	39.2
20.3	20.3
22.3	22.3
37	37
27.5	27.5
21.8	21.8
25.4	25.4
42.6	42.6
32.5	32.5
37.7	37.7

Mol. weight [kDa]	Q-value	Score	MS/MS count	Student's T-test p-value C_R
32.21		323.31	194	0.0000151259854875
42.662		323.31	174	0.000311519140397759
38.144		323.31	440	1.14969034718314E-05
112.1		100.93	259	0.00272787050753832
33.597		231.24	88	0.00945528502037795
66.603		323.31	267	0.00390897781665615
71.125	0.0006959	14.616	73	0.00704138804648386
13.791		170.52	85	0.00737184192196051
4.3092		323.31	58	0.00205584818348044
19.581		323.31	271	0.00192477408539638
24.36		323.31	394	0.00714625944253694
17.754		152.87	50	0.0387185445511247
13.957		323.31	166	0.0149794124564188
20.551		236.99	498	0.0300729091948452
55.067		323.31	414	0.000276909997991713
22.621		323.31	90	1.37337821088312E-05
76.43		180.89	97	0.00293688081466565
12.774		69.315	39	0.000957257068278874
64.023		323.31	270	0.0039284683760383
22.652		23.488	83	0.00353694506694516
29.548		323.31	112	0.0582036717370132
29.755		323.31	538	7.99337077263005E-06
25.032	0.0007082	16.251	38	0.0817871488836982
46.517		35.193	27	0.0751512444317363
24.759		119.86	116	0.0151447909182967
56.39		293.95	202	0.0099056225606758
57.422		104.49	118	0.000102419076864983
30.118		74.432	93	0.0295045214015024
38.032		134.82	145	0.0168054998507124
22.02		250.34	168	0.00201839396897485
26.57		323.31	450	0.0104938029735215
54.046		323.31	144	0.027820372455985
34.519		323.31	117	0.0652859100165011
10.151		323.31	113	0.00630806968547636
21.156		323.31	317	2.65957334393869E-05
30.044		323.31	518	0.00139126529391667
22.278		323.31	359	0.0164533487813691
22.437		323.31	519	0.000507773693543019
32.722		323.31	604	0.000195757878636275
11.147		29.844	64	0.0145337812328133
36.324		45.973	106	0.000281940771946676
12.915		28.121	96	0.0106703258455862
37.421		323.31	180	0.117803460708731
51.204		323.31	277	0.00145308168225624
35.523		323.31	190	0.00430961480589468

22.292	0.0007582	22.438	80	0.00435637645300226
61.246	0.0007179	17.313	38	0.00227819819831142
64.155		58.066	102	0.00743403261632141
62.023		203.4	241	0.00659492937429436
152.91		323.31	415	0.000312530011463739
16.533		89.613	117	0.00967737321678217
42.811		59.693	74	0.00510397851957586
81.944		91.996	81	0.00159710379279158
33.498		323.31	153	0.00128651032714942
19.03		323.31	234	0.00946419276442132
64.473		99.88	64	0.0105189200269165
22.963		63.219	93	0.0301805307681498
16.181		284.07	83	0.0226219362729911
13.222	0.0006169	7.349	41	0.0656736039424338
69.309		323.31	508	0.00110940016321892
34.991	0.0006901	13.989	105	0.0013452379288135
27.834		27.818	39	0.00103252424308954
89.824		323.31	233	0.00014304031734137
26.742		64.228	35	0.000707759462630622
82.596		170.66	65	0.00813823698640099
66.895		323.31	150	0.00642725586760038
50.683		323.31	383	0.00138044847540439
32.183		323.31	89	0.00951342634536971
22.214		323.31	101	0.0185526106731201
27.399		38.241	59	0.013916313884884
45.866		323.31	207	0.0404781947992032
9.2178		186.76	77	0.0467801435907306
8.3471		323.31	59	0.00191955291181912
89.89		63.683	128	0.000108144927693403
31.042		137.75	104	0.00231527282897213
66.059		255.51	115	0.018743131350887
62.555	0.0007299	18.997	54	0.00112712993351853
41.959		275.94	59	0.0581959720146229
20.238		127.22	127	0.0295511988537899
11.874		30.448	78	0.00492287043106415
17.947		211.16	76	0.0893835223293399
18.613		323.31	242	0.0178711589291299
17.432	0.0005794	4.3233	29	0.00343037092325538
55.783		122.54	40	0.0260105420831611
29.339		23.022	72	0.023945366045367
34.632		101.46	132	0.000990468950380577
23.87		323.31	249	0.00587400230703885
73.157		323.31	380	0.000692228319591693
15.516		105.69	107	0.0016445748024429
38.263		74.742	54	0.000382165182061595
16.175		89.982	171	0.00726797506000484
17.078		107.52	88	0.025850956415696
20.874		136.08	130	0.0565152650262315

23.563	0.0006545	10.461	39	0.0131796859465408
15.808		24.762	64	0.0279908136782167
45.705		98.145	42	0.0272441856761651
119.56		323.31	164	0.00069427171902882
44.135		323.31	99	0.00193955315905206
31.194		66.52	104	0.017570799104302
30.394		102.9	41	0.19293286301869
50.488		158.37	48	0.0637009256335553
25.124		323.31	52	0.0167407116383016
58.63		323.31	199	0.00624695976704986
104.21		323.31	697	5.68490716860391E-05
17.83		233.81	129	0.0378957449030781
73.453		323.31	483	0.000432838167613403
80.859		323.31	68	0.000647894702910687
36.174		323.31	62	0.00521352399974252
11.442		323.31	106	0.0266060476880044
56.109		180.54	129	0.000318021316718751
31.653		43.682	80	0.00646800135019638
179.05		100.18	156	0.002319522479683
22.134		72.103	118	0.0121402934767116
32.962		74.282	94	0.00285768682122259
40.884		183.76	111	0.0184446940867921
41.986		221.25	174	0.00272789645233561
35.079		227.83	83	0.00777894358843281
34.303		137.79	101	0.00164244070204315
78.779		323.31	144	0.000619985586987824
94.671		323.31	332	6.38268174996356E-07
109.78		323.31	454	0.00673107465233946
31.721		269.85	188	0.0165416436650571

Student's T-test q-value C_R	Student's T-test Difference C_R
	-3.59419759114583
	-2.89708773295085
	-2.98152923583984
0.0175	-1.21769587198893
0.03285	-0.910172780354817
0.0326285714285714	0.922046025594078
0.047672131147541	-0.784305572509766
0.0425263157894737	0.801412582397461
0.00939130434782609	1.3902104695638
0.0351058823529412	0.869169235229496
0.0201176470588235	1.23914655049642
0.0438347107438017	0.830351511637371
0.0207179487179487	1.2196159362793
0.0408380952380952	-0.856760025024414
0.0268333333333333	0.993163426717121
	1.78851890563965
	-1.83763186136882
0.0324444444444444	-0.870188395182293
0.0390204081632653	0.83160400390625
0.0406593406593407	0.839778264363606
0.0402173913043478	0.920879364013672
0.0255593220338983	0.924942016601563
0.0339154929577465	-1.04522450764974
0.04	-0.920078277587891
0.0401090909090909	-0.826901117960613
0.0336923076923077	0.915684382120766
	-2.38933690388997
0.0327619047619048	-0.940685907999676
0.024280701754386	-1.04486020406087
0.0429026548672566	-0.785032272338867
0.0393394495412844	0.823728561401367
0.041	0.90307299296061
0.0389473684210526	0.921798706054688
0.00981818181818182	1.58491198221843
	-4.83265686035156
0.0410847457627119	0.774902343750004
0.0332658227848101	0.928176244099934
0.028	1.01780764261882
	-3.22341855367025
0.031030303030303	0.978530883789063
0.009	-1.2679697672526
0.0394226804123711	0.851139068603516
0.0320487804878049	1.04598236083984
0.019	1.18351936340332
	-1.61733436584473

0.0192380952380952	1.13400459289551
0.0234666666666667	1.0660285949707
0.0283225806451613	-0.979415893554688
0.02768	1.02683639526367
0.0404	-0.799065907796223
0.0247142857142857	-1.02851804097493
0.0405393258426966	-0.856781005859375
0.0305671641791045	-0.923255284627277
0.0424642857142857	-0.78101921081543
0.0274375	-0.979516347249351
0.0329473684210526	0.925503412882485
0.0400747663551402	-0.855238596598308
0.0202	1.23932393391927
0.0212631578947368	-1.34553972880046
0.0396078431372549	0.808417002360027
0.0187906976744186	-1.09680493672689
0.0121428571428571	1.24124336242676
	-1.63039080301921
	1.87223815917969
0.0385416666666667	-0.846031188964844
0.0325194805194805	0.910367329915363
0.041781512605042	-0.770631154378254
0.0393617021276596	0.853178024291992
0.0333866666666667	0.947086970011391
0.0412307692307692	0.83976936340332
0.0338378378378378	0.982587814331055
0.0247213114754098	-1.06999333699545
0.016969696969697	-1.20732879638672
	-1.58760452270508
0.0312463768115942	0.920964558919273
0.0397849462365591	0.872919082641602
	2.04479598999023
0.0278730158730159	1.07076772054037
0.0184864864864865	-1.31376457214355
0.0117241379310345	-1.2799186706543
0.0414358974358974	0.885983784993488
0.0331566265060241	0.921833674112957
0.026	-0.984525680541992
0.024	-1.17487589518229
0.0404528301886792	-0.848559061686199
0.0301176470588235	-0.915542602539063
0.0140645161290323	1.2614205678304
	-1.62314542134603
0.0343013698630137	0.895591100056965
0.0261132075471698	-0.970536549886067
0.0266153846153846	-1.02641359965007
0.0357241379310345	0.912665049235024
0.0411111111111111	-0.922977447509766

0.0256296296296296	-1.04228973388672
0.0406126126126126	-0.840792973836265
0.0404271844660194	-0.868814468383789
	1.62910143534343
	1.6945686340332
0.0271372549019608	1.05866305033366
0.0397037037037037	0.996818542480469
0.0251333333333333	-1.10594749450684
0.0282448979591837	-1.06491533915202
0.0442	0.785537083943684
0.0298461538461538	0.901121139526367
0.0274042553191489	-1.13511276245117
0.0346976744186046	0.850489934285484
	2.3445192972819
0.00830769230769231	1.32975769042969
0.0421565217391304	0.83062426249186
0.0417931034482759	-0.763683954874676
0.0334444444444444	-0.926301320393879
	-3.96801376342773
0.008	-1.35222625732422
0.0386262626262626	0.822746912638348
0.0145333333333333	1.34358469645182
0.0197073170731707	-1.12944984436035
	1.64934285481771
0.00864	1.29046694437663
0.0195428571428571	1.16863377888998
	1.59959602355957
	2.43935076395671
0.0251636363636364	1.05045318603516

Fold change C_R	Student's T-test Test statistic C_R
0.0828015973029901	-1.67671289661185
0.134242395176224	-1.28821134620296
0.126610658357321	-1.41225317331502
0.429968873360389	-0.557438568736182
0.53212135937735	-0.414746843299115
1.89480058913051	0.428070327163544
0.580631371309397	-0.364090714177522
1.74280672145737	0.371099700414874
2.62116917271953	0.633286195211619
1.82661075788979	0.409997013948704
2.36058846558029	0.552066450840046
1.77811854689838	0.36516688829497
2.32884712176222	0.530678482282751
0.552191266321428	-0.379094846538951
1.99054491734552	0.476840409859444
3.45460055461961	0.86412607441475
0.279780658971906	-0.804587983383495
0.547075405801305	-0.414391657760751
1.7796629112181	0.388773271273708
1.78977504046898	0.393070871061735
1.89326894355691	0.391833146305014
1.8986079333608	0.455303519555745
0.484569495424129	-0.426283825992644
0.528480345274678	-0.385787184842245
0.563738843300012	-0.375376573875405
1.88646375564161	0.416534137186574
0.190870109292417	-1.10892240338204
0.520985126863892	-0.411907573722462
0.484691872427923	-0.461416495385671
0.580338975165158	-0.372104915410703
1.76997448261842	0.377610917396262
1.87004500778387	0.398305236481014
1.89447579350125	0.389660559042678
2.99989495156605	0.688483409603675
0.0350933902795729	-2.17439891582241
1.71107422456672	0.369328949722378
1.90286899884275	0.415582669181764
2.02483962083339	0.484866577551689
0.107066677730561	-1.43520295400634
1.97045784266025	0.437405311130757
0.415243713695076	-0.602017980155851
1.80392463771801	0.388938074593406
2.06477182580895	0.414035116231085
2.27130171502721	0.550062047819439
0.3259371343241	-0.710179460185124

2.19467086199744	0.516619202344708
2.09366206101561	0.494937779315833
0.507185043220231	-0.44610859728164
2.03755131740745	0.467139816059438
0.574721167968914	-0.386262127687208
0.490213445209254	-0.463027053592829
0.552183235969029	-0.397774566554349
0.527317841481115	-0.43523966193928
0.581955517874869	-0.372464602225168
0.507149729563282	-0.443339795728603
1.89934688290372	0.419924084667475
0.552773899937804	-0.378448692939221
2.36087872443135	0.528809193382219
0.393506745346373	-0.530804275600689
1.75128878577216	0.385608090356956
0.46755081100555	-0.513082297995941
2.36402183757687	0.578568853974278
0.323000700082538	-0.77092056371317
3.66100097834397	0.851506059675074
0.556313033485155	-0.389296341232427
1.87952399005845	0.418675526010703
0.586160983120971	-0.367423045909282
1.80647592032505	0.39088796584859
1.92797583618058	0.42148659983126
1.78976399817178	0.381558428405322
1.97600666670022	0.421890631867
0.476321198879109	-0.450236579054009
0.433069718506252	-0.557275582303414
0.332723455245349	-0.754499193943096
1.89338074932802	0.431668221989931
1.83136465303639	0.3917277314754
4.12614920146914	0.910809042983215
2.10055086401298	0.44482599963166
0.402269824981063	-0.548210267745074
0.411818723521443	-0.574553095725218
1.84802437411756	0.369638148869826
1.89452171238286	0.41195463433644
0.505391855226985	-0.456083667508001
0.442921859337232	-0.502006167570946
0.555339122034374	-0.378906867924066
0.530144444045378	-0.434717062987865
2.39731679560395	0.564230013135408
0.324626927159705	-0.747542744238873
1.86037195946489	0.422740115280948
0.510316236845064	-0.464843529536861
0.490929035253176	-0.465801654579353
1.8825198133809	0.403141355136737
0.527419403192558	-0.393214661052731

0.485556224582897	-0.46419645762977
0.558336596322074	-0.373794990283234
0.547596652190789	-0.385209351068689
3.09320282409819	0.750023264020833
3.23680091767853	0.758428148717459
2.08300030458239	0.466045038137608
1.99559442279565	0.377943783767322
0.464597245385974	-0.454242811040283
0.478000708216042	-0.469286057651423
1.72373390858525	0.365486556622804
1.86751669399482	0.439516994987553
0.45529932876571	-0.478600570634179
1.80311315230007	0.40898523809749
5.07891136643447	1.04515088228819
2.51360453813301	0.593556360286074
1.77845474352124	0.370422688965805
0.588990408313407	-0.369649966435709
0.526205662887512	-0.425344922154906
0.0639011735378326	-1.54069119080938
0.391687160506508	-0.585194202535466
1.76877055370238	0.38695768905943
2.5378111161172	0.57178535478592
0.457089998049356	-0.520224392217091
3.13690721012024	0.706836121606011
2.44607212586298	0.594481298134198
2.24798713453782	0.551276782845982
3.03058440470979	0.788403957349776
5.42397588383904	0.986235335574067
2.07118035425433	0.463855696715235

Table S6. Differentially abundant proteins in the comparison Control_6h vs Radia

Uniprot Accession	Gene name	Protein name	LFQ intensity C_6h_1
O32504	pprA	DNA repair prote	26.4724636077881
O32508	cinA	CinA-like protein	26.3323287963867
P42443	recA	Protein RecA	30.7394065856934
Q46577	uvrA	UvrABC system p	28.4357509613037
Q59337	katA	Catalase	32.4800758361816
Q9RRG8	rpmB	50S ribosomal pro	28.3523712158203
Q9RRY0	hemC	Porphobilinogen	28.2844066619873
Q9RS16	psuG	Pseudouridine-5'-	28.9643039703369
Q9RSL0	rpmD	50S ribosomal pro	25.3042774200439
Q9RSN7	infC	Translation initiat	31.2849349975586
Q9RSY2	DR_1987	Uncharacterized	31.2415561676025
Q9RT38	glpK	Glycerol kinase	31.6629295349121
Q9RU72	prmA	Ribosomal protein	27.5988922119141
Q9RU79	rpsB	30S ribosomal pro	32.7138710021973
Q9RUE8	DR_1438	Probable ABC tra	28.9812259674072
Q9RUF5	purD	Phosphoribosylan	27.6476669311523
Q9RUK6	msrB	Peptide methionin	27.8720874786377
Q9RUW8	rsr	60 kDa SS-A/Ro r	26.3517303466797
Q9RUZ0	DR_1240	Citrate lyase subu	28.7536926269531
Q9RV79	miaB	tRNA-2-methylth	29.0710697174072
Q9RV96	hemE	Uroporphyrinogen	29.7106151580811
Q9RV97	ilvD	Dihydroxy-acid d	27.2846279144287
Q9RVD6	trpS2	Tryptophan--tRNA	26.1388359069824
Q9RVE7	hpf	Ribosome hiberna	27.1487998962402
Q9RVY3	DR_0888	Uncharacterized	33.1346549987793
Q9RW94	aroC	Chorismate syntha	26.9847888946533
Q9RWN9	murC	UDP-N-acetylmu	27.4475040435791
Q9RX20	pdxH	Pyridoxine/pyrido	26.99733543396
Q9RX35	ttcA	tRNA-cytidine(32	28.4123592376709
Q9RXH2	rpsO	30S ribosomal pro	28.3452816009521
Q9RXI7	ddrD	DNA damage resp	28.5068817138672
Q9RXJ1	rplX	50S ribosomal pro	30.2477359771729
Q9RXY1	rplM	50S ribosomal pro	27.659761428833
Q9RY51	ssb	Single-stranded D	30.2041988372803
Q9RYM8	DR_A0283	Probable subtilas	31.6167106628418
Q9RYM9	DR_A0282	Uncharacterized	31.0364284515381
Q9RYX0	pdxY	Pyridoxal kinase	27.7197971343994
Q9WXF2	recN	DNA repair prote	27.0864887237549
Q9RRB8	DR_2574	Transcriptional r	29.7108612060547
Q9RRE7	DR_2545	NMO domain-con	29.2874050140381
Q9RRG5	DR_2527	Uncharacterized p	28.2597942352295
Q9RRH4	DR_2517	Uncharacterized p	26.5261917114258
Q9RRH7	DR_2514	PlsC domain-cont	28.6140022277832
Q9RRI7	DR_2503	Prolyl endopeptid	29.3374538421631
Q9RRS5	dnaX	DNA polymerase	26.1286468505859

Q9RRW1	DR_2374	Ribonucleoside-d	29.1031951904297
Q9RS52	uvrB	UvrABC system p	26.9133071899414
Q9RSD4	DR_2190	NADH-dependen	31.1160106658936
Q9RSD8	DR_2186	O-acetylhomoseri	26.5724639892578
Q9RSH0	DR_2154	Amino acid ABC	27.106294631958
Q9RSQ4	DR_2070	Membrane lipopr	31.411096572876
Q9RSU0	DR_2033	Glutamine syntha	29.4728202819824
Q9RSU8	DR_2024	Uncharacterized p	24.8130855560303
Q9RSY9	DR_1980	Epimerase domai	27.4316539764404
Q9RSZ9	DR_1970	Transcription elo	31.0855007171631
Q9RT24	DR_1945	Malonyl CoA-acy	30.9206790924072
Q9RT53	gryA	DNA gyrase subu	29.3591957092285
Q9RT93	DR_1872	Uncharacterized p	25.8609580993652
Q9RTA3	DR_1862	SUA5-related pro	26.470495223999
Q9RTE8	DR_1817	Phospho-2-dehyd	25.6546630859375
Q9RTN3	DR_1724	Amino_oxidase d	31.4053249359131
Q9RTU7	DR_1659	Oligopeptidase A	29.6933860778809
Q9RTV2	DR_1654	Uncharacterized p	29.6825771331787
Q9RU54	DR_1540	Isocitrate dehyd	32.114429473877
Q9RU76	DR_1516	Acetolactate synt	26.1331233978271
Q9RUB0	DR_1481	Uncharacterized p	29.1929340362549
Q9RUG1	DR_1425	Uncharacterized p	27.0534133911133
Q9RUH2	DR_1414	Uncharacterized p	27.3255157470703
Q9RUK5	DR_1379	Transcriptional re	28.0941104888916
Q9RUS6	DR_1306	Uncharacterized p	27.6449890136719
Q9RUT9	DR_1292	Molybdenum cofa	25.8184108734131
Q9RUU2	DR_1289	DNA helicase	26.8014392852783
Q9RV15	accD	Acetyl-coenzyme	28.9067859649658
Q9RVB5	DR_1114	Heat shock protei	30.0502510070801
Q9RVN7	DR_0990	Acetoin utilizatio	27.361686706543
Q9RVQ0	DR_0972	Uncharacterized p	30.746150970459
Q9RVU4	DR_0928	Endonuclease III,	27.4728202819824
Q9RVW4	DR_0907	Cold shock protei	27.4638080596924
Q9RVW5	DR_0906	DNA topoisomera	29.9579544067383
Q9RVX1	DR_0900	DUF3197 domain	27.9509372711182
Q9RW33	DR_0836	NurA domain-co	27.8383388519287
Q9RW48	DR_0821	Lipase, putative	27.3951740264893
Q9RWH2	DR_0696	V-type ATP synt	30.7758026123047
Q9RWH4	DR_0694	Uncharacterized p	31.3134899139404
Q9RWM2	DR_0644	Uncharacterized p	28.6933517456055
Q9RWQ4	DR_0612	Arginine utilizati	29.2590312957764
Q9RWV9	DR_0556	CoA_binding dom	26.4180736541748
Q9RWX7	DR_0538	Uncharacterized p	28.3094730377197
Q9RWX8	DR_0537	Uncharacterized p	27.1345729827881
Q9RX16	DR_0499	3-hydroxyisobuty	29.1953773498535
Q9RX81	DR_0434	Cytochrome C6, p	25.1121139526367
Q9RXC5	DR_0390	DhaL domain-co	29.1414604187012
Q9RXD2	DR_0383	S-layer-like array	26.6415481567383

Q9RXE8	DR_0365	Peptide ABC tran	27.7730388641357
Q9RXU3	DR_0213	N-acetyltransfera	26.5731296539307
Q9RXV1	DR_0205	ABC transporter,	25.6308155059814
Q9RXY9	DR_0165	Acyl-peptide hydı	26.940221786499
Q9RY02	DR_0152	Uncharacterized p	29.4752197265625
Q9RYB8	DR_0032	Dihydrolipoamide	29.8344707489014
Q9RYC0	DR_0030	2-oxo acid dehydı	27.730785369873
Q9RYC2	DR_0028	Aminotransferase	28.9773273468018
Q9RYI7	DR_A0325	N-glycosidase F, ı	29.0786209106445
Q9RYN5	DR_A0275	Cytochrome C6, p	28.7068500518799
Q9RYP8	DR_A0261	Branched-chain aı	26.3245906829834
Q9RYR1	DR_A0247	Drug transport prı	27.932300567627
Q9RYS1	DR_A0237	Oxidoreductase	27.5136737823486
Q9RYS9	DR_A0229	DUF1990 domain	25.7634963989258
Q9RYV4	DR_A0202	Superoxide dismu	26.5312576293945
Q9RYY7	DR_A0166	Uncharacterized p	25.5018196105957
Q9RZ09	DR_A0144	Uncharacterized p	27.2656192779541
Q9RZA3	DR_A0051	Uncharacterized p	26.4719829559326
Q9RZB3	DR_A0041	dTDP-glucose 4,6	28.0007762908936
Q9RZC4	DR_A0030	1-pyrroline-5-car	29.2945652008057
Q9RZC5	argD	Acetylornithine a	32.0951232910156
Q9RZE4	DR_A0005	Alcohol dehydrog	27.8235950469971
Q9RZQ2	DR_B0068	Uncharacterized p	29.1441860198975
Q9RZQ3	DR_B0067	Extracellular nucl	30.1003665924072

ation_6h

Control_6h		
**LFQ intensity C_6h_3**	**LFQ intensity C_6h_5**	**LFQ intensity R_6h_2**
26.5134468078613	26.1942844390869	30.2086238861084
26.6017284393311	26.3271961212158	29.142068862915
30.6307067871094	30.4633407592773	33.6970634460449
28.3496322631836	28.1963386535645	30.0476531982422
32.6572341918945	32.2988204956055	33.5134735107422
29.3825950622559	28.5372695922852	29.9688415527344
28.5288753509521	28.6020126342773	27.3084316253662
28.5459098815918	28.8478355407715	30.2346649169922
26.5215721130371	25.9904479980469	24.8979988098145
31.0019588470459	30.726146697998	32.0319671630859
31.1812744140625	30.5531635284424	32.0285797119141
31.8476715087891	31.7641429901123	30.4961948394775
27.8904113769531	27.9551563262939	26.3457717895508
32.7167129516602	32.9656295776367	30.8998756408691
29.1716918945313	28.9386520385742	27.9163856506348
27.2154903411865	27.788990020752	26.0142707824707
27.7228622436523	27.4545822143555	28.7525768280029
26.406702041626	26.070198059082	28.4145774841309
29.4091644287109	29.5872955322266	28.0597171783447
29.4967269897461	29.0950679779053	28.1165428161621
29.6098346710205	28.9421806335449	30.3085613250732
26.9205932617188	26.6981639862061	28.0165901184082
26.4097480773926	27.0115871429443	27.6448516845703
26.7902946472168	26.4460697174072	28.3734760284424
32.8646507263184	32.9588356018066	32.2880744934082
26.3761367797852	26.2005634307861	27.3822021484375
28.3298854827881	28.1184749603271	26.8438587188721
27.5377140045166	27.1689224243164	26.1810894012451
28.4188060760498	28.3762111663818	27.0828380584717
28.1497192382813	27.6574459075928	26.6357498168945
28.661563873291	28.7389240264893	32.0874633789063
29.4033241271973	32.1502532958984	31.8720283508301
26.5438766479492	28.1726455688477	28.9210205078125
30.992130279541	30.2013969421387	32.8860740661621
31.567512512207	31.6893329620361	30.2471714019775
30.6513004302979	30.8736438751221	29.9469833374023
27.8313484191895	28.2980651855469	27.0791797637939
27.2478046417236	27.0292854309082	28.1717872619629
29.559175491333	30.0939598083496	28.854211807251
29.3048686981201	29.3774528503418	28.1119251251221
28.0343837738037	28.0973300933838	28.8781242370605
26.9464817047119	27.4115905761719	27.9648818969727
28.3957462310791	28.4255447387695	27.0199851989746
29.1372394561768	29.1400470733643	28.4122371673584
26.2648124694824	26.1517238616943	26.7888031005859

28.9275054931641	28.9252433776855	31.0562725067139
26.2725410461426	27.1102828979492	28.3490409851074
31.2732543945313	31.354736328125	32.1384010314941
26.9584789276123	26.4946804046631	27.8290519714355
26.7021331787109	27.4429264068604	28.4941921234131
31.2482452392578	31.3040523529053	31.9194011688232
29.5877075195313	29.439661026001	30.6630573272705
25.3187236785889	25.3247451782227	27.2488899230957
27.1044960021973	27.4566974639893	28.3868141174316
31.3098087310791	30.8404865264893	30.0598201751709
31.3726978302002	30.9831848144531	30.5406551361084
29.2828388214111	29.3692169189453	30.9559459686279
26.5472354888916	25.9592761993408	25.1106014251709
26.4122371673584	26.270206451416	24.3769645690918
25.8552169799805	25.9360466003418	27.0888137817383
32.0627174377441	32.0059776306152	30.7757244110107
30.1426048278809	30.0199584960938	29.1105823516846
29.4802360534668	29.4321517944336	30.8891067504883
32.3292427062988	32.1837615966797	31.3672389984131
25.8572120666504	26.065523147583	26.7532787322998
29.0920963287354	28.7597694396973	30.1168155670166
27.0330715179443	27.5922775268555	28.0983333587646
27.9410610198975	27.7210369110107	26.7611637115479
28.1894283294678	27.3810482025146	25.4973487854004
27.1955413818359	27.6767387390137	28.5949478149414
26.9216156005859	26.2512226104736	25.6948204040527
26.4267845153809	26.1921215057373	27.9356212615967
28.8716773986816	29.0917167663574	28.137508392334
29.9264869689941	29.8032283782959	31.059175491333
27.1161460876465	26.7147636413574	26.0240077972412
30.3848400115967	30.4854850769043	31.4318618774414
28.4265842437744	27.8949279785156	27.295352935791
28.3259029388428	27.3923931121826	26.0053462982178
30.2445640563965	30.2910976409912	31.6988430023193
28.0841579437256	28.3711910247803	26.8772468566895
27.9176387786865	27.7888660430908	28.9013862609863
27.6445083618164	27.74560546875	29.0129451751709
30.8857650756836	31.7660388946533	31.9844760894775
32.3331680297852	31.6956520080566	30.851921081543
28.8488216400146	28.5801410675049	29.8036422729492
29.0388126373291	29.1722164154053	28.3139762878418
24.6821212768555	26.282190322876	26.7593879699707
28.3781547546387	28.4592018127441	27.0555858612061
27.0355911254883	26.9216156005859	25.499813079834
29.208646774292	29.3400249481201	28.6838817596436
26.2785778045654	25.7789897918701	24.4491596221924
28.9543533325195	29.0791301727295	27.9537696838379
27.2581787109375	28.0052280426025	29.061315536499

27.7116813659668	27.8430805206299	26.633810043335
26.1832313537598	25.1809768676758	27.0609550476074
25.5563716888428	25.740571975708	26.6049842834473
27.5647087097168	27.3919830322266	26.1778430938721
29.3404712677002	29.3670310974121	30.3602428436279
29.9540748596191	29.8901786804199	28.9013862609863
27.8083305358887	27.9890727996826	26.7356986999512
28.8121013641357	29.0591506958008	27.4912128448486
28.9701042175293	29.0360355377197	28.1617813110352
28.9942092895508	28.447229385376	27.0572395324707
26.0355491638184	26.0623149871826	25.0580863952637
27.8335819244385	27.8117027282715	28.9320201873779
27.5420761108398	28.1096858978271	28.6764698028564
25.6760787963867	25.5141544342041	26.7021331787109
26.3384971618652	27.0326519012451	27.5208072662354
25.8588485717773	25.5854606628418	27.6747875213623
27.325174331665	27.1288833618164	28.0636310577393
25.9698734283447	26.2054767608643	24.7251529693604
28.1438465118408	28.338623046875	26.1179695129395
29.0724754333496	29.5309791564941	28.5432205200195
31.849723815918	32.0737380981445	30.9297351837158
27.8650817871094	28.0442161560059	29.4952030181885
28.1462268829346	29.0740585327148	24.9488677978516
29.7869510650635	29.9641647338867	28.052791595459

Radiation_6h			
LFQ intensity R_6h_4	LFQ intensity R_6h_5	Peptides	Razor + unique peptides
30.1678218841553	29.7253665924072	10	10
29.0430698394775	29.367696762085	16	16
33.8261032104492	33.5608100891113	19	19
29.8925266265869	29.7945003509521	15	15
33.6333084106445	33.1622657775879	25	25
30.3948879241943	30.2321491241455	3	3
27.4804668426514	28.0093460083008	8	8
29.6566791534424	29.292459487915	11	11
24.7507762908936	25.0946235656738	3	3
31.9925689697266	31.8469619750977	5	5
32.127124786377	31.6675891876221	5	5
30.7365055084229	30.8795700073242	14	14
26.7006797790527	27.3250885009766	6	6
31.3009471893311	31.7526245117188	12	12
26.9793472290039	27.6389274597168	17	17
26.7025299072266	26.8460140228271	8	8
28.8525772094727	28.6931858062744	3	3
28.869441986084	28.5872421264648	11	11
27.3605995178223	28.7487754821777	7	7
28.049108505249	27.935396194458	5	5
30.5629272460938	30.1533966064453	6	6
28.0765838623047	27.6027908325195	9	9
27.5415592193604	27.3452816009521	3	3
27.9412860870361	28.0017967224121	7	7
32.0022583007813	31.9355087280273	13	13
27.7391166687012	27.4032440185547	6	6
26.8326759338379	27.3482398986816	4	4
25.6591892242432	26.5943431854248	2	2
27.4132061004639	27.3169994354248	4	4
27.0461444854736	27.2165088653564	3	3
32.3202056884766	32.0336112976074	13	13
32.1597557067871	32.1421165466309	7	7
29.1396083831787	29.7457981109619	4	4
32.8197402954102	32.963924407959	10	10
30.7016544342041	30.6601696014404	33	33
29.7706623077393	29.8810443878174	22	22
26.9419002532959	27.1357479095459	4	4
28.1723117828369	27.8688545227051	4	4
27.9413967132568	29.4220180511475	2	2
27.8013172149658	28.0925998687744	14	14
29.2043151855469	29.0004005432129	11	11
28.0867919921875	28.0418701171875	6	6
27.3280029296875	27.3831100463867	4	4
28.3009033203125	28.5008926391602	10	10
27.1628856658936	27.1420936584473	8	8

31.2849903106689	31.2283706665039	23	23
28.3337707519531	28.5638008117676	11	11
31.9514713287354	32.1926651000977	8	8
27.5493659973145	27.2639179229736	4	4
28.3485355377197	28.2931613922119	7	7
32.4690551757813	32.5253562927246	14	14
30.9200401306152	30.4979076385498	9	9
26.4979419708252	26.7013397216797	2	2
28.0339641571045	28.0958213806152	6	6
30.2230854034424	30.039701461792	6	6
27.726634979248	30.1798515319824	3	3
30.9626502990723	30.6790065765381	15	15
24.8964748382568	25.6720371246338	2	2
25.5124378204346	26.2460117340088	2	2
27.0495758056641	27.7739810943604	4	4
30.9207496643066	30.8340187072754	6	6
28.9605522155762	29.1358699798584	20	20
31.1689586639404	30.720775604248	1	1
31.2343215942383	31.5767307281494	18	18
26.8053817749023	27.2887687683105	6	6
30.1057453155518	29.8651123046875	7	7
27.9673023223877	28.4073829650879	4	4
26.4446334838867	26.9141063690186	6	6
24.3866004943848	25.7114315032959	5	5
29.0943374633789	28.4850826263428	6	6
24.9913558959961	25.4575443267822	5	5
28.0128650665283	27.7908554077148	9	9
27.7146968841553	27.9556560516357	9	9
31.3645801544189	31.0616245269775	5	5
26.2815532684326	25.9355754852295	6	6
31.5928115844727	31.3301525115967	7	7
26.1936264038086	26.8604125976563	3	3
25.4722156524658	26.6275615692139	4	4
31.6916236877441	31.6891670227051	22	22
26.6308975219727	27.1173362731934	6	6
29.1817245483398	29.0195617675781	4	4
28.3801803588867	28.581111907959	3	3
32.1311187744141	32.4538078308105	1	1
30.3927402496338	30.7990798950195	10	10
29.585111618042	29.4743690490723	3	3
28.1443328857422	28.0927505493164	10	10
26.9504928588867	26.6700668334961	5	5
27.3310813903809	27.4785404205322	10	10
25.8623065948486	25.4825916290283	6	6
27.9731178283691	28.1293754577637	9	9
24.3991737365723	23.9599609375	2	2
28.0137176513672	28.1103820800781	10	10
28.5820465087891	29.0612640380859	5	5

26.8390598297119	26.892728805542	5	5
26.7848148345947	26.908504486084	4	4
26.753662109375	26.3294906616211	4	4
25.8815231323242	26.3836536407471	8	8
30.5034160614014	30.3055210113525	12	12
28.7438106536865	28.980899810791	15	15
27.0936584472656	26.7261142730713	8	8
27.7336330413818	27.8336429595947	3	3
28.3070430755615	28.2561988830566	20	20
27.67431640625	28.2744884490967	4	4
25.1482181549072	25.2293834686279	2	2
29.1495742797852	29.1645660400391	3	3
28.9270820617676	28.641996383667	15	15
26.7755527496338	26.6331176757813	3	3
27.8069801330566	27.6045589447021	8	8
27.9656524658203	27.8081474304199	19	19
28.1622619628906	28.1117267608643	9	9
24.9070148468018	25.0843200683594	3	3
26.6003112792969	27.4334850311279	5	5
28.2386779785156	28.4371032714844	9	9
31.0587882995605	30.9350872039795	14	14
29.3373908996582	29.5724277496338	14	14
25.3291320800781	25.5294876098633	22	22
28.2442245483398	28.2927665710449	24	24

Unique peptides	Sequence coverage [%]
10	37.3
16	45.3
19	62.3
15	20.5
25	41.8
3	34.6
8	36.6
11	35.5
3	38.2
5	47.2
5	24.8
14	42.3
6	34.4
12	61
17	54.3
8	30.4
3	26.1
11	26.2
7	38.4
5	14.3
6	30.5
9	18
3	13.1
7	50.3
13	60.9
6	20.9
4	10.2
2	12.1
4	14.4
3	47.3
13	54.5
7	38.3
4	25.9
10	43.9
33	55.8
22	65.1
4	23.5
4	12.4
2	29.8
14	40.3
11	62.8
6	34.9
4	22.4
10	15
8	20.8

23	23.6
11	18.4
8	26.2
4	18.2
7	33.4
14	40.1
9	16.1
2	21.7
6	16.5
6	48.4
3	13.4
15	17.6
2	45.2
2	15.2
4	15.8
6	17.9
20	45.3
1	14.8
18	47.2
6	13
7	29.8
4	32
6	19.4
5	36.3
6	34.5
5	35.7
9	17.6
9	32
5	29.7
6	49.3
7	49.4
3	11.8
4	27.1
22	33.9
6	63.8
4	18.1
3	8.6
1	16.8
10	86.7
3	20.9
10	23.9
5	51.4
10	16.3
6	27.7
9	36.1
2	12.5
10	27.4
5	22.9

5	16.8
4	17.7
4	16.4
8	19.2
12	58.1
15	45.7
8	31.1
3	5.9
20	43.3
4	28.1
2	6.6
3	6.3
15	29.4
3	26.8
8	25.1
19	16.7
9	36.3
3	53.7
5	22.3
9	23.6
14	38.1
14	37
22	42.6
24	32.5

Unique + razor sequence coverage [%]	Unique sequence coverage [%]
37.3	37.3
45.3	45.3
62.3	62.3
20.5	20.5
41.8	41.8
34.6	34.6
36.6	36.6
35.5	35.5
38.2	38.2
47.2	47.2
24.8	24.8
42.3	42.3
34.4	34.4
61	61
54.3	54.3
30.4	30.4
26.1	26.1
26.2	26.2
38.4	38.4
14.3	14.3
30.5	30.5
18	18
13.1	13.1
50.3	50.3
60.9	60.9
20.9	20.9
10.2	10.2
12.1	12.1
14.4	14.4
47.3	47.3
54.5	54.5
38.3	38.3
25.9	25.9
43.9	43.9
55.8	55.8
65.1	65.1
23.5	23.5
12.4	12.4
29.8	29.8
40.3	40.3
62.8	62.8
34.9	34.9
22.4	22.4
15	15
20.8	20.8

23.6	23.6
18.4	18.4
26.2	26.2
18.2	18.2
33.4	33.4
40.1	40.1
16.1	16.1
21.7	21.7
16.5	16.5
48.4	48.4
13.4	13.4
17.6	17.6
45.2	45.2
15.2	15.2
15.8	15.8
17.9	17.9
45.3	45.3
14.8	14.8
47.2	47.2
13	13
29.8	29.8
32	32
19.4	19.4
36.3	36.3
34.5	34.5
35.7	35.7
17.6	17.6
32	32
29.7	29.7
49.3	49.3
49.4	49.4
11.8	11.8
27.1	27.1
33.9	33.9
63.8	63.8
18.1	18.1
8.6	8.6
16.8	16.8
86.7	86.7
20.9	20.9
23.9	23.9
51.4	51.4
16.3	16.3
27.7	27.7
36.1	36.1
12.5	12.5
27.4	27.4
22.9	22.9

16.8	16.8
17.7	17.7
16.4	16.4
19.2	19.2
58.1	58.1
45.7	45.7
31.1	31.1
5.9	5.9
43.3	43.3
28.1	28.1
6.6	6.6
6.3	6.3
29.4	29.4
26.8	26.8
25.1	25.1
16.7	16.7
36.3	36.3
53.7	53.7
22.3	22.3
23.6	23.6
38.1	38.1
37	37
42.6	42.6
32.5	32.5

Table S4. Differentially abundant proteins in the comparison Control_6h vs Radiation_6h

Mol. weight [kDa]	Q-value	Score	MS/MS count	Student's T-test p-value C_R
32.21		323.31	194	3.88070020570005E-05
42.662		323.31	174	3.08310167420951E-05
38.144		323.31	440	9.97623775819098E-06
112.1		100.93	259	9.89474183027509E-05
60.512		323.31	806	0.00544474560721352
8.9624		323.31	137	0.0133475601485111
33.587		310.38	114	0.019697782811797
32.501		67.311	157	0.0353211268687577
6.0671	0.0005992	5.8666	23	0.0489400177808421
23.617		323.31	257	0.00506654692180359
20.551		236.99	498	0.0219498524007691
55.067		323.31	414	0.00104835150334907
29.548		323.31	112	0.0287848043416974
29.755		323.31	538	0.00469834486485519
44.741		323.31	223	0.00611651445765236
43.351		181.5	113	0.02911560914014
17.661		136.5	156	0.00115826145936049
57.422		104.49	118	0.000154409692512964
29.98		216.32	193	0.0655320533344271
55.752		28.407	46	0.00130444990977586
38.032		134.82	145	0.0267228304310597
64.678		323.31	123	0.0147834138887468
38.179	0.0007067	16.144	40	0.0220541651315552
22.02		250.34	168	0.00578293053357874
26.57		323.31	450	0.00244650237370832
41.162		119.89	66	0.0201618329704769
50.47		296.93	95	0.0387197639033852
24.808		79.114	78	0.0255776831815378
33.268		191.29	103	0.000334342155720724
10.151		323.31	113	0.0154217844519366
21.156		323.31	317	6.01567147600291E-06
12.358		36.69	399	0.149238798010644
19.19		93.803	104	0.028583740102484
32.722		323.31	604	0.000808931572178716
76.754		323.31	1076	0.0018847510257668
54.834		323.31	721	0.00130180234106105
31.54		43.129	82	0.00852898219982529
59.798		323.31	95	0.00139604062325165
14.993		323.31	71	0.0846442120745524
51.204		323.31	277	0.000222623185096389
20.324		323.31	328	0.00152785049141502
35.523		323.31	190	0.0143337731032281
22.292	0.0007582	22.438	80	0.000728196658322385
75.236		323.31	196	0.000810058053250717
64.155		58.066	102	0.00271062867916903

152.91		323.31	415	1.69065041271881E-05
81.944		91.996	81	0.00332020166382688
39.579		323.31	356	0.00111950220854388
44.084		125.21	62	0.0159220200303493
32.986		179.27	127	0.00433248871794154
40.266		323.31	370	0.00781963447000542
86.943		323.31	319	0.00079896145292412
13.222	0.0006169	7.349	41	0.0040818067636158
34.991	0.0006901	13.989	105	0.00587744146974348
18.064		323.31	228	0.00275213779313288
31.284		140.41	110	0.1465574870376
89.824		323.31	233	9.60416182852079E-05
8.3483		231.04	40	0.0467464348529282
23.259		73.918	28	0.139751208998926
38.728		70.263	61	0.00396898132390842
47.068	0.0007231	18.009	56	0.010226505890524
76.967		323.31	300	0.00365796134979394
18.746		225.23	80	0.000774918661346707
47.471		323.31	587	0.00229525946158624
63.252		116.7	76	0.00800220232937791
33.085		323.31	367	0.00278468146245254
22.239		323.31	83	0.0143436374819592
53.823		323.31	120	0.0135801370739012
20.744		67.472	48	0.00510854512111204
24.97		260.96	155	0.00745658791311781
28.534		48.013	77	0.06771583243363
89.89		63.683	128	0.00159421758356308
31.042		137.75	104	0.00189041537750134
20.238		127.22	127	0.000569908197036346
22.866		95.265	40	0.0102513393150966
23.55		323.31	338	0.00229785555846791
38.26	0.0067603	1.6314	23	0.0532169953298519
14.371		323.31	105	0.0195944501634115
73.157		323.31	380	0.000125623311652444
15.516		105.69	107	0.00254312046477421
38.263		74.742	54	0.000187409456399765
49.499		94.965	64	0.00763560786752334
10.023		323.31	140	0.0377944382327537
11.72		323.31	327	0.0293025715891275
20.874		136.08	130	0.00181876844567191
57.561		216.94	208	0.000463045545048389
15.808		24.762	64	0.1508726871617
119.56		323.31	164	0.00113378285865062
44.135		323.31	99	0.000512607003166014
31.194		66.52	104	0.0110707302565709
36.267	0.0006374	8.8475	18	0.0174081361821271
58.63		323.31	199	0.000133486535547004
30.662		35.317	118	0.0197584858588805

36.567		93.173	96	0.000352253853695406
33.426		323.31	61	0.0902389048139854
38.537	0.000673	12.228	42	0.00244140002619887
72.625		69.248	68	0.00822289875970936
30.466		323.31	287	0.000158317465076215
55.589		323.31	388	0.000196955581821536
37.04		75.758	120	0.00228420588650148
59.761	0.0006281	8.2065	52	0.0005386073809504
69.82		323.31	353	0.000119903507008517
28.294		169.51	69	0.0530623065381337
63.013		41.618	73	0.000681318601567493
74.588		88.831	146	0.000128268656697141
77.371		319.61	265	0.00864731794296405
29.102		42.268	73	0.000231635749020863
48.294		310.71	123	0.010690544105141
179.05		100.18	156	9.24838978464402E-05
28.694		323.31	189	0.000174935777110352
12.666		105.71	97	0.00182554454955042
40.884		183.76	111	0.0219386674611896
57.035		125.4	247	0.00501378587599341
48.951		323.31	453	0.000317623891818278
41.986		221.25	174	8.72208610571683E-05
94.671		323.31	332	0.000639802366448048
109.78		323.31	454	0.000113875987200418

Student's T-test q-value C_R	Student's T-test Difference C_R
	-3.64053916931152
	-2.76386070251465
	-3.08350753784179
0.009	-1.5843194325765
0.0377283950617284	-0.957639058430992
0.00385714285714286	-1.44121424357096
0.0490909090909091	0.872350056966145
0.0420194174757282	-0.941918055216473
0.0394883720930233	1.02429962158203
0.0383132530120482	-0.952819188435871
0.03975	-0.94909985860189
0.0167692307692308	1.05415789286296
0.036	1.02430661519369
0.00432	1.48092206319173
0.0054	1.51896985371907
0.0369473684210526	1.02977752685547
0.0136666666666667	-1.08293596903483
0.0154285714285714	-2.34754371643066
0.0237818181818182	1.19368680318196
0.00552380952380952	1.18727238972982
0.0426538461538462	-0.920751571655273
0.0389387755102041	-0.930859883626301
0.0399529411764706	-0.990507125854492
0.00682352941176471	-1.31046485900879
0.0407472527472527	0.9107666015625
0.0404285714285714	-0.987691243489582
0.04004	0.957029978434246
0.0234461538461538	1.08978335062663
0.00527272727272727	1.1314442952474
0.0244137931034483	1.08468119303386
	-3.51130358378092
0.00968888888888889	-1.45752906799316
0.006	-1.81004778544108
0.0135	-2.4240042368571
0.0133877551020408	1.08818689982096
0.0227462686567164	0.987560907999676
0.0413725490196078	0.8974609375
0.0384657534246575	-0.949791590372723
0.040304347826087	1.04878997802734
0.004	1.3212947845459
0.0394468085106383	-0.897110621134441
0.024983606557377	-1.06976000467936
0.00627027027027027	1.23473167419434
0.0506551724137931	0.800235748291016
0.0469811320754717	-0.849533081054688

0.012	-2.20456314086914
0.0072	-1.65016047159831
0.0464	-0.846178690592449
0.048697247706422	-0.872237523396809
0.00662857142857143	-1.29484494527181
0.0379459459459459	-0.983472824096676
0.0058	-1.19360542297363
0.00771428571428571	-1.6638724009196
0.0498018018018018	-0.84125073750814
0.0372222222222222	0.971062978108726
0.00565853658536585	1.60980669657389
0.00675	-1.52878379821777
0.0504561403508772	0.896118799845379
0.048	1.00584157307943
0.00490909090909091	-1.48881467183431
0.0382	0.981175740559898
0.0417821782178218	0.882981618245445
0.00415384615384615	-1.39462534586588
0.0500173913043478	0.816380818684895
0.0398709677419355	-0.930523554484051
0.0230909090909091	-1.01429112752279
0.039340206185567	-0.931418736775715
0.0416629213483146	0.955903371175129
0.018	2.68973541259766
0.0053953488372093	-1.21903292338054
0.0465420560747664	0.949176152547203
0.0045	-1.43966547648112
0.024	1.02077293395996
0.00644444444444444	-1.23513793945313
0.0364675324675325	0.983819961547852
0.0412	-0.912783304850262
0.0254	1.14831352233887
0.00568421052631579	1.69232686360677
0.00635294117647059	-1.5286725362142
0.00610526315789474	1.26026852925618
0.00594871794871795	-1.18594296773275
0.0248421052631579	-1.06298319498698
0.03744	-1.04726537068685
0.0238125	1.09952290852865
0.0410454545454545	-0.913602828979492
0.0224117647058823	0.973000208536785
0.0493571428571429	-0.999187469482422
0.0139574468085106	1.09387397766113
0.00514285714285714	1.41568946838379
0.0355443037974684	0.985891342163086
0.00372413793103448	1.45379575093587
0.0149803921568627	1.03235816955566
0.00469565217391304	-1.5998903910319

0.0241904761904762	0.987400690714516
0.0500530973451327	-0.938978830973308
0.0415172413793103	-0.920125961303711
0.00947826086956522	1.15129788716634
0.0252857142857143	-0.995485941569012
0.0219259259259259	1.01754252115886
0.0235428571428571	0.99090576171875
0.00725	1.26336352030436
0.0502222222222222	0.786579132080078
0.038780487804878	1.04741477966309
0.0245806451612903	0.995588938395183
0.00703030303030303	-1.22285842895508
0.0238840579710145	-1.02670415242513
0.01528	-1.05235799153646
0.0349295774647887	-1.00997988382975
0.0108	-2.1674861907959
0.0390315789473684	-0.872647603352867
0.00748387096774194	1.31028175354004
0.00773333333333333	1.44382667541504
0.0404444444444444	0.893006006876629
0.0182641509433962	1.03165817260742
0.00830769230769231	-1.55737622578939
	3.5189946492513
0.00981818181818182	1.75389989217122

Fold change C_R	Student's T-test Test statistic C_R
0.0801841462824705	-1.66662709093517
0.147229563566222	-1.29631494779946
0.117970043417452	-1.46071029566614
0.333481946742867	-0.753845088810512
0.514898845896462	-0.440260504151194
0.368257230337641	-0.615760583107243
1.83064247128926	0.390885133215999
0.520540364588727	-0.409295266347396
2.03397172174239	0.432895619804379
0.516621937509032	-0.438916647632076
0.517955529362414	-0.419828048734724
2.07650578765856	0.49631958902877
2.03398158165209	0.444094844811099
2.79127073503991	0.65525662335729
2.86586341558417	0.6641806167873
2.04170938200841	0.445930792566656
0.472067160811739	-0.508253552240768
0.19648026048122	-1.08249920646749
2.28736532495368	0.482461803356492
2.27721797037405	0.552776900551083
0.528233765329329	-0.40578870565247
0.524545605848742	-0.418040611427633
0.503300827219148	-0.435874106201658
0.403190944221693	-0.584051529286642
1.88004422785741	0.426791517201162
0.504284139616473	-0.436210627211546
1.94130927849241	0.413281610763881
2.12842071617546	0.470944950020057
2.19077951454703	0.539041492905167
2.12090675265657	0.478350928306557
0.0876985266888783	-1.66310384967042
0.364116224561766	-0.517283218921852
0.285181482931559	-0.712504123973702
0.186338251117304	-1.06954380995267
2.1260667608783	0.506291745181379
1.98282988468327	0.465194222120273
1.86278469970305	0.410505746465435
0.517707243754574	-0.447931865869017
2.0687939740779	0.426391392924704
2.49890279764706	0.627936833843597
0.536961061814552	-0.423874744581249
0.476398242269084	-0.473729252024248
2.35337573108342	0.579132192583621
1.7413856601504	0.383259196698056
0.554964317594291	-0.399129930550273

0.216950356075545	-1.05452396302646
0.318604716317335	-0.729007373858607
0.556256158760461	-0.402708395804513
0.546298920216925	-0.393394074598737
0.407579968809298	-0.582639317413308
0.505760816972435	-0.447215207508557
0.437208870460251	-0.560180513261389
0.315590919214514	-0.729407312103704
0.558159465563962	-0.389976699319044
1.96028440130408	0.452195414239594
3.05210944532265	0.556012885141316
0.346569404292818	-0.728912131677194
1.8610525589171	0.3870504057026
2.00811455693776	0.394922611130031
0.356305171451306	-0.66180370296415
1.97407354706633	0.443070398299183
1.84418273846133	0.411686033305063
0.380343447049833	-0.648191554089935
1.76098280078544	0.385436834570746
0.524667905111087	-0.424969934079142
0.495071523313267	-0.470779623783481
0.524342453286597	-0.418647661693281
1.93979389282182	0.429228730569318
6.45195069033547	1.08309507695394
0.429570574239741	-0.543363409260787
1.93076978164988	0.398531289859622
0.368652775419289	-0.657684672132867
2.02900572244257	0.476954693836251
0.424801881324358	-0.581525924766473
1.97769501457987	0.444118285537258
0.531159368260376	-0.428125077265695
2.21654633687139	0.47395619232107
3.23177523332124	0.691077744381669
0.346596133083127	-0.726485489206059
2.39540322485992	0.57616608689189
0.439537155787716	-0.567573812837174
0.478641304445368	-0.480172193501103
0.483884499089171	-0.447041740394765
2.14283818285704	0.47171497331563
0.530857728416781	-0.430094666653299
1.96291841144029	0.464980944345462
0.50028168093305	-0.389776407936303
2.13446421815628	0.513246554827244
2.66787203379696	0.66207947913308
1.98053657240707	0.443988304532376
2.7392781201011	0.612922656697847
2.04536478908111	0.498371213387851
0.329902041097248	-0.65964133312198

1.98260969540372	0.472994053121655
0.521601950305204	-0.387646241967566
0.528462878319127	-0.43091462586227
2.22113623934488	0.514792174583529
0.501566903506398	-0.480454860130569
2.02446755390429	0.489729201377956
1.98743236133781	0.462365588445001
2.40054756189549	0.59453607476669
1.72497939880211	0.383138884376174
2.0668229084749	0.439117154477492
1.9938943090478	0.473044012440706
0.428433017269604	-0.586840957845809
0.490830174144736	-0.463755557674804
0.482179429045098	-0.505013076689207
0.496553171342854	-0.454200662152241
0.222598196521672	-1.01440466638092
0.546143659129439	-0.422649362118939
2.47989966874032	0.60151900337627
2.72041485604112	0.602470587613865
1.8570414366672	0.41349455891666
2.04437261694285	0.493832883899214
0.339768444160251	-0.742746608860725
11.463650685872	1.48864146834783
3.37269040371728	0.828635023231179

Table S7. Differentially abundant proteins in the comparison Control_12h vs Radia

Uniprot Accession	Gene name	Protein name	LFQ intensity C_12h_3
Q9RZV3	DR_B0008	Cob(I)alamin ade	25.6087665557861
Q9RZ22	DR_A0131	Uncharacterized p	26.5929183959961
Q9RZ24	DR_A0129	Uncharacterized p	26.8820362091064
Q9RZ33	DR_A0120	Bac_luciferase do	27.9069004058838
Q9RZ81	DR_A0073	Cation-transporti	26.1749897003174
Q9RZ87	DR_A0067	CoA transferase,	30.6003646850586
Q9RZA6	DR_A0048	Mannose-6-phosp	26.7235126495361
Q9RZB2	DR_A0042	Glucose-1-phosph	26.6695251464844
Q9RZB3	DR_A0041	dTDP-glucose 4,6	27.139461517334
Q9RZC4	DR_A0030	1-pyrroline-5-car	29.4599056243896
Q9RZD6	DR_A0013	Ferredoxin-nitrite	29.2692356109619
Q9RZE4	DR_A0005	Alcohol dehydrog	29.5657253265381
Q9RZF3	DR_C0037	Nodulation protei	27.9178657531738
Q9RZH5	DR_C0009	KAP NTPase dom	26.5703659057617
Q9RZK2	DR_B0125	Iron ABC transpo	27.1327114105225
Q9RZN4	DR_B0089	Uncharacterized p	29.2045021057129
Q9RZP7	DR_B0073	PTS system, fruct	28.1671524047852
Q9RZS7	DR_B0037	Uncharacterized p	29.0048809051514
Q9RZT2	DR_B0031	Chromosome part	27.137508392334
Q9RZU0	DR_B0023	Hydrolase_4 dom	27.2446327209473
Q9RZU7	DR_B0014	Hemin ABC trans	27.193286895752
Q9RY25	DR_0127	Uncharacterized p	26.9829406738281
Q9RY46	DR_0105	Uncharacterized p	30.5863838195801
Q9RY54	DR_0096	ABC transporter,	25.2539291381836
Q9RY55	DR_0095	ABC transporter,	25.8423252105713
Q9RY56	DR_0094	Uncharacterized p	31.5207042694092
Q9RY79	DR_0071	Peptidyl-prolyl ci	29.1136150360107
Q9RYC0	DR_0030	2-oxo acid dehydr	27.2897357940674
Q9RYC7	purE	N5-carboxyaminc	26.3907051086426
Q9RYE8	DR_0001	Beta sliding clam	29.2331104278564
Q9RYF4	DR_A0364	Oxidoreductase, s	30.1438217163086
Q9RYK8	DR_A0304	Uncharacterized p	27.8145179748535
Q9RYL1	DR_A0301	Methylamine utili	27.730655670166
Q9RYL2	DR_A0300	Uncharacterized p	28.6913566589355
Q9RYL8	DR_A0294	bMG10 domain-c	25.3605155944824
Q9RYN0	DR_A0281	Uncharacterized p	28.9074745178223
Q9RYN5	DR_A0275	Cytochrome C6, p	29.0407199859619
Q9RYN6	DR_A0274	Flavin monoamin	32.1964492797852
Q9RYN9	DR_A0271	Sugar transporter,	26.670877456665
Q9RYP8	DR_A0261	Branched-chain a	25.1064147949219
Q9RYQ0	DR_A0259	Catalase	31.8250217437744
Q9RYQ2	DR_A0257	Ankyrin-related p	28.1355037689209
Q9RYQ3	DR_A0256	Phenylacetate-coe	27.8370151519775
Q9RYQ4	DR_A0255	Aculeacin A acyla	30.2628211975098
Q9RYR1	DR_A0247	Drug transport pro	26.8597011566162

Q9RYR7	DR_A0241	Beta lactamase-re	29.0272827148438
Q9RYS1	DR_A0237	Oxidoreductase	28.5074481964111
Q9RYS5	DR_A0233	Oxidoreductase, i	25.5271911621094
Q9RYS7	DR_A0231	Oxidoreductase	29.115650177002
Q9RYS8	DR_A0230	DUF1990 domain	29.0810623168945
Q9RYT5	DR_A0223	4-hydroxyphenyla	29.0593299865723
Q9RYT6	DR_A0222	TDP-glucose-4,6-	28.3916969299316
Q9RYT8	DR_A0220	Aldehyde dehydro	29.4969940185547
Q9RYV3	DR_A0203	Oxidoreductase, p	29.0155792236328
Q9RYV4	DR_A0202	Superoxide dismu	28.7387943267822
Q9RYW8	DR_A0188	Excinuclease ABC	28.4685153961182
Q9RYW9	DR_A0185	Exopolyphosphata	27.5515689849854
Q9RYX6	DR_A0178	Xanthine dehydro	28.8252639770508
Q9RZ07	DR_A0146	Uncharacterized p	32.1827850341797
Q9RZ08	DR_A0145	Peroxidase, putati	28.9582023620605
Q9RZ09	DR_A0144	Uncharacterized p	27.5689182281494
Q9RZ10	DR_A0143	3-hydroxyacyl-Co	28.7257251739502
Q9RX33	DR_0482	B-cell receptor as	24.9261035919189
Q9RX59	DR_0456	Biopolymer transp	28.4606475830078
Q9RX86	DR_0429	Cytochrome-relat	28.530216217041
Q9RXB8	DR_0397	Molybdopterin ox	28.9720230102539
Q9RXC3	DR_2592	Uncharacterized p	26.2152500152588
Q9RXD2	DR_0383	S-layer-like array-	29.5117321014404
Q9RXF0	DR_0363	Peptide ABC tran	32.7216110229492
Q9RXG0	rnr	Ribonuclease R	29.6354370117188
Q9RXG3	DR_0350	Uncharacterized p	26.556224822998
Q9RXI1	DR_0332	Uncharacterized p	29.6010036468506
Q9RXI3	DR_0330	Uncharacterized p	28.380428314209
Q9RXM4	DR_0286	Uncharacterized p	28.2872714996338
Q9RXN0	DR_0280	Branched-chain ai	28.0271244049072
Q9RXU0	DR_0216	SufE domain-cont	28.3172149658203
Q9RXX3	DR_0182	Glutamate synthas	29.3361358642578
Q9RXZ4	DR_0160	Uncharacterized p	28.6105213165283
Q9RY02	DR_0152	Uncharacterized p	30.9281826019287
Q9RY13	hflx	GTPase HflX	27.0744934082031
Q9RTS5	DR_1681	GidA-related prot	27.5671787261963
Q9RTT5	DR_1671	Uncharacterized p	29.9102783203125
Q9RTV1	DR_1655	ABC transporter,	28.3665714263916
Q9RTV5	DR_1649	Immunogenic pro	29.3875732421875
Q9RTW5	DR_1638	Corrinoid adenosy	26.5558013916016
Q9RTW8	DR_1634	FecR domain-con	28.0518589019775
Q9RTX7	DR_1623	PrcB_C domain-c	30.3266735076904
Q9RTY0	DR_1620	Ketoacyl reductas	30.3006210327148
Q9RTZ3	DR_1604	Oligosaccharide d	27.5659446716309
Q9RTZ7	DR_1600	Uncharacterized p	28.872615814209
Q9RTZ8	DR_1599	Serine protease	28.5243625640869
Q9RTZ9	DR_1598	Protease, putative	30.1526718139648
Q9RU07	DR_1589	Fructose-bisphosp	32.0710868835449

Q9RU08	DR_1588	Aminotransferase	29.9377536773682
Q9RU55	DR_1539	Uncharacterized p	28.0782127380371
Q9RU56	DR_1538	Osmotically induc	28.7512683868408
Q9RU91	DR_1501	NADH dehydroge	27.0437469482422
Q9RU92	DR_1500	NADH-quinone o	28.0860328674316
Q9RU93	DR_1499	NADH-quinone o	29.3989696502686
Q9RUA1	DR_1491	Carboxyl-termina	27.1894283294678
Q9RUB0	DR_1481	Uncharacterized p	30.132345199585
Q9RUB8	DR_1472	Alpha-amylase	27.2463550567627
Q9RUC0	DR_1469	MurNAc-LAA do	27.2569198608398
Q9RUD0	DR_1459	Serine protease, s	29.7887096405029
Q9RUD5	DR_1452	Aminotransferase	29.8741264343262
Q9RUE0	DR_1447	Uncharacterized p	32.2422103881836
Q9RUF4	DR_1432	Uncharacterized p	29.8566341400146
Q9RUF9	DR_1427	Signal peptidase I	29.0547847747803
Q9RUG9	DR_1417	Penicillin-insensi	30.1390705108643
Q9RUK0	ychF	Ribosome-bindin	26.8729095458984
Q9RUK9	DR_1375	Glycosyl hydrolas	29.2612972259521
Q9RUL0	topA	DNA topoisomera	28.6682758331299
Q9RUR7	DR_1315	DUF2382 domain	30.483757019043
Q9RUV4	DR_1277	ABC transporter,	26.37965965271
Q9RUW2	DR_1268	DinB_2 domain-c	26.745735168457
Q9RV11	DR_1219	Ferrous iron trans	26.8178749084473
Q9RV31	DR_1199	Protease I	26.8631343841553
Q9RV32	bipA	50S ribosomal sul	28.24049949646
Q9RV70	DR_1160	Uricase	29.4911365509033
Q9RV78	DR_1151	3-hydroxybutyryl	28.9912376403809
Q9RV83	DR_1146	General stress pro	30.6706733703613
Q9RV99	DR_1130	Coproporphyrinog	28.4691753387451
Q9RVA5	DR_1124	SLH family prote	31.0500564575195
Q9RVF7	DR_1072	Acetyl-CoA acety	26.2879409790039
Q9RVG6	DR_1063	Peptidyl-prolyl ci	32.4064407348633
Q9RVL6	mqnD	1,4-dihydroxy-6-r	28.8171119689941
Q9RVQ1	DR_0971	Electron transfer f	28.9095916748047
Q9RVQ3	DR_0969	Uncharacterized p	30.0126533508301
Q9RVR2	DR_0960	WD_REPEATS_	30.9780788421631
Q9RVT5	DR_0937	Tetratricopeptide	28.5075244903564
Q9RVU0	DR_0932	Polyprenyl syntha	27.8053207397461
Q9RVW4	DR_0907	Cold shock protei	25.6511631011963
Q9RVW5	DR_0906	DNA topoisomera	29.908561706543
Q9RVX7	DR_0894	Uncharacterized p	29.6377716064453
Q9RVZ4	DR_0876	Nudix hydrolase c	29.0718612670898
Q9RVZ5	DR_0875	Aminopeptidase	26.6544437408447
Q9RW23	DR_0846	Bacterioferritin c	31.5578708648682
Q9RW25	DR_0844	Thioredox_DsbH	28.3341541290283
Q9RW56	DR_0813	1-pyrroline-5-car	30.7272033691406
Q9RW64	tatA	Sec-independent p	27.1197128295898
Q9RW82	DR_0787	S4 RNA-binding	29.391429901123

Q9RW88	DR_0781	Response regulat	30.5105075836182
Q9RW97	DR_0772	Uncharacterized p	26.2425632476807
Q9RWH2	DR_0696	V-type ATP synth	29.7590866088867
Q9RWK8	DR_0658	Lactamase_B dom	29.481294631958
Q9RWP6	DR_0620	TGc domain-cont	28.2171096801758
Q9RWR9	DR_0597	Uncharacterized p	27.1817722320557
Q9RWS4	DR_0591	Uncharacterized p	25.6724414825439
Q9RWT0	DR_0585	Aminopeptidase	30.9230194091797
Q9RWU6	DR_0569	Uncharacterized p	28.6026134490967
Q9RWV3	DR_0562	Maltose/maltodex	27.4873085021973
Q9RWV5	DR_0560	Thioredoxin doma	29.8567218780518
Q9RX16	DR_0499	3-hydroxyisobuty	27.6931858062744
Q9RX28	DR_0487	Uncharacterized p	24.4150619506836
O32504	pprA	DNA repair prote	26.5487632751465
O32508	cinA	CinA-like protein	26.0941410064697
P42443	recA	Protein RecA	30.5434226989746
P56861	cysC	Adenylyl-sulfate	27.2669582366943
P56864	sat	Sulfate adenylyltr	26.7193412780762
P83589	DR_2477	Probable 3-hydro	31.1844711303711
Q59337	katA	Catalase	32.8910255432129
Q9R342	tufA	Elongation factor	32.1091766357422
Q9RR63	tyrS	Tyrosine--tRNA l	27.5952682495117
Q9RR76	gpsA	Glycerol-3-phosp	25.5100536346436
Q9RRB6	slpA	S-layer protein S	31.3996829986572
Q9RRB7	ppaC	Probable mangane	31.0319671630859
Q9RRC4	argS	Arginine--tRNA l	29.2837657928467
Q9RRE4	DR_2548	Probable transcri	27.6239395141602
Q9RRJ5	hisA	1-(5-phosphoribos	27.9749794006348
Q9RRT0	menG	Demethylmenaqu	25.3558521270752
Q9RRT8	surE	5'-nucleotidase Su	28.1994323730469
Q9RSG0	ispH	4-hydroxy-3-meth	28.5450630187988
Q9RSN7	infC	Translation initiat	30.0715026855469
Q9RSP3	thrS	Threonine--tRNA	29.3777828216553
Q9RSQ7	nusB	Transcription anti	24.8931922912598
Q9RSS6	nusG	Transcription tern	31.2994747161865
Q9RSS8	rplA	50S ribosomal pro	29.5510177612305
Q9RSW7	rplT	50S ribosomal pro	29.3410224914551
Q9RTG5	infB	Translation initiat	29.5513668060303
Q9RTJ3	DR_1769	Desiccation/radia	30.6511287689209
Q9RTL8	pgi	Glucose-6-phosph	27.5245494842529
Q9RU24	DR_1571	Probable ABC tra	33.5049781799316
Q9RU89	nuoD	NADH-quinone o	26.5133266448975
Q9RUK6	msrB	Peptide methionin	28.2477149963379
Q9RUR8	DR_1314	Uncharacterized	33.5600128173828
Q9RUS3	rpsT	30S ribosomal pro	29.838249206543
Q9RV58	DR_1172	Protein DR_1172	29.0098533630371
Q9RV66	panC	Pantothenate synt	28.5713081359863
Q9RV69	DR_1161	5-hydroxyisourate	30.1117267608643

Q9RV96	hemE	Uroporphyrinoger	30.9003791809082
Q9RV97	ilvD	Dihydroxy-acid d	28.0596656799316
Q9RV98	hemH	Ferrochelatase	29.5313148498535
Q9RVE7	hpf	Ribosome hiberna	28.7592926025391
Q9RVY3	DR_0888	Uncharacterized	32.1758728027344
Q9RXI7	ddrD	DNA damage resp	28.4221782684326
Q9RXJ0	rplE	50S ribosomal pro	30.1568965911865
Q9RXJ6	rpsC	30S ribosomal pro	28.3999080657959
Q9RXK2	rplC	50S ribosomal pro	31.0847396850586
Q9RXK5	fusA	Elongation factor	31.1978282928467
Q9RXK6	rpsG	30S ribosomal pro	31.6721935272217
Q9RXP1	DR_0269	Uncharacterized	27.3668632507324
Q9RXT4	purL	Phosphoribosylfo	29.0146484375
Q9RXT9	DR_0217	Thiosulfate sulfur	30.2452449798584
Q9RXU7	trmFO	Methylenetetrahy	26.6723613739014
Q9RY24	grpE	Protein GrpE	30.0175724029541
Q9RY51	ssb	Single-stranded D	29.4711151123047
Q9RY66	obg	GTPase Obg	27.7440032958984
Q9RYE3	DR_0006	UPF0173 metal-d	27.5809688568115
Q9RYM8	DR_A0283	Probable subtilas	32.8999671936035
Q9RYM9	DR_A0282	Uncharacterized	31.7853450775146
Q9RYP0	ade	Adenine deamina	28.110631942749
Q9RYR5	hmp	Flavohemoprotein	27.1476345062256
Q9RZ89	hup	DNA-binding pro	29.9918594360352
Q9RZE7	parB2	Probable chromos	26.1862010955811
Q9RZN1	dps2	DNA protection d	26.561653137207
Q9RRA0	DR_2594	Magnesium proto	30.1074924468994
Q9RRA2	DR_0392	BON domain-con	30.1135902404785
Q9RRC5	DR_2567	N-acetylmuramoy	29.5956077575684
Q9RRC7	DR_2565	Iron-sulfur bindin	27.5299186706543
Q9RRE0	mltG	Endolytic murein	27.4466381072998
Q9RRF0	DR_2542	Peptidyl-prolyl ci	28.3754234313965
Q9RRF4	DR_2538	Cytochrome P450	26.6982955932617
Q9RRG1	DR_2531	Thiosulfate sulfur	29.0057373046875
Q9RRH4	DR_2517	Uncharacterized p	29.464334487915
Q9RRI7	DR_2503	Prolyl endopeptid	28.6152648925781
Q9RRK0	DR_2489	Uncharacterized p	27.6005954742432
Q9RRK9	DR_2480	Acetyl-CoA acety	30.0240612030029
Q9RRM4	DR_2464	Peptidyl-prolyl ci	29.5154228210449
Q9RRP3	DR_2445	Uncharacterized p	25.6544437408447
Q9RRP5	DR_2443	Ribosomal protein	28.7594509124756
Q9RRR8	DR_2418	DNA-binding resp	25.4605464935303
Q9RRV0	DR_2385	Phenylacetic acid	28.3002490997314
Q9RRV1	DR_2384	Phenylacetic acid	31.3590908050537
Q9RRV2	DR_2383	Phenylacetic acid	30.4109439849854
Q9RRV3	DR_2382	Uncharacterized p	29.3381767272949
Q9RRV4	DR_2381	Aldehyde dehydro	30.6139488220215

Q9RRV7	DR_2378	Uncharacterized p	25.6315650939941
Q9RRW1	DR_2374	Ribonucleoside-d	29.4104595184326
Q9RRX1	DR_2361	Acyl-CoA dehydr	30.9024658203125
Q9RRZ0	DR_2342	Uncharacterized p	28.3802623748779
Q9RRZ5	DR_2334	Uncharacterized p	26.7192096710205
Q9RRZ6	DR_2333	NADH oxidase-re	25.3904418945313
Q9RS17	DR_2310	Uncharacterized p	32.172061920166
Q9RS52	uvrB	UvrABC system p	27.2190971374512
Q9RS70	DR_2257	Erythromycin este	25.0263957977295
Q9RS85	DR_2242	Thiol-specific ant	28.0055503845215
Q9RS98	DR_2229	Uncharacterized p	29.669340133667
Q9RSA6	DR_2220	Tellurium resistar	27.0061378479004
Q9RSD4	DR_2190	NADH-dependen	32.2991485595703
Q9RSE4	DR_2180	Robl_LC7 domai	27.0004539489746
Q9RSE5	DR_2179	Uncharacterized p	28.088207244873
Q9RSL6	DR_2108	Molybdate metab	28.4267845153809
Q9RSL7	DR_2107	ABC transporter,	28.0362720489502
Q9RSL8	DR_2106	Uncharacterized p	28.7158432006836
Q9RSM3	DR_2101	Uncharacterized p	29.0544738769531
Q9RSM9	DR_2095	C-type cytochrom	26.3962211608887
Q9RST1	DR_2042	Uncharacterized p	28.9882068634033
Q9RSU3	DR_2030	Uncharacterized p	29.4035682678223
Q9RSV3	DR_2019	Thioredoxin-like_	28.1077919006348
Q9RSZ3	mutS2	Endonuclease Mu	26.1575374603271
Q9RSZ5	lon	Lon protease	27.4084358215332
Q9RT43	DR_1923	Uncharacterized p	29.091968536377
Q9RT53	gryA	DNA gyrase subu	29.3669681549072
Q9RT82	DR_1883	MOSC domain-co	26.5947704315186
Q9RT96	DR_1869	Polyferredoxin, pu	25.9061431884766
Q9RTE2	DR_1823	Lactamase_B don	28.8265075683594
Q9RTG7	nusA	Transcription tern	30.5415782928467
Q9RTJ4	DR_1768	Uncharacterized p	31.251916885376
Q9RTK5	DR_1755	Uncharacterized p	28.5178833007813
Q9RTL6	DR_1744	Uncharacterized p	28.8132934570313
Q9RTM0	DR_1740	Uncharacterized p	28.6668872833252
Q9RTP5	DR_1712	Extracellular solu	28.697566986084

ation_12h

**Control_12h**		
**LFQ intensity C_12h_4**	**LFQ intensity C_12h_5**	**LFQ intensity R_12h_2**
25.9617938995361	25.7559299468994	26.6891250610352
26.5936317443848	26.5216617584229	25.6930923461914
26.7794399261475	26.9731178283691	26.280969619751
28.042652130127	27.936014175415	27.2968406677246
25.9810905456543	25.7807426452637	26.6031455993652
30.4801006317139	30.1410217285156	29.2457218170166
26.3412303924561	26.4276161193848	25.7224712371826
26.6274223327637	26.6518478393555	27.3303985595703
26.7861881256104	27.1038951873779	28.4503002166748
29.1146087646484	29.4012718200684	30.3005123138428
29.056827545166	29.5202465057373	30.8329639434814
29.7687549591064	29.520694732666	28.7711811065674
27.5065784454346	27.5700054168701	28.3901386260986
26.8485221862793	26.583911895752	27.5940589904785
27.3553142547607	27.3074760437012	31.7175121307373
29.0556125640869	28.6044178009033	27.3228549957275
28.0870952606201	28.0830421447754	29.0781116485596
29.1644687652588	29.0693283081055	28.3952980041504
27.325345993042	27.1933822631836	28.75439453125
27.7376346588135	27.5125427246094	25.1396179199219
28.2772407531738	27.4228610992432	31.0160446166992
26.7471446990967	27.107292175293	27.8136005401611
30.3992767333984	30.6495018005371	28.7335357666016
25.2192993164063	25.4303932189941	26.908390045166
26.0207710266113	25.977840423584	27.0191383361816
31.3107299804688	31.8336791992188	30.0652236938477
29.0154991149902	29.2533168792725	30.4008026123047
27.5901374816895	27.3203601837158	28.078519821167
27.0469760894775	26.5475444793701	27.5654354095459
29.4511070251465	29.2400665283203	30.0986099243164
30.0943374633789	30.2102508544922	29.4537963867188
27.8757820129395	28.0179176330566	28.8281440734863
27.7078704833984	27.4497890472412	25.1360607147217
29.0749778747559	27.953104019165	27.7661037445068
26.256685256958	25.2051219940186	24.8527183532715
27.881628036499	28.8318920135498	30.086893081665
29.0449199676514	28.8615379333496	27.4085178375244
32.2698020935059	31.9661273956299	30.4408493041992
26.5994606018066	26.5479698181152	25.7026882171631
25.1340255737305	25.1359825134277	25.6860294342041
31.9295597076416	32.0662841796875	30.997522354126
28.2946968078613	28.1807289123535	27.3154449462891
27.6990909576416	27.7650909423828	26.0641250610352
29.9678802490234	29.9359302520752	28.7823810577393
26.5093288421631	26.7438106536865	27.5099029541016

29.0173072814941	29.0085716247559	27.8985633850098
28.9185199737549	28.9012718200684	27.5859184265137
25.7608337402344	25.6157131195068	24.4656925201416
29.1397304534912	29.3728942871094	28.1555213928223
29.3607654571533	29.2159538269043	28.1904640197754
28.6874904632568	28.770299911499	27.8473873138428
27.9063282012939	28.3739738464355	27.6118221282959
29.420051574707	30.1058692932129	29.1655235290527
29.2028675079346	29.1030464172363	26.8145790100098
28.9861450195313	28.7904815673828	27.679895401001
28.1383380889893	28.3577499389648	27.4104595184326
27.4478206634521	27.4303779602051	26.6754608154297
28.844877243042	28.666446685791	29.2345504760742
32.2919769287109	32.417724609375	31.3076400756836
28.9557933807373	29.113639831543	27.494649887085
27.8689136505127	28.0543441772461	26.9329204559326
28.8954467773438	28.9266567230225	27.9375858306885
25.1821517944336	24.9261932373047	26.2460842132568
28.3121585845947	28.1870212554932	29.4353942871094
28.1829090118408	28.3840560913086	26.686990737915
28.8866672515869	28.7791271209717	25.6863231658936
26.3396015167236	26.2214031219482	25.2787017822266
29.4470520019531	29.263313293457	30.2605800628662
32.5523376464844	32.8827819824219	31.9826412200928
29.6574630737305	29.4358901977539	30.1752185821533
26.8197021484375	26.7168579101563	27.581901550293
29.1048717498779	28.971529006958	28.0303382873535
28.2307300567627	28.1146583557129	27.2412719726563
28.3607234954834	27.6681060791016	27.1034965515137
28.1675357818604	27.8227443695068	28.8449077606201
29.1831226348877	28.5471973419189	29.8075485229492
29.2746658325195	29.4305171966553	28.8313179016113
28.458028793335	28.730785369873	29.5004558563232
30.7722682952881	30.8475227355957	28.8562335968018
26.7152862548828	26.6673622131348	27.2825965881348
27.6343650817871	27.5924911499023	26.2165641784668
29.9636135101318	29.7387294769287	28.475528717041
28.4944972991943	28.496898651123	30.0885372161865
29.3276805877686	29.5841808319092	28.1991519927979
26.9121646881104	26.8570938110352	27.6094989776611
28.0062980651855	28.0797901153564	27.3666973114014
29.9647159576416	30.2857074737549	29.0884094238281
30.4771118164063	30.3829860687256	29.4040775299072
27.4121570587158	27.855489730835	26.4010963439941
28.7643947601318	28.7156791687012	27.3832740783691
28.6180686950684	28.7112216949463	27.7239036560059
30.3408527374268	30.1917819976807	29.1740760803223
32.4021759033203	32.0685615539551	31.3529491424561

30.2613658905029	29.9914531707764	29.5173759460449
28.7407894134521	29.0770683288574	26.580753326416
29.1155014038086	29.0768127441406	28.4561100006104
27.1602916717529	27.1948852539063	26.1309471130371
27.7925319671631	27.9830493927002	26.4580764770508
29.2045478820801	29.5854530334473	26.795259475708
27.1975116729736	27.1287860870361	26.0109901428223
30.6956939697266	30.1039962768555	29.0251178741455
27.1108798980713	27.3139762878418	26.0570755004883
27.1308479309082	27.0047454833984	26.6753253936768
29.9430751800537	30.1152038574219	29.5315551757813
29.7016544342041	29.5883502960205	28.4269847869873
32.1940155029297	32.1833190917969	31.1469192504883
29.7747364044189	29.8680305480957	28.4329681396484
29.6522750854492	29.2305469512939	30.3419132232666
29.8847618103027	29.9722690582275	29.1501083374023
27.2761306762695	27.2265567779541	27.8460731506348
29.1043968200684	29.3595104217529	28.5082416534424
28.3852100372314	28.4064502716064	27.4865417480469
30.2008113861084	30.4822196960449	29.7265205383301
26.4636211395264	27.3633594512939	28.6525993347168
26.5475444793701	26.6072425842285	25.5402603149414
26.3889007568359	26.3505344390869	28.9013290405273
27.6482162475586	27.3666973114014	25.6194686889648
28.173360824585	28.0495223999023	29.3940505981445
29.3565330505371	29.424503326416	27.581901550293
29.4001922607422	28.3767910003662	29.4918823242188
30.6065559387207	31.0182361602783	30.1819858551025
28.4449005126953	28.2341613769531	27.5955543518066
31.0274925231934	31.1366291046143	32.6475105285645
24.8179721832275	25.0956707000732	27.0654811859131
32.4358177185059	32.2698860168457	31.3023643493652
28.2338428497314	28.1676788330078	27.701473236084
29.1606044769287	29.2868537902832	30.1529140472412
30.3880252838135	30.181510925293	29.4663391113281
30.6887912750244	30.4885540008545	29.4442901611328
28.3366680145264	28.3794364929199	27.6481475830078
28.1536865234375	28.0796890258789	26.7360858917236
25.7158622741699	26.2252521514893	26.7951354980469
30.0657386779785	29.8186817169189	30.6106967926025
29.5269947052002	29.8332653045654	29.0965003967285
28.8675880432129	28.9127655029297	28.2565135955811
26.5350761413574	26.688591003418	25.9208431243896
31.4329090118408	31.4991912841797	30.7139110565186
28.3232841491699	28.1545085906982	27.3300552368164
30.7513484954834	30.6430625915527	31.6132469177246
27.7224712371826	26.8610038757324	26.1861248016357
29.1534214019775	29.3019065856934	28.6323204040527

30.301929473877	30.1698532104492	29.6498107910156
26.3130416870117	26.1905403137207	26.7499599456787
28.9523258209229	29.6141242980957	30.3311672210693
29.4993724822998	29.7787666320801	28.5606708526611
27.6581268310547	28.1172866821289	26.8358116149902
27.033805847168	27.1924419403076	25.37477684021
25.0395069122314	24.7281665802002	25.713846206665
31.0616893768311	31.1586437225342	30.4118537902832
28.607349395752	28.629753112793	26.6191883087158
26.7988471984863	27.1103820800781	27.8316497802734
29.5809860229492	29.1044216156006	28.8343353271484
27.7601490020752	27.2963161468506	28.2771511077881
24.0680885314941	24.0244522094727	25.210334777832
27.0849685668945	26.6463642120361	29.3203182220459
26.0437259674072	26.318172454834	28.0206756591797
30.520751953125	30.7507095336914	32.5177040100098
27.489990234375	27.6063251495361	29.2407264709473
26.8551940917969	26.9414539337158	31.2730865478516
31.0361022949219	31.198881149292	33.5636558532715
33.059886932373	32.8548774719238	32.3942222595215
32.586986541748	32.373966217041	32.966625213623
27.3370513916016	27.4017791748047	28.4364280700684
24.7427825927734	25.3186893463135	26.7137145996094
31.2300891876221	31.5522003173828	32.6797180175781
30.9125652313232	31.0898628234863	31.6508712768555
29.3107089996338	29.2910976409912	30.1692562103271
27.4908313751221	27.3646945953369	28.470495223999
28.0242729187012	27.9811992645264	28.6769733428955
24.9990787506104	24.4429092407227	26.1010932922363
28.2251777648926	28.3052597045898	29.2702159881592
28.6582641601563	28.6209716796875	27.5914211273193
30.1026210784912	30.0467414855957	31.0299301147461
29.36594581604	29.0869693756104	29.9294528961182
25.3106346130371	25.7749500274658	27.0627059936523
30.9985313415527	31.2215881347656	31.7295551300049
29.8141040802002	30.2558612823486	31.0447254180908
28.7497024536133	29.4817771911621	30.2636051177979
29.3738479614258	29.5185203552246	30.3155975341797
30.8096199035645	30.4648017883301	29.8267345428467
27.9202537536621	27.5712356567383	26.8593463897705
33.2156257629395	33.5154724121094	32.3325805664063
26.214916229248	25.9924373626709	24.4735164642334
27.6509571075439	28.7236423492432	26.7886791229248
33.6841583251953	33.5255432128906	32.219108581543
30.3753414154053	29.8831443786621	30.8696041107178
29.0685844421387	29.2694129943848	28.209135055542
28.7996501922607	28.7001495361328	27.9502143859863
29.9208202362061	29.9797821044922	28.2787475585938

31.1943683624268	30.7798004150391	29.4147796630859
28.1649971008301	27.8291721343994	27.0778579711914
29.5072021484375	29.6756954193115	28.0636310577393
29.1520919799805	29.1510276794434	28.0784683227539
32.2395935058594	32.337718963623	33.4083137512207
28.3219528198242	28.6970043182373	30.6814384460449
30.42822265625	30.4456901550293	31.4356918334961
28.8660259246826	28.4138927459717	30.165548324585
31.1386432647705	31.0466117858887	31.9180507659912
31.4173984527588	31.2373447418213	32.1067123413086
31.7665920257568	31.5046482086182	32.4624137878418
27.3135433197021	27.0545520782471	27.762622833252
28.8244457244873	29.0241928100586	29.521369934082
29.9895610809326	30.2306613922119	31.1961879730225
26.8469696044922	26.4151744842529	27.5239505767822
30.1873054504395	29.9537982940674	30.6565933227539
29.9543533325195	29.7531509399414	31.7507495880127
27.336540222168	26.9805450439453	28.0936069488525
28.7641429901123	28.7400493621826	29.6953125
32.7731513977051	32.817741394043	31.7504711151123
32.0183334350586	31.9092426300049	31.0503158569336
27.8623065948486	28.0452060699463	27.1897106170654
26.7177734375	26.8184852600098	25.2405624389648
31.2789688110352	31.3228664398193	32.2062950134277
25.9703330993652	26.0550079345703	26.6956443786621
25.8001327514648	25.2253437042236	27.0441646575928
29.9747333526611	29.8861865997314	30.6192073822021
30.0389156341553	30.2043151855469	29.0638885498047
29.3918190002441	29.6660079956055	29.0476779937744
27.4704170227051	27.4957180023193	28.2096920013428
27.3027782440186	27.3239727020264	27.9485988616943
28.3810062408447	28.3849620819092	29.2235450744629
26.5440540313721	26.4014377593994	28.3049983978271
29.0699939727783	29.0821285247803	28.2819328308105
29.5232410430908	29.3506011962891	28.2469902038574
28.49587059021	28.6820735931396	29.2099952697754
27.0325469970703	26.7666091918945	25.3508377075195
30.4471702575684	29.9704875946045	32.8780899047852
29.5994606018066	30.193546295166	30.5361213684082
25.9849185943604	26.6204490661621	27.6458148956299
28.8245372772217	28.7572593688965	29.7310447692871
25.670202255249	26.0531635284424	26.9864177703857
28.4109859466553	28.4870014190674	27.7430400848389
31.5496788024902	31.3181858062744	29.3287296295166
30.3493156433105	30.2481899261475	28.1716442108154
28.7787189483643	29.2597274780273	27.8312873840332
30.7377967834473	31.0363636016846	29.5686092376709

25.3524208068848	25.6027488708496	23.8490009307861
29.549108505249	29.4686698913574	31.3683319091797
31.0786457061768	31.0259761810303	32.0938987731934
28.1773109436035	27.3716907501221	27.064453125
26.5922069549561	26.3270263671875	27.4030818939209
25.5139141082764	25.776180267334	26.4582004547119
31.8815040588379	32.1024322509766	31.4363384246826
27.0864887237549	27.081111907959	27.7912273406982
25.1509304046631	25.0520458221436	25.808895111084
28.0179710388184	27.719274520874	27.1133670806885
29.7359580993652	29.8275680541992	28.903169631958
27.4198913574219	27.2182655334473	28.8317718505859
32.132682800293	32.1705093383789	30.9416770935059
28.581937789917	27.7612915039063	29.6303596496582
27.6492443084717	28.5145378112793	29.6427364349365
28.4403133392334	28.5784149169922	27.6868572235107
27.8843688964844	27.3470573425293	29.565580368042
28.6075611114502	28.6074542999268	29.7399044036865
29.0723209381104	29.0712985992432	30.5312023162842
26.2666015625	26.2723979949951	25.5361728668213
28.7707099914551	29.0593814849854	28.2977600097656
29.4090442657471	29.0626029968262	30.096097946167
27.9964733123779	27.9252433776855	27.0707111358643
26.0864276885986	26.2274017333984	26.9689502716064
27.8782997131348	27.7027282714844	28.6915893554688
28.9464817047119	28.8779773712158	28.127555847168
29.3776798248291	29.2793445587158	30.1021194458008
26.670202255249	26.47047996521	25.3172073364258
25.9337539672852	25.8009223937988	24.572998046875
29.3964614868164	29.3134365081787	28.310037612915
30.182222366333	30.3652591705322	31.1659069061279
30.9371929168701	31.1383380889893	29.9482383728027
28.4346790313721	28.5844860076904	29.6353340148926
28.9075889587402	28.6405830383301	27.748872756958
28.760181427002	28.1185741424561	27.7463760375977
28.3912868499756	28.5406360626221	27.5770111083984

Radiation_12h		
LFQ intensity R_12h_3	LFQ intensity R_12h_4	Peptides
26.3468036651611	26.7328567504883	8
26.1708717346191	25.6652755737305	6
26.3817729949951	25.8964977264404	5
27.2422714233398	27.1847076416016	5
26.7774353027344	26.5579776763916	7
29.6798114776611	29.7301216125488	6
25.6730632781982	25.9254913330078	5
27.0885105133057	27.5050640106201	5
28.214656829834	28.3368797302246	5
29.8732624053955	30.2713069915771	9
30.8362789154053	30.8718814849854	13
28.9478759765625	28.9597511291504	14
28.8960247039795	28.4014949798584	6
27.6587409973145	27.6085834503174	8
30.6174564361572	31.881067276001	8
27.5379371643066	27.5160808563232	5
29.0990619659424	28.9956378936768	8
28.3501377105713	28.1330547332764	8
28.3620204925537	28.0928516387939	6
25.8973064422607	25.1106014251709	6
30.549036026001	31.1614685058594	11
27.3960742950439	27.756591796875	4
29.653470993042	29.2344818115234	6
27.1322212219238	26.7962493896484	5
27.3243160247803	27.2723083496094	7
29.92578125	29.852367401123	4
30.2297458648682	30.2855968475342	23
27.908275604248	28.1588478088379	8
27.4361896514893	27.6537609100342	3
29.9182643890381	30.3085613250732	10
29.3118991851807	29.6838493347168	10
29.0506649017334	28.9941539764404	5
26.0851306915283	25.8737773895264	5
27.5331172943115	27.5486316680908	3
24.7761039733887	24.8683471679688	4
29.9596118927002	29.9869861602783	2
27.994047164917	27.495641708374	4
30.6395320892334	30.9526882171631	17
25.9770774841309	25.658073425293	4
26.0509872436523	25.6330070495605	2
30.5295276641846	30.6483879089355	25
27.2206649780273	27.1438465118408	5
26.3515625	25.9785842895508	7
28.9715576171875	29.1590156555176	14
27.7781867980957	27.7273483276367	3

27.7973022460938	27.821346282959	6
27.6877918243408	27.8147621154785	15
24.3485431671143	24.2241287231445	8
27.9891262054443	28.0823822021484	14
28.1190204620361	27.9862003326416	7
28.3096904754639	27.9013862609863	12
27.4732074737549	27.3263740539551	4
28.8589019775391	28.8774814605713	8
26.4466209411621	26.7008113861084	12
27.3937854766846	28.2314167022705	8
27.7561454772949	27.689525604248	12
26.4749870300293	27.1788311004639	3
29.4364280700684	29.6485767364502	11
31.1223812103271	31.0545253753662	17
27.7829437255859	27.3292865753174	6
26.7486801147461	26.7192096710205	9
27.7908554077148	28.1574020385742	9
26.0495758056641	26.491870880127	7
29.4504585266113	29.3899955749512	3
27.223705291748	27.071325302124	1
26.0286731719971	26.3535652160645	13
24.8884620666504	25.4556331634521	4
30.1243553161621	30.9591293334961	5
31.9018898010254	31.8922939300537	33
30.3315944671631	30.2701930999756	18
27.1027946472168	27.6897926330566	9
28.3507289886475	28.294038772583	14
27.0103073120117	27.485237121582	8
26.5540447235107	27.3043460845947	4
28.638650894165	28.8056888580322	8
29.6495532989502	29.8273410797119	3
28.8296566009521	28.2935562133789	8
29.3761692047119	29.228048324585	8
28.7677135467529	28.9900207519531	12
27.6716861724854	28.1596660614014	5
26.5288467407227	26.2395248413086	3
27.5428886413574	28.4589290618896	3
29.704460144043	30.0464820861816	9
27.9840831756592	27.2408180236816	8
27.582763671875	27.6724281311035	3
26.9526042938232	27.4195690155029	8
28.7658824920654	29.0114803314209	21
29.8498058319092	29.6136493682861	8
27.0679454803467	26.0443515777588	7
27.9544906616211	27.8054428100586	5
27.6418933868408	27.5850582122803	14
28.9735832214355	29.2165775299072	29
31.4009552001953	31.4089221954346	15

29.3934574127197	29.3124618530273	8
26.9572620391846	26.8542423248291	2
28.1096363067627	28.1632690429688	3
26.0549659729004	26.0793437957764	6
27.0212574005127	26.7978591918945	7
27.5093002319336	27.0983848571777	7
26.2669944763184	26.0408039093018	3
28.8498954772949	28.9642200469971	7
25.5536785125732	25.7006797790527	6
26.0199642181396	26.5917778015137	10
28.8640193939209	29.3350276947021	13
28.5903148651123	28.2896480560303	14
31.1294002532959	31.0455703735352	16
28.8930759429932	28.4918251037598	6
30.1204051971436	30.2076930999756	7
29.1641330718994	29.0747737884521	15
27.6852531433105	27.9865798950195	13
28.3133277893066	28.487232208252	11
27.6341571807861	27.5462055206299	15
29.6855201721191	29.732177734375	13
28.3895645141602	28.9951248168945	6
25.8842754364014	25.7414207458496	3
28.8229579925537	28.8000831604004	18
26.3688297271729	25.5140647888184	6
28.6192588806152	29.5842704772949	7
27.4359512329102	27.814884185791	11
30.052583694458	29.7031078338623	7
29.7081832885742	30.278636932373	7
27.4850063323975	27.0751056671143	6
32.5433540344238	32.5101318359375	19
27.1872577667236	26.0645980834961	6
31.2944431304932	31.2586727142334	22
27.6728324890137	27.650203704834	6
30.1169395446777	30.2489814758301	12
29.284252166748	29.5676136016846	14
29.5297698974609	29.8117027282715	11
27.4558353424072	28.0677909851074	9
26.7196025848389	26.6463642120361	7
26.638650894165	26.3379516601563	4
30.6434078216553	30.612455368042	22
28.7815685272217	29.0653018951416	25
28.3435039520264	28.2685661315918	4
25.2793750762939	25.4904479980469	7
30.7353763580322	30.2676296234131	7
27.7905445098877	27.4649753570557	10
31.5201892852783	31.6919975280762	18
25.9462356567383	25.9605579376221	5
28.3343677520752	28.4800815582275	13

29.5395278930664	29.7415466308594	6
27.0012054443359	27.0010986328125	3
30.4276237487793	30.8950424194336	1
28.7840671539307	28.5757484436035	2
27.457010269165	26.7110900878906	2
25.1262855529785	25.3836688995361	5
27.6908569335938	25.0988864898682	1
30.3545799255371	30.4202117919922	12
26.5027751922607	27.4911365509033	6
27.9991111755371	27.7289714813232	3
28.592134475708	28.8045825958252	9
28.210901260376	28.5781269073486	9
25.2614822387695	25.169361114502	2
29.2300891876221	29.0895214080811	10
28.258358001709	28.0672264099121	16
32.3592224121094	32.544921875	19
28.4921684265137	29.3167839050293	5
30.7990016937256	30.9002361297607	9
33.8404312133789	33.8161201477051	10
32.1629981994629	32.3309516906738	25
33.2574348449707	33.2277679443359	20
27.9992713928223	27.9853858947754	8
25.354305267334	25.7666091918945	3
32.5507125854492	32.4122581481934	62
31.7423000335693	31.6123237609863	14
30.1343040466309	30.2220478057861	15
28.3780307769775	27.975471496582	7
28.5979709625244	28.8590202331543	7
26.4518013000488	26.5462341308594	4
29.1283912658691	28.8520412445068	5
27.9192314147949	27.5229034423828	6
30.9225234985352	31.264331817627	5
30.0028972625732	30.0870189666748	10
26.1690940856934	26.2349662780762	5
31.8581981658936	31.9228420257568	7
30.4560127258301	30.9402770996094	9
29.5747566223145	30.1088638305664	7
30.027889251709	30.4634380340576	13
29.6050186157227	30.0766086578369	15
27.1961040496826	26.977165222168	10
32.0878715515137	32.0484657287598	32
24.5914936065674	24.1175136566162	5
27.2757759094238	27.0730628967285	3
32.4690551757813	32.2584190368652	41
30.6724452972412	30.7638263702393	4
28.4915199279785	28.2882843017578	25
27.8376770019531	27.7955684661865	6
28.383810043335	28.4129638671875	4

29.6064491271973	29.3077373504639	6
27.1176338195801	27.1058959960938	9
27.5312576293945	27.5237274169922	9
27.594913482666	27.5346755981445	7
33.2860946655273	33.3688011169434	13
30.7582759857178	30.7051200866699	13
31.3923835754395	31.6578712463379	8
29.637788772583	30.4456901550293	8
31.5618362426758	31.9206428527832	10
32.1454010009766	32.0582389831543	33
32.207633972168	32.4892234802246	10
27.8983345031738	28.0133972167969	2
29.6624965667725	29.8276443481445	11
31.1522483825684	31.3355922698975	15
27.5838394165039	27.3525390625	6
30.7491111755371	30.817798614502	5
31.8398494720459	31.7443065643311	10
28.3598861694336	28.2674503326416	11
29.7340202331543	29.8989391326904	5
31.4053764343262	31.6283626556396	33
30.855489730835	30.9188327789307	22
27.3865661621094	27.1575946807861	7
25.262020111084	25.2352771759033	5
32.2611389160156	32.3575973510742	9
26.826265335083	26.9110221862793	8
26.2558040618896	27.1744575500488	2
30.9529647827148	31.0584011077881	18
29.2104816436768	28.9066429138184	4
28.4198112487793	29.0824069976807	9
28.3840980529785	28.2878437042236	21
28.3972988128662	28.1653327941895	6
29.2471485137939	29.1533489227295	7
27.3984394073486	27.9686756134033	4
28.3119869232178	28.2198810577393	5
28.2751541137695	28.4530906677246	6
29.2970600128174	29.3847160339355	10
25.5148468017578	25.5132808685303	5
32.6888542175293	32.8950080871582	16
30.7317409515381	30.3060646057129	3
27.7325973510742	27.6876583099365	2
30.0173072814941	30.0091857910156	5
26.0536403656006	26.5278625488281	6
26.9616832733154	26.5260124206543	4
29.8977851867676	29.3844890594482	10
28.3640689849854	28.4930858612061	7
27.946928024292	27.8734378814697	5
29.3126354217529	29.3774318695068	13

24.5014247894287	24.5841121673584	4
31.4873085021973	31.3835525512695	23
32.2006378173828	32.1572265625	21
26.8332805633545	26.993236541748	6
27.4680881500244	27.6706066131592	4
26.2166385650635	26.3774280548096	6
31.3306331634521	31.2639961242676	22
27.5601959228516	28.0319671630859	11
25.7598438262939	25.9080238342285	5
27.5063514709473	27.1390705108643	4
29.1517524719238	28.81298828125	6
28.8042736053467	28.968786239624	4
31.0693283081055	31.1308116912842	8
28.8333110809326	29.2939071655273	5
28.7582130432129	29.6274757385254	3
27.6809692382813	27.7423973083496	8
29.5554504394531	29.7774353027344	10
29.6467761993408	29.924222946167	15
30.5054054260254	30.6496734619141	17
25.547513961792	25.466625213623	4
28.4168338775635	28.1659545898438	9
30.4195098876953	30.2096691131592	2
27.2042446136475	27.1246509552002	5
27.8018093109131	27.3157901763916	6
28.2369918823242	28.3059558868408	17
27.5394172668457	28.0629119873047	5
30.0241928100586	30.0523242950439	15
25.0546131134033	25.3136138916016	5
25.1289615631104	24.5292186737061	4
27.931568145752	27.8016872406006	3
30.7158756256104	31.4415912628174	15
29.9371929168701	29.9578151702881	9
29.2127571105957	29.3187236785889	5
27.6237316131592	27.7470149993896	6
27.7227306365967	27.9779281616211	8
27.0869941711426	27.70530128479	10

Razor + unique peptides	Unique peptides	Sequence coverage [%]
8	8	58
6	6	10.8
5	5	21
5	5	24.3
7	7	14.7
6	6	39.2
5	5	29.3
5	5	20.3
5	5	22.3
9	9	23.6
13	13	30.7
14	14	37
6	6	11.9
8	8	16.2
8	8	27.5
5	5	15.9
8	8	27.9
8	8	24.5
6	6	18
6	6	33.3
11	11	37.7
4	4	21.1
6	6	43.6
5	5	9.3
7	7	16.6
4	4	27.6
23	23	44.8
8	8	31.1
3	3	24.9
10	10	28.2
10	10	38.5
5	5	17.6
5	5	14.8
3	3	25.4
4	4	32.2
2	2	27.4
4	4	28.1
17	17	39.5
4	4	7.9
2	2	6.6
25	25	41.6
5	5	31.1
7	7	20.4
14	14	27.1
3	3	6.3

6	6	20.4
15	15	29.4
8	8	42.2
14	14	25.7
7	7	37.2
12	12	33.5
4	4	24.3
8	8	22.3
12	12	29.8
8	8	25.1
12	12	18.9
3	3	7.4
11	11	20.8
17	17	43.2
6	6	14.6
9	9	36.3
9	9	41.8
7	7	31.7
3	3	12.6
1	1	12
13	13	28.6
4	4	13.1
5	5	22.9
33	33	58.8
18	18	35.5
9	9	22.8
14	14	31.2
8	8	18.5
4	4	23.6
8	8	29.1
3	3	18.4
8	8	22.3
8	8	18.9
12	12	58.1
5	5	10.3
3	3	16.6
3	3	23.3
9	9	35.3
8	8	37.6
3	3	29.7
8	8	26.3
21	21	83.3
8	8	23.8
7	7	28.5
5	5	45.1
14	14	41.1
29	29	37.1
15	15	59.7

8	8	25.5
2	2	12.7
3	3	32
6	6	39.6
7	7	22.7
7	7	15.1
3	3	11.9
7	7	29.8
6	6	17
10	10	32.5
13	13	41.5
14	14	43.9
16	16	45.7
6	6	38.2
7	7	40.9
15	15	17.3
13	13	46.6
11	11	21.8
15	15	19.6
12	12	49
6	6	34.2
3	3	20.9
18	18	33.2
6	6	65.3
7	7	18
11	11	60.4
7	7	30.8
7	7	27.5
6	6	22.5
19	19	46.6
6	6	29.5
22	22	69.2
6	6	28.6
12	12	53.8
14	14	43.9
11	11	41.7
9	9	18
7	7	32.8
4	4	27.1
22	22	33.9
25	25	59.1
4	4	36.9
7	7	24.2
7	7	54.9
10	10	17
18	18	51.1
5	5	53
13	13	54

6	6	34.2
3	3	16
1	1	16.8
2	2	11.8
2	2	2.2
5	5	35.6
1	1	12.5
12	12	48.2
6	6	23.1
3	3	11.5
9	9	40.7
9	9	36.1
2	2	7.3
10	10	37.3
16	16	45.3
19	19	62.3
5	5	29.2
9	9	28.4
10	10	30.1
25	25	41.8
20	20	43
8	8	30.4
3	3	19.8
62	62	65.4
14	14	56.7
15	15	31.2
7	7	24.2
7	7	38.2
4	4	24.9
5	5	20.1
6	6	29.6
5	5	47.2
10	10	23.6
5	5	57.3
7	7	55.3
9	9	52.4
7	7	49.2
13	13	29.6
15	15	46.2
10	10	26.1
32	32	58.6
5	5	19.7
3	3	26.1
41	40	90.5
4	4	31.5
25	25	70.8
6	6	21.7
4	4	44.5

6	6	30.5
9	9	18
9	9	34.2
7	7	50.3
13	13	60.9
13	13	54.5
8	8	38.3
8	8	45
10	10	39.8
33	33	51.9
10	10	60.9
2	2	13.7
11	11	25.2
15	15	65.7
6	6	28.6
5	5	20.4
10	10	43.9
11	11	27.6
5	5	37
33	33	55.8
22	22	65.1
7	7	14.4
5	5	16.6
9	9	66.4
8	8	36.2
2	2	14.1
18	18	45
4	4	30.6
9	9	33.1
21	21	26.5
6	6	21.9
7	7	42
4	4	19.1
5	5	26.9
6	6	34.9
10	10	15
5	5	17.8
16	16	59.9
3	3	25.3
2	2	32.5
5	5	24.2
6	6	25.7
4	4	31.7
10	10	41.4
7	7	48.8
5	5	17
13	13	24.3

4	4	63.4
23	23	23.6
21	21	42.3
6	6	45
4	4	24.6
6	6	20.6
22	22	41
11	11	18.4
5	5	11.6
4	4	31.8
6	6	54.3
4	4	42
8	8	26.2
5	5	49.6
3	3	32.5
8	8	18.8
10	10	44
15	15	33.5
17	17	45.3
4	4	19.5
9	9	21.7
2	2	21.4
5	5	18.8
6	6	9.8
16	16	22.8
5	5	31.9
15	15	17.6
5	5	36.7
4	4	28.4
3	3	10.7
15	15	39.7
9	9	85.1
5	5	23
6	6	67.6
8	8	26.3
10	10	25.9

Unique + razor sequence coverage [%]	Unique sequence coverage [%]
58	58
10.8	10.8
21	21
24.3	24.3
14.7	14.7
39.2	39.2
29.3	29.3
20.3	20.3
22.3	22.3
23.6	23.6
30.7	30.7
37	37
11.9	11.9
16.2	16.2
27.5	27.5
15.9	15.9
27.9	27.9
24.5	24.5
18	18
33.3	33.3
37.7	37.7
21.1	21.1
43.6	43.6
9.3	9.3
16.6	16.6
27.6	27.6
44.8	44.8
31.1	31.1
24.9	24.9
28.2	28.2
38.5	38.5
17.6	17.6
14.8	14.8
25.4	25.4
32.2	32.2
27.4	27.4
28.1	28.1
39.5	39.5
7.9	7.9
6.6	6.6
41.6	41.6
31.1	31.1
20.4	20.4
27.1	27.1
6.3	6.3

20.4	20.4
29.4	29.4
42.2	42.2
25.7	25.7
37.2	37.2
33.5	33.5
24.3	24.3
22.3	22.3
29.8	29.8
25.1	25.1
18.9	18.9
7.4	7.4
20.8	20.8
43.2	43.2
14.6	14.6
36.3	36.3
41.8	41.8
31.7	31.7
12.6	12.6
12	12
28.6	28.6
13.1	13.1
22.9	22.9
58.8	58.8
35.5	35.5
22.8	22.8
31.2	31.2
18.5	18.5
23.6	23.6
29.1	29.1
18.4	18.4
22.3	22.3
18.9	18.9
58.1	58.1
10.3	10.3
16.6	16.6
23.3	23.3
35.3	35.3
37.6	37.6
29.7	29.7
26.3	26.3
83.3	83.3
23.8	23.8
28.5	28.5
45.1	45.1
41.1	41.1
37.1	37.1
59.7	59.7

25.5	25.5
12.7	12.7
32	32
39.6	39.6
22.7	22.7
15.1	15.1
11.9	11.9
29.8	29.8
17	17
32.5	32.5
41.5	41.5
43.9	43.9
45.7	45.7
38.2	38.2
40.9	40.9
17.3	17.3
46.6	46.6
21.8	21.8
19.6	19.6
49	49
34.2	34.2
20.9	20.9
33.2	33.2
65.3	65.3
18	18
60.4	60.4
30.8	30.8
27.5	27.5
22.5	22.5
46.6	46.6
29.5	29.5
69.2	69.2
28.6	28.6
53.8	53.8
43.9	43.9
41.7	41.7
18	18
32.8	32.8
27.1	27.1
33.9	33.9
59.1	59.1
36.9	36.9
24.2	24.2
54.9	54.9
17	17
51.1	51.1
53	53
54	54

34.2	34.2
16	16
16.8	16.8
11.8	11.8
2.2	2.2
35.6	35.6
12.5	12.5
48.2	48.2
23.1	23.1
11.5	11.5
40.7	40.7
36.1	36.1
7.3	7.3
37.3	37.3
45.3	45.3
62.3	62.3
29.2	29.2
28.4	28.4
30.1	30.1
41.8	41.8
43	43
30.4	30.4
19.8	19.8
65.4	65.4
56.7	56.7
31.2	31.2
24.2	24.2
38.2	38.2
24.9	24.9
20.1	20.1
29.6	29.6
47.2	47.2
23.6	23.6
57.3	57.3
55.3	55.3
52.4	52.4
49.2	49.2
29.6	29.6
46.2	46.2
26.1	26.1
58.6	58.6
19.7	19.7
26.1	26.1
90.5	90.5
31.5	31.5
70.8	70.8
21.7	21.7
44.5	44.5

30.5	30.5
18	18
34.2	34.2
50.3	50.3
60.9	60.9
54.5	54.5
38.3	38.3
45	45
39.8	39.8
51.9	51.9
60.9	60.9
13.7	13.7
25.2	25.2
65.7	65.7
28.6	28.6
20.4	20.4
43.9	43.9
27.6	27.6
37	37
55.8	55.8
65.1	65.1
14.4	14.4
16.6	16.6
66.4	66.4
36.2	36.2
14.1	14.1
45	45
30.6	30.6
33.1	33.1
26.5	26.5
21.9	21.9
42	42
19.1	19.1
26.9	26.9
34.9	34.9
15	15
17.8	17.8
59.9	59.9
25.3	25.3
32.5	32.5
24.2	24.2
25.7	25.7
31.7	31.7
41.4	41.4
48.8	48.8
17	17
24.3	24.3

63.4	63.4
23.6	23.6
42.3	42.3
45	45
24.6	24.6
20.6	20.6
41	41
18.4	18.4
11.6	11.6
31.8	31.8
54.3	54.3
42	42
26.2	26.2
49.6	49.6
32.5	32.5
18.8	18.8
44	44
33.5	33.5
45.3	45.3
19.5	19.5
21.7	21.7
21.4	21.4
18.8	18.8
9.8	9.8
21.6	21.6
31.9	31.9
17.6	17.6
36.7	36.7
28.4	28.4
10.7	10.7
39.7	39.7
85.1	85.1
23	23
67.6	67.6
26.3	26.3
25.9	25.9

Mol. weight [kDa]	Q-value	Score	MS/MS count	Student's T-test p-value C_R
23.402		323.31	131	0.00693247246201904
100.77		167.73	76	0.0118730588892496
36.93		69.322	100	0.011910416690981
36.294		323.31	84	0.000162601976634107
75.532		41.905	81	0.00721095190057114
22.134		72.103	118	0.0143080117187453
33.259		53.424	25	0.00651000171527867
32.962		74.282	94	0.00560206020318896
40.884		183.76	111	0.000544052157523642
57.035		125.4	247	0.00915096808885703
61.722		323.31	232	0.000311954012475399
41.986		221.25	174	0.00175381470594025
77.447		274.21	44	0.012926950467844
69.439		272.95	122	0.000503927365355341
35.079		227.83	83	0.000503751580704537
50.051		323.31	77	0.00149064356896017
60.514		323.31	131	2.26368332759986E-05
69.702		208.43	123	0.0010821803608361
42.143		44.28	77	0.00407241041600536
38.779		113.65	61	0.00198410458883949
31.721		269.85	188	0.000973382385017486
29.031		323.31	42	0.0134475005089919
17.83		233.81	129	0.00838939548599754
62.604		203.58	117	0.000155973994795749
66.085		166.16	138	0.000316135208955853
19.371		148.07	132	0.000611349101554366
73.453		323.31	483	0.000160312485301199
37.04		75.758	120	0.00579526249917255
18.113		27.543	62	0.0127955278805661
42.481		173.45	188	0.00389893613213038
41.904		117.19	203	0.00419962586283957
53.019		323.31	109	0.000302186963957282
36.174		323.31	62	0.00304999446642782
14.8		69.177	104	0.0471269878947696
32.533		323.31	67	0.0779873747780139
11.442		323.31	106	0.0114593585035513
28.294		169.51	69	0.00216425378698912
58.603		323.31	398	0.00110528149978879
48.171		60.123	70	0.00145265838808026
63.013		41.618	73	0.00725836633312469
84.048		323.31	856	0.0014958378040004
19.535		27.326	49	0.00014116885254382
46.934		223.14	103	0.00016573798300011
84.042		323.31	259	0.00196509683765523
74.588		88.831	146	0.00183704764765416

52.793		164.35	126	2.86534572419623E-06
77.371		319.61	265	0.00195888536370431
20.038		53.386	82	0.000189292157612009
79.507		219.96	297	0.000283806791858228
22.647		323.31	159	0.000368322118943335
56.109		180.54	129	0.0112937373195304
31.653		43.682	80	0.0135939452026818
56.898		97.883	103	0.0414009850106069
48.85		110.95	177	3.54996837987513E-05
48.294		310.71	123	0.0141195599113047
100.31		57.56	156	0.00807940391873645
54.583	0.0006006	5.9603	39	0.0301866327141472
86.382		323.31	443	0.00749261008338471
40.182		323.31	473	0.000364728077563814
51.657		73.524	103	0.000493086125997113
28.694		323.31	189	0.00275070844083716
37.993		323.31	193	0.00198534425375006
35.25		41.862	78	0.00124241512281623
23.949		323.31	111	0.000168184423510172
25.124		323.31	52	0.00190511230438318
76.275		314.66	231	0.000141153311590829
48.662	0.0006993	15.409	41	0.00365715809930317
30.662		35.317	118	0.0180269161969229
64.842		323.31	776	0.00134674401782172
84.741		323.31	346	0.00123954311564907
60.533		252.73	174	0.0178371465674757
71.139		163.21	169	0.00969823824687956
56.812		73.427	78	0.00317484380276421
23.28		144.48	95	0.0235480514862549
39.044		137.55	96	0.00306846644808635
16.317		184.68	54	0.0152031971504173
53.293		282.7	105	0.0196457249172353
69.279		161.12	123	0.00232222650315815
30.466		323.31	287	1.49181623750337E-05
57.452	0.000578	4.25	43	0.0357337274663115
27.598		26.283	70	0.000242986477851742
18.302		127.34	79	0.0055983353290504
35.49		323.31	228	0.000317861216348877
33.447		138.94	133	0.00566000892947266
20.25		112.81	55	0.00174975633260593
45.542		158.1	151	0.00584395960658841
37.281		323.31	394	0.00118438001791776
34.94		89.064	210	0.00525845575803145
48.499	0.0007474	21.128	44	0.0276477696184492
26.891	0.0007353	19.856	84	0.00379810807022832
50.683		323.31	383	0.000136271278532304
102.4		323.31	449	0.000301743338036718
32.421		323.31	308	0.00211135721471171

45.195		319.63	292	0.00490254679978294
11.275		99.257	46	0.00428857897397313
18.663		127.75	82	0.00948391315653797
21.984		118.03	87	3.33259962629946E-05
47.984		111.87	85	0.00295541545127829
77.931		323.31	138	0.000644053378752377
46.524		54.793	39	0.000219093548581177
33.085		323.31	367	0.00238824134881716
53.183		116.43	41	0.000833103456504243
50.821		323.31	170	0.0324976484833224
63.53		323.31	300	0.032402996388886
44.673		96.644	205	0.000433203997913763
47.265		323.31	553	6.96146003922371E-06
9.2178		186.76	77	0.00114071628524185
29.738		138.25	136	0.0084699589153713
111.16		323.31	317	0.000399444700992019
39.408		323.31	203	0.00974082746282535
63.666		272.44	177	0.00113134915367446
111.12		323.31	290	0.000758366984248571
22.759		271.46	312	0.002098794433616
33.971		127.35	145	0.00571741788638814
15.06	0.0006382	8.9263	61	0.00140646958111718
80.109		234.78	197	0.000109553443010263
20.665		323.31	203	0.0145745670988457
66.059		255.51	115	0.0253958621089659
34.331		323.31	87	0.000101163748346128
31.687		180.57	76	0.0715822475303604
21.043		196.33	243	0.031222389917994
49.622		39.477	60	0.00468269559172839
42.8		323.31	1109	9.44925067073741E-06
40.629		54.683	89	0.075255406496344
36.1		323.31	576	3.32055526930172E-05
32.947		56.641	124	0.0241118977948597
27.259		323.31	394	0.000859628308194355
46.258		323.31	305	0.00524009352862891
40.187		323.31	348	0.00338221944987668
63.392		74.379	143	0.0219365459026628
34.632		101.46	132	0.000277834894037177
14.371		323.31	105	0.0323296660654023
73.157		323.31	380	0.000693113514354407
52.642		323.31	395	0.00699541074832628
17.528		26.956	76	0.000614416427664317
51.281		95.353	94	0.00545078540656621
19.057		323.31	189	0.00412971983403158
75.927		156.88	129	0.00751003636506367
56.626		323.31	607	0.0001108823914892
14.533		158.73	84	0.010731325258869
25.45		260.18	187	0.00193351127479819

25.06		51.743	127	0.00402329404521621
22.631		23.007	56	0.00182208579349009
10.023		323.31	140	0.0216157371601307
39.037		26.132	97	0.00141053804690918
105.89	0.009264	1.4966	14	0.0257885673420127
23.702		127.57	98	4.85655456154565E-05
25.744		71.168	12	0.285887663506871
40.339		289.77	358	0.000797611863980067
36.536		115.71	58	0.00504296464798087
50.748		38.901	28	0.0280757601143718
40.173		323.31	200	0.0295971943675769
31.194		66.52	104	0.0136401851098479
42.062		274.14	72	0.00117058682581733
32.21		323.31	194	0.000160753595398955
42.662		323.31	174	6.07947157705921E-05
38.144		323.31	440	3.66570599585722E-05
20.588		115.7	82	0.00514729931971297
42.943		323.31	231	1.24546719264215E-05
37.54		312.55	378	1.44198470174862E-05
60.512		323.31	806	0.00239962944574456
44.452		323.31	976	0.00880359325586946
45.504		157.01	140	0.0141298875548519
33.597		231.24	88	0.1791726317542
123.73		323.31	2405	0.000673908327937978
34.038		323.31	403	0.000535387742122569
66.603		323.31	267	5.08471572233748E-06
25.894		50.803	54	0.00992865680490626
25.269		195.44	122	0.000806610845980685
26.567		200.97	55	0.00859297587911747
28.297		112.27	95	0.00269385568334675
36.317		70.358	137	0.00183514702797766
23.617		323.31	257	0.000614657804342788
73.942		323.31	244	0.00228361941485116
21.147		323.31	81	0.0388910209639552
20.967		323.31	341	0.00338745877967974
24.36		323.31	394	0.0265743900957096
13.957		323.31	166	0.061067672305351
64.023		323.31	270	0.00478310080657628
58.433		323.31	471	0.00881424476206511
60.164		68.385	139	0.0142054576554409
65.62		323.31	1115	0.000691157958383581
44.737		35.968	46	0.00088249234174162
17.661		136.5	156	0.0271047344994139
30.606		323.31	1403	0.000153006839595859
10.003		323.31	265	0.0153059265241951
30.929		323.31	259	0.00240138837792062
30.118		74.432	93	0.000503937135210157
12.868		151.75	100	1.89892191413511E-05

38.032		134.82	145	0.000554166051855888
64.678		323.31	123	0.000779524823996142
35.018		323.31	170	0.000559534509269409
22.02		250.34	168	0.00401777235252261
26.57		323.31	450	4.90002610609509E-05
21.156		323.31	317	4.04921525139809E-05
20.35		211.06	383	0.000759423390343974
27.307		323.31	213	0.00568982247406644
22.437		323.31	519	0.00435316448536452
76.797		323.31	1223	0.000342521625612535
17.941		279.58	359	0.00332010999972106
35.742		323.31	54	0.00585290325074447
78.366		128.36	238	0.00285833046732633
32.27		323.31	384	0.00042179679846506
50.17		232.98	121	0.00419080362152667
24.724		55.348	138	0.00120240721896407
32.722		323.31	604	0.000138908216174169
47.836		323.31	171	0.0192761269656644
24.488		32.011	124	0.0232281713270911
76.754		323.31	1076	0.000328810904499679
54.834		323.31	721	0.000404245261541949
55.904		27.662	101	0.00179704843326485
43.296	0.0007189	17.456	36	0.000223015689706558
12.289		223.7	415	0.0324486002147502
32.891		248.32	157	0.00112553425723665
26.089		193.91	32	0.116440559984694
53.228		323.31	357	0.00381260737968209
34.323		323.31	165	0.000449157407311701
37.176		128.46	140	0.0383140244935508
121.55		323.31	336	0.000117582915148392
37.421		323.31	180	0.00405944754023906
19.073		292.34	169	8.14755366123954E-06
43.477		26.285	24	0.00847409609340522
29.844		323.31	152	2.67069897473392E-05
35.523		323.31	190	0.000166131559463276
75.236		323.31	196	0.000706876277494873
51.18		97.074	89	0.0026644170337997
40.858		323.31	730	8.40960617909432E-05
17.393		323.31	149	0.0374525149391925
12.91		65.563	53	0.0048965449928304
21.052	0.0006803	12.907	58	0.000296927993651849
40.691		89.746	69	0.068155785451189
25.164		191.78	66	0.021346952343311
29.318		323.31	164	0.000653557742774454
18.166		109.5	124	4.49381230282628E-05
36.547		81.981	69	0.00222650704386869
75.492		62.869	182	0.000725275624626909

9.7595	0.0006489	9.7976	23	0.00808502443895643
152.91		323.31	415	3.84888561885192E-06
66.193		323.31	1026	4.60480505356417E-05
22.863		105.64	70	0.0325825281987619
42.811		59.693	74	0.00234985208569941
42.912		41.86	62	0.00413735947825732
84.409		323.31	1144	0.00217168797086712
81.944		91.996	81	0.00974311740212691
58.59		38.5	43	0.000204449702072671
16.578		179.75	79	0.0144819307739358
19.03		323.31	234	0.00209003439707158
16.619		124.5	93	0.000219012338928531
39.579		323.31	356	0.000104856196523962
12.916		323.31	129	0.0452397313319037
16.181		284.07	83	0.0306815875870364
52.43		58.944	153	0.000118211503339791
27.979		249.68	185	0.00105836371998528
52.221		323.31	392	0.00022580775793924
49.305		323.31	294	4.79329781339612E-06
21.12		323.31	48	8.62273037356257E-05
70.502		139.21	148	0.00464696120750803
16.866		27.424	93	0.00307828595910745
37.44		137.53	92	0.000183187971410139
84.141		41.68	107	0.00793115200951035
90.135		323.31	267	0.0191136621668188
29.523		193.43	146	0.00568261283682853
89.824		323.31	233	4.90612835532518E-05
24.622		156.11	83	0.000208132285671732
35.843		136.19	62	0.00449943634881788
28.559	0.0005695	3.8771	81	0.00764330788662493
44.177		180.95	149	0.0341347681397457
15.174		323.31	225	0.000228722565658685
35.746		323.31	138	0.00283250327915501
17.991		323.31	156	0.000257887959053977
51.341		46.81	113	0.0317454101761546
57.628		211.88	127	0.00641978987461278

Student's T-test q-value C_R	Student's T-test Difference C_R
0.0166086956521739	-0.814098358154297
0.0358766519823789	0.726324081420898
0.0445573770491803	0.691784540812172
0.0293461538461538	0.720582326253258
0.0473204633204633	-0.667245229085285
0.0148342857142857	0.855277379353844
0.0348288288288288	0.723777770996094
0.0492623574144487	-0.658392588297527
	-1.32409731547038
0.0167912087912088	-0.82309850056966
	-1.56493822733561
0.0314905660377358	0.725455602010094
0.0136196319018405	-0.897736231486
0.00930434782608696	-0.952861150105797
	-4.14017804463704
	1.4958864847819
0.00965413533834586	-0.945173899332683
0.0178810810810811	0.786729176839192
0.00217021276595745	-1.1843433380127
	2.11576143900554
	-3.27772013346354
0.0395274261603376	-0.709629694620769
0.00158823529411765	1.33789126078288
	-1.64441299438477
0.0016875	-1.2582753499349
	1.60724703470866
0.00138461538461538	-1.1779047648112
0.0503940520446097	-0.648469924926758
0.0135365853658537	-0.890053431193031
0.0181063829787234	-0.800383885701496
0.0463686274509804	0.666288375854492
0.00511711711711712	-1.05491511027018
	1.9311154683431
0.0137888198757764	0.957195281982422
0.0372640692640693	0.775051116943359
	-1.47083218892415
	1.34965705871582
	1.4664363861084
0.0144561403508772	0.826822916666668
0.0478007662835249	-0.664533615112305
0.00136708860759494	1.21514256795247
0.00885496183206107	0.976991017659504
	1.63564173380534
0.00507142857142857	1.08455912272136
0.0107428571428571	-0.96753247578939

0.0015	1.17864990234375
0.00498245614035088	1.07958920796712
0.0018	1.2884578704834
0.00229213483146067	1.13374837239583
0.00210309278350515	1.12069892883301
0.0182032085561497	0.819552103678383
0.0313427230046948	0.753531773885094
0.0462450592885375	0.707002639770508
	2.45316060384114
0.00892307692307692	1.07010777791341
0.0394723404255319	0.702824274698891
0.0466135458167331	0.700162887573242
0.0490757575757576	-0.66098912556966
0.00226666666666667	1.13597997029622
	1.47358512878418
0.00920634920634921	1.030455271403
0.0128205128205128	0.887328465779625
0.00152112676056338	-1.25102742513021
0.00206060606060606	-1.10534032185872
	1.37171999613444
	2.85641860961914
0.00950819672131148	1.05115254720052
0.00944117647058824	-1.04065577189128
0.0166994535519126	0.793301900227863
0.0396949152542373	-0.682738622029621
0.0310521327014218	-0.760567982991539
0.0105915492957746	1.00076611836751
0.00958208955223881	0.996333440144856
0.00935483870967742	1.11807123819987
0.025910447761194	-0.757280985514324
0.00913385826771653	-1.07896931966146
0.0447258064516129	0.69559605916341
0.0207715736040609	-0.76844596862793
	1.97800191243489
0.0149195402298851	-0.885602315266929
0.00171428571428571	1.26969973246257
	1.71175829569498
	-1.49383735656738
	1.62512715657552
0.0142060606060606	-0.846543629964195
0.0179157894736842	0.799692153930664
0.00144	1.23710823059082
0.0257821782178218	0.764395395914715
0.0090625	1.10673268636068
0.00983050847457627	1.06982739766439
0.00878787878787879	0.967599232991539
0.002	1.10702323913574
0.0177849462365591	0.792999267578129

0.0488905660377359	0.655759175618488
	1.83460426330566
0.033041095890411	0.73818906148275
0.004	1.0445556640625
0.00242857142857143	1.1948070526123
	2.26200866699219
0.00407407407407407	1.06564585367838
	1.36426734924316
	1.45325915018717
0.0464285714285714	0.701815287272133
0.044752	0.705462137858074
0.00174193548387097	1.28572781880697
0.00224175824175824	1.09921836853027
0.00142105263157895	1.22717730204264
0.0135286624203822	-0.910801569620769
0.0129032258064516	0.869028727213539
0.0379655172413793	-0.71410306294759
0.0153898305084746	0.80546760559082
0.0113055555555556	0.931010564168297
0.0446639004149378	0.674189885457356
	-1.9435494740804
0.0131578947368421	0.911521911621094
	-2.32235336303711
	1.45856157938639
0.0108201438848921	-1.04473241170247
	1.81314531962077
0.0287804878048781	-0.826450983683269
0.0445182186234818	0.708886464436848
0.00937226277372263	0.997523625691731
	-1.49560610453288
0.00208163265306122	-1.37191772460938
0.00201980198019802	1.08555475870768
0.0378025751072961	0.731374740600586
0.00900869565217391	-1.05392837524414
0.0286407766990291	0.754661560058594
0.00291588785046729	1.12322044372559
0.0471384615384615	0.68395169576009
	1.31221453348795
0.0412773109243698	-0.726486841837563
0.037589519650655	-0.691192626953125
0.0447407407407407	0.684886932373047
0.0446991869918699	0.661210378011067
0.00958677685950413	1.06248156229655
0.0130718954248366	0.924351374308269
0.0317009345794393	0.742123921712238
0.012	-0.901273091634113
0.00204	1.2034231821696
0.0168839779005525	0.799996058146156

0.0429	0.683801651000977
0.0448816326530612	-0.66870625813802
0.00928	-1.10943222045898
0.0105174825174825	0.946315765380859
0.0125066666666667	0.996203740437828
	1.84109624226888
0.0235376884422111	-1.02115821838379
0.0472062256809339	0.652235666910805
	1.74220530192057
0.0416234309623431	-0.721064885457356
0.0319814814814815	0.770359039306641
0.028921568627451	-0.772176106770836
0.00974789915966386	-1.04452514648438
	-2.45327758789063
	-1.96340688069662
	-1.86898803710938
	-1.56213506062825
	-4.15211168924968
	-2.6002508799235
0.050479704797048	0.639205932617188
0.0191384615384615	-0.793899536132813
0.0449256198347107	-0.695662180582683
0.0504119850187266	-0.754367828369141
0.0024	-1.15357208251953
0.0449317269076305	-0.657033284505207
0.0124238410596026	-0.880011876424152
0.0245	-0.781510670979817
0.031553488372093	-0.71783765157064
	-1.43376286824544
0.0138698224852071	-0.840259552001953
0.0119183673469388	0.930247624715172
0.0089922480620155	-0.998640060424805
0.0312	-0.729557037353516
0.00966666666666667	-1.16266314188639
0.0461875	-0.663667043050133
0.0134430379746835	-0.94001070658366
0.032331797235023	-0.791574478149414
0.0181030927835052	-0.787729899088543
0.0177291666666667	0.805729548136394
0.0506015037593985	0.66114107767741
0.00161194029850746	1.25571950276693
	1.8460521697998
0.00893103448275862	1.16159884134929
0.00177049180327869	1.2743771870931
0.0352286995515695	-0.736380259195965
0.0178219895287958	0.786303838094078
0.0141204819277108	0.829216003417969
	1.64560254414876

	1.51519393920898
0.0125906040268456	0.917482376098633
	1.86519877115886
0.00147945205479452	1.28478495279948
0.002125	-1.10334142049153
	-2.23456637064616
0.0023448275862069	-1.15171241760254
	-1.52306683858236
0.0362631578947368	-0.710178375244141
0.013952380952381	-0.819260279337566
0.0313492822966507	-0.738612492879231
0.0502941176470588	-0.646465301513672
0.0349864253393665	-0.716074625651043
0.00403669724770642	-1.07285372416178
0.0145411764705882	-0.841941197713215
0.0391111111111111	-0.688275655110679
	-2.05209541320801
0.0140359281437126	-0.88661829630534
0.00166153846153846	-1.41437021891276
0.00156521739130435	1.23554992675781
0.00951111111111111	0.962760925292969
0.0236565656565657	0.761424382527668
	1.64867782592773
0.00145945945945946	-1.41044553120931
0.029064039408867	-0.740463256835938
0.0150057803468208	-0.96243222554525
0.0133584905660377	-0.887386957804363
0.00502654867256637	1.05860265096029
0.0470232558139535	0.701179504394531
0.01475	-0.795193354288735
0.017072625698324	-0.81261380513509
0.0137037037037037	-0.827550252278648
0.00163636363636364	-1.34277534484863
0.0158426966292135	0.781352996826172
0.00219354838709677	1.12098058064779
0.0350714285714286	-0.699520746866863
	1.6735954284668
	-2.67341105143229
0.0351858407079646	-0.755165735880531
	-1.60208638509114
0.00237209302325581	-1.13876342773438
0.0326238532110092	-0.79466946919759
0.00255	1.32250022888184
	1.87198384602864
	1.99321683247884
0.0014025974025974	1.2416566212972
	1.37647755940755

0.00231818181818182	1.21739896138509
	-1.93698501586914
0.00251851851851852	-1.14822514851888
0.0118378378378378	1.01276461283366
0.0106666666666667	-0.967777887980144
0.0176373056994819	-0.790576934814453
0.0349155555555556	0.708343505859379
0.0485038167938931	-0.665564219156899
0.0208775510204082	-0.749130249023438
0.0502238805970149	0.661335627237957
0.018010582010582	0.788318634033203
	-1.65351231892904
0.00248780487804878	1.15350786844889
0.00154285714285714	-1.47129821777344
0.00198058252427184	-1.25881195068359
0.0169777777777778	0.778429667154946
	-1.87692260742188
0.00221739130434783	-1.12668164571126
	-1.496062596639
0.0143720930232558	0.79496955871582
0.0502074074074074	0.645916620890301
0.0112275862068966	-0.95002047220866
0.012987012987013	0.876633961995442
0.00214736842105263	-1.2050609588623
0.0334545454545455	-0.748357772827148
0.00943089430894309	1.06218083699544
0.0289661835748792	-0.718214670817058
	1.35000610351563
0.00294339622641509	1.13654708862305
0.003	1.16437085469564
0.0374260869565217	-0.744771321614582
0.0024578313253012	1.16140047709147
0.013875	-0.876588821411133
0.00297142857142857	1.08061536153157
0.0465511811023622	0.699536005655922
0.00991452991452992	1.08672777811686

Fold change C_R	Student's T-test Test statistic C_R
0.568763836185858	-0.377018903554641
1.65441833726892	0.33535756759813
1.61528030328328	0.320555614440485
1.64784703366914	0.351084442423118
0.629707943214924	-0.312963296643441
1.80910654904564	0.387653116748981
1.65150091735763	0.338348106662427
0.633583825793587	-0.310359827402602
0.399399016277069	-0.621261471304779
0.565226691170866	-0.378562653785575
0.33799217994365	-0.733160341189351
1.65342270351751	0.345839060720112
0.536728264470928	-0.406191273140686
0.516606911461709	-0.455338969210975
0.0567129477659937	-1.72328623777499
2.82037399744645	0.682143934222148
0.519366944510062	-0.462914021059113
1.72515881133959	0.37583622418007
0.44002477556251	-0.538342383067291
4.33418711612443	0.922170043041004
0.103111694139827	-1.37835026834256
0.611477070272609	-0.327312774772916
2.52781566249084	0.587742119823713
0.319876520615231	-0.776242184845276
0.418043405401312	-0.59675724734809
3.04669912533675	0.742631463565293
0.441992941408046	-0.564827289721857
0.637956550806292	-0.305782796715102
0.539594133646047	-0.403157269206827
0.574196369663883	-0.375141701579875
1.58698487218367	0.31526001098632
0.4813255424309	-0.504759318615044
3.81349938829772	0.839088335358609
1.94153172584827	0.40946232816662
1.71125068258115	0.332790784564328
0.360774133719081	-0.630627178968221
2.54851537813817	0.6156770548341
2.76338463791832	0.674276904698271
1.77377487527588	0.392620409866147
0.630892622237323	-0.311734321890119
2.32163724295748	0.563385928033891
1.96835578888099	0.472295812168308
3.10725735036352	0.77166393557054
2.12072730461665	0.50433115257567
0.511379957824589	-0.453842074454279

2.26364841980601	0.580324120560993
2.11343421757676	0.502210572718876
2.44266813591186	0.614288623990757
2.1942811263164	0.541138244944729
2.17452294049062	0.53353459581816
1.76485799310948	0.37518531202503
1.68591496865189	0.345812796538656
1.63240907919874	0.315830367030272
5.47614483437783	1.15634301835489
2.09959021328318	0.474107578761677
1.62768809996349	0.327869070879882
1.62468821788166	0.316422105156296
0.632444538260767	-0.31000207963083
2.19767792340864	0.540522700539067
2.77711155157331	0.68779815662784
2.04266875487758	0.477850867535571
1.84974764982421	0.417869359609065
0.420148889156689	-0.5808491838517
0.464792816938158	-0.531121048088336
2.58778901206556	0.626707036089152
7.24215276735033	1.29804233947602
2.07218462349367	0.483875731766157
0.486106465518544	-0.458629581160984
1.73303633227929	0.377832989616723
0.622981565251368	-0.327628728008552
0.590263900540415	-0.346341030002341
2.00106234761857	0.451720565761675
1.99492352232729	0.461859475078061
2.17056592053135	0.483196402343848
0.591610275083564	-0.357477201269993
0.473366882126776	-0.476366108200442
1.61955342428071	0.318404915653734
0.587049487704472	-0.363936863975845
3.93947099373697	0.951533375281194
0.541261502626936	-0.387674791335965
2.41111377901656	0.603935011087949
3.27559796273196	0.739251062947702
0.355066866139863	-0.701655775590679
3.08469351823628	0.706431083888882
0.556115467835183	-0.400465245384516
1.74072964548126	0.37209183639217
2.35725564966999	0.575350292443445
1.69865797615656	0.357448809448173
2.15357368471112	0.475622163708615
2.09918220853507	0.491368899454098
1.95558362883685	0.468042136712323
2.15400744916325	0.528577594665687
1.73267283316327	0.375445210051163

1.5754447662874	0.309829551225744
3.56673557445427	0.792760130640731
1.6680806710184	0.342085815454268
2.06273095983383	0.50931331923657
2.28914214870625	0.546840224951009
4.79658847058889	1.01239513594619
2.09310669441723	0.511423839976
2.57445552886712	0.620288071369815
2.73825945342318	0.672513579785976
1.62655013066593	0.316356249658633
1.63066693196197	0.317867505935222
2.43805017824862	0.606409828466003
2.14238589665226	0.539853714162006
2.34108497621192	0.571439901538639
0.531889488271432	-0.416152057014562
1.82643286793484	0.417885485703395
0.609583998148065	-0.33153454896669
1.74771216763019	0.384175160229308
1.90661105172938	0.443208870857133
1.59570048961278	0.32177800576197
0.259976031634386	-0.823412570337636
1.88102876586367	0.430823046876929
0.199941054004487	-1.0787091204959
2.74834207052229	0.619676608948974
0.484734807808642	-0.454157500452889
3.51407582055355	0.856474145047417
0.56391476250762	-0.353277089997718
1.63454201859788	0.319639098733711
1.99656996091969	0.458628604547968
0.354631819953962	-0.728464796189212
0.386377308889874	-0.532932205021437
2.12219137090682	0.528801229228933
1.66022035806378	0.331408341705656
0.481654858893286	-0.497683058519193
1.68723573940674	0.353210155685241
2.17832685347138	0.515165798363247
1.6065342027194	0.312617318371068
2.48322421919033	0.622052021805022
0.604373855702221	-0.32640395505863
0.619341649599032	-0.333430582279635
1.60757598678909	0.320886196562285
1.58140882205531	0.319786387721325
2.08852087329945	0.484178304324318
1.89783079849028	0.428599513609716
1.67263646641542	0.34540935805316
0.535414052033214	-0.437613118027323
2.3028543741755	0.530897443554161
1.74109636941447	0.379050226702413

1.60636712686763	0.323298155729946
0.629070555635264	-0.319810672093777
0.463476398412774	-0.481675385724512
1.92694549607865	0.446316588318336
1.99474418479658	0.435438576516871
3.58282168263121	0.877267788868514
0.492720629511796	-0.360858679877334
1.57160173642674	0.314874207001122
3.34546163985411	0.753555597918523
0.606649495345491	-0.325677737268797
1.70569422186503	0.345051464597433
0.585533611381672	-0.353620370219977
0.484804452383611	-0.491214239971954
0.182595410889458	-1.12636816132676
0.256422207839739	-0.929922469262322
0.273765387763597	-0.892840893211052
0.338649539569373	-0.684749793136904
0.0562457660600887	-1.92407097074139
0.164909809079511	-1.23665649530073
1.55747168227732	0.30532168104232
0.576782966132259	-0.366487450501334
0.61742586208234	-0.320999865678872
0.592806089071949	-0.306180606553322
0.449510872861972	-0.543905491729917
0.63418106826408	-0.318193579820665
0.543362958221021	-0.434200369287387
0.581757306030371	-0.360244029300931
0.608008057713239	-0.345308608351648
0.370164162349533	-0.623875484332878
0.558543073775263	-0.395072621009047
1.90560304643753	0.437402591703982
0.500471541352688	-0.475044596290709
0.603089056865426	-0.346522572216438
0.446687212003875	-0.487676959217948
0.631271689284187	-0.315044476412373
0.52122901224151	-0.413339984528225
0.577713263256004	-0.343217585046575
0.579254840855144	-0.368267435977678
1.74802951933921	0.371513695749957
1.58133286038372	0.306207317101658
2.38786206686312	0.588855076553034
3.59515050958815	0.836254982972745
2.23705207643373	0.496207166260775
2.41894369256574	0.609361704934905
0.600243483360678	-0.337599698815317
1.72465027092955	0.371757329232266
1.7767195860661	0.39854266355289
3.12878504230618	0.795124834935919

2.85837250014378	0.704423286814366
1.88881627286981	0.436924915478621
3.64318124880862	0.853127223664294
2.43645732351051	0.579739429921429
0.465437248990817	-0.535793788320504
0.212485106615857	-1.05683771120018
0.450090675647722	-0.542107411784115
0.347945477627054	-0.667421467661172
0.611244559714574	-0.334654157530599
0.566732451893326	-0.395372082765646
0.599315464867788	-0.348735404494009
0.638843606977478	-0.304843426607909
0.608751519895294	-0.33939781090295
0.475377747046867	-0.510971054038941
0.557892399634167	-0.392827530617227
0.620595157588701	-0.330284528479775
0.241133598152645	-0.957357834471593
0.540880467257134	-0.396881340229629
0.375173486431831	-0.590453169021519
2.3547108725097	0.586224323923608
1.94903625250639	0.460995048874725
1.69516344507879	0.362035756095441
3.13546154549986	0.774006484548766
0.376195492708628	-0.57834272417097
0.598547124872395	-0.354509402687313
0.513190999897521	-0.387795421794127
0.54059236530717	-0.41328278399425
2.08291310023497	0.504116645365309
1.62583348240676	0.314363573958549
0.576265935345734	-0.387275353966019
0.569349403214502	-0.380242447501957
0.563485248531527	-0.408000958008311
0.394261476284523	-0.589420689568977
1.71874199810194	0.383765616684837
2.17494750569875	0.538405649322328
0.615776729393283	-0.33724360961445
3.1900862455051	0.743190445890992
0.15675560652908	-1.23505654991108
0.592478318032107	-0.336177007854839
0.329400263651602	-0.701265692873217
0.454148673781775	-0.543134730947666
0.576475232490323	-0.342480722763256
2.50099162927667	0.560352120435804
3.66035568698789	0.852976193793566
3.98123720961392	0.947008279443744
2.3646991067874	0.570059459671607
2.59633682835781	0.641104139664113

2.32527115419818	0.541352972401793
0.261161653181239	-0.942621698068273
0.451179946615471	-0.55719710526286
2.01777402495594	0.437388108740291
0.511292976031	-0.45205434556617
0.578112857960698	-0.370454769550662
1.63392697411393	0.337155625038744
0.630442098539419	-0.310506496200782
0.594962131510088	-0.364044273032994
1.58154611983817	0.306161097867651
1.72706051410129	0.373399438163933
0.317865353705969	-0.776356355800512
2.22454127710405	0.555878982222957
0.360657612904124	-0.585765229374913
0.417887945872076	-0.527909841203014
1.71526284120511	0.379305624791163
0.272263858886518	-0.844988222883754
0.457967890175228	-0.539298479927235
0.354519626446169	-0.731644708880074
1.73504076405409	0.387930313369194
1.56473312868144	0.305674495934051
0.51762511663684	-0.442144042761309
1.83608641693907	0.424361593089631
0.433751015706104	-0.536788279889368
0.59528078315557	-0.340632110881889
2.08808557297921	0.483555046461407
0.607849187831917	-0.352309452091848
2.54913203906205	0.641443932728058
2.19854199266111	0.517264009890407
2.24135450995459	0.521155763986234
0.596762455510394	-0.33314530189573
2.23674451270283	0.555106918653302
0.544653715823135	-0.410747309154943
2.1149379861844	0.517420377795585
1.62398240938375	0.315665856414318
2.12391757353099	0.492151644549336
