# Supplementary material for: Comprehensive Temporal Protein Dynamics during Postirradiation Recovery in Deinococcus radiodurans
Source: Oxid Med Cell Longev. 2022 Nov 11;2022:1622829. doi: 10.1155/2022/1622829 (PMC9674996; doi:10.1155/2022/1622829)
Supplement: Supplementary file 4 [file 1622829.f4.odt]

Table S8 Protein IDs in cluster 1 and cluster 2

Cluster1

Protein IDs	Majority protein IDs
sp|Q9RXW9	EX7L_DEIRA
tr|Q9RZU7	Q9RZU7_DEIRA
tr|Q9RV11	Q9RV11_DEIRA
tr|Q9RXG0	Q9RXG0_DEIRA
tr|Q9RYZ6	Q9RYZ6_DEIRA
sp|Q9RWB4	RL19_DEIRA
sp|Q9RY52	RS6_DEIRA
tr|Q9RXG1	Q9RXG1_DEIRA
tr|Q9RTD6	Q9RTD6_DEIRA
tr|Q9RTG7	Q9RTG7_DEIRA
tr|Q9RZP7	Q9RZP7_DEIRA
tr|Q9RXQ2	Q9RXQ2_DEIRA
tr|Q9RX10	Q9RX10_DEIRA
sp|Q9RZA4	BPHY_DEIRA
tr|Q9RUK0	Q9RUK0_DEIRA
tr|Q9RW99	Q9RW99_DEIRA
tr|Q9RRY4	Q9RRY4_DEIRA
sp|Q9RXJ7	RL22_DEIRA
sp|Q9RU68	RSMA_DEIRA
sp|Q9RVV9	RPOB_DEIRA
tr|Q9RUA2	Q9RUA2_DEIRA
sp|P56867	HPI_DEIRA
sp|Q9RSJ7	RS4_DEIRA
tr|Q9RY79	Q9RY79_DEIRA
sp|Q9RSS8	RL1_DEIRA
sp|Q9RSW7	RL20_DEIRA
sp|Q9RVW0	RPOC_DEIRA
sp|Q9RXJ0	RL5_DEIRA
sp|Q9RSK9	RL15_DEIRA
sp|Q9RY49	RL9_DEIRA
tr|Q9RVS6	Q9RVS6_DEIRA
tr|Q9RZJ2	Q9RZJ2_DEIRA
tr|Q9RXP5	Q9RXP5_DEIRA
tr|Q9RZT2	Q9RZT2_DEIRA
sp|Q9RUF3	SYM_DEIRA
tr|Q9RSW3	Q9RSW3_DEIRA
tr|Q9RX33	Q9RX33_DEIRA
tr|Q9RW56	Q9RW56_DEIRA
tr|Q9RTC9	Q9RTC9_DEIRA
tr|Q9RT09	Q9RT09_DEIRA
sp|Q9RYD8	PARB1_DEIRA
sp|Q9RRC4	SYR_DEIRA
sp|Q9RWU0	SECA_DEIRA
tr|Q9RSY6	Q9RSY6_DEIRA

Cluster2

Protein IDs
tr|Q9RUU2
tr|Q9RVW5
sp|Q9RT63
sp|Q9RY51
tr|Q9RYM2
tr|Q9RT53
tr|Q9RRS5
sp|Q9RXI7
sp|P42443
sp|O32504
sp|O32508
sp|Q46577
tr|Q9RS52
tr|Q9RYX6
tr|Q9RY55
tr|Q9RU16
tr|Q9RSS3
sp|Q9RYE3
tr|Q9RRC7
tr|Q9RSZ5
tr|Q9RUG4
tr|Q9RY13
tr|Q9RSU3
tr|Q9RRA0
tr|Q9RRW1
sp|Q9RT44
tr|Q9RSZ3
tr|Q9RWP5
tr|Q9RYC6
tr|Q9RVB2
tr|Q9RYD0

sp|Q9RY32	EFP_DEIRA
sp|Q9RTG5	IF2_DEIRA
sp|Q9RWQ9	CH60_DEIRA
tr|Q9RWB7	Q9RWB7_DEIRA
tr|Q9RUX8	Q9RUX8_DEIRA
sp|Q9RWK0	CARB_DEIRA
sp|Q9RT23	FABH1_DEIRA
sp|Q9RUP5	TPIS_DEIRA
tr|Q9RZB3	Q9RZB3_DEIRA
tr|Q9RWX7	Q9RWX7_DEIRA
tr|Q9RU54	Q9RU54_DEIRA
tr|Q9RT93	Q9RT93_DEIRA
tr|Q9RS41	Q9RS41_DEIRA
sp|Q9RT38	GLPK_DEIRA
sp|Q9RUW4	SYP_DEIRA
tr|Q9RZC5	Q9RZC5_DEIRA
tr|Q9RZC4	Q9RZC4_DEIRA
tr|Q9RTU7	Q9RTU7_DEIRA
tr|Q9RRI7	Q9RRI7_DEIRA
sp|Q9RSE6	PUR8_DEIRA
sp|Q9RWM0	ERA_DEIRA
tr|Q9RZB2	Q9RZB2_DEIRA
tr|Q9RT97	Q9RT97_DEIRA
sp|Q9RVY3	Y888_DEIRA
tr|Q9RRD6	Q9RRD6_DEIRA
tr|Q9RU26	Q9RU26_DEIRA
tr|Q9RY67	Q9RY67_DEIRA
tr|Q9RYE8	Q9RYE8_DEIRA
sp|Q9RWR0	CH10_DEIRA
tr|Q9RT02	Q9RT02_DEIRA
tr|Q9RTV1	Q9RTV1_DEIRA
tr|Q9RRF0	Q9RRF0_DEIRA
sp|Q9RY24	GRPE_DEIRA
sp|Q9RRB7	PPAC_DEIRA
sp|Q9RXT9	THtr|_DEIRA
tr|Q9RXZ4	Q9RXZ4_DEIRA
sp|Q9RSL3	RL6_DEIRA
sp|Q9RXK1	RL4_DEIRA
sp|Q9RU79	RS2_DEIRA
sp|Q9RXJ9	RL2_DEIRA
tr|Q9RWH4	Q9RWH4_DEIRA
sp|Q9RUP8	SYI_DEIRA
tr|Q9RWG9	Q9RWG9_DEIRA
sp|Q9R342	EFTU_DEIRA
sp|P56864	SAT_DEIRA
sp|Q9RXK5	EFG_DEIRA
tr|Q9Rtr|4	Q9Rtr|4_DEIRA
sp|Q9RWH0	VATC_DEIRA

sp|Q9RSJ6	RPOA_DEIRA
sp|Q9RSS6	NUSG_DEIRA
tr|Q9RSM3	Q9RSM3_DEIRA
tr|Q9RZD6	Q9RZD6_DEIRA
tr|Q9RVP1	Q9RVP1_DEIRA
sp|P56861	CYSC_DEIRA
tr|Q9RSL8	Q9RSL8_DEIRA
tr|Q9RRK9	Q9RRK9_DEIRA
sp|P83589	HCDH_DEIRA
sp|Q9RXU7	tr|MFO_DEIRA
tr|Q9RZ81	Q9RZ81_DEIRA
tr|Q9RYD9	Q9RYD9_DEIRA
sp|Q9RS19	DER_DEIRA
sp|Q9RY66	OBG_DEIRA
tr|Q9RW96	Q9RW96_DEIRA
tr|Q9RX57	Q9RX57_DEIRA
tr|Q9RWV1	Q9RWV1_DEIRA
sp|Q9RSZ6	CLPX_DEIRA
sp|Q9RT21	TIG_DEIRA
tr|Q9RXD5	Q9RXD5_DEIRA
tr|Q9RY75	Q9RY75_DEIRA
tr|Q9RX59	Q9RX59_DEIRA
sp|Q9Rsp|3	SYT_DEIRA
tr|Q9RRE0	Q9RRE0_DEIRA
tr|Q9RY54	Q9RY54_DEIRA
sp|Q9RXT4	PURL_DEIRA
tr|Q9RXS7	Q9RXS7_DEIRA

Majority protein IDs
Q9RUU2_DEIRA
Q9RVW5_DEIRA
RECDL_DEIRA
SSB_DEIRA
Q9RYM2_DEIRA
Q9RT53_DEIRA
Q9RRS5_DEIRA
DDRD_DEIRA
RECA_DEIRA
PPRA_DEIRA
CINAL_DEIRA
UVRA_DEIRA
Q9RS52_DEIRA
Q9RYX6_DEIRA
Q9RY55_DEIRA
Q9RU16_DEIRA
Q9RSS3_DEIRA
Y006_DEIRA
Q9RRC7_DEIRA
Q9RSZ5_DEIRA
Q9RUG4_DEIRA
Q9RY13_DEIRA
Q9RSU3_DEIRA
Q9RRA0_DEIRA
Q9RRW1_DEIRA
SBCC_DEIRA
Q9RSZ3_DEIRA
Q9RWP5_DEIRA
Q9RYC6_DEIRA
Q9RVB2_DEIRA
Q9RYD0_DEIRA
